# Structure and expression analysis of seven salt-related *ERF* genes of *Populus*

**DOI:** 10.7717/peerj.10206

**Published:** 2020-10-20

**Authors:** Juanjuan Huang, Shengji Wang, Xingdou Wang, Yan Fan, Youzhi Han

**Affiliations:** College of Forestry, Shanxi Agricultural University, Taigu, China

**Keywords:** ERF, Transcription factor, Salt stress, Gene express, *Populus*

## Abstract

Ethylene response factors (ERFs) are plant-specific transcription factors (TFs) that play important roles in plant growth and stress defense and have received a great amount of attention in recent years. In this study, seven *ERF* genes related to abiotic stress tolerance and response were identified in plants of the *Populus* genus. Systematic bioinformatics, including sequence phylogeny, genome organisation, gene structure, gene ontology (GO) annotation, etc. were detected. Expression-pattern of these seven *ERF* genes were analyzed using RT-qPCR and cross validated using RNA-Seq. Data from a phylogenetic tree and multiple alignment of protein sequences indicated that these seven ERF TFs belong to three subfamilies and contain AP2, YRG, and RAYD conserved domains, which may interact with downstream target genes to regulate the plant stress response. An analysis of the structure and promoter region of these seven *ERF* genes showed that they have multiple stress-related motifs and* cis*-elements, which may play roles in the plant stress-tolerance process through a transcriptional regulation mechanism; moreover, the cellular_component and molecular_function terms associated with these *ERFs* determined by GO annotation supported this hypothesis. In addition, the spatio-temporal expression pattern of these seven *ERFs*, as detected using RT-qPCR and RNA-seq, suggested that they play a critical role in mediating the salt response and tolerance in a dynamic and tissue-specific manner. The results of this study provide a solid basis to explore the functions of the stress-related ERF TFs in *Populus* abiotic stress tolerance and development process.

## Introduction

Plant growth, development, and biomass productivity are seriously affected by abiotic and biotic stresses from the environment (Singh, Foley & Oñate Sánchez, 2002; Meng et al., 2016; Zhu, 2016). To adapt to various environmental effects, plants have developed a series of response strategies that are regulated by multiple signaling pathways (Song et al., 2016; Raza et al., 2019; Gong et al., 2020). Many genes play important roles in the stress-response process, with a significant overlap between the patterns of expression of the genes that are induced (Durrant et al., 2000; Seki et al., 2001; Chen et al., 2002; Scarpeci et al., 2017; Yao et al., 2017; Li et al., 2019). The pattern that originally appeared to be several linear pathways is now being revealed as a complex regulatory network of signals that allow plants to respond optimally to their changing environment (Singh, Foley & Oñate Sánchez, 2002; Song et al., 2016). The regulation of the expression of stress-response genes occurs at the transcriptional level, and the regulation of the spatio-temporal expression patterns of their target genes plays an important role in plant stress endurance. Plants have evolved and coded a large number of transcription factors (TFs) to achieve the transcriptional regulatory process. These TFs are often divided into different gene families, such as APETALA2/ethylene responsive factor (AP2/ERF), NAC, WRKY, MYB, and bZIP, whereas some TFs are unique to plants (Riechmann et al., 2000).

The AP2/ERF family, which plays important roles in plant growth and stress response endurance, has been studied extensively in recent years. The members of this family are characterized by the presence of an AP2 DNA-binding domain, for interaction with GCC-box, DRE, or C-repeat *cis*-acting elements at the promoter regions of downstream target genes in a direct or indirect way. The AP2/ERF family is divided into five subfamilies, including the ERF subfamily, according to the number and similarity of the AP2 DNA-binding domains (Sakuma et al., 2002). Many *ERF* genes were isolated and cloned from plants and microorganisms in recent decades (Magnani, Sjölander & Hake, 2004; Shigyo, Hasebe & Ito, 2006; Zhang et al., 2009). Several ERFs were shown to be involved in diverse processes of plant development and stress-response processes, including vegetative and reproductive development, cell proliferation, and salt, drought, and hormone responses. Overexpression of these *ERFs* has been studied in *Arabidopsis*, tomato, tobacco, *Populus* plants, and rice, leading to the improvement of drought or salt tolerance in the transgenic plants (Zhang et al., 2009; Zhang & Huang, 2010; Yang et al., 2011; Cheng et al., 2019; Li et al., 2019). *Populus* plants overexpressing *ERF76* exhibited increased salinity-stress tolerance with an increased root length, fresh weight, and abscisic acid and gibberellin concentration compared with the control plants (Yao et al., 2016b). Overexpression of *AP37* in rice improved the drought and saline resistance of the transgenic plants at the reproductive growth stage. In addition, rice plants overexpressing *AP37* showed no undesirable phenotypes and produced a higher grain yield compared with the control plants under severe drought conditions (Oh et al., 2009). When exposed to normoxia or hypoxia conditions, the expression of *AtERF73*/*HRE1* is induced or reduced in order to regulate ethylene responses of *Arabidopsis* (Yang et al., 2011).

In 2008, a genome-wide analysis of the *ERF* family was performed in *Populus trichocarpa*, leading to the identification of 200 ERF TFs (Zhuang et al., 2008). These ERF TFs were classified into four subfamilies based on a phylogenetic analysis. Subsequently, the expression pattern of genes in the ERF family was analyzed under salt and other abiotic stresses in *Populus* plants using the RT–qPCR and RNA-seq methods (Wang et al., 2014b; Yao et al., 2017). A transcriptome analysis of *ERF* genes under multiple abiotic stresses identified seven genes that were induced by NaCl, KCl, PEG, and CdCl_2_ (Yao et al., 2019). However, no systematic analyses of *ERF* genes, including gene/protein sequence, gene structure, promoter *cis*-element prediction, and expression pattern analyses, have been reported. In this study, we performed a systematic analysis of these seven important *ERF* genes of *Populus*. Multiple alignment and a phylogenetic tree were used to analyze the sequence characters of these genes. Intron–exon and conserved motifs in the sequence were detected, together with *cis*-elements in the promoter regions, to predict the function of these genes in transcriptional regulation. A chromosome location and synteny analysis indicated that *ERF* genes have undergone the whole-genome and tandem duplication events in their evolutionary history. Gene ontology (GO) annotation and enrichment showed that these seven *ERF* genes play important roles in the cellular_component and molecular_function areas of *Populus*. Finally, the spatio-temporal expression patterns of these seven genes suggested that they play a critical role in mediating salt response and tolerance in a dynamic and tissue-specific manner.

## Materials and Methods

### Screening and phylogenetic analysis

In recent years, we have focused exclusively on the expression and function of transcription factors of *Populus* in response to abiotic stresses (Wang et al., 2014a; Yao et al., 2016b; Wang et al., 2018). Seven *ERFs* induced by multiple abiotic stresses were selected for the detection of expression patterns related to salt stress in this study (Yao et al., 2019). The systematic search of the seven ERFs in *Populus* was performed using PlantTFDB (Version 4.0, http://planttfdb.gao-lab.org/) (Jin et al., 2017). The orthologous sequences in *Arabidopsis* were searched and downloaded from the *Arabidopsis* genome TAIR 9.0 (http://www.Arabidopsis.org/index.jsp). Multiple sequence alignments of ERF proteins were performed using Clustal X 1.83 and BioEdit 7.0.5.3 (Thompson et al., 1997; Hall, 1999). Phylogenetic trees constructed with MEGA 10.0.5 using the Neighbor Joining (NJ) method were carried out with 1,000 iterations bootstrap test (Kumar et al., 2018). The information of these seven ERF TFs is listed in Table S1.

### Gene structure and conserved motifs

Gene structure of seven ERF TFs were detected using TBtools (http://www.tbtools.com/) by comparison with their corresponding genomic DNA sequences from Phytozome (https://phytozome.jgi.doe.gov/pz/portal.html#!info?alias=Org_Ptrichocarpa) (Chen et al., 2018). Conserved motifs in seven *Populus* ERF TFs were detected using the MEME software, version 5.0.2 (Bailey et al., 2009), which was run using parameters: any number of repetitions; maximum number of motifs, 6; optimum motif width, between 6 and 50 residues.

### Chromosome location and synteny analysis

The chromosome location of these seven TFs were illustrated using the “Map Genes On Genome From Sequence Files” methods of TBtools (Chen et al., 2018). The subject sequences used in this study were downloaded from Phytozome (https://phytozome.jgi.doe.gov/pz/portal.html#!info?alias=Org_Ptrichocarpa). Gene synteny of intra-species and collinearity characteristics of the seven TFs were analyzed using MCScanX with the default parameters and considering pBLAST ≤ 1e^−5^ (Wang et al., 2012). Colinear blocks between two sets of linkage groups (LGs) are linked using Circos-plots tool of TBtools (Chen et al., 2018). In this study, all of the 209 ERFs of *Populus* were obtained from PlantTFDB and used for synteny analysis (Jin et al., 2017).

### Cis-elements of promoter

The 2,000 bp upstream sequence of the translation start site were downloaded from the Phytozome v12.1 database and signed as the promoter sequence, respectively. The prediction and determination of the location of *cis*-elements in promoter sequence was performed with the PlantCRAE software (Lescot et al., 2002).

### GO annotation and enrichment

GO annotation and enrichment of seven ERF TFs were performed with OmicsBox 1.2.4 (https://www.biobam.com/omicsbox). According to the manual, blast annotation was proceed with the default configurations. On the basis of GO classification, these seven ERF TFs were involved into biological processes, molecular functions and cellular components three terms. GO enrichment was carried out using Fisher’s exact test methods in this study.

### Plant materials and salt stress treatment

The hybrid aspen 84K poplar (*P. alba* × *P. glandulosa*) were used as the materials in this study. Twigs from the same clone were cut and planted in Murashige and Skoog (MS) medium for 20 days under the controlled conditions: relative humidity, 60%–70%; light/dark cycle, 16/8 h; average temperature, 25 ± 2 °C. To monitor changes in gene expression patterns according to the different treatments, a time-course experiment was designed in this study. Strong and healthy strains with a similar state were subjected to the following treatments: 0.05 M NaCl for 0, 2, 12, 24, and 48 h. The first time point (hour 0) served as a control. After the completion of each treatment, root, stem, leaf, and shoot tissues were harvested from six seedlings, respectively. The samples from six seedlings per time point were pooled per tissue type, frozen immediately in liquid nitrogen, and stored at −70 °C for RNA isolation and RT–qPCR analysis. Total RNA was extracted according to the manuals (Wang et al., 2018).

### RT–qPCR

RT–qPCR was performed as described in our previous study (Wang et al., 2014a). Two housekeeping genes, *Actin* and *EF1*, were used as reference to monitor the expression change of target genes (Regier & Frey, 2010). The primers used for RT–qPCR are listed in Table S2. The relative expression level was calculated as 2^−ΔΔ*Ct*^.

### Validation of the expression data using RNA-Seq

RNA sequencing was used to cross-validate the gene expression data of RT–qPCR and the results of the two methods were compared. Twenty-six-day-old seedlings with the same genetic background and similar state of the 84K poplar and grown in MS medium were divided into two groups and treated with 100 mM NaCl (S1, S2, and S3) or regular water (W1, W2, and W3) for 24 h, respectively. Roots, stems, leaves, and shoots were respectively collected from each of the replicates. Three biological replicates were tested. Total 24 samples were collected and shipped to Majorbio Bio-pharm Technology Co.,Ltd. (Shanghai, China) for RNA-Seq using the Illumina Novaseq 6000 platform. Sequencing-library construction and RNA-Seq data analysis were proceed referred to previous study (Wang et al., 2014a). The results of gene expression level are indicated as transcripts per million reads (TPM).

### Statistics

A single-variable analysis was used to compare gene expression under salt and normal conditions, as well as between different tissues, using a *t*-test. We used an unsupervised clustering analysis to identify genes with similar expression patterns based on RNA-seq data. A correlation analysis was used to assess the relationships of genes. The data represent the mean ± SD of expression level. Statistics was calculated in R (v3.5.1, http://cran.r-project.org/).

## Results

### Screening and phylogenetic analysis

In this study, seven *ERF* genes of *Populus* that were reported to be related to abiotic stresses (Yao et al., 2019) were selected as the targets for the detection of expression patterns under salt stress. These genes were designated as *PtERF001*–*PtERF007*, for convenience (Table S1). The average length of the proteins encoded by them was about 215 amino acids, with molecular weights ranging from 14,649.4 to 54,060.9 Da and isoelectric point values ranging from 4.7314 to 10.2631 (Tables S1). Orthologs of the seven PtERFs were searched in *Arabidopsis*, yielding five hits with identification percent about 35%–64% (Tables S1). *PtERF002* and *PtERF003* hit the same ortholog, *AtERF1*, in *Arabidopsis*, whereas *PtERF004* and *PtERF005* hit *AtERF016* (Table S3). Detailed information on the seven PtERFs is provided in Tables S1 and S3, including TF_ID and orthologs in *Arabidopsis*, cDNA and protein sequences.

**Figure 1:**
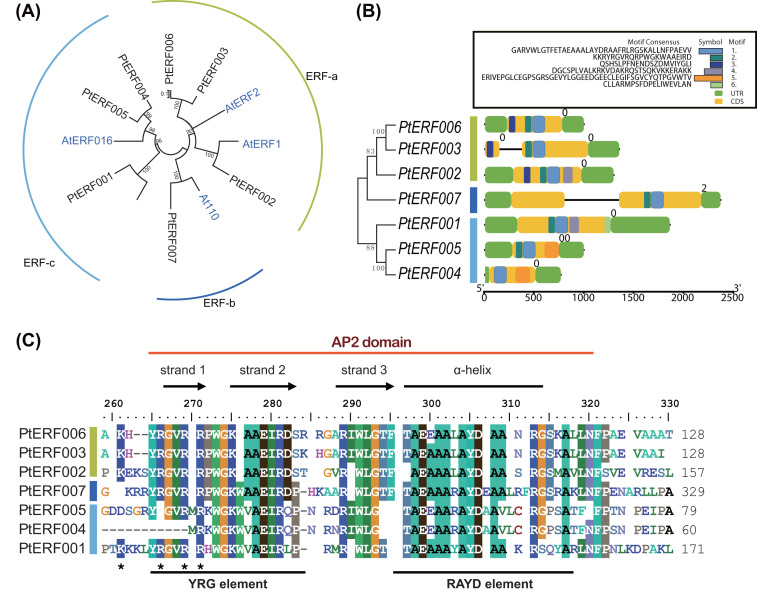
Phylogenetic tree and gene structure analysis of seven ERF TFs. (A) Phylogenetic tree of full-length amino acid sequences of seven PtERFs was constructed using MEGA 10.0.5 with the NJ method. (B) Gene structure and conserved motif were detected using the programme TBtools. The black lines indicate the introns. Conserved motifs were discovered using the MEME programme and were shown by colourful boxes. The sizes of conserved motifs, UTR, CDS and introns are estimated using the scale at the bottom. (C) Multiple alignments of the AP2 domain of ERFs were performed using the programme BioEdit 7.0.5.3.

Phylogenetic relationships between these seven PtERFs and the five *Arabidopsis* orthologs were detected via multiple alignments of the full-length protein sequence using NJ algorithm methods. The seven PtERFs were divided into three subfamilies: ERF-a, ERF-b, and ERF-c (Fig. 1A), with three PtERFs each belonging to subfamilies ERF-a and ERF-c, respectively. Only one TF, PtERF007, was attributed to subfamily ERF-b. Multiple alignment conducted using the AP2 domain sequence showed similar profile with that of the full-length amino acid sequences (Fig. 1C). On the other hand, gene structure and expression patterns of these seven *PtERFs* supported the classification of the subfamily in this study.

### Gene structure and conserved motifs

The structures, including the UTRs, CDS (coding sequence) and introns, of seven *ERFs* were investigated. Genes in the same subfamily shared similar exon/intron structures in terms of number of introns, with the exception of *PtERF003* from subfamily ERF-a, which possessed one intron (Fig. 1B). In addition, *PtERF007* in subfamily ERF-b also harbored one intron. The conserved motifs that were predicted using MEME showed that motif-1 was conserved among all seven PtERF proteins (Fig. 1B). Motif-3 was only found in members of subfamily ERF-a, whereas motif-5 was only contained in subfamily ERF-c. PtERF001 of subfamily ERF-c did not have motif-5, but carried motif-6. These findings indicate that the structure and function of the poplar ERF TFs are similar, especially among those belonging to the same subfamily.

### Analysis of chromosome location and synteny of ERF genes

**Figure 2:**
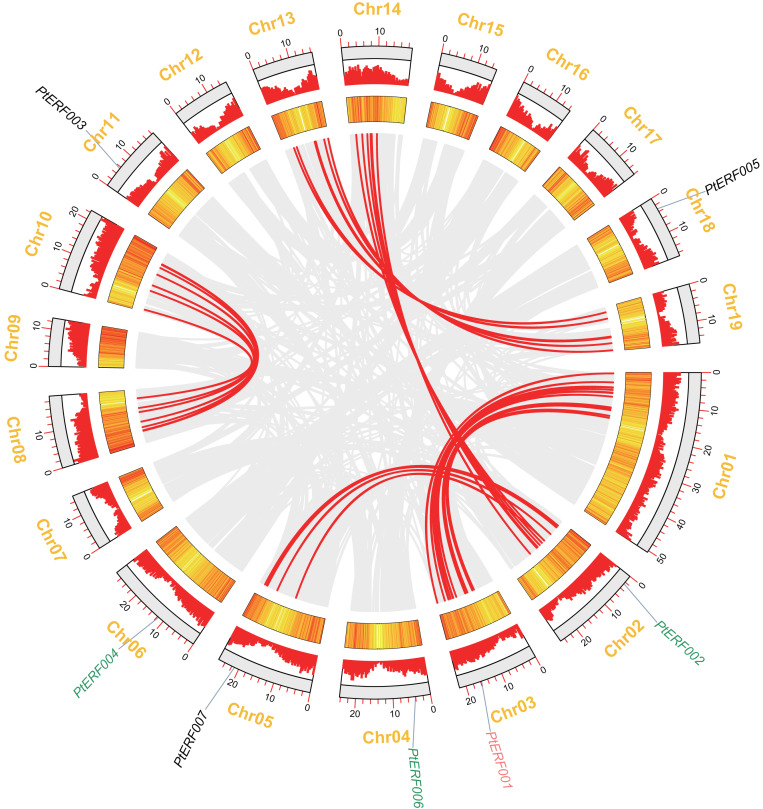
Syntenic relationships of seven *ERF* genes among 19 Populus LGs are detected using the MCScanX programme. The colinear blocks of *Populus* genome and *ERFs* are marked by grey and red connecting lines, respectively. The scales represent the distance of chromosomes. Bar and Heatmap graphs indicate the density of genes on each of LGs. The seven *ERFs* were mapped on 7 LGs. Whole-genome wide and tandemly duplicated genes are labelled in red and green, respectively.

In this study, seven *PtERF* genes were mapped on seven out of 19 LGs (Fig. 2). A synteny analysis of a total of 73013 genes of *Populus* was performed using MCScanX, which led to the identification of 21519 (29.47%) collinear genes (Fig. 2, Table S4). In contrast, 83 (39.71%) collinear genes were identified among the 209 *ERF* genes and considered to be the result of a whole-genome duplication event. They were located mainly on eight LGs of *Populus* (Fig. 2, Table S6). Our target gene *PtERF001* was included in these 83 collinear genes. In this study, 30 *ERF* transcription factor genes of poplar, including *PtERF002*, *PtERF004*, and *PtERF006*, related to tandem duplications that impact the expansion of the *ERF* gene family were identified (Fig. 2, Table S6) and were distributed on 12 of the 19 LGs (Table S5).

### Cis-elements in promoter sequences

Putative *cis*-elements in the promoter region of the seven *PtERFs* were detected using PlantCRAE. The core-sequence and function annotations of each putative *cis*-element are listed in Table S7. Most of the promoters of these target *ERFs* had multiple stress-related *cis*-acting regulatory elements, such as the ABRE, LTR, MBS, and TGACG motifs; however, the type and number of *cis*-elements varied among the different genes (Table S7). For example, the ABRE *cis*-element existed in all but *PtERF007*, and the CGTCA motif was not found in the promoter region of *PtERF001*. Whereas, the number of the same *cis*-element of promoter varied among these seven *Populus ERF* genes (Dataset S1).

### GO annotation and enrichment

**Figure 3:**
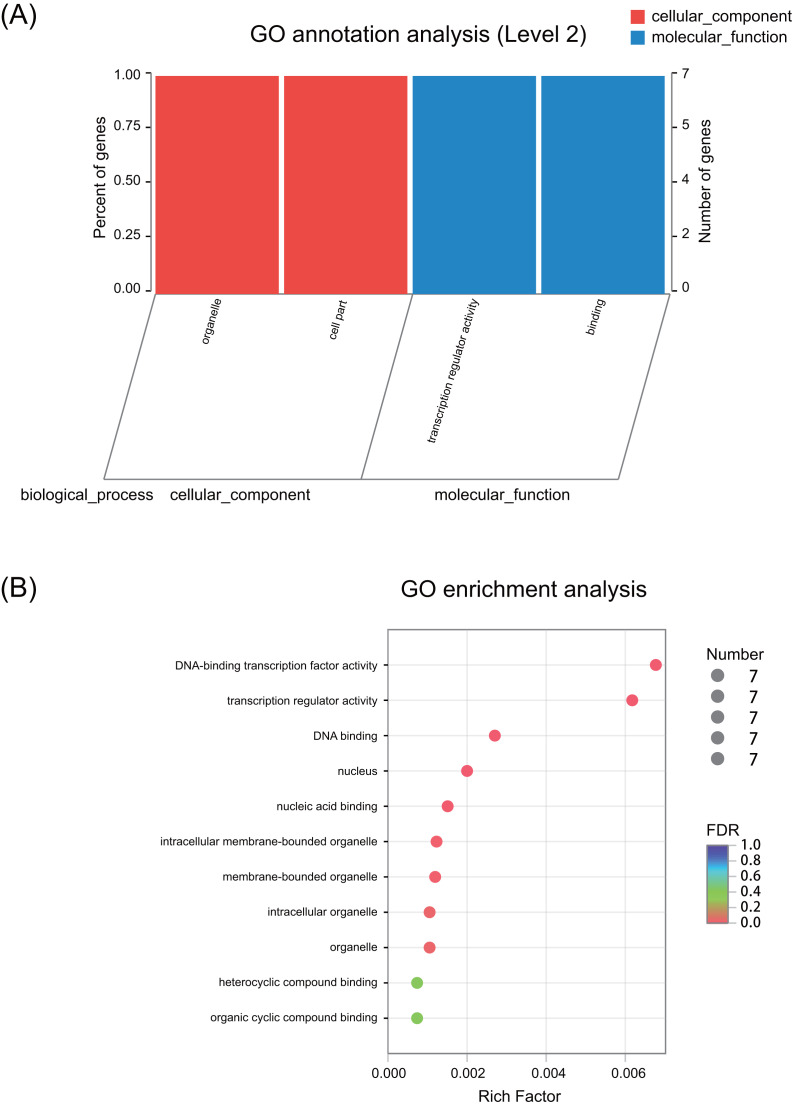
GO annotation and enrichment of the seven *ERF* genes. (A) GO annotation of seven ERF TFs for their involvement in cellular components and molecular functions. The abscissa represents the secondary classification term of GO, the left ordinate represents the percentage of the total number of genes included in the secondary classification, the right ordinate represents the number of mapped to the secondary classification, and the two colors represent the two major classifications. (B) GO enrichment of the seven* ERF* genes. The vertical axis represents the GO Term, while the horizontal axis represents the ratio of the Rich factor (the number of genes enriched in the GO Term to the Background number. The larger the Rich factor is, the greater the enrichment degree is). The size of the points represents the number of genes in the GO Term, and the color of the points corresponds to different FDR (Pvaule_corrected) ranges.

GO annotation results showed that these seven *ERF* genes functioned mainly in the cellular_component and molecular_function terms (Fig. 3A and Fig. S1). None of the genes were predicted to play a role in the biological_process term. The GO enrichment analysis showed that these seven *ERF* genes were highly enriched in heterocyclic compound binding and organic cyclic compound binding activities, although they were also enriched in the DNA-binding transcription factor activity and transcription regulator activity, which may be involved in the plant stress response process (Fig. 3B, Table S8). Moreover, cellular_component prediction showed these seven *ERF* genes function mainly in intracellular organelles (Fig. 3, Table S8).

### Expression patterns under salt stress

**Figure 4:**
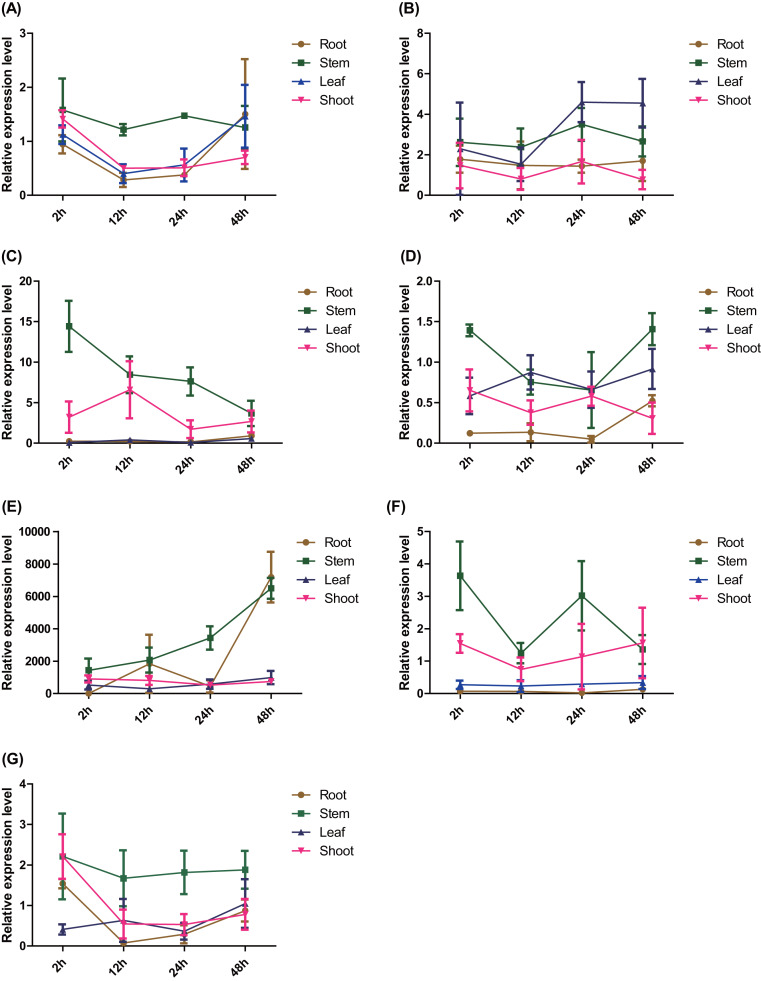
Spatio-temporal expression analysis of seven *ERF* genes under salt stress conditions based on RTq-PCR of *Populus,* respectively. The time point hour 0 serves as a control. Data represents Mean ± SD. (A) *PtERF001*, (B) *PtERF002*, (C) *PtERF003*, (D) *PtERF004*, (E) *PtERF005*, (F) *PtERF006*, (G) *PtERF007*.

The spatio-temporal expression patterns of the seven *ERFs* were detected using RT–qPCR. The expression of *PtERF003*, *PtERF004*, and *PtERF006* in roots treated with salt stress was suppressed to varying degrees (Figs. 4C, 4D, 4F), while *PtERF002* was overexpressed (Fig. 4B). The expression of *PtERF001* and *PtERF005* showed a dynamic pattern of “suppressed–induced amplification”, although the suppressed/induced degree and initial time points were different (Figs. 4A, 4E). However, *PtERF007* displayed the opposite expression pattern compared with *PtERF001* and *PtERF005*, which was induced at the 2 h time point and was suppressed thereafter (Fig. 4G). In stems, all seven *ERF* genes were induced by salt stress across the experimental period, with the exception of *PtERF004*, which was only induced at the initial and final time points (Fig. 4). In leaves, the expression of *PtERF002* and *PtERF005* was increased under salt stress, while *PtERF003*, *PtERF004*, and *PtERF006* were downregulated. The expression patterns of *PtERF001* and *PtERF007* exhibited similarities in that they were overexpressed at the 48 h time point, but *PtERF007* was suppressed at the initial time point (2 h). In shoots, the variation in the expression of these seven *ERF* genes was more complex compared with the tissues described above. The expression levels of *PtERF003* and *PtERF005* increased after treatment with salt stress, whereas the expression of *PtERF004* decreased significantly (Table S9). The expression of *PtERF001* and *PtERF007* showed an “induced–suppressed” profile and *PtERF006* displayed an “induced–suppressed–recovered” pattern; in contrast, the expression of *PtERF002* was relatively unstable. In general, these seven *ERF* genes showed specific, albeit similar, expression patterns.

**Figure 5:**
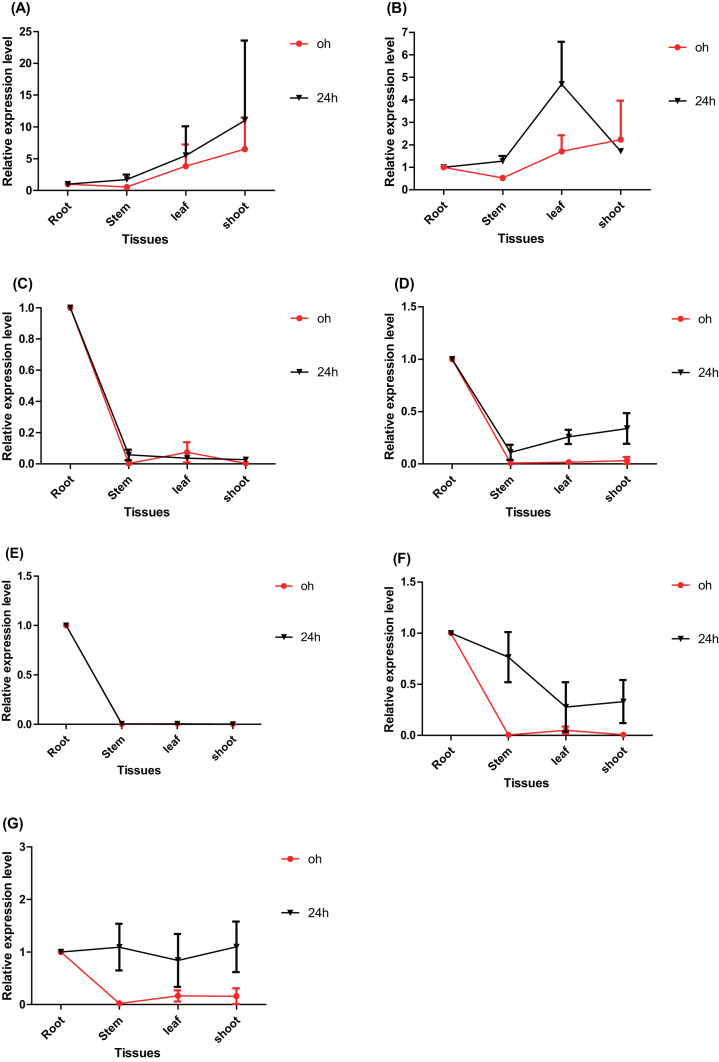
Tissue-specific expression analysis of seven *ERF* genes based on RT-qPCR of *Populus*. Expression level of each genes in the root tissues under the normal (0 h) and salt stress (24 h) conditions was used as the control and normalized as 1.0, respectively. Black indicates treated with NaCl for 24 h and red denotes the control (normal condition). Data represents Mean ± SD. (A) *PtERF001*, (B) *PtERF002*, (C) *PtERF003*, (D) *PtERF004*, (E) *PtERF005*, (F) *PtERF006*, (G) *PtERF007*.

The tissue-specific expression pattern of the seven *ERFs* was also investigated in this study. The expression level of each gene in the root tissues under normal conditions (0 h) and salt stress (24 h) conditions was used as the control and normalized as 1.0, respectively (Fig. 5). Under normal conditions, the tissue-specific expression patterns of *PtERF001* and *PtERF002* were similar, with lowest expression in stems and highest expression in shoots. The remaining five genes were significantly overexpressed in roots (Fig. 5, Table S10). After treatment with salt stress for about 24 h, the relative expression levels of individual genes in different tissues varied significantly, with the exception of *PtERF001* and *PtERF005* (Fig. 5, Table S10).

### Validation of the expression using RNA-Seq

**Figure 6:**
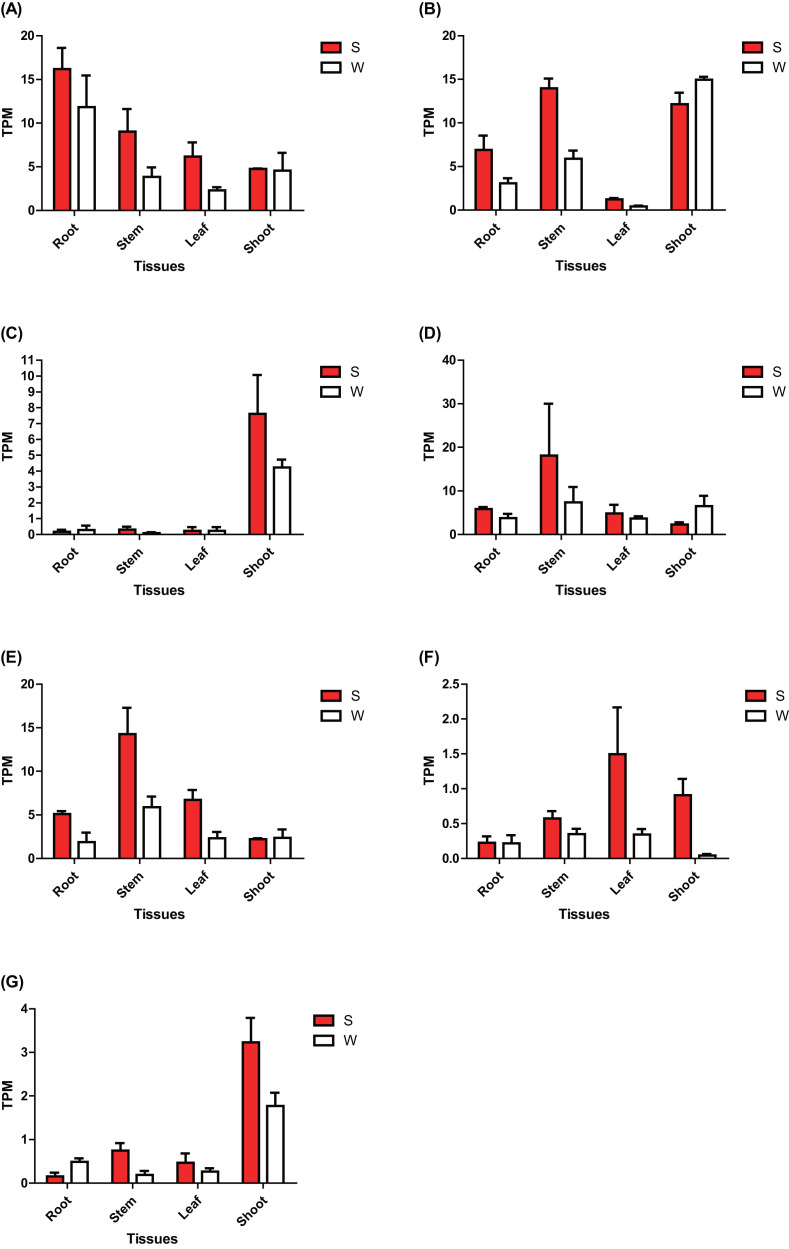
Expression analysis of seven *ERF*s under salt stress using RNA-Seq data. The expression is measured as TPM (Transcripts Per Million reads). Data represents Mean ± SD. S indicates treated with NaCl for 24 hours and W denotes the control (normal condition). (A) *PtERF001*, (B) *PtERF002*, (C) *PtERF003*, (D) *PtERF004*, (E) *PtERF005*, (F) *PtERF006*, (G) *PtERF007*.

**Figure 7:**
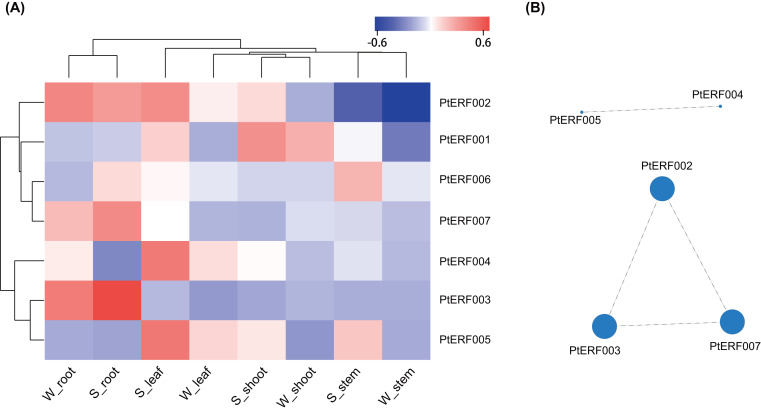
Heatmap clustering and correlations of the seven *ERF* genes based on RNA-Seq. (A) Heatmap clustering of the seven *ERF* genes. Each column represents a sample, and each row represents a gene. The color in the figure represents the expression level of the gene in the sample [log10(TPM+1)]. Red represents the high expression level of the gene in the sample, while blue represents the low expression level. On the left are the tree diagram of gene clustering and the module diagram of sub-clustering, and on the right are the names of genes. The upper part is the tree diagram of sample clustering, and the lower part is the name of samples. The closer the branches of the two samples are, the closer the expression patterns of all genes in the two samples are, that is, the closer the variation trend of gene expression is. (B) Visualization of inter-gene expression correlations. Each node in the figure represents a gene, and the internode line represents the correlation of gene expression. The larger the node, the more the number of expression correlations between this gene and other genes.

The expression of these seven *ERF* genes of *Populus* was also cross-validated using RNA-seq in this study. In root tissues, the expression (TPM) of *PtERF001*, *PtERF002*, *PtERF004*, and *PtERF005* was induced after exposure to salt stress, while *PtERF003* and *PtERF007* were suppressed and *PtERF006* showed no difference compared with the control (Fig. 6, Table S11). In stems, the expression patterns of these seven genes were respectively similar to those observed in the roots, with the exception of *PtERF003*, *PtERF006* and *PtERF007*. It is worth noting that all seven *ERF* genes were overexpressed in stems and leaves after treatment with salt stress; the expression of *PtERF007* in stems and *PtERF006* in leaves in particular was about 3.8 times and 4.4 times that of the control under normal conditions, respectively (Fig. 6, Table S11). In shoot tissues, *PtERF006* and *PtERF007* were significantly induced by salt stress, whereas *PtERF004* was repressed by this treatment (Fig. 6, Table S11). The remaining genes showed no significant differences in expression levels between the tested groups in shoots. Tissue-specific expression patterns were also identified using RNA-seq data. The tissues with high gene expression remained high after the stress treatment (Fig. 7A). In addition, the seven *ERF* genes could be divided into two subgroups based on their expression patterns: *PtERF003*, *PtERF004*, and *PtERF005* belonged to one subgroup, while the remaining four genes belonged to the other subgroup (Fig. 7A). The expression correlations detected among these seven genes showed that *PtERF004* was correlated with *PtERF005*, while *PtERF002*, *PtERF003*, and *PtERF007* were co-expressed significantly (Fig. 7B, Table S12).

## Discussion

Seven *ERF* genes of *Populus* were selected in the present study because they play potential roles in plant growth and stress response (Yao et al., 2019). A homology analysis showed that these seven genes have only five orthologs in *Arabidopsis thaliana*, which is always used as the model in plant physiology, biochemistry, and molecular biology studies (Table S1). The proportion (7/5) of *ERF* genes between *Populus* and *Arabidopsis* is consistent with the total number of *Populus ERF* genes, which is about 1.4–1.6-fold that of *Arabidopsis* (Zhuang et al., 2008). This increased number of genes in *Populus* indicated that plants in this genus have undergone gene duplication events during their evolutionary history (Tuskan et al., 2006; Lan et al., 2009; Wang et al., 2019). The synteny analysis showed that *PtERF001* was involved in the whole-genome duplications, while *PtERF002*, *PtERF004*, and *PtERF006* were related with tandem duplications (Fig. 2). As well known, the whole-genome duplication events along with the tandem duplication events has an important impact on the increase of ERF transcription factor genes of *Populus* in the long history of biological evolution (Kalluri et al., 2007; Lan et al., 2009; Wilkins et al., 2009).

The prediction of gene function based on the homology analysis indicated that these seven *ERF* genes mainly act as transcriptional activators and may bind to the pathogenesis-related GCC-box element in promoter region of the target genes (Table S1). Moreover, they may be involved in the stress response and signal transduction pathways by regulating gene expression. The phylogenetic relationship analysis divided these seven ERF TFs into three subfamilies based on the full-length protein sequence (Fig. 1). This was somewhat different from the classification used in a previous study, in which PtERF001 was located in a subfamily other than that of PtERF004 and PtERF005 (Yao et al., 2019). Multiple alignment showed that these seven ERF proteins have an AP2 domain and other conserved elements, such as YRG and RAYD elements (Fig. 1C). Proteins in the ERF-c subfamily contained a conserved 14th valine (V) residue and the conserved 14th V residue in the AP2 domain always determines the specificity of DNA-binding to DRE/GCC element (Sakuma et al., 2002; Djemal & Khoudi, 2015). GO annotation and enrichment also showed that these seven genes were enriched in DNA-binding transcription factor activity and transcription regulator activity (Fig. 3 and Fig. S1, Table S8). These findings suggest that the seven ERF TFs, at least the members of the ERF-c subfamily, possibly function in *Populus* stress tolerance.

The gene structure analysis showed that genes in the same subfamily had relatively similar and conserved intron/exon structure and motifs (Fig. 1B). Six conserved motifs among the seven *ERF* genes were identified in this study. The conserved motif-1 in the AP2 domain was present in all seven genes. Moreover, motif-3 and motif-5 were conserved in the ERF-a and ERF-c subfamilies, respectively. This result supports the reliability of the phylogenetic analysis and subfamily classification and suggests that a similar gene structure often represents a similar gene function (Wang et al., 2019). In addition to gene structure, putative *cis*-elements in the promoter region were investigated. Multiple stress-related elements were found that varied significantly in number and type among the different genes (Table S7). However, genes in the same subfamily had similar *cis*-elements, regardless of the dissimilarities between elements.

Some genes in the same subfamily showed similar expression changes in response to salt stress. For example, *PtERF003* and *PtERF006* of subfamily ERF-a were both suppressed in roots after treatment with salt stress (Fig. 4). Nevertheless, most of the genes exhibited a specific spatio-temporal expression pattern. Studies have shown that overexpression of *PtERF004* or *PtERF007* improved the salinity tolerance of transgenic plants (Yao et al., 2016a; Yao et al., 2016b; Cheng et al., 2019). Under normal conditions, five *ERFs* except *PtERF001* and *PtERF002* showed similar tissue-specific expression patterns with highest expression in roots (Fig. 5). It is reported that *ERF38* of poplar is mainly expressed in leaves and stems but roots and the expression reach the peak after 12 h under salt stress condition (Cheng et al., 2019). On the contrary, *ERF3* and *AP37* of rice play function in the root tissues and promote crown root development as long as increase grain yield under drought stress conditions (Oh et al., 2009; Zhao et al., 2015). In *Populus*, *PtaERF003* is suggested playing important roles in adventitious and lateral root proliferation (Trupiano et al., 2013). In addition, RNA-seq was used to verify the expression of the seven genes. A correlation analysis of expression indicated that *PtERF004* and *PtERF005* were strongly correlated and that *PtERF002*, *PtERF003*, *and PtERF007* were co-expressed significantly (Fig. 7B). These results are somewhat consistent with the classification reported in this study and indicate that genes in same subfamily may have similar functions in the plant stress-response process. Generally, the expression patterns detected here in response to salt stress showed that these seven *ERF* genes participated in the salt-stress-response process. The molecular mechanism of the seven ERF TFs will be detected with the overexpression and inhibiting expression transgenic poplar in our following research work. We have been and will continue to monitor the molecular basis and functions of *Populus* ERF TFs.

## Conclusions

In conclusion, a systematic bioinformatics and expression-pattern analysis of seven stress-related *ERF* genes of *Populus* was performed in this study. Phylogenetic trees and multiple alignment of protein sequences indicated these seven ERFs belong to three subfamilies and carry AP2 conserved domains, thus possibly interacting with the downstream target genes to regulate the plant stress response. Gene structure and promoter analyses showed that the seven ERFs with stress-related motifs and *cis*-elements may play roles in the plant stress-tolerance process via a transcriptional regulation process; the enrichment in the cellular_component and molecular_function terms supported this hypothesis. In addition, the spatio-temporal expression pattern of these seven *ERF* genes suggests that they play a critical role in mediating salt response and tolerance in a dynamic and tissue-specific manner. These results indicate that the seven *ERF* genes have important functions in the salt stress endurance and tissue development of *Populus* plants.

##  Supplemental Information

10.7717/peerj.10206/supp-1 Supplemental Information 1 The information of 7 ERF TFs of *Populus*

10.7717/peerj.10206/supp-2 Supplemental Information 2 Primes for RTq-PCR**

10.7717/peerj.10206/supp-3 Supplemental Information 3 The detailed information of the 7 ERF TFs, including cDNA and amino acids sequences and physicochemical property

10.7717/peerj.10206/supp-4 Supplemental Information 4 Collinear gene of *Populus* detected by MCScanX using default parameters considering pBLAST ≤ 1e −5

10.7717/peerj.10206/supp-5 Supplemental Information 5 Collinear gene of *ERF* genes of *Populus* detected by MCScanX

10.7717/peerj.10206/supp-6 Supplemental Information 6 Tandem genes of *ERF* genes of* Populus* detected by MCScanX

10.7717/peerj.10206/supp-7 Supplemental Information 7 Abiotic stress and phytohormone related *cis*-elements in 7 *ERF* promoters detected using the PlantCRAE programme

10.7717/peerj.10206/supp-8 Supplemental Information 8 Statistical analysis of GO enrichment

10.7717/peerj.10206/supp-9 Supplemental Information 9 One-way ANOVA statistical analysis of gene expression among different time points

10.7717/peerj.10206/supp-10 Supplemental Information 10 One-way ANOVA statistical analysis of gene expression among different tissues

10.7717/peerj.10206/supp-11 Supplemental Information 11 Statistical analysis of RNA-seq data between the salt and control samples

10.7717/peerj.10206/supp-12 Supplemental Information 12 Express correlation analysis of 7 *ERF* genes based on RNA-seq data

10.7717/peerj.10206/supp-13 Supplemental Information 13 Directed acyclic graph of GO terms The colored box was the GO term with significant enrichment in the gene set. The closer the color is to red, the more significant it is. The connection between GO terms represents the relationship between two GO terms.

10.7717/peerj.10206/supp-14 Supplemental Information 14 Raw data of expression level of 7 ERF genes based on RNA-seq and gene annotation from different databases

10.7717/peerj.10206/supp-15 Supplemental Information 15 Raw data of gene count of RNA-seq

10.7717/peerj.10206/supp-16 Supplemental Information 16 Raw data of gene TPM of RNA-seq

10.7717/peerj.10206/supp-17 Supplemental Information 17 Detection of *cis*-elements in 7 *ERF* promoters using the PlantCRAE programme

## Additional Information and Declarations

### Data Availability

The following information was supplied regarding data availability: The gene expression level of the 7 ERFs based on RNA-seq data and gene annotation prediction from different databases are available in the Supplemental Files. The sequences are available from Phytozome: https://phytozome.jgi.doe.gov/pz/portal.html#!info?alias=Org_Ptrichocarpa.
