# Supplementary material for: Structure and expression analysis of seven salt-related ERF genes of Populus
Source: PeerJ. 2020 Oct 20;8:e10206. doi: 10.7717/peerj.10206 (PMC7583627; doi:10.7717/peerj.10206)
Supplement: Supplemental Information 1 [file peerj-08-10206-s001.docx]

**Table S1 The information of 7 ERF TFs of *Populus***

| **TF NO.** | ***Populus* ID** | ***Ortholog in Arabidopsis*** | | **Function** |
| --- | --- | --- | --- | --- |
|  |  | **ID** | ***Description*** |  |
| PthERF001 | Potri.003G139300.1 | [AT1G64380.1](http://planttfdb.cbi.pku.edu.cn/tf.php?sp=Ath&did=AT1G64380.1) | AtERF061 | Probably acts as a transcriptional activator. Binds to the GCC-box pathogenesis-related promoter element. May be involved in the regulation of gene expression by stress factors and by components of stress signal transduction pathways (By similarity). |
| PthERF002 | Potri.002G039100.1 | [AT3G23240.1](http://planttfdb.cbi.pku.edu.cn/tf.php?sp=Ath&did=AT3G23240.1) | AtERF1 | Acts as a transcriptional activator. Binds to the GCC-box pathogenesis-related promoter element. Involved in the regulation of gene expression during the plant development, and/or mediated by stress factors and by components of stress signal transduction pathways. Seems to be a key integrator of ethylene and jasmonate signals in the regulation of ethylene/jasmonate-dependent defenses. Can mediate resistance to necrotizing fungi (*Botrytis cinerea* and *Plectosphaerella cucumerina*) and to soil borne fungi (*Fusarium oxysporum* conglutinans and *Fusiarium oxysporum* lycopersici), but probably not to necrotizing bacteria (*Pseudomonas syringae* tomato). |
| PthERF003 | Potri.011G061700.1 |  |  |  |
| PthERF004 | Potri.006G138900.1 | [AT5G21960.1](http://planttfdb.cbi.pku.edu.cn/tf.php?sp=Ath&did=AT5G21960.1) | AtERF016 | Probably acts as a transcriptional activator. Binds to the GCC-box pathogenesis-related promoter element. May be involved in the regulation of gene expression by stress factors and by components of stress signal transduction pathways (By similarity). |
| PthERF005 | Potri.018G038100.1 |  |  |  |
| PthERF006 | Potri.004G051700.1 | [AT5G47220.1](http://planttfdb.cbi.pku.edu.cn/tf.php?sp=Ath&did=AT5G47220.1) | AtERF2 | Acts as a transcriptional activator. Binds to the GCC-box pathogenesis-related promoter element. Involved in the regulation of gene expression by stress factors and by components of stress signal transduction pathways. Involved in disease resistance pathways. |
| PthERF007 | Potri.005G195000.1 | [AT5G50080.1](http://planttfdb.cbi.pku.edu.cn/tf.php?sp=Ath&did=AT5G50080.1) | AtERF110 | Probably acts as a transcriptional activator. Binds to the GCC-box pathogenesis-related promoter element. May be involved in the regulation of gene expression by stress factors and by components of stress signal transduction pathways (By similarity). |

*Red represents stress response, purple represents hormone related.*
