# Supplementary material for: Structure and expression analysis of seven salt-related ERF genes of Populus
Source: PeerJ. 2020 Oct 20;8:e10206. doi: 10.7717/peerj.10206 (PMC7583627; doi:10.7717/peerj.10206)
Supplement: Supplemental Information 2 [file peerj-08-10206-s002.docx]

**Table S2 Primers for RTq-PCR**

| **Gene** | **Gene_ID** | ***Forward and reverse primers (5’-3’)*** | |
| --- | --- | --- | --- |
| PthERF001 | Potri.003G139300.1 | CACTCCAGCAACTATTTGGCTC | GCTTGAATCTTGGCATCCACAG |
| PthERF002 | Potri.002G039100.1 | GAACCTTTGATAGTGCAGAGGCG | CCCAAATCCTCCAAAACCACCAC |
| PthERF003 | Potri.011G061700.1 | TAGCAAAGAAGCATTACCGGGG | GATATAGCAGCCACCACTTCAG |
| PthERF004 | Potri.006G138900.1 | CCGCAGAGGAAGCAGCAAGAGC | GATTCCACAATCCTCTCTGCAG |
| PthERF005 | Potri.018G038100.1 | CAAGGGAGTGAGGATGAGAAAG | CGTTGGAAAATGAAACGTCGCC |
| PthERF006 | Potri.004G051700.1 | AAAGAAGCACTACAGAGGCGTG | TGTGATTCAGGTTGCGATGCTG |
| PthERF007 | Potri.005G195000.1 | GTCATCTGGTGCAACTGCAACTG | CCAGACTCTTGCTGCTTTGTGTG |
| Actin | JM986590 | ACCCTCCAATCCAGACACTG | TTGCTGACCGTATGAGCAAG |
| EF1 | FJ438462 | AAGCCATGGGATGATGAGAC | ACTGGAGCCAATTTTGATGC |
