# Supplementary figures and images for: Structure and expression analysis of seven salt-related ERF genes of Populus
Source: PeerJ. 2020 Oct 20;8:e10206. doi: 10.7717/peerj.10206 (PMC7583627; doi:10.7717/peerj.10206)

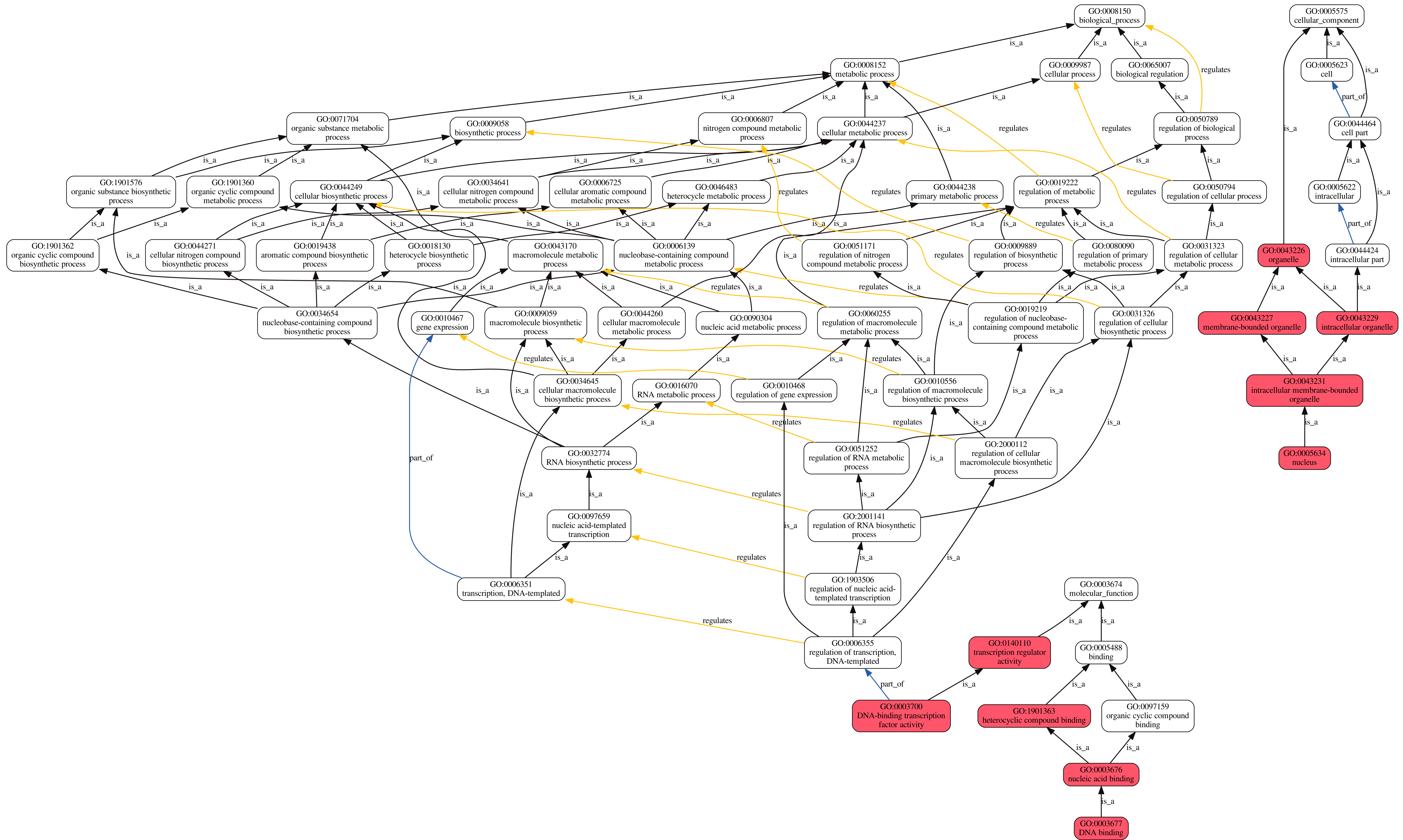

Supplement: Supplemental Information 13 — The colored box was the GO term with significant enrichment in the gene set. The closer the color is to red, the more significant it is. The connection between GO terms represents the relationship between two GO terms. [file peerj-08-10206-s013.pdf]
