# Supplementary material for: Structure and expression analysis of seven salt-related ERF genes of Populus
Source: PeerJ. 2020 Oct 20;8:e10206. doi: 10.7717/peerj.10206 (PMC7583627; doi:10.7717/peerj.10206)
Supplement: Supplemental Information 17 [file peerj-08-10206-s017.gz › Potri.002G039100.1_plantcare.html]

Content-Type: text/html; charset=ISO-8859-1


PlantCARE


Webmaster Firefox specific output  
To save the result:
click on the frame with the right mouse button and save the source code as a text file with extension .html  
REFERENCE:PlantCARE: a database of plant cis-acting regulatory elements and a portal to tools for in silico analysis of promoter sequences.  
Lescot, M., Déhais, P., Moreau, Y., De Moor, B., Rouzé ,P.,and Rombauts, S.  
Nucleic Acids Res., Database issue(2002), 30(1):325-327.   


---

>Potri.002G039100.1   
+ TTTTTTTTTA TCCGTAATTG ATTATTCAAA ACCTTTCCAG CTTAAAATTT TTTCCATAAT TTAAAAACTA   
  
  
+ TTAAAGGTTG AAAACACTTT TTTTTTCTCT GAGATTTAAA ACTAATACTA TAAAAACTCT AACTCTAACT   
  
  
+ TTCCGTTGTT TATTATTTAT CACCGAATCT TCTTGATAAA TGTATTTCTT AAAGTATATT TTATATAAAA   
  
  
+ ATATTTTTTT TATTTTTAAA ATTTTCCTTT AATATACATC ATGTAAAGTT TCAAATCGAT TTAAAAATTA   
  
  
+ AACATAATTT TTTTAAAAAA TTAACGGAAC CGCAAAAAAA TAAAGAGATG GTCGCCATCA ACCGTGTAGC   
  
  
+ AACCCTTTCT TTTCGGAGGA CTTTCAAAAT TCAGGCATTT CCATGGAGAT TTTTTGTCTT CTTGAAGACC   
  
  
+ GGGAACGGTT TCAAGCAATC CCACGAAAGG GGGCCTGAAT CTAGACAACG TTGCTATCAA ACAGCTTCAT   
  
  
+ CTGTGTTAAA TATTTTATGG ATTTGATTTA CCTGTCCCCT CTGCCAGTCT TTTCAAATCA AATTTGCCAC   
  
  
+ AAGAAACCAT GACTTTTATT TCTTTCATGT GCACATCATT ACCTTAAGTG ATAACGATTA AAGGTGGAGG   
  
  
+ TTTGCCTAAA ATTACCAGTT GATATTAAAT ACGCAACAAC TTAATTTCAC ATGAATCCTC ACCCGTATTA   
  
  
+ GCTAGGGTGC CAAACTCTCT TGATCACCAT CCGTGATTAA GCGGTGCGTG GTTTTATTGA TTAAAACTAA   
  
  
+ TGTGATTTAA ATCTGATTAT CCCCGAGTTA ATTATGAAGG CTCGGAACTC CATTTTCTTC TCCTTTTTTT   
  
  
+ CACTTTTTTC TTGGACAGGG GACGAAAGTT GGTTCATGTG GCATCAAAAA AGTAGTGTTT AATAATTTAG   
  
  
+ ATGCTTTTAA CATTCATTGG ACAAGACTAA AAAGATCAGA TTTCATAGCA AATCTATCAC TTAGTCGACA   
  
  
+ TAAATATGTA AAAAACTCGA GATTTTATTA ATGGGTCTTG AATTTAATTA AAACCTTTAA ATTAAATAAA   
  
  
+ TATTATTCTC GTCACGAGTC AATCTCTTGT AACATCGTCA TTTCTTTAAG ATCAGTGACC TTGTTGAAAA   
  
  
+ CTAAAAACAC ACACAATTTT AATCATTCTT CTCTTTTTCG TGTATTCAAC TCACTTAAAT TCAACATATA   
  
  
+ GATTGAAAGA TTACAAGTCT AGACCTATTA TAATTCAATA AAGATTCTAA TTAGCAAGTT GAATAAGTGT   
  
  
+ TCTAAACACC AATTACTTTT TTCATCGATG ATTTCTATTC AAACTACGAT GTAAACTAAT ATCTAACAAT   
  
  
+ TGTCTTGAAT TTCGAACAAA TAATCACAGC TGCAGCTTTC AAAACATTAC CCAGAACCAT TTTCCTTGAC   
  
  
+ TTCTTTTTTA TTTTCTTATG CTTGAAATAA TACAGAACAG ATTAACGAGT TCTGTACACT GAAAATGAAA   
  
  
+ CAAGACTTTG AACTTCTAAC TTGGTATATT CTATTTTGTT ATACCGTCCC ATCATGTTAA GACTGGAAAA   
  
  
+ CTAATTAAAA GGTATTTCAT GAGATTATGA TGCATAATTA TTTTTAAAAT AATTTTTTAT TTAAAAATAT   
  
  
+ ATTAAAATAA AATTTCTTAT TTATTTTTAA TATCAACAGA TTAAAACTAT CATAAATCAC TAAATAATTA   
  
  
+ TCAATTTAAT ATTTTTCAAA CCAAAAACAC ATTTAAAATA CACTTCAAAA CTGTGTCAAC TTGAAGCTCA   
  
  
+ AAATTCACAG AGAAGTTAAA AACATTTAAC TGTAAATGCG AAGCTACACT GTCCAAACCT TCCAGTGACA   
  
  
+ ATGAAAAGCT TGCCACTTTG TATTGAAATA TTGGTACCAC GTGTAAGACA TATGCGAACA AGGCAATGGT   
  
  
+ GACGTCGGCA AGGTATAGGC AATCGTGGGA CCAATCCCGC CGCCGTACGT CACTAAACAC ACGTGCCTTA   
  
  
+ AAAACAAATT CCCAAGCCTT CCTAGCAACA AAGAACAAG  

- AAAAAAAAAT AGGCATTAAC TAATAAGTTT TGGAAAGGTC GAATTTTAAA AAAGGTATTA AATTTTTGAT   
  
  
- AATTTCCAAC TTTTGTGAAA AAAAAAGAGA CTCTAAATTT TGATTATGAT ATTTTTGAGA TTGAGATTGA   
  
  
- AAGGCAACAA ATAATAAATA GTGGCTTAGA AGAACTATTT ACATAAAGAA TTTCATATAA AATATATTTT   
  
  
- TATAAAAAAA ATAAAAATTT TAAAAGGAAA TTATATGTAG TACATTTCAA AGTTTAGCTA AATTTTTAAT   
  
  
- TTGTATTAAA AAAATTTTTT AATTGCCTTG GCGTTTTTTT ATTTCTCTAC CAGCGGTAGT TGGCACATCG   
  
  
- TTGGGAAAGA AAAGCCTCCT GAAAGTTTTA AGTCCGTAAA GGTACCTCTA AAAAACAGAA GAACTTCTGG   
  
  
- CCCTTGCCAA AGTTCGTTAG GGTGCTTTCC CCCGGACTTA GATCTGTTGC AACGATAGTT TGTCGAAGTA   
  
  
- GACACAATTT ATAAAATACC TAAACTAAAT GGACAGGGGA GACGGTCAGA AAAGTTTAGT TTAAACGGTG   
  
  
- TTCTTTGGTA CTGAAAATAA AGAAAGTACA CGTGTAGTAA TGGAATTCAC TATTGCTAAT TTCCACCTCC   
  
  
- AAACGGATTT TAATGGTCAA CTATAATTTA TGCGTTGTTG AATTAAAGTG TACTTAGGAG TGGGCATAAT   
  
  
- CGATCCCACG GTTTGAGAGA ACTAGTGGTA GGCACTAATT CGCCACGCAC CAAAATAACT AATTTTGATT   
  
  
- ACACTAAATT TAGACTAATA GGGGCTCAAT TAATACTTCC GAGCCTTGAG GTAAAAGAAG AGGAAAAAAA   
  
  
- GTGAAAAAAG AACCTGTCCC CTGCTTTCAA CCAAGTACAC CGTAGTTTTT TCATCACAAA TTATTAAATC   
  
  
- TACGAAAATT GTAAGTAACC TGTTCTGATT TTTCTAGTCT AAAGTATCGT TTAGATAGTG AATCAGCTGT   
  
  
- ATTTATACAT TTTTTGAGCT CTAAAATAAT TACCCAGAAC TTAAATTAAT TTTGGAAATT TAATTTATTT   
  
  
- ATAATAAGAG CAGTGCTCAG TTAGAGAACA TTGTAGCAGT AAAGAAATTC TAGTCACTGG AACAACTTTT   
  
  
- GATTTTTGTG TGTGTTAAAA TTAGTAAGAA GAGAAAAAGC ACATAAGTTG AGTGAATTTA AGTTGTATAT   
  
  
- CTAACTTTCT AATGTTCAGA TCTGGATAAT ATTAAGTTAT TTCTAAGATT AATCGTTCAA CTTATTCACA   
  
  
- AGATTTGTGG TTAATGAAAA AAGTAGCTAC TAAAGATAAG TTTGATGCTA CATTTGATTA TAGATTGTTA   
  
  
- ACAGAACTTA AAGCTTGTTT ATTAGTGTCG ACGTCGAAAG TTTTGTAATG GGTCTTGGTA AAAGGAACTG   
  
  
- AAGAAAAAAT AAAAGAATAC GAACTTTATT ATGTCTTGTC TAATTGCTCA AGACATGTGA CTTTTACTTT   
  
  
- GTTCTGAAAC TTGAAGATTG AACCATATAA GATAAAACAA TATGGCAGGG TAGTACAATT CTGACCTTTT   
  
  
- GATTAATTTT CCATAAAGTA CTCTAATACT ACGTATTAAT AAAAATTTTA TTAAAAAATA AATTTTTATA   
  
  
- TAATTTTATT TTAAAGAATA AATAAAAATT ATAGTTGTCT AATTTTGATA GTATTTAGTG ATTTATTAAT   
  
  
- AGTTAAATTA TAAAAAGTTT GGTTTTTGTG TAAATTTTAT GTGAAGTTTT GACACAGTTG AACTTCGAGT   
  
  
- TTTAAGTGTC TCTTCAATTT TTGTAAATTG ACATTTACGC TTCGATGTGA CAGGTTTGGA AGGTCACTGT   
  
  
- TACTTTTCGA ACGGTGAAAC ATAACTTTAT AACCATGGTG CACATTCTGT ATACGCTTGT TCCGTTACCA   
  
  
- CTGCAGCCGT TCCATATCCG TTAGCACCCT GGTTAGGGCG GCGGCATGCA GTGATTTGTG TGCACGGAAT   
  
  
- TTTTGTTTAA GGGTTCGGAA GGATCGTTGT TTCTTGTTC

  
  
Motifs Found  

+   

| Site Name | Organism | Position | Strand | Matrix score. | sequence | function |
| --- | --- | --- | --- | --- | --- | --- |
|  | organism | 167 | + | 4 | motif\_sequence | short\_function |
|  | organism | 853 | - | 4 | motif\_sequence | short\_function |
|  | organism | 587 | + | 4 | motif\_sequence | short\_function |
|  | organism | 1452 | + | 4 | motif\_sequence | short\_function |
|  | organism | 522 | + | 4 | motif\_sequence | short\_function |
|  | organism | 1435 | - | 4 | motif\_sequence | short\_function |
|  | organism | 591 | - | 4 | motif\_sequence | short\_function |
|  | organism | 1799 | + | 4 | motif\_sequence | short\_function |
|  | organism | 395 | - | 4 | motif\_sequence | short\_function |
|  | organism | 529 | + | 4 | motif\_sequence | short\_function |

>Potri.002G039100.1   
+ TTTTTTTTTA TCCGTAATTG ATTATTCAAA ACCTTTCCAG CTTAAAATTT TTTCCATAAT TTAAAAACTA   
  
  
+ TTAAAGGTTG AAAACACTTT TTTTTTCTCT GAGATTTAAA ACTAATACTA TAAAAACTCT AACTCTAACT   
  
  
+ TTCCGTTGTT TATTATTTAT CACCGAATCT TCTTGATAAA TGTATTTCTT AAAGTATATT TTATATAAAA   
  
  
+ ATATTTTTTT TATTTTTAAA ATTTTCCTTT AATATACATC ATGTAAAGTT TCAAATCGAT TTAAAAATTA   
  
  
+ AACATAATTT TTTTAAAAAA TTAACGGAAC CGCAAAAAAA TAAAGAGATG GTCGCCATCA ACCGTGTAGC   
  
  
+ AACCCTTTCT TTTCGGAGGA CTTTCAAAAT TCAGGCATTT CCATGGAGAT TTTTTGTCTT CTTGAAGACC   
  
  
+ GGGAACGGTT TCAAGCAATC CCACGAAAGG GGGCCTGAAT CTAGACAACG TTGCTATCAA ACAGCTTCAT   
  
  
+ CTGTGTTAAA TATTTTATGG ATTTGATTTA CCTGTCCCCT CTGCCAGTCT TTTCAAATCA AATTTGCCAC   
  
  
+ AAGAAACCAT GACTTTTATT TCTTTCATGT GCACATCATT ACCTTAAGTG ATAACGATTA AAGGTGGAGG   
  
  
+ TTTGCCTAAA ATTACCAGTT GATATTAAAT ACGCAACAAC TTAATTTCAC ATGAATCCTC ACCCGTATTA   
  
  
+ GCTAGGGTGC CAAACTCTCT TGATCACCAT CCGTGATTAA GCGGTGCGTG GTTTTATTGA TTAAAACTAA   
  
  
+ TGTGATTTAA ATCTGATTAT CCCCGAGTTA ATTATGAAGG CTCGGAACTC CATTTTCTTC TCCTTTTTTT   
  
  
+ CACTTTTTTC TTGGACAGGG GACGAAAGTT GGTTCATGTG GCATCAAAAA AGTAGTGTTT AATAATTTAG   
  
  
+ ATGCTTTTAA CATTCATTGG ACAAGACTAA AAAGATCAGA TTTCATAGCA AATCTATCAC TTAGTCGACA   
  
  
+ TAAATATGTA AAAAACTCGA GATTTTATTA ATGGGTCTTG AATTTAATTA AAACCTTTAA ATTAAATAAA   
  
  
+ TATTATTCTC GTCACGAGTC AATCTCTTGT AACATCGTCA TTTCTTTAAG ATCAGTGACC TTGTTGAAAA   
  
  
+ CTAAAAACAC ACACAATTTT AATCATTCTT CTCTTTTTCG TGTATTCAAC TCACTTAAAT TCAACATATA   
  
  
+ GATTGAAAGA TTACAAGTCT AGACCTATTA TAATTCAATA AAGATTCTAA TTAGCAAGTT GAATAAGTGT   
  
  
+ TCTAAACACC AATTACTTTT TTCATCGATG ATTTCTATTC AAACTACGAT GTAAACTAAT ATCTAACAAT   
  
  
+ TGTCTTGAAT TTCGAACAAA TAATCACAGC TGCAGCTTTC AAAACATTAC CCAGAACCAT TTTCCTTGAC   
  
  
+ TTCTTTTTTA TTTTCTTATG CTTGAAATAA TACAGAACAG ATTAACGAGT TCTGTACACT GAAAATGAAA   
  
  
+ CAAGACTTTG AACTTCTAAC TTGGTATATT CTATTTTGTT ATACCGTCCC ATCATGTTAA GACTGGAAAA   
  
  
+ CTAATTAAAA GGTATTTCAT GAGATTATGA TGCATAATTA TTTTTAAAAT AATTTTTTAT TTAAAAATAT   
  
  
+ ATTAAAATAA AATTTCTTAT TTATTTTTAA TATCAACAGA TTAAAACTAT CATAAATCAC TAAATAATTA   
  
  
+ TCAATTTAAT ATTTTTCAAA CCAAAAACAC ATTTAAAATA CACTTCAAAA CTGTGTCAAC TTGAAGCTCA   
  
  
+ AAATTCACAG AGAAGTTAAA AACATTTAAC TGTAAATGCG AAGCTACACT GTCCAAACCT TCCAGTGACA   
  
  
+ ATGAAAAGCT TGCCACTTTG TATTGAAATA TTGGTACCAC GTGTAAGACA TATGCGAACA AGGCAATGGT   
  
  
+ GACGTCGGCA AGGTATAGGC AATCGTGGGA CCAATCCCGC CGCCGTACGT CACTAAACAC ACGTGCCTTA   
  
  
+ AAAACAAATT CCCAAGCCTT CCTAGCAACA AAGAACAAG  

- AAAAAAAAAT AGGCATTAAC TAATAAGTTT TGGAAAGGTC GAATTTTAAA AAAGGTATTA AATTTTTGAT   
  
  
- AATTTCCAAC TTTTGTGAAA AAAAAAGAGA CTCTAAATTT TGATTATGAT ATTTTTGAGA TTGAGATTGA   
  
  
- AAGGCAACAA ATAATAAATA GTGGCTTAGA AGAACTATTT ACATAAAGAA TTTCATATAA AATATATTTT   
  
  
- TATAAAAAAA ATAAAAATTT TAAAAGGAAA TTATATGTAG TACATTTCAA AGTTTAGCTA AATTTTTAAT   
  
  
- TTGTATTAAA AAAATTTTTT AATTGCCTTG GCGTTTTTTT ATTTCTCTAC CAGCGGTAGT TGGCACATCG   
  
  
- TTGGGAAAGA AAAGCCTCCT GAAAGTTTTA AGTCCGTAAA GGTACCTCTA AAAAACAGAA GAACTTCTGG   
  
  
- CCCTTGCCAA AGTTCGTTAG GGTGCTTTCC CCCGGACTTA GATCTGTTGC AACGATAGTT TGTCGAAGTA   
  
  
- GACACAATTT ATAAAATACC TAAACTAAAT GGACAGGGGA GACGGTCAGA AAAGTTTAGT TTAAACGGTG   
  
  
- TTCTTTGGTA CTGAAAATAA AGAAAGTACA CGTGTAGTAA TGGAATTCAC TATTGCTAAT TTCCACCTCC   
  
  
- AAACGGATTT TAATGGTCAA CTATAATTTA TGCGTTGTTG AATTAAAGTG TACTTAGGAG TGGGCATAAT   
  
  
- CGATCCCACG GTTTGAGAGA ACTAGTGGTA GGCACTAATT CGCCACGCAC CAAAATAACT AATTTTGATT   
  
  
- ACACTAAATT TAGACTAATA GGGGCTCAAT TAATACTTCC GAGCCTTGAG GTAAAAGAAG AGGAAAAAAA   
  
  
- GTGAAAAAAG AACCTGTCCC CTGCTTTCAA CCAAGTACAC CGTAGTTTTT TCATCACAAA TTATTAAATC   
  
  
- TACGAAAATT GTAAGTAACC TGTTCTGATT TTTCTAGTCT AAAGTATCGT TTAGATAGTG AATCAGCTGT   
  
  
- ATTTATACAT TTTTTGAGCT CTAAAATAAT TACCCAGAAC TTAAATTAAT TTTGGAAATT TAATTTATTT   
  
  
- ATAATAAGAG CAGTGCTCAG TTAGAGAACA TTGTAGCAGT AAAGAAATTC TAGTCACTGG AACAACTTTT   
  
  
- GATTTTTGTG TGTGTTAAAA TTAGTAAGAA GAGAAAAAGC ACATAAGTTG AGTGAATTTA AGTTGTATAT   
  
  
- CTAACTTTCT AATGTTCAGA TCTGGATAAT ATTAAGTTAT TTCTAAGATT AATCGTTCAA CTTATTCACA   
  
  
- AGATTTGTGG TTAATGAAAA AAGTAGCTAC TAAAGATAAG TTTGATGCTA CATTTGATTA TAGATTGTTA   
  
  
- ACAGAACTTA AAGCTTGTTT ATTAGTGTCG ACGTCGAAAG TTTTGTAATG GGTCTTGGTA AAAGGAACTG   
  
  
- AAGAAAAAAT AAAAGAATAC GAACTTTATT ATGTCTTGTC TAATTGCTCA AGACATGTGA CTTTTACTTT   
  
  
- GTTCTGAAAC TTGAAGATTG AACCATATAA GATAAAACAA TATGGCAGGG TAGTACAATT CTGACCTTTT   
  
  
- GATTAATTTT CCATAAAGTA CTCTAATACT ACGTATTAAT AAAAATTTTA TTAAAAAATA AATTTTTATA   
  
  
- TAATTTTATT TTAAAGAATA AATAAAAATT ATAGTTGTCT AATTTTGATA GTATTTAGTG ATTTATTAAT   
  
  
- AGTTAAATTA TAAAAAGTTT GGTTTTTGTG TAAATTTTAT GTGAAGTTTT GACACAGTTG AACTTCGAGT   
  
  
- TTTAAGTGTC TCTTCAATTT TTGTAAATTG ACATTTACGC TTCGATGTGA CAGGTTTGGA AGGTCACTGT   
  
  
- TACTTTTCGA ACGGTGAAAC ATAACTTTAT AACCATGGTG CACATTCTGT ATACGCTTGT TCCGTTACCA   
  
  
- CTGCAGCCGT TCCATATCCG TTAGCACCCT GGTTAGGGCG GCGGCATGCA GTGATTTGTG TGCACGGAAT   
  
  
- TTTTGTTTAA GGGTTCGGAA GGATCGTTGT TTCTTGTTC

+     A-box

| Site Name | Organism | Position | Strand | Matrix score. | sequence | function |
| --- | --- | --- | --- | --- | --- | --- |
| A-box | Petroselinum crispum | 1514 | + | 6 | CCGTCC | cis-acting regulatory element |

>Potri.002G039100.1   
+ TTTTTTTTTA TCCGTAATTG ATTATTCAAA ACCTTTCCAG CTTAAAATTT TTTCCATAAT TTAAAAACTA   
  
  
+ TTAAAGGTTG AAAACACTTT TTTTTTCTCT GAGATTTAAA ACTAATACTA TAAAAACTCT AACTCTAACT   
  
  
+ TTCCGTTGTT TATTATTTAT CACCGAATCT TCTTGATAAA TGTATTTCTT AAAGTATATT TTATATAAAA   
  
  
+ ATATTTTTTT TATTTTTAAA ATTTTCCTTT AATATACATC ATGTAAAGTT TCAAATCGAT TTAAAAATTA   
  
  
+ AACATAATTT TTTTAAAAAA TTAACGGAAC CGCAAAAAAA TAAAGAGATG GTCGCCATCA ACCGTGTAGC   
  
  
+ AACCCTTTCT TTTCGGAGGA CTTTCAAAAT TCAGGCATTT CCATGGAGAT TTTTTGTCTT CTTGAAGACC   
  
  
+ GGGAACGGTT TCAAGCAATC CCACGAAAGG GGGCCTGAAT CTAGACAACG TTGCTATCAA ACAGCTTCAT   
  
  
+ CTGTGTTAAA TATTTTATGG ATTTGATTTA CCTGTCCCCT CTGCCAGTCT TTTCAAATCA AATTTGCCAC   
  
  
+ AAGAAACCAT GACTTTTATT TCTTTCATGT GCACATCATT ACCTTAAGTG ATAACGATTA AAGGTGGAGG   
  
  
+ TTTGCCTAAA ATTACCAGTT GATATTAAAT ACGCAACAAC TTAATTTCAC ATGAATCCTC ACCCGTATTA   
  
  
+ GCTAGGGTGC CAAACTCTCT TGATCACCAT CCGTGATTAA GCGGTGCGTG GTTTTATTGA TTAAAACTAA   
  
  
+ TGTGATTTAA ATCTGATTAT CCCCGAGTTA ATTATGAAGG CTCGGAACTC CATTTTCTTC TCCTTTTTTT   
  
  
+ CACTTTTTTC TTGGACAGGG GACGAAAGTT GGTTCATGTG GCATCAAAAA AGTAGTGTTT AATAATTTAG   
  
  
+ ATGCTTTTAA CATTCATTGG ACAAGACTAA AAAGATCAGA TTTCATAGCA AATCTATCAC TTAGTCGACA   
  
  
+ TAAATATGTA AAAAACTCGA GATTTTATTA ATGGGTCTTG AATTTAATTA AAACCTTTAA ATTAAATAAA   
  
  
+ TATTATTCTC GTCACGAGTC AATCTCTTGT AACATCGTCA TTTCTTTAAG ATCAGTGACC TTGTTGAAAA   
  
  
+ CTAAAAACAC ACACAATTTT AATCATTCTT CTCTTTTTCG TGTATTCAAC TCACTTAAAT TCAACATATA   
  
  
+ GATTGAAAGA TTACAAGTCT AGACCTATTA TAATTCAATA AAGATTCTAA TTAGCAAGTT GAATAAGTGT   
  
  
+ TCTAAACACC AATTACTTTT TTCATCGATG ATTTCTATTC AAACTACGAT GTAAACTAAT ATCTAACAAT   
  
  
+ TGTCTTGAAT TTCGAACAAA TAATCACAGC TGCAGCTTTC AAAACATTAC CCAGAACCAT TTTCCTTGAC   
  
  
+ TTCTTTTTTA TTTTCTTATG CTTGAAATAA TACAGAACAG ATTAACGAGT TCTGTACACT GAAAATGAAA   
  
  
+ CAAGACTTTG AACTTCTAAC TTGGTATATT CTATTTTGTT ATACCGTCCC ATCATGTTAA GACTGGAAAA   
  
  
+ CTAATTAAAA GGTATTTCAT GAGATTATGA TGCATAATTA TTTTTAAAAT AATTTTTTAT TTAAAAATAT   
  
  
+ ATTAAAATAA AATTTCTTAT TTATTTTTAA TATCAACAGA TTAAAACTAT CATAAATCAC TAAATAATTA   
  
  
+ TCAATTTAAT ATTTTTCAAA CCAAAAACAC ATTTAAAATA CACTTCAAAA CTGTGTCAAC TTGAAGCTCA   
  
  
+ AAATTCACAG AGAAGTTAAA AACATTTAAC TGTAAATGCG AAGCTACACT GTCCAAACCT TCCAGTGACA   
  
  
+ ATGAAAAGCT TGCCACTTTG TATTGAAATA TTGGTACCAC GTGTAAGACA TATGCGAACA AGGCAATGGT   
  
  
+ GACGTCGGCA AGGTATAGGC AATCGTGGGA CCAATCCCGC CGCCGTACGT CACTAAACAC ACGTGCCTTA   
  
  
+ AAAACAAATT CCCAAGCCTT CCTAGCAACA AAGAACAAG  

- AAAAAAAAAT AGGCATTAAC TAATAAGTTT TGGAAAGGTC GAATTTTAAA AAAGGTATTA AATTTTTGAT   
  
  
- AATTTCCAAC TTTTGTGAAA AAAAAAGAGA CTCTAAATTT TGATTATGAT ATTTTTGAGA TTGAGATTGA   
  
  
- AAGGCAACAA ATAATAAATA GTGGCTTAGA AGAACTATTT ACATAAAGAA TTTCATATAA AATATATTTT   
  
  
- TATAAAAAAA ATAAAAATTT TAAAAGGAAA TTATATGTAG TACATTTCAA AGTTTAGCTA AATTTTTAAT   
  
  
- TTGTATTAAA AAAATTTTTT AATTGCCTTG GCGTTTTTTT ATTTCTCTAC CAGCGGTAGT TGGCACATCG   
  
  
- TTGGGAAAGA AAAGCCTCCT GAAAGTTTTA AGTCCGTAAA GGTACCTCTA AAAAACAGAA GAACTTCTGG   
  
  
- CCCTTGCCAA AGTTCGTTAG GGTGCTTTCC CCCGGACTTA GATCTGTTGC AACGATAGTT TGTCGAAGTA   
  
  
- GACACAATTT ATAAAATACC TAAACTAAAT GGACAGGGGA GACGGTCAGA AAAGTTTAGT TTAAACGGTG   
  
  
- TTCTTTGGTA CTGAAAATAA AGAAAGTACA CGTGTAGTAA TGGAATTCAC TATTGCTAAT TTCCACCTCC   
  
  
- AAACGGATTT TAATGGTCAA CTATAATTTA TGCGTTGTTG AATTAAAGTG TACTTAGGAG TGGGCATAAT   
  
  
- CGATCCCACG GTTTGAGAGA ACTAGTGGTA GGCACTAATT CGCCACGCAC CAAAATAACT AATTTTGATT   
  
  
- ACACTAAATT TAGACTAATA GGGGCTCAAT TAATACTTCC GAGCCTTGAG GTAAAAGAAG AGGAAAAAAA   
  
  
- GTGAAAAAAG AACCTGTCCC CTGCTTTCAA CCAAGTACAC CGTAGTTTTT TCATCACAAA TTATTAAATC   
  
  
- TACGAAAATT GTAAGTAACC TGTTCTGATT TTTCTAGTCT AAAGTATCGT TTAGATAGTG AATCAGCTGT   
  
  
- ATTTATACAT TTTTTGAGCT CTAAAATAAT TACCCAGAAC TTAAATTAAT TTTGGAAATT TAATTTATTT   
  
  
- ATAATAAGAG CAGTGCTCAG TTAGAGAACA TTGTAGCAGT AAAGAAATTC TAGTCACTGG AACAACTTTT   
  
  
- GATTTTTGTG TGTGTTAAAA TTAGTAAGAA GAGAAAAAGC ACATAAGTTG AGTGAATTTA AGTTGTATAT   
  
  
- CTAACTTTCT AATGTTCAGA TCTGGATAAT ATTAAGTTAT TTCTAAGATT AATCGTTCAA CTTATTCACA   
  
  
- AGATTTGTGG TTAATGAAAA AAGTAGCTAC TAAAGATAAG TTTGATGCTA CATTTGATTA TAGATTGTTA   
  
  
- ACAGAACTTA AAGCTTGTTT ATTAGTGTCG ACGTCGAAAG TTTTGTAATG GGTCTTGGTA AAAGGAACTG   
  
  
- AAGAAAAAAT AAAAGAATAC GAACTTTATT ATGTCTTGTC TAATTGCTCA AGACATGTGA CTTTTACTTT   
  
  
- GTTCTGAAAC TTGAAGATTG AACCATATAA GATAAAACAA TATGGCAGGG TAGTACAATT CTGACCTTTT   
  
  
- GATTAATTTT CCATAAAGTA CTCTAATACT ACGTATTAAT AAAAATTTTA TTAAAAAATA AATTTTTATA   
  
  
- TAATTTTATT TTAAAGAATA AATAAAAATT ATAGTTGTCT AATTTTGATA GTATTTAGTG ATTTATTAAT   
  
  
- AGTTAAATTA TAAAAAGTTT GGTTTTTGTG TAAATTTTAT GTGAAGTTTT GACACAGTTG AACTTCGAGT   
  
  
- TTTAAGTGTC TCTTCAATTT TTGTAAATTG ACATTTACGC TTCGATGTGA CAGGTTTGGA AGGTCACTGT   
  
  
- TACTTTTCGA ACGGTGAAAC ATAACTTTAT AACCATGGTG CACATTCTGT ATACGCTTGT TCCGTTACCA   
  
  
- CTGCAGCCGT TCCATATCCG TTAGCACCCT GGTTAGGGCG GCGGCATGCA GTGATTTGTG TGCACGGAAT   
  
  
- TTTTGTTTAA GGGTTCGGAA GGATCGTTGT TTCTTGTTC

+     AAGAA-motif

| Site Name | Organism | Position | Strand | Matrix score. | sequence | function |
| --- | --- | --- | --- | --- | --- | --- |
| AAGAA-motif | Avena sativa | 580 | - | 7 | GAAAGAA |  |

>Potri.002G039100.1   
+ TTTTTTTTTA TCCGTAATTG ATTATTCAAA ACCTTTCCAG CTTAAAATTT TTTCCATAAT TTAAAAACTA   
  
  
+ TTAAAGGTTG AAAACACTTT TTTTTTCTCT GAGATTTAAA ACTAATACTA TAAAAACTCT AACTCTAACT   
  
  
+ TTCCGTTGTT TATTATTTAT CACCGAATCT TCTTGATAAA TGTATTTCTT AAAGTATATT TTATATAAAA   
  
  
+ ATATTTTTTT TATTTTTAAA ATTTTCCTTT AATATACATC ATGTAAAGTT TCAAATCGAT TTAAAAATTA   
  
  
+ AACATAATTT TTTTAAAAAA TTAACGGAAC CGCAAAAAAA TAAAGAGATG GTCGCCATCA ACCGTGTAGC   
  
  
+ AACCCTTTCT TTTCGGAGGA CTTTCAAAAT TCAGGCATTT CCATGGAGAT TTTTTGTCTT CTTGAAGACC   
  
  
+ GGGAACGGTT TCAAGCAATC CCACGAAAGG GGGCCTGAAT CTAGACAACG TTGCTATCAA ACAGCTTCAT   
  
  
+ CTGTGTTAAA TATTTTATGG ATTTGATTTA CCTGTCCCCT CTGCCAGTCT TTTCAAATCA AATTTGCCAC   
  
  
+ AAGAAACCAT GACTTTTATT TCTTTCATGT GCACATCATT ACCTTAAGTG ATAACGATTA AAGGTGGAGG   
  
  
+ TTTGCCTAAA ATTACCAGTT GATATTAAAT ACGCAACAAC TTAATTTCAC ATGAATCCTC ACCCGTATTA   
  
  
+ GCTAGGGTGC CAAACTCTCT TGATCACCAT CCGTGATTAA GCGGTGCGTG GTTTTATTGA TTAAAACTAA   
  
  
+ TGTGATTTAA ATCTGATTAT CCCCGAGTTA ATTATGAAGG CTCGGAACTC CATTTTCTTC TCCTTTTTTT   
  
  
+ CACTTTTTTC TTGGACAGGG GACGAAAGTT GGTTCATGTG GCATCAAAAA AGTAGTGTTT AATAATTTAG   
  
  
+ ATGCTTTTAA CATTCATTGG ACAAGACTAA AAAGATCAGA TTTCATAGCA AATCTATCAC TTAGTCGACA   
  
  
+ TAAATATGTA AAAAACTCGA GATTTTATTA ATGGGTCTTG AATTTAATTA AAACCTTTAA ATTAAATAAA   
  
  
+ TATTATTCTC GTCACGAGTC AATCTCTTGT AACATCGTCA TTTCTTTAAG ATCAGTGACC TTGTTGAAAA   
  
  
+ CTAAAAACAC ACACAATTTT AATCATTCTT CTCTTTTTCG TGTATTCAAC TCACTTAAAT TCAACATATA   
  
  
+ GATTGAAAGA TTACAAGTCT AGACCTATTA TAATTCAATA AAGATTCTAA TTAGCAAGTT GAATAAGTGT   
  
  
+ TCTAAACACC AATTACTTTT TTCATCGATG ATTTCTATTC AAACTACGAT GTAAACTAAT ATCTAACAAT   
  
  
+ TGTCTTGAAT TTCGAACAAA TAATCACAGC TGCAGCTTTC AAAACATTAC CCAGAACCAT TTTCCTTGAC   
  
  
+ TTCTTTTTTA TTTTCTTATG CTTGAAATAA TACAGAACAG ATTAACGAGT TCTGTACACT GAAAATGAAA   
  
  
+ CAAGACTTTG AACTTCTAAC TTGGTATATT CTATTTTGTT ATACCGTCCC ATCATGTTAA GACTGGAAAA   
  
  
+ CTAATTAAAA GGTATTTCAT GAGATTATGA TGCATAATTA TTTTTAAAAT AATTTTTTAT TTAAAAATAT   
  
  
+ ATTAAAATAA AATTTCTTAT TTATTTTTAA TATCAACAGA TTAAAACTAT CATAAATCAC TAAATAATTA   
  
  
+ TCAATTTAAT ATTTTTCAAA CCAAAAACAC ATTTAAAATA CACTTCAAAA CTGTGTCAAC TTGAAGCTCA   
  
  
+ AAATTCACAG AGAAGTTAAA AACATTTAAC TGTAAATGCG AAGCTACACT GTCCAAACCT TCCAGTGACA   
  
  
+ ATGAAAAGCT TGCCACTTTG TATTGAAATA TTGGTACCAC GTGTAAGACA TATGCGAACA AGGCAATGGT   
  
  
+ GACGTCGGCA AGGTATAGGC AATCGTGGGA CCAATCCCGC CGCCGTACGT CACTAAACAC ACGTGCCTTA   
  
  
+ AAAACAAATT CCCAAGCCTT CCTAGCAACA AAGAACAAG  

- AAAAAAAAAT AGGCATTAAC TAATAAGTTT TGGAAAGGTC GAATTTTAAA AAAGGTATTA AATTTTTGAT   
  
  
- AATTTCCAAC TTTTGTGAAA AAAAAAGAGA CTCTAAATTT TGATTATGAT ATTTTTGAGA TTGAGATTGA   
  
  
- AAGGCAACAA ATAATAAATA GTGGCTTAGA AGAACTATTT ACATAAAGAA TTTCATATAA AATATATTTT   
  
  
- TATAAAAAAA ATAAAAATTT TAAAAGGAAA TTATATGTAG TACATTTCAA AGTTTAGCTA AATTTTTAAT   
  
  
- TTGTATTAAA AAAATTTTTT AATTGCCTTG GCGTTTTTTT ATTTCTCTAC CAGCGGTAGT TGGCACATCG   
  
  
- TTGGGAAAGA AAAGCCTCCT GAAAGTTTTA AGTCCGTAAA GGTACCTCTA AAAAACAGAA GAACTTCTGG   
  
  
- CCCTTGCCAA AGTTCGTTAG GGTGCTTTCC CCCGGACTTA GATCTGTTGC AACGATAGTT TGTCGAAGTA   
  
  
- GACACAATTT ATAAAATACC TAAACTAAAT GGACAGGGGA GACGGTCAGA AAAGTTTAGT TTAAACGGTG   
  
  
- TTCTTTGGTA CTGAAAATAA AGAAAGTACA CGTGTAGTAA TGGAATTCAC TATTGCTAAT TTCCACCTCC   
  
  
- AAACGGATTT TAATGGTCAA CTATAATTTA TGCGTTGTTG AATTAAAGTG TACTTAGGAG TGGGCATAAT   
  
  
- CGATCCCACG GTTTGAGAGA ACTAGTGGTA GGCACTAATT CGCCACGCAC CAAAATAACT AATTTTGATT   
  
  
- ACACTAAATT TAGACTAATA GGGGCTCAAT TAATACTTCC GAGCCTTGAG GTAAAAGAAG AGGAAAAAAA   
  
  
- GTGAAAAAAG AACCTGTCCC CTGCTTTCAA CCAAGTACAC CGTAGTTTTT TCATCACAAA TTATTAAATC   
  
  
- TACGAAAATT GTAAGTAACC TGTTCTGATT TTTCTAGTCT AAAGTATCGT TTAGATAGTG AATCAGCTGT   
  
  
- ATTTATACAT TTTTTGAGCT CTAAAATAAT TACCCAGAAC TTAAATTAAT TTTGGAAATT TAATTTATTT   
  
  
- ATAATAAGAG CAGTGCTCAG TTAGAGAACA TTGTAGCAGT AAAGAAATTC TAGTCACTGG AACAACTTTT   
  
  
- GATTTTTGTG TGTGTTAAAA TTAGTAAGAA GAGAAAAAGC ACATAAGTTG AGTGAATTTA AGTTGTATAT   
  
  
- CTAACTTTCT AATGTTCAGA TCTGGATAAT ATTAAGTTAT TTCTAAGATT AATCGTTCAA CTTATTCACA   
  
  
- AGATTTGTGG TTAATGAAAA AAGTAGCTAC TAAAGATAAG TTTGATGCTA CATTTGATTA TAGATTGTTA   
  
  
- ACAGAACTTA AAGCTTGTTT ATTAGTGTCG ACGTCGAAAG TTTTGTAATG GGTCTTGGTA AAAGGAACTG   
  
  
- AAGAAAAAAT AAAAGAATAC GAACTTTATT ATGTCTTGTC TAATTGCTCA AGACATGTGA CTTTTACTTT   
  
  
- GTTCTGAAAC TTGAAGATTG AACCATATAA GATAAAACAA TATGGCAGGG TAGTACAATT CTGACCTTTT   
  
  
- GATTAATTTT CCATAAAGTA CTCTAATACT ACGTATTAAT AAAAATTTTA TTAAAAAATA AATTTTTATA   
  
  
- TAATTTTATT TTAAAGAATA AATAAAAATT ATAGTTGTCT AATTTTGATA GTATTTAGTG ATTTATTAAT   
  
  
- AGTTAAATTA TAAAAAGTTT GGTTTTTGTG TAAATTTTAT GTGAAGTTTT GACACAGTTG AACTTCGAGT   
  
  
- TTTAAGTGTC TCTTCAATTT TTGTAAATTG ACATTTACGC TTCGATGTGA CAGGTTTGGA AGGTCACTGT   
  
  
- TACTTTTCGA ACGGTGAAAC ATAACTTTAT AACCATGGTG CACATTCTGT ATACGCTTGT TCCGTTACCA   
  
  
- CTGCAGCCGT TCCATATCCG TTAGCACCCT GGTTAGGGCG GCGGCATGCA GTGATTTGTG TGCACGGAAT   
  
  
- TTTTGTTTAA GGGTTCGGAA GGATCGTTGT TTCTTGTTC

+     ABRE

| Site Name | Organism | Position | Strand | Matrix score. | sequence | function |
| --- | --- | --- | --- | --- | --- | --- |
| ABRE | Arabidopsis thaliana | 1858 | - | 6 | CACGTG | cis-acting element involved in the abscisic acid responsiveness |
| ABRE | Arabidopsis thaliana | 1950 | - | 6 | CACGTG | cis-acting element involved in the abscisic acid responsiveness |
| ABRE | Arabidopsis thaliana | 1859 | + | 5 | ACGTG | cis-acting element involved in the abscisic acid responsiveness |
| ABRE | Arabidopsis thaliana | 1951 | + | 5 | ACGTG | cis-acting element involved in the abscisic acid responsiveness |

>Potri.002G039100.1   
+ TTTTTTTTTA TCCGTAATTG ATTATTCAAA ACCTTTCCAG CTTAAAATTT TTTCCATAAT TTAAAAACTA   
  
  
+ TTAAAGGTTG AAAACACTTT TTTTTTCTCT GAGATTTAAA ACTAATACTA TAAAAACTCT AACTCTAACT   
  
  
+ TTCCGTTGTT TATTATTTAT CACCGAATCT TCTTGATAAA TGTATTTCTT AAAGTATATT TTATATAAAA   
  
  
+ ATATTTTTTT TATTTTTAAA ATTTTCCTTT AATATACATC ATGTAAAGTT TCAAATCGAT TTAAAAATTA   
  
  
+ AACATAATTT TTTTAAAAAA TTAACGGAAC CGCAAAAAAA TAAAGAGATG GTCGCCATCA ACCGTGTAGC   
  
  
+ AACCCTTTCT TTTCGGAGGA CTTTCAAAAT TCAGGCATTT CCATGGAGAT TTTTTGTCTT CTTGAAGACC   
  
  
+ GGGAACGGTT TCAAGCAATC CCACGAAAGG GGGCCTGAAT CTAGACAACG TTGCTATCAA ACAGCTTCAT   
  
  
+ CTGTGTTAAA TATTTTATGG ATTTGATTTA CCTGTCCCCT CTGCCAGTCT TTTCAAATCA AATTTGCCAC   
  
  
+ AAGAAACCAT GACTTTTATT TCTTTCATGT GCACATCATT ACCTTAAGTG ATAACGATTA AAGGTGGAGG   
  
  
+ TTTGCCTAAA ATTACCAGTT GATATTAAAT ACGCAACAAC TTAATTTCAC ATGAATCCTC ACCCGTATTA   
  
  
+ GCTAGGGTGC CAAACTCTCT TGATCACCAT CCGTGATTAA GCGGTGCGTG GTTTTATTGA TTAAAACTAA   
  
  
+ TGTGATTTAA ATCTGATTAT CCCCGAGTTA ATTATGAAGG CTCGGAACTC CATTTTCTTC TCCTTTTTTT   
  
  
+ CACTTTTTTC TTGGACAGGG GACGAAAGTT GGTTCATGTG GCATCAAAAA AGTAGTGTTT AATAATTTAG   
  
  
+ ATGCTTTTAA CATTCATTGG ACAAGACTAA AAAGATCAGA TTTCATAGCA AATCTATCAC TTAGTCGACA   
  
  
+ TAAATATGTA AAAAACTCGA GATTTTATTA ATGGGTCTTG AATTTAATTA AAACCTTTAA ATTAAATAAA   
  
  
+ TATTATTCTC GTCACGAGTC AATCTCTTGT AACATCGTCA TTTCTTTAAG ATCAGTGACC TTGTTGAAAA   
  
  
+ CTAAAAACAC ACACAATTTT AATCATTCTT CTCTTTTTCG TGTATTCAAC TCACTTAAAT TCAACATATA   
  
  
+ GATTGAAAGA TTACAAGTCT AGACCTATTA TAATTCAATA AAGATTCTAA TTAGCAAGTT GAATAAGTGT   
  
  
+ TCTAAACACC AATTACTTTT TTCATCGATG ATTTCTATTC AAACTACGAT GTAAACTAAT ATCTAACAAT   
  
  
+ TGTCTTGAAT TTCGAACAAA TAATCACAGC TGCAGCTTTC AAAACATTAC CCAGAACCAT TTTCCTTGAC   
  
  
+ TTCTTTTTTA TTTTCTTATG CTTGAAATAA TACAGAACAG ATTAACGAGT TCTGTACACT GAAAATGAAA   
  
  
+ CAAGACTTTG AACTTCTAAC TTGGTATATT CTATTTTGTT ATACCGTCCC ATCATGTTAA GACTGGAAAA   
  
  
+ CTAATTAAAA GGTATTTCAT GAGATTATGA TGCATAATTA TTTTTAAAAT AATTTTTTAT TTAAAAATAT   
  
  
+ ATTAAAATAA AATTTCTTAT TTATTTTTAA TATCAACAGA TTAAAACTAT CATAAATCAC TAAATAATTA   
  
  
+ TCAATTTAAT ATTTTTCAAA CCAAAAACAC ATTTAAAATA CACTTCAAAA CTGTGTCAAC TTGAAGCTCA   
  
  
+ AAATTCACAG AGAAGTTAAA AACATTTAAC TGTAAATGCG AAGCTACACT GTCCAAACCT TCCAGTGACA   
  
  
+ ATGAAAAGCT TGCCACTTTG TATTGAAATA TTGGTACCAC GTGTAAGACA TATGCGAACA AGGCAATGGT   
  
  
+ GACGTCGGCA AGGTATAGGC AATCGTGGGA CCAATCCCGC CGCCGTACGT CACTAAACAC ACGTGCCTTA   
  
  
+ AAAACAAATT CCCAAGCCTT CCTAGCAACA AAGAACAAG  

- AAAAAAAAAT AGGCATTAAC TAATAAGTTT TGGAAAGGTC GAATTTTAAA AAAGGTATTA AATTTTTGAT   
  
  
- AATTTCCAAC TTTTGTGAAA AAAAAAGAGA CTCTAAATTT TGATTATGAT ATTTTTGAGA TTGAGATTGA   
  
  
- AAGGCAACAA ATAATAAATA GTGGCTTAGA AGAACTATTT ACATAAAGAA TTTCATATAA AATATATTTT   
  
  
- TATAAAAAAA ATAAAAATTT TAAAAGGAAA TTATATGTAG TACATTTCAA AGTTTAGCTA AATTTTTAAT   
  
  
- TTGTATTAAA AAAATTTTTT AATTGCCTTG GCGTTTTTTT ATTTCTCTAC CAGCGGTAGT TGGCACATCG   
  
  
- TTGGGAAAGA AAAGCCTCCT GAAAGTTTTA AGTCCGTAAA GGTACCTCTA AAAAACAGAA GAACTTCTGG   
  
  
- CCCTTGCCAA AGTTCGTTAG GGTGCTTTCC CCCGGACTTA GATCTGTTGC AACGATAGTT TGTCGAAGTA   
  
  
- GACACAATTT ATAAAATACC TAAACTAAAT GGACAGGGGA GACGGTCAGA AAAGTTTAGT TTAAACGGTG   
  
  
- TTCTTTGGTA CTGAAAATAA AGAAAGTACA CGTGTAGTAA TGGAATTCAC TATTGCTAAT TTCCACCTCC   
  
  
- AAACGGATTT TAATGGTCAA CTATAATTTA TGCGTTGTTG AATTAAAGTG TACTTAGGAG TGGGCATAAT   
  
  
- CGATCCCACG GTTTGAGAGA ACTAGTGGTA GGCACTAATT CGCCACGCAC CAAAATAACT AATTTTGATT   
  
  
- ACACTAAATT TAGACTAATA GGGGCTCAAT TAATACTTCC GAGCCTTGAG GTAAAAGAAG AGGAAAAAAA   
  
  
- GTGAAAAAAG AACCTGTCCC CTGCTTTCAA CCAAGTACAC CGTAGTTTTT TCATCACAAA TTATTAAATC   
  
  
- TACGAAAATT GTAAGTAACC TGTTCTGATT TTTCTAGTCT AAAGTATCGT TTAGATAGTG AATCAGCTGT   
  
  
- ATTTATACAT TTTTTGAGCT CTAAAATAAT TACCCAGAAC TTAAATTAAT TTTGGAAATT TAATTTATTT   
  
  
- ATAATAAGAG CAGTGCTCAG TTAGAGAACA TTGTAGCAGT AAAGAAATTC TAGTCACTGG AACAACTTTT   
  
  
- GATTTTTGTG TGTGTTAAAA TTAGTAAGAA GAGAAAAAGC ACATAAGTTG AGTGAATTTA AGTTGTATAT   
  
  
- CTAACTTTCT AATGTTCAGA TCTGGATAAT ATTAAGTTAT TTCTAAGATT AATCGTTCAA CTTATTCACA   
  
  
- AGATTTGTGG TTAATGAAAA AAGTAGCTAC TAAAGATAAG TTTGATGCTA CATTTGATTA TAGATTGTTA   
  
  
- ACAGAACTTA AAGCTTGTTT ATTAGTGTCG ACGTCGAAAG TTTTGTAATG GGTCTTGGTA AAAGGAACTG   
  
  
- AAGAAAAAAT AAAAGAATAC GAACTTTATT ATGTCTTGTC TAATTGCTCA AGACATGTGA CTTTTACTTT   
  
  
- GTTCTGAAAC TTGAAGATTG AACCATATAA GATAAAACAA TATGGCAGGG TAGTACAATT CTGACCTTTT   
  
  
- GATTAATTTT CCATAAAGTA CTCTAATACT ACGTATTAAT AAAAATTTTA TTAAAAAATA AATTTTTATA   
  
  
- TAATTTTATT TTAAAGAATA AATAAAAATT ATAGTTGTCT AATTTTGATA GTATTTAGTG ATTTATTAAT   
  
  
- AGTTAAATTA TAAAAAGTTT GGTTTTTGTG TAAATTTTAT GTGAAGTTTT GACACAGTTG AACTTCGAGT   
  
  
- TTTAAGTGTC TCTTCAATTT TTGTAAATTG ACATTTACGC TTCGATGTGA CAGGTTTGGA AGGTCACTGT   
  
  
- TACTTTTCGA ACGGTGAAAC ATAACTTTAT AACCATGGTG CACATTCTGT ATACGCTTGT TCCGTTACCA   
  
  
- CTGCAGCCGT TCCATATCCG TTAGCACCCT GGTTAGGGCG GCGGCATGCA GTGATTTGTG TGCACGGAAT   
  
  
- TTTTGTTTAA GGGTTCGGAA GGATCGTTGT TTCTTGTTC

+     ARE

| Site Name | Organism | Position | Strand | Matrix score. | sequence | function |
| --- | --- | --- | --- | --- | --- | --- |
| ARE | Zea mays | 564 | + | 6 | AAACCA | cis-acting regulatory element essential for the anaerobic induction |
| ARE | Zea mays | 1698 | + | 6 | AAACCA | cis-acting regulatory element essential for the anaerobic induction |
| ARE | Zea mays | 749 | - | 6 | AAACCA | cis-acting regulatory element essential for the anaerobic induction |

>Potri.002G039100.1   
+ TTTTTTTTTA TCCGTAATTG ATTATTCAAA ACCTTTCCAG CTTAAAATTT TTTCCATAAT TTAAAAACTA   
  
  
+ TTAAAGGTTG AAAACACTTT TTTTTTCTCT GAGATTTAAA ACTAATACTA TAAAAACTCT AACTCTAACT   
  
  
+ TTCCGTTGTT TATTATTTAT CACCGAATCT TCTTGATAAA TGTATTTCTT AAAGTATATT TTATATAAAA   
  
  
+ ATATTTTTTT TATTTTTAAA ATTTTCCTTT AATATACATC ATGTAAAGTT TCAAATCGAT TTAAAAATTA   
  
  
+ AACATAATTT TTTTAAAAAA TTAACGGAAC CGCAAAAAAA TAAAGAGATG GTCGCCATCA ACCGTGTAGC   
  
  
+ AACCCTTTCT TTTCGGAGGA CTTTCAAAAT TCAGGCATTT CCATGGAGAT TTTTTGTCTT CTTGAAGACC   
  
  
+ GGGAACGGTT TCAAGCAATC CCACGAAAGG GGGCCTGAAT CTAGACAACG TTGCTATCAA ACAGCTTCAT   
  
  
+ CTGTGTTAAA TATTTTATGG ATTTGATTTA CCTGTCCCCT CTGCCAGTCT TTTCAAATCA AATTTGCCAC   
  
  
+ AAGAAACCAT GACTTTTATT TCTTTCATGT GCACATCATT ACCTTAAGTG ATAACGATTA AAGGTGGAGG   
  
  
+ TTTGCCTAAA ATTACCAGTT GATATTAAAT ACGCAACAAC TTAATTTCAC ATGAATCCTC ACCCGTATTA   
  
  
+ GCTAGGGTGC CAAACTCTCT TGATCACCAT CCGTGATTAA GCGGTGCGTG GTTTTATTGA TTAAAACTAA   
  
  
+ TGTGATTTAA ATCTGATTAT CCCCGAGTTA ATTATGAAGG CTCGGAACTC CATTTTCTTC TCCTTTTTTT   
  
  
+ CACTTTTTTC TTGGACAGGG GACGAAAGTT GGTTCATGTG GCATCAAAAA AGTAGTGTTT AATAATTTAG   
  
  
+ ATGCTTTTAA CATTCATTGG ACAAGACTAA AAAGATCAGA TTTCATAGCA AATCTATCAC TTAGTCGACA   
  
  
+ TAAATATGTA AAAAACTCGA GATTTTATTA ATGGGTCTTG AATTTAATTA AAACCTTTAA ATTAAATAAA   
  
  
+ TATTATTCTC GTCACGAGTC AATCTCTTGT AACATCGTCA TTTCTTTAAG ATCAGTGACC TTGTTGAAAA   
  
  
+ CTAAAAACAC ACACAATTTT AATCATTCTT CTCTTTTTCG TGTATTCAAC TCACTTAAAT TCAACATATA   
  
  
+ GATTGAAAGA TTACAAGTCT AGACCTATTA TAATTCAATA AAGATTCTAA TTAGCAAGTT GAATAAGTGT   
  
  
+ TCTAAACACC AATTACTTTT TTCATCGATG ATTTCTATTC AAACTACGAT GTAAACTAAT ATCTAACAAT   
  
  
+ TGTCTTGAAT TTCGAACAAA TAATCACAGC TGCAGCTTTC AAAACATTAC CCAGAACCAT TTTCCTTGAC   
  
  
+ TTCTTTTTTA TTTTCTTATG CTTGAAATAA TACAGAACAG ATTAACGAGT TCTGTACACT GAAAATGAAA   
  
  
+ CAAGACTTTG AACTTCTAAC TTGGTATATT CTATTTTGTT ATACCGTCCC ATCATGTTAA GACTGGAAAA   
  
  
+ CTAATTAAAA GGTATTTCAT GAGATTATGA TGCATAATTA TTTTTAAAAT AATTTTTTAT TTAAAAATAT   
  
  
+ ATTAAAATAA AATTTCTTAT TTATTTTTAA TATCAACAGA TTAAAACTAT CATAAATCAC TAAATAATTA   
  
  
+ TCAATTTAAT ATTTTTCAAA CCAAAAACAC ATTTAAAATA CACTTCAAAA CTGTGTCAAC TTGAAGCTCA   
  
  
+ AAATTCACAG AGAAGTTAAA AACATTTAAC TGTAAATGCG AAGCTACACT GTCCAAACCT TCCAGTGACA   
  
  
+ ATGAAAAGCT TGCCACTTTG TATTGAAATA TTGGTACCAC GTGTAAGACA TATGCGAACA AGGCAATGGT   
  
  
+ GACGTCGGCA AGGTATAGGC AATCGTGGGA CCAATCCCGC CGCCGTACGT CACTAAACAC ACGTGCCTTA   
  
  
+ AAAACAAATT CCCAAGCCTT CCTAGCAACA AAGAACAAG  

- AAAAAAAAAT AGGCATTAAC TAATAAGTTT TGGAAAGGTC GAATTTTAAA AAAGGTATTA AATTTTTGAT   
  
  
- AATTTCCAAC TTTTGTGAAA AAAAAAGAGA CTCTAAATTT TGATTATGAT ATTTTTGAGA TTGAGATTGA   
  
  
- AAGGCAACAA ATAATAAATA GTGGCTTAGA AGAACTATTT ACATAAAGAA TTTCATATAA AATATATTTT   
  
  
- TATAAAAAAA ATAAAAATTT TAAAAGGAAA TTATATGTAG TACATTTCAA AGTTTAGCTA AATTTTTAAT   
  
  
- TTGTATTAAA AAAATTTTTT AATTGCCTTG GCGTTTTTTT ATTTCTCTAC CAGCGGTAGT TGGCACATCG   
  
  
- TTGGGAAAGA AAAGCCTCCT GAAAGTTTTA AGTCCGTAAA GGTACCTCTA AAAAACAGAA GAACTTCTGG   
  
  
- CCCTTGCCAA AGTTCGTTAG GGTGCTTTCC CCCGGACTTA GATCTGTTGC AACGATAGTT TGTCGAAGTA   
  
  
- GACACAATTT ATAAAATACC TAAACTAAAT GGACAGGGGA GACGGTCAGA AAAGTTTAGT TTAAACGGTG   
  
  
- TTCTTTGGTA CTGAAAATAA AGAAAGTACA CGTGTAGTAA TGGAATTCAC TATTGCTAAT TTCCACCTCC   
  
  
- AAACGGATTT TAATGGTCAA CTATAATTTA TGCGTTGTTG AATTAAAGTG TACTTAGGAG TGGGCATAAT   
  
  
- CGATCCCACG GTTTGAGAGA ACTAGTGGTA GGCACTAATT CGCCACGCAC CAAAATAACT AATTTTGATT   
  
  
- ACACTAAATT TAGACTAATA GGGGCTCAAT TAATACTTCC GAGCCTTGAG GTAAAAGAAG AGGAAAAAAA   
  
  
- GTGAAAAAAG AACCTGTCCC CTGCTTTCAA CCAAGTACAC CGTAGTTTTT TCATCACAAA TTATTAAATC   
  
  
- TACGAAAATT GTAAGTAACC TGTTCTGATT TTTCTAGTCT AAAGTATCGT TTAGATAGTG AATCAGCTGT   
  
  
- ATTTATACAT TTTTTGAGCT CTAAAATAAT TACCCAGAAC TTAAATTAAT TTTGGAAATT TAATTTATTT   
  
  
- ATAATAAGAG CAGTGCTCAG TTAGAGAACA TTGTAGCAGT AAAGAAATTC TAGTCACTGG AACAACTTTT   
  
  
- GATTTTTGTG TGTGTTAAAA TTAGTAAGAA GAGAAAAAGC ACATAAGTTG AGTGAATTTA AGTTGTATAT   
  
  
- CTAACTTTCT AATGTTCAGA TCTGGATAAT ATTAAGTTAT TTCTAAGATT AATCGTTCAA CTTATTCACA   
  
  
- AGATTTGTGG TTAATGAAAA AAGTAGCTAC TAAAGATAAG TTTGATGCTA CATTTGATTA TAGATTGTTA   
  
  
- ACAGAACTTA AAGCTTGTTT ATTAGTGTCG ACGTCGAAAG TTTTGTAATG GGTCTTGGTA AAAGGAACTG   
  
  
- AAGAAAAAAT AAAAGAATAC GAACTTTATT ATGTCTTGTC TAATTGCTCA AGACATGTGA CTTTTACTTT   
  
  
- GTTCTGAAAC TTGAAGATTG AACCATATAA GATAAAACAA TATGGCAGGG TAGTACAATT CTGACCTTTT   
  
  
- GATTAATTTT CCATAAAGTA CTCTAATACT ACGTATTAAT AAAAATTTTA TTAAAAAATA AATTTTTATA   
  
  
- TAATTTTATT TTAAAGAATA AATAAAAATT ATAGTTGTCT AATTTTGATA GTATTTAGTG ATTTATTAAT   
  
  
- AGTTAAATTA TAAAAAGTTT GGTTTTTGTG TAAATTTTAT GTGAAGTTTT GACACAGTTG AACTTCGAGT   
  
  
- TTTAAGTGTC TCTTCAATTT TTGTAAATTG ACATTTACGC TTCGATGTGA CAGGTTTGGA AGGTCACTGT   
  
  
- TACTTTTCGA ACGGTGAAAC ATAACTTTAT AACCATGGTG CACATTCTGT ATACGCTTGT TCCGTTACCA   
  
  
- CTGCAGCCGT TCCATATCCG TTAGCACCCT GGTTAGGGCG GCGGCATGCA GTGATTTGTG TGCACGGAAT   
  
  
- TTTTGTTTAA GGGTTCGGAA GGATCGTTGT TTCTTGTTC

+     AT-rich element

| Site Name | Organism | Position | Strand | Matrix score. | sequence | function |
| --- | --- | --- | --- | --- | --- | --- |
| AT-rich element | Glycine max | 1288 | - | 10 | ATAGAAATCAA | binding site of AT-rich DNA binding protein (ATBP-1) |

>Potri.002G039100.1   
+ TTTTTTTTTA TCCGTAATTG ATTATTCAAA ACCTTTCCAG CTTAAAATTT TTTCCATAAT TTAAAAACTA   
  
  
+ TTAAAGGTTG AAAACACTTT TTTTTTCTCT GAGATTTAAA ACTAATACTA TAAAAACTCT AACTCTAACT   
  
  
+ TTCCGTTGTT TATTATTTAT CACCGAATCT TCTTGATAAA TGTATTTCTT AAAGTATATT TTATATAAAA   
  
  
+ ATATTTTTTT TATTTTTAAA ATTTTCCTTT AATATACATC ATGTAAAGTT TCAAATCGAT TTAAAAATTA   
  
  
+ AACATAATTT TTTTAAAAAA TTAACGGAAC CGCAAAAAAA TAAAGAGATG GTCGCCATCA ACCGTGTAGC   
  
  
+ AACCCTTTCT TTTCGGAGGA CTTTCAAAAT TCAGGCATTT CCATGGAGAT TTTTTGTCTT CTTGAAGACC   
  
  
+ GGGAACGGTT TCAAGCAATC CCACGAAAGG GGGCCTGAAT CTAGACAACG TTGCTATCAA ACAGCTTCAT   
  
  
+ CTGTGTTAAA TATTTTATGG ATTTGATTTA CCTGTCCCCT CTGCCAGTCT TTTCAAATCA AATTTGCCAC   
  
  
+ AAGAAACCAT GACTTTTATT TCTTTCATGT GCACATCATT ACCTTAAGTG ATAACGATTA AAGGTGGAGG   
  
  
+ TTTGCCTAAA ATTACCAGTT GATATTAAAT ACGCAACAAC TTAATTTCAC ATGAATCCTC ACCCGTATTA   
  
  
+ GCTAGGGTGC CAAACTCTCT TGATCACCAT CCGTGATTAA GCGGTGCGTG GTTTTATTGA TTAAAACTAA   
  
  
+ TGTGATTTAA ATCTGATTAT CCCCGAGTTA ATTATGAAGG CTCGGAACTC CATTTTCTTC TCCTTTTTTT   
  
  
+ CACTTTTTTC TTGGACAGGG GACGAAAGTT GGTTCATGTG GCATCAAAAA AGTAGTGTTT AATAATTTAG   
  
  
+ ATGCTTTTAA CATTCATTGG ACAAGACTAA AAAGATCAGA TTTCATAGCA AATCTATCAC TTAGTCGACA   
  
  
+ TAAATATGTA AAAAACTCGA GATTTTATTA ATGGGTCTTG AATTTAATTA AAACCTTTAA ATTAAATAAA   
  
  
+ TATTATTCTC GTCACGAGTC AATCTCTTGT AACATCGTCA TTTCTTTAAG ATCAGTGACC TTGTTGAAAA   
  
  
+ CTAAAAACAC ACACAATTTT AATCATTCTT CTCTTTTTCG TGTATTCAAC TCACTTAAAT TCAACATATA   
  
  
+ GATTGAAAGA TTACAAGTCT AGACCTATTA TAATTCAATA AAGATTCTAA TTAGCAAGTT GAATAAGTGT   
  
  
+ TCTAAACACC AATTACTTTT TTCATCGATG ATTTCTATTC AAACTACGAT GTAAACTAAT ATCTAACAAT   
  
  
+ TGTCTTGAAT TTCGAACAAA TAATCACAGC TGCAGCTTTC AAAACATTAC CCAGAACCAT TTTCCTTGAC   
  
  
+ TTCTTTTTTA TTTTCTTATG CTTGAAATAA TACAGAACAG ATTAACGAGT TCTGTACACT GAAAATGAAA   
  
  
+ CAAGACTTTG AACTTCTAAC TTGGTATATT CTATTTTGTT ATACCGTCCC ATCATGTTAA GACTGGAAAA   
  
  
+ CTAATTAAAA GGTATTTCAT GAGATTATGA TGCATAATTA TTTTTAAAAT AATTTTTTAT TTAAAAATAT   
  
  
+ ATTAAAATAA AATTTCTTAT TTATTTTTAA TATCAACAGA TTAAAACTAT CATAAATCAC TAAATAATTA   
  
  
+ TCAATTTAAT ATTTTTCAAA CCAAAAACAC ATTTAAAATA CACTTCAAAA CTGTGTCAAC TTGAAGCTCA   
  
  
+ AAATTCACAG AGAAGTTAAA AACATTTAAC TGTAAATGCG AAGCTACACT GTCCAAACCT TCCAGTGACA   
  
  
+ ATGAAAAGCT TGCCACTTTG TATTGAAATA TTGGTACCAC GTGTAAGACA TATGCGAACA AGGCAATGGT   
  
  
+ GACGTCGGCA AGGTATAGGC AATCGTGGGA CCAATCCCGC CGCCGTACGT CACTAAACAC ACGTGCCTTA   
  
  
+ AAAACAAATT CCCAAGCCTT CCTAGCAACA AAGAACAAG  

- AAAAAAAAAT AGGCATTAAC TAATAAGTTT TGGAAAGGTC GAATTTTAAA AAAGGTATTA AATTTTTGAT   
  
  
- AATTTCCAAC TTTTGTGAAA AAAAAAGAGA CTCTAAATTT TGATTATGAT ATTTTTGAGA TTGAGATTGA   
  
  
- AAGGCAACAA ATAATAAATA GTGGCTTAGA AGAACTATTT ACATAAAGAA TTTCATATAA AATATATTTT   
  
  
- TATAAAAAAA ATAAAAATTT TAAAAGGAAA TTATATGTAG TACATTTCAA AGTTTAGCTA AATTTTTAAT   
  
  
- TTGTATTAAA AAAATTTTTT AATTGCCTTG GCGTTTTTTT ATTTCTCTAC CAGCGGTAGT TGGCACATCG   
  
  
- TTGGGAAAGA AAAGCCTCCT GAAAGTTTTA AGTCCGTAAA GGTACCTCTA AAAAACAGAA GAACTTCTGG   
  
  
- CCCTTGCCAA AGTTCGTTAG GGTGCTTTCC CCCGGACTTA GATCTGTTGC AACGATAGTT TGTCGAAGTA   
  
  
- GACACAATTT ATAAAATACC TAAACTAAAT GGACAGGGGA GACGGTCAGA AAAGTTTAGT TTAAACGGTG   
  
  
- TTCTTTGGTA CTGAAAATAA AGAAAGTACA CGTGTAGTAA TGGAATTCAC TATTGCTAAT TTCCACCTCC   
  
  
- AAACGGATTT TAATGGTCAA CTATAATTTA TGCGTTGTTG AATTAAAGTG TACTTAGGAG TGGGCATAAT   
  
  
- CGATCCCACG GTTTGAGAGA ACTAGTGGTA GGCACTAATT CGCCACGCAC CAAAATAACT AATTTTGATT   
  
  
- ACACTAAATT TAGACTAATA GGGGCTCAAT TAATACTTCC GAGCCTTGAG GTAAAAGAAG AGGAAAAAAA   
  
  
- GTGAAAAAAG AACCTGTCCC CTGCTTTCAA CCAAGTACAC CGTAGTTTTT TCATCACAAA TTATTAAATC   
  
  
- TACGAAAATT GTAAGTAACC TGTTCTGATT TTTCTAGTCT AAAGTATCGT TTAGATAGTG AATCAGCTGT   
  
  
- ATTTATACAT TTTTTGAGCT CTAAAATAAT TACCCAGAAC TTAAATTAAT TTTGGAAATT TAATTTATTT   
  
  
- ATAATAAGAG CAGTGCTCAG TTAGAGAACA TTGTAGCAGT AAAGAAATTC TAGTCACTGG AACAACTTTT   
  
  
- GATTTTTGTG TGTGTTAAAA TTAGTAAGAA GAGAAAAAGC ACATAAGTTG AGTGAATTTA AGTTGTATAT   
  
  
- CTAACTTTCT AATGTTCAGA TCTGGATAAT ATTAAGTTAT TTCTAAGATT AATCGTTCAA CTTATTCACA   
  
  
- AGATTTGTGG TTAATGAAAA AAGTAGCTAC TAAAGATAAG TTTGATGCTA CATTTGATTA TAGATTGTTA   
  
  
- ACAGAACTTA AAGCTTGTTT ATTAGTGTCG ACGTCGAAAG TTTTGTAATG GGTCTTGGTA AAAGGAACTG   
  
  
- AAGAAAAAAT AAAAGAATAC GAACTTTATT ATGTCTTGTC TAATTGCTCA AGACATGTGA CTTTTACTTT   
  
  
- GTTCTGAAAC TTGAAGATTG AACCATATAA GATAAAACAA TATGGCAGGG TAGTACAATT CTGACCTTTT   
  
  
- GATTAATTTT CCATAAAGTA CTCTAATACT ACGTATTAAT AAAAATTTTA TTAAAAAATA AATTTTTATA   
  
  
- TAATTTTATT TTAAAGAATA AATAAAAATT ATAGTTGTCT AATTTTGATA GTATTTAGTG ATTTATTAAT   
  
  
- AGTTAAATTA TAAAAAGTTT GGTTTTTGTG TAAATTTTAT GTGAAGTTTT GACACAGTTG AACTTCGAGT   
  
  
- TTTAAGTGTC TCTTCAATTT TTGTAAATTG ACATTTACGC TTCGATGTGA CAGGTTTGGA AGGTCACTGT   
  
  
- TACTTTTCGA ACGGTGAAAC ATAACTTTAT AACCATGGTG CACATTCTGT ATACGCTTGT TCCGTTACCA   
  
  
- CTGCAGCCGT TCCATATCCG TTAGCACCCT GGTTAGGGCG GCGGCATGCA GTGATTTGTG TGCACGGAAT   
  
  
- TTTTGTTTAA GGGTTCGGAA GGATCGTTGT TTCTTGTTC

+     AT1-motif

| Site Name | Organism | Position | Strand | Matrix score. | sequence | function |
| --- | --- | --- | --- | --- | --- | --- |
| AT1-motif | Solanum tuberosum | 1588 | + | 13 | AATTATTTTTTATT | part of a light responsive module |

>Potri.002G039100.1   
+ TTTTTTTTTA TCCGTAATTG ATTATTCAAA ACCTTTCCAG CTTAAAATTT TTTCCATAAT TTAAAAACTA   
  
  
+ TTAAAGGTTG AAAACACTTT TTTTTTCTCT GAGATTTAAA ACTAATACTA TAAAAACTCT AACTCTAACT   
  
  
+ TTCCGTTGTT TATTATTTAT CACCGAATCT TCTTGATAAA TGTATTTCTT AAAGTATATT TTATATAAAA   
  
  
+ ATATTTTTTT TATTTTTAAA ATTTTCCTTT AATATACATC ATGTAAAGTT TCAAATCGAT TTAAAAATTA   
  
  
+ AACATAATTT TTTTAAAAAA TTAACGGAAC CGCAAAAAAA TAAAGAGATG GTCGCCATCA ACCGTGTAGC   
  
  
+ AACCCTTTCT TTTCGGAGGA CTTTCAAAAT TCAGGCATTT CCATGGAGAT TTTTTGTCTT CTTGAAGACC   
  
  
+ GGGAACGGTT TCAAGCAATC CCACGAAAGG GGGCCTGAAT CTAGACAACG TTGCTATCAA ACAGCTTCAT   
  
  
+ CTGTGTTAAA TATTTTATGG ATTTGATTTA CCTGTCCCCT CTGCCAGTCT TTTCAAATCA AATTTGCCAC   
  
  
+ AAGAAACCAT GACTTTTATT TCTTTCATGT GCACATCATT ACCTTAAGTG ATAACGATTA AAGGTGGAGG   
  
  
+ TTTGCCTAAA ATTACCAGTT GATATTAAAT ACGCAACAAC TTAATTTCAC ATGAATCCTC ACCCGTATTA   
  
  
+ GCTAGGGTGC CAAACTCTCT TGATCACCAT CCGTGATTAA GCGGTGCGTG GTTTTATTGA TTAAAACTAA   
  
  
+ TGTGATTTAA ATCTGATTAT CCCCGAGTTA ATTATGAAGG CTCGGAACTC CATTTTCTTC TCCTTTTTTT   
  
  
+ CACTTTTTTC TTGGACAGGG GACGAAAGTT GGTTCATGTG GCATCAAAAA AGTAGTGTTT AATAATTTAG   
  
  
+ ATGCTTTTAA CATTCATTGG ACAAGACTAA AAAGATCAGA TTTCATAGCA AATCTATCAC TTAGTCGACA   
  
  
+ TAAATATGTA AAAAACTCGA GATTTTATTA ATGGGTCTTG AATTTAATTA AAACCTTTAA ATTAAATAAA   
  
  
+ TATTATTCTC GTCACGAGTC AATCTCTTGT AACATCGTCA TTTCTTTAAG ATCAGTGACC TTGTTGAAAA   
  
  
+ CTAAAAACAC ACACAATTTT AATCATTCTT CTCTTTTTCG TGTATTCAAC TCACTTAAAT TCAACATATA   
  
  
+ GATTGAAAGA TTACAAGTCT AGACCTATTA TAATTCAATA AAGATTCTAA TTAGCAAGTT GAATAAGTGT   
  
  
+ TCTAAACACC AATTACTTTT TTCATCGATG ATTTCTATTC AAACTACGAT GTAAACTAAT ATCTAACAAT   
  
  
+ TGTCTTGAAT TTCGAACAAA TAATCACAGC TGCAGCTTTC AAAACATTAC CCAGAACCAT TTTCCTTGAC   
  
  
+ TTCTTTTTTA TTTTCTTATG CTTGAAATAA TACAGAACAG ATTAACGAGT TCTGTACACT GAAAATGAAA   
  
  
+ CAAGACTTTG AACTTCTAAC TTGGTATATT CTATTTTGTT ATACCGTCCC ATCATGTTAA GACTGGAAAA   
  
  
+ CTAATTAAAA GGTATTTCAT GAGATTATGA TGCATAATTA TTTTTAAAAT AATTTTTTAT TTAAAAATAT   
  
  
+ ATTAAAATAA AATTTCTTAT TTATTTTTAA TATCAACAGA TTAAAACTAT CATAAATCAC TAAATAATTA   
  
  
+ TCAATTTAAT ATTTTTCAAA CCAAAAACAC ATTTAAAATA CACTTCAAAA CTGTGTCAAC TTGAAGCTCA   
  
  
+ AAATTCACAG AGAAGTTAAA AACATTTAAC TGTAAATGCG AAGCTACACT GTCCAAACCT TCCAGTGACA   
  
  
+ ATGAAAAGCT TGCCACTTTG TATTGAAATA TTGGTACCAC GTGTAAGACA TATGCGAACA AGGCAATGGT   
  
  
+ GACGTCGGCA AGGTATAGGC AATCGTGGGA CCAATCCCGC CGCCGTACGT CACTAAACAC ACGTGCCTTA   
  
  
+ AAAACAAATT CCCAAGCCTT CCTAGCAACA AAGAACAAG  

- AAAAAAAAAT AGGCATTAAC TAATAAGTTT TGGAAAGGTC GAATTTTAAA AAAGGTATTA AATTTTTGAT   
  
  
- AATTTCCAAC TTTTGTGAAA AAAAAAGAGA CTCTAAATTT TGATTATGAT ATTTTTGAGA TTGAGATTGA   
  
  
- AAGGCAACAA ATAATAAATA GTGGCTTAGA AGAACTATTT ACATAAAGAA TTTCATATAA AATATATTTT   
  
  
- TATAAAAAAA ATAAAAATTT TAAAAGGAAA TTATATGTAG TACATTTCAA AGTTTAGCTA AATTTTTAAT   
  
  
- TTGTATTAAA AAAATTTTTT AATTGCCTTG GCGTTTTTTT ATTTCTCTAC CAGCGGTAGT TGGCACATCG   
  
  
- TTGGGAAAGA AAAGCCTCCT GAAAGTTTTA AGTCCGTAAA GGTACCTCTA AAAAACAGAA GAACTTCTGG   
  
  
- CCCTTGCCAA AGTTCGTTAG GGTGCTTTCC CCCGGACTTA GATCTGTTGC AACGATAGTT TGTCGAAGTA   
  
  
- GACACAATTT ATAAAATACC TAAACTAAAT GGACAGGGGA GACGGTCAGA AAAGTTTAGT TTAAACGGTG   
  
  
- TTCTTTGGTA CTGAAAATAA AGAAAGTACA CGTGTAGTAA TGGAATTCAC TATTGCTAAT TTCCACCTCC   
  
  
- AAACGGATTT TAATGGTCAA CTATAATTTA TGCGTTGTTG AATTAAAGTG TACTTAGGAG TGGGCATAAT   
  
  
- CGATCCCACG GTTTGAGAGA ACTAGTGGTA GGCACTAATT CGCCACGCAC CAAAATAACT AATTTTGATT   
  
  
- ACACTAAATT TAGACTAATA GGGGCTCAAT TAATACTTCC GAGCCTTGAG GTAAAAGAAG AGGAAAAAAA   
  
  
- GTGAAAAAAG AACCTGTCCC CTGCTTTCAA CCAAGTACAC CGTAGTTTTT TCATCACAAA TTATTAAATC   
  
  
- TACGAAAATT GTAAGTAACC TGTTCTGATT TTTCTAGTCT AAAGTATCGT TTAGATAGTG AATCAGCTGT   
  
  
- ATTTATACAT TTTTTGAGCT CTAAAATAAT TACCCAGAAC TTAAATTAAT TTTGGAAATT TAATTTATTT   
  
  
- ATAATAAGAG CAGTGCTCAG TTAGAGAACA TTGTAGCAGT AAAGAAATTC TAGTCACTGG AACAACTTTT   
  
  
- GATTTTTGTG TGTGTTAAAA TTAGTAAGAA GAGAAAAAGC ACATAAGTTG AGTGAATTTA AGTTGTATAT   
  
  
- CTAACTTTCT AATGTTCAGA TCTGGATAAT ATTAAGTTAT TTCTAAGATT AATCGTTCAA CTTATTCACA   
  
  
- AGATTTGTGG TTAATGAAAA AAGTAGCTAC TAAAGATAAG TTTGATGCTA CATTTGATTA TAGATTGTTA   
  
  
- ACAGAACTTA AAGCTTGTTT ATTAGTGTCG ACGTCGAAAG TTTTGTAATG GGTCTTGGTA AAAGGAACTG   
  
  
- AAGAAAAAAT AAAAGAATAC GAACTTTATT ATGTCTTGTC TAATTGCTCA AGACATGTGA CTTTTACTTT   
  
  
- GTTCTGAAAC TTGAAGATTG AACCATATAA GATAAAACAA TATGGCAGGG TAGTACAATT CTGACCTTTT   
  
  
- GATTAATTTT CCATAAAGTA CTCTAATACT ACGTATTAAT AAAAATTTTA TTAAAAAATA AATTTTTATA   
  
  
- TAATTTTATT TTAAAGAATA AATAAAAATT ATAGTTGTCT AATTTTGATA GTATTTAGTG ATTTATTAAT   
  
  
- AGTTAAATTA TAAAAAGTTT GGTTTTTGTG TAAATTTTAT GTGAAGTTTT GACACAGTTG AACTTCGAGT   
  
  
- TTTAAGTGTC TCTTCAATTT TTGTAAATTG ACATTTACGC TTCGATGTGA CAGGTTTGGA AGGTCACTGT   
  
  
- TACTTTTCGA ACGGTGAAAC ATAACTTTAT AACCATGGTG CACATTCTGT ATACGCTTGT TCCGTTACCA   
  
  
- CTGCAGCCGT TCCATATCCG TTAGCACCCT GGTTAGGGCG GCGGCATGCA GTGATTTGTG TGCACGGAAT   
  
  
- TTTTGTTTAA GGGTTCGGAA GGATCGTTGT TTCTTGTTC

+     ATCT-motif

| Site Name | Organism | Position | Strand | Matrix score. | sequence | function |
| --- | --- | --- | --- | --- | --- | --- |
| ATCT-motif | Pisum sativum | 509 | - | 9 | AATCTAATCC | part of a conserved DNA module involved in light responsiveness |

>Potri.002G039100.1   
+ TTTTTTTTTA TCCGTAATTG ATTATTCAAA ACCTTTCCAG CTTAAAATTT TTTCCATAAT TTAAAAACTA   
  
  
+ TTAAAGGTTG AAAACACTTT TTTTTTCTCT GAGATTTAAA ACTAATACTA TAAAAACTCT AACTCTAACT   
  
  
+ TTCCGTTGTT TATTATTTAT CACCGAATCT TCTTGATAAA TGTATTTCTT AAAGTATATT TTATATAAAA   
  
  
+ ATATTTTTTT TATTTTTAAA ATTTTCCTTT AATATACATC ATGTAAAGTT TCAAATCGAT TTAAAAATTA   
  
  
+ AACATAATTT TTTTAAAAAA TTAACGGAAC CGCAAAAAAA TAAAGAGATG GTCGCCATCA ACCGTGTAGC   
  
  
+ AACCCTTTCT TTTCGGAGGA CTTTCAAAAT TCAGGCATTT CCATGGAGAT TTTTTGTCTT CTTGAAGACC   
  
  
+ GGGAACGGTT TCAAGCAATC CCACGAAAGG GGGCCTGAAT CTAGACAACG TTGCTATCAA ACAGCTTCAT   
  
  
+ CTGTGTTAAA TATTTTATGG ATTTGATTTA CCTGTCCCCT CTGCCAGTCT TTTCAAATCA AATTTGCCAC   
  
  
+ AAGAAACCAT GACTTTTATT TCTTTCATGT GCACATCATT ACCTTAAGTG ATAACGATTA AAGGTGGAGG   
  
  
+ TTTGCCTAAA ATTACCAGTT GATATTAAAT ACGCAACAAC TTAATTTCAC ATGAATCCTC ACCCGTATTA   
  
  
+ GCTAGGGTGC CAAACTCTCT TGATCACCAT CCGTGATTAA GCGGTGCGTG GTTTTATTGA TTAAAACTAA   
  
  
+ TGTGATTTAA ATCTGATTAT CCCCGAGTTA ATTATGAAGG CTCGGAACTC CATTTTCTTC TCCTTTTTTT   
  
  
+ CACTTTTTTC TTGGACAGGG GACGAAAGTT GGTTCATGTG GCATCAAAAA AGTAGTGTTT AATAATTTAG   
  
  
+ ATGCTTTTAA CATTCATTGG ACAAGACTAA AAAGATCAGA TTTCATAGCA AATCTATCAC TTAGTCGACA   
  
  
+ TAAATATGTA AAAAACTCGA GATTTTATTA ATGGGTCTTG AATTTAATTA AAACCTTTAA ATTAAATAAA   
  
  
+ TATTATTCTC GTCACGAGTC AATCTCTTGT AACATCGTCA TTTCTTTAAG ATCAGTGACC TTGTTGAAAA   
  
  
+ CTAAAAACAC ACACAATTTT AATCATTCTT CTCTTTTTCG TGTATTCAAC TCACTTAAAT TCAACATATA   
  
  
+ GATTGAAAGA TTACAAGTCT AGACCTATTA TAATTCAATA AAGATTCTAA TTAGCAAGTT GAATAAGTGT   
  
  
+ TCTAAACACC AATTACTTTT TTCATCGATG ATTTCTATTC AAACTACGAT GTAAACTAAT ATCTAACAAT   
  
  
+ TGTCTTGAAT TTCGAACAAA TAATCACAGC TGCAGCTTTC AAAACATTAC CCAGAACCAT TTTCCTTGAC   
  
  
+ TTCTTTTTTA TTTTCTTATG CTTGAAATAA TACAGAACAG ATTAACGAGT TCTGTACACT GAAAATGAAA   
  
  
+ CAAGACTTTG AACTTCTAAC TTGGTATATT CTATTTTGTT ATACCGTCCC ATCATGTTAA GACTGGAAAA   
  
  
+ CTAATTAAAA GGTATTTCAT GAGATTATGA TGCATAATTA TTTTTAAAAT AATTTTTTAT TTAAAAATAT   
  
  
+ ATTAAAATAA AATTTCTTAT TTATTTTTAA TATCAACAGA TTAAAACTAT CATAAATCAC TAAATAATTA   
  
  
+ TCAATTTAAT ATTTTTCAAA CCAAAAACAC ATTTAAAATA CACTTCAAAA CTGTGTCAAC TTGAAGCTCA   
  
  
+ AAATTCACAG AGAAGTTAAA AACATTTAAC TGTAAATGCG AAGCTACACT GTCCAAACCT TCCAGTGACA   
  
  
+ ATGAAAAGCT TGCCACTTTG TATTGAAATA TTGGTACCAC GTGTAAGACA TATGCGAACA AGGCAATGGT   
  
  
+ GACGTCGGCA AGGTATAGGC AATCGTGGGA CCAATCCCGC CGCCGTACGT CACTAAACAC ACGTGCCTTA   
  
  
+ AAAACAAATT CCCAAGCCTT CCTAGCAACA AAGAACAAG  

- AAAAAAAAAT AGGCATTAAC TAATAAGTTT TGGAAAGGTC GAATTTTAAA AAAGGTATTA AATTTTTGAT   
  
  
- AATTTCCAAC TTTTGTGAAA AAAAAAGAGA CTCTAAATTT TGATTATGAT ATTTTTGAGA TTGAGATTGA   
  
  
- AAGGCAACAA ATAATAAATA GTGGCTTAGA AGAACTATTT ACATAAAGAA TTTCATATAA AATATATTTT   
  
  
- TATAAAAAAA ATAAAAATTT TAAAAGGAAA TTATATGTAG TACATTTCAA AGTTTAGCTA AATTTTTAAT   
  
  
- TTGTATTAAA AAAATTTTTT AATTGCCTTG GCGTTTTTTT ATTTCTCTAC CAGCGGTAGT TGGCACATCG   
  
  
- TTGGGAAAGA AAAGCCTCCT GAAAGTTTTA AGTCCGTAAA GGTACCTCTA AAAAACAGAA GAACTTCTGG   
  
  
- CCCTTGCCAA AGTTCGTTAG GGTGCTTTCC CCCGGACTTA GATCTGTTGC AACGATAGTT TGTCGAAGTA   
  
  
- GACACAATTT ATAAAATACC TAAACTAAAT GGACAGGGGA GACGGTCAGA AAAGTTTAGT TTAAACGGTG   
  
  
- TTCTTTGGTA CTGAAAATAA AGAAAGTACA CGTGTAGTAA TGGAATTCAC TATTGCTAAT TTCCACCTCC   
  
  
- AAACGGATTT TAATGGTCAA CTATAATTTA TGCGTTGTTG AATTAAAGTG TACTTAGGAG TGGGCATAAT   
  
  
- CGATCCCACG GTTTGAGAGA ACTAGTGGTA GGCACTAATT CGCCACGCAC CAAAATAACT AATTTTGATT   
  
  
- ACACTAAATT TAGACTAATA GGGGCTCAAT TAATACTTCC GAGCCTTGAG GTAAAAGAAG AGGAAAAAAA   
  
  
- GTGAAAAAAG AACCTGTCCC CTGCTTTCAA CCAAGTACAC CGTAGTTTTT TCATCACAAA TTATTAAATC   
  
  
- TACGAAAATT GTAAGTAACC TGTTCTGATT TTTCTAGTCT AAAGTATCGT TTAGATAGTG AATCAGCTGT   
  
  
- ATTTATACAT TTTTTGAGCT CTAAAATAAT TACCCAGAAC TTAAATTAAT TTTGGAAATT TAATTTATTT   
  
  
- ATAATAAGAG CAGTGCTCAG TTAGAGAACA TTGTAGCAGT AAAGAAATTC TAGTCACTGG AACAACTTTT   
  
  
- GATTTTTGTG TGTGTTAAAA TTAGTAAGAA GAGAAAAAGC ACATAAGTTG AGTGAATTTA AGTTGTATAT   
  
  
- CTAACTTTCT AATGTTCAGA TCTGGATAAT ATTAAGTTAT TTCTAAGATT AATCGTTCAA CTTATTCACA   
  
  
- AGATTTGTGG TTAATGAAAA AAGTAGCTAC TAAAGATAAG TTTGATGCTA CATTTGATTA TAGATTGTTA   
  
  
- ACAGAACTTA AAGCTTGTTT ATTAGTGTCG ACGTCGAAAG TTTTGTAATG GGTCTTGGTA AAAGGAACTG   
  
  
- AAGAAAAAAT AAAAGAATAC GAACTTTATT ATGTCTTGTC TAATTGCTCA AGACATGTGA CTTTTACTTT   
  
  
- GTTCTGAAAC TTGAAGATTG AACCATATAA GATAAAACAA TATGGCAGGG TAGTACAATT CTGACCTTTT   
  
  
- GATTAATTTT CCATAAAGTA CTCTAATACT ACGTATTAAT AAAAATTTTA TTAAAAAATA AATTTTTATA   
  
  
- TAATTTTATT TTAAAGAATA AATAAAAATT ATAGTTGTCT AATTTTGATA GTATTTAGTG ATTTATTAAT   
  
  
- AGTTAAATTA TAAAAAGTTT GGTTTTTGTG TAAATTTTAT GTGAAGTTTT GACACAGTTG AACTTCGAGT   
  
  
- TTTAAGTGTC TCTTCAATTT TTGTAAATTG ACATTTACGC TTCGATGTGA CAGGTTTGGA AGGTCACTGT   
  
  
- TACTTTTCGA ACGGTGAAAC ATAACTTTAT AACCATGGTG CACATTCTGT ATACGCTTGT TCCGTTACCA   
  
  
- CTGCAGCCGT TCCATATCCG TTAGCACCCT GGTTAGGGCG GCGGCATGCA GTGATTTGTG TGCACGGAAT   
  
  
- TTTTGTTTAA GGGTTCGGAA GGATCGTTGT TTCTTGTTC

+     AT~TATA-box

| Site Name | Organism | Position | Strand | Matrix score. | sequence | function |
| --- | --- | --- | --- | --- | --- | --- |
| AT~TATA-box | Arabidopsis thaliana | 200 | - | 8 | TATATAAA |  |
| AT~TATA-box | Arabidopsis thaliana | 202 | + | 6 | TATATA |  |

>Potri.002G039100.1   
+ TTTTTTTTTA TCCGTAATTG ATTATTCAAA ACCTTTCCAG CTTAAAATTT TTTCCATAAT TTAAAAACTA   
  
  
+ TTAAAGGTTG AAAACACTTT TTTTTTCTCT GAGATTTAAA ACTAATACTA TAAAAACTCT AACTCTAACT   
  
  
+ TTCCGTTGTT TATTATTTAT CACCGAATCT TCTTGATAAA TGTATTTCTT AAAGTATATT TTATATAAAA   
  
  
+ ATATTTTTTT TATTTTTAAA ATTTTCCTTT AATATACATC ATGTAAAGTT TCAAATCGAT TTAAAAATTA   
  
  
+ AACATAATTT TTTTAAAAAA TTAACGGAAC CGCAAAAAAA TAAAGAGATG GTCGCCATCA ACCGTGTAGC   
  
  
+ AACCCTTTCT TTTCGGAGGA CTTTCAAAAT TCAGGCATTT CCATGGAGAT TTTTTGTCTT CTTGAAGACC   
  
  
+ GGGAACGGTT TCAAGCAATC CCACGAAAGG GGGCCTGAAT CTAGACAACG TTGCTATCAA ACAGCTTCAT   
  
  
+ CTGTGTTAAA TATTTTATGG ATTTGATTTA CCTGTCCCCT CTGCCAGTCT TTTCAAATCA AATTTGCCAC   
  
  
+ AAGAAACCAT GACTTTTATT TCTTTCATGT GCACATCATT ACCTTAAGTG ATAACGATTA AAGGTGGAGG   
  
  
+ TTTGCCTAAA ATTACCAGTT GATATTAAAT ACGCAACAAC TTAATTTCAC ATGAATCCTC ACCCGTATTA   
  
  
+ GCTAGGGTGC CAAACTCTCT TGATCACCAT CCGTGATTAA GCGGTGCGTG GTTTTATTGA TTAAAACTAA   
  
  
+ TGTGATTTAA ATCTGATTAT CCCCGAGTTA ATTATGAAGG CTCGGAACTC CATTTTCTTC TCCTTTTTTT   
  
  
+ CACTTTTTTC TTGGACAGGG GACGAAAGTT GGTTCATGTG GCATCAAAAA AGTAGTGTTT AATAATTTAG   
  
  
+ ATGCTTTTAA CATTCATTGG ACAAGACTAA AAAGATCAGA TTTCATAGCA AATCTATCAC TTAGTCGACA   
  
  
+ TAAATATGTA AAAAACTCGA GATTTTATTA ATGGGTCTTG AATTTAATTA AAACCTTTAA ATTAAATAAA   
  
  
+ TATTATTCTC GTCACGAGTC AATCTCTTGT AACATCGTCA TTTCTTTAAG ATCAGTGACC TTGTTGAAAA   
  
  
+ CTAAAAACAC ACACAATTTT AATCATTCTT CTCTTTTTCG TGTATTCAAC TCACTTAAAT TCAACATATA   
  
  
+ GATTGAAAGA TTACAAGTCT AGACCTATTA TAATTCAATA AAGATTCTAA TTAGCAAGTT GAATAAGTGT   
  
  
+ TCTAAACACC AATTACTTTT TTCATCGATG ATTTCTATTC AAACTACGAT GTAAACTAAT ATCTAACAAT   
  
  
+ TGTCTTGAAT TTCGAACAAA TAATCACAGC TGCAGCTTTC AAAACATTAC CCAGAACCAT TTTCCTTGAC   
  
  
+ TTCTTTTTTA TTTTCTTATG CTTGAAATAA TACAGAACAG ATTAACGAGT TCTGTACACT GAAAATGAAA   
  
  
+ CAAGACTTTG AACTTCTAAC TTGGTATATT CTATTTTGTT ATACCGTCCC ATCATGTTAA GACTGGAAAA   
  
  
+ CTAATTAAAA GGTATTTCAT GAGATTATGA TGCATAATTA TTTTTAAAAT AATTTTTTAT TTAAAAATAT   
  
  
+ ATTAAAATAA AATTTCTTAT TTATTTTTAA TATCAACAGA TTAAAACTAT CATAAATCAC TAAATAATTA   
  
  
+ TCAATTTAAT ATTTTTCAAA CCAAAAACAC ATTTAAAATA CACTTCAAAA CTGTGTCAAC TTGAAGCTCA   
  
  
+ AAATTCACAG AGAAGTTAAA AACATTTAAC TGTAAATGCG AAGCTACACT GTCCAAACCT TCCAGTGACA   
  
  
+ ATGAAAAGCT TGCCACTTTG TATTGAAATA TTGGTACCAC GTGTAAGACA TATGCGAACA AGGCAATGGT   
  
  
+ GACGTCGGCA AGGTATAGGC AATCGTGGGA CCAATCCCGC CGCCGTACGT CACTAAACAC ACGTGCCTTA   
  
  
+ AAAACAAATT CCCAAGCCTT CCTAGCAACA AAGAACAAG  

- AAAAAAAAAT AGGCATTAAC TAATAAGTTT TGGAAAGGTC GAATTTTAAA AAAGGTATTA AATTTTTGAT   
  
  
- AATTTCCAAC TTTTGTGAAA AAAAAAGAGA CTCTAAATTT TGATTATGAT ATTTTTGAGA TTGAGATTGA   
  
  
- AAGGCAACAA ATAATAAATA GTGGCTTAGA AGAACTATTT ACATAAAGAA TTTCATATAA AATATATTTT   
  
  
- TATAAAAAAA ATAAAAATTT TAAAAGGAAA TTATATGTAG TACATTTCAA AGTTTAGCTA AATTTTTAAT   
  
  
- TTGTATTAAA AAAATTTTTT AATTGCCTTG GCGTTTTTTT ATTTCTCTAC CAGCGGTAGT TGGCACATCG   
  
  
- TTGGGAAAGA AAAGCCTCCT GAAAGTTTTA AGTCCGTAAA GGTACCTCTA AAAAACAGAA GAACTTCTGG   
  
  
- CCCTTGCCAA AGTTCGTTAG GGTGCTTTCC CCCGGACTTA GATCTGTTGC AACGATAGTT TGTCGAAGTA   
  
  
- GACACAATTT ATAAAATACC TAAACTAAAT GGACAGGGGA GACGGTCAGA AAAGTTTAGT TTAAACGGTG   
  
  
- TTCTTTGGTA CTGAAAATAA AGAAAGTACA CGTGTAGTAA TGGAATTCAC TATTGCTAAT TTCCACCTCC   
  
  
- AAACGGATTT TAATGGTCAA CTATAATTTA TGCGTTGTTG AATTAAAGTG TACTTAGGAG TGGGCATAAT   
  
  
- CGATCCCACG GTTTGAGAGA ACTAGTGGTA GGCACTAATT CGCCACGCAC CAAAATAACT AATTTTGATT   
  
  
- ACACTAAATT TAGACTAATA GGGGCTCAAT TAATACTTCC GAGCCTTGAG GTAAAAGAAG AGGAAAAAAA   
  
  
- GTGAAAAAAG AACCTGTCCC CTGCTTTCAA CCAAGTACAC CGTAGTTTTT TCATCACAAA TTATTAAATC   
  
  
- TACGAAAATT GTAAGTAACC TGTTCTGATT TTTCTAGTCT AAAGTATCGT TTAGATAGTG AATCAGCTGT   
  
  
- ATTTATACAT TTTTTGAGCT CTAAAATAAT TACCCAGAAC TTAAATTAAT TTTGGAAATT TAATTTATTT   
  
  
- ATAATAAGAG CAGTGCTCAG TTAGAGAACA TTGTAGCAGT AAAGAAATTC TAGTCACTGG AACAACTTTT   
  
  
- GATTTTTGTG TGTGTTAAAA TTAGTAAGAA GAGAAAAAGC ACATAAGTTG AGTGAATTTA AGTTGTATAT   
  
  
- CTAACTTTCT AATGTTCAGA TCTGGATAAT ATTAAGTTAT TTCTAAGATT AATCGTTCAA CTTATTCACA   
  
  
- AGATTTGTGG TTAATGAAAA AAGTAGCTAC TAAAGATAAG TTTGATGCTA CATTTGATTA TAGATTGTTA   
  
  
- ACAGAACTTA AAGCTTGTTT ATTAGTGTCG ACGTCGAAAG TTTTGTAATG GGTCTTGGTA AAAGGAACTG   
  
  
- AAGAAAAAAT AAAAGAATAC GAACTTTATT ATGTCTTGTC TAATTGCTCA AGACATGTGA CTTTTACTTT   
  
  
- GTTCTGAAAC TTGAAGATTG AACCATATAA GATAAAACAA TATGGCAGGG TAGTACAATT CTGACCTTTT   
  
  
- GATTAATTTT CCATAAAGTA CTCTAATACT ACGTATTAAT AAAAATTTTA TTAAAAAATA AATTTTTATA   
  
  
- TAATTTTATT TTAAAGAATA AATAAAAATT ATAGTTGTCT AATTTTGATA GTATTTAGTG ATTTATTAAT   
  
  
- AGTTAAATTA TAAAAAGTTT GGTTTTTGTG TAAATTTTAT GTGAAGTTTT GACACAGTTG AACTTCGAGT   
  
  
- TTTAAGTGTC TCTTCAATTT TTGTAAATTG ACATTTACGC TTCGATGTGA CAGGTTTGGA AGGTCACTGT   
  
  
- TACTTTTCGA ACGGTGAAAC ATAACTTTAT AACCATGGTG CACATTCTGT ATACGCTTGT TCCGTTACCA   
  
  
- CTGCAGCCGT TCCATATCCG TTAGCACCCT GGTTAGGGCG GCGGCATGCA GTGATTTGTG TGCACGGAAT   
  
  
- TTTTGTTTAA GGGTTCGGAA GGATCGTTGT TTCTTGTTC

+     Box 4

| Site Name | Organism | Position | Strand | Matrix score. | sequence | function |
| --- | --- | --- | --- | --- | --- | --- |
| Box 4 | Petroselinum crispum | 1007 | - | 6 | ATTAAT | part of a conserved DNA module involved in light responsiveness |

>Potri.002G039100.1   
+ TTTTTTTTTA TCCGTAATTG ATTATTCAAA ACCTTTCCAG CTTAAAATTT TTTCCATAAT TTAAAAACTA   
  
  
+ TTAAAGGTTG AAAACACTTT TTTTTTCTCT GAGATTTAAA ACTAATACTA TAAAAACTCT AACTCTAACT   
  
  
+ TTCCGTTGTT TATTATTTAT CACCGAATCT TCTTGATAAA TGTATTTCTT AAAGTATATT TTATATAAAA   
  
  
+ ATATTTTTTT TATTTTTAAA ATTTTCCTTT AATATACATC ATGTAAAGTT TCAAATCGAT TTAAAAATTA   
  
  
+ AACATAATTT TTTTAAAAAA TTAACGGAAC CGCAAAAAAA TAAAGAGATG GTCGCCATCA ACCGTGTAGC   
  
  
+ AACCCTTTCT TTTCGGAGGA CTTTCAAAAT TCAGGCATTT CCATGGAGAT TTTTTGTCTT CTTGAAGACC   
  
  
+ GGGAACGGTT TCAAGCAATC CCACGAAAGG GGGCCTGAAT CTAGACAACG TTGCTATCAA ACAGCTTCAT   
  
  
+ CTGTGTTAAA TATTTTATGG ATTTGATTTA CCTGTCCCCT CTGCCAGTCT TTTCAAATCA AATTTGCCAC   
  
  
+ AAGAAACCAT GACTTTTATT TCTTTCATGT GCACATCATT ACCTTAAGTG ATAACGATTA AAGGTGGAGG   
  
  
+ TTTGCCTAAA ATTACCAGTT GATATTAAAT ACGCAACAAC TTAATTTCAC ATGAATCCTC ACCCGTATTA   
  
  
+ GCTAGGGTGC CAAACTCTCT TGATCACCAT CCGTGATTAA GCGGTGCGTG GTTTTATTGA TTAAAACTAA   
  
  
+ TGTGATTTAA ATCTGATTAT CCCCGAGTTA ATTATGAAGG CTCGGAACTC CATTTTCTTC TCCTTTTTTT   
  
  
+ CACTTTTTTC TTGGACAGGG GACGAAAGTT GGTTCATGTG GCATCAAAAA AGTAGTGTTT AATAATTTAG   
  
  
+ ATGCTTTTAA CATTCATTGG ACAAGACTAA AAAGATCAGA TTTCATAGCA AATCTATCAC TTAGTCGACA   
  
  
+ TAAATATGTA AAAAACTCGA GATTTTATTA ATGGGTCTTG AATTTAATTA AAACCTTTAA ATTAAATAAA   
  
  
+ TATTATTCTC GTCACGAGTC AATCTCTTGT AACATCGTCA TTTCTTTAAG ATCAGTGACC TTGTTGAAAA   
  
  
+ CTAAAAACAC ACACAATTTT AATCATTCTT CTCTTTTTCG TGTATTCAAC TCACTTAAAT TCAACATATA   
  
  
+ GATTGAAAGA TTACAAGTCT AGACCTATTA TAATTCAATA AAGATTCTAA TTAGCAAGTT GAATAAGTGT   
  
  
+ TCTAAACACC AATTACTTTT TTCATCGATG ATTTCTATTC AAACTACGAT GTAAACTAAT ATCTAACAAT   
  
  
+ TGTCTTGAAT TTCGAACAAA TAATCACAGC TGCAGCTTTC AAAACATTAC CCAGAACCAT TTTCCTTGAC   
  
  
+ TTCTTTTTTA TTTTCTTATG CTTGAAATAA TACAGAACAG ATTAACGAGT TCTGTACACT GAAAATGAAA   
  
  
+ CAAGACTTTG AACTTCTAAC TTGGTATATT CTATTTTGTT ATACCGTCCC ATCATGTTAA GACTGGAAAA   
  
  
+ CTAATTAAAA GGTATTTCAT GAGATTATGA TGCATAATTA TTTTTAAAAT AATTTTTTAT TTAAAAATAT   
  
  
+ ATTAAAATAA AATTTCTTAT TTATTTTTAA TATCAACAGA TTAAAACTAT CATAAATCAC TAAATAATTA   
  
  
+ TCAATTTAAT ATTTTTCAAA CCAAAAACAC ATTTAAAATA CACTTCAAAA CTGTGTCAAC TTGAAGCTCA   
  
  
+ AAATTCACAG AGAAGTTAAA AACATTTAAC TGTAAATGCG AAGCTACACT GTCCAAACCT TCCAGTGACA   
  
  
+ ATGAAAAGCT TGCCACTTTG TATTGAAATA TTGGTACCAC GTGTAAGACA TATGCGAACA AGGCAATGGT   
  
  
+ GACGTCGGCA AGGTATAGGC AATCGTGGGA CCAATCCCGC CGCCGTACGT CACTAAACAC ACGTGCCTTA   
  
  
+ AAAACAAATT CCCAAGCCTT CCTAGCAACA AAGAACAAG  

- AAAAAAAAAT AGGCATTAAC TAATAAGTTT TGGAAAGGTC GAATTTTAAA AAAGGTATTA AATTTTTGAT   
  
  
- AATTTCCAAC TTTTGTGAAA AAAAAAGAGA CTCTAAATTT TGATTATGAT ATTTTTGAGA TTGAGATTGA   
  
  
- AAGGCAACAA ATAATAAATA GTGGCTTAGA AGAACTATTT ACATAAAGAA TTTCATATAA AATATATTTT   
  
  
- TATAAAAAAA ATAAAAATTT TAAAAGGAAA TTATATGTAG TACATTTCAA AGTTTAGCTA AATTTTTAAT   
  
  
- TTGTATTAAA AAAATTTTTT AATTGCCTTG GCGTTTTTTT ATTTCTCTAC CAGCGGTAGT TGGCACATCG   
  
  
- TTGGGAAAGA AAAGCCTCCT GAAAGTTTTA AGTCCGTAAA GGTACCTCTA AAAAACAGAA GAACTTCTGG   
  
  
- CCCTTGCCAA AGTTCGTTAG GGTGCTTTCC CCCGGACTTA GATCTGTTGC AACGATAGTT TGTCGAAGTA   
  
  
- GACACAATTT ATAAAATACC TAAACTAAAT GGACAGGGGA GACGGTCAGA AAAGTTTAGT TTAAACGGTG   
  
  
- TTCTTTGGTA CTGAAAATAA AGAAAGTACA CGTGTAGTAA TGGAATTCAC TATTGCTAAT TTCCACCTCC   
  
  
- AAACGGATTT TAATGGTCAA CTATAATTTA TGCGTTGTTG AATTAAAGTG TACTTAGGAG TGGGCATAAT   
  
  
- CGATCCCACG GTTTGAGAGA ACTAGTGGTA GGCACTAATT CGCCACGCAC CAAAATAACT AATTTTGATT   
  
  
- ACACTAAATT TAGACTAATA GGGGCTCAAT TAATACTTCC GAGCCTTGAG GTAAAAGAAG AGGAAAAAAA   
  
  
- GTGAAAAAAG AACCTGTCCC CTGCTTTCAA CCAAGTACAC CGTAGTTTTT TCATCACAAA TTATTAAATC   
  
  
- TACGAAAATT GTAAGTAACC TGTTCTGATT TTTCTAGTCT AAAGTATCGT TTAGATAGTG AATCAGCTGT   
  
  
- ATTTATACAT TTTTTGAGCT CTAAAATAAT TACCCAGAAC TTAAATTAAT TTTGGAAATT TAATTTATTT   
  
  
- ATAATAAGAG CAGTGCTCAG TTAGAGAACA TTGTAGCAGT AAAGAAATTC TAGTCACTGG AACAACTTTT   
  
  
- GATTTTTGTG TGTGTTAAAA TTAGTAAGAA GAGAAAAAGC ACATAAGTTG AGTGAATTTA AGTTGTATAT   
  
  
- CTAACTTTCT AATGTTCAGA TCTGGATAAT ATTAAGTTAT TTCTAAGATT AATCGTTCAA CTTATTCACA   
  
  
- AGATTTGTGG TTAATGAAAA AAGTAGCTAC TAAAGATAAG TTTGATGCTA CATTTGATTA TAGATTGTTA   
  
  
- ACAGAACTTA AAGCTTGTTT ATTAGTGTCG ACGTCGAAAG TTTTGTAATG GGTCTTGGTA AAAGGAACTG   
  
  
- AAGAAAAAAT AAAAGAATAC GAACTTTATT ATGTCTTGTC TAATTGCTCA AGACATGTGA CTTTTACTTT   
  
  
- GTTCTGAAAC TTGAAGATTG AACCATATAA GATAAAACAA TATGGCAGGG TAGTACAATT CTGACCTTTT   
  
  
- GATTAATTTT CCATAAAGTA CTCTAATACT ACGTATTAAT AAAAATTTTA TTAAAAAATA AATTTTTATA   
  
  
- TAATTTTATT TTAAAGAATA AATAAAAATT ATAGTTGTCT AATTTTGATA GTATTTAGTG ATTTATTAAT   
  
  
- AGTTAAATTA TAAAAAGTTT GGTTTTTGTG TAAATTTTAT GTGAAGTTTT GACACAGTTG AACTTCGAGT   
  
  
- TTTAAGTGTC TCTTCAATTT TTGTAAATTG ACATTTACGC TTCGATGTGA CAGGTTTGGA AGGTCACTGT   
  
  
- TACTTTTCGA ACGGTGAAAC ATAACTTTAT AACCATGGTG CACATTCTGT ATACGCTTGT TCCGTTACCA   
  
  
- CTGCAGCCGT TCCATATCCG TTAGCACCCT GGTTAGGGCG GCGGCATGCA GTGATTTGTG TGCACGGAAT   
  
  
- TTTTGTTTAA GGGTTCGGAA GGATCGTTGT TTCTTGTTC

+     CAAT-box

| Site Name | Organism | Position | Strand | Matrix score. | sequence | function |
| --- | --- | --- | --- | --- | --- | --- |
| CAAT-box | Nicotiana glutinosa | 17 | - | 4 | CAAT |  |
| CAAT-box | Pisum sativum | 262 | + | 5 | CAAAT | common cis-acting element in promoter and enhancer regions |
| CAAT-box | Nicotiana glutinosa | 436 | + | 4 | CAAT |  |
| CAAT-box | Pisum sativum | 511 | - | 5 | CAAAT | common cis-acting element in promoter and enhancer regions |
| CAAT-box | Pisum sativum | 544 | + | 5 | CAAAT | common cis-acting element in promoter and enhancer regions |
| CAAT-box | Pisum sativum | 549 | + | 5 | CAAAT | common cis-acting element in promoter and enhancer regions |
| CAAT-box | Pisum sativum | 552 | - | 5 | CAAAT | common cis-acting element in promoter and enhancer regions |
| CAAT-box | Nicotiana glutinosa | 756 | - | 4 | CAAT |  |
| CAAT-box | Arabidopsis thaliana | 926 | - | 5 | CCAAT | common cis-acting element in promoter and enhancer regions |
| CAAT-box | Pisum sativum | 959 | + | 5 | CAAAT | common cis-acting element in promoter and enhancer regions |
| CAAT-box | Nicotiana glutinosa | 1070 | + | 4 | CAAT |  |
| CAAT-box | Nicotiana glutinosa | 1134 | + | 4 | CAAT |  |
| CAAT-box | Nicotiana glutinosa | 1192 | - | 4 | CAAT |  |
| CAAT-box | Nicotiana glutinosa | 1226 | + | 4 | CAAT |  |
| CAAT-box | Arabidopsis thaliana | 1269 | + | 5 | CCAAT | common cis-acting element in promoter and enhancer regions |
| CAAT-box | Nicotiana glutinosa | 1270 | + | 4 | CAAT |  |
| CAAT-box | Nicotiana glutinosa | 1327 | + | 4 | CAAT |  |
| CAAT-box | Nicotiana glutinosa | 1329 | - | 4 | CAAT |  |
| CAAT-box | Pisum sativum | 1347 | + | 5 | CAAAT | common cis-acting element in promoter and enhancer regions |
| CAAT-box | Nicotiana glutinosa | 1682 | + | 4 | CAAT |  |
| CAAT-box | Nicotiana glutinosa | 1819 | + | 4 | CAAT |  |
| CAAT-box | Nicotiana glutinosa | 1842 | - | 4 | CAAT |  |
| CAAT-box | Arabidopsis thaliana | 1850 | - | 5 | CCAAT | common cis-acting element in promoter and enhancer regions |
| CAAT-box | Nicotiana glutinosa | 1884 | + | 4 | CAAT |  |
| CAAT-box | Nicotiana glutinosa | 1910 | + | 4 | CAAT |  |
| CAAT-box | Arabidopsis thaliana | 1921 | + | 5 | CCAAT | common cis-acting element in promoter and enhancer regions |
| CAAT-box | Nicotiana glutinosa | 1922 | + | 4 | CAAT |  |
| CAAT-box | Pisum sativum | 1965 | + | 5 | CAAAT | common cis-acting element in promoter and enhancer regions |

>Potri.002G039100.1   
+ TTTTTTTTTA TCCGTAATTG ATTATTCAAA ACCTTTCCAG CTTAAAATTT TTTCCATAAT TTAAAAACTA   
  
  
+ TTAAAGGTTG AAAACACTTT TTTTTTCTCT GAGATTTAAA ACTAATACTA TAAAAACTCT AACTCTAACT   
  
  
+ TTCCGTTGTT TATTATTTAT CACCGAATCT TCTTGATAAA TGTATTTCTT AAAGTATATT TTATATAAAA   
  
  
+ ATATTTTTTT TATTTTTAAA ATTTTCCTTT AATATACATC ATGTAAAGTT TCAAATCGAT TTAAAAATTA   
  
  
+ AACATAATTT TTTTAAAAAA TTAACGGAAC CGCAAAAAAA TAAAGAGATG GTCGCCATCA ACCGTGTAGC   
  
  
+ AACCCTTTCT TTTCGGAGGA CTTTCAAAAT TCAGGCATTT CCATGGAGAT TTTTTGTCTT CTTGAAGACC   
  
  
+ GGGAACGGTT TCAAGCAATC CCACGAAAGG GGGCCTGAAT CTAGACAACG TTGCTATCAA ACAGCTTCAT   
  
  
+ CTGTGTTAAA TATTTTATGG ATTTGATTTA CCTGTCCCCT CTGCCAGTCT TTTCAAATCA AATTTGCCAC   
  
  
+ AAGAAACCAT GACTTTTATT TCTTTCATGT GCACATCATT ACCTTAAGTG ATAACGATTA AAGGTGGAGG   
  
  
+ TTTGCCTAAA ATTACCAGTT GATATTAAAT ACGCAACAAC TTAATTTCAC ATGAATCCTC ACCCGTATTA   
  
  
+ GCTAGGGTGC CAAACTCTCT TGATCACCAT CCGTGATTAA GCGGTGCGTG GTTTTATTGA TTAAAACTAA   
  
  
+ TGTGATTTAA ATCTGATTAT CCCCGAGTTA ATTATGAAGG CTCGGAACTC CATTTTCTTC TCCTTTTTTT   
  
  
+ CACTTTTTTC TTGGACAGGG GACGAAAGTT GGTTCATGTG GCATCAAAAA AGTAGTGTTT AATAATTTAG   
  
  
+ ATGCTTTTAA CATTCATTGG ACAAGACTAA AAAGATCAGA TTTCATAGCA AATCTATCAC TTAGTCGACA   
  
  
+ TAAATATGTA AAAAACTCGA GATTTTATTA ATGGGTCTTG AATTTAATTA AAACCTTTAA ATTAAATAAA   
  
  
+ TATTATTCTC GTCACGAGTC AATCTCTTGT AACATCGTCA TTTCTTTAAG ATCAGTGACC TTGTTGAAAA   
  
  
+ CTAAAAACAC ACACAATTTT AATCATTCTT CTCTTTTTCG TGTATTCAAC TCACTTAAAT TCAACATATA   
  
  
+ GATTGAAAGA TTACAAGTCT AGACCTATTA TAATTCAATA AAGATTCTAA TTAGCAAGTT GAATAAGTGT   
  
  
+ TCTAAACACC AATTACTTTT TTCATCGATG ATTTCTATTC AAACTACGAT GTAAACTAAT ATCTAACAAT   
  
  
+ TGTCTTGAAT TTCGAACAAA TAATCACAGC TGCAGCTTTC AAAACATTAC CCAGAACCAT TTTCCTTGAC   
  
  
+ TTCTTTTTTA TTTTCTTATG CTTGAAATAA TACAGAACAG ATTAACGAGT TCTGTACACT GAAAATGAAA   
  
  
+ CAAGACTTTG AACTTCTAAC TTGGTATATT CTATTTTGTT ATACCGTCCC ATCATGTTAA GACTGGAAAA   
  
  
+ CTAATTAAAA GGTATTTCAT GAGATTATGA TGCATAATTA TTTTTAAAAT AATTTTTTAT TTAAAAATAT   
  
  
+ ATTAAAATAA AATTTCTTAT TTATTTTTAA TATCAACAGA TTAAAACTAT CATAAATCAC TAAATAATTA   
  
  
+ TCAATTTAAT ATTTTTCAAA CCAAAAACAC ATTTAAAATA CACTTCAAAA CTGTGTCAAC TTGAAGCTCA   
  
  
+ AAATTCACAG AGAAGTTAAA AACATTTAAC TGTAAATGCG AAGCTACACT GTCCAAACCT TCCAGTGACA   
  
  
+ ATGAAAAGCT TGCCACTTTG TATTGAAATA TTGGTACCAC GTGTAAGACA TATGCGAACA AGGCAATGGT   
  
  
+ GACGTCGGCA AGGTATAGGC AATCGTGGGA CCAATCCCGC CGCCGTACGT CACTAAACAC ACGTGCCTTA   
  
  
+ AAAACAAATT CCCAAGCCTT CCTAGCAACA AAGAACAAG  

- AAAAAAAAAT AGGCATTAAC TAATAAGTTT TGGAAAGGTC GAATTTTAAA AAAGGTATTA AATTTTTGAT   
  
  
- AATTTCCAAC TTTTGTGAAA AAAAAAGAGA CTCTAAATTT TGATTATGAT ATTTTTGAGA TTGAGATTGA   
  
  
- AAGGCAACAA ATAATAAATA GTGGCTTAGA AGAACTATTT ACATAAAGAA TTTCATATAA AATATATTTT   
  
  
- TATAAAAAAA ATAAAAATTT TAAAAGGAAA TTATATGTAG TACATTTCAA AGTTTAGCTA AATTTTTAAT   
  
  
- TTGTATTAAA AAAATTTTTT AATTGCCTTG GCGTTTTTTT ATTTCTCTAC CAGCGGTAGT TGGCACATCG   
  
  
- TTGGGAAAGA AAAGCCTCCT GAAAGTTTTA AGTCCGTAAA GGTACCTCTA AAAAACAGAA GAACTTCTGG   
  
  
- CCCTTGCCAA AGTTCGTTAG GGTGCTTTCC CCCGGACTTA GATCTGTTGC AACGATAGTT TGTCGAAGTA   
  
  
- GACACAATTT ATAAAATACC TAAACTAAAT GGACAGGGGA GACGGTCAGA AAAGTTTAGT TTAAACGGTG   
  
  
- TTCTTTGGTA CTGAAAATAA AGAAAGTACA CGTGTAGTAA TGGAATTCAC TATTGCTAAT TTCCACCTCC   
  
  
- AAACGGATTT TAATGGTCAA CTATAATTTA TGCGTTGTTG AATTAAAGTG TACTTAGGAG TGGGCATAAT   
  
  
- CGATCCCACG GTTTGAGAGA ACTAGTGGTA GGCACTAATT CGCCACGCAC CAAAATAACT AATTTTGATT   
  
  
- ACACTAAATT TAGACTAATA GGGGCTCAAT TAATACTTCC GAGCCTTGAG GTAAAAGAAG AGGAAAAAAA   
  
  
- GTGAAAAAAG AACCTGTCCC CTGCTTTCAA CCAAGTACAC CGTAGTTTTT TCATCACAAA TTATTAAATC   
  
  
- TACGAAAATT GTAAGTAACC TGTTCTGATT TTTCTAGTCT AAAGTATCGT TTAGATAGTG AATCAGCTGT   
  
  
- ATTTATACAT TTTTTGAGCT CTAAAATAAT TACCCAGAAC TTAAATTAAT TTTGGAAATT TAATTTATTT   
  
  
- ATAATAAGAG CAGTGCTCAG TTAGAGAACA TTGTAGCAGT AAAGAAATTC TAGTCACTGG AACAACTTTT   
  
  
- GATTTTTGTG TGTGTTAAAA TTAGTAAGAA GAGAAAAAGC ACATAAGTTG AGTGAATTTA AGTTGTATAT   
  
  
- CTAACTTTCT AATGTTCAGA TCTGGATAAT ATTAAGTTAT TTCTAAGATT AATCGTTCAA CTTATTCACA   
  
  
- AGATTTGTGG TTAATGAAAA AAGTAGCTAC TAAAGATAAG TTTGATGCTA CATTTGATTA TAGATTGTTA   
  
  
- ACAGAACTTA AAGCTTGTTT ATTAGTGTCG ACGTCGAAAG TTTTGTAATG GGTCTTGGTA AAAGGAACTG   
  
  
- AAGAAAAAAT AAAAGAATAC GAACTTTATT ATGTCTTGTC TAATTGCTCA AGACATGTGA CTTTTACTTT   
  
  
- GTTCTGAAAC TTGAAGATTG AACCATATAA GATAAAACAA TATGGCAGGG TAGTACAATT CTGACCTTTT   
  
  
- GATTAATTTT CCATAAAGTA CTCTAATACT ACGTATTAAT AAAAATTTTA TTAAAAAATA AATTTTTATA   
  
  
- TAATTTTATT TTAAAGAATA AATAAAAATT ATAGTTGTCT AATTTTGATA GTATTTAGTG ATTTATTAAT   
  
  
- AGTTAAATTA TAAAAAGTTT GGTTTTTGTG TAAATTTTAT GTGAAGTTTT GACACAGTTG AACTTCGAGT   
  
  
- TTTAAGTGTC TCTTCAATTT TTGTAAATTG ACATTTACGC TTCGATGTGA CAGGTTTGGA AGGTCACTGT   
  
  
- TACTTTTCGA ACGGTGAAAC ATAACTTTAT AACCATGGTG CACATTCTGT ATACGCTTGT TCCGTTACCA   
  
  
- CTGCAGCCGT TCCATATCCG TTAGCACCCT GGTTAGGGCG GCGGCATGCA GTGATTTGTG TGCACGGAAT   
  
  
- TTTTGTTTAA GGGTTCGGAA GGATCGTTGT TTCTTGTTC

+     CARE

| Site Name | Organism | Position | Strand | Matrix score. | sequence | function |
| --- | --- | --- | --- | --- | --- | --- |
| CARE | Oryza sativa | 1167 | + | 8 | CAACTCAC |  |

>Potri.002G039100.1   
+ TTTTTTTTTA TCCGTAATTG ATTATTCAAA ACCTTTCCAG CTTAAAATTT TTTCCATAAT TTAAAAACTA   
  
  
+ TTAAAGGTTG AAAACACTTT TTTTTTCTCT GAGATTTAAA ACTAATACTA TAAAAACTCT AACTCTAACT   
  
  
+ TTCCGTTGTT TATTATTTAT CACCGAATCT TCTTGATAAA TGTATTTCTT AAAGTATATT TTATATAAAA   
  
  
+ ATATTTTTTT TATTTTTAAA ATTTTCCTTT AATATACATC ATGTAAAGTT TCAAATCGAT TTAAAAATTA   
  
  
+ AACATAATTT TTTTAAAAAA TTAACGGAAC CGCAAAAAAA TAAAGAGATG GTCGCCATCA ACCGTGTAGC   
  
  
+ AACCCTTTCT TTTCGGAGGA CTTTCAAAAT TCAGGCATTT CCATGGAGAT TTTTTGTCTT CTTGAAGACC   
  
  
+ GGGAACGGTT TCAAGCAATC CCACGAAAGG GGGCCTGAAT CTAGACAACG TTGCTATCAA ACAGCTTCAT   
  
  
+ CTGTGTTAAA TATTTTATGG ATTTGATTTA CCTGTCCCCT CTGCCAGTCT TTTCAAATCA AATTTGCCAC   
  
  
+ AAGAAACCAT GACTTTTATT TCTTTCATGT GCACATCATT ACCTTAAGTG ATAACGATTA AAGGTGGAGG   
  
  
+ TTTGCCTAAA ATTACCAGTT GATATTAAAT ACGCAACAAC TTAATTTCAC ATGAATCCTC ACCCGTATTA   
  
  
+ GCTAGGGTGC CAAACTCTCT TGATCACCAT CCGTGATTAA GCGGTGCGTG GTTTTATTGA TTAAAACTAA   
  
  
+ TGTGATTTAA ATCTGATTAT CCCCGAGTTA ATTATGAAGG CTCGGAACTC CATTTTCTTC TCCTTTTTTT   
  
  
+ CACTTTTTTC TTGGACAGGG GACGAAAGTT GGTTCATGTG GCATCAAAAA AGTAGTGTTT AATAATTTAG   
  
  
+ ATGCTTTTAA CATTCATTGG ACAAGACTAA AAAGATCAGA TTTCATAGCA AATCTATCAC TTAGTCGACA   
  
  
+ TAAATATGTA AAAAACTCGA GATTTTATTA ATGGGTCTTG AATTTAATTA AAACCTTTAA ATTAAATAAA   
  
  
+ TATTATTCTC GTCACGAGTC AATCTCTTGT AACATCGTCA TTTCTTTAAG ATCAGTGACC TTGTTGAAAA   
  
  
+ CTAAAAACAC ACACAATTTT AATCATTCTT CTCTTTTTCG TGTATTCAAC TCACTTAAAT TCAACATATA   
  
  
+ GATTGAAAGA TTACAAGTCT AGACCTATTA TAATTCAATA AAGATTCTAA TTAGCAAGTT GAATAAGTGT   
  
  
+ TCTAAACACC AATTACTTTT TTCATCGATG ATTTCTATTC AAACTACGAT GTAAACTAAT ATCTAACAAT   
  
  
+ TGTCTTGAAT TTCGAACAAA TAATCACAGC TGCAGCTTTC AAAACATTAC CCAGAACCAT TTTCCTTGAC   
  
  
+ TTCTTTTTTA TTTTCTTATG CTTGAAATAA TACAGAACAG ATTAACGAGT TCTGTACACT GAAAATGAAA   
  
  
+ CAAGACTTTG AACTTCTAAC TTGGTATATT CTATTTTGTT ATACCGTCCC ATCATGTTAA GACTGGAAAA   
  
  
+ CTAATTAAAA GGTATTTCAT GAGATTATGA TGCATAATTA TTTTTAAAAT AATTTTTTAT TTAAAAATAT   
  
  
+ ATTAAAATAA AATTTCTTAT TTATTTTTAA TATCAACAGA TTAAAACTAT CATAAATCAC TAAATAATTA   
  
  
+ TCAATTTAAT ATTTTTCAAA CCAAAAACAC ATTTAAAATA CACTTCAAAA CTGTGTCAAC TTGAAGCTCA   
  
  
+ AAATTCACAG AGAAGTTAAA AACATTTAAC TGTAAATGCG AAGCTACACT GTCCAAACCT TCCAGTGACA   
  
  
+ ATGAAAAGCT TGCCACTTTG TATTGAAATA TTGGTACCAC GTGTAAGACA TATGCGAACA AGGCAATGGT   
  
  
+ GACGTCGGCA AGGTATAGGC AATCGTGGGA CCAATCCCGC CGCCGTACGT CACTAAACAC ACGTGCCTTA   
  
  
+ AAAACAAATT CCCAAGCCTT CCTAGCAACA AAGAACAAG  

- AAAAAAAAAT AGGCATTAAC TAATAAGTTT TGGAAAGGTC GAATTTTAAA AAAGGTATTA AATTTTTGAT   
  
  
- AATTTCCAAC TTTTGTGAAA AAAAAAGAGA CTCTAAATTT TGATTATGAT ATTTTTGAGA TTGAGATTGA   
  
  
- AAGGCAACAA ATAATAAATA GTGGCTTAGA AGAACTATTT ACATAAAGAA TTTCATATAA AATATATTTT   
  
  
- TATAAAAAAA ATAAAAATTT TAAAAGGAAA TTATATGTAG TACATTTCAA AGTTTAGCTA AATTTTTAAT   
  
  
- TTGTATTAAA AAAATTTTTT AATTGCCTTG GCGTTTTTTT ATTTCTCTAC CAGCGGTAGT TGGCACATCG   
  
  
- TTGGGAAAGA AAAGCCTCCT GAAAGTTTTA AGTCCGTAAA GGTACCTCTA AAAAACAGAA GAACTTCTGG   
  
  
- CCCTTGCCAA AGTTCGTTAG GGTGCTTTCC CCCGGACTTA GATCTGTTGC AACGATAGTT TGTCGAAGTA   
  
  
- GACACAATTT ATAAAATACC TAAACTAAAT GGACAGGGGA GACGGTCAGA AAAGTTTAGT TTAAACGGTG   
  
  
- TTCTTTGGTA CTGAAAATAA AGAAAGTACA CGTGTAGTAA TGGAATTCAC TATTGCTAAT TTCCACCTCC   
  
  
- AAACGGATTT TAATGGTCAA CTATAATTTA TGCGTTGTTG AATTAAAGTG TACTTAGGAG TGGGCATAAT   
  
  
- CGATCCCACG GTTTGAGAGA ACTAGTGGTA GGCACTAATT CGCCACGCAC CAAAATAACT AATTTTGATT   
  
  
- ACACTAAATT TAGACTAATA GGGGCTCAAT TAATACTTCC GAGCCTTGAG GTAAAAGAAG AGGAAAAAAA   
  
  
- GTGAAAAAAG AACCTGTCCC CTGCTTTCAA CCAAGTACAC CGTAGTTTTT TCATCACAAA TTATTAAATC   
  
  
- TACGAAAATT GTAAGTAACC TGTTCTGATT TTTCTAGTCT AAAGTATCGT TTAGATAGTG AATCAGCTGT   
  
  
- ATTTATACAT TTTTTGAGCT CTAAAATAAT TACCCAGAAC TTAAATTAAT TTTGGAAATT TAATTTATTT   
  
  
- ATAATAAGAG CAGTGCTCAG TTAGAGAACA TTGTAGCAGT AAAGAAATTC TAGTCACTGG AACAACTTTT   
  
  
- GATTTTTGTG TGTGTTAAAA TTAGTAAGAA GAGAAAAAGC ACATAAGTTG AGTGAATTTA AGTTGTATAT   
  
  
- CTAACTTTCT AATGTTCAGA TCTGGATAAT ATTAAGTTAT TTCTAAGATT AATCGTTCAA CTTATTCACA   
  
  
- AGATTTGTGG TTAATGAAAA AAGTAGCTAC TAAAGATAAG TTTGATGCTA CATTTGATTA TAGATTGTTA   
  
  
- ACAGAACTTA AAGCTTGTTT ATTAGTGTCG ACGTCGAAAG TTTTGTAATG GGTCTTGGTA AAAGGAACTG   
  
  
- AAGAAAAAAT AAAAGAATAC GAACTTTATT ATGTCTTGTC TAATTGCTCA AGACATGTGA CTTTTACTTT   
  
  
- GTTCTGAAAC TTGAAGATTG AACCATATAA GATAAAACAA TATGGCAGGG TAGTACAATT CTGACCTTTT   
  
  
- GATTAATTTT CCATAAAGTA CTCTAATACT ACGTATTAAT AAAAATTTTA TTAAAAAATA AATTTTTATA   
  
  
- TAATTTTATT TTAAAGAATA AATAAAAATT ATAGTTGTCT AATTTTGATA GTATTTAGTG ATTTATTAAT   
  
  
- AGTTAAATTA TAAAAAGTTT GGTTTTTGTG TAAATTTTAT GTGAAGTTTT GACACAGTTG AACTTCGAGT   
  
  
- TTTAAGTGTC TCTTCAATTT TTGTAAATTG ACATTTACGC TTCGATGTGA CAGGTTTGGA AGGTCACTGT   
  
  
- TACTTTTCGA ACGGTGAAAC ATAACTTTAT AACCATGGTG CACATTCTGT ATACGCTTGT TCCGTTACCA   
  
  
- CTGCAGCCGT TCCATATCCG TTAGCACCCT GGTTAGGGCG GCGGCATGCA GTGATTTGTG TGCACGGAAT   
  
  
- TTTTGTTTAA GGGTTCGGAA GGATCGTTGT TTCTTGTTC

+     CAT-box

| Site Name | Organism | Position | Strand | Matrix score. | sequence | function |
| --- | --- | --- | --- | --- | --- | --- |
| CAT-box | Arabidopsis thaliana | 1832 | + | 6 | GCCACT | cis-acting regulatory element related to meristem expression |

>Potri.002G039100.1   
+ TTTTTTTTTA TCCGTAATTG ATTATTCAAA ACCTTTCCAG CTTAAAATTT TTTCCATAAT TTAAAAACTA   
  
  
+ TTAAAGGTTG AAAACACTTT TTTTTTCTCT GAGATTTAAA ACTAATACTA TAAAAACTCT AACTCTAACT   
  
  
+ TTCCGTTGTT TATTATTTAT CACCGAATCT TCTTGATAAA TGTATTTCTT AAAGTATATT TTATATAAAA   
  
  
+ ATATTTTTTT TATTTTTAAA ATTTTCCTTT AATATACATC ATGTAAAGTT TCAAATCGAT TTAAAAATTA   
  
  
+ AACATAATTT TTTTAAAAAA TTAACGGAAC CGCAAAAAAA TAAAGAGATG GTCGCCATCA ACCGTGTAGC   
  
  
+ AACCCTTTCT TTTCGGAGGA CTTTCAAAAT TCAGGCATTT CCATGGAGAT TTTTTGTCTT CTTGAAGACC   
  
  
+ GGGAACGGTT TCAAGCAATC CCACGAAAGG GGGCCTGAAT CTAGACAACG TTGCTATCAA ACAGCTTCAT   
  
  
+ CTGTGTTAAA TATTTTATGG ATTTGATTTA CCTGTCCCCT CTGCCAGTCT TTTCAAATCA AATTTGCCAC   
  
  
+ AAGAAACCAT GACTTTTATT TCTTTCATGT GCACATCATT ACCTTAAGTG ATAACGATTA AAGGTGGAGG   
  
  
+ TTTGCCTAAA ATTACCAGTT GATATTAAAT ACGCAACAAC TTAATTTCAC ATGAATCCTC ACCCGTATTA   
  
  
+ GCTAGGGTGC CAAACTCTCT TGATCACCAT CCGTGATTAA GCGGTGCGTG GTTTTATTGA TTAAAACTAA   
  
  
+ TGTGATTTAA ATCTGATTAT CCCCGAGTTA ATTATGAAGG CTCGGAACTC CATTTTCTTC TCCTTTTTTT   
  
  
+ CACTTTTTTC TTGGACAGGG GACGAAAGTT GGTTCATGTG GCATCAAAAA AGTAGTGTTT AATAATTTAG   
  
  
+ ATGCTTTTAA CATTCATTGG ACAAGACTAA AAAGATCAGA TTTCATAGCA AATCTATCAC TTAGTCGACA   
  
  
+ TAAATATGTA AAAAACTCGA GATTTTATTA ATGGGTCTTG AATTTAATTA AAACCTTTAA ATTAAATAAA   
  
  
+ TATTATTCTC GTCACGAGTC AATCTCTTGT AACATCGTCA TTTCTTTAAG ATCAGTGACC TTGTTGAAAA   
  
  
+ CTAAAAACAC ACACAATTTT AATCATTCTT CTCTTTTTCG TGTATTCAAC TCACTTAAAT TCAACATATA   
  
  
+ GATTGAAAGA TTACAAGTCT AGACCTATTA TAATTCAATA AAGATTCTAA TTAGCAAGTT GAATAAGTGT   
  
  
+ TCTAAACACC AATTACTTTT TTCATCGATG ATTTCTATTC AAACTACGAT GTAAACTAAT ATCTAACAAT   
  
  
+ TGTCTTGAAT TTCGAACAAA TAATCACAGC TGCAGCTTTC AAAACATTAC CCAGAACCAT TTTCCTTGAC   
  
  
+ TTCTTTTTTA TTTTCTTATG CTTGAAATAA TACAGAACAG ATTAACGAGT TCTGTACACT GAAAATGAAA   
  
  
+ CAAGACTTTG AACTTCTAAC TTGGTATATT CTATTTTGTT ATACCGTCCC ATCATGTTAA GACTGGAAAA   
  
  
+ CTAATTAAAA GGTATTTCAT GAGATTATGA TGCATAATTA TTTTTAAAAT AATTTTTTAT TTAAAAATAT   
  
  
+ ATTAAAATAA AATTTCTTAT TTATTTTTAA TATCAACAGA TTAAAACTAT CATAAATCAC TAAATAATTA   
  
  
+ TCAATTTAAT ATTTTTCAAA CCAAAAACAC ATTTAAAATA CACTTCAAAA CTGTGTCAAC TTGAAGCTCA   
  
  
+ AAATTCACAG AGAAGTTAAA AACATTTAAC TGTAAATGCG AAGCTACACT GTCCAAACCT TCCAGTGACA   
  
  
+ ATGAAAAGCT TGCCACTTTG TATTGAAATA TTGGTACCAC GTGTAAGACA TATGCGAACA AGGCAATGGT   
  
  
+ GACGTCGGCA AGGTATAGGC AATCGTGGGA CCAATCCCGC CGCCGTACGT CACTAAACAC ACGTGCCTTA   
  
  
+ AAAACAAATT CCCAAGCCTT CCTAGCAACA AAGAACAAG  

- AAAAAAAAAT AGGCATTAAC TAATAAGTTT TGGAAAGGTC GAATTTTAAA AAAGGTATTA AATTTTTGAT   
  
  
- AATTTCCAAC TTTTGTGAAA AAAAAAGAGA CTCTAAATTT TGATTATGAT ATTTTTGAGA TTGAGATTGA   
  
  
- AAGGCAACAA ATAATAAATA GTGGCTTAGA AGAACTATTT ACATAAAGAA TTTCATATAA AATATATTTT   
  
  
- TATAAAAAAA ATAAAAATTT TAAAAGGAAA TTATATGTAG TACATTTCAA AGTTTAGCTA AATTTTTAAT   
  
  
- TTGTATTAAA AAAATTTTTT AATTGCCTTG GCGTTTTTTT ATTTCTCTAC CAGCGGTAGT TGGCACATCG   
  
  
- TTGGGAAAGA AAAGCCTCCT GAAAGTTTTA AGTCCGTAAA GGTACCTCTA AAAAACAGAA GAACTTCTGG   
  
  
- CCCTTGCCAA AGTTCGTTAG GGTGCTTTCC CCCGGACTTA GATCTGTTGC AACGATAGTT TGTCGAAGTA   
  
  
- GACACAATTT ATAAAATACC TAAACTAAAT GGACAGGGGA GACGGTCAGA AAAGTTTAGT TTAAACGGTG   
  
  
- TTCTTTGGTA CTGAAAATAA AGAAAGTACA CGTGTAGTAA TGGAATTCAC TATTGCTAAT TTCCACCTCC   
  
  
- AAACGGATTT TAATGGTCAA CTATAATTTA TGCGTTGTTG AATTAAAGTG TACTTAGGAG TGGGCATAAT   
  
  
- CGATCCCACG GTTTGAGAGA ACTAGTGGTA GGCACTAATT CGCCACGCAC CAAAATAACT AATTTTGATT   
  
  
- ACACTAAATT TAGACTAATA GGGGCTCAAT TAATACTTCC GAGCCTTGAG GTAAAAGAAG AGGAAAAAAA   
  
  
- GTGAAAAAAG AACCTGTCCC CTGCTTTCAA CCAAGTACAC CGTAGTTTTT TCATCACAAA TTATTAAATC   
  
  
- TACGAAAATT GTAAGTAACC TGTTCTGATT TTTCTAGTCT AAAGTATCGT TTAGATAGTG AATCAGCTGT   
  
  
- ATTTATACAT TTTTTGAGCT CTAAAATAAT TACCCAGAAC TTAAATTAAT TTTGGAAATT TAATTTATTT   
  
  
- ATAATAAGAG CAGTGCTCAG TTAGAGAACA TTGTAGCAGT AAAGAAATTC TAGTCACTGG AACAACTTTT   
  
  
- GATTTTTGTG TGTGTTAAAA TTAGTAAGAA GAGAAAAAGC ACATAAGTTG AGTGAATTTA AGTTGTATAT   
  
  
- CTAACTTTCT AATGTTCAGA TCTGGATAAT ATTAAGTTAT TTCTAAGATT AATCGTTCAA CTTATTCACA   
  
  
- AGATTTGTGG TTAATGAAAA AAGTAGCTAC TAAAGATAAG TTTGATGCTA CATTTGATTA TAGATTGTTA   
  
  
- ACAGAACTTA AAGCTTGTTT ATTAGTGTCG ACGTCGAAAG TTTTGTAATG GGTCTTGGTA AAAGGAACTG   
  
  
- AAGAAAAAAT AAAAGAATAC GAACTTTATT ATGTCTTGTC TAATTGCTCA AGACATGTGA CTTTTACTTT   
  
  
- GTTCTGAAAC TTGAAGATTG AACCATATAA GATAAAACAA TATGGCAGGG TAGTACAATT CTGACCTTTT   
  
  
- GATTAATTTT CCATAAAGTA CTCTAATACT ACGTATTAAT AAAAATTTTA TTAAAAAATA AATTTTTATA   
  
  
- TAATTTTATT TTAAAGAATA AATAAAAATT ATAGTTGTCT AATTTTGATA GTATTTAGTG ATTTATTAAT   
  
  
- AGTTAAATTA TAAAAAGTTT GGTTTTTGTG TAAATTTTAT GTGAAGTTTT GACACAGTTG AACTTCGAGT   
  
  
- TTTAAGTGTC TCTTCAATTT TTGTAAATTG ACATTTACGC TTCGATGTGA CAGGTTTGGA AGGTCACTGT   
  
  
- TACTTTTCGA ACGGTGAAAC ATAACTTTAT AACCATGGTG CACATTCTGT ATACGCTTGT TCCGTTACCA   
  
  
- CTGCAGCCGT TCCATATCCG TTAGCACCCT GGTTAGGGCG GCGGCATGCA GTGATTTGTG TGCACGGAAT   
  
  
- TTTTGTTTAA GGGTTCGGAA GGATCGTTGT TTCTTGTTC

+     CCAAT-box

| Site Name | Organism | Position | Strand | Matrix score. | sequence | function |
| --- | --- | --- | --- | --- | --- | --- |
| CCAAT-box | Hordeum vulgare | 143 | - | 6 | CAACGG | MYBHv1 binding site |

>Potri.002G039100.1   
+ TTTTTTTTTA TCCGTAATTG ATTATTCAAA ACCTTTCCAG CTTAAAATTT TTTCCATAAT TTAAAAACTA   
  
  
+ TTAAAGGTTG AAAACACTTT TTTTTTCTCT GAGATTTAAA ACTAATACTA TAAAAACTCT AACTCTAACT   
  
  
+ TTCCGTTGTT TATTATTTAT CACCGAATCT TCTTGATAAA TGTATTTCTT AAAGTATATT TTATATAAAA   
  
  
+ ATATTTTTTT TATTTTTAAA ATTTTCCTTT AATATACATC ATGTAAAGTT TCAAATCGAT TTAAAAATTA   
  
  
+ AACATAATTT TTTTAAAAAA TTAACGGAAC CGCAAAAAAA TAAAGAGATG GTCGCCATCA ACCGTGTAGC   
  
  
+ AACCCTTTCT TTTCGGAGGA CTTTCAAAAT TCAGGCATTT CCATGGAGAT TTTTTGTCTT CTTGAAGACC   
  
  
+ GGGAACGGTT TCAAGCAATC CCACGAAAGG GGGCCTGAAT CTAGACAACG TTGCTATCAA ACAGCTTCAT   
  
  
+ CTGTGTTAAA TATTTTATGG ATTTGATTTA CCTGTCCCCT CTGCCAGTCT TTTCAAATCA AATTTGCCAC   
  
  
+ AAGAAACCAT GACTTTTATT TCTTTCATGT GCACATCATT ACCTTAAGTG ATAACGATTA AAGGTGGAGG   
  
  
+ TTTGCCTAAA ATTACCAGTT GATATTAAAT ACGCAACAAC TTAATTTCAC ATGAATCCTC ACCCGTATTA   
  
  
+ GCTAGGGTGC CAAACTCTCT TGATCACCAT CCGTGATTAA GCGGTGCGTG GTTTTATTGA TTAAAACTAA   
  
  
+ TGTGATTTAA ATCTGATTAT CCCCGAGTTA ATTATGAAGG CTCGGAACTC CATTTTCTTC TCCTTTTTTT   
  
  
+ CACTTTTTTC TTGGACAGGG GACGAAAGTT GGTTCATGTG GCATCAAAAA AGTAGTGTTT AATAATTTAG   
  
  
+ ATGCTTTTAA CATTCATTGG ACAAGACTAA AAAGATCAGA TTTCATAGCA AATCTATCAC TTAGTCGACA   
  
  
+ TAAATATGTA AAAAACTCGA GATTTTATTA ATGGGTCTTG AATTTAATTA AAACCTTTAA ATTAAATAAA   
  
  
+ TATTATTCTC GTCACGAGTC AATCTCTTGT AACATCGTCA TTTCTTTAAG ATCAGTGACC TTGTTGAAAA   
  
  
+ CTAAAAACAC ACACAATTTT AATCATTCTT CTCTTTTTCG TGTATTCAAC TCACTTAAAT TCAACATATA   
  
  
+ GATTGAAAGA TTACAAGTCT AGACCTATTA TAATTCAATA AAGATTCTAA TTAGCAAGTT GAATAAGTGT   
  
  
+ TCTAAACACC AATTACTTTT TTCATCGATG ATTTCTATTC AAACTACGAT GTAAACTAAT ATCTAACAAT   
  
  
+ TGTCTTGAAT TTCGAACAAA TAATCACAGC TGCAGCTTTC AAAACATTAC CCAGAACCAT TTTCCTTGAC   
  
  
+ TTCTTTTTTA TTTTCTTATG CTTGAAATAA TACAGAACAG ATTAACGAGT TCTGTACACT GAAAATGAAA   
  
  
+ CAAGACTTTG AACTTCTAAC TTGGTATATT CTATTTTGTT ATACCGTCCC ATCATGTTAA GACTGGAAAA   
  
  
+ CTAATTAAAA GGTATTTCAT GAGATTATGA TGCATAATTA TTTTTAAAAT AATTTTTTAT TTAAAAATAT   
  
  
+ ATTAAAATAA AATTTCTTAT TTATTTTTAA TATCAACAGA TTAAAACTAT CATAAATCAC TAAATAATTA   
  
  
+ TCAATTTAAT ATTTTTCAAA CCAAAAACAC ATTTAAAATA CACTTCAAAA CTGTGTCAAC TTGAAGCTCA   
  
  
+ AAATTCACAG AGAAGTTAAA AACATTTAAC TGTAAATGCG AAGCTACACT GTCCAAACCT TCCAGTGACA   
  
  
+ ATGAAAAGCT TGCCACTTTG TATTGAAATA TTGGTACCAC GTGTAAGACA TATGCGAACA AGGCAATGGT   
  
  
+ GACGTCGGCA AGGTATAGGC AATCGTGGGA CCAATCCCGC CGCCGTACGT CACTAAACAC ACGTGCCTTA   
  
  
+ AAAACAAATT CCCAAGCCTT CCTAGCAACA AAGAACAAG  

- AAAAAAAAAT AGGCATTAAC TAATAAGTTT TGGAAAGGTC GAATTTTAAA AAAGGTATTA AATTTTTGAT   
  
  
- AATTTCCAAC TTTTGTGAAA AAAAAAGAGA CTCTAAATTT TGATTATGAT ATTTTTGAGA TTGAGATTGA   
  
  
- AAGGCAACAA ATAATAAATA GTGGCTTAGA AGAACTATTT ACATAAAGAA TTTCATATAA AATATATTTT   
  
  
- TATAAAAAAA ATAAAAATTT TAAAAGGAAA TTATATGTAG TACATTTCAA AGTTTAGCTA AATTTTTAAT   
  
  
- TTGTATTAAA AAAATTTTTT AATTGCCTTG GCGTTTTTTT ATTTCTCTAC CAGCGGTAGT TGGCACATCG   
  
  
- TTGGGAAAGA AAAGCCTCCT GAAAGTTTTA AGTCCGTAAA GGTACCTCTA AAAAACAGAA GAACTTCTGG   
  
  
- CCCTTGCCAA AGTTCGTTAG GGTGCTTTCC CCCGGACTTA GATCTGTTGC AACGATAGTT TGTCGAAGTA   
  
  
- GACACAATTT ATAAAATACC TAAACTAAAT GGACAGGGGA GACGGTCAGA AAAGTTTAGT TTAAACGGTG   
  
  
- TTCTTTGGTA CTGAAAATAA AGAAAGTACA CGTGTAGTAA TGGAATTCAC TATTGCTAAT TTCCACCTCC   
  
  
- AAACGGATTT TAATGGTCAA CTATAATTTA TGCGTTGTTG AATTAAAGTG TACTTAGGAG TGGGCATAAT   
  
  
- CGATCCCACG GTTTGAGAGA ACTAGTGGTA GGCACTAATT CGCCACGCAC CAAAATAACT AATTTTGATT   
  
  
- ACACTAAATT TAGACTAATA GGGGCTCAAT TAATACTTCC GAGCCTTGAG GTAAAAGAAG AGGAAAAAAA   
  
  
- GTGAAAAAAG AACCTGTCCC CTGCTTTCAA CCAAGTACAC CGTAGTTTTT TCATCACAAA TTATTAAATC   
  
  
- TACGAAAATT GTAAGTAACC TGTTCTGATT TTTCTAGTCT AAAGTATCGT TTAGATAGTG AATCAGCTGT   
  
  
- ATTTATACAT TTTTTGAGCT CTAAAATAAT TACCCAGAAC TTAAATTAAT TTTGGAAATT TAATTTATTT   
  
  
- ATAATAAGAG CAGTGCTCAG TTAGAGAACA TTGTAGCAGT AAAGAAATTC TAGTCACTGG AACAACTTTT   
  
  
- GATTTTTGTG TGTGTTAAAA TTAGTAAGAA GAGAAAAAGC ACATAAGTTG AGTGAATTTA AGTTGTATAT   
  
  
- CTAACTTTCT AATGTTCAGA TCTGGATAAT ATTAAGTTAT TTCTAAGATT AATCGTTCAA CTTATTCACA   
  
  
- AGATTTGTGG TTAATGAAAA AAGTAGCTAC TAAAGATAAG TTTGATGCTA CATTTGATTA TAGATTGTTA   
  
  
- ACAGAACTTA AAGCTTGTTT ATTAGTGTCG ACGTCGAAAG TTTTGTAATG GGTCTTGGTA AAAGGAACTG   
  
  
- AAGAAAAAAT AAAAGAATAC GAACTTTATT ATGTCTTGTC TAATTGCTCA AGACATGTGA CTTTTACTTT   
  
  
- GTTCTGAAAC TTGAAGATTG AACCATATAA GATAAAACAA TATGGCAGGG TAGTACAATT CTGACCTTTT   
  
  
- GATTAATTTT CCATAAAGTA CTCTAATACT ACGTATTAAT AAAAATTTTA TTAAAAAATA AATTTTTATA   
  
  
- TAATTTTATT TTAAAGAATA AATAAAAATT ATAGTTGTCT AATTTTGATA GTATTTAGTG ATTTATTAAT   
  
  
- AGTTAAATTA TAAAAAGTTT GGTTTTTGTG TAAATTTTAT GTGAAGTTTT GACACAGTTG AACTTCGAGT   
  
  
- TTTAAGTGTC TCTTCAATTT TTGTAAATTG ACATTTACGC TTCGATGTGA CAGGTTTGGA AGGTCACTGT   
  
  
- TACTTTTCGA ACGGTGAAAC ATAACTTTAT AACCATGGTG CACATTCTGT ATACGCTTGT TCCGTTACCA   
  
  
- CTGCAGCCGT TCCATATCCG TTAGCACCCT GGTTAGGGCG GCGGCATGCA GTGATTTGTG TGCACGGAAT   
  
  
- TTTTGTTTAA GGGTTCGGAA GGATCGTTGT TTCTTGTTC

+     CCGTCC motif

| Site Name | Organism | Position | Strand | Matrix score. | sequence | function |
| --- | --- | --- | --- | --- | --- | --- |
| CCGTCC motif | Nicotiana tabacum | 1514 | + | 6 | CCGTCC |  |

>Potri.002G039100.1   
+ TTTTTTTTTA TCCGTAATTG ATTATTCAAA ACCTTTCCAG CTTAAAATTT TTTCCATAAT TTAAAAACTA   
  
  
+ TTAAAGGTTG AAAACACTTT TTTTTTCTCT GAGATTTAAA ACTAATACTA TAAAAACTCT AACTCTAACT   
  
  
+ TTCCGTTGTT TATTATTTAT CACCGAATCT TCTTGATAAA TGTATTTCTT AAAGTATATT TTATATAAAA   
  
  
+ ATATTTTTTT TATTTTTAAA ATTTTCCTTT AATATACATC ATGTAAAGTT TCAAATCGAT TTAAAAATTA   
  
  
+ AACATAATTT TTTTAAAAAA TTAACGGAAC CGCAAAAAAA TAAAGAGATG GTCGCCATCA ACCGTGTAGC   
  
  
+ AACCCTTTCT TTTCGGAGGA CTTTCAAAAT TCAGGCATTT CCATGGAGAT TTTTTGTCTT CTTGAAGACC   
  
  
+ GGGAACGGTT TCAAGCAATC CCACGAAAGG GGGCCTGAAT CTAGACAACG TTGCTATCAA ACAGCTTCAT   
  
  
+ CTGTGTTAAA TATTTTATGG ATTTGATTTA CCTGTCCCCT CTGCCAGTCT TTTCAAATCA AATTTGCCAC   
  
  
+ AAGAAACCAT GACTTTTATT TCTTTCATGT GCACATCATT ACCTTAAGTG ATAACGATTA AAGGTGGAGG   
  
  
+ TTTGCCTAAA ATTACCAGTT GATATTAAAT ACGCAACAAC TTAATTTCAC ATGAATCCTC ACCCGTATTA   
  
  
+ GCTAGGGTGC CAAACTCTCT TGATCACCAT CCGTGATTAA GCGGTGCGTG GTTTTATTGA TTAAAACTAA   
  
  
+ TGTGATTTAA ATCTGATTAT CCCCGAGTTA ATTATGAAGG CTCGGAACTC CATTTTCTTC TCCTTTTTTT   
  
  
+ CACTTTTTTC TTGGACAGGG GACGAAAGTT GGTTCATGTG GCATCAAAAA AGTAGTGTTT AATAATTTAG   
  
  
+ ATGCTTTTAA CATTCATTGG ACAAGACTAA AAAGATCAGA TTTCATAGCA AATCTATCAC TTAGTCGACA   
  
  
+ TAAATATGTA AAAAACTCGA GATTTTATTA ATGGGTCTTG AATTTAATTA AAACCTTTAA ATTAAATAAA   
  
  
+ TATTATTCTC GTCACGAGTC AATCTCTTGT AACATCGTCA TTTCTTTAAG ATCAGTGACC TTGTTGAAAA   
  
  
+ CTAAAAACAC ACACAATTTT AATCATTCTT CTCTTTTTCG TGTATTCAAC TCACTTAAAT TCAACATATA   
  
  
+ GATTGAAAGA TTACAAGTCT AGACCTATTA TAATTCAATA AAGATTCTAA TTAGCAAGTT GAATAAGTGT   
  
  
+ TCTAAACACC AATTACTTTT TTCATCGATG ATTTCTATTC AAACTACGAT GTAAACTAAT ATCTAACAAT   
  
  
+ TGTCTTGAAT TTCGAACAAA TAATCACAGC TGCAGCTTTC AAAACATTAC CCAGAACCAT TTTCCTTGAC   
  
  
+ TTCTTTTTTA TTTTCTTATG CTTGAAATAA TACAGAACAG ATTAACGAGT TCTGTACACT GAAAATGAAA   
  
  
+ CAAGACTTTG AACTTCTAAC TTGGTATATT CTATTTTGTT ATACCGTCCC ATCATGTTAA GACTGGAAAA   
  
  
+ CTAATTAAAA GGTATTTCAT GAGATTATGA TGCATAATTA TTTTTAAAAT AATTTTTTAT TTAAAAATAT   
  
  
+ ATTAAAATAA AATTTCTTAT TTATTTTTAA TATCAACAGA TTAAAACTAT CATAAATCAC TAAATAATTA   
  
  
+ TCAATTTAAT ATTTTTCAAA CCAAAAACAC ATTTAAAATA CACTTCAAAA CTGTGTCAAC TTGAAGCTCA   
  
  
+ AAATTCACAG AGAAGTTAAA AACATTTAAC TGTAAATGCG AAGCTACACT GTCCAAACCT TCCAGTGACA   
  
  
+ ATGAAAAGCT TGCCACTTTG TATTGAAATA TTGGTACCAC GTGTAAGACA TATGCGAACA AGGCAATGGT   
  
  
+ GACGTCGGCA AGGTATAGGC AATCGTGGGA CCAATCCCGC CGCCGTACGT CACTAAACAC ACGTGCCTTA   
  
  
+ AAAACAAATT CCCAAGCCTT CCTAGCAACA AAGAACAAG  

- AAAAAAAAAT AGGCATTAAC TAATAAGTTT TGGAAAGGTC GAATTTTAAA AAAGGTATTA AATTTTTGAT   
  
  
- AATTTCCAAC TTTTGTGAAA AAAAAAGAGA CTCTAAATTT TGATTATGAT ATTTTTGAGA TTGAGATTGA   
  
  
- AAGGCAACAA ATAATAAATA GTGGCTTAGA AGAACTATTT ACATAAAGAA TTTCATATAA AATATATTTT   
  
  
- TATAAAAAAA ATAAAAATTT TAAAAGGAAA TTATATGTAG TACATTTCAA AGTTTAGCTA AATTTTTAAT   
  
  
- TTGTATTAAA AAAATTTTTT AATTGCCTTG GCGTTTTTTT ATTTCTCTAC CAGCGGTAGT TGGCACATCG   
  
  
- TTGGGAAAGA AAAGCCTCCT GAAAGTTTTA AGTCCGTAAA GGTACCTCTA AAAAACAGAA GAACTTCTGG   
  
  
- CCCTTGCCAA AGTTCGTTAG GGTGCTTTCC CCCGGACTTA GATCTGTTGC AACGATAGTT TGTCGAAGTA   
  
  
- GACACAATTT ATAAAATACC TAAACTAAAT GGACAGGGGA GACGGTCAGA AAAGTTTAGT TTAAACGGTG   
  
  
- TTCTTTGGTA CTGAAAATAA AGAAAGTACA CGTGTAGTAA TGGAATTCAC TATTGCTAAT TTCCACCTCC   
  
  
- AAACGGATTT TAATGGTCAA CTATAATTTA TGCGTTGTTG AATTAAAGTG TACTTAGGAG TGGGCATAAT   
  
  
- CGATCCCACG GTTTGAGAGA ACTAGTGGTA GGCACTAATT CGCCACGCAC CAAAATAACT AATTTTGATT   
  
  
- ACACTAAATT TAGACTAATA GGGGCTCAAT TAATACTTCC GAGCCTTGAG GTAAAAGAAG AGGAAAAAAA   
  
  
- GTGAAAAAAG AACCTGTCCC CTGCTTTCAA CCAAGTACAC CGTAGTTTTT TCATCACAAA TTATTAAATC   
  
  
- TACGAAAATT GTAAGTAACC TGTTCTGATT TTTCTAGTCT AAAGTATCGT TTAGATAGTG AATCAGCTGT   
  
  
- ATTTATACAT TTTTTGAGCT CTAAAATAAT TACCCAGAAC TTAAATTAAT TTTGGAAATT TAATTTATTT   
  
  
- ATAATAAGAG CAGTGCTCAG TTAGAGAACA TTGTAGCAGT AAAGAAATTC TAGTCACTGG AACAACTTTT   
  
  
- GATTTTTGTG TGTGTTAAAA TTAGTAAGAA GAGAAAAAGC ACATAAGTTG AGTGAATTTA AGTTGTATAT   
  
  
- CTAACTTTCT AATGTTCAGA TCTGGATAAT ATTAAGTTAT TTCTAAGATT AATCGTTCAA CTTATTCACA   
  
  
- AGATTTGTGG TTAATGAAAA AAGTAGCTAC TAAAGATAAG TTTGATGCTA CATTTGATTA TAGATTGTTA   
  
  
- ACAGAACTTA AAGCTTGTTT ATTAGTGTCG ACGTCGAAAG TTTTGTAATG GGTCTTGGTA AAAGGAACTG   
  
  
- AAGAAAAAAT AAAAGAATAC GAACTTTATT ATGTCTTGTC TAATTGCTCA AGACATGTGA CTTTTACTTT   
  
  
- GTTCTGAAAC TTGAAGATTG AACCATATAA GATAAAACAA TATGGCAGGG TAGTACAATT CTGACCTTTT   
  
  
- GATTAATTTT CCATAAAGTA CTCTAATACT ACGTATTAAT AAAAATTTTA TTAAAAAATA AATTTTTATA   
  
  
- TAATTTTATT TTAAAGAATA AATAAAAATT ATAGTTGTCT AATTTTGATA GTATTTAGTG ATTTATTAAT   
  
  
- AGTTAAATTA TAAAAAGTTT GGTTTTTGTG TAAATTTTAT GTGAAGTTTT GACACAGTTG AACTTCGAGT   
  
  
- TTTAAGTGTC TCTTCAATTT TTGTAAATTG ACATTTACGC TTCGATGTGA CAGGTTTGGA AGGTCACTGT   
  
  
- TACTTTTCGA ACGGTGAAAC ATAACTTTAT AACCATGGTG CACATTCTGT ATACGCTTGT TCCGTTACCA   
  
  
- CTGCAGCCGT TCCATATCCG TTAGCACCCT GGTTAGGGCG GCGGCATGCA GTGATTTGTG TGCACGGAAT   
  
  
- TTTTGTTTAA GGGTTCGGAA GGATCGTTGT TTCTTGTTC

+     CCGTCC-box

| Site Name | Organism | Position | Strand | Matrix score. | sequence | function |
| --- | --- | --- | --- | --- | --- | --- |
| CCGTCC-box | Petroselinum hortense | 1514 | + | 6 | CCGTCC |  |

>Potri.002G039100.1   
+ TTTTTTTTTA TCCGTAATTG ATTATTCAAA ACCTTTCCAG CTTAAAATTT TTTCCATAAT TTAAAAACTA   
  
  
+ TTAAAGGTTG AAAACACTTT TTTTTTCTCT GAGATTTAAA ACTAATACTA TAAAAACTCT AACTCTAACT   
  
  
+ TTCCGTTGTT TATTATTTAT CACCGAATCT TCTTGATAAA TGTATTTCTT AAAGTATATT TTATATAAAA   
  
  
+ ATATTTTTTT TATTTTTAAA ATTTTCCTTT AATATACATC ATGTAAAGTT TCAAATCGAT TTAAAAATTA   
  
  
+ AACATAATTT TTTTAAAAAA TTAACGGAAC CGCAAAAAAA TAAAGAGATG GTCGCCATCA ACCGTGTAGC   
  
  
+ AACCCTTTCT TTTCGGAGGA CTTTCAAAAT TCAGGCATTT CCATGGAGAT TTTTTGTCTT CTTGAAGACC   
  
  
+ GGGAACGGTT TCAAGCAATC CCACGAAAGG GGGCCTGAAT CTAGACAACG TTGCTATCAA ACAGCTTCAT   
  
  
+ CTGTGTTAAA TATTTTATGG ATTTGATTTA CCTGTCCCCT CTGCCAGTCT TTTCAAATCA AATTTGCCAC   
  
  
+ AAGAAACCAT GACTTTTATT TCTTTCATGT GCACATCATT ACCTTAAGTG ATAACGATTA AAGGTGGAGG   
  
  
+ TTTGCCTAAA ATTACCAGTT GATATTAAAT ACGCAACAAC TTAATTTCAC ATGAATCCTC ACCCGTATTA   
  
  
+ GCTAGGGTGC CAAACTCTCT TGATCACCAT CCGTGATTAA GCGGTGCGTG GTTTTATTGA TTAAAACTAA   
  
  
+ TGTGATTTAA ATCTGATTAT CCCCGAGTTA ATTATGAAGG CTCGGAACTC CATTTTCTTC TCCTTTTTTT   
  
  
+ CACTTTTTTC TTGGACAGGG GACGAAAGTT GGTTCATGTG GCATCAAAAA AGTAGTGTTT AATAATTTAG   
  
  
+ ATGCTTTTAA CATTCATTGG ACAAGACTAA AAAGATCAGA TTTCATAGCA AATCTATCAC TTAGTCGACA   
  
  
+ TAAATATGTA AAAAACTCGA GATTTTATTA ATGGGTCTTG AATTTAATTA AAACCTTTAA ATTAAATAAA   
  
  
+ TATTATTCTC GTCACGAGTC AATCTCTTGT AACATCGTCA TTTCTTTAAG ATCAGTGACC TTGTTGAAAA   
  
  
+ CTAAAAACAC ACACAATTTT AATCATTCTT CTCTTTTTCG TGTATTCAAC TCACTTAAAT TCAACATATA   
  
  
+ GATTGAAAGA TTACAAGTCT AGACCTATTA TAATTCAATA AAGATTCTAA TTAGCAAGTT GAATAAGTGT   
  
  
+ TCTAAACACC AATTACTTTT TTCATCGATG ATTTCTATTC AAACTACGAT GTAAACTAAT ATCTAACAAT   
  
  
+ TGTCTTGAAT TTCGAACAAA TAATCACAGC TGCAGCTTTC AAAACATTAC CCAGAACCAT TTTCCTTGAC   
  
  
+ TTCTTTTTTA TTTTCTTATG CTTGAAATAA TACAGAACAG ATTAACGAGT TCTGTACACT GAAAATGAAA   
  
  
+ CAAGACTTTG AACTTCTAAC TTGGTATATT CTATTTTGTT ATACCGTCCC ATCATGTTAA GACTGGAAAA   
  
  
+ CTAATTAAAA GGTATTTCAT GAGATTATGA TGCATAATTA TTTTTAAAAT AATTTTTTAT TTAAAAATAT   
  
  
+ ATTAAAATAA AATTTCTTAT TTATTTTTAA TATCAACAGA TTAAAACTAT CATAAATCAC TAAATAATTA   
  
  
+ TCAATTTAAT ATTTTTCAAA CCAAAAACAC ATTTAAAATA CACTTCAAAA CTGTGTCAAC TTGAAGCTCA   
  
  
+ AAATTCACAG AGAAGTTAAA AACATTTAAC TGTAAATGCG AAGCTACACT GTCCAAACCT TCCAGTGACA   
  
  
+ ATGAAAAGCT TGCCACTTTG TATTGAAATA TTGGTACCAC GTGTAAGACA TATGCGAACA AGGCAATGGT   
  
  
+ GACGTCGGCA AGGTATAGGC AATCGTGGGA CCAATCCCGC CGCCGTACGT CACTAAACAC ACGTGCCTTA   
  
  
+ AAAACAAATT CCCAAGCCTT CCTAGCAACA AAGAACAAG  

- AAAAAAAAAT AGGCATTAAC TAATAAGTTT TGGAAAGGTC GAATTTTAAA AAAGGTATTA AATTTTTGAT   
  
  
- AATTTCCAAC TTTTGTGAAA AAAAAAGAGA CTCTAAATTT TGATTATGAT ATTTTTGAGA TTGAGATTGA   
  
  
- AAGGCAACAA ATAATAAATA GTGGCTTAGA AGAACTATTT ACATAAAGAA TTTCATATAA AATATATTTT   
  
  
- TATAAAAAAA ATAAAAATTT TAAAAGGAAA TTATATGTAG TACATTTCAA AGTTTAGCTA AATTTTTAAT   
  
  
- TTGTATTAAA AAAATTTTTT AATTGCCTTG GCGTTTTTTT ATTTCTCTAC CAGCGGTAGT TGGCACATCG   
  
  
- TTGGGAAAGA AAAGCCTCCT GAAAGTTTTA AGTCCGTAAA GGTACCTCTA AAAAACAGAA GAACTTCTGG   
  
  
- CCCTTGCCAA AGTTCGTTAG GGTGCTTTCC CCCGGACTTA GATCTGTTGC AACGATAGTT TGTCGAAGTA   
  
  
- GACACAATTT ATAAAATACC TAAACTAAAT GGACAGGGGA GACGGTCAGA AAAGTTTAGT TTAAACGGTG   
  
  
- TTCTTTGGTA CTGAAAATAA AGAAAGTACA CGTGTAGTAA TGGAATTCAC TATTGCTAAT TTCCACCTCC   
  
  
- AAACGGATTT TAATGGTCAA CTATAATTTA TGCGTTGTTG AATTAAAGTG TACTTAGGAG TGGGCATAAT   
  
  
- CGATCCCACG GTTTGAGAGA ACTAGTGGTA GGCACTAATT CGCCACGCAC CAAAATAACT AATTTTGATT   
  
  
- ACACTAAATT TAGACTAATA GGGGCTCAAT TAATACTTCC GAGCCTTGAG GTAAAAGAAG AGGAAAAAAA   
  
  
- GTGAAAAAAG AACCTGTCCC CTGCTTTCAA CCAAGTACAC CGTAGTTTTT TCATCACAAA TTATTAAATC   
  
  
- TACGAAAATT GTAAGTAACC TGTTCTGATT TTTCTAGTCT AAAGTATCGT TTAGATAGTG AATCAGCTGT   
  
  
- ATTTATACAT TTTTTGAGCT CTAAAATAAT TACCCAGAAC TTAAATTAAT TTTGGAAATT TAATTTATTT   
  
  
- ATAATAAGAG CAGTGCTCAG TTAGAGAACA TTGTAGCAGT AAAGAAATTC TAGTCACTGG AACAACTTTT   
  
  
- GATTTTTGTG TGTGTTAAAA TTAGTAAGAA GAGAAAAAGC ACATAAGTTG AGTGAATTTA AGTTGTATAT   
  
  
- CTAACTTTCT AATGTTCAGA TCTGGATAAT ATTAAGTTAT TTCTAAGATT AATCGTTCAA CTTATTCACA   
  
  
- AGATTTGTGG TTAATGAAAA AAGTAGCTAC TAAAGATAAG TTTGATGCTA CATTTGATTA TAGATTGTTA   
  
  
- ACAGAACTTA AAGCTTGTTT ATTAGTGTCG ACGTCGAAAG TTTTGTAATG GGTCTTGGTA AAAGGAACTG   
  
  
- AAGAAAAAAT AAAAGAATAC GAACTTTATT ATGTCTTGTC TAATTGCTCA AGACATGTGA CTTTTACTTT   
  
  
- GTTCTGAAAC TTGAAGATTG AACCATATAA GATAAAACAA TATGGCAGGG TAGTACAATT CTGACCTTTT   
  
  
- GATTAATTTT CCATAAAGTA CTCTAATACT ACGTATTAAT AAAAATTTTA TTAAAAAATA AATTTTTATA   
  
  
- TAATTTTATT TTAAAGAATA AATAAAAATT ATAGTTGTCT AATTTTGATA GTATTTAGTG ATTTATTAAT   
  
  
- AGTTAAATTA TAAAAAGTTT GGTTTTTGTG TAAATTTTAT GTGAAGTTTT GACACAGTTG AACTTCGAGT   
  
  
- TTTAAGTGTC TCTTCAATTT TTGTAAATTG ACATTTACGC TTCGATGTGA CAGGTTTGGA AGGTCACTGT   
  
  
- TACTTTTCGA ACGGTGAAAC ATAACTTTAT AACCATGGTG CACATTCTGT ATACGCTTGT TCCGTTACCA   
  
  
- CTGCAGCCGT TCCATATCCG TTAGCACCCT GGTTAGGGCG GCGGCATGCA GTGATTTGTG TGCACGGAAT   
  
  
- TTTTGTTTAA GGGTTCGGAA GGATCGTTGT TTCTTGTTC

+     CGTCA-motif

| Site Name | Organism | Position | Strand | Matrix score. | sequence | function |
| --- | --- | --- | --- | --- | --- | --- |
| CGTCA-motif | Hordeum vulgare | 1060 | + | 5 | CGTCA | cis-acting regulatory element involved in the MeJA-responsiveness |
| CGTCA-motif | Hordeum vulgare | 1890 | - | 5 | CGTCA | cis-acting regulatory element involved in the MeJA-responsiveness |
| CGTCA-motif | Hordeum vulgare | 1086 | + | 5 | CGTCA | cis-acting regulatory element involved in the MeJA-responsiveness |
| CGTCA-motif | Hordeum vulgare | 1938 | + | 5 | CGTCA | cis-acting regulatory element involved in the MeJA-responsiveness |

>Potri.002G039100.1   
+ TTTTTTTTTA TCCGTAATTG ATTATTCAAA ACCTTTCCAG CTTAAAATTT TTTCCATAAT TTAAAAACTA   
  
  
+ TTAAAGGTTG AAAACACTTT TTTTTTCTCT GAGATTTAAA ACTAATACTA TAAAAACTCT AACTCTAACT   
  
  
+ TTCCGTTGTT TATTATTTAT CACCGAATCT TCTTGATAAA TGTATTTCTT AAAGTATATT TTATATAAAA   
  
  
+ ATATTTTTTT TATTTTTAAA ATTTTCCTTT AATATACATC ATGTAAAGTT TCAAATCGAT TTAAAAATTA   
  
  
+ AACATAATTT TTTTAAAAAA TTAACGGAAC CGCAAAAAAA TAAAGAGATG GTCGCCATCA ACCGTGTAGC   
  
  
+ AACCCTTTCT TTTCGGAGGA CTTTCAAAAT TCAGGCATTT CCATGGAGAT TTTTTGTCTT CTTGAAGACC   
  
  
+ GGGAACGGTT TCAAGCAATC CCACGAAAGG GGGCCTGAAT CTAGACAACG TTGCTATCAA ACAGCTTCAT   
  
  
+ CTGTGTTAAA TATTTTATGG ATTTGATTTA CCTGTCCCCT CTGCCAGTCT TTTCAAATCA AATTTGCCAC   
  
  
+ AAGAAACCAT GACTTTTATT TCTTTCATGT GCACATCATT ACCTTAAGTG ATAACGATTA AAGGTGGAGG   
  
  
+ TTTGCCTAAA ATTACCAGTT GATATTAAAT ACGCAACAAC TTAATTTCAC ATGAATCCTC ACCCGTATTA   
  
  
+ GCTAGGGTGC CAAACTCTCT TGATCACCAT CCGTGATTAA GCGGTGCGTG GTTTTATTGA TTAAAACTAA   
  
  
+ TGTGATTTAA ATCTGATTAT CCCCGAGTTA ATTATGAAGG CTCGGAACTC CATTTTCTTC TCCTTTTTTT   
  
  
+ CACTTTTTTC TTGGACAGGG GACGAAAGTT GGTTCATGTG GCATCAAAAA AGTAGTGTTT AATAATTTAG   
  
  
+ ATGCTTTTAA CATTCATTGG ACAAGACTAA AAAGATCAGA TTTCATAGCA AATCTATCAC TTAGTCGACA   
  
  
+ TAAATATGTA AAAAACTCGA GATTTTATTA ATGGGTCTTG AATTTAATTA AAACCTTTAA ATTAAATAAA   
  
  
+ TATTATTCTC GTCACGAGTC AATCTCTTGT AACATCGTCA TTTCTTTAAG ATCAGTGACC TTGTTGAAAA   
  
  
+ CTAAAAACAC ACACAATTTT AATCATTCTT CTCTTTTTCG TGTATTCAAC TCACTTAAAT TCAACATATA   
  
  
+ GATTGAAAGA TTACAAGTCT AGACCTATTA TAATTCAATA AAGATTCTAA TTAGCAAGTT GAATAAGTGT   
  
  
+ TCTAAACACC AATTACTTTT TTCATCGATG ATTTCTATTC AAACTACGAT GTAAACTAAT ATCTAACAAT   
  
  
+ TGTCTTGAAT TTCGAACAAA TAATCACAGC TGCAGCTTTC AAAACATTAC CCAGAACCAT TTTCCTTGAC   
  
  
+ TTCTTTTTTA TTTTCTTATG CTTGAAATAA TACAGAACAG ATTAACGAGT TCTGTACACT GAAAATGAAA   
  
  
+ CAAGACTTTG AACTTCTAAC TTGGTATATT CTATTTTGTT ATACCGTCCC ATCATGTTAA GACTGGAAAA   
  
  
+ CTAATTAAAA GGTATTTCAT GAGATTATGA TGCATAATTA TTTTTAAAAT AATTTTTTAT TTAAAAATAT   
  
  
+ ATTAAAATAA AATTTCTTAT TTATTTTTAA TATCAACAGA TTAAAACTAT CATAAATCAC TAAATAATTA   
  
  
+ TCAATTTAAT ATTTTTCAAA CCAAAAACAC ATTTAAAATA CACTTCAAAA CTGTGTCAAC TTGAAGCTCA   
  
  
+ AAATTCACAG AGAAGTTAAA AACATTTAAC TGTAAATGCG AAGCTACACT GTCCAAACCT TCCAGTGACA   
  
  
+ ATGAAAAGCT TGCCACTTTG TATTGAAATA TTGGTACCAC GTGTAAGACA TATGCGAACA AGGCAATGGT   
  
  
+ GACGTCGGCA AGGTATAGGC AATCGTGGGA CCAATCCCGC CGCCGTACGT CACTAAACAC ACGTGCCTTA   
  
  
+ AAAACAAATT CCCAAGCCTT CCTAGCAACA AAGAACAAG  

- AAAAAAAAAT AGGCATTAAC TAATAAGTTT TGGAAAGGTC GAATTTTAAA AAAGGTATTA AATTTTTGAT   
  
  
- AATTTCCAAC TTTTGTGAAA AAAAAAGAGA CTCTAAATTT TGATTATGAT ATTTTTGAGA TTGAGATTGA   
  
  
- AAGGCAACAA ATAATAAATA GTGGCTTAGA AGAACTATTT ACATAAAGAA TTTCATATAA AATATATTTT   
  
  
- TATAAAAAAA ATAAAAATTT TAAAAGGAAA TTATATGTAG TACATTTCAA AGTTTAGCTA AATTTTTAAT   
  
  
- TTGTATTAAA AAAATTTTTT AATTGCCTTG GCGTTTTTTT ATTTCTCTAC CAGCGGTAGT TGGCACATCG   
  
  
- TTGGGAAAGA AAAGCCTCCT GAAAGTTTTA AGTCCGTAAA GGTACCTCTA AAAAACAGAA GAACTTCTGG   
  
  
- CCCTTGCCAA AGTTCGTTAG GGTGCTTTCC CCCGGACTTA GATCTGTTGC AACGATAGTT TGTCGAAGTA   
  
  
- GACACAATTT ATAAAATACC TAAACTAAAT GGACAGGGGA GACGGTCAGA AAAGTTTAGT TTAAACGGTG   
  
  
- TTCTTTGGTA CTGAAAATAA AGAAAGTACA CGTGTAGTAA TGGAATTCAC TATTGCTAAT TTCCACCTCC   
  
  
- AAACGGATTT TAATGGTCAA CTATAATTTA TGCGTTGTTG AATTAAAGTG TACTTAGGAG TGGGCATAAT   
  
  
- CGATCCCACG GTTTGAGAGA ACTAGTGGTA GGCACTAATT CGCCACGCAC CAAAATAACT AATTTTGATT   
  
  
- ACACTAAATT TAGACTAATA GGGGCTCAAT TAATACTTCC GAGCCTTGAG GTAAAAGAAG AGGAAAAAAA   
  
  
- GTGAAAAAAG AACCTGTCCC CTGCTTTCAA CCAAGTACAC CGTAGTTTTT TCATCACAAA TTATTAAATC   
  
  
- TACGAAAATT GTAAGTAACC TGTTCTGATT TTTCTAGTCT AAAGTATCGT TTAGATAGTG AATCAGCTGT   
  
  
- ATTTATACAT TTTTTGAGCT CTAAAATAAT TACCCAGAAC TTAAATTAAT TTTGGAAATT TAATTTATTT   
  
  
- ATAATAAGAG CAGTGCTCAG TTAGAGAACA TTGTAGCAGT AAAGAAATTC TAGTCACTGG AACAACTTTT   
  
  
- GATTTTTGTG TGTGTTAAAA TTAGTAAGAA GAGAAAAAGC ACATAAGTTG AGTGAATTTA AGTTGTATAT   
  
  
- CTAACTTTCT AATGTTCAGA TCTGGATAAT ATTAAGTTAT TTCTAAGATT AATCGTTCAA CTTATTCACA   
  
  
- AGATTTGTGG TTAATGAAAA AAGTAGCTAC TAAAGATAAG TTTGATGCTA CATTTGATTA TAGATTGTTA   
  
  
- ACAGAACTTA AAGCTTGTTT ATTAGTGTCG ACGTCGAAAG TTTTGTAATG GGTCTTGGTA AAAGGAACTG   
  
  
- AAGAAAAAAT AAAAGAATAC GAACTTTATT ATGTCTTGTC TAATTGCTCA AGACATGTGA CTTTTACTTT   
  
  
- GTTCTGAAAC TTGAAGATTG AACCATATAA GATAAAACAA TATGGCAGGG TAGTACAATT CTGACCTTTT   
  
  
- GATTAATTTT CCATAAAGTA CTCTAATACT ACGTATTAAT AAAAATTTTA TTAAAAAATA AATTTTTATA   
  
  
- TAATTTTATT TTAAAGAATA AATAAAAATT ATAGTTGTCT AATTTTGATA GTATTTAGTG ATTTATTAAT   
  
  
- AGTTAAATTA TAAAAAGTTT GGTTTTTGTG TAAATTTTAT GTGAAGTTTT GACACAGTTG AACTTCGAGT   
  
  
- TTTAAGTGTC TCTTCAATTT TTGTAAATTG ACATTTACGC TTCGATGTGA CAGGTTTGGA AGGTCACTGT   
  
  
- TACTTTTCGA ACGGTGAAAC ATAACTTTAT AACCATGGTG CACATTCTGT ATACGCTTGT TCCGTTACCA   
  
  
- CTGCAGCCGT TCCATATCCG TTAGCACCCT GGTTAGGGCG GCGGCATGCA GTGATTTGTG TGCACGGAAT   
  
  
- TTTTGTTTAA GGGTTCGGAA GGATCGTTGT TTCTTGTTC

+     DRE core

| Site Name | Organism | Position | Strand | Matrix score. | sequence | function |
| --- | --- | --- | --- | --- | --- | --- |
| DRE core | Arabidopsis thaliana | 1894 | - | 6 | GCCGAC |  |

>Potri.002G039100.1   
+ TTTTTTTTTA TCCGTAATTG ATTATTCAAA ACCTTTCCAG CTTAAAATTT TTTCCATAAT TTAAAAACTA   
  
  
+ TTAAAGGTTG AAAACACTTT TTTTTTCTCT GAGATTTAAA ACTAATACTA TAAAAACTCT AACTCTAACT   
  
  
+ TTCCGTTGTT TATTATTTAT CACCGAATCT TCTTGATAAA TGTATTTCTT AAAGTATATT TTATATAAAA   
  
  
+ ATATTTTTTT TATTTTTAAA ATTTTCCTTT AATATACATC ATGTAAAGTT TCAAATCGAT TTAAAAATTA   
  
  
+ AACATAATTT TTTTAAAAAA TTAACGGAAC CGCAAAAAAA TAAAGAGATG GTCGCCATCA ACCGTGTAGC   
  
  
+ AACCCTTTCT TTTCGGAGGA CTTTCAAAAT TCAGGCATTT CCATGGAGAT TTTTTGTCTT CTTGAAGACC   
  
  
+ GGGAACGGTT TCAAGCAATC CCACGAAAGG GGGCCTGAAT CTAGACAACG TTGCTATCAA ACAGCTTCAT   
  
  
+ CTGTGTTAAA TATTTTATGG ATTTGATTTA CCTGTCCCCT CTGCCAGTCT TTTCAAATCA AATTTGCCAC   
  
  
+ AAGAAACCAT GACTTTTATT TCTTTCATGT GCACATCATT ACCTTAAGTG ATAACGATTA AAGGTGGAGG   
  
  
+ TTTGCCTAAA ATTACCAGTT GATATTAAAT ACGCAACAAC TTAATTTCAC ATGAATCCTC ACCCGTATTA   
  
  
+ GCTAGGGTGC CAAACTCTCT TGATCACCAT CCGTGATTAA GCGGTGCGTG GTTTTATTGA TTAAAACTAA   
  
  
+ TGTGATTTAA ATCTGATTAT CCCCGAGTTA ATTATGAAGG CTCGGAACTC CATTTTCTTC TCCTTTTTTT   
  
  
+ CACTTTTTTC TTGGACAGGG GACGAAAGTT GGTTCATGTG GCATCAAAAA AGTAGTGTTT AATAATTTAG   
  
  
+ ATGCTTTTAA CATTCATTGG ACAAGACTAA AAAGATCAGA TTTCATAGCA AATCTATCAC TTAGTCGACA   
  
  
+ TAAATATGTA AAAAACTCGA GATTTTATTA ATGGGTCTTG AATTTAATTA AAACCTTTAA ATTAAATAAA   
  
  
+ TATTATTCTC GTCACGAGTC AATCTCTTGT AACATCGTCA TTTCTTTAAG ATCAGTGACC TTGTTGAAAA   
  
  
+ CTAAAAACAC ACACAATTTT AATCATTCTT CTCTTTTTCG TGTATTCAAC TCACTTAAAT TCAACATATA   
  
  
+ GATTGAAAGA TTACAAGTCT AGACCTATTA TAATTCAATA AAGATTCTAA TTAGCAAGTT GAATAAGTGT   
  
  
+ TCTAAACACC AATTACTTTT TTCATCGATG ATTTCTATTC AAACTACGAT GTAAACTAAT ATCTAACAAT   
  
  
+ TGTCTTGAAT TTCGAACAAA TAATCACAGC TGCAGCTTTC AAAACATTAC CCAGAACCAT TTTCCTTGAC   
  
  
+ TTCTTTTTTA TTTTCTTATG CTTGAAATAA TACAGAACAG ATTAACGAGT TCTGTACACT GAAAATGAAA   
  
  
+ CAAGACTTTG AACTTCTAAC TTGGTATATT CTATTTTGTT ATACCGTCCC ATCATGTTAA GACTGGAAAA   
  
  
+ CTAATTAAAA GGTATTTCAT GAGATTATGA TGCATAATTA TTTTTAAAAT AATTTTTTAT TTAAAAATAT   
  
  
+ ATTAAAATAA AATTTCTTAT TTATTTTTAA TATCAACAGA TTAAAACTAT CATAAATCAC TAAATAATTA   
  
  
+ TCAATTTAAT ATTTTTCAAA CCAAAAACAC ATTTAAAATA CACTTCAAAA CTGTGTCAAC TTGAAGCTCA   
  
  
+ AAATTCACAG AGAAGTTAAA AACATTTAAC TGTAAATGCG AAGCTACACT GTCCAAACCT TCCAGTGACA   
  
  
+ ATGAAAAGCT TGCCACTTTG TATTGAAATA TTGGTACCAC GTGTAAGACA TATGCGAACA AGGCAATGGT   
  
  
+ GACGTCGGCA AGGTATAGGC AATCGTGGGA CCAATCCCGC CGCCGTACGT CACTAAACAC ACGTGCCTTA   
  
  
+ AAAACAAATT CCCAAGCCTT CCTAGCAACA AAGAACAAG  

- AAAAAAAAAT AGGCATTAAC TAATAAGTTT TGGAAAGGTC GAATTTTAAA AAAGGTATTA AATTTTTGAT   
  
  
- AATTTCCAAC TTTTGTGAAA AAAAAAGAGA CTCTAAATTT TGATTATGAT ATTTTTGAGA TTGAGATTGA   
  
  
- AAGGCAACAA ATAATAAATA GTGGCTTAGA AGAACTATTT ACATAAAGAA TTTCATATAA AATATATTTT   
  
  
- TATAAAAAAA ATAAAAATTT TAAAAGGAAA TTATATGTAG TACATTTCAA AGTTTAGCTA AATTTTTAAT   
  
  
- TTGTATTAAA AAAATTTTTT AATTGCCTTG GCGTTTTTTT ATTTCTCTAC CAGCGGTAGT TGGCACATCG   
  
  
- TTGGGAAAGA AAAGCCTCCT GAAAGTTTTA AGTCCGTAAA GGTACCTCTA AAAAACAGAA GAACTTCTGG   
  
  
- CCCTTGCCAA AGTTCGTTAG GGTGCTTTCC CCCGGACTTA GATCTGTTGC AACGATAGTT TGTCGAAGTA   
  
  
- GACACAATTT ATAAAATACC TAAACTAAAT GGACAGGGGA GACGGTCAGA AAAGTTTAGT TTAAACGGTG   
  
  
- TTCTTTGGTA CTGAAAATAA AGAAAGTACA CGTGTAGTAA TGGAATTCAC TATTGCTAAT TTCCACCTCC   
  
  
- AAACGGATTT TAATGGTCAA CTATAATTTA TGCGTTGTTG AATTAAAGTG TACTTAGGAG TGGGCATAAT   
  
  
- CGATCCCACG GTTTGAGAGA ACTAGTGGTA GGCACTAATT CGCCACGCAC CAAAATAACT AATTTTGATT   
  
  
- ACACTAAATT TAGACTAATA GGGGCTCAAT TAATACTTCC GAGCCTTGAG GTAAAAGAAG AGGAAAAAAA   
  
  
- GTGAAAAAAG AACCTGTCCC CTGCTTTCAA CCAAGTACAC CGTAGTTTTT TCATCACAAA TTATTAAATC   
  
  
- TACGAAAATT GTAAGTAACC TGTTCTGATT TTTCTAGTCT AAAGTATCGT TTAGATAGTG AATCAGCTGT   
  
  
- ATTTATACAT TTTTTGAGCT CTAAAATAAT TACCCAGAAC TTAAATTAAT TTTGGAAATT TAATTTATTT   
  
  
- ATAATAAGAG CAGTGCTCAG TTAGAGAACA TTGTAGCAGT AAAGAAATTC TAGTCACTGG AACAACTTTT   
  
  
- GATTTTTGTG TGTGTTAAAA TTAGTAAGAA GAGAAAAAGC ACATAAGTTG AGTGAATTTA AGTTGTATAT   
  
  
- CTAACTTTCT AATGTTCAGA TCTGGATAAT ATTAAGTTAT TTCTAAGATT AATCGTTCAA CTTATTCACA   
  
  
- AGATTTGTGG TTAATGAAAA AAGTAGCTAC TAAAGATAAG TTTGATGCTA CATTTGATTA TAGATTGTTA   
  
  
- ACAGAACTTA AAGCTTGTTT ATTAGTGTCG ACGTCGAAAG TTTTGTAATG GGTCTTGGTA AAAGGAACTG   
  
  
- AAGAAAAAAT AAAAGAATAC GAACTTTATT ATGTCTTGTC TAATTGCTCA AGACATGTGA CTTTTACTTT   
  
  
- GTTCTGAAAC TTGAAGATTG AACCATATAA GATAAAACAA TATGGCAGGG TAGTACAATT CTGACCTTTT   
  
  
- GATTAATTTT CCATAAAGTA CTCTAATACT ACGTATTAAT AAAAATTTTA TTAAAAAATA AATTTTTATA   
  
  
- TAATTTTATT TTAAAGAATA AATAAAAATT ATAGTTGTCT AATTTTGATA GTATTTAGTG ATTTATTAAT   
  
  
- AGTTAAATTA TAAAAAGTTT GGTTTTTGTG TAAATTTTAT GTGAAGTTTT GACACAGTTG AACTTCGAGT   
  
  
- TTTAAGTGTC TCTTCAATTT TTGTAAATTG ACATTTACGC TTCGATGTGA CAGGTTTGGA AGGTCACTGT   
  
  
- TACTTTTCGA ACGGTGAAAC ATAACTTTAT AACCATGGTG CACATTCTGT ATACGCTTGT TCCGTTACCA   
  
  
- CTGCAGCCGT TCCATATCCG TTAGCACCCT GGTTAGGGCG GCGGCATGCA GTGATTTGTG TGCACGGAAT   
  
  
- TTTTGTTTAA GGGTTCGGAA GGATCGTTGT TTCTTGTTC

+     ERE

| Site Name | Organism | Position | Strand | Matrix score. | sequence | function |
| --- | --- | --- | --- | --- | --- | --- |
| ERE | Nicotiana glutinos | 225 | - | 8 | ATTTTAAA |  |
| ERE | Nicotiana glutinos | 1583 | - | 8 | ATTTTAAA |  |
| ERE | Nicotiana glutinos | 950 | + | 8 | ATTTCATA |  |
| ERE | Nicotiana glutinos | 1712 | - | 8 | ATTTTAAA |  |

>Potri.002G039100.1   
+ TTTTTTTTTA TCCGTAATTG ATTATTCAAA ACCTTTCCAG CTTAAAATTT TTTCCATAAT TTAAAAACTA   
  
  
+ TTAAAGGTTG AAAACACTTT TTTTTTCTCT GAGATTTAAA ACTAATACTA TAAAAACTCT AACTCTAACT   
  
  
+ TTCCGTTGTT TATTATTTAT CACCGAATCT TCTTGATAAA TGTATTTCTT AAAGTATATT TTATATAAAA   
  
  
+ ATATTTTTTT TATTTTTAAA ATTTTCCTTT AATATACATC ATGTAAAGTT TCAAATCGAT TTAAAAATTA   
  
  
+ AACATAATTT TTTTAAAAAA TTAACGGAAC CGCAAAAAAA TAAAGAGATG GTCGCCATCA ACCGTGTAGC   
  
  
+ AACCCTTTCT TTTCGGAGGA CTTTCAAAAT TCAGGCATTT CCATGGAGAT TTTTTGTCTT CTTGAAGACC   
  
  
+ GGGAACGGTT TCAAGCAATC CCACGAAAGG GGGCCTGAAT CTAGACAACG TTGCTATCAA ACAGCTTCAT   
  
  
+ CTGTGTTAAA TATTTTATGG ATTTGATTTA CCTGTCCCCT CTGCCAGTCT TTTCAAATCA AATTTGCCAC   
  
  
+ AAGAAACCAT GACTTTTATT TCTTTCATGT GCACATCATT ACCTTAAGTG ATAACGATTA AAGGTGGAGG   
  
  
+ TTTGCCTAAA ATTACCAGTT GATATTAAAT ACGCAACAAC TTAATTTCAC ATGAATCCTC ACCCGTATTA   
  
  
+ GCTAGGGTGC CAAACTCTCT TGATCACCAT CCGTGATTAA GCGGTGCGTG GTTTTATTGA TTAAAACTAA   
  
  
+ TGTGATTTAA ATCTGATTAT CCCCGAGTTA ATTATGAAGG CTCGGAACTC CATTTTCTTC TCCTTTTTTT   
  
  
+ CACTTTTTTC TTGGACAGGG GACGAAAGTT GGTTCATGTG GCATCAAAAA AGTAGTGTTT AATAATTTAG   
  
  
+ ATGCTTTTAA CATTCATTGG ACAAGACTAA AAAGATCAGA TTTCATAGCA AATCTATCAC TTAGTCGACA   
  
  
+ TAAATATGTA AAAAACTCGA GATTTTATTA ATGGGTCTTG AATTTAATTA AAACCTTTAA ATTAAATAAA   
  
  
+ TATTATTCTC GTCACGAGTC AATCTCTTGT AACATCGTCA TTTCTTTAAG ATCAGTGACC TTGTTGAAAA   
  
  
+ CTAAAAACAC ACACAATTTT AATCATTCTT CTCTTTTTCG TGTATTCAAC TCACTTAAAT TCAACATATA   
  
  
+ GATTGAAAGA TTACAAGTCT AGACCTATTA TAATTCAATA AAGATTCTAA TTAGCAAGTT GAATAAGTGT   
  
  
+ TCTAAACACC AATTACTTTT TTCATCGATG ATTTCTATTC AAACTACGAT GTAAACTAAT ATCTAACAAT   
  
  
+ TGTCTTGAAT TTCGAACAAA TAATCACAGC TGCAGCTTTC AAAACATTAC CCAGAACCAT TTTCCTTGAC   
  
  
+ TTCTTTTTTA TTTTCTTATG CTTGAAATAA TACAGAACAG ATTAACGAGT TCTGTACACT GAAAATGAAA   
  
  
+ CAAGACTTTG AACTTCTAAC TTGGTATATT CTATTTTGTT ATACCGTCCC ATCATGTTAA GACTGGAAAA   
  
  
+ CTAATTAAAA GGTATTTCAT GAGATTATGA TGCATAATTA TTTTTAAAAT AATTTTTTAT TTAAAAATAT   
  
  
+ ATTAAAATAA AATTTCTTAT TTATTTTTAA TATCAACAGA TTAAAACTAT CATAAATCAC TAAATAATTA   
  
  
+ TCAATTTAAT ATTTTTCAAA CCAAAAACAC ATTTAAAATA CACTTCAAAA CTGTGTCAAC TTGAAGCTCA   
  
  
+ AAATTCACAG AGAAGTTAAA AACATTTAAC TGTAAATGCG AAGCTACACT GTCCAAACCT TCCAGTGACA   
  
  
+ ATGAAAAGCT TGCCACTTTG TATTGAAATA TTGGTACCAC GTGTAAGACA TATGCGAACA AGGCAATGGT   
  
  
+ GACGTCGGCA AGGTATAGGC AATCGTGGGA CCAATCCCGC CGCCGTACGT CACTAAACAC ACGTGCCTTA   
  
  
+ AAAACAAATT CCCAAGCCTT CCTAGCAACA AAGAACAAG  

- AAAAAAAAAT AGGCATTAAC TAATAAGTTT TGGAAAGGTC GAATTTTAAA AAAGGTATTA AATTTTTGAT   
  
  
- AATTTCCAAC TTTTGTGAAA AAAAAAGAGA CTCTAAATTT TGATTATGAT ATTTTTGAGA TTGAGATTGA   
  
  
- AAGGCAACAA ATAATAAATA GTGGCTTAGA AGAACTATTT ACATAAAGAA TTTCATATAA AATATATTTT   
  
  
- TATAAAAAAA ATAAAAATTT TAAAAGGAAA TTATATGTAG TACATTTCAA AGTTTAGCTA AATTTTTAAT   
  
  
- TTGTATTAAA AAAATTTTTT AATTGCCTTG GCGTTTTTTT ATTTCTCTAC CAGCGGTAGT TGGCACATCG   
  
  
- TTGGGAAAGA AAAGCCTCCT GAAAGTTTTA AGTCCGTAAA GGTACCTCTA AAAAACAGAA GAACTTCTGG   
  
  
- CCCTTGCCAA AGTTCGTTAG GGTGCTTTCC CCCGGACTTA GATCTGTTGC AACGATAGTT TGTCGAAGTA   
  
  
- GACACAATTT ATAAAATACC TAAACTAAAT GGACAGGGGA GACGGTCAGA AAAGTTTAGT TTAAACGGTG   
  
  
- TTCTTTGGTA CTGAAAATAA AGAAAGTACA CGTGTAGTAA TGGAATTCAC TATTGCTAAT TTCCACCTCC   
  
  
- AAACGGATTT TAATGGTCAA CTATAATTTA TGCGTTGTTG AATTAAAGTG TACTTAGGAG TGGGCATAAT   
  
  
- CGATCCCACG GTTTGAGAGA ACTAGTGGTA GGCACTAATT CGCCACGCAC CAAAATAACT AATTTTGATT   
  
  
- ACACTAAATT TAGACTAATA GGGGCTCAAT TAATACTTCC GAGCCTTGAG GTAAAAGAAG AGGAAAAAAA   
  
  
- GTGAAAAAAG AACCTGTCCC CTGCTTTCAA CCAAGTACAC CGTAGTTTTT TCATCACAAA TTATTAAATC   
  
  
- TACGAAAATT GTAAGTAACC TGTTCTGATT TTTCTAGTCT AAAGTATCGT TTAGATAGTG AATCAGCTGT   
  
  
- ATTTATACAT TTTTTGAGCT CTAAAATAAT TACCCAGAAC TTAAATTAAT TTTGGAAATT TAATTTATTT   
  
  
- ATAATAAGAG CAGTGCTCAG TTAGAGAACA TTGTAGCAGT AAAGAAATTC TAGTCACTGG AACAACTTTT   
  
  
- GATTTTTGTG TGTGTTAAAA TTAGTAAGAA GAGAAAAAGC ACATAAGTTG AGTGAATTTA AGTTGTATAT   
  
  
- CTAACTTTCT AATGTTCAGA TCTGGATAAT ATTAAGTTAT TTCTAAGATT AATCGTTCAA CTTATTCACA   
  
  
- AGATTTGTGG TTAATGAAAA AAGTAGCTAC TAAAGATAAG TTTGATGCTA CATTTGATTA TAGATTGTTA   
  
  
- ACAGAACTTA AAGCTTGTTT ATTAGTGTCG ACGTCGAAAG TTTTGTAATG GGTCTTGGTA AAAGGAACTG   
  
  
- AAGAAAAAAT AAAAGAATAC GAACTTTATT ATGTCTTGTC TAATTGCTCA AGACATGTGA CTTTTACTTT   
  
  
- GTTCTGAAAC TTGAAGATTG AACCATATAA GATAAAACAA TATGGCAGGG TAGTACAATT CTGACCTTTT   
  
  
- GATTAATTTT CCATAAAGTA CTCTAATACT ACGTATTAAT AAAAATTTTA TTAAAAAATA AATTTTTATA   
  
  
- TAATTTTATT TTAAAGAATA AATAAAAATT ATAGTTGTCT AATTTTGATA GTATTTAGTG ATTTATTAAT   
  
  
- AGTTAAATTA TAAAAAGTTT GGTTTTTGTG TAAATTTTAT GTGAAGTTTT GACACAGTTG AACTTCGAGT   
  
  
- TTTAAGTGTC TCTTCAATTT TTGTAAATTG ACATTTACGC TTCGATGTGA CAGGTTTGGA AGGTCACTGT   
  
  
- TACTTTTCGA ACGGTGAAAC ATAACTTTAT AACCATGGTG CACATTCTGT ATACGCTTGT TCCGTTACCA   
  
  
- CTGCAGCCGT TCCATATCCG TTAGCACCCT GGTTAGGGCG GCGGCATGCA GTGATTTGTG TGCACGGAAT   
  
  
- TTTTGTTTAA GGGTTCGGAA GGATCGTTGT TTCTTGTTC

+     G-Box

| Site Name | Organism | Position | Strand | Matrix score. | sequence | function |
| --- | --- | --- | --- | --- | --- | --- |
| G-Box | Pisum sativum | 1858 | - | 6 | CACGTG | cis-acting regulatory element involved in light responsiveness |
| G-Box | Pisum sativum | 1950 | - | 6 | CACGTG | cis-acting regulatory element involved in light responsiveness |

>Potri.002G039100.1   
+ TTTTTTTTTA TCCGTAATTG ATTATTCAAA ACCTTTCCAG CTTAAAATTT TTTCCATAAT TTAAAAACTA   
  
  
+ TTAAAGGTTG AAAACACTTT TTTTTTCTCT GAGATTTAAA ACTAATACTA TAAAAACTCT AACTCTAACT   
  
  
+ TTCCGTTGTT TATTATTTAT CACCGAATCT TCTTGATAAA TGTATTTCTT AAAGTATATT TTATATAAAA   
  
  
+ ATATTTTTTT TATTTTTAAA ATTTTCCTTT AATATACATC ATGTAAAGTT TCAAATCGAT TTAAAAATTA   
  
  
+ AACATAATTT TTTTAAAAAA TTAACGGAAC CGCAAAAAAA TAAAGAGATG GTCGCCATCA ACCGTGTAGC   
  
  
+ AACCCTTTCT TTTCGGAGGA CTTTCAAAAT TCAGGCATTT CCATGGAGAT TTTTTGTCTT CTTGAAGACC   
  
  
+ GGGAACGGTT TCAAGCAATC CCACGAAAGG GGGCCTGAAT CTAGACAACG TTGCTATCAA ACAGCTTCAT   
  
  
+ CTGTGTTAAA TATTTTATGG ATTTGATTTA CCTGTCCCCT CTGCCAGTCT TTTCAAATCA AATTTGCCAC   
  
  
+ AAGAAACCAT GACTTTTATT TCTTTCATGT GCACATCATT ACCTTAAGTG ATAACGATTA AAGGTGGAGG   
  
  
+ TTTGCCTAAA ATTACCAGTT GATATTAAAT ACGCAACAAC TTAATTTCAC ATGAATCCTC ACCCGTATTA   
  
  
+ GCTAGGGTGC CAAACTCTCT TGATCACCAT CCGTGATTAA GCGGTGCGTG GTTTTATTGA TTAAAACTAA   
  
  
+ TGTGATTTAA ATCTGATTAT CCCCGAGTTA ATTATGAAGG CTCGGAACTC CATTTTCTTC TCCTTTTTTT   
  
  
+ CACTTTTTTC TTGGACAGGG GACGAAAGTT GGTTCATGTG GCATCAAAAA AGTAGTGTTT AATAATTTAG   
  
  
+ ATGCTTTTAA CATTCATTGG ACAAGACTAA AAAGATCAGA TTTCATAGCA AATCTATCAC TTAGTCGACA   
  
  
+ TAAATATGTA AAAAACTCGA GATTTTATTA ATGGGTCTTG AATTTAATTA AAACCTTTAA ATTAAATAAA   
  
  
+ TATTATTCTC GTCACGAGTC AATCTCTTGT AACATCGTCA TTTCTTTAAG ATCAGTGACC TTGTTGAAAA   
  
  
+ CTAAAAACAC ACACAATTTT AATCATTCTT CTCTTTTTCG TGTATTCAAC TCACTTAAAT TCAACATATA   
  
  
+ GATTGAAAGA TTACAAGTCT AGACCTATTA TAATTCAATA AAGATTCTAA TTAGCAAGTT GAATAAGTGT   
  
  
+ TCTAAACACC AATTACTTTT TTCATCGATG ATTTCTATTC AAACTACGAT GTAAACTAAT ATCTAACAAT   
  
  
+ TGTCTTGAAT TTCGAACAAA TAATCACAGC TGCAGCTTTC AAAACATTAC CCAGAACCAT TTTCCTTGAC   
  
  
+ TTCTTTTTTA TTTTCTTATG CTTGAAATAA TACAGAACAG ATTAACGAGT TCTGTACACT GAAAATGAAA   
  
  
+ CAAGACTTTG AACTTCTAAC TTGGTATATT CTATTTTGTT ATACCGTCCC ATCATGTTAA GACTGGAAAA   
  
  
+ CTAATTAAAA GGTATTTCAT GAGATTATGA TGCATAATTA TTTTTAAAAT AATTTTTTAT TTAAAAATAT   
  
  
+ ATTAAAATAA AATTTCTTAT TTATTTTTAA TATCAACAGA TTAAAACTAT CATAAATCAC TAAATAATTA   
  
  
+ TCAATTTAAT ATTTTTCAAA CCAAAAACAC ATTTAAAATA CACTTCAAAA CTGTGTCAAC TTGAAGCTCA   
  
  
+ AAATTCACAG AGAAGTTAAA AACATTTAAC TGTAAATGCG AAGCTACACT GTCCAAACCT TCCAGTGACA   
  
  
+ ATGAAAAGCT TGCCACTTTG TATTGAAATA TTGGTACCAC GTGTAAGACA TATGCGAACA AGGCAATGGT   
  
  
+ GACGTCGGCA AGGTATAGGC AATCGTGGGA CCAATCCCGC CGCCGTACGT CACTAAACAC ACGTGCCTTA   
  
  
+ AAAACAAATT CCCAAGCCTT CCTAGCAACA AAGAACAAG  

- AAAAAAAAAT AGGCATTAAC TAATAAGTTT TGGAAAGGTC GAATTTTAAA AAAGGTATTA AATTTTTGAT   
  
  
- AATTTCCAAC TTTTGTGAAA AAAAAAGAGA CTCTAAATTT TGATTATGAT ATTTTTGAGA TTGAGATTGA   
  
  
- AAGGCAACAA ATAATAAATA GTGGCTTAGA AGAACTATTT ACATAAAGAA TTTCATATAA AATATATTTT   
  
  
- TATAAAAAAA ATAAAAATTT TAAAAGGAAA TTATATGTAG TACATTTCAA AGTTTAGCTA AATTTTTAAT   
  
  
- TTGTATTAAA AAAATTTTTT AATTGCCTTG GCGTTTTTTT ATTTCTCTAC CAGCGGTAGT TGGCACATCG   
  
  
- TTGGGAAAGA AAAGCCTCCT GAAAGTTTTA AGTCCGTAAA GGTACCTCTA AAAAACAGAA GAACTTCTGG   
  
  
- CCCTTGCCAA AGTTCGTTAG GGTGCTTTCC CCCGGACTTA GATCTGTTGC AACGATAGTT TGTCGAAGTA   
  
  
- GACACAATTT ATAAAATACC TAAACTAAAT GGACAGGGGA GACGGTCAGA AAAGTTTAGT TTAAACGGTG   
  
  
- TTCTTTGGTA CTGAAAATAA AGAAAGTACA CGTGTAGTAA TGGAATTCAC TATTGCTAAT TTCCACCTCC   
  
  
- AAACGGATTT TAATGGTCAA CTATAATTTA TGCGTTGTTG AATTAAAGTG TACTTAGGAG TGGGCATAAT   
  
  
- CGATCCCACG GTTTGAGAGA ACTAGTGGTA GGCACTAATT CGCCACGCAC CAAAATAACT AATTTTGATT   
  
  
- ACACTAAATT TAGACTAATA GGGGCTCAAT TAATACTTCC GAGCCTTGAG GTAAAAGAAG AGGAAAAAAA   
  
  
- GTGAAAAAAG AACCTGTCCC CTGCTTTCAA CCAAGTACAC CGTAGTTTTT TCATCACAAA TTATTAAATC   
  
  
- TACGAAAATT GTAAGTAACC TGTTCTGATT TTTCTAGTCT AAAGTATCGT TTAGATAGTG AATCAGCTGT   
  
  
- ATTTATACAT TTTTTGAGCT CTAAAATAAT TACCCAGAAC TTAAATTAAT TTTGGAAATT TAATTTATTT   
  
  
- ATAATAAGAG CAGTGCTCAG TTAGAGAACA TTGTAGCAGT AAAGAAATTC TAGTCACTGG AACAACTTTT   
  
  
- GATTTTTGTG TGTGTTAAAA TTAGTAAGAA GAGAAAAAGC ACATAAGTTG AGTGAATTTA AGTTGTATAT   
  
  
- CTAACTTTCT AATGTTCAGA TCTGGATAAT ATTAAGTTAT TTCTAAGATT AATCGTTCAA CTTATTCACA   
  
  
- AGATTTGTGG TTAATGAAAA AAGTAGCTAC TAAAGATAAG TTTGATGCTA CATTTGATTA TAGATTGTTA   
  
  
- ACAGAACTTA AAGCTTGTTT ATTAGTGTCG ACGTCGAAAG TTTTGTAATG GGTCTTGGTA AAAGGAACTG   
  
  
- AAGAAAAAAT AAAAGAATAC GAACTTTATT ATGTCTTGTC TAATTGCTCA AGACATGTGA CTTTTACTTT   
  
  
- GTTCTGAAAC TTGAAGATTG AACCATATAA GATAAAACAA TATGGCAGGG TAGTACAATT CTGACCTTTT   
  
  
- GATTAATTTT CCATAAAGTA CTCTAATACT ACGTATTAAT AAAAATTTTA TTAAAAAATA AATTTTTATA   
  
  
- TAATTTTATT TTAAAGAATA AATAAAAATT ATAGTTGTCT AATTTTGATA GTATTTAGTG ATTTATTAAT   
  
  
- AGTTAAATTA TAAAAAGTTT GGTTTTTGTG TAAATTTTAT GTGAAGTTTT GACACAGTTG AACTTCGAGT   
  
  
- TTTAAGTGTC TCTTCAATTT TTGTAAATTG ACATTTACGC TTCGATGTGA CAGGTTTGGA AGGTCACTGT   
  
  
- TACTTTTCGA ACGGTGAAAC ATAACTTTAT AACCATGGTG CACATTCTGT ATACGCTTGT TCCGTTACCA   
  
  
- CTGCAGCCGT TCCATATCCG TTAGCACCCT GGTTAGGGCG GCGGCATGCA GTGATTTGTG TGCACGGAAT   
  
  
- TTTTGTTTAA GGGTTCGGAA GGATCGTTGT TTCTTGTTC

+     G-box

| Site Name | Organism | Position | Strand | Matrix score. | sequence | function |
| --- | --- | --- | --- | --- | --- | --- |
| G-box | Lycopersicon esculentum | 1853 | - | 10.5 | ACACGTG(G/t)CACC | cis-acting regulatory element involved in light responsiveness |
| G-box | Arabidopsis thaliana | 1950 | - | 6 | CACGTG | cis-acting regulatory element involved in light responsiveness |
| G-box | Arabidopsis thaliana | 1858 | - | 6 | CACGTG | cis-acting regulatory element involved in light responsiveness |

>Potri.002G039100.1   
+ TTTTTTTTTA TCCGTAATTG ATTATTCAAA ACCTTTCCAG CTTAAAATTT TTTCCATAAT TTAAAAACTA   
  
  
+ TTAAAGGTTG AAAACACTTT TTTTTTCTCT GAGATTTAAA ACTAATACTA TAAAAACTCT AACTCTAACT   
  
  
+ TTCCGTTGTT TATTATTTAT CACCGAATCT TCTTGATAAA TGTATTTCTT AAAGTATATT TTATATAAAA   
  
  
+ ATATTTTTTT TATTTTTAAA ATTTTCCTTT AATATACATC ATGTAAAGTT TCAAATCGAT TTAAAAATTA   
  
  
+ AACATAATTT TTTTAAAAAA TTAACGGAAC CGCAAAAAAA TAAAGAGATG GTCGCCATCA ACCGTGTAGC   
  
  
+ AACCCTTTCT TTTCGGAGGA CTTTCAAAAT TCAGGCATTT CCATGGAGAT TTTTTGTCTT CTTGAAGACC   
  
  
+ GGGAACGGTT TCAAGCAATC CCACGAAAGG GGGCCTGAAT CTAGACAACG TTGCTATCAA ACAGCTTCAT   
  
  
+ CTGTGTTAAA TATTTTATGG ATTTGATTTA CCTGTCCCCT CTGCCAGTCT TTTCAAATCA AATTTGCCAC   
  
  
+ AAGAAACCAT GACTTTTATT TCTTTCATGT GCACATCATT ACCTTAAGTG ATAACGATTA AAGGTGGAGG   
  
  
+ TTTGCCTAAA ATTACCAGTT GATATTAAAT ACGCAACAAC TTAATTTCAC ATGAATCCTC ACCCGTATTA   
  
  
+ GCTAGGGTGC CAAACTCTCT TGATCACCAT CCGTGATTAA GCGGTGCGTG GTTTTATTGA TTAAAACTAA   
  
  
+ TGTGATTTAA ATCTGATTAT CCCCGAGTTA ATTATGAAGG CTCGGAACTC CATTTTCTTC TCCTTTTTTT   
  
  
+ CACTTTTTTC TTGGACAGGG GACGAAAGTT GGTTCATGTG GCATCAAAAA AGTAGTGTTT AATAATTTAG   
  
  
+ ATGCTTTTAA CATTCATTGG ACAAGACTAA AAAGATCAGA TTTCATAGCA AATCTATCAC TTAGTCGACA   
  
  
+ TAAATATGTA AAAAACTCGA GATTTTATTA ATGGGTCTTG AATTTAATTA AAACCTTTAA ATTAAATAAA   
  
  
+ TATTATTCTC GTCACGAGTC AATCTCTTGT AACATCGTCA TTTCTTTAAG ATCAGTGACC TTGTTGAAAA   
  
  
+ CTAAAAACAC ACACAATTTT AATCATTCTT CTCTTTTTCG TGTATTCAAC TCACTTAAAT TCAACATATA   
  
  
+ GATTGAAAGA TTACAAGTCT AGACCTATTA TAATTCAATA AAGATTCTAA TTAGCAAGTT GAATAAGTGT   
  
  
+ TCTAAACACC AATTACTTTT TTCATCGATG ATTTCTATTC AAACTACGAT GTAAACTAAT ATCTAACAAT   
  
  
+ TGTCTTGAAT TTCGAACAAA TAATCACAGC TGCAGCTTTC AAAACATTAC CCAGAACCAT TTTCCTTGAC   
  
  
+ TTCTTTTTTA TTTTCTTATG CTTGAAATAA TACAGAACAG ATTAACGAGT TCTGTACACT GAAAATGAAA   
  
  
+ CAAGACTTTG AACTTCTAAC TTGGTATATT CTATTTTGTT ATACCGTCCC ATCATGTTAA GACTGGAAAA   
  
  
+ CTAATTAAAA GGTATTTCAT GAGATTATGA TGCATAATTA TTTTTAAAAT AATTTTTTAT TTAAAAATAT   
  
  
+ ATTAAAATAA AATTTCTTAT TTATTTTTAA TATCAACAGA TTAAAACTAT CATAAATCAC TAAATAATTA   
  
  
+ TCAATTTAAT ATTTTTCAAA CCAAAAACAC ATTTAAAATA CACTTCAAAA CTGTGTCAAC TTGAAGCTCA   
  
  
+ AAATTCACAG AGAAGTTAAA AACATTTAAC TGTAAATGCG AAGCTACACT GTCCAAACCT TCCAGTGACA   
  
  
+ ATGAAAAGCT TGCCACTTTG TATTGAAATA TTGGTACCAC GTGTAAGACA TATGCGAACA AGGCAATGGT   
  
  
+ GACGTCGGCA AGGTATAGGC AATCGTGGGA CCAATCCCGC CGCCGTACGT CACTAAACAC ACGTGCCTTA   
  
  
+ AAAACAAATT CCCAAGCCTT CCTAGCAACA AAGAACAAG  

- AAAAAAAAAT AGGCATTAAC TAATAAGTTT TGGAAAGGTC GAATTTTAAA AAAGGTATTA AATTTTTGAT   
  
  
- AATTTCCAAC TTTTGTGAAA AAAAAAGAGA CTCTAAATTT TGATTATGAT ATTTTTGAGA TTGAGATTGA   
  
  
- AAGGCAACAA ATAATAAATA GTGGCTTAGA AGAACTATTT ACATAAAGAA TTTCATATAA AATATATTTT   
  
  
- TATAAAAAAA ATAAAAATTT TAAAAGGAAA TTATATGTAG TACATTTCAA AGTTTAGCTA AATTTTTAAT   
  
  
- TTGTATTAAA AAAATTTTTT AATTGCCTTG GCGTTTTTTT ATTTCTCTAC CAGCGGTAGT TGGCACATCG   
  
  
- TTGGGAAAGA AAAGCCTCCT GAAAGTTTTA AGTCCGTAAA GGTACCTCTA AAAAACAGAA GAACTTCTGG   
  
  
- CCCTTGCCAA AGTTCGTTAG GGTGCTTTCC CCCGGACTTA GATCTGTTGC AACGATAGTT TGTCGAAGTA   
  
  
- GACACAATTT ATAAAATACC TAAACTAAAT GGACAGGGGA GACGGTCAGA AAAGTTTAGT TTAAACGGTG   
  
  
- TTCTTTGGTA CTGAAAATAA AGAAAGTACA CGTGTAGTAA TGGAATTCAC TATTGCTAAT TTCCACCTCC   
  
  
- AAACGGATTT TAATGGTCAA CTATAATTTA TGCGTTGTTG AATTAAAGTG TACTTAGGAG TGGGCATAAT   
  
  
- CGATCCCACG GTTTGAGAGA ACTAGTGGTA GGCACTAATT CGCCACGCAC CAAAATAACT AATTTTGATT   
  
  
- ACACTAAATT TAGACTAATA GGGGCTCAAT TAATACTTCC GAGCCTTGAG GTAAAAGAAG AGGAAAAAAA   
  
  
- GTGAAAAAAG AACCTGTCCC CTGCTTTCAA CCAAGTACAC CGTAGTTTTT TCATCACAAA TTATTAAATC   
  
  
- TACGAAAATT GTAAGTAACC TGTTCTGATT TTTCTAGTCT AAAGTATCGT TTAGATAGTG AATCAGCTGT   
  
  
- ATTTATACAT TTTTTGAGCT CTAAAATAAT TACCCAGAAC TTAAATTAAT TTTGGAAATT TAATTTATTT   
  
  
- ATAATAAGAG CAGTGCTCAG TTAGAGAACA TTGTAGCAGT AAAGAAATTC TAGTCACTGG AACAACTTTT   
  
  
- GATTTTTGTG TGTGTTAAAA TTAGTAAGAA GAGAAAAAGC ACATAAGTTG AGTGAATTTA AGTTGTATAT   
  
  
- CTAACTTTCT AATGTTCAGA TCTGGATAAT ATTAAGTTAT TTCTAAGATT AATCGTTCAA CTTATTCACA   
  
  
- AGATTTGTGG TTAATGAAAA AAGTAGCTAC TAAAGATAAG TTTGATGCTA CATTTGATTA TAGATTGTTA   
  
  
- ACAGAACTTA AAGCTTGTTT ATTAGTGTCG ACGTCGAAAG TTTTGTAATG GGTCTTGGTA AAAGGAACTG   
  
  
- AAGAAAAAAT AAAAGAATAC GAACTTTATT ATGTCTTGTC TAATTGCTCA AGACATGTGA CTTTTACTTT   
  
  
- GTTCTGAAAC TTGAAGATTG AACCATATAA GATAAAACAA TATGGCAGGG TAGTACAATT CTGACCTTTT   
  
  
- GATTAATTTT CCATAAAGTA CTCTAATACT ACGTATTAAT AAAAATTTTA TTAAAAAATA AATTTTTATA   
  
  
- TAATTTTATT TTAAAGAATA AATAAAAATT ATAGTTGTCT AATTTTGATA GTATTTAGTG ATTTATTAAT   
  
  
- AGTTAAATTA TAAAAAGTTT GGTTTTTGTG TAAATTTTAT GTGAAGTTTT GACACAGTTG AACTTCGAGT   
  
  
- TTTAAGTGTC TCTTCAATTT TTGTAAATTG ACATTTACGC TTCGATGTGA CAGGTTTGGA AGGTCACTGT   
  
  
- TACTTTTCGA ACGGTGAAAC ATAACTTTAT AACCATGGTG CACATTCTGT ATACGCTTGT TCCGTTACCA   
  
  
- CTGCAGCCGT TCCATATCCG TTAGCACCCT GGTTAGGGCG GCGGCATGCA GTGATTTGTG TGCACGGAAT   
  
  
- TTTTGTTTAA GGGTTCGGAA GGATCGTTGT TTCTTGTTC

+     GARE-motif

| Site Name | Organism | Position | Strand | Matrix score. | sequence | function |
| --- | --- | --- | --- | --- | --- | --- |
| GARE-motif | Brassica oleracea | 1644 | - | 7 | TCTGTTG | gibberellin-responsive element |

>Potri.002G039100.1   
+ TTTTTTTTTA TCCGTAATTG ATTATTCAAA ACCTTTCCAG CTTAAAATTT TTTCCATAAT TTAAAAACTA   
  
  
+ TTAAAGGTTG AAAACACTTT TTTTTTCTCT GAGATTTAAA ACTAATACTA TAAAAACTCT AACTCTAACT   
  
  
+ TTCCGTTGTT TATTATTTAT CACCGAATCT TCTTGATAAA TGTATTTCTT AAAGTATATT TTATATAAAA   
  
  
+ ATATTTTTTT TATTTTTAAA ATTTTCCTTT AATATACATC ATGTAAAGTT TCAAATCGAT TTAAAAATTA   
  
  
+ AACATAATTT TTTTAAAAAA TTAACGGAAC CGCAAAAAAA TAAAGAGATG GTCGCCATCA ACCGTGTAGC   
  
  
+ AACCCTTTCT TTTCGGAGGA CTTTCAAAAT TCAGGCATTT CCATGGAGAT TTTTTGTCTT CTTGAAGACC   
  
  
+ GGGAACGGTT TCAAGCAATC CCACGAAAGG GGGCCTGAAT CTAGACAACG TTGCTATCAA ACAGCTTCAT   
  
  
+ CTGTGTTAAA TATTTTATGG ATTTGATTTA CCTGTCCCCT CTGCCAGTCT TTTCAAATCA AATTTGCCAC   
  
  
+ AAGAAACCAT GACTTTTATT TCTTTCATGT GCACATCATT ACCTTAAGTG ATAACGATTA AAGGTGGAGG   
  
  
+ TTTGCCTAAA ATTACCAGTT GATATTAAAT ACGCAACAAC TTAATTTCAC ATGAATCCTC ACCCGTATTA   
  
  
+ GCTAGGGTGC CAAACTCTCT TGATCACCAT CCGTGATTAA GCGGTGCGTG GTTTTATTGA TTAAAACTAA   
  
  
+ TGTGATTTAA ATCTGATTAT CCCCGAGTTA ATTATGAAGG CTCGGAACTC CATTTTCTTC TCCTTTTTTT   
  
  
+ CACTTTTTTC TTGGACAGGG GACGAAAGTT GGTTCATGTG GCATCAAAAA AGTAGTGTTT AATAATTTAG   
  
  
+ ATGCTTTTAA CATTCATTGG ACAAGACTAA AAAGATCAGA TTTCATAGCA AATCTATCAC TTAGTCGACA   
  
  
+ TAAATATGTA AAAAACTCGA GATTTTATTA ATGGGTCTTG AATTTAATTA AAACCTTTAA ATTAAATAAA   
  
  
+ TATTATTCTC GTCACGAGTC AATCTCTTGT AACATCGTCA TTTCTTTAAG ATCAGTGACC TTGTTGAAAA   
  
  
+ CTAAAAACAC ACACAATTTT AATCATTCTT CTCTTTTTCG TGTATTCAAC TCACTTAAAT TCAACATATA   
  
  
+ GATTGAAAGA TTACAAGTCT AGACCTATTA TAATTCAATA AAGATTCTAA TTAGCAAGTT GAATAAGTGT   
  
  
+ TCTAAACACC AATTACTTTT TTCATCGATG ATTTCTATTC AAACTACGAT GTAAACTAAT ATCTAACAAT   
  
  
+ TGTCTTGAAT TTCGAACAAA TAATCACAGC TGCAGCTTTC AAAACATTAC CCAGAACCAT TTTCCTTGAC   
  
  
+ TTCTTTTTTA TTTTCTTATG CTTGAAATAA TACAGAACAG ATTAACGAGT TCTGTACACT GAAAATGAAA   
  
  
+ CAAGACTTTG AACTTCTAAC TTGGTATATT CTATTTTGTT ATACCGTCCC ATCATGTTAA GACTGGAAAA   
  
  
+ CTAATTAAAA GGTATTTCAT GAGATTATGA TGCATAATTA TTTTTAAAAT AATTTTTTAT TTAAAAATAT   
  
  
+ ATTAAAATAA AATTTCTTAT TTATTTTTAA TATCAACAGA TTAAAACTAT CATAAATCAC TAAATAATTA   
  
  
+ TCAATTTAAT ATTTTTCAAA CCAAAAACAC ATTTAAAATA CACTTCAAAA CTGTGTCAAC TTGAAGCTCA   
  
  
+ AAATTCACAG AGAAGTTAAA AACATTTAAC TGTAAATGCG AAGCTACACT GTCCAAACCT TCCAGTGACA   
  
  
+ ATGAAAAGCT TGCCACTTTG TATTGAAATA TTGGTACCAC GTGTAAGACA TATGCGAACA AGGCAATGGT   
  
  
+ GACGTCGGCA AGGTATAGGC AATCGTGGGA CCAATCCCGC CGCCGTACGT CACTAAACAC ACGTGCCTTA   
  
  
+ AAAACAAATT CCCAAGCCTT CCTAGCAACA AAGAACAAG  

- AAAAAAAAAT AGGCATTAAC TAATAAGTTT TGGAAAGGTC GAATTTTAAA AAAGGTATTA AATTTTTGAT   
  
  
- AATTTCCAAC TTTTGTGAAA AAAAAAGAGA CTCTAAATTT TGATTATGAT ATTTTTGAGA TTGAGATTGA   
  
  
- AAGGCAACAA ATAATAAATA GTGGCTTAGA AGAACTATTT ACATAAAGAA TTTCATATAA AATATATTTT   
  
  
- TATAAAAAAA ATAAAAATTT TAAAAGGAAA TTATATGTAG TACATTTCAA AGTTTAGCTA AATTTTTAAT   
  
  
- TTGTATTAAA AAAATTTTTT AATTGCCTTG GCGTTTTTTT ATTTCTCTAC CAGCGGTAGT TGGCACATCG   
  
  
- TTGGGAAAGA AAAGCCTCCT GAAAGTTTTA AGTCCGTAAA GGTACCTCTA AAAAACAGAA GAACTTCTGG   
  
  
- CCCTTGCCAA AGTTCGTTAG GGTGCTTTCC CCCGGACTTA GATCTGTTGC AACGATAGTT TGTCGAAGTA   
  
  
- GACACAATTT ATAAAATACC TAAACTAAAT GGACAGGGGA GACGGTCAGA AAAGTTTAGT TTAAACGGTG   
  
  
- TTCTTTGGTA CTGAAAATAA AGAAAGTACA CGTGTAGTAA TGGAATTCAC TATTGCTAAT TTCCACCTCC   
  
  
- AAACGGATTT TAATGGTCAA CTATAATTTA TGCGTTGTTG AATTAAAGTG TACTTAGGAG TGGGCATAAT   
  
  
- CGATCCCACG GTTTGAGAGA ACTAGTGGTA GGCACTAATT CGCCACGCAC CAAAATAACT AATTTTGATT   
  
  
- ACACTAAATT TAGACTAATA GGGGCTCAAT TAATACTTCC GAGCCTTGAG GTAAAAGAAG AGGAAAAAAA   
  
  
- GTGAAAAAAG AACCTGTCCC CTGCTTTCAA CCAAGTACAC CGTAGTTTTT TCATCACAAA TTATTAAATC   
  
  
- TACGAAAATT GTAAGTAACC TGTTCTGATT TTTCTAGTCT AAAGTATCGT TTAGATAGTG AATCAGCTGT   
  
  
- ATTTATACAT TTTTTGAGCT CTAAAATAAT TACCCAGAAC TTAAATTAAT TTTGGAAATT TAATTTATTT   
  
  
- ATAATAAGAG CAGTGCTCAG TTAGAGAACA TTGTAGCAGT AAAGAAATTC TAGTCACTGG AACAACTTTT   
  
  
- GATTTTTGTG TGTGTTAAAA TTAGTAAGAA GAGAAAAAGC ACATAAGTTG AGTGAATTTA AGTTGTATAT   
  
  
- CTAACTTTCT AATGTTCAGA TCTGGATAAT ATTAAGTTAT TTCTAAGATT AATCGTTCAA CTTATTCACA   
  
  
- AGATTTGTGG TTAATGAAAA AAGTAGCTAC TAAAGATAAG TTTGATGCTA CATTTGATTA TAGATTGTTA   
  
  
- ACAGAACTTA AAGCTTGTTT ATTAGTGTCG ACGTCGAAAG TTTTGTAATG GGTCTTGGTA AAAGGAACTG   
  
  
- AAGAAAAAAT AAAAGAATAC GAACTTTATT ATGTCTTGTC TAATTGCTCA AGACATGTGA CTTTTACTTT   
  
  
- GTTCTGAAAC TTGAAGATTG AACCATATAA GATAAAACAA TATGGCAGGG TAGTACAATT CTGACCTTTT   
  
  
- GATTAATTTT CCATAAAGTA CTCTAATACT ACGTATTAAT AAAAATTTTA TTAAAAAATA AATTTTTATA   
  
  
- TAATTTTATT TTAAAGAATA AATAAAAATT ATAGTTGTCT AATTTTGATA GTATTTAGTG ATTTATTAAT   
  
  
- AGTTAAATTA TAAAAAGTTT GGTTTTTGTG TAAATTTTAT GTGAAGTTTT GACACAGTTG AACTTCGAGT   
  
  
- TTTAAGTGTC TCTTCAATTT TTGTAAATTG ACATTTACGC TTCGATGTGA CAGGTTTGGA AGGTCACTGT   
  
  
- TACTTTTCGA ACGGTGAAAC ATAACTTTAT AACCATGGTG CACATTCTGT ATACGCTTGT TCCGTTACCA   
  
  
- CTGCAGCCGT TCCATATCCG TTAGCACCCT GGTTAGGGCG GCGGCATGCA GTGATTTGTG TGCACGGAAT   
  
  
- TTTTGTTTAA GGGTTCGGAA GGATCGTTGT TTCTTGTTC

+     GATA-motif

| Site Name | Organism | Position | Strand | Matrix score. | sequence | function |
| --- | --- | --- | --- | --- | --- | --- |
| GATA-motif | Arabidopsis thaliana | 942 | + | 10 | AAGATAAGATT | part of a light responsive element |

>Potri.002G039100.1   
+ TTTTTTTTTA TCCGTAATTG ATTATTCAAA ACCTTTCCAG CTTAAAATTT TTTCCATAAT TTAAAAACTA   
  
  
+ TTAAAGGTTG AAAACACTTT TTTTTTCTCT GAGATTTAAA ACTAATACTA TAAAAACTCT AACTCTAACT   
  
  
+ TTCCGTTGTT TATTATTTAT CACCGAATCT TCTTGATAAA TGTATTTCTT AAAGTATATT TTATATAAAA   
  
  
+ ATATTTTTTT TATTTTTAAA ATTTTCCTTT AATATACATC ATGTAAAGTT TCAAATCGAT TTAAAAATTA   
  
  
+ AACATAATTT TTTTAAAAAA TTAACGGAAC CGCAAAAAAA TAAAGAGATG GTCGCCATCA ACCGTGTAGC   
  
  
+ AACCCTTTCT TTTCGGAGGA CTTTCAAAAT TCAGGCATTT CCATGGAGAT TTTTTGTCTT CTTGAAGACC   
  
  
+ GGGAACGGTT TCAAGCAATC CCACGAAAGG GGGCCTGAAT CTAGACAACG TTGCTATCAA ACAGCTTCAT   
  
  
+ CTGTGTTAAA TATTTTATGG ATTTGATTTA CCTGTCCCCT CTGCCAGTCT TTTCAAATCA AATTTGCCAC   
  
  
+ AAGAAACCAT GACTTTTATT TCTTTCATGT GCACATCATT ACCTTAAGTG ATAACGATTA AAGGTGGAGG   
  
  
+ TTTGCCTAAA ATTACCAGTT GATATTAAAT ACGCAACAAC TTAATTTCAC ATGAATCCTC ACCCGTATTA   
  
  
+ GCTAGGGTGC CAAACTCTCT TGATCACCAT CCGTGATTAA GCGGTGCGTG GTTTTATTGA TTAAAACTAA   
  
  
+ TGTGATTTAA ATCTGATTAT CCCCGAGTTA ATTATGAAGG CTCGGAACTC CATTTTCTTC TCCTTTTTTT   
  
  
+ CACTTTTTTC TTGGACAGGG GACGAAAGTT GGTTCATGTG GCATCAAAAA AGTAGTGTTT AATAATTTAG   
  
  
+ ATGCTTTTAA CATTCATTGG ACAAGACTAA AAAGATCAGA TTTCATAGCA AATCTATCAC TTAGTCGACA   
  
  
+ TAAATATGTA AAAAACTCGA GATTTTATTA ATGGGTCTTG AATTTAATTA AAACCTTTAA ATTAAATAAA   
  
  
+ TATTATTCTC GTCACGAGTC AATCTCTTGT AACATCGTCA TTTCTTTAAG ATCAGTGACC TTGTTGAAAA   
  
  
+ CTAAAAACAC ACACAATTTT AATCATTCTT CTCTTTTTCG TGTATTCAAC TCACTTAAAT TCAACATATA   
  
  
+ GATTGAAAGA TTACAAGTCT AGACCTATTA TAATTCAATA AAGATTCTAA TTAGCAAGTT GAATAAGTGT   
  
  
+ TCTAAACACC AATTACTTTT TTCATCGATG ATTTCTATTC AAACTACGAT GTAAACTAAT ATCTAACAAT   
  
  
+ TGTCTTGAAT TTCGAACAAA TAATCACAGC TGCAGCTTTC AAAACATTAC CCAGAACCAT TTTCCTTGAC   
  
  
+ TTCTTTTTTA TTTTCTTATG CTTGAAATAA TACAGAACAG ATTAACGAGT TCTGTACACT GAAAATGAAA   
  
  
+ CAAGACTTTG AACTTCTAAC TTGGTATATT CTATTTTGTT ATACCGTCCC ATCATGTTAA GACTGGAAAA   
  
  
+ CTAATTAAAA GGTATTTCAT GAGATTATGA TGCATAATTA TTTTTAAAAT AATTTTTTAT TTAAAAATAT   
  
  
+ ATTAAAATAA AATTTCTTAT TTATTTTTAA TATCAACAGA TTAAAACTAT CATAAATCAC TAAATAATTA   
  
  
+ TCAATTTAAT ATTTTTCAAA CCAAAAACAC ATTTAAAATA CACTTCAAAA CTGTGTCAAC TTGAAGCTCA   
  
  
+ AAATTCACAG AGAAGTTAAA AACATTTAAC TGTAAATGCG AAGCTACACT GTCCAAACCT TCCAGTGACA   
  
  
+ ATGAAAAGCT TGCCACTTTG TATTGAAATA TTGGTACCAC GTGTAAGACA TATGCGAACA AGGCAATGGT   
  
  
+ GACGTCGGCA AGGTATAGGC AATCGTGGGA CCAATCCCGC CGCCGTACGT CACTAAACAC ACGTGCCTTA   
  
  
+ AAAACAAATT CCCAAGCCTT CCTAGCAACA AAGAACAAG  

- AAAAAAAAAT AGGCATTAAC TAATAAGTTT TGGAAAGGTC GAATTTTAAA AAAGGTATTA AATTTTTGAT   
  
  
- AATTTCCAAC TTTTGTGAAA AAAAAAGAGA CTCTAAATTT TGATTATGAT ATTTTTGAGA TTGAGATTGA   
  
  
- AAGGCAACAA ATAATAAATA GTGGCTTAGA AGAACTATTT ACATAAAGAA TTTCATATAA AATATATTTT   
  
  
- TATAAAAAAA ATAAAAATTT TAAAAGGAAA TTATATGTAG TACATTTCAA AGTTTAGCTA AATTTTTAAT   
  
  
- TTGTATTAAA AAAATTTTTT AATTGCCTTG GCGTTTTTTT ATTTCTCTAC CAGCGGTAGT TGGCACATCG   
  
  
- TTGGGAAAGA AAAGCCTCCT GAAAGTTTTA AGTCCGTAAA GGTACCTCTA AAAAACAGAA GAACTTCTGG   
  
  
- CCCTTGCCAA AGTTCGTTAG GGTGCTTTCC CCCGGACTTA GATCTGTTGC AACGATAGTT TGTCGAAGTA   
  
  
- GACACAATTT ATAAAATACC TAAACTAAAT GGACAGGGGA GACGGTCAGA AAAGTTTAGT TTAAACGGTG   
  
  
- TTCTTTGGTA CTGAAAATAA AGAAAGTACA CGTGTAGTAA TGGAATTCAC TATTGCTAAT TTCCACCTCC   
  
  
- AAACGGATTT TAATGGTCAA CTATAATTTA TGCGTTGTTG AATTAAAGTG TACTTAGGAG TGGGCATAAT   
  
  
- CGATCCCACG GTTTGAGAGA ACTAGTGGTA GGCACTAATT CGCCACGCAC CAAAATAACT AATTTTGATT   
  
  
- ACACTAAATT TAGACTAATA GGGGCTCAAT TAATACTTCC GAGCCTTGAG GTAAAAGAAG AGGAAAAAAA   
  
  
- GTGAAAAAAG AACCTGTCCC CTGCTTTCAA CCAAGTACAC CGTAGTTTTT TCATCACAAA TTATTAAATC   
  
  
- TACGAAAATT GTAAGTAACC TGTTCTGATT TTTCTAGTCT AAAGTATCGT TTAGATAGTG AATCAGCTGT   
  
  
- ATTTATACAT TTTTTGAGCT CTAAAATAAT TACCCAGAAC TTAAATTAAT TTTGGAAATT TAATTTATTT   
  
  
- ATAATAAGAG CAGTGCTCAG TTAGAGAACA TTGTAGCAGT AAAGAAATTC TAGTCACTGG AACAACTTTT   
  
  
- GATTTTTGTG TGTGTTAAAA TTAGTAAGAA GAGAAAAAGC ACATAAGTTG AGTGAATTTA AGTTGTATAT   
  
  
- CTAACTTTCT AATGTTCAGA TCTGGATAAT ATTAAGTTAT TTCTAAGATT AATCGTTCAA CTTATTCACA   
  
  
- AGATTTGTGG TTAATGAAAA AAGTAGCTAC TAAAGATAAG TTTGATGCTA CATTTGATTA TAGATTGTTA   
  
  
- ACAGAACTTA AAGCTTGTTT ATTAGTGTCG ACGTCGAAAG TTTTGTAATG GGTCTTGGTA AAAGGAACTG   
  
  
- AAGAAAAAAT AAAAGAATAC GAACTTTATT ATGTCTTGTC TAATTGCTCA AGACATGTGA CTTTTACTTT   
  
  
- GTTCTGAAAC TTGAAGATTG AACCATATAA GATAAAACAA TATGGCAGGG TAGTACAATT CTGACCTTTT   
  
  
- GATTAATTTT CCATAAAGTA CTCTAATACT ACGTATTAAT AAAAATTTTA TTAAAAAATA AATTTTTATA   
  
  
- TAATTTTATT TTAAAGAATA AATAAAAATT ATAGTTGTCT AATTTTGATA GTATTTAGTG ATTTATTAAT   
  
  
- AGTTAAATTA TAAAAAGTTT GGTTTTTGTG TAAATTTTAT GTGAAGTTTT GACACAGTTG AACTTCGAGT   
  
  
- TTTAAGTGTC TCTTCAATTT TTGTAAATTG ACATTTACGC TTCGATGTGA CAGGTTTGGA AGGTCACTGT   
  
  
- TACTTTTCGA ACGGTGAAAC ATAACTTTAT AACCATGGTG CACATTCTGT ATACGCTTGT TCCGTTACCA   
  
  
- CTGCAGCCGT TCCATATCCG TTAGCACCCT GGTTAGGGCG GCGGCATGCA GTGATTTGTG TGCACGGAAT   
  
  
- TTTTGTTTAA GGGTTCGGAA GGATCGTTGT TTCTTGTTC

+     LTR

| Site Name | Organism | Position | Strand | Matrix score. | sequence | function |
| --- | --- | --- | --- | --- | --- | --- |
| LTR | Hordeum vulgare | 361 | - | 6 | CCGAAA | cis-acting element involved in low-temperature responsiveness |

>Potri.002G039100.1   
+ TTTTTTTTTA TCCGTAATTG ATTATTCAAA ACCTTTCCAG CTTAAAATTT TTTCCATAAT TTAAAAACTA   
  
  
+ TTAAAGGTTG AAAACACTTT TTTTTTCTCT GAGATTTAAA ACTAATACTA TAAAAACTCT AACTCTAACT   
  
  
+ TTCCGTTGTT TATTATTTAT CACCGAATCT TCTTGATAAA TGTATTTCTT AAAGTATATT TTATATAAAA   
  
  
+ ATATTTTTTT TATTTTTAAA ATTTTCCTTT AATATACATC ATGTAAAGTT TCAAATCGAT TTAAAAATTA   
  
  
+ AACATAATTT TTTTAAAAAA TTAACGGAAC CGCAAAAAAA TAAAGAGATG GTCGCCATCA ACCGTGTAGC   
  
  
+ AACCCTTTCT TTTCGGAGGA CTTTCAAAAT TCAGGCATTT CCATGGAGAT TTTTTGTCTT CTTGAAGACC   
  
  
+ GGGAACGGTT TCAAGCAATC CCACGAAAGG GGGCCTGAAT CTAGACAACG TTGCTATCAA ACAGCTTCAT   
  
  
+ CTGTGTTAAA TATTTTATGG ATTTGATTTA CCTGTCCCCT CTGCCAGTCT TTTCAAATCA AATTTGCCAC   
  
  
+ AAGAAACCAT GACTTTTATT TCTTTCATGT GCACATCATT ACCTTAAGTG ATAACGATTA AAGGTGGAGG   
  
  
+ TTTGCCTAAA ATTACCAGTT GATATTAAAT ACGCAACAAC TTAATTTCAC ATGAATCCTC ACCCGTATTA   
  
  
+ GCTAGGGTGC CAAACTCTCT TGATCACCAT CCGTGATTAA GCGGTGCGTG GTTTTATTGA TTAAAACTAA   
  
  
+ TGTGATTTAA ATCTGATTAT CCCCGAGTTA ATTATGAAGG CTCGGAACTC CATTTTCTTC TCCTTTTTTT   
  
  
+ CACTTTTTTC TTGGACAGGG GACGAAAGTT GGTTCATGTG GCATCAAAAA AGTAGTGTTT AATAATTTAG   
  
  
+ ATGCTTTTAA CATTCATTGG ACAAGACTAA AAAGATCAGA TTTCATAGCA AATCTATCAC TTAGTCGACA   
  
  
+ TAAATATGTA AAAAACTCGA GATTTTATTA ATGGGTCTTG AATTTAATTA AAACCTTTAA ATTAAATAAA   
  
  
+ TATTATTCTC GTCACGAGTC AATCTCTTGT AACATCGTCA TTTCTTTAAG ATCAGTGACC TTGTTGAAAA   
  
  
+ CTAAAAACAC ACACAATTTT AATCATTCTT CTCTTTTTCG TGTATTCAAC TCACTTAAAT TCAACATATA   
  
  
+ GATTGAAAGA TTACAAGTCT AGACCTATTA TAATTCAATA AAGATTCTAA TTAGCAAGTT GAATAAGTGT   
  
  
+ TCTAAACACC AATTACTTTT TTCATCGATG ATTTCTATTC AAACTACGAT GTAAACTAAT ATCTAACAAT   
  
  
+ TGTCTTGAAT TTCGAACAAA TAATCACAGC TGCAGCTTTC AAAACATTAC CCAGAACCAT TTTCCTTGAC   
  
  
+ TTCTTTTTTA TTTTCTTATG CTTGAAATAA TACAGAACAG ATTAACGAGT TCTGTACACT GAAAATGAAA   
  
  
+ CAAGACTTTG AACTTCTAAC TTGGTATATT CTATTTTGTT ATACCGTCCC ATCATGTTAA GACTGGAAAA   
  
  
+ CTAATTAAAA GGTATTTCAT GAGATTATGA TGCATAATTA TTTTTAAAAT AATTTTTTAT TTAAAAATAT   
  
  
+ ATTAAAATAA AATTTCTTAT TTATTTTTAA TATCAACAGA TTAAAACTAT CATAAATCAC TAAATAATTA   
  
  
+ TCAATTTAAT ATTTTTCAAA CCAAAAACAC ATTTAAAATA CACTTCAAAA CTGTGTCAAC TTGAAGCTCA   
  
  
+ AAATTCACAG AGAAGTTAAA AACATTTAAC TGTAAATGCG AAGCTACACT GTCCAAACCT TCCAGTGACA   
  
  
+ ATGAAAAGCT TGCCACTTTG TATTGAAATA TTGGTACCAC GTGTAAGACA TATGCGAACA AGGCAATGGT   
  
  
+ GACGTCGGCA AGGTATAGGC AATCGTGGGA CCAATCCCGC CGCCGTACGT CACTAAACAC ACGTGCCTTA   
  
  
+ AAAACAAATT CCCAAGCCTT CCTAGCAACA AAGAACAAG  

- AAAAAAAAAT AGGCATTAAC TAATAAGTTT TGGAAAGGTC GAATTTTAAA AAAGGTATTA AATTTTTGAT   
  
  
- AATTTCCAAC TTTTGTGAAA AAAAAAGAGA CTCTAAATTT TGATTATGAT ATTTTTGAGA TTGAGATTGA   
  
  
- AAGGCAACAA ATAATAAATA GTGGCTTAGA AGAACTATTT ACATAAAGAA TTTCATATAA AATATATTTT   
  
  
- TATAAAAAAA ATAAAAATTT TAAAAGGAAA TTATATGTAG TACATTTCAA AGTTTAGCTA AATTTTTAAT   
  
  
- TTGTATTAAA AAAATTTTTT AATTGCCTTG GCGTTTTTTT ATTTCTCTAC CAGCGGTAGT TGGCACATCG   
  
  
- TTGGGAAAGA AAAGCCTCCT GAAAGTTTTA AGTCCGTAAA GGTACCTCTA AAAAACAGAA GAACTTCTGG   
  
  
- CCCTTGCCAA AGTTCGTTAG GGTGCTTTCC CCCGGACTTA GATCTGTTGC AACGATAGTT TGTCGAAGTA   
  
  
- GACACAATTT ATAAAATACC TAAACTAAAT GGACAGGGGA GACGGTCAGA AAAGTTTAGT TTAAACGGTG   
  
  
- TTCTTTGGTA CTGAAAATAA AGAAAGTACA CGTGTAGTAA TGGAATTCAC TATTGCTAAT TTCCACCTCC   
  
  
- AAACGGATTT TAATGGTCAA CTATAATTTA TGCGTTGTTG AATTAAAGTG TACTTAGGAG TGGGCATAAT   
  
  
- CGATCCCACG GTTTGAGAGA ACTAGTGGTA GGCACTAATT CGCCACGCAC CAAAATAACT AATTTTGATT   
  
  
- ACACTAAATT TAGACTAATA GGGGCTCAAT TAATACTTCC GAGCCTTGAG GTAAAAGAAG AGGAAAAAAA   
  
  
- GTGAAAAAAG AACCTGTCCC CTGCTTTCAA CCAAGTACAC CGTAGTTTTT TCATCACAAA TTATTAAATC   
  
  
- TACGAAAATT GTAAGTAACC TGTTCTGATT TTTCTAGTCT AAAGTATCGT TTAGATAGTG AATCAGCTGT   
  
  
- ATTTATACAT TTTTTGAGCT CTAAAATAAT TACCCAGAAC TTAAATTAAT TTTGGAAATT TAATTTATTT   
  
  
- ATAATAAGAG CAGTGCTCAG TTAGAGAACA TTGTAGCAGT AAAGAAATTC TAGTCACTGG AACAACTTTT   
  
  
- GATTTTTGTG TGTGTTAAAA TTAGTAAGAA GAGAAAAAGC ACATAAGTTG AGTGAATTTA AGTTGTATAT   
  
  
- CTAACTTTCT AATGTTCAGA TCTGGATAAT ATTAAGTTAT TTCTAAGATT AATCGTTCAA CTTATTCACA   
  
  
- AGATTTGTGG TTAATGAAAA AAGTAGCTAC TAAAGATAAG TTTGATGCTA CATTTGATTA TAGATTGTTA   
  
  
- ACAGAACTTA AAGCTTGTTT ATTAGTGTCG ACGTCGAAAG TTTTGTAATG GGTCTTGGTA AAAGGAACTG   
  
  
- AAGAAAAAAT AAAAGAATAC GAACTTTATT ATGTCTTGTC TAATTGCTCA AGACATGTGA CTTTTACTTT   
  
  
- GTTCTGAAAC TTGAAGATTG AACCATATAA GATAAAACAA TATGGCAGGG TAGTACAATT CTGACCTTTT   
  
  
- GATTAATTTT CCATAAAGTA CTCTAATACT ACGTATTAAT AAAAATTTTA TTAAAAAATA AATTTTTATA   
  
  
- TAATTTTATT TTAAAGAATA AATAAAAATT ATAGTTGTCT AATTTTGATA GTATTTAGTG ATTTATTAAT   
  
  
- AGTTAAATTA TAAAAAGTTT GGTTTTTGTG TAAATTTTAT GTGAAGTTTT GACACAGTTG AACTTCGAGT   
  
  
- TTTAAGTGTC TCTTCAATTT TTGTAAATTG ACATTTACGC TTCGATGTGA CAGGTTTGGA AGGTCACTGT   
  
  
- TACTTTTCGA ACGGTGAAAC ATAACTTTAT AACCATGGTG CACATTCTGT ATACGCTTGT TCCGTTACCA   
  
  
- CTGCAGCCGT TCCATATCCG TTAGCACCCT GGTTAGGGCG GCGGCATGCA GTGATTTGTG TGCACGGAAT   
  
  
- TTTTGTTTAA GGGTTCGGAA GGATCGTTGT TTCTTGTTC

+     MBS

| Site Name | Organism | Position | Strand | Matrix score. | sequence | function |
| --- | --- | --- | --- | --- | --- | --- |
| MBS | Arabidopsis thaliana | 646 | - | 6 | CAACTG | MYB binding site involved in drought-inducibility |

>Potri.002G039100.1   
+ TTTTTTTTTA TCCGTAATTG ATTATTCAAA ACCTTTCCAG CTTAAAATTT TTTCCATAAT TTAAAAACTA   
  
  
+ TTAAAGGTTG AAAACACTTT TTTTTTCTCT GAGATTTAAA ACTAATACTA TAAAAACTCT AACTCTAACT   
  
  
+ TTCCGTTGTT TATTATTTAT CACCGAATCT TCTTGATAAA TGTATTTCTT AAAGTATATT TTATATAAAA   
  
  
+ ATATTTTTTT TATTTTTAAA ATTTTCCTTT AATATACATC ATGTAAAGTT TCAAATCGAT TTAAAAATTA   
  
  
+ AACATAATTT TTTTAAAAAA TTAACGGAAC CGCAAAAAAA TAAAGAGATG GTCGCCATCA ACCGTGTAGC   
  
  
+ AACCCTTTCT TTTCGGAGGA CTTTCAAAAT TCAGGCATTT CCATGGAGAT TTTTTGTCTT CTTGAAGACC   
  
  
+ GGGAACGGTT TCAAGCAATC CCACGAAAGG GGGCCTGAAT CTAGACAACG TTGCTATCAA ACAGCTTCAT   
  
  
+ CTGTGTTAAA TATTTTATGG ATTTGATTTA CCTGTCCCCT CTGCCAGTCT TTTCAAATCA AATTTGCCAC   
  
  
+ AAGAAACCAT GACTTTTATT TCTTTCATGT GCACATCATT ACCTTAAGTG ATAACGATTA AAGGTGGAGG   
  
  
+ TTTGCCTAAA ATTACCAGTT GATATTAAAT ACGCAACAAC TTAATTTCAC ATGAATCCTC ACCCGTATTA   
  
  
+ GCTAGGGTGC CAAACTCTCT TGATCACCAT CCGTGATTAA GCGGTGCGTG GTTTTATTGA TTAAAACTAA   
  
  
+ TGTGATTTAA ATCTGATTAT CCCCGAGTTA ATTATGAAGG CTCGGAACTC CATTTTCTTC TCCTTTTTTT   
  
  
+ CACTTTTTTC TTGGACAGGG GACGAAAGTT GGTTCATGTG GCATCAAAAA AGTAGTGTTT AATAATTTAG   
  
  
+ ATGCTTTTAA CATTCATTGG ACAAGACTAA AAAGATCAGA TTTCATAGCA AATCTATCAC TTAGTCGACA   
  
  
+ TAAATATGTA AAAAACTCGA GATTTTATTA ATGGGTCTTG AATTTAATTA AAACCTTTAA ATTAAATAAA   
  
  
+ TATTATTCTC GTCACGAGTC AATCTCTTGT AACATCGTCA TTTCTTTAAG ATCAGTGACC TTGTTGAAAA   
  
  
+ CTAAAAACAC ACACAATTTT AATCATTCTT CTCTTTTTCG TGTATTCAAC TCACTTAAAT TCAACATATA   
  
  
+ GATTGAAAGA TTACAAGTCT AGACCTATTA TAATTCAATA AAGATTCTAA TTAGCAAGTT GAATAAGTGT   
  
  
+ TCTAAACACC AATTACTTTT TTCATCGATG ATTTCTATTC AAACTACGAT GTAAACTAAT ATCTAACAAT   
  
  
+ TGTCTTGAAT TTCGAACAAA TAATCACAGC TGCAGCTTTC AAAACATTAC CCAGAACCAT TTTCCTTGAC   
  
  
+ TTCTTTTTTA TTTTCTTATG CTTGAAATAA TACAGAACAG ATTAACGAGT TCTGTACACT GAAAATGAAA   
  
  
+ CAAGACTTTG AACTTCTAAC TTGGTATATT CTATTTTGTT ATACCGTCCC ATCATGTTAA GACTGGAAAA   
  
  
+ CTAATTAAAA GGTATTTCAT GAGATTATGA TGCATAATTA TTTTTAAAAT AATTTTTTAT TTAAAAATAT   
  
  
+ ATTAAAATAA AATTTCTTAT TTATTTTTAA TATCAACAGA TTAAAACTAT CATAAATCAC TAAATAATTA   
  
  
+ TCAATTTAAT ATTTTTCAAA CCAAAAACAC ATTTAAAATA CACTTCAAAA CTGTGTCAAC TTGAAGCTCA   
  
  
+ AAATTCACAG AGAAGTTAAA AACATTTAAC TGTAAATGCG AAGCTACACT GTCCAAACCT TCCAGTGACA   
  
  
+ ATGAAAAGCT TGCCACTTTG TATTGAAATA TTGGTACCAC GTGTAAGACA TATGCGAACA AGGCAATGGT   
  
  
+ GACGTCGGCA AGGTATAGGC AATCGTGGGA CCAATCCCGC CGCCGTACGT CACTAAACAC ACGTGCCTTA   
  
  
+ AAAACAAATT CCCAAGCCTT CCTAGCAACA AAGAACAAG  

- AAAAAAAAAT AGGCATTAAC TAATAAGTTT TGGAAAGGTC GAATTTTAAA AAAGGTATTA AATTTTTGAT   
  
  
- AATTTCCAAC TTTTGTGAAA AAAAAAGAGA CTCTAAATTT TGATTATGAT ATTTTTGAGA TTGAGATTGA   
  
  
- AAGGCAACAA ATAATAAATA GTGGCTTAGA AGAACTATTT ACATAAAGAA TTTCATATAA AATATATTTT   
  
  
- TATAAAAAAA ATAAAAATTT TAAAAGGAAA TTATATGTAG TACATTTCAA AGTTTAGCTA AATTTTTAAT   
  
  
- TTGTATTAAA AAAATTTTTT AATTGCCTTG GCGTTTTTTT ATTTCTCTAC CAGCGGTAGT TGGCACATCG   
  
  
- TTGGGAAAGA AAAGCCTCCT GAAAGTTTTA AGTCCGTAAA GGTACCTCTA AAAAACAGAA GAACTTCTGG   
  
  
- CCCTTGCCAA AGTTCGTTAG GGTGCTTTCC CCCGGACTTA GATCTGTTGC AACGATAGTT TGTCGAAGTA   
  
  
- GACACAATTT ATAAAATACC TAAACTAAAT GGACAGGGGA GACGGTCAGA AAAGTTTAGT TTAAACGGTG   
  
  
- TTCTTTGGTA CTGAAAATAA AGAAAGTACA CGTGTAGTAA TGGAATTCAC TATTGCTAAT TTCCACCTCC   
  
  
- AAACGGATTT TAATGGTCAA CTATAATTTA TGCGTTGTTG AATTAAAGTG TACTTAGGAG TGGGCATAAT   
  
  
- CGATCCCACG GTTTGAGAGA ACTAGTGGTA GGCACTAATT CGCCACGCAC CAAAATAACT AATTTTGATT   
  
  
- ACACTAAATT TAGACTAATA GGGGCTCAAT TAATACTTCC GAGCCTTGAG GTAAAAGAAG AGGAAAAAAA   
  
  
- GTGAAAAAAG AACCTGTCCC CTGCTTTCAA CCAAGTACAC CGTAGTTTTT TCATCACAAA TTATTAAATC   
  
  
- TACGAAAATT GTAAGTAACC TGTTCTGATT TTTCTAGTCT AAAGTATCGT TTAGATAGTG AATCAGCTGT   
  
  
- ATTTATACAT TTTTTGAGCT CTAAAATAAT TACCCAGAAC TTAAATTAAT TTTGGAAATT TAATTTATTT   
  
  
- ATAATAAGAG CAGTGCTCAG TTAGAGAACA TTGTAGCAGT AAAGAAATTC TAGTCACTGG AACAACTTTT   
  
  
- GATTTTTGTG TGTGTTAAAA TTAGTAAGAA GAGAAAAAGC ACATAAGTTG AGTGAATTTA AGTTGTATAT   
  
  
- CTAACTTTCT AATGTTCAGA TCTGGATAAT ATTAAGTTAT TTCTAAGATT AATCGTTCAA CTTATTCACA   
  
  
- AGATTTGTGG TTAATGAAAA AAGTAGCTAC TAAAGATAAG TTTGATGCTA CATTTGATTA TAGATTGTTA   
  
  
- ACAGAACTTA AAGCTTGTTT ATTAGTGTCG ACGTCGAAAG TTTTGTAATG GGTCTTGGTA AAAGGAACTG   
  
  
- AAGAAAAAAT AAAAGAATAC GAACTTTATT ATGTCTTGTC TAATTGCTCA AGACATGTGA CTTTTACTTT   
  
  
- GTTCTGAAAC TTGAAGATTG AACCATATAA GATAAAACAA TATGGCAGGG TAGTACAATT CTGACCTTTT   
  
  
- GATTAATTTT CCATAAAGTA CTCTAATACT ACGTATTAAT AAAAATTTTA TTAAAAAATA AATTTTTATA   
  
  
- TAATTTTATT TTAAAGAATA AATAAAAATT ATAGTTGTCT AATTTTGATA GTATTTAGTG ATTTATTAAT   
  
  
- AGTTAAATTA TAAAAAGTTT GGTTTTTGTG TAAATTTTAT GTGAAGTTTT GACACAGTTG AACTTCGAGT   
  
  
- TTTAAGTGTC TCTTCAATTT TTGTAAATTG ACATTTACGC TTCGATGTGA CAGGTTTGGA AGGTCACTGT   
  
  
- TACTTTTCGA ACGGTGAAAC ATAACTTTAT AACCATGGTG CACATTCTGT ATACGCTTGT TCCGTTACCA   
  
  
- CTGCAGCCGT TCCATATCCG TTAGCACCCT GGTTAGGGCG GCGGCATGCA GTGATTTGTG TGCACGGAAT   
  
  
- TTTTGTTTAA GGGTTCGGAA GGATCGTTGT TTCTTGTTC

+     MYB

| Site Name | Organism | Position | Strand | Matrix score. | sequence | function |
| --- | --- | --- | --- | --- | --- | --- |
| MYB | Arabidopsis thaliana | 1644 | + | 6 | CAACAG |  |

>Potri.002G039100.1   
+ TTTTTTTTTA TCCGTAATTG ATTATTCAAA ACCTTTCCAG CTTAAAATTT TTTCCATAAT TTAAAAACTA   
  
  
+ TTAAAGGTTG AAAACACTTT TTTTTTCTCT GAGATTTAAA ACTAATACTA TAAAAACTCT AACTCTAACT   
  
  
+ TTCCGTTGTT TATTATTTAT CACCGAATCT TCTTGATAAA TGTATTTCTT AAAGTATATT TTATATAAAA   
  
  
+ ATATTTTTTT TATTTTTAAA ATTTTCCTTT AATATACATC ATGTAAAGTT TCAAATCGAT TTAAAAATTA   
  
  
+ AACATAATTT TTTTAAAAAA TTAACGGAAC CGCAAAAAAA TAAAGAGATG GTCGCCATCA ACCGTGTAGC   
  
  
+ AACCCTTTCT TTTCGGAGGA CTTTCAAAAT TCAGGCATTT CCATGGAGAT TTTTTGTCTT CTTGAAGACC   
  
  
+ GGGAACGGTT TCAAGCAATC CCACGAAAGG GGGCCTGAAT CTAGACAACG TTGCTATCAA ACAGCTTCAT   
  
  
+ CTGTGTTAAA TATTTTATGG ATTTGATTTA CCTGTCCCCT CTGCCAGTCT TTTCAAATCA AATTTGCCAC   
  
  
+ AAGAAACCAT GACTTTTATT TCTTTCATGT GCACATCATT ACCTTAAGTG ATAACGATTA AAGGTGGAGG   
  
  
+ TTTGCCTAAA ATTACCAGTT GATATTAAAT ACGCAACAAC TTAATTTCAC ATGAATCCTC ACCCGTATTA   
  
  
+ GCTAGGGTGC CAAACTCTCT TGATCACCAT CCGTGATTAA GCGGTGCGTG GTTTTATTGA TTAAAACTAA   
  
  
+ TGTGATTTAA ATCTGATTAT CCCCGAGTTA ATTATGAAGG CTCGGAACTC CATTTTCTTC TCCTTTTTTT   
  
  
+ CACTTTTTTC TTGGACAGGG GACGAAAGTT GGTTCATGTG GCATCAAAAA AGTAGTGTTT AATAATTTAG   
  
  
+ ATGCTTTTAA CATTCATTGG ACAAGACTAA AAAGATCAGA TTTCATAGCA AATCTATCAC TTAGTCGACA   
  
  
+ TAAATATGTA AAAAACTCGA GATTTTATTA ATGGGTCTTG AATTTAATTA AAACCTTTAA ATTAAATAAA   
  
  
+ TATTATTCTC GTCACGAGTC AATCTCTTGT AACATCGTCA TTTCTTTAAG ATCAGTGACC TTGTTGAAAA   
  
  
+ CTAAAAACAC ACACAATTTT AATCATTCTT CTCTTTTTCG TGTATTCAAC TCACTTAAAT TCAACATATA   
  
  
+ GATTGAAAGA TTACAAGTCT AGACCTATTA TAATTCAATA AAGATTCTAA TTAGCAAGTT GAATAAGTGT   
  
  
+ TCTAAACACC AATTACTTTT TTCATCGATG ATTTCTATTC AAACTACGAT GTAAACTAAT ATCTAACAAT   
  
  
+ TGTCTTGAAT TTCGAACAAA TAATCACAGC TGCAGCTTTC AAAACATTAC CCAGAACCAT TTTCCTTGAC   
  
  
+ TTCTTTTTTA TTTTCTTATG CTTGAAATAA TACAGAACAG ATTAACGAGT TCTGTACACT GAAAATGAAA   
  
  
+ CAAGACTTTG AACTTCTAAC TTGGTATATT CTATTTTGTT ATACCGTCCC ATCATGTTAA GACTGGAAAA   
  
  
+ CTAATTAAAA GGTATTTCAT GAGATTATGA TGCATAATTA TTTTTAAAAT AATTTTTTAT TTAAAAATAT   
  
  
+ ATTAAAATAA AATTTCTTAT TTATTTTTAA TATCAACAGA TTAAAACTAT CATAAATCAC TAAATAATTA   
  
  
+ TCAATTTAAT ATTTTTCAAA CCAAAAACAC ATTTAAAATA CACTTCAAAA CTGTGTCAAC TTGAAGCTCA   
  
  
+ AAATTCACAG AGAAGTTAAA AACATTTAAC TGTAAATGCG AAGCTACACT GTCCAAACCT TCCAGTGACA   
  
  
+ ATGAAAAGCT TGCCACTTTG TATTGAAATA TTGGTACCAC GTGTAAGACA TATGCGAACA AGGCAATGGT   
  
  
+ GACGTCGGCA AGGTATAGGC AATCGTGGGA CCAATCCCGC CGCCGTACGT CACTAAACAC ACGTGCCTTA   
  
  
+ AAAACAAATT CCCAAGCCTT CCTAGCAACA AAGAACAAG  

- AAAAAAAAAT AGGCATTAAC TAATAAGTTT TGGAAAGGTC GAATTTTAAA AAAGGTATTA AATTTTTGAT   
  
  
- AATTTCCAAC TTTTGTGAAA AAAAAAGAGA CTCTAAATTT TGATTATGAT ATTTTTGAGA TTGAGATTGA   
  
  
- AAGGCAACAA ATAATAAATA GTGGCTTAGA AGAACTATTT ACATAAAGAA TTTCATATAA AATATATTTT   
  
  
- TATAAAAAAA ATAAAAATTT TAAAAGGAAA TTATATGTAG TACATTTCAA AGTTTAGCTA AATTTTTAAT   
  
  
- TTGTATTAAA AAAATTTTTT AATTGCCTTG GCGTTTTTTT ATTTCTCTAC CAGCGGTAGT TGGCACATCG   
  
  
- TTGGGAAAGA AAAGCCTCCT GAAAGTTTTA AGTCCGTAAA GGTACCTCTA AAAAACAGAA GAACTTCTGG   
  
  
- CCCTTGCCAA AGTTCGTTAG GGTGCTTTCC CCCGGACTTA GATCTGTTGC AACGATAGTT TGTCGAAGTA   
  
  
- GACACAATTT ATAAAATACC TAAACTAAAT GGACAGGGGA GACGGTCAGA AAAGTTTAGT TTAAACGGTG   
  
  
- TTCTTTGGTA CTGAAAATAA AGAAAGTACA CGTGTAGTAA TGGAATTCAC TATTGCTAAT TTCCACCTCC   
  
  
- AAACGGATTT TAATGGTCAA CTATAATTTA TGCGTTGTTG AATTAAAGTG TACTTAGGAG TGGGCATAAT   
  
  
- CGATCCCACG GTTTGAGAGA ACTAGTGGTA GGCACTAATT CGCCACGCAC CAAAATAACT AATTTTGATT   
  
  
- ACACTAAATT TAGACTAATA GGGGCTCAAT TAATACTTCC GAGCCTTGAG GTAAAAGAAG AGGAAAAAAA   
  
  
- GTGAAAAAAG AACCTGTCCC CTGCTTTCAA CCAAGTACAC CGTAGTTTTT TCATCACAAA TTATTAAATC   
  
  
- TACGAAAATT GTAAGTAACC TGTTCTGATT TTTCTAGTCT AAAGTATCGT TTAGATAGTG AATCAGCTGT   
  
  
- ATTTATACAT TTTTTGAGCT CTAAAATAAT TACCCAGAAC TTAAATTAAT TTTGGAAATT TAATTTATTT   
  
  
- ATAATAAGAG CAGTGCTCAG TTAGAGAACA TTGTAGCAGT AAAGAAATTC TAGTCACTGG AACAACTTTT   
  
  
- GATTTTTGTG TGTGTTAAAA TTAGTAAGAA GAGAAAAAGC ACATAAGTTG AGTGAATTTA AGTTGTATAT   
  
  
- CTAACTTTCT AATGTTCAGA TCTGGATAAT ATTAAGTTAT TTCTAAGATT AATCGTTCAA CTTATTCACA   
  
  
- AGATTTGTGG TTAATGAAAA AAGTAGCTAC TAAAGATAAG TTTGATGCTA CATTTGATTA TAGATTGTTA   
  
  
- ACAGAACTTA AAGCTTGTTT ATTAGTGTCG ACGTCGAAAG TTTTGTAATG GGTCTTGGTA AAAGGAACTG   
  
  
- AAGAAAAAAT AAAAGAATAC GAACTTTATT ATGTCTTGTC TAATTGCTCA AGACATGTGA CTTTTACTTT   
  
  
- GTTCTGAAAC TTGAAGATTG AACCATATAA GATAAAACAA TATGGCAGGG TAGTACAATT CTGACCTTTT   
  
  
- GATTAATTTT CCATAAAGTA CTCTAATACT ACGTATTAAT AAAAATTTTA TTAAAAAATA AATTTTTATA   
  
  
- TAATTTTATT TTAAAGAATA AATAAAAATT ATAGTTGTCT AATTTTGATA GTATTTAGTG ATTTATTAAT   
  
  
- AGTTAAATTA TAAAAAGTTT GGTTTTTGTG TAAATTTTAT GTGAAGTTTT GACACAGTTG AACTTCGAGT   
  
  
- TTTAAGTGTC TCTTCAATTT TTGTAAATTG ACATTTACGC TTCGATGTGA CAGGTTTGGA AGGTCACTGT   
  
  
- TACTTTTCGA ACGGTGAAAC ATAACTTTAT AACCATGGTG CACATTCTGT ATACGCTTGT TCCGTTACCA   
  
  
- CTGCAGCCGT TCCATATCCG TTAGCACCCT GGTTAGGGCG GCGGCATGCA GTGATTTGTG TGCACGGAAT   
  
  
- TTTTGTTTAA GGGTTCGGAA GGATCGTTGT TTCTTGTTC

+     MYB recognition site

| Site Name | Organism | Position | Strand | Matrix score. | sequence | function |
| --- | --- | --- | --- | --- | --- | --- |
| MYB recognition site | Arabidopsis thaliana | 143 | + | 6 | CCGTTG |  |

>Potri.002G039100.1   
+ TTTTTTTTTA TCCGTAATTG ATTATTCAAA ACCTTTCCAG CTTAAAATTT TTTCCATAAT TTAAAAACTA   
  
  
+ TTAAAGGTTG AAAACACTTT TTTTTTCTCT GAGATTTAAA ACTAATACTA TAAAAACTCT AACTCTAACT   
  
  
+ TTCCGTTGTT TATTATTTAT CACCGAATCT TCTTGATAAA TGTATTTCTT AAAGTATATT TTATATAAAA   
  
  
+ ATATTTTTTT TATTTTTAAA ATTTTCCTTT AATATACATC ATGTAAAGTT TCAAATCGAT TTAAAAATTA   
  
  
+ AACATAATTT TTTTAAAAAA TTAACGGAAC CGCAAAAAAA TAAAGAGATG GTCGCCATCA ACCGTGTAGC   
  
  
+ AACCCTTTCT TTTCGGAGGA CTTTCAAAAT TCAGGCATTT CCATGGAGAT TTTTTGTCTT CTTGAAGACC   
  
  
+ GGGAACGGTT TCAAGCAATC CCACGAAAGG GGGCCTGAAT CTAGACAACG TTGCTATCAA ACAGCTTCAT   
  
  
+ CTGTGTTAAA TATTTTATGG ATTTGATTTA CCTGTCCCCT CTGCCAGTCT TTTCAAATCA AATTTGCCAC   
  
  
+ AAGAAACCAT GACTTTTATT TCTTTCATGT GCACATCATT ACCTTAAGTG ATAACGATTA AAGGTGGAGG   
  
  
+ TTTGCCTAAA ATTACCAGTT GATATTAAAT ACGCAACAAC TTAATTTCAC ATGAATCCTC ACCCGTATTA   
  
  
+ GCTAGGGTGC CAAACTCTCT TGATCACCAT CCGTGATTAA GCGGTGCGTG GTTTTATTGA TTAAAACTAA   
  
  
+ TGTGATTTAA ATCTGATTAT CCCCGAGTTA ATTATGAAGG CTCGGAACTC CATTTTCTTC TCCTTTTTTT   
  
  
+ CACTTTTTTC TTGGACAGGG GACGAAAGTT GGTTCATGTG GCATCAAAAA AGTAGTGTTT AATAATTTAG   
  
  
+ ATGCTTTTAA CATTCATTGG ACAAGACTAA AAAGATCAGA TTTCATAGCA AATCTATCAC TTAGTCGACA   
  
  
+ TAAATATGTA AAAAACTCGA GATTTTATTA ATGGGTCTTG AATTTAATTA AAACCTTTAA ATTAAATAAA   
  
  
+ TATTATTCTC GTCACGAGTC AATCTCTTGT AACATCGTCA TTTCTTTAAG ATCAGTGACC TTGTTGAAAA   
  
  
+ CTAAAAACAC ACACAATTTT AATCATTCTT CTCTTTTTCG TGTATTCAAC TCACTTAAAT TCAACATATA   
  
  
+ GATTGAAAGA TTACAAGTCT AGACCTATTA TAATTCAATA AAGATTCTAA TTAGCAAGTT GAATAAGTGT   
  
  
+ TCTAAACACC AATTACTTTT TTCATCGATG ATTTCTATTC AAACTACGAT GTAAACTAAT ATCTAACAAT   
  
  
+ TGTCTTGAAT TTCGAACAAA TAATCACAGC TGCAGCTTTC AAAACATTAC CCAGAACCAT TTTCCTTGAC   
  
  
+ TTCTTTTTTA TTTTCTTATG CTTGAAATAA TACAGAACAG ATTAACGAGT TCTGTACACT GAAAATGAAA   
  
  
+ CAAGACTTTG AACTTCTAAC TTGGTATATT CTATTTTGTT ATACCGTCCC ATCATGTTAA GACTGGAAAA   
  
  
+ CTAATTAAAA GGTATTTCAT GAGATTATGA TGCATAATTA TTTTTAAAAT AATTTTTTAT TTAAAAATAT   
  
  
+ ATTAAAATAA AATTTCTTAT TTATTTTTAA TATCAACAGA TTAAAACTAT CATAAATCAC TAAATAATTA   
  
  
+ TCAATTTAAT ATTTTTCAAA CCAAAAACAC ATTTAAAATA CACTTCAAAA CTGTGTCAAC TTGAAGCTCA   
  
  
+ AAATTCACAG AGAAGTTAAA AACATTTAAC TGTAAATGCG AAGCTACACT GTCCAAACCT TCCAGTGACA   
  
  
+ ATGAAAAGCT TGCCACTTTG TATTGAAATA TTGGTACCAC GTGTAAGACA TATGCGAACA AGGCAATGGT   
  
  
+ GACGTCGGCA AGGTATAGGC AATCGTGGGA CCAATCCCGC CGCCGTACGT CACTAAACAC ACGTGCCTTA   
  
  
+ AAAACAAATT CCCAAGCCTT CCTAGCAACA AAGAACAAG  

- AAAAAAAAAT AGGCATTAAC TAATAAGTTT TGGAAAGGTC GAATTTTAAA AAAGGTATTA AATTTTTGAT   
  
  
- AATTTCCAAC TTTTGTGAAA AAAAAAGAGA CTCTAAATTT TGATTATGAT ATTTTTGAGA TTGAGATTGA   
  
  
- AAGGCAACAA ATAATAAATA GTGGCTTAGA AGAACTATTT ACATAAAGAA TTTCATATAA AATATATTTT   
  
  
- TATAAAAAAA ATAAAAATTT TAAAAGGAAA TTATATGTAG TACATTTCAA AGTTTAGCTA AATTTTTAAT   
  
  
- TTGTATTAAA AAAATTTTTT AATTGCCTTG GCGTTTTTTT ATTTCTCTAC CAGCGGTAGT TGGCACATCG   
  
  
- TTGGGAAAGA AAAGCCTCCT GAAAGTTTTA AGTCCGTAAA GGTACCTCTA AAAAACAGAA GAACTTCTGG   
  
  
- CCCTTGCCAA AGTTCGTTAG GGTGCTTTCC CCCGGACTTA GATCTGTTGC AACGATAGTT TGTCGAAGTA   
  
  
- GACACAATTT ATAAAATACC TAAACTAAAT GGACAGGGGA GACGGTCAGA AAAGTTTAGT TTAAACGGTG   
  
  
- TTCTTTGGTA CTGAAAATAA AGAAAGTACA CGTGTAGTAA TGGAATTCAC TATTGCTAAT TTCCACCTCC   
  
  
- AAACGGATTT TAATGGTCAA CTATAATTTA TGCGTTGTTG AATTAAAGTG TACTTAGGAG TGGGCATAAT   
  
  
- CGATCCCACG GTTTGAGAGA ACTAGTGGTA GGCACTAATT CGCCACGCAC CAAAATAACT AATTTTGATT   
  
  
- ACACTAAATT TAGACTAATA GGGGCTCAAT TAATACTTCC GAGCCTTGAG GTAAAAGAAG AGGAAAAAAA   
  
  
- GTGAAAAAAG AACCTGTCCC CTGCTTTCAA CCAAGTACAC CGTAGTTTTT TCATCACAAA TTATTAAATC   
  
  
- TACGAAAATT GTAAGTAACC TGTTCTGATT TTTCTAGTCT AAAGTATCGT TTAGATAGTG AATCAGCTGT   
  
  
- ATTTATACAT TTTTTGAGCT CTAAAATAAT TACCCAGAAC TTAAATTAAT TTTGGAAATT TAATTTATTT   
  
  
- ATAATAAGAG CAGTGCTCAG TTAGAGAACA TTGTAGCAGT AAAGAAATTC TAGTCACTGG AACAACTTTT   
  
  
- GATTTTTGTG TGTGTTAAAA TTAGTAAGAA GAGAAAAAGC ACATAAGTTG AGTGAATTTA AGTTGTATAT   
  
  
- CTAACTTTCT AATGTTCAGA TCTGGATAAT ATTAAGTTAT TTCTAAGATT AATCGTTCAA CTTATTCACA   
  
  
- AGATTTGTGG TTAATGAAAA AAGTAGCTAC TAAAGATAAG TTTGATGCTA CATTTGATTA TAGATTGTTA   
  
  
- ACAGAACTTA AAGCTTGTTT ATTAGTGTCG ACGTCGAAAG TTTTGTAATG GGTCTTGGTA AAAGGAACTG   
  
  
- AAGAAAAAAT AAAAGAATAC GAACTTTATT ATGTCTTGTC TAATTGCTCA AGACATGTGA CTTTTACTTT   
  
  
- GTTCTGAAAC TTGAAGATTG AACCATATAA GATAAAACAA TATGGCAGGG TAGTACAATT CTGACCTTTT   
  
  
- GATTAATTTT CCATAAAGTA CTCTAATACT ACGTATTAAT AAAAATTTTA TTAAAAAATA AATTTTTATA   
  
  
- TAATTTTATT TTAAAGAATA AATAAAAATT ATAGTTGTCT AATTTTGATA GTATTTAGTG ATTTATTAAT   
  
  
- AGTTAAATTA TAAAAAGTTT GGTTTTTGTG TAAATTTTAT GTGAAGTTTT GACACAGTTG AACTTCGAGT   
  
  
- TTTAAGTGTC TCTTCAATTT TTGTAAATTG ACATTTACGC TTCGATGTGA CAGGTTTGGA AGGTCACTGT   
  
  
- TACTTTTCGA ACGGTGAAAC ATAACTTTAT AACCATGGTG CACATTCTGT ATACGCTTGT TCCGTTACCA   
  
  
- CTGCAGCCGT TCCATATCCG TTAGCACCCT GGTTAGGGCG GCGGCATGCA GTGATTTGTG TGCACGGAAT   
  
  
- TTTTGTTTAA GGGTTCGGAA GGATCGTTGT TTCTTGTTC

+     MYC

| Site Name | Organism | Position | Strand | Matrix score. | sequence | function |
| --- | --- | --- | --- | --- | --- | --- |
| MYC | Arabidopsis thaliana | 586 | + | 6 | CATGTG |  |
| MYC | Arabidopsis thaliana | 875 | + | 6 | CATGTG |  |
| MYC | Arabidopsis thaliana | 678 | - | 6 | CATGTG |  |
| MYC | Arabidopsis thaliana | 1327 | - | 6 | CAATTG |  |

>Potri.002G039100.1   
+ TTTTTTTTTA TCCGTAATTG ATTATTCAAA ACCTTTCCAG CTTAAAATTT TTTCCATAAT TTAAAAACTA   
  
  
+ TTAAAGGTTG AAAACACTTT TTTTTTCTCT GAGATTTAAA ACTAATACTA TAAAAACTCT AACTCTAACT   
  
  
+ TTCCGTTGTT TATTATTTAT CACCGAATCT TCTTGATAAA TGTATTTCTT AAAGTATATT TTATATAAAA   
  
  
+ ATATTTTTTT TATTTTTAAA ATTTTCCTTT AATATACATC ATGTAAAGTT TCAAATCGAT TTAAAAATTA   
  
  
+ AACATAATTT TTTTAAAAAA TTAACGGAAC CGCAAAAAAA TAAAGAGATG GTCGCCATCA ACCGTGTAGC   
  
  
+ AACCCTTTCT TTTCGGAGGA CTTTCAAAAT TCAGGCATTT CCATGGAGAT TTTTTGTCTT CTTGAAGACC   
  
  
+ GGGAACGGTT TCAAGCAATC CCACGAAAGG GGGCCTGAAT CTAGACAACG TTGCTATCAA ACAGCTTCAT   
  
  
+ CTGTGTTAAA TATTTTATGG ATTTGATTTA CCTGTCCCCT CTGCCAGTCT TTTCAAATCA AATTTGCCAC   
  
  
+ AAGAAACCAT GACTTTTATT TCTTTCATGT GCACATCATT ACCTTAAGTG ATAACGATTA AAGGTGGAGG   
  
  
+ TTTGCCTAAA ATTACCAGTT GATATTAAAT ACGCAACAAC TTAATTTCAC ATGAATCCTC ACCCGTATTA   
  
  
+ GCTAGGGTGC CAAACTCTCT TGATCACCAT CCGTGATTAA GCGGTGCGTG GTTTTATTGA TTAAAACTAA   
  
  
+ TGTGATTTAA ATCTGATTAT CCCCGAGTTA ATTATGAAGG CTCGGAACTC CATTTTCTTC TCCTTTTTTT   
  
  
+ CACTTTTTTC TTGGACAGGG GACGAAAGTT GGTTCATGTG GCATCAAAAA AGTAGTGTTT AATAATTTAG   
  
  
+ ATGCTTTTAA CATTCATTGG ACAAGACTAA AAAGATCAGA TTTCATAGCA AATCTATCAC TTAGTCGACA   
  
  
+ TAAATATGTA AAAAACTCGA GATTTTATTA ATGGGTCTTG AATTTAATTA AAACCTTTAA ATTAAATAAA   
  
  
+ TATTATTCTC GTCACGAGTC AATCTCTTGT AACATCGTCA TTTCTTTAAG ATCAGTGACC TTGTTGAAAA   
  
  
+ CTAAAAACAC ACACAATTTT AATCATTCTT CTCTTTTTCG TGTATTCAAC TCACTTAAAT TCAACATATA   
  
  
+ GATTGAAAGA TTACAAGTCT AGACCTATTA TAATTCAATA AAGATTCTAA TTAGCAAGTT GAATAAGTGT   
  
  
+ TCTAAACACC AATTACTTTT TTCATCGATG ATTTCTATTC AAACTACGAT GTAAACTAAT ATCTAACAAT   
  
  
+ TGTCTTGAAT TTCGAACAAA TAATCACAGC TGCAGCTTTC AAAACATTAC CCAGAACCAT TTTCCTTGAC   
  
  
+ TTCTTTTTTA TTTTCTTATG CTTGAAATAA TACAGAACAG ATTAACGAGT TCTGTACACT GAAAATGAAA   
  
  
+ CAAGACTTTG AACTTCTAAC TTGGTATATT CTATTTTGTT ATACCGTCCC ATCATGTTAA GACTGGAAAA   
  
  
+ CTAATTAAAA GGTATTTCAT GAGATTATGA TGCATAATTA TTTTTAAAAT AATTTTTTAT TTAAAAATAT   
  
  
+ ATTAAAATAA AATTTCTTAT TTATTTTTAA TATCAACAGA TTAAAACTAT CATAAATCAC TAAATAATTA   
  
  
+ TCAATTTAAT ATTTTTCAAA CCAAAAACAC ATTTAAAATA CACTTCAAAA CTGTGTCAAC TTGAAGCTCA   
  
  
+ AAATTCACAG AGAAGTTAAA AACATTTAAC TGTAAATGCG AAGCTACACT GTCCAAACCT TCCAGTGACA   
  
  
+ ATGAAAAGCT TGCCACTTTG TATTGAAATA TTGGTACCAC GTGTAAGACA TATGCGAACA AGGCAATGGT   
  
  
+ GACGTCGGCA AGGTATAGGC AATCGTGGGA CCAATCCCGC CGCCGTACGT CACTAAACAC ACGTGCCTTA   
  
  
+ AAAACAAATT CCCAAGCCTT CCTAGCAACA AAGAACAAG  

- AAAAAAAAAT AGGCATTAAC TAATAAGTTT TGGAAAGGTC GAATTTTAAA AAAGGTATTA AATTTTTGAT   
  
  
- AATTTCCAAC TTTTGTGAAA AAAAAAGAGA CTCTAAATTT TGATTATGAT ATTTTTGAGA TTGAGATTGA   
  
  
- AAGGCAACAA ATAATAAATA GTGGCTTAGA AGAACTATTT ACATAAAGAA TTTCATATAA AATATATTTT   
  
  
- TATAAAAAAA ATAAAAATTT TAAAAGGAAA TTATATGTAG TACATTTCAA AGTTTAGCTA AATTTTTAAT   
  
  
- TTGTATTAAA AAAATTTTTT AATTGCCTTG GCGTTTTTTT ATTTCTCTAC CAGCGGTAGT TGGCACATCG   
  
  
- TTGGGAAAGA AAAGCCTCCT GAAAGTTTTA AGTCCGTAAA GGTACCTCTA AAAAACAGAA GAACTTCTGG   
  
  
- CCCTTGCCAA AGTTCGTTAG GGTGCTTTCC CCCGGACTTA GATCTGTTGC AACGATAGTT TGTCGAAGTA   
  
  
- GACACAATTT ATAAAATACC TAAACTAAAT GGACAGGGGA GACGGTCAGA AAAGTTTAGT TTAAACGGTG   
  
  
- TTCTTTGGTA CTGAAAATAA AGAAAGTACA CGTGTAGTAA TGGAATTCAC TATTGCTAAT TTCCACCTCC   
  
  
- AAACGGATTT TAATGGTCAA CTATAATTTA TGCGTTGTTG AATTAAAGTG TACTTAGGAG TGGGCATAAT   
  
  
- CGATCCCACG GTTTGAGAGA ACTAGTGGTA GGCACTAATT CGCCACGCAC CAAAATAACT AATTTTGATT   
  
  
- ACACTAAATT TAGACTAATA GGGGCTCAAT TAATACTTCC GAGCCTTGAG GTAAAAGAAG AGGAAAAAAA   
  
  
- GTGAAAAAAG AACCTGTCCC CTGCTTTCAA CCAAGTACAC CGTAGTTTTT TCATCACAAA TTATTAAATC   
  
  
- TACGAAAATT GTAAGTAACC TGTTCTGATT TTTCTAGTCT AAAGTATCGT TTAGATAGTG AATCAGCTGT   
  
  
- ATTTATACAT TTTTTGAGCT CTAAAATAAT TACCCAGAAC TTAAATTAAT TTTGGAAATT TAATTTATTT   
  
  
- ATAATAAGAG CAGTGCTCAG TTAGAGAACA TTGTAGCAGT AAAGAAATTC TAGTCACTGG AACAACTTTT   
  
  
- GATTTTTGTG TGTGTTAAAA TTAGTAAGAA GAGAAAAAGC ACATAAGTTG AGTGAATTTA AGTTGTATAT   
  
  
- CTAACTTTCT AATGTTCAGA TCTGGATAAT ATTAAGTTAT TTCTAAGATT AATCGTTCAA CTTATTCACA   
  
  
- AGATTTGTGG TTAATGAAAA AAGTAGCTAC TAAAGATAAG TTTGATGCTA CATTTGATTA TAGATTGTTA   
  
  
- ACAGAACTTA AAGCTTGTTT ATTAGTGTCG ACGTCGAAAG TTTTGTAATG GGTCTTGGTA AAAGGAACTG   
  
  
- AAGAAAAAAT AAAAGAATAC GAACTTTATT ATGTCTTGTC TAATTGCTCA AGACATGTGA CTTTTACTTT   
  
  
- GTTCTGAAAC TTGAAGATTG AACCATATAA GATAAAACAA TATGGCAGGG TAGTACAATT CTGACCTTTT   
  
  
- GATTAATTTT CCATAAAGTA CTCTAATACT ACGTATTAAT AAAAATTTTA TTAAAAAATA AATTTTTATA   
  
  
- TAATTTTATT TTAAAGAATA AATAAAAATT ATAGTTGTCT AATTTTGATA GTATTTAGTG ATTTATTAAT   
  
  
- AGTTAAATTA TAAAAAGTTT GGTTTTTGTG TAAATTTTAT GTGAAGTTTT GACACAGTTG AACTTCGAGT   
  
  
- TTTAAGTGTC TCTTCAATTT TTGTAAATTG ACATTTACGC TTCGATGTGA CAGGTTTGGA AGGTCACTGT   
  
  
- TACTTTTCGA ACGGTGAAAC ATAACTTTAT AACCATGGTG CACATTCTGT ATACGCTTGT TCCGTTACCA   
  
  
- CTGCAGCCGT TCCATATCCG TTAGCACCCT GGTTAGGGCG GCGGCATGCA GTGATTTGTG TGCACGGAAT   
  
  
- TTTTGTTTAA GGGTTCGGAA GGATCGTTGT TTCTTGTTC

+     Myb

| Site Name | Organism | Position | Strand | Matrix score. | sequence | function |
| --- | --- | --- | --- | --- | --- | --- |
| Myb | Arabidopsis thaliana | 646 | - | 6 | CAACTG |  |
| Myb | Arabidopsis thaliana | 1777 | + | 6 | TAACTG |  |

>Potri.002G039100.1   
+ TTTTTTTTTA TCCGTAATTG ATTATTCAAA ACCTTTCCAG CTTAAAATTT TTTCCATAAT TTAAAAACTA   
  
  
+ TTAAAGGTTG AAAACACTTT TTTTTTCTCT GAGATTTAAA ACTAATACTA TAAAAACTCT AACTCTAACT   
  
  
+ TTCCGTTGTT TATTATTTAT CACCGAATCT TCTTGATAAA TGTATTTCTT AAAGTATATT TTATATAAAA   
  
  
+ ATATTTTTTT TATTTTTAAA ATTTTCCTTT AATATACATC ATGTAAAGTT TCAAATCGAT TTAAAAATTA   
  
  
+ AACATAATTT TTTTAAAAAA TTAACGGAAC CGCAAAAAAA TAAAGAGATG GTCGCCATCA ACCGTGTAGC   
  
  
+ AACCCTTTCT TTTCGGAGGA CTTTCAAAAT TCAGGCATTT CCATGGAGAT TTTTTGTCTT CTTGAAGACC   
  
  
+ GGGAACGGTT TCAAGCAATC CCACGAAAGG GGGCCTGAAT CTAGACAACG TTGCTATCAA ACAGCTTCAT   
  
  
+ CTGTGTTAAA TATTTTATGG ATTTGATTTA CCTGTCCCCT CTGCCAGTCT TTTCAAATCA AATTTGCCAC   
  
  
+ AAGAAACCAT GACTTTTATT TCTTTCATGT GCACATCATT ACCTTAAGTG ATAACGATTA AAGGTGGAGG   
  
  
+ TTTGCCTAAA ATTACCAGTT GATATTAAAT ACGCAACAAC TTAATTTCAC ATGAATCCTC ACCCGTATTA   
  
  
+ GCTAGGGTGC CAAACTCTCT TGATCACCAT CCGTGATTAA GCGGTGCGTG GTTTTATTGA TTAAAACTAA   
  
  
+ TGTGATTTAA ATCTGATTAT CCCCGAGTTA ATTATGAAGG CTCGGAACTC CATTTTCTTC TCCTTTTTTT   
  
  
+ CACTTTTTTC TTGGACAGGG GACGAAAGTT GGTTCATGTG GCATCAAAAA AGTAGTGTTT AATAATTTAG   
  
  
+ ATGCTTTTAA CATTCATTGG ACAAGACTAA AAAGATCAGA TTTCATAGCA AATCTATCAC TTAGTCGACA   
  
  
+ TAAATATGTA AAAAACTCGA GATTTTATTA ATGGGTCTTG AATTTAATTA AAACCTTTAA ATTAAATAAA   
  
  
+ TATTATTCTC GTCACGAGTC AATCTCTTGT AACATCGTCA TTTCTTTAAG ATCAGTGACC TTGTTGAAAA   
  
  
+ CTAAAAACAC ACACAATTTT AATCATTCTT CTCTTTTTCG TGTATTCAAC TCACTTAAAT TCAACATATA   
  
  
+ GATTGAAAGA TTACAAGTCT AGACCTATTA TAATTCAATA AAGATTCTAA TTAGCAAGTT GAATAAGTGT   
  
  
+ TCTAAACACC AATTACTTTT TTCATCGATG ATTTCTATTC AAACTACGAT GTAAACTAAT ATCTAACAAT   
  
  
+ TGTCTTGAAT TTCGAACAAA TAATCACAGC TGCAGCTTTC AAAACATTAC CCAGAACCAT TTTCCTTGAC   
  
  
+ TTCTTTTTTA TTTTCTTATG CTTGAAATAA TACAGAACAG ATTAACGAGT TCTGTACACT GAAAATGAAA   
  
  
+ CAAGACTTTG AACTTCTAAC TTGGTATATT CTATTTTGTT ATACCGTCCC ATCATGTTAA GACTGGAAAA   
  
  
+ CTAATTAAAA GGTATTTCAT GAGATTATGA TGCATAATTA TTTTTAAAAT AATTTTTTAT TTAAAAATAT   
  
  
+ ATTAAAATAA AATTTCTTAT TTATTTTTAA TATCAACAGA TTAAAACTAT CATAAATCAC TAAATAATTA   
  
  
+ TCAATTTAAT ATTTTTCAAA CCAAAAACAC ATTTAAAATA CACTTCAAAA CTGTGTCAAC TTGAAGCTCA   
  
  
+ AAATTCACAG AGAAGTTAAA AACATTTAAC TGTAAATGCG AAGCTACACT GTCCAAACCT TCCAGTGACA   
  
  
+ ATGAAAAGCT TGCCACTTTG TATTGAAATA TTGGTACCAC GTGTAAGACA TATGCGAACA AGGCAATGGT   
  
  
+ GACGTCGGCA AGGTATAGGC AATCGTGGGA CCAATCCCGC CGCCGTACGT CACTAAACAC ACGTGCCTTA   
  
  
+ AAAACAAATT CCCAAGCCTT CCTAGCAACA AAGAACAAG  

- AAAAAAAAAT AGGCATTAAC TAATAAGTTT TGGAAAGGTC GAATTTTAAA AAAGGTATTA AATTTTTGAT   
  
  
- AATTTCCAAC TTTTGTGAAA AAAAAAGAGA CTCTAAATTT TGATTATGAT ATTTTTGAGA TTGAGATTGA   
  
  
- AAGGCAACAA ATAATAAATA GTGGCTTAGA AGAACTATTT ACATAAAGAA TTTCATATAA AATATATTTT   
  
  
- TATAAAAAAA ATAAAAATTT TAAAAGGAAA TTATATGTAG TACATTTCAA AGTTTAGCTA AATTTTTAAT   
  
  
- TTGTATTAAA AAAATTTTTT AATTGCCTTG GCGTTTTTTT ATTTCTCTAC CAGCGGTAGT TGGCACATCG   
  
  
- TTGGGAAAGA AAAGCCTCCT GAAAGTTTTA AGTCCGTAAA GGTACCTCTA AAAAACAGAA GAACTTCTGG   
  
  
- CCCTTGCCAA AGTTCGTTAG GGTGCTTTCC CCCGGACTTA GATCTGTTGC AACGATAGTT TGTCGAAGTA   
  
  
- GACACAATTT ATAAAATACC TAAACTAAAT GGACAGGGGA GACGGTCAGA AAAGTTTAGT TTAAACGGTG   
  
  
- TTCTTTGGTA CTGAAAATAA AGAAAGTACA CGTGTAGTAA TGGAATTCAC TATTGCTAAT TTCCACCTCC   
  
  
- AAACGGATTT TAATGGTCAA CTATAATTTA TGCGTTGTTG AATTAAAGTG TACTTAGGAG TGGGCATAAT   
  
  
- CGATCCCACG GTTTGAGAGA ACTAGTGGTA GGCACTAATT CGCCACGCAC CAAAATAACT AATTTTGATT   
  
  
- ACACTAAATT TAGACTAATA GGGGCTCAAT TAATACTTCC GAGCCTTGAG GTAAAAGAAG AGGAAAAAAA   
  
  
- GTGAAAAAAG AACCTGTCCC CTGCTTTCAA CCAAGTACAC CGTAGTTTTT TCATCACAAA TTATTAAATC   
  
  
- TACGAAAATT GTAAGTAACC TGTTCTGATT TTTCTAGTCT AAAGTATCGT TTAGATAGTG AATCAGCTGT   
  
  
- ATTTATACAT TTTTTGAGCT CTAAAATAAT TACCCAGAAC TTAAATTAAT TTTGGAAATT TAATTTATTT   
  
  
- ATAATAAGAG CAGTGCTCAG TTAGAGAACA TTGTAGCAGT AAAGAAATTC TAGTCACTGG AACAACTTTT   
  
  
- GATTTTTGTG TGTGTTAAAA TTAGTAAGAA GAGAAAAAGC ACATAAGTTG AGTGAATTTA AGTTGTATAT   
  
  
- CTAACTTTCT AATGTTCAGA TCTGGATAAT ATTAAGTTAT TTCTAAGATT AATCGTTCAA CTTATTCACA   
  
  
- AGATTTGTGG TTAATGAAAA AAGTAGCTAC TAAAGATAAG TTTGATGCTA CATTTGATTA TAGATTGTTA   
  
  
- ACAGAACTTA AAGCTTGTTT ATTAGTGTCG ACGTCGAAAG TTTTGTAATG GGTCTTGGTA AAAGGAACTG   
  
  
- AAGAAAAAAT AAAAGAATAC GAACTTTATT ATGTCTTGTC TAATTGCTCA AGACATGTGA CTTTTACTTT   
  
  
- GTTCTGAAAC TTGAAGATTG AACCATATAA GATAAAACAA TATGGCAGGG TAGTACAATT CTGACCTTTT   
  
  
- GATTAATTTT CCATAAAGTA CTCTAATACT ACGTATTAAT AAAAATTTTA TTAAAAAATA AATTTTTATA   
  
  
- TAATTTTATT TTAAAGAATA AATAAAAATT ATAGTTGTCT AATTTTGATA GTATTTAGTG ATTTATTAAT   
  
  
- AGTTAAATTA TAAAAAGTTT GGTTTTTGTG TAAATTTTAT GTGAAGTTTT GACACAGTTG AACTTCGAGT   
  
  
- TTTAAGTGTC TCTTCAATTT TTGTAAATTG ACATTTACGC TTCGATGTGA CAGGTTTGGA AGGTCACTGT   
  
  
- TACTTTTCGA ACGGTGAAAC ATAACTTTAT AACCATGGTG CACATTCTGT ATACGCTTGT TCCGTTACCA   
  
  
- CTGCAGCCGT TCCATATCCG TTAGCACCCT GGTTAGGGCG GCGGCATGCA GTGATTTGTG TGCACGGAAT   
  
  
- TTTTGTTTAA GGGTTCGGAA GGATCGTTGT TTCTTGTTC

+     Myb-binding site

| Site Name | Organism | Position | Strand | Matrix score. | sequence | function |
| --- | --- | --- | --- | --- | --- | --- |
| Myb-binding site | Nicotiana tabacum | 1644 | + | 6 | CAACAG |  |

>Potri.002G039100.1   
+ TTTTTTTTTA TCCGTAATTG ATTATTCAAA ACCTTTCCAG CTTAAAATTT TTTCCATAAT TTAAAAACTA   
  
  
+ TTAAAGGTTG AAAACACTTT TTTTTTCTCT GAGATTTAAA ACTAATACTA TAAAAACTCT AACTCTAACT   
  
  
+ TTCCGTTGTT TATTATTTAT CACCGAATCT TCTTGATAAA TGTATTTCTT AAAGTATATT TTATATAAAA   
  
  
+ ATATTTTTTT TATTTTTAAA ATTTTCCTTT AATATACATC ATGTAAAGTT TCAAATCGAT TTAAAAATTA   
  
  
+ AACATAATTT TTTTAAAAAA TTAACGGAAC CGCAAAAAAA TAAAGAGATG GTCGCCATCA ACCGTGTAGC   
  
  
+ AACCCTTTCT TTTCGGAGGA CTTTCAAAAT TCAGGCATTT CCATGGAGAT TTTTTGTCTT CTTGAAGACC   
  
  
+ GGGAACGGTT TCAAGCAATC CCACGAAAGG GGGCCTGAAT CTAGACAACG TTGCTATCAA ACAGCTTCAT   
  
  
+ CTGTGTTAAA TATTTTATGG ATTTGATTTA CCTGTCCCCT CTGCCAGTCT TTTCAAATCA AATTTGCCAC   
  
  
+ AAGAAACCAT GACTTTTATT TCTTTCATGT GCACATCATT ACCTTAAGTG ATAACGATTA AAGGTGGAGG   
  
  
+ TTTGCCTAAA ATTACCAGTT GATATTAAAT ACGCAACAAC TTAATTTCAC ATGAATCCTC ACCCGTATTA   
  
  
+ GCTAGGGTGC CAAACTCTCT TGATCACCAT CCGTGATTAA GCGGTGCGTG GTTTTATTGA TTAAAACTAA   
  
  
+ TGTGATTTAA ATCTGATTAT CCCCGAGTTA ATTATGAAGG CTCGGAACTC CATTTTCTTC TCCTTTTTTT   
  
  
+ CACTTTTTTC TTGGACAGGG GACGAAAGTT GGTTCATGTG GCATCAAAAA AGTAGTGTTT AATAATTTAG   
  
  
+ ATGCTTTTAA CATTCATTGG ACAAGACTAA AAAGATCAGA TTTCATAGCA AATCTATCAC TTAGTCGACA   
  
  
+ TAAATATGTA AAAAACTCGA GATTTTATTA ATGGGTCTTG AATTTAATTA AAACCTTTAA ATTAAATAAA   
  
  
+ TATTATTCTC GTCACGAGTC AATCTCTTGT AACATCGTCA TTTCTTTAAG ATCAGTGACC TTGTTGAAAA   
  
  
+ CTAAAAACAC ACACAATTTT AATCATTCTT CTCTTTTTCG TGTATTCAAC TCACTTAAAT TCAACATATA   
  
  
+ GATTGAAAGA TTACAAGTCT AGACCTATTA TAATTCAATA AAGATTCTAA TTAGCAAGTT GAATAAGTGT   
  
  
+ TCTAAACACC AATTACTTTT TTCATCGATG ATTTCTATTC AAACTACGAT GTAAACTAAT ATCTAACAAT   
  
  
+ TGTCTTGAAT TTCGAACAAA TAATCACAGC TGCAGCTTTC AAAACATTAC CCAGAACCAT TTTCCTTGAC   
  
  
+ TTCTTTTTTA TTTTCTTATG CTTGAAATAA TACAGAACAG ATTAACGAGT TCTGTACACT GAAAATGAAA   
  
  
+ CAAGACTTTG AACTTCTAAC TTGGTATATT CTATTTTGTT ATACCGTCCC ATCATGTTAA GACTGGAAAA   
  
  
+ CTAATTAAAA GGTATTTCAT GAGATTATGA TGCATAATTA TTTTTAAAAT AATTTTTTAT TTAAAAATAT   
  
  
+ ATTAAAATAA AATTTCTTAT TTATTTTTAA TATCAACAGA TTAAAACTAT CATAAATCAC TAAATAATTA   
  
  
+ TCAATTTAAT ATTTTTCAAA CCAAAAACAC ATTTAAAATA CACTTCAAAA CTGTGTCAAC TTGAAGCTCA   
  
  
+ AAATTCACAG AGAAGTTAAA AACATTTAAC TGTAAATGCG AAGCTACACT GTCCAAACCT TCCAGTGACA   
  
  
+ ATGAAAAGCT TGCCACTTTG TATTGAAATA TTGGTACCAC GTGTAAGACA TATGCGAACA AGGCAATGGT   
  
  
+ GACGTCGGCA AGGTATAGGC AATCGTGGGA CCAATCCCGC CGCCGTACGT CACTAAACAC ACGTGCCTTA   
  
  
+ AAAACAAATT CCCAAGCCTT CCTAGCAACA AAGAACAAG  

- AAAAAAAAAT AGGCATTAAC TAATAAGTTT TGGAAAGGTC GAATTTTAAA AAAGGTATTA AATTTTTGAT   
  
  
- AATTTCCAAC TTTTGTGAAA AAAAAAGAGA CTCTAAATTT TGATTATGAT ATTTTTGAGA TTGAGATTGA   
  
  
- AAGGCAACAA ATAATAAATA GTGGCTTAGA AGAACTATTT ACATAAAGAA TTTCATATAA AATATATTTT   
  
  
- TATAAAAAAA ATAAAAATTT TAAAAGGAAA TTATATGTAG TACATTTCAA AGTTTAGCTA AATTTTTAAT   
  
  
- TTGTATTAAA AAAATTTTTT AATTGCCTTG GCGTTTTTTT ATTTCTCTAC CAGCGGTAGT TGGCACATCG   
  
  
- TTGGGAAAGA AAAGCCTCCT GAAAGTTTTA AGTCCGTAAA GGTACCTCTA AAAAACAGAA GAACTTCTGG   
  
  
- CCCTTGCCAA AGTTCGTTAG GGTGCTTTCC CCCGGACTTA GATCTGTTGC AACGATAGTT TGTCGAAGTA   
  
  
- GACACAATTT ATAAAATACC TAAACTAAAT GGACAGGGGA GACGGTCAGA AAAGTTTAGT TTAAACGGTG   
  
  
- TTCTTTGGTA CTGAAAATAA AGAAAGTACA CGTGTAGTAA TGGAATTCAC TATTGCTAAT TTCCACCTCC   
  
  
- AAACGGATTT TAATGGTCAA CTATAATTTA TGCGTTGTTG AATTAAAGTG TACTTAGGAG TGGGCATAAT   
  
  
- CGATCCCACG GTTTGAGAGA ACTAGTGGTA GGCACTAATT CGCCACGCAC CAAAATAACT AATTTTGATT   
  
  
- ACACTAAATT TAGACTAATA GGGGCTCAAT TAATACTTCC GAGCCTTGAG GTAAAAGAAG AGGAAAAAAA   
  
  
- GTGAAAAAAG AACCTGTCCC CTGCTTTCAA CCAAGTACAC CGTAGTTTTT TCATCACAAA TTATTAAATC   
  
  
- TACGAAAATT GTAAGTAACC TGTTCTGATT TTTCTAGTCT AAAGTATCGT TTAGATAGTG AATCAGCTGT   
  
  
- ATTTATACAT TTTTTGAGCT CTAAAATAAT TACCCAGAAC TTAAATTAAT TTTGGAAATT TAATTTATTT   
  
  
- ATAATAAGAG CAGTGCTCAG TTAGAGAACA TTGTAGCAGT AAAGAAATTC TAGTCACTGG AACAACTTTT   
  
  
- GATTTTTGTG TGTGTTAAAA TTAGTAAGAA GAGAAAAAGC ACATAAGTTG AGTGAATTTA AGTTGTATAT   
  
  
- CTAACTTTCT AATGTTCAGA TCTGGATAAT ATTAAGTTAT TTCTAAGATT AATCGTTCAA CTTATTCACA   
  
  
- AGATTTGTGG TTAATGAAAA AAGTAGCTAC TAAAGATAAG TTTGATGCTA CATTTGATTA TAGATTGTTA   
  
  
- ACAGAACTTA AAGCTTGTTT ATTAGTGTCG ACGTCGAAAG TTTTGTAATG GGTCTTGGTA AAAGGAACTG   
  
  
- AAGAAAAAAT AAAAGAATAC GAACTTTATT ATGTCTTGTC TAATTGCTCA AGACATGTGA CTTTTACTTT   
  
  
- GTTCTGAAAC TTGAAGATTG AACCATATAA GATAAAACAA TATGGCAGGG TAGTACAATT CTGACCTTTT   
  
  
- GATTAATTTT CCATAAAGTA CTCTAATACT ACGTATTAAT AAAAATTTTA TTAAAAAATA AATTTTTATA   
  
  
- TAATTTTATT TTAAAGAATA AATAAAAATT ATAGTTGTCT AATTTTGATA GTATTTAGTG ATTTATTAAT   
  
  
- AGTTAAATTA TAAAAAGTTT GGTTTTTGTG TAAATTTTAT GTGAAGTTTT GACACAGTTG AACTTCGAGT   
  
  
- TTTAAGTGTC TCTTCAATTT TTGTAAATTG ACATTTACGC TTCGATGTGA CAGGTTTGGA AGGTCACTGT   
  
  
- TACTTTTCGA ACGGTGAAAC ATAACTTTAT AACCATGGTG CACATTCTGT ATACGCTTGT TCCGTTACCA   
  
  
- CTGCAGCCGT TCCATATCCG TTAGCACCCT GGTTAGGGCG GCGGCATGCA GTGATTTGTG TGCACGGAAT   
  
  
- TTTTGTTTAA GGGTTCGGAA GGATCGTTGT TTCTTGTTC

+     STRE

| Site Name | Organism | Position | Strand | Matrix score. | sequence | function |
| --- | --- | --- | --- | --- | --- | --- |
| STRE | Arabidopsis thaliana | 448 | + | 5 | AGGGG |  |
| STRE | Arabidopsis thaliana | 857 | + | 5 | AGGGG |  |
| STRE | Arabidopsis thaliana | 526 | - | 5 | AGGGG |  |

>Potri.002G039100.1   
+ TTTTTTTTTA TCCGTAATTG ATTATTCAAA ACCTTTCCAG CTTAAAATTT TTTCCATAAT TTAAAAACTA   
  
  
+ TTAAAGGTTG AAAACACTTT TTTTTTCTCT GAGATTTAAA ACTAATACTA TAAAAACTCT AACTCTAACT   
  
  
+ TTCCGTTGTT TATTATTTAT CACCGAATCT TCTTGATAAA TGTATTTCTT AAAGTATATT TTATATAAAA   
  
  
+ ATATTTTTTT TATTTTTAAA ATTTTCCTTT AATATACATC ATGTAAAGTT TCAAATCGAT TTAAAAATTA   
  
  
+ AACATAATTT TTTTAAAAAA TTAACGGAAC CGCAAAAAAA TAAAGAGATG GTCGCCATCA ACCGTGTAGC   
  
  
+ AACCCTTTCT TTTCGGAGGA CTTTCAAAAT TCAGGCATTT CCATGGAGAT TTTTTGTCTT CTTGAAGACC   
  
  
+ GGGAACGGTT TCAAGCAATC CCACGAAAGG GGGCCTGAAT CTAGACAACG TTGCTATCAA ACAGCTTCAT   
  
  
+ CTGTGTTAAA TATTTTATGG ATTTGATTTA CCTGTCCCCT CTGCCAGTCT TTTCAAATCA AATTTGCCAC   
  
  
+ AAGAAACCAT GACTTTTATT TCTTTCATGT GCACATCATT ACCTTAAGTG ATAACGATTA AAGGTGGAGG   
  
  
+ TTTGCCTAAA ATTACCAGTT GATATTAAAT ACGCAACAAC TTAATTTCAC ATGAATCCTC ACCCGTATTA   
  
  
+ GCTAGGGTGC CAAACTCTCT TGATCACCAT CCGTGATTAA GCGGTGCGTG GTTTTATTGA TTAAAACTAA   
  
  
+ TGTGATTTAA ATCTGATTAT CCCCGAGTTA ATTATGAAGG CTCGGAACTC CATTTTCTTC TCCTTTTTTT   
  
  
+ CACTTTTTTC TTGGACAGGG GACGAAAGTT GGTTCATGTG GCATCAAAAA AGTAGTGTTT AATAATTTAG   
  
  
+ ATGCTTTTAA CATTCATTGG ACAAGACTAA AAAGATCAGA TTTCATAGCA AATCTATCAC TTAGTCGACA   
  
  
+ TAAATATGTA AAAAACTCGA GATTTTATTA ATGGGTCTTG AATTTAATTA AAACCTTTAA ATTAAATAAA   
  
  
+ TATTATTCTC GTCACGAGTC AATCTCTTGT AACATCGTCA TTTCTTTAAG ATCAGTGACC TTGTTGAAAA   
  
  
+ CTAAAAACAC ACACAATTTT AATCATTCTT CTCTTTTTCG TGTATTCAAC TCACTTAAAT TCAACATATA   
  
  
+ GATTGAAAGA TTACAAGTCT AGACCTATTA TAATTCAATA AAGATTCTAA TTAGCAAGTT GAATAAGTGT   
  
  
+ TCTAAACACC AATTACTTTT TTCATCGATG ATTTCTATTC AAACTACGAT GTAAACTAAT ATCTAACAAT   
  
  
+ TGTCTTGAAT TTCGAACAAA TAATCACAGC TGCAGCTTTC AAAACATTAC CCAGAACCAT TTTCCTTGAC   
  
  
+ TTCTTTTTTA TTTTCTTATG CTTGAAATAA TACAGAACAG ATTAACGAGT TCTGTACACT GAAAATGAAA   
  
  
+ CAAGACTTTG AACTTCTAAC TTGGTATATT CTATTTTGTT ATACCGTCCC ATCATGTTAA GACTGGAAAA   
  
  
+ CTAATTAAAA GGTATTTCAT GAGATTATGA TGCATAATTA TTTTTAAAAT AATTTTTTAT TTAAAAATAT   
  
  
+ ATTAAAATAA AATTTCTTAT TTATTTTTAA TATCAACAGA TTAAAACTAT CATAAATCAC TAAATAATTA   
  
  
+ TCAATTTAAT ATTTTTCAAA CCAAAAACAC ATTTAAAATA CACTTCAAAA CTGTGTCAAC TTGAAGCTCA   
  
  
+ AAATTCACAG AGAAGTTAAA AACATTTAAC TGTAAATGCG AAGCTACACT GTCCAAACCT TCCAGTGACA   
  
  
+ ATGAAAAGCT TGCCACTTTG TATTGAAATA TTGGTACCAC GTGTAAGACA TATGCGAACA AGGCAATGGT   
  
  
+ GACGTCGGCA AGGTATAGGC AATCGTGGGA CCAATCCCGC CGCCGTACGT CACTAAACAC ACGTGCCTTA   
  
  
+ AAAACAAATT CCCAAGCCTT CCTAGCAACA AAGAACAAG  

- AAAAAAAAAT AGGCATTAAC TAATAAGTTT TGGAAAGGTC GAATTTTAAA AAAGGTATTA AATTTTTGAT   
  
  
- AATTTCCAAC TTTTGTGAAA AAAAAAGAGA CTCTAAATTT TGATTATGAT ATTTTTGAGA TTGAGATTGA   
  
  
- AAGGCAACAA ATAATAAATA GTGGCTTAGA AGAACTATTT ACATAAAGAA TTTCATATAA AATATATTTT   
  
  
- TATAAAAAAA ATAAAAATTT TAAAAGGAAA TTATATGTAG TACATTTCAA AGTTTAGCTA AATTTTTAAT   
  
  
- TTGTATTAAA AAAATTTTTT AATTGCCTTG GCGTTTTTTT ATTTCTCTAC CAGCGGTAGT TGGCACATCG   
  
  
- TTGGGAAAGA AAAGCCTCCT GAAAGTTTTA AGTCCGTAAA GGTACCTCTA AAAAACAGAA GAACTTCTGG   
  
  
- CCCTTGCCAA AGTTCGTTAG GGTGCTTTCC CCCGGACTTA GATCTGTTGC AACGATAGTT TGTCGAAGTA   
  
  
- GACACAATTT ATAAAATACC TAAACTAAAT GGACAGGGGA GACGGTCAGA AAAGTTTAGT TTAAACGGTG   
  
  
- TTCTTTGGTA CTGAAAATAA AGAAAGTACA CGTGTAGTAA TGGAATTCAC TATTGCTAAT TTCCACCTCC   
  
  
- AAACGGATTT TAATGGTCAA CTATAATTTA TGCGTTGTTG AATTAAAGTG TACTTAGGAG TGGGCATAAT   
  
  
- CGATCCCACG GTTTGAGAGA ACTAGTGGTA GGCACTAATT CGCCACGCAC CAAAATAACT AATTTTGATT   
  
  
- ACACTAAATT TAGACTAATA GGGGCTCAAT TAATACTTCC GAGCCTTGAG GTAAAAGAAG AGGAAAAAAA   
  
  
- GTGAAAAAAG AACCTGTCCC CTGCTTTCAA CCAAGTACAC CGTAGTTTTT TCATCACAAA TTATTAAATC   
  
  
- TACGAAAATT GTAAGTAACC TGTTCTGATT TTTCTAGTCT AAAGTATCGT TTAGATAGTG AATCAGCTGT   
  
  
- ATTTATACAT TTTTTGAGCT CTAAAATAAT TACCCAGAAC TTAAATTAAT TTTGGAAATT TAATTTATTT   
  
  
- ATAATAAGAG CAGTGCTCAG TTAGAGAACA TTGTAGCAGT AAAGAAATTC TAGTCACTGG AACAACTTTT   
  
  
- GATTTTTGTG TGTGTTAAAA TTAGTAAGAA GAGAAAAAGC ACATAAGTTG AGTGAATTTA AGTTGTATAT   
  
  
- CTAACTTTCT AATGTTCAGA TCTGGATAAT ATTAAGTTAT TTCTAAGATT AATCGTTCAA CTTATTCACA   
  
  
- AGATTTGTGG TTAATGAAAA AAGTAGCTAC TAAAGATAAG TTTGATGCTA CATTTGATTA TAGATTGTTA   
  
  
- ACAGAACTTA AAGCTTGTTT ATTAGTGTCG ACGTCGAAAG TTTTGTAATG GGTCTTGGTA AAAGGAACTG   
  
  
- AAGAAAAAAT AAAAGAATAC GAACTTTATT ATGTCTTGTC TAATTGCTCA AGACATGTGA CTTTTACTTT   
  
  
- GTTCTGAAAC TTGAAGATTG AACCATATAA GATAAAACAA TATGGCAGGG TAGTACAATT CTGACCTTTT   
  
  
- GATTAATTTT CCATAAAGTA CTCTAATACT ACGTATTAAT AAAAATTTTA TTAAAAAATA AATTTTTATA   
  
  
- TAATTTTATT TTAAAGAATA AATAAAAATT ATAGTTGTCT AATTTTGATA GTATTTAGTG ATTTATTAAT   
  
  
- AGTTAAATTA TAAAAAGTTT GGTTTTTGTG TAAATTTTAT GTGAAGTTTT GACACAGTTG AACTTCGAGT   
  
  
- TTTAAGTGTC TCTTCAATTT TTGTAAATTG ACATTTACGC TTCGATGTGA CAGGTTTGGA AGGTCACTGT   
  
  
- TACTTTTCGA ACGGTGAAAC ATAACTTTAT AACCATGGTG CACATTCTGT ATACGCTTGT TCCGTTACCA   
  
  
- CTGCAGCCGT TCCATATCCG TTAGCACCCT GGTTAGGGCG GCGGCATGCA GTGATTTGTG TGCACGGAAT   
  
  
- TTTTGTTTAA GGGTTCGGAA GGATCGTTGT TTCTTGTTC

+     TATA

| Site Name | Organism | Position | Strand | Matrix score. | sequence | function |
| --- | --- | --- | --- | --- | --- | --- |
| TATA | Arabidopsis thaliana | 198 | - | 8 | TATAAAAT |  |

>Potri.002G039100.1   
+ TTTTTTTTTA TCCGTAATTG ATTATTCAAA ACCTTTCCAG CTTAAAATTT TTTCCATAAT TTAAAAACTA   
  
  
+ TTAAAGGTTG AAAACACTTT TTTTTTCTCT GAGATTTAAA ACTAATACTA TAAAAACTCT AACTCTAACT   
  
  
+ TTCCGTTGTT TATTATTTAT CACCGAATCT TCTTGATAAA TGTATTTCTT AAAGTATATT TTATATAAAA   
  
  
+ ATATTTTTTT TATTTTTAAA ATTTTCCTTT AATATACATC ATGTAAAGTT TCAAATCGAT TTAAAAATTA   
  
  
+ AACATAATTT TTTTAAAAAA TTAACGGAAC CGCAAAAAAA TAAAGAGATG GTCGCCATCA ACCGTGTAGC   
  
  
+ AACCCTTTCT TTTCGGAGGA CTTTCAAAAT TCAGGCATTT CCATGGAGAT TTTTTGTCTT CTTGAAGACC   
  
  
+ GGGAACGGTT TCAAGCAATC CCACGAAAGG GGGCCTGAAT CTAGACAACG TTGCTATCAA ACAGCTTCAT   
  
  
+ CTGTGTTAAA TATTTTATGG ATTTGATTTA CCTGTCCCCT CTGCCAGTCT TTTCAAATCA AATTTGCCAC   
  
  
+ AAGAAACCAT GACTTTTATT TCTTTCATGT GCACATCATT ACCTTAAGTG ATAACGATTA AAGGTGGAGG   
  
  
+ TTTGCCTAAA ATTACCAGTT GATATTAAAT ACGCAACAAC TTAATTTCAC ATGAATCCTC ACCCGTATTA   
  
  
+ GCTAGGGTGC CAAACTCTCT TGATCACCAT CCGTGATTAA GCGGTGCGTG GTTTTATTGA TTAAAACTAA   
  
  
+ TGTGATTTAA ATCTGATTAT CCCCGAGTTA ATTATGAAGG CTCGGAACTC CATTTTCTTC TCCTTTTTTT   
  
  
+ CACTTTTTTC TTGGACAGGG GACGAAAGTT GGTTCATGTG GCATCAAAAA AGTAGTGTTT AATAATTTAG   
  
  
+ ATGCTTTTAA CATTCATTGG ACAAGACTAA AAAGATCAGA TTTCATAGCA AATCTATCAC TTAGTCGACA   
  
  
+ TAAATATGTA AAAAACTCGA GATTTTATTA ATGGGTCTTG AATTTAATTA AAACCTTTAA ATTAAATAAA   
  
  
+ TATTATTCTC GTCACGAGTC AATCTCTTGT AACATCGTCA TTTCTTTAAG ATCAGTGACC TTGTTGAAAA   
  
  
+ CTAAAAACAC ACACAATTTT AATCATTCTT CTCTTTTTCG TGTATTCAAC TCACTTAAAT TCAACATATA   
  
  
+ GATTGAAAGA TTACAAGTCT AGACCTATTA TAATTCAATA AAGATTCTAA TTAGCAAGTT GAATAAGTGT   
  
  
+ TCTAAACACC AATTACTTTT TTCATCGATG ATTTCTATTC AAACTACGAT GTAAACTAAT ATCTAACAAT   
  
  
+ TGTCTTGAAT TTCGAACAAA TAATCACAGC TGCAGCTTTC AAAACATTAC CCAGAACCAT TTTCCTTGAC   
  
  
+ TTCTTTTTTA TTTTCTTATG CTTGAAATAA TACAGAACAG ATTAACGAGT TCTGTACACT GAAAATGAAA   
  
  
+ CAAGACTTTG AACTTCTAAC TTGGTATATT CTATTTTGTT ATACCGTCCC ATCATGTTAA GACTGGAAAA   
  
  
+ CTAATTAAAA GGTATTTCAT GAGATTATGA TGCATAATTA TTTTTAAAAT AATTTTTTAT TTAAAAATAT   
  
  
+ ATTAAAATAA AATTTCTTAT TTATTTTTAA TATCAACAGA TTAAAACTAT CATAAATCAC TAAATAATTA   
  
  
+ TCAATTTAAT ATTTTTCAAA CCAAAAACAC ATTTAAAATA CACTTCAAAA CTGTGTCAAC TTGAAGCTCA   
  
  
+ AAATTCACAG AGAAGTTAAA AACATTTAAC TGTAAATGCG AAGCTACACT GTCCAAACCT TCCAGTGACA   
  
  
+ ATGAAAAGCT TGCCACTTTG TATTGAAATA TTGGTACCAC GTGTAAGACA TATGCGAACA AGGCAATGGT   
  
  
+ GACGTCGGCA AGGTATAGGC AATCGTGGGA CCAATCCCGC CGCCGTACGT CACTAAACAC ACGTGCCTTA   
  
  
+ AAAACAAATT CCCAAGCCTT CCTAGCAACA AAGAACAAG  

- AAAAAAAAAT AGGCATTAAC TAATAAGTTT TGGAAAGGTC GAATTTTAAA AAAGGTATTA AATTTTTGAT   
  
  
- AATTTCCAAC TTTTGTGAAA AAAAAAGAGA CTCTAAATTT TGATTATGAT ATTTTTGAGA TTGAGATTGA   
  
  
- AAGGCAACAA ATAATAAATA GTGGCTTAGA AGAACTATTT ACATAAAGAA TTTCATATAA AATATATTTT   
  
  
- TATAAAAAAA ATAAAAATTT TAAAAGGAAA TTATATGTAG TACATTTCAA AGTTTAGCTA AATTTTTAAT   
  
  
- TTGTATTAAA AAAATTTTTT AATTGCCTTG GCGTTTTTTT ATTTCTCTAC CAGCGGTAGT TGGCACATCG   
  
  
- TTGGGAAAGA AAAGCCTCCT GAAAGTTTTA AGTCCGTAAA GGTACCTCTA AAAAACAGAA GAACTTCTGG   
  
  
- CCCTTGCCAA AGTTCGTTAG GGTGCTTTCC CCCGGACTTA GATCTGTTGC AACGATAGTT TGTCGAAGTA   
  
  
- GACACAATTT ATAAAATACC TAAACTAAAT GGACAGGGGA GACGGTCAGA AAAGTTTAGT TTAAACGGTG   
  
  
- TTCTTTGGTA CTGAAAATAA AGAAAGTACA CGTGTAGTAA TGGAATTCAC TATTGCTAAT TTCCACCTCC   
  
  
- AAACGGATTT TAATGGTCAA CTATAATTTA TGCGTTGTTG AATTAAAGTG TACTTAGGAG TGGGCATAAT   
  
  
- CGATCCCACG GTTTGAGAGA ACTAGTGGTA GGCACTAATT CGCCACGCAC CAAAATAACT AATTTTGATT   
  
  
- ACACTAAATT TAGACTAATA GGGGCTCAAT TAATACTTCC GAGCCTTGAG GTAAAAGAAG AGGAAAAAAA   
  
  
- GTGAAAAAAG AACCTGTCCC CTGCTTTCAA CCAAGTACAC CGTAGTTTTT TCATCACAAA TTATTAAATC   
  
  
- TACGAAAATT GTAAGTAACC TGTTCTGATT TTTCTAGTCT AAAGTATCGT TTAGATAGTG AATCAGCTGT   
  
  
- ATTTATACAT TTTTTGAGCT CTAAAATAAT TACCCAGAAC TTAAATTAAT TTTGGAAATT TAATTTATTT   
  
  
- ATAATAAGAG CAGTGCTCAG TTAGAGAACA TTGTAGCAGT AAAGAAATTC TAGTCACTGG AACAACTTTT   
  
  
- GATTTTTGTG TGTGTTAAAA TTAGTAAGAA GAGAAAAAGC ACATAAGTTG AGTGAATTTA AGTTGTATAT   
  
  
- CTAACTTTCT AATGTTCAGA TCTGGATAAT ATTAAGTTAT TTCTAAGATT AATCGTTCAA CTTATTCACA   
  
  
- AGATTTGTGG TTAATGAAAA AAGTAGCTAC TAAAGATAAG TTTGATGCTA CATTTGATTA TAGATTGTTA   
  
  
- ACAGAACTTA AAGCTTGTTT ATTAGTGTCG ACGTCGAAAG TTTTGTAATG GGTCTTGGTA AAAGGAACTG   
  
  
- AAGAAAAAAT AAAAGAATAC GAACTTTATT ATGTCTTGTC TAATTGCTCA AGACATGTGA CTTTTACTTT   
  
  
- GTTCTGAAAC TTGAAGATTG AACCATATAA GATAAAACAA TATGGCAGGG TAGTACAATT CTGACCTTTT   
  
  
- GATTAATTTT CCATAAAGTA CTCTAATACT ACGTATTAAT AAAAATTTTA TTAAAAAATA AATTTTTATA   
  
  
- TAATTTTATT TTAAAGAATA AATAAAAATT ATAGTTGTCT AATTTTGATA GTATTTAGTG ATTTATTAAT   
  
  
- AGTTAAATTA TAAAAAGTTT GGTTTTTGTG TAAATTTTAT GTGAAGTTTT GACACAGTTG AACTTCGAGT   
  
  
- TTTAAGTGTC TCTTCAATTT TTGTAAATTG ACATTTACGC TTCGATGTGA CAGGTTTGGA AGGTCACTGT   
  
  
- TACTTTTCGA ACGGTGAAAC ATAACTTTAT AACCATGGTG CACATTCTGT ATACGCTTGT TCCGTTACCA   
  
  
- CTGCAGCCGT TCCATATCCG TTAGCACCCT GGTTAGGGCG GCGGCATGCA GTGATTTGTG TGCACGGAAT   
  
  
- TTTTGTTTAA GGGTTCGGAA GGATCGTTGT TTCTTGTTC

+     TATA-box

| Site Name | Organism | Position | Strand | Matrix score. | sequence | function |
| --- | --- | --- | --- | --- | --- | --- |
| TATA-box | Arabidopsis thaliana | 117 | + | 9 | ccTATAAAaa | core promoter element around -30 of transcription start |
| TATA-box | Arabidopsis thaliana | 1904 | - | 4 | TATA | core promoter element around -30 of transcription start |
| TATA-box | Arabidopsis thaliana | 119 | + | 4 | TATA | core promoter element around -30 of transcription start |
| TATA-box | Arabidopsis thaliana | 1608 | - | 4 | TATA | core promoter element around -30 of transcription start |
| TATA-box | Arabidopsis thaliana | 195 | + | 4 | TATA | core promoter element around -30 of transcription start |
| TATA-box | Pisum sativum | 199 | - | 7 | TATAAAA | core promoter element around -30 of transcription start |
| TATA-box | Helianthus annuus | 200 | - | 6 | TATAAA | core promoter element around -30 of transcription start |
| TATA-box | Arabidopsis thaliana | 201 | - | 7 | TATATAA | core promoter element around -30 of transcription start |
| TATA-box | Arabidopsis thaliana | 202 | + | 6 | TATATA | core promoter element around -30 of transcription start |
| TATA-box | Brassica oleracea | 203 | + | 6 | ATATAA | core promoter element around -30 of transcription start |
| TATA-box | Arabidopsis thaliana | 204 | + | 4 | TATA | core promoter element around -30 of transcription start |
| TATA-box | Arabidopsis thaliana | 243 | + | 4 | TATA | core promoter element around -30 of transcription start |
| TATA-box | Arabidopsis thaliana | 1187 | - | 4 | TATA | core promoter element around -30 of transcription start |
| TATA-box | Brassica napus | 1217 | + | 6 | ATTATA | core promoter element around -30 of transcription start |
| TATA-box | Arabidopsis thaliana | 1218 | - | 5 | TATAA | core promoter element around -30 of transcription start |
| TATA-box | Arabidopsis thaliana | 1219 | - | 4 | TATA | core promoter element around -30 of transcription start |
| TATA-box | Arabidopsis thaliana | 1229 | + | 8 | TAAAGATT | core promoter element around -30 of transcription start |
| TATA-box | Arabidopsis thaliana | 1495 | - | 4 | TATA | core promoter element around -30 of transcription start |
| TATA-box | Arabidopsis thaliana | 1509 | - | 5 | TATAA | core promoter element around -30 of transcription start |
| TATA-box | Arabidopsis thaliana | 1510 | - | 4 | TATA | core promoter element around -30 of transcription start |
| TATA-box | Arabidopsis thaliana | 1598 | + | 8 | TATTTAAA | core promoter element around -30 of transcription start |
| TATA-box | Brassica napus | 1607 | - | 6 | ATATAT | core promoter element around -30 of transcription start |

>Potri.002G039100.1   
+ TTTTTTTTTA TCCGTAATTG ATTATTCAAA ACCTTTCCAG CTTAAAATTT TTTCCATAAT TTAAAAACTA   
  
  
+ TTAAAGGTTG AAAACACTTT TTTTTTCTCT GAGATTTAAA ACTAATACTA TAAAAACTCT AACTCTAACT   
  
  
+ TTCCGTTGTT TATTATTTAT CACCGAATCT TCTTGATAAA TGTATTTCTT AAAGTATATT TTATATAAAA   
  
  
+ ATATTTTTTT TATTTTTAAA ATTTTCCTTT AATATACATC ATGTAAAGTT TCAAATCGAT TTAAAAATTA   
  
  
+ AACATAATTT TTTTAAAAAA TTAACGGAAC CGCAAAAAAA TAAAGAGATG GTCGCCATCA ACCGTGTAGC   
  
  
+ AACCCTTTCT TTTCGGAGGA CTTTCAAAAT TCAGGCATTT CCATGGAGAT TTTTTGTCTT CTTGAAGACC   
  
  
+ GGGAACGGTT TCAAGCAATC CCACGAAAGG GGGCCTGAAT CTAGACAACG TTGCTATCAA ACAGCTTCAT   
  
  
+ CTGTGTTAAA TATTTTATGG ATTTGATTTA CCTGTCCCCT CTGCCAGTCT TTTCAAATCA AATTTGCCAC   
  
  
+ AAGAAACCAT GACTTTTATT TCTTTCATGT GCACATCATT ACCTTAAGTG ATAACGATTA AAGGTGGAGG   
  
  
+ TTTGCCTAAA ATTACCAGTT GATATTAAAT ACGCAACAAC TTAATTTCAC ATGAATCCTC ACCCGTATTA   
  
  
+ GCTAGGGTGC CAAACTCTCT TGATCACCAT CCGTGATTAA GCGGTGCGTG GTTTTATTGA TTAAAACTAA   
  
  
+ TGTGATTTAA ATCTGATTAT CCCCGAGTTA ATTATGAAGG CTCGGAACTC CATTTTCTTC TCCTTTTTTT   
  
  
+ CACTTTTTTC TTGGACAGGG GACGAAAGTT GGTTCATGTG GCATCAAAAA AGTAGTGTTT AATAATTTAG   
  
  
+ ATGCTTTTAA CATTCATTGG ACAAGACTAA AAAGATCAGA TTTCATAGCA AATCTATCAC TTAGTCGACA   
  
  
+ TAAATATGTA AAAAACTCGA GATTTTATTA ATGGGTCTTG AATTTAATTA AAACCTTTAA ATTAAATAAA   
  
  
+ TATTATTCTC GTCACGAGTC AATCTCTTGT AACATCGTCA TTTCTTTAAG ATCAGTGACC TTGTTGAAAA   
  
  
+ CTAAAAACAC ACACAATTTT AATCATTCTT CTCTTTTTCG TGTATTCAAC TCACTTAAAT TCAACATATA   
  
  
+ GATTGAAAGA TTACAAGTCT AGACCTATTA TAATTCAATA AAGATTCTAA TTAGCAAGTT GAATAAGTGT   
  
  
+ TCTAAACACC AATTACTTTT TTCATCGATG ATTTCTATTC AAACTACGAT GTAAACTAAT ATCTAACAAT   
  
  
+ TGTCTTGAAT TTCGAACAAA TAATCACAGC TGCAGCTTTC AAAACATTAC CCAGAACCAT TTTCCTTGAC   
  
  
+ TTCTTTTTTA TTTTCTTATG CTTGAAATAA TACAGAACAG ATTAACGAGT TCTGTACACT GAAAATGAAA   
  
  
+ CAAGACTTTG AACTTCTAAC TTGGTATATT CTATTTTGTT ATACCGTCCC ATCATGTTAA GACTGGAAAA   
  
  
+ CTAATTAAAA GGTATTTCAT GAGATTATGA TGCATAATTA TTTTTAAAAT AATTTTTTAT TTAAAAATAT   
  
  
+ ATTAAAATAA AATTTCTTAT TTATTTTTAA TATCAACAGA TTAAAACTAT CATAAATCAC TAAATAATTA   
  
  
+ TCAATTTAAT ATTTTTCAAA CCAAAAACAC ATTTAAAATA CACTTCAAAA CTGTGTCAAC TTGAAGCTCA   
  
  
+ AAATTCACAG AGAAGTTAAA AACATTTAAC TGTAAATGCG AAGCTACACT GTCCAAACCT TCCAGTGACA   
  
  
+ ATGAAAAGCT TGCCACTTTG TATTGAAATA TTGGTACCAC GTGTAAGACA TATGCGAACA AGGCAATGGT   
  
  
+ GACGTCGGCA AGGTATAGGC AATCGTGGGA CCAATCCCGC CGCCGTACGT CACTAAACAC ACGTGCCTTA   
  
  
+ AAAACAAATT CCCAAGCCTT CCTAGCAACA AAGAACAAG  

- AAAAAAAAAT AGGCATTAAC TAATAAGTTT TGGAAAGGTC GAATTTTAAA AAAGGTATTA AATTTTTGAT   
  
  
- AATTTCCAAC TTTTGTGAAA AAAAAAGAGA CTCTAAATTT TGATTATGAT ATTTTTGAGA TTGAGATTGA   
  
  
- AAGGCAACAA ATAATAAATA GTGGCTTAGA AGAACTATTT ACATAAAGAA TTTCATATAA AATATATTTT   
  
  
- TATAAAAAAA ATAAAAATTT TAAAAGGAAA TTATATGTAG TACATTTCAA AGTTTAGCTA AATTTTTAAT   
  
  
- TTGTATTAAA AAAATTTTTT AATTGCCTTG GCGTTTTTTT ATTTCTCTAC CAGCGGTAGT TGGCACATCG   
  
  
- TTGGGAAAGA AAAGCCTCCT GAAAGTTTTA AGTCCGTAAA GGTACCTCTA AAAAACAGAA GAACTTCTGG   
  
  
- CCCTTGCCAA AGTTCGTTAG GGTGCTTTCC CCCGGACTTA GATCTGTTGC AACGATAGTT TGTCGAAGTA   
  
  
- GACACAATTT ATAAAATACC TAAACTAAAT GGACAGGGGA GACGGTCAGA AAAGTTTAGT TTAAACGGTG   
  
  
- TTCTTTGGTA CTGAAAATAA AGAAAGTACA CGTGTAGTAA TGGAATTCAC TATTGCTAAT TTCCACCTCC   
  
  
- AAACGGATTT TAATGGTCAA CTATAATTTA TGCGTTGTTG AATTAAAGTG TACTTAGGAG TGGGCATAAT   
  
  
- CGATCCCACG GTTTGAGAGA ACTAGTGGTA GGCACTAATT CGCCACGCAC CAAAATAACT AATTTTGATT   
  
  
- ACACTAAATT TAGACTAATA GGGGCTCAAT TAATACTTCC GAGCCTTGAG GTAAAAGAAG AGGAAAAAAA   
  
  
- GTGAAAAAAG AACCTGTCCC CTGCTTTCAA CCAAGTACAC CGTAGTTTTT TCATCACAAA TTATTAAATC   
  
  
- TACGAAAATT GTAAGTAACC TGTTCTGATT TTTCTAGTCT AAAGTATCGT TTAGATAGTG AATCAGCTGT   
  
  
- ATTTATACAT TTTTTGAGCT CTAAAATAAT TACCCAGAAC TTAAATTAAT TTTGGAAATT TAATTTATTT   
  
  
- ATAATAAGAG CAGTGCTCAG TTAGAGAACA TTGTAGCAGT AAAGAAATTC TAGTCACTGG AACAACTTTT   
  
  
- GATTTTTGTG TGTGTTAAAA TTAGTAAGAA GAGAAAAAGC ACATAAGTTG AGTGAATTTA AGTTGTATAT   
  
  
- CTAACTTTCT AATGTTCAGA TCTGGATAAT ATTAAGTTAT TTCTAAGATT AATCGTTCAA CTTATTCACA   
  
  
- AGATTTGTGG TTAATGAAAA AAGTAGCTAC TAAAGATAAG TTTGATGCTA CATTTGATTA TAGATTGTTA   
  
  
- ACAGAACTTA AAGCTTGTTT ATTAGTGTCG ACGTCGAAAG TTTTGTAATG GGTCTTGGTA AAAGGAACTG   
  
  
- AAGAAAAAAT AAAAGAATAC GAACTTTATT ATGTCTTGTC TAATTGCTCA AGACATGTGA CTTTTACTTT   
  
  
- GTTCTGAAAC TTGAAGATTG AACCATATAA GATAAAACAA TATGGCAGGG TAGTACAATT CTGACCTTTT   
  
  
- GATTAATTTT CCATAAAGTA CTCTAATACT ACGTATTAAT AAAAATTTTA TTAAAAAATA AATTTTTATA   
  
  
- TAATTTTATT TTAAAGAATA AATAAAAATT ATAGTTGTCT AATTTTGATA GTATTTAGTG ATTTATTAAT   
  
  
- AGTTAAATTA TAAAAAGTTT GGTTTTTGTG TAAATTTTAT GTGAAGTTTT GACACAGTTG AACTTCGAGT   
  
  
- TTTAAGTGTC TCTTCAATTT TTGTAAATTG ACATTTACGC TTCGATGTGA CAGGTTTGGA AGGTCACTGT   
  
  
- TACTTTTCGA ACGGTGAAAC ATAACTTTAT AACCATGGTG CACATTCTGT ATACGCTTGT TCCGTTACCA   
  
  
- CTGCAGCCGT TCCATATCCG TTAGCACCCT GGTTAGGGCG GCGGCATGCA GTGATTTGTG TGCACGGAAT   
  
  
- TTTTGTTTAA GGGTTCGGAA GGATCGTTGT TTCTTGTTC

+     TCA-element

| Site Name | Organism | Position | Strand | Matrix score. | sequence | function |
| --- | --- | --- | --- | --- | --- | --- |
| TCA-element | Nicotiana tabacum | 322 | - | 9 | CCATCTTTTT | cis-acting element involved in salicylic acid responsiveness |

>Potri.002G039100.1   
+ TTTTTTTTTA TCCGTAATTG ATTATTCAAA ACCTTTCCAG CTTAAAATTT TTTCCATAAT TTAAAAACTA   
  
  
+ TTAAAGGTTG AAAACACTTT TTTTTTCTCT GAGATTTAAA ACTAATACTA TAAAAACTCT AACTCTAACT   
  
  
+ TTCCGTTGTT TATTATTTAT CACCGAATCT TCTTGATAAA TGTATTTCTT AAAGTATATT TTATATAAAA   
  
  
+ ATATTTTTTT TATTTTTAAA ATTTTCCTTT AATATACATC ATGTAAAGTT TCAAATCGAT TTAAAAATTA   
  
  
+ AACATAATTT TTTTAAAAAA TTAACGGAAC CGCAAAAAAA TAAAGAGATG GTCGCCATCA ACCGTGTAGC   
  
  
+ AACCCTTTCT TTTCGGAGGA CTTTCAAAAT TCAGGCATTT CCATGGAGAT TTTTTGTCTT CTTGAAGACC   
  
  
+ GGGAACGGTT TCAAGCAATC CCACGAAAGG GGGCCTGAAT CTAGACAACG TTGCTATCAA ACAGCTTCAT   
  
  
+ CTGTGTTAAA TATTTTATGG ATTTGATTTA CCTGTCCCCT CTGCCAGTCT TTTCAAATCA AATTTGCCAC   
  
  
+ AAGAAACCAT GACTTTTATT TCTTTCATGT GCACATCATT ACCTTAAGTG ATAACGATTA AAGGTGGAGG   
  
  
+ TTTGCCTAAA ATTACCAGTT GATATTAAAT ACGCAACAAC TTAATTTCAC ATGAATCCTC ACCCGTATTA   
  
  
+ GCTAGGGTGC CAAACTCTCT TGATCACCAT CCGTGATTAA GCGGTGCGTG GTTTTATTGA TTAAAACTAA   
  
  
+ TGTGATTTAA ATCTGATTAT CCCCGAGTTA ATTATGAAGG CTCGGAACTC CATTTTCTTC TCCTTTTTTT   
  
  
+ CACTTTTTTC TTGGACAGGG GACGAAAGTT GGTTCATGTG GCATCAAAAA AGTAGTGTTT AATAATTTAG   
  
  
+ ATGCTTTTAA CATTCATTGG ACAAGACTAA AAAGATCAGA TTTCATAGCA AATCTATCAC TTAGTCGACA   
  
  
+ TAAATATGTA AAAAACTCGA GATTTTATTA ATGGGTCTTG AATTTAATTA AAACCTTTAA ATTAAATAAA   
  
  
+ TATTATTCTC GTCACGAGTC AATCTCTTGT AACATCGTCA TTTCTTTAAG ATCAGTGACC TTGTTGAAAA   
  
  
+ CTAAAAACAC ACACAATTTT AATCATTCTT CTCTTTTTCG TGTATTCAAC TCACTTAAAT TCAACATATA   
  
  
+ GATTGAAAGA TTACAAGTCT AGACCTATTA TAATTCAATA AAGATTCTAA TTAGCAAGTT GAATAAGTGT   
  
  
+ TCTAAACACC AATTACTTTT TTCATCGATG ATTTCTATTC AAACTACGAT GTAAACTAAT ATCTAACAAT   
  
  
+ TGTCTTGAAT TTCGAACAAA TAATCACAGC TGCAGCTTTC AAAACATTAC CCAGAACCAT TTTCCTTGAC   
  
  
+ TTCTTTTTTA TTTTCTTATG CTTGAAATAA TACAGAACAG ATTAACGAGT TCTGTACACT GAAAATGAAA   
  
  
+ CAAGACTTTG AACTTCTAAC TTGGTATATT CTATTTTGTT ATACCGTCCC ATCATGTTAA GACTGGAAAA   
  
  
+ CTAATTAAAA GGTATTTCAT GAGATTATGA TGCATAATTA TTTTTAAAAT AATTTTTTAT TTAAAAATAT   
  
  
+ ATTAAAATAA AATTTCTTAT TTATTTTTAA TATCAACAGA TTAAAACTAT CATAAATCAC TAAATAATTA   
  
  
+ TCAATTTAAT ATTTTTCAAA CCAAAAACAC ATTTAAAATA CACTTCAAAA CTGTGTCAAC TTGAAGCTCA   
  
  
+ AAATTCACAG AGAAGTTAAA AACATTTAAC TGTAAATGCG AAGCTACACT GTCCAAACCT TCCAGTGACA   
  
  
+ ATGAAAAGCT TGCCACTTTG TATTGAAATA TTGGTACCAC GTGTAAGACA TATGCGAACA AGGCAATGGT   
  
  
+ GACGTCGGCA AGGTATAGGC AATCGTGGGA CCAATCCCGC CGCCGTACGT CACTAAACAC ACGTGCCTTA   
  
  
+ AAAACAAATT CCCAAGCCTT CCTAGCAACA AAGAACAAG  

- AAAAAAAAAT AGGCATTAAC TAATAAGTTT TGGAAAGGTC GAATTTTAAA AAAGGTATTA AATTTTTGAT   
  
  
- AATTTCCAAC TTTTGTGAAA AAAAAAGAGA CTCTAAATTT TGATTATGAT ATTTTTGAGA TTGAGATTGA   
  
  
- AAGGCAACAA ATAATAAATA GTGGCTTAGA AGAACTATTT ACATAAAGAA TTTCATATAA AATATATTTT   
  
  
- TATAAAAAAA ATAAAAATTT TAAAAGGAAA TTATATGTAG TACATTTCAA AGTTTAGCTA AATTTTTAAT   
  
  
- TTGTATTAAA AAAATTTTTT AATTGCCTTG GCGTTTTTTT ATTTCTCTAC CAGCGGTAGT TGGCACATCG   
  
  
- TTGGGAAAGA AAAGCCTCCT GAAAGTTTTA AGTCCGTAAA GGTACCTCTA AAAAACAGAA GAACTTCTGG   
  
  
- CCCTTGCCAA AGTTCGTTAG GGTGCTTTCC CCCGGACTTA GATCTGTTGC AACGATAGTT TGTCGAAGTA   
  
  
- GACACAATTT ATAAAATACC TAAACTAAAT GGACAGGGGA GACGGTCAGA AAAGTTTAGT TTAAACGGTG   
  
  
- TTCTTTGGTA CTGAAAATAA AGAAAGTACA CGTGTAGTAA TGGAATTCAC TATTGCTAAT TTCCACCTCC   
  
  
- AAACGGATTT TAATGGTCAA CTATAATTTA TGCGTTGTTG AATTAAAGTG TACTTAGGAG TGGGCATAAT   
  
  
- CGATCCCACG GTTTGAGAGA ACTAGTGGTA GGCACTAATT CGCCACGCAC CAAAATAACT AATTTTGATT   
  
  
- ACACTAAATT TAGACTAATA GGGGCTCAAT TAATACTTCC GAGCCTTGAG GTAAAAGAAG AGGAAAAAAA   
  
  
- GTGAAAAAAG AACCTGTCCC CTGCTTTCAA CCAAGTACAC CGTAGTTTTT TCATCACAAA TTATTAAATC   
  
  
- TACGAAAATT GTAAGTAACC TGTTCTGATT TTTCTAGTCT AAAGTATCGT TTAGATAGTG AATCAGCTGT   
  
  
- ATTTATACAT TTTTTGAGCT CTAAAATAAT TACCCAGAAC TTAAATTAAT TTTGGAAATT TAATTTATTT   
  
  
- ATAATAAGAG CAGTGCTCAG TTAGAGAACA TTGTAGCAGT AAAGAAATTC TAGTCACTGG AACAACTTTT   
  
  
- GATTTTTGTG TGTGTTAAAA TTAGTAAGAA GAGAAAAAGC ACATAAGTTG AGTGAATTTA AGTTGTATAT   
  
  
- CTAACTTTCT AATGTTCAGA TCTGGATAAT ATTAAGTTAT TTCTAAGATT AATCGTTCAA CTTATTCACA   
  
  
- AGATTTGTGG TTAATGAAAA AAGTAGCTAC TAAAGATAAG TTTGATGCTA CATTTGATTA TAGATTGTTA   
  
  
- ACAGAACTTA AAGCTTGTTT ATTAGTGTCG ACGTCGAAAG TTTTGTAATG GGTCTTGGTA AAAGGAACTG   
  
  
- AAGAAAAAAT AAAAGAATAC GAACTTTATT ATGTCTTGTC TAATTGCTCA AGACATGTGA CTTTTACTTT   
  
  
- GTTCTGAAAC TTGAAGATTG AACCATATAA GATAAAACAA TATGGCAGGG TAGTACAATT CTGACCTTTT   
  
  
- GATTAATTTT CCATAAAGTA CTCTAATACT ACGTATTAAT AAAAATTTTA TTAAAAAATA AATTTTTATA   
  
  
- TAATTTTATT TTAAAGAATA AATAAAAATT ATAGTTGTCT AATTTTGATA GTATTTAGTG ATTTATTAAT   
  
  
- AGTTAAATTA TAAAAAGTTT GGTTTTTGTG TAAATTTTAT GTGAAGTTTT GACACAGTTG AACTTCGAGT   
  
  
- TTTAAGTGTC TCTTCAATTT TTGTAAATTG ACATTTACGC TTCGATGTGA CAGGTTTGGA AGGTCACTGT   
  
  
- TACTTTTCGA ACGGTGAAAC ATAACTTTAT AACCATGGTG CACATTCTGT ATACGCTTGT TCCGTTACCA   
  
  
- CTGCAGCCGT TCCATATCCG TTAGCACCCT GGTTAGGGCG GCGGCATGCA GTGATTTGTG TGCACGGAAT   
  
  
- TTTTGTTTAA GGGTTCGGAA GGATCGTTGT TTCTTGTTC

+     TCT-motif

| Site Name | Organism | Position | Strand | Matrix score. | sequence | function |
| --- | --- | --- | --- | --- | --- | --- |
| TCT-motif | Arabidopsis thaliana | 1863 | - | 6 | TCTTAC | part of a light responsive element |

>Potri.002G039100.1   
+ TTTTTTTTTA TCCGTAATTG ATTATTCAAA ACCTTTCCAG CTTAAAATTT TTTCCATAAT TTAAAAACTA   
  
  
+ TTAAAGGTTG AAAACACTTT TTTTTTCTCT GAGATTTAAA ACTAATACTA TAAAAACTCT AACTCTAACT   
  
  
+ TTCCGTTGTT TATTATTTAT CACCGAATCT TCTTGATAAA TGTATTTCTT AAAGTATATT TTATATAAAA   
  
  
+ ATATTTTTTT TATTTTTAAA ATTTTCCTTT AATATACATC ATGTAAAGTT TCAAATCGAT TTAAAAATTA   
  
  
+ AACATAATTT TTTTAAAAAA TTAACGGAAC CGCAAAAAAA TAAAGAGATG GTCGCCATCA ACCGTGTAGC   
  
  
+ AACCCTTTCT TTTCGGAGGA CTTTCAAAAT TCAGGCATTT CCATGGAGAT TTTTTGTCTT CTTGAAGACC   
  
  
+ GGGAACGGTT TCAAGCAATC CCACGAAAGG GGGCCTGAAT CTAGACAACG TTGCTATCAA ACAGCTTCAT   
  
  
+ CTGTGTTAAA TATTTTATGG ATTTGATTTA CCTGTCCCCT CTGCCAGTCT TTTCAAATCA AATTTGCCAC   
  
  
+ AAGAAACCAT GACTTTTATT TCTTTCATGT GCACATCATT ACCTTAAGTG ATAACGATTA AAGGTGGAGG   
  
  
+ TTTGCCTAAA ATTACCAGTT GATATTAAAT ACGCAACAAC TTAATTTCAC ATGAATCCTC ACCCGTATTA   
  
  
+ GCTAGGGTGC CAAACTCTCT TGATCACCAT CCGTGATTAA GCGGTGCGTG GTTTTATTGA TTAAAACTAA   
  
  
+ TGTGATTTAA ATCTGATTAT CCCCGAGTTA ATTATGAAGG CTCGGAACTC CATTTTCTTC TCCTTTTTTT   
  
  
+ CACTTTTTTC TTGGACAGGG GACGAAAGTT GGTTCATGTG GCATCAAAAA AGTAGTGTTT AATAATTTAG   
  
  
+ ATGCTTTTAA CATTCATTGG ACAAGACTAA AAAGATCAGA TTTCATAGCA AATCTATCAC TTAGTCGACA   
  
  
+ TAAATATGTA AAAAACTCGA GATTTTATTA ATGGGTCTTG AATTTAATTA AAACCTTTAA ATTAAATAAA   
  
  
+ TATTATTCTC GTCACGAGTC AATCTCTTGT AACATCGTCA TTTCTTTAAG ATCAGTGACC TTGTTGAAAA   
  
  
+ CTAAAAACAC ACACAATTTT AATCATTCTT CTCTTTTTCG TGTATTCAAC TCACTTAAAT TCAACATATA   
  
  
+ GATTGAAAGA TTACAAGTCT AGACCTATTA TAATTCAATA AAGATTCTAA TTAGCAAGTT GAATAAGTGT   
  
  
+ TCTAAACACC AATTACTTTT TTCATCGATG ATTTCTATTC AAACTACGAT GTAAACTAAT ATCTAACAAT   
  
  
+ TGTCTTGAAT TTCGAACAAA TAATCACAGC TGCAGCTTTC AAAACATTAC CCAGAACCAT TTTCCTTGAC   
  
  
+ TTCTTTTTTA TTTTCTTATG CTTGAAATAA TACAGAACAG ATTAACGAGT TCTGTACACT GAAAATGAAA   
  
  
+ CAAGACTTTG AACTTCTAAC TTGGTATATT CTATTTTGTT ATACCGTCCC ATCATGTTAA GACTGGAAAA   
  
  
+ CTAATTAAAA GGTATTTCAT GAGATTATGA TGCATAATTA TTTTTAAAAT AATTTTTTAT TTAAAAATAT   
  
  
+ ATTAAAATAA AATTTCTTAT TTATTTTTAA TATCAACAGA TTAAAACTAT CATAAATCAC TAAATAATTA   
  
  
+ TCAATTTAAT ATTTTTCAAA CCAAAAACAC ATTTAAAATA CACTTCAAAA CTGTGTCAAC TTGAAGCTCA   
  
  
+ AAATTCACAG AGAAGTTAAA AACATTTAAC TGTAAATGCG AAGCTACACT GTCCAAACCT TCCAGTGACA   
  
  
+ ATGAAAAGCT TGCCACTTTG TATTGAAATA TTGGTACCAC GTGTAAGACA TATGCGAACA AGGCAATGGT   
  
  
+ GACGTCGGCA AGGTATAGGC AATCGTGGGA CCAATCCCGC CGCCGTACGT CACTAAACAC ACGTGCCTTA   
  
  
+ AAAACAAATT CCCAAGCCTT CCTAGCAACA AAGAACAAG  

- AAAAAAAAAT AGGCATTAAC TAATAAGTTT TGGAAAGGTC GAATTTTAAA AAAGGTATTA AATTTTTGAT   
  
  
- AATTTCCAAC TTTTGTGAAA AAAAAAGAGA CTCTAAATTT TGATTATGAT ATTTTTGAGA TTGAGATTGA   
  
  
- AAGGCAACAA ATAATAAATA GTGGCTTAGA AGAACTATTT ACATAAAGAA TTTCATATAA AATATATTTT   
  
  
- TATAAAAAAA ATAAAAATTT TAAAAGGAAA TTATATGTAG TACATTTCAA AGTTTAGCTA AATTTTTAAT   
  
  
- TTGTATTAAA AAAATTTTTT AATTGCCTTG GCGTTTTTTT ATTTCTCTAC CAGCGGTAGT TGGCACATCG   
  
  
- TTGGGAAAGA AAAGCCTCCT GAAAGTTTTA AGTCCGTAAA GGTACCTCTA AAAAACAGAA GAACTTCTGG   
  
  
- CCCTTGCCAA AGTTCGTTAG GGTGCTTTCC CCCGGACTTA GATCTGTTGC AACGATAGTT TGTCGAAGTA   
  
  
- GACACAATTT ATAAAATACC TAAACTAAAT GGACAGGGGA GACGGTCAGA AAAGTTTAGT TTAAACGGTG   
  
  
- TTCTTTGGTA CTGAAAATAA AGAAAGTACA CGTGTAGTAA TGGAATTCAC TATTGCTAAT TTCCACCTCC   
  
  
- AAACGGATTT TAATGGTCAA CTATAATTTA TGCGTTGTTG AATTAAAGTG TACTTAGGAG TGGGCATAAT   
  
  
- CGATCCCACG GTTTGAGAGA ACTAGTGGTA GGCACTAATT CGCCACGCAC CAAAATAACT AATTTTGATT   
  
  
- ACACTAAATT TAGACTAATA GGGGCTCAAT TAATACTTCC GAGCCTTGAG GTAAAAGAAG AGGAAAAAAA   
  
  
- GTGAAAAAAG AACCTGTCCC CTGCTTTCAA CCAAGTACAC CGTAGTTTTT TCATCACAAA TTATTAAATC   
  
  
- TACGAAAATT GTAAGTAACC TGTTCTGATT TTTCTAGTCT AAAGTATCGT TTAGATAGTG AATCAGCTGT   
  
  
- ATTTATACAT TTTTTGAGCT CTAAAATAAT TACCCAGAAC TTAAATTAAT TTTGGAAATT TAATTTATTT   
  
  
- ATAATAAGAG CAGTGCTCAG TTAGAGAACA TTGTAGCAGT AAAGAAATTC TAGTCACTGG AACAACTTTT   
  
  
- GATTTTTGTG TGTGTTAAAA TTAGTAAGAA GAGAAAAAGC ACATAAGTTG AGTGAATTTA AGTTGTATAT   
  
  
- CTAACTTTCT AATGTTCAGA TCTGGATAAT ATTAAGTTAT TTCTAAGATT AATCGTTCAA CTTATTCACA   
  
  
- AGATTTGTGG TTAATGAAAA AAGTAGCTAC TAAAGATAAG TTTGATGCTA CATTTGATTA TAGATTGTTA   
  
  
- ACAGAACTTA AAGCTTGTTT ATTAGTGTCG ACGTCGAAAG TTTTGTAATG GGTCTTGGTA AAAGGAACTG   
  
  
- AAGAAAAAAT AAAAGAATAC GAACTTTATT ATGTCTTGTC TAATTGCTCA AGACATGTGA CTTTTACTTT   
  
  
- GTTCTGAAAC TTGAAGATTG AACCATATAA GATAAAACAA TATGGCAGGG TAGTACAATT CTGACCTTTT   
  
  
- GATTAATTTT CCATAAAGTA CTCTAATACT ACGTATTAAT AAAAATTTTA TTAAAAAATA AATTTTTATA   
  
  
- TAATTTTATT TTAAAGAATA AATAAAAATT ATAGTTGTCT AATTTTGATA GTATTTAGTG ATTTATTAAT   
  
  
- AGTTAAATTA TAAAAAGTTT GGTTTTTGTG TAAATTTTAT GTGAAGTTTT GACACAGTTG AACTTCGAGT   
  
  
- TTTAAGTGTC TCTTCAATTT TTGTAAATTG ACATTTACGC TTCGATGTGA CAGGTTTGGA AGGTCACTGT   
  
  
- TACTTTTCGA ACGGTGAAAC ATAACTTTAT AACCATGGTG CACATTCTGT ATACGCTTGT TCCGTTACCA   
  
  
- CTGCAGCCGT TCCATATCCG TTAGCACCCT GGTTAGGGCG GCGGCATGCA GTGATTTGTG TGCACGGAAT   
  
  
- TTTTGTTTAA GGGTTCGGAA GGATCGTTGT TTCTTGTTC

+     TGACG-motif

| Site Name | Organism | Position | Strand | Matrix score. | sequence | function |
| --- | --- | --- | --- | --- | --- | --- |
| TGACG-motif | Hordeum vulgare | 1060 | - | 5 | TGACG | cis-acting regulatory element involved in the MeJA-responsiveness |
| TGACG-motif | Hordeum vulgare | 1890 | + | 5 | TGACG | cis-acting regulatory element involved in the MeJA-responsiveness |
| TGACG-motif | Hordeum vulgare | 1086 | - | 5 | TGACG | cis-acting regulatory element involved in the MeJA-responsiveness |
| TGACG-motif | Hordeum vulgare | 1938 | - | 5 | TGACG | cis-acting regulatory element involved in the MeJA-responsiveness |

>Potri.002G039100.1   
+ TTTTTTTTTA TCCGTAATTG ATTATTCAAA ACCTTTCCAG CTTAAAATTT TTTCCATAAT TTAAAAACTA   
  
  
+ TTAAAGGTTG AAAACACTTT TTTTTTCTCT GAGATTTAAA ACTAATACTA TAAAAACTCT AACTCTAACT   
  
  
+ TTCCGTTGTT TATTATTTAT CACCGAATCT TCTTGATAAA TGTATTTCTT AAAGTATATT TTATATAAAA   
  
  
+ ATATTTTTTT TATTTTTAAA ATTTTCCTTT AATATACATC ATGTAAAGTT TCAAATCGAT TTAAAAATTA   
  
  
+ AACATAATTT TTTTAAAAAA TTAACGGAAC CGCAAAAAAA TAAAGAGATG GTCGCCATCA ACCGTGTAGC   
  
  
+ AACCCTTTCT TTTCGGAGGA CTTTCAAAAT TCAGGCATTT CCATGGAGAT TTTTTGTCTT CTTGAAGACC   
  
  
+ GGGAACGGTT TCAAGCAATC CCACGAAAGG GGGCCTGAAT CTAGACAACG TTGCTATCAA ACAGCTTCAT   
  
  
+ CTGTGTTAAA TATTTTATGG ATTTGATTTA CCTGTCCCCT CTGCCAGTCT TTTCAAATCA AATTTGCCAC   
  
  
+ AAGAAACCAT GACTTTTATT TCTTTCATGT GCACATCATT ACCTTAAGTG ATAACGATTA AAGGTGGAGG   
  
  
+ TTTGCCTAAA ATTACCAGTT GATATTAAAT ACGCAACAAC TTAATTTCAC ATGAATCCTC ACCCGTATTA   
  
  
+ GCTAGGGTGC CAAACTCTCT TGATCACCAT CCGTGATTAA GCGGTGCGTG GTTTTATTGA TTAAAACTAA   
  
  
+ TGTGATTTAA ATCTGATTAT CCCCGAGTTA ATTATGAAGG CTCGGAACTC CATTTTCTTC TCCTTTTTTT   
  
  
+ CACTTTTTTC TTGGACAGGG GACGAAAGTT GGTTCATGTG GCATCAAAAA AGTAGTGTTT AATAATTTAG   
  
  
+ ATGCTTTTAA CATTCATTGG ACAAGACTAA AAAGATCAGA TTTCATAGCA AATCTATCAC TTAGTCGACA   
  
  
+ TAAATATGTA AAAAACTCGA GATTTTATTA ATGGGTCTTG AATTTAATTA AAACCTTTAA ATTAAATAAA   
  
  
+ TATTATTCTC GTCACGAGTC AATCTCTTGT AACATCGTCA TTTCTTTAAG ATCAGTGACC TTGTTGAAAA   
  
  
+ CTAAAAACAC ACACAATTTT AATCATTCTT CTCTTTTTCG TGTATTCAAC TCACTTAAAT TCAACATATA   
  
  
+ GATTGAAAGA TTACAAGTCT AGACCTATTA TAATTCAATA AAGATTCTAA TTAGCAAGTT GAATAAGTGT   
  
  
+ TCTAAACACC AATTACTTTT TTCATCGATG ATTTCTATTC AAACTACGAT GTAAACTAAT ATCTAACAAT   
  
  
+ TGTCTTGAAT TTCGAACAAA TAATCACAGC TGCAGCTTTC AAAACATTAC CCAGAACCAT TTTCCTTGAC   
  
  
+ TTCTTTTTTA TTTTCTTATG CTTGAAATAA TACAGAACAG ATTAACGAGT TCTGTACACT GAAAATGAAA   
  
  
+ CAAGACTTTG AACTTCTAAC TTGGTATATT CTATTTTGTT ATACCGTCCC ATCATGTTAA GACTGGAAAA   
  
  
+ CTAATTAAAA GGTATTTCAT GAGATTATGA TGCATAATTA TTTTTAAAAT AATTTTTTAT TTAAAAATAT   
  
  
+ ATTAAAATAA AATTTCTTAT TTATTTTTAA TATCAACAGA TTAAAACTAT CATAAATCAC TAAATAATTA   
  
  
+ TCAATTTAAT ATTTTTCAAA CCAAAAACAC ATTTAAAATA CACTTCAAAA CTGTGTCAAC TTGAAGCTCA   
  
  
+ AAATTCACAG AGAAGTTAAA AACATTTAAC TGTAAATGCG AAGCTACACT GTCCAAACCT TCCAGTGACA   
  
  
+ ATGAAAAGCT TGCCACTTTG TATTGAAATA TTGGTACCAC GTGTAAGACA TATGCGAACA AGGCAATGGT   
  
  
+ GACGTCGGCA AGGTATAGGC AATCGTGGGA CCAATCCCGC CGCCGTACGT CACTAAACAC ACGTGCCTTA   
  
  
+ AAAACAAATT CCCAAGCCTT CCTAGCAACA AAGAACAAG  

- AAAAAAAAAT AGGCATTAAC TAATAAGTTT TGGAAAGGTC GAATTTTAAA AAAGGTATTA AATTTTTGAT   
  
  
- AATTTCCAAC TTTTGTGAAA AAAAAAGAGA CTCTAAATTT TGATTATGAT ATTTTTGAGA TTGAGATTGA   
  
  
- AAGGCAACAA ATAATAAATA GTGGCTTAGA AGAACTATTT ACATAAAGAA TTTCATATAA AATATATTTT   
  
  
- TATAAAAAAA ATAAAAATTT TAAAAGGAAA TTATATGTAG TACATTTCAA AGTTTAGCTA AATTTTTAAT   
  
  
- TTGTATTAAA AAAATTTTTT AATTGCCTTG GCGTTTTTTT ATTTCTCTAC CAGCGGTAGT TGGCACATCG   
  
  
- TTGGGAAAGA AAAGCCTCCT GAAAGTTTTA AGTCCGTAAA GGTACCTCTA AAAAACAGAA GAACTTCTGG   
  
  
- CCCTTGCCAA AGTTCGTTAG GGTGCTTTCC CCCGGACTTA GATCTGTTGC AACGATAGTT TGTCGAAGTA   
  
  
- GACACAATTT ATAAAATACC TAAACTAAAT GGACAGGGGA GACGGTCAGA AAAGTTTAGT TTAAACGGTG   
  
  
- TTCTTTGGTA CTGAAAATAA AGAAAGTACA CGTGTAGTAA TGGAATTCAC TATTGCTAAT TTCCACCTCC   
  
  
- AAACGGATTT TAATGGTCAA CTATAATTTA TGCGTTGTTG AATTAAAGTG TACTTAGGAG TGGGCATAAT   
  
  
- CGATCCCACG GTTTGAGAGA ACTAGTGGTA GGCACTAATT CGCCACGCAC CAAAATAACT AATTTTGATT   
  
  
- ACACTAAATT TAGACTAATA GGGGCTCAAT TAATACTTCC GAGCCTTGAG GTAAAAGAAG AGGAAAAAAA   
  
  
- GTGAAAAAAG AACCTGTCCC CTGCTTTCAA CCAAGTACAC CGTAGTTTTT TCATCACAAA TTATTAAATC   
  
  
- TACGAAAATT GTAAGTAACC TGTTCTGATT TTTCTAGTCT AAAGTATCGT TTAGATAGTG AATCAGCTGT   
  
  
- ATTTATACAT TTTTTGAGCT CTAAAATAAT TACCCAGAAC TTAAATTAAT TTTGGAAATT TAATTTATTT   
  
  
- ATAATAAGAG CAGTGCTCAG TTAGAGAACA TTGTAGCAGT AAAGAAATTC TAGTCACTGG AACAACTTTT   
  
  
- GATTTTTGTG TGTGTTAAAA TTAGTAAGAA GAGAAAAAGC ACATAAGTTG AGTGAATTTA AGTTGTATAT   
  
  
- CTAACTTTCT AATGTTCAGA TCTGGATAAT ATTAAGTTAT TTCTAAGATT AATCGTTCAA CTTATTCACA   
  
  
- AGATTTGTGG TTAATGAAAA AAGTAGCTAC TAAAGATAAG TTTGATGCTA CATTTGATTA TAGATTGTTA   
  
  
- ACAGAACTTA AAGCTTGTTT ATTAGTGTCG ACGTCGAAAG TTTTGTAATG GGTCTTGGTA AAAGGAACTG   
  
  
- AAGAAAAAAT AAAAGAATAC GAACTTTATT ATGTCTTGTC TAATTGCTCA AGACATGTGA CTTTTACTTT   
  
  
- GTTCTGAAAC TTGAAGATTG AACCATATAA GATAAAACAA TATGGCAGGG TAGTACAATT CTGACCTTTT   
  
  
- GATTAATTTT CCATAAAGTA CTCTAATACT ACGTATTAAT AAAAATTTTA TTAAAAAATA AATTTTTATA   
  
  
- TAATTTTATT TTAAAGAATA AATAAAAATT ATAGTTGTCT AATTTTGATA GTATTTAGTG ATTTATTAAT   
  
  
- AGTTAAATTA TAAAAAGTTT GGTTTTTGTG TAAATTTTAT GTGAAGTTTT GACACAGTTG AACTTCGAGT   
  
  
- TTTAAGTGTC TCTTCAATTT TTGTAAATTG ACATTTACGC TTCGATGTGA CAGGTTTGGA AGGTCACTGT   
  
  
- TACTTTTCGA ACGGTGAAAC ATAACTTTAT AACCATGGTG CACATTCTGT ATACGCTTGT TCCGTTACCA   
  
  
- CTGCAGCCGT TCCATATCCG TTAGCACCCT GGTTAGGGCG GCGGCATGCA GTGATTTGTG TGCACGGAAT   
  
  
- TTTTGTTTAA GGGTTCGGAA GGATCGTTGT TTCTTGTTC

+     Unnamed\_\_1

| Site Name | Organism | Position | Strand | Matrix score. | sequence | function |
| --- | --- | --- | --- | --- | --- | --- |
| Unnamed\_\_1 | Zea mays | 441 | - | 5 | CGTGG |  |
| Unnamed\_\_1 | Zea mays | 1914 | + | 5 | CGTGG |  |
| Unnamed\_\_1 | Glycine max | 1020 | + | 12 | GAATTTAATTAA | 60K protein binding site |
| Unnamed\_\_1 | Zea mays | 747 | + | 5 | CGTGG |  |
| Unnamed\_\_1 | Zea mays | 1857 | - | 5 | CGTGG |  |

>Potri.002G039100.1   
+ TTTTTTTTTA TCCGTAATTG ATTATTCAAA ACCTTTCCAG CTTAAAATTT TTTCCATAAT TTAAAAACTA   
  
  
+ TTAAAGGTTG AAAACACTTT TTTTTTCTCT GAGATTTAAA ACTAATACTA TAAAAACTCT AACTCTAACT   
  
  
+ TTCCGTTGTT TATTATTTAT CACCGAATCT TCTTGATAAA TGTATTTCTT AAAGTATATT TTATATAAAA   
  
  
+ ATATTTTTTT TATTTTTAAA ATTTTCCTTT AATATACATC ATGTAAAGTT TCAAATCGAT TTAAAAATTA   
  
  
+ AACATAATTT TTTTAAAAAA TTAACGGAAC CGCAAAAAAA TAAAGAGATG GTCGCCATCA ACCGTGTAGC   
  
  
+ AACCCTTTCT TTTCGGAGGA CTTTCAAAAT TCAGGCATTT CCATGGAGAT TTTTTGTCTT CTTGAAGACC   
  
  
+ GGGAACGGTT TCAAGCAATC CCACGAAAGG GGGCCTGAAT CTAGACAACG TTGCTATCAA ACAGCTTCAT   
  
  
+ CTGTGTTAAA TATTTTATGG ATTTGATTTA CCTGTCCCCT CTGCCAGTCT TTTCAAATCA AATTTGCCAC   
  
  
+ AAGAAACCAT GACTTTTATT TCTTTCATGT GCACATCATT ACCTTAAGTG ATAACGATTA AAGGTGGAGG   
  
  
+ TTTGCCTAAA ATTACCAGTT GATATTAAAT ACGCAACAAC TTAATTTCAC ATGAATCCTC ACCCGTATTA   
  
  
+ GCTAGGGTGC CAAACTCTCT TGATCACCAT CCGTGATTAA GCGGTGCGTG GTTTTATTGA TTAAAACTAA   
  
  
+ TGTGATTTAA ATCTGATTAT CCCCGAGTTA ATTATGAAGG CTCGGAACTC CATTTTCTTC TCCTTTTTTT   
  
  
+ CACTTTTTTC TTGGACAGGG GACGAAAGTT GGTTCATGTG GCATCAAAAA AGTAGTGTTT AATAATTTAG   
  
  
+ ATGCTTTTAA CATTCATTGG ACAAGACTAA AAAGATCAGA TTTCATAGCA AATCTATCAC TTAGTCGACA   
  
  
+ TAAATATGTA AAAAACTCGA GATTTTATTA ATGGGTCTTG AATTTAATTA AAACCTTTAA ATTAAATAAA   
  
  
+ TATTATTCTC GTCACGAGTC AATCTCTTGT AACATCGTCA TTTCTTTAAG ATCAGTGACC TTGTTGAAAA   
  
  
+ CTAAAAACAC ACACAATTTT AATCATTCTT CTCTTTTTCG TGTATTCAAC TCACTTAAAT TCAACATATA   
  
  
+ GATTGAAAGA TTACAAGTCT AGACCTATTA TAATTCAATA AAGATTCTAA TTAGCAAGTT GAATAAGTGT   
  
  
+ TCTAAACACC AATTACTTTT TTCATCGATG ATTTCTATTC AAACTACGAT GTAAACTAAT ATCTAACAAT   
  
  
+ TGTCTTGAAT TTCGAACAAA TAATCACAGC TGCAGCTTTC AAAACATTAC CCAGAACCAT TTTCCTTGAC   
  
  
+ TTCTTTTTTA TTTTCTTATG CTTGAAATAA TACAGAACAG ATTAACGAGT TCTGTACACT GAAAATGAAA   
  
  
+ CAAGACTTTG AACTTCTAAC TTGGTATATT CTATTTTGTT ATACCGTCCC ATCATGTTAA GACTGGAAAA   
  
  
+ CTAATTAAAA GGTATTTCAT GAGATTATGA TGCATAATTA TTTTTAAAAT AATTTTTTAT TTAAAAATAT   
  
  
+ ATTAAAATAA AATTTCTTAT TTATTTTTAA TATCAACAGA TTAAAACTAT CATAAATCAC TAAATAATTA   
  
  
+ TCAATTTAAT ATTTTTCAAA CCAAAAACAC ATTTAAAATA CACTTCAAAA CTGTGTCAAC TTGAAGCTCA   
  
  
+ AAATTCACAG AGAAGTTAAA AACATTTAAC TGTAAATGCG AAGCTACACT GTCCAAACCT TCCAGTGACA   
  
  
+ ATGAAAAGCT TGCCACTTTG TATTGAAATA TTGGTACCAC GTGTAAGACA TATGCGAACA AGGCAATGGT   
  
  
+ GACGTCGGCA AGGTATAGGC AATCGTGGGA CCAATCCCGC CGCCGTACGT CACTAAACAC ACGTGCCTTA   
  
  
+ AAAACAAATT CCCAAGCCTT CCTAGCAACA AAGAACAAG  

- AAAAAAAAAT AGGCATTAAC TAATAAGTTT TGGAAAGGTC GAATTTTAAA AAAGGTATTA AATTTTTGAT   
  
  
- AATTTCCAAC TTTTGTGAAA AAAAAAGAGA CTCTAAATTT TGATTATGAT ATTTTTGAGA TTGAGATTGA   
  
  
- AAGGCAACAA ATAATAAATA GTGGCTTAGA AGAACTATTT ACATAAAGAA TTTCATATAA AATATATTTT   
  
  
- TATAAAAAAA ATAAAAATTT TAAAAGGAAA TTATATGTAG TACATTTCAA AGTTTAGCTA AATTTTTAAT   
  
  
- TTGTATTAAA AAAATTTTTT AATTGCCTTG GCGTTTTTTT ATTTCTCTAC CAGCGGTAGT TGGCACATCG   
  
  
- TTGGGAAAGA AAAGCCTCCT GAAAGTTTTA AGTCCGTAAA GGTACCTCTA AAAAACAGAA GAACTTCTGG   
  
  
- CCCTTGCCAA AGTTCGTTAG GGTGCTTTCC CCCGGACTTA GATCTGTTGC AACGATAGTT TGTCGAAGTA   
  
  
- GACACAATTT ATAAAATACC TAAACTAAAT GGACAGGGGA GACGGTCAGA AAAGTTTAGT TTAAACGGTG   
  
  
- TTCTTTGGTA CTGAAAATAA AGAAAGTACA CGTGTAGTAA TGGAATTCAC TATTGCTAAT TTCCACCTCC   
  
  
- AAACGGATTT TAATGGTCAA CTATAATTTA TGCGTTGTTG AATTAAAGTG TACTTAGGAG TGGGCATAAT   
  
  
- CGATCCCACG GTTTGAGAGA ACTAGTGGTA GGCACTAATT CGCCACGCAC CAAAATAACT AATTTTGATT   
  
  
- ACACTAAATT TAGACTAATA GGGGCTCAAT TAATACTTCC GAGCCTTGAG GTAAAAGAAG AGGAAAAAAA   
  
  
- GTGAAAAAAG AACCTGTCCC CTGCTTTCAA CCAAGTACAC CGTAGTTTTT TCATCACAAA TTATTAAATC   
  
  
- TACGAAAATT GTAAGTAACC TGTTCTGATT TTTCTAGTCT AAAGTATCGT TTAGATAGTG AATCAGCTGT   
  
  
- ATTTATACAT TTTTTGAGCT CTAAAATAAT TACCCAGAAC TTAAATTAAT TTTGGAAATT TAATTTATTT   
  
  
- ATAATAAGAG CAGTGCTCAG TTAGAGAACA TTGTAGCAGT AAAGAAATTC TAGTCACTGG AACAACTTTT   
  
  
- GATTTTTGTG TGTGTTAAAA TTAGTAAGAA GAGAAAAAGC ACATAAGTTG AGTGAATTTA AGTTGTATAT   
  
  
- CTAACTTTCT AATGTTCAGA TCTGGATAAT ATTAAGTTAT TTCTAAGATT AATCGTTCAA CTTATTCACA   
  
  
- AGATTTGTGG TTAATGAAAA AAGTAGCTAC TAAAGATAAG TTTGATGCTA CATTTGATTA TAGATTGTTA   
  
  
- ACAGAACTTA AAGCTTGTTT ATTAGTGTCG ACGTCGAAAG TTTTGTAATG GGTCTTGGTA AAAGGAACTG   
  
  
- AAGAAAAAAT AAAAGAATAC GAACTTTATT ATGTCTTGTC TAATTGCTCA AGACATGTGA CTTTTACTTT   
  
  
- GTTCTGAAAC TTGAAGATTG AACCATATAA GATAAAACAA TATGGCAGGG TAGTACAATT CTGACCTTTT   
  
  
- GATTAATTTT CCATAAAGTA CTCTAATACT ACGTATTAAT AAAAATTTTA TTAAAAAATA AATTTTTATA   
  
  
- TAATTTTATT TTAAAGAATA AATAAAAATT ATAGTTGTCT AATTTTGATA GTATTTAGTG ATTTATTAAT   
  
  
- AGTTAAATTA TAAAAAGTTT GGTTTTTGTG TAAATTTTAT GTGAAGTTTT GACACAGTTG AACTTCGAGT   
  
  
- TTTAAGTGTC TCTTCAATTT TTGTAAATTG ACATTTACGC TTCGATGTGA CAGGTTTGGA AGGTCACTGT   
  
  
- TACTTTTCGA ACGGTGAAAC ATAACTTTAT AACCATGGTG CACATTCTGT ATACGCTTGT TCCGTTACCA   
  
  
- CTGCAGCCGT TCCATATCCG TTAGCACCCT GGTTAGGGCG GCGGCATGCA GTGATTTGTG TGCACGGAAT   
  
  
- TTTTGTTTAA GGGTTCGGAA GGATCGTTGT TTCTTGTTC

+     Unnamed\_\_4

| Site Name | Organism | Position | Strand | Matrix score. | sequence | function |
| --- | --- | --- | --- | --- | --- | --- |
| Unnamed\_\_4 | Petroselinum hortense | 365 | - | 4 | CTCC |  |
| Unnamed\_\_4 | Petroselinum hortense | 830 | + | 4 | CTCC |  |
| Unnamed\_\_4 | Petroselinum hortense | 626 | - | 4 | CTCC |  |
| Unnamed\_\_4 | Petroselinum hortense | 395 | - | 4 | CTCC |  |
| Unnamed\_\_4 | Petroselinum hortense | 818 | + | 4 | CTCC |  |

>Potri.002G039100.1   
+ TTTTTTTTTA TCCGTAATTG ATTATTCAAA ACCTTTCCAG CTTAAAATTT TTTCCATAAT TTAAAAACTA   
  
  
+ TTAAAGGTTG AAAACACTTT TTTTTTCTCT GAGATTTAAA ACTAATACTA TAAAAACTCT AACTCTAACT   
  
  
+ TTCCGTTGTT TATTATTTAT CACCGAATCT TCTTGATAAA TGTATTTCTT AAAGTATATT TTATATAAAA   
  
  
+ ATATTTTTTT TATTTTTAAA ATTTTCCTTT AATATACATC ATGTAAAGTT TCAAATCGAT TTAAAAATTA   
  
  
+ AACATAATTT TTTTAAAAAA TTAACGGAAC CGCAAAAAAA TAAAGAGATG GTCGCCATCA ACCGTGTAGC   
  
  
+ AACCCTTTCT TTTCGGAGGA CTTTCAAAAT TCAGGCATTT CCATGGAGAT TTTTTGTCTT CTTGAAGACC   
  
  
+ GGGAACGGTT TCAAGCAATC CCACGAAAGG GGGCCTGAAT CTAGACAACG TTGCTATCAA ACAGCTTCAT   
  
  
+ CTGTGTTAAA TATTTTATGG ATTTGATTTA CCTGTCCCCT CTGCCAGTCT TTTCAAATCA AATTTGCCAC   
  
  
+ AAGAAACCAT GACTTTTATT TCTTTCATGT GCACATCATT ACCTTAAGTG ATAACGATTA AAGGTGGAGG   
  
  
+ TTTGCCTAAA ATTACCAGTT GATATTAAAT ACGCAACAAC TTAATTTCAC ATGAATCCTC ACCCGTATTA   
  
  
+ GCTAGGGTGC CAAACTCTCT TGATCACCAT CCGTGATTAA GCGGTGCGTG GTTTTATTGA TTAAAACTAA   
  
  
+ TGTGATTTAA ATCTGATTAT CCCCGAGTTA ATTATGAAGG CTCGGAACTC CATTTTCTTC TCCTTTTTTT   
  
  
+ CACTTTTTTC TTGGACAGGG GACGAAAGTT GGTTCATGTG GCATCAAAAA AGTAGTGTTT AATAATTTAG   
  
  
+ ATGCTTTTAA CATTCATTGG ACAAGACTAA AAAGATCAGA TTTCATAGCA AATCTATCAC TTAGTCGACA   
  
  
+ TAAATATGTA AAAAACTCGA GATTTTATTA ATGGGTCTTG AATTTAATTA AAACCTTTAA ATTAAATAAA   
  
  
+ TATTATTCTC GTCACGAGTC AATCTCTTGT AACATCGTCA TTTCTTTAAG ATCAGTGACC TTGTTGAAAA   
  
  
+ CTAAAAACAC ACACAATTTT AATCATTCTT CTCTTTTTCG TGTATTCAAC TCACTTAAAT TCAACATATA   
  
  
+ GATTGAAAGA TTACAAGTCT AGACCTATTA TAATTCAATA AAGATTCTAA TTAGCAAGTT GAATAAGTGT   
  
  
+ TCTAAACACC AATTACTTTT TTCATCGATG ATTTCTATTC AAACTACGAT GTAAACTAAT ATCTAACAAT   
  
  
+ TGTCTTGAAT TTCGAACAAA TAATCACAGC TGCAGCTTTC AAAACATTAC CCAGAACCAT TTTCCTTGAC   
  
  
+ TTCTTTTTTA TTTTCTTATG CTTGAAATAA TACAGAACAG ATTAACGAGT TCTGTACACT GAAAATGAAA   
  
  
+ CAAGACTTTG AACTTCTAAC TTGGTATATT CTATTTTGTT ATACCGTCCC ATCATGTTAA GACTGGAAAA   
  
  
+ CTAATTAAAA GGTATTTCAT GAGATTATGA TGCATAATTA TTTTTAAAAT AATTTTTTAT TTAAAAATAT   
  
  
+ ATTAAAATAA AATTTCTTAT TTATTTTTAA TATCAACAGA TTAAAACTAT CATAAATCAC TAAATAATTA   
  
  
+ TCAATTTAAT ATTTTTCAAA CCAAAAACAC ATTTAAAATA CACTTCAAAA CTGTGTCAAC TTGAAGCTCA   
  
  
+ AAATTCACAG AGAAGTTAAA AACATTTAAC TGTAAATGCG AAGCTACACT GTCCAAACCT TCCAGTGACA   
  
  
+ ATGAAAAGCT TGCCACTTTG TATTGAAATA TTGGTACCAC GTGTAAGACA TATGCGAACA AGGCAATGGT   
  
  
+ GACGTCGGCA AGGTATAGGC AATCGTGGGA CCAATCCCGC CGCCGTACGT CACTAAACAC ACGTGCCTTA   
  
  
+ AAAACAAATT CCCAAGCCTT CCTAGCAACA AAGAACAAG  

- AAAAAAAAAT AGGCATTAAC TAATAAGTTT TGGAAAGGTC GAATTTTAAA AAAGGTATTA AATTTTTGAT   
  
  
- AATTTCCAAC TTTTGTGAAA AAAAAAGAGA CTCTAAATTT TGATTATGAT ATTTTTGAGA TTGAGATTGA   
  
  
- AAGGCAACAA ATAATAAATA GTGGCTTAGA AGAACTATTT ACATAAAGAA TTTCATATAA AATATATTTT   
  
  
- TATAAAAAAA ATAAAAATTT TAAAAGGAAA TTATATGTAG TACATTTCAA AGTTTAGCTA AATTTTTAAT   
  
  
- TTGTATTAAA AAAATTTTTT AATTGCCTTG GCGTTTTTTT ATTTCTCTAC CAGCGGTAGT TGGCACATCG   
  
  
- TTGGGAAAGA AAAGCCTCCT GAAAGTTTTA AGTCCGTAAA GGTACCTCTA AAAAACAGAA GAACTTCTGG   
  
  
- CCCTTGCCAA AGTTCGTTAG GGTGCTTTCC CCCGGACTTA GATCTGTTGC AACGATAGTT TGTCGAAGTA   
  
  
- GACACAATTT ATAAAATACC TAAACTAAAT GGACAGGGGA GACGGTCAGA AAAGTTTAGT TTAAACGGTG   
  
  
- TTCTTTGGTA CTGAAAATAA AGAAAGTACA CGTGTAGTAA TGGAATTCAC TATTGCTAAT TTCCACCTCC   
  
  
- AAACGGATTT TAATGGTCAA CTATAATTTA TGCGTTGTTG AATTAAAGTG TACTTAGGAG TGGGCATAAT   
  
  
- CGATCCCACG GTTTGAGAGA ACTAGTGGTA GGCACTAATT CGCCACGCAC CAAAATAACT AATTTTGATT   
  
  
- ACACTAAATT TAGACTAATA GGGGCTCAAT TAATACTTCC GAGCCTTGAG GTAAAAGAAG AGGAAAAAAA   
  
  
- GTGAAAAAAG AACCTGTCCC CTGCTTTCAA CCAAGTACAC CGTAGTTTTT TCATCACAAA TTATTAAATC   
  
  
- TACGAAAATT GTAAGTAACC TGTTCTGATT TTTCTAGTCT AAAGTATCGT TTAGATAGTG AATCAGCTGT   
  
  
- ATTTATACAT TTTTTGAGCT CTAAAATAAT TACCCAGAAC TTAAATTAAT TTTGGAAATT TAATTTATTT   
  
  
- ATAATAAGAG CAGTGCTCAG TTAGAGAACA TTGTAGCAGT AAAGAAATTC TAGTCACTGG AACAACTTTT   
  
  
- GATTTTTGTG TGTGTTAAAA TTAGTAAGAA GAGAAAAAGC ACATAAGTTG AGTGAATTTA AGTTGTATAT   
  
  
- CTAACTTTCT AATGTTCAGA TCTGGATAAT ATTAAGTTAT TTCTAAGATT AATCGTTCAA CTTATTCACA   
  
  
- AGATTTGTGG TTAATGAAAA AAGTAGCTAC TAAAGATAAG TTTGATGCTA CATTTGATTA TAGATTGTTA   
  
  
- ACAGAACTTA AAGCTTGTTT ATTAGTGTCG ACGTCGAAAG TTTTGTAATG GGTCTTGGTA AAAGGAACTG   
  
  
- AAGAAAAAAT AAAAGAATAC GAACTTTATT ATGTCTTGTC TAATTGCTCA AGACATGTGA CTTTTACTTT   
  
  
- GTTCTGAAAC TTGAAGATTG AACCATATAA GATAAAACAA TATGGCAGGG TAGTACAATT CTGACCTTTT   
  
  
- GATTAATTTT CCATAAAGTA CTCTAATACT ACGTATTAAT AAAAATTTTA TTAAAAAATA AATTTTTATA   
  
  
- TAATTTTATT TTAAAGAATA AATAAAAATT ATAGTTGTCT AATTTTGATA GTATTTAGTG ATTTATTAAT   
  
  
- AGTTAAATTA TAAAAAGTTT GGTTTTTGTG TAAATTTTAT GTGAAGTTTT GACACAGTTG AACTTCGAGT   
  
  
- TTTAAGTGTC TCTTCAATTT TTGTAAATTG ACATTTACGC TTCGATGTGA CAGGTTTGGA AGGTCACTGT   
  
  
- TACTTTTCGA ACGGTGAAAC ATAACTTTAT AACCATGGTG CACATTCTGT ATACGCTTGT TCCGTTACCA   
  
  
- CTGCAGCCGT TCCATATCCG TTAGCACCCT GGTTAGGGCG GCGGCATGCA GTGATTTGTG TGCACGGAAT   
  
  
- TTTTGTTTAA GGGTTCGGAA GGATCGTTGT TTCTTGTTC

+     WRE3

| Site Name | Organism | Position | Strand | Matrix score. | sequence | function |
| --- | --- | --- | --- | --- | --- | --- |
| WRE3 | Pisum sativum | 622 | - | 6 | CCACCT |  |

>Potri.002G039100.1   
+ TTTTTTTTTA TCCGTAATTG ATTATTCAAA ACCTTTCCAG CTTAAAATTT TTTCCATAAT TTAAAAACTA   
  
  
+ TTAAAGGTTG AAAACACTTT TTTTTTCTCT GAGATTTAAA ACTAATACTA TAAAAACTCT AACTCTAACT   
  
  
+ TTCCGTTGTT TATTATTTAT CACCGAATCT TCTTGATAAA TGTATTTCTT AAAGTATATT TTATATAAAA   
  
  
+ ATATTTTTTT TATTTTTAAA ATTTTCCTTT AATATACATC ATGTAAAGTT TCAAATCGAT TTAAAAATTA   
  
  
+ AACATAATTT TTTTAAAAAA TTAACGGAAC CGCAAAAAAA TAAAGAGATG GTCGCCATCA ACCGTGTAGC   
  
  
+ AACCCTTTCT TTTCGGAGGA CTTTCAAAAT TCAGGCATTT CCATGGAGAT TTTTTGTCTT CTTGAAGACC   
  
  
+ GGGAACGGTT TCAAGCAATC CCACGAAAGG GGGCCTGAAT CTAGACAACG TTGCTATCAA ACAGCTTCAT   
  
  
+ CTGTGTTAAA TATTTTATGG ATTTGATTTA CCTGTCCCCT CTGCCAGTCT TTTCAAATCA AATTTGCCAC   
  
  
+ AAGAAACCAT GACTTTTATT TCTTTCATGT GCACATCATT ACCTTAAGTG ATAACGATTA AAGGTGGAGG   
  
  
+ TTTGCCTAAA ATTACCAGTT GATATTAAAT ACGCAACAAC TTAATTTCAC ATGAATCCTC ACCCGTATTA   
  
  
+ GCTAGGGTGC CAAACTCTCT TGATCACCAT CCGTGATTAA GCGGTGCGTG GTTTTATTGA TTAAAACTAA   
  
  
+ TGTGATTTAA ATCTGATTAT CCCCGAGTTA ATTATGAAGG CTCGGAACTC CATTTTCTTC TCCTTTTTTT   
  
  
+ CACTTTTTTC TTGGACAGGG GACGAAAGTT GGTTCATGTG GCATCAAAAA AGTAGTGTTT AATAATTTAG   
  
  
+ ATGCTTTTAA CATTCATTGG ACAAGACTAA AAAGATCAGA TTTCATAGCA AATCTATCAC TTAGTCGACA   
  
  
+ TAAATATGTA AAAAACTCGA GATTTTATTA ATGGGTCTTG AATTTAATTA AAACCTTTAA ATTAAATAAA   
  
  
+ TATTATTCTC GTCACGAGTC AATCTCTTGT AACATCGTCA TTTCTTTAAG ATCAGTGACC TTGTTGAAAA   
  
  
+ CTAAAAACAC ACACAATTTT AATCATTCTT CTCTTTTTCG TGTATTCAAC TCACTTAAAT TCAACATATA   
  
  
+ GATTGAAAGA TTACAAGTCT AGACCTATTA TAATTCAATA AAGATTCTAA TTAGCAAGTT GAATAAGTGT   
  
  
+ TCTAAACACC AATTACTTTT TTCATCGATG ATTTCTATTC AAACTACGAT GTAAACTAAT ATCTAACAAT   
  
  
+ TGTCTTGAAT TTCGAACAAA TAATCACAGC TGCAGCTTTC AAAACATTAC CCAGAACCAT TTTCCTTGAC   
  
  
+ TTCTTTTTTA TTTTCTTATG CTTGAAATAA TACAGAACAG ATTAACGAGT TCTGTACACT GAAAATGAAA   
  
  
+ CAAGACTTTG AACTTCTAAC TTGGTATATT CTATTTTGTT ATACCGTCCC ATCATGTTAA GACTGGAAAA   
  
  
+ CTAATTAAAA GGTATTTCAT GAGATTATGA TGCATAATTA TTTTTAAAAT AATTTTTTAT TTAAAAATAT   
  
  
+ ATTAAAATAA AATTTCTTAT TTATTTTTAA TATCAACAGA TTAAAACTAT CATAAATCAC TAAATAATTA   
  
  
+ TCAATTTAAT ATTTTTCAAA CCAAAAACAC ATTTAAAATA CACTTCAAAA CTGTGTCAAC TTGAAGCTCA   
  
  
+ AAATTCACAG AGAAGTTAAA AACATTTAAC TGTAAATGCG AAGCTACACT GTCCAAACCT TCCAGTGACA   
  
  
+ ATGAAAAGCT TGCCACTTTG TATTGAAATA TTGGTACCAC GTGTAAGACA TATGCGAACA AGGCAATGGT   
  
  
+ GACGTCGGCA AGGTATAGGC AATCGTGGGA CCAATCCCGC CGCCGTACGT CACTAAACAC ACGTGCCTTA   
  
  
+ AAAACAAATT CCCAAGCCTT CCTAGCAACA AAGAACAAG  

- AAAAAAAAAT AGGCATTAAC TAATAAGTTT TGGAAAGGTC GAATTTTAAA AAAGGTATTA AATTTTTGAT   
  
  
- AATTTCCAAC TTTTGTGAAA AAAAAAGAGA CTCTAAATTT TGATTATGAT ATTTTTGAGA TTGAGATTGA   
  
  
- AAGGCAACAA ATAATAAATA GTGGCTTAGA AGAACTATTT ACATAAAGAA TTTCATATAA AATATATTTT   
  
  
- TATAAAAAAA ATAAAAATTT TAAAAGGAAA TTATATGTAG TACATTTCAA AGTTTAGCTA AATTTTTAAT   
  
  
- TTGTATTAAA AAAATTTTTT AATTGCCTTG GCGTTTTTTT ATTTCTCTAC CAGCGGTAGT TGGCACATCG   
  
  
- TTGGGAAAGA AAAGCCTCCT GAAAGTTTTA AGTCCGTAAA GGTACCTCTA AAAAACAGAA GAACTTCTGG   
  
  
- CCCTTGCCAA AGTTCGTTAG GGTGCTTTCC CCCGGACTTA GATCTGTTGC AACGATAGTT TGTCGAAGTA   
  
  
- GACACAATTT ATAAAATACC TAAACTAAAT GGACAGGGGA GACGGTCAGA AAAGTTTAGT TTAAACGGTG   
  
  
- TTCTTTGGTA CTGAAAATAA AGAAAGTACA CGTGTAGTAA TGGAATTCAC TATTGCTAAT TTCCACCTCC   
  
  
- AAACGGATTT TAATGGTCAA CTATAATTTA TGCGTTGTTG AATTAAAGTG TACTTAGGAG TGGGCATAAT   
  
  
- CGATCCCACG GTTTGAGAGA ACTAGTGGTA GGCACTAATT CGCCACGCAC CAAAATAACT AATTTTGATT   
  
  
- ACACTAAATT TAGACTAATA GGGGCTCAAT TAATACTTCC GAGCCTTGAG GTAAAAGAAG AGGAAAAAAA   
  
  
- GTGAAAAAAG AACCTGTCCC CTGCTTTCAA CCAAGTACAC CGTAGTTTTT TCATCACAAA TTATTAAATC   
  
  
- TACGAAAATT GTAAGTAACC TGTTCTGATT TTTCTAGTCT AAAGTATCGT TTAGATAGTG AATCAGCTGT   
  
  
- ATTTATACAT TTTTTGAGCT CTAAAATAAT TACCCAGAAC TTAAATTAAT TTTGGAAATT TAATTTATTT   
  
  
- ATAATAAGAG CAGTGCTCAG TTAGAGAACA TTGTAGCAGT AAAGAAATTC TAGTCACTGG AACAACTTTT   
  
  
- GATTTTTGTG TGTGTTAAAA TTAGTAAGAA GAGAAAAAGC ACATAAGTTG AGTGAATTTA AGTTGTATAT   
  
  
- CTAACTTTCT AATGTTCAGA TCTGGATAAT ATTAAGTTAT TTCTAAGATT AATCGTTCAA CTTATTCACA   
  
  
- AGATTTGTGG TTAATGAAAA AAGTAGCTAC TAAAGATAAG TTTGATGCTA CATTTGATTA TAGATTGTTA   
  
  
- ACAGAACTTA AAGCTTGTTT ATTAGTGTCG ACGTCGAAAG TTTTGTAATG GGTCTTGGTA AAAGGAACTG   
  
  
- AAGAAAAAAT AAAAGAATAC GAACTTTATT ATGTCTTGTC TAATTGCTCA AGACATGTGA CTTTTACTTT   
  
  
- GTTCTGAAAC TTGAAGATTG AACCATATAA GATAAAACAA TATGGCAGGG TAGTACAATT CTGACCTTTT   
  
  
- GATTAATTTT CCATAAAGTA CTCTAATACT ACGTATTAAT AAAAATTTTA TTAAAAAATA AATTTTTATA   
  
  
- TAATTTTATT TTAAAGAATA AATAAAAATT ATAGTTGTCT AATTTTGATA GTATTTAGTG ATTTATTAAT   
  
  
- AGTTAAATTA TAAAAAGTTT GGTTTTTGTG TAAATTTTAT GTGAAGTTTT GACACAGTTG AACTTCGAGT   
  
  
- TTTAAGTGTC TCTTCAATTT TTGTAAATTG ACATTTACGC TTCGATGTGA CAGGTTTGGA AGGTCACTGT   
  
  
- TACTTTTCGA ACGGTGAAAC ATAACTTTAT AACCATGGTG CACATTCTGT ATACGCTTGT TCCGTTACCA   
  
  
- CTGCAGCCGT TCCATATCCG TTAGCACCCT GGTTAGGGCG GCGGCATGCA GTGATTTGTG TGCACGGAAT   
  
  
- TTTTGTTTAA GGGTTCGGAA GGATCGTTGT TTCTTGTTC

+     WUN-motif

| Site Name | Organism | Position | Strand | Matrix score. | sequence | function |
| --- | --- | --- | --- | --- | --- | --- |
| WUN-motif | Nicotiana glutinosa | 1620 | + | 9 | AAATTTCTT |  |

>Potri.002G039100.1   
+ TTTTTTTTTA TCCGTAATTG ATTATTCAAA ACCTTTCCAG CTTAAAATTT TTTCCATAAT TTAAAAACTA   
  
  
+ TTAAAGGTTG AAAACACTTT TTTTTTCTCT GAGATTTAAA ACTAATACTA TAAAAACTCT AACTCTAACT   
  
  
+ TTCCGTTGTT TATTATTTAT CACCGAATCT TCTTGATAAA TGTATTTCTT AAAGTATATT TTATATAAAA   
  
  
+ ATATTTTTTT TATTTTTAAA ATTTTCCTTT AATATACATC ATGTAAAGTT TCAAATCGAT TTAAAAATTA   
  
  
+ AACATAATTT TTTTAAAAAA TTAACGGAAC CGCAAAAAAA TAAAGAGATG GTCGCCATCA ACCGTGTAGC   
  
  
+ AACCCTTTCT TTTCGGAGGA CTTTCAAAAT TCAGGCATTT CCATGGAGAT TTTTTGTCTT CTTGAAGACC   
  
  
+ GGGAACGGTT TCAAGCAATC CCACGAAAGG GGGCCTGAAT CTAGACAACG TTGCTATCAA ACAGCTTCAT   
  
  
+ CTGTGTTAAA TATTTTATGG ATTTGATTTA CCTGTCCCCT CTGCCAGTCT TTTCAAATCA AATTTGCCAC   
  
  
+ AAGAAACCAT GACTTTTATT TCTTTCATGT GCACATCATT ACCTTAAGTG ATAACGATTA AAGGTGGAGG   
  
  
+ TTTGCCTAAA ATTACCAGTT GATATTAAAT ACGCAACAAC TTAATTTCAC ATGAATCCTC ACCCGTATTA   
  
  
+ GCTAGGGTGC CAAACTCTCT TGATCACCAT CCGTGATTAA GCGGTGCGTG GTTTTATTGA TTAAAACTAA   
  
  
+ TGTGATTTAA ATCTGATTAT CCCCGAGTTA ATTATGAAGG CTCGGAACTC CATTTTCTTC TCCTTTTTTT   
  
  
+ CACTTTTTTC TTGGACAGGG GACGAAAGTT GGTTCATGTG GCATCAAAAA AGTAGTGTTT AATAATTTAG   
  
  
+ ATGCTTTTAA CATTCATTGG ACAAGACTAA AAAGATCAGA TTTCATAGCA AATCTATCAC TTAGTCGACA   
  
  
+ TAAATATGTA AAAAACTCGA GATTTTATTA ATGGGTCTTG AATTTAATTA AAACCTTTAA ATTAAATAAA   
  
  
+ TATTATTCTC GTCACGAGTC AATCTCTTGT AACATCGTCA TTTCTTTAAG ATCAGTGACC TTGTTGAAAA   
  
  
+ CTAAAAACAC ACACAATTTT AATCATTCTT CTCTTTTTCG TGTATTCAAC TCACTTAAAT TCAACATATA   
  
  
+ GATTGAAAGA TTACAAGTCT AGACCTATTA TAATTCAATA AAGATTCTAA TTAGCAAGTT GAATAAGTGT   
  
  
+ TCTAAACACC AATTACTTTT TTCATCGATG ATTTCTATTC AAACTACGAT GTAAACTAAT ATCTAACAAT   
  
  
+ TGTCTTGAAT TTCGAACAAA TAATCACAGC TGCAGCTTTC AAAACATTAC CCAGAACCAT TTTCCTTGAC   
  
  
+ TTCTTTTTTA TTTTCTTATG CTTGAAATAA TACAGAACAG ATTAACGAGT TCTGTACACT GAAAATGAAA   
  
  
+ CAAGACTTTG AACTTCTAAC TTGGTATATT CTATTTTGTT ATACCGTCCC ATCATGTTAA GACTGGAAAA   
  
  
+ CTAATTAAAA GGTATTTCAT GAGATTATGA TGCATAATTA TTTTTAAAAT AATTTTTTAT TTAAAAATAT   
  
  
+ ATTAAAATAA AATTTCTTAT TTATTTTTAA TATCAACAGA TTAAAACTAT CATAAATCAC TAAATAATTA   
  
  
+ TCAATTTAAT ATTTTTCAAA CCAAAAACAC ATTTAAAATA CACTTCAAAA CTGTGTCAAC TTGAAGCTCA   
  
  
+ AAATTCACAG AGAAGTTAAA AACATTTAAC TGTAAATGCG AAGCTACACT GTCCAAACCT TCCAGTGACA   
  
  
+ ATGAAAAGCT TGCCACTTTG TATTGAAATA TTGGTACCAC GTGTAAGACA TATGCGAACA AGGCAATGGT   
  
  
+ GACGTCGGCA AGGTATAGGC AATCGTGGGA CCAATCCCGC CGCCGTACGT CACTAAACAC ACGTGCCTTA   
  
  
+ AAAACAAATT CCCAAGCCTT CCTAGCAACA AAGAACAAG  

- AAAAAAAAAT AGGCATTAAC TAATAAGTTT TGGAAAGGTC GAATTTTAAA AAAGGTATTA AATTTTTGAT   
  
  
- AATTTCCAAC TTTTGTGAAA AAAAAAGAGA CTCTAAATTT TGATTATGAT ATTTTTGAGA TTGAGATTGA   
  
  
- AAGGCAACAA ATAATAAATA GTGGCTTAGA AGAACTATTT ACATAAAGAA TTTCATATAA AATATATTTT   
  
  
- TATAAAAAAA ATAAAAATTT TAAAAGGAAA TTATATGTAG TACATTTCAA AGTTTAGCTA AATTTTTAAT   
  
  
- TTGTATTAAA AAAATTTTTT AATTGCCTTG GCGTTTTTTT ATTTCTCTAC CAGCGGTAGT TGGCACATCG   
  
  
- TTGGGAAAGA AAAGCCTCCT GAAAGTTTTA AGTCCGTAAA GGTACCTCTA AAAAACAGAA GAACTTCTGG   
  
  
- CCCTTGCCAA AGTTCGTTAG GGTGCTTTCC CCCGGACTTA GATCTGTTGC AACGATAGTT TGTCGAAGTA   
  
  
- GACACAATTT ATAAAATACC TAAACTAAAT GGACAGGGGA GACGGTCAGA AAAGTTTAGT TTAAACGGTG   
  
  
- TTCTTTGGTA CTGAAAATAA AGAAAGTACA CGTGTAGTAA TGGAATTCAC TATTGCTAAT TTCCACCTCC   
  
  
- AAACGGATTT TAATGGTCAA CTATAATTTA TGCGTTGTTG AATTAAAGTG TACTTAGGAG TGGGCATAAT   
  
  
- CGATCCCACG GTTTGAGAGA ACTAGTGGTA GGCACTAATT CGCCACGCAC CAAAATAACT AATTTTGATT   
  
  
- ACACTAAATT TAGACTAATA GGGGCTCAAT TAATACTTCC GAGCCTTGAG GTAAAAGAAG AGGAAAAAAA   
  
  
- GTGAAAAAAG AACCTGTCCC CTGCTTTCAA CCAAGTACAC CGTAGTTTTT TCATCACAAA TTATTAAATC   
  
  
- TACGAAAATT GTAAGTAACC TGTTCTGATT TTTCTAGTCT AAAGTATCGT TTAGATAGTG AATCAGCTGT   
  
  
- ATTTATACAT TTTTTGAGCT CTAAAATAAT TACCCAGAAC TTAAATTAAT TTTGGAAATT TAATTTATTT   
  
  
- ATAATAAGAG CAGTGCTCAG TTAGAGAACA TTGTAGCAGT AAAGAAATTC TAGTCACTGG AACAACTTTT   
  
  
- GATTTTTGTG TGTGTTAAAA TTAGTAAGAA GAGAAAAAGC ACATAAGTTG AGTGAATTTA AGTTGTATAT   
  
  
- CTAACTTTCT AATGTTCAGA TCTGGATAAT ATTAAGTTAT TTCTAAGATT AATCGTTCAA CTTATTCACA   
  
  
- AGATTTGTGG TTAATGAAAA AAGTAGCTAC TAAAGATAAG TTTGATGCTA CATTTGATTA TAGATTGTTA   
  
  
- ACAGAACTTA AAGCTTGTTT ATTAGTGTCG ACGTCGAAAG TTTTGTAATG GGTCTTGGTA AAAGGAACTG   
  
  
- AAGAAAAAAT AAAAGAATAC GAACTTTATT ATGTCTTGTC TAATTGCTCA AGACATGTGA CTTTTACTTT   
  
  
- GTTCTGAAAC TTGAAGATTG AACCATATAA GATAAAACAA TATGGCAGGG TAGTACAATT CTGACCTTTT   
  
  
- GATTAATTTT CCATAAAGTA CTCTAATACT ACGTATTAAT AAAAATTTTA TTAAAAAATA AATTTTTATA   
  
  
- TAATTTTATT TTAAAGAATA AATAAAAATT ATAGTTGTCT AATTTTGATA GTATTTAGTG ATTTATTAAT   
  
  
- AGTTAAATTA TAAAAAGTTT GGTTTTTGTG TAAATTTTAT GTGAAGTTTT GACACAGTTG AACTTCGAGT   
  
  
- TTTAAGTGTC TCTTCAATTT TTGTAAATTG ACATTTACGC TTCGATGTGA CAGGTTTGGA AGGTCACTGT   
  
  
- TACTTTTCGA ACGGTGAAAC ATAACTTTAT AACCATGGTG CACATTCTGT ATACGCTTGT TCCGTTACCA   
  
  
- CTGCAGCCGT TCCATATCCG TTAGCACCCT GGTTAGGGCG GCGGCATGCA GTGATTTGTG TGCACGGAAT   
  
  
- TTTTGTTTAA GGGTTCGGAA GGATCGTTGT TTCTTGTTC

+     as-1

| Site Name | Organism | Position | Strand | Matrix score. | sequence | function |
| --- | --- | --- | --- | --- | --- | --- |
| as-1 | Arabidopsis thaliana | 1060 | - | 5 | TGACG |  |
| as-1 | Arabidopsis thaliana | 1890 | + | 5 | TGACG |  |
| as-1 | Arabidopsis thaliana | 1086 | - | 5 | TGACG |  |
| as-1 | Arabidopsis thaliana | 1938 | - | 5 | TGACG |  |

>Potri.002G039100.1   
+ TTTTTTTTTA TCCGTAATTG ATTATTCAAA ACCTTTCCAG CTTAAAATTT TTTCCATAAT TTAAAAACTA   
  
  
+ TTAAAGGTTG AAAACACTTT TTTTTTCTCT GAGATTTAAA ACTAATACTA TAAAAACTCT AACTCTAACT   
  
  
+ TTCCGTTGTT TATTATTTAT CACCGAATCT TCTTGATAAA TGTATTTCTT AAAGTATATT TTATATAAAA   
  
  
+ ATATTTTTTT TATTTTTAAA ATTTTCCTTT AATATACATC ATGTAAAGTT TCAAATCGAT TTAAAAATTA   
  
  
+ AACATAATTT TTTTAAAAAA TTAACGGAAC CGCAAAAAAA TAAAGAGATG GTCGCCATCA ACCGTGTAGC   
  
  
+ AACCCTTTCT TTTCGGAGGA CTTTCAAAAT TCAGGCATTT CCATGGAGAT TTTTTGTCTT CTTGAAGACC   
  
  
+ GGGAACGGTT TCAAGCAATC CCACGAAAGG GGGCCTGAAT CTAGACAACG TTGCTATCAA ACAGCTTCAT   
  
  
+ CTGTGTTAAA TATTTTATGG ATTTGATTTA CCTGTCCCCT CTGCCAGTCT TTTCAAATCA AATTTGCCAC   
  
  
+ AAGAAACCAT GACTTTTATT TCTTTCATGT GCACATCATT ACCTTAAGTG ATAACGATTA AAGGTGGAGG   
  
  
+ TTTGCCTAAA ATTACCAGTT GATATTAAAT ACGCAACAAC TTAATTTCAC ATGAATCCTC ACCCGTATTA   
  
  
+ GCTAGGGTGC CAAACTCTCT TGATCACCAT CCGTGATTAA GCGGTGCGTG GTTTTATTGA TTAAAACTAA   
  
  
+ TGTGATTTAA ATCTGATTAT CCCCGAGTTA ATTATGAAGG CTCGGAACTC CATTTTCTTC TCCTTTTTTT   
  
  
+ CACTTTTTTC TTGGACAGGG GACGAAAGTT GGTTCATGTG GCATCAAAAA AGTAGTGTTT AATAATTTAG   
  
  
+ ATGCTTTTAA CATTCATTGG ACAAGACTAA AAAGATCAGA TTTCATAGCA AATCTATCAC TTAGTCGACA   
  
  
+ TAAATATGTA AAAAACTCGA GATTTTATTA ATGGGTCTTG AATTTAATTA AAACCTTTAA ATTAAATAAA   
  
  
+ TATTATTCTC GTCACGAGTC AATCTCTTGT AACATCGTCA TTTCTTTAAG ATCAGTGACC TTGTTGAAAA   
  
  
+ CTAAAAACAC ACACAATTTT AATCATTCTT CTCTTTTTCG TGTATTCAAC TCACTTAAAT TCAACATATA   
  
  
+ GATTGAAAGA TTACAAGTCT AGACCTATTA TAATTCAATA AAGATTCTAA TTAGCAAGTT GAATAAGTGT   
  
  
+ TCTAAACACC AATTACTTTT TTCATCGATG ATTTCTATTC AAACTACGAT GTAAACTAAT ATCTAACAAT   
  
  
+ TGTCTTGAAT TTCGAACAAA TAATCACAGC TGCAGCTTTC AAAACATTAC CCAGAACCAT TTTCCTTGAC   
  
  
+ TTCTTTTTTA TTTTCTTATG CTTGAAATAA TACAGAACAG ATTAACGAGT TCTGTACACT GAAAATGAAA   
  
  
+ CAAGACTTTG AACTTCTAAC TTGGTATATT CTATTTTGTT ATACCGTCCC ATCATGTTAA GACTGGAAAA   
  
  
+ CTAATTAAAA GGTATTTCAT GAGATTATGA TGCATAATTA TTTTTAAAAT AATTTTTTAT TTAAAAATAT   
  
  
+ ATTAAAATAA AATTTCTTAT TTATTTTTAA TATCAACAGA TTAAAACTAT CATAAATCAC TAAATAATTA   
  
  
+ TCAATTTAAT ATTTTTCAAA CCAAAAACAC ATTTAAAATA CACTTCAAAA CTGTGTCAAC TTGAAGCTCA   
  
  
+ AAATTCACAG AGAAGTTAAA AACATTTAAC TGTAAATGCG AAGCTACACT GTCCAAACCT TCCAGTGACA   
  
  
+ ATGAAAAGCT TGCCACTTTG TATTGAAATA TTGGTACCAC GTGTAAGACA TATGCGAACA AGGCAATGGT   
  
  
+ GACGTCGGCA AGGTATAGGC AATCGTGGGA CCAATCCCGC CGCCGTACGT CACTAAACAC ACGTGCCTTA   
  
  
+ AAAACAAATT CCCAAGCCTT CCTAGCAACA AAGAACAAG  

- AAAAAAAAAT AGGCATTAAC TAATAAGTTT TGGAAAGGTC GAATTTTAAA AAAGGTATTA AATTTTTGAT   
  
  
- AATTTCCAAC TTTTGTGAAA AAAAAAGAGA CTCTAAATTT TGATTATGAT ATTTTTGAGA TTGAGATTGA   
  
  
- AAGGCAACAA ATAATAAATA GTGGCTTAGA AGAACTATTT ACATAAAGAA TTTCATATAA AATATATTTT   
  
  
- TATAAAAAAA ATAAAAATTT TAAAAGGAAA TTATATGTAG TACATTTCAA AGTTTAGCTA AATTTTTAAT   
  
  
- TTGTATTAAA AAAATTTTTT AATTGCCTTG GCGTTTTTTT ATTTCTCTAC CAGCGGTAGT TGGCACATCG   
  
  
- TTGGGAAAGA AAAGCCTCCT GAAAGTTTTA AGTCCGTAAA GGTACCTCTA AAAAACAGAA GAACTTCTGG   
  
  
- CCCTTGCCAA AGTTCGTTAG GGTGCTTTCC CCCGGACTTA GATCTGTTGC AACGATAGTT TGTCGAAGTA   
  
  
- GACACAATTT ATAAAATACC TAAACTAAAT GGACAGGGGA GACGGTCAGA AAAGTTTAGT TTAAACGGTG   
  
  
- TTCTTTGGTA CTGAAAATAA AGAAAGTACA CGTGTAGTAA TGGAATTCAC TATTGCTAAT TTCCACCTCC   
  
  
- AAACGGATTT TAATGGTCAA CTATAATTTA TGCGTTGTTG AATTAAAGTG TACTTAGGAG TGGGCATAAT   
  
  
- CGATCCCACG GTTTGAGAGA ACTAGTGGTA GGCACTAATT CGCCACGCAC CAAAATAACT AATTTTGATT   
  
  
- ACACTAAATT TAGACTAATA GGGGCTCAAT TAATACTTCC GAGCCTTGAG GTAAAAGAAG AGGAAAAAAA   
  
  
- GTGAAAAAAG AACCTGTCCC CTGCTTTCAA CCAAGTACAC CGTAGTTTTT TCATCACAAA TTATTAAATC   
  
  
- TACGAAAATT GTAAGTAACC TGTTCTGATT TTTCTAGTCT AAAGTATCGT TTAGATAGTG AATCAGCTGT   
  
  
- ATTTATACAT TTTTTGAGCT CTAAAATAAT TACCCAGAAC TTAAATTAAT TTTGGAAATT TAATTTATTT   
  
  
- ATAATAAGAG CAGTGCTCAG TTAGAGAACA TTGTAGCAGT AAAGAAATTC TAGTCACTGG AACAACTTTT   
  
  
- GATTTTTGTG TGTGTTAAAA TTAGTAAGAA GAGAAAAAGC ACATAAGTTG AGTGAATTTA AGTTGTATAT   
  
  
- CTAACTTTCT AATGTTCAGA TCTGGATAAT ATTAAGTTAT TTCTAAGATT AATCGTTCAA CTTATTCACA   
  
  
- AGATTTGTGG TTAATGAAAA AAGTAGCTAC TAAAGATAAG TTTGATGCTA CATTTGATTA TAGATTGTTA   
  
  
- ACAGAACTTA AAGCTTGTTT ATTAGTGTCG ACGTCGAAAG TTTTGTAATG GGTCTTGGTA AAAGGAACTG   
  
  
- AAGAAAAAAT AAAAGAATAC GAACTTTATT ATGTCTTGTC TAATTGCTCA AGACATGTGA CTTTTACTTT   
  
  
- GTTCTGAAAC TTGAAGATTG AACCATATAA GATAAAACAA TATGGCAGGG TAGTACAATT CTGACCTTTT   
  
  
- GATTAATTTT CCATAAAGTA CTCTAATACT ACGTATTAAT AAAAATTTTA TTAAAAAATA AATTTTTATA   
  
  
- TAATTTTATT TTAAAGAATA AATAAAAATT ATAGTTGTCT AATTTTGATA GTATTTAGTG ATTTATTAAT   
  
  
- AGTTAAATTA TAAAAAGTTT GGTTTTTGTG TAAATTTTAT GTGAAGTTTT GACACAGTTG AACTTCGAGT   
  
  
- TTTAAGTGTC TCTTCAATTT TTGTAAATTG ACATTTACGC TTCGATGTGA CAGGTTTGGA AGGTCACTGT   
  
  
- TACTTTTCGA ACGGTGAAAC ATAACTTTAT AACCATGGTG CACATTCTGT ATACGCTTGT TCCGTTACCA   
  
  
- CTGCAGCCGT TCCATATCCG TTAGCACCCT GGTTAGGGCG GCGGCATGCA GTGATTTGTG TGCACGGAAT   
  
  
- TTTTGTTTAA GGGTTCGGAA GGATCGTTGT TTCTTGTTC
