# Supplementary material for: Structure and expression analysis of seven salt-related ERF genes of Populus
Source: PeerJ. 2020 Oct 20;8:e10206. doi: 10.7717/peerj.10206 (PMC7583627; doi:10.7717/peerj.10206)
Supplement: Supplemental Information 17 [file peerj-08-10206-s017.gz › Potri.003G139300.1_plantcare.html]

Content-Type: text/html; charset=ISO-8859-1


PlantCARE


Webmaster Firefox specific output  
To save the result:
click on the frame with the right mouse button and save the source code as a text file with extension .html  
REFERENCE:PlantCARE: a database of plant cis-acting regulatory elements and a portal to tools for in silico analysis of promoter sequences.  
Lescot, M., Déhais, P., Moreau, Y., De Moor, B., Rouzé ,P.,and Rombauts, S.  
Nucleic Acids Res., Database issue(2002), 30(1):325-327.   


---

>Potri.003G139300.1   
+ CAATCAAATA TCACGTCTTG CCTACAAGCA TGGTTTTCAA CTGTCAATTT CTGCTTGAAA GTGCTTCCAT   
  
  
+ ATTAGCACGA GGACTCAGAA GGATAACGAG AATATCAAGC AACTTATGCC AGTCCTTTGT AATTAGCAGA   
  
  
+ GTCGAGCAGT TTTAAATAAC TTTTCGATCC TTCATGTTTA AACTGAGAAT GATGTATAGA ACTAGAAGAC   
  
  
+ TGTCGGCAAT TTCTTTCATT TTGTGTGTGT TTGGCAGTGT TATAGCGATT GCTTTTCAAA TAGTTTTTCG   
  
  
+ TGCCGAAATA CATGCCAATG ATGTTTTTTT ATTTTTTTAA AATCATTTTT AATATCAACA CATCAAAACG   
  
  
+ ATCCAAAAGA TACAAACCGC ACTCAATTTT AGCAAAAAAA AAAATTCAAA ATTTAACAAA ACGCAGGTTT   
  
  
+ AAACGCACAG CCAAACAGTC TGTTATGGCT GGTTTTTAAC TTTATTCTAC GACCAGATCA TAAAAAATGA   
  
  
+ TATCTTGACT AATTATGTGT CGATTAATTA AATTTACGAG TAAATATGTT AAACCAGTGT TCACGACTAT   
  
  
+ GCTTCTTGAA ACGACGTGGT ATTCATCATA TTTATTTACT TCTAGAGGTT TTTTGTATAA AAAAATAAAT   
  
  
+ AAAAAAAATG CATTATGAGA TTAGCCGGCT AGATAGCGGG ATTGAGCCAG AGCCTGAATT GCAGCTGCTT   
  
  
+ TAGATAGGGA TGGTGATGGA AAAGAAAGGG AAAATTTATC CAGCACGAAC AGAAAAGCAA AACGCCCTTG   
  
  
+ CCCGCAACAA GCCAGGACGC GTGGCGAAGG AGGCTTTAAA TGAATGCGAC AGATGTTATT ATTGAGGTTT   
  
  
+ GACTTGTTTC CCGGTTGTTA TTGTTTTTAT AAAAAAATTA ATTTGTTTTT TTTAAAAAAA GTTTACATTA   
  
  
+ TTTTAATATG TCGACGGAAA TAATAAATCT TAAAAAATAA AAAATATTAT TTTAATATAA TTTTAAATAA   
  
  
+ AAATATATTT TATATAATAT AGTTTGTGTT TTGTTTAAAT CATAAATTTA AAAATATTAG ATTAATAAAT   
  
  
+ TCACCCTATT TTTTTTATGA ATCCCAATTA GGATTTTATT TTTAAATATT AGTTAAGAAA AAATTATAAT   
  
  
+ TTATAATTTA TTAATAACCG TAACTATAAA AAACTACCGT AATACAAAAT CAAATGCAAA ATTCTCAATC   
  
  
+ TAAATTCATG TACTGTTCTG ACACATAATT AACTCCCCAT TCAGTTACTG TTTTTGTTTT TATCACTTGA   
  
  
+ GTTAATTCCT CTTTTTTTTT CTAGAAGAAA TTTTAATTTC ATTGTTTATA TAAATGGTAA AAAAACAAAA   
  
  
+ ATGAAAATCG TGAACCATCA AACATGTGCA TGCTAAATGC TGCATTTGCT ACCTATAATT GTATTTTCTT   
  
  
+ ATAATATTGT TTTGAAATAT TGAAAAAACA CTATTTCTTT TAATCTGTGT TTTTAGAGCT CTATTGATTG   
  
  
+ TTTTTTTTTT TTTTACACTG TGATGATAAA AACCAATAAC CACGCCTCAT TCTATTATTA TTATTATCAT   
  
  
+ TATCACAAGA AATTATAAAA AATTTATTGG ATTAAGGAAT GTTTAGAATT GCGTTTTAAA TTGTATTTTT   
  
  
+ AAAATTTTTG ATTTTTTATT TAAAATTAAT TTTTTAGATT ATTTTTATAT ATTGATATCT AAACTAATTT   
  
  
+ TAAAAAATTA AAAAATATAT TATTTTAATA TATTTTTAAA TAAAAAATAA TTTAAAAAAC AACTTCTATT   
  
  
+ ATACAGGATA AAATACCCAT TACCGATTTA ACATTTTGAG AAGAAAAATC TACATCCAAG GTTCTTATTC   
  
  
+ CTTGAGGACA TTATGGTCAT TACATATCCA GGACAAACCT TTGCTTGGAC TAAAATTCAA ACCAAATGGA   
  
  
+ ATTTCATAGC TGTCAAATAC AGTGAAGCTG GGGATCCATG GAAAGTCCCC TGCTTTACTC TGCAGCTATA   
  
  
+ AATACACCAA CTAGGACACA ACCATAAACC ACAGTAACC  

- GTTAGTTTAT AGTGCAGAAC GGATGTTCGT ACCAAAAGTT GACAGTTAAA GACGAACTTT CACGAAGGTA   
  
  
- TAATCGTGCT CCTGAGTCTT CCTATTGCTC TTATAGTTCG TTGAATACGG TCAGGAAACA TTAATCGTCT   
  
  
- CAGCTCGTCA AAATTTATTG AAAAGCTAGG AAGTACAAAT TTGACTCTTA CTACATATCT TGATCTTCTG   
  
  
- ACAGCCGTTA AAGAAAGTAA AACACACACA AACCGTCACA ATATCGCTAA CGAAAAGTTT ATCAAAAAGC   
  
  
- ACGGCTTTAT GTACGGTTAC TACAAAAAAA TAAAAAAATT TTAGTAAAAA TTATAGTTGT GTAGTTTTGC   
  
  
- TAGGTTTTCT ATGTTTGGCG TGAGTTAAAA TCGTTTTTTT TTTTAAGTTT TAAATTGTTT TGCGTCCAAA   
  
  
- TTTGCGTGTC GGTTTGTCAG ACAATACCGA CCAAAAATTG AAATAAGATG CTGGTCTAGT ATTTTTTACT   
  
  
- ATAGAACTGA TTAATACACA GCTAATTAAT TTAAATGCTC ATTTATACAA TTTGGTCACA AGTGCTGATA   
  
  
- CGAAGAACTT TGCTGCACCA TAAGTAGTAT AAATAAATGA AGATCTCCAA AAAACATATT TTTTTATTTA   
  
  
- TTTTTTTTAC GTAATACTCT AATCGGCCGA TCTATCGCCC TAACTCGGTC TCGGACTTAA CGTCGACGAA   
  
  
- ATCTATCCCT ACCACTACCT TTTCTTTCCC TTTTAAATAG GTCGTGCTTG TCTTTTCGTT TTGCGGGAAC   
  
  
- GGGCGTTGTT CGGTCCTGCG CACCGCTTCC TCCGAAATTT ACTTACGCTG TCTACAATAA TAACTCCAAA   
  
  
- CTGAACAAAG GGCCAACAAT AACAAAAATA TTTTTTTAAT TAAACAAAAA AAATTTTTTT CAAATGTAAT   
  
  
- AAAATTATAC AGCTGCCTTT ATTATTTAGA ATTTTTTATT TTTTATAATA AAATTATATT AAAATTTATT   
  
  
- TTTATATAAA ATATATTATA TCAAACACAA AACAAATTTA GTATTTAAAT TTTTATAATC TAATTATTTA   
  
  
- AGTGGGATAA AAAAAATACT TAGGGTTAAT CCTAAAATAA AAATTTATAA TCAATTCTTT TTTAATATTA   
  
  
- AATATTAAAT AATTATTGGC ATTGATATTT TTTGATGGCA TTATGTTTTA GTTTACGTTT TAAGAGTTAG   
  
  
- ATTTAAGTAC ATGACAAGAC TGTGTATTAA TTGAGGGGTA AGTCAATGAC AAAAACAAAA ATAGTGAACT   
  
  
- CAATTAAGGA GAAAAAAAAA GATCTTCTTT AAAATTAAAG TAACAAATAT ATTTACCATT TTTTTGTTTT   
  
  
- TACTTTTAGC ACTTGGTAGT TTGTACACGT ACGATTTACG ACGTAAACGA TGGATATTAA CATAAAAGAA   
  
  
- TATTATAACA AAACTTTATA ACTTTTTTGT GATAAAGAAA ATTAGACACA AAAATCTCGA GATAACTAAC   
  
  
- AAAAAAAAAA AAAATGTGAC ACTACTATTT TTGGTTATTG GTGCGGAGTA AGATAATAAT AATAATAGTA   
  
  
- ATAGTGTTCT TTAATATTTT TTAAATAACC TAATTCCTTA CAAATCTTAA CGCAAAATTT AACATAAAAA   
  
  
- TTTTAAAAAC TAAAAAATAA ATTTTAATTA AAAAATCTAA TAAAAATATA TAACTATAGA TTTGATTAAA   
  
  
- ATTTTTTAAT TTTTTATATA ATAAAATTAT ATAAAAATTT ATTTTTTATT AAATTTTTTG TTGAAGATAA   
  
  
- TATGTCCTAT TTTATGGGTA ATGGCTAAAT TGTAAAACTC TTCTTTTTAG ATGTAGGTTC CAAGAATAAG   
  
  
- GAACTCCTGT AATACCAGTA ATGTATAGGT CCTGTTTGGA AACGAACCTG ATTTTAAGTT TGGTTTACCT   
  
  
- TAAAGTATCG ACAGTTTATG TCACTTCGAC CCCTAGGTAC CTTTCAGGGG ACGAAATGAG ACGTCGATAT   
  
  
- TTATGTGGTT GATCCTGTGT TGGTATTTGG TGTCATTGG

  
  
Motifs Found  

+   

| Site Name | Organism | Position | Strand | Matrix score. | sequence | function |
| --- | --- | --- | --- | --- | --- | --- |
|  | organism | 1948 | + | 4 | motif\_sequence | short\_function |
|  | organism | 1826 | - | 4 | motif\_sequence | short\_function |
|  | organism | 1798 | + | 4 | motif\_sequence | short\_function |
|  | organism | 1198 | + | 4 | motif\_sequence | short\_function |
|  | organism | 1354 | + | 4 | motif\_sequence | short\_function |
|  | organism | 747 | - | 4 | motif\_sequence | short\_function |
|  | organism | 425 | - | 4 | motif\_sequence | short\_function |
|  | organism | 1203 | + | 4 | motif\_sequence | short\_function |
|  | organism | 136 | - | 4 | motif\_sequence | short\_function |

>Potri.003G139300.1   
+ CAATCAAATA TCACGTCTTG CCTACAAGCA TGGTTTTCAA CTGTCAATTT CTGCTTGAAA GTGCTTCCAT   
  
  
+ ATTAGCACGA GGACTCAGAA GGATAACGAG AATATCAAGC AACTTATGCC AGTCCTTTGT AATTAGCAGA   
  
  
+ GTCGAGCAGT TTTAAATAAC TTTTCGATCC TTCATGTTTA AACTGAGAAT GATGTATAGA ACTAGAAGAC   
  
  
+ TGTCGGCAAT TTCTTTCATT TTGTGTGTGT TTGGCAGTGT TATAGCGATT GCTTTTCAAA TAGTTTTTCG   
  
  
+ TGCCGAAATA CATGCCAATG ATGTTTTTTT ATTTTTTTAA AATCATTTTT AATATCAACA CATCAAAACG   
  
  
+ ATCCAAAAGA TACAAACCGC ACTCAATTTT AGCAAAAAAA AAAATTCAAA ATTTAACAAA ACGCAGGTTT   
  
  
+ AAACGCACAG CCAAACAGTC TGTTATGGCT GGTTTTTAAC TTTATTCTAC GACCAGATCA TAAAAAATGA   
  
  
+ TATCTTGACT AATTATGTGT CGATTAATTA AATTTACGAG TAAATATGTT AAACCAGTGT TCACGACTAT   
  
  
+ GCTTCTTGAA ACGACGTGGT ATTCATCATA TTTATTTACT TCTAGAGGTT TTTTGTATAA AAAAATAAAT   
  
  
+ AAAAAAAATG CATTATGAGA TTAGCCGGCT AGATAGCGGG ATTGAGCCAG AGCCTGAATT GCAGCTGCTT   
  
  
+ TAGATAGGGA TGGTGATGGA AAAGAAAGGG AAAATTTATC CAGCACGAAC AGAAAAGCAA AACGCCCTTG   
  
  
+ CCCGCAACAA GCCAGGACGC GTGGCGAAGG AGGCTTTAAA TGAATGCGAC AGATGTTATT ATTGAGGTTT   
  
  
+ GACTTGTTTC CCGGTTGTTA TTGTTTTTAT AAAAAAATTA ATTTGTTTTT TTTAAAAAAA GTTTACATTA   
  
  
+ TTTTAATATG TCGACGGAAA TAATAAATCT TAAAAAATAA AAAATATTAT TTTAATATAA TTTTAAATAA   
  
  
+ AAATATATTT TATATAATAT AGTTTGTGTT TTGTTTAAAT CATAAATTTA AAAATATTAG ATTAATAAAT   
  
  
+ TCACCCTATT TTTTTTATGA ATCCCAATTA GGATTTTATT TTTAAATATT AGTTAAGAAA AAATTATAAT   
  
  
+ TTATAATTTA TTAATAACCG TAACTATAAA AAACTACCGT AATACAAAAT CAAATGCAAA ATTCTCAATC   
  
  
+ TAAATTCATG TACTGTTCTG ACACATAATT AACTCCCCAT TCAGTTACTG TTTTTGTTTT TATCACTTGA   
  
  
+ GTTAATTCCT CTTTTTTTTT CTAGAAGAAA TTTTAATTTC ATTGTTTATA TAAATGGTAA AAAAACAAAA   
  
  
+ ATGAAAATCG TGAACCATCA AACATGTGCA TGCTAAATGC TGCATTTGCT ACCTATAATT GTATTTTCTT   
  
  
+ ATAATATTGT TTTGAAATAT TGAAAAAACA CTATTTCTTT TAATCTGTGT TTTTAGAGCT CTATTGATTG   
  
  
+ TTTTTTTTTT TTTTACACTG TGATGATAAA AACCAATAAC CACGCCTCAT TCTATTATTA TTATTATCAT   
  
  
+ TATCACAAGA AATTATAAAA AATTTATTGG ATTAAGGAAT GTTTAGAATT GCGTTTTAAA TTGTATTTTT   
  
  
+ AAAATTTTTG ATTTTTTATT TAAAATTAAT TTTTTAGATT ATTTTTATAT ATTGATATCT AAACTAATTT   
  
  
+ TAAAAAATTA AAAAATATAT TATTTTAATA TATTTTTAAA TAAAAAATAA TTTAAAAAAC AACTTCTATT   
  
  
+ ATACAGGATA AAATACCCAT TACCGATTTA ACATTTTGAG AAGAAAAATC TACATCCAAG GTTCTTATTC   
  
  
+ CTTGAGGACA TTATGGTCAT TACATATCCA GGACAAACCT TTGCTTGGAC TAAAATTCAA ACCAAATGGA   
  
  
+ ATTTCATAGC TGTCAAATAC AGTGAAGCTG GGGATCCATG GAAAGTCCCC TGCTTTACTC TGCAGCTATA   
  
  
+ AATACACCAA CTAGGACACA ACCATAAACC ACAGTAACC  

- GTTAGTTTAT AGTGCAGAAC GGATGTTCGT ACCAAAAGTT GACAGTTAAA GACGAACTTT CACGAAGGTA   
  
  
- TAATCGTGCT CCTGAGTCTT CCTATTGCTC TTATAGTTCG TTGAATACGG TCAGGAAACA TTAATCGTCT   
  
  
- CAGCTCGTCA AAATTTATTG AAAAGCTAGG AAGTACAAAT TTGACTCTTA CTACATATCT TGATCTTCTG   
  
  
- ACAGCCGTTA AAGAAAGTAA AACACACACA AACCGTCACA ATATCGCTAA CGAAAAGTTT ATCAAAAAGC   
  
  
- ACGGCTTTAT GTACGGTTAC TACAAAAAAA TAAAAAAATT TTAGTAAAAA TTATAGTTGT GTAGTTTTGC   
  
  
- TAGGTTTTCT ATGTTTGGCG TGAGTTAAAA TCGTTTTTTT TTTTAAGTTT TAAATTGTTT TGCGTCCAAA   
  
  
- TTTGCGTGTC GGTTTGTCAG ACAATACCGA CCAAAAATTG AAATAAGATG CTGGTCTAGT ATTTTTTACT   
  
  
- ATAGAACTGA TTAATACACA GCTAATTAAT TTAAATGCTC ATTTATACAA TTTGGTCACA AGTGCTGATA   
  
  
- CGAAGAACTT TGCTGCACCA TAAGTAGTAT AAATAAATGA AGATCTCCAA AAAACATATT TTTTTATTTA   
  
  
- TTTTTTTTAC GTAATACTCT AATCGGCCGA TCTATCGCCC TAACTCGGTC TCGGACTTAA CGTCGACGAA   
  
  
- ATCTATCCCT ACCACTACCT TTTCTTTCCC TTTTAAATAG GTCGTGCTTG TCTTTTCGTT TTGCGGGAAC   
  
  
- GGGCGTTGTT CGGTCCTGCG CACCGCTTCC TCCGAAATTT ACTTACGCTG TCTACAATAA TAACTCCAAA   
  
  
- CTGAACAAAG GGCCAACAAT AACAAAAATA TTTTTTTAAT TAAACAAAAA AAATTTTTTT CAAATGTAAT   
  
  
- AAAATTATAC AGCTGCCTTT ATTATTTAGA ATTTTTTATT TTTTATAATA AAATTATATT AAAATTTATT   
  
  
- TTTATATAAA ATATATTATA TCAAACACAA AACAAATTTA GTATTTAAAT TTTTATAATC TAATTATTTA   
  
  
- AGTGGGATAA AAAAAATACT TAGGGTTAAT CCTAAAATAA AAATTTATAA TCAATTCTTT TTTAATATTA   
  
  
- AATATTAAAT AATTATTGGC ATTGATATTT TTTGATGGCA TTATGTTTTA GTTTACGTTT TAAGAGTTAG   
  
  
- ATTTAAGTAC ATGACAAGAC TGTGTATTAA TTGAGGGGTA AGTCAATGAC AAAAACAAAA ATAGTGAACT   
  
  
- CAATTAAGGA GAAAAAAAAA GATCTTCTTT AAAATTAAAG TAACAAATAT ATTTACCATT TTTTTGTTTT   
  
  
- TACTTTTAGC ACTTGGTAGT TTGTACACGT ACGATTTACG ACGTAAACGA TGGATATTAA CATAAAAGAA   
  
  
- TATTATAACA AAACTTTATA ACTTTTTTGT GATAAAGAAA ATTAGACACA AAAATCTCGA GATAACTAAC   
  
  
- AAAAAAAAAA AAAATGTGAC ACTACTATTT TTGGTTATTG GTGCGGAGTA AGATAATAAT AATAATAGTA   
  
  
- ATAGTGTTCT TTAATATTTT TTAAATAACC TAATTCCTTA CAAATCTTAA CGCAAAATTT AACATAAAAA   
  
  
- TTTTAAAAAC TAAAAAATAA ATTTTAATTA AAAAATCTAA TAAAAATATA TAACTATAGA TTTGATTAAA   
  
  
- ATTTTTTAAT TTTTTATATA ATAAAATTAT ATAAAAATTT ATTTTTTATT AAATTTTTTG TTGAAGATAA   
  
  
- TATGTCCTAT TTTATGGGTA ATGGCTAAAT TGTAAAACTC TTCTTTTTAG ATGTAGGTTC CAAGAATAAG   
  
  
- GAACTCCTGT AATACCAGTA ATGTATAGGT CCTGTTTGGA AACGAACCTG ATTTTAAGTT TGGTTTACCT   
  
  
- TAAAGTATCG ACAGTTTATG TCACTTCGAC CCCTAGGTAC CTTTCAGGGG ACGAAATGAG ACGTCGATAT   
  
  
- TTATGTGGTT GATCCTGTGT TGGTATTTGG TGTCATTGG

+     AAGAA-motif

| Site Name | Organism | Position | Strand | Matrix score. | sequence | function |
| --- | --- | --- | --- | --- | --- | --- |
| AAGAA-motif | Avena sativa | 1316 | + | 9 | gGTAAAGAAA |  |
| AAGAA-motif | Avena sativa | 718 | + | 9 | gGTAAAGAAA |  |
| AAGAA-motif | Avena sativa | 221 | - | 7 | GAAAGAA |  |

>Potri.003G139300.1   
+ CAATCAAATA TCACGTCTTG CCTACAAGCA TGGTTTTCAA CTGTCAATTT CTGCTTGAAA GTGCTTCCAT   
  
  
+ ATTAGCACGA GGACTCAGAA GGATAACGAG AATATCAAGC AACTTATGCC AGTCCTTTGT AATTAGCAGA   
  
  
+ GTCGAGCAGT TTTAAATAAC TTTTCGATCC TTCATGTTTA AACTGAGAAT GATGTATAGA ACTAGAAGAC   
  
  
+ TGTCGGCAAT TTCTTTCATT TTGTGTGTGT TTGGCAGTGT TATAGCGATT GCTTTTCAAA TAGTTTTTCG   
  
  
+ TGCCGAAATA CATGCCAATG ATGTTTTTTT ATTTTTTTAA AATCATTTTT AATATCAACA CATCAAAACG   
  
  
+ ATCCAAAAGA TACAAACCGC ACTCAATTTT AGCAAAAAAA AAAATTCAAA ATTTAACAAA ACGCAGGTTT   
  
  
+ AAACGCACAG CCAAACAGTC TGTTATGGCT GGTTTTTAAC TTTATTCTAC GACCAGATCA TAAAAAATGA   
  
  
+ TATCTTGACT AATTATGTGT CGATTAATTA AATTTACGAG TAAATATGTT AAACCAGTGT TCACGACTAT   
  
  
+ GCTTCTTGAA ACGACGTGGT ATTCATCATA TTTATTTACT TCTAGAGGTT TTTTGTATAA AAAAATAAAT   
  
  
+ AAAAAAAATG CATTATGAGA TTAGCCGGCT AGATAGCGGG ATTGAGCCAG AGCCTGAATT GCAGCTGCTT   
  
  
+ TAGATAGGGA TGGTGATGGA AAAGAAAGGG AAAATTTATC CAGCACGAAC AGAAAAGCAA AACGCCCTTG   
  
  
+ CCCGCAACAA GCCAGGACGC GTGGCGAAGG AGGCTTTAAA TGAATGCGAC AGATGTTATT ATTGAGGTTT   
  
  
+ GACTTGTTTC CCGGTTGTTA TTGTTTTTAT AAAAAAATTA ATTTGTTTTT TTTAAAAAAA GTTTACATTA   
  
  
+ TTTTAATATG TCGACGGAAA TAATAAATCT TAAAAAATAA AAAATATTAT TTTAATATAA TTTTAAATAA   
  
  
+ AAATATATTT TATATAATAT AGTTTGTGTT TTGTTTAAAT CATAAATTTA AAAATATTAG ATTAATAAAT   
  
  
+ TCACCCTATT TTTTTTATGA ATCCCAATTA GGATTTTATT TTTAAATATT AGTTAAGAAA AAATTATAAT   
  
  
+ TTATAATTTA TTAATAACCG TAACTATAAA AAACTACCGT AATACAAAAT CAAATGCAAA ATTCTCAATC   
  
  
+ TAAATTCATG TACTGTTCTG ACACATAATT AACTCCCCAT TCAGTTACTG TTTTTGTTTT TATCACTTGA   
  
  
+ GTTAATTCCT CTTTTTTTTT CTAGAAGAAA TTTTAATTTC ATTGTTTATA TAAATGGTAA AAAAACAAAA   
  
  
+ ATGAAAATCG TGAACCATCA AACATGTGCA TGCTAAATGC TGCATTTGCT ACCTATAATT GTATTTTCTT   
  
  
+ ATAATATTGT TTTGAAATAT TGAAAAAACA CTATTTCTTT TAATCTGTGT TTTTAGAGCT CTATTGATTG   
  
  
+ TTTTTTTTTT TTTTACACTG TGATGATAAA AACCAATAAC CACGCCTCAT TCTATTATTA TTATTATCAT   
  
  
+ TATCACAAGA AATTATAAAA AATTTATTGG ATTAAGGAAT GTTTAGAATT GCGTTTTAAA TTGTATTTTT   
  
  
+ AAAATTTTTG ATTTTTTATT TAAAATTAAT TTTTTAGATT ATTTTTATAT ATTGATATCT AAACTAATTT   
  
  
+ TAAAAAATTA AAAAATATAT TATTTTAATA TATTTTTAAA TAAAAAATAA TTTAAAAAAC AACTTCTATT   
  
  
+ ATACAGGATA AAATACCCAT TACCGATTTA ACATTTTGAG AAGAAAAATC TACATCCAAG GTTCTTATTC   
  
  
+ CTTGAGGACA TTATGGTCAT TACATATCCA GGACAAACCT TTGCTTGGAC TAAAATTCAA ACCAAATGGA   
  
  
+ ATTTCATAGC TGTCAAATAC AGTGAAGCTG GGGATCCATG GAAAGTCCCC TGCTTTACTC TGCAGCTATA   
  
  
+ AATACACCAA CTAGGACACA ACCATAAACC ACAGTAACC  

- GTTAGTTTAT AGTGCAGAAC GGATGTTCGT ACCAAAAGTT GACAGTTAAA GACGAACTTT CACGAAGGTA   
  
  
- TAATCGTGCT CCTGAGTCTT CCTATTGCTC TTATAGTTCG TTGAATACGG TCAGGAAACA TTAATCGTCT   
  
  
- CAGCTCGTCA AAATTTATTG AAAAGCTAGG AAGTACAAAT TTGACTCTTA CTACATATCT TGATCTTCTG   
  
  
- ACAGCCGTTA AAGAAAGTAA AACACACACA AACCGTCACA ATATCGCTAA CGAAAAGTTT ATCAAAAAGC   
  
  
- ACGGCTTTAT GTACGGTTAC TACAAAAAAA TAAAAAAATT TTAGTAAAAA TTATAGTTGT GTAGTTTTGC   
  
  
- TAGGTTTTCT ATGTTTGGCG TGAGTTAAAA TCGTTTTTTT TTTTAAGTTT TAAATTGTTT TGCGTCCAAA   
  
  
- TTTGCGTGTC GGTTTGTCAG ACAATACCGA CCAAAAATTG AAATAAGATG CTGGTCTAGT ATTTTTTACT   
  
  
- ATAGAACTGA TTAATACACA GCTAATTAAT TTAAATGCTC ATTTATACAA TTTGGTCACA AGTGCTGATA   
  
  
- CGAAGAACTT TGCTGCACCA TAAGTAGTAT AAATAAATGA AGATCTCCAA AAAACATATT TTTTTATTTA   
  
  
- TTTTTTTTAC GTAATACTCT AATCGGCCGA TCTATCGCCC TAACTCGGTC TCGGACTTAA CGTCGACGAA   
  
  
- ATCTATCCCT ACCACTACCT TTTCTTTCCC TTTTAAATAG GTCGTGCTTG TCTTTTCGTT TTGCGGGAAC   
  
  
- GGGCGTTGTT CGGTCCTGCG CACCGCTTCC TCCGAAATTT ACTTACGCTG TCTACAATAA TAACTCCAAA   
  
  
- CTGAACAAAG GGCCAACAAT AACAAAAATA TTTTTTTAAT TAAACAAAAA AAATTTTTTT CAAATGTAAT   
  
  
- AAAATTATAC AGCTGCCTTT ATTATTTAGA ATTTTTTATT TTTTATAATA AAATTATATT AAAATTTATT   
  
  
- TTTATATAAA ATATATTATA TCAAACACAA AACAAATTTA GTATTTAAAT TTTTATAATC TAATTATTTA   
  
  
- AGTGGGATAA AAAAAATACT TAGGGTTAAT CCTAAAATAA AAATTTATAA TCAATTCTTT TTTAATATTA   
  
  
- AATATTAAAT AATTATTGGC ATTGATATTT TTTGATGGCA TTATGTTTTA GTTTACGTTT TAAGAGTTAG   
  
  
- ATTTAAGTAC ATGACAAGAC TGTGTATTAA TTGAGGGGTA AGTCAATGAC AAAAACAAAA ATAGTGAACT   
  
  
- CAATTAAGGA GAAAAAAAAA GATCTTCTTT AAAATTAAAG TAACAAATAT ATTTACCATT TTTTTGTTTT   
  
  
- TACTTTTAGC ACTTGGTAGT TTGTACACGT ACGATTTACG ACGTAAACGA TGGATATTAA CATAAAAGAA   
  
  
- TATTATAACA AAACTTTATA ACTTTTTTGT GATAAAGAAA ATTAGACACA AAAATCTCGA GATAACTAAC   
  
  
- AAAAAAAAAA AAAATGTGAC ACTACTATTT TTGGTTATTG GTGCGGAGTA AGATAATAAT AATAATAGTA   
  
  
- ATAGTGTTCT TTAATATTTT TTAAATAACC TAATTCCTTA CAAATCTTAA CGCAAAATTT AACATAAAAA   
  
  
- TTTTAAAAAC TAAAAAATAA ATTTTAATTA AAAAATCTAA TAAAAATATA TAACTATAGA TTTGATTAAA   
  
  
- ATTTTTTAAT TTTTTATATA ATAAAATTAT ATAAAAATTT ATTTTTTATT AAATTTTTTG TTGAAGATAA   
  
  
- TATGTCCTAT TTTATGGGTA ATGGCTAAAT TGTAAAACTC TTCTTTTTAG ATGTAGGTTC CAAGAATAAG   
  
  
- GAACTCCTGT AATACCAGTA ATGTATAGGT CCTGTTTGGA AACGAACCTG ATTTTAAGTT TGGTTTACCT   
  
  
- TAAAGTATCG ACAGTTTATG TCACTTCGAC CCCTAGGTAC CTTTCAGGGG ACGAAATGAG ACGTCGATAT   
  
  
- TTATGTGGTT GATCCTGTGT TGGTATTTGG TGTCATTGG

+     ABRE

| Site Name | Organism | Position | Strand | Matrix score. | sequence | function |
| --- | --- | --- | --- | --- | --- | --- |
| ABRE | Arabidopsis thaliana | 574 | + | 5 | ACGTG | cis-acting element involved in the abscisic acid responsiveness |
| ABRE | Arabidopsis thaliana | 12 | - | 5 | ACGTG | cis-acting element involved in the abscisic acid responsiveness |
| ABRE | Oryza sativa | 786 | + | 9 | GCCGCGTGGC | cis-acting element involved in the abscisic acid responsiveness |

>Potri.003G139300.1   
+ CAATCAAATA TCACGTCTTG CCTACAAGCA TGGTTTTCAA CTGTCAATTT CTGCTTGAAA GTGCTTCCAT   
  
  
+ ATTAGCACGA GGACTCAGAA GGATAACGAG AATATCAAGC AACTTATGCC AGTCCTTTGT AATTAGCAGA   
  
  
+ GTCGAGCAGT TTTAAATAAC TTTTCGATCC TTCATGTTTA AACTGAGAAT GATGTATAGA ACTAGAAGAC   
  
  
+ TGTCGGCAAT TTCTTTCATT TTGTGTGTGT TTGGCAGTGT TATAGCGATT GCTTTTCAAA TAGTTTTTCG   
  
  
+ TGCCGAAATA CATGCCAATG ATGTTTTTTT ATTTTTTTAA AATCATTTTT AATATCAACA CATCAAAACG   
  
  
+ ATCCAAAAGA TACAAACCGC ACTCAATTTT AGCAAAAAAA AAAATTCAAA ATTTAACAAA ACGCAGGTTT   
  
  
+ AAACGCACAG CCAAACAGTC TGTTATGGCT GGTTTTTAAC TTTATTCTAC GACCAGATCA TAAAAAATGA   
  
  
+ TATCTTGACT AATTATGTGT CGATTAATTA AATTTACGAG TAAATATGTT AAACCAGTGT TCACGACTAT   
  
  
+ GCTTCTTGAA ACGACGTGGT ATTCATCATA TTTATTTACT TCTAGAGGTT TTTTGTATAA AAAAATAAAT   
  
  
+ AAAAAAAATG CATTATGAGA TTAGCCGGCT AGATAGCGGG ATTGAGCCAG AGCCTGAATT GCAGCTGCTT   
  
  
+ TAGATAGGGA TGGTGATGGA AAAGAAAGGG AAAATTTATC CAGCACGAAC AGAAAAGCAA AACGCCCTTG   
  
  
+ CCCGCAACAA GCCAGGACGC GTGGCGAAGG AGGCTTTAAA TGAATGCGAC AGATGTTATT ATTGAGGTTT   
  
  
+ GACTTGTTTC CCGGTTGTTA TTGTTTTTAT AAAAAAATTA ATTTGTTTTT TTTAAAAAAA GTTTACATTA   
  
  
+ TTTTAATATG TCGACGGAAA TAATAAATCT TAAAAAATAA AAAATATTAT TTTAATATAA TTTTAAATAA   
  
  
+ AAATATATTT TATATAATAT AGTTTGTGTT TTGTTTAAAT CATAAATTTA AAAATATTAG ATTAATAAAT   
  
  
+ TCACCCTATT TTTTTTATGA ATCCCAATTA GGATTTTATT TTTAAATATT AGTTAAGAAA AAATTATAAT   
  
  
+ TTATAATTTA TTAATAACCG TAACTATAAA AAACTACCGT AATACAAAAT CAAATGCAAA ATTCTCAATC   
  
  
+ TAAATTCATG TACTGTTCTG ACACATAATT AACTCCCCAT TCAGTTACTG TTTTTGTTTT TATCACTTGA   
  
  
+ GTTAATTCCT CTTTTTTTTT CTAGAAGAAA TTTTAATTTC ATTGTTTATA TAAATGGTAA AAAAACAAAA   
  
  
+ ATGAAAATCG TGAACCATCA AACATGTGCA TGCTAAATGC TGCATTTGCT ACCTATAATT GTATTTTCTT   
  
  
+ ATAATATTGT TTTGAAATAT TGAAAAAACA CTATTTCTTT TAATCTGTGT TTTTAGAGCT CTATTGATTG   
  
  
+ TTTTTTTTTT TTTTACACTG TGATGATAAA AACCAATAAC CACGCCTCAT TCTATTATTA TTATTATCAT   
  
  
+ TATCACAAGA AATTATAAAA AATTTATTGG ATTAAGGAAT GTTTAGAATT GCGTTTTAAA TTGTATTTTT   
  
  
+ AAAATTTTTG ATTTTTTATT TAAAATTAAT TTTTTAGATT ATTTTTATAT ATTGATATCT AAACTAATTT   
  
  
+ TAAAAAATTA AAAAATATAT TATTTTAATA TATTTTTAAA TAAAAAATAA TTTAAAAAAC AACTTCTATT   
  
  
+ ATACAGGATA AAATACCCAT TACCGATTTA ACATTTTGAG AAGAAAAATC TACATCCAAG GTTCTTATTC   
  
  
+ CTTGAGGACA TTATGGTCAT TACATATCCA GGACAAACCT TTGCTTGGAC TAAAATTCAA ACCAAATGGA   
  
  
+ ATTTCATAGC TGTCAAATAC AGTGAAGCTG GGGATCCATG GAAAGTCCCC TGCTTTACTC TGCAGCTATA   
  
  
+ AATACACCAA CTAGGACACA ACCATAAACC ACAGTAACC  

- GTTAGTTTAT AGTGCAGAAC GGATGTTCGT ACCAAAAGTT GACAGTTAAA GACGAACTTT CACGAAGGTA   
  
  
- TAATCGTGCT CCTGAGTCTT CCTATTGCTC TTATAGTTCG TTGAATACGG TCAGGAAACA TTAATCGTCT   
  
  
- CAGCTCGTCA AAATTTATTG AAAAGCTAGG AAGTACAAAT TTGACTCTTA CTACATATCT TGATCTTCTG   
  
  
- ACAGCCGTTA AAGAAAGTAA AACACACACA AACCGTCACA ATATCGCTAA CGAAAAGTTT ATCAAAAAGC   
  
  
- ACGGCTTTAT GTACGGTTAC TACAAAAAAA TAAAAAAATT TTAGTAAAAA TTATAGTTGT GTAGTTTTGC   
  
  
- TAGGTTTTCT ATGTTTGGCG TGAGTTAAAA TCGTTTTTTT TTTTAAGTTT TAAATTGTTT TGCGTCCAAA   
  
  
- TTTGCGTGTC GGTTTGTCAG ACAATACCGA CCAAAAATTG AAATAAGATG CTGGTCTAGT ATTTTTTACT   
  
  
- ATAGAACTGA TTAATACACA GCTAATTAAT TTAAATGCTC ATTTATACAA TTTGGTCACA AGTGCTGATA   
  
  
- CGAAGAACTT TGCTGCACCA TAAGTAGTAT AAATAAATGA AGATCTCCAA AAAACATATT TTTTTATTTA   
  
  
- TTTTTTTTAC GTAATACTCT AATCGGCCGA TCTATCGCCC TAACTCGGTC TCGGACTTAA CGTCGACGAA   
  
  
- ATCTATCCCT ACCACTACCT TTTCTTTCCC TTTTAAATAG GTCGTGCTTG TCTTTTCGTT TTGCGGGAAC   
  
  
- GGGCGTTGTT CGGTCCTGCG CACCGCTTCC TCCGAAATTT ACTTACGCTG TCTACAATAA TAACTCCAAA   
  
  
- CTGAACAAAG GGCCAACAAT AACAAAAATA TTTTTTTAAT TAAACAAAAA AAATTTTTTT CAAATGTAAT   
  
  
- AAAATTATAC AGCTGCCTTT ATTATTTAGA ATTTTTTATT TTTTATAATA AAATTATATT AAAATTTATT   
  
  
- TTTATATAAA ATATATTATA TCAAACACAA AACAAATTTA GTATTTAAAT TTTTATAATC TAATTATTTA   
  
  
- AGTGGGATAA AAAAAATACT TAGGGTTAAT CCTAAAATAA AAATTTATAA TCAATTCTTT TTTAATATTA   
  
  
- AATATTAAAT AATTATTGGC ATTGATATTT TTTGATGGCA TTATGTTTTA GTTTACGTTT TAAGAGTTAG   
  
  
- ATTTAAGTAC ATGACAAGAC TGTGTATTAA TTGAGGGGTA AGTCAATGAC AAAAACAAAA ATAGTGAACT   
  
  
- CAATTAAGGA GAAAAAAAAA GATCTTCTTT AAAATTAAAG TAACAAATAT ATTTACCATT TTTTTGTTTT   
  
  
- TACTTTTAGC ACTTGGTAGT TTGTACACGT ACGATTTACG ACGTAAACGA TGGATATTAA CATAAAAGAA   
  
  
- TATTATAACA AAACTTTATA ACTTTTTTGT GATAAAGAAA ATTAGACACA AAAATCTCGA GATAACTAAC   
  
  
- AAAAAAAAAA AAAATGTGAC ACTACTATTT TTGGTTATTG GTGCGGAGTA AGATAATAAT AATAATAGTA   
  
  
- ATAGTGTTCT TTAATATTTT TTAAATAACC TAATTCCTTA CAAATCTTAA CGCAAAATTT AACATAAAAA   
  
  
- TTTTAAAAAC TAAAAAATAA ATTTTAATTA AAAAATCTAA TAAAAATATA TAACTATAGA TTTGATTAAA   
  
  
- ATTTTTTAAT TTTTTATATA ATAAAATTAT ATAAAAATTT ATTTTTTATT AAATTTTTTG TTGAAGATAA   
  
  
- TATGTCCTAT TTTATGGGTA ATGGCTAAAT TGTAAAACTC TTCTTTTTAG ATGTAGGTTC CAAGAATAAG   
  
  
- GAACTCCTGT AATACCAGTA ATGTATAGGT CCTGTTTGGA AACGAACCTG ATTTTAAGTT TGGTTTACCT   
  
  
- TAAAGTATCG ACAGTTTATG TCACTTCGAC CCCTAGGTAC CTTTCAGGGG ACGAAATGAG ACGTCGATAT   
  
  
- TTATGTGGTT GATCCTGTGT TGGTATTTGG TGTCATTGG

+     ARE

| Site Name | Organism | Position | Strand | Matrix score. | sequence | function |
| --- | --- | --- | --- | --- | --- | --- |
| ARE | Zea mays | 1879 | + | 6 | AAACCA | cis-acting regulatory element essential for the anaerobic induction |
| ARE | Zea mays | 1986 | + | 6 | AAACCA | cis-acting regulatory element essential for the anaerobic induction |
| ARE | Zea mays | 31 | - | 6 | AAACCA | cis-acting regulatory element essential for the anaerobic induction |
| ARE | Zea mays | 541 | + | 6 | AAACCA | cis-acting regulatory element essential for the anaerobic induction |
| ARE | Zea mays | 450 | - | 6 | AAACCA | cis-acting regulatory element essential for the anaerobic induction |
| ARE | Zea mays | 1500 | + | 6 | AAACCA | cis-acting regulatory element essential for the anaerobic induction |

>Potri.003G139300.1   
+ CAATCAAATA TCACGTCTTG CCTACAAGCA TGGTTTTCAA CTGTCAATTT CTGCTTGAAA GTGCTTCCAT   
  
  
+ ATTAGCACGA GGACTCAGAA GGATAACGAG AATATCAAGC AACTTATGCC AGTCCTTTGT AATTAGCAGA   
  
  
+ GTCGAGCAGT TTTAAATAAC TTTTCGATCC TTCATGTTTA AACTGAGAAT GATGTATAGA ACTAGAAGAC   
  
  
+ TGTCGGCAAT TTCTTTCATT TTGTGTGTGT TTGGCAGTGT TATAGCGATT GCTTTTCAAA TAGTTTTTCG   
  
  
+ TGCCGAAATA CATGCCAATG ATGTTTTTTT ATTTTTTTAA AATCATTTTT AATATCAACA CATCAAAACG   
  
  
+ ATCCAAAAGA TACAAACCGC ACTCAATTTT AGCAAAAAAA AAAATTCAAA ATTTAACAAA ACGCAGGTTT   
  
  
+ AAACGCACAG CCAAACAGTC TGTTATGGCT GGTTTTTAAC TTTATTCTAC GACCAGATCA TAAAAAATGA   
  
  
+ TATCTTGACT AATTATGTGT CGATTAATTA AATTTACGAG TAAATATGTT AAACCAGTGT TCACGACTAT   
  
  
+ GCTTCTTGAA ACGACGTGGT ATTCATCATA TTTATTTACT TCTAGAGGTT TTTTGTATAA AAAAATAAAT   
  
  
+ AAAAAAAATG CATTATGAGA TTAGCCGGCT AGATAGCGGG ATTGAGCCAG AGCCTGAATT GCAGCTGCTT   
  
  
+ TAGATAGGGA TGGTGATGGA AAAGAAAGGG AAAATTTATC CAGCACGAAC AGAAAAGCAA AACGCCCTTG   
  
  
+ CCCGCAACAA GCCAGGACGC GTGGCGAAGG AGGCTTTAAA TGAATGCGAC AGATGTTATT ATTGAGGTTT   
  
  
+ GACTTGTTTC CCGGTTGTTA TTGTTTTTAT AAAAAAATTA ATTTGTTTTT TTTAAAAAAA GTTTACATTA   
  
  
+ TTTTAATATG TCGACGGAAA TAATAAATCT TAAAAAATAA AAAATATTAT TTTAATATAA TTTTAAATAA   
  
  
+ AAATATATTT TATATAATAT AGTTTGTGTT TTGTTTAAAT CATAAATTTA AAAATATTAG ATTAATAAAT   
  
  
+ TCACCCTATT TTTTTTATGA ATCCCAATTA GGATTTTATT TTTAAATATT AGTTAAGAAA AAATTATAAT   
  
  
+ TTATAATTTA TTAATAACCG TAACTATAAA AAACTACCGT AATACAAAAT CAAATGCAAA ATTCTCAATC   
  
  
+ TAAATTCATG TACTGTTCTG ACACATAATT AACTCCCCAT TCAGTTACTG TTTTTGTTTT TATCACTTGA   
  
  
+ GTTAATTCCT CTTTTTTTTT CTAGAAGAAA TTTTAATTTC ATTGTTTATA TAAATGGTAA AAAAACAAAA   
  
  
+ ATGAAAATCG TGAACCATCA AACATGTGCA TGCTAAATGC TGCATTTGCT ACCTATAATT GTATTTTCTT   
  
  
+ ATAATATTGT TTTGAAATAT TGAAAAAACA CTATTTCTTT TAATCTGTGT TTTTAGAGCT CTATTGATTG   
  
  
+ TTTTTTTTTT TTTTACACTG TGATGATAAA AACCAATAAC CACGCCTCAT TCTATTATTA TTATTATCAT   
  
  
+ TATCACAAGA AATTATAAAA AATTTATTGG ATTAAGGAAT GTTTAGAATT GCGTTTTAAA TTGTATTTTT   
  
  
+ AAAATTTTTG ATTTTTTATT TAAAATTAAT TTTTTAGATT ATTTTTATAT ATTGATATCT AAACTAATTT   
  
  
+ TAAAAAATTA AAAAATATAT TATTTTAATA TATTTTTAAA TAAAAAATAA TTTAAAAAAC AACTTCTATT   
  
  
+ ATACAGGATA AAATACCCAT TACCGATTTA ACATTTTGAG AAGAAAAATC TACATCCAAG GTTCTTATTC   
  
  
+ CTTGAGGACA TTATGGTCAT TACATATCCA GGACAAACCT TTGCTTGGAC TAAAATTCAA ACCAAATGGA   
  
  
+ ATTTCATAGC TGTCAAATAC AGTGAAGCTG GGGATCCATG GAAAGTCCCC TGCTTTACTC TGCAGCTATA   
  
  
+ AATACACCAA CTAGGACACA ACCATAAACC ACAGTAACC  

- GTTAGTTTAT AGTGCAGAAC GGATGTTCGT ACCAAAAGTT GACAGTTAAA GACGAACTTT CACGAAGGTA   
  
  
- TAATCGTGCT CCTGAGTCTT CCTATTGCTC TTATAGTTCG TTGAATACGG TCAGGAAACA TTAATCGTCT   
  
  
- CAGCTCGTCA AAATTTATTG AAAAGCTAGG AAGTACAAAT TTGACTCTTA CTACATATCT TGATCTTCTG   
  
  
- ACAGCCGTTA AAGAAAGTAA AACACACACA AACCGTCACA ATATCGCTAA CGAAAAGTTT ATCAAAAAGC   
  
  
- ACGGCTTTAT GTACGGTTAC TACAAAAAAA TAAAAAAATT TTAGTAAAAA TTATAGTTGT GTAGTTTTGC   
  
  
- TAGGTTTTCT ATGTTTGGCG TGAGTTAAAA TCGTTTTTTT TTTTAAGTTT TAAATTGTTT TGCGTCCAAA   
  
  
- TTTGCGTGTC GGTTTGTCAG ACAATACCGA CCAAAAATTG AAATAAGATG CTGGTCTAGT ATTTTTTACT   
  
  
- ATAGAACTGA TTAATACACA GCTAATTAAT TTAAATGCTC ATTTATACAA TTTGGTCACA AGTGCTGATA   
  
  
- CGAAGAACTT TGCTGCACCA TAAGTAGTAT AAATAAATGA AGATCTCCAA AAAACATATT TTTTTATTTA   
  
  
- TTTTTTTTAC GTAATACTCT AATCGGCCGA TCTATCGCCC TAACTCGGTC TCGGACTTAA CGTCGACGAA   
  
  
- ATCTATCCCT ACCACTACCT TTTCTTTCCC TTTTAAATAG GTCGTGCTTG TCTTTTCGTT TTGCGGGAAC   
  
  
- GGGCGTTGTT CGGTCCTGCG CACCGCTTCC TCCGAAATTT ACTTACGCTG TCTACAATAA TAACTCCAAA   
  
  
- CTGAACAAAG GGCCAACAAT AACAAAAATA TTTTTTTAAT TAAACAAAAA AAATTTTTTT CAAATGTAAT   
  
  
- AAAATTATAC AGCTGCCTTT ATTATTTAGA ATTTTTTATT TTTTATAATA AAATTATATT AAAATTTATT   
  
  
- TTTATATAAA ATATATTATA TCAAACACAA AACAAATTTA GTATTTAAAT TTTTATAATC TAATTATTTA   
  
  
- AGTGGGATAA AAAAAATACT TAGGGTTAAT CCTAAAATAA AAATTTATAA TCAATTCTTT TTTAATATTA   
  
  
- AATATTAAAT AATTATTGGC ATTGATATTT TTTGATGGCA TTATGTTTTA GTTTACGTTT TAAGAGTTAG   
  
  
- ATTTAAGTAC ATGACAAGAC TGTGTATTAA TTGAGGGGTA AGTCAATGAC AAAAACAAAA ATAGTGAACT   
  
  
- CAATTAAGGA GAAAAAAAAA GATCTTCTTT AAAATTAAAG TAACAAATAT ATTTACCATT TTTTTGTTTT   
  
  
- TACTTTTAGC ACTTGGTAGT TTGTACACGT ACGATTTACG ACGTAAACGA TGGATATTAA CATAAAAGAA   
  
  
- TATTATAACA AAACTTTATA ACTTTTTTGT GATAAAGAAA ATTAGACACA AAAATCTCGA GATAACTAAC   
  
  
- AAAAAAAAAA AAAATGTGAC ACTACTATTT TTGGTTATTG GTGCGGAGTA AGATAATAAT AATAATAGTA   
  
  
- ATAGTGTTCT TTAATATTTT TTAAATAACC TAATTCCTTA CAAATCTTAA CGCAAAATTT AACATAAAAA   
  
  
- TTTTAAAAAC TAAAAAATAA ATTTTAATTA AAAAATCTAA TAAAAATATA TAACTATAGA TTTGATTAAA   
  
  
- ATTTTTTAAT TTTTTATATA ATAAAATTAT ATAAAAATTT ATTTTTTATT AAATTTTTTG TTGAAGATAA   
  
  
- TATGTCCTAT TTTATGGGTA ATGGCTAAAT TGTAAAACTC TTCTTTTTAG ATGTAGGTTC CAAGAATAAG   
  
  
- GAACTCCTGT AATACCAGTA ATGTATAGGT CCTGTTTGGA AACGAACCTG ATTTTAAGTT TGGTTTACCT   
  
  
- TAAAGTATCG ACAGTTTATG TCACTTCGAC CCCTAGGTAC CTTTCAGGGG ACGAAATGAG ACGTCGATAT   
  
  
- TTATGTGGTT GATCCTGTGT TGGTATTTGG TGTCATTGG

+     AT1-motif

| Site Name | Organism | Position | Strand | Matrix score. | sequence | function |
| --- | --- | --- | --- | --- | --- | --- |
| AT1-motif | Solanum tuberosum | 1719 | - | 14 | AATTATTTTTTATT | part of a light responsive module |

>Potri.003G139300.1   
+ CAATCAAATA TCACGTCTTG CCTACAAGCA TGGTTTTCAA CTGTCAATTT CTGCTTGAAA GTGCTTCCAT   
  
  
+ ATTAGCACGA GGACTCAGAA GGATAACGAG AATATCAAGC AACTTATGCC AGTCCTTTGT AATTAGCAGA   
  
  
+ GTCGAGCAGT TTTAAATAAC TTTTCGATCC TTCATGTTTA AACTGAGAAT GATGTATAGA ACTAGAAGAC   
  
  
+ TGTCGGCAAT TTCTTTCATT TTGTGTGTGT TTGGCAGTGT TATAGCGATT GCTTTTCAAA TAGTTTTTCG   
  
  
+ TGCCGAAATA CATGCCAATG ATGTTTTTTT ATTTTTTTAA AATCATTTTT AATATCAACA CATCAAAACG   
  
  
+ ATCCAAAAGA TACAAACCGC ACTCAATTTT AGCAAAAAAA AAAATTCAAA ATTTAACAAA ACGCAGGTTT   
  
  
+ AAACGCACAG CCAAACAGTC TGTTATGGCT GGTTTTTAAC TTTATTCTAC GACCAGATCA TAAAAAATGA   
  
  
+ TATCTTGACT AATTATGTGT CGATTAATTA AATTTACGAG TAAATATGTT AAACCAGTGT TCACGACTAT   
  
  
+ GCTTCTTGAA ACGACGTGGT ATTCATCATA TTTATTTACT TCTAGAGGTT TTTTGTATAA AAAAATAAAT   
  
  
+ AAAAAAAATG CATTATGAGA TTAGCCGGCT AGATAGCGGG ATTGAGCCAG AGCCTGAATT GCAGCTGCTT   
  
  
+ TAGATAGGGA TGGTGATGGA AAAGAAAGGG AAAATTTATC CAGCACGAAC AGAAAAGCAA AACGCCCTTG   
  
  
+ CCCGCAACAA GCCAGGACGC GTGGCGAAGG AGGCTTTAAA TGAATGCGAC AGATGTTATT ATTGAGGTTT   
  
  
+ GACTTGTTTC CCGGTTGTTA TTGTTTTTAT AAAAAAATTA ATTTGTTTTT TTTAAAAAAA GTTTACATTA   
  
  
+ TTTTAATATG TCGACGGAAA TAATAAATCT TAAAAAATAA AAAATATTAT TTTAATATAA TTTTAAATAA   
  
  
+ AAATATATTT TATATAATAT AGTTTGTGTT TTGTTTAAAT CATAAATTTA AAAATATTAG ATTAATAAAT   
  
  
+ TCACCCTATT TTTTTTATGA ATCCCAATTA GGATTTTATT TTTAAATATT AGTTAAGAAA AAATTATAAT   
  
  
+ TTATAATTTA TTAATAACCG TAACTATAAA AAACTACCGT AATACAAAAT CAAATGCAAA ATTCTCAATC   
  
  
+ TAAATTCATG TACTGTTCTG ACACATAATT AACTCCCCAT TCAGTTACTG TTTTTGTTTT TATCACTTGA   
  
  
+ GTTAATTCCT CTTTTTTTTT CTAGAAGAAA TTTTAATTTC ATTGTTTATA TAAATGGTAA AAAAACAAAA   
  
  
+ ATGAAAATCG TGAACCATCA AACATGTGCA TGCTAAATGC TGCATTTGCT ACCTATAATT GTATTTTCTT   
  
  
+ ATAATATTGT TTTGAAATAT TGAAAAAACA CTATTTCTTT TAATCTGTGT TTTTAGAGCT CTATTGATTG   
  
  
+ TTTTTTTTTT TTTTACACTG TGATGATAAA AACCAATAAC CACGCCTCAT TCTATTATTA TTATTATCAT   
  
  
+ TATCACAAGA AATTATAAAA AATTTATTGG ATTAAGGAAT GTTTAGAATT GCGTTTTAAA TTGTATTTTT   
  
  
+ AAAATTTTTG ATTTTTTATT TAAAATTAAT TTTTTAGATT ATTTTTATAT ATTGATATCT AAACTAATTT   
  
  
+ TAAAAAATTA AAAAATATAT TATTTTAATA TATTTTTAAA TAAAAAATAA TTTAAAAAAC AACTTCTATT   
  
  
+ ATACAGGATA AAATACCCAT TACCGATTTA ACATTTTGAG AAGAAAAATC TACATCCAAG GTTCTTATTC   
  
  
+ CTTGAGGACA TTATGGTCAT TACATATCCA GGACAAACCT TTGCTTGGAC TAAAATTCAA ACCAAATGGA   
  
  
+ ATTTCATAGC TGTCAAATAC AGTGAAGCTG GGGATCCATG GAAAGTCCCC TGCTTTACTC TGCAGCTATA   
  
  
+ AATACACCAA CTAGGACACA ACCATAAACC ACAGTAACC  

- GTTAGTTTAT AGTGCAGAAC GGATGTTCGT ACCAAAAGTT GACAGTTAAA GACGAACTTT CACGAAGGTA   
  
  
- TAATCGTGCT CCTGAGTCTT CCTATTGCTC TTATAGTTCG TTGAATACGG TCAGGAAACA TTAATCGTCT   
  
  
- CAGCTCGTCA AAATTTATTG AAAAGCTAGG AAGTACAAAT TTGACTCTTA CTACATATCT TGATCTTCTG   
  
  
- ACAGCCGTTA AAGAAAGTAA AACACACACA AACCGTCACA ATATCGCTAA CGAAAAGTTT ATCAAAAAGC   
  
  
- ACGGCTTTAT GTACGGTTAC TACAAAAAAA TAAAAAAATT TTAGTAAAAA TTATAGTTGT GTAGTTTTGC   
  
  
- TAGGTTTTCT ATGTTTGGCG TGAGTTAAAA TCGTTTTTTT TTTTAAGTTT TAAATTGTTT TGCGTCCAAA   
  
  
- TTTGCGTGTC GGTTTGTCAG ACAATACCGA CCAAAAATTG AAATAAGATG CTGGTCTAGT ATTTTTTACT   
  
  
- ATAGAACTGA TTAATACACA GCTAATTAAT TTAAATGCTC ATTTATACAA TTTGGTCACA AGTGCTGATA   
  
  
- CGAAGAACTT TGCTGCACCA TAAGTAGTAT AAATAAATGA AGATCTCCAA AAAACATATT TTTTTATTTA   
  
  
- TTTTTTTTAC GTAATACTCT AATCGGCCGA TCTATCGCCC TAACTCGGTC TCGGACTTAA CGTCGACGAA   
  
  
- ATCTATCCCT ACCACTACCT TTTCTTTCCC TTTTAAATAG GTCGTGCTTG TCTTTTCGTT TTGCGGGAAC   
  
  
- GGGCGTTGTT CGGTCCTGCG CACCGCTTCC TCCGAAATTT ACTTACGCTG TCTACAATAA TAACTCCAAA   
  
  
- CTGAACAAAG GGCCAACAAT AACAAAAATA TTTTTTTAAT TAAACAAAAA AAATTTTTTT CAAATGTAAT   
  
  
- AAAATTATAC AGCTGCCTTT ATTATTTAGA ATTTTTTATT TTTTATAATA AAATTATATT AAAATTTATT   
  
  
- TTTATATAAA ATATATTATA TCAAACACAA AACAAATTTA GTATTTAAAT TTTTATAATC TAATTATTTA   
  
  
- AGTGGGATAA AAAAAATACT TAGGGTTAAT CCTAAAATAA AAATTTATAA TCAATTCTTT TTTAATATTA   
  
  
- AATATTAAAT AATTATTGGC ATTGATATTT TTTGATGGCA TTATGTTTTA GTTTACGTTT TAAGAGTTAG   
  
  
- ATTTAAGTAC ATGACAAGAC TGTGTATTAA TTGAGGGGTA AGTCAATGAC AAAAACAAAA ATAGTGAACT   
  
  
- CAATTAAGGA GAAAAAAAAA GATCTTCTTT AAAATTAAAG TAACAAATAT ATTTACCATT TTTTTGTTTT   
  
  
- TACTTTTAGC ACTTGGTAGT TTGTACACGT ACGATTTACG ACGTAAACGA TGGATATTAA CATAAAAGAA   
  
  
- TATTATAACA AAACTTTATA ACTTTTTTGT GATAAAGAAA ATTAGACACA AAAATCTCGA GATAACTAAC   
  
  
- AAAAAAAAAA AAAATGTGAC ACTACTATTT TTGGTTATTG GTGCGGAGTA AGATAATAAT AATAATAGTA   
  
  
- ATAGTGTTCT TTAATATTTT TTAAATAACC TAATTCCTTA CAAATCTTAA CGCAAAATTT AACATAAAAA   
  
  
- TTTTAAAAAC TAAAAAATAA ATTTTAATTA AAAAATCTAA TAAAAATATA TAACTATAGA TTTGATTAAA   
  
  
- ATTTTTTAAT TTTTTATATA ATAAAATTAT ATAAAAATTT ATTTTTTATT AAATTTTTTG TTGAAGATAA   
  
  
- TATGTCCTAT TTTATGGGTA ATGGCTAAAT TGTAAAACTC TTCTTTTTAG ATGTAGGTTC CAAGAATAAG   
  
  
- GAACTCCTGT AATACCAGTA ATGTATAGGT CCTGTTTGGA AACGAACCTG ATTTTAAGTT TGGTTTACCT   
  
  
- TAAAGTATCG ACAGTTTATG TCACTTCGAC CCCTAGGTAC CTTTCAGGGG ACGAAATGAG ACGTCGATAT   
  
  
- TTATGTGGTT GATCCTGTGT TGGTATTTGG TGTCATTGG

+     AT~TATA-box

| Site Name | Organism | Position | Strand | Matrix score. | sequence | function |
| --- | --- | --- | --- | --- | --- | --- |
| AT~TATA-box | Arabidopsis thaliana | 1656 | - | 6 | TATATA |  |
| AT~TATA-box | Arabidopsis thaliana | 1654 | - | 8 | TATATAAA |  |
| AT~TATA-box | Arabidopsis thaliana | 991 | + | 6 | TATATA |  |
| AT~TATA-box | Arabidopsis thaliana | 989 | - | 8 | TATATAAA |  |
| AT~TATA-box | Arabidopsis thaliana | 1307 | - | 6 | TATATA |  |
| AT~TATA-box | Arabidopsis thaliana | 1305 | - | 8 | TATATAAA |  |

>Potri.003G139300.1   
+ CAATCAAATA TCACGTCTTG CCTACAAGCA TGGTTTTCAA CTGTCAATTT CTGCTTGAAA GTGCTTCCAT   
  
  
+ ATTAGCACGA GGACTCAGAA GGATAACGAG AATATCAAGC AACTTATGCC AGTCCTTTGT AATTAGCAGA   
  
  
+ GTCGAGCAGT TTTAAATAAC TTTTCGATCC TTCATGTTTA AACTGAGAAT GATGTATAGA ACTAGAAGAC   
  
  
+ TGTCGGCAAT TTCTTTCATT TTGTGTGTGT TTGGCAGTGT TATAGCGATT GCTTTTCAAA TAGTTTTTCG   
  
  
+ TGCCGAAATA CATGCCAATG ATGTTTTTTT ATTTTTTTAA AATCATTTTT AATATCAACA CATCAAAACG   
  
  
+ ATCCAAAAGA TACAAACCGC ACTCAATTTT AGCAAAAAAA AAAATTCAAA ATTTAACAAA ACGCAGGTTT   
  
  
+ AAACGCACAG CCAAACAGTC TGTTATGGCT GGTTTTTAAC TTTATTCTAC GACCAGATCA TAAAAAATGA   
  
  
+ TATCTTGACT AATTATGTGT CGATTAATTA AATTTACGAG TAAATATGTT AAACCAGTGT TCACGACTAT   
  
  
+ GCTTCTTGAA ACGACGTGGT ATTCATCATA TTTATTTACT TCTAGAGGTT TTTTGTATAA AAAAATAAAT   
  
  
+ AAAAAAAATG CATTATGAGA TTAGCCGGCT AGATAGCGGG ATTGAGCCAG AGCCTGAATT GCAGCTGCTT   
  
  
+ TAGATAGGGA TGGTGATGGA AAAGAAAGGG AAAATTTATC CAGCACGAAC AGAAAAGCAA AACGCCCTTG   
  
  
+ CCCGCAACAA GCCAGGACGC GTGGCGAAGG AGGCTTTAAA TGAATGCGAC AGATGTTATT ATTGAGGTTT   
  
  
+ GACTTGTTTC CCGGTTGTTA TTGTTTTTAT AAAAAAATTA ATTTGTTTTT TTTAAAAAAA GTTTACATTA   
  
  
+ TTTTAATATG TCGACGGAAA TAATAAATCT TAAAAAATAA AAAATATTAT TTTAATATAA TTTTAAATAA   
  
  
+ AAATATATTT TATATAATAT AGTTTGTGTT TTGTTTAAAT CATAAATTTA AAAATATTAG ATTAATAAAT   
  
  
+ TCACCCTATT TTTTTTATGA ATCCCAATTA GGATTTTATT TTTAAATATT AGTTAAGAAA AAATTATAAT   
  
  
+ TTATAATTTA TTAATAACCG TAACTATAAA AAACTACCGT AATACAAAAT CAAATGCAAA ATTCTCAATC   
  
  
+ TAAATTCATG TACTGTTCTG ACACATAATT AACTCCCCAT TCAGTTACTG TTTTTGTTTT TATCACTTGA   
  
  
+ GTTAATTCCT CTTTTTTTTT CTAGAAGAAA TTTTAATTTC ATTGTTTATA TAAATGGTAA AAAAACAAAA   
  
  
+ ATGAAAATCG TGAACCATCA AACATGTGCA TGCTAAATGC TGCATTTGCT ACCTATAATT GTATTTTCTT   
  
  
+ ATAATATTGT TTTGAAATAT TGAAAAAACA CTATTTCTTT TAATCTGTGT TTTTAGAGCT CTATTGATTG   
  
  
+ TTTTTTTTTT TTTTACACTG TGATGATAAA AACCAATAAC CACGCCTCAT TCTATTATTA TTATTATCAT   
  
  
+ TATCACAAGA AATTATAAAA AATTTATTGG ATTAAGGAAT GTTTAGAATT GCGTTTTAAA TTGTATTTTT   
  
  
+ AAAATTTTTG ATTTTTTATT TAAAATTAAT TTTTTAGATT ATTTTTATAT ATTGATATCT AAACTAATTT   
  
  
+ TAAAAAATTA AAAAATATAT TATTTTAATA TATTTTTAAA TAAAAAATAA TTTAAAAAAC AACTTCTATT   
  
  
+ ATACAGGATA AAATACCCAT TACCGATTTA ACATTTTGAG AAGAAAAATC TACATCCAAG GTTCTTATTC   
  
  
+ CTTGAGGACA TTATGGTCAT TACATATCCA GGACAAACCT TTGCTTGGAC TAAAATTCAA ACCAAATGGA   
  
  
+ ATTTCATAGC TGTCAAATAC AGTGAAGCTG GGGATCCATG GAAAGTCCCC TGCTTTACTC TGCAGCTATA   
  
  
+ AATACACCAA CTAGGACACA ACCATAAACC ACAGTAACC  

- GTTAGTTTAT AGTGCAGAAC GGATGTTCGT ACCAAAAGTT GACAGTTAAA GACGAACTTT CACGAAGGTA   
  
  
- TAATCGTGCT CCTGAGTCTT CCTATTGCTC TTATAGTTCG TTGAATACGG TCAGGAAACA TTAATCGTCT   
  
  
- CAGCTCGTCA AAATTTATTG AAAAGCTAGG AAGTACAAAT TTGACTCTTA CTACATATCT TGATCTTCTG   
  
  
- ACAGCCGTTA AAGAAAGTAA AACACACACA AACCGTCACA ATATCGCTAA CGAAAAGTTT ATCAAAAAGC   
  
  
- ACGGCTTTAT GTACGGTTAC TACAAAAAAA TAAAAAAATT TTAGTAAAAA TTATAGTTGT GTAGTTTTGC   
  
  
- TAGGTTTTCT ATGTTTGGCG TGAGTTAAAA TCGTTTTTTT TTTTAAGTTT TAAATTGTTT TGCGTCCAAA   
  
  
- TTTGCGTGTC GGTTTGTCAG ACAATACCGA CCAAAAATTG AAATAAGATG CTGGTCTAGT ATTTTTTACT   
  
  
- ATAGAACTGA TTAATACACA GCTAATTAAT TTAAATGCTC ATTTATACAA TTTGGTCACA AGTGCTGATA   
  
  
- CGAAGAACTT TGCTGCACCA TAAGTAGTAT AAATAAATGA AGATCTCCAA AAAACATATT TTTTTATTTA   
  
  
- TTTTTTTTAC GTAATACTCT AATCGGCCGA TCTATCGCCC TAACTCGGTC TCGGACTTAA CGTCGACGAA   
  
  
- ATCTATCCCT ACCACTACCT TTTCTTTCCC TTTTAAATAG GTCGTGCTTG TCTTTTCGTT TTGCGGGAAC   
  
  
- GGGCGTTGTT CGGTCCTGCG CACCGCTTCC TCCGAAATTT ACTTACGCTG TCTACAATAA TAACTCCAAA   
  
  
- CTGAACAAAG GGCCAACAAT AACAAAAATA TTTTTTTAAT TAAACAAAAA AAATTTTTTT CAAATGTAAT   
  
  
- AAAATTATAC AGCTGCCTTT ATTATTTAGA ATTTTTTATT TTTTATAATA AAATTATATT AAAATTTATT   
  
  
- TTTATATAAA ATATATTATA TCAAACACAA AACAAATTTA GTATTTAAAT TTTTATAATC TAATTATTTA   
  
  
- AGTGGGATAA AAAAAATACT TAGGGTTAAT CCTAAAATAA AAATTTATAA TCAATTCTTT TTTAATATTA   
  
  
- AATATTAAAT AATTATTGGC ATTGATATTT TTTGATGGCA TTATGTTTTA GTTTACGTTT TAAGAGTTAG   
  
  
- ATTTAAGTAC ATGACAAGAC TGTGTATTAA TTGAGGGGTA AGTCAATGAC AAAAACAAAA ATAGTGAACT   
  
  
- CAATTAAGGA GAAAAAAAAA GATCTTCTTT AAAATTAAAG TAACAAATAT ATTTACCATT TTTTTGTTTT   
  
  
- TACTTTTAGC ACTTGGTAGT TTGTACACGT ACGATTTACG ACGTAAACGA TGGATATTAA CATAAAAGAA   
  
  
- TATTATAACA AAACTTTATA ACTTTTTTGT GATAAAGAAA ATTAGACACA AAAATCTCGA GATAACTAAC   
  
  
- AAAAAAAAAA AAAATGTGAC ACTACTATTT TTGGTTATTG GTGCGGAGTA AGATAATAAT AATAATAGTA   
  
  
- ATAGTGTTCT TTAATATTTT TTAAATAACC TAATTCCTTA CAAATCTTAA CGCAAAATTT AACATAAAAA   
  
  
- TTTTAAAAAC TAAAAAATAA ATTTTAATTA AAAAATCTAA TAAAAATATA TAACTATAGA TTTGATTAAA   
  
  
- ATTTTTTAAT TTTTTATATA ATAAAATTAT ATAAAAATTT ATTTTTTATT AAATTTTTTG TTGAAGATAA   
  
  
- TATGTCCTAT TTTATGGGTA ATGGCTAAAT TGTAAAACTC TTCTTTTTAG ATGTAGGTTC CAAGAATAAG   
  
  
- GAACTCCTGT AATACCAGTA ATGTATAGGT CCTGTTTGGA AACGAACCTG ATTTTAAGTT TGGTTTACCT   
  
  
- TAAAGTATCG ACAGTTTATG TCACTTCGAC CCCTAGGTAC CTTTCAGGGG ACGAAATGAG ACGTCGATAT   
  
  
- TTATGTGGTT GATCCTGTGT TGGTATTTGG TGTCATTGG

+     Box 4

| Site Name | Organism | Position | Strand | Matrix score. | sequence | function |
| --- | --- | --- | --- | --- | --- | --- |
| Box 4 | Petroselinum crispum | 513 | + | 6 | ATTAAT | part of a conserved DNA module involved in light responsiveness |
| Box 4 | Petroselinum crispum | 1635 | - | 6 | ATTAAT | part of a conserved DNA module involved in light responsiveness |
| Box 4 | Petroselinum crispum | 1130 | - | 6 | ATTAAT | part of a conserved DNA module involved in light responsiveness |
| Box 4 | Petroselinum crispum | 1041 | - | 6 | ATTAAT | part of a conserved DNA module involved in light responsiveness |
| Box 4 | Petroselinum crispum | 877 | + | 6 | ATTAAT | part of a conserved DNA module involved in light responsiveness |

>Potri.003G139300.1   
+ CAATCAAATA TCACGTCTTG CCTACAAGCA TGGTTTTCAA CTGTCAATTT CTGCTTGAAA GTGCTTCCAT   
  
  
+ ATTAGCACGA GGACTCAGAA GGATAACGAG AATATCAAGC AACTTATGCC AGTCCTTTGT AATTAGCAGA   
  
  
+ GTCGAGCAGT TTTAAATAAC TTTTCGATCC TTCATGTTTA AACTGAGAAT GATGTATAGA ACTAGAAGAC   
  
  
+ TGTCGGCAAT TTCTTTCATT TTGTGTGTGT TTGGCAGTGT TATAGCGATT GCTTTTCAAA TAGTTTTTCG   
  
  
+ TGCCGAAATA CATGCCAATG ATGTTTTTTT ATTTTTTTAA AATCATTTTT AATATCAACA CATCAAAACG   
  
  
+ ATCCAAAAGA TACAAACCGC ACTCAATTTT AGCAAAAAAA AAAATTCAAA ATTTAACAAA ACGCAGGTTT   
  
  
+ AAACGCACAG CCAAACAGTC TGTTATGGCT GGTTTTTAAC TTTATTCTAC GACCAGATCA TAAAAAATGA   
  
  
+ TATCTTGACT AATTATGTGT CGATTAATTA AATTTACGAG TAAATATGTT AAACCAGTGT TCACGACTAT   
  
  
+ GCTTCTTGAA ACGACGTGGT ATTCATCATA TTTATTTACT TCTAGAGGTT TTTTGTATAA AAAAATAAAT   
  
  
+ AAAAAAAATG CATTATGAGA TTAGCCGGCT AGATAGCGGG ATTGAGCCAG AGCCTGAATT GCAGCTGCTT   
  
  
+ TAGATAGGGA TGGTGATGGA AAAGAAAGGG AAAATTTATC CAGCACGAAC AGAAAAGCAA AACGCCCTTG   
  
  
+ CCCGCAACAA GCCAGGACGC GTGGCGAAGG AGGCTTTAAA TGAATGCGAC AGATGTTATT ATTGAGGTTT   
  
  
+ GACTTGTTTC CCGGTTGTTA TTGTTTTTAT AAAAAAATTA ATTTGTTTTT TTTAAAAAAA GTTTACATTA   
  
  
+ TTTTAATATG TCGACGGAAA TAATAAATCT TAAAAAATAA AAAATATTAT TTTAATATAA TTTTAAATAA   
  
  
+ AAATATATTT TATATAATAT AGTTTGTGTT TTGTTTAAAT CATAAATTTA AAAATATTAG ATTAATAAAT   
  
  
+ TCACCCTATT TTTTTTATGA ATCCCAATTA GGATTTTATT TTTAAATATT AGTTAAGAAA AAATTATAAT   
  
  
+ TTATAATTTA TTAATAACCG TAACTATAAA AAACTACCGT AATACAAAAT CAAATGCAAA ATTCTCAATC   
  
  
+ TAAATTCATG TACTGTTCTG ACACATAATT AACTCCCCAT TCAGTTACTG TTTTTGTTTT TATCACTTGA   
  
  
+ GTTAATTCCT CTTTTTTTTT CTAGAAGAAA TTTTAATTTC ATTGTTTATA TAAATGGTAA AAAAACAAAA   
  
  
+ ATGAAAATCG TGAACCATCA AACATGTGCA TGCTAAATGC TGCATTTGCT ACCTATAATT GTATTTTCTT   
  
  
+ ATAATATTGT TTTGAAATAT TGAAAAAACA CTATTTCTTT TAATCTGTGT TTTTAGAGCT CTATTGATTG   
  
  
+ TTTTTTTTTT TTTTACACTG TGATGATAAA AACCAATAAC CACGCCTCAT TCTATTATTA TTATTATCAT   
  
  
+ TATCACAAGA AATTATAAAA AATTTATTGG ATTAAGGAAT GTTTAGAATT GCGTTTTAAA TTGTATTTTT   
  
  
+ AAAATTTTTG ATTTTTTATT TAAAATTAAT TTTTTAGATT ATTTTTATAT ATTGATATCT AAACTAATTT   
  
  
+ TAAAAAATTA AAAAATATAT TATTTTAATA TATTTTTAAA TAAAAAATAA TTTAAAAAAC AACTTCTATT   
  
  
+ ATACAGGATA AAATACCCAT TACCGATTTA ACATTTTGAG AAGAAAAATC TACATCCAAG GTTCTTATTC   
  
  
+ CTTGAGGACA TTATGGTCAT TACATATCCA GGACAAACCT TTGCTTGGAC TAAAATTCAA ACCAAATGGA   
  
  
+ ATTTCATAGC TGTCAAATAC AGTGAAGCTG GGGATCCATG GAAAGTCCCC TGCTTTACTC TGCAGCTATA   
  
  
+ AATACACCAA CTAGGACACA ACCATAAACC ACAGTAACC  

- GTTAGTTTAT AGTGCAGAAC GGATGTTCGT ACCAAAAGTT GACAGTTAAA GACGAACTTT CACGAAGGTA   
  
  
- TAATCGTGCT CCTGAGTCTT CCTATTGCTC TTATAGTTCG TTGAATACGG TCAGGAAACA TTAATCGTCT   
  
  
- CAGCTCGTCA AAATTTATTG AAAAGCTAGG AAGTACAAAT TTGACTCTTA CTACATATCT TGATCTTCTG   
  
  
- ACAGCCGTTA AAGAAAGTAA AACACACACA AACCGTCACA ATATCGCTAA CGAAAAGTTT ATCAAAAAGC   
  
  
- ACGGCTTTAT GTACGGTTAC TACAAAAAAA TAAAAAAATT TTAGTAAAAA TTATAGTTGT GTAGTTTTGC   
  
  
- TAGGTTTTCT ATGTTTGGCG TGAGTTAAAA TCGTTTTTTT TTTTAAGTTT TAAATTGTTT TGCGTCCAAA   
  
  
- TTTGCGTGTC GGTTTGTCAG ACAATACCGA CCAAAAATTG AAATAAGATG CTGGTCTAGT ATTTTTTACT   
  
  
- ATAGAACTGA TTAATACACA GCTAATTAAT TTAAATGCTC ATTTATACAA TTTGGTCACA AGTGCTGATA   
  
  
- CGAAGAACTT TGCTGCACCA TAAGTAGTAT AAATAAATGA AGATCTCCAA AAAACATATT TTTTTATTTA   
  
  
- TTTTTTTTAC GTAATACTCT AATCGGCCGA TCTATCGCCC TAACTCGGTC TCGGACTTAA CGTCGACGAA   
  
  
- ATCTATCCCT ACCACTACCT TTTCTTTCCC TTTTAAATAG GTCGTGCTTG TCTTTTCGTT TTGCGGGAAC   
  
  
- GGGCGTTGTT CGGTCCTGCG CACCGCTTCC TCCGAAATTT ACTTACGCTG TCTACAATAA TAACTCCAAA   
  
  
- CTGAACAAAG GGCCAACAAT AACAAAAATA TTTTTTTAAT TAAACAAAAA AAATTTTTTT CAAATGTAAT   
  
  
- AAAATTATAC AGCTGCCTTT ATTATTTAGA ATTTTTTATT TTTTATAATA AAATTATATT AAAATTTATT   
  
  
- TTTATATAAA ATATATTATA TCAAACACAA AACAAATTTA GTATTTAAAT TTTTATAATC TAATTATTTA   
  
  
- AGTGGGATAA AAAAAATACT TAGGGTTAAT CCTAAAATAA AAATTTATAA TCAATTCTTT TTTAATATTA   
  
  
- AATATTAAAT AATTATTGGC ATTGATATTT TTTGATGGCA TTATGTTTTA GTTTACGTTT TAAGAGTTAG   
  
  
- ATTTAAGTAC ATGACAAGAC TGTGTATTAA TTGAGGGGTA AGTCAATGAC AAAAACAAAA ATAGTGAACT   
  
  
- CAATTAAGGA GAAAAAAAAA GATCTTCTTT AAAATTAAAG TAACAAATAT ATTTACCATT TTTTTGTTTT   
  
  
- TACTTTTAGC ACTTGGTAGT TTGTACACGT ACGATTTACG ACGTAAACGA TGGATATTAA CATAAAAGAA   
  
  
- TATTATAACA AAACTTTATA ACTTTTTTGT GATAAAGAAA ATTAGACACA AAAATCTCGA GATAACTAAC   
  
  
- AAAAAAAAAA AAAATGTGAC ACTACTATTT TTGGTTATTG GTGCGGAGTA AGATAATAAT AATAATAGTA   
  
  
- ATAGTGTTCT TTAATATTTT TTAAATAACC TAATTCCTTA CAAATCTTAA CGCAAAATTT AACATAAAAA   
  
  
- TTTTAAAAAC TAAAAAATAA ATTTTAATTA AAAAATCTAA TAAAAATATA TAACTATAGA TTTGATTAAA   
  
  
- ATTTTTTAAT TTTTTATATA ATAAAATTAT ATAAAAATTT ATTTTTTATT AAATTTTTTG TTGAAGATAA   
  
  
- TATGTCCTAT TTTATGGGTA ATGGCTAAAT TGTAAAACTC TTCTTTTTAG ATGTAGGTTC CAAGAATAAG   
  
  
- GAACTCCTGT AATACCAGTA ATGTATAGGT CCTGTTTGGA AACGAACCTG ATTTTAAGTT TGGTTTACCT   
  
  
- TAAAGTATCG ACAGTTTATG TCACTTCGAC CCCTAGGTAC CTTTCAGGGG ACGAAATGAG ACGTCGATAT   
  
  
- TTATGTGGTT GATCCTGTGT TGGTATTTGG TGTCATTGG

+     CAAT-box

| Site Name | Organism | Position | Strand | Matrix score. | sequence | function |
| --- | --- | --- | --- | --- | --- | --- |
| CAAT-box | Arabidopsis thaliana | 1566 | - | 5 | CCAAT | common cis-acting element in promoter and enhancer regions |
| CAAT-box | Arabidopsis thaliana | 1503 | + | 5 | CCAAT | common cis-acting element in promoter and enhancer regions |
| CAAT-box | Nicotiana glutinosa | 217 | + | 4 | CAAT |  |
| CAAT-box | Nicotiana glutinosa | 688 | - | 4 | CAAT |  |
| CAAT-box | Nicotiana glutinosa | 1301 | - | 4 | CAAT |  |
| CAAT-box | Nicotiana glutinosa | 296 | + | 4 | CAAT |  |
| CAAT-box | Arabidopsis thaliana | 295 | + | 5 | CCAAT | common cis-acting element in promoter and enhancer regions |
| CAAT-box | Nicotiana glutinosa | 374 | + | 4 | CAAT |  |
| CAAT-box | Nicotiana glutinosa | 1388 | - | 4 | CAAT |  |
| CAAT-box | Nicotiana glutinosa | 1075 | + | 4 | CAAT |  |
| CAAT-box | Pisum sativum | 1883 | + | 5 | CAAAT | common cis-acting element in promoter and enhancer regions |
| CAAT-box | Nicotiana glutinosa | 1661 | - | 4 | CAAT |  |
| CAAT-box | Arabidopsis thaliana | 1074 | + | 5 | CCAAT | common cis-acting element in promoter and enhancer regions |
| CAAT-box | Nicotiana glutinosa | 258 | - | 4 | CAAT |  |
| CAAT-box | Nicotiana glutinosa | 1 | + | 4 | CAAT |  |
| CAAT-box | Nicotiana glutinosa | 1463 | - | 4 | CAAT |  |
| CAAT-box | Nicotiana glutinosa | 860 | - | 4 | CAAT |  |
| CAAT-box | Pisum sativum | 1374 | - | 5 | CAAAT | common cis-acting element in promoter and enhancer regions |
| CAAT-box | Nicotiana glutinosa | 1419 | - | 4 | CAAT |  |
| CAAT-box | Nicotiana glutinosa | 671 | - | 4 | CAAT |  |
| CAAT-box | Nicotiana glutinosa | 831 | - | 4 | CAAT |  |
| CAAT-box | Nicotiana glutinosa | 1600 | - | 4 | CAAT |  |
| CAAT-box | Pisum sativum | 267 | + | 5 | CAAAT | common cis-acting element in promoter and enhancer regions |
| CAAT-box | Nicotiana glutinosa | 1406 | - | 4 | CAAT |  |
| CAAT-box | Nicotiana glutinosa | 1186 | + | 4 | CAAT |  |
| CAAT-box | Nicotiana glutinosa | 1504 | + | 4 | CAAT |  |
| CAAT-box | Pisum sativum | 5 | + | 5 | CAAAT | common cis-acting element in promoter and enhancer regions |
| CAAT-box | Nicotiana glutinosa | 1467 | - | 4 | CAAT |  |
| CAAT-box | Pisum sativum | 881 | - | 5 | CAAAT | common cis-acting element in promoter and enhancer regions |
| CAAT-box | Pisum sativum | 1904 | + | 5 | CAAAT | common cis-acting element in promoter and enhancer regions |
| CAAT-box | Nicotiana glutinosa | 45 | + | 4 | CAAT |  |
| CAAT-box | Nicotiana glutinosa | 1588 | - | 4 | CAAT |  |
| CAAT-box | Pisum sativum | 1171 | + | 5 | CAAAT | common cis-acting element in promoter and enhancer regions |

>Potri.003G139300.1   
+ CAATCAAATA TCACGTCTTG CCTACAAGCA TGGTTTTCAA CTGTCAATTT CTGCTTGAAA GTGCTTCCAT   
  
  
+ ATTAGCACGA GGACTCAGAA GGATAACGAG AATATCAAGC AACTTATGCC AGTCCTTTGT AATTAGCAGA   
  
  
+ GTCGAGCAGT TTTAAATAAC TTTTCGATCC TTCATGTTTA AACTGAGAAT GATGTATAGA ACTAGAAGAC   
  
  
+ TGTCGGCAAT TTCTTTCATT TTGTGTGTGT TTGGCAGTGT TATAGCGATT GCTTTTCAAA TAGTTTTTCG   
  
  
+ TGCCGAAATA CATGCCAATG ATGTTTTTTT ATTTTTTTAA AATCATTTTT AATATCAACA CATCAAAACG   
  
  
+ ATCCAAAAGA TACAAACCGC ACTCAATTTT AGCAAAAAAA AAAATTCAAA ATTTAACAAA ACGCAGGTTT   
  
  
+ AAACGCACAG CCAAACAGTC TGTTATGGCT GGTTTTTAAC TTTATTCTAC GACCAGATCA TAAAAAATGA   
  
  
+ TATCTTGACT AATTATGTGT CGATTAATTA AATTTACGAG TAAATATGTT AAACCAGTGT TCACGACTAT   
  
  
+ GCTTCTTGAA ACGACGTGGT ATTCATCATA TTTATTTACT TCTAGAGGTT TTTTGTATAA AAAAATAAAT   
  
  
+ AAAAAAAATG CATTATGAGA TTAGCCGGCT AGATAGCGGG ATTGAGCCAG AGCCTGAATT GCAGCTGCTT   
  
  
+ TAGATAGGGA TGGTGATGGA AAAGAAAGGG AAAATTTATC CAGCACGAAC AGAAAAGCAA AACGCCCTTG   
  
  
+ CCCGCAACAA GCCAGGACGC GTGGCGAAGG AGGCTTTAAA TGAATGCGAC AGATGTTATT ATTGAGGTTT   
  
  
+ GACTTGTTTC CCGGTTGTTA TTGTTTTTAT AAAAAAATTA ATTTGTTTTT TTTAAAAAAA GTTTACATTA   
  
  
+ TTTTAATATG TCGACGGAAA TAATAAATCT TAAAAAATAA AAAATATTAT TTTAATATAA TTTTAAATAA   
  
  
+ AAATATATTT TATATAATAT AGTTTGTGTT TTGTTTAAAT CATAAATTTA AAAATATTAG ATTAATAAAT   
  
  
+ TCACCCTATT TTTTTTATGA ATCCCAATTA GGATTTTATT TTTAAATATT AGTTAAGAAA AAATTATAAT   
  
  
+ TTATAATTTA TTAATAACCG TAACTATAAA AAACTACCGT AATACAAAAT CAAATGCAAA ATTCTCAATC   
  
  
+ TAAATTCATG TACTGTTCTG ACACATAATT AACTCCCCAT TCAGTTACTG TTTTTGTTTT TATCACTTGA   
  
  
+ GTTAATTCCT CTTTTTTTTT CTAGAAGAAA TTTTAATTTC ATTGTTTATA TAAATGGTAA AAAAACAAAA   
  
  
+ ATGAAAATCG TGAACCATCA AACATGTGCA TGCTAAATGC TGCATTTGCT ACCTATAATT GTATTTTCTT   
  
  
+ ATAATATTGT TTTGAAATAT TGAAAAAACA CTATTTCTTT TAATCTGTGT TTTTAGAGCT CTATTGATTG   
  
  
+ TTTTTTTTTT TTTTACACTG TGATGATAAA AACCAATAAC CACGCCTCAT TCTATTATTA TTATTATCAT   
  
  
+ TATCACAAGA AATTATAAAA AATTTATTGG ATTAAGGAAT GTTTAGAATT GCGTTTTAAA TTGTATTTTT   
  
  
+ AAAATTTTTG ATTTTTTATT TAAAATTAAT TTTTTAGATT ATTTTTATAT ATTGATATCT AAACTAATTT   
  
  
+ TAAAAAATTA AAAAATATAT TATTTTAATA TATTTTTAAA TAAAAAATAA TTTAAAAAAC AACTTCTATT   
  
  
+ ATACAGGATA AAATACCCAT TACCGATTTA ACATTTTGAG AAGAAAAATC TACATCCAAG GTTCTTATTC   
  
  
+ CTTGAGGACA TTATGGTCAT TACATATCCA GGACAAACCT TTGCTTGGAC TAAAATTCAA ACCAAATGGA   
  
  
+ ATTTCATAGC TGTCAAATAC AGTGAAGCTG GGGATCCATG GAAAGTCCCC TGCTTTACTC TGCAGCTATA   
  
  
+ AATACACCAA CTAGGACACA ACCATAAACC ACAGTAACC  

- GTTAGTTTAT AGTGCAGAAC GGATGTTCGT ACCAAAAGTT GACAGTTAAA GACGAACTTT CACGAAGGTA   
  
  
- TAATCGTGCT CCTGAGTCTT CCTATTGCTC TTATAGTTCG TTGAATACGG TCAGGAAACA TTAATCGTCT   
  
  
- CAGCTCGTCA AAATTTATTG AAAAGCTAGG AAGTACAAAT TTGACTCTTA CTACATATCT TGATCTTCTG   
  
  
- ACAGCCGTTA AAGAAAGTAA AACACACACA AACCGTCACA ATATCGCTAA CGAAAAGTTT ATCAAAAAGC   
  
  
- ACGGCTTTAT GTACGGTTAC TACAAAAAAA TAAAAAAATT TTAGTAAAAA TTATAGTTGT GTAGTTTTGC   
  
  
- TAGGTTTTCT ATGTTTGGCG TGAGTTAAAA TCGTTTTTTT TTTTAAGTTT TAAATTGTTT TGCGTCCAAA   
  
  
- TTTGCGTGTC GGTTTGTCAG ACAATACCGA CCAAAAATTG AAATAAGATG CTGGTCTAGT ATTTTTTACT   
  
  
- ATAGAACTGA TTAATACACA GCTAATTAAT TTAAATGCTC ATTTATACAA TTTGGTCACA AGTGCTGATA   
  
  
- CGAAGAACTT TGCTGCACCA TAAGTAGTAT AAATAAATGA AGATCTCCAA AAAACATATT TTTTTATTTA   
  
  
- TTTTTTTTAC GTAATACTCT AATCGGCCGA TCTATCGCCC TAACTCGGTC TCGGACTTAA CGTCGACGAA   
  
  
- ATCTATCCCT ACCACTACCT TTTCTTTCCC TTTTAAATAG GTCGTGCTTG TCTTTTCGTT TTGCGGGAAC   
  
  
- GGGCGTTGTT CGGTCCTGCG CACCGCTTCC TCCGAAATTT ACTTACGCTG TCTACAATAA TAACTCCAAA   
  
  
- CTGAACAAAG GGCCAACAAT AACAAAAATA TTTTTTTAAT TAAACAAAAA AAATTTTTTT CAAATGTAAT   
  
  
- AAAATTATAC AGCTGCCTTT ATTATTTAGA ATTTTTTATT TTTTATAATA AAATTATATT AAAATTTATT   
  
  
- TTTATATAAA ATATATTATA TCAAACACAA AACAAATTTA GTATTTAAAT TTTTATAATC TAATTATTTA   
  
  
- AGTGGGATAA AAAAAATACT TAGGGTTAAT CCTAAAATAA AAATTTATAA TCAATTCTTT TTTAATATTA   
  
  
- AATATTAAAT AATTATTGGC ATTGATATTT TTTGATGGCA TTATGTTTTA GTTTACGTTT TAAGAGTTAG   
  
  
- ATTTAAGTAC ATGACAAGAC TGTGTATTAA TTGAGGGGTA AGTCAATGAC AAAAACAAAA ATAGTGAACT   
  
  
- CAATTAAGGA GAAAAAAAAA GATCTTCTTT AAAATTAAAG TAACAAATAT ATTTACCATT TTTTTGTTTT   
  
  
- TACTTTTAGC ACTTGGTAGT TTGTACACGT ACGATTTACG ACGTAAACGA TGGATATTAA CATAAAAGAA   
  
  
- TATTATAACA AAACTTTATA ACTTTTTTGT GATAAAGAAA ATTAGACACA AAAATCTCGA GATAACTAAC   
  
  
- AAAAAAAAAA AAAATGTGAC ACTACTATTT TTGGTTATTG GTGCGGAGTA AGATAATAAT AATAATAGTA   
  
  
- ATAGTGTTCT TTAATATTTT TTAAATAACC TAATTCCTTA CAAATCTTAA CGCAAAATTT AACATAAAAA   
  
  
- TTTTAAAAAC TAAAAAATAA ATTTTAATTA AAAAATCTAA TAAAAATATA TAACTATAGA TTTGATTAAA   
  
  
- ATTTTTTAAT TTTTTATATA ATAAAATTAT ATAAAAATTT ATTTTTTATT AAATTTTTTG TTGAAGATAA   
  
  
- TATGTCCTAT TTTATGGGTA ATGGCTAAAT TGTAAAACTC TTCTTTTTAG ATGTAGGTTC CAAGAATAAG   
  
  
- GAACTCCTGT AATACCAGTA ATGTATAGGT CCTGTTTGGA AACGAACCTG ATTTTAAGTT TGGTTTACCT   
  
  
- TAAAGTATCG ACAGTTTATG TCACTTCGAC CCCTAGGTAC CTTTCAGGGG ACGAAATGAG ACGTCGATAT   
  
  
- TTATGTGGTT GATCCTGTGT TGGTATTTGG TGTCATTGG

+     DRE core

| Site Name | Organism | Position | Strand | Matrix score. | sequence | function |
| --- | --- | --- | --- | --- | --- | --- |
| DRE core | Arabidopsis thaliana | 212 | - | 6 | GCCGAC |  |

>Potri.003G139300.1   
+ CAATCAAATA TCACGTCTTG CCTACAAGCA TGGTTTTCAA CTGTCAATTT CTGCTTGAAA GTGCTTCCAT   
  
  
+ ATTAGCACGA GGACTCAGAA GGATAACGAG AATATCAAGC AACTTATGCC AGTCCTTTGT AATTAGCAGA   
  
  
+ GTCGAGCAGT TTTAAATAAC TTTTCGATCC TTCATGTTTA AACTGAGAAT GATGTATAGA ACTAGAAGAC   
  
  
+ TGTCGGCAAT TTCTTTCATT TTGTGTGTGT TTGGCAGTGT TATAGCGATT GCTTTTCAAA TAGTTTTTCG   
  
  
+ TGCCGAAATA CATGCCAATG ATGTTTTTTT ATTTTTTTAA AATCATTTTT AATATCAACA CATCAAAACG   
  
  
+ ATCCAAAAGA TACAAACCGC ACTCAATTTT AGCAAAAAAA AAAATTCAAA ATTTAACAAA ACGCAGGTTT   
  
  
+ AAACGCACAG CCAAACAGTC TGTTATGGCT GGTTTTTAAC TTTATTCTAC GACCAGATCA TAAAAAATGA   
  
  
+ TATCTTGACT AATTATGTGT CGATTAATTA AATTTACGAG TAAATATGTT AAACCAGTGT TCACGACTAT   
  
  
+ GCTTCTTGAA ACGACGTGGT ATTCATCATA TTTATTTACT TCTAGAGGTT TTTTGTATAA AAAAATAAAT   
  
  
+ AAAAAAAATG CATTATGAGA TTAGCCGGCT AGATAGCGGG ATTGAGCCAG AGCCTGAATT GCAGCTGCTT   
  
  
+ TAGATAGGGA TGGTGATGGA AAAGAAAGGG AAAATTTATC CAGCACGAAC AGAAAAGCAA AACGCCCTTG   
  
  
+ CCCGCAACAA GCCAGGACGC GTGGCGAAGG AGGCTTTAAA TGAATGCGAC AGATGTTATT ATTGAGGTTT   
  
  
+ GACTTGTTTC CCGGTTGTTA TTGTTTTTAT AAAAAAATTA ATTTGTTTTT TTTAAAAAAA GTTTACATTA   
  
  
+ TTTTAATATG TCGACGGAAA TAATAAATCT TAAAAAATAA AAAATATTAT TTTAATATAA TTTTAAATAA   
  
  
+ AAATATATTT TATATAATAT AGTTTGTGTT TTGTTTAAAT CATAAATTTA AAAATATTAG ATTAATAAAT   
  
  
+ TCACCCTATT TTTTTTATGA ATCCCAATTA GGATTTTATT TTTAAATATT AGTTAAGAAA AAATTATAAT   
  
  
+ TTATAATTTA TTAATAACCG TAACTATAAA AAACTACCGT AATACAAAAT CAAATGCAAA ATTCTCAATC   
  
  
+ TAAATTCATG TACTGTTCTG ACACATAATT AACTCCCCAT TCAGTTACTG TTTTTGTTTT TATCACTTGA   
  
  
+ GTTAATTCCT CTTTTTTTTT CTAGAAGAAA TTTTAATTTC ATTGTTTATA TAAATGGTAA AAAAACAAAA   
  
  
+ ATGAAAATCG TGAACCATCA AACATGTGCA TGCTAAATGC TGCATTTGCT ACCTATAATT GTATTTTCTT   
  
  
+ ATAATATTGT TTTGAAATAT TGAAAAAACA CTATTTCTTT TAATCTGTGT TTTTAGAGCT CTATTGATTG   
  
  
+ TTTTTTTTTT TTTTACACTG TGATGATAAA AACCAATAAC CACGCCTCAT TCTATTATTA TTATTATCAT   
  
  
+ TATCACAAGA AATTATAAAA AATTTATTGG ATTAAGGAAT GTTTAGAATT GCGTTTTAAA TTGTATTTTT   
  
  
+ AAAATTTTTG ATTTTTTATT TAAAATTAAT TTTTTAGATT ATTTTTATAT ATTGATATCT AAACTAATTT   
  
  
+ TAAAAAATTA AAAAATATAT TATTTTAATA TATTTTTAAA TAAAAAATAA TTTAAAAAAC AACTTCTATT   
  
  
+ ATACAGGATA AAATACCCAT TACCGATTTA ACATTTTGAG AAGAAAAATC TACATCCAAG GTTCTTATTC   
  
  
+ CTTGAGGACA TTATGGTCAT TACATATCCA GGACAAACCT TTGCTTGGAC TAAAATTCAA ACCAAATGGA   
  
  
+ ATTTCATAGC TGTCAAATAC AGTGAAGCTG GGGATCCATG GAAAGTCCCC TGCTTTACTC TGCAGCTATA   
  
  
+ AATACACCAA CTAGGACACA ACCATAAACC ACAGTAACC  

- GTTAGTTTAT AGTGCAGAAC GGATGTTCGT ACCAAAAGTT GACAGTTAAA GACGAACTTT CACGAAGGTA   
  
  
- TAATCGTGCT CCTGAGTCTT CCTATTGCTC TTATAGTTCG TTGAATACGG TCAGGAAACA TTAATCGTCT   
  
  
- CAGCTCGTCA AAATTTATTG AAAAGCTAGG AAGTACAAAT TTGACTCTTA CTACATATCT TGATCTTCTG   
  
  
- ACAGCCGTTA AAGAAAGTAA AACACACACA AACCGTCACA ATATCGCTAA CGAAAAGTTT ATCAAAAAGC   
  
  
- ACGGCTTTAT GTACGGTTAC TACAAAAAAA TAAAAAAATT TTAGTAAAAA TTATAGTTGT GTAGTTTTGC   
  
  
- TAGGTTTTCT ATGTTTGGCG TGAGTTAAAA TCGTTTTTTT TTTTAAGTTT TAAATTGTTT TGCGTCCAAA   
  
  
- TTTGCGTGTC GGTTTGTCAG ACAATACCGA CCAAAAATTG AAATAAGATG CTGGTCTAGT ATTTTTTACT   
  
  
- ATAGAACTGA TTAATACACA GCTAATTAAT TTAAATGCTC ATTTATACAA TTTGGTCACA AGTGCTGATA   
  
  
- CGAAGAACTT TGCTGCACCA TAAGTAGTAT AAATAAATGA AGATCTCCAA AAAACATATT TTTTTATTTA   
  
  
- TTTTTTTTAC GTAATACTCT AATCGGCCGA TCTATCGCCC TAACTCGGTC TCGGACTTAA CGTCGACGAA   
  
  
- ATCTATCCCT ACCACTACCT TTTCTTTCCC TTTTAAATAG GTCGTGCTTG TCTTTTCGTT TTGCGGGAAC   
  
  
- GGGCGTTGTT CGGTCCTGCG CACCGCTTCC TCCGAAATTT ACTTACGCTG TCTACAATAA TAACTCCAAA   
  
  
- CTGAACAAAG GGCCAACAAT AACAAAAATA TTTTTTTAAT TAAACAAAAA AAATTTTTTT CAAATGTAAT   
  
  
- AAAATTATAC AGCTGCCTTT ATTATTTAGA ATTTTTTATT TTTTATAATA AAATTATATT AAAATTTATT   
  
  
- TTTATATAAA ATATATTATA TCAAACACAA AACAAATTTA GTATTTAAAT TTTTATAATC TAATTATTTA   
  
  
- AGTGGGATAA AAAAAATACT TAGGGTTAAT CCTAAAATAA AAATTTATAA TCAATTCTTT TTTAATATTA   
  
  
- AATATTAAAT AATTATTGGC ATTGATATTT TTTGATGGCA TTATGTTTTA GTTTACGTTT TAAGAGTTAG   
  
  
- ATTTAAGTAC ATGACAAGAC TGTGTATTAA TTGAGGGGTA AGTCAATGAC AAAAACAAAA ATAGTGAACT   
  
  
- CAATTAAGGA GAAAAAAAAA GATCTTCTTT AAAATTAAAG TAACAAATAT ATTTACCATT TTTTTGTTTT   
  
  
- TACTTTTAGC ACTTGGTAGT TTGTACACGT ACGATTTACG ACGTAAACGA TGGATATTAA CATAAAAGAA   
  
  
- TATTATAACA AAACTTTATA ACTTTTTTGT GATAAAGAAA ATTAGACACA AAAATCTCGA GATAACTAAC   
  
  
- AAAAAAAAAA AAAATGTGAC ACTACTATTT TTGGTTATTG GTGCGGAGTA AGATAATAAT AATAATAGTA   
  
  
- ATAGTGTTCT TTAATATTTT TTAAATAACC TAATTCCTTA CAAATCTTAA CGCAAAATTT AACATAAAAA   
  
  
- TTTTAAAAAC TAAAAAATAA ATTTTAATTA AAAAATCTAA TAAAAATATA TAACTATAGA TTTGATTAAA   
  
  
- ATTTTTTAAT TTTTTATATA ATAAAATTAT ATAAAAATTT ATTTTTTATT AAATTTTTTG TTGAAGATAA   
  
  
- TATGTCCTAT TTTATGGGTA ATGGCTAAAT TGTAAAACTC TTCTTTTTAG ATGTAGGTTC CAAGAATAAG   
  
  
- GAACTCCTGT AATACCAGTA ATGTATAGGT CCTGTTTGGA AACGAACCTG ATTTTAAGTT TGGTTTACCT   
  
  
- TAAAGTATCG ACAGTTTATG TCACTTCGAC CCCTAGGTAC CTTTCAGGGG ACGAAATGAG ACGTCGATAT   
  
  
- TTATGTGGTT GATCCTGTGT TGGTATTTGG TGTCATTGG

+     ERE

| Site Name | Organism | Position | Strand | Matrix score. | sequence | function |
| --- | --- | --- | --- | --- | --- | --- |
| ERE | Nicotiana glutinos | 316 | - | 8 | ATTTTAAA |  |
| ERE | Nicotiana glutinos | 1677 | + | 8 | ATTTTAAA |  |
| ERE | Nicotiana glutinos | 1608 | - | 8 | ATTTTAAA |  |
| ERE | Nicotiana glutinos | 1891 | + | 8 | ATTTCATA |  |
| ERE | Nicotiana glutinos | 970 | + | 8 | ATTTTAAA |  |
| ERE | Nicotiana glutinos | 1629 | - | 8 | ATTTTAAA |  |

>Potri.003G139300.1   
+ CAATCAAATA TCACGTCTTG CCTACAAGCA TGGTTTTCAA CTGTCAATTT CTGCTTGAAA GTGCTTCCAT   
  
  
+ ATTAGCACGA GGACTCAGAA GGATAACGAG AATATCAAGC AACTTATGCC AGTCCTTTGT AATTAGCAGA   
  
  
+ GTCGAGCAGT TTTAAATAAC TTTTCGATCC TTCATGTTTA AACTGAGAAT GATGTATAGA ACTAGAAGAC   
  
  
+ TGTCGGCAAT TTCTTTCATT TTGTGTGTGT TTGGCAGTGT TATAGCGATT GCTTTTCAAA TAGTTTTTCG   
  
  
+ TGCCGAAATA CATGCCAATG ATGTTTTTTT ATTTTTTTAA AATCATTTTT AATATCAACA CATCAAAACG   
  
  
+ ATCCAAAAGA TACAAACCGC ACTCAATTTT AGCAAAAAAA AAAATTCAAA ATTTAACAAA ACGCAGGTTT   
  
  
+ AAACGCACAG CCAAACAGTC TGTTATGGCT GGTTTTTAAC TTTATTCTAC GACCAGATCA TAAAAAATGA   
  
  
+ TATCTTGACT AATTATGTGT CGATTAATTA AATTTACGAG TAAATATGTT AAACCAGTGT TCACGACTAT   
  
  
+ GCTTCTTGAA ACGACGTGGT ATTCATCATA TTTATTTACT TCTAGAGGTT TTTTGTATAA AAAAATAAAT   
  
  
+ AAAAAAAATG CATTATGAGA TTAGCCGGCT AGATAGCGGG ATTGAGCCAG AGCCTGAATT GCAGCTGCTT   
  
  
+ TAGATAGGGA TGGTGATGGA AAAGAAAGGG AAAATTTATC CAGCACGAAC AGAAAAGCAA AACGCCCTTG   
  
  
+ CCCGCAACAA GCCAGGACGC GTGGCGAAGG AGGCTTTAAA TGAATGCGAC AGATGTTATT ATTGAGGTTT   
  
  
+ GACTTGTTTC CCGGTTGTTA TTGTTTTTAT AAAAAAATTA ATTTGTTTTT TTTAAAAAAA GTTTACATTA   
  
  
+ TTTTAATATG TCGACGGAAA TAATAAATCT TAAAAAATAA AAAATATTAT TTTAATATAA TTTTAAATAA   
  
  
+ AAATATATTT TATATAATAT AGTTTGTGTT TTGTTTAAAT CATAAATTTA AAAATATTAG ATTAATAAAT   
  
  
+ TCACCCTATT TTTTTTATGA ATCCCAATTA GGATTTTATT TTTAAATATT AGTTAAGAAA AAATTATAAT   
  
  
+ TTATAATTTA TTAATAACCG TAACTATAAA AAACTACCGT AATACAAAAT CAAATGCAAA ATTCTCAATC   
  
  
+ TAAATTCATG TACTGTTCTG ACACATAATT AACTCCCCAT TCAGTTACTG TTTTTGTTTT TATCACTTGA   
  
  
+ GTTAATTCCT CTTTTTTTTT CTAGAAGAAA TTTTAATTTC ATTGTTTATA TAAATGGTAA AAAAACAAAA   
  
  
+ ATGAAAATCG TGAACCATCA AACATGTGCA TGCTAAATGC TGCATTTGCT ACCTATAATT GTATTTTCTT   
  
  
+ ATAATATTGT TTTGAAATAT TGAAAAAACA CTATTTCTTT TAATCTGTGT TTTTAGAGCT CTATTGATTG   
  
  
+ TTTTTTTTTT TTTTACACTG TGATGATAAA AACCAATAAC CACGCCTCAT TCTATTATTA TTATTATCAT   
  
  
+ TATCACAAGA AATTATAAAA AATTTATTGG ATTAAGGAAT GTTTAGAATT GCGTTTTAAA TTGTATTTTT   
  
  
+ AAAATTTTTG ATTTTTTATT TAAAATTAAT TTTTTAGATT ATTTTTATAT ATTGATATCT AAACTAATTT   
  
  
+ TAAAAAATTA AAAAATATAT TATTTTAATA TATTTTTAAA TAAAAAATAA TTTAAAAAAC AACTTCTATT   
  
  
+ ATACAGGATA AAATACCCAT TACCGATTTA ACATTTTGAG AAGAAAAATC TACATCCAAG GTTCTTATTC   
  
  
+ CTTGAGGACA TTATGGTCAT TACATATCCA GGACAAACCT TTGCTTGGAC TAAAATTCAA ACCAAATGGA   
  
  
+ ATTTCATAGC TGTCAAATAC AGTGAAGCTG GGGATCCATG GAAAGTCCCC TGCTTTACTC TGCAGCTATA   
  
  
+ AATACACCAA CTAGGACACA ACCATAAACC ACAGTAACC  

- GTTAGTTTAT AGTGCAGAAC GGATGTTCGT ACCAAAAGTT GACAGTTAAA GACGAACTTT CACGAAGGTA   
  
  
- TAATCGTGCT CCTGAGTCTT CCTATTGCTC TTATAGTTCG TTGAATACGG TCAGGAAACA TTAATCGTCT   
  
  
- CAGCTCGTCA AAATTTATTG AAAAGCTAGG AAGTACAAAT TTGACTCTTA CTACATATCT TGATCTTCTG   
  
  
- ACAGCCGTTA AAGAAAGTAA AACACACACA AACCGTCACA ATATCGCTAA CGAAAAGTTT ATCAAAAAGC   
  
  
- ACGGCTTTAT GTACGGTTAC TACAAAAAAA TAAAAAAATT TTAGTAAAAA TTATAGTTGT GTAGTTTTGC   
  
  
- TAGGTTTTCT ATGTTTGGCG TGAGTTAAAA TCGTTTTTTT TTTTAAGTTT TAAATTGTTT TGCGTCCAAA   
  
  
- TTTGCGTGTC GGTTTGTCAG ACAATACCGA CCAAAAATTG AAATAAGATG CTGGTCTAGT ATTTTTTACT   
  
  
- ATAGAACTGA TTAATACACA GCTAATTAAT TTAAATGCTC ATTTATACAA TTTGGTCACA AGTGCTGATA   
  
  
- CGAAGAACTT TGCTGCACCA TAAGTAGTAT AAATAAATGA AGATCTCCAA AAAACATATT TTTTTATTTA   
  
  
- TTTTTTTTAC GTAATACTCT AATCGGCCGA TCTATCGCCC TAACTCGGTC TCGGACTTAA CGTCGACGAA   
  
  
- ATCTATCCCT ACCACTACCT TTTCTTTCCC TTTTAAATAG GTCGTGCTTG TCTTTTCGTT TTGCGGGAAC   
  
  
- GGGCGTTGTT CGGTCCTGCG CACCGCTTCC TCCGAAATTT ACTTACGCTG TCTACAATAA TAACTCCAAA   
  
  
- CTGAACAAAG GGCCAACAAT AACAAAAATA TTTTTTTAAT TAAACAAAAA AAATTTTTTT CAAATGTAAT   
  
  
- AAAATTATAC AGCTGCCTTT ATTATTTAGA ATTTTTTATT TTTTATAATA AAATTATATT AAAATTTATT   
  
  
- TTTATATAAA ATATATTATA TCAAACACAA AACAAATTTA GTATTTAAAT TTTTATAATC TAATTATTTA   
  
  
- AGTGGGATAA AAAAAATACT TAGGGTTAAT CCTAAAATAA AAATTTATAA TCAATTCTTT TTTAATATTA   
  
  
- AATATTAAAT AATTATTGGC ATTGATATTT TTTGATGGCA TTATGTTTTA GTTTACGTTT TAAGAGTTAG   
  
  
- ATTTAAGTAC ATGACAAGAC TGTGTATTAA TTGAGGGGTA AGTCAATGAC AAAAACAAAA ATAGTGAACT   
  
  
- CAATTAAGGA GAAAAAAAAA GATCTTCTTT AAAATTAAAG TAACAAATAT ATTTACCATT TTTTTGTTTT   
  
  
- TACTTTTAGC ACTTGGTAGT TTGTACACGT ACGATTTACG ACGTAAACGA TGGATATTAA CATAAAAGAA   
  
  
- TATTATAACA AAACTTTATA ACTTTTTTGT GATAAAGAAA ATTAGACACA AAAATCTCGA GATAACTAAC   
  
  
- AAAAAAAAAA AAAATGTGAC ACTACTATTT TTGGTTATTG GTGCGGAGTA AGATAATAAT AATAATAGTA   
  
  
- ATAGTGTTCT TTAATATTTT TTAAATAACC TAATTCCTTA CAAATCTTAA CGCAAAATTT AACATAAAAA   
  
  
- TTTTAAAAAC TAAAAAATAA ATTTTAATTA AAAAATCTAA TAAAAATATA TAACTATAGA TTTGATTAAA   
  
  
- ATTTTTTAAT TTTTTATATA ATAAAATTAT ATAAAAATTT ATTTTTTATT AAATTTTTTG TTGAAGATAA   
  
  
- TATGTCCTAT TTTATGGGTA ATGGCTAAAT TGTAAAACTC TTCTTTTTAG ATGTAGGTTC CAAGAATAAG   
  
  
- GAACTCCTGT AATACCAGTA ATGTATAGGT CCTGTTTGGA AACGAACCTG ATTTTAAGTT TGGTTTACCT   
  
  
- TAAAGTATCG ACAGTTTATG TCACTTCGAC CCCTAGGTAC CTTTCAGGGG ACGAAATGAG ACGTCGATAT   
  
  
- TTATGTGGTT GATCCTGTGT TGGTATTTGG TGTCATTGG

+     G-box

| Site Name | Organism | Position | Strand | Matrix score. | sequence | function |
| --- | --- | --- | --- | --- | --- | --- |
| G-box | Zea mays | 12 | + | 6 | CACGTC | cis-acting regulatory element involved in light responsiveness |
| G-box | Zea mays | 573 | - | 6 | CACGTC | cis-acting regulatory element involved in light responsiveness |
| G-box | Zea mays | 552 | + | 6 | CACGAC | cis-acting regulatory element involved in light responsiveness |

>Potri.003G139300.1   
+ CAATCAAATA TCACGTCTTG CCTACAAGCA TGGTTTTCAA CTGTCAATTT CTGCTTGAAA GTGCTTCCAT   
  
  
+ ATTAGCACGA GGACTCAGAA GGATAACGAG AATATCAAGC AACTTATGCC AGTCCTTTGT AATTAGCAGA   
  
  
+ GTCGAGCAGT TTTAAATAAC TTTTCGATCC TTCATGTTTA AACTGAGAAT GATGTATAGA ACTAGAAGAC   
  
  
+ TGTCGGCAAT TTCTTTCATT TTGTGTGTGT TTGGCAGTGT TATAGCGATT GCTTTTCAAA TAGTTTTTCG   
  
  
+ TGCCGAAATA CATGCCAATG ATGTTTTTTT ATTTTTTTAA AATCATTTTT AATATCAACA CATCAAAACG   
  
  
+ ATCCAAAAGA TACAAACCGC ACTCAATTTT AGCAAAAAAA AAAATTCAAA ATTTAACAAA ACGCAGGTTT   
  
  
+ AAACGCACAG CCAAACAGTC TGTTATGGCT GGTTTTTAAC TTTATTCTAC GACCAGATCA TAAAAAATGA   
  
  
+ TATCTTGACT AATTATGTGT CGATTAATTA AATTTACGAG TAAATATGTT AAACCAGTGT TCACGACTAT   
  
  
+ GCTTCTTGAA ACGACGTGGT ATTCATCATA TTTATTTACT TCTAGAGGTT TTTTGTATAA AAAAATAAAT   
  
  
+ AAAAAAAATG CATTATGAGA TTAGCCGGCT AGATAGCGGG ATTGAGCCAG AGCCTGAATT GCAGCTGCTT   
  
  
+ TAGATAGGGA TGGTGATGGA AAAGAAAGGG AAAATTTATC CAGCACGAAC AGAAAAGCAA AACGCCCTTG   
  
  
+ CCCGCAACAA GCCAGGACGC GTGGCGAAGG AGGCTTTAAA TGAATGCGAC AGATGTTATT ATTGAGGTTT   
  
  
+ GACTTGTTTC CCGGTTGTTA TTGTTTTTAT AAAAAAATTA ATTTGTTTTT TTTAAAAAAA GTTTACATTA   
  
  
+ TTTTAATATG TCGACGGAAA TAATAAATCT TAAAAAATAA AAAATATTAT TTTAATATAA TTTTAAATAA   
  
  
+ AAATATATTT TATATAATAT AGTTTGTGTT TTGTTTAAAT CATAAATTTA AAAATATTAG ATTAATAAAT   
  
  
+ TCACCCTATT TTTTTTATGA ATCCCAATTA GGATTTTATT TTTAAATATT AGTTAAGAAA AAATTATAAT   
  
  
+ TTATAATTTA TTAATAACCG TAACTATAAA AAACTACCGT AATACAAAAT CAAATGCAAA ATTCTCAATC   
  
  
+ TAAATTCATG TACTGTTCTG ACACATAATT AACTCCCCAT TCAGTTACTG TTTTTGTTTT TATCACTTGA   
  
  
+ GTTAATTCCT CTTTTTTTTT CTAGAAGAAA TTTTAATTTC ATTGTTTATA TAAATGGTAA AAAAACAAAA   
  
  
+ ATGAAAATCG TGAACCATCA AACATGTGCA TGCTAAATGC TGCATTTGCT ACCTATAATT GTATTTTCTT   
  
  
+ ATAATATTGT TTTGAAATAT TGAAAAAACA CTATTTCTTT TAATCTGTGT TTTTAGAGCT CTATTGATTG   
  
  
+ TTTTTTTTTT TTTTACACTG TGATGATAAA AACCAATAAC CACGCCTCAT TCTATTATTA TTATTATCAT   
  
  
+ TATCACAAGA AATTATAAAA AATTTATTGG ATTAAGGAAT GTTTAGAATT GCGTTTTAAA TTGTATTTTT   
  
  
+ AAAATTTTTG ATTTTTTATT TAAAATTAAT TTTTTAGATT ATTTTTATAT ATTGATATCT AAACTAATTT   
  
  
+ TAAAAAATTA AAAAATATAT TATTTTAATA TATTTTTAAA TAAAAAATAA TTTAAAAAAC AACTTCTATT   
  
  
+ ATACAGGATA AAATACCCAT TACCGATTTA ACATTTTGAG AAGAAAAATC TACATCCAAG GTTCTTATTC   
  
  
+ CTTGAGGACA TTATGGTCAT TACATATCCA GGACAAACCT TTGCTTGGAC TAAAATTCAA ACCAAATGGA   
  
  
+ ATTTCATAGC TGTCAAATAC AGTGAAGCTG GGGATCCATG GAAAGTCCCC TGCTTTACTC TGCAGCTATA   
  
  
+ AATACACCAA CTAGGACACA ACCATAAACC ACAGTAACC  

- GTTAGTTTAT AGTGCAGAAC GGATGTTCGT ACCAAAAGTT GACAGTTAAA GACGAACTTT CACGAAGGTA   
  
  
- TAATCGTGCT CCTGAGTCTT CCTATTGCTC TTATAGTTCG TTGAATACGG TCAGGAAACA TTAATCGTCT   
  
  
- CAGCTCGTCA AAATTTATTG AAAAGCTAGG AAGTACAAAT TTGACTCTTA CTACATATCT TGATCTTCTG   
  
  
- ACAGCCGTTA AAGAAAGTAA AACACACACA AACCGTCACA ATATCGCTAA CGAAAAGTTT ATCAAAAAGC   
  
  
- ACGGCTTTAT GTACGGTTAC TACAAAAAAA TAAAAAAATT TTAGTAAAAA TTATAGTTGT GTAGTTTTGC   
  
  
- TAGGTTTTCT ATGTTTGGCG TGAGTTAAAA TCGTTTTTTT TTTTAAGTTT TAAATTGTTT TGCGTCCAAA   
  
  
- TTTGCGTGTC GGTTTGTCAG ACAATACCGA CCAAAAATTG AAATAAGATG CTGGTCTAGT ATTTTTTACT   
  
  
- ATAGAACTGA TTAATACACA GCTAATTAAT TTAAATGCTC ATTTATACAA TTTGGTCACA AGTGCTGATA   
  
  
- CGAAGAACTT TGCTGCACCA TAAGTAGTAT AAATAAATGA AGATCTCCAA AAAACATATT TTTTTATTTA   
  
  
- TTTTTTTTAC GTAATACTCT AATCGGCCGA TCTATCGCCC TAACTCGGTC TCGGACTTAA CGTCGACGAA   
  
  
- ATCTATCCCT ACCACTACCT TTTCTTTCCC TTTTAAATAG GTCGTGCTTG TCTTTTCGTT TTGCGGGAAC   
  
  
- GGGCGTTGTT CGGTCCTGCG CACCGCTTCC TCCGAAATTT ACTTACGCTG TCTACAATAA TAACTCCAAA   
  
  
- CTGAACAAAG GGCCAACAAT AACAAAAATA TTTTTTTAAT TAAACAAAAA AAATTTTTTT CAAATGTAAT   
  
  
- AAAATTATAC AGCTGCCTTT ATTATTTAGA ATTTTTTATT TTTTATAATA AAATTATATT AAAATTTATT   
  
  
- TTTATATAAA ATATATTATA TCAAACACAA AACAAATTTA GTATTTAAAT TTTTATAATC TAATTATTTA   
  
  
- AGTGGGATAA AAAAAATACT TAGGGTTAAT CCTAAAATAA AAATTTATAA TCAATTCTTT TTTAATATTA   
  
  
- AATATTAAAT AATTATTGGC ATTGATATTT TTTGATGGCA TTATGTTTTA GTTTACGTTT TAAGAGTTAG   
  
  
- ATTTAAGTAC ATGACAAGAC TGTGTATTAA TTGAGGGGTA AGTCAATGAC AAAAACAAAA ATAGTGAACT   
  
  
- CAATTAAGGA GAAAAAAAAA GATCTTCTTT AAAATTAAAG TAACAAATAT ATTTACCATT TTTTTGTTTT   
  
  
- TACTTTTAGC ACTTGGTAGT TTGTACACGT ACGATTTACG ACGTAAACGA TGGATATTAA CATAAAAGAA   
  
  
- TATTATAACA AAACTTTATA ACTTTTTTGT GATAAAGAAA ATTAGACACA AAAATCTCGA GATAACTAAC   
  
  
- AAAAAAAAAA AAAATGTGAC ACTACTATTT TTGGTTATTG GTGCGGAGTA AGATAATAAT AATAATAGTA   
  
  
- ATAGTGTTCT TTAATATTTT TTAAATAACC TAATTCCTTA CAAATCTTAA CGCAAAATTT AACATAAAAA   
  
  
- TTTTAAAAAC TAAAAAATAA ATTTTAATTA AAAAATCTAA TAAAAATATA TAACTATAGA TTTGATTAAA   
  
  
- ATTTTTTAAT TTTTTATATA ATAAAATTAT ATAAAAATTT ATTTTTTATT AAATTTTTTG TTGAAGATAA   
  
  
- TATGTCCTAT TTTATGGGTA ATGGCTAAAT TGTAAAACTC TTCTTTTTAG ATGTAGGTTC CAAGAATAAG   
  
  
- GAACTCCTGT AATACCAGTA ATGTATAGGT CCTGTTTGGA AACGAACCTG ATTTTAAGTT TGGTTTACCT   
  
  
- TAAAGTATCG ACAGTTTATG TCACTTCGAC CCCTAGGTAC CTTTCAGGGG ACGAAATGAG ACGTCGATAT   
  
  
- TTATGTGGTT GATCCTGTGT TGGTATTTGG TGTCATTGG

+     GATA-motif

| Site Name | Organism | Position | Strand | Matrix score. | sequence | function |
| --- | --- | --- | --- | --- | --- | --- |
| GATA-motif | Solanum tuberosum | 89 | + | 9 | AAGGATAAGG | part of a light responsive element |
| GATA-motif | Pisum sativum | 703 | + | 7 | GATAGGG | part of a light responsive element |

>Potri.003G139300.1   
+ CAATCAAATA TCACGTCTTG CCTACAAGCA TGGTTTTCAA CTGTCAATTT CTGCTTGAAA GTGCTTCCAT   
  
  
+ ATTAGCACGA GGACTCAGAA GGATAACGAG AATATCAAGC AACTTATGCC AGTCCTTTGT AATTAGCAGA   
  
  
+ GTCGAGCAGT TTTAAATAAC TTTTCGATCC TTCATGTTTA AACTGAGAAT GATGTATAGA ACTAGAAGAC   
  
  
+ TGTCGGCAAT TTCTTTCATT TTGTGTGTGT TTGGCAGTGT TATAGCGATT GCTTTTCAAA TAGTTTTTCG   
  
  
+ TGCCGAAATA CATGCCAATG ATGTTTTTTT ATTTTTTTAA AATCATTTTT AATATCAACA CATCAAAACG   
  
  
+ ATCCAAAAGA TACAAACCGC ACTCAATTTT AGCAAAAAAA AAAATTCAAA ATTTAACAAA ACGCAGGTTT   
  
  
+ AAACGCACAG CCAAACAGTC TGTTATGGCT GGTTTTTAAC TTTATTCTAC GACCAGATCA TAAAAAATGA   
  
  
+ TATCTTGACT AATTATGTGT CGATTAATTA AATTTACGAG TAAATATGTT AAACCAGTGT TCACGACTAT   
  
  
+ GCTTCTTGAA ACGACGTGGT ATTCATCATA TTTATTTACT TCTAGAGGTT TTTTGTATAA AAAAATAAAT   
  
  
+ AAAAAAAATG CATTATGAGA TTAGCCGGCT AGATAGCGGG ATTGAGCCAG AGCCTGAATT GCAGCTGCTT   
  
  
+ TAGATAGGGA TGGTGATGGA AAAGAAAGGG AAAATTTATC CAGCACGAAC AGAAAAGCAA AACGCCCTTG   
  
  
+ CCCGCAACAA GCCAGGACGC GTGGCGAAGG AGGCTTTAAA TGAATGCGAC AGATGTTATT ATTGAGGTTT   
  
  
+ GACTTGTTTC CCGGTTGTTA TTGTTTTTAT AAAAAAATTA ATTTGTTTTT TTTAAAAAAA GTTTACATTA   
  
  
+ TTTTAATATG TCGACGGAAA TAATAAATCT TAAAAAATAA AAAATATTAT TTTAATATAA TTTTAAATAA   
  
  
+ AAATATATTT TATATAATAT AGTTTGTGTT TTGTTTAAAT CATAAATTTA AAAATATTAG ATTAATAAAT   
  
  
+ TCACCCTATT TTTTTTATGA ATCCCAATTA GGATTTTATT TTTAAATATT AGTTAAGAAA AAATTATAAT   
  
  
+ TTATAATTTA TTAATAACCG TAACTATAAA AAACTACCGT AATACAAAAT CAAATGCAAA ATTCTCAATC   
  
  
+ TAAATTCATG TACTGTTCTG ACACATAATT AACTCCCCAT TCAGTTACTG TTTTTGTTTT TATCACTTGA   
  
  
+ GTTAATTCCT CTTTTTTTTT CTAGAAGAAA TTTTAATTTC ATTGTTTATA TAAATGGTAA AAAAACAAAA   
  
  
+ ATGAAAATCG TGAACCATCA AACATGTGCA TGCTAAATGC TGCATTTGCT ACCTATAATT GTATTTTCTT   
  
  
+ ATAATATTGT TTTGAAATAT TGAAAAAACA CTATTTCTTT TAATCTGTGT TTTTAGAGCT CTATTGATTG   
  
  
+ TTTTTTTTTT TTTTACACTG TGATGATAAA AACCAATAAC CACGCCTCAT TCTATTATTA TTATTATCAT   
  
  
+ TATCACAAGA AATTATAAAA AATTTATTGG ATTAAGGAAT GTTTAGAATT GCGTTTTAAA TTGTATTTTT   
  
  
+ AAAATTTTTG ATTTTTTATT TAAAATTAAT TTTTTAGATT ATTTTTATAT ATTGATATCT AAACTAATTT   
  
  
+ TAAAAAATTA AAAAATATAT TATTTTAATA TATTTTTAAA TAAAAAATAA TTTAAAAAAC AACTTCTATT   
  
  
+ ATACAGGATA AAATACCCAT TACCGATTTA ACATTTTGAG AAGAAAAATC TACATCCAAG GTTCTTATTC   
  
  
+ CTTGAGGACA TTATGGTCAT TACATATCCA GGACAAACCT TTGCTTGGAC TAAAATTCAA ACCAAATGGA   
  
  
+ ATTTCATAGC TGTCAAATAC AGTGAAGCTG GGGATCCATG GAAAGTCCCC TGCTTTACTC TGCAGCTATA   
  
  
+ AATACACCAA CTAGGACACA ACCATAAACC ACAGTAACC  

- GTTAGTTTAT AGTGCAGAAC GGATGTTCGT ACCAAAAGTT GACAGTTAAA GACGAACTTT CACGAAGGTA   
  
  
- TAATCGTGCT CCTGAGTCTT CCTATTGCTC TTATAGTTCG TTGAATACGG TCAGGAAACA TTAATCGTCT   
  
  
- CAGCTCGTCA AAATTTATTG AAAAGCTAGG AAGTACAAAT TTGACTCTTA CTACATATCT TGATCTTCTG   
  
  
- ACAGCCGTTA AAGAAAGTAA AACACACACA AACCGTCACA ATATCGCTAA CGAAAAGTTT ATCAAAAAGC   
  
  
- ACGGCTTTAT GTACGGTTAC TACAAAAAAA TAAAAAAATT TTAGTAAAAA TTATAGTTGT GTAGTTTTGC   
  
  
- TAGGTTTTCT ATGTTTGGCG TGAGTTAAAA TCGTTTTTTT TTTTAAGTTT TAAATTGTTT TGCGTCCAAA   
  
  
- TTTGCGTGTC GGTTTGTCAG ACAATACCGA CCAAAAATTG AAATAAGATG CTGGTCTAGT ATTTTTTACT   
  
  
- ATAGAACTGA TTAATACACA GCTAATTAAT TTAAATGCTC ATTTATACAA TTTGGTCACA AGTGCTGATA   
  
  
- CGAAGAACTT TGCTGCACCA TAAGTAGTAT AAATAAATGA AGATCTCCAA AAAACATATT TTTTTATTTA   
  
  
- TTTTTTTTAC GTAATACTCT AATCGGCCGA TCTATCGCCC TAACTCGGTC TCGGACTTAA CGTCGACGAA   
  
  
- ATCTATCCCT ACCACTACCT TTTCTTTCCC TTTTAAATAG GTCGTGCTTG TCTTTTCGTT TTGCGGGAAC   
  
  
- GGGCGTTGTT CGGTCCTGCG CACCGCTTCC TCCGAAATTT ACTTACGCTG TCTACAATAA TAACTCCAAA   
  
  
- CTGAACAAAG GGCCAACAAT AACAAAAATA TTTTTTTAAT TAAACAAAAA AAATTTTTTT CAAATGTAAT   
  
  
- AAAATTATAC AGCTGCCTTT ATTATTTAGA ATTTTTTATT TTTTATAATA AAATTATATT AAAATTTATT   
  
  
- TTTATATAAA ATATATTATA TCAAACACAA AACAAATTTA GTATTTAAAT TTTTATAATC TAATTATTTA   
  
  
- AGTGGGATAA AAAAAATACT TAGGGTTAAT CCTAAAATAA AAATTTATAA TCAATTCTTT TTTAATATTA   
  
  
- AATATTAAAT AATTATTGGC ATTGATATTT TTTGATGGCA TTATGTTTTA GTTTACGTTT TAAGAGTTAG   
  
  
- ATTTAAGTAC ATGACAAGAC TGTGTATTAA TTGAGGGGTA AGTCAATGAC AAAAACAAAA ATAGTGAACT   
  
  
- CAATTAAGGA GAAAAAAAAA GATCTTCTTT AAAATTAAAG TAACAAATAT ATTTACCATT TTTTTGTTTT   
  
  
- TACTTTTAGC ACTTGGTAGT TTGTACACGT ACGATTTACG ACGTAAACGA TGGATATTAA CATAAAAGAA   
  
  
- TATTATAACA AAACTTTATA ACTTTTTTGT GATAAAGAAA ATTAGACACA AAAATCTCGA GATAACTAAC   
  
  
- AAAAAAAAAA AAAATGTGAC ACTACTATTT TTGGTTATTG GTGCGGAGTA AGATAATAAT AATAATAGTA   
  
  
- ATAGTGTTCT TTAATATTTT TTAAATAACC TAATTCCTTA CAAATCTTAA CGCAAAATTT AACATAAAAA   
  
  
- TTTTAAAAAC TAAAAAATAA ATTTTAATTA AAAAATCTAA TAAAAATATA TAACTATAGA TTTGATTAAA   
  
  
- ATTTTTTAAT TTTTTATATA ATAAAATTAT ATAAAAATTT ATTTTTTATT AAATTTTTTG TTGAAGATAA   
  
  
- TATGTCCTAT TTTATGGGTA ATGGCTAAAT TGTAAAACTC TTCTTTTTAG ATGTAGGTTC CAAGAATAAG   
  
  
- GAACTCCTGT AATACCAGTA ATGTATAGGT CCTGTTTGGA AACGAACCTG ATTTTAAGTT TGGTTTACCT   
  
  
- TAAAGTATCG ACAGTTTATG TCACTTCGAC CCCTAGGTAC CTTTCAGGGG ACGAAATGAG ACGTCGATAT   
  
  
- TTATGTGGTT GATCCTGTGT TGGTATTTGG TGTCATTGG

+     LTR

| Site Name | Organism | Position | Strand | Matrix score. | sequence | function |
| --- | --- | --- | --- | --- | --- | --- |
| LTR | Hordeum vulgare | 283 | + | 6 | CCGAAA | cis-acting element involved in low-temperature responsiveness |

>Potri.003G139300.1   
+ CAATCAAATA TCACGTCTTG CCTACAAGCA TGGTTTTCAA CTGTCAATTT CTGCTTGAAA GTGCTTCCAT   
  
  
+ ATTAGCACGA GGACTCAGAA GGATAACGAG AATATCAAGC AACTTATGCC AGTCCTTTGT AATTAGCAGA   
  
  
+ GTCGAGCAGT TTTAAATAAC TTTTCGATCC TTCATGTTTA AACTGAGAAT GATGTATAGA ACTAGAAGAC   
  
  
+ TGTCGGCAAT TTCTTTCATT TTGTGTGTGT TTGGCAGTGT TATAGCGATT GCTTTTCAAA TAGTTTTTCG   
  
  
+ TGCCGAAATA CATGCCAATG ATGTTTTTTT ATTTTTTTAA AATCATTTTT AATATCAACA CATCAAAACG   
  
  
+ ATCCAAAAGA TACAAACCGC ACTCAATTTT AGCAAAAAAA AAAATTCAAA ATTTAACAAA ACGCAGGTTT   
  
  
+ AAACGCACAG CCAAACAGTC TGTTATGGCT GGTTTTTAAC TTTATTCTAC GACCAGATCA TAAAAAATGA   
  
  
+ TATCTTGACT AATTATGTGT CGATTAATTA AATTTACGAG TAAATATGTT AAACCAGTGT TCACGACTAT   
  
  
+ GCTTCTTGAA ACGACGTGGT ATTCATCATA TTTATTTACT TCTAGAGGTT TTTTGTATAA AAAAATAAAT   
  
  
+ AAAAAAAATG CATTATGAGA TTAGCCGGCT AGATAGCGGG ATTGAGCCAG AGCCTGAATT GCAGCTGCTT   
  
  
+ TAGATAGGGA TGGTGATGGA AAAGAAAGGG AAAATTTATC CAGCACGAAC AGAAAAGCAA AACGCCCTTG   
  
  
+ CCCGCAACAA GCCAGGACGC GTGGCGAAGG AGGCTTTAAA TGAATGCGAC AGATGTTATT ATTGAGGTTT   
  
  
+ GACTTGTTTC CCGGTTGTTA TTGTTTTTAT AAAAAAATTA ATTTGTTTTT TTTAAAAAAA GTTTACATTA   
  
  
+ TTTTAATATG TCGACGGAAA TAATAAATCT TAAAAAATAA AAAATATTAT TTTAATATAA TTTTAAATAA   
  
  
+ AAATATATTT TATATAATAT AGTTTGTGTT TTGTTTAAAT CATAAATTTA AAAATATTAG ATTAATAAAT   
  
  
+ TCACCCTATT TTTTTTATGA ATCCCAATTA GGATTTTATT TTTAAATATT AGTTAAGAAA AAATTATAAT   
  
  
+ TTATAATTTA TTAATAACCG TAACTATAAA AAACTACCGT AATACAAAAT CAAATGCAAA ATTCTCAATC   
  
  
+ TAAATTCATG TACTGTTCTG ACACATAATT AACTCCCCAT TCAGTTACTG TTTTTGTTTT TATCACTTGA   
  
  
+ GTTAATTCCT CTTTTTTTTT CTAGAAGAAA TTTTAATTTC ATTGTTTATA TAAATGGTAA AAAAACAAAA   
  
  
+ ATGAAAATCG TGAACCATCA AACATGTGCA TGCTAAATGC TGCATTTGCT ACCTATAATT GTATTTTCTT   
  
  
+ ATAATATTGT TTTGAAATAT TGAAAAAACA CTATTTCTTT TAATCTGTGT TTTTAGAGCT CTATTGATTG   
  
  
+ TTTTTTTTTT TTTTACACTG TGATGATAAA AACCAATAAC CACGCCTCAT TCTATTATTA TTATTATCAT   
  
  
+ TATCACAAGA AATTATAAAA AATTTATTGG ATTAAGGAAT GTTTAGAATT GCGTTTTAAA TTGTATTTTT   
  
  
+ AAAATTTTTG ATTTTTTATT TAAAATTAAT TTTTTAGATT ATTTTTATAT ATTGATATCT AAACTAATTT   
  
  
+ TAAAAAATTA AAAAATATAT TATTTTAATA TATTTTTAAA TAAAAAATAA TTTAAAAAAC AACTTCTATT   
  
  
+ ATACAGGATA AAATACCCAT TACCGATTTA ACATTTTGAG AAGAAAAATC TACATCCAAG GTTCTTATTC   
  
  
+ CTTGAGGACA TTATGGTCAT TACATATCCA GGACAAACCT TTGCTTGGAC TAAAATTCAA ACCAAATGGA   
  
  
+ ATTTCATAGC TGTCAAATAC AGTGAAGCTG GGGATCCATG GAAAGTCCCC TGCTTTACTC TGCAGCTATA   
  
  
+ AATACACCAA CTAGGACACA ACCATAAACC ACAGTAACC  

- GTTAGTTTAT AGTGCAGAAC GGATGTTCGT ACCAAAAGTT GACAGTTAAA GACGAACTTT CACGAAGGTA   
  
  
- TAATCGTGCT CCTGAGTCTT CCTATTGCTC TTATAGTTCG TTGAATACGG TCAGGAAACA TTAATCGTCT   
  
  
- CAGCTCGTCA AAATTTATTG AAAAGCTAGG AAGTACAAAT TTGACTCTTA CTACATATCT TGATCTTCTG   
  
  
- ACAGCCGTTA AAGAAAGTAA AACACACACA AACCGTCACA ATATCGCTAA CGAAAAGTTT ATCAAAAAGC   
  
  
- ACGGCTTTAT GTACGGTTAC TACAAAAAAA TAAAAAAATT TTAGTAAAAA TTATAGTTGT GTAGTTTTGC   
  
  
- TAGGTTTTCT ATGTTTGGCG TGAGTTAAAA TCGTTTTTTT TTTTAAGTTT TAAATTGTTT TGCGTCCAAA   
  
  
- TTTGCGTGTC GGTTTGTCAG ACAATACCGA CCAAAAATTG AAATAAGATG CTGGTCTAGT ATTTTTTACT   
  
  
- ATAGAACTGA TTAATACACA GCTAATTAAT TTAAATGCTC ATTTATACAA TTTGGTCACA AGTGCTGATA   
  
  
- CGAAGAACTT TGCTGCACCA TAAGTAGTAT AAATAAATGA AGATCTCCAA AAAACATATT TTTTTATTTA   
  
  
- TTTTTTTTAC GTAATACTCT AATCGGCCGA TCTATCGCCC TAACTCGGTC TCGGACTTAA CGTCGACGAA   
  
  
- ATCTATCCCT ACCACTACCT TTTCTTTCCC TTTTAAATAG GTCGTGCTTG TCTTTTCGTT TTGCGGGAAC   
  
  
- GGGCGTTGTT CGGTCCTGCG CACCGCTTCC TCCGAAATTT ACTTACGCTG TCTACAATAA TAACTCCAAA   
  
  
- CTGAACAAAG GGCCAACAAT AACAAAAATA TTTTTTTAAT TAAACAAAAA AAATTTTTTT CAAATGTAAT   
  
  
- AAAATTATAC AGCTGCCTTT ATTATTTAGA ATTTTTTATT TTTTATAATA AAATTATATT AAAATTTATT   
  
  
- TTTATATAAA ATATATTATA TCAAACACAA AACAAATTTA GTATTTAAAT TTTTATAATC TAATTATTTA   
  
  
- AGTGGGATAA AAAAAATACT TAGGGTTAAT CCTAAAATAA AAATTTATAA TCAATTCTTT TTTAATATTA   
  
  
- AATATTAAAT AATTATTGGC ATTGATATTT TTTGATGGCA TTATGTTTTA GTTTACGTTT TAAGAGTTAG   
  
  
- ATTTAAGTAC ATGACAAGAC TGTGTATTAA TTGAGGGGTA AGTCAATGAC AAAAACAAAA ATAGTGAACT   
  
  
- CAATTAAGGA GAAAAAAAAA GATCTTCTTT AAAATTAAAG TAACAAATAT ATTTACCATT TTTTTGTTTT   
  
  
- TACTTTTAGC ACTTGGTAGT TTGTACACGT ACGATTTACG ACGTAAACGA TGGATATTAA CATAAAAGAA   
  
  
- TATTATAACA AAACTTTATA ACTTTTTTGT GATAAAGAAA ATTAGACACA AAAATCTCGA GATAACTAAC   
  
  
- AAAAAAAAAA AAAATGTGAC ACTACTATTT TTGGTTATTG GTGCGGAGTA AGATAATAAT AATAATAGTA   
  
  
- ATAGTGTTCT TTAATATTTT TTAAATAACC TAATTCCTTA CAAATCTTAA CGCAAAATTT AACATAAAAA   
  
  
- TTTTAAAAAC TAAAAAATAA ATTTTAATTA AAAAATCTAA TAAAAATATA TAACTATAGA TTTGATTAAA   
  
  
- ATTTTTTAAT TTTTTATATA ATAAAATTAT ATAAAAATTT ATTTTTTATT AAATTTTTTG TTGAAGATAA   
  
  
- TATGTCCTAT TTTATGGGTA ATGGCTAAAT TGTAAAACTC TTCTTTTTAG ATGTAGGTTC CAAGAATAAG   
  
  
- GAACTCCTGT AATACCAGTA ATGTATAGGT CCTGTTTGGA AACGAACCTG ATTTTAAGTT TGGTTTACCT   
  
  
- TAAAGTATCG ACAGTTTATG TCACTTCGAC CCCTAGGTAC CTTTCAGGGG ACGAAATGAG ACGTCGATAT   
  
  
- TTATGTGGTT GATCCTGTGT TGGTATTTGG TGTCATTGG

+     MBS

| Site Name | Organism | Position | Strand | Matrix score. | sequence | function |
| --- | --- | --- | --- | --- | --- | --- |
| MBS | Arabidopsis thaliana | 38 | + | 6 | CAACTG | MYB binding site involved in drought-inducibility |

>Potri.003G139300.1   
+ CAATCAAATA TCACGTCTTG CCTACAAGCA TGGTTTTCAA CTGTCAATTT CTGCTTGAAA GTGCTTCCAT   
  
  
+ ATTAGCACGA GGACTCAGAA GGATAACGAG AATATCAAGC AACTTATGCC AGTCCTTTGT AATTAGCAGA   
  
  
+ GTCGAGCAGT TTTAAATAAC TTTTCGATCC TTCATGTTTA AACTGAGAAT GATGTATAGA ACTAGAAGAC   
  
  
+ TGTCGGCAAT TTCTTTCATT TTGTGTGTGT TTGGCAGTGT TATAGCGATT GCTTTTCAAA TAGTTTTTCG   
  
  
+ TGCCGAAATA CATGCCAATG ATGTTTTTTT ATTTTTTTAA AATCATTTTT AATATCAACA CATCAAAACG   
  
  
+ ATCCAAAAGA TACAAACCGC ACTCAATTTT AGCAAAAAAA AAAATTCAAA ATTTAACAAA ACGCAGGTTT   
  
  
+ AAACGCACAG CCAAACAGTC TGTTATGGCT GGTTTTTAAC TTTATTCTAC GACCAGATCA TAAAAAATGA   
  
  
+ TATCTTGACT AATTATGTGT CGATTAATTA AATTTACGAG TAAATATGTT AAACCAGTGT TCACGACTAT   
  
  
+ GCTTCTTGAA ACGACGTGGT ATTCATCATA TTTATTTACT TCTAGAGGTT TTTTGTATAA AAAAATAAAT   
  
  
+ AAAAAAAATG CATTATGAGA TTAGCCGGCT AGATAGCGGG ATTGAGCCAG AGCCTGAATT GCAGCTGCTT   
  
  
+ TAGATAGGGA TGGTGATGGA AAAGAAAGGG AAAATTTATC CAGCACGAAC AGAAAAGCAA AACGCCCTTG   
  
  
+ CCCGCAACAA GCCAGGACGC GTGGCGAAGG AGGCTTTAAA TGAATGCGAC AGATGTTATT ATTGAGGTTT   
  
  
+ GACTTGTTTC CCGGTTGTTA TTGTTTTTAT AAAAAAATTA ATTTGTTTTT TTTAAAAAAA GTTTACATTA   
  
  
+ TTTTAATATG TCGACGGAAA TAATAAATCT TAAAAAATAA AAAATATTAT TTTAATATAA TTTTAAATAA   
  
  
+ AAATATATTT TATATAATAT AGTTTGTGTT TTGTTTAAAT CATAAATTTA AAAATATTAG ATTAATAAAT   
  
  
+ TCACCCTATT TTTTTTATGA ATCCCAATTA GGATTTTATT TTTAAATATT AGTTAAGAAA AAATTATAAT   
  
  
+ TTATAATTTA TTAATAACCG TAACTATAAA AAACTACCGT AATACAAAAT CAAATGCAAA ATTCTCAATC   
  
  
+ TAAATTCATG TACTGTTCTG ACACATAATT AACTCCCCAT TCAGTTACTG TTTTTGTTTT TATCACTTGA   
  
  
+ GTTAATTCCT CTTTTTTTTT CTAGAAGAAA TTTTAATTTC ATTGTTTATA TAAATGGTAA AAAAACAAAA   
  
  
+ ATGAAAATCG TGAACCATCA AACATGTGCA TGCTAAATGC TGCATTTGCT ACCTATAATT GTATTTTCTT   
  
  
+ ATAATATTGT TTTGAAATAT TGAAAAAACA CTATTTCTTT TAATCTGTGT TTTTAGAGCT CTATTGATTG   
  
  
+ TTTTTTTTTT TTTTACACTG TGATGATAAA AACCAATAAC CACGCCTCAT TCTATTATTA TTATTATCAT   
  
  
+ TATCACAAGA AATTATAAAA AATTTATTGG ATTAAGGAAT GTTTAGAATT GCGTTTTAAA TTGTATTTTT   
  
  
+ AAAATTTTTG ATTTTTTATT TAAAATTAAT TTTTTAGATT ATTTTTATAT ATTGATATCT AAACTAATTT   
  
  
+ TAAAAAATTA AAAAATATAT TATTTTAATA TATTTTTAAA TAAAAAATAA TTTAAAAAAC AACTTCTATT   
  
  
+ ATACAGGATA AAATACCCAT TACCGATTTA ACATTTTGAG AAGAAAAATC TACATCCAAG GTTCTTATTC   
  
  
+ CTTGAGGACA TTATGGTCAT TACATATCCA GGACAAACCT TTGCTTGGAC TAAAATTCAA ACCAAATGGA   
  
  
+ ATTTCATAGC TGTCAAATAC AGTGAAGCTG GGGATCCATG GAAAGTCCCC TGCTTTACTC TGCAGCTATA   
  
  
+ AATACACCAA CTAGGACACA ACCATAAACC ACAGTAACC  

- GTTAGTTTAT AGTGCAGAAC GGATGTTCGT ACCAAAAGTT GACAGTTAAA GACGAACTTT CACGAAGGTA   
  
  
- TAATCGTGCT CCTGAGTCTT CCTATTGCTC TTATAGTTCG TTGAATACGG TCAGGAAACA TTAATCGTCT   
  
  
- CAGCTCGTCA AAATTTATTG AAAAGCTAGG AAGTACAAAT TTGACTCTTA CTACATATCT TGATCTTCTG   
  
  
- ACAGCCGTTA AAGAAAGTAA AACACACACA AACCGTCACA ATATCGCTAA CGAAAAGTTT ATCAAAAAGC   
  
  
- ACGGCTTTAT GTACGGTTAC TACAAAAAAA TAAAAAAATT TTAGTAAAAA TTATAGTTGT GTAGTTTTGC   
  
  
- TAGGTTTTCT ATGTTTGGCG TGAGTTAAAA TCGTTTTTTT TTTTAAGTTT TAAATTGTTT TGCGTCCAAA   
  
  
- TTTGCGTGTC GGTTTGTCAG ACAATACCGA CCAAAAATTG AAATAAGATG CTGGTCTAGT ATTTTTTACT   
  
  
- ATAGAACTGA TTAATACACA GCTAATTAAT TTAAATGCTC ATTTATACAA TTTGGTCACA AGTGCTGATA   
  
  
- CGAAGAACTT TGCTGCACCA TAAGTAGTAT AAATAAATGA AGATCTCCAA AAAACATATT TTTTTATTTA   
  
  
- TTTTTTTTAC GTAATACTCT AATCGGCCGA TCTATCGCCC TAACTCGGTC TCGGACTTAA CGTCGACGAA   
  
  
- ATCTATCCCT ACCACTACCT TTTCTTTCCC TTTTAAATAG GTCGTGCTTG TCTTTTCGTT TTGCGGGAAC   
  
  
- GGGCGTTGTT CGGTCCTGCG CACCGCTTCC TCCGAAATTT ACTTACGCTG TCTACAATAA TAACTCCAAA   
  
  
- CTGAACAAAG GGCCAACAAT AACAAAAATA TTTTTTTAAT TAAACAAAAA AAATTTTTTT CAAATGTAAT   
  
  
- AAAATTATAC AGCTGCCTTT ATTATTTAGA ATTTTTTATT TTTTATAATA AAATTATATT AAAATTTATT   
  
  
- TTTATATAAA ATATATTATA TCAAACACAA AACAAATTTA GTATTTAAAT TTTTATAATC TAATTATTTA   
  
  
- AGTGGGATAA AAAAAATACT TAGGGTTAAT CCTAAAATAA AAATTTATAA TCAATTCTTT TTTAATATTA   
  
  
- AATATTAAAT AATTATTGGC ATTGATATTT TTTGATGGCA TTATGTTTTA GTTTACGTTT TAAGAGTTAG   
  
  
- ATTTAAGTAC ATGACAAGAC TGTGTATTAA TTGAGGGGTA AGTCAATGAC AAAAACAAAA ATAGTGAACT   
  
  
- CAATTAAGGA GAAAAAAAAA GATCTTCTTT AAAATTAAAG TAACAAATAT ATTTACCATT TTTTTGTTTT   
  
  
- TACTTTTAGC ACTTGGTAGT TTGTACACGT ACGATTTACG ACGTAAACGA TGGATATTAA CATAAAAGAA   
  
  
- TATTATAACA AAACTTTATA ACTTTTTTGT GATAAAGAAA ATTAGACACA AAAATCTCGA GATAACTAAC   
  
  
- AAAAAAAAAA AAAATGTGAC ACTACTATTT TTGGTTATTG GTGCGGAGTA AGATAATAAT AATAATAGTA   
  
  
- ATAGTGTTCT TTAATATTTT TTAAATAACC TAATTCCTTA CAAATCTTAA CGCAAAATTT AACATAAAAA   
  
  
- TTTTAAAAAC TAAAAAATAA ATTTTAATTA AAAAATCTAA TAAAAATATA TAACTATAGA TTTGATTAAA   
  
  
- ATTTTTTAAT TTTTTATATA ATAAAATTAT ATAAAAATTT ATTTTTTATT AAATTTTTTG TTGAAGATAA   
  
  
- TATGTCCTAT TTTATGGGTA ATGGCTAAAT TGTAAAACTC TTCTTTTTAG ATGTAGGTTC CAAGAATAAG   
  
  
- GAACTCCTGT AATACCAGTA ATGTATAGGT CCTGTTTGGA AACGAACCTG ATTTTAAGTT TGGTTTACCT   
  
  
- TAAAGTATCG ACAGTTTATG TCACTTCGAC CCCTAGGTAC CTTTCAGGGG ACGAAATGAG ACGTCGATAT   
  
  
- TTATGTGGTT GATCCTGTGT TGGTATTTGG TGTCATTGG

+     MYB

| Site Name | Organism | Position | Strand | Matrix score. | sequence | function |
| --- | --- | --- | --- | --- | --- | --- |
| MYB | Arabidopsis thaliana | 1979 | + | 6 | CAACCA |  |
| MYB | Arabidopsis thaliana | 1507 | + | 6 | TAACCA |  |

>Potri.003G139300.1   
+ CAATCAAATA TCACGTCTTG CCTACAAGCA TGGTTTTCAA CTGTCAATTT CTGCTTGAAA GTGCTTCCAT   
  
  
+ ATTAGCACGA GGACTCAGAA GGATAACGAG AATATCAAGC AACTTATGCC AGTCCTTTGT AATTAGCAGA   
  
  
+ GTCGAGCAGT TTTAAATAAC TTTTCGATCC TTCATGTTTA AACTGAGAAT GATGTATAGA ACTAGAAGAC   
  
  
+ TGTCGGCAAT TTCTTTCATT TTGTGTGTGT TTGGCAGTGT TATAGCGATT GCTTTTCAAA TAGTTTTTCG   
  
  
+ TGCCGAAATA CATGCCAATG ATGTTTTTTT ATTTTTTTAA AATCATTTTT AATATCAACA CATCAAAACG   
  
  
+ ATCCAAAAGA TACAAACCGC ACTCAATTTT AGCAAAAAAA AAAATTCAAA ATTTAACAAA ACGCAGGTTT   
  
  
+ AAACGCACAG CCAAACAGTC TGTTATGGCT GGTTTTTAAC TTTATTCTAC GACCAGATCA TAAAAAATGA   
  
  
+ TATCTTGACT AATTATGTGT CGATTAATTA AATTTACGAG TAAATATGTT AAACCAGTGT TCACGACTAT   
  
  
+ GCTTCTTGAA ACGACGTGGT ATTCATCATA TTTATTTACT TCTAGAGGTT TTTTGTATAA AAAAATAAAT   
  
  
+ AAAAAAAATG CATTATGAGA TTAGCCGGCT AGATAGCGGG ATTGAGCCAG AGCCTGAATT GCAGCTGCTT   
  
  
+ TAGATAGGGA TGGTGATGGA AAAGAAAGGG AAAATTTATC CAGCACGAAC AGAAAAGCAA AACGCCCTTG   
  
  
+ CCCGCAACAA GCCAGGACGC GTGGCGAAGG AGGCTTTAAA TGAATGCGAC AGATGTTATT ATTGAGGTTT   
  
  
+ GACTTGTTTC CCGGTTGTTA TTGTTTTTAT AAAAAAATTA ATTTGTTTTT TTTAAAAAAA GTTTACATTA   
  
  
+ TTTTAATATG TCGACGGAAA TAATAAATCT TAAAAAATAA AAAATATTAT TTTAATATAA TTTTAAATAA   
  
  
+ AAATATATTT TATATAATAT AGTTTGTGTT TTGTTTAAAT CATAAATTTA AAAATATTAG ATTAATAAAT   
  
  
+ TCACCCTATT TTTTTTATGA ATCCCAATTA GGATTTTATT TTTAAATATT AGTTAAGAAA AAATTATAAT   
  
  
+ TTATAATTTA TTAATAACCG TAACTATAAA AAACTACCGT AATACAAAAT CAAATGCAAA ATTCTCAATC   
  
  
+ TAAATTCATG TACTGTTCTG ACACATAATT AACTCCCCAT TCAGTTACTG TTTTTGTTTT TATCACTTGA   
  
  
+ GTTAATTCCT CTTTTTTTTT CTAGAAGAAA TTTTAATTTC ATTGTTTATA TAAATGGTAA AAAAACAAAA   
  
  
+ ATGAAAATCG TGAACCATCA AACATGTGCA TGCTAAATGC TGCATTTGCT ACCTATAATT GTATTTTCTT   
  
  
+ ATAATATTGT TTTGAAATAT TGAAAAAACA CTATTTCTTT TAATCTGTGT TTTTAGAGCT CTATTGATTG   
  
  
+ TTTTTTTTTT TTTTACACTG TGATGATAAA AACCAATAAC CACGCCTCAT TCTATTATTA TTATTATCAT   
  
  
+ TATCACAAGA AATTATAAAA AATTTATTGG ATTAAGGAAT GTTTAGAATT GCGTTTTAAA TTGTATTTTT   
  
  
+ AAAATTTTTG ATTTTTTATT TAAAATTAAT TTTTTAGATT ATTTTTATAT ATTGATATCT AAACTAATTT   
  
  
+ TAAAAAATTA AAAAATATAT TATTTTAATA TATTTTTAAA TAAAAAATAA TTTAAAAAAC AACTTCTATT   
  
  
+ ATACAGGATA AAATACCCAT TACCGATTTA ACATTTTGAG AAGAAAAATC TACATCCAAG GTTCTTATTC   
  
  
+ CTTGAGGACA TTATGGTCAT TACATATCCA GGACAAACCT TTGCTTGGAC TAAAATTCAA ACCAAATGGA   
  
  
+ ATTTCATAGC TGTCAAATAC AGTGAAGCTG GGGATCCATG GAAAGTCCCC TGCTTTACTC TGCAGCTATA   
  
  
+ AATACACCAA CTAGGACACA ACCATAAACC ACAGTAACC  

- GTTAGTTTAT AGTGCAGAAC GGATGTTCGT ACCAAAAGTT GACAGTTAAA GACGAACTTT CACGAAGGTA   
  
  
- TAATCGTGCT CCTGAGTCTT CCTATTGCTC TTATAGTTCG TTGAATACGG TCAGGAAACA TTAATCGTCT   
  
  
- CAGCTCGTCA AAATTTATTG AAAAGCTAGG AAGTACAAAT TTGACTCTTA CTACATATCT TGATCTTCTG   
  
  
- ACAGCCGTTA AAGAAAGTAA AACACACACA AACCGTCACA ATATCGCTAA CGAAAAGTTT ATCAAAAAGC   
  
  
- ACGGCTTTAT GTACGGTTAC TACAAAAAAA TAAAAAAATT TTAGTAAAAA TTATAGTTGT GTAGTTTTGC   
  
  
- TAGGTTTTCT ATGTTTGGCG TGAGTTAAAA TCGTTTTTTT TTTTAAGTTT TAAATTGTTT TGCGTCCAAA   
  
  
- TTTGCGTGTC GGTTTGTCAG ACAATACCGA CCAAAAATTG AAATAAGATG CTGGTCTAGT ATTTTTTACT   
  
  
- ATAGAACTGA TTAATACACA GCTAATTAAT TTAAATGCTC ATTTATACAA TTTGGTCACA AGTGCTGATA   
  
  
- CGAAGAACTT TGCTGCACCA TAAGTAGTAT AAATAAATGA AGATCTCCAA AAAACATATT TTTTTATTTA   
  
  
- TTTTTTTTAC GTAATACTCT AATCGGCCGA TCTATCGCCC TAACTCGGTC TCGGACTTAA CGTCGACGAA   
  
  
- ATCTATCCCT ACCACTACCT TTTCTTTCCC TTTTAAATAG GTCGTGCTTG TCTTTTCGTT TTGCGGGAAC   
  
  
- GGGCGTTGTT CGGTCCTGCG CACCGCTTCC TCCGAAATTT ACTTACGCTG TCTACAATAA TAACTCCAAA   
  
  
- CTGAACAAAG GGCCAACAAT AACAAAAATA TTTTTTTAAT TAAACAAAAA AAATTTTTTT CAAATGTAAT   
  
  
- AAAATTATAC AGCTGCCTTT ATTATTTAGA ATTTTTTATT TTTTATAATA AAATTATATT AAAATTTATT   
  
  
- TTTATATAAA ATATATTATA TCAAACACAA AACAAATTTA GTATTTAAAT TTTTATAATC TAATTATTTA   
  
  
- AGTGGGATAA AAAAAATACT TAGGGTTAAT CCTAAAATAA AAATTTATAA TCAATTCTTT TTTAATATTA   
  
  
- AATATTAAAT AATTATTGGC ATTGATATTT TTTGATGGCA TTATGTTTTA GTTTACGTTT TAAGAGTTAG   
  
  
- ATTTAAGTAC ATGACAAGAC TGTGTATTAA TTGAGGGGTA AGTCAATGAC AAAAACAAAA ATAGTGAACT   
  
  
- CAATTAAGGA GAAAAAAAAA GATCTTCTTT AAAATTAAAG TAACAAATAT ATTTACCATT TTTTTGTTTT   
  
  
- TACTTTTAGC ACTTGGTAGT TTGTACACGT ACGATTTACG ACGTAAACGA TGGATATTAA CATAAAAGAA   
  
  
- TATTATAACA AAACTTTATA ACTTTTTTGT GATAAAGAAA ATTAGACACA AAAATCTCGA GATAACTAAC   
  
  
- AAAAAAAAAA AAAATGTGAC ACTACTATTT TTGGTTATTG GTGCGGAGTA AGATAATAAT AATAATAGTA   
  
  
- ATAGTGTTCT TTAATATTTT TTAAATAACC TAATTCCTTA CAAATCTTAA CGCAAAATTT AACATAAAAA   
  
  
- TTTTAAAAAC TAAAAAATAA ATTTTAATTA AAAAATCTAA TAAAAATATA TAACTATAGA TTTGATTAAA   
  
  
- ATTTTTTAAT TTTTTATATA ATAAAATTAT ATAAAAATTT ATTTTTTATT AAATTTTTTG TTGAAGATAA   
  
  
- TATGTCCTAT TTTATGGGTA ATGGCTAAAT TGTAAAACTC TTCTTTTTAG ATGTAGGTTC CAAGAATAAG   
  
  
- GAACTCCTGT AATACCAGTA ATGTATAGGT CCTGTTTGGA AACGAACCTG ATTTTAAGTT TGGTTTACCT   
  
  
- TAAAGTATCG ACAGTTTATG TCACTTCGAC CCCTAGGTAC CTTTCAGGGG ACGAAATGAG ACGTCGATAT   
  
  
- TTATGTGGTT GATCCTGTGT TGGTATTTGG TGTCATTGG

+     MYB-like sequence

| Site Name | Organism | Position | Strand | Matrix score. | sequence | function |
| --- | --- | --- | --- | --- | --- | --- |
| MYB-like sequence | Arabidopsis thaliana | 1507 | + | 6 | TAACCA |  |

>Potri.003G139300.1   
+ CAATCAAATA TCACGTCTTG CCTACAAGCA TGGTTTTCAA CTGTCAATTT CTGCTTGAAA GTGCTTCCAT   
  
  
+ ATTAGCACGA GGACTCAGAA GGATAACGAG AATATCAAGC AACTTATGCC AGTCCTTTGT AATTAGCAGA   
  
  
+ GTCGAGCAGT TTTAAATAAC TTTTCGATCC TTCATGTTTA AACTGAGAAT GATGTATAGA ACTAGAAGAC   
  
  
+ TGTCGGCAAT TTCTTTCATT TTGTGTGTGT TTGGCAGTGT TATAGCGATT GCTTTTCAAA TAGTTTTTCG   
  
  
+ TGCCGAAATA CATGCCAATG ATGTTTTTTT ATTTTTTTAA AATCATTTTT AATATCAACA CATCAAAACG   
  
  
+ ATCCAAAAGA TACAAACCGC ACTCAATTTT AGCAAAAAAA AAAATTCAAA ATTTAACAAA ACGCAGGTTT   
  
  
+ AAACGCACAG CCAAACAGTC TGTTATGGCT GGTTTTTAAC TTTATTCTAC GACCAGATCA TAAAAAATGA   
  
  
+ TATCTTGACT AATTATGTGT CGATTAATTA AATTTACGAG TAAATATGTT AAACCAGTGT TCACGACTAT   
  
  
+ GCTTCTTGAA ACGACGTGGT ATTCATCATA TTTATTTACT TCTAGAGGTT TTTTGTATAA AAAAATAAAT   
  
  
+ AAAAAAAATG CATTATGAGA TTAGCCGGCT AGATAGCGGG ATTGAGCCAG AGCCTGAATT GCAGCTGCTT   
  
  
+ TAGATAGGGA TGGTGATGGA AAAGAAAGGG AAAATTTATC CAGCACGAAC AGAAAAGCAA AACGCCCTTG   
  
  
+ CCCGCAACAA GCCAGGACGC GTGGCGAAGG AGGCTTTAAA TGAATGCGAC AGATGTTATT ATTGAGGTTT   
  
  
+ GACTTGTTTC CCGGTTGTTA TTGTTTTTAT AAAAAAATTA ATTTGTTTTT TTTAAAAAAA GTTTACATTA   
  
  
+ TTTTAATATG TCGACGGAAA TAATAAATCT TAAAAAATAA AAAATATTAT TTTAATATAA TTTTAAATAA   
  
  
+ AAATATATTT TATATAATAT AGTTTGTGTT TTGTTTAAAT CATAAATTTA AAAATATTAG ATTAATAAAT   
  
  
+ TCACCCTATT TTTTTTATGA ATCCCAATTA GGATTTTATT TTTAAATATT AGTTAAGAAA AAATTATAAT   
  
  
+ TTATAATTTA TTAATAACCG TAACTATAAA AAACTACCGT AATACAAAAT CAAATGCAAA ATTCTCAATC   
  
  
+ TAAATTCATG TACTGTTCTG ACACATAATT AACTCCCCAT TCAGTTACTG TTTTTGTTTT TATCACTTGA   
  
  
+ GTTAATTCCT CTTTTTTTTT CTAGAAGAAA TTTTAATTTC ATTGTTTATA TAAATGGTAA AAAAACAAAA   
  
  
+ ATGAAAATCG TGAACCATCA AACATGTGCA TGCTAAATGC TGCATTTGCT ACCTATAATT GTATTTTCTT   
  
  
+ ATAATATTGT TTTGAAATAT TGAAAAAACA CTATTTCTTT TAATCTGTGT TTTTAGAGCT CTATTGATTG   
  
  
+ TTTTTTTTTT TTTTACACTG TGATGATAAA AACCAATAAC CACGCCTCAT TCTATTATTA TTATTATCAT   
  
  
+ TATCACAAGA AATTATAAAA AATTTATTGG ATTAAGGAAT GTTTAGAATT GCGTTTTAAA TTGTATTTTT   
  
  
+ AAAATTTTTG ATTTTTTATT TAAAATTAAT TTTTTAGATT ATTTTTATAT ATTGATATCT AAACTAATTT   
  
  
+ TAAAAAATTA AAAAATATAT TATTTTAATA TATTTTTAAA TAAAAAATAA TTTAAAAAAC AACTTCTATT   
  
  
+ ATACAGGATA AAATACCCAT TACCGATTTA ACATTTTGAG AAGAAAAATC TACATCCAAG GTTCTTATTC   
  
  
+ CTTGAGGACA TTATGGTCAT TACATATCCA GGACAAACCT TTGCTTGGAC TAAAATTCAA ACCAAATGGA   
  
  
+ ATTTCATAGC TGTCAAATAC AGTGAAGCTG GGGATCCATG GAAAGTCCCC TGCTTTACTC TGCAGCTATA   
  
  
+ AATACACCAA CTAGGACACA ACCATAAACC ACAGTAACC  

- GTTAGTTTAT AGTGCAGAAC GGATGTTCGT ACCAAAAGTT GACAGTTAAA GACGAACTTT CACGAAGGTA   
  
  
- TAATCGTGCT CCTGAGTCTT CCTATTGCTC TTATAGTTCG TTGAATACGG TCAGGAAACA TTAATCGTCT   
  
  
- CAGCTCGTCA AAATTTATTG AAAAGCTAGG AAGTACAAAT TTGACTCTTA CTACATATCT TGATCTTCTG   
  
  
- ACAGCCGTTA AAGAAAGTAA AACACACACA AACCGTCACA ATATCGCTAA CGAAAAGTTT ATCAAAAAGC   
  
  
- ACGGCTTTAT GTACGGTTAC TACAAAAAAA TAAAAAAATT TTAGTAAAAA TTATAGTTGT GTAGTTTTGC   
  
  
- TAGGTTTTCT ATGTTTGGCG TGAGTTAAAA TCGTTTTTTT TTTTAAGTTT TAAATTGTTT TGCGTCCAAA   
  
  
- TTTGCGTGTC GGTTTGTCAG ACAATACCGA CCAAAAATTG AAATAAGATG CTGGTCTAGT ATTTTTTACT   
  
  
- ATAGAACTGA TTAATACACA GCTAATTAAT TTAAATGCTC ATTTATACAA TTTGGTCACA AGTGCTGATA   
  
  
- CGAAGAACTT TGCTGCACCA TAAGTAGTAT AAATAAATGA AGATCTCCAA AAAACATATT TTTTTATTTA   
  
  
- TTTTTTTTAC GTAATACTCT AATCGGCCGA TCTATCGCCC TAACTCGGTC TCGGACTTAA CGTCGACGAA   
  
  
- ATCTATCCCT ACCACTACCT TTTCTTTCCC TTTTAAATAG GTCGTGCTTG TCTTTTCGTT TTGCGGGAAC   
  
  
- GGGCGTTGTT CGGTCCTGCG CACCGCTTCC TCCGAAATTT ACTTACGCTG TCTACAATAA TAACTCCAAA   
  
  
- CTGAACAAAG GGCCAACAAT AACAAAAATA TTTTTTTAAT TAAACAAAAA AAATTTTTTT CAAATGTAAT   
  
  
- AAAATTATAC AGCTGCCTTT ATTATTTAGA ATTTTTTATT TTTTATAATA AAATTATATT AAAATTTATT   
  
  
- TTTATATAAA ATATATTATA TCAAACACAA AACAAATTTA GTATTTAAAT TTTTATAATC TAATTATTTA   
  
  
- AGTGGGATAA AAAAAATACT TAGGGTTAAT CCTAAAATAA AAATTTATAA TCAATTCTTT TTTAATATTA   
  
  
- AATATTAAAT AATTATTGGC ATTGATATTT TTTGATGGCA TTATGTTTTA GTTTACGTTT TAAGAGTTAG   
  
  
- ATTTAAGTAC ATGACAAGAC TGTGTATTAA TTGAGGGGTA AGTCAATGAC AAAAACAAAA ATAGTGAACT   
  
  
- CAATTAAGGA GAAAAAAAAA GATCTTCTTT AAAATTAAAG TAACAAATAT ATTTACCATT TTTTTGTTTT   
  
  
- TACTTTTAGC ACTTGGTAGT TTGTACACGT ACGATTTACG ACGTAAACGA TGGATATTAA CATAAAAGAA   
  
  
- TATTATAACA AAACTTTATA ACTTTTTTGT GATAAAGAAA ATTAGACACA AAAATCTCGA GATAACTAAC   
  
  
- AAAAAAAAAA AAAATGTGAC ACTACTATTT TTGGTTATTG GTGCGGAGTA AGATAATAAT AATAATAGTA   
  
  
- ATAGTGTTCT TTAATATTTT TTAAATAACC TAATTCCTTA CAAATCTTAA CGCAAAATTT AACATAAAAA   
  
  
- TTTTAAAAAC TAAAAAATAA ATTTTAATTA AAAAATCTAA TAAAAATATA TAACTATAGA TTTGATTAAA   
  
  
- ATTTTTTAAT TTTTTATATA ATAAAATTAT ATAAAAATTT ATTTTTTATT AAATTTTTTG TTGAAGATAA   
  
  
- TATGTCCTAT TTTATGGGTA ATGGCTAAAT TGTAAAACTC TTCTTTTTAG ATGTAGGTTC CAAGAATAAG   
  
  
- GAACTCCTGT AATACCAGTA ATGTATAGGT CCTGTTTGGA AACGAACCTG ATTTTAAGTT TGGTTTACCT   
  
  
- TAAAGTATCG ACAGTTTATG TCACTTCGAC CCCTAGGTAC CTTTCAGGGG ACGAAATGAG ACGTCGATAT   
  
  
- TTATGTGGTT GATCCTGTGT TGGTATTTGG TGTCATTGG

+     MYC

| Site Name | Organism | Position | Strand | Matrix score. | sequence | function |
| --- | --- | --- | --- | --- | --- | --- |
| MYC | Arabidopsis thaliana | 1353 | + | 6 | CATGTG |  |
| MYC | Arabidopsis thaliana | 1171 | - | 6 | CATTTG |  |
| MYC | Arabidopsis thaliana | 1883 | - | 6 | CATTTG |  |
| MYC | Arabidopsis thaliana | 1373 | + | 6 | CATTTG |  |

>Potri.003G139300.1   
+ CAATCAAATA TCACGTCTTG CCTACAAGCA TGGTTTTCAA CTGTCAATTT CTGCTTGAAA GTGCTTCCAT   
  
  
+ ATTAGCACGA GGACTCAGAA GGATAACGAG AATATCAAGC AACTTATGCC AGTCCTTTGT AATTAGCAGA   
  
  
+ GTCGAGCAGT TTTAAATAAC TTTTCGATCC TTCATGTTTA AACTGAGAAT GATGTATAGA ACTAGAAGAC   
  
  
+ TGTCGGCAAT TTCTTTCATT TTGTGTGTGT TTGGCAGTGT TATAGCGATT GCTTTTCAAA TAGTTTTTCG   
  
  
+ TGCCGAAATA CATGCCAATG ATGTTTTTTT ATTTTTTTAA AATCATTTTT AATATCAACA CATCAAAACG   
  
  
+ ATCCAAAAGA TACAAACCGC ACTCAATTTT AGCAAAAAAA AAAATTCAAA ATTTAACAAA ACGCAGGTTT   
  
  
+ AAACGCACAG CCAAACAGTC TGTTATGGCT GGTTTTTAAC TTTATTCTAC GACCAGATCA TAAAAAATGA   
  
  
+ TATCTTGACT AATTATGTGT CGATTAATTA AATTTACGAG TAAATATGTT AAACCAGTGT TCACGACTAT   
  
  
+ GCTTCTTGAA ACGACGTGGT ATTCATCATA TTTATTTACT TCTAGAGGTT TTTTGTATAA AAAAATAAAT   
  
  
+ AAAAAAAATG CATTATGAGA TTAGCCGGCT AGATAGCGGG ATTGAGCCAG AGCCTGAATT GCAGCTGCTT   
  
  
+ TAGATAGGGA TGGTGATGGA AAAGAAAGGG AAAATTTATC CAGCACGAAC AGAAAAGCAA AACGCCCTTG   
  
  
+ CCCGCAACAA GCCAGGACGC GTGGCGAAGG AGGCTTTAAA TGAATGCGAC AGATGTTATT ATTGAGGTTT   
  
  
+ GACTTGTTTC CCGGTTGTTA TTGTTTTTAT AAAAAAATTA ATTTGTTTTT TTTAAAAAAA GTTTACATTA   
  
  
+ TTTTAATATG TCGACGGAAA TAATAAATCT TAAAAAATAA AAAATATTAT TTTAATATAA TTTTAAATAA   
  
  
+ AAATATATTT TATATAATAT AGTTTGTGTT TTGTTTAAAT CATAAATTTA AAAATATTAG ATTAATAAAT   
  
  
+ TCACCCTATT TTTTTTATGA ATCCCAATTA GGATTTTATT TTTAAATATT AGTTAAGAAA AAATTATAAT   
  
  
+ TTATAATTTA TTAATAACCG TAACTATAAA AAACTACCGT AATACAAAAT CAAATGCAAA ATTCTCAATC   
  
  
+ TAAATTCATG TACTGTTCTG ACACATAATT AACTCCCCAT TCAGTTACTG TTTTTGTTTT TATCACTTGA   
  
  
+ GTTAATTCCT CTTTTTTTTT CTAGAAGAAA TTTTAATTTC ATTGTTTATA TAAATGGTAA AAAAACAAAA   
  
  
+ ATGAAAATCG TGAACCATCA AACATGTGCA TGCTAAATGC TGCATTTGCT ACCTATAATT GTATTTTCTT   
  
  
+ ATAATATTGT TTTGAAATAT TGAAAAAACA CTATTTCTTT TAATCTGTGT TTTTAGAGCT CTATTGATTG   
  
  
+ TTTTTTTTTT TTTTACACTG TGATGATAAA AACCAATAAC CACGCCTCAT TCTATTATTA TTATTATCAT   
  
  
+ TATCACAAGA AATTATAAAA AATTTATTGG ATTAAGGAAT GTTTAGAATT GCGTTTTAAA TTGTATTTTT   
  
  
+ AAAATTTTTG ATTTTTTATT TAAAATTAAT TTTTTAGATT ATTTTTATAT ATTGATATCT AAACTAATTT   
  
  
+ TAAAAAATTA AAAAATATAT TATTTTAATA TATTTTTAAA TAAAAAATAA TTTAAAAAAC AACTTCTATT   
  
  
+ ATACAGGATA AAATACCCAT TACCGATTTA ACATTTTGAG AAGAAAAATC TACATCCAAG GTTCTTATTC   
  
  
+ CTTGAGGACA TTATGGTCAT TACATATCCA GGACAAACCT TTGCTTGGAC TAAAATTCAA ACCAAATGGA   
  
  
+ ATTTCATAGC TGTCAAATAC AGTGAAGCTG GGGATCCATG GAAAGTCCCC TGCTTTACTC TGCAGCTATA   
  
  
+ AATACACCAA CTAGGACACA ACCATAAACC ACAGTAACC  

- GTTAGTTTAT AGTGCAGAAC GGATGTTCGT ACCAAAAGTT GACAGTTAAA GACGAACTTT CACGAAGGTA   
  
  
- TAATCGTGCT CCTGAGTCTT CCTATTGCTC TTATAGTTCG TTGAATACGG TCAGGAAACA TTAATCGTCT   
  
  
- CAGCTCGTCA AAATTTATTG AAAAGCTAGG AAGTACAAAT TTGACTCTTA CTACATATCT TGATCTTCTG   
  
  
- ACAGCCGTTA AAGAAAGTAA AACACACACA AACCGTCACA ATATCGCTAA CGAAAAGTTT ATCAAAAAGC   
  
  
- ACGGCTTTAT GTACGGTTAC TACAAAAAAA TAAAAAAATT TTAGTAAAAA TTATAGTTGT GTAGTTTTGC   
  
  
- TAGGTTTTCT ATGTTTGGCG TGAGTTAAAA TCGTTTTTTT TTTTAAGTTT TAAATTGTTT TGCGTCCAAA   
  
  
- TTTGCGTGTC GGTTTGTCAG ACAATACCGA CCAAAAATTG AAATAAGATG CTGGTCTAGT ATTTTTTACT   
  
  
- ATAGAACTGA TTAATACACA GCTAATTAAT TTAAATGCTC ATTTATACAA TTTGGTCACA AGTGCTGATA   
  
  
- CGAAGAACTT TGCTGCACCA TAAGTAGTAT AAATAAATGA AGATCTCCAA AAAACATATT TTTTTATTTA   
  
  
- TTTTTTTTAC GTAATACTCT AATCGGCCGA TCTATCGCCC TAACTCGGTC TCGGACTTAA CGTCGACGAA   
  
  
- ATCTATCCCT ACCACTACCT TTTCTTTCCC TTTTAAATAG GTCGTGCTTG TCTTTTCGTT TTGCGGGAAC   
  
  
- GGGCGTTGTT CGGTCCTGCG CACCGCTTCC TCCGAAATTT ACTTACGCTG TCTACAATAA TAACTCCAAA   
  
  
- CTGAACAAAG GGCCAACAAT AACAAAAATA TTTTTTTAAT TAAACAAAAA AAATTTTTTT CAAATGTAAT   
  
  
- AAAATTATAC AGCTGCCTTT ATTATTTAGA ATTTTTTATT TTTTATAATA AAATTATATT AAAATTTATT   
  
  
- TTTATATAAA ATATATTATA TCAAACACAA AACAAATTTA GTATTTAAAT TTTTATAATC TAATTATTTA   
  
  
- AGTGGGATAA AAAAAATACT TAGGGTTAAT CCTAAAATAA AAATTTATAA TCAATTCTTT TTTAATATTA   
  
  
- AATATTAAAT AATTATTGGC ATTGATATTT TTTGATGGCA TTATGTTTTA GTTTACGTTT TAAGAGTTAG   
  
  
- ATTTAAGTAC ATGACAAGAC TGTGTATTAA TTGAGGGGTA AGTCAATGAC AAAAACAAAA ATAGTGAACT   
  
  
- CAATTAAGGA GAAAAAAAAA GATCTTCTTT AAAATTAAAG TAACAAATAT ATTTACCATT TTTTTGTTTT   
  
  
- TACTTTTAGC ACTTGGTAGT TTGTACACGT ACGATTTACG ACGTAAACGA TGGATATTAA CATAAAAGAA   
  
  
- TATTATAACA AAACTTTATA ACTTTTTTGT GATAAAGAAA ATTAGACACA AAAATCTCGA GATAACTAAC   
  
  
- AAAAAAAAAA AAAATGTGAC ACTACTATTT TTGGTTATTG GTGCGGAGTA AGATAATAAT AATAATAGTA   
  
  
- ATAGTGTTCT TTAATATTTT TTAAATAACC TAATTCCTTA CAAATCTTAA CGCAAAATTT AACATAAAAA   
  
  
- TTTTAAAAAC TAAAAAATAA ATTTTAATTA AAAAATCTAA TAAAAATATA TAACTATAGA TTTGATTAAA   
  
  
- ATTTTTTAAT TTTTTATATA ATAAAATTAT ATAAAAATTT ATTTTTTATT AAATTTTTTG TTGAAGATAA   
  
  
- TATGTCCTAT TTTATGGGTA ATGGCTAAAT TGTAAAACTC TTCTTTTTAG ATGTAGGTTC CAAGAATAAG   
  
  
- GAACTCCTGT AATACCAGTA ATGTATAGGT CCTGTTTGGA AACGAACCTG ATTTTAAGTT TGGTTTACCT   
  
  
- TAAAGTATCG ACAGTTTATG TCACTTCGAC CCCTAGGTAC CTTTCAGGGG ACGAAATGAG ACGTCGATAT   
  
  
- TTATGTGGTT GATCCTGTGT TGGTATTTGG TGTCATTGG

+     Myb

| Site Name | Organism | Position | Strand | Matrix score. | sequence | function |
| --- | --- | --- | --- | --- | --- | --- |
| Myb | Arabidopsis thaliana | 1232 | - | 6 | TAACTG |  |
| Myb | Arabidopsis thaliana | 38 | + | 6 | CAACTG |  |

>Potri.003G139300.1   
+ CAATCAAATA TCACGTCTTG CCTACAAGCA TGGTTTTCAA CTGTCAATTT CTGCTTGAAA GTGCTTCCAT   
  
  
+ ATTAGCACGA GGACTCAGAA GGATAACGAG AATATCAAGC AACTTATGCC AGTCCTTTGT AATTAGCAGA   
  
  
+ GTCGAGCAGT TTTAAATAAC TTTTCGATCC TTCATGTTTA AACTGAGAAT GATGTATAGA ACTAGAAGAC   
  
  
+ TGTCGGCAAT TTCTTTCATT TTGTGTGTGT TTGGCAGTGT TATAGCGATT GCTTTTCAAA TAGTTTTTCG   
  
  
+ TGCCGAAATA CATGCCAATG ATGTTTTTTT ATTTTTTTAA AATCATTTTT AATATCAACA CATCAAAACG   
  
  
+ ATCCAAAAGA TACAAACCGC ACTCAATTTT AGCAAAAAAA AAAATTCAAA ATTTAACAAA ACGCAGGTTT   
  
  
+ AAACGCACAG CCAAACAGTC TGTTATGGCT GGTTTTTAAC TTTATTCTAC GACCAGATCA TAAAAAATGA   
  
  
+ TATCTTGACT AATTATGTGT CGATTAATTA AATTTACGAG TAAATATGTT AAACCAGTGT TCACGACTAT   
  
  
+ GCTTCTTGAA ACGACGTGGT ATTCATCATA TTTATTTACT TCTAGAGGTT TTTTGTATAA AAAAATAAAT   
  
  
+ AAAAAAAATG CATTATGAGA TTAGCCGGCT AGATAGCGGG ATTGAGCCAG AGCCTGAATT GCAGCTGCTT   
  
  
+ TAGATAGGGA TGGTGATGGA AAAGAAAGGG AAAATTTATC CAGCACGAAC AGAAAAGCAA AACGCCCTTG   
  
  
+ CCCGCAACAA GCCAGGACGC GTGGCGAAGG AGGCTTTAAA TGAATGCGAC AGATGTTATT ATTGAGGTTT   
  
  
+ GACTTGTTTC CCGGTTGTTA TTGTTTTTAT AAAAAAATTA ATTTGTTTTT TTTAAAAAAA GTTTACATTA   
  
  
+ TTTTAATATG TCGACGGAAA TAATAAATCT TAAAAAATAA AAAATATTAT TTTAATATAA TTTTAAATAA   
  
  
+ AAATATATTT TATATAATAT AGTTTGTGTT TTGTTTAAAT CATAAATTTA AAAATATTAG ATTAATAAAT   
  
  
+ TCACCCTATT TTTTTTATGA ATCCCAATTA GGATTTTATT TTTAAATATT AGTTAAGAAA AAATTATAAT   
  
  
+ TTATAATTTA TTAATAACCG TAACTATAAA AAACTACCGT AATACAAAAT CAAATGCAAA ATTCTCAATC   
  
  
+ TAAATTCATG TACTGTTCTG ACACATAATT AACTCCCCAT TCAGTTACTG TTTTTGTTTT TATCACTTGA   
  
  
+ GTTAATTCCT CTTTTTTTTT CTAGAAGAAA TTTTAATTTC ATTGTTTATA TAAATGGTAA AAAAACAAAA   
  
  
+ ATGAAAATCG TGAACCATCA AACATGTGCA TGCTAAATGC TGCATTTGCT ACCTATAATT GTATTTTCTT   
  
  
+ ATAATATTGT TTTGAAATAT TGAAAAAACA CTATTTCTTT TAATCTGTGT TTTTAGAGCT CTATTGATTG   
  
  
+ TTTTTTTTTT TTTTACACTG TGATGATAAA AACCAATAAC CACGCCTCAT TCTATTATTA TTATTATCAT   
  
  
+ TATCACAAGA AATTATAAAA AATTTATTGG ATTAAGGAAT GTTTAGAATT GCGTTTTAAA TTGTATTTTT   
  
  
+ AAAATTTTTG ATTTTTTATT TAAAATTAAT TTTTTAGATT ATTTTTATAT ATTGATATCT AAACTAATTT   
  
  
+ TAAAAAATTA AAAAATATAT TATTTTAATA TATTTTTAAA TAAAAAATAA TTTAAAAAAC AACTTCTATT   
  
  
+ ATACAGGATA AAATACCCAT TACCGATTTA ACATTTTGAG AAGAAAAATC TACATCCAAG GTTCTTATTC   
  
  
+ CTTGAGGACA TTATGGTCAT TACATATCCA GGACAAACCT TTGCTTGGAC TAAAATTCAA ACCAAATGGA   
  
  
+ ATTTCATAGC TGTCAAATAC AGTGAAGCTG GGGATCCATG GAAAGTCCCC TGCTTTACTC TGCAGCTATA   
  
  
+ AATACACCAA CTAGGACACA ACCATAAACC ACAGTAACC  

- GTTAGTTTAT AGTGCAGAAC GGATGTTCGT ACCAAAAGTT GACAGTTAAA GACGAACTTT CACGAAGGTA   
  
  
- TAATCGTGCT CCTGAGTCTT CCTATTGCTC TTATAGTTCG TTGAATACGG TCAGGAAACA TTAATCGTCT   
  
  
- CAGCTCGTCA AAATTTATTG AAAAGCTAGG AAGTACAAAT TTGACTCTTA CTACATATCT TGATCTTCTG   
  
  
- ACAGCCGTTA AAGAAAGTAA AACACACACA AACCGTCACA ATATCGCTAA CGAAAAGTTT ATCAAAAAGC   
  
  
- ACGGCTTTAT GTACGGTTAC TACAAAAAAA TAAAAAAATT TTAGTAAAAA TTATAGTTGT GTAGTTTTGC   
  
  
- TAGGTTTTCT ATGTTTGGCG TGAGTTAAAA TCGTTTTTTT TTTTAAGTTT TAAATTGTTT TGCGTCCAAA   
  
  
- TTTGCGTGTC GGTTTGTCAG ACAATACCGA CCAAAAATTG AAATAAGATG CTGGTCTAGT ATTTTTTACT   
  
  
- ATAGAACTGA TTAATACACA GCTAATTAAT TTAAATGCTC ATTTATACAA TTTGGTCACA AGTGCTGATA   
  
  
- CGAAGAACTT TGCTGCACCA TAAGTAGTAT AAATAAATGA AGATCTCCAA AAAACATATT TTTTTATTTA   
  
  
- TTTTTTTTAC GTAATACTCT AATCGGCCGA TCTATCGCCC TAACTCGGTC TCGGACTTAA CGTCGACGAA   
  
  
- ATCTATCCCT ACCACTACCT TTTCTTTCCC TTTTAAATAG GTCGTGCTTG TCTTTTCGTT TTGCGGGAAC   
  
  
- GGGCGTTGTT CGGTCCTGCG CACCGCTTCC TCCGAAATTT ACTTACGCTG TCTACAATAA TAACTCCAAA   
  
  
- CTGAACAAAG GGCCAACAAT AACAAAAATA TTTTTTTAAT TAAACAAAAA AAATTTTTTT CAAATGTAAT   
  
  
- AAAATTATAC AGCTGCCTTT ATTATTTAGA ATTTTTTATT TTTTATAATA AAATTATATT AAAATTTATT   
  
  
- TTTATATAAA ATATATTATA TCAAACACAA AACAAATTTA GTATTTAAAT TTTTATAATC TAATTATTTA   
  
  
- AGTGGGATAA AAAAAATACT TAGGGTTAAT CCTAAAATAA AAATTTATAA TCAATTCTTT TTTAATATTA   
  
  
- AATATTAAAT AATTATTGGC ATTGATATTT TTTGATGGCA TTATGTTTTA GTTTACGTTT TAAGAGTTAG   
  
  
- ATTTAAGTAC ATGACAAGAC TGTGTATTAA TTGAGGGGTA AGTCAATGAC AAAAACAAAA ATAGTGAACT   
  
  
- CAATTAAGGA GAAAAAAAAA GATCTTCTTT AAAATTAAAG TAACAAATAT ATTTACCATT TTTTTGTTTT   
  
  
- TACTTTTAGC ACTTGGTAGT TTGTACACGT ACGATTTACG ACGTAAACGA TGGATATTAA CATAAAAGAA   
  
  
- TATTATAACA AAACTTTATA ACTTTTTTGT GATAAAGAAA ATTAGACACA AAAATCTCGA GATAACTAAC   
  
  
- AAAAAAAAAA AAAATGTGAC ACTACTATTT TTGGTTATTG GTGCGGAGTA AGATAATAAT AATAATAGTA   
  
  
- ATAGTGTTCT TTAATATTTT TTAAATAACC TAATTCCTTA CAAATCTTAA CGCAAAATTT AACATAAAAA   
  
  
- TTTTAAAAAC TAAAAAATAA ATTTTAATTA AAAAATCTAA TAAAAATATA TAACTATAGA TTTGATTAAA   
  
  
- ATTTTTTAAT TTTTTATATA ATAAAATTAT ATAAAAATTT ATTTTTTATT AAATTTTTTG TTGAAGATAA   
  
  
- TATGTCCTAT TTTATGGGTA ATGGCTAAAT TGTAAAACTC TTCTTTTTAG ATGTAGGTTC CAAGAATAAG   
  
  
- GAACTCCTGT AATACCAGTA ATGTATAGGT CCTGTTTGGA AACGAACCTG ATTTTAAGTT TGGTTTACCT   
  
  
- TAAAGTATCG ACAGTTTATG TCACTTCGAC CCCTAGGTAC CTTTCAGGGG ACGAAATGAG ACGTCGATAT   
  
  
- TTATGTGGTT GATCCTGTGT TGGTATTTGG TGTCATTGG

+     STRE

| Site Name | Organism | Position | Strand | Matrix score. | sequence | function |
| --- | --- | --- | --- | --- | --- | --- |
| STRE | Arabidopsis thaliana | 1937 | - | 5 | AGGGG |  |

>Potri.003G139300.1   
+ CAATCAAATA TCACGTCTTG CCTACAAGCA TGGTTTTCAA CTGTCAATTT CTGCTTGAAA GTGCTTCCAT   
  
  
+ ATTAGCACGA GGACTCAGAA GGATAACGAG AATATCAAGC AACTTATGCC AGTCCTTTGT AATTAGCAGA   
  
  
+ GTCGAGCAGT TTTAAATAAC TTTTCGATCC TTCATGTTTA AACTGAGAAT GATGTATAGA ACTAGAAGAC   
  
  
+ TGTCGGCAAT TTCTTTCATT TTGTGTGTGT TTGGCAGTGT TATAGCGATT GCTTTTCAAA TAGTTTTTCG   
  
  
+ TGCCGAAATA CATGCCAATG ATGTTTTTTT ATTTTTTTAA AATCATTTTT AATATCAACA CATCAAAACG   
  
  
+ ATCCAAAAGA TACAAACCGC ACTCAATTTT AGCAAAAAAA AAAATTCAAA ATTTAACAAA ACGCAGGTTT   
  
  
+ AAACGCACAG CCAAACAGTC TGTTATGGCT GGTTTTTAAC TTTATTCTAC GACCAGATCA TAAAAAATGA   
  
  
+ TATCTTGACT AATTATGTGT CGATTAATTA AATTTACGAG TAAATATGTT AAACCAGTGT TCACGACTAT   
  
  
+ GCTTCTTGAA ACGACGTGGT ATTCATCATA TTTATTTACT TCTAGAGGTT TTTTGTATAA AAAAATAAAT   
  
  
+ AAAAAAAATG CATTATGAGA TTAGCCGGCT AGATAGCGGG ATTGAGCCAG AGCCTGAATT GCAGCTGCTT   
  
  
+ TAGATAGGGA TGGTGATGGA AAAGAAAGGG AAAATTTATC CAGCACGAAC AGAAAAGCAA AACGCCCTTG   
  
  
+ CCCGCAACAA GCCAGGACGC GTGGCGAAGG AGGCTTTAAA TGAATGCGAC AGATGTTATT ATTGAGGTTT   
  
  
+ GACTTGTTTC CCGGTTGTTA TTGTTTTTAT AAAAAAATTA ATTTGTTTTT TTTAAAAAAA GTTTACATTA   
  
  
+ TTTTAATATG TCGACGGAAA TAATAAATCT TAAAAAATAA AAAATATTAT TTTAATATAA TTTTAAATAA   
  
  
+ AAATATATTT TATATAATAT AGTTTGTGTT TTGTTTAAAT CATAAATTTA AAAATATTAG ATTAATAAAT   
  
  
+ TCACCCTATT TTTTTTATGA ATCCCAATTA GGATTTTATT TTTAAATATT AGTTAAGAAA AAATTATAAT   
  
  
+ TTATAATTTA TTAATAACCG TAACTATAAA AAACTACCGT AATACAAAAT CAAATGCAAA ATTCTCAATC   
  
  
+ TAAATTCATG TACTGTTCTG ACACATAATT AACTCCCCAT TCAGTTACTG TTTTTGTTTT TATCACTTGA   
  
  
+ GTTAATTCCT CTTTTTTTTT CTAGAAGAAA TTTTAATTTC ATTGTTTATA TAAATGGTAA AAAAACAAAA   
  
  
+ ATGAAAATCG TGAACCATCA AACATGTGCA TGCTAAATGC TGCATTTGCT ACCTATAATT GTATTTTCTT   
  
  
+ ATAATATTGT TTTGAAATAT TGAAAAAACA CTATTTCTTT TAATCTGTGT TTTTAGAGCT CTATTGATTG   
  
  
+ TTTTTTTTTT TTTTACACTG TGATGATAAA AACCAATAAC CACGCCTCAT TCTATTATTA TTATTATCAT   
  
  
+ TATCACAAGA AATTATAAAA AATTTATTGG ATTAAGGAAT GTTTAGAATT GCGTTTTAAA TTGTATTTTT   
  
  
+ AAAATTTTTG ATTTTTTATT TAAAATTAAT TTTTTAGATT ATTTTTATAT ATTGATATCT AAACTAATTT   
  
  
+ TAAAAAATTA AAAAATATAT TATTTTAATA TATTTTTAAA TAAAAAATAA TTTAAAAAAC AACTTCTATT   
  
  
+ ATACAGGATA AAATACCCAT TACCGATTTA ACATTTTGAG AAGAAAAATC TACATCCAAG GTTCTTATTC   
  
  
+ CTTGAGGACA TTATGGTCAT TACATATCCA GGACAAACCT TTGCTTGGAC TAAAATTCAA ACCAAATGGA   
  
  
+ ATTTCATAGC TGTCAAATAC AGTGAAGCTG GGGATCCATG GAAAGTCCCC TGCTTTACTC TGCAGCTATA   
  
  
+ AATACACCAA CTAGGACACA ACCATAAACC ACAGTAACC  

- GTTAGTTTAT AGTGCAGAAC GGATGTTCGT ACCAAAAGTT GACAGTTAAA GACGAACTTT CACGAAGGTA   
  
  
- TAATCGTGCT CCTGAGTCTT CCTATTGCTC TTATAGTTCG TTGAATACGG TCAGGAAACA TTAATCGTCT   
  
  
- CAGCTCGTCA AAATTTATTG AAAAGCTAGG AAGTACAAAT TTGACTCTTA CTACATATCT TGATCTTCTG   
  
  
- ACAGCCGTTA AAGAAAGTAA AACACACACA AACCGTCACA ATATCGCTAA CGAAAAGTTT ATCAAAAAGC   
  
  
- ACGGCTTTAT GTACGGTTAC TACAAAAAAA TAAAAAAATT TTAGTAAAAA TTATAGTTGT GTAGTTTTGC   
  
  
- TAGGTTTTCT ATGTTTGGCG TGAGTTAAAA TCGTTTTTTT TTTTAAGTTT TAAATTGTTT TGCGTCCAAA   
  
  
- TTTGCGTGTC GGTTTGTCAG ACAATACCGA CCAAAAATTG AAATAAGATG CTGGTCTAGT ATTTTTTACT   
  
  
- ATAGAACTGA TTAATACACA GCTAATTAAT TTAAATGCTC ATTTATACAA TTTGGTCACA AGTGCTGATA   
  
  
- CGAAGAACTT TGCTGCACCA TAAGTAGTAT AAATAAATGA AGATCTCCAA AAAACATATT TTTTTATTTA   
  
  
- TTTTTTTTAC GTAATACTCT AATCGGCCGA TCTATCGCCC TAACTCGGTC TCGGACTTAA CGTCGACGAA   
  
  
- ATCTATCCCT ACCACTACCT TTTCTTTCCC TTTTAAATAG GTCGTGCTTG TCTTTTCGTT TTGCGGGAAC   
  
  
- GGGCGTTGTT CGGTCCTGCG CACCGCTTCC TCCGAAATTT ACTTACGCTG TCTACAATAA TAACTCCAAA   
  
  
- CTGAACAAAG GGCCAACAAT AACAAAAATA TTTTTTTAAT TAAACAAAAA AAATTTTTTT CAAATGTAAT   
  
  
- AAAATTATAC AGCTGCCTTT ATTATTTAGA ATTTTTTATT TTTTATAATA AAATTATATT AAAATTTATT   
  
  
- TTTATATAAA ATATATTATA TCAAACACAA AACAAATTTA GTATTTAAAT TTTTATAATC TAATTATTTA   
  
  
- AGTGGGATAA AAAAAATACT TAGGGTTAAT CCTAAAATAA AAATTTATAA TCAATTCTTT TTTAATATTA   
  
  
- AATATTAAAT AATTATTGGC ATTGATATTT TTTGATGGCA TTATGTTTTA GTTTACGTTT TAAGAGTTAG   
  
  
- ATTTAAGTAC ATGACAAGAC TGTGTATTAA TTGAGGGGTA AGTCAATGAC AAAAACAAAA ATAGTGAACT   
  
  
- CAATTAAGGA GAAAAAAAAA GATCTTCTTT AAAATTAAAG TAACAAATAT ATTTACCATT TTTTTGTTTT   
  
  
- TACTTTTAGC ACTTGGTAGT TTGTACACGT ACGATTTACG ACGTAAACGA TGGATATTAA CATAAAAGAA   
  
  
- TATTATAACA AAACTTTATA ACTTTTTTGT GATAAAGAAA ATTAGACACA AAAATCTCGA GATAACTAAC   
  
  
- AAAAAAAAAA AAAATGTGAC ACTACTATTT TTGGTTATTG GTGCGGAGTA AGATAATAAT AATAATAGTA   
  
  
- ATAGTGTTCT TTAATATTTT TTAAATAACC TAATTCCTTA CAAATCTTAA CGCAAAATTT AACATAAAAA   
  
  
- TTTTAAAAAC TAAAAAATAA ATTTTAATTA AAAAATCTAA TAAAAATATA TAACTATAGA TTTGATTAAA   
  
  
- ATTTTTTAAT TTTTTATATA ATAAAATTAT ATAAAAATTT ATTTTTTATT AAATTTTTTG TTGAAGATAA   
  
  
- TATGTCCTAT TTTATGGGTA ATGGCTAAAT TGTAAAACTC TTCTTTTTAG ATGTAGGTTC CAAGAATAAG   
  
  
- GAACTCCTGT AATACCAGTA ATGTATAGGT CCTGTTTGGA AACGAACCTG ATTTTAAGTT TGGTTTACCT   
  
  
- TAAAGTATCG ACAGTTTATG TCACTTCGAC CCCTAGGTAC CTTTCAGGGG ACGAAATGAG ACGTCGATAT   
  
  
- TTATGTGGTT GATCCTGTGT TGGTATTTGG TGTCATTGG

+     TATA

| Site Name | Organism | Position | Strand | Matrix score. | sequence | function |
| --- | --- | --- | --- | --- | --- | --- |
| TATA | Arabidopsis thaliana | 987 | - | 8 | TATAAAAT |  |

>Potri.003G139300.1   
+ CAATCAAATA TCACGTCTTG CCTACAAGCA TGGTTTTCAA CTGTCAATTT CTGCTTGAAA GTGCTTCCAT   
  
  
+ ATTAGCACGA GGACTCAGAA GGATAACGAG AATATCAAGC AACTTATGCC AGTCCTTTGT AATTAGCAGA   
  
  
+ GTCGAGCAGT TTTAAATAAC TTTTCGATCC TTCATGTTTA AACTGAGAAT GATGTATAGA ACTAGAAGAC   
  
  
+ TGTCGGCAAT TTCTTTCATT TTGTGTGTGT TTGGCAGTGT TATAGCGATT GCTTTTCAAA TAGTTTTTCG   
  
  
+ TGCCGAAATA CATGCCAATG ATGTTTTTTT ATTTTTTTAA AATCATTTTT AATATCAACA CATCAAAACG   
  
  
+ ATCCAAAAGA TACAAACCGC ACTCAATTTT AGCAAAAAAA AAAATTCAAA ATTTAACAAA ACGCAGGTTT   
  
  
+ AAACGCACAG CCAAACAGTC TGTTATGGCT GGTTTTTAAC TTTATTCTAC GACCAGATCA TAAAAAATGA   
  
  
+ TATCTTGACT AATTATGTGT CGATTAATTA AATTTACGAG TAAATATGTT AAACCAGTGT TCACGACTAT   
  
  
+ GCTTCTTGAA ACGACGTGGT ATTCATCATA TTTATTTACT TCTAGAGGTT TTTTGTATAA AAAAATAAAT   
  
  
+ AAAAAAAATG CATTATGAGA TTAGCCGGCT AGATAGCGGG ATTGAGCCAG AGCCTGAATT GCAGCTGCTT   
  
  
+ TAGATAGGGA TGGTGATGGA AAAGAAAGGG AAAATTTATC CAGCACGAAC AGAAAAGCAA AACGCCCTTG   
  
  
+ CCCGCAACAA GCCAGGACGC GTGGCGAAGG AGGCTTTAAA TGAATGCGAC AGATGTTATT ATTGAGGTTT   
  
  
+ GACTTGTTTC CCGGTTGTTA TTGTTTTTAT AAAAAAATTA ATTTGTTTTT TTTAAAAAAA GTTTACATTA   
  
  
+ TTTTAATATG TCGACGGAAA TAATAAATCT TAAAAAATAA AAAATATTAT TTTAATATAA TTTTAAATAA   
  
  
+ AAATATATTT TATATAATAT AGTTTGTGTT TTGTTTAAAT CATAAATTTA AAAATATTAG ATTAATAAAT   
  
  
+ TCACCCTATT TTTTTTATGA ATCCCAATTA GGATTTTATT TTTAAATATT AGTTAAGAAA AAATTATAAT   
  
  
+ TTATAATTTA TTAATAACCG TAACTATAAA AAACTACCGT AATACAAAAT CAAATGCAAA ATTCTCAATC   
  
  
+ TAAATTCATG TACTGTTCTG ACACATAATT AACTCCCCAT TCAGTTACTG TTTTTGTTTT TATCACTTGA   
  
  
+ GTTAATTCCT CTTTTTTTTT CTAGAAGAAA TTTTAATTTC ATTGTTTATA TAAATGGTAA AAAAACAAAA   
  
  
+ ATGAAAATCG TGAACCATCA AACATGTGCA TGCTAAATGC TGCATTTGCT ACCTATAATT GTATTTTCTT   
  
  
+ ATAATATTGT TTTGAAATAT TGAAAAAACA CTATTTCTTT TAATCTGTGT TTTTAGAGCT CTATTGATTG   
  
  
+ TTTTTTTTTT TTTTACACTG TGATGATAAA AACCAATAAC CACGCCTCAT TCTATTATTA TTATTATCAT   
  
  
+ TATCACAAGA AATTATAAAA AATTTATTGG ATTAAGGAAT GTTTAGAATT GCGTTTTAAA TTGTATTTTT   
  
  
+ AAAATTTTTG ATTTTTTATT TAAAATTAAT TTTTTAGATT ATTTTTATAT ATTGATATCT AAACTAATTT   
  
  
+ TAAAAAATTA AAAAATATAT TATTTTAATA TATTTTTAAA TAAAAAATAA TTTAAAAAAC AACTTCTATT   
  
  
+ ATACAGGATA AAATACCCAT TACCGATTTA ACATTTTGAG AAGAAAAATC TACATCCAAG GTTCTTATTC   
  
  
+ CTTGAGGACA TTATGGTCAT TACATATCCA GGACAAACCT TTGCTTGGAC TAAAATTCAA ACCAAATGGA   
  
  
+ ATTTCATAGC TGTCAAATAC AGTGAAGCTG GGGATCCATG GAAAGTCCCC TGCTTTACTC TGCAGCTATA   
  
  
+ AATACACCAA CTAGGACACA ACCATAAACC ACAGTAACC  

- GTTAGTTTAT AGTGCAGAAC GGATGTTCGT ACCAAAAGTT GACAGTTAAA GACGAACTTT CACGAAGGTA   
  
  
- TAATCGTGCT CCTGAGTCTT CCTATTGCTC TTATAGTTCG TTGAATACGG TCAGGAAACA TTAATCGTCT   
  
  
- CAGCTCGTCA AAATTTATTG AAAAGCTAGG AAGTACAAAT TTGACTCTTA CTACATATCT TGATCTTCTG   
  
  
- ACAGCCGTTA AAGAAAGTAA AACACACACA AACCGTCACA ATATCGCTAA CGAAAAGTTT ATCAAAAAGC   
  
  
- ACGGCTTTAT GTACGGTTAC TACAAAAAAA TAAAAAAATT TTAGTAAAAA TTATAGTTGT GTAGTTTTGC   
  
  
- TAGGTTTTCT ATGTTTGGCG TGAGTTAAAA TCGTTTTTTT TTTTAAGTTT TAAATTGTTT TGCGTCCAAA   
  
  
- TTTGCGTGTC GGTTTGTCAG ACAATACCGA CCAAAAATTG AAATAAGATG CTGGTCTAGT ATTTTTTACT   
  
  
- ATAGAACTGA TTAATACACA GCTAATTAAT TTAAATGCTC ATTTATACAA TTTGGTCACA AGTGCTGATA   
  
  
- CGAAGAACTT TGCTGCACCA TAAGTAGTAT AAATAAATGA AGATCTCCAA AAAACATATT TTTTTATTTA   
  
  
- TTTTTTTTAC GTAATACTCT AATCGGCCGA TCTATCGCCC TAACTCGGTC TCGGACTTAA CGTCGACGAA   
  
  
- ATCTATCCCT ACCACTACCT TTTCTTTCCC TTTTAAATAG GTCGTGCTTG TCTTTTCGTT TTGCGGGAAC   
  
  
- GGGCGTTGTT CGGTCCTGCG CACCGCTTCC TCCGAAATTT ACTTACGCTG TCTACAATAA TAACTCCAAA   
  
  
- CTGAACAAAG GGCCAACAAT AACAAAAATA TTTTTTTAAT TAAACAAAAA AAATTTTTTT CAAATGTAAT   
  
  
- AAAATTATAC AGCTGCCTTT ATTATTTAGA ATTTTTTATT TTTTATAATA AAATTATATT AAAATTTATT   
  
  
- TTTATATAAA ATATATTATA TCAAACACAA AACAAATTTA GTATTTAAAT TTTTATAATC TAATTATTTA   
  
  
- AGTGGGATAA AAAAAATACT TAGGGTTAAT CCTAAAATAA AAATTTATAA TCAATTCTTT TTTAATATTA   
  
  
- AATATTAAAT AATTATTGGC ATTGATATTT TTTGATGGCA TTATGTTTTA GTTTACGTTT TAAGAGTTAG   
  
  
- ATTTAAGTAC ATGACAAGAC TGTGTATTAA TTGAGGGGTA AGTCAATGAC AAAAACAAAA ATAGTGAACT   
  
  
- CAATTAAGGA GAAAAAAAAA GATCTTCTTT AAAATTAAAG TAACAAATAT ATTTACCATT TTTTTGTTTT   
  
  
- TACTTTTAGC ACTTGGTAGT TTGTACACGT ACGATTTACG ACGTAAACGA TGGATATTAA CATAAAAGAA   
  
  
- TATTATAACA AAACTTTATA ACTTTTTTGT GATAAAGAAA ATTAGACACA AAAATCTCGA GATAACTAAC   
  
  
- AAAAAAAAAA AAAATGTGAC ACTACTATTT TTGGTTATTG GTGCGGAGTA AGATAATAAT AATAATAGTA   
  
  
- ATAGTGTTCT TTAATATTTT TTAAATAACC TAATTCCTTA CAAATCTTAA CGCAAAATTT AACATAAAAA   
  
  
- TTTTAAAAAC TAAAAAATAA ATTTTAATTA AAAAATCTAA TAAAAATATA TAACTATAGA TTTGATTAAA   
  
  
- ATTTTTTAAT TTTTTATATA ATAAAATTAT ATAAAAATTT ATTTTTTATT AAATTTTTTG TTGAAGATAA   
  
  
- TATGTCCTAT TTTATGGGTA ATGGCTAAAT TGTAAAACTC TTCTTTTTAG ATGTAGGTTC CAAGAATAAG   
  
  
- GAACTCCTGT AATACCAGTA ATGTATAGGT CCTGTTTGGA AACGAACCTG ATTTTAAGTT TGGTTTACCT   
  
  
- TAAAGTATCG ACAGTTTATG TCACTTCGAC CCCTAGGTAC CTTTCAGGGG ACGAAATGAG ACGTCGATAT   
  
  
- TTATGTGGTT GATCCTGTGT TGGTATTTGG TGTCATTGG

+     TATA-box

| Site Name | Organism | Position | Strand | Matrix score. | sequence | function |
| --- | --- | --- | --- | --- | --- | --- |
| TATA-box | Brassica napus | 1748 | + | 6 | ATTATA | core promoter element around -30 of transcription start |
| TATA-box | Arabidopsis thaliana | 1709 | - | 4 | TATA | core promoter element around -30 of transcription start |
| TATA-box | Arabidopsis thaliana | 1715 | - | 8 | TATTTAAA | core promoter element around -30 of transcription start |
| TATA-box | Arabidopsis thaliana | 1384 | - | 4 | TATA | core promoter element around -30 of transcription start |
| TATA-box | Helianthus annuus | 1305 | - | 6 | TATAAA | core promoter element around -30 of transcription start |
| TATA-box | Arabidopsis thaliana | 1655 | - | 7 | TATATAA | core promoter element around -30 of transcription start |
| TATA-box | Arabidopsis thaliana | 1696 | - | 4 | TATA | core promoter element around -30 of transcription start |
| TATA-box | Arabidopsis thaliana | 1749 | - | 5 | TATAA | core promoter element around -30 of transcription start |
| TATA-box | Arabidopsis thaliana | 1309 | - | 4 | TATA | core promoter element around -30 of transcription start |
| TATA-box | Brassica napus | 1708 | - | 6 | ATATAT | core promoter element around -30 of transcription start |
| TATA-box | Arabidopsis thaliana | 1143 | + | 9 | ccTATAAAaa | core promoter element around -30 of transcription start |
| TATA-box | Arabidopsis thaliana | 1306 | - | 7 | TATATAA | core promoter element around -30 of transcription start |
| TATA-box | Arabidopsis thaliana | 984 | + | 4 | TATA | core promoter element around -30 of transcription start |
| TATA-box | Arabidopsis thaliana | 1957 | - | 4 | TATA | core promoter element around -30 of transcription start |
| TATA-box | Arabidopsis thaliana | 1307 | - | 6 | TATATA | core promoter element around -30 of transcription start |
| TATA-box | Arabidopsis thaliana | 1122 | - | 4 | TATA | core promoter element around -30 of transcription start |
| TATA-box | Arabidopsis thaliana | 1121 | - | 5 | TATAA | core promoter element around -30 of transcription start |
| TATA-box | Arabidopsis thaliana | 972 | - | 8 | TATTTAAA | core promoter element around -30 of transcription start |
| TATA-box | Brassica napus | 983 | + | 6 | ATATAT | core promoter element around -30 of transcription start |
| TATA-box | Arabidopsis thaliana | 966 | + | 4 | TATA | core promoter element around -30 of transcription start |
| TATA-box | Arabidopsis thaliana | 1656 | - | 6 | TATATA | core promoter element around -30 of transcription start |
| TATA-box | Avena sativa | 975 | - | 12 | TATATTTATATTT | core promoter element around -30 of transcription start |
| TATA-box | Arabidopsis thaliana | 1750 | - | 4 | TATA | core promoter element around -30 of transcription start |
| TATA-box | Helianthus annuus | 989 | - | 6 | TATAAA | core promoter element around -30 of transcription start |
| TATA-box | Brassica napus | 1657 | - | 6 | ATATAT | core promoter element around -30 of transcription start |
| TATA-box | Arabidopsis thaliana | 868 | + | 4 | TATA | core promoter element around -30 of transcription start |
| TATA-box | Arabidopsis thaliana | 1658 | - | 4 | TATA | core promoter element around -30 of transcription start |
| TATA-box | Arabidopsis thaliana | 1554 | - | 4 | TATA | core promoter element around -30 of transcription start |
| TATA-box | Brassica oleracea | 965 | + | 6 | ATATAA | core promoter element around -30 of transcription start |
| TATA-box | Arabidopsis thaliana | 867 | - | 5 | TATAA | core promoter element around -30 of transcription start |
| TATA-box | Helianthus annuus | 1654 | - | 6 | TATAAA | core promoter element around -30 of transcription start |
| TATA-box | Helianthus annuus | 866 | - | 6 | TATAAA | core promoter element around -30 of transcription start |
| TATA-box | Brassica napus | 1695 | - | 6 | ATATAT | core promoter element around -30 of transcription start |
| TATA-box | Helianthus annuus | 614 | - | 6 | TATACA | core promoter element around -30 of transcription start |
| TATA-box | Brassica oleracea | 1308 | + | 6 | ATATAA | core promoter element around -30 of transcription start |
| TATA-box | Arabidopsis thaliana | 616 | + | 4 | TATA | core promoter element around -30 of transcription start |
| TATA-box | Zea mays | 1396 | - | 8 | TATAAGAA | core promoter element around -30 of transcription start |
| TATA-box | Oryza sativa | 1163 | + | 7 | TACAAAA | core promoter element around -30 of transcription start |
| TATA-box | Arabidopsis thaliana | 1553 | - | 5 | TATAA | core promoter element around -30 of transcription start |
| TATA-box | Arabidopsis thaliana | 250 | - | 5 | TATAA | core promoter element around -30 of transcription start |
| TATA-box | Arabidopsis thaliana | 1627 | + | 8 | TATTTAAA | core promoter element around -30 of transcription start |
| TATA-box | Pisum sativum | 988 | - | 7 | TATAAAA | core promoter element around -30 of transcription start |
| TATA-box | Arabidopsis thaliana | 1399 | - | 5 | TATAA | core promoter element around -30 of transcription start |
| TATA-box | Helianthus annuus | 1120 | - | 6 | TATAAA | core promoter element around -30 of transcription start |
| TATA-box | Arabidopsis thaliana | 1400 | - | 4 | TATA | core promoter element around -30 of transcription start |
| TATA-box | Brassica napus | 1552 | + | 6 | ATTATA | core promoter element around -30 of transcription start |
| TATA-box | Arabidopsis thaliana | 251 | + | 4 | TATA | core promoter element around -30 of transcription start |
| TATA-box | Brassica juncea | 1119 | - | 7 | TATAAAT | core promoter element around -30 of transcription start |
| TATA-box | Pisum sativum | 1653 | - | 7 | TATAAAA | core promoter element around -30 of transcription start |
| TATA-box | Arabidopsis thaliana | 195 | + | 4 | TATA | core promoter element around -30 of transcription start |
| TATA-box | Arabidopsis thaliana | 1115 | - | 4 | TATA | core promoter element around -30 of transcription start |
| TATA-box | Arabidopsis thaliana | 1114 | - | 5 | TATAA | core promoter element around -30 of transcription start |
| TATA-box | Brassica napus | 1113 | + | 6 | ATTATA | core promoter element around -30 of transcription start |
| TATA-box | Arabidopsis thaliana | 1091 | - | 8 | TATTTAAA | core promoter element around -30 of transcription start |
| TATA-box | Arabidopsis thaliana | 998 | + | 4 | TATA | core promoter element around -30 of transcription start |
| TATA-box | Arabidopsis thaliana | 993 | + | 4 | TATA | core promoter element around -30 of transcription start |
| TATA-box | Brassica oleracea | 992 | + | 6 | ATATAA | core promoter element around -30 of transcription start |
| TATA-box | Arabidopsis thaliana | 991 | + | 6 | TATATA | core promoter element around -30 of transcription start |
| TATA-box | Helianthus annuus | 193 | - | 6 | TATACA | core promoter element around -30 of transcription start |
| TATA-box | Arabidopsis thaliana | 1145 | - | 4 | TATA | core promoter element around -30 of transcription start |
| TATA-box | Arabidopsis thaliana | 990 | - | 7 | TATATAA | core promoter element around -30 of transcription start |
| TATA-box | Arabidopsis thaliana | 151 | - | 8 | TATTTAAA | core promoter element around -30 of transcription start |
| TATA-box | Pisum sativum | 865 | - | 7 | TATAAAA | core promoter element around -30 of transcription start |
| TATA-box | Oryza sativa | 611 | - | 7 | TACAAAA | core promoter element around -30 of transcription start |

>Potri.003G139300.1   
+ CAATCAAATA TCACGTCTTG CCTACAAGCA TGGTTTTCAA CTGTCAATTT CTGCTTGAAA GTGCTTCCAT   
  
  
+ ATTAGCACGA GGACTCAGAA GGATAACGAG AATATCAAGC AACTTATGCC AGTCCTTTGT AATTAGCAGA   
  
  
+ GTCGAGCAGT TTTAAATAAC TTTTCGATCC TTCATGTTTA AACTGAGAAT GATGTATAGA ACTAGAAGAC   
  
  
+ TGTCGGCAAT TTCTTTCATT TTGTGTGTGT TTGGCAGTGT TATAGCGATT GCTTTTCAAA TAGTTTTTCG   
  
  
+ TGCCGAAATA CATGCCAATG ATGTTTTTTT ATTTTTTTAA AATCATTTTT AATATCAACA CATCAAAACG   
  
  
+ ATCCAAAAGA TACAAACCGC ACTCAATTTT AGCAAAAAAA AAAATTCAAA ATTTAACAAA ACGCAGGTTT   
  
  
+ AAACGCACAG CCAAACAGTC TGTTATGGCT GGTTTTTAAC TTTATTCTAC GACCAGATCA TAAAAAATGA   
  
  
+ TATCTTGACT AATTATGTGT CGATTAATTA AATTTACGAG TAAATATGTT AAACCAGTGT TCACGACTAT   
  
  
+ GCTTCTTGAA ACGACGTGGT ATTCATCATA TTTATTTACT TCTAGAGGTT TTTTGTATAA AAAAATAAAT   
  
  
+ AAAAAAAATG CATTATGAGA TTAGCCGGCT AGATAGCGGG ATTGAGCCAG AGCCTGAATT GCAGCTGCTT   
  
  
+ TAGATAGGGA TGGTGATGGA AAAGAAAGGG AAAATTTATC CAGCACGAAC AGAAAAGCAA AACGCCCTTG   
  
  
+ CCCGCAACAA GCCAGGACGC GTGGCGAAGG AGGCTTTAAA TGAATGCGAC AGATGTTATT ATTGAGGTTT   
  
  
+ GACTTGTTTC CCGGTTGTTA TTGTTTTTAT AAAAAAATTA ATTTGTTTTT TTTAAAAAAA GTTTACATTA   
  
  
+ TTTTAATATG TCGACGGAAA TAATAAATCT TAAAAAATAA AAAATATTAT TTTAATATAA TTTTAAATAA   
  
  
+ AAATATATTT TATATAATAT AGTTTGTGTT TTGTTTAAAT CATAAATTTA AAAATATTAG ATTAATAAAT   
  
  
+ TCACCCTATT TTTTTTATGA ATCCCAATTA GGATTTTATT TTTAAATATT AGTTAAGAAA AAATTATAAT   
  
  
+ TTATAATTTA TTAATAACCG TAACTATAAA AAACTACCGT AATACAAAAT CAAATGCAAA ATTCTCAATC   
  
  
+ TAAATTCATG TACTGTTCTG ACACATAATT AACTCCCCAT TCAGTTACTG TTTTTGTTTT TATCACTTGA   
  
  
+ GTTAATTCCT CTTTTTTTTT CTAGAAGAAA TTTTAATTTC ATTGTTTATA TAAATGGTAA AAAAACAAAA   
  
  
+ ATGAAAATCG TGAACCATCA AACATGTGCA TGCTAAATGC TGCATTTGCT ACCTATAATT GTATTTTCTT   
  
  
+ ATAATATTGT TTTGAAATAT TGAAAAAACA CTATTTCTTT TAATCTGTGT TTTTAGAGCT CTATTGATTG   
  
  
+ TTTTTTTTTT TTTTACACTG TGATGATAAA AACCAATAAC CACGCCTCAT TCTATTATTA TTATTATCAT   
  
  
+ TATCACAAGA AATTATAAAA AATTTATTGG ATTAAGGAAT GTTTAGAATT GCGTTTTAAA TTGTATTTTT   
  
  
+ AAAATTTTTG ATTTTTTATT TAAAATTAAT TTTTTAGATT ATTTTTATAT ATTGATATCT AAACTAATTT   
  
  
+ TAAAAAATTA AAAAATATAT TATTTTAATA TATTTTTAAA TAAAAAATAA TTTAAAAAAC AACTTCTATT   
  
  
+ ATACAGGATA AAATACCCAT TACCGATTTA ACATTTTGAG AAGAAAAATC TACATCCAAG GTTCTTATTC   
  
  
+ CTTGAGGACA TTATGGTCAT TACATATCCA GGACAAACCT TTGCTTGGAC TAAAATTCAA ACCAAATGGA   
  
  
+ ATTTCATAGC TGTCAAATAC AGTGAAGCTG GGGATCCATG GAAAGTCCCC TGCTTTACTC TGCAGCTATA   
  
  
+ AATACACCAA CTAGGACACA ACCATAAACC ACAGTAACC  

- GTTAGTTTAT AGTGCAGAAC GGATGTTCGT ACCAAAAGTT GACAGTTAAA GACGAACTTT CACGAAGGTA   
  
  
- TAATCGTGCT CCTGAGTCTT CCTATTGCTC TTATAGTTCG TTGAATACGG TCAGGAAACA TTAATCGTCT   
  
  
- CAGCTCGTCA AAATTTATTG AAAAGCTAGG AAGTACAAAT TTGACTCTTA CTACATATCT TGATCTTCTG   
  
  
- ACAGCCGTTA AAGAAAGTAA AACACACACA AACCGTCACA ATATCGCTAA CGAAAAGTTT ATCAAAAAGC   
  
  
- ACGGCTTTAT GTACGGTTAC TACAAAAAAA TAAAAAAATT TTAGTAAAAA TTATAGTTGT GTAGTTTTGC   
  
  
- TAGGTTTTCT ATGTTTGGCG TGAGTTAAAA TCGTTTTTTT TTTTAAGTTT TAAATTGTTT TGCGTCCAAA   
  
  
- TTTGCGTGTC GGTTTGTCAG ACAATACCGA CCAAAAATTG AAATAAGATG CTGGTCTAGT ATTTTTTACT   
  
  
- ATAGAACTGA TTAATACACA GCTAATTAAT TTAAATGCTC ATTTATACAA TTTGGTCACA AGTGCTGATA   
  
  
- CGAAGAACTT TGCTGCACCA TAAGTAGTAT AAATAAATGA AGATCTCCAA AAAACATATT TTTTTATTTA   
  
  
- TTTTTTTTAC GTAATACTCT AATCGGCCGA TCTATCGCCC TAACTCGGTC TCGGACTTAA CGTCGACGAA   
  
  
- ATCTATCCCT ACCACTACCT TTTCTTTCCC TTTTAAATAG GTCGTGCTTG TCTTTTCGTT TTGCGGGAAC   
  
  
- GGGCGTTGTT CGGTCCTGCG CACCGCTTCC TCCGAAATTT ACTTACGCTG TCTACAATAA TAACTCCAAA   
  
  
- CTGAACAAAG GGCCAACAAT AACAAAAATA TTTTTTTAAT TAAACAAAAA AAATTTTTTT CAAATGTAAT   
  
  
- AAAATTATAC AGCTGCCTTT ATTATTTAGA ATTTTTTATT TTTTATAATA AAATTATATT AAAATTTATT   
  
  
- TTTATATAAA ATATATTATA TCAAACACAA AACAAATTTA GTATTTAAAT TTTTATAATC TAATTATTTA   
  
  
- AGTGGGATAA AAAAAATACT TAGGGTTAAT CCTAAAATAA AAATTTATAA TCAATTCTTT TTTAATATTA   
  
  
- AATATTAAAT AATTATTGGC ATTGATATTT TTTGATGGCA TTATGTTTTA GTTTACGTTT TAAGAGTTAG   
  
  
- ATTTAAGTAC ATGACAAGAC TGTGTATTAA TTGAGGGGTA AGTCAATGAC AAAAACAAAA ATAGTGAACT   
  
  
- CAATTAAGGA GAAAAAAAAA GATCTTCTTT AAAATTAAAG TAACAAATAT ATTTACCATT TTTTTGTTTT   
  
  
- TACTTTTAGC ACTTGGTAGT TTGTACACGT ACGATTTACG ACGTAAACGA TGGATATTAA CATAAAAGAA   
  
  
- TATTATAACA AAACTTTATA ACTTTTTTGT GATAAAGAAA ATTAGACACA AAAATCTCGA GATAACTAAC   
  
  
- AAAAAAAAAA AAAATGTGAC ACTACTATTT TTGGTTATTG GTGCGGAGTA AGATAATAAT AATAATAGTA   
  
  
- ATAGTGTTCT TTAATATTTT TTAAATAACC TAATTCCTTA CAAATCTTAA CGCAAAATTT AACATAAAAA   
  
  
- TTTTAAAAAC TAAAAAATAA ATTTTAATTA AAAAATCTAA TAAAAATATA TAACTATAGA TTTGATTAAA   
  
  
- ATTTTTTAAT TTTTTATATA ATAAAATTAT ATAAAAATTT ATTTTTTATT AAATTTTTTG TTGAAGATAA   
  
  
- TATGTCCTAT TTTATGGGTA ATGGCTAAAT TGTAAAACTC TTCTTTTTAG ATGTAGGTTC CAAGAATAAG   
  
  
- GAACTCCTGT AATACCAGTA ATGTATAGGT CCTGTTTGGA AACGAACCTG ATTTTAAGTT TGGTTTACCT   
  
  
- TAAAGTATCG ACAGTTTATG TCACTTCGAC CCCTAGGTAC CTTTCAGGGG ACGAAATGAG ACGTCGATAT   
  
  
- TTATGTGGTT GATCCTGTGT TGGTATTTGG TGTCATTGG

+     TC-rich repeats

| Site Name | Organism | Position | Strand | Matrix score. | sequence | function |
| --- | --- | --- | --- | --- | --- | --- |
| TC-rich repeats | Nicotiana tabacum | 1317 | - | 9 | GTTTTCTTAC | cis-acting element involved in defense and stress responsiveness |

>Potri.003G139300.1   
+ CAATCAAATA TCACGTCTTG CCTACAAGCA TGGTTTTCAA CTGTCAATTT CTGCTTGAAA GTGCTTCCAT   
  
  
+ ATTAGCACGA GGACTCAGAA GGATAACGAG AATATCAAGC AACTTATGCC AGTCCTTTGT AATTAGCAGA   
  
  
+ GTCGAGCAGT TTTAAATAAC TTTTCGATCC TTCATGTTTA AACTGAGAAT GATGTATAGA ACTAGAAGAC   
  
  
+ TGTCGGCAAT TTCTTTCATT TTGTGTGTGT TTGGCAGTGT TATAGCGATT GCTTTTCAAA TAGTTTTTCG   
  
  
+ TGCCGAAATA CATGCCAATG ATGTTTTTTT ATTTTTTTAA AATCATTTTT AATATCAACA CATCAAAACG   
  
  
+ ATCCAAAAGA TACAAACCGC ACTCAATTTT AGCAAAAAAA AAAATTCAAA ATTTAACAAA ACGCAGGTTT   
  
  
+ AAACGCACAG CCAAACAGTC TGTTATGGCT GGTTTTTAAC TTTATTCTAC GACCAGATCA TAAAAAATGA   
  
  
+ TATCTTGACT AATTATGTGT CGATTAATTA AATTTACGAG TAAATATGTT AAACCAGTGT TCACGACTAT   
  
  
+ GCTTCTTGAA ACGACGTGGT ATTCATCATA TTTATTTACT TCTAGAGGTT TTTTGTATAA AAAAATAAAT   
  
  
+ AAAAAAAATG CATTATGAGA TTAGCCGGCT AGATAGCGGG ATTGAGCCAG AGCCTGAATT GCAGCTGCTT   
  
  
+ TAGATAGGGA TGGTGATGGA AAAGAAAGGG AAAATTTATC CAGCACGAAC AGAAAAGCAA AACGCCCTTG   
  
  
+ CCCGCAACAA GCCAGGACGC GTGGCGAAGG AGGCTTTAAA TGAATGCGAC AGATGTTATT ATTGAGGTTT   
  
  
+ GACTTGTTTC CCGGTTGTTA TTGTTTTTAT AAAAAAATTA ATTTGTTTTT TTTAAAAAAA GTTTACATTA   
  
  
+ TTTTAATATG TCGACGGAAA TAATAAATCT TAAAAAATAA AAAATATTAT TTTAATATAA TTTTAAATAA   
  
  
+ AAATATATTT TATATAATAT AGTTTGTGTT TTGTTTAAAT CATAAATTTA AAAATATTAG ATTAATAAAT   
  
  
+ TCACCCTATT TTTTTTATGA ATCCCAATTA GGATTTTATT TTTAAATATT AGTTAAGAAA AAATTATAAT   
  
  
+ TTATAATTTA TTAATAACCG TAACTATAAA AAACTACCGT AATACAAAAT CAAATGCAAA ATTCTCAATC   
  
  
+ TAAATTCATG TACTGTTCTG ACACATAATT AACTCCCCAT TCAGTTACTG TTTTTGTTTT TATCACTTGA   
  
  
+ GTTAATTCCT CTTTTTTTTT CTAGAAGAAA TTTTAATTTC ATTGTTTATA TAAATGGTAA AAAAACAAAA   
  
  
+ ATGAAAATCG TGAACCATCA AACATGTGCA TGCTAAATGC TGCATTTGCT ACCTATAATT GTATTTTCTT   
  
  
+ ATAATATTGT TTTGAAATAT TGAAAAAACA CTATTTCTTT TAATCTGTGT TTTTAGAGCT CTATTGATTG   
  
  
+ TTTTTTTTTT TTTTACACTG TGATGATAAA AACCAATAAC CACGCCTCAT TCTATTATTA TTATTATCAT   
  
  
+ TATCACAAGA AATTATAAAA AATTTATTGG ATTAAGGAAT GTTTAGAATT GCGTTTTAAA TTGTATTTTT   
  
  
+ AAAATTTTTG ATTTTTTATT TAAAATTAAT TTTTTAGATT ATTTTTATAT ATTGATATCT AAACTAATTT   
  
  
+ TAAAAAATTA AAAAATATAT TATTTTAATA TATTTTTAAA TAAAAAATAA TTTAAAAAAC AACTTCTATT   
  
  
+ ATACAGGATA AAATACCCAT TACCGATTTA ACATTTTGAG AAGAAAAATC TACATCCAAG GTTCTTATTC   
  
  
+ CTTGAGGACA TTATGGTCAT TACATATCCA GGACAAACCT TTGCTTGGAC TAAAATTCAA ACCAAATGGA   
  
  
+ ATTTCATAGC TGTCAAATAC AGTGAAGCTG GGGATCCATG GAAAGTCCCC TGCTTTACTC TGCAGCTATA   
  
  
+ AATACACCAA CTAGGACACA ACCATAAACC ACAGTAACC  

- GTTAGTTTAT AGTGCAGAAC GGATGTTCGT ACCAAAAGTT GACAGTTAAA GACGAACTTT CACGAAGGTA   
  
  
- TAATCGTGCT CCTGAGTCTT CCTATTGCTC TTATAGTTCG TTGAATACGG TCAGGAAACA TTAATCGTCT   
  
  
- CAGCTCGTCA AAATTTATTG AAAAGCTAGG AAGTACAAAT TTGACTCTTA CTACATATCT TGATCTTCTG   
  
  
- ACAGCCGTTA AAGAAAGTAA AACACACACA AACCGTCACA ATATCGCTAA CGAAAAGTTT ATCAAAAAGC   
  
  
- ACGGCTTTAT GTACGGTTAC TACAAAAAAA TAAAAAAATT TTAGTAAAAA TTATAGTTGT GTAGTTTTGC   
  
  
- TAGGTTTTCT ATGTTTGGCG TGAGTTAAAA TCGTTTTTTT TTTTAAGTTT TAAATTGTTT TGCGTCCAAA   
  
  
- TTTGCGTGTC GGTTTGTCAG ACAATACCGA CCAAAAATTG AAATAAGATG CTGGTCTAGT ATTTTTTACT   
  
  
- ATAGAACTGA TTAATACACA GCTAATTAAT TTAAATGCTC ATTTATACAA TTTGGTCACA AGTGCTGATA   
  
  
- CGAAGAACTT TGCTGCACCA TAAGTAGTAT AAATAAATGA AGATCTCCAA AAAACATATT TTTTTATTTA   
  
  
- TTTTTTTTAC GTAATACTCT AATCGGCCGA TCTATCGCCC TAACTCGGTC TCGGACTTAA CGTCGACGAA   
  
  
- ATCTATCCCT ACCACTACCT TTTCTTTCCC TTTTAAATAG GTCGTGCTTG TCTTTTCGTT TTGCGGGAAC   
  
  
- GGGCGTTGTT CGGTCCTGCG CACCGCTTCC TCCGAAATTT ACTTACGCTG TCTACAATAA TAACTCCAAA   
  
  
- CTGAACAAAG GGCCAACAAT AACAAAAATA TTTTTTTAAT TAAACAAAAA AAATTTTTTT CAAATGTAAT   
  
  
- AAAATTATAC AGCTGCCTTT ATTATTTAGA ATTTTTTATT TTTTATAATA AAATTATATT AAAATTTATT   
  
  
- TTTATATAAA ATATATTATA TCAAACACAA AACAAATTTA GTATTTAAAT TTTTATAATC TAATTATTTA   
  
  
- AGTGGGATAA AAAAAATACT TAGGGTTAAT CCTAAAATAA AAATTTATAA TCAATTCTTT TTTAATATTA   
  
  
- AATATTAAAT AATTATTGGC ATTGATATTT TTTGATGGCA TTATGTTTTA GTTTACGTTT TAAGAGTTAG   
  
  
- ATTTAAGTAC ATGACAAGAC TGTGTATTAA TTGAGGGGTA AGTCAATGAC AAAAACAAAA ATAGTGAACT   
  
  
- CAATTAAGGA GAAAAAAAAA GATCTTCTTT AAAATTAAAG TAACAAATAT ATTTACCATT TTTTTGTTTT   
  
  
- TACTTTTAGC ACTTGGTAGT TTGTACACGT ACGATTTACG ACGTAAACGA TGGATATTAA CATAAAAGAA   
  
  
- TATTATAACA AAACTTTATA ACTTTTTTGT GATAAAGAAA ATTAGACACA AAAATCTCGA GATAACTAAC   
  
  
- AAAAAAAAAA AAAATGTGAC ACTACTATTT TTGGTTATTG GTGCGGAGTA AGATAATAAT AATAATAGTA   
  
  
- ATAGTGTTCT TTAATATTTT TTAAATAACC TAATTCCTTA CAAATCTTAA CGCAAAATTT AACATAAAAA   
  
  
- TTTTAAAAAC TAAAAAATAA ATTTTAATTA AAAAATCTAA TAAAAATATA TAACTATAGA TTTGATTAAA   
  
  
- ATTTTTTAAT TTTTTATATA ATAAAATTAT ATAAAAATTT ATTTTTTATT AAATTTTTTG TTGAAGATAA   
  
  
- TATGTCCTAT TTTATGGGTA ATGGCTAAAT TGTAAAACTC TTCTTTTTAG ATGTAGGTTC CAAGAATAAG   
  
  
- GAACTCCTGT AATACCAGTA ATGTATAGGT CCTGTTTGGA AACGAACCTG ATTTTAAGTT TGGTTTACCT   
  
  
- TAAAGTATCG ACAGTTTATG TCACTTCGAC CCCTAGGTAC CTTTCAGGGG ACGAAATGAG ACGTCGATAT   
  
  
- TTATGTGGTT GATCCTGTGT TGGTATTTGG TGTCATTGG

+     TGA-element

| Site Name | Organism | Position | Strand | Matrix score. | sequence | function |
| --- | --- | --- | --- | --- | --- | --- |
| TGA-element | Brassica oleracea | 570 | + | 6 | AACGAC | auxin-responsive element |

>Potri.003G139300.1   
+ CAATCAAATA TCACGTCTTG CCTACAAGCA TGGTTTTCAA CTGTCAATTT CTGCTTGAAA GTGCTTCCAT   
  
  
+ ATTAGCACGA GGACTCAGAA GGATAACGAG AATATCAAGC AACTTATGCC AGTCCTTTGT AATTAGCAGA   
  
  
+ GTCGAGCAGT TTTAAATAAC TTTTCGATCC TTCATGTTTA AACTGAGAAT GATGTATAGA ACTAGAAGAC   
  
  
+ TGTCGGCAAT TTCTTTCATT TTGTGTGTGT TTGGCAGTGT TATAGCGATT GCTTTTCAAA TAGTTTTTCG   
  
  
+ TGCCGAAATA CATGCCAATG ATGTTTTTTT ATTTTTTTAA AATCATTTTT AATATCAACA CATCAAAACG   
  
  
+ ATCCAAAAGA TACAAACCGC ACTCAATTTT AGCAAAAAAA AAAATTCAAA ATTTAACAAA ACGCAGGTTT   
  
  
+ AAACGCACAG CCAAACAGTC TGTTATGGCT GGTTTTTAAC TTTATTCTAC GACCAGATCA TAAAAAATGA   
  
  
+ TATCTTGACT AATTATGTGT CGATTAATTA AATTTACGAG TAAATATGTT AAACCAGTGT TCACGACTAT   
  
  
+ GCTTCTTGAA ACGACGTGGT ATTCATCATA TTTATTTACT TCTAGAGGTT TTTTGTATAA AAAAATAAAT   
  
  
+ AAAAAAAATG CATTATGAGA TTAGCCGGCT AGATAGCGGG ATTGAGCCAG AGCCTGAATT GCAGCTGCTT   
  
  
+ TAGATAGGGA TGGTGATGGA AAAGAAAGGG AAAATTTATC CAGCACGAAC AGAAAAGCAA AACGCCCTTG   
  
  
+ CCCGCAACAA GCCAGGACGC GTGGCGAAGG AGGCTTTAAA TGAATGCGAC AGATGTTATT ATTGAGGTTT   
  
  
+ GACTTGTTTC CCGGTTGTTA TTGTTTTTAT AAAAAAATTA ATTTGTTTTT TTTAAAAAAA GTTTACATTA   
  
  
+ TTTTAATATG TCGACGGAAA TAATAAATCT TAAAAAATAA AAAATATTAT TTTAATATAA TTTTAAATAA   
  
  
+ AAATATATTT TATATAATAT AGTTTGTGTT TTGTTTAAAT CATAAATTTA AAAATATTAG ATTAATAAAT   
  
  
+ TCACCCTATT TTTTTTATGA ATCCCAATTA GGATTTTATT TTTAAATATT AGTTAAGAAA AAATTATAAT   
  
  
+ TTATAATTTA TTAATAACCG TAACTATAAA AAACTACCGT AATACAAAAT CAAATGCAAA ATTCTCAATC   
  
  
+ TAAATTCATG TACTGTTCTG ACACATAATT AACTCCCCAT TCAGTTACTG TTTTTGTTTT TATCACTTGA   
  
  
+ GTTAATTCCT CTTTTTTTTT CTAGAAGAAA TTTTAATTTC ATTGTTTATA TAAATGGTAA AAAAACAAAA   
  
  
+ ATGAAAATCG TGAACCATCA AACATGTGCA TGCTAAATGC TGCATTTGCT ACCTATAATT GTATTTTCTT   
  
  
+ ATAATATTGT TTTGAAATAT TGAAAAAACA CTATTTCTTT TAATCTGTGT TTTTAGAGCT CTATTGATTG   
  
  
+ TTTTTTTTTT TTTTACACTG TGATGATAAA AACCAATAAC CACGCCTCAT TCTATTATTA TTATTATCAT   
  
  
+ TATCACAAGA AATTATAAAA AATTTATTGG ATTAAGGAAT GTTTAGAATT GCGTTTTAAA TTGTATTTTT   
  
  
+ AAAATTTTTG ATTTTTTATT TAAAATTAAT TTTTTAGATT ATTTTTATAT ATTGATATCT AAACTAATTT   
  
  
+ TAAAAAATTA AAAAATATAT TATTTTAATA TATTTTTAAA TAAAAAATAA TTTAAAAAAC AACTTCTATT   
  
  
+ ATACAGGATA AAATACCCAT TACCGATTTA ACATTTTGAG AAGAAAAATC TACATCCAAG GTTCTTATTC   
  
  
+ CTTGAGGACA TTATGGTCAT TACATATCCA GGACAAACCT TTGCTTGGAC TAAAATTCAA ACCAAATGGA   
  
  
+ ATTTCATAGC TGTCAAATAC AGTGAAGCTG GGGATCCATG GAAAGTCCCC TGCTTTACTC TGCAGCTATA   
  
  
+ AATACACCAA CTAGGACACA ACCATAAACC ACAGTAACC  

- GTTAGTTTAT AGTGCAGAAC GGATGTTCGT ACCAAAAGTT GACAGTTAAA GACGAACTTT CACGAAGGTA   
  
  
- TAATCGTGCT CCTGAGTCTT CCTATTGCTC TTATAGTTCG TTGAATACGG TCAGGAAACA TTAATCGTCT   
  
  
- CAGCTCGTCA AAATTTATTG AAAAGCTAGG AAGTACAAAT TTGACTCTTA CTACATATCT TGATCTTCTG   
  
  
- ACAGCCGTTA AAGAAAGTAA AACACACACA AACCGTCACA ATATCGCTAA CGAAAAGTTT ATCAAAAAGC   
  
  
- ACGGCTTTAT GTACGGTTAC TACAAAAAAA TAAAAAAATT TTAGTAAAAA TTATAGTTGT GTAGTTTTGC   
  
  
- TAGGTTTTCT ATGTTTGGCG TGAGTTAAAA TCGTTTTTTT TTTTAAGTTT TAAATTGTTT TGCGTCCAAA   
  
  
- TTTGCGTGTC GGTTTGTCAG ACAATACCGA CCAAAAATTG AAATAAGATG CTGGTCTAGT ATTTTTTACT   
  
  
- ATAGAACTGA TTAATACACA GCTAATTAAT TTAAATGCTC ATTTATACAA TTTGGTCACA AGTGCTGATA   
  
  
- CGAAGAACTT TGCTGCACCA TAAGTAGTAT AAATAAATGA AGATCTCCAA AAAACATATT TTTTTATTTA   
  
  
- TTTTTTTTAC GTAATACTCT AATCGGCCGA TCTATCGCCC TAACTCGGTC TCGGACTTAA CGTCGACGAA   
  
  
- ATCTATCCCT ACCACTACCT TTTCTTTCCC TTTTAAATAG GTCGTGCTTG TCTTTTCGTT TTGCGGGAAC   
  
  
- GGGCGTTGTT CGGTCCTGCG CACCGCTTCC TCCGAAATTT ACTTACGCTG TCTACAATAA TAACTCCAAA   
  
  
- CTGAACAAAG GGCCAACAAT AACAAAAATA TTTTTTTAAT TAAACAAAAA AAATTTTTTT CAAATGTAAT   
  
  
- AAAATTATAC AGCTGCCTTT ATTATTTAGA ATTTTTTATT TTTTATAATA AAATTATATT AAAATTTATT   
  
  
- TTTATATAAA ATATATTATA TCAAACACAA AACAAATTTA GTATTTAAAT TTTTATAATC TAATTATTTA   
  
  
- AGTGGGATAA AAAAAATACT TAGGGTTAAT CCTAAAATAA AAATTTATAA TCAATTCTTT TTTAATATTA   
  
  
- AATATTAAAT AATTATTGGC ATTGATATTT TTTGATGGCA TTATGTTTTA GTTTACGTTT TAAGAGTTAG   
  
  
- ATTTAAGTAC ATGACAAGAC TGTGTATTAA TTGAGGGGTA AGTCAATGAC AAAAACAAAA ATAGTGAACT   
  
  
- CAATTAAGGA GAAAAAAAAA GATCTTCTTT AAAATTAAAG TAACAAATAT ATTTACCATT TTTTTGTTTT   
  
  
- TACTTTTAGC ACTTGGTAGT TTGTACACGT ACGATTTACG ACGTAAACGA TGGATATTAA CATAAAAGAA   
  
  
- TATTATAACA AAACTTTATA ACTTTTTTGT GATAAAGAAA ATTAGACACA AAAATCTCGA GATAACTAAC   
  
  
- AAAAAAAAAA AAAATGTGAC ACTACTATTT TTGGTTATTG GTGCGGAGTA AGATAATAAT AATAATAGTA   
  
  
- ATAGTGTTCT TTAATATTTT TTAAATAACC TAATTCCTTA CAAATCTTAA CGCAAAATTT AACATAAAAA   
  
  
- TTTTAAAAAC TAAAAAATAA ATTTTAATTA AAAAATCTAA TAAAAATATA TAACTATAGA TTTGATTAAA   
  
  
- ATTTTTTAAT TTTTTATATA ATAAAATTAT ATAAAAATTT ATTTTTTATT AAATTTTTTG TTGAAGATAA   
  
  
- TATGTCCTAT TTTATGGGTA ATGGCTAAAT TGTAAAACTC TTCTTTTTAG ATGTAGGTTC CAAGAATAAG   
  
  
- GAACTCCTGT AATACCAGTA ATGTATAGGT CCTGTTTGGA AACGAACCTG ATTTTAAGTT TGGTTTACCT   
  
  
- TAAAGTATCG ACAGTTTATG TCACTTCGAC CCCTAGGTAC CTTTCAGGGG ACGAAATGAG ACGTCGATAT   
  
  
- TTATGTGGTT GATCCTGTGT TGGTATTTGG TGTCATTGG

+     Unnamed\_\_1

| Site Name | Organism | Position | Strand | Matrix score. | sequence | function |
| --- | --- | --- | --- | --- | --- | --- |
| Unnamed\_\_1 | Glycine max | 514 | - | 11 | GAATTTAATTAA | 60K protein binding site |
| Unnamed\_\_1 | Zea mays | 790 | + | 5 | CGTGG |  |
| Unnamed\_\_1 | Zea mays | 575 | + | 5 | CGTGG |  |
| Unnamed\_\_1 | Zea mays | 1510 | - | 5 | CGTGG |  |

>Potri.003G139300.1   
+ CAATCAAATA TCACGTCTTG CCTACAAGCA TGGTTTTCAA CTGTCAATTT CTGCTTGAAA GTGCTTCCAT   
  
  
+ ATTAGCACGA GGACTCAGAA GGATAACGAG AATATCAAGC AACTTATGCC AGTCCTTTGT AATTAGCAGA   
  
  
+ GTCGAGCAGT TTTAAATAAC TTTTCGATCC TTCATGTTTA AACTGAGAAT GATGTATAGA ACTAGAAGAC   
  
  
+ TGTCGGCAAT TTCTTTCATT TTGTGTGTGT TTGGCAGTGT TATAGCGATT GCTTTTCAAA TAGTTTTTCG   
  
  
+ TGCCGAAATA CATGCCAATG ATGTTTTTTT ATTTTTTTAA AATCATTTTT AATATCAACA CATCAAAACG   
  
  
+ ATCCAAAAGA TACAAACCGC ACTCAATTTT AGCAAAAAAA AAAATTCAAA ATTTAACAAA ACGCAGGTTT   
  
  
+ AAACGCACAG CCAAACAGTC TGTTATGGCT GGTTTTTAAC TTTATTCTAC GACCAGATCA TAAAAAATGA   
  
  
+ TATCTTGACT AATTATGTGT CGATTAATTA AATTTACGAG TAAATATGTT AAACCAGTGT TCACGACTAT   
  
  
+ GCTTCTTGAA ACGACGTGGT ATTCATCATA TTTATTTACT TCTAGAGGTT TTTTGTATAA AAAAATAAAT   
  
  
+ AAAAAAAATG CATTATGAGA TTAGCCGGCT AGATAGCGGG ATTGAGCCAG AGCCTGAATT GCAGCTGCTT   
  
  
+ TAGATAGGGA TGGTGATGGA AAAGAAAGGG AAAATTTATC CAGCACGAAC AGAAAAGCAA AACGCCCTTG   
  
  
+ CCCGCAACAA GCCAGGACGC GTGGCGAAGG AGGCTTTAAA TGAATGCGAC AGATGTTATT ATTGAGGTTT   
  
  
+ GACTTGTTTC CCGGTTGTTA TTGTTTTTAT AAAAAAATTA ATTTGTTTTT TTTAAAAAAA GTTTACATTA   
  
  
+ TTTTAATATG TCGACGGAAA TAATAAATCT TAAAAAATAA AAAATATTAT TTTAATATAA TTTTAAATAA   
  
  
+ AAATATATTT TATATAATAT AGTTTGTGTT TTGTTTAAAT CATAAATTTA AAAATATTAG ATTAATAAAT   
  
  
+ TCACCCTATT TTTTTTATGA ATCCCAATTA GGATTTTATT TTTAAATATT AGTTAAGAAA AAATTATAAT   
  
  
+ TTATAATTTA TTAATAACCG TAACTATAAA AAACTACCGT AATACAAAAT CAAATGCAAA ATTCTCAATC   
  
  
+ TAAATTCATG TACTGTTCTG ACACATAATT AACTCCCCAT TCAGTTACTG TTTTTGTTTT TATCACTTGA   
  
  
+ GTTAATTCCT CTTTTTTTTT CTAGAAGAAA TTTTAATTTC ATTGTTTATA TAAATGGTAA AAAAACAAAA   
  
  
+ ATGAAAATCG TGAACCATCA AACATGTGCA TGCTAAATGC TGCATTTGCT ACCTATAATT GTATTTTCTT   
  
  
+ ATAATATTGT TTTGAAATAT TGAAAAAACA CTATTTCTTT TAATCTGTGT TTTTAGAGCT CTATTGATTG   
  
  
+ TTTTTTTTTT TTTTACACTG TGATGATAAA AACCAATAAC CACGCCTCAT TCTATTATTA TTATTATCAT   
  
  
+ TATCACAAGA AATTATAAAA AATTTATTGG ATTAAGGAAT GTTTAGAATT GCGTTTTAAA TTGTATTTTT   
  
  
+ AAAATTTTTG ATTTTTTATT TAAAATTAAT TTTTTAGATT ATTTTTATAT ATTGATATCT AAACTAATTT   
  
  
+ TAAAAAATTA AAAAATATAT TATTTTAATA TATTTTTAAA TAAAAAATAA TTTAAAAAAC AACTTCTATT   
  
  
+ ATACAGGATA AAATACCCAT TACCGATTTA ACATTTTGAG AAGAAAAATC TACATCCAAG GTTCTTATTC   
  
  
+ CTTGAGGACA TTATGGTCAT TACATATCCA GGACAAACCT TTGCTTGGAC TAAAATTCAA ACCAAATGGA   
  
  
+ ATTTCATAGC TGTCAAATAC AGTGAAGCTG GGGATCCATG GAAAGTCCCC TGCTTTACTC TGCAGCTATA   
  
  
+ AATACACCAA CTAGGACACA ACCATAAACC ACAGTAACC  

- GTTAGTTTAT AGTGCAGAAC GGATGTTCGT ACCAAAAGTT GACAGTTAAA GACGAACTTT CACGAAGGTA   
  
  
- TAATCGTGCT CCTGAGTCTT CCTATTGCTC TTATAGTTCG TTGAATACGG TCAGGAAACA TTAATCGTCT   
  
  
- CAGCTCGTCA AAATTTATTG AAAAGCTAGG AAGTACAAAT TTGACTCTTA CTACATATCT TGATCTTCTG   
  
  
- ACAGCCGTTA AAGAAAGTAA AACACACACA AACCGTCACA ATATCGCTAA CGAAAAGTTT ATCAAAAAGC   
  
  
- ACGGCTTTAT GTACGGTTAC TACAAAAAAA TAAAAAAATT TTAGTAAAAA TTATAGTTGT GTAGTTTTGC   
  
  
- TAGGTTTTCT ATGTTTGGCG TGAGTTAAAA TCGTTTTTTT TTTTAAGTTT TAAATTGTTT TGCGTCCAAA   
  
  
- TTTGCGTGTC GGTTTGTCAG ACAATACCGA CCAAAAATTG AAATAAGATG CTGGTCTAGT ATTTTTTACT   
  
  
- ATAGAACTGA TTAATACACA GCTAATTAAT TTAAATGCTC ATTTATACAA TTTGGTCACA AGTGCTGATA   
  
  
- CGAAGAACTT TGCTGCACCA TAAGTAGTAT AAATAAATGA AGATCTCCAA AAAACATATT TTTTTATTTA   
  
  
- TTTTTTTTAC GTAATACTCT AATCGGCCGA TCTATCGCCC TAACTCGGTC TCGGACTTAA CGTCGACGAA   
  
  
- ATCTATCCCT ACCACTACCT TTTCTTTCCC TTTTAAATAG GTCGTGCTTG TCTTTTCGTT TTGCGGGAAC   
  
  
- GGGCGTTGTT CGGTCCTGCG CACCGCTTCC TCCGAAATTT ACTTACGCTG TCTACAATAA TAACTCCAAA   
  
  
- CTGAACAAAG GGCCAACAAT AACAAAAATA TTTTTTTAAT TAAACAAAAA AAATTTTTTT CAAATGTAAT   
  
  
- AAAATTATAC AGCTGCCTTT ATTATTTAGA ATTTTTTATT TTTTATAATA AAATTATATT AAAATTTATT   
  
  
- TTTATATAAA ATATATTATA TCAAACACAA AACAAATTTA GTATTTAAAT TTTTATAATC TAATTATTTA   
  
  
- AGTGGGATAA AAAAAATACT TAGGGTTAAT CCTAAAATAA AAATTTATAA TCAATTCTTT TTTAATATTA   
  
  
- AATATTAAAT AATTATTGGC ATTGATATTT TTTGATGGCA TTATGTTTTA GTTTACGTTT TAAGAGTTAG   
  
  
- ATTTAAGTAC ATGACAAGAC TGTGTATTAA TTGAGGGGTA AGTCAATGAC AAAAACAAAA ATAGTGAACT   
  
  
- CAATTAAGGA GAAAAAAAAA GATCTTCTTT AAAATTAAAG TAACAAATAT ATTTACCATT TTTTTGTTTT   
  
  
- TACTTTTAGC ACTTGGTAGT TTGTACACGT ACGATTTACG ACGTAAACGA TGGATATTAA CATAAAAGAA   
  
  
- TATTATAACA AAACTTTATA ACTTTTTTGT GATAAAGAAA ATTAGACACA AAAATCTCGA GATAACTAAC   
  
  
- AAAAAAAAAA AAAATGTGAC ACTACTATTT TTGGTTATTG GTGCGGAGTA AGATAATAAT AATAATAGTA   
  
  
- ATAGTGTTCT TTAATATTTT TTAAATAACC TAATTCCTTA CAAATCTTAA CGCAAAATTT AACATAAAAA   
  
  
- TTTTAAAAAC TAAAAAATAA ATTTTAATTA AAAAATCTAA TAAAAATATA TAACTATAGA TTTGATTAAA   
  
  
- ATTTTTTAAT TTTTTATATA ATAAAATTAT ATAAAAATTT ATTTTTTATT AAATTTTTTG TTGAAGATAA   
  
  
- TATGTCCTAT TTTATGGGTA ATGGCTAAAT TGTAAAACTC TTCTTTTTAG ATGTAGGTTC CAAGAATAAG   
  
  
- GAACTCCTGT AATACCAGTA ATGTATAGGT CCTGTTTGGA AACGAACCTG ATTTTAAGTT TGGTTTACCT   
  
  
- TAAAGTATCG ACAGTTTATG TCACTTCGAC CCCTAGGTAC CTTTCAGGGG ACGAAATGAG ACGTCGATAT   
  
  
- TTATGTGGTT GATCCTGTGT TGGTATTTGG TGTCATTGG

+     Unnamed\_\_4

| Site Name | Organism | Position | Strand | Matrix score. | sequence | function |
| --- | --- | --- | --- | --- | --- | --- |
| Unnamed\_\_4 | Petroselinum hortense | 799 | - | 4 | CTCC |  |
| Unnamed\_\_4 | Petroselinum hortense | 1223 | + | 4 | CTCC |  |

>Potri.003G139300.1   
+ CAATCAAATA TCACGTCTTG CCTACAAGCA TGGTTTTCAA CTGTCAATTT CTGCTTGAAA GTGCTTCCAT   
  
  
+ ATTAGCACGA GGACTCAGAA GGATAACGAG AATATCAAGC AACTTATGCC AGTCCTTTGT AATTAGCAGA   
  
  
+ GTCGAGCAGT TTTAAATAAC TTTTCGATCC TTCATGTTTA AACTGAGAAT GATGTATAGA ACTAGAAGAC   
  
  
+ TGTCGGCAAT TTCTTTCATT TTGTGTGTGT TTGGCAGTGT TATAGCGATT GCTTTTCAAA TAGTTTTTCG   
  
  
+ TGCCGAAATA CATGCCAATG ATGTTTTTTT ATTTTTTTAA AATCATTTTT AATATCAACA CATCAAAACG   
  
  
+ ATCCAAAAGA TACAAACCGC ACTCAATTTT AGCAAAAAAA AAAATTCAAA ATTTAACAAA ACGCAGGTTT   
  
  
+ AAACGCACAG CCAAACAGTC TGTTATGGCT GGTTTTTAAC TTTATTCTAC GACCAGATCA TAAAAAATGA   
  
  
+ TATCTTGACT AATTATGTGT CGATTAATTA AATTTACGAG TAAATATGTT AAACCAGTGT TCACGACTAT   
  
  
+ GCTTCTTGAA ACGACGTGGT ATTCATCATA TTTATTTACT TCTAGAGGTT TTTTGTATAA AAAAATAAAT   
  
  
+ AAAAAAAATG CATTATGAGA TTAGCCGGCT AGATAGCGGG ATTGAGCCAG AGCCTGAATT GCAGCTGCTT   
  
  
+ TAGATAGGGA TGGTGATGGA AAAGAAAGGG AAAATTTATC CAGCACGAAC AGAAAAGCAA AACGCCCTTG   
  
  
+ CCCGCAACAA GCCAGGACGC GTGGCGAAGG AGGCTTTAAA TGAATGCGAC AGATGTTATT ATTGAGGTTT   
  
  
+ GACTTGTTTC CCGGTTGTTA TTGTTTTTAT AAAAAAATTA ATTTGTTTTT TTTAAAAAAA GTTTACATTA   
  
  
+ TTTTAATATG TCGACGGAAA TAATAAATCT TAAAAAATAA AAAATATTAT TTTAATATAA TTTTAAATAA   
  
  
+ AAATATATTT TATATAATAT AGTTTGTGTT TTGTTTAAAT CATAAATTTA AAAATATTAG ATTAATAAAT   
  
  
+ TCACCCTATT TTTTTTATGA ATCCCAATTA GGATTTTATT TTTAAATATT AGTTAAGAAA AAATTATAAT   
  
  
+ TTATAATTTA TTAATAACCG TAACTATAAA AAACTACCGT AATACAAAAT CAAATGCAAA ATTCTCAATC   
  
  
+ TAAATTCATG TACTGTTCTG ACACATAATT AACTCCCCAT TCAGTTACTG TTTTTGTTTT TATCACTTGA   
  
  
+ GTTAATTCCT CTTTTTTTTT CTAGAAGAAA TTTTAATTTC ATTGTTTATA TAAATGGTAA AAAAACAAAA   
  
  
+ ATGAAAATCG TGAACCATCA AACATGTGCA TGCTAAATGC TGCATTTGCT ACCTATAATT GTATTTTCTT   
  
  
+ ATAATATTGT TTTGAAATAT TGAAAAAACA CTATTTCTTT TAATCTGTGT TTTTAGAGCT CTATTGATTG   
  
  
+ TTTTTTTTTT TTTTACACTG TGATGATAAA AACCAATAAC CACGCCTCAT TCTATTATTA TTATTATCAT   
  
  
+ TATCACAAGA AATTATAAAA AATTTATTGG ATTAAGGAAT GTTTAGAATT GCGTTTTAAA TTGTATTTTT   
  
  
+ AAAATTTTTG ATTTTTTATT TAAAATTAAT TTTTTAGATT ATTTTTATAT ATTGATATCT AAACTAATTT   
  
  
+ TAAAAAATTA AAAAATATAT TATTTTAATA TATTTTTAAA TAAAAAATAA TTTAAAAAAC AACTTCTATT   
  
  
+ ATACAGGATA AAATACCCAT TACCGATTTA ACATTTTGAG AAGAAAAATC TACATCCAAG GTTCTTATTC   
  
  
+ CTTGAGGACA TTATGGTCAT TACATATCCA GGACAAACCT TTGCTTGGAC TAAAATTCAA ACCAAATGGA   
  
  
+ ATTTCATAGC TGTCAAATAC AGTGAAGCTG GGGATCCATG GAAAGTCCCC TGCTTTACTC TGCAGCTATA   
  
  
+ AATACACCAA CTAGGACACA ACCATAAACC ACAGTAACC  

- GTTAGTTTAT AGTGCAGAAC GGATGTTCGT ACCAAAAGTT GACAGTTAAA GACGAACTTT CACGAAGGTA   
  
  
- TAATCGTGCT CCTGAGTCTT CCTATTGCTC TTATAGTTCG TTGAATACGG TCAGGAAACA TTAATCGTCT   
  
  
- CAGCTCGTCA AAATTTATTG AAAAGCTAGG AAGTACAAAT TTGACTCTTA CTACATATCT TGATCTTCTG   
  
  
- ACAGCCGTTA AAGAAAGTAA AACACACACA AACCGTCACA ATATCGCTAA CGAAAAGTTT ATCAAAAAGC   
  
  
- ACGGCTTTAT GTACGGTTAC TACAAAAAAA TAAAAAAATT TTAGTAAAAA TTATAGTTGT GTAGTTTTGC   
  
  
- TAGGTTTTCT ATGTTTGGCG TGAGTTAAAA TCGTTTTTTT TTTTAAGTTT TAAATTGTTT TGCGTCCAAA   
  
  
- TTTGCGTGTC GGTTTGTCAG ACAATACCGA CCAAAAATTG AAATAAGATG CTGGTCTAGT ATTTTTTACT   
  
  
- ATAGAACTGA TTAATACACA GCTAATTAAT TTAAATGCTC ATTTATACAA TTTGGTCACA AGTGCTGATA   
  
  
- CGAAGAACTT TGCTGCACCA TAAGTAGTAT AAATAAATGA AGATCTCCAA AAAACATATT TTTTTATTTA   
  
  
- TTTTTTTTAC GTAATACTCT AATCGGCCGA TCTATCGCCC TAACTCGGTC TCGGACTTAA CGTCGACGAA   
  
  
- ATCTATCCCT ACCACTACCT TTTCTTTCCC TTTTAAATAG GTCGTGCTTG TCTTTTCGTT TTGCGGGAAC   
  
  
- GGGCGTTGTT CGGTCCTGCG CACCGCTTCC TCCGAAATTT ACTTACGCTG TCTACAATAA TAACTCCAAA   
  
  
- CTGAACAAAG GGCCAACAAT AACAAAAATA TTTTTTTAAT TAAACAAAAA AAATTTTTTT CAAATGTAAT   
  
  
- AAAATTATAC AGCTGCCTTT ATTATTTAGA ATTTTTTATT TTTTATAATA AAATTATATT AAAATTTATT   
  
  
- TTTATATAAA ATATATTATA TCAAACACAA AACAAATTTA GTATTTAAAT TTTTATAATC TAATTATTTA   
  
  
- AGTGGGATAA AAAAAATACT TAGGGTTAAT CCTAAAATAA AAATTTATAA TCAATTCTTT TTTAATATTA   
  
  
- AATATTAAAT AATTATTGGC ATTGATATTT TTTGATGGCA TTATGTTTTA GTTTACGTTT TAAGAGTTAG   
  
  
- ATTTAAGTAC ATGACAAGAC TGTGTATTAA TTGAGGGGTA AGTCAATGAC AAAAACAAAA ATAGTGAACT   
  
  
- CAATTAAGGA GAAAAAAAAA GATCTTCTTT AAAATTAAAG TAACAAATAT ATTTACCATT TTTTTGTTTT   
  
  
- TACTTTTAGC ACTTGGTAGT TTGTACACGT ACGATTTACG ACGTAAACGA TGGATATTAA CATAAAAGAA   
  
  
- TATTATAACA AAACTTTATA ACTTTTTTGT GATAAAGAAA ATTAGACACA AAAATCTCGA GATAACTAAC   
  
  
- AAAAAAAAAA AAAATGTGAC ACTACTATTT TTGGTTATTG GTGCGGAGTA AGATAATAAT AATAATAGTA   
  
  
- ATAGTGTTCT TTAATATTTT TTAAATAACC TAATTCCTTA CAAATCTTAA CGCAAAATTT AACATAAAAA   
  
  
- TTTTAAAAAC TAAAAAATAA ATTTTAATTA AAAAATCTAA TAAAAATATA TAACTATAGA TTTGATTAAA   
  
  
- ATTTTTTAAT TTTTTATATA ATAAAATTAT ATAAAAATTT ATTTTTTATT AAATTTTTTG TTGAAGATAA   
  
  
- TATGTCCTAT TTTATGGGTA ATGGCTAAAT TGTAAAACTC TTCTTTTTAG ATGTAGGTTC CAAGAATAAG   
  
  
- GAACTCCTGT AATACCAGTA ATGTATAGGT CCTGTTTGGA AACGAACCTG ATTTTAAGTT TGGTTTACCT   
  
  
- TAAAGTATCG ACAGTTTATG TCACTTCGAC CCCTAGGTAC CTTTCAGGGG ACGAAATGAG ACGTCGATAT   
  
  
- TTATGTGGTT GATCCTGTGT TGGTATTTGG TGTCATTGG

+     WUN-motif

| Site Name | Organism | Position | Strand | Matrix score. | sequence | function |
| --- | --- | --- | --- | --- | --- | --- |
| WUN-motif | Nicotiana glutinosa | 1285 | - | 9 | AAATTTCTT |  |

>Potri.003G139300.1   
+ CAATCAAATA TCACGTCTTG CCTACAAGCA TGGTTTTCAA CTGTCAATTT CTGCTTGAAA GTGCTTCCAT   
  
  
+ ATTAGCACGA GGACTCAGAA GGATAACGAG AATATCAAGC AACTTATGCC AGTCCTTTGT AATTAGCAGA   
  
  
+ GTCGAGCAGT TTTAAATAAC TTTTCGATCC TTCATGTTTA AACTGAGAAT GATGTATAGA ACTAGAAGAC   
  
  
+ TGTCGGCAAT TTCTTTCATT TTGTGTGTGT TTGGCAGTGT TATAGCGATT GCTTTTCAAA TAGTTTTTCG   
  
  
+ TGCCGAAATA CATGCCAATG ATGTTTTTTT ATTTTTTTAA AATCATTTTT AATATCAACA CATCAAAACG   
  
  
+ ATCCAAAAGA TACAAACCGC ACTCAATTTT AGCAAAAAAA AAAATTCAAA ATTTAACAAA ACGCAGGTTT   
  
  
+ AAACGCACAG CCAAACAGTC TGTTATGGCT GGTTTTTAAC TTTATTCTAC GACCAGATCA TAAAAAATGA   
  
  
+ TATCTTGACT AATTATGTGT CGATTAATTA AATTTACGAG TAAATATGTT AAACCAGTGT TCACGACTAT   
  
  
+ GCTTCTTGAA ACGACGTGGT ATTCATCATA TTTATTTACT TCTAGAGGTT TTTTGTATAA AAAAATAAAT   
  
  
+ AAAAAAAATG CATTATGAGA TTAGCCGGCT AGATAGCGGG ATTGAGCCAG AGCCTGAATT GCAGCTGCTT   
  
  
+ TAGATAGGGA TGGTGATGGA AAAGAAAGGG AAAATTTATC CAGCACGAAC AGAAAAGCAA AACGCCCTTG   
  
  
+ CCCGCAACAA GCCAGGACGC GTGGCGAAGG AGGCTTTAAA TGAATGCGAC AGATGTTATT ATTGAGGTTT   
  
  
+ GACTTGTTTC CCGGTTGTTA TTGTTTTTAT AAAAAAATTA ATTTGTTTTT TTTAAAAAAA GTTTACATTA   
  
  
+ TTTTAATATG TCGACGGAAA TAATAAATCT TAAAAAATAA AAAATATTAT TTTAATATAA TTTTAAATAA   
  
  
+ AAATATATTT TATATAATAT AGTTTGTGTT TTGTTTAAAT CATAAATTTA AAAATATTAG ATTAATAAAT   
  
  
+ TCACCCTATT TTTTTTATGA ATCCCAATTA GGATTTTATT TTTAAATATT AGTTAAGAAA AAATTATAAT   
  
  
+ TTATAATTTA TTAATAACCG TAACTATAAA AAACTACCGT AATACAAAAT CAAATGCAAA ATTCTCAATC   
  
  
+ TAAATTCATG TACTGTTCTG ACACATAATT AACTCCCCAT TCAGTTACTG TTTTTGTTTT TATCACTTGA   
  
  
+ GTTAATTCCT CTTTTTTTTT CTAGAAGAAA TTTTAATTTC ATTGTTTATA TAAATGGTAA AAAAACAAAA   
  
  
+ ATGAAAATCG TGAACCATCA AACATGTGCA TGCTAAATGC TGCATTTGCT ACCTATAATT GTATTTTCTT   
  
  
+ ATAATATTGT TTTGAAATAT TGAAAAAACA CTATTTCTTT TAATCTGTGT TTTTAGAGCT CTATTGATTG   
  
  
+ TTTTTTTTTT TTTTACACTG TGATGATAAA AACCAATAAC CACGCCTCAT TCTATTATTA TTATTATCAT   
  
  
+ TATCACAAGA AATTATAAAA AATTTATTGG ATTAAGGAAT GTTTAGAATT GCGTTTTAAA TTGTATTTTT   
  
  
+ AAAATTTTTG ATTTTTTATT TAAAATTAAT TTTTTAGATT ATTTTTATAT ATTGATATCT AAACTAATTT   
  
  
+ TAAAAAATTA AAAAATATAT TATTTTAATA TATTTTTAAA TAAAAAATAA TTTAAAAAAC AACTTCTATT   
  
  
+ ATACAGGATA AAATACCCAT TACCGATTTA ACATTTTGAG AAGAAAAATC TACATCCAAG GTTCTTATTC   
  
  
+ CTTGAGGACA TTATGGTCAT TACATATCCA GGACAAACCT TTGCTTGGAC TAAAATTCAA ACCAAATGGA   
  
  
+ ATTTCATAGC TGTCAAATAC AGTGAAGCTG GGGATCCATG GAAAGTCCCC TGCTTTACTC TGCAGCTATA   
  
  
+ AATACACCAA CTAGGACACA ACCATAAACC ACAGTAACC  

- GTTAGTTTAT AGTGCAGAAC GGATGTTCGT ACCAAAAGTT GACAGTTAAA GACGAACTTT CACGAAGGTA   
  
  
- TAATCGTGCT CCTGAGTCTT CCTATTGCTC TTATAGTTCG TTGAATACGG TCAGGAAACA TTAATCGTCT   
  
  
- CAGCTCGTCA AAATTTATTG AAAAGCTAGG AAGTACAAAT TTGACTCTTA CTACATATCT TGATCTTCTG   
  
  
- ACAGCCGTTA AAGAAAGTAA AACACACACA AACCGTCACA ATATCGCTAA CGAAAAGTTT ATCAAAAAGC   
  
  
- ACGGCTTTAT GTACGGTTAC TACAAAAAAA TAAAAAAATT TTAGTAAAAA TTATAGTTGT GTAGTTTTGC   
  
  
- TAGGTTTTCT ATGTTTGGCG TGAGTTAAAA TCGTTTTTTT TTTTAAGTTT TAAATTGTTT TGCGTCCAAA   
  
  
- TTTGCGTGTC GGTTTGTCAG ACAATACCGA CCAAAAATTG AAATAAGATG CTGGTCTAGT ATTTTTTACT   
  
  
- ATAGAACTGA TTAATACACA GCTAATTAAT TTAAATGCTC ATTTATACAA TTTGGTCACA AGTGCTGATA   
  
  
- CGAAGAACTT TGCTGCACCA TAAGTAGTAT AAATAAATGA AGATCTCCAA AAAACATATT TTTTTATTTA   
  
  
- TTTTTTTTAC GTAATACTCT AATCGGCCGA TCTATCGCCC TAACTCGGTC TCGGACTTAA CGTCGACGAA   
  
  
- ATCTATCCCT ACCACTACCT TTTCTTTCCC TTTTAAATAG GTCGTGCTTG TCTTTTCGTT TTGCGGGAAC   
  
  
- GGGCGTTGTT CGGTCCTGCG CACCGCTTCC TCCGAAATTT ACTTACGCTG TCTACAATAA TAACTCCAAA   
  
  
- CTGAACAAAG GGCCAACAAT AACAAAAATA TTTTTTTAAT TAAACAAAAA AAATTTTTTT CAAATGTAAT   
  
  
- AAAATTATAC AGCTGCCTTT ATTATTTAGA ATTTTTTATT TTTTATAATA AAATTATATT AAAATTTATT   
  
  
- TTTATATAAA ATATATTATA TCAAACACAA AACAAATTTA GTATTTAAAT TTTTATAATC TAATTATTTA   
  
  
- AGTGGGATAA AAAAAATACT TAGGGTTAAT CCTAAAATAA AAATTTATAA TCAATTCTTT TTTAATATTA   
  
  
- AATATTAAAT AATTATTGGC ATTGATATTT TTTGATGGCA TTATGTTTTA GTTTACGTTT TAAGAGTTAG   
  
  
- ATTTAAGTAC ATGACAAGAC TGTGTATTAA TTGAGGGGTA AGTCAATGAC AAAAACAAAA ATAGTGAACT   
  
  
- CAATTAAGGA GAAAAAAAAA GATCTTCTTT AAAATTAAAG TAACAAATAT ATTTACCATT TTTTTGTTTT   
  
  
- TACTTTTAGC ACTTGGTAGT TTGTACACGT ACGATTTACG ACGTAAACGA TGGATATTAA CATAAAAGAA   
  
  
- TATTATAACA AAACTTTATA ACTTTTTTGT GATAAAGAAA ATTAGACACA AAAATCTCGA GATAACTAAC   
  
  
- AAAAAAAAAA AAAATGTGAC ACTACTATTT TTGGTTATTG GTGCGGAGTA AGATAATAAT AATAATAGTA   
  
  
- ATAGTGTTCT TTAATATTTT TTAAATAACC TAATTCCTTA CAAATCTTAA CGCAAAATTT AACATAAAAA   
  
  
- TTTTAAAAAC TAAAAAATAA ATTTTAATTA AAAAATCTAA TAAAAATATA TAACTATAGA TTTGATTAAA   
  
  
- ATTTTTTAAT TTTTTATATA ATAAAATTAT ATAAAAATTT ATTTTTTATT AAATTTTTTG TTGAAGATAA   
  
  
- TATGTCCTAT TTTATGGGTA ATGGCTAAAT TGTAAAACTC TTCTTTTTAG ATGTAGGTTC CAAGAATAAG   
  
  
- GAACTCCTGT AATACCAGTA ATGTATAGGT CCTGTTTGGA AACGAACCTG ATTTTAAGTT TGGTTTACCT   
  
  
- TAAAGTATCG ACAGTTTATG TCACTTCGAC CCCTAGGTAC CTTTCAGGGG ACGAAATGAG ACGTCGATAT   
  
  
- TTATGTGGTT GATCCTGTGT TGGTATTTGG TGTCATTGG

+     chs-CMA2a

| Site Name | Organism | Position | Strand | Matrix score. | sequence | function |
| --- | --- | --- | --- | --- | --- | --- |
| chs-CMA2a | Petroselinum crispum | 1253 | + | 8 | TCACTTGA | part of a light responsive element |

>Potri.003G139300.1   
+ CAATCAAATA TCACGTCTTG CCTACAAGCA TGGTTTTCAA CTGTCAATTT CTGCTTGAAA GTGCTTCCAT   
  
  
+ ATTAGCACGA GGACTCAGAA GGATAACGAG AATATCAAGC AACTTATGCC AGTCCTTTGT AATTAGCAGA   
  
  
+ GTCGAGCAGT TTTAAATAAC TTTTCGATCC TTCATGTTTA AACTGAGAAT GATGTATAGA ACTAGAAGAC   
  
  
+ TGTCGGCAAT TTCTTTCATT TTGTGTGTGT TTGGCAGTGT TATAGCGATT GCTTTTCAAA TAGTTTTTCG   
  
  
+ TGCCGAAATA CATGCCAATG ATGTTTTTTT ATTTTTTTAA AATCATTTTT AATATCAACA CATCAAAACG   
  
  
+ ATCCAAAAGA TACAAACCGC ACTCAATTTT AGCAAAAAAA AAAATTCAAA ATTTAACAAA ACGCAGGTTT   
  
  
+ AAACGCACAG CCAAACAGTC TGTTATGGCT GGTTTTTAAC TTTATTCTAC GACCAGATCA TAAAAAATGA   
  
  
+ TATCTTGACT AATTATGTGT CGATTAATTA AATTTACGAG TAAATATGTT AAACCAGTGT TCACGACTAT   
  
  
+ GCTTCTTGAA ACGACGTGGT ATTCATCATA TTTATTTACT TCTAGAGGTT TTTTGTATAA AAAAATAAAT   
  
  
+ AAAAAAAATG CATTATGAGA TTAGCCGGCT AGATAGCGGG ATTGAGCCAG AGCCTGAATT GCAGCTGCTT   
  
  
+ TAGATAGGGA TGGTGATGGA AAAGAAAGGG AAAATTTATC CAGCACGAAC AGAAAAGCAA AACGCCCTTG   
  
  
+ CCCGCAACAA GCCAGGACGC GTGGCGAAGG AGGCTTTAAA TGAATGCGAC AGATGTTATT ATTGAGGTTT   
  
  
+ GACTTGTTTC CCGGTTGTTA TTGTTTTTAT AAAAAAATTA ATTTGTTTTT TTTAAAAAAA GTTTACATTA   
  
  
+ TTTTAATATG TCGACGGAAA TAATAAATCT TAAAAAATAA AAAATATTAT TTTAATATAA TTTTAAATAA   
  
  
+ AAATATATTT TATATAATAT AGTTTGTGTT TTGTTTAAAT CATAAATTTA AAAATATTAG ATTAATAAAT   
  
  
+ TCACCCTATT TTTTTTATGA ATCCCAATTA GGATTTTATT TTTAAATATT AGTTAAGAAA AAATTATAAT   
  
  
+ TTATAATTTA TTAATAACCG TAACTATAAA AAACTACCGT AATACAAAAT CAAATGCAAA ATTCTCAATC   
  
  
+ TAAATTCATG TACTGTTCTG ACACATAATT AACTCCCCAT TCAGTTACTG TTTTTGTTTT TATCACTTGA   
  
  
+ GTTAATTCCT CTTTTTTTTT CTAGAAGAAA TTTTAATTTC ATTGTTTATA TAAATGGTAA AAAAACAAAA   
  
  
+ ATGAAAATCG TGAACCATCA AACATGTGCA TGCTAAATGC TGCATTTGCT ACCTATAATT GTATTTTCTT   
  
  
+ ATAATATTGT TTTGAAATAT TGAAAAAACA CTATTTCTTT TAATCTGTGT TTTTAGAGCT CTATTGATTG   
  
  
+ TTTTTTTTTT TTTTACACTG TGATGATAAA AACCAATAAC CACGCCTCAT TCTATTATTA TTATTATCAT   
  
  
+ TATCACAAGA AATTATAAAA AATTTATTGG ATTAAGGAAT GTTTAGAATT GCGTTTTAAA TTGTATTTTT   
  
  
+ AAAATTTTTG ATTTTTTATT TAAAATTAAT TTTTTAGATT ATTTTTATAT ATTGATATCT AAACTAATTT   
  
  
+ TAAAAAATTA AAAAATATAT TATTTTAATA TATTTTTAAA TAAAAAATAA TTTAAAAAAC AACTTCTATT   
  
  
+ ATACAGGATA AAATACCCAT TACCGATTTA ACATTTTGAG AAGAAAAATC TACATCCAAG GTTCTTATTC   
  
  
+ CTTGAGGACA TTATGGTCAT TACATATCCA GGACAAACCT TTGCTTGGAC TAAAATTCAA ACCAAATGGA   
  
  
+ ATTTCATAGC TGTCAAATAC AGTGAAGCTG GGGATCCATG GAAAGTCCCC TGCTTTACTC TGCAGCTATA   
  
  
+ AATACACCAA CTAGGACACA ACCATAAACC ACAGTAACC  

- GTTAGTTTAT AGTGCAGAAC GGATGTTCGT ACCAAAAGTT GACAGTTAAA GACGAACTTT CACGAAGGTA   
  
  
- TAATCGTGCT CCTGAGTCTT CCTATTGCTC TTATAGTTCG TTGAATACGG TCAGGAAACA TTAATCGTCT   
  
  
- CAGCTCGTCA AAATTTATTG AAAAGCTAGG AAGTACAAAT TTGACTCTTA CTACATATCT TGATCTTCTG   
  
  
- ACAGCCGTTA AAGAAAGTAA AACACACACA AACCGTCACA ATATCGCTAA CGAAAAGTTT ATCAAAAAGC   
  
  
- ACGGCTTTAT GTACGGTTAC TACAAAAAAA TAAAAAAATT TTAGTAAAAA TTATAGTTGT GTAGTTTTGC   
  
  
- TAGGTTTTCT ATGTTTGGCG TGAGTTAAAA TCGTTTTTTT TTTTAAGTTT TAAATTGTTT TGCGTCCAAA   
  
  
- TTTGCGTGTC GGTTTGTCAG ACAATACCGA CCAAAAATTG AAATAAGATG CTGGTCTAGT ATTTTTTACT   
  
  
- ATAGAACTGA TTAATACACA GCTAATTAAT TTAAATGCTC ATTTATACAA TTTGGTCACA AGTGCTGATA   
  
  
- CGAAGAACTT TGCTGCACCA TAAGTAGTAT AAATAAATGA AGATCTCCAA AAAACATATT TTTTTATTTA   
  
  
- TTTTTTTTAC GTAATACTCT AATCGGCCGA TCTATCGCCC TAACTCGGTC TCGGACTTAA CGTCGACGAA   
  
  
- ATCTATCCCT ACCACTACCT TTTCTTTCCC TTTTAAATAG GTCGTGCTTG TCTTTTCGTT TTGCGGGAAC   
  
  
- GGGCGTTGTT CGGTCCTGCG CACCGCTTCC TCCGAAATTT ACTTACGCTG TCTACAATAA TAACTCCAAA   
  
  
- CTGAACAAAG GGCCAACAAT AACAAAAATA TTTTTTTAAT TAAACAAAAA AAATTTTTTT CAAATGTAAT   
  
  
- AAAATTATAC AGCTGCCTTT ATTATTTAGA ATTTTTTATT TTTTATAATA AAATTATATT AAAATTTATT   
  
  
- TTTATATAAA ATATATTATA TCAAACACAA AACAAATTTA GTATTTAAAT TTTTATAATC TAATTATTTA   
  
  
- AGTGGGATAA AAAAAATACT TAGGGTTAAT CCTAAAATAA AAATTTATAA TCAATTCTTT TTTAATATTA   
  
  
- AATATTAAAT AATTATTGGC ATTGATATTT TTTGATGGCA TTATGTTTTA GTTTACGTTT TAAGAGTTAG   
  
  
- ATTTAAGTAC ATGACAAGAC TGTGTATTAA TTGAGGGGTA AGTCAATGAC AAAAACAAAA ATAGTGAACT   
  
  
- CAATTAAGGA GAAAAAAAAA GATCTTCTTT AAAATTAAAG TAACAAATAT ATTTACCATT TTTTTGTTTT   
  
  
- TACTTTTAGC ACTTGGTAGT TTGTACACGT ACGATTTACG ACGTAAACGA TGGATATTAA CATAAAAGAA   
  
  
- TATTATAACA AAACTTTATA ACTTTTTTGT GATAAAGAAA ATTAGACACA AAAATCTCGA GATAACTAAC   
  
  
- AAAAAAAAAA AAAATGTGAC ACTACTATTT TTGGTTATTG GTGCGGAGTA AGATAATAAT AATAATAGTA   
  
  
- ATAGTGTTCT TTAATATTTT TTAAATAACC TAATTCCTTA CAAATCTTAA CGCAAAATTT AACATAAAAA   
  
  
- TTTTAAAAAC TAAAAAATAA ATTTTAATTA AAAAATCTAA TAAAAATATA TAACTATAGA TTTGATTAAA   
  
  
- ATTTTTTAAT TTTTTATATA ATAAAATTAT ATAAAAATTT ATTTTTTATT AAATTTTTTG TTGAAGATAA   
  
  
- TATGTCCTAT TTTATGGGTA ATGGCTAAAT TGTAAAACTC TTCTTTTTAG ATGTAGGTTC CAAGAATAAG   
  
  
- GAACTCCTGT AATACCAGTA ATGTATAGGT CCTGTTTGGA AACGAACCTG ATTTTAAGTT TGGTTTACCT   
  
  
- TAAAGTATCG ACAGTTTATG TCACTTCGAC CCCTAGGTAC CTTTCAGGGG ACGAAATGAG ACGTCGATAT   
  
  
- TTATGTGGTT GATCCTGTGT TGGTATTTGG TGTCATTGG
