# Supplementary material for: Structure and expression analysis of seven salt-related ERF genes of Populus
Source: PeerJ. 2020 Oct 20;8:e10206. doi: 10.7717/peerj.10206 (PMC7583627; doi:10.7717/peerj.10206)
Supplement: Supplemental Information 17 [file peerj-08-10206-s017.gz › Potri.004G051700.1_plantcare.html]

Content-Type: text/html; charset=ISO-8859-1


PlantCARE


Webmaster Firefox specific output  
To save the result:
click on the frame with the right mouse button and save the source code as a text file with extension .html  
REFERENCE:PlantCARE: a database of plant cis-acting regulatory elements and a portal to tools for in silico analysis of promoter sequences.  
Lescot, M., Déhais, P., Moreau, Y., De Moor, B., Rouzé ,P.,and Rombauts, S.  
Nucleic Acids Res., Database issue(2002), 30(1):325-327.   


---

>Potri.004G051700.1   
+ TTATCAAACT TTCATGATGT GAATCTCAGG TTTGACGGGT TAACTTGGTT TGAAGGGTTA ACCCAGTTAA   
  
  
+ TTCAAATTTT TTTTTTCTTC ATTAGTTTTT TTTTCTTCCT GTTGATTTTT TTCATTGAAC TTATATATTT   
  
  
+ TAATTATAAT AATATAATAA TAATTACATA TACTAAAATA CTGAGTTGTC TTCTTTTTTT TTTACGTTTT   
  
  
+ TAAATTATTA TTTTTTTGTT TTTGTTTTTT TCTTTGTGTT TTTTTTATTT TAATGAATTT TTTTTGTTTA   
  
  
+ ATTTAGTTTG TTAATGTTTA ATTTTTTTTA TTTAGTTATC ATATTTTCAT AATACGGATC CCGAGTTTGA   
  
  
+ TGAGTTAATC CAGAATTTTT GCTTTTCTTC TTTTTAATTA ATTTTTTTTT GTTTAGTTTA GTTTGTTAAT   
  
  
+ GTTAAATTTT TTTTATTTAA TTATCAGACT TTTATGACAC GTATCTCGGG TTTGACGGGT TAACGCGTCA   
  
  
+ ATTTTTTTTT CTATTTAGTT ATCAAATTTT TATGACGCGA ATTTCAGGTT TGACAGGTTA ACCTGGTTTG   
  
  
+ AAGGGTTAAC CCATTTAATT CAATTTTTTT TCTTTTTCTT CATTAGTTTT TTTCTTCCAT TTAATTTTTT   
  
  
+ TTCTTTTTCT TCATTAATGT TATTATTAAT ATTATAAATA TCACTCTTGG ATCAGGCGTT GCAGCTAAAT   
  
  
+ CTAAAACTCA TAGGTATAGC TTTATAGAAA TTTGAATCTT AGTTTTTTTT TATATTTTTT ATACGAAAAA   
  
  
+ ATTAACCCGT GCAGGTAATG TGACTAGTAA TAACATTATT TTCTGTTGAA CCGAACAAGA AAGAATCGTA   
  
  
+ GTTAGGTGTC AGAGACTCAG AGTCGCTGCT TCTCACATTC AAACCTTGTA AGCGTTTGGT CATTGTAAGA   
  
  
+ CCCTCTCTTT CTTTTATCAA ATAACCTTTC TTTATTTCTT AAATAAATTA AGGACCCTTC TTTTTTTATA   
  
  
+ AAAAATAAAA ATAAAAAATT CTTCCTTCTT CCCATAATTG ATTCAATCGC AAATGAGTCA TGTCATGACC   
  
  
+ CGGAGACCCT TTGAAAAGTC AAATTAGATA AAGATTGAGA CGATGGTTTC AAGAGACACA ACCACTCAAG   
  
  
+ TATTTACCAT TGTGATGAGC TGACAAGGAT TGTCATCCAG AATGGTCTCT CCTCACGATG TCACCAGCCA   
  
  
+ GTGGAATCCA AATTAAATTG AATTTATTGG GAAATGGGAT AACTCTTCCT TTTATATATT AAAAATTAAA   
  
  
+ AAATATATAC AGTGCCAATT CTACTGTACT GGTTAATTAC CCTATCAACA TTTAAGTAGT TTATGTAACG   
  
  
+ TTGTATTAGT TGTTTTTTAA AATAATTTTT TTTTAATATA TTAAAATAAT TTTTTTAAAA AATTTATTTT   
  
  
+ TGATATTAAT ATGTTAAAAC AATTTAAAAA CATTGAAAAA AAATAAAATA AAATAAATTC AAGTTTTGCA   
  
  
+ CAAAAAAACA CTATTGCACC GCGTACATAA ATGATGTCTT AGTGTATTTA GCATTGTGAT AGCTTTTGTG   
  
  
+ ATCATTCTGG TTTTAAAAAA ACAAATTTAA AAGAAATATT TTTAGTTGTG GTTTTAAAAA GTAGATTTTA   
  
  
+ AAAAATACAT GTTTGATTAA ATCTACTATG AGATAAATTT TTACATGTAA AATAAATAAA AACATATTTC   
  
  
+ CATCAATTAA AAAAAAACAT GTTCTTTCAG TTTCTACATG ACTAATATTT AAAATACAAT TACTTATAAA   
  
  
+ GTCCGGTACG AAAGCACCCT CTAAATGGAG AATGAACTGT ATGTATTCTT CCCATGGACC TGTGATTTTT   
  
  
+ CCGTAGGAAA GGCAATTTCC ATTCAGAATA CAAGAAGATA CATGTCTTTT GAAACTCTTA TTAGCTGTCT   
  
  
+ ACCTATCAAC CAAGAATGAC GCTTCTCATC GTATGTCACT ATAAAATTTG GTTGATGACA CCAACACTTT   
  
  
+ CCCATTGGTA TTCATGGCTT TTTAGCTGCA CGATCCAAC  

- AATAGTTTGA AAGTACTACA CTTAGAGTCC AAACTGCCCA ATTGAACCAA ACTTCCCAAT TGGGTCAATT   
  
  
- AAGTTTAAAA AAAAAAGAAG TAATCAAAAA AAAAGAAGGA CAACTAAAAA AAGTAACTTG AATATATAAA   
  
  
- ATTAATATTA TTATATTATT ATTAATGTAT ATGATTTTAT GACTCAACAG AAGAAAAAAA AAATGCAAAA   
  
  
- ATTTAATAAT AAAAAAACAA AAACAAAAAA AGAAACACAA AAAAAATAAA ATTACTTAAA AAAAACAAAT   
  
  
- TAAATCAAAC AATTACAAAT TAAAAAAAAT AAATCAATAG TATAAAAGTA TTATGCCTAG GGCTCAAACT   
  
  
- ACTCAATTAG GTCTTAAAAA CGAAAAGAAG AAAAATTAAT TAAAAAAAAA CAAATCAAAT CAAACAATTA   
  
  
- CAATTTAAAA AAAATAAATT AATAGTCTGA AAATACTGTG CATAGAGCCC AAACTGCCCA ATTGCGCAGT   
  
  
- TAAAAAAAAA GATAAATCAA TAGTTTAAAA ATACTGCGCT TAAAGTCCAA ACTGTCCAAT TGGACCAAAC   
  
  
- TTCCCAATTG GGTAAATTAA GTTAAAAAAA AGAAAAAGAA GTAATCAAAA AAAGAAGGTA AATTAAAAAA   
  
  
- AAGAAAAAGA AGTAATTACA ATAATAATTA TAATATTTAT AGTGAGAACC TAGTCCGCAA CGTCGATTTA   
  
  
- GATTTTGAGT ATCCATATCG AAATATCTTT AAACTTAGAA TCAAAAAAAA ATATAAAAAA TATGCTTTTT   
  
  
- TAATTGGGCA CGTCCATTAC ACTGATCATT ATTGTAATAA AAGACAACTT GGCTTGTTCT TTCTTAGCAT   
  
  
- CAATCCACAG TCTCTGAGTC TCAGCGACGA AGAGTGTAAG TTTGGAACAT TCGCAAACCA GTAACATTCT   
  
  
- GGGAGAGAAA GAAAATAGTT TATTGGAAAG AAATAAAGAA TTTATTTAAT TCCTGGGAAG AAAAAAATAT   
  
  
- TTTTTATTTT TATTTTTTAA GAAGGAAGAA GGGTATTAAC TAAGTTAGCG TTTACTCAGT ACAGTACTGG   
  
  
- GCCTCTGGGA AACTTTTCAG TTTAATCTAT TTCTAACTCT GCTACCAAAG TTCTCTGTGT TGGTGAGTTC   
  
  
- ATAAATGGTA ACACTACTCG ACTGTTCCTA ACAGTAGGTC TTACCAGAGA GGAGTGCTAC AGTGGTCGGT   
  
  
- CACCTTAGGT TTAATTTAAC TTAAATAACC CTTTACCCTA TTGAGAAGGA AAATATATAA TTTTTAATTT   
  
  
- TTTATATATG TCACGGTTAA GATGACATGA CCAATTAATG GGATAGTTGT AAATTCATCA AATACATTGC   
  
  
- AACATAATCA ACAAAAAATT TTATTAAAAA AAAATTATAT AATTTTATTA AAAAAATTTT TTAAATAAAA   
  
  
- ACTATAATTA TACAATTTTG TTAAATTTTT GTAACTTTTT TTTATTTTAT TTTATTTAAG TTCAAAACGT   
  
  
- GTTTTTTTGT GATAACGTGG CGCATGTATT TACTACAGAA TCACATAAAT CGTAACACTA TCGAAAACAC   
  
  
- TAGTAAGACC AAAATTTTTT TGTTTAAATT TTCTTTATAA AAATCAACAC CAAAATTTTT CATCTAAAAT   
  
  
- TTTTTATGTA CAAACTAATT TAGATGATAC TCTATTTAAA AATGTACATT TTATTTATTT TTGTATAAAG   
  
  
- GTAGTTAATT TTTTTTTGTA CAAGAAAGTC AAAGATGTAC TGATTATAAA TTTTATGTTA ATGAATATTT   
  
  
- CAGGCCATGC TTTCGTGGGA GATTTACCTC TTACTTGACA TACATAAGAA GGGTACCTGG ACACTAAAAA   
  
  
- GGCATCCTTT CCGTTAAAGG TAAGTCTTAT GTTCTTCTAT GTACAGAAAA CTTTGAGAAT AATCGACAGA   
  
  
- TGGATAGTTG GTTCTTACTG CGAAGAGTAG CATACAGTGA TATTTTAAAC CAACTACTGT GGTTGTGAAA   
  
  
- GGGTAACCAT AAGTACCGAA AAATCGACGT GCTAGGTTG

  
  
Motifs Found  

+   

| Site Name | Organism | Position | Strand | Matrix score. | sequence | function |
| --- | --- | --- | --- | --- | --- | --- |
|  | organism | 1233 | + | 4 | motif\_sequence | short\_function |
|  | organism | 1601 | - | 4 | motif\_sequence | short\_function |
|  | organism | 1631 | + | 4 | motif\_sequence | short\_function |
|  | organism | 1167 | + | 4 | motif\_sequence | short\_function |
|  | organism | 1493 | - | 4 | motif\_sequence | short\_function |
|  | organism | 1284 | + | 4 | motif\_sequence | short\_function |
|  | organism | 1699 | + | 4 | motif\_sequence | short\_function |
|  | organism | 1854 | - | 4 | motif\_sequence | short\_function |

>Potri.004G051700.1   
+ TTATCAAACT TTCATGATGT GAATCTCAGG TTTGACGGGT TAACTTGGTT TGAAGGGTTA ACCCAGTTAA   
  
  
+ TTCAAATTTT TTTTTTCTTC ATTAGTTTTT TTTTCTTCCT GTTGATTTTT TTCATTGAAC TTATATATTT   
  
  
+ TAATTATAAT AATATAATAA TAATTACATA TACTAAAATA CTGAGTTGTC TTCTTTTTTT TTTACGTTTT   
  
  
+ TAAATTATTA TTTTTTTGTT TTTGTTTTTT TCTTTGTGTT TTTTTTATTT TAATGAATTT TTTTTGTTTA   
  
  
+ ATTTAGTTTG TTAATGTTTA ATTTTTTTTA TTTAGTTATC ATATTTTCAT AATACGGATC CCGAGTTTGA   
  
  
+ TGAGTTAATC CAGAATTTTT GCTTTTCTTC TTTTTAATTA ATTTTTTTTT GTTTAGTTTA GTTTGTTAAT   
  
  
+ GTTAAATTTT TTTTATTTAA TTATCAGACT TTTATGACAC GTATCTCGGG TTTGACGGGT TAACGCGTCA   
  
  
+ ATTTTTTTTT CTATTTAGTT ATCAAATTTT TATGACGCGA ATTTCAGGTT TGACAGGTTA ACCTGGTTTG   
  
  
+ AAGGGTTAAC CCATTTAATT CAATTTTTTT TCTTTTTCTT CATTAGTTTT TTTCTTCCAT TTAATTTTTT   
  
  
+ TTCTTTTTCT TCATTAATGT TATTATTAAT ATTATAAATA TCACTCTTGG ATCAGGCGTT GCAGCTAAAT   
  
  
+ CTAAAACTCA TAGGTATAGC TTTATAGAAA TTTGAATCTT AGTTTTTTTT TATATTTTTT ATACGAAAAA   
  
  
+ ATTAACCCGT GCAGGTAATG TGACTAGTAA TAACATTATT TTCTGTTGAA CCGAACAAGA AAGAATCGTA   
  
  
+ GTTAGGTGTC AGAGACTCAG AGTCGCTGCT TCTCACATTC AAACCTTGTA AGCGTTTGGT CATTGTAAGA   
  
  
+ CCCTCTCTTT CTTTTATCAA ATAACCTTTC TTTATTTCTT AAATAAATTA AGGACCCTTC TTTTTTTATA   
  
  
+ AAAAATAAAA ATAAAAAATT CTTCCTTCTT CCCATAATTG ATTCAATCGC AAATGAGTCA TGTCATGACC   
  
  
+ CGGAGACCCT TTGAAAAGTC AAATTAGATA AAGATTGAGA CGATGGTTTC AAGAGACACA ACCACTCAAG   
  
  
+ TATTTACCAT TGTGATGAGC TGACAAGGAT TGTCATCCAG AATGGTCTCT CCTCACGATG TCACCAGCCA   
  
  
+ GTGGAATCCA AATTAAATTG AATTTATTGG GAAATGGGAT AACTCTTCCT TTTATATATT AAAAATTAAA   
  
  
+ AAATATATAC AGTGCCAATT CTACTGTACT GGTTAATTAC CCTATCAACA TTTAAGTAGT TTATGTAACG   
  
  
+ TTGTATTAGT TGTTTTTTAA AATAATTTTT TTTTAATATA TTAAAATAAT TTTTTTAAAA AATTTATTTT   
  
  
+ TGATATTAAT ATGTTAAAAC AATTTAAAAA CATTGAAAAA AAATAAAATA AAATAAATTC AAGTTTTGCA   
  
  
+ CAAAAAAACA CTATTGCACC GCGTACATAA ATGATGTCTT AGTGTATTTA GCATTGTGAT AGCTTTTGTG   
  
  
+ ATCATTCTGG TTTTAAAAAA ACAAATTTAA AAGAAATATT TTTAGTTGTG GTTTTAAAAA GTAGATTTTA   
  
  
+ AAAAATACAT GTTTGATTAA ATCTACTATG AGATAAATTT TTACATGTAA AATAAATAAA AACATATTTC   
  
  
+ CATCAATTAA AAAAAAACAT GTTCTTTCAG TTTCTACATG ACTAATATTT AAAATACAAT TACTTATAAA   
  
  
+ GTCCGGTACG AAAGCACCCT CTAAATGGAG AATGAACTGT ATGTATTCTT CCCATGGACC TGTGATTTTT   
  
  
+ CCGTAGGAAA GGCAATTTCC ATTCAGAATA CAAGAAGATA CATGTCTTTT GAAACTCTTA TTAGCTGTCT   
  
  
+ ACCTATCAAC CAAGAATGAC GCTTCTCATC GTATGTCACT ATAAAATTTG GTTGATGACA CCAACACTTT   
  
  
+ CCCATTGGTA TTCATGGCTT TTTAGCTGCA CGATCCAAC  

- AATAGTTTGA AAGTACTACA CTTAGAGTCC AAACTGCCCA ATTGAACCAA ACTTCCCAAT TGGGTCAATT   
  
  
- AAGTTTAAAA AAAAAAGAAG TAATCAAAAA AAAAGAAGGA CAACTAAAAA AAGTAACTTG AATATATAAA   
  
  
- ATTAATATTA TTATATTATT ATTAATGTAT ATGATTTTAT GACTCAACAG AAGAAAAAAA AAATGCAAAA   
  
  
- ATTTAATAAT AAAAAAACAA AAACAAAAAA AGAAACACAA AAAAAATAAA ATTACTTAAA AAAAACAAAT   
  
  
- TAAATCAAAC AATTACAAAT TAAAAAAAAT AAATCAATAG TATAAAAGTA TTATGCCTAG GGCTCAAACT   
  
  
- ACTCAATTAG GTCTTAAAAA CGAAAAGAAG AAAAATTAAT TAAAAAAAAA CAAATCAAAT CAAACAATTA   
  
  
- CAATTTAAAA AAAATAAATT AATAGTCTGA AAATACTGTG CATAGAGCCC AAACTGCCCA ATTGCGCAGT   
  
  
- TAAAAAAAAA GATAAATCAA TAGTTTAAAA ATACTGCGCT TAAAGTCCAA ACTGTCCAAT TGGACCAAAC   
  
  
- TTCCCAATTG GGTAAATTAA GTTAAAAAAA AGAAAAAGAA GTAATCAAAA AAAGAAGGTA AATTAAAAAA   
  
  
- AAGAAAAAGA AGTAATTACA ATAATAATTA TAATATTTAT AGTGAGAACC TAGTCCGCAA CGTCGATTTA   
  
  
- GATTTTGAGT ATCCATATCG AAATATCTTT AAACTTAGAA TCAAAAAAAA ATATAAAAAA TATGCTTTTT   
  
  
- TAATTGGGCA CGTCCATTAC ACTGATCATT ATTGTAATAA AAGACAACTT GGCTTGTTCT TTCTTAGCAT   
  
  
- CAATCCACAG TCTCTGAGTC TCAGCGACGA AGAGTGTAAG TTTGGAACAT TCGCAAACCA GTAACATTCT   
  
  
- GGGAGAGAAA GAAAATAGTT TATTGGAAAG AAATAAAGAA TTTATTTAAT TCCTGGGAAG AAAAAAATAT   
  
  
- TTTTTATTTT TATTTTTTAA GAAGGAAGAA GGGTATTAAC TAAGTTAGCG TTTACTCAGT ACAGTACTGG   
  
  
- GCCTCTGGGA AACTTTTCAG TTTAATCTAT TTCTAACTCT GCTACCAAAG TTCTCTGTGT TGGTGAGTTC   
  
  
- ATAAATGGTA ACACTACTCG ACTGTTCCTA ACAGTAGGTC TTACCAGAGA GGAGTGCTAC AGTGGTCGGT   
  
  
- CACCTTAGGT TTAATTTAAC TTAAATAACC CTTTACCCTA TTGAGAAGGA AAATATATAA TTTTTAATTT   
  
  
- TTTATATATG TCACGGTTAA GATGACATGA CCAATTAATG GGATAGTTGT AAATTCATCA AATACATTGC   
  
  
- AACATAATCA ACAAAAAATT TTATTAAAAA AAAATTATAT AATTTTATTA AAAAAATTTT TTAAATAAAA   
  
  
- ACTATAATTA TACAATTTTG TTAAATTTTT GTAACTTTTT TTTATTTTAT TTTATTTAAG TTCAAAACGT   
  
  
- GTTTTTTTGT GATAACGTGG CGCATGTATT TACTACAGAA TCACATAAAT CGTAACACTA TCGAAAACAC   
  
  
- TAGTAAGACC AAAATTTTTT TGTTTAAATT TTCTTTATAA AAATCAACAC CAAAATTTTT CATCTAAAAT   
  
  
- TTTTTATGTA CAAACTAATT TAGATGATAC TCTATTTAAA AATGTACATT TTATTTATTT TTGTATAAAG   
  
  
- GTAGTTAATT TTTTTTTGTA CAAGAAAGTC AAAGATGTAC TGATTATAAA TTTTATGTTA ATGAATATTT   
  
  
- CAGGCCATGC TTTCGTGGGA GATTTACCTC TTACTTGACA TACATAAGAA GGGTACCTGG ACACTAAAAA   
  
  
- GGCATCCTTT CCGTTAAAGG TAAGTCTTAT GTTCTTCTAT GTACAGAAAA CTTTGAGAAT AATCGACAGA   
  
  
- TGGATAGTTG GTTCTTACTG CGAAGAGTAG CATACAGTGA TATTTTAAAC CAACTACTGT GGTTGTGAAA   
  
  
- GGGTAACCAT AAGTACCGAA AAATCGACGT GCTAGGTTG

+     AAGAA-motif

| Site Name | Organism | Position | Strand | Matrix score. | sequence | function |
| --- | --- | --- | --- | --- | --- | --- |
| AAGAA-motif | Avena sativa | 1702 | - | 7 | GAAAGAA |  |
| AAGAA-motif | Avena sativa | 829 | + | 7 | GAAAGAA |  |

>Potri.004G051700.1   
+ TTATCAAACT TTCATGATGT GAATCTCAGG TTTGACGGGT TAACTTGGTT TGAAGGGTTA ACCCAGTTAA   
  
  
+ TTCAAATTTT TTTTTTCTTC ATTAGTTTTT TTTTCTTCCT GTTGATTTTT TTCATTGAAC TTATATATTT   
  
  
+ TAATTATAAT AATATAATAA TAATTACATA TACTAAAATA CTGAGTTGTC TTCTTTTTTT TTTACGTTTT   
  
  
+ TAAATTATTA TTTTTTTGTT TTTGTTTTTT TCTTTGTGTT TTTTTTATTT TAATGAATTT TTTTTGTTTA   
  
  
+ ATTTAGTTTG TTAATGTTTA ATTTTTTTTA TTTAGTTATC ATATTTTCAT AATACGGATC CCGAGTTTGA   
  
  
+ TGAGTTAATC CAGAATTTTT GCTTTTCTTC TTTTTAATTA ATTTTTTTTT GTTTAGTTTA GTTTGTTAAT   
  
  
+ GTTAAATTTT TTTTATTTAA TTATCAGACT TTTATGACAC GTATCTCGGG TTTGACGGGT TAACGCGTCA   
  
  
+ ATTTTTTTTT CTATTTAGTT ATCAAATTTT TATGACGCGA ATTTCAGGTT TGACAGGTTA ACCTGGTTTG   
  
  
+ AAGGGTTAAC CCATTTAATT CAATTTTTTT TCTTTTTCTT CATTAGTTTT TTTCTTCCAT TTAATTTTTT   
  
  
+ TTCTTTTTCT TCATTAATGT TATTATTAAT ATTATAAATA TCACTCTTGG ATCAGGCGTT GCAGCTAAAT   
  
  
+ CTAAAACTCA TAGGTATAGC TTTATAGAAA TTTGAATCTT AGTTTTTTTT TATATTTTTT ATACGAAAAA   
  
  
+ ATTAACCCGT GCAGGTAATG TGACTAGTAA TAACATTATT TTCTGTTGAA CCGAACAAGA AAGAATCGTA   
  
  
+ GTTAGGTGTC AGAGACTCAG AGTCGCTGCT TCTCACATTC AAACCTTGTA AGCGTTTGGT CATTGTAAGA   
  
  
+ CCCTCTCTTT CTTTTATCAA ATAACCTTTC TTTATTTCTT AAATAAATTA AGGACCCTTC TTTTTTTATA   
  
  
+ AAAAATAAAA ATAAAAAATT CTTCCTTCTT CCCATAATTG ATTCAATCGC AAATGAGTCA TGTCATGACC   
  
  
+ CGGAGACCCT TTGAAAAGTC AAATTAGATA AAGATTGAGA CGATGGTTTC AAGAGACACA ACCACTCAAG   
  
  
+ TATTTACCAT TGTGATGAGC TGACAAGGAT TGTCATCCAG AATGGTCTCT CCTCACGATG TCACCAGCCA   
  
  
+ GTGGAATCCA AATTAAATTG AATTTATTGG GAAATGGGAT AACTCTTCCT TTTATATATT AAAAATTAAA   
  
  
+ AAATATATAC AGTGCCAATT CTACTGTACT GGTTAATTAC CCTATCAACA TTTAAGTAGT TTATGTAACG   
  
  
+ TTGTATTAGT TGTTTTTTAA AATAATTTTT TTTTAATATA TTAAAATAAT TTTTTTAAAA AATTTATTTT   
  
  
+ TGATATTAAT ATGTTAAAAC AATTTAAAAA CATTGAAAAA AAATAAAATA AAATAAATTC AAGTTTTGCA   
  
  
+ CAAAAAAACA CTATTGCACC GCGTACATAA ATGATGTCTT AGTGTATTTA GCATTGTGAT AGCTTTTGTG   
  
  
+ ATCATTCTGG TTTTAAAAAA ACAAATTTAA AAGAAATATT TTTAGTTGTG GTTTTAAAAA GTAGATTTTA   
  
  
+ AAAAATACAT GTTTGATTAA ATCTACTATG AGATAAATTT TTACATGTAA AATAAATAAA AACATATTTC   
  
  
+ CATCAATTAA AAAAAAACAT GTTCTTTCAG TTTCTACATG ACTAATATTT AAAATACAAT TACTTATAAA   
  
  
+ GTCCGGTACG AAAGCACCCT CTAAATGGAG AATGAACTGT ATGTATTCTT CCCATGGACC TGTGATTTTT   
  
  
+ CCGTAGGAAA GGCAATTTCC ATTCAGAATA CAAGAAGATA CATGTCTTTT GAAACTCTTA TTAGCTGTCT   
  
  
+ ACCTATCAAC CAAGAATGAC GCTTCTCATC GTATGTCACT ATAAAATTTG GTTGATGACA CCAACACTTT   
  
  
+ CCCATTGGTA TTCATGGCTT TTTAGCTGCA CGATCCAAC  

- AATAGTTTGA AAGTACTACA CTTAGAGTCC AAACTGCCCA ATTGAACCAA ACTTCCCAAT TGGGTCAATT   
  
  
- AAGTTTAAAA AAAAAAGAAG TAATCAAAAA AAAAGAAGGA CAACTAAAAA AAGTAACTTG AATATATAAA   
  
  
- ATTAATATTA TTATATTATT ATTAATGTAT ATGATTTTAT GACTCAACAG AAGAAAAAAA AAATGCAAAA   
  
  
- ATTTAATAAT AAAAAAACAA AAACAAAAAA AGAAACACAA AAAAAATAAA ATTACTTAAA AAAAACAAAT   
  
  
- TAAATCAAAC AATTACAAAT TAAAAAAAAT AAATCAATAG TATAAAAGTA TTATGCCTAG GGCTCAAACT   
  
  
- ACTCAATTAG GTCTTAAAAA CGAAAAGAAG AAAAATTAAT TAAAAAAAAA CAAATCAAAT CAAACAATTA   
  
  
- CAATTTAAAA AAAATAAATT AATAGTCTGA AAATACTGTG CATAGAGCCC AAACTGCCCA ATTGCGCAGT   
  
  
- TAAAAAAAAA GATAAATCAA TAGTTTAAAA ATACTGCGCT TAAAGTCCAA ACTGTCCAAT TGGACCAAAC   
  
  
- TTCCCAATTG GGTAAATTAA GTTAAAAAAA AGAAAAAGAA GTAATCAAAA AAAGAAGGTA AATTAAAAAA   
  
  
- AAGAAAAAGA AGTAATTACA ATAATAATTA TAATATTTAT AGTGAGAACC TAGTCCGCAA CGTCGATTTA   
  
  
- GATTTTGAGT ATCCATATCG AAATATCTTT AAACTTAGAA TCAAAAAAAA ATATAAAAAA TATGCTTTTT   
  
  
- TAATTGGGCA CGTCCATTAC ACTGATCATT ATTGTAATAA AAGACAACTT GGCTTGTTCT TTCTTAGCAT   
  
  
- CAATCCACAG TCTCTGAGTC TCAGCGACGA AGAGTGTAAG TTTGGAACAT TCGCAAACCA GTAACATTCT   
  
  
- GGGAGAGAAA GAAAATAGTT TATTGGAAAG AAATAAAGAA TTTATTTAAT TCCTGGGAAG AAAAAAATAT   
  
  
- TTTTTATTTT TATTTTTTAA GAAGGAAGAA GGGTATTAAC TAAGTTAGCG TTTACTCAGT ACAGTACTGG   
  
  
- GCCTCTGGGA AACTTTTCAG TTTAATCTAT TTCTAACTCT GCTACCAAAG TTCTCTGTGT TGGTGAGTTC   
  
  
- ATAAATGGTA ACACTACTCG ACTGTTCCTA ACAGTAGGTC TTACCAGAGA GGAGTGCTAC AGTGGTCGGT   
  
  
- CACCTTAGGT TTAATTTAAC TTAAATAACC CTTTACCCTA TTGAGAAGGA AAATATATAA TTTTTAATTT   
  
  
- TTTATATATG TCACGGTTAA GATGACATGA CCAATTAATG GGATAGTTGT AAATTCATCA AATACATTGC   
  
  
- AACATAATCA ACAAAAAATT TTATTAAAAA AAAATTATAT AATTTTATTA AAAAAATTTT TTAAATAAAA   
  
  
- ACTATAATTA TACAATTTTG TTAAATTTTT GTAACTTTTT TTTATTTTAT TTTATTTAAG TTCAAAACGT   
  
  
- GTTTTTTTGT GATAACGTGG CGCATGTATT TACTACAGAA TCACATAAAT CGTAACACTA TCGAAAACAC   
  
  
- TAGTAAGACC AAAATTTTTT TGTTTAAATT TTCTTTATAA AAATCAACAC CAAAATTTTT CATCTAAAAT   
  
  
- TTTTTATGTA CAAACTAATT TAGATGATAC TCTATTTAAA AATGTACATT TTATTTATTT TTGTATAAAG   
  
  
- GTAGTTAATT TTTTTTTGTA CAAGAAAGTC AAAGATGTAC TGATTATAAA TTTTATGTTA ATGAATATTT   
  
  
- CAGGCCATGC TTTCGTGGGA GATTTACCTC TTACTTGACA TACATAAGAA GGGTACCTGG ACACTAAAAA   
  
  
- GGCATCCTTT CCGTTAAAGG TAAGTCTTAT GTTCTTCTAT GTACAGAAAA CTTTGAGAAT AATCGACAGA   
  
  
- TGGATAGTTG GTTCTTACTG CGAAGAGTAG CATACAGTGA TATTTTAAAC CAACTACTGT GGTTGTGAAA   
  
  
- GGGTAACCAT AAGTACCGAA AAATCGACGT GCTAGGTTG

+     ABRE

| Site Name | Organism | Position | Strand | Matrix score. | sequence | function |
| --- | --- | --- | --- | --- | --- | --- |
| ABRE | Arabidopsis thaliana | 458 | - | 5 | ACGTG | cis-acting element involved in the abscisic acid responsiveness |
| ABRE | Oryza sativa | 456 | - | 8 | TACGTGTC | cis-acting element involved in the abscisic acid responsiveness |

>Potri.004G051700.1   
+ TTATCAAACT TTCATGATGT GAATCTCAGG TTTGACGGGT TAACTTGGTT TGAAGGGTTA ACCCAGTTAA   
  
  
+ TTCAAATTTT TTTTTTCTTC ATTAGTTTTT TTTTCTTCCT GTTGATTTTT TTCATTGAAC TTATATATTT   
  
  
+ TAATTATAAT AATATAATAA TAATTACATA TACTAAAATA CTGAGTTGTC TTCTTTTTTT TTTACGTTTT   
  
  
+ TAAATTATTA TTTTTTTGTT TTTGTTTTTT TCTTTGTGTT TTTTTTATTT TAATGAATTT TTTTTGTTTA   
  
  
+ ATTTAGTTTG TTAATGTTTA ATTTTTTTTA TTTAGTTATC ATATTTTCAT AATACGGATC CCGAGTTTGA   
  
  
+ TGAGTTAATC CAGAATTTTT GCTTTTCTTC TTTTTAATTA ATTTTTTTTT GTTTAGTTTA GTTTGTTAAT   
  
  
+ GTTAAATTTT TTTTATTTAA TTATCAGACT TTTATGACAC GTATCTCGGG TTTGACGGGT TAACGCGTCA   
  
  
+ ATTTTTTTTT CTATTTAGTT ATCAAATTTT TATGACGCGA ATTTCAGGTT TGACAGGTTA ACCTGGTTTG   
  
  
+ AAGGGTTAAC CCATTTAATT CAATTTTTTT TCTTTTTCTT CATTAGTTTT TTTCTTCCAT TTAATTTTTT   
  
  
+ TTCTTTTTCT TCATTAATGT TATTATTAAT ATTATAAATA TCACTCTTGG ATCAGGCGTT GCAGCTAAAT   
  
  
+ CTAAAACTCA TAGGTATAGC TTTATAGAAA TTTGAATCTT AGTTTTTTTT TATATTTTTT ATACGAAAAA   
  
  
+ ATTAACCCGT GCAGGTAATG TGACTAGTAA TAACATTATT TTCTGTTGAA CCGAACAAGA AAGAATCGTA   
  
  
+ GTTAGGTGTC AGAGACTCAG AGTCGCTGCT TCTCACATTC AAACCTTGTA AGCGTTTGGT CATTGTAAGA   
  
  
+ CCCTCTCTTT CTTTTATCAA ATAACCTTTC TTTATTTCTT AAATAAATTA AGGACCCTTC TTTTTTTATA   
  
  
+ AAAAATAAAA ATAAAAAATT CTTCCTTCTT CCCATAATTG ATTCAATCGC AAATGAGTCA TGTCATGACC   
  
  
+ CGGAGACCCT TTGAAAAGTC AAATTAGATA AAGATTGAGA CGATGGTTTC AAGAGACACA ACCACTCAAG   
  
  
+ TATTTACCAT TGTGATGAGC TGACAAGGAT TGTCATCCAG AATGGTCTCT CCTCACGATG TCACCAGCCA   
  
  
+ GTGGAATCCA AATTAAATTG AATTTATTGG GAAATGGGAT AACTCTTCCT TTTATATATT AAAAATTAAA   
  
  
+ AAATATATAC AGTGCCAATT CTACTGTACT GGTTAATTAC CCTATCAACA TTTAAGTAGT TTATGTAACG   
  
  
+ TTGTATTAGT TGTTTTTTAA AATAATTTTT TTTTAATATA TTAAAATAAT TTTTTTAAAA AATTTATTTT   
  
  
+ TGATATTAAT ATGTTAAAAC AATTTAAAAA CATTGAAAAA AAATAAAATA AAATAAATTC AAGTTTTGCA   
  
  
+ CAAAAAAACA CTATTGCACC GCGTACATAA ATGATGTCTT AGTGTATTTA GCATTGTGAT AGCTTTTGTG   
  
  
+ ATCATTCTGG TTTTAAAAAA ACAAATTTAA AAGAAATATT TTTAGTTGTG GTTTTAAAAA GTAGATTTTA   
  
  
+ AAAAATACAT GTTTGATTAA ATCTACTATG AGATAAATTT TTACATGTAA AATAAATAAA AACATATTTC   
  
  
+ CATCAATTAA AAAAAAACAT GTTCTTTCAG TTTCTACATG ACTAATATTT AAAATACAAT TACTTATAAA   
  
  
+ GTCCGGTACG AAAGCACCCT CTAAATGGAG AATGAACTGT ATGTATTCTT CCCATGGACC TGTGATTTTT   
  
  
+ CCGTAGGAAA GGCAATTTCC ATTCAGAATA CAAGAAGATA CATGTCTTTT GAAACTCTTA TTAGCTGTCT   
  
  
+ ACCTATCAAC CAAGAATGAC GCTTCTCATC GTATGTCACT ATAAAATTTG GTTGATGACA CCAACACTTT   
  
  
+ CCCATTGGTA TTCATGGCTT TTTAGCTGCA CGATCCAAC  

- AATAGTTTGA AAGTACTACA CTTAGAGTCC AAACTGCCCA ATTGAACCAA ACTTCCCAAT TGGGTCAATT   
  
  
- AAGTTTAAAA AAAAAAGAAG TAATCAAAAA AAAAGAAGGA CAACTAAAAA AAGTAACTTG AATATATAAA   
  
  
- ATTAATATTA TTATATTATT ATTAATGTAT ATGATTTTAT GACTCAACAG AAGAAAAAAA AAATGCAAAA   
  
  
- ATTTAATAAT AAAAAAACAA AAACAAAAAA AGAAACACAA AAAAAATAAA ATTACTTAAA AAAAACAAAT   
  
  
- TAAATCAAAC AATTACAAAT TAAAAAAAAT AAATCAATAG TATAAAAGTA TTATGCCTAG GGCTCAAACT   
  
  
- ACTCAATTAG GTCTTAAAAA CGAAAAGAAG AAAAATTAAT TAAAAAAAAA CAAATCAAAT CAAACAATTA   
  
  
- CAATTTAAAA AAAATAAATT AATAGTCTGA AAATACTGTG CATAGAGCCC AAACTGCCCA ATTGCGCAGT   
  
  
- TAAAAAAAAA GATAAATCAA TAGTTTAAAA ATACTGCGCT TAAAGTCCAA ACTGTCCAAT TGGACCAAAC   
  
  
- TTCCCAATTG GGTAAATTAA GTTAAAAAAA AGAAAAAGAA GTAATCAAAA AAAGAAGGTA AATTAAAAAA   
  
  
- AAGAAAAAGA AGTAATTACA ATAATAATTA TAATATTTAT AGTGAGAACC TAGTCCGCAA CGTCGATTTA   
  
  
- GATTTTGAGT ATCCATATCG AAATATCTTT AAACTTAGAA TCAAAAAAAA ATATAAAAAA TATGCTTTTT   
  
  
- TAATTGGGCA CGTCCATTAC ACTGATCATT ATTGTAATAA AAGACAACTT GGCTTGTTCT TTCTTAGCAT   
  
  
- CAATCCACAG TCTCTGAGTC TCAGCGACGA AGAGTGTAAG TTTGGAACAT TCGCAAACCA GTAACATTCT   
  
  
- GGGAGAGAAA GAAAATAGTT TATTGGAAAG AAATAAAGAA TTTATTTAAT TCCTGGGAAG AAAAAAATAT   
  
  
- TTTTTATTTT TATTTTTTAA GAAGGAAGAA GGGTATTAAC TAAGTTAGCG TTTACTCAGT ACAGTACTGG   
  
  
- GCCTCTGGGA AACTTTTCAG TTTAATCTAT TTCTAACTCT GCTACCAAAG TTCTCTGTGT TGGTGAGTTC   
  
  
- ATAAATGGTA ACACTACTCG ACTGTTCCTA ACAGTAGGTC TTACCAGAGA GGAGTGCTAC AGTGGTCGGT   
  
  
- CACCTTAGGT TTAATTTAAC TTAAATAACC CTTTACCCTA TTGAGAAGGA AAATATATAA TTTTTAATTT   
  
  
- TTTATATATG TCACGGTTAA GATGACATGA CCAATTAATG GGATAGTTGT AAATTCATCA AATACATTGC   
  
  
- AACATAATCA ACAAAAAATT TTATTAAAAA AAAATTATAT AATTTTATTA AAAAAATTTT TTAAATAAAA   
  
  
- ACTATAATTA TACAATTTTG TTAAATTTTT GTAACTTTTT TTTATTTTAT TTTATTTAAG TTCAAAACGT   
  
  
- GTTTTTTTGT GATAACGTGG CGCATGTATT TACTACAGAA TCACATAAAT CGTAACACTA TCGAAAACAC   
  
  
- TAGTAAGACC AAAATTTTTT TGTTTAAATT TTCTTTATAA AAATCAACAC CAAAATTTTT CATCTAAAAT   
  
  
- TTTTTATGTA CAAACTAATT TAGATGATAC TCTATTTAAA AATGTACATT TTATTTATTT TTGTATAAAG   
  
  
- GTAGTTAATT TTTTTTTGTA CAAGAAAGTC AAAGATGTAC TGATTATAAA TTTTATGTTA ATGAATATTT   
  
  
- CAGGCCATGC TTTCGTGGGA GATTTACCTC TTACTTGACA TACATAAGAA GGGTACCTGG ACACTAAAAA   
  
  
- GGCATCCTTT CCGTTAAAGG TAAGTCTTAT GTTCTTCTAT GTACAGAAAA CTTTGAGAAT AATCGACAGA   
  
  
- TGGATAGTTG GTTCTTACTG CGAAGAGTAG CATACAGTGA TATTTTAAAC CAACTACTGT GGTTGTGAAA   
  
  
- GGGTAACCAT AAGTACCGAA AAATCGACGT GCTAGGTTG

+     ABRE3a

| Site Name | Organism | Position | Strand | Matrix score. | sequence | function |
| --- | --- | --- | --- | --- | --- | --- |
| ABRE3a | Zea mays | 458 | - | 6 | TACGTG |  |

>Potri.004G051700.1   
+ TTATCAAACT TTCATGATGT GAATCTCAGG TTTGACGGGT TAACTTGGTT TGAAGGGTTA ACCCAGTTAA   
  
  
+ TTCAAATTTT TTTTTTCTTC ATTAGTTTTT TTTTCTTCCT GTTGATTTTT TTCATTGAAC TTATATATTT   
  
  
+ TAATTATAAT AATATAATAA TAATTACATA TACTAAAATA CTGAGTTGTC TTCTTTTTTT TTTACGTTTT   
  
  
+ TAAATTATTA TTTTTTTGTT TTTGTTTTTT TCTTTGTGTT TTTTTTATTT TAATGAATTT TTTTTGTTTA   
  
  
+ ATTTAGTTTG TTAATGTTTA ATTTTTTTTA TTTAGTTATC ATATTTTCAT AATACGGATC CCGAGTTTGA   
  
  
+ TGAGTTAATC CAGAATTTTT GCTTTTCTTC TTTTTAATTA ATTTTTTTTT GTTTAGTTTA GTTTGTTAAT   
  
  
+ GTTAAATTTT TTTTATTTAA TTATCAGACT TTTATGACAC GTATCTCGGG TTTGACGGGT TAACGCGTCA   
  
  
+ ATTTTTTTTT CTATTTAGTT ATCAAATTTT TATGACGCGA ATTTCAGGTT TGACAGGTTA ACCTGGTTTG   
  
  
+ AAGGGTTAAC CCATTTAATT CAATTTTTTT TCTTTTTCTT CATTAGTTTT TTTCTTCCAT TTAATTTTTT   
  
  
+ TTCTTTTTCT TCATTAATGT TATTATTAAT ATTATAAATA TCACTCTTGG ATCAGGCGTT GCAGCTAAAT   
  
  
+ CTAAAACTCA TAGGTATAGC TTTATAGAAA TTTGAATCTT AGTTTTTTTT TATATTTTTT ATACGAAAAA   
  
  
+ ATTAACCCGT GCAGGTAATG TGACTAGTAA TAACATTATT TTCTGTTGAA CCGAACAAGA AAGAATCGTA   
  
  
+ GTTAGGTGTC AGAGACTCAG AGTCGCTGCT TCTCACATTC AAACCTTGTA AGCGTTTGGT CATTGTAAGA   
  
  
+ CCCTCTCTTT CTTTTATCAA ATAACCTTTC TTTATTTCTT AAATAAATTA AGGACCCTTC TTTTTTTATA   
  
  
+ AAAAATAAAA ATAAAAAATT CTTCCTTCTT CCCATAATTG ATTCAATCGC AAATGAGTCA TGTCATGACC   
  
  
+ CGGAGACCCT TTGAAAAGTC AAATTAGATA AAGATTGAGA CGATGGTTTC AAGAGACACA ACCACTCAAG   
  
  
+ TATTTACCAT TGTGATGAGC TGACAAGGAT TGTCATCCAG AATGGTCTCT CCTCACGATG TCACCAGCCA   
  
  
+ GTGGAATCCA AATTAAATTG AATTTATTGG GAAATGGGAT AACTCTTCCT TTTATATATT AAAAATTAAA   
  
  
+ AAATATATAC AGTGCCAATT CTACTGTACT GGTTAATTAC CCTATCAACA TTTAAGTAGT TTATGTAACG   
  
  
+ TTGTATTAGT TGTTTTTTAA AATAATTTTT TTTTAATATA TTAAAATAAT TTTTTTAAAA AATTTATTTT   
  
  
+ TGATATTAAT ATGTTAAAAC AATTTAAAAA CATTGAAAAA AAATAAAATA AAATAAATTC AAGTTTTGCA   
  
  
+ CAAAAAAACA CTATTGCACC GCGTACATAA ATGATGTCTT AGTGTATTTA GCATTGTGAT AGCTTTTGTG   
  
  
+ ATCATTCTGG TTTTAAAAAA ACAAATTTAA AAGAAATATT TTTAGTTGTG GTTTTAAAAA GTAGATTTTA   
  
  
+ AAAAATACAT GTTTGATTAA ATCTACTATG AGATAAATTT TTACATGTAA AATAAATAAA AACATATTTC   
  
  
+ CATCAATTAA AAAAAAACAT GTTCTTTCAG TTTCTACATG ACTAATATTT AAAATACAAT TACTTATAAA   
  
  
+ GTCCGGTACG AAAGCACCCT CTAAATGGAG AATGAACTGT ATGTATTCTT CCCATGGACC TGTGATTTTT   
  
  
+ CCGTAGGAAA GGCAATTTCC ATTCAGAATA CAAGAAGATA CATGTCTTTT GAAACTCTTA TTAGCTGTCT   
  
  
+ ACCTATCAAC CAAGAATGAC GCTTCTCATC GTATGTCACT ATAAAATTTG GTTGATGACA CCAACACTTT   
  
  
+ CCCATTGGTA TTCATGGCTT TTTAGCTGCA CGATCCAAC  

- AATAGTTTGA AAGTACTACA CTTAGAGTCC AAACTGCCCA ATTGAACCAA ACTTCCCAAT TGGGTCAATT   
  
  
- AAGTTTAAAA AAAAAAGAAG TAATCAAAAA AAAAGAAGGA CAACTAAAAA AAGTAACTTG AATATATAAA   
  
  
- ATTAATATTA TTATATTATT ATTAATGTAT ATGATTTTAT GACTCAACAG AAGAAAAAAA AAATGCAAAA   
  
  
- ATTTAATAAT AAAAAAACAA AAACAAAAAA AGAAACACAA AAAAAATAAA ATTACTTAAA AAAAACAAAT   
  
  
- TAAATCAAAC AATTACAAAT TAAAAAAAAT AAATCAATAG TATAAAAGTA TTATGCCTAG GGCTCAAACT   
  
  
- ACTCAATTAG GTCTTAAAAA CGAAAAGAAG AAAAATTAAT TAAAAAAAAA CAAATCAAAT CAAACAATTA   
  
  
- CAATTTAAAA AAAATAAATT AATAGTCTGA AAATACTGTG CATAGAGCCC AAACTGCCCA ATTGCGCAGT   
  
  
- TAAAAAAAAA GATAAATCAA TAGTTTAAAA ATACTGCGCT TAAAGTCCAA ACTGTCCAAT TGGACCAAAC   
  
  
- TTCCCAATTG GGTAAATTAA GTTAAAAAAA AGAAAAAGAA GTAATCAAAA AAAGAAGGTA AATTAAAAAA   
  
  
- AAGAAAAAGA AGTAATTACA ATAATAATTA TAATATTTAT AGTGAGAACC TAGTCCGCAA CGTCGATTTA   
  
  
- GATTTTGAGT ATCCATATCG AAATATCTTT AAACTTAGAA TCAAAAAAAA ATATAAAAAA TATGCTTTTT   
  
  
- TAATTGGGCA CGTCCATTAC ACTGATCATT ATTGTAATAA AAGACAACTT GGCTTGTTCT TTCTTAGCAT   
  
  
- CAATCCACAG TCTCTGAGTC TCAGCGACGA AGAGTGTAAG TTTGGAACAT TCGCAAACCA GTAACATTCT   
  
  
- GGGAGAGAAA GAAAATAGTT TATTGGAAAG AAATAAAGAA TTTATTTAAT TCCTGGGAAG AAAAAAATAT   
  
  
- TTTTTATTTT TATTTTTTAA GAAGGAAGAA GGGTATTAAC TAAGTTAGCG TTTACTCAGT ACAGTACTGG   
  
  
- GCCTCTGGGA AACTTTTCAG TTTAATCTAT TTCTAACTCT GCTACCAAAG TTCTCTGTGT TGGTGAGTTC   
  
  
- ATAAATGGTA ACACTACTCG ACTGTTCCTA ACAGTAGGTC TTACCAGAGA GGAGTGCTAC AGTGGTCGGT   
  
  
- CACCTTAGGT TTAATTTAAC TTAAATAACC CTTTACCCTA TTGAGAAGGA AAATATATAA TTTTTAATTT   
  
  
- TTTATATATG TCACGGTTAA GATGACATGA CCAATTAATG GGATAGTTGT AAATTCATCA AATACATTGC   
  
  
- AACATAATCA ACAAAAAATT TTATTAAAAA AAAATTATAT AATTTTATTA AAAAAATTTT TTAAATAAAA   
  
  
- ACTATAATTA TACAATTTTG TTAAATTTTT GTAACTTTTT TTTATTTTAT TTTATTTAAG TTCAAAACGT   
  
  
- GTTTTTTTGT GATAACGTGG CGCATGTATT TACTACAGAA TCACATAAAT CGTAACACTA TCGAAAACAC   
  
  
- TAGTAAGACC AAAATTTTTT TGTTTAAATT TTCTTTATAA AAATCAACAC CAAAATTTTT CATCTAAAAT   
  
  
- TTTTTATGTA CAAACTAATT TAGATGATAC TCTATTTAAA AATGTACATT TTATTTATTT TTGTATAAAG   
  
  
- GTAGTTAATT TTTTTTTGTA CAAGAAAGTC AAAGATGTAC TGATTATAAA TTTTATGTTA ATGAATATTT   
  
  
- CAGGCCATGC TTTCGTGGGA GATTTACCTC TTACTTGACA TACATAAGAA GGGTACCTGG ACACTAAAAA   
  
  
- GGCATCCTTT CCGTTAAAGG TAAGTCTTAT GTTCTTCTAT GTACAGAAAA CTTTGAGAAT AATCGACAGA   
  
  
- TGGATAGTTG GTTCTTACTG CGAAGAGTAG CATACAGTGA TATTTTAAAC CAACTACTGT GGTTGTGAAA   
  
  
- GGGTAACCAT AAGTACCGAA AAATCGACGT GCTAGGTTG

+     ABRE4

| Site Name | Organism | Position | Strand | Matrix score. | sequence | function |
| --- | --- | --- | --- | --- | --- | --- |
| ABRE4 | Zea mays | 458 | + | 6 | CACGTA |  |

>Potri.004G051700.1   
+ TTATCAAACT TTCATGATGT GAATCTCAGG TTTGACGGGT TAACTTGGTT TGAAGGGTTA ACCCAGTTAA   
  
  
+ TTCAAATTTT TTTTTTCTTC ATTAGTTTTT TTTTCTTCCT GTTGATTTTT TTCATTGAAC TTATATATTT   
  
  
+ TAATTATAAT AATATAATAA TAATTACATA TACTAAAATA CTGAGTTGTC TTCTTTTTTT TTTACGTTTT   
  
  
+ TAAATTATTA TTTTTTTGTT TTTGTTTTTT TCTTTGTGTT TTTTTTATTT TAATGAATTT TTTTTGTTTA   
  
  
+ ATTTAGTTTG TTAATGTTTA ATTTTTTTTA TTTAGTTATC ATATTTTCAT AATACGGATC CCGAGTTTGA   
  
  
+ TGAGTTAATC CAGAATTTTT GCTTTTCTTC TTTTTAATTA ATTTTTTTTT GTTTAGTTTA GTTTGTTAAT   
  
  
+ GTTAAATTTT TTTTATTTAA TTATCAGACT TTTATGACAC GTATCTCGGG TTTGACGGGT TAACGCGTCA   
  
  
+ ATTTTTTTTT CTATTTAGTT ATCAAATTTT TATGACGCGA ATTTCAGGTT TGACAGGTTA ACCTGGTTTG   
  
  
+ AAGGGTTAAC CCATTTAATT CAATTTTTTT TCTTTTTCTT CATTAGTTTT TTTCTTCCAT TTAATTTTTT   
  
  
+ TTCTTTTTCT TCATTAATGT TATTATTAAT ATTATAAATA TCACTCTTGG ATCAGGCGTT GCAGCTAAAT   
  
  
+ CTAAAACTCA TAGGTATAGC TTTATAGAAA TTTGAATCTT AGTTTTTTTT TATATTTTTT ATACGAAAAA   
  
  
+ ATTAACCCGT GCAGGTAATG TGACTAGTAA TAACATTATT TTCTGTTGAA CCGAACAAGA AAGAATCGTA   
  
  
+ GTTAGGTGTC AGAGACTCAG AGTCGCTGCT TCTCACATTC AAACCTTGTA AGCGTTTGGT CATTGTAAGA   
  
  
+ CCCTCTCTTT CTTTTATCAA ATAACCTTTC TTTATTTCTT AAATAAATTA AGGACCCTTC TTTTTTTATA   
  
  
+ AAAAATAAAA ATAAAAAATT CTTCCTTCTT CCCATAATTG ATTCAATCGC AAATGAGTCA TGTCATGACC   
  
  
+ CGGAGACCCT TTGAAAAGTC AAATTAGATA AAGATTGAGA CGATGGTTTC AAGAGACACA ACCACTCAAG   
  
  
+ TATTTACCAT TGTGATGAGC TGACAAGGAT TGTCATCCAG AATGGTCTCT CCTCACGATG TCACCAGCCA   
  
  
+ GTGGAATCCA AATTAAATTG AATTTATTGG GAAATGGGAT AACTCTTCCT TTTATATATT AAAAATTAAA   
  
  
+ AAATATATAC AGTGCCAATT CTACTGTACT GGTTAATTAC CCTATCAACA TTTAAGTAGT TTATGTAACG   
  
  
+ TTGTATTAGT TGTTTTTTAA AATAATTTTT TTTTAATATA TTAAAATAAT TTTTTTAAAA AATTTATTTT   
  
  
+ TGATATTAAT ATGTTAAAAC AATTTAAAAA CATTGAAAAA AAATAAAATA AAATAAATTC AAGTTTTGCA   
  
  
+ CAAAAAAACA CTATTGCACC GCGTACATAA ATGATGTCTT AGTGTATTTA GCATTGTGAT AGCTTTTGTG   
  
  
+ ATCATTCTGG TTTTAAAAAA ACAAATTTAA AAGAAATATT TTTAGTTGTG GTTTTAAAAA GTAGATTTTA   
  
  
+ AAAAATACAT GTTTGATTAA ATCTACTATG AGATAAATTT TTACATGTAA AATAAATAAA AACATATTTC   
  
  
+ CATCAATTAA AAAAAAACAT GTTCTTTCAG TTTCTACATG ACTAATATTT AAAATACAAT TACTTATAAA   
  
  
+ GTCCGGTACG AAAGCACCCT CTAAATGGAG AATGAACTGT ATGTATTCTT CCCATGGACC TGTGATTTTT   
  
  
+ CCGTAGGAAA GGCAATTTCC ATTCAGAATA CAAGAAGATA CATGTCTTTT GAAACTCTTA TTAGCTGTCT   
  
  
+ ACCTATCAAC CAAGAATGAC GCTTCTCATC GTATGTCACT ATAAAATTTG GTTGATGACA CCAACACTTT   
  
  
+ CCCATTGGTA TTCATGGCTT TTTAGCTGCA CGATCCAAC  

- AATAGTTTGA AAGTACTACA CTTAGAGTCC AAACTGCCCA ATTGAACCAA ACTTCCCAAT TGGGTCAATT   
  
  
- AAGTTTAAAA AAAAAAGAAG TAATCAAAAA AAAAGAAGGA CAACTAAAAA AAGTAACTTG AATATATAAA   
  
  
- ATTAATATTA TTATATTATT ATTAATGTAT ATGATTTTAT GACTCAACAG AAGAAAAAAA AAATGCAAAA   
  
  
- ATTTAATAAT AAAAAAACAA AAACAAAAAA AGAAACACAA AAAAAATAAA ATTACTTAAA AAAAACAAAT   
  
  
- TAAATCAAAC AATTACAAAT TAAAAAAAAT AAATCAATAG TATAAAAGTA TTATGCCTAG GGCTCAAACT   
  
  
- ACTCAATTAG GTCTTAAAAA CGAAAAGAAG AAAAATTAAT TAAAAAAAAA CAAATCAAAT CAAACAATTA   
  
  
- CAATTTAAAA AAAATAAATT AATAGTCTGA AAATACTGTG CATAGAGCCC AAACTGCCCA ATTGCGCAGT   
  
  
- TAAAAAAAAA GATAAATCAA TAGTTTAAAA ATACTGCGCT TAAAGTCCAA ACTGTCCAAT TGGACCAAAC   
  
  
- TTCCCAATTG GGTAAATTAA GTTAAAAAAA AGAAAAAGAA GTAATCAAAA AAAGAAGGTA AATTAAAAAA   
  
  
- AAGAAAAAGA AGTAATTACA ATAATAATTA TAATATTTAT AGTGAGAACC TAGTCCGCAA CGTCGATTTA   
  
  
- GATTTTGAGT ATCCATATCG AAATATCTTT AAACTTAGAA TCAAAAAAAA ATATAAAAAA TATGCTTTTT   
  
  
- TAATTGGGCA CGTCCATTAC ACTGATCATT ATTGTAATAA AAGACAACTT GGCTTGTTCT TTCTTAGCAT   
  
  
- CAATCCACAG TCTCTGAGTC TCAGCGACGA AGAGTGTAAG TTTGGAACAT TCGCAAACCA GTAACATTCT   
  
  
- GGGAGAGAAA GAAAATAGTT TATTGGAAAG AAATAAAGAA TTTATTTAAT TCCTGGGAAG AAAAAAATAT   
  
  
- TTTTTATTTT TATTTTTTAA GAAGGAAGAA GGGTATTAAC TAAGTTAGCG TTTACTCAGT ACAGTACTGG   
  
  
- GCCTCTGGGA AACTTTTCAG TTTAATCTAT TTCTAACTCT GCTACCAAAG TTCTCTGTGT TGGTGAGTTC   
  
  
- ATAAATGGTA ACACTACTCG ACTGTTCCTA ACAGTAGGTC TTACCAGAGA GGAGTGCTAC AGTGGTCGGT   
  
  
- CACCTTAGGT TTAATTTAAC TTAAATAACC CTTTACCCTA TTGAGAAGGA AAATATATAA TTTTTAATTT   
  
  
- TTTATATATG TCACGGTTAA GATGACATGA CCAATTAATG GGATAGTTGT AAATTCATCA AATACATTGC   
  
  
- AACATAATCA ACAAAAAATT TTATTAAAAA AAAATTATAT AATTTTATTA AAAAAATTTT TTAAATAAAA   
  
  
- ACTATAATTA TACAATTTTG TTAAATTTTT GTAACTTTTT TTTATTTTAT TTTATTTAAG TTCAAAACGT   
  
  
- GTTTTTTTGT GATAACGTGG CGCATGTATT TACTACAGAA TCACATAAAT CGTAACACTA TCGAAAACAC   
  
  
- TAGTAAGACC AAAATTTTTT TGTTTAAATT TTCTTTATAA AAATCAACAC CAAAATTTTT CATCTAAAAT   
  
  
- TTTTTATGTA CAAACTAATT TAGATGATAC TCTATTTAAA AATGTACATT TTATTTATTT TTGTATAAAG   
  
  
- GTAGTTAATT TTTTTTTGTA CAAGAAAGTC AAAGATGTAC TGATTATAAA TTTTATGTTA ATGAATATTT   
  
  
- CAGGCCATGC TTTCGTGGGA GATTTACCTC TTACTTGACA TACATAAGAA GGGTACCTGG ACACTAAAAA   
  
  
- GGCATCCTTT CCGTTAAAGG TAAGTCTTAT GTTCTTCTAT GTACAGAAAA CTTTGAGAAT AATCGACAGA   
  
  
- TGGATAGTTG GTTCTTACTG CGAAGAGTAG CATACAGTGA TATTTTAAAC CAACTACTGT GGTTGTGAAA   
  
  
- GGGTAACCAT AAGTACCGAA AAATCGACGT GCTAGGTTG

+     ACE

| Site Name | Organism | Position | Strand | Matrix score. | sequence | function |
| --- | --- | --- | --- | --- | --- | --- |
| ACE | Petroselinum crispum | 456 | + | 9 | GACACGTATG | cis-acting element involved in light responsiveness |

>Potri.004G051700.1   
+ TTATCAAACT TTCATGATGT GAATCTCAGG TTTGACGGGT TAACTTGGTT TGAAGGGTTA ACCCAGTTAA   
  
  
+ TTCAAATTTT TTTTTTCTTC ATTAGTTTTT TTTTCTTCCT GTTGATTTTT TTCATTGAAC TTATATATTT   
  
  
+ TAATTATAAT AATATAATAA TAATTACATA TACTAAAATA CTGAGTTGTC TTCTTTTTTT TTTACGTTTT   
  
  
+ TAAATTATTA TTTTTTTGTT TTTGTTTTTT TCTTTGTGTT TTTTTTATTT TAATGAATTT TTTTTGTTTA   
  
  
+ ATTTAGTTTG TTAATGTTTA ATTTTTTTTA TTTAGTTATC ATATTTTCAT AATACGGATC CCGAGTTTGA   
  
  
+ TGAGTTAATC CAGAATTTTT GCTTTTCTTC TTTTTAATTA ATTTTTTTTT GTTTAGTTTA GTTTGTTAAT   
  
  
+ GTTAAATTTT TTTTATTTAA TTATCAGACT TTTATGACAC GTATCTCGGG TTTGACGGGT TAACGCGTCA   
  
  
+ ATTTTTTTTT CTATTTAGTT ATCAAATTTT TATGACGCGA ATTTCAGGTT TGACAGGTTA ACCTGGTTTG   
  
  
+ AAGGGTTAAC CCATTTAATT CAATTTTTTT TCTTTTTCTT CATTAGTTTT TTTCTTCCAT TTAATTTTTT   
  
  
+ TTCTTTTTCT TCATTAATGT TATTATTAAT ATTATAAATA TCACTCTTGG ATCAGGCGTT GCAGCTAAAT   
  
  
+ CTAAAACTCA TAGGTATAGC TTTATAGAAA TTTGAATCTT AGTTTTTTTT TATATTTTTT ATACGAAAAA   
  
  
+ ATTAACCCGT GCAGGTAATG TGACTAGTAA TAACATTATT TTCTGTTGAA CCGAACAAGA AAGAATCGTA   
  
  
+ GTTAGGTGTC AGAGACTCAG AGTCGCTGCT TCTCACATTC AAACCTTGTA AGCGTTTGGT CATTGTAAGA   
  
  
+ CCCTCTCTTT CTTTTATCAA ATAACCTTTC TTTATTTCTT AAATAAATTA AGGACCCTTC TTTTTTTATA   
  
  
+ AAAAATAAAA ATAAAAAATT CTTCCTTCTT CCCATAATTG ATTCAATCGC AAATGAGTCA TGTCATGACC   
  
  
+ CGGAGACCCT TTGAAAAGTC AAATTAGATA AAGATTGAGA CGATGGTTTC AAGAGACACA ACCACTCAAG   
  
  
+ TATTTACCAT TGTGATGAGC TGACAAGGAT TGTCATCCAG AATGGTCTCT CCTCACGATG TCACCAGCCA   
  
  
+ GTGGAATCCA AATTAAATTG AATTTATTGG GAAATGGGAT AACTCTTCCT TTTATATATT AAAAATTAAA   
  
  
+ AAATATATAC AGTGCCAATT CTACTGTACT GGTTAATTAC CCTATCAACA TTTAAGTAGT TTATGTAACG   
  
  
+ TTGTATTAGT TGTTTTTTAA AATAATTTTT TTTTAATATA TTAAAATAAT TTTTTTAAAA AATTTATTTT   
  
  
+ TGATATTAAT ATGTTAAAAC AATTTAAAAA CATTGAAAAA AAATAAAATA AAATAAATTC AAGTTTTGCA   
  
  
+ CAAAAAAACA CTATTGCACC GCGTACATAA ATGATGTCTT AGTGTATTTA GCATTGTGAT AGCTTTTGTG   
  
  
+ ATCATTCTGG TTTTAAAAAA ACAAATTTAA AAGAAATATT TTTAGTTGTG GTTTTAAAAA GTAGATTTTA   
  
  
+ AAAAATACAT GTTTGATTAA ATCTACTATG AGATAAATTT TTACATGTAA AATAAATAAA AACATATTTC   
  
  
+ CATCAATTAA AAAAAAACAT GTTCTTTCAG TTTCTACATG ACTAATATTT AAAATACAAT TACTTATAAA   
  
  
+ GTCCGGTACG AAAGCACCCT CTAAATGGAG AATGAACTGT ATGTATTCTT CCCATGGACC TGTGATTTTT   
  
  
+ CCGTAGGAAA GGCAATTTCC ATTCAGAATA CAAGAAGATA CATGTCTTTT GAAACTCTTA TTAGCTGTCT   
  
  
+ ACCTATCAAC CAAGAATGAC GCTTCTCATC GTATGTCACT ATAAAATTTG GTTGATGACA CCAACACTTT   
  
  
+ CCCATTGGTA TTCATGGCTT TTTAGCTGCA CGATCCAAC  

- AATAGTTTGA AAGTACTACA CTTAGAGTCC AAACTGCCCA ATTGAACCAA ACTTCCCAAT TGGGTCAATT   
  
  
- AAGTTTAAAA AAAAAAGAAG TAATCAAAAA AAAAGAAGGA CAACTAAAAA AAGTAACTTG AATATATAAA   
  
  
- ATTAATATTA TTATATTATT ATTAATGTAT ATGATTTTAT GACTCAACAG AAGAAAAAAA AAATGCAAAA   
  
  
- ATTTAATAAT AAAAAAACAA AAACAAAAAA AGAAACACAA AAAAAATAAA ATTACTTAAA AAAAACAAAT   
  
  
- TAAATCAAAC AATTACAAAT TAAAAAAAAT AAATCAATAG TATAAAAGTA TTATGCCTAG GGCTCAAACT   
  
  
- ACTCAATTAG GTCTTAAAAA CGAAAAGAAG AAAAATTAAT TAAAAAAAAA CAAATCAAAT CAAACAATTA   
  
  
- CAATTTAAAA AAAATAAATT AATAGTCTGA AAATACTGTG CATAGAGCCC AAACTGCCCA ATTGCGCAGT   
  
  
- TAAAAAAAAA GATAAATCAA TAGTTTAAAA ATACTGCGCT TAAAGTCCAA ACTGTCCAAT TGGACCAAAC   
  
  
- TTCCCAATTG GGTAAATTAA GTTAAAAAAA AGAAAAAGAA GTAATCAAAA AAAGAAGGTA AATTAAAAAA   
  
  
- AAGAAAAAGA AGTAATTACA ATAATAATTA TAATATTTAT AGTGAGAACC TAGTCCGCAA CGTCGATTTA   
  
  
- GATTTTGAGT ATCCATATCG AAATATCTTT AAACTTAGAA TCAAAAAAAA ATATAAAAAA TATGCTTTTT   
  
  
- TAATTGGGCA CGTCCATTAC ACTGATCATT ATTGTAATAA AAGACAACTT GGCTTGTTCT TTCTTAGCAT   
  
  
- CAATCCACAG TCTCTGAGTC TCAGCGACGA AGAGTGTAAG TTTGGAACAT TCGCAAACCA GTAACATTCT   
  
  
- GGGAGAGAAA GAAAATAGTT TATTGGAAAG AAATAAAGAA TTTATTTAAT TCCTGGGAAG AAAAAAATAT   
  
  
- TTTTTATTTT TATTTTTTAA GAAGGAAGAA GGGTATTAAC TAAGTTAGCG TTTACTCAGT ACAGTACTGG   
  
  
- GCCTCTGGGA AACTTTTCAG TTTAATCTAT TTCTAACTCT GCTACCAAAG TTCTCTGTGT TGGTGAGTTC   
  
  
- ATAAATGGTA ACACTACTCG ACTGTTCCTA ACAGTAGGTC TTACCAGAGA GGAGTGCTAC AGTGGTCGGT   
  
  
- CACCTTAGGT TTAATTTAAC TTAAATAACC CTTTACCCTA TTGAGAAGGA AAATATATAA TTTTTAATTT   
  
  
- TTTATATATG TCACGGTTAA GATGACATGA CCAATTAATG GGATAGTTGT AAATTCATCA AATACATTGC   
  
  
- AACATAATCA ACAAAAAATT TTATTAAAAA AAAATTATAT AATTTTATTA AAAAAATTTT TTAAATAAAA   
  
  
- ACTATAATTA TACAATTTTG TTAAATTTTT GTAACTTTTT TTTATTTTAT TTTATTTAAG TTCAAAACGT   
  
  
- GTTTTTTTGT GATAACGTGG CGCATGTATT TACTACAGAA TCACATAAAT CGTAACACTA TCGAAAACAC   
  
  
- TAGTAAGACC AAAATTTTTT TGTTTAAATT TTCTTTATAA AAATCAACAC CAAAATTTTT CATCTAAAAT   
  
  
- TTTTTATGTA CAAACTAATT TAGATGATAC TCTATTTAAA AATGTACATT TTATTTATTT TTGTATAAAG   
  
  
- GTAGTTAATT TTTTTTTGTA CAAGAAAGTC AAAGATGTAC TGATTATAAA TTTTATGTTA ATGAATATTT   
  
  
- CAGGCCATGC TTTCGTGGGA GATTTACCTC TTACTTGACA TACATAAGAA GGGTACCTGG ACACTAAAAA   
  
  
- GGCATCCTTT CCGTTAAAGG TAAGTCTTAT GTTCTTCTAT GTACAGAAAA CTTTGAGAAT AATCGACAGA   
  
  
- TGGATAGTTG GTTCTTACTG CGAAGAGTAG CATACAGTGA TATTTTAAAC CAACTACTGT GGTTGTGAAA   
  
  
- GGGTAACCAT AAGTACCGAA AAATCGACGT GCTAGGTTG

+     ARE

| Site Name | Organism | Position | Strand | Matrix score. | sequence | function |
| --- | --- | --- | --- | --- | --- | --- |
| ARE | Zea mays | 46 | - | 6 | AAACCA | cis-acting regulatory element essential for the anaerobic induction |
| ARE | Zea mays | 1589 | - | 6 | AAACCA | cis-acting regulatory element essential for the anaerobic induction |
| ARE | Zea mays | 1094 | - | 6 | AAACCA | cis-acting regulatory element essential for the anaerobic induction |
| ARE | Zea mays | 554 | - | 6 | AAACCA | cis-acting regulatory element essential for the anaerobic induction |
| ARE | Zea mays | 1548 | - | 6 | AAACCA | cis-acting regulatory element essential for the anaerobic induction |

>Potri.004G051700.1   
+ TTATCAAACT TTCATGATGT GAATCTCAGG TTTGACGGGT TAACTTGGTT TGAAGGGTTA ACCCAGTTAA   
  
  
+ TTCAAATTTT TTTTTTCTTC ATTAGTTTTT TTTTCTTCCT GTTGATTTTT TTCATTGAAC TTATATATTT   
  
  
+ TAATTATAAT AATATAATAA TAATTACATA TACTAAAATA CTGAGTTGTC TTCTTTTTTT TTTACGTTTT   
  
  
+ TAAATTATTA TTTTTTTGTT TTTGTTTTTT TCTTTGTGTT TTTTTTATTT TAATGAATTT TTTTTGTTTA   
  
  
+ ATTTAGTTTG TTAATGTTTA ATTTTTTTTA TTTAGTTATC ATATTTTCAT AATACGGATC CCGAGTTTGA   
  
  
+ TGAGTTAATC CAGAATTTTT GCTTTTCTTC TTTTTAATTA ATTTTTTTTT GTTTAGTTTA GTTTGTTAAT   
  
  
+ GTTAAATTTT TTTTATTTAA TTATCAGACT TTTATGACAC GTATCTCGGG TTTGACGGGT TAACGCGTCA   
  
  
+ ATTTTTTTTT CTATTTAGTT ATCAAATTTT TATGACGCGA ATTTCAGGTT TGACAGGTTA ACCTGGTTTG   
  
  
+ AAGGGTTAAC CCATTTAATT CAATTTTTTT TCTTTTTCTT CATTAGTTTT TTTCTTCCAT TTAATTTTTT   
  
  
+ TTCTTTTTCT TCATTAATGT TATTATTAAT ATTATAAATA TCACTCTTGG ATCAGGCGTT GCAGCTAAAT   
  
  
+ CTAAAACTCA TAGGTATAGC TTTATAGAAA TTTGAATCTT AGTTTTTTTT TATATTTTTT ATACGAAAAA   
  
  
+ ATTAACCCGT GCAGGTAATG TGACTAGTAA TAACATTATT TTCTGTTGAA CCGAACAAGA AAGAATCGTA   
  
  
+ GTTAGGTGTC AGAGACTCAG AGTCGCTGCT TCTCACATTC AAACCTTGTA AGCGTTTGGT CATTGTAAGA   
  
  
+ CCCTCTCTTT CTTTTATCAA ATAACCTTTC TTTATTTCTT AAATAAATTA AGGACCCTTC TTTTTTTATA   
  
  
+ AAAAATAAAA ATAAAAAATT CTTCCTTCTT CCCATAATTG ATTCAATCGC AAATGAGTCA TGTCATGACC   
  
  
+ CGGAGACCCT TTGAAAAGTC AAATTAGATA AAGATTGAGA CGATGGTTTC AAGAGACACA ACCACTCAAG   
  
  
+ TATTTACCAT TGTGATGAGC TGACAAGGAT TGTCATCCAG AATGGTCTCT CCTCACGATG TCACCAGCCA   
  
  
+ GTGGAATCCA AATTAAATTG AATTTATTGG GAAATGGGAT AACTCTTCCT TTTATATATT AAAAATTAAA   
  
  
+ AAATATATAC AGTGCCAATT CTACTGTACT GGTTAATTAC CCTATCAACA TTTAAGTAGT TTATGTAACG   
  
  
+ TTGTATTAGT TGTTTTTTAA AATAATTTTT TTTTAATATA TTAAAATAAT TTTTTTAAAA AATTTATTTT   
  
  
+ TGATATTAAT ATGTTAAAAC AATTTAAAAA CATTGAAAAA AAATAAAATA AAATAAATTC AAGTTTTGCA   
  
  
+ CAAAAAAACA CTATTGCACC GCGTACATAA ATGATGTCTT AGTGTATTTA GCATTGTGAT AGCTTTTGTG   
  
  
+ ATCATTCTGG TTTTAAAAAA ACAAATTTAA AAGAAATATT TTTAGTTGTG GTTTTAAAAA GTAGATTTTA   
  
  
+ AAAAATACAT GTTTGATTAA ATCTACTATG AGATAAATTT TTACATGTAA AATAAATAAA AACATATTTC   
  
  
+ CATCAATTAA AAAAAAACAT GTTCTTTCAG TTTCTACATG ACTAATATTT AAAATACAAT TACTTATAAA   
  
  
+ GTCCGGTACG AAAGCACCCT CTAAATGGAG AATGAACTGT ATGTATTCTT CCCATGGACC TGTGATTTTT   
  
  
+ CCGTAGGAAA GGCAATTTCC ATTCAGAATA CAAGAAGATA CATGTCTTTT GAAACTCTTA TTAGCTGTCT   
  
  
+ ACCTATCAAC CAAGAATGAC GCTTCTCATC GTATGTCACT ATAAAATTTG GTTGATGACA CCAACACTTT   
  
  
+ CCCATTGGTA TTCATGGCTT TTTAGCTGCA CGATCCAAC  

- AATAGTTTGA AAGTACTACA CTTAGAGTCC AAACTGCCCA ATTGAACCAA ACTTCCCAAT TGGGTCAATT   
  
  
- AAGTTTAAAA AAAAAAGAAG TAATCAAAAA AAAAGAAGGA CAACTAAAAA AAGTAACTTG AATATATAAA   
  
  
- ATTAATATTA TTATATTATT ATTAATGTAT ATGATTTTAT GACTCAACAG AAGAAAAAAA AAATGCAAAA   
  
  
- ATTTAATAAT AAAAAAACAA AAACAAAAAA AGAAACACAA AAAAAATAAA ATTACTTAAA AAAAACAAAT   
  
  
- TAAATCAAAC AATTACAAAT TAAAAAAAAT AAATCAATAG TATAAAAGTA TTATGCCTAG GGCTCAAACT   
  
  
- ACTCAATTAG GTCTTAAAAA CGAAAAGAAG AAAAATTAAT TAAAAAAAAA CAAATCAAAT CAAACAATTA   
  
  
- CAATTTAAAA AAAATAAATT AATAGTCTGA AAATACTGTG CATAGAGCCC AAACTGCCCA ATTGCGCAGT   
  
  
- TAAAAAAAAA GATAAATCAA TAGTTTAAAA ATACTGCGCT TAAAGTCCAA ACTGTCCAAT TGGACCAAAC   
  
  
- TTCCCAATTG GGTAAATTAA GTTAAAAAAA AGAAAAAGAA GTAATCAAAA AAAGAAGGTA AATTAAAAAA   
  
  
- AAGAAAAAGA AGTAATTACA ATAATAATTA TAATATTTAT AGTGAGAACC TAGTCCGCAA CGTCGATTTA   
  
  
- GATTTTGAGT ATCCATATCG AAATATCTTT AAACTTAGAA TCAAAAAAAA ATATAAAAAA TATGCTTTTT   
  
  
- TAATTGGGCA CGTCCATTAC ACTGATCATT ATTGTAATAA AAGACAACTT GGCTTGTTCT TTCTTAGCAT   
  
  
- CAATCCACAG TCTCTGAGTC TCAGCGACGA AGAGTGTAAG TTTGGAACAT TCGCAAACCA GTAACATTCT   
  
  
- GGGAGAGAAA GAAAATAGTT TATTGGAAAG AAATAAAGAA TTTATTTAAT TCCTGGGAAG AAAAAAATAT   
  
  
- TTTTTATTTT TATTTTTTAA GAAGGAAGAA GGGTATTAAC TAAGTTAGCG TTTACTCAGT ACAGTACTGG   
  
  
- GCCTCTGGGA AACTTTTCAG TTTAATCTAT TTCTAACTCT GCTACCAAAG TTCTCTGTGT TGGTGAGTTC   
  
  
- ATAAATGGTA ACACTACTCG ACTGTTCCTA ACAGTAGGTC TTACCAGAGA GGAGTGCTAC AGTGGTCGGT   
  
  
- CACCTTAGGT TTAATTTAAC TTAAATAACC CTTTACCCTA TTGAGAAGGA AAATATATAA TTTTTAATTT   
  
  
- TTTATATATG TCACGGTTAA GATGACATGA CCAATTAATG GGATAGTTGT AAATTCATCA AATACATTGC   
  
  
- AACATAATCA ACAAAAAATT TTATTAAAAA AAAATTATAT AATTTTATTA AAAAAATTTT TTAAATAAAA   
  
  
- ACTATAATTA TACAATTTTG TTAAATTTTT GTAACTTTTT TTTATTTTAT TTTATTTAAG TTCAAAACGT   
  
  
- GTTTTTTTGT GATAACGTGG CGCATGTATT TACTACAGAA TCACATAAAT CGTAACACTA TCGAAAACAC   
  
  
- TAGTAAGACC AAAATTTTTT TGTTTAAATT TTCTTTATAA AAATCAACAC CAAAATTTTT CATCTAAAAT   
  
  
- TTTTTATGTA CAAACTAATT TAGATGATAC TCTATTTAAA AATGTACATT TTATTTATTT TTGTATAAAG   
  
  
- GTAGTTAATT TTTTTTTGTA CAAGAAAGTC AAAGATGTAC TGATTATAAA TTTTATGTTA ATGAATATTT   
  
  
- CAGGCCATGC TTTCGTGGGA GATTTACCTC TTACTTGACA TACATAAGAA GGGTACCTGG ACACTAAAAA   
  
  
- GGCATCCTTT CCGTTAAAGG TAAGTCTTAT GTTCTTCTAT GTACAGAAAA CTTTGAGAAT AATCGACAGA   
  
  
- TGGATAGTTG GTTCTTACTG CGAAGAGTAG CATACAGTGA TATTTTAAAC CAACTACTGT GGTTGTGAAA   
  
  
- GGGTAACCAT AAGTACCGAA AAATCGACGT GCTAGGTTG

+     AT-rich sequence

| Site Name | Organism | Position | Strand | Matrix score. | sequence | function |
| --- | --- | --- | --- | --- | --- | --- |
| AT-rich sequence | Pisum sativum | 174 | + | 9 | TAAAATACT | element for maximal elicitor-mediated activation (2copies) |

>Potri.004G051700.1   
+ TTATCAAACT TTCATGATGT GAATCTCAGG TTTGACGGGT TAACTTGGTT TGAAGGGTTA ACCCAGTTAA   
  
  
+ TTCAAATTTT TTTTTTCTTC ATTAGTTTTT TTTTCTTCCT GTTGATTTTT TTCATTGAAC TTATATATTT   
  
  
+ TAATTATAAT AATATAATAA TAATTACATA TACTAAAATA CTGAGTTGTC TTCTTTTTTT TTTACGTTTT   
  
  
+ TAAATTATTA TTTTTTTGTT TTTGTTTTTT TCTTTGTGTT TTTTTTATTT TAATGAATTT TTTTTGTTTA   
  
  
+ ATTTAGTTTG TTAATGTTTA ATTTTTTTTA TTTAGTTATC ATATTTTCAT AATACGGATC CCGAGTTTGA   
  
  
+ TGAGTTAATC CAGAATTTTT GCTTTTCTTC TTTTTAATTA ATTTTTTTTT GTTTAGTTTA GTTTGTTAAT   
  
  
+ GTTAAATTTT TTTTATTTAA TTATCAGACT TTTATGACAC GTATCTCGGG TTTGACGGGT TAACGCGTCA   
  
  
+ ATTTTTTTTT CTATTTAGTT ATCAAATTTT TATGACGCGA ATTTCAGGTT TGACAGGTTA ACCTGGTTTG   
  
  
+ AAGGGTTAAC CCATTTAATT CAATTTTTTT TCTTTTTCTT CATTAGTTTT TTTCTTCCAT TTAATTTTTT   
  
  
+ TTCTTTTTCT TCATTAATGT TATTATTAAT ATTATAAATA TCACTCTTGG ATCAGGCGTT GCAGCTAAAT   
  
  
+ CTAAAACTCA TAGGTATAGC TTTATAGAAA TTTGAATCTT AGTTTTTTTT TATATTTTTT ATACGAAAAA   
  
  
+ ATTAACCCGT GCAGGTAATG TGACTAGTAA TAACATTATT TTCTGTTGAA CCGAACAAGA AAGAATCGTA   
  
  
+ GTTAGGTGTC AGAGACTCAG AGTCGCTGCT TCTCACATTC AAACCTTGTA AGCGTTTGGT CATTGTAAGA   
  
  
+ CCCTCTCTTT CTTTTATCAA ATAACCTTTC TTTATTTCTT AAATAAATTA AGGACCCTTC TTTTTTTATA   
  
  
+ AAAAATAAAA ATAAAAAATT CTTCCTTCTT CCCATAATTG ATTCAATCGC AAATGAGTCA TGTCATGACC   
  
  
+ CGGAGACCCT TTGAAAAGTC AAATTAGATA AAGATTGAGA CGATGGTTTC AAGAGACACA ACCACTCAAG   
  
  
+ TATTTACCAT TGTGATGAGC TGACAAGGAT TGTCATCCAG AATGGTCTCT CCTCACGATG TCACCAGCCA   
  
  
+ GTGGAATCCA AATTAAATTG AATTTATTGG GAAATGGGAT AACTCTTCCT TTTATATATT AAAAATTAAA   
  
  
+ AAATATATAC AGTGCCAATT CTACTGTACT GGTTAATTAC CCTATCAACA TTTAAGTAGT TTATGTAACG   
  
  
+ TTGTATTAGT TGTTTTTTAA AATAATTTTT TTTTAATATA TTAAAATAAT TTTTTTAAAA AATTTATTTT   
  
  
+ TGATATTAAT ATGTTAAAAC AATTTAAAAA CATTGAAAAA AAATAAAATA AAATAAATTC AAGTTTTGCA   
  
  
+ CAAAAAAACA CTATTGCACC GCGTACATAA ATGATGTCTT AGTGTATTTA GCATTGTGAT AGCTTTTGTG   
  
  
+ ATCATTCTGG TTTTAAAAAA ACAAATTTAA AAGAAATATT TTTAGTTGTG GTTTTAAAAA GTAGATTTTA   
  
  
+ AAAAATACAT GTTTGATTAA ATCTACTATG AGATAAATTT TTACATGTAA AATAAATAAA AACATATTTC   
  
  
+ CATCAATTAA AAAAAAACAT GTTCTTTCAG TTTCTACATG ACTAATATTT AAAATACAAT TACTTATAAA   
  
  
+ GTCCGGTACG AAAGCACCCT CTAAATGGAG AATGAACTGT ATGTATTCTT CCCATGGACC TGTGATTTTT   
  
  
+ CCGTAGGAAA GGCAATTTCC ATTCAGAATA CAAGAAGATA CATGTCTTTT GAAACTCTTA TTAGCTGTCT   
  
  
+ ACCTATCAAC CAAGAATGAC GCTTCTCATC GTATGTCACT ATAAAATTTG GTTGATGACA CCAACACTTT   
  
  
+ CCCATTGGTA TTCATGGCTT TTTAGCTGCA CGATCCAAC  

- AATAGTTTGA AAGTACTACA CTTAGAGTCC AAACTGCCCA ATTGAACCAA ACTTCCCAAT TGGGTCAATT   
  
  
- AAGTTTAAAA AAAAAAGAAG TAATCAAAAA AAAAGAAGGA CAACTAAAAA AAGTAACTTG AATATATAAA   
  
  
- ATTAATATTA TTATATTATT ATTAATGTAT ATGATTTTAT GACTCAACAG AAGAAAAAAA AAATGCAAAA   
  
  
- ATTTAATAAT AAAAAAACAA AAACAAAAAA AGAAACACAA AAAAAATAAA ATTACTTAAA AAAAACAAAT   
  
  
- TAAATCAAAC AATTACAAAT TAAAAAAAAT AAATCAATAG TATAAAAGTA TTATGCCTAG GGCTCAAACT   
  
  
- ACTCAATTAG GTCTTAAAAA CGAAAAGAAG AAAAATTAAT TAAAAAAAAA CAAATCAAAT CAAACAATTA   
  
  
- CAATTTAAAA AAAATAAATT AATAGTCTGA AAATACTGTG CATAGAGCCC AAACTGCCCA ATTGCGCAGT   
  
  
- TAAAAAAAAA GATAAATCAA TAGTTTAAAA ATACTGCGCT TAAAGTCCAA ACTGTCCAAT TGGACCAAAC   
  
  
- TTCCCAATTG GGTAAATTAA GTTAAAAAAA AGAAAAAGAA GTAATCAAAA AAAGAAGGTA AATTAAAAAA   
  
  
- AAGAAAAAGA AGTAATTACA ATAATAATTA TAATATTTAT AGTGAGAACC TAGTCCGCAA CGTCGATTTA   
  
  
- GATTTTGAGT ATCCATATCG AAATATCTTT AAACTTAGAA TCAAAAAAAA ATATAAAAAA TATGCTTTTT   
  
  
- TAATTGGGCA CGTCCATTAC ACTGATCATT ATTGTAATAA AAGACAACTT GGCTTGTTCT TTCTTAGCAT   
  
  
- CAATCCACAG TCTCTGAGTC TCAGCGACGA AGAGTGTAAG TTTGGAACAT TCGCAAACCA GTAACATTCT   
  
  
- GGGAGAGAAA GAAAATAGTT TATTGGAAAG AAATAAAGAA TTTATTTAAT TCCTGGGAAG AAAAAAATAT   
  
  
- TTTTTATTTT TATTTTTTAA GAAGGAAGAA GGGTATTAAC TAAGTTAGCG TTTACTCAGT ACAGTACTGG   
  
  
- GCCTCTGGGA AACTTTTCAG TTTAATCTAT TTCTAACTCT GCTACCAAAG TTCTCTGTGT TGGTGAGTTC   
  
  
- ATAAATGGTA ACACTACTCG ACTGTTCCTA ACAGTAGGTC TTACCAGAGA GGAGTGCTAC AGTGGTCGGT   
  
  
- CACCTTAGGT TTAATTTAAC TTAAATAACC CTTTACCCTA TTGAGAAGGA AAATATATAA TTTTTAATTT   
  
  
- TTTATATATG TCACGGTTAA GATGACATGA CCAATTAATG GGATAGTTGT AAATTCATCA AATACATTGC   
  
  
- AACATAATCA ACAAAAAATT TTATTAAAAA AAAATTATAT AATTTTATTA AAAAAATTTT TTAAATAAAA   
  
  
- ACTATAATTA TACAATTTTG TTAAATTTTT GTAACTTTTT TTTATTTTAT TTTATTTAAG TTCAAAACGT   
  
  
- GTTTTTTTGT GATAACGTGG CGCATGTATT TACTACAGAA TCACATAAAT CGTAACACTA TCGAAAACAC   
  
  
- TAGTAAGACC AAAATTTTTT TGTTTAAATT TTCTTTATAA AAATCAACAC CAAAATTTTT CATCTAAAAT   
  
  
- TTTTTATGTA CAAACTAATT TAGATGATAC TCTATTTAAA AATGTACATT TTATTTATTT TTGTATAAAG   
  
  
- GTAGTTAATT TTTTTTTGTA CAAGAAAGTC AAAGATGTAC TGATTATAAA TTTTATGTTA ATGAATATTT   
  
  
- CAGGCCATGC TTTCGTGGGA GATTTACCTC TTACTTGACA TACATAAGAA GGGTACCTGG ACACTAAAAA   
  
  
- GGCATCCTTT CCGTTAAAGG TAAGTCTTAT GTTCTTCTAT GTACAGAAAA CTTTGAGAAT AATCGACAGA   
  
  
- TGGATAGTTG GTTCTTACTG CGAAGAGTAG CATACAGTGA TATTTTAAAC CAACTACTGT GGTTGTGAAA   
  
  
- GGGTAACCAT AAGTACCGAA AAATCGACGT GCTAGGTTG

+     AT~ABRE

| Site Name | Organism | Position | Strand | Matrix score. | sequence | function |
| --- | --- | --- | --- | --- | --- | --- |
| AT~ABRE | Arabidopsis thaliana | 456 | - | 8 | TACGTGTC |  |

>Potri.004G051700.1   
+ TTATCAAACT TTCATGATGT GAATCTCAGG TTTGACGGGT TAACTTGGTT TGAAGGGTTA ACCCAGTTAA   
  
  
+ TTCAAATTTT TTTTTTCTTC ATTAGTTTTT TTTTCTTCCT GTTGATTTTT TTCATTGAAC TTATATATTT   
  
  
+ TAATTATAAT AATATAATAA TAATTACATA TACTAAAATA CTGAGTTGTC TTCTTTTTTT TTTACGTTTT   
  
  
+ TAAATTATTA TTTTTTTGTT TTTGTTTTTT TCTTTGTGTT TTTTTTATTT TAATGAATTT TTTTTGTTTA   
  
  
+ ATTTAGTTTG TTAATGTTTA ATTTTTTTTA TTTAGTTATC ATATTTTCAT AATACGGATC CCGAGTTTGA   
  
  
+ TGAGTTAATC CAGAATTTTT GCTTTTCTTC TTTTTAATTA ATTTTTTTTT GTTTAGTTTA GTTTGTTAAT   
  
  
+ GTTAAATTTT TTTTATTTAA TTATCAGACT TTTATGACAC GTATCTCGGG TTTGACGGGT TAACGCGTCA   
  
  
+ ATTTTTTTTT CTATTTAGTT ATCAAATTTT TATGACGCGA ATTTCAGGTT TGACAGGTTA ACCTGGTTTG   
  
  
+ AAGGGTTAAC CCATTTAATT CAATTTTTTT TCTTTTTCTT CATTAGTTTT TTTCTTCCAT TTAATTTTTT   
  
  
+ TTCTTTTTCT TCATTAATGT TATTATTAAT ATTATAAATA TCACTCTTGG ATCAGGCGTT GCAGCTAAAT   
  
  
+ CTAAAACTCA TAGGTATAGC TTTATAGAAA TTTGAATCTT AGTTTTTTTT TATATTTTTT ATACGAAAAA   
  
  
+ ATTAACCCGT GCAGGTAATG TGACTAGTAA TAACATTATT TTCTGTTGAA CCGAACAAGA AAGAATCGTA   
  
  
+ GTTAGGTGTC AGAGACTCAG AGTCGCTGCT TCTCACATTC AAACCTTGTA AGCGTTTGGT CATTGTAAGA   
  
  
+ CCCTCTCTTT CTTTTATCAA ATAACCTTTC TTTATTTCTT AAATAAATTA AGGACCCTTC TTTTTTTATA   
  
  
+ AAAAATAAAA ATAAAAAATT CTTCCTTCTT CCCATAATTG ATTCAATCGC AAATGAGTCA TGTCATGACC   
  
  
+ CGGAGACCCT TTGAAAAGTC AAATTAGATA AAGATTGAGA CGATGGTTTC AAGAGACACA ACCACTCAAG   
  
  
+ TATTTACCAT TGTGATGAGC TGACAAGGAT TGTCATCCAG AATGGTCTCT CCTCACGATG TCACCAGCCA   
  
  
+ GTGGAATCCA AATTAAATTG AATTTATTGG GAAATGGGAT AACTCTTCCT TTTATATATT AAAAATTAAA   
  
  
+ AAATATATAC AGTGCCAATT CTACTGTACT GGTTAATTAC CCTATCAACA TTTAAGTAGT TTATGTAACG   
  
  
+ TTGTATTAGT TGTTTTTTAA AATAATTTTT TTTTAATATA TTAAAATAAT TTTTTTAAAA AATTTATTTT   
  
  
+ TGATATTAAT ATGTTAAAAC AATTTAAAAA CATTGAAAAA AAATAAAATA AAATAAATTC AAGTTTTGCA   
  
  
+ CAAAAAAACA CTATTGCACC GCGTACATAA ATGATGTCTT AGTGTATTTA GCATTGTGAT AGCTTTTGTG   
  
  
+ ATCATTCTGG TTTTAAAAAA ACAAATTTAA AAGAAATATT TTTAGTTGTG GTTTTAAAAA GTAGATTTTA   
  
  
+ AAAAATACAT GTTTGATTAA ATCTACTATG AGATAAATTT TTACATGTAA AATAAATAAA AACATATTTC   
  
  
+ CATCAATTAA AAAAAAACAT GTTCTTTCAG TTTCTACATG ACTAATATTT AAAATACAAT TACTTATAAA   
  
  
+ GTCCGGTACG AAAGCACCCT CTAAATGGAG AATGAACTGT ATGTATTCTT CCCATGGACC TGTGATTTTT   
  
  
+ CCGTAGGAAA GGCAATTTCC ATTCAGAATA CAAGAAGATA CATGTCTTTT GAAACTCTTA TTAGCTGTCT   
  
  
+ ACCTATCAAC CAAGAATGAC GCTTCTCATC GTATGTCACT ATAAAATTTG GTTGATGACA CCAACACTTT   
  
  
+ CCCATTGGTA TTCATGGCTT TTTAGCTGCA CGATCCAAC  

- AATAGTTTGA AAGTACTACA CTTAGAGTCC AAACTGCCCA ATTGAACCAA ACTTCCCAAT TGGGTCAATT   
  
  
- AAGTTTAAAA AAAAAAGAAG TAATCAAAAA AAAAGAAGGA CAACTAAAAA AAGTAACTTG AATATATAAA   
  
  
- ATTAATATTA TTATATTATT ATTAATGTAT ATGATTTTAT GACTCAACAG AAGAAAAAAA AAATGCAAAA   
  
  
- ATTTAATAAT AAAAAAACAA AAACAAAAAA AGAAACACAA AAAAAATAAA ATTACTTAAA AAAAACAAAT   
  
  
- TAAATCAAAC AATTACAAAT TAAAAAAAAT AAATCAATAG TATAAAAGTA TTATGCCTAG GGCTCAAACT   
  
  
- ACTCAATTAG GTCTTAAAAA CGAAAAGAAG AAAAATTAAT TAAAAAAAAA CAAATCAAAT CAAACAATTA   
  
  
- CAATTTAAAA AAAATAAATT AATAGTCTGA AAATACTGTG CATAGAGCCC AAACTGCCCA ATTGCGCAGT   
  
  
- TAAAAAAAAA GATAAATCAA TAGTTTAAAA ATACTGCGCT TAAAGTCCAA ACTGTCCAAT TGGACCAAAC   
  
  
- TTCCCAATTG GGTAAATTAA GTTAAAAAAA AGAAAAAGAA GTAATCAAAA AAAGAAGGTA AATTAAAAAA   
  
  
- AAGAAAAAGA AGTAATTACA ATAATAATTA TAATATTTAT AGTGAGAACC TAGTCCGCAA CGTCGATTTA   
  
  
- GATTTTGAGT ATCCATATCG AAATATCTTT AAACTTAGAA TCAAAAAAAA ATATAAAAAA TATGCTTTTT   
  
  
- TAATTGGGCA CGTCCATTAC ACTGATCATT ATTGTAATAA AAGACAACTT GGCTTGTTCT TTCTTAGCAT   
  
  
- CAATCCACAG TCTCTGAGTC TCAGCGACGA AGAGTGTAAG TTTGGAACAT TCGCAAACCA GTAACATTCT   
  
  
- GGGAGAGAAA GAAAATAGTT TATTGGAAAG AAATAAAGAA TTTATTTAAT TCCTGGGAAG AAAAAAATAT   
  
  
- TTTTTATTTT TATTTTTTAA GAAGGAAGAA GGGTATTAAC TAAGTTAGCG TTTACTCAGT ACAGTACTGG   
  
  
- GCCTCTGGGA AACTTTTCAG TTTAATCTAT TTCTAACTCT GCTACCAAAG TTCTCTGTGT TGGTGAGTTC   
  
  
- ATAAATGGTA ACACTACTCG ACTGTTCCTA ACAGTAGGTC TTACCAGAGA GGAGTGCTAC AGTGGTCGGT   
  
  
- CACCTTAGGT TTAATTTAAC TTAAATAACC CTTTACCCTA TTGAGAAGGA AAATATATAA TTTTTAATTT   
  
  
- TTTATATATG TCACGGTTAA GATGACATGA CCAATTAATG GGATAGTTGT AAATTCATCA AATACATTGC   
  
  
- AACATAATCA ACAAAAAATT TTATTAAAAA AAAATTATAT AATTTTATTA AAAAAATTTT TTAAATAAAA   
  
  
- ACTATAATTA TACAATTTTG TTAAATTTTT GTAACTTTTT TTTATTTTAT TTTATTTAAG TTCAAAACGT   
  
  
- GTTTTTTTGT GATAACGTGG CGCATGTATT TACTACAGAA TCACATAAAT CGTAACACTA TCGAAAACAC   
  
  
- TAGTAAGACC AAAATTTTTT TGTTTAAATT TTCTTTATAA AAATCAACAC CAAAATTTTT CATCTAAAAT   
  
  
- TTTTTATGTA CAAACTAATT TAGATGATAC TCTATTTAAA AATGTACATT TTATTTATTT TTGTATAAAG   
  
  
- GTAGTTAATT TTTTTTTGTA CAAGAAAGTC AAAGATGTAC TGATTATAAA TTTTATGTTA ATGAATATTT   
  
  
- CAGGCCATGC TTTCGTGGGA GATTTACCTC TTACTTGACA TACATAAGAA GGGTACCTGG ACACTAAAAA   
  
  
- GGCATCCTTT CCGTTAAAGG TAAGTCTTAT GTTCTTCTAT GTACAGAAAA CTTTGAGAAT AATCGACAGA   
  
  
- TGGATAGTTG GTTCTTACTG CGAAGAGTAG CATACAGTGA TATTTTAAAC CAACTACTGT GGTTGTGAAA   
  
  
- GGGTAACCAT AAGTACCGAA AAATCGACGT GCTAGGTTG

+     AT~TATA-box

| Site Name | Organism | Position | Strand | Matrix score. | sequence | function |
| --- | --- | --- | --- | --- | --- | --- |
| AT~TATA-box | Arabidopsis thaliana | 1264 | - | 6 | TATATA |  |
| AT~TATA-box | Arabidopsis thaliana | 1243 | - | 6 | TATATA |  |
| AT~TATA-box | Arabidopsis thaliana | 1241 | - | 8 | TATATAAA |  |
| AT~TATA-box | Arabidopsis thaliana | 132 | + | 6 | TATATA |  |

>Potri.004G051700.1   
+ TTATCAAACT TTCATGATGT GAATCTCAGG TTTGACGGGT TAACTTGGTT TGAAGGGTTA ACCCAGTTAA   
  
  
+ TTCAAATTTT TTTTTTCTTC ATTAGTTTTT TTTTCTTCCT GTTGATTTTT TTCATTGAAC TTATATATTT   
  
  
+ TAATTATAAT AATATAATAA TAATTACATA TACTAAAATA CTGAGTTGTC TTCTTTTTTT TTTACGTTTT   
  
  
+ TAAATTATTA TTTTTTTGTT TTTGTTTTTT TCTTTGTGTT TTTTTTATTT TAATGAATTT TTTTTGTTTA   
  
  
+ ATTTAGTTTG TTAATGTTTA ATTTTTTTTA TTTAGTTATC ATATTTTCAT AATACGGATC CCGAGTTTGA   
  
  
+ TGAGTTAATC CAGAATTTTT GCTTTTCTTC TTTTTAATTA ATTTTTTTTT GTTTAGTTTA GTTTGTTAAT   
  
  
+ GTTAAATTTT TTTTATTTAA TTATCAGACT TTTATGACAC GTATCTCGGG TTTGACGGGT TAACGCGTCA   
  
  
+ ATTTTTTTTT CTATTTAGTT ATCAAATTTT TATGACGCGA ATTTCAGGTT TGACAGGTTA ACCTGGTTTG   
  
  
+ AAGGGTTAAC CCATTTAATT CAATTTTTTT TCTTTTTCTT CATTAGTTTT TTTCTTCCAT TTAATTTTTT   
  
  
+ TTCTTTTTCT TCATTAATGT TATTATTAAT ATTATAAATA TCACTCTTGG ATCAGGCGTT GCAGCTAAAT   
  
  
+ CTAAAACTCA TAGGTATAGC TTTATAGAAA TTTGAATCTT AGTTTTTTTT TATATTTTTT ATACGAAAAA   
  
  
+ ATTAACCCGT GCAGGTAATG TGACTAGTAA TAACATTATT TTCTGTTGAA CCGAACAAGA AAGAATCGTA   
  
  
+ GTTAGGTGTC AGAGACTCAG AGTCGCTGCT TCTCACATTC AAACCTTGTA AGCGTTTGGT CATTGTAAGA   
  
  
+ CCCTCTCTTT CTTTTATCAA ATAACCTTTC TTTATTTCTT AAATAAATTA AGGACCCTTC TTTTTTTATA   
  
  
+ AAAAATAAAA ATAAAAAATT CTTCCTTCTT CCCATAATTG ATTCAATCGC AAATGAGTCA TGTCATGACC   
  
  
+ CGGAGACCCT TTGAAAAGTC AAATTAGATA AAGATTGAGA CGATGGTTTC AAGAGACACA ACCACTCAAG   
  
  
+ TATTTACCAT TGTGATGAGC TGACAAGGAT TGTCATCCAG AATGGTCTCT CCTCACGATG TCACCAGCCA   
  
  
+ GTGGAATCCA AATTAAATTG AATTTATTGG GAAATGGGAT AACTCTTCCT TTTATATATT AAAAATTAAA   
  
  
+ AAATATATAC AGTGCCAATT CTACTGTACT GGTTAATTAC CCTATCAACA TTTAAGTAGT TTATGTAACG   
  
  
+ TTGTATTAGT TGTTTTTTAA AATAATTTTT TTTTAATATA TTAAAATAAT TTTTTTAAAA AATTTATTTT   
  
  
+ TGATATTAAT ATGTTAAAAC AATTTAAAAA CATTGAAAAA AAATAAAATA AAATAAATTC AAGTTTTGCA   
  
  
+ CAAAAAAACA CTATTGCACC GCGTACATAA ATGATGTCTT AGTGTATTTA GCATTGTGAT AGCTTTTGTG   
  
  
+ ATCATTCTGG TTTTAAAAAA ACAAATTTAA AAGAAATATT TTTAGTTGTG GTTTTAAAAA GTAGATTTTA   
  
  
+ AAAAATACAT GTTTGATTAA ATCTACTATG AGATAAATTT TTACATGTAA AATAAATAAA AACATATTTC   
  
  
+ CATCAATTAA AAAAAAACAT GTTCTTTCAG TTTCTACATG ACTAATATTT AAAATACAAT TACTTATAAA   
  
  
+ GTCCGGTACG AAAGCACCCT CTAAATGGAG AATGAACTGT ATGTATTCTT CCCATGGACC TGTGATTTTT   
  
  
+ CCGTAGGAAA GGCAATTTCC ATTCAGAATA CAAGAAGATA CATGTCTTTT GAAACTCTTA TTAGCTGTCT   
  
  
+ ACCTATCAAC CAAGAATGAC GCTTCTCATC GTATGTCACT ATAAAATTTG GTTGATGACA CCAACACTTT   
  
  
+ CCCATTGGTA TTCATGGCTT TTTAGCTGCA CGATCCAAC  

- AATAGTTTGA AAGTACTACA CTTAGAGTCC AAACTGCCCA ATTGAACCAA ACTTCCCAAT TGGGTCAATT   
  
  
- AAGTTTAAAA AAAAAAGAAG TAATCAAAAA AAAAGAAGGA CAACTAAAAA AAGTAACTTG AATATATAAA   
  
  
- ATTAATATTA TTATATTATT ATTAATGTAT ATGATTTTAT GACTCAACAG AAGAAAAAAA AAATGCAAAA   
  
  
- ATTTAATAAT AAAAAAACAA AAACAAAAAA AGAAACACAA AAAAAATAAA ATTACTTAAA AAAAACAAAT   
  
  
- TAAATCAAAC AATTACAAAT TAAAAAAAAT AAATCAATAG TATAAAAGTA TTATGCCTAG GGCTCAAACT   
  
  
- ACTCAATTAG GTCTTAAAAA CGAAAAGAAG AAAAATTAAT TAAAAAAAAA CAAATCAAAT CAAACAATTA   
  
  
- CAATTTAAAA AAAATAAATT AATAGTCTGA AAATACTGTG CATAGAGCCC AAACTGCCCA ATTGCGCAGT   
  
  
- TAAAAAAAAA GATAAATCAA TAGTTTAAAA ATACTGCGCT TAAAGTCCAA ACTGTCCAAT TGGACCAAAC   
  
  
- TTCCCAATTG GGTAAATTAA GTTAAAAAAA AGAAAAAGAA GTAATCAAAA AAAGAAGGTA AATTAAAAAA   
  
  
- AAGAAAAAGA AGTAATTACA ATAATAATTA TAATATTTAT AGTGAGAACC TAGTCCGCAA CGTCGATTTA   
  
  
- GATTTTGAGT ATCCATATCG AAATATCTTT AAACTTAGAA TCAAAAAAAA ATATAAAAAA TATGCTTTTT   
  
  
- TAATTGGGCA CGTCCATTAC ACTGATCATT ATTGTAATAA AAGACAACTT GGCTTGTTCT TTCTTAGCAT   
  
  
- CAATCCACAG TCTCTGAGTC TCAGCGACGA AGAGTGTAAG TTTGGAACAT TCGCAAACCA GTAACATTCT   
  
  
- GGGAGAGAAA GAAAATAGTT TATTGGAAAG AAATAAAGAA TTTATTTAAT TCCTGGGAAG AAAAAAATAT   
  
  
- TTTTTATTTT TATTTTTTAA GAAGGAAGAA GGGTATTAAC TAAGTTAGCG TTTACTCAGT ACAGTACTGG   
  
  
- GCCTCTGGGA AACTTTTCAG TTTAATCTAT TTCTAACTCT GCTACCAAAG TTCTCTGTGT TGGTGAGTTC   
  
  
- ATAAATGGTA ACACTACTCG ACTGTTCCTA ACAGTAGGTC TTACCAGAGA GGAGTGCTAC AGTGGTCGGT   
  
  
- CACCTTAGGT TTAATTTAAC TTAAATAACC CTTTACCCTA TTGAGAAGGA AAATATATAA TTTTTAATTT   
  
  
- TTTATATATG TCACGGTTAA GATGACATGA CCAATTAATG GGATAGTTGT AAATTCATCA AATACATTGC   
  
  
- AACATAATCA ACAAAAAATT TTATTAAAAA AAAATTATAT AATTTTATTA AAAAAATTTT TTAAATAAAA   
  
  
- ACTATAATTA TACAATTTTG TTAAATTTTT GTAACTTTTT TTTATTTTAT TTTATTTAAG TTCAAAACGT   
  
  
- GTTTTTTTGT GATAACGTGG CGCATGTATT TACTACAGAA TCACATAAAT CGTAACACTA TCGAAAACAC   
  
  
- TAGTAAGACC AAAATTTTTT TGTTTAAATT TTCTTTATAA AAATCAACAC CAAAATTTTT CATCTAAAAT   
  
  
- TTTTTATGTA CAAACTAATT TAGATGATAC TCTATTTAAA AATGTACATT TTATTTATTT TTGTATAAAG   
  
  
- GTAGTTAATT TTTTTTTGTA CAAGAAAGTC AAAGATGTAC TGATTATAAA TTTTATGTTA ATGAATATTT   
  
  
- CAGGCCATGC TTTCGTGGGA GATTTACCTC TTACTTGACA TACATAAGAA GGGTACCTGG ACACTAAAAA   
  
  
- GGCATCCTTT CCGTTAAAGG TAAGTCTTAT GTTCTTCTAT GTACAGAAAA CTTTGAGAAT AATCGACAGA   
  
  
- TGGATAGTTG GTTCTTACTG CGAAGAGTAG CATACAGTGA TATTTTAAAC CAACTACTGT GGTTGTGAAA   
  
  
- GGGTAACCAT AAGTACCGAA AAATCGACGT GCTAGGTTG

+     AuxRR-core

| Site Name | Organism | Position | Strand | Matrix score. | sequence | function |
| --- | --- | --- | --- | --- | --- | --- |
| AuxRR-core | Nicotiana tabacum | 1804 | - | 7 | GGTCCAT | cis-acting regulatory element involved in auxin responsiveness |

>Potri.004G051700.1   
+ TTATCAAACT TTCATGATGT GAATCTCAGG TTTGACGGGT TAACTTGGTT TGAAGGGTTA ACCCAGTTAA   
  
  
+ TTCAAATTTT TTTTTTCTTC ATTAGTTTTT TTTTCTTCCT GTTGATTTTT TTCATTGAAC TTATATATTT   
  
  
+ TAATTATAAT AATATAATAA TAATTACATA TACTAAAATA CTGAGTTGTC TTCTTTTTTT TTTACGTTTT   
  
  
+ TAAATTATTA TTTTTTTGTT TTTGTTTTTT TCTTTGTGTT TTTTTTATTT TAATGAATTT TTTTTGTTTA   
  
  
+ ATTTAGTTTG TTAATGTTTA ATTTTTTTTA TTTAGTTATC ATATTTTCAT AATACGGATC CCGAGTTTGA   
  
  
+ TGAGTTAATC CAGAATTTTT GCTTTTCTTC TTTTTAATTA ATTTTTTTTT GTTTAGTTTA GTTTGTTAAT   
  
  
+ GTTAAATTTT TTTTATTTAA TTATCAGACT TTTATGACAC GTATCTCGGG TTTGACGGGT TAACGCGTCA   
  
  
+ ATTTTTTTTT CTATTTAGTT ATCAAATTTT TATGACGCGA ATTTCAGGTT TGACAGGTTA ACCTGGTTTG   
  
  
+ AAGGGTTAAC CCATTTAATT CAATTTTTTT TCTTTTTCTT CATTAGTTTT TTTCTTCCAT TTAATTTTTT   
  
  
+ TTCTTTTTCT TCATTAATGT TATTATTAAT ATTATAAATA TCACTCTTGG ATCAGGCGTT GCAGCTAAAT   
  
  
+ CTAAAACTCA TAGGTATAGC TTTATAGAAA TTTGAATCTT AGTTTTTTTT TATATTTTTT ATACGAAAAA   
  
  
+ ATTAACCCGT GCAGGTAATG TGACTAGTAA TAACATTATT TTCTGTTGAA CCGAACAAGA AAGAATCGTA   
  
  
+ GTTAGGTGTC AGAGACTCAG AGTCGCTGCT TCTCACATTC AAACCTTGTA AGCGTTTGGT CATTGTAAGA   
  
  
+ CCCTCTCTTT CTTTTATCAA ATAACCTTTC TTTATTTCTT AAATAAATTA AGGACCCTTC TTTTTTTATA   
  
  
+ AAAAATAAAA ATAAAAAATT CTTCCTTCTT CCCATAATTG ATTCAATCGC AAATGAGTCA TGTCATGACC   
  
  
+ CGGAGACCCT TTGAAAAGTC AAATTAGATA AAGATTGAGA CGATGGTTTC AAGAGACACA ACCACTCAAG   
  
  
+ TATTTACCAT TGTGATGAGC TGACAAGGAT TGTCATCCAG AATGGTCTCT CCTCACGATG TCACCAGCCA   
  
  
+ GTGGAATCCA AATTAAATTG AATTTATTGG GAAATGGGAT AACTCTTCCT TTTATATATT AAAAATTAAA   
  
  
+ AAATATATAC AGTGCCAATT CTACTGTACT GGTTAATTAC CCTATCAACA TTTAAGTAGT TTATGTAACG   
  
  
+ TTGTATTAGT TGTTTTTTAA AATAATTTTT TTTTAATATA TTAAAATAAT TTTTTTAAAA AATTTATTTT   
  
  
+ TGATATTAAT ATGTTAAAAC AATTTAAAAA CATTGAAAAA AAATAAAATA AAATAAATTC AAGTTTTGCA   
  
  
+ CAAAAAAACA CTATTGCACC GCGTACATAA ATGATGTCTT AGTGTATTTA GCATTGTGAT AGCTTTTGTG   
  
  
+ ATCATTCTGG TTTTAAAAAA ACAAATTTAA AAGAAATATT TTTAGTTGTG GTTTTAAAAA GTAGATTTTA   
  
  
+ AAAAATACAT GTTTGATTAA ATCTACTATG AGATAAATTT TTACATGTAA AATAAATAAA AACATATTTC   
  
  
+ CATCAATTAA AAAAAAACAT GTTCTTTCAG TTTCTACATG ACTAATATTT AAAATACAAT TACTTATAAA   
  
  
+ GTCCGGTACG AAAGCACCCT CTAAATGGAG AATGAACTGT ATGTATTCTT CCCATGGACC TGTGATTTTT   
  
  
+ CCGTAGGAAA GGCAATTTCC ATTCAGAATA CAAGAAGATA CATGTCTTTT GAAACTCTTA TTAGCTGTCT   
  
  
+ ACCTATCAAC CAAGAATGAC GCTTCTCATC GTATGTCACT ATAAAATTTG GTTGATGACA CCAACACTTT   
  
  
+ CCCATTGGTA TTCATGGCTT TTTAGCTGCA CGATCCAAC  

- AATAGTTTGA AAGTACTACA CTTAGAGTCC AAACTGCCCA ATTGAACCAA ACTTCCCAAT TGGGTCAATT   
  
  
- AAGTTTAAAA AAAAAAGAAG TAATCAAAAA AAAAGAAGGA CAACTAAAAA AAGTAACTTG AATATATAAA   
  
  
- ATTAATATTA TTATATTATT ATTAATGTAT ATGATTTTAT GACTCAACAG AAGAAAAAAA AAATGCAAAA   
  
  
- ATTTAATAAT AAAAAAACAA AAACAAAAAA AGAAACACAA AAAAAATAAA ATTACTTAAA AAAAACAAAT   
  
  
- TAAATCAAAC AATTACAAAT TAAAAAAAAT AAATCAATAG TATAAAAGTA TTATGCCTAG GGCTCAAACT   
  
  
- ACTCAATTAG GTCTTAAAAA CGAAAAGAAG AAAAATTAAT TAAAAAAAAA CAAATCAAAT CAAACAATTA   
  
  
- CAATTTAAAA AAAATAAATT AATAGTCTGA AAATACTGTG CATAGAGCCC AAACTGCCCA ATTGCGCAGT   
  
  
- TAAAAAAAAA GATAAATCAA TAGTTTAAAA ATACTGCGCT TAAAGTCCAA ACTGTCCAAT TGGACCAAAC   
  
  
- TTCCCAATTG GGTAAATTAA GTTAAAAAAA AGAAAAAGAA GTAATCAAAA AAAGAAGGTA AATTAAAAAA   
  
  
- AAGAAAAAGA AGTAATTACA ATAATAATTA TAATATTTAT AGTGAGAACC TAGTCCGCAA CGTCGATTTA   
  
  
- GATTTTGAGT ATCCATATCG AAATATCTTT AAACTTAGAA TCAAAAAAAA ATATAAAAAA TATGCTTTTT   
  
  
- TAATTGGGCA CGTCCATTAC ACTGATCATT ATTGTAATAA AAGACAACTT GGCTTGTTCT TTCTTAGCAT   
  
  
- CAATCCACAG TCTCTGAGTC TCAGCGACGA AGAGTGTAAG TTTGGAACAT TCGCAAACCA GTAACATTCT   
  
  
- GGGAGAGAAA GAAAATAGTT TATTGGAAAG AAATAAAGAA TTTATTTAAT TCCTGGGAAG AAAAAAATAT   
  
  
- TTTTTATTTT TATTTTTTAA GAAGGAAGAA GGGTATTAAC TAAGTTAGCG TTTACTCAGT ACAGTACTGG   
  
  
- GCCTCTGGGA AACTTTTCAG TTTAATCTAT TTCTAACTCT GCTACCAAAG TTCTCTGTGT TGGTGAGTTC   
  
  
- ATAAATGGTA ACACTACTCG ACTGTTCCTA ACAGTAGGTC TTACCAGAGA GGAGTGCTAC AGTGGTCGGT   
  
  
- CACCTTAGGT TTAATTTAAC TTAAATAACC CTTTACCCTA TTGAGAAGGA AAATATATAA TTTTTAATTT   
  
  
- TTTATATATG TCACGGTTAA GATGACATGA CCAATTAATG GGATAGTTGT AAATTCATCA AATACATTGC   
  
  
- AACATAATCA ACAAAAAATT TTATTAAAAA AAAATTATAT AATTTTATTA AAAAAATTTT TTAAATAAAA   
  
  
- ACTATAATTA TACAATTTTG TTAAATTTTT GTAACTTTTT TTTATTTTAT TTTATTTAAG TTCAAAACGT   
  
  
- GTTTTTTTGT GATAACGTGG CGCATGTATT TACTACAGAA TCACATAAAT CGTAACACTA TCGAAAACAC   
  
  
- TAGTAAGACC AAAATTTTTT TGTTTAAATT TTCTTTATAA AAATCAACAC CAAAATTTTT CATCTAAAAT   
  
  
- TTTTTATGTA CAAACTAATT TAGATGATAC TCTATTTAAA AATGTACATT TTATTTATTT TTGTATAAAG   
  
  
- GTAGTTAATT TTTTTTTGTA CAAGAAAGTC AAAGATGTAC TGATTATAAA TTTTATGTTA ATGAATATTT   
  
  
- CAGGCCATGC TTTCGTGGGA GATTTACCTC TTACTTGACA TACATAAGAA GGGTACCTGG ACACTAAAAA   
  
  
- GGCATCCTTT CCGTTAAAGG TAAGTCTTAT GTTCTTCTAT GTACAGAAAA CTTTGAGAAT AATCGACAGA   
  
  
- TGGATAGTTG GTTCTTACTG CGAAGAGTAG CATACAGTGA TATTTTAAAC CAACTACTGT GGTTGTGAAA   
  
  
- GGGTAACCAT AAGTACCGAA AAATCGACGT GCTAGGTTG

+     Box 4

| Site Name | Organism | Position | Strand | Matrix score. | sequence | function |
| --- | --- | --- | --- | --- | --- | --- |
| Box 4 | Petroselinum crispum | 387 | + | 6 | ATTAAT | part of a conserved DNA module involved in light responsiveness |
| Box 4 | Petroselinum crispum | 655 | + | 6 | ATTAAT | part of a conserved DNA module involved in light responsiveness |
| Box 4 | Petroselinum crispum | 643 | + | 6 | ATTAAT | part of a conserved DNA module involved in light responsiveness |
| Box 4 | Petroselinum crispum | 1405 | - | 6 | ATTAAT | part of a conserved DNA module involved in light responsiveness |

>Potri.004G051700.1   
+ TTATCAAACT TTCATGATGT GAATCTCAGG TTTGACGGGT TAACTTGGTT TGAAGGGTTA ACCCAGTTAA   
  
  
+ TTCAAATTTT TTTTTTCTTC ATTAGTTTTT TTTTCTTCCT GTTGATTTTT TTCATTGAAC TTATATATTT   
  
  
+ TAATTATAAT AATATAATAA TAATTACATA TACTAAAATA CTGAGTTGTC TTCTTTTTTT TTTACGTTTT   
  
  
+ TAAATTATTA TTTTTTTGTT TTTGTTTTTT TCTTTGTGTT TTTTTTATTT TAATGAATTT TTTTTGTTTA   
  
  
+ ATTTAGTTTG TTAATGTTTA ATTTTTTTTA TTTAGTTATC ATATTTTCAT AATACGGATC CCGAGTTTGA   
  
  
+ TGAGTTAATC CAGAATTTTT GCTTTTCTTC TTTTTAATTA ATTTTTTTTT GTTTAGTTTA GTTTGTTAAT   
  
  
+ GTTAAATTTT TTTTATTTAA TTATCAGACT TTTATGACAC GTATCTCGGG TTTGACGGGT TAACGCGTCA   
  
  
+ ATTTTTTTTT CTATTTAGTT ATCAAATTTT TATGACGCGA ATTTCAGGTT TGACAGGTTA ACCTGGTTTG   
  
  
+ AAGGGTTAAC CCATTTAATT CAATTTTTTT TCTTTTTCTT CATTAGTTTT TTTCTTCCAT TTAATTTTTT   
  
  
+ TTCTTTTTCT TCATTAATGT TATTATTAAT ATTATAAATA TCACTCTTGG ATCAGGCGTT GCAGCTAAAT   
  
  
+ CTAAAACTCA TAGGTATAGC TTTATAGAAA TTTGAATCTT AGTTTTTTTT TATATTTTTT ATACGAAAAA   
  
  
+ ATTAACCCGT GCAGGTAATG TGACTAGTAA TAACATTATT TTCTGTTGAA CCGAACAAGA AAGAATCGTA   
  
  
+ GTTAGGTGTC AGAGACTCAG AGTCGCTGCT TCTCACATTC AAACCTTGTA AGCGTTTGGT CATTGTAAGA   
  
  
+ CCCTCTCTTT CTTTTATCAA ATAACCTTTC TTTATTTCTT AAATAAATTA AGGACCCTTC TTTTTTTATA   
  
  
+ AAAAATAAAA ATAAAAAATT CTTCCTTCTT CCCATAATTG ATTCAATCGC AAATGAGTCA TGTCATGACC   
  
  
+ CGGAGACCCT TTGAAAAGTC AAATTAGATA AAGATTGAGA CGATGGTTTC AAGAGACACA ACCACTCAAG   
  
  
+ TATTTACCAT TGTGATGAGC TGACAAGGAT TGTCATCCAG AATGGTCTCT CCTCACGATG TCACCAGCCA   
  
  
+ GTGGAATCCA AATTAAATTG AATTTATTGG GAAATGGGAT AACTCTTCCT TTTATATATT AAAAATTAAA   
  
  
+ AAATATATAC AGTGCCAATT CTACTGTACT GGTTAATTAC CCTATCAACA TTTAAGTAGT TTATGTAACG   
  
  
+ TTGTATTAGT TGTTTTTTAA AATAATTTTT TTTTAATATA TTAAAATAAT TTTTTTAAAA AATTTATTTT   
  
  
+ TGATATTAAT ATGTTAAAAC AATTTAAAAA CATTGAAAAA AAATAAAATA AAATAAATTC AAGTTTTGCA   
  
  
+ CAAAAAAACA CTATTGCACC GCGTACATAA ATGATGTCTT AGTGTATTTA GCATTGTGAT AGCTTTTGTG   
  
  
+ ATCATTCTGG TTTTAAAAAA ACAAATTTAA AAGAAATATT TTTAGTTGTG GTTTTAAAAA GTAGATTTTA   
  
  
+ AAAAATACAT GTTTGATTAA ATCTACTATG AGATAAATTT TTACATGTAA AATAAATAAA AACATATTTC   
  
  
+ CATCAATTAA AAAAAAACAT GTTCTTTCAG TTTCTACATG ACTAATATTT AAAATACAAT TACTTATAAA   
  
  
+ GTCCGGTACG AAAGCACCCT CTAAATGGAG AATGAACTGT ATGTATTCTT CCCATGGACC TGTGATTTTT   
  
  
+ CCGTAGGAAA GGCAATTTCC ATTCAGAATA CAAGAAGATA CATGTCTTTT GAAACTCTTA TTAGCTGTCT   
  
  
+ ACCTATCAAC CAAGAATGAC GCTTCTCATC GTATGTCACT ATAAAATTTG GTTGATGACA CCAACACTTT   
  
  
+ CCCATTGGTA TTCATGGCTT TTTAGCTGCA CGATCCAAC  

- AATAGTTTGA AAGTACTACA CTTAGAGTCC AAACTGCCCA ATTGAACCAA ACTTCCCAAT TGGGTCAATT   
  
  
- AAGTTTAAAA AAAAAAGAAG TAATCAAAAA AAAAGAAGGA CAACTAAAAA AAGTAACTTG AATATATAAA   
  
  
- ATTAATATTA TTATATTATT ATTAATGTAT ATGATTTTAT GACTCAACAG AAGAAAAAAA AAATGCAAAA   
  
  
- ATTTAATAAT AAAAAAACAA AAACAAAAAA AGAAACACAA AAAAAATAAA ATTACTTAAA AAAAACAAAT   
  
  
- TAAATCAAAC AATTACAAAT TAAAAAAAAT AAATCAATAG TATAAAAGTA TTATGCCTAG GGCTCAAACT   
  
  
- ACTCAATTAG GTCTTAAAAA CGAAAAGAAG AAAAATTAAT TAAAAAAAAA CAAATCAAAT CAAACAATTA   
  
  
- CAATTTAAAA AAAATAAATT AATAGTCTGA AAATACTGTG CATAGAGCCC AAACTGCCCA ATTGCGCAGT   
  
  
- TAAAAAAAAA GATAAATCAA TAGTTTAAAA ATACTGCGCT TAAAGTCCAA ACTGTCCAAT TGGACCAAAC   
  
  
- TTCCCAATTG GGTAAATTAA GTTAAAAAAA AGAAAAAGAA GTAATCAAAA AAAGAAGGTA AATTAAAAAA   
  
  
- AAGAAAAAGA AGTAATTACA ATAATAATTA TAATATTTAT AGTGAGAACC TAGTCCGCAA CGTCGATTTA   
  
  
- GATTTTGAGT ATCCATATCG AAATATCTTT AAACTTAGAA TCAAAAAAAA ATATAAAAAA TATGCTTTTT   
  
  
- TAATTGGGCA CGTCCATTAC ACTGATCATT ATTGTAATAA AAGACAACTT GGCTTGTTCT TTCTTAGCAT   
  
  
- CAATCCACAG TCTCTGAGTC TCAGCGACGA AGAGTGTAAG TTTGGAACAT TCGCAAACCA GTAACATTCT   
  
  
- GGGAGAGAAA GAAAATAGTT TATTGGAAAG AAATAAAGAA TTTATTTAAT TCCTGGGAAG AAAAAAATAT   
  
  
- TTTTTATTTT TATTTTTTAA GAAGGAAGAA GGGTATTAAC TAAGTTAGCG TTTACTCAGT ACAGTACTGG   
  
  
- GCCTCTGGGA AACTTTTCAG TTTAATCTAT TTCTAACTCT GCTACCAAAG TTCTCTGTGT TGGTGAGTTC   
  
  
- ATAAATGGTA ACACTACTCG ACTGTTCCTA ACAGTAGGTC TTACCAGAGA GGAGTGCTAC AGTGGTCGGT   
  
  
- CACCTTAGGT TTAATTTAAC TTAAATAACC CTTTACCCTA TTGAGAAGGA AAATATATAA TTTTTAATTT   
  
  
- TTTATATATG TCACGGTTAA GATGACATGA CCAATTAATG GGATAGTTGT AAATTCATCA AATACATTGC   
  
  
- AACATAATCA ACAAAAAATT TTATTAAAAA AAAATTATAT AATTTTATTA AAAAAATTTT TTAAATAAAA   
  
  
- ACTATAATTA TACAATTTTG TTAAATTTTT GTAACTTTTT TTTATTTTAT TTTATTTAAG TTCAAAACGT   
  
  
- GTTTTTTTGT GATAACGTGG CGCATGTATT TACTACAGAA TCACATAAAT CGTAACACTA TCGAAAACAC   
  
  
- TAGTAAGACC AAAATTTTTT TGTTTAAATT TTCTTTATAA AAATCAACAC CAAAATTTTT CATCTAAAAT   
  
  
- TTTTTATGTA CAAACTAATT TAGATGATAC TCTATTTAAA AATGTACATT TTATTTATTT TTGTATAAAG   
  
  
- GTAGTTAATT TTTTTTTGTA CAAGAAAGTC AAAGATGTAC TGATTATAAA TTTTATGTTA ATGAATATTT   
  
  
- CAGGCCATGC TTTCGTGGGA GATTTACCTC TTACTTGACA TACATAAGAA GGGTACCTGG ACACTAAAAA   
  
  
- GGCATCCTTT CCGTTAAAGG TAAGTCTTAT GTTCTTCTAT GTACAGAAAA CTTTGAGAAT AATCGACAGA   
  
  
- TGGATAGTTG GTTCTTACTG CGAAGAGTAG CATACAGTGA TATTTTAAAC CAACTACTGT GGTTGTGAAA   
  
  
- GGGTAACCAT AAGTACCGAA AAATCGACGT GCTAGGTTG

+     CAAT-box

| Site Name | Organism | Position | Strand | Matrix score. | sequence | function |
| --- | --- | --- | --- | --- | --- | --- |
| CAAT-box | Pisum sativum | 1936 | - | 5 | CAAAT | common cis-acting element in promoter and enhancer regions |
| CAAT-box | Arabidopsis thaliana | 1964 | - | 5 | CCAAT | common cis-acting element in promoter and enhancer regions |
| CAAT-box | Nicotiana glutinosa | 1737 | + | 4 | CAAT |  |
| CAAT-box | Nicotiana glutinosa | 1833 | + | 4 | CAAT |  |
| CAAT-box | Pisum sativum | 1562 | + | 5 | CAAAT | common cis-acting element in promoter and enhancer regions |
| CAAT-box | Nicotiana glutinosa | 1684 | + | 4 | CAAT |  |
| CAAT-box | Nicotiana glutinosa | 1483 | - | 4 | CAAT |  |
| CAAT-box | Nicotiana glutinosa | 1276 | + | 4 | CAAT |  |
| CAAT-box | Nicotiana glutinosa | 1523 | - | 4 | CAAT |  |
| CAAT-box | Nicotiana glutinosa | 1432 | - | 4 | CAAT |  |
| CAAT-box | Nicotiana glutinosa | 1420 | + | 4 | CAAT |  |
| CAAT-box | Arabidopsis thaliana | 1275 | + | 5 | CCAAT | common cis-acting element in promoter and enhancer regions |
| CAAT-box | Nicotiana glutinosa | 1149 | - | 4 | CAAT |  |
| CAAT-box | Arabidopsis thaliana | 1216 | - | 5 | CCAAT | common cis-acting element in promoter and enhancer regions |
| CAAT-box | Nicotiana glutinosa | 1207 | - | 4 | CAAT |  |
| CAAT-box | Nicotiana glutinosa | 1129 | - | 4 | CAAT |  |
| CAAT-box | Pisum sativum | 1199 | + | 5 | CAAAT | common cis-acting element in promoter and enhancer regions |
| CAAT-box | Nicotiana glutinosa | 1084 | - | 4 | CAAT |  |
| CAAT-box | Nicotiana glutinosa | 1017 | - | 4 | CAAT |  |
| CAAT-box | Pisum sativum | 1030 | + | 5 | CAAAT | common cis-acting element in promoter and enhancer regions |
| CAAT-box | Nicotiana glutinosa | 1024 | + | 4 | CAAT |  |
| CAAT-box | Pisum sativum | 1070 | + | 5 | CAAAT | common cis-acting element in promoter and enhancer regions |
| CAAT-box | Nicotiana glutinosa | 581 | + | 4 | CAAT |  |
| CAAT-box | Pisum sativum | 73 | + | 5 | CAAAT | common cis-acting element in promoter and enhancer regions |
| CAAT-box | Nicotiana glutinosa | 124 | - | 4 | CAAT |  |
| CAAT-box | Pisum sativum | 730 | - | 5 | CAAAT | common cis-acting element in promoter and enhancer regions |
| CAAT-box | Pisum sativum | 513 | + | 5 | CAAAT | common cis-acting element in promoter and enhancer regions |
| CAAT-box | Nicotiana glutinosa | 489 | + | 4 | CAAT |  |
| CAAT-box | Nicotiana glutinosa | 902 | - | 4 | CAAT |  |
| CAAT-box | Pisum sativum | 928 | + | 5 | CAAAT | common cis-acting element in promoter and enhancer regions |

>Potri.004G051700.1   
+ TTATCAAACT TTCATGATGT GAATCTCAGG TTTGACGGGT TAACTTGGTT TGAAGGGTTA ACCCAGTTAA   
  
  
+ TTCAAATTTT TTTTTTCTTC ATTAGTTTTT TTTTCTTCCT GTTGATTTTT TTCATTGAAC TTATATATTT   
  
  
+ TAATTATAAT AATATAATAA TAATTACATA TACTAAAATA CTGAGTTGTC TTCTTTTTTT TTTACGTTTT   
  
  
+ TAAATTATTA TTTTTTTGTT TTTGTTTTTT TCTTTGTGTT TTTTTTATTT TAATGAATTT TTTTTGTTTA   
  
  
+ ATTTAGTTTG TTAATGTTTA ATTTTTTTTA TTTAGTTATC ATATTTTCAT AATACGGATC CCGAGTTTGA   
  
  
+ TGAGTTAATC CAGAATTTTT GCTTTTCTTC TTTTTAATTA ATTTTTTTTT GTTTAGTTTA GTTTGTTAAT   
  
  
+ GTTAAATTTT TTTTATTTAA TTATCAGACT TTTATGACAC GTATCTCGGG TTTGACGGGT TAACGCGTCA   
  
  
+ ATTTTTTTTT CTATTTAGTT ATCAAATTTT TATGACGCGA ATTTCAGGTT TGACAGGTTA ACCTGGTTTG   
  
  
+ AAGGGTTAAC CCATTTAATT CAATTTTTTT TCTTTTTCTT CATTAGTTTT TTTCTTCCAT TTAATTTTTT   
  
  
+ TTCTTTTTCT TCATTAATGT TATTATTAAT ATTATAAATA TCACTCTTGG ATCAGGCGTT GCAGCTAAAT   
  
  
+ CTAAAACTCA TAGGTATAGC TTTATAGAAA TTTGAATCTT AGTTTTTTTT TATATTTTTT ATACGAAAAA   
  
  
+ ATTAACCCGT GCAGGTAATG TGACTAGTAA TAACATTATT TTCTGTTGAA CCGAACAAGA AAGAATCGTA   
  
  
+ GTTAGGTGTC AGAGACTCAG AGTCGCTGCT TCTCACATTC AAACCTTGTA AGCGTTTGGT CATTGTAAGA   
  
  
+ CCCTCTCTTT CTTTTATCAA ATAACCTTTC TTTATTTCTT AAATAAATTA AGGACCCTTC TTTTTTTATA   
  
  
+ AAAAATAAAA ATAAAAAATT CTTCCTTCTT CCCATAATTG ATTCAATCGC AAATGAGTCA TGTCATGACC   
  
  
+ CGGAGACCCT TTGAAAAGTC AAATTAGATA AAGATTGAGA CGATGGTTTC AAGAGACACA ACCACTCAAG   
  
  
+ TATTTACCAT TGTGATGAGC TGACAAGGAT TGTCATCCAG AATGGTCTCT CCTCACGATG TCACCAGCCA   
  
  
+ GTGGAATCCA AATTAAATTG AATTTATTGG GAAATGGGAT AACTCTTCCT TTTATATATT AAAAATTAAA   
  
  
+ AAATATATAC AGTGCCAATT CTACTGTACT GGTTAATTAC CCTATCAACA TTTAAGTAGT TTATGTAACG   
  
  
+ TTGTATTAGT TGTTTTTTAA AATAATTTTT TTTTAATATA TTAAAATAAT TTTTTTAAAA AATTTATTTT   
  
  
+ TGATATTAAT ATGTTAAAAC AATTTAAAAA CATTGAAAAA AAATAAAATA AAATAAATTC AAGTTTTGCA   
  
  
+ CAAAAAAACA CTATTGCACC GCGTACATAA ATGATGTCTT AGTGTATTTA GCATTGTGAT AGCTTTTGTG   
  
  
+ ATCATTCTGG TTTTAAAAAA ACAAATTTAA AAGAAATATT TTTAGTTGTG GTTTTAAAAA GTAGATTTTA   
  
  
+ AAAAATACAT GTTTGATTAA ATCTACTATG AGATAAATTT TTACATGTAA AATAAATAAA AACATATTTC   
  
  
+ CATCAATTAA AAAAAAACAT GTTCTTTCAG TTTCTACATG ACTAATATTT AAAATACAAT TACTTATAAA   
  
  
+ GTCCGGTACG AAAGCACCCT CTAAATGGAG AATGAACTGT ATGTATTCTT CCCATGGACC TGTGATTTTT   
  
  
+ CCGTAGGAAA GGCAATTTCC ATTCAGAATA CAAGAAGATA CATGTCTTTT GAAACTCTTA TTAGCTGTCT   
  
  
+ ACCTATCAAC CAAGAATGAC GCTTCTCATC GTATGTCACT ATAAAATTTG GTTGATGACA CCAACACTTT   
  
  
+ CCCATTGGTA TTCATGGCTT TTTAGCTGCA CGATCCAAC  

- AATAGTTTGA AAGTACTACA CTTAGAGTCC AAACTGCCCA ATTGAACCAA ACTTCCCAAT TGGGTCAATT   
  
  
- AAGTTTAAAA AAAAAAGAAG TAATCAAAAA AAAAGAAGGA CAACTAAAAA AAGTAACTTG AATATATAAA   
  
  
- ATTAATATTA TTATATTATT ATTAATGTAT ATGATTTTAT GACTCAACAG AAGAAAAAAA AAATGCAAAA   
  
  
- ATTTAATAAT AAAAAAACAA AAACAAAAAA AGAAACACAA AAAAAATAAA ATTACTTAAA AAAAACAAAT   
  
  
- TAAATCAAAC AATTACAAAT TAAAAAAAAT AAATCAATAG TATAAAAGTA TTATGCCTAG GGCTCAAACT   
  
  
- ACTCAATTAG GTCTTAAAAA CGAAAAGAAG AAAAATTAAT TAAAAAAAAA CAAATCAAAT CAAACAATTA   
  
  
- CAATTTAAAA AAAATAAATT AATAGTCTGA AAATACTGTG CATAGAGCCC AAACTGCCCA ATTGCGCAGT   
  
  
- TAAAAAAAAA GATAAATCAA TAGTTTAAAA ATACTGCGCT TAAAGTCCAA ACTGTCCAAT TGGACCAAAC   
  
  
- TTCCCAATTG GGTAAATTAA GTTAAAAAAA AGAAAAAGAA GTAATCAAAA AAAGAAGGTA AATTAAAAAA   
  
  
- AAGAAAAAGA AGTAATTACA ATAATAATTA TAATATTTAT AGTGAGAACC TAGTCCGCAA CGTCGATTTA   
  
  
- GATTTTGAGT ATCCATATCG AAATATCTTT AAACTTAGAA TCAAAAAAAA ATATAAAAAA TATGCTTTTT   
  
  
- TAATTGGGCA CGTCCATTAC ACTGATCATT ATTGTAATAA AAGACAACTT GGCTTGTTCT TTCTTAGCAT   
  
  
- CAATCCACAG TCTCTGAGTC TCAGCGACGA AGAGTGTAAG TTTGGAACAT TCGCAAACCA GTAACATTCT   
  
  
- GGGAGAGAAA GAAAATAGTT TATTGGAAAG AAATAAAGAA TTTATTTAAT TCCTGGGAAG AAAAAAATAT   
  
  
- TTTTTATTTT TATTTTTTAA GAAGGAAGAA GGGTATTAAC TAAGTTAGCG TTTACTCAGT ACAGTACTGG   
  
  
- GCCTCTGGGA AACTTTTCAG TTTAATCTAT TTCTAACTCT GCTACCAAAG TTCTCTGTGT TGGTGAGTTC   
  
  
- ATAAATGGTA ACACTACTCG ACTGTTCCTA ACAGTAGGTC TTACCAGAGA GGAGTGCTAC AGTGGTCGGT   
  
  
- CACCTTAGGT TTAATTTAAC TTAAATAACC CTTTACCCTA TTGAGAAGGA AAATATATAA TTTTTAATTT   
  
  
- TTTATATATG TCACGGTTAA GATGACATGA CCAATTAATG GGATAGTTGT AAATTCATCA AATACATTGC   
  
  
- AACATAATCA ACAAAAAATT TTATTAAAAA AAAATTATAT AATTTTATTA AAAAAATTTT TTAAATAAAA   
  
  
- ACTATAATTA TACAATTTTG TTAAATTTTT GTAACTTTTT TTTATTTTAT TTTATTTAAG TTCAAAACGT   
  
  
- GTTTTTTTGT GATAACGTGG CGCATGTATT TACTACAGAA TCACATAAAT CGTAACACTA TCGAAAACAC   
  
  
- TAGTAAGACC AAAATTTTTT TGTTTAAATT TTCTTTATAA AAATCAACAC CAAAATTTTT CATCTAAAAT   
  
  
- TTTTTATGTA CAAACTAATT TAGATGATAC TCTATTTAAA AATGTACATT TTATTTATTT TTGTATAAAG   
  
  
- GTAGTTAATT TTTTTTTGTA CAAGAAAGTC AAAGATGTAC TGATTATAAA TTTTATGTTA ATGAATATTT   
  
  
- CAGGCCATGC TTTCGTGGGA GATTTACCTC TTACTTGACA TACATAAGAA GGGTACCTGG ACACTAAAAA   
  
  
- GGCATCCTTT CCGTTAAAGG TAAGTCTTAT GTTCTTCTAT GTACAGAAAA CTTTGAGAAT AATCGACAGA   
  
  
- TGGATAGTTG GTTCTTACTG CGAAGAGTAG CATACAGTGA TATTTTAAAC CAACTACTGT GGTTGTGAAA   
  
  
- GGGTAACCAT AAGTACCGAA AAATCGACGT GCTAGGTTG

+     CGTCA-motif

| Site Name | Organism | Position | Strand | Matrix score. | sequence | function |
| --- | --- | --- | --- | --- | --- | --- |
| CGTCA-motif | Hordeum vulgare | 1907 | - | 5 | CGTCA | cis-acting regulatory element involved in the MeJA-responsiveness |
| CGTCA-motif | Hordeum vulgare | 473 | - | 5 | CGTCA | cis-acting regulatory element involved in the MeJA-responsiveness |
| CGTCA-motif | Hordeum vulgare | 33 | - | 5 | CGTCA | cis-acting regulatory element involved in the MeJA-responsiveness |
| CGTCA-motif | Hordeum vulgare | 523 | - | 5 | CGTCA | cis-acting regulatory element involved in the MeJA-responsiveness |
| CGTCA-motif | Hordeum vulgare | 486 | + | 5 | CGTCA | cis-acting regulatory element involved in the MeJA-responsiveness |

>Potri.004G051700.1   
+ TTATCAAACT TTCATGATGT GAATCTCAGG TTTGACGGGT TAACTTGGTT TGAAGGGTTA ACCCAGTTAA   
  
  
+ TTCAAATTTT TTTTTTCTTC ATTAGTTTTT TTTTCTTCCT GTTGATTTTT TTCATTGAAC TTATATATTT   
  
  
+ TAATTATAAT AATATAATAA TAATTACATA TACTAAAATA CTGAGTTGTC TTCTTTTTTT TTTACGTTTT   
  
  
+ TAAATTATTA TTTTTTTGTT TTTGTTTTTT TCTTTGTGTT TTTTTTATTT TAATGAATTT TTTTTGTTTA   
  
  
+ ATTTAGTTTG TTAATGTTTA ATTTTTTTTA TTTAGTTATC ATATTTTCAT AATACGGATC CCGAGTTTGA   
  
  
+ TGAGTTAATC CAGAATTTTT GCTTTTCTTC TTTTTAATTA ATTTTTTTTT GTTTAGTTTA GTTTGTTAAT   
  
  
+ GTTAAATTTT TTTTATTTAA TTATCAGACT TTTATGACAC GTATCTCGGG TTTGACGGGT TAACGCGTCA   
  
  
+ ATTTTTTTTT CTATTTAGTT ATCAAATTTT TATGACGCGA ATTTCAGGTT TGACAGGTTA ACCTGGTTTG   
  
  
+ AAGGGTTAAC CCATTTAATT CAATTTTTTT TCTTTTTCTT CATTAGTTTT TTTCTTCCAT TTAATTTTTT   
  
  
+ TTCTTTTTCT TCATTAATGT TATTATTAAT ATTATAAATA TCACTCTTGG ATCAGGCGTT GCAGCTAAAT   
  
  
+ CTAAAACTCA TAGGTATAGC TTTATAGAAA TTTGAATCTT AGTTTTTTTT TATATTTTTT ATACGAAAAA   
  
  
+ ATTAACCCGT GCAGGTAATG TGACTAGTAA TAACATTATT TTCTGTTGAA CCGAACAAGA AAGAATCGTA   
  
  
+ GTTAGGTGTC AGAGACTCAG AGTCGCTGCT TCTCACATTC AAACCTTGTA AGCGTTTGGT CATTGTAAGA   
  
  
+ CCCTCTCTTT CTTTTATCAA ATAACCTTTC TTTATTTCTT AAATAAATTA AGGACCCTTC TTTTTTTATA   
  
  
+ AAAAATAAAA ATAAAAAATT CTTCCTTCTT CCCATAATTG ATTCAATCGC AAATGAGTCA TGTCATGACC   
  
  
+ CGGAGACCCT TTGAAAAGTC AAATTAGATA AAGATTGAGA CGATGGTTTC AAGAGACACA ACCACTCAAG   
  
  
+ TATTTACCAT TGTGATGAGC TGACAAGGAT TGTCATCCAG AATGGTCTCT CCTCACGATG TCACCAGCCA   
  
  
+ GTGGAATCCA AATTAAATTG AATTTATTGG GAAATGGGAT AACTCTTCCT TTTATATATT AAAAATTAAA   
  
  
+ AAATATATAC AGTGCCAATT CTACTGTACT GGTTAATTAC CCTATCAACA TTTAAGTAGT TTATGTAACG   
  
  
+ TTGTATTAGT TGTTTTTTAA AATAATTTTT TTTTAATATA TTAAAATAAT TTTTTTAAAA AATTTATTTT   
  
  
+ TGATATTAAT ATGTTAAAAC AATTTAAAAA CATTGAAAAA AAATAAAATA AAATAAATTC AAGTTTTGCA   
  
  
+ CAAAAAAACA CTATTGCACC GCGTACATAA ATGATGTCTT AGTGTATTTA GCATTGTGAT AGCTTTTGTG   
  
  
+ ATCATTCTGG TTTTAAAAAA ACAAATTTAA AAGAAATATT TTTAGTTGTG GTTTTAAAAA GTAGATTTTA   
  
  
+ AAAAATACAT GTTTGATTAA ATCTACTATG AGATAAATTT TTACATGTAA AATAAATAAA AACATATTTC   
  
  
+ CATCAATTAA AAAAAAACAT GTTCTTTCAG TTTCTACATG ACTAATATTT AAAATACAAT TACTTATAAA   
  
  
+ GTCCGGTACG AAAGCACCCT CTAAATGGAG AATGAACTGT ATGTATTCTT CCCATGGACC TGTGATTTTT   
  
  
+ CCGTAGGAAA GGCAATTTCC ATTCAGAATA CAAGAAGATA CATGTCTTTT GAAACTCTTA TTAGCTGTCT   
  
  
+ ACCTATCAAC CAAGAATGAC GCTTCTCATC GTATGTCACT ATAAAATTTG GTTGATGACA CCAACACTTT   
  
  
+ CCCATTGGTA TTCATGGCTT TTTAGCTGCA CGATCCAAC  

- AATAGTTTGA AAGTACTACA CTTAGAGTCC AAACTGCCCA ATTGAACCAA ACTTCCCAAT TGGGTCAATT   
  
  
- AAGTTTAAAA AAAAAAGAAG TAATCAAAAA AAAAGAAGGA CAACTAAAAA AAGTAACTTG AATATATAAA   
  
  
- ATTAATATTA TTATATTATT ATTAATGTAT ATGATTTTAT GACTCAACAG AAGAAAAAAA AAATGCAAAA   
  
  
- ATTTAATAAT AAAAAAACAA AAACAAAAAA AGAAACACAA AAAAAATAAA ATTACTTAAA AAAAACAAAT   
  
  
- TAAATCAAAC AATTACAAAT TAAAAAAAAT AAATCAATAG TATAAAAGTA TTATGCCTAG GGCTCAAACT   
  
  
- ACTCAATTAG GTCTTAAAAA CGAAAAGAAG AAAAATTAAT TAAAAAAAAA CAAATCAAAT CAAACAATTA   
  
  
- CAATTTAAAA AAAATAAATT AATAGTCTGA AAATACTGTG CATAGAGCCC AAACTGCCCA ATTGCGCAGT   
  
  
- TAAAAAAAAA GATAAATCAA TAGTTTAAAA ATACTGCGCT TAAAGTCCAA ACTGTCCAAT TGGACCAAAC   
  
  
- TTCCCAATTG GGTAAATTAA GTTAAAAAAA AGAAAAAGAA GTAATCAAAA AAAGAAGGTA AATTAAAAAA   
  
  
- AAGAAAAAGA AGTAATTACA ATAATAATTA TAATATTTAT AGTGAGAACC TAGTCCGCAA CGTCGATTTA   
  
  
- GATTTTGAGT ATCCATATCG AAATATCTTT AAACTTAGAA TCAAAAAAAA ATATAAAAAA TATGCTTTTT   
  
  
- TAATTGGGCA CGTCCATTAC ACTGATCATT ATTGTAATAA AAGACAACTT GGCTTGTTCT TTCTTAGCAT   
  
  
- CAATCCACAG TCTCTGAGTC TCAGCGACGA AGAGTGTAAG TTTGGAACAT TCGCAAACCA GTAACATTCT   
  
  
- GGGAGAGAAA GAAAATAGTT TATTGGAAAG AAATAAAGAA TTTATTTAAT TCCTGGGAAG AAAAAAATAT   
  
  
- TTTTTATTTT TATTTTTTAA GAAGGAAGAA GGGTATTAAC TAAGTTAGCG TTTACTCAGT ACAGTACTGG   
  
  
- GCCTCTGGGA AACTTTTCAG TTTAATCTAT TTCTAACTCT GCTACCAAAG TTCTCTGTGT TGGTGAGTTC   
  
  
- ATAAATGGTA ACACTACTCG ACTGTTCCTA ACAGTAGGTC TTACCAGAGA GGAGTGCTAC AGTGGTCGGT   
  
  
- CACCTTAGGT TTAATTTAAC TTAAATAACC CTTTACCCTA TTGAGAAGGA AAATATATAA TTTTTAATTT   
  
  
- TTTATATATG TCACGGTTAA GATGACATGA CCAATTAATG GGATAGTTGT AAATTCATCA AATACATTGC   
  
  
- AACATAATCA ACAAAAAATT TTATTAAAAA AAAATTATAT AATTTTATTA AAAAAATTTT TTAAATAAAA   
  
  
- ACTATAATTA TACAATTTTG TTAAATTTTT GTAACTTTTT TTTATTTTAT TTTATTTAAG TTCAAAACGT   
  
  
- GTTTTTTTGT GATAACGTGG CGCATGTATT TACTACAGAA TCACATAAAT CGTAACACTA TCGAAAACAC   
  
  
- TAGTAAGACC AAAATTTTTT TGTTTAAATT TTCTTTATAA AAATCAACAC CAAAATTTTT CATCTAAAAT   
  
  
- TTTTTATGTA CAAACTAATT TAGATGATAC TCTATTTAAA AATGTACATT TTATTTATTT TTGTATAAAG   
  
  
- GTAGTTAATT TTTTTTTGTA CAAGAAAGTC AAAGATGTAC TGATTATAAA TTTTATGTTA ATGAATATTT   
  
  
- CAGGCCATGC TTTCGTGGGA GATTTACCTC TTACTTGACA TACATAAGAA GGGTACCTGG ACACTAAAAA   
  
  
- GGCATCCTTT CCGTTAAAGG TAAGTCTTAT GTTCTTCTAT GTACAGAAAA CTTTGAGAAT AATCGACAGA   
  
  
- TGGATAGTTG GTTCTTACTG CGAAGAGTAG CATACAGTGA TATTTTAAAC CAACTACTGT GGTTGTGAAA   
  
  
- GGGTAACCAT AAGTACCGAA AAATCGACGT GCTAGGTTG

+     ERE

| Site Name | Organism | Position | Strand | Matrix score. | sequence | function |
| --- | --- | --- | --- | --- | --- | --- |
| ERE | Nicotiana glutinos | 1728 | - | 8 | ATTTTAAA |  |
| ERE | Nicotiana glutinos | 1605 | + | 8 | ATTTTAAA |  |
| ERE | Nicotiana glutinos | 1346 | - | 8 | ATTTTAAA |  |

>Potri.004G051700.1   
+ TTATCAAACT TTCATGATGT GAATCTCAGG TTTGACGGGT TAACTTGGTT TGAAGGGTTA ACCCAGTTAA   
  
  
+ TTCAAATTTT TTTTTTCTTC ATTAGTTTTT TTTTCTTCCT GTTGATTTTT TTCATTGAAC TTATATATTT   
  
  
+ TAATTATAAT AATATAATAA TAATTACATA TACTAAAATA CTGAGTTGTC TTCTTTTTTT TTTACGTTTT   
  
  
+ TAAATTATTA TTTTTTTGTT TTTGTTTTTT TCTTTGTGTT TTTTTTATTT TAATGAATTT TTTTTGTTTA   
  
  
+ ATTTAGTTTG TTAATGTTTA ATTTTTTTTA TTTAGTTATC ATATTTTCAT AATACGGATC CCGAGTTTGA   
  
  
+ TGAGTTAATC CAGAATTTTT GCTTTTCTTC TTTTTAATTA ATTTTTTTTT GTTTAGTTTA GTTTGTTAAT   
  
  
+ GTTAAATTTT TTTTATTTAA TTATCAGACT TTTATGACAC GTATCTCGGG TTTGACGGGT TAACGCGTCA   
  
  
+ ATTTTTTTTT CTATTTAGTT ATCAAATTTT TATGACGCGA ATTTCAGGTT TGACAGGTTA ACCTGGTTTG   
  
  
+ AAGGGTTAAC CCATTTAATT CAATTTTTTT TCTTTTTCTT CATTAGTTTT TTTCTTCCAT TTAATTTTTT   
  
  
+ TTCTTTTTCT TCATTAATGT TATTATTAAT ATTATAAATA TCACTCTTGG ATCAGGCGTT GCAGCTAAAT   
  
  
+ CTAAAACTCA TAGGTATAGC TTTATAGAAA TTTGAATCTT AGTTTTTTTT TATATTTTTT ATACGAAAAA   
  
  
+ ATTAACCCGT GCAGGTAATG TGACTAGTAA TAACATTATT TTCTGTTGAA CCGAACAAGA AAGAATCGTA   
  
  
+ GTTAGGTGTC AGAGACTCAG AGTCGCTGCT TCTCACATTC AAACCTTGTA AGCGTTTGGT CATTGTAAGA   
  
  
+ CCCTCTCTTT CTTTTATCAA ATAACCTTTC TTTATTTCTT AAATAAATTA AGGACCCTTC TTTTTTTATA   
  
  
+ AAAAATAAAA ATAAAAAATT CTTCCTTCTT CCCATAATTG ATTCAATCGC AAATGAGTCA TGTCATGACC   
  
  
+ CGGAGACCCT TTGAAAAGTC AAATTAGATA AAGATTGAGA CGATGGTTTC AAGAGACACA ACCACTCAAG   
  
  
+ TATTTACCAT TGTGATGAGC TGACAAGGAT TGTCATCCAG AATGGTCTCT CCTCACGATG TCACCAGCCA   
  
  
+ GTGGAATCCA AATTAAATTG AATTTATTGG GAAATGGGAT AACTCTTCCT TTTATATATT AAAAATTAAA   
  
  
+ AAATATATAC AGTGCCAATT CTACTGTACT GGTTAATTAC CCTATCAACA TTTAAGTAGT TTATGTAACG   
  
  
+ TTGTATTAGT TGTTTTTTAA AATAATTTTT TTTTAATATA TTAAAATAAT TTTTTTAAAA AATTTATTTT   
  
  
+ TGATATTAAT ATGTTAAAAC AATTTAAAAA CATTGAAAAA AAATAAAATA AAATAAATTC AAGTTTTGCA   
  
  
+ CAAAAAAACA CTATTGCACC GCGTACATAA ATGATGTCTT AGTGTATTTA GCATTGTGAT AGCTTTTGTG   
  
  
+ ATCATTCTGG TTTTAAAAAA ACAAATTTAA AAGAAATATT TTTAGTTGTG GTTTTAAAAA GTAGATTTTA   
  
  
+ AAAAATACAT GTTTGATTAA ATCTACTATG AGATAAATTT TTACATGTAA AATAAATAAA AACATATTTC   
  
  
+ CATCAATTAA AAAAAAACAT GTTCTTTCAG TTTCTACATG ACTAATATTT AAAATACAAT TACTTATAAA   
  
  
+ GTCCGGTACG AAAGCACCCT CTAAATGGAG AATGAACTGT ATGTATTCTT CCCATGGACC TGTGATTTTT   
  
  
+ CCGTAGGAAA GGCAATTTCC ATTCAGAATA CAAGAAGATA CATGTCTTTT GAAACTCTTA TTAGCTGTCT   
  
  
+ ACCTATCAAC CAAGAATGAC GCTTCTCATC GTATGTCACT ATAAAATTTG GTTGATGACA CCAACACTTT   
  
  
+ CCCATTGGTA TTCATGGCTT TTTAGCTGCA CGATCCAAC  

- AATAGTTTGA AAGTACTACA CTTAGAGTCC AAACTGCCCA ATTGAACCAA ACTTCCCAAT TGGGTCAATT   
  
  
- AAGTTTAAAA AAAAAAGAAG TAATCAAAAA AAAAGAAGGA CAACTAAAAA AAGTAACTTG AATATATAAA   
  
  
- ATTAATATTA TTATATTATT ATTAATGTAT ATGATTTTAT GACTCAACAG AAGAAAAAAA AAATGCAAAA   
  
  
- ATTTAATAAT AAAAAAACAA AAACAAAAAA AGAAACACAA AAAAAATAAA ATTACTTAAA AAAAACAAAT   
  
  
- TAAATCAAAC AATTACAAAT TAAAAAAAAT AAATCAATAG TATAAAAGTA TTATGCCTAG GGCTCAAACT   
  
  
- ACTCAATTAG GTCTTAAAAA CGAAAAGAAG AAAAATTAAT TAAAAAAAAA CAAATCAAAT CAAACAATTA   
  
  
- CAATTTAAAA AAAATAAATT AATAGTCTGA AAATACTGTG CATAGAGCCC AAACTGCCCA ATTGCGCAGT   
  
  
- TAAAAAAAAA GATAAATCAA TAGTTTAAAA ATACTGCGCT TAAAGTCCAA ACTGTCCAAT TGGACCAAAC   
  
  
- TTCCCAATTG GGTAAATTAA GTTAAAAAAA AGAAAAAGAA GTAATCAAAA AAAGAAGGTA AATTAAAAAA   
  
  
- AAGAAAAAGA AGTAATTACA ATAATAATTA TAATATTTAT AGTGAGAACC TAGTCCGCAA CGTCGATTTA   
  
  
- GATTTTGAGT ATCCATATCG AAATATCTTT AAACTTAGAA TCAAAAAAAA ATATAAAAAA TATGCTTTTT   
  
  
- TAATTGGGCA CGTCCATTAC ACTGATCATT ATTGTAATAA AAGACAACTT GGCTTGTTCT TTCTTAGCAT   
  
  
- CAATCCACAG TCTCTGAGTC TCAGCGACGA AGAGTGTAAG TTTGGAACAT TCGCAAACCA GTAACATTCT   
  
  
- GGGAGAGAAA GAAAATAGTT TATTGGAAAG AAATAAAGAA TTTATTTAAT TCCTGGGAAG AAAAAAATAT   
  
  
- TTTTTATTTT TATTTTTTAA GAAGGAAGAA GGGTATTAAC TAAGTTAGCG TTTACTCAGT ACAGTACTGG   
  
  
- GCCTCTGGGA AACTTTTCAG TTTAATCTAT TTCTAACTCT GCTACCAAAG TTCTCTGTGT TGGTGAGTTC   
  
  
- ATAAATGGTA ACACTACTCG ACTGTTCCTA ACAGTAGGTC TTACCAGAGA GGAGTGCTAC AGTGGTCGGT   
  
  
- CACCTTAGGT TTAATTTAAC TTAAATAACC CTTTACCCTA TTGAGAAGGA AAATATATAA TTTTTAATTT   
  
  
- TTTATATATG TCACGGTTAA GATGACATGA CCAATTAATG GGATAGTTGT AAATTCATCA AATACATTGC   
  
  
- AACATAATCA ACAAAAAATT TTATTAAAAA AAAATTATAT AATTTTATTA AAAAAATTTT TTAAATAAAA   
  
  
- ACTATAATTA TACAATTTTG TTAAATTTTT GTAACTTTTT TTTATTTTAT TTTATTTAAG TTCAAAACGT   
  
  
- GTTTTTTTGT GATAACGTGG CGCATGTATT TACTACAGAA TCACATAAAT CGTAACACTA TCGAAAACAC   
  
  
- TAGTAAGACC AAAATTTTTT TGTTTAAATT TTCTTTATAA AAATCAACAC CAAAATTTTT CATCTAAAAT   
  
  
- TTTTTATGTA CAAACTAATT TAGATGATAC TCTATTTAAA AATGTACATT TTATTTATTT TTGTATAAAG   
  
  
- GTAGTTAATT TTTTTTTGTA CAAGAAAGTC AAAGATGTAC TGATTATAAA TTTTATGTTA ATGAATATTT   
  
  
- CAGGCCATGC TTTCGTGGGA GATTTACCTC TTACTTGACA TACATAAGAA GGGTACCTGG ACACTAAAAA   
  
  
- GGCATCCTTT CCGTTAAAGG TAAGTCTTAT GTTCTTCTAT GTACAGAAAA CTTTGAGAAT AATCGACAGA   
  
  
- TGGATAGTTG GTTCTTACTG CGAAGAGTAG CATACAGTGA TATTTTAAAC CAACTACTGT GGTTGTGAAA   
  
  
- GGGTAACCAT AAGTACCGAA AAATCGACGT GCTAGGTTG

+     G-box

| Site Name | Organism | Position | Strand | Matrix score. | sequence | function |
| --- | --- | --- | --- | --- | --- | --- |
| G-box | Arabidopsis thaliana | 458 | - | 6 | TACGTG | cis-acting regulatory element involved in light responsiveness |

>Potri.004G051700.1   
+ TTATCAAACT TTCATGATGT GAATCTCAGG TTTGACGGGT TAACTTGGTT TGAAGGGTTA ACCCAGTTAA   
  
  
+ TTCAAATTTT TTTTTTCTTC ATTAGTTTTT TTTTCTTCCT GTTGATTTTT TTCATTGAAC TTATATATTT   
  
  
+ TAATTATAAT AATATAATAA TAATTACATA TACTAAAATA CTGAGTTGTC TTCTTTTTTT TTTACGTTTT   
  
  
+ TAAATTATTA TTTTTTTGTT TTTGTTTTTT TCTTTGTGTT TTTTTTATTT TAATGAATTT TTTTTGTTTA   
  
  
+ ATTTAGTTTG TTAATGTTTA ATTTTTTTTA TTTAGTTATC ATATTTTCAT AATACGGATC CCGAGTTTGA   
  
  
+ TGAGTTAATC CAGAATTTTT GCTTTTCTTC TTTTTAATTA ATTTTTTTTT GTTTAGTTTA GTTTGTTAAT   
  
  
+ GTTAAATTTT TTTTATTTAA TTATCAGACT TTTATGACAC GTATCTCGGG TTTGACGGGT TAACGCGTCA   
  
  
+ ATTTTTTTTT CTATTTAGTT ATCAAATTTT TATGACGCGA ATTTCAGGTT TGACAGGTTA ACCTGGTTTG   
  
  
+ AAGGGTTAAC CCATTTAATT CAATTTTTTT TCTTTTTCTT CATTAGTTTT TTTCTTCCAT TTAATTTTTT   
  
  
+ TTCTTTTTCT TCATTAATGT TATTATTAAT ATTATAAATA TCACTCTTGG ATCAGGCGTT GCAGCTAAAT   
  
  
+ CTAAAACTCA TAGGTATAGC TTTATAGAAA TTTGAATCTT AGTTTTTTTT TATATTTTTT ATACGAAAAA   
  
  
+ ATTAACCCGT GCAGGTAATG TGACTAGTAA TAACATTATT TTCTGTTGAA CCGAACAAGA AAGAATCGTA   
  
  
+ GTTAGGTGTC AGAGACTCAG AGTCGCTGCT TCTCACATTC AAACCTTGTA AGCGTTTGGT CATTGTAAGA   
  
  
+ CCCTCTCTTT CTTTTATCAA ATAACCTTTC TTTATTTCTT AAATAAATTA AGGACCCTTC TTTTTTTATA   
  
  
+ AAAAATAAAA ATAAAAAATT CTTCCTTCTT CCCATAATTG ATTCAATCGC AAATGAGTCA TGTCATGACC   
  
  
+ CGGAGACCCT TTGAAAAGTC AAATTAGATA AAGATTGAGA CGATGGTTTC AAGAGACACA ACCACTCAAG   
  
  
+ TATTTACCAT TGTGATGAGC TGACAAGGAT TGTCATCCAG AATGGTCTCT CCTCACGATG TCACCAGCCA   
  
  
+ GTGGAATCCA AATTAAATTG AATTTATTGG GAAATGGGAT AACTCTTCCT TTTATATATT AAAAATTAAA   
  
  
+ AAATATATAC AGTGCCAATT CTACTGTACT GGTTAATTAC CCTATCAACA TTTAAGTAGT TTATGTAACG   
  
  
+ TTGTATTAGT TGTTTTTTAA AATAATTTTT TTTTAATATA TTAAAATAAT TTTTTTAAAA AATTTATTTT   
  
  
+ TGATATTAAT ATGTTAAAAC AATTTAAAAA CATTGAAAAA AAATAAAATA AAATAAATTC AAGTTTTGCA   
  
  
+ CAAAAAAACA CTATTGCACC GCGTACATAA ATGATGTCTT AGTGTATTTA GCATTGTGAT AGCTTTTGTG   
  
  
+ ATCATTCTGG TTTTAAAAAA ACAAATTTAA AAGAAATATT TTTAGTTGTG GTTTTAAAAA GTAGATTTTA   
  
  
+ AAAAATACAT GTTTGATTAA ATCTACTATG AGATAAATTT TTACATGTAA AATAAATAAA AACATATTTC   
  
  
+ CATCAATTAA AAAAAAACAT GTTCTTTCAG TTTCTACATG ACTAATATTT AAAATACAAT TACTTATAAA   
  
  
+ GTCCGGTACG AAAGCACCCT CTAAATGGAG AATGAACTGT ATGTATTCTT CCCATGGACC TGTGATTTTT   
  
  
+ CCGTAGGAAA GGCAATTTCC ATTCAGAATA CAAGAAGATA CATGTCTTTT GAAACTCTTA TTAGCTGTCT   
  
  
+ ACCTATCAAC CAAGAATGAC GCTTCTCATC GTATGTCACT ATAAAATTTG GTTGATGACA CCAACACTTT   
  
  
+ CCCATTGGTA TTCATGGCTT TTTAGCTGCA CGATCCAAC  

- AATAGTTTGA AAGTACTACA CTTAGAGTCC AAACTGCCCA ATTGAACCAA ACTTCCCAAT TGGGTCAATT   
  
  
- AAGTTTAAAA AAAAAAGAAG TAATCAAAAA AAAAGAAGGA CAACTAAAAA AAGTAACTTG AATATATAAA   
  
  
- ATTAATATTA TTATATTATT ATTAATGTAT ATGATTTTAT GACTCAACAG AAGAAAAAAA AAATGCAAAA   
  
  
- ATTTAATAAT AAAAAAACAA AAACAAAAAA AGAAACACAA AAAAAATAAA ATTACTTAAA AAAAACAAAT   
  
  
- TAAATCAAAC AATTACAAAT TAAAAAAAAT AAATCAATAG TATAAAAGTA TTATGCCTAG GGCTCAAACT   
  
  
- ACTCAATTAG GTCTTAAAAA CGAAAAGAAG AAAAATTAAT TAAAAAAAAA CAAATCAAAT CAAACAATTA   
  
  
- CAATTTAAAA AAAATAAATT AATAGTCTGA AAATACTGTG CATAGAGCCC AAACTGCCCA ATTGCGCAGT   
  
  
- TAAAAAAAAA GATAAATCAA TAGTTTAAAA ATACTGCGCT TAAAGTCCAA ACTGTCCAAT TGGACCAAAC   
  
  
- TTCCCAATTG GGTAAATTAA GTTAAAAAAA AGAAAAAGAA GTAATCAAAA AAAGAAGGTA AATTAAAAAA   
  
  
- AAGAAAAAGA AGTAATTACA ATAATAATTA TAATATTTAT AGTGAGAACC TAGTCCGCAA CGTCGATTTA   
  
  
- GATTTTGAGT ATCCATATCG AAATATCTTT AAACTTAGAA TCAAAAAAAA ATATAAAAAA TATGCTTTTT   
  
  
- TAATTGGGCA CGTCCATTAC ACTGATCATT ATTGTAATAA AAGACAACTT GGCTTGTTCT TTCTTAGCAT   
  
  
- CAATCCACAG TCTCTGAGTC TCAGCGACGA AGAGTGTAAG TTTGGAACAT TCGCAAACCA GTAACATTCT   
  
  
- GGGAGAGAAA GAAAATAGTT TATTGGAAAG AAATAAAGAA TTTATTTAAT TCCTGGGAAG AAAAAAATAT   
  
  
- TTTTTATTTT TATTTTTTAA GAAGGAAGAA GGGTATTAAC TAAGTTAGCG TTTACTCAGT ACAGTACTGG   
  
  
- GCCTCTGGGA AACTTTTCAG TTTAATCTAT TTCTAACTCT GCTACCAAAG TTCTCTGTGT TGGTGAGTTC   
  
  
- ATAAATGGTA ACACTACTCG ACTGTTCCTA ACAGTAGGTC TTACCAGAGA GGAGTGCTAC AGTGGTCGGT   
  
  
- CACCTTAGGT TTAATTTAAC TTAAATAACC CTTTACCCTA TTGAGAAGGA AAATATATAA TTTTTAATTT   
  
  
- TTTATATATG TCACGGTTAA GATGACATGA CCAATTAATG GGATAGTTGT AAATTCATCA AATACATTGC   
  
  
- AACATAATCA ACAAAAAATT TTATTAAAAA AAAATTATAT AATTTTATTA AAAAAATTTT TTAAATAAAA   
  
  
- ACTATAATTA TACAATTTTG TTAAATTTTT GTAACTTTTT TTTATTTTAT TTTATTTAAG TTCAAAACGT   
  
  
- GTTTTTTTGT GATAACGTGG CGCATGTATT TACTACAGAA TCACATAAAT CGTAACACTA TCGAAAACAC   
  
  
- TAGTAAGACC AAAATTTTTT TGTTTAAATT TTCTTTATAA AAATCAACAC CAAAATTTTT CATCTAAAAT   
  
  
- TTTTTATGTA CAAACTAATT TAGATGATAC TCTATTTAAA AATGTACATT TTATTTATTT TTGTATAAAG   
  
  
- GTAGTTAATT TTTTTTTGTA CAAGAAAGTC AAAGATGTAC TGATTATAAA TTTTATGTTA ATGAATATTT   
  
  
- CAGGCCATGC TTTCGTGGGA GATTTACCTC TTACTTGACA TACATAAGAA GGGTACCTGG ACACTAAAAA   
  
  
- GGCATCCTTT CCGTTAAAGG TAAGTCTTAT GTTCTTCTAT GTACAGAAAA CTTTGAGAAT AATCGACAGA   
  
  
- TGGATAGTTG GTTCTTACTG CGAAGAGTAG CATACAGTGA TATTTTAAAC CAACTACTGT GGTTGTGAAA   
  
  
- GGGTAACCAT AAGTACCGAA AAATCGACGT GCTAGGTTG

+     GARE-motif

| Site Name | Organism | Position | Strand | Matrix score. | sequence | function |
| --- | --- | --- | --- | --- | --- | --- |
| GARE-motif | Brassica oleracea | 812 | + | 7 | TCTGTTG | gibberellin-responsive element |

>Potri.004G051700.1   
+ TTATCAAACT TTCATGATGT GAATCTCAGG TTTGACGGGT TAACTTGGTT TGAAGGGTTA ACCCAGTTAA   
  
  
+ TTCAAATTTT TTTTTTCTTC ATTAGTTTTT TTTTCTTCCT GTTGATTTTT TTCATTGAAC TTATATATTT   
  
  
+ TAATTATAAT AATATAATAA TAATTACATA TACTAAAATA CTGAGTTGTC TTCTTTTTTT TTTACGTTTT   
  
  
+ TAAATTATTA TTTTTTTGTT TTTGTTTTTT TCTTTGTGTT TTTTTTATTT TAATGAATTT TTTTTGTTTA   
  
  
+ ATTTAGTTTG TTAATGTTTA ATTTTTTTTA TTTAGTTATC ATATTTTCAT AATACGGATC CCGAGTTTGA   
  
  
+ TGAGTTAATC CAGAATTTTT GCTTTTCTTC TTTTTAATTA ATTTTTTTTT GTTTAGTTTA GTTTGTTAAT   
  
  
+ GTTAAATTTT TTTTATTTAA TTATCAGACT TTTATGACAC GTATCTCGGG TTTGACGGGT TAACGCGTCA   
  
  
+ ATTTTTTTTT CTATTTAGTT ATCAAATTTT TATGACGCGA ATTTCAGGTT TGACAGGTTA ACCTGGTTTG   
  
  
+ AAGGGTTAAC CCATTTAATT CAATTTTTTT TCTTTTTCTT CATTAGTTTT TTTCTTCCAT TTAATTTTTT   
  
  
+ TTCTTTTTCT TCATTAATGT TATTATTAAT ATTATAAATA TCACTCTTGG ATCAGGCGTT GCAGCTAAAT   
  
  
+ CTAAAACTCA TAGGTATAGC TTTATAGAAA TTTGAATCTT AGTTTTTTTT TATATTTTTT ATACGAAAAA   
  
  
+ ATTAACCCGT GCAGGTAATG TGACTAGTAA TAACATTATT TTCTGTTGAA CCGAACAAGA AAGAATCGTA   
  
  
+ GTTAGGTGTC AGAGACTCAG AGTCGCTGCT TCTCACATTC AAACCTTGTA AGCGTTTGGT CATTGTAAGA   
  
  
+ CCCTCTCTTT CTTTTATCAA ATAACCTTTC TTTATTTCTT AAATAAATTA AGGACCCTTC TTTTTTTATA   
  
  
+ AAAAATAAAA ATAAAAAATT CTTCCTTCTT CCCATAATTG ATTCAATCGC AAATGAGTCA TGTCATGACC   
  
  
+ CGGAGACCCT TTGAAAAGTC AAATTAGATA AAGATTGAGA CGATGGTTTC AAGAGACACA ACCACTCAAG   
  
  
+ TATTTACCAT TGTGATGAGC TGACAAGGAT TGTCATCCAG AATGGTCTCT CCTCACGATG TCACCAGCCA   
  
  
+ GTGGAATCCA AATTAAATTG AATTTATTGG GAAATGGGAT AACTCTTCCT TTTATATATT AAAAATTAAA   
  
  
+ AAATATATAC AGTGCCAATT CTACTGTACT GGTTAATTAC CCTATCAACA TTTAAGTAGT TTATGTAACG   
  
  
+ TTGTATTAGT TGTTTTTTAA AATAATTTTT TTTTAATATA TTAAAATAAT TTTTTTAAAA AATTTATTTT   
  
  
+ TGATATTAAT ATGTTAAAAC AATTTAAAAA CATTGAAAAA AAATAAAATA AAATAAATTC AAGTTTTGCA   
  
  
+ CAAAAAAACA CTATTGCACC GCGTACATAA ATGATGTCTT AGTGTATTTA GCATTGTGAT AGCTTTTGTG   
  
  
+ ATCATTCTGG TTTTAAAAAA ACAAATTTAA AAGAAATATT TTTAGTTGTG GTTTTAAAAA GTAGATTTTA   
  
  
+ AAAAATACAT GTTTGATTAA ATCTACTATG AGATAAATTT TTACATGTAA AATAAATAAA AACATATTTC   
  
  
+ CATCAATTAA AAAAAAACAT GTTCTTTCAG TTTCTACATG ACTAATATTT AAAATACAAT TACTTATAAA   
  
  
+ GTCCGGTACG AAAGCACCCT CTAAATGGAG AATGAACTGT ATGTATTCTT CCCATGGACC TGTGATTTTT   
  
  
+ CCGTAGGAAA GGCAATTTCC ATTCAGAATA CAAGAAGATA CATGTCTTTT GAAACTCTTA TTAGCTGTCT   
  
  
+ ACCTATCAAC CAAGAATGAC GCTTCTCATC GTATGTCACT ATAAAATTTG GTTGATGACA CCAACACTTT   
  
  
+ CCCATTGGTA TTCATGGCTT TTTAGCTGCA CGATCCAAC  

- AATAGTTTGA AAGTACTACA CTTAGAGTCC AAACTGCCCA ATTGAACCAA ACTTCCCAAT TGGGTCAATT   
  
  
- AAGTTTAAAA AAAAAAGAAG TAATCAAAAA AAAAGAAGGA CAACTAAAAA AAGTAACTTG AATATATAAA   
  
  
- ATTAATATTA TTATATTATT ATTAATGTAT ATGATTTTAT GACTCAACAG AAGAAAAAAA AAATGCAAAA   
  
  
- ATTTAATAAT AAAAAAACAA AAACAAAAAA AGAAACACAA AAAAAATAAA ATTACTTAAA AAAAACAAAT   
  
  
- TAAATCAAAC AATTACAAAT TAAAAAAAAT AAATCAATAG TATAAAAGTA TTATGCCTAG GGCTCAAACT   
  
  
- ACTCAATTAG GTCTTAAAAA CGAAAAGAAG AAAAATTAAT TAAAAAAAAA CAAATCAAAT CAAACAATTA   
  
  
- CAATTTAAAA AAAATAAATT AATAGTCTGA AAATACTGTG CATAGAGCCC AAACTGCCCA ATTGCGCAGT   
  
  
- TAAAAAAAAA GATAAATCAA TAGTTTAAAA ATACTGCGCT TAAAGTCCAA ACTGTCCAAT TGGACCAAAC   
  
  
- TTCCCAATTG GGTAAATTAA GTTAAAAAAA AGAAAAAGAA GTAATCAAAA AAAGAAGGTA AATTAAAAAA   
  
  
- AAGAAAAAGA AGTAATTACA ATAATAATTA TAATATTTAT AGTGAGAACC TAGTCCGCAA CGTCGATTTA   
  
  
- GATTTTGAGT ATCCATATCG AAATATCTTT AAACTTAGAA TCAAAAAAAA ATATAAAAAA TATGCTTTTT   
  
  
- TAATTGGGCA CGTCCATTAC ACTGATCATT ATTGTAATAA AAGACAACTT GGCTTGTTCT TTCTTAGCAT   
  
  
- CAATCCACAG TCTCTGAGTC TCAGCGACGA AGAGTGTAAG TTTGGAACAT TCGCAAACCA GTAACATTCT   
  
  
- GGGAGAGAAA GAAAATAGTT TATTGGAAAG AAATAAAGAA TTTATTTAAT TCCTGGGAAG AAAAAAATAT   
  
  
- TTTTTATTTT TATTTTTTAA GAAGGAAGAA GGGTATTAAC TAAGTTAGCG TTTACTCAGT ACAGTACTGG   
  
  
- GCCTCTGGGA AACTTTTCAG TTTAATCTAT TTCTAACTCT GCTACCAAAG TTCTCTGTGT TGGTGAGTTC   
  
  
- ATAAATGGTA ACACTACTCG ACTGTTCCTA ACAGTAGGTC TTACCAGAGA GGAGTGCTAC AGTGGTCGGT   
  
  
- CACCTTAGGT TTAATTTAAC TTAAATAACC CTTTACCCTA TTGAGAAGGA AAATATATAA TTTTTAATTT   
  
  
- TTTATATATG TCACGGTTAA GATGACATGA CCAATTAATG GGATAGTTGT AAATTCATCA AATACATTGC   
  
  
- AACATAATCA ACAAAAAATT TTATTAAAAA AAAATTATAT AATTTTATTA AAAAAATTTT TTAAATAAAA   
  
  
- ACTATAATTA TACAATTTTG TTAAATTTTT GTAACTTTTT TTTATTTTAT TTTATTTAAG TTCAAAACGT   
  
  
- GTTTTTTTGT GATAACGTGG CGCATGTATT TACTACAGAA TCACATAAAT CGTAACACTA TCGAAAACAC   
  
  
- TAGTAAGACC AAAATTTTTT TGTTTAAATT TTCTTTATAA AAATCAACAC CAAAATTTTT CATCTAAAAT   
  
  
- TTTTTATGTA CAAACTAATT TAGATGATAC TCTATTTAAA AATGTACATT TTATTTATTT TTGTATAAAG   
  
  
- GTAGTTAATT TTTTTTTGTA CAAGAAAGTC AAAGATGTAC TGATTATAAA TTTTATGTTA ATGAATATTT   
  
  
- CAGGCCATGC TTTCGTGGGA GATTTACCTC TTACTTGACA TACATAAGAA GGGTACCTGG ACACTAAAAA   
  
  
- GGCATCCTTT CCGTTAAAGG TAAGTCTTAT GTTCTTCTAT GTACAGAAAA CTTTGAGAAT AATCGACAGA   
  
  
- TGGATAGTTG GTTCTTACTG CGAAGAGTAG CATACAGTGA TATTTTAAAC CAACTACTGT GGTTGTGAAA   
  
  
- GGGTAACCAT AAGTACCGAA AAATCGACGT GCTAGGTTG

+     GATA-motif

| Site Name | Organism | Position | Strand | Matrix score. | sequence | function |
| --- | --- | --- | --- | --- | --- | --- |
| GATA-motif | Pisum sativum | 1300 | - | 7 | GATAGGG | part of a light responsive element |

>Potri.004G051700.1   
+ TTATCAAACT TTCATGATGT GAATCTCAGG TTTGACGGGT TAACTTGGTT TGAAGGGTTA ACCCAGTTAA   
  
  
+ TTCAAATTTT TTTTTTCTTC ATTAGTTTTT TTTTCTTCCT GTTGATTTTT TTCATTGAAC TTATATATTT   
  
  
+ TAATTATAAT AATATAATAA TAATTACATA TACTAAAATA CTGAGTTGTC TTCTTTTTTT TTTACGTTTT   
  
  
+ TAAATTATTA TTTTTTTGTT TTTGTTTTTT TCTTTGTGTT TTTTTTATTT TAATGAATTT TTTTTGTTTA   
  
  
+ ATTTAGTTTG TTAATGTTTA ATTTTTTTTA TTTAGTTATC ATATTTTCAT AATACGGATC CCGAGTTTGA   
  
  
+ TGAGTTAATC CAGAATTTTT GCTTTTCTTC TTTTTAATTA ATTTTTTTTT GTTTAGTTTA GTTTGTTAAT   
  
  
+ GTTAAATTTT TTTTATTTAA TTATCAGACT TTTATGACAC GTATCTCGGG TTTGACGGGT TAACGCGTCA   
  
  
+ ATTTTTTTTT CTATTTAGTT ATCAAATTTT TATGACGCGA ATTTCAGGTT TGACAGGTTA ACCTGGTTTG   
  
  
+ AAGGGTTAAC CCATTTAATT CAATTTTTTT TCTTTTTCTT CATTAGTTTT TTTCTTCCAT TTAATTTTTT   
  
  
+ TTCTTTTTCT TCATTAATGT TATTATTAAT ATTATAAATA TCACTCTTGG ATCAGGCGTT GCAGCTAAAT   
  
  
+ CTAAAACTCA TAGGTATAGC TTTATAGAAA TTTGAATCTT AGTTTTTTTT TATATTTTTT ATACGAAAAA   
  
  
+ ATTAACCCGT GCAGGTAATG TGACTAGTAA TAACATTATT TTCTGTTGAA CCGAACAAGA AAGAATCGTA   
  
  
+ GTTAGGTGTC AGAGACTCAG AGTCGCTGCT TCTCACATTC AAACCTTGTA AGCGTTTGGT CATTGTAAGA   
  
  
+ CCCTCTCTTT CTTTTATCAA ATAACCTTTC TTTATTTCTT AAATAAATTA AGGACCCTTC TTTTTTTATA   
  
  
+ AAAAATAAAA ATAAAAAATT CTTCCTTCTT CCCATAATTG ATTCAATCGC AAATGAGTCA TGTCATGACC   
  
  
+ CGGAGACCCT TTGAAAAGTC AAATTAGATA AAGATTGAGA CGATGGTTTC AAGAGACACA ACCACTCAAG   
  
  
+ TATTTACCAT TGTGATGAGC TGACAAGGAT TGTCATCCAG AATGGTCTCT CCTCACGATG TCACCAGCCA   
  
  
+ GTGGAATCCA AATTAAATTG AATTTATTGG GAAATGGGAT AACTCTTCCT TTTATATATT AAAAATTAAA   
  
  
+ AAATATATAC AGTGCCAATT CTACTGTACT GGTTAATTAC CCTATCAACA TTTAAGTAGT TTATGTAACG   
  
  
+ TTGTATTAGT TGTTTTTTAA AATAATTTTT TTTTAATATA TTAAAATAAT TTTTTTAAAA AATTTATTTT   
  
  
+ TGATATTAAT ATGTTAAAAC AATTTAAAAA CATTGAAAAA AAATAAAATA AAATAAATTC AAGTTTTGCA   
  
  
+ CAAAAAAACA CTATTGCACC GCGTACATAA ATGATGTCTT AGTGTATTTA GCATTGTGAT AGCTTTTGTG   
  
  
+ ATCATTCTGG TTTTAAAAAA ACAAATTTAA AAGAAATATT TTTAGTTGTG GTTTTAAAAA GTAGATTTTA   
  
  
+ AAAAATACAT GTTTGATTAA ATCTACTATG AGATAAATTT TTACATGTAA AATAAATAAA AACATATTTC   
  
  
+ CATCAATTAA AAAAAAACAT GTTCTTTCAG TTTCTACATG ACTAATATTT AAAATACAAT TACTTATAAA   
  
  
+ GTCCGGTACG AAAGCACCCT CTAAATGGAG AATGAACTGT ATGTATTCTT CCCATGGACC TGTGATTTTT   
  
  
+ CCGTAGGAAA GGCAATTTCC ATTCAGAATA CAAGAAGATA CATGTCTTTT GAAACTCTTA TTAGCTGTCT   
  
  
+ ACCTATCAAC CAAGAATGAC GCTTCTCATC GTATGTCACT ATAAAATTTG GTTGATGACA CCAACACTTT   
  
  
+ CCCATTGGTA TTCATGGCTT TTTAGCTGCA CGATCCAAC  

- AATAGTTTGA AAGTACTACA CTTAGAGTCC AAACTGCCCA ATTGAACCAA ACTTCCCAAT TGGGTCAATT   
  
  
- AAGTTTAAAA AAAAAAGAAG TAATCAAAAA AAAAGAAGGA CAACTAAAAA AAGTAACTTG AATATATAAA   
  
  
- ATTAATATTA TTATATTATT ATTAATGTAT ATGATTTTAT GACTCAACAG AAGAAAAAAA AAATGCAAAA   
  
  
- ATTTAATAAT AAAAAAACAA AAACAAAAAA AGAAACACAA AAAAAATAAA ATTACTTAAA AAAAACAAAT   
  
  
- TAAATCAAAC AATTACAAAT TAAAAAAAAT AAATCAATAG TATAAAAGTA TTATGCCTAG GGCTCAAACT   
  
  
- ACTCAATTAG GTCTTAAAAA CGAAAAGAAG AAAAATTAAT TAAAAAAAAA CAAATCAAAT CAAACAATTA   
  
  
- CAATTTAAAA AAAATAAATT AATAGTCTGA AAATACTGTG CATAGAGCCC AAACTGCCCA ATTGCGCAGT   
  
  
- TAAAAAAAAA GATAAATCAA TAGTTTAAAA ATACTGCGCT TAAAGTCCAA ACTGTCCAAT TGGACCAAAC   
  
  
- TTCCCAATTG GGTAAATTAA GTTAAAAAAA AGAAAAAGAA GTAATCAAAA AAAGAAGGTA AATTAAAAAA   
  
  
- AAGAAAAAGA AGTAATTACA ATAATAATTA TAATATTTAT AGTGAGAACC TAGTCCGCAA CGTCGATTTA   
  
  
- GATTTTGAGT ATCCATATCG AAATATCTTT AAACTTAGAA TCAAAAAAAA ATATAAAAAA TATGCTTTTT   
  
  
- TAATTGGGCA CGTCCATTAC ACTGATCATT ATTGTAATAA AAGACAACTT GGCTTGTTCT TTCTTAGCAT   
  
  
- CAATCCACAG TCTCTGAGTC TCAGCGACGA AGAGTGTAAG TTTGGAACAT TCGCAAACCA GTAACATTCT   
  
  
- GGGAGAGAAA GAAAATAGTT TATTGGAAAG AAATAAAGAA TTTATTTAAT TCCTGGGAAG AAAAAAATAT   
  
  
- TTTTTATTTT TATTTTTTAA GAAGGAAGAA GGGTATTAAC TAAGTTAGCG TTTACTCAGT ACAGTACTGG   
  
  
- GCCTCTGGGA AACTTTTCAG TTTAATCTAT TTCTAACTCT GCTACCAAAG TTCTCTGTGT TGGTGAGTTC   
  
  
- ATAAATGGTA ACACTACTCG ACTGTTCCTA ACAGTAGGTC TTACCAGAGA GGAGTGCTAC AGTGGTCGGT   
  
  
- CACCTTAGGT TTAATTTAAC TTAAATAACC CTTTACCCTA TTGAGAAGGA AAATATATAA TTTTTAATTT   
  
  
- TTTATATATG TCACGGTTAA GATGACATGA CCAATTAATG GGATAGTTGT AAATTCATCA AATACATTGC   
  
  
- AACATAATCA ACAAAAAATT TTATTAAAAA AAAATTATAT AATTTTATTA AAAAAATTTT TTAAATAAAA   
  
  
- ACTATAATTA TACAATTTTG TTAAATTTTT GTAACTTTTT TTTATTTTAT TTTATTTAAG TTCAAAACGT   
  
  
- GTTTTTTTGT GATAACGTGG CGCATGTATT TACTACAGAA TCACATAAAT CGTAACACTA TCGAAAACAC   
  
  
- TAGTAAGACC AAAATTTTTT TGTTTAAATT TTCTTTATAA AAATCAACAC CAAAATTTTT CATCTAAAAT   
  
  
- TTTTTATGTA CAAACTAATT TAGATGATAC TCTATTTAAA AATGTACATT TTATTTATTT TTGTATAAAG   
  
  
- GTAGTTAATT TTTTTTTGTA CAAGAAAGTC AAAGATGTAC TGATTATAAA TTTTATGTTA ATGAATATTT   
  
  
- CAGGCCATGC TTTCGTGGGA GATTTACCTC TTACTTGACA TACATAAGAA GGGTACCTGG ACACTAAAAA   
  
  
- GGCATCCTTT CCGTTAAAGG TAAGTCTTAT GTTCTTCTAT GTACAGAAAA CTTTGAGAAT AATCGACAGA   
  
  
- TGGATAGTTG GTTCTTACTG CGAAGAGTAG CATACAGTGA TATTTTAAAC CAACTACTGT GGTTGTGAAA   
  
  
- GGGTAACCAT AAGTACCGAA AAATCGACGT GCTAGGTTG

+     GCN4\_motif

| Site Name | Organism | Position | Strand | Matrix score. | sequence | function |
| --- | --- | --- | --- | --- | --- | --- |
| GCN4\_motif | Oryza sativa | 1034 | + | 7 | TGAGTCA | cis-regulatory element involved in endosperm expression |

>Potri.004G051700.1   
+ TTATCAAACT TTCATGATGT GAATCTCAGG TTTGACGGGT TAACTTGGTT TGAAGGGTTA ACCCAGTTAA   
  
  
+ TTCAAATTTT TTTTTTCTTC ATTAGTTTTT TTTTCTTCCT GTTGATTTTT TTCATTGAAC TTATATATTT   
  
  
+ TAATTATAAT AATATAATAA TAATTACATA TACTAAAATA CTGAGTTGTC TTCTTTTTTT TTTACGTTTT   
  
  
+ TAAATTATTA TTTTTTTGTT TTTGTTTTTT TCTTTGTGTT TTTTTTATTT TAATGAATTT TTTTTGTTTA   
  
  
+ ATTTAGTTTG TTAATGTTTA ATTTTTTTTA TTTAGTTATC ATATTTTCAT AATACGGATC CCGAGTTTGA   
  
  
+ TGAGTTAATC CAGAATTTTT GCTTTTCTTC TTTTTAATTA ATTTTTTTTT GTTTAGTTTA GTTTGTTAAT   
  
  
+ GTTAAATTTT TTTTATTTAA TTATCAGACT TTTATGACAC GTATCTCGGG TTTGACGGGT TAACGCGTCA   
  
  
+ ATTTTTTTTT CTATTTAGTT ATCAAATTTT TATGACGCGA ATTTCAGGTT TGACAGGTTA ACCTGGTTTG   
  
  
+ AAGGGTTAAC CCATTTAATT CAATTTTTTT TCTTTTTCTT CATTAGTTTT TTTCTTCCAT TTAATTTTTT   
  
  
+ TTCTTTTTCT TCATTAATGT TATTATTAAT ATTATAAATA TCACTCTTGG ATCAGGCGTT GCAGCTAAAT   
  
  
+ CTAAAACTCA TAGGTATAGC TTTATAGAAA TTTGAATCTT AGTTTTTTTT TATATTTTTT ATACGAAAAA   
  
  
+ ATTAACCCGT GCAGGTAATG TGACTAGTAA TAACATTATT TTCTGTTGAA CCGAACAAGA AAGAATCGTA   
  
  
+ GTTAGGTGTC AGAGACTCAG AGTCGCTGCT TCTCACATTC AAACCTTGTA AGCGTTTGGT CATTGTAAGA   
  
  
+ CCCTCTCTTT CTTTTATCAA ATAACCTTTC TTTATTTCTT AAATAAATTA AGGACCCTTC TTTTTTTATA   
  
  
+ AAAAATAAAA ATAAAAAATT CTTCCTTCTT CCCATAATTG ATTCAATCGC AAATGAGTCA TGTCATGACC   
  
  
+ CGGAGACCCT TTGAAAAGTC AAATTAGATA AAGATTGAGA CGATGGTTTC AAGAGACACA ACCACTCAAG   
  
  
+ TATTTACCAT TGTGATGAGC TGACAAGGAT TGTCATCCAG AATGGTCTCT CCTCACGATG TCACCAGCCA   
  
  
+ GTGGAATCCA AATTAAATTG AATTTATTGG GAAATGGGAT AACTCTTCCT TTTATATATT AAAAATTAAA   
  
  
+ AAATATATAC AGTGCCAATT CTACTGTACT GGTTAATTAC CCTATCAACA TTTAAGTAGT TTATGTAACG   
  
  
+ TTGTATTAGT TGTTTTTTAA AATAATTTTT TTTTAATATA TTAAAATAAT TTTTTTAAAA AATTTATTTT   
  
  
+ TGATATTAAT ATGTTAAAAC AATTTAAAAA CATTGAAAAA AAATAAAATA AAATAAATTC AAGTTTTGCA   
  
  
+ CAAAAAAACA CTATTGCACC GCGTACATAA ATGATGTCTT AGTGTATTTA GCATTGTGAT AGCTTTTGTG   
  
  
+ ATCATTCTGG TTTTAAAAAA ACAAATTTAA AAGAAATATT TTTAGTTGTG GTTTTAAAAA GTAGATTTTA   
  
  
+ AAAAATACAT GTTTGATTAA ATCTACTATG AGATAAATTT TTACATGTAA AATAAATAAA AACATATTTC   
  
  
+ CATCAATTAA AAAAAAACAT GTTCTTTCAG TTTCTACATG ACTAATATTT AAAATACAAT TACTTATAAA   
  
  
+ GTCCGGTACG AAAGCACCCT CTAAATGGAG AATGAACTGT ATGTATTCTT CCCATGGACC TGTGATTTTT   
  
  
+ CCGTAGGAAA GGCAATTTCC ATTCAGAATA CAAGAAGATA CATGTCTTTT GAAACTCTTA TTAGCTGTCT   
  
  
+ ACCTATCAAC CAAGAATGAC GCTTCTCATC GTATGTCACT ATAAAATTTG GTTGATGACA CCAACACTTT   
  
  
+ CCCATTGGTA TTCATGGCTT TTTAGCTGCA CGATCCAAC  

- AATAGTTTGA AAGTACTACA CTTAGAGTCC AAACTGCCCA ATTGAACCAA ACTTCCCAAT TGGGTCAATT   
  
  
- AAGTTTAAAA AAAAAAGAAG TAATCAAAAA AAAAGAAGGA CAACTAAAAA AAGTAACTTG AATATATAAA   
  
  
- ATTAATATTA TTATATTATT ATTAATGTAT ATGATTTTAT GACTCAACAG AAGAAAAAAA AAATGCAAAA   
  
  
- ATTTAATAAT AAAAAAACAA AAACAAAAAA AGAAACACAA AAAAAATAAA ATTACTTAAA AAAAACAAAT   
  
  
- TAAATCAAAC AATTACAAAT TAAAAAAAAT AAATCAATAG TATAAAAGTA TTATGCCTAG GGCTCAAACT   
  
  
- ACTCAATTAG GTCTTAAAAA CGAAAAGAAG AAAAATTAAT TAAAAAAAAA CAAATCAAAT CAAACAATTA   
  
  
- CAATTTAAAA AAAATAAATT AATAGTCTGA AAATACTGTG CATAGAGCCC AAACTGCCCA ATTGCGCAGT   
  
  
- TAAAAAAAAA GATAAATCAA TAGTTTAAAA ATACTGCGCT TAAAGTCCAA ACTGTCCAAT TGGACCAAAC   
  
  
- TTCCCAATTG GGTAAATTAA GTTAAAAAAA AGAAAAAGAA GTAATCAAAA AAAGAAGGTA AATTAAAAAA   
  
  
- AAGAAAAAGA AGTAATTACA ATAATAATTA TAATATTTAT AGTGAGAACC TAGTCCGCAA CGTCGATTTA   
  
  
- GATTTTGAGT ATCCATATCG AAATATCTTT AAACTTAGAA TCAAAAAAAA ATATAAAAAA TATGCTTTTT   
  
  
- TAATTGGGCA CGTCCATTAC ACTGATCATT ATTGTAATAA AAGACAACTT GGCTTGTTCT TTCTTAGCAT   
  
  
- CAATCCACAG TCTCTGAGTC TCAGCGACGA AGAGTGTAAG TTTGGAACAT TCGCAAACCA GTAACATTCT   
  
  
- GGGAGAGAAA GAAAATAGTT TATTGGAAAG AAATAAAGAA TTTATTTAAT TCCTGGGAAG AAAAAAATAT   
  
  
- TTTTTATTTT TATTTTTTAA GAAGGAAGAA GGGTATTAAC TAAGTTAGCG TTTACTCAGT ACAGTACTGG   
  
  
- GCCTCTGGGA AACTTTTCAG TTTAATCTAT TTCTAACTCT GCTACCAAAG TTCTCTGTGT TGGTGAGTTC   
  
  
- ATAAATGGTA ACACTACTCG ACTGTTCCTA ACAGTAGGTC TTACCAGAGA GGAGTGCTAC AGTGGTCGGT   
  
  
- CACCTTAGGT TTAATTTAAC TTAAATAACC CTTTACCCTA TTGAGAAGGA AAATATATAA TTTTTAATTT   
  
  
- TTTATATATG TCACGGTTAA GATGACATGA CCAATTAATG GGATAGTTGT AAATTCATCA AATACATTGC   
  
  
- AACATAATCA ACAAAAAATT TTATTAAAAA AAAATTATAT AATTTTATTA AAAAAATTTT TTAAATAAAA   
  
  
- ACTATAATTA TACAATTTTG TTAAATTTTT GTAACTTTTT TTTATTTTAT TTTATTTAAG TTCAAAACGT   
  
  
- GTTTTTTTGT GATAACGTGG CGCATGTATT TACTACAGAA TCACATAAAT CGTAACACTA TCGAAAACAC   
  
  
- TAGTAAGACC AAAATTTTTT TGTTTAAATT TTCTTTATAA AAATCAACAC CAAAATTTTT CATCTAAAAT   
  
  
- TTTTTATGTA CAAACTAATT TAGATGATAC TCTATTTAAA AATGTACATT TTATTTATTT TTGTATAAAG   
  
  
- GTAGTTAATT TTTTTTTGTA CAAGAAAGTC AAAGATGTAC TGATTATAAA TTTTATGTTA ATGAATATTT   
  
  
- CAGGCCATGC TTTCGTGGGA GATTTACCTC TTACTTGACA TACATAAGAA GGGTACCTGG ACACTAAAAA   
  
  
- GGCATCCTTT CCGTTAAAGG TAAGTCTTAT GTTCTTCTAT GTACAGAAAA CTTTGAGAAT AATCGACAGA   
  
  
- TGGATAGTTG GTTCTTACTG CGAAGAGTAG CATACAGTGA TATTTTAAAC CAACTACTGT GGTTGTGAAA   
  
  
- GGGTAACCAT AAGTACCGAA AAATCGACGT GCTAGGTTG

+     GT1-motif

| Site Name | Organism | Position | Strand | Matrix score. | sequence | function |
| --- | --- | --- | --- | --- | --- | --- |
| GT1-motif | Avena sativa | 1291 | + | 7 | GGTTAAT | light responsive element |
| GT1-motif | Arabidopsis thaliana | 566 | - | 6 | GGTTAA | light responsive element |
| GT1-motif | Arabidopsis thaliana | 564 | + | 6 | GGTTAA | light responsive element |
| GT1-motif | Arabidopsis thaliana | 772 | - | 6 | GGTTAA | light responsive element |
| GT1-motif | Avena sativa | 771 | - | 7 | GGTTAAT | light responsive element |
| GT1-motif | Arabidopsis thaliana | 548 | - | 6 | GGTTAA | light responsive element |
| GT1-motif | Arabidopsis thaliana | 546 | + | 6 | GGTTAA | light responsive element |
| GT1-motif | Arabidopsis thaliana | 58 | - | 6 | GGTTAA | light responsive element |
| GT1-motif | Arabidopsis thaliana | 56 | + | 6 | GGTTAA | light responsive element |
| GT1-motif | Arabidopsis thaliana | 38 | + | 6 | GGTTAA | light responsive element |
| GT1-motif | Arabidopsis thaliana | 478 | + | 6 | GGTTAA | light responsive element |

>Potri.004G051700.1   
+ TTATCAAACT TTCATGATGT GAATCTCAGG TTTGACGGGT TAACTTGGTT TGAAGGGTTA ACCCAGTTAA   
  
  
+ TTCAAATTTT TTTTTTCTTC ATTAGTTTTT TTTTCTTCCT GTTGATTTTT TTCATTGAAC TTATATATTT   
  
  
+ TAATTATAAT AATATAATAA TAATTACATA TACTAAAATA CTGAGTTGTC TTCTTTTTTT TTTACGTTTT   
  
  
+ TAAATTATTA TTTTTTTGTT TTTGTTTTTT TCTTTGTGTT TTTTTTATTT TAATGAATTT TTTTTGTTTA   
  
  
+ ATTTAGTTTG TTAATGTTTA ATTTTTTTTA TTTAGTTATC ATATTTTCAT AATACGGATC CCGAGTTTGA   
  
  
+ TGAGTTAATC CAGAATTTTT GCTTTTCTTC TTTTTAATTA ATTTTTTTTT GTTTAGTTTA GTTTGTTAAT   
  
  
+ GTTAAATTTT TTTTATTTAA TTATCAGACT TTTATGACAC GTATCTCGGG TTTGACGGGT TAACGCGTCA   
  
  
+ ATTTTTTTTT CTATTTAGTT ATCAAATTTT TATGACGCGA ATTTCAGGTT TGACAGGTTA ACCTGGTTTG   
  
  
+ AAGGGTTAAC CCATTTAATT CAATTTTTTT TCTTTTTCTT CATTAGTTTT TTTCTTCCAT TTAATTTTTT   
  
  
+ TTCTTTTTCT TCATTAATGT TATTATTAAT ATTATAAATA TCACTCTTGG ATCAGGCGTT GCAGCTAAAT   
  
  
+ CTAAAACTCA TAGGTATAGC TTTATAGAAA TTTGAATCTT AGTTTTTTTT TATATTTTTT ATACGAAAAA   
  
  
+ ATTAACCCGT GCAGGTAATG TGACTAGTAA TAACATTATT TTCTGTTGAA CCGAACAAGA AAGAATCGTA   
  
  
+ GTTAGGTGTC AGAGACTCAG AGTCGCTGCT TCTCACATTC AAACCTTGTA AGCGTTTGGT CATTGTAAGA   
  
  
+ CCCTCTCTTT CTTTTATCAA ATAACCTTTC TTTATTTCTT AAATAAATTA AGGACCCTTC TTTTTTTATA   
  
  
+ AAAAATAAAA ATAAAAAATT CTTCCTTCTT CCCATAATTG ATTCAATCGC AAATGAGTCA TGTCATGACC   
  
  
+ CGGAGACCCT TTGAAAAGTC AAATTAGATA AAGATTGAGA CGATGGTTTC AAGAGACACA ACCACTCAAG   
  
  
+ TATTTACCAT TGTGATGAGC TGACAAGGAT TGTCATCCAG AATGGTCTCT CCTCACGATG TCACCAGCCA   
  
  
+ GTGGAATCCA AATTAAATTG AATTTATTGG GAAATGGGAT AACTCTTCCT TTTATATATT AAAAATTAAA   
  
  
+ AAATATATAC AGTGCCAATT CTACTGTACT GGTTAATTAC CCTATCAACA TTTAAGTAGT TTATGTAACG   
  
  
+ TTGTATTAGT TGTTTTTTAA AATAATTTTT TTTTAATATA TTAAAATAAT TTTTTTAAAA AATTTATTTT   
  
  
+ TGATATTAAT ATGTTAAAAC AATTTAAAAA CATTGAAAAA AAATAAAATA AAATAAATTC AAGTTTTGCA   
  
  
+ CAAAAAAACA CTATTGCACC GCGTACATAA ATGATGTCTT AGTGTATTTA GCATTGTGAT AGCTTTTGTG   
  
  
+ ATCATTCTGG TTTTAAAAAA ACAAATTTAA AAGAAATATT TTTAGTTGTG GTTTTAAAAA GTAGATTTTA   
  
  
+ AAAAATACAT GTTTGATTAA ATCTACTATG AGATAAATTT TTACATGTAA AATAAATAAA AACATATTTC   
  
  
+ CATCAATTAA AAAAAAACAT GTTCTTTCAG TTTCTACATG ACTAATATTT AAAATACAAT TACTTATAAA   
  
  
+ GTCCGGTACG AAAGCACCCT CTAAATGGAG AATGAACTGT ATGTATTCTT CCCATGGACC TGTGATTTTT   
  
  
+ CCGTAGGAAA GGCAATTTCC ATTCAGAATA CAAGAAGATA CATGTCTTTT GAAACTCTTA TTAGCTGTCT   
  
  
+ ACCTATCAAC CAAGAATGAC GCTTCTCATC GTATGTCACT ATAAAATTTG GTTGATGACA CCAACACTTT   
  
  
+ CCCATTGGTA TTCATGGCTT TTTAGCTGCA CGATCCAAC  

- AATAGTTTGA AAGTACTACA CTTAGAGTCC AAACTGCCCA ATTGAACCAA ACTTCCCAAT TGGGTCAATT   
  
  
- AAGTTTAAAA AAAAAAGAAG TAATCAAAAA AAAAGAAGGA CAACTAAAAA AAGTAACTTG AATATATAAA   
  
  
- ATTAATATTA TTATATTATT ATTAATGTAT ATGATTTTAT GACTCAACAG AAGAAAAAAA AAATGCAAAA   
  
  
- ATTTAATAAT AAAAAAACAA AAACAAAAAA AGAAACACAA AAAAAATAAA ATTACTTAAA AAAAACAAAT   
  
  
- TAAATCAAAC AATTACAAAT TAAAAAAAAT AAATCAATAG TATAAAAGTA TTATGCCTAG GGCTCAAACT   
  
  
- ACTCAATTAG GTCTTAAAAA CGAAAAGAAG AAAAATTAAT TAAAAAAAAA CAAATCAAAT CAAACAATTA   
  
  
- CAATTTAAAA AAAATAAATT AATAGTCTGA AAATACTGTG CATAGAGCCC AAACTGCCCA ATTGCGCAGT   
  
  
- TAAAAAAAAA GATAAATCAA TAGTTTAAAA ATACTGCGCT TAAAGTCCAA ACTGTCCAAT TGGACCAAAC   
  
  
- TTCCCAATTG GGTAAATTAA GTTAAAAAAA AGAAAAAGAA GTAATCAAAA AAAGAAGGTA AATTAAAAAA   
  
  
- AAGAAAAAGA AGTAATTACA ATAATAATTA TAATATTTAT AGTGAGAACC TAGTCCGCAA CGTCGATTTA   
  
  
- GATTTTGAGT ATCCATATCG AAATATCTTT AAACTTAGAA TCAAAAAAAA ATATAAAAAA TATGCTTTTT   
  
  
- TAATTGGGCA CGTCCATTAC ACTGATCATT ATTGTAATAA AAGACAACTT GGCTTGTTCT TTCTTAGCAT   
  
  
- CAATCCACAG TCTCTGAGTC TCAGCGACGA AGAGTGTAAG TTTGGAACAT TCGCAAACCA GTAACATTCT   
  
  
- GGGAGAGAAA GAAAATAGTT TATTGGAAAG AAATAAAGAA TTTATTTAAT TCCTGGGAAG AAAAAAATAT   
  
  
- TTTTTATTTT TATTTTTTAA GAAGGAAGAA GGGTATTAAC TAAGTTAGCG TTTACTCAGT ACAGTACTGG   
  
  
- GCCTCTGGGA AACTTTTCAG TTTAATCTAT TTCTAACTCT GCTACCAAAG TTCTCTGTGT TGGTGAGTTC   
  
  
- ATAAATGGTA ACACTACTCG ACTGTTCCTA ACAGTAGGTC TTACCAGAGA GGAGTGCTAC AGTGGTCGGT   
  
  
- CACCTTAGGT TTAATTTAAC TTAAATAACC CTTTACCCTA TTGAGAAGGA AAATATATAA TTTTTAATTT   
  
  
- TTTATATATG TCACGGTTAA GATGACATGA CCAATTAATG GGATAGTTGT AAATTCATCA AATACATTGC   
  
  
- AACATAATCA ACAAAAAATT TTATTAAAAA AAAATTATAT AATTTTATTA AAAAAATTTT TTAAATAAAA   
  
  
- ACTATAATTA TACAATTTTG TTAAATTTTT GTAACTTTTT TTTATTTTAT TTTATTTAAG TTCAAAACGT   
  
  
- GTTTTTTTGT GATAACGTGG CGCATGTATT TACTACAGAA TCACATAAAT CGTAACACTA TCGAAAACAC   
  
  
- TAGTAAGACC AAAATTTTTT TGTTTAAATT TTCTTTATAA AAATCAACAC CAAAATTTTT CATCTAAAAT   
  
  
- TTTTTATGTA CAAACTAATT TAGATGATAC TCTATTTAAA AATGTACATT TTATTTATTT TTGTATAAAG   
  
  
- GTAGTTAATT TTTTTTTGTA CAAGAAAGTC AAAGATGTAC TGATTATAAA TTTTATGTTA ATGAATATTT   
  
  
- CAGGCCATGC TTTCGTGGGA GATTTACCTC TTACTTGACA TACATAAGAA GGGTACCTGG ACACTAAAAA   
  
  
- GGCATCCTTT CCGTTAAAGG TAAGTCTTAT GTTCTTCTAT GTACAGAAAA CTTTGAGAAT AATCGACAGA   
  
  
- TGGATAGTTG GTTCTTACTG CGAAGAGTAG CATACAGTGA TATTTTAAAC CAACTACTGT GGTTGTGAAA   
  
  
- GGGTAACCAT AAGTACCGAA AAATCGACGT GCTAGGTTG

+     MYB

| Site Name | Organism | Position | Strand | Matrix score. | sequence | function |
| --- | --- | --- | --- | --- | --- | --- |
| MYB | Arabidopsis thaliana | 1939 | - | 6 | CAACCA |  |
| MYB | Arabidopsis thaliana | 1897 | + | 6 | CAACCA |  |
| MYB | Arabidopsis thaliana | 1290 | - | 6 | TAACCA |  |
| MYB | Arabidopsis thaliana | 1109 | + | 6 | CAACCA |  |
| MYB | Arabidopsis thaliana | 813 | - | 6 | CAACAG |  |
| MYB | Arabidopsis thaliana | 109 | - | 6 | CAACAG |  |

>Potri.004G051700.1   
+ TTATCAAACT TTCATGATGT GAATCTCAGG TTTGACGGGT TAACTTGGTT TGAAGGGTTA ACCCAGTTAA   
  
  
+ TTCAAATTTT TTTTTTCTTC ATTAGTTTTT TTTTCTTCCT GTTGATTTTT TTCATTGAAC TTATATATTT   
  
  
+ TAATTATAAT AATATAATAA TAATTACATA TACTAAAATA CTGAGTTGTC TTCTTTTTTT TTTACGTTTT   
  
  
+ TAAATTATTA TTTTTTTGTT TTTGTTTTTT TCTTTGTGTT TTTTTTATTT TAATGAATTT TTTTTGTTTA   
  
  
+ ATTTAGTTTG TTAATGTTTA ATTTTTTTTA TTTAGTTATC ATATTTTCAT AATACGGATC CCGAGTTTGA   
  
  
+ TGAGTTAATC CAGAATTTTT GCTTTTCTTC TTTTTAATTA ATTTTTTTTT GTTTAGTTTA GTTTGTTAAT   
  
  
+ GTTAAATTTT TTTTATTTAA TTATCAGACT TTTATGACAC GTATCTCGGG TTTGACGGGT TAACGCGTCA   
  
  
+ ATTTTTTTTT CTATTTAGTT ATCAAATTTT TATGACGCGA ATTTCAGGTT TGACAGGTTA ACCTGGTTTG   
  
  
+ AAGGGTTAAC CCATTTAATT CAATTTTTTT TCTTTTTCTT CATTAGTTTT TTTCTTCCAT TTAATTTTTT   
  
  
+ TTCTTTTTCT TCATTAATGT TATTATTAAT ATTATAAATA TCACTCTTGG ATCAGGCGTT GCAGCTAAAT   
  
  
+ CTAAAACTCA TAGGTATAGC TTTATAGAAA TTTGAATCTT AGTTTTTTTT TATATTTTTT ATACGAAAAA   
  
  
+ ATTAACCCGT GCAGGTAATG TGACTAGTAA TAACATTATT TTCTGTTGAA CCGAACAAGA AAGAATCGTA   
  
  
+ GTTAGGTGTC AGAGACTCAG AGTCGCTGCT TCTCACATTC AAACCTTGTA AGCGTTTGGT CATTGTAAGA   
  
  
+ CCCTCTCTTT CTTTTATCAA ATAACCTTTC TTTATTTCTT AAATAAATTA AGGACCCTTC TTTTTTTATA   
  
  
+ AAAAATAAAA ATAAAAAATT CTTCCTTCTT CCCATAATTG ATTCAATCGC AAATGAGTCA TGTCATGACC   
  
  
+ CGGAGACCCT TTGAAAAGTC AAATTAGATA AAGATTGAGA CGATGGTTTC AAGAGACACA ACCACTCAAG   
  
  
+ TATTTACCAT TGTGATGAGC TGACAAGGAT TGTCATCCAG AATGGTCTCT CCTCACGATG TCACCAGCCA   
  
  
+ GTGGAATCCA AATTAAATTG AATTTATTGG GAAATGGGAT AACTCTTCCT TTTATATATT AAAAATTAAA   
  
  
+ AAATATATAC AGTGCCAATT CTACTGTACT GGTTAATTAC CCTATCAACA TTTAAGTAGT TTATGTAACG   
  
  
+ TTGTATTAGT TGTTTTTTAA AATAATTTTT TTTTAATATA TTAAAATAAT TTTTTTAAAA AATTTATTTT   
  
  
+ TGATATTAAT ATGTTAAAAC AATTTAAAAA CATTGAAAAA AAATAAAATA AAATAAATTC AAGTTTTGCA   
  
  
+ CAAAAAAACA CTATTGCACC GCGTACATAA ATGATGTCTT AGTGTATTTA GCATTGTGAT AGCTTTTGTG   
  
  
+ ATCATTCTGG TTTTAAAAAA ACAAATTTAA AAGAAATATT TTTAGTTGTG GTTTTAAAAA GTAGATTTTA   
  
  
+ AAAAATACAT GTTTGATTAA ATCTACTATG AGATAAATTT TTACATGTAA AATAAATAAA AACATATTTC   
  
  
+ CATCAATTAA AAAAAAACAT GTTCTTTCAG TTTCTACATG ACTAATATTT AAAATACAAT TACTTATAAA   
  
  
+ GTCCGGTACG AAAGCACCCT CTAAATGGAG AATGAACTGT ATGTATTCTT CCCATGGACC TGTGATTTTT   
  
  
+ CCGTAGGAAA GGCAATTTCC ATTCAGAATA CAAGAAGATA CATGTCTTTT GAAACTCTTA TTAGCTGTCT   
  
  
+ ACCTATCAAC CAAGAATGAC GCTTCTCATC GTATGTCACT ATAAAATTTG GTTGATGACA CCAACACTTT   
  
  
+ CCCATTGGTA TTCATGGCTT TTTAGCTGCA CGATCCAAC  

- AATAGTTTGA AAGTACTACA CTTAGAGTCC AAACTGCCCA ATTGAACCAA ACTTCCCAAT TGGGTCAATT   
  
  
- AAGTTTAAAA AAAAAAGAAG TAATCAAAAA AAAAGAAGGA CAACTAAAAA AAGTAACTTG AATATATAAA   
  
  
- ATTAATATTA TTATATTATT ATTAATGTAT ATGATTTTAT GACTCAACAG AAGAAAAAAA AAATGCAAAA   
  
  
- ATTTAATAAT AAAAAAACAA AAACAAAAAA AGAAACACAA AAAAAATAAA ATTACTTAAA AAAAACAAAT   
  
  
- TAAATCAAAC AATTACAAAT TAAAAAAAAT AAATCAATAG TATAAAAGTA TTATGCCTAG GGCTCAAACT   
  
  
- ACTCAATTAG GTCTTAAAAA CGAAAAGAAG AAAAATTAAT TAAAAAAAAA CAAATCAAAT CAAACAATTA   
  
  
- CAATTTAAAA AAAATAAATT AATAGTCTGA AAATACTGTG CATAGAGCCC AAACTGCCCA ATTGCGCAGT   
  
  
- TAAAAAAAAA GATAAATCAA TAGTTTAAAA ATACTGCGCT TAAAGTCCAA ACTGTCCAAT TGGACCAAAC   
  
  
- TTCCCAATTG GGTAAATTAA GTTAAAAAAA AGAAAAAGAA GTAATCAAAA AAAGAAGGTA AATTAAAAAA   
  
  
- AAGAAAAAGA AGTAATTACA ATAATAATTA TAATATTTAT AGTGAGAACC TAGTCCGCAA CGTCGATTTA   
  
  
- GATTTTGAGT ATCCATATCG AAATATCTTT AAACTTAGAA TCAAAAAAAA ATATAAAAAA TATGCTTTTT   
  
  
- TAATTGGGCA CGTCCATTAC ACTGATCATT ATTGTAATAA AAGACAACTT GGCTTGTTCT TTCTTAGCAT   
  
  
- CAATCCACAG TCTCTGAGTC TCAGCGACGA AGAGTGTAAG TTTGGAACAT TCGCAAACCA GTAACATTCT   
  
  
- GGGAGAGAAA GAAAATAGTT TATTGGAAAG AAATAAAGAA TTTATTTAAT TCCTGGGAAG AAAAAAATAT   
  
  
- TTTTTATTTT TATTTTTTAA GAAGGAAGAA GGGTATTAAC TAAGTTAGCG TTTACTCAGT ACAGTACTGG   
  
  
- GCCTCTGGGA AACTTTTCAG TTTAATCTAT TTCTAACTCT GCTACCAAAG TTCTCTGTGT TGGTGAGTTC   
  
  
- ATAAATGGTA ACACTACTCG ACTGTTCCTA ACAGTAGGTC TTACCAGAGA GGAGTGCTAC AGTGGTCGGT   
  
  
- CACCTTAGGT TTAATTTAAC TTAAATAACC CTTTACCCTA TTGAGAAGGA AAATATATAA TTTTTAATTT   
  
  
- TTTATATATG TCACGGTTAA GATGACATGA CCAATTAATG GGATAGTTGT AAATTCATCA AATACATTGC   
  
  
- AACATAATCA ACAAAAAATT TTATTAAAAA AAAATTATAT AATTTTATTA AAAAAATTTT TTAAATAAAA   
  
  
- ACTATAATTA TACAATTTTG TTAAATTTTT GTAACTTTTT TTTATTTTAT TTTATTTAAG TTCAAAACGT   
  
  
- GTTTTTTTGT GATAACGTGG CGCATGTATT TACTACAGAA TCACATAAAT CGTAACACTA TCGAAAACAC   
  
  
- TAGTAAGACC AAAATTTTTT TGTTTAAATT TTCTTTATAA AAATCAACAC CAAAATTTTT CATCTAAAAT   
  
  
- TTTTTATGTA CAAACTAATT TAGATGATAC TCTATTTAAA AATGTACATT TTATTTATTT TTGTATAAAG   
  
  
- GTAGTTAATT TTTTTTTGTA CAAGAAAGTC AAAGATGTAC TGATTATAAA TTTTATGTTA ATGAATATTT   
  
  
- CAGGCCATGC TTTCGTGGGA GATTTACCTC TTACTTGACA TACATAAGAA GGGTACCTGG ACACTAAAAA   
  
  
- GGCATCCTTT CCGTTAAAGG TAAGTCTTAT GTTCTTCTAT GTACAGAAAA CTTTGAGAAT AATCGACAGA   
  
  
- TGGATAGTTG GTTCTTACTG CGAAGAGTAG CATACAGTGA TATTTTAAAC CAACTACTGT GGTTGTGAAA   
  
  
- GGGTAACCAT AAGTACCGAA AAATCGACGT GCTAGGTTG

+     MYB-like sequence

| Site Name | Organism | Position | Strand | Matrix score. | sequence | function |
| --- | --- | --- | --- | --- | --- | --- |
| MYB-like sequence | Arabidopsis thaliana | 1290 | - | 6 | TAACCA |  |

>Potri.004G051700.1   
+ TTATCAAACT TTCATGATGT GAATCTCAGG TTTGACGGGT TAACTTGGTT TGAAGGGTTA ACCCAGTTAA   
  
  
+ TTCAAATTTT TTTTTTCTTC ATTAGTTTTT TTTTCTTCCT GTTGATTTTT TTCATTGAAC TTATATATTT   
  
  
+ TAATTATAAT AATATAATAA TAATTACATA TACTAAAATA CTGAGTTGTC TTCTTTTTTT TTTACGTTTT   
  
  
+ TAAATTATTA TTTTTTTGTT TTTGTTTTTT TCTTTGTGTT TTTTTTATTT TAATGAATTT TTTTTGTTTA   
  
  
+ ATTTAGTTTG TTAATGTTTA ATTTTTTTTA TTTAGTTATC ATATTTTCAT AATACGGATC CCGAGTTTGA   
  
  
+ TGAGTTAATC CAGAATTTTT GCTTTTCTTC TTTTTAATTA ATTTTTTTTT GTTTAGTTTA GTTTGTTAAT   
  
  
+ GTTAAATTTT TTTTATTTAA TTATCAGACT TTTATGACAC GTATCTCGGG TTTGACGGGT TAACGCGTCA   
  
  
+ ATTTTTTTTT CTATTTAGTT ATCAAATTTT TATGACGCGA ATTTCAGGTT TGACAGGTTA ACCTGGTTTG   
  
  
+ AAGGGTTAAC CCATTTAATT CAATTTTTTT TCTTTTTCTT CATTAGTTTT TTTCTTCCAT TTAATTTTTT   
  
  
+ TTCTTTTTCT TCATTAATGT TATTATTAAT ATTATAAATA TCACTCTTGG ATCAGGCGTT GCAGCTAAAT   
  
  
+ CTAAAACTCA TAGGTATAGC TTTATAGAAA TTTGAATCTT AGTTTTTTTT TATATTTTTT ATACGAAAAA   
  
  
+ ATTAACCCGT GCAGGTAATG TGACTAGTAA TAACATTATT TTCTGTTGAA CCGAACAAGA AAGAATCGTA   
  
  
+ GTTAGGTGTC AGAGACTCAG AGTCGCTGCT TCTCACATTC AAACCTTGTA AGCGTTTGGT CATTGTAAGA   
  
  
+ CCCTCTCTTT CTTTTATCAA ATAACCTTTC TTTATTTCTT AAATAAATTA AGGACCCTTC TTTTTTTATA   
  
  
+ AAAAATAAAA ATAAAAAATT CTTCCTTCTT CCCATAATTG ATTCAATCGC AAATGAGTCA TGTCATGACC   
  
  
+ CGGAGACCCT TTGAAAAGTC AAATTAGATA AAGATTGAGA CGATGGTTTC AAGAGACACA ACCACTCAAG   
  
  
+ TATTTACCAT TGTGATGAGC TGACAAGGAT TGTCATCCAG AATGGTCTCT CCTCACGATG TCACCAGCCA   
  
  
+ GTGGAATCCA AATTAAATTG AATTTATTGG GAAATGGGAT AACTCTTCCT TTTATATATT AAAAATTAAA   
  
  
+ AAATATATAC AGTGCCAATT CTACTGTACT GGTTAATTAC CCTATCAACA TTTAAGTAGT TTATGTAACG   
  
  
+ TTGTATTAGT TGTTTTTTAA AATAATTTTT TTTTAATATA TTAAAATAAT TTTTTTAAAA AATTTATTTT   
  
  
+ TGATATTAAT ATGTTAAAAC AATTTAAAAA CATTGAAAAA AAATAAAATA AAATAAATTC AAGTTTTGCA   
  
  
+ CAAAAAAACA CTATTGCACC GCGTACATAA ATGATGTCTT AGTGTATTTA GCATTGTGAT AGCTTTTGTG   
  
  
+ ATCATTCTGG TTTTAAAAAA ACAAATTTAA AAGAAATATT TTTAGTTGTG GTTTTAAAAA GTAGATTTTA   
  
  
+ AAAAATACAT GTTTGATTAA ATCTACTATG AGATAAATTT TTACATGTAA AATAAATAAA AACATATTTC   
  
  
+ CATCAATTAA AAAAAAACAT GTTCTTTCAG TTTCTACATG ACTAATATTT AAAATACAAT TACTTATAAA   
  
  
+ GTCCGGTACG AAAGCACCCT CTAAATGGAG AATGAACTGT ATGTATTCTT CCCATGGACC TGTGATTTTT   
  
  
+ CCGTAGGAAA GGCAATTTCC ATTCAGAATA CAAGAAGATA CATGTCTTTT GAAACTCTTA TTAGCTGTCT   
  
  
+ ACCTATCAAC CAAGAATGAC GCTTCTCATC GTATGTCACT ATAAAATTTG GTTGATGACA CCAACACTTT   
  
  
+ CCCATTGGTA TTCATGGCTT TTTAGCTGCA CGATCCAAC  

- AATAGTTTGA AAGTACTACA CTTAGAGTCC AAACTGCCCA ATTGAACCAA ACTTCCCAAT TGGGTCAATT   
  
  
- AAGTTTAAAA AAAAAAGAAG TAATCAAAAA AAAAGAAGGA CAACTAAAAA AAGTAACTTG AATATATAAA   
  
  
- ATTAATATTA TTATATTATT ATTAATGTAT ATGATTTTAT GACTCAACAG AAGAAAAAAA AAATGCAAAA   
  
  
- ATTTAATAAT AAAAAAACAA AAACAAAAAA AGAAACACAA AAAAAATAAA ATTACTTAAA AAAAACAAAT   
  
  
- TAAATCAAAC AATTACAAAT TAAAAAAAAT AAATCAATAG TATAAAAGTA TTATGCCTAG GGCTCAAACT   
  
  
- ACTCAATTAG GTCTTAAAAA CGAAAAGAAG AAAAATTAAT TAAAAAAAAA CAAATCAAAT CAAACAATTA   
  
  
- CAATTTAAAA AAAATAAATT AATAGTCTGA AAATACTGTG CATAGAGCCC AAACTGCCCA ATTGCGCAGT   
  
  
- TAAAAAAAAA GATAAATCAA TAGTTTAAAA ATACTGCGCT TAAAGTCCAA ACTGTCCAAT TGGACCAAAC   
  
  
- TTCCCAATTG GGTAAATTAA GTTAAAAAAA AGAAAAAGAA GTAATCAAAA AAAGAAGGTA AATTAAAAAA   
  
  
- AAGAAAAAGA AGTAATTACA ATAATAATTA TAATATTTAT AGTGAGAACC TAGTCCGCAA CGTCGATTTA   
  
  
- GATTTTGAGT ATCCATATCG AAATATCTTT AAACTTAGAA TCAAAAAAAA ATATAAAAAA TATGCTTTTT   
  
  
- TAATTGGGCA CGTCCATTAC ACTGATCATT ATTGTAATAA AAGACAACTT GGCTTGTTCT TTCTTAGCAT   
  
  
- CAATCCACAG TCTCTGAGTC TCAGCGACGA AGAGTGTAAG TTTGGAACAT TCGCAAACCA GTAACATTCT   
  
  
- GGGAGAGAAA GAAAATAGTT TATTGGAAAG AAATAAAGAA TTTATTTAAT TCCTGGGAAG AAAAAAATAT   
  
  
- TTTTTATTTT TATTTTTTAA GAAGGAAGAA GGGTATTAAC TAAGTTAGCG TTTACTCAGT ACAGTACTGG   
  
  
- GCCTCTGGGA AACTTTTCAG TTTAATCTAT TTCTAACTCT GCTACCAAAG TTCTCTGTGT TGGTGAGTTC   
  
  
- ATAAATGGTA ACACTACTCG ACTGTTCCTA ACAGTAGGTC TTACCAGAGA GGAGTGCTAC AGTGGTCGGT   
  
  
- CACCTTAGGT TTAATTTAAC TTAAATAACC CTTTACCCTA TTGAGAAGGA AAATATATAA TTTTTAATTT   
  
  
- TTTATATATG TCACGGTTAA GATGACATGA CCAATTAATG GGATAGTTGT AAATTCATCA AATACATTGC   
  
  
- AACATAATCA ACAAAAAATT TTATTAAAAA AAAATTATAT AATTTTATTA AAAAAATTTT TTAAATAAAA   
  
  
- ACTATAATTA TACAATTTTG TTAAATTTTT GTAACTTTTT TTTATTTTAT TTTATTTAAG TTCAAAACGT   
  
  
- GTTTTTTTGT GATAACGTGG CGCATGTATT TACTACAGAA TCACATAAAT CGTAACACTA TCGAAAACAC   
  
  
- TAGTAAGACC AAAATTTTTT TGTTTAAATT TTCTTTATAA AAATCAACAC CAAAATTTTT CATCTAAAAT   
  
  
- TTTTTATGTA CAAACTAATT TAGATGATAC TCTATTTAAA AATGTACATT TTATTTATTT TTGTATAAAG   
  
  
- GTAGTTAATT TTTTTTTGTA CAAGAAAGTC AAAGATGTAC TGATTATAAA TTTTATGTTA ATGAATATTT   
  
  
- CAGGCCATGC TTTCGTGGGA GATTTACCTC TTACTTGACA TACATAAGAA GGGTACCTGG ACACTAAAAA   
  
  
- GGCATCCTTT CCGTTAAAGG TAAGTCTTAT GTTCTTCTAT GTACAGAAAA CTTTGAGAAT AATCGACAGA   
  
  
- TGGATAGTTG GTTCTTACTG CGAAGAGTAG CATACAGTGA TATTTTAAAC CAACTACTGT GGTTGTGAAA   
  
  
- GGGTAACCAT AAGTACCGAA AAATCGACGT GCTAGGTTG

+     MYC

| Site Name | Organism | Position | Strand | Matrix score. | sequence | function |
| --- | --- | --- | --- | --- | --- | --- |
| MYC | Arabidopsis thaliana | 1030 | - | 6 | CATTTG |  |

>Potri.004G051700.1   
+ TTATCAAACT TTCATGATGT GAATCTCAGG TTTGACGGGT TAACTTGGTT TGAAGGGTTA ACCCAGTTAA   
  
  
+ TTCAAATTTT TTTTTTCTTC ATTAGTTTTT TTTTCTTCCT GTTGATTTTT TTCATTGAAC TTATATATTT   
  
  
+ TAATTATAAT AATATAATAA TAATTACATA TACTAAAATA CTGAGTTGTC TTCTTTTTTT TTTACGTTTT   
  
  
+ TAAATTATTA TTTTTTTGTT TTTGTTTTTT TCTTTGTGTT TTTTTTATTT TAATGAATTT TTTTTGTTTA   
  
  
+ ATTTAGTTTG TTAATGTTTA ATTTTTTTTA TTTAGTTATC ATATTTTCAT AATACGGATC CCGAGTTTGA   
  
  
+ TGAGTTAATC CAGAATTTTT GCTTTTCTTC TTTTTAATTA ATTTTTTTTT GTTTAGTTTA GTTTGTTAAT   
  
  
+ GTTAAATTTT TTTTATTTAA TTATCAGACT TTTATGACAC GTATCTCGGG TTTGACGGGT TAACGCGTCA   
  
  
+ ATTTTTTTTT CTATTTAGTT ATCAAATTTT TATGACGCGA ATTTCAGGTT TGACAGGTTA ACCTGGTTTG   
  
  
+ AAGGGTTAAC CCATTTAATT CAATTTTTTT TCTTTTTCTT CATTAGTTTT TTTCTTCCAT TTAATTTTTT   
  
  
+ TTCTTTTTCT TCATTAATGT TATTATTAAT ATTATAAATA TCACTCTTGG ATCAGGCGTT GCAGCTAAAT   
  
  
+ CTAAAACTCA TAGGTATAGC TTTATAGAAA TTTGAATCTT AGTTTTTTTT TATATTTTTT ATACGAAAAA   
  
  
+ ATTAACCCGT GCAGGTAATG TGACTAGTAA TAACATTATT TTCTGTTGAA CCGAACAAGA AAGAATCGTA   
  
  
+ GTTAGGTGTC AGAGACTCAG AGTCGCTGCT TCTCACATTC AAACCTTGTA AGCGTTTGGT CATTGTAAGA   
  
  
+ CCCTCTCTTT CTTTTATCAA ATAACCTTTC TTTATTTCTT AAATAAATTA AGGACCCTTC TTTTTTTATA   
  
  
+ AAAAATAAAA ATAAAAAATT CTTCCTTCTT CCCATAATTG ATTCAATCGC AAATGAGTCA TGTCATGACC   
  
  
+ CGGAGACCCT TTGAAAAGTC AAATTAGATA AAGATTGAGA CGATGGTTTC AAGAGACACA ACCACTCAAG   
  
  
+ TATTTACCAT TGTGATGAGC TGACAAGGAT TGTCATCCAG AATGGTCTCT CCTCACGATG TCACCAGCCA   
  
  
+ GTGGAATCCA AATTAAATTG AATTTATTGG GAAATGGGAT AACTCTTCCT TTTATATATT AAAAATTAAA   
  
  
+ AAATATATAC AGTGCCAATT CTACTGTACT GGTTAATTAC CCTATCAACA TTTAAGTAGT TTATGTAACG   
  
  
+ TTGTATTAGT TGTTTTTTAA AATAATTTTT TTTTAATATA TTAAAATAAT TTTTTTAAAA AATTTATTTT   
  
  
+ TGATATTAAT ATGTTAAAAC AATTTAAAAA CATTGAAAAA AAATAAAATA AAATAAATTC AAGTTTTGCA   
  
  
+ CAAAAAAACA CTATTGCACC GCGTACATAA ATGATGTCTT AGTGTATTTA GCATTGTGAT AGCTTTTGTG   
  
  
+ ATCATTCTGG TTTTAAAAAA ACAAATTTAA AAGAAATATT TTTAGTTGTG GTTTTAAAAA GTAGATTTTA   
  
  
+ AAAAATACAT GTTTGATTAA ATCTACTATG AGATAAATTT TTACATGTAA AATAAATAAA AACATATTTC   
  
  
+ CATCAATTAA AAAAAAACAT GTTCTTTCAG TTTCTACATG ACTAATATTT AAAATACAAT TACTTATAAA   
  
  
+ GTCCGGTACG AAAGCACCCT CTAAATGGAG AATGAACTGT ATGTATTCTT CCCATGGACC TGTGATTTTT   
  
  
+ CCGTAGGAAA GGCAATTTCC ATTCAGAATA CAAGAAGATA CATGTCTTTT GAAACTCTTA TTAGCTGTCT   
  
  
+ ACCTATCAAC CAAGAATGAC GCTTCTCATC GTATGTCACT ATAAAATTTG GTTGATGACA CCAACACTTT   
  
  
+ CCCATTGGTA TTCATGGCTT TTTAGCTGCA CGATCCAAC  

- AATAGTTTGA AAGTACTACA CTTAGAGTCC AAACTGCCCA ATTGAACCAA ACTTCCCAAT TGGGTCAATT   
  
  
- AAGTTTAAAA AAAAAAGAAG TAATCAAAAA AAAAGAAGGA CAACTAAAAA AAGTAACTTG AATATATAAA   
  
  
- ATTAATATTA TTATATTATT ATTAATGTAT ATGATTTTAT GACTCAACAG AAGAAAAAAA AAATGCAAAA   
  
  
- ATTTAATAAT AAAAAAACAA AAACAAAAAA AGAAACACAA AAAAAATAAA ATTACTTAAA AAAAACAAAT   
  
  
- TAAATCAAAC AATTACAAAT TAAAAAAAAT AAATCAATAG TATAAAAGTA TTATGCCTAG GGCTCAAACT   
  
  
- ACTCAATTAG GTCTTAAAAA CGAAAAGAAG AAAAATTAAT TAAAAAAAAA CAAATCAAAT CAAACAATTA   
  
  
- CAATTTAAAA AAAATAAATT AATAGTCTGA AAATACTGTG CATAGAGCCC AAACTGCCCA ATTGCGCAGT   
  
  
- TAAAAAAAAA GATAAATCAA TAGTTTAAAA ATACTGCGCT TAAAGTCCAA ACTGTCCAAT TGGACCAAAC   
  
  
- TTCCCAATTG GGTAAATTAA GTTAAAAAAA AGAAAAAGAA GTAATCAAAA AAAGAAGGTA AATTAAAAAA   
  
  
- AAGAAAAAGA AGTAATTACA ATAATAATTA TAATATTTAT AGTGAGAACC TAGTCCGCAA CGTCGATTTA   
  
  
- GATTTTGAGT ATCCATATCG AAATATCTTT AAACTTAGAA TCAAAAAAAA ATATAAAAAA TATGCTTTTT   
  
  
- TAATTGGGCA CGTCCATTAC ACTGATCATT ATTGTAATAA AAGACAACTT GGCTTGTTCT TTCTTAGCAT   
  
  
- CAATCCACAG TCTCTGAGTC TCAGCGACGA AGAGTGTAAG TTTGGAACAT TCGCAAACCA GTAACATTCT   
  
  
- GGGAGAGAAA GAAAATAGTT TATTGGAAAG AAATAAAGAA TTTATTTAAT TCCTGGGAAG AAAAAAATAT   
  
  
- TTTTTATTTT TATTTTTTAA GAAGGAAGAA GGGTATTAAC TAAGTTAGCG TTTACTCAGT ACAGTACTGG   
  
  
- GCCTCTGGGA AACTTTTCAG TTTAATCTAT TTCTAACTCT GCTACCAAAG TTCTCTGTGT TGGTGAGTTC   
  
  
- ATAAATGGTA ACACTACTCG ACTGTTCCTA ACAGTAGGTC TTACCAGAGA GGAGTGCTAC AGTGGTCGGT   
  
  
- CACCTTAGGT TTAATTTAAC TTAAATAACC CTTTACCCTA TTGAGAAGGA AAATATATAA TTTTTAATTT   
  
  
- TTTATATATG TCACGGTTAA GATGACATGA CCAATTAATG GGATAGTTGT AAATTCATCA AATACATTGC   
  
  
- AACATAATCA ACAAAAAATT TTATTAAAAA AAAATTATAT AATTTTATTA AAAAAATTTT TTAAATAAAA   
  
  
- ACTATAATTA TACAATTTTG TTAAATTTTT GTAACTTTTT TTTATTTTAT TTTATTTAAG TTCAAAACGT   
  
  
- GTTTTTTTGT GATAACGTGG CGCATGTATT TACTACAGAA TCACATAAAT CGTAACACTA TCGAAAACAC   
  
  
- TAGTAAGACC AAAATTTTTT TGTTTAAATT TTCTTTATAA AAATCAACAC CAAAATTTTT CATCTAAAAT   
  
  
- TTTTTATGTA CAAACTAATT TAGATGATAC TCTATTTAAA AATGTACATT TTATTTATTT TTGTATAAAG   
  
  
- GTAGTTAATT TTTTTTTGTA CAAGAAAGTC AAAGATGTAC TGATTATAAA TTTTATGTTA ATGAATATTT   
  
  
- CAGGCCATGC TTTCGTGGGA GATTTACCTC TTACTTGACA TACATAAGAA GGGTACCTGG ACACTAAAAA   
  
  
- GGCATCCTTT CCGTTAAAGG TAAGTCTTAT GTTCTTCTAT GTACAGAAAA CTTTGAGAAT AATCGACAGA   
  
  
- TGGATAGTTG GTTCTTACTG CGAAGAGTAG CATACAGTGA TATTTTAAAC CAACTACTGT GGTTGTGAAA   
  
  
- GGGTAACCAT AAGTACCGAA AAATCGACGT GCTAGGTTG

+     Myb

| Site Name | Organism | Position | Strand | Matrix score. | sequence | function |
| --- | --- | --- | --- | --- | --- | --- |
| Myb | Arabidopsis thaliana | 64 | - | 6 | TAACTG |  |

>Potri.004G051700.1   
+ TTATCAAACT TTCATGATGT GAATCTCAGG TTTGACGGGT TAACTTGGTT TGAAGGGTTA ACCCAGTTAA   
  
  
+ TTCAAATTTT TTTTTTCTTC ATTAGTTTTT TTTTCTTCCT GTTGATTTTT TTCATTGAAC TTATATATTT   
  
  
+ TAATTATAAT AATATAATAA TAATTACATA TACTAAAATA CTGAGTTGTC TTCTTTTTTT TTTACGTTTT   
  
  
+ TAAATTATTA TTTTTTTGTT TTTGTTTTTT TCTTTGTGTT TTTTTTATTT TAATGAATTT TTTTTGTTTA   
  
  
+ ATTTAGTTTG TTAATGTTTA ATTTTTTTTA TTTAGTTATC ATATTTTCAT AATACGGATC CCGAGTTTGA   
  
  
+ TGAGTTAATC CAGAATTTTT GCTTTTCTTC TTTTTAATTA ATTTTTTTTT GTTTAGTTTA GTTTGTTAAT   
  
  
+ GTTAAATTTT TTTTATTTAA TTATCAGACT TTTATGACAC GTATCTCGGG TTTGACGGGT TAACGCGTCA   
  
  
+ ATTTTTTTTT CTATTTAGTT ATCAAATTTT TATGACGCGA ATTTCAGGTT TGACAGGTTA ACCTGGTTTG   
  
  
+ AAGGGTTAAC CCATTTAATT CAATTTTTTT TCTTTTTCTT CATTAGTTTT TTTCTTCCAT TTAATTTTTT   
  
  
+ TTCTTTTTCT TCATTAATGT TATTATTAAT ATTATAAATA TCACTCTTGG ATCAGGCGTT GCAGCTAAAT   
  
  
+ CTAAAACTCA TAGGTATAGC TTTATAGAAA TTTGAATCTT AGTTTTTTTT TATATTTTTT ATACGAAAAA   
  
  
+ ATTAACCCGT GCAGGTAATG TGACTAGTAA TAACATTATT TTCTGTTGAA CCGAACAAGA AAGAATCGTA   
  
  
+ GTTAGGTGTC AGAGACTCAG AGTCGCTGCT TCTCACATTC AAACCTTGTA AGCGTTTGGT CATTGTAAGA   
  
  
+ CCCTCTCTTT CTTTTATCAA ATAACCTTTC TTTATTTCTT AAATAAATTA AGGACCCTTC TTTTTTTATA   
  
  
+ AAAAATAAAA ATAAAAAATT CTTCCTTCTT CCCATAATTG ATTCAATCGC AAATGAGTCA TGTCATGACC   
  
  
+ CGGAGACCCT TTGAAAAGTC AAATTAGATA AAGATTGAGA CGATGGTTTC AAGAGACACA ACCACTCAAG   
  
  
+ TATTTACCAT TGTGATGAGC TGACAAGGAT TGTCATCCAG AATGGTCTCT CCTCACGATG TCACCAGCCA   
  
  
+ GTGGAATCCA AATTAAATTG AATTTATTGG GAAATGGGAT AACTCTTCCT TTTATATATT AAAAATTAAA   
  
  
+ AAATATATAC AGTGCCAATT CTACTGTACT GGTTAATTAC CCTATCAACA TTTAAGTAGT TTATGTAACG   
  
  
+ TTGTATTAGT TGTTTTTTAA AATAATTTTT TTTTAATATA TTAAAATAAT TTTTTTAAAA AATTTATTTT   
  
  
+ TGATATTAAT ATGTTAAAAC AATTTAAAAA CATTGAAAAA AAATAAAATA AAATAAATTC AAGTTTTGCA   
  
  
+ CAAAAAAACA CTATTGCACC GCGTACATAA ATGATGTCTT AGTGTATTTA GCATTGTGAT AGCTTTTGTG   
  
  
+ ATCATTCTGG TTTTAAAAAA ACAAATTTAA AAGAAATATT TTTAGTTGTG GTTTTAAAAA GTAGATTTTA   
  
  
+ AAAAATACAT GTTTGATTAA ATCTACTATG AGATAAATTT TTACATGTAA AATAAATAAA AACATATTTC   
  
  
+ CATCAATTAA AAAAAAACAT GTTCTTTCAG TTTCTACATG ACTAATATTT AAAATACAAT TACTTATAAA   
  
  
+ GTCCGGTACG AAAGCACCCT CTAAATGGAG AATGAACTGT ATGTATTCTT CCCATGGACC TGTGATTTTT   
  
  
+ CCGTAGGAAA GGCAATTTCC ATTCAGAATA CAAGAAGATA CATGTCTTTT GAAACTCTTA TTAGCTGTCT   
  
  
+ ACCTATCAAC CAAGAATGAC GCTTCTCATC GTATGTCACT ATAAAATTTG GTTGATGACA CCAACACTTT   
  
  
+ CCCATTGGTA TTCATGGCTT TTTAGCTGCA CGATCCAAC  

- AATAGTTTGA AAGTACTACA CTTAGAGTCC AAACTGCCCA ATTGAACCAA ACTTCCCAAT TGGGTCAATT   
  
  
- AAGTTTAAAA AAAAAAGAAG TAATCAAAAA AAAAGAAGGA CAACTAAAAA AAGTAACTTG AATATATAAA   
  
  
- ATTAATATTA TTATATTATT ATTAATGTAT ATGATTTTAT GACTCAACAG AAGAAAAAAA AAATGCAAAA   
  
  
- ATTTAATAAT AAAAAAACAA AAACAAAAAA AGAAACACAA AAAAAATAAA ATTACTTAAA AAAAACAAAT   
  
  
- TAAATCAAAC AATTACAAAT TAAAAAAAAT AAATCAATAG TATAAAAGTA TTATGCCTAG GGCTCAAACT   
  
  
- ACTCAATTAG GTCTTAAAAA CGAAAAGAAG AAAAATTAAT TAAAAAAAAA CAAATCAAAT CAAACAATTA   
  
  
- CAATTTAAAA AAAATAAATT AATAGTCTGA AAATACTGTG CATAGAGCCC AAACTGCCCA ATTGCGCAGT   
  
  
- TAAAAAAAAA GATAAATCAA TAGTTTAAAA ATACTGCGCT TAAAGTCCAA ACTGTCCAAT TGGACCAAAC   
  
  
- TTCCCAATTG GGTAAATTAA GTTAAAAAAA AGAAAAAGAA GTAATCAAAA AAAGAAGGTA AATTAAAAAA   
  
  
- AAGAAAAAGA AGTAATTACA ATAATAATTA TAATATTTAT AGTGAGAACC TAGTCCGCAA CGTCGATTTA   
  
  
- GATTTTGAGT ATCCATATCG AAATATCTTT AAACTTAGAA TCAAAAAAAA ATATAAAAAA TATGCTTTTT   
  
  
- TAATTGGGCA CGTCCATTAC ACTGATCATT ATTGTAATAA AAGACAACTT GGCTTGTTCT TTCTTAGCAT   
  
  
- CAATCCACAG TCTCTGAGTC TCAGCGACGA AGAGTGTAAG TTTGGAACAT TCGCAAACCA GTAACATTCT   
  
  
- GGGAGAGAAA GAAAATAGTT TATTGGAAAG AAATAAAGAA TTTATTTAAT TCCTGGGAAG AAAAAAATAT   
  
  
- TTTTTATTTT TATTTTTTAA GAAGGAAGAA GGGTATTAAC TAAGTTAGCG TTTACTCAGT ACAGTACTGG   
  
  
- GCCTCTGGGA AACTTTTCAG TTTAATCTAT TTCTAACTCT GCTACCAAAG TTCTCTGTGT TGGTGAGTTC   
  
  
- ATAAATGGTA ACACTACTCG ACTGTTCCTA ACAGTAGGTC TTACCAGAGA GGAGTGCTAC AGTGGTCGGT   
  
  
- CACCTTAGGT TTAATTTAAC TTAAATAACC CTTTACCCTA TTGAGAAGGA AAATATATAA TTTTTAATTT   
  
  
- TTTATATATG TCACGGTTAA GATGACATGA CCAATTAATG GGATAGTTGT AAATTCATCA AATACATTGC   
  
  
- AACATAATCA ACAAAAAATT TTATTAAAAA AAAATTATAT AATTTTATTA AAAAAATTTT TTAAATAAAA   
  
  
- ACTATAATTA TACAATTTTG TTAAATTTTT GTAACTTTTT TTTATTTTAT TTTATTTAAG TTCAAAACGT   
  
  
- GTTTTTTTGT GATAACGTGG CGCATGTATT TACTACAGAA TCACATAAAT CGTAACACTA TCGAAAACAC   
  
  
- TAGTAAGACC AAAATTTTTT TGTTTAAATT TTCTTTATAA AAATCAACAC CAAAATTTTT CATCTAAAAT   
  
  
- TTTTTATGTA CAAACTAATT TAGATGATAC TCTATTTAAA AATGTACATT TTATTTATTT TTGTATAAAG   
  
  
- GTAGTTAATT TTTTTTTGTA CAAGAAAGTC AAAGATGTAC TGATTATAAA TTTTATGTTA ATGAATATTT   
  
  
- CAGGCCATGC TTTCGTGGGA GATTTACCTC TTACTTGACA TACATAAGAA GGGTACCTGG ACACTAAAAA   
  
  
- GGCATCCTTT CCGTTAAAGG TAAGTCTTAT GTTCTTCTAT GTACAGAAAA CTTTGAGAAT AATCGACAGA   
  
  
- TGGATAGTTG GTTCTTACTG CGAAGAGTAG CATACAGTGA TATTTTAAAC CAACTACTGT GGTTGTGAAA   
  
  
- GGGTAACCAT AAGTACCGAA AAATCGACGT GCTAGGTTG

+     Myb-binding site

| Site Name | Organism | Position | Strand | Matrix score. | sequence | function |
| --- | --- | --- | --- | --- | --- | --- |
| Myb-binding site | Nicotiana tabacum | 813 | - | 6 | CAACAG |  |
| Myb-binding site | Nicotiana tabacum | 109 | - | 6 | CAACAG |  |

>Potri.004G051700.1   
+ TTATCAAACT TTCATGATGT GAATCTCAGG TTTGACGGGT TAACTTGGTT TGAAGGGTTA ACCCAGTTAA   
  
  
+ TTCAAATTTT TTTTTTCTTC ATTAGTTTTT TTTTCTTCCT GTTGATTTTT TTCATTGAAC TTATATATTT   
  
  
+ TAATTATAAT AATATAATAA TAATTACATA TACTAAAATA CTGAGTTGTC TTCTTTTTTT TTTACGTTTT   
  
  
+ TAAATTATTA TTTTTTTGTT TTTGTTTTTT TCTTTGTGTT TTTTTTATTT TAATGAATTT TTTTTGTTTA   
  
  
+ ATTTAGTTTG TTAATGTTTA ATTTTTTTTA TTTAGTTATC ATATTTTCAT AATACGGATC CCGAGTTTGA   
  
  
+ TGAGTTAATC CAGAATTTTT GCTTTTCTTC TTTTTAATTA ATTTTTTTTT GTTTAGTTTA GTTTGTTAAT   
  
  
+ GTTAAATTTT TTTTATTTAA TTATCAGACT TTTATGACAC GTATCTCGGG TTTGACGGGT TAACGCGTCA   
  
  
+ ATTTTTTTTT CTATTTAGTT ATCAAATTTT TATGACGCGA ATTTCAGGTT TGACAGGTTA ACCTGGTTTG   
  
  
+ AAGGGTTAAC CCATTTAATT CAATTTTTTT TCTTTTTCTT CATTAGTTTT TTTCTTCCAT TTAATTTTTT   
  
  
+ TTCTTTTTCT TCATTAATGT TATTATTAAT ATTATAAATA TCACTCTTGG ATCAGGCGTT GCAGCTAAAT   
  
  
+ CTAAAACTCA TAGGTATAGC TTTATAGAAA TTTGAATCTT AGTTTTTTTT TATATTTTTT ATACGAAAAA   
  
  
+ ATTAACCCGT GCAGGTAATG TGACTAGTAA TAACATTATT TTCTGTTGAA CCGAACAAGA AAGAATCGTA   
  
  
+ GTTAGGTGTC AGAGACTCAG AGTCGCTGCT TCTCACATTC AAACCTTGTA AGCGTTTGGT CATTGTAAGA   
  
  
+ CCCTCTCTTT CTTTTATCAA ATAACCTTTC TTTATTTCTT AAATAAATTA AGGACCCTTC TTTTTTTATA   
  
  
+ AAAAATAAAA ATAAAAAATT CTTCCTTCTT CCCATAATTG ATTCAATCGC AAATGAGTCA TGTCATGACC   
  
  
+ CGGAGACCCT TTGAAAAGTC AAATTAGATA AAGATTGAGA CGATGGTTTC AAGAGACACA ACCACTCAAG   
  
  
+ TATTTACCAT TGTGATGAGC TGACAAGGAT TGTCATCCAG AATGGTCTCT CCTCACGATG TCACCAGCCA   
  
  
+ GTGGAATCCA AATTAAATTG AATTTATTGG GAAATGGGAT AACTCTTCCT TTTATATATT AAAAATTAAA   
  
  
+ AAATATATAC AGTGCCAATT CTACTGTACT GGTTAATTAC CCTATCAACA TTTAAGTAGT TTATGTAACG   
  
  
+ TTGTATTAGT TGTTTTTTAA AATAATTTTT TTTTAATATA TTAAAATAAT TTTTTTAAAA AATTTATTTT   
  
  
+ TGATATTAAT ATGTTAAAAC AATTTAAAAA CATTGAAAAA AAATAAAATA AAATAAATTC AAGTTTTGCA   
  
  
+ CAAAAAAACA CTATTGCACC GCGTACATAA ATGATGTCTT AGTGTATTTA GCATTGTGAT AGCTTTTGTG   
  
  
+ ATCATTCTGG TTTTAAAAAA ACAAATTTAA AAGAAATATT TTTAGTTGTG GTTTTAAAAA GTAGATTTTA   
  
  
+ AAAAATACAT GTTTGATTAA ATCTACTATG AGATAAATTT TTACATGTAA AATAAATAAA AACATATTTC   
  
  
+ CATCAATTAA AAAAAAACAT GTTCTTTCAG TTTCTACATG ACTAATATTT AAAATACAAT TACTTATAAA   
  
  
+ GTCCGGTACG AAAGCACCCT CTAAATGGAG AATGAACTGT ATGTATTCTT CCCATGGACC TGTGATTTTT   
  
  
+ CCGTAGGAAA GGCAATTTCC ATTCAGAATA CAAGAAGATA CATGTCTTTT GAAACTCTTA TTAGCTGTCT   
  
  
+ ACCTATCAAC CAAGAATGAC GCTTCTCATC GTATGTCACT ATAAAATTTG GTTGATGACA CCAACACTTT   
  
  
+ CCCATTGGTA TTCATGGCTT TTTAGCTGCA CGATCCAAC  

- AATAGTTTGA AAGTACTACA CTTAGAGTCC AAACTGCCCA ATTGAACCAA ACTTCCCAAT TGGGTCAATT   
  
  
- AAGTTTAAAA AAAAAAGAAG TAATCAAAAA AAAAGAAGGA CAACTAAAAA AAGTAACTTG AATATATAAA   
  
  
- ATTAATATTA TTATATTATT ATTAATGTAT ATGATTTTAT GACTCAACAG AAGAAAAAAA AAATGCAAAA   
  
  
- ATTTAATAAT AAAAAAACAA AAACAAAAAA AGAAACACAA AAAAAATAAA ATTACTTAAA AAAAACAAAT   
  
  
- TAAATCAAAC AATTACAAAT TAAAAAAAAT AAATCAATAG TATAAAAGTA TTATGCCTAG GGCTCAAACT   
  
  
- ACTCAATTAG GTCTTAAAAA CGAAAAGAAG AAAAATTAAT TAAAAAAAAA CAAATCAAAT CAAACAATTA   
  
  
- CAATTTAAAA AAAATAAATT AATAGTCTGA AAATACTGTG CATAGAGCCC AAACTGCCCA ATTGCGCAGT   
  
  
- TAAAAAAAAA GATAAATCAA TAGTTTAAAA ATACTGCGCT TAAAGTCCAA ACTGTCCAAT TGGACCAAAC   
  
  
- TTCCCAATTG GGTAAATTAA GTTAAAAAAA AGAAAAAGAA GTAATCAAAA AAAGAAGGTA AATTAAAAAA   
  
  
- AAGAAAAAGA AGTAATTACA ATAATAATTA TAATATTTAT AGTGAGAACC TAGTCCGCAA CGTCGATTTA   
  
  
- GATTTTGAGT ATCCATATCG AAATATCTTT AAACTTAGAA TCAAAAAAAA ATATAAAAAA TATGCTTTTT   
  
  
- TAATTGGGCA CGTCCATTAC ACTGATCATT ATTGTAATAA AAGACAACTT GGCTTGTTCT TTCTTAGCAT   
  
  
- CAATCCACAG TCTCTGAGTC TCAGCGACGA AGAGTGTAAG TTTGGAACAT TCGCAAACCA GTAACATTCT   
  
  
- GGGAGAGAAA GAAAATAGTT TATTGGAAAG AAATAAAGAA TTTATTTAAT TCCTGGGAAG AAAAAAATAT   
  
  
- TTTTTATTTT TATTTTTTAA GAAGGAAGAA GGGTATTAAC TAAGTTAGCG TTTACTCAGT ACAGTACTGG   
  
  
- GCCTCTGGGA AACTTTTCAG TTTAATCTAT TTCTAACTCT GCTACCAAAG TTCTCTGTGT TGGTGAGTTC   
  
  
- ATAAATGGTA ACACTACTCG ACTGTTCCTA ACAGTAGGTC TTACCAGAGA GGAGTGCTAC AGTGGTCGGT   
  
  
- CACCTTAGGT TTAATTTAAC TTAAATAACC CTTTACCCTA TTGAGAAGGA AAATATATAA TTTTTAATTT   
  
  
- TTTATATATG TCACGGTTAA GATGACATGA CCAATTAATG GGATAGTTGT AAATTCATCA AATACATTGC   
  
  
- AACATAATCA ACAAAAAATT TTATTAAAAA AAAATTATAT AATTTTATTA AAAAAATTTT TTAAATAAAA   
  
  
- ACTATAATTA TACAATTTTG TTAAATTTTT GTAACTTTTT TTTATTTTAT TTTATTTAAG TTCAAAACGT   
  
  
- GTTTTTTTGT GATAACGTGG CGCATGTATT TACTACAGAA TCACATAAAT CGTAACACTA TCGAAAACAC   
  
  
- TAGTAAGACC AAAATTTTTT TGTTTAAATT TTCTTTATAA AAATCAACAC CAAAATTTTT CATCTAAAAT   
  
  
- TTTTTATGTA CAAACTAATT TAGATGATAC TCTATTTAAA AATGTACATT TTATTTATTT TTGTATAAAG   
  
  
- GTAGTTAATT TTTTTTTGTA CAAGAAAGTC AAAGATGTAC TGATTATAAA TTTTATGTTA ATGAATATTT   
  
  
- CAGGCCATGC TTTCGTGGGA GATTTACCTC TTACTTGACA TACATAAGAA GGGTACCTGG ACACTAAAAA   
  
  
- GGCATCCTTT CCGTTAAAGG TAAGTCTTAT GTTCTTCTAT GTACAGAAAA CTTTGAGAAT AATCGACAGA   
  
  
- TGGATAGTTG GTTCTTACTG CGAAGAGTAG CATACAGTGA TATTTTAAAC CAACTACTGT GGTTGTGAAA   
  
  
- GGGTAACCAT AAGTACCGAA AAATCGACGT GCTAGGTTG

+     TATA

| Site Name | Organism | Position | Strand | Matrix score. | sequence | function |
| --- | --- | --- | --- | --- | --- | --- |
| TATA | Arabidopsis thaliana | 1930 | + | 8 | TATAAAAT |  |

>Potri.004G051700.1   
+ TTATCAAACT TTCATGATGT GAATCTCAGG TTTGACGGGT TAACTTGGTT TGAAGGGTTA ACCCAGTTAA   
  
  
+ TTCAAATTTT TTTTTTCTTC ATTAGTTTTT TTTTCTTCCT GTTGATTTTT TTCATTGAAC TTATATATTT   
  
  
+ TAATTATAAT AATATAATAA TAATTACATA TACTAAAATA CTGAGTTGTC TTCTTTTTTT TTTACGTTTT   
  
  
+ TAAATTATTA TTTTTTTGTT TTTGTTTTTT TCTTTGTGTT TTTTTTATTT TAATGAATTT TTTTTGTTTA   
  
  
+ ATTTAGTTTG TTAATGTTTA ATTTTTTTTA TTTAGTTATC ATATTTTCAT AATACGGATC CCGAGTTTGA   
  
  
+ TGAGTTAATC CAGAATTTTT GCTTTTCTTC TTTTTAATTA ATTTTTTTTT GTTTAGTTTA GTTTGTTAAT   
  
  
+ GTTAAATTTT TTTTATTTAA TTATCAGACT TTTATGACAC GTATCTCGGG TTTGACGGGT TAACGCGTCA   
  
  
+ ATTTTTTTTT CTATTTAGTT ATCAAATTTT TATGACGCGA ATTTCAGGTT TGACAGGTTA ACCTGGTTTG   
  
  
+ AAGGGTTAAC CCATTTAATT CAATTTTTTT TCTTTTTCTT CATTAGTTTT TTTCTTCCAT TTAATTTTTT   
  
  
+ TTCTTTTTCT TCATTAATGT TATTATTAAT ATTATAAATA TCACTCTTGG ATCAGGCGTT GCAGCTAAAT   
  
  
+ CTAAAACTCA TAGGTATAGC TTTATAGAAA TTTGAATCTT AGTTTTTTTT TATATTTTTT ATACGAAAAA   
  
  
+ ATTAACCCGT GCAGGTAATG TGACTAGTAA TAACATTATT TTCTGTTGAA CCGAACAAGA AAGAATCGTA   
  
  
+ GTTAGGTGTC AGAGACTCAG AGTCGCTGCT TCTCACATTC AAACCTTGTA AGCGTTTGGT CATTGTAAGA   
  
  
+ CCCTCTCTTT CTTTTATCAA ATAACCTTTC TTTATTTCTT AAATAAATTA AGGACCCTTC TTTTTTTATA   
  
  
+ AAAAATAAAA ATAAAAAATT CTTCCTTCTT CCCATAATTG ATTCAATCGC AAATGAGTCA TGTCATGACC   
  
  
+ CGGAGACCCT TTGAAAAGTC AAATTAGATA AAGATTGAGA CGATGGTTTC AAGAGACACA ACCACTCAAG   
  
  
+ TATTTACCAT TGTGATGAGC TGACAAGGAT TGTCATCCAG AATGGTCTCT CCTCACGATG TCACCAGCCA   
  
  
+ GTGGAATCCA AATTAAATTG AATTTATTGG GAAATGGGAT AACTCTTCCT TTTATATATT AAAAATTAAA   
  
  
+ AAATATATAC AGTGCCAATT CTACTGTACT GGTTAATTAC CCTATCAACA TTTAAGTAGT TTATGTAACG   
  
  
+ TTGTATTAGT TGTTTTTTAA AATAATTTTT TTTTAATATA TTAAAATAAT TTTTTTAAAA AATTTATTTT   
  
  
+ TGATATTAAT ATGTTAAAAC AATTTAAAAA CATTGAAAAA AAATAAAATA AAATAAATTC AAGTTTTGCA   
  
  
+ CAAAAAAACA CTATTGCACC GCGTACATAA ATGATGTCTT AGTGTATTTA GCATTGTGAT AGCTTTTGTG   
  
  
+ ATCATTCTGG TTTTAAAAAA ACAAATTTAA AAGAAATATT TTTAGTTGTG GTTTTAAAAA GTAGATTTTA   
  
  
+ AAAAATACAT GTTTGATTAA ATCTACTATG AGATAAATTT TTACATGTAA AATAAATAAA AACATATTTC   
  
  
+ CATCAATTAA AAAAAAACAT GTTCTTTCAG TTTCTACATG ACTAATATTT AAAATACAAT TACTTATAAA   
  
  
+ GTCCGGTACG AAAGCACCCT CTAAATGGAG AATGAACTGT ATGTATTCTT CCCATGGACC TGTGATTTTT   
  
  
+ CCGTAGGAAA GGCAATTTCC ATTCAGAATA CAAGAAGATA CATGTCTTTT GAAACTCTTA TTAGCTGTCT   
  
  
+ ACCTATCAAC CAAGAATGAC GCTTCTCATC GTATGTCACT ATAAAATTTG GTTGATGACA CCAACACTTT   
  
  
+ CCCATTGGTA TTCATGGCTT TTTAGCTGCA CGATCCAAC  

- AATAGTTTGA AAGTACTACA CTTAGAGTCC AAACTGCCCA ATTGAACCAA ACTTCCCAAT TGGGTCAATT   
  
  
- AAGTTTAAAA AAAAAAGAAG TAATCAAAAA AAAAGAAGGA CAACTAAAAA AAGTAACTTG AATATATAAA   
  
  
- ATTAATATTA TTATATTATT ATTAATGTAT ATGATTTTAT GACTCAACAG AAGAAAAAAA AAATGCAAAA   
  
  
- ATTTAATAAT AAAAAAACAA AAACAAAAAA AGAAACACAA AAAAAATAAA ATTACTTAAA AAAAACAAAT   
  
  
- TAAATCAAAC AATTACAAAT TAAAAAAAAT AAATCAATAG TATAAAAGTA TTATGCCTAG GGCTCAAACT   
  
  
- ACTCAATTAG GTCTTAAAAA CGAAAAGAAG AAAAATTAAT TAAAAAAAAA CAAATCAAAT CAAACAATTA   
  
  
- CAATTTAAAA AAAATAAATT AATAGTCTGA AAATACTGTG CATAGAGCCC AAACTGCCCA ATTGCGCAGT   
  
  
- TAAAAAAAAA GATAAATCAA TAGTTTAAAA ATACTGCGCT TAAAGTCCAA ACTGTCCAAT TGGACCAAAC   
  
  
- TTCCCAATTG GGTAAATTAA GTTAAAAAAA AGAAAAAGAA GTAATCAAAA AAAGAAGGTA AATTAAAAAA   
  
  
- AAGAAAAAGA AGTAATTACA ATAATAATTA TAATATTTAT AGTGAGAACC TAGTCCGCAA CGTCGATTTA   
  
  
- GATTTTGAGT ATCCATATCG AAATATCTTT AAACTTAGAA TCAAAAAAAA ATATAAAAAA TATGCTTTTT   
  
  
- TAATTGGGCA CGTCCATTAC ACTGATCATT ATTGTAATAA AAGACAACTT GGCTTGTTCT TTCTTAGCAT   
  
  
- CAATCCACAG TCTCTGAGTC TCAGCGACGA AGAGTGTAAG TTTGGAACAT TCGCAAACCA GTAACATTCT   
  
  
- GGGAGAGAAA GAAAATAGTT TATTGGAAAG AAATAAAGAA TTTATTTAAT TCCTGGGAAG AAAAAAATAT   
  
  
- TTTTTATTTT TATTTTTTAA GAAGGAAGAA GGGTATTAAC TAAGTTAGCG TTTACTCAGT ACAGTACTGG   
  
  
- GCCTCTGGGA AACTTTTCAG TTTAATCTAT TTCTAACTCT GCTACCAAAG TTCTCTGTGT TGGTGAGTTC   
  
  
- ATAAATGGTA ACACTACTCG ACTGTTCCTA ACAGTAGGTC TTACCAGAGA GGAGTGCTAC AGTGGTCGGT   
  
  
- CACCTTAGGT TTAATTTAAC TTAAATAACC CTTTACCCTA TTGAGAAGGA AAATATATAA TTTTTAATTT   
  
  
- TTTATATATG TCACGGTTAA GATGACATGA CCAATTAATG GGATAGTTGT AAATTCATCA AATACATTGC   
  
  
- AACATAATCA ACAAAAAATT TTATTAAAAA AAAATTATAT AATTTTATTA AAAAAATTTT TTAAATAAAA   
  
  
- ACTATAATTA TACAATTTTG TTAAATTTTT GTAACTTTTT TTTATTTTAT TTTATTTAAG TTCAAAACGT   
  
  
- GTTTTTTTGT GATAACGTGG CGCATGTATT TACTACAGAA TCACATAAAT CGTAACACTA TCGAAAACAC   
  
  
- TAGTAAGACC AAAATTTTTT TGTTTAAATT TTCTTTATAA AAATCAACAC CAAAATTTTT CATCTAAAAT   
  
  
- TTTTTATGTA CAAACTAATT TAGATGATAC TCTATTTAAA AATGTACATT TTATTTATTT TTGTATAAAG   
  
  
- GTAGTTAATT TTTTTTTGTA CAAGAAAGTC AAAGATGTAC TGATTATAAA TTTTATGTTA ATGAATATTT   
  
  
- CAGGCCATGC TTTCGTGGGA GATTTACCTC TTACTTGACA TACATAAGAA GGGTACCTGG ACACTAAAAA   
  
  
- GGCATCCTTT CCGTTAAAGG TAAGTCTTAT GTTCTTCTAT GTACAGAAAA CTTTGAGAAT AATCGACAGA   
  
  
- TGGATAGTTG GTTCTTACTG CGAAGAGTAG CATACAGTGA TATTTTAAAC CAACTACTGT GGTTGTGAAA   
  
  
- GGGTAACCAT AAGTACCGAA AAATCGACGT GCTAGGTTG

+     TATA-box

| Site Name | Organism | Position | Strand | Matrix score. | sequence | function |
| --- | --- | --- | --- | --- | --- | --- |
| TATA-box | Arabidopsis thaliana | 1744 | - | 5 | TATAA | core promoter element around -30 of transcription start |
| TATA-box | Arabidopsis thaliana | 1726 | + | 8 | TATTTAAA | core promoter element around -30 of transcription start |
| TATA-box | Oryza sativa | 1494 | + | 8 | TACATAAA | core promoter element around -30 of transcription start |
| TATA-box | Arabidopsis thaliana | 1367 | - | 4 | TATA | core promoter element around -30 of transcription start |
| TATA-box | Pisum sativum | 757 | - | 7 | TATAAAA | core promoter element around -30 of transcription start |
| TATA-box | Brassica napus | 1366 | - | 6 | ATATAT | core promoter element around -30 of transcription start |
| TATA-box | Arabidopsis thaliana | 1930 | - | 4 | TATA | core promoter element around -30 of transcription start |
| TATA-box | Arabidopsis thaliana | 1745 | - | 4 | TATA | core promoter element around -30 of transcription start |
| TATA-box | Oryza sativa | 1320 | - | 8 | TACATAAA | core promoter element around -30 of transcription start |
| TATA-box | Arabidopsis thaliana | 1266 | - | 4 | TATA | core promoter element around -30 of transcription start |
| TATA-box | Arabidopsis thaliana | 1264 | - | 6 | TATATA | core promoter element around -30 of transcription start |
| TATA-box | Brassica napus | 1263 | - | 6 | ATATAT | core promoter element around -30 of transcription start |
| TATA-box | Arabidopsis thaliana | 1245 | - | 4 | TATA | core promoter element around -30 of transcription start |
| TATA-box | Helianthus annuus | 758 | - | 6 | TATAAA | core promoter element around -30 of transcription start |
| TATA-box | Brassica napus | 1244 | - | 6 | ATATAT | core promoter element around -30 of transcription start |
| TATA-box | Arabidopsis thaliana | 1242 | - | 7 | TATATAA | core promoter element around -30 of transcription start |
| TATA-box | Arabidopsis thaliana | 1243 | - | 6 | TATATA | core promoter element around -30 of transcription start |
| TATA-box | Helianthus annuus | 1241 | - | 6 | TATAAA | core promoter element around -30 of transcription start |
| TATA-box | Arabidopsis thaliana | 1079 | + | 8 | TAAAGATT | core promoter element around -30 of transcription start |
| TATA-box | Pisum sativum | 1240 | - | 7 | TATAAAA | core promoter element around -30 of transcription start |
| TATA-box | Arabidopsis thaliana | 976 | - | 5 | TATAA | core promoter element around -30 of transcription start |
| TATA-box | Arabidopsis thaliana | 977 | + | 4 | TATA | core promoter element around -30 of transcription start |
| TATA-box | Pisum sativum | 974 | - | 7 | TATAAAA | core promoter element around -30 of transcription start |
| TATA-box | Helianthus annuus | 975 | - | 6 | TATAAA | core promoter element around -30 of transcription start |
| TATA-box | Arabidopsis thaliana | 759 | - | 5 | TATAA | core promoter element around -30 of transcription start |
| TATA-box | Arabidopsis thaliana | 760 | + | 4 | TATA | core promoter element around -30 of transcription start |
| TATA-box | Arabidopsis thaliana | 756 | - | 9 | ccTATAAAaa | core promoter element around -30 of transcription start |
| TATA-box | Arabidopsis thaliana | 751 | + | 4 | TATA | core promoter element around -30 of transcription start |
| TATA-box | Helianthus annuus | 749 | - | 6 | TATAAA | core promoter element around -30 of transcription start |
| TATA-box | Arabidopsis thaliana | 750 | - | 5 | TATAA | core promoter element around -30 of transcription start |
| TATA-box | Pisum sativum | 748 | - | 7 | TATAAAA | core promoter element around -30 of transcription start |
| TATA-box | Arabidopsis thaliana | 723 | + | 4 | TATA | core promoter element around -30 of transcription start |
| TATA-box | Arabidopsis thaliana | 722 | - | 5 | TATAA | core promoter element around -30 of transcription start |
| TATA-box | Helianthus annuus | 721 | - | 6 | TATAAA | core promoter element around -30 of transcription start |
| TATA-box | Arabidopsis thaliana | 715 | + | 4 | TATA | core promoter element around -30 of transcription start |
| TATA-box | Arabidopsis thaliana | 663 | + | 4 | TATA | core promoter element around -30 of transcription start |
| TATA-box | Arabidopsis thaliana | 662 | - | 5 | TATAA | core promoter element around -30 of transcription start |
| TATA-box | Brassica napus | 661 | + | 6 | ATTATA | core promoter element around -30 of transcription start |
| TATA-box | Arabidopsis thaliana | 145 | + | 4 | TATA | core promoter element around -30 of transcription start |
| TATA-box | Arabidopsis thaliana | 169 | + | 4 | TATA | core promoter element around -30 of transcription start |
| TATA-box | Arabidopsis thaliana | 153 | + | 4 | TATA | core promoter element around -30 of transcription start |
| TATA-box | Brassica oleracea | 152 | + | 6 | ATATAA | core promoter element around -30 of transcription start |
| TATA-box | Brassica napus | 143 | + | 6 | ATTATA | core promoter element around -30 of transcription start |
| TATA-box | Arabidopsis thaliana | 134 | + | 4 | TATA | core promoter element around -30 of transcription start |
| TATA-box | Brassica napus | 133 | + | 6 | ATATAT | core promoter element around -30 of transcription start |
| TATA-box | Arabidopsis thaliana | 132 | + | 6 | TATATA | core promoter element around -30 of transcription start |
| TATA-box | Arabidopsis thaliana | 131 | - | 7 | TATATAA | core promoter element around -30 of transcription start |
| TATA-box | Arabidopsis thaliana | 144 | - | 5 | TATAA | core promoter element around -30 of transcription start |

>Potri.004G051700.1   
+ TTATCAAACT TTCATGATGT GAATCTCAGG TTTGACGGGT TAACTTGGTT TGAAGGGTTA ACCCAGTTAA   
  
  
+ TTCAAATTTT TTTTTTCTTC ATTAGTTTTT TTTTCTTCCT GTTGATTTTT TTCATTGAAC TTATATATTT   
  
  
+ TAATTATAAT AATATAATAA TAATTACATA TACTAAAATA CTGAGTTGTC TTCTTTTTTT TTTACGTTTT   
  
  
+ TAAATTATTA TTTTTTTGTT TTTGTTTTTT TCTTTGTGTT TTTTTTATTT TAATGAATTT TTTTTGTTTA   
  
  
+ ATTTAGTTTG TTAATGTTTA ATTTTTTTTA TTTAGTTATC ATATTTTCAT AATACGGATC CCGAGTTTGA   
  
  
+ TGAGTTAATC CAGAATTTTT GCTTTTCTTC TTTTTAATTA ATTTTTTTTT GTTTAGTTTA GTTTGTTAAT   
  
  
+ GTTAAATTTT TTTTATTTAA TTATCAGACT TTTATGACAC GTATCTCGGG TTTGACGGGT TAACGCGTCA   
  
  
+ ATTTTTTTTT CTATTTAGTT ATCAAATTTT TATGACGCGA ATTTCAGGTT TGACAGGTTA ACCTGGTTTG   
  
  
+ AAGGGTTAAC CCATTTAATT CAATTTTTTT TCTTTTTCTT CATTAGTTTT TTTCTTCCAT TTAATTTTTT   
  
  
+ TTCTTTTTCT TCATTAATGT TATTATTAAT ATTATAAATA TCACTCTTGG ATCAGGCGTT GCAGCTAAAT   
  
  
+ CTAAAACTCA TAGGTATAGC TTTATAGAAA TTTGAATCTT AGTTTTTTTT TATATTTTTT ATACGAAAAA   
  
  
+ ATTAACCCGT GCAGGTAATG TGACTAGTAA TAACATTATT TTCTGTTGAA CCGAACAAGA AAGAATCGTA   
  
  
+ GTTAGGTGTC AGAGACTCAG AGTCGCTGCT TCTCACATTC AAACCTTGTA AGCGTTTGGT CATTGTAAGA   
  
  
+ CCCTCTCTTT CTTTTATCAA ATAACCTTTC TTTATTTCTT AAATAAATTA AGGACCCTTC TTTTTTTATA   
  
  
+ AAAAATAAAA ATAAAAAATT CTTCCTTCTT CCCATAATTG ATTCAATCGC AAATGAGTCA TGTCATGACC   
  
  
+ CGGAGACCCT TTGAAAAGTC AAATTAGATA AAGATTGAGA CGATGGTTTC AAGAGACACA ACCACTCAAG   
  
  
+ TATTTACCAT TGTGATGAGC TGACAAGGAT TGTCATCCAG AATGGTCTCT CCTCACGATG TCACCAGCCA   
  
  
+ GTGGAATCCA AATTAAATTG AATTTATTGG GAAATGGGAT AACTCTTCCT TTTATATATT AAAAATTAAA   
  
  
+ AAATATATAC AGTGCCAATT CTACTGTACT GGTTAATTAC CCTATCAACA TTTAAGTAGT TTATGTAACG   
  
  
+ TTGTATTAGT TGTTTTTTAA AATAATTTTT TTTTAATATA TTAAAATAAT TTTTTTAAAA AATTTATTTT   
  
  
+ TGATATTAAT ATGTTAAAAC AATTTAAAAA CATTGAAAAA AAATAAAATA AAATAAATTC AAGTTTTGCA   
  
  
+ CAAAAAAACA CTATTGCACC GCGTACATAA ATGATGTCTT AGTGTATTTA GCATTGTGAT AGCTTTTGTG   
  
  
+ ATCATTCTGG TTTTAAAAAA ACAAATTTAA AAGAAATATT TTTAGTTGTG GTTTTAAAAA GTAGATTTTA   
  
  
+ AAAAATACAT GTTTGATTAA ATCTACTATG AGATAAATTT TTACATGTAA AATAAATAAA AACATATTTC   
  
  
+ CATCAATTAA AAAAAAACAT GTTCTTTCAG TTTCTACATG ACTAATATTT AAAATACAAT TACTTATAAA   
  
  
+ GTCCGGTACG AAAGCACCCT CTAAATGGAG AATGAACTGT ATGTATTCTT CCCATGGACC TGTGATTTTT   
  
  
+ CCGTAGGAAA GGCAATTTCC ATTCAGAATA CAAGAAGATA CATGTCTTTT GAAACTCTTA TTAGCTGTCT   
  
  
+ ACCTATCAAC CAAGAATGAC GCTTCTCATC GTATGTCACT ATAAAATTTG GTTGATGACA CCAACACTTT   
  
  
+ CCCATTGGTA TTCATGGCTT TTTAGCTGCA CGATCCAAC  

- AATAGTTTGA AAGTACTACA CTTAGAGTCC AAACTGCCCA ATTGAACCAA ACTTCCCAAT TGGGTCAATT   
  
  
- AAGTTTAAAA AAAAAAGAAG TAATCAAAAA AAAAGAAGGA CAACTAAAAA AAGTAACTTG AATATATAAA   
  
  
- ATTAATATTA TTATATTATT ATTAATGTAT ATGATTTTAT GACTCAACAG AAGAAAAAAA AAATGCAAAA   
  
  
- ATTTAATAAT AAAAAAACAA AAACAAAAAA AGAAACACAA AAAAAATAAA ATTACTTAAA AAAAACAAAT   
  
  
- TAAATCAAAC AATTACAAAT TAAAAAAAAT AAATCAATAG TATAAAAGTA TTATGCCTAG GGCTCAAACT   
  
  
- ACTCAATTAG GTCTTAAAAA CGAAAAGAAG AAAAATTAAT TAAAAAAAAA CAAATCAAAT CAAACAATTA   
  
  
- CAATTTAAAA AAAATAAATT AATAGTCTGA AAATACTGTG CATAGAGCCC AAACTGCCCA ATTGCGCAGT   
  
  
- TAAAAAAAAA GATAAATCAA TAGTTTAAAA ATACTGCGCT TAAAGTCCAA ACTGTCCAAT TGGACCAAAC   
  
  
- TTCCCAATTG GGTAAATTAA GTTAAAAAAA AGAAAAAGAA GTAATCAAAA AAAGAAGGTA AATTAAAAAA   
  
  
- AAGAAAAAGA AGTAATTACA ATAATAATTA TAATATTTAT AGTGAGAACC TAGTCCGCAA CGTCGATTTA   
  
  
- GATTTTGAGT ATCCATATCG AAATATCTTT AAACTTAGAA TCAAAAAAAA ATATAAAAAA TATGCTTTTT   
  
  
- TAATTGGGCA CGTCCATTAC ACTGATCATT ATTGTAATAA AAGACAACTT GGCTTGTTCT TTCTTAGCAT   
  
  
- CAATCCACAG TCTCTGAGTC TCAGCGACGA AGAGTGTAAG TTTGGAACAT TCGCAAACCA GTAACATTCT   
  
  
- GGGAGAGAAA GAAAATAGTT TATTGGAAAG AAATAAAGAA TTTATTTAAT TCCTGGGAAG AAAAAAATAT   
  
  
- TTTTTATTTT TATTTTTTAA GAAGGAAGAA GGGTATTAAC TAAGTTAGCG TTTACTCAGT ACAGTACTGG   
  
  
- GCCTCTGGGA AACTTTTCAG TTTAATCTAT TTCTAACTCT GCTACCAAAG TTCTCTGTGT TGGTGAGTTC   
  
  
- ATAAATGGTA ACACTACTCG ACTGTTCCTA ACAGTAGGTC TTACCAGAGA GGAGTGCTAC AGTGGTCGGT   
  
  
- CACCTTAGGT TTAATTTAAC TTAAATAACC CTTTACCCTA TTGAGAAGGA AAATATATAA TTTTTAATTT   
  
  
- TTTATATATG TCACGGTTAA GATGACATGA CCAATTAATG GGATAGTTGT AAATTCATCA AATACATTGC   
  
  
- AACATAATCA ACAAAAAATT TTATTAAAAA AAAATTATAT AATTTTATTA AAAAAATTTT TTAAATAAAA   
  
  
- ACTATAATTA TACAATTTTG TTAAATTTTT GTAACTTTTT TTTATTTTAT TTTATTTAAG TTCAAAACGT   
  
  
- GTTTTTTTGT GATAACGTGG CGCATGTATT TACTACAGAA TCACATAAAT CGTAACACTA TCGAAAACAC   
  
  
- TAGTAAGACC AAAATTTTTT TGTTTAAATT TTCTTTATAA AAATCAACAC CAAAATTTTT CATCTAAAAT   
  
  
- TTTTTATGTA CAAACTAATT TAGATGATAC TCTATTTAAA AATGTACATT TTATTTATTT TTGTATAAAG   
  
  
- GTAGTTAATT TTTTTTTGTA CAAGAAAGTC AAAGATGTAC TGATTATAAA TTTTATGTTA ATGAATATTT   
  
  
- CAGGCCATGC TTTCGTGGGA GATTTACCTC TTACTTGACA TACATAAGAA GGGTACCTGG ACACTAAAAA   
  
  
- GGCATCCTTT CCGTTAAAGG TAAGTCTTAT GTTCTTCTAT GTACAGAAAA CTTTGAGAAT AATCGACAGA   
  
  
- TGGATAGTTG GTTCTTACTG CGAAGAGTAG CATACAGTGA TATTTTAAAC CAACTACTGT GGTTGTGAAA   
  
  
- GGGTAACCAT AAGTACCGAA AAATCGACGT GCTAGGTTG

+     TATC-box

| Site Name | Organism | Position | Strand | Matrix score. | sequence | function |
| --- | --- | --- | --- | --- | --- | --- |
| TATC-box | Oryza sativa | 1225 | - | 7 | TATCCCA | cis-acting element involved in gibberellin-responsiveness |

>Potri.004G051700.1   
+ TTATCAAACT TTCATGATGT GAATCTCAGG TTTGACGGGT TAACTTGGTT TGAAGGGTTA ACCCAGTTAA   
  
  
+ TTCAAATTTT TTTTTTCTTC ATTAGTTTTT TTTTCTTCCT GTTGATTTTT TTCATTGAAC TTATATATTT   
  
  
+ TAATTATAAT AATATAATAA TAATTACATA TACTAAAATA CTGAGTTGTC TTCTTTTTTT TTTACGTTTT   
  
  
+ TAAATTATTA TTTTTTTGTT TTTGTTTTTT TCTTTGTGTT TTTTTTATTT TAATGAATTT TTTTTGTTTA   
  
  
+ ATTTAGTTTG TTAATGTTTA ATTTTTTTTA TTTAGTTATC ATATTTTCAT AATACGGATC CCGAGTTTGA   
  
  
+ TGAGTTAATC CAGAATTTTT GCTTTTCTTC TTTTTAATTA ATTTTTTTTT GTTTAGTTTA GTTTGTTAAT   
  
  
+ GTTAAATTTT TTTTATTTAA TTATCAGACT TTTATGACAC GTATCTCGGG TTTGACGGGT TAACGCGTCA   
  
  
+ ATTTTTTTTT CTATTTAGTT ATCAAATTTT TATGACGCGA ATTTCAGGTT TGACAGGTTA ACCTGGTTTG   
  
  
+ AAGGGTTAAC CCATTTAATT CAATTTTTTT TCTTTTTCTT CATTAGTTTT TTTCTTCCAT TTAATTTTTT   
  
  
+ TTCTTTTTCT TCATTAATGT TATTATTAAT ATTATAAATA TCACTCTTGG ATCAGGCGTT GCAGCTAAAT   
  
  
+ CTAAAACTCA TAGGTATAGC TTTATAGAAA TTTGAATCTT AGTTTTTTTT TATATTTTTT ATACGAAAAA   
  
  
+ ATTAACCCGT GCAGGTAATG TGACTAGTAA TAACATTATT TTCTGTTGAA CCGAACAAGA AAGAATCGTA   
  
  
+ GTTAGGTGTC AGAGACTCAG AGTCGCTGCT TCTCACATTC AAACCTTGTA AGCGTTTGGT CATTGTAAGA   
  
  
+ CCCTCTCTTT CTTTTATCAA ATAACCTTTC TTTATTTCTT AAATAAATTA AGGACCCTTC TTTTTTTATA   
  
  
+ AAAAATAAAA ATAAAAAATT CTTCCTTCTT CCCATAATTG ATTCAATCGC AAATGAGTCA TGTCATGACC   
  
  
+ CGGAGACCCT TTGAAAAGTC AAATTAGATA AAGATTGAGA CGATGGTTTC AAGAGACACA ACCACTCAAG   
  
  
+ TATTTACCAT TGTGATGAGC TGACAAGGAT TGTCATCCAG AATGGTCTCT CCTCACGATG TCACCAGCCA   
  
  
+ GTGGAATCCA AATTAAATTG AATTTATTGG GAAATGGGAT AACTCTTCCT TTTATATATT AAAAATTAAA   
  
  
+ AAATATATAC AGTGCCAATT CTACTGTACT GGTTAATTAC CCTATCAACA TTTAAGTAGT TTATGTAACG   
  
  
+ TTGTATTAGT TGTTTTTTAA AATAATTTTT TTTTAATATA TTAAAATAAT TTTTTTAAAA AATTTATTTT   
  
  
+ TGATATTAAT ATGTTAAAAC AATTTAAAAA CATTGAAAAA AAATAAAATA AAATAAATTC AAGTTTTGCA   
  
  
+ CAAAAAAACA CTATTGCACC GCGTACATAA ATGATGTCTT AGTGTATTTA GCATTGTGAT AGCTTTTGTG   
  
  
+ ATCATTCTGG TTTTAAAAAA ACAAATTTAA AAGAAATATT TTTAGTTGTG GTTTTAAAAA GTAGATTTTA   
  
  
+ AAAAATACAT GTTTGATTAA ATCTACTATG AGATAAATTT TTACATGTAA AATAAATAAA AACATATTTC   
  
  
+ CATCAATTAA AAAAAAACAT GTTCTTTCAG TTTCTACATG ACTAATATTT AAAATACAAT TACTTATAAA   
  
  
+ GTCCGGTACG AAAGCACCCT CTAAATGGAG AATGAACTGT ATGTATTCTT CCCATGGACC TGTGATTTTT   
  
  
+ CCGTAGGAAA GGCAATTTCC ATTCAGAATA CAAGAAGATA CATGTCTTTT GAAACTCTTA TTAGCTGTCT   
  
  
+ ACCTATCAAC CAAGAATGAC GCTTCTCATC GTATGTCACT ATAAAATTTG GTTGATGACA CCAACACTTT   
  
  
+ CCCATTGGTA TTCATGGCTT TTTAGCTGCA CGATCCAAC  

- AATAGTTTGA AAGTACTACA CTTAGAGTCC AAACTGCCCA ATTGAACCAA ACTTCCCAAT TGGGTCAATT   
  
  
- AAGTTTAAAA AAAAAAGAAG TAATCAAAAA AAAAGAAGGA CAACTAAAAA AAGTAACTTG AATATATAAA   
  
  
- ATTAATATTA TTATATTATT ATTAATGTAT ATGATTTTAT GACTCAACAG AAGAAAAAAA AAATGCAAAA   
  
  
- ATTTAATAAT AAAAAAACAA AAACAAAAAA AGAAACACAA AAAAAATAAA ATTACTTAAA AAAAACAAAT   
  
  
- TAAATCAAAC AATTACAAAT TAAAAAAAAT AAATCAATAG TATAAAAGTA TTATGCCTAG GGCTCAAACT   
  
  
- ACTCAATTAG GTCTTAAAAA CGAAAAGAAG AAAAATTAAT TAAAAAAAAA CAAATCAAAT CAAACAATTA   
  
  
- CAATTTAAAA AAAATAAATT AATAGTCTGA AAATACTGTG CATAGAGCCC AAACTGCCCA ATTGCGCAGT   
  
  
- TAAAAAAAAA GATAAATCAA TAGTTTAAAA ATACTGCGCT TAAAGTCCAA ACTGTCCAAT TGGACCAAAC   
  
  
- TTCCCAATTG GGTAAATTAA GTTAAAAAAA AGAAAAAGAA GTAATCAAAA AAAGAAGGTA AATTAAAAAA   
  
  
- AAGAAAAAGA AGTAATTACA ATAATAATTA TAATATTTAT AGTGAGAACC TAGTCCGCAA CGTCGATTTA   
  
  
- GATTTTGAGT ATCCATATCG AAATATCTTT AAACTTAGAA TCAAAAAAAA ATATAAAAAA TATGCTTTTT   
  
  
- TAATTGGGCA CGTCCATTAC ACTGATCATT ATTGTAATAA AAGACAACTT GGCTTGTTCT TTCTTAGCAT   
  
  
- CAATCCACAG TCTCTGAGTC TCAGCGACGA AGAGTGTAAG TTTGGAACAT TCGCAAACCA GTAACATTCT   
  
  
- GGGAGAGAAA GAAAATAGTT TATTGGAAAG AAATAAAGAA TTTATTTAAT TCCTGGGAAG AAAAAAATAT   
  
  
- TTTTTATTTT TATTTTTTAA GAAGGAAGAA GGGTATTAAC TAAGTTAGCG TTTACTCAGT ACAGTACTGG   
  
  
- GCCTCTGGGA AACTTTTCAG TTTAATCTAT TTCTAACTCT GCTACCAAAG TTCTCTGTGT TGGTGAGTTC   
  
  
- ATAAATGGTA ACACTACTCG ACTGTTCCTA ACAGTAGGTC TTACCAGAGA GGAGTGCTAC AGTGGTCGGT   
  
  
- CACCTTAGGT TTAATTTAAC TTAAATAACC CTTTACCCTA TTGAGAAGGA AAATATATAA TTTTTAATTT   
  
  
- TTTATATATG TCACGGTTAA GATGACATGA CCAATTAATG GGATAGTTGT AAATTCATCA AATACATTGC   
  
  
- AACATAATCA ACAAAAAATT TTATTAAAAA AAAATTATAT AATTTTATTA AAAAAATTTT TTAAATAAAA   
  
  
- ACTATAATTA TACAATTTTG TTAAATTTTT GTAACTTTTT TTTATTTTAT TTTATTTAAG TTCAAAACGT   
  
  
- GTTTTTTTGT GATAACGTGG CGCATGTATT TACTACAGAA TCACATAAAT CGTAACACTA TCGAAAACAC   
  
  
- TAGTAAGACC AAAATTTTTT TGTTTAAATT TTCTTTATAA AAATCAACAC CAAAATTTTT CATCTAAAAT   
  
  
- TTTTTATGTA CAAACTAATT TAGATGATAC TCTATTTAAA AATGTACATT TTATTTATTT TTGTATAAAG   
  
  
- GTAGTTAATT TTTTTTTGTA CAAGAAAGTC AAAGATGTAC TGATTATAAA TTTTATGTTA ATGAATATTT   
  
  
- CAGGCCATGC TTTCGTGGGA GATTTACCTC TTACTTGACA TACATAAGAA GGGTACCTGG ACACTAAAAA   
  
  
- GGCATCCTTT CCGTTAAAGG TAAGTCTTAT GTTCTTCTAT GTACAGAAAA CTTTGAGAAT AATCGACAGA   
  
  
- TGGATAGTTG GTTCTTACTG CGAAGAGTAG CATACAGTGA TATTTTAAAC CAACTACTGT GGTTGTGAAA   
  
  
- GGGTAACCAT AAGTACCGAA AAATCGACGT GCTAGGTTG

+     TCA-element

| Site Name | Organism | Position | Strand | Matrix score. | sequence | function |
| --- | --- | --- | --- | --- | --- | --- |
| TCA-element | Nicotiana tabacum | 966 | + | 9 | CCATCTTTTT | cis-acting element involved in salicylic acid responsiveness |

>Potri.004G051700.1   
+ TTATCAAACT TTCATGATGT GAATCTCAGG TTTGACGGGT TAACTTGGTT TGAAGGGTTA ACCCAGTTAA   
  
  
+ TTCAAATTTT TTTTTTCTTC ATTAGTTTTT TTTTCTTCCT GTTGATTTTT TTCATTGAAC TTATATATTT   
  
  
+ TAATTATAAT AATATAATAA TAATTACATA TACTAAAATA CTGAGTTGTC TTCTTTTTTT TTTACGTTTT   
  
  
+ TAAATTATTA TTTTTTTGTT TTTGTTTTTT TCTTTGTGTT TTTTTTATTT TAATGAATTT TTTTTGTTTA   
  
  
+ ATTTAGTTTG TTAATGTTTA ATTTTTTTTA TTTAGTTATC ATATTTTCAT AATACGGATC CCGAGTTTGA   
  
  
+ TGAGTTAATC CAGAATTTTT GCTTTTCTTC TTTTTAATTA ATTTTTTTTT GTTTAGTTTA GTTTGTTAAT   
  
  
+ GTTAAATTTT TTTTATTTAA TTATCAGACT TTTATGACAC GTATCTCGGG TTTGACGGGT TAACGCGTCA   
  
  
+ ATTTTTTTTT CTATTTAGTT ATCAAATTTT TATGACGCGA ATTTCAGGTT TGACAGGTTA ACCTGGTTTG   
  
  
+ AAGGGTTAAC CCATTTAATT CAATTTTTTT TCTTTTTCTT CATTAGTTTT TTTCTTCCAT TTAATTTTTT   
  
  
+ TTCTTTTTCT TCATTAATGT TATTATTAAT ATTATAAATA TCACTCTTGG ATCAGGCGTT GCAGCTAAAT   
  
  
+ CTAAAACTCA TAGGTATAGC TTTATAGAAA TTTGAATCTT AGTTTTTTTT TATATTTTTT ATACGAAAAA   
  
  
+ ATTAACCCGT GCAGGTAATG TGACTAGTAA TAACATTATT TTCTGTTGAA CCGAACAAGA AAGAATCGTA   
  
  
+ GTTAGGTGTC AGAGACTCAG AGTCGCTGCT TCTCACATTC AAACCTTGTA AGCGTTTGGT CATTGTAAGA   
  
  
+ CCCTCTCTTT CTTTTATCAA ATAACCTTTC TTTATTTCTT AAATAAATTA AGGACCCTTC TTTTTTTATA   
  
  
+ AAAAATAAAA ATAAAAAATT CTTCCTTCTT CCCATAATTG ATTCAATCGC AAATGAGTCA TGTCATGACC   
  
  
+ CGGAGACCCT TTGAAAAGTC AAATTAGATA AAGATTGAGA CGATGGTTTC AAGAGACACA ACCACTCAAG   
  
  
+ TATTTACCAT TGTGATGAGC TGACAAGGAT TGTCATCCAG AATGGTCTCT CCTCACGATG TCACCAGCCA   
  
  
+ GTGGAATCCA AATTAAATTG AATTTATTGG GAAATGGGAT AACTCTTCCT TTTATATATT AAAAATTAAA   
  
  
+ AAATATATAC AGTGCCAATT CTACTGTACT GGTTAATTAC CCTATCAACA TTTAAGTAGT TTATGTAACG   
  
  
+ TTGTATTAGT TGTTTTTTAA AATAATTTTT TTTTAATATA TTAAAATAAT TTTTTTAAAA AATTTATTTT   
  
  
+ TGATATTAAT ATGTTAAAAC AATTTAAAAA CATTGAAAAA AAATAAAATA AAATAAATTC AAGTTTTGCA   
  
  
+ CAAAAAAACA CTATTGCACC GCGTACATAA ATGATGTCTT AGTGTATTTA GCATTGTGAT AGCTTTTGTG   
  
  
+ ATCATTCTGG TTTTAAAAAA ACAAATTTAA AAGAAATATT TTTAGTTGTG GTTTTAAAAA GTAGATTTTA   
  
  
+ AAAAATACAT GTTTGATTAA ATCTACTATG AGATAAATTT TTACATGTAA AATAAATAAA AACATATTTC   
  
  
+ CATCAATTAA AAAAAAACAT GTTCTTTCAG TTTCTACATG ACTAATATTT AAAATACAAT TACTTATAAA   
  
  
+ GTCCGGTACG AAAGCACCCT CTAAATGGAG AATGAACTGT ATGTATTCTT CCCATGGACC TGTGATTTTT   
  
  
+ CCGTAGGAAA GGCAATTTCC ATTCAGAATA CAAGAAGATA CATGTCTTTT GAAACTCTTA TTAGCTGTCT   
  
  
+ ACCTATCAAC CAAGAATGAC GCTTCTCATC GTATGTCACT ATAAAATTTG GTTGATGACA CCAACACTTT   
  
  
+ CCCATTGGTA TTCATGGCTT TTTAGCTGCA CGATCCAAC  

- AATAGTTTGA AAGTACTACA CTTAGAGTCC AAACTGCCCA ATTGAACCAA ACTTCCCAAT TGGGTCAATT   
  
  
- AAGTTTAAAA AAAAAAGAAG TAATCAAAAA AAAAGAAGGA CAACTAAAAA AAGTAACTTG AATATATAAA   
  
  
- ATTAATATTA TTATATTATT ATTAATGTAT ATGATTTTAT GACTCAACAG AAGAAAAAAA AAATGCAAAA   
  
  
- ATTTAATAAT AAAAAAACAA AAACAAAAAA AGAAACACAA AAAAAATAAA ATTACTTAAA AAAAACAAAT   
  
  
- TAAATCAAAC AATTACAAAT TAAAAAAAAT AAATCAATAG TATAAAAGTA TTATGCCTAG GGCTCAAACT   
  
  
- ACTCAATTAG GTCTTAAAAA CGAAAAGAAG AAAAATTAAT TAAAAAAAAA CAAATCAAAT CAAACAATTA   
  
  
- CAATTTAAAA AAAATAAATT AATAGTCTGA AAATACTGTG CATAGAGCCC AAACTGCCCA ATTGCGCAGT   
  
  
- TAAAAAAAAA GATAAATCAA TAGTTTAAAA ATACTGCGCT TAAAGTCCAA ACTGTCCAAT TGGACCAAAC   
  
  
- TTCCCAATTG GGTAAATTAA GTTAAAAAAA AGAAAAAGAA GTAATCAAAA AAAGAAGGTA AATTAAAAAA   
  
  
- AAGAAAAAGA AGTAATTACA ATAATAATTA TAATATTTAT AGTGAGAACC TAGTCCGCAA CGTCGATTTA   
  
  
- GATTTTGAGT ATCCATATCG AAATATCTTT AAACTTAGAA TCAAAAAAAA ATATAAAAAA TATGCTTTTT   
  
  
- TAATTGGGCA CGTCCATTAC ACTGATCATT ATTGTAATAA AAGACAACTT GGCTTGTTCT TTCTTAGCAT   
  
  
- CAATCCACAG TCTCTGAGTC TCAGCGACGA AGAGTGTAAG TTTGGAACAT TCGCAAACCA GTAACATTCT   
  
  
- GGGAGAGAAA GAAAATAGTT TATTGGAAAG AAATAAAGAA TTTATTTAAT TCCTGGGAAG AAAAAAATAT   
  
  
- TTTTTATTTT TATTTTTTAA GAAGGAAGAA GGGTATTAAC TAAGTTAGCG TTTACTCAGT ACAGTACTGG   
  
  
- GCCTCTGGGA AACTTTTCAG TTTAATCTAT TTCTAACTCT GCTACCAAAG TTCTCTGTGT TGGTGAGTTC   
  
  
- ATAAATGGTA ACACTACTCG ACTGTTCCTA ACAGTAGGTC TTACCAGAGA GGAGTGCTAC AGTGGTCGGT   
  
  
- CACCTTAGGT TTAATTTAAC TTAAATAACC CTTTACCCTA TTGAGAAGGA AAATATATAA TTTTTAATTT   
  
  
- TTTATATATG TCACGGTTAA GATGACATGA CCAATTAATG GGATAGTTGT AAATTCATCA AATACATTGC   
  
  
- AACATAATCA ACAAAAAATT TTATTAAAAA AAAATTATAT AATTTTATTA AAAAAATTTT TTAAATAAAA   
  
  
- ACTATAATTA TACAATTTTG TTAAATTTTT GTAACTTTTT TTTATTTTAT TTTATTTAAG TTCAAAACGT   
  
  
- GTTTTTTTGT GATAACGTGG CGCATGTATT TACTACAGAA TCACATAAAT CGTAACACTA TCGAAAACAC   
  
  
- TAGTAAGACC AAAATTTTTT TGTTTAAATT TTCTTTATAA AAATCAACAC CAAAATTTTT CATCTAAAAT   
  
  
- TTTTTATGTA CAAACTAATT TAGATGATAC TCTATTTAAA AATGTACATT TTATTTATTT TTGTATAAAG   
  
  
- GTAGTTAATT TTTTTTTGTA CAAGAAAGTC AAAGATGTAC TGATTATAAA TTTTATGTTA ATGAATATTT   
  
  
- CAGGCCATGC TTTCGTGGGA GATTTACCTC TTACTTGACA TACATAAGAA GGGTACCTGG ACACTAAAAA   
  
  
- GGCATCCTTT CCGTTAAAGG TAAGTCTTAT GTTCTTCTAT GTACAGAAAA CTTTGAGAAT AATCGACAGA   
  
  
- TGGATAGTTG GTTCTTACTG CGAAGAGTAG CATACAGTGA TATTTTAAAC CAACTACTGT GGTTGTGAAA   
  
  
- GGGTAACCAT AAGTACCGAA AAATCGACGT GCTAGGTTG

+     TCT-motif

| Site Name | Organism | Position | Strand | Matrix score. | sequence | function |
| --- | --- | --- | --- | --- | --- | --- |
| TCT-motif | Arabidopsis thaliana | 905 | - | 6 | TCTTAC | part of a light responsive element |

>Potri.004G051700.1   
+ TTATCAAACT TTCATGATGT GAATCTCAGG TTTGACGGGT TAACTTGGTT TGAAGGGTTA ACCCAGTTAA   
  
  
+ TTCAAATTTT TTTTTTCTTC ATTAGTTTTT TTTTCTTCCT GTTGATTTTT TTCATTGAAC TTATATATTT   
  
  
+ TAATTATAAT AATATAATAA TAATTACATA TACTAAAATA CTGAGTTGTC TTCTTTTTTT TTTACGTTTT   
  
  
+ TAAATTATTA TTTTTTTGTT TTTGTTTTTT TCTTTGTGTT TTTTTTATTT TAATGAATTT TTTTTGTTTA   
  
  
+ ATTTAGTTTG TTAATGTTTA ATTTTTTTTA TTTAGTTATC ATATTTTCAT AATACGGATC CCGAGTTTGA   
  
  
+ TGAGTTAATC CAGAATTTTT GCTTTTCTTC TTTTTAATTA ATTTTTTTTT GTTTAGTTTA GTTTGTTAAT   
  
  
+ GTTAAATTTT TTTTATTTAA TTATCAGACT TTTATGACAC GTATCTCGGG TTTGACGGGT TAACGCGTCA   
  
  
+ ATTTTTTTTT CTATTTAGTT ATCAAATTTT TATGACGCGA ATTTCAGGTT TGACAGGTTA ACCTGGTTTG   
  
  
+ AAGGGTTAAC CCATTTAATT CAATTTTTTT TCTTTTTCTT CATTAGTTTT TTTCTTCCAT TTAATTTTTT   
  
  
+ TTCTTTTTCT TCATTAATGT TATTATTAAT ATTATAAATA TCACTCTTGG ATCAGGCGTT GCAGCTAAAT   
  
  
+ CTAAAACTCA TAGGTATAGC TTTATAGAAA TTTGAATCTT AGTTTTTTTT TATATTTTTT ATACGAAAAA   
  
  
+ ATTAACCCGT GCAGGTAATG TGACTAGTAA TAACATTATT TTCTGTTGAA CCGAACAAGA AAGAATCGTA   
  
  
+ GTTAGGTGTC AGAGACTCAG AGTCGCTGCT TCTCACATTC AAACCTTGTA AGCGTTTGGT CATTGTAAGA   
  
  
+ CCCTCTCTTT CTTTTATCAA ATAACCTTTC TTTATTTCTT AAATAAATTA AGGACCCTTC TTTTTTTATA   
  
  
+ AAAAATAAAA ATAAAAAATT CTTCCTTCTT CCCATAATTG ATTCAATCGC AAATGAGTCA TGTCATGACC   
  
  
+ CGGAGACCCT TTGAAAAGTC AAATTAGATA AAGATTGAGA CGATGGTTTC AAGAGACACA ACCACTCAAG   
  
  
+ TATTTACCAT TGTGATGAGC TGACAAGGAT TGTCATCCAG AATGGTCTCT CCTCACGATG TCACCAGCCA   
  
  
+ GTGGAATCCA AATTAAATTG AATTTATTGG GAAATGGGAT AACTCTTCCT TTTATATATT AAAAATTAAA   
  
  
+ AAATATATAC AGTGCCAATT CTACTGTACT GGTTAATTAC CCTATCAACA TTTAAGTAGT TTATGTAACG   
  
  
+ TTGTATTAGT TGTTTTTTAA AATAATTTTT TTTTAATATA TTAAAATAAT TTTTTTAAAA AATTTATTTT   
  
  
+ TGATATTAAT ATGTTAAAAC AATTTAAAAA CATTGAAAAA AAATAAAATA AAATAAATTC AAGTTTTGCA   
  
  
+ CAAAAAAACA CTATTGCACC GCGTACATAA ATGATGTCTT AGTGTATTTA GCATTGTGAT AGCTTTTGTG   
  
  
+ ATCATTCTGG TTTTAAAAAA ACAAATTTAA AAGAAATATT TTTAGTTGTG GTTTTAAAAA GTAGATTTTA   
  
  
+ AAAAATACAT GTTTGATTAA ATCTACTATG AGATAAATTT TTACATGTAA AATAAATAAA AACATATTTC   
  
  
+ CATCAATTAA AAAAAAACAT GTTCTTTCAG TTTCTACATG ACTAATATTT AAAATACAAT TACTTATAAA   
  
  
+ GTCCGGTACG AAAGCACCCT CTAAATGGAG AATGAACTGT ATGTATTCTT CCCATGGACC TGTGATTTTT   
  
  
+ CCGTAGGAAA GGCAATTTCC ATTCAGAATA CAAGAAGATA CATGTCTTTT GAAACTCTTA TTAGCTGTCT   
  
  
+ ACCTATCAAC CAAGAATGAC GCTTCTCATC GTATGTCACT ATAAAATTTG GTTGATGACA CCAACACTTT   
  
  
+ CCCATTGGTA TTCATGGCTT TTTAGCTGCA CGATCCAAC  

- AATAGTTTGA AAGTACTACA CTTAGAGTCC AAACTGCCCA ATTGAACCAA ACTTCCCAAT TGGGTCAATT   
  
  
- AAGTTTAAAA AAAAAAGAAG TAATCAAAAA AAAAGAAGGA CAACTAAAAA AAGTAACTTG AATATATAAA   
  
  
- ATTAATATTA TTATATTATT ATTAATGTAT ATGATTTTAT GACTCAACAG AAGAAAAAAA AAATGCAAAA   
  
  
- ATTTAATAAT AAAAAAACAA AAACAAAAAA AGAAACACAA AAAAAATAAA ATTACTTAAA AAAAACAAAT   
  
  
- TAAATCAAAC AATTACAAAT TAAAAAAAAT AAATCAATAG TATAAAAGTA TTATGCCTAG GGCTCAAACT   
  
  
- ACTCAATTAG GTCTTAAAAA CGAAAAGAAG AAAAATTAAT TAAAAAAAAA CAAATCAAAT CAAACAATTA   
  
  
- CAATTTAAAA AAAATAAATT AATAGTCTGA AAATACTGTG CATAGAGCCC AAACTGCCCA ATTGCGCAGT   
  
  
- TAAAAAAAAA GATAAATCAA TAGTTTAAAA ATACTGCGCT TAAAGTCCAA ACTGTCCAAT TGGACCAAAC   
  
  
- TTCCCAATTG GGTAAATTAA GTTAAAAAAA AGAAAAAGAA GTAATCAAAA AAAGAAGGTA AATTAAAAAA   
  
  
- AAGAAAAAGA AGTAATTACA ATAATAATTA TAATATTTAT AGTGAGAACC TAGTCCGCAA CGTCGATTTA   
  
  
- GATTTTGAGT ATCCATATCG AAATATCTTT AAACTTAGAA TCAAAAAAAA ATATAAAAAA TATGCTTTTT   
  
  
- TAATTGGGCA CGTCCATTAC ACTGATCATT ATTGTAATAA AAGACAACTT GGCTTGTTCT TTCTTAGCAT   
  
  
- CAATCCACAG TCTCTGAGTC TCAGCGACGA AGAGTGTAAG TTTGGAACAT TCGCAAACCA GTAACATTCT   
  
  
- GGGAGAGAAA GAAAATAGTT TATTGGAAAG AAATAAAGAA TTTATTTAAT TCCTGGGAAG AAAAAAATAT   
  
  
- TTTTTATTTT TATTTTTTAA GAAGGAAGAA GGGTATTAAC TAAGTTAGCG TTTACTCAGT ACAGTACTGG   
  
  
- GCCTCTGGGA AACTTTTCAG TTTAATCTAT TTCTAACTCT GCTACCAAAG TTCTCTGTGT TGGTGAGTTC   
  
  
- ATAAATGGTA ACACTACTCG ACTGTTCCTA ACAGTAGGTC TTACCAGAGA GGAGTGCTAC AGTGGTCGGT   
  
  
- CACCTTAGGT TTAATTTAAC TTAAATAACC CTTTACCCTA TTGAGAAGGA AAATATATAA TTTTTAATTT   
  
  
- TTTATATATG TCACGGTTAA GATGACATGA CCAATTAATG GGATAGTTGT AAATTCATCA AATACATTGC   
  
  
- AACATAATCA ACAAAAAATT TTATTAAAAA AAAATTATAT AATTTTATTA AAAAAATTTT TTAAATAAAA   
  
  
- ACTATAATTA TACAATTTTG TTAAATTTTT GTAACTTTTT TTTATTTTAT TTTATTTAAG TTCAAAACGT   
  
  
- GTTTTTTTGT GATAACGTGG CGCATGTATT TACTACAGAA TCACATAAAT CGTAACACTA TCGAAAACAC   
  
  
- TAGTAAGACC AAAATTTTTT TGTTTAAATT TTCTTTATAA AAATCAACAC CAAAATTTTT CATCTAAAAT   
  
  
- TTTTTATGTA CAAACTAATT TAGATGATAC TCTATTTAAA AATGTACATT TTATTTATTT TTGTATAAAG   
  
  
- GTAGTTAATT TTTTTTTGTA CAAGAAAGTC AAAGATGTAC TGATTATAAA TTTTATGTTA ATGAATATTT   
  
  
- CAGGCCATGC TTTCGTGGGA GATTTACCTC TTACTTGACA TACATAAGAA GGGTACCTGG ACACTAAAAA   
  
  
- GGCATCCTTT CCGTTAAAGG TAAGTCTTAT GTTCTTCTAT GTACAGAAAA CTTTGAGAAT AATCGACAGA   
  
  
- TGGATAGTTG GTTCTTACTG CGAAGAGTAG CATACAGTGA TATTTTAAAC CAACTACTGT GGTTGTGAAA   
  
  
- GGGTAACCAT AAGTACCGAA AAATCGACGT GCTAGGTTG

+     TGACG-motif

| Site Name | Organism | Position | Strand | Matrix score. | sequence | function |
| --- | --- | --- | --- | --- | --- | --- |
| TGACG-motif | Hordeum vulgare | 523 | + | 5 | TGACG | cis-acting regulatory element involved in the MeJA-responsiveness |
| TGACG-motif | Hordeum vulgare | 486 | - | 5 | TGACG | cis-acting regulatory element involved in the MeJA-responsiveness |
| TGACG-motif | Hordeum vulgare | 473 | + | 5 | TGACG | cis-acting regulatory element involved in the MeJA-responsiveness |
| TGACG-motif | Hordeum vulgare | 33 | + | 5 | TGACG | cis-acting regulatory element involved in the MeJA-responsiveness |
| TGACG-motif | Hordeum vulgare | 1907 | + | 5 | TGACG | cis-acting regulatory element involved in the MeJA-responsiveness |

>Potri.004G051700.1   
+ TTATCAAACT TTCATGATGT GAATCTCAGG TTTGACGGGT TAACTTGGTT TGAAGGGTTA ACCCAGTTAA   
  
  
+ TTCAAATTTT TTTTTTCTTC ATTAGTTTTT TTTTCTTCCT GTTGATTTTT TTCATTGAAC TTATATATTT   
  
  
+ TAATTATAAT AATATAATAA TAATTACATA TACTAAAATA CTGAGTTGTC TTCTTTTTTT TTTACGTTTT   
  
  
+ TAAATTATTA TTTTTTTGTT TTTGTTTTTT TCTTTGTGTT TTTTTTATTT TAATGAATTT TTTTTGTTTA   
  
  
+ ATTTAGTTTG TTAATGTTTA ATTTTTTTTA TTTAGTTATC ATATTTTCAT AATACGGATC CCGAGTTTGA   
  
  
+ TGAGTTAATC CAGAATTTTT GCTTTTCTTC TTTTTAATTA ATTTTTTTTT GTTTAGTTTA GTTTGTTAAT   
  
  
+ GTTAAATTTT TTTTATTTAA TTATCAGACT TTTATGACAC GTATCTCGGG TTTGACGGGT TAACGCGTCA   
  
  
+ ATTTTTTTTT CTATTTAGTT ATCAAATTTT TATGACGCGA ATTTCAGGTT TGACAGGTTA ACCTGGTTTG   
  
  
+ AAGGGTTAAC CCATTTAATT CAATTTTTTT TCTTTTTCTT CATTAGTTTT TTTCTTCCAT TTAATTTTTT   
  
  
+ TTCTTTTTCT TCATTAATGT TATTATTAAT ATTATAAATA TCACTCTTGG ATCAGGCGTT GCAGCTAAAT   
  
  
+ CTAAAACTCA TAGGTATAGC TTTATAGAAA TTTGAATCTT AGTTTTTTTT TATATTTTTT ATACGAAAAA   
  
  
+ ATTAACCCGT GCAGGTAATG TGACTAGTAA TAACATTATT TTCTGTTGAA CCGAACAAGA AAGAATCGTA   
  
  
+ GTTAGGTGTC AGAGACTCAG AGTCGCTGCT TCTCACATTC AAACCTTGTA AGCGTTTGGT CATTGTAAGA   
  
  
+ CCCTCTCTTT CTTTTATCAA ATAACCTTTC TTTATTTCTT AAATAAATTA AGGACCCTTC TTTTTTTATA   
  
  
+ AAAAATAAAA ATAAAAAATT CTTCCTTCTT CCCATAATTG ATTCAATCGC AAATGAGTCA TGTCATGACC   
  
  
+ CGGAGACCCT TTGAAAAGTC AAATTAGATA AAGATTGAGA CGATGGTTTC AAGAGACACA ACCACTCAAG   
  
  
+ TATTTACCAT TGTGATGAGC TGACAAGGAT TGTCATCCAG AATGGTCTCT CCTCACGATG TCACCAGCCA   
  
  
+ GTGGAATCCA AATTAAATTG AATTTATTGG GAAATGGGAT AACTCTTCCT TTTATATATT AAAAATTAAA   
  
  
+ AAATATATAC AGTGCCAATT CTACTGTACT GGTTAATTAC CCTATCAACA TTTAAGTAGT TTATGTAACG   
  
  
+ TTGTATTAGT TGTTTTTTAA AATAATTTTT TTTTAATATA TTAAAATAAT TTTTTTAAAA AATTTATTTT   
  
  
+ TGATATTAAT ATGTTAAAAC AATTTAAAAA CATTGAAAAA AAATAAAATA AAATAAATTC AAGTTTTGCA   
  
  
+ CAAAAAAACA CTATTGCACC GCGTACATAA ATGATGTCTT AGTGTATTTA GCATTGTGAT AGCTTTTGTG   
  
  
+ ATCATTCTGG TTTTAAAAAA ACAAATTTAA AAGAAATATT TTTAGTTGTG GTTTTAAAAA GTAGATTTTA   
  
  
+ AAAAATACAT GTTTGATTAA ATCTACTATG AGATAAATTT TTACATGTAA AATAAATAAA AACATATTTC   
  
  
+ CATCAATTAA AAAAAAACAT GTTCTTTCAG TTTCTACATG ACTAATATTT AAAATACAAT TACTTATAAA   
  
  
+ GTCCGGTACG AAAGCACCCT CTAAATGGAG AATGAACTGT ATGTATTCTT CCCATGGACC TGTGATTTTT   
  
  
+ CCGTAGGAAA GGCAATTTCC ATTCAGAATA CAAGAAGATA CATGTCTTTT GAAACTCTTA TTAGCTGTCT   
  
  
+ ACCTATCAAC CAAGAATGAC GCTTCTCATC GTATGTCACT ATAAAATTTG GTTGATGACA CCAACACTTT   
  
  
+ CCCATTGGTA TTCATGGCTT TTTAGCTGCA CGATCCAAC  

- AATAGTTTGA AAGTACTACA CTTAGAGTCC AAACTGCCCA ATTGAACCAA ACTTCCCAAT TGGGTCAATT   
  
  
- AAGTTTAAAA AAAAAAGAAG TAATCAAAAA AAAAGAAGGA CAACTAAAAA AAGTAACTTG AATATATAAA   
  
  
- ATTAATATTA TTATATTATT ATTAATGTAT ATGATTTTAT GACTCAACAG AAGAAAAAAA AAATGCAAAA   
  
  
- ATTTAATAAT AAAAAAACAA AAACAAAAAA AGAAACACAA AAAAAATAAA ATTACTTAAA AAAAACAAAT   
  
  
- TAAATCAAAC AATTACAAAT TAAAAAAAAT AAATCAATAG TATAAAAGTA TTATGCCTAG GGCTCAAACT   
  
  
- ACTCAATTAG GTCTTAAAAA CGAAAAGAAG AAAAATTAAT TAAAAAAAAA CAAATCAAAT CAAACAATTA   
  
  
- CAATTTAAAA AAAATAAATT AATAGTCTGA AAATACTGTG CATAGAGCCC AAACTGCCCA ATTGCGCAGT   
  
  
- TAAAAAAAAA GATAAATCAA TAGTTTAAAA ATACTGCGCT TAAAGTCCAA ACTGTCCAAT TGGACCAAAC   
  
  
- TTCCCAATTG GGTAAATTAA GTTAAAAAAA AGAAAAAGAA GTAATCAAAA AAAGAAGGTA AATTAAAAAA   
  
  
- AAGAAAAAGA AGTAATTACA ATAATAATTA TAATATTTAT AGTGAGAACC TAGTCCGCAA CGTCGATTTA   
  
  
- GATTTTGAGT ATCCATATCG AAATATCTTT AAACTTAGAA TCAAAAAAAA ATATAAAAAA TATGCTTTTT   
  
  
- TAATTGGGCA CGTCCATTAC ACTGATCATT ATTGTAATAA AAGACAACTT GGCTTGTTCT TTCTTAGCAT   
  
  
- CAATCCACAG TCTCTGAGTC TCAGCGACGA AGAGTGTAAG TTTGGAACAT TCGCAAACCA GTAACATTCT   
  
  
- GGGAGAGAAA GAAAATAGTT TATTGGAAAG AAATAAAGAA TTTATTTAAT TCCTGGGAAG AAAAAAATAT   
  
  
- TTTTTATTTT TATTTTTTAA GAAGGAAGAA GGGTATTAAC TAAGTTAGCG TTTACTCAGT ACAGTACTGG   
  
  
- GCCTCTGGGA AACTTTTCAG TTTAATCTAT TTCTAACTCT GCTACCAAAG TTCTCTGTGT TGGTGAGTTC   
  
  
- ATAAATGGTA ACACTACTCG ACTGTTCCTA ACAGTAGGTC TTACCAGAGA GGAGTGCTAC AGTGGTCGGT   
  
  
- CACCTTAGGT TTAATTTAAC TTAAATAACC CTTTACCCTA TTGAGAAGGA AAATATATAA TTTTTAATTT   
  
  
- TTTATATATG TCACGGTTAA GATGACATGA CCAATTAATG GGATAGTTGT AAATTCATCA AATACATTGC   
  
  
- AACATAATCA ACAAAAAATT TTATTAAAAA AAAATTATAT AATTTTATTA AAAAAATTTT TTAAATAAAA   
  
  
- ACTATAATTA TACAATTTTG TTAAATTTTT GTAACTTTTT TTTATTTTAT TTTATTTAAG TTCAAAACGT   
  
  
- GTTTTTTTGT GATAACGTGG CGCATGTATT TACTACAGAA TCACATAAAT CGTAACACTA TCGAAAACAC   
  
  
- TAGTAAGACC AAAATTTTTT TGTTTAAATT TTCTTTATAA AAATCAACAC CAAAATTTTT CATCTAAAAT   
  
  
- TTTTTATGTA CAAACTAATT TAGATGATAC TCTATTTAAA AATGTACATT TTATTTATTT TTGTATAAAG   
  
  
- GTAGTTAATT TTTTTTTGTA CAAGAAAGTC AAAGATGTAC TGATTATAAA TTTTATGTTA ATGAATATTT   
  
  
- CAGGCCATGC TTTCGTGGGA GATTTACCTC TTACTTGACA TACATAAGAA GGGTACCTGG ACACTAAAAA   
  
  
- GGCATCCTTT CCGTTAAAGG TAAGTCTTAT GTTCTTCTAT GTACAGAAAA CTTTGAGAAT AATCGACAGA   
  
  
- TGGATAGTTG GTTCTTACTG CGAAGAGTAG CATACAGTGA TATTTTAAAC CAACTACTGT GGTTGTGAAA   
  
  
- GGGTAACCAT AAGTACCGAA AAATCGACGT GCTAGGTTG

+     Unnamed\_\_4

| Site Name | Organism | Position | Strand | Matrix score. | sequence | function |
| --- | --- | --- | --- | --- | --- | --- |
| Unnamed\_\_4 | Petroselinum hortense | 1777 | - | 4 | CTCC |  |
| Unnamed\_\_4 | Petroselinum hortense | 1169 | + | 4 | CTCC |  |
| Unnamed\_\_4 | Petroselinum hortense | 1052 | - | 4 | CTCC |  |

>Potri.004G051700.1   
+ TTATCAAACT TTCATGATGT GAATCTCAGG TTTGACGGGT TAACTTGGTT TGAAGGGTTA ACCCAGTTAA   
  
  
+ TTCAAATTTT TTTTTTCTTC ATTAGTTTTT TTTTCTTCCT GTTGATTTTT TTCATTGAAC TTATATATTT   
  
  
+ TAATTATAAT AATATAATAA TAATTACATA TACTAAAATA CTGAGTTGTC TTCTTTTTTT TTTACGTTTT   
  
  
+ TAAATTATTA TTTTTTTGTT TTTGTTTTTT TCTTTGTGTT TTTTTTATTT TAATGAATTT TTTTTGTTTA   
  
  
+ ATTTAGTTTG TTAATGTTTA ATTTTTTTTA TTTAGTTATC ATATTTTCAT AATACGGATC CCGAGTTTGA   
  
  
+ TGAGTTAATC CAGAATTTTT GCTTTTCTTC TTTTTAATTA ATTTTTTTTT GTTTAGTTTA GTTTGTTAAT   
  
  
+ GTTAAATTTT TTTTATTTAA TTATCAGACT TTTATGACAC GTATCTCGGG TTTGACGGGT TAACGCGTCA   
  
  
+ ATTTTTTTTT CTATTTAGTT ATCAAATTTT TATGACGCGA ATTTCAGGTT TGACAGGTTA ACCTGGTTTG   
  
  
+ AAGGGTTAAC CCATTTAATT CAATTTTTTT TCTTTTTCTT CATTAGTTTT TTTCTTCCAT TTAATTTTTT   
  
  
+ TTCTTTTTCT TCATTAATGT TATTATTAAT ATTATAAATA TCACTCTTGG ATCAGGCGTT GCAGCTAAAT   
  
  
+ CTAAAACTCA TAGGTATAGC TTTATAGAAA TTTGAATCTT AGTTTTTTTT TATATTTTTT ATACGAAAAA   
  
  
+ ATTAACCCGT GCAGGTAATG TGACTAGTAA TAACATTATT TTCTGTTGAA CCGAACAAGA AAGAATCGTA   
  
  
+ GTTAGGTGTC AGAGACTCAG AGTCGCTGCT TCTCACATTC AAACCTTGTA AGCGTTTGGT CATTGTAAGA   
  
  
+ CCCTCTCTTT CTTTTATCAA ATAACCTTTC TTTATTTCTT AAATAAATTA AGGACCCTTC TTTTTTTATA   
  
  
+ AAAAATAAAA ATAAAAAATT CTTCCTTCTT CCCATAATTG ATTCAATCGC AAATGAGTCA TGTCATGACC   
  
  
+ CGGAGACCCT TTGAAAAGTC AAATTAGATA AAGATTGAGA CGATGGTTTC AAGAGACACA ACCACTCAAG   
  
  
+ TATTTACCAT TGTGATGAGC TGACAAGGAT TGTCATCCAG AATGGTCTCT CCTCACGATG TCACCAGCCA   
  
  
+ GTGGAATCCA AATTAAATTG AATTTATTGG GAAATGGGAT AACTCTTCCT TTTATATATT AAAAATTAAA   
  
  
+ AAATATATAC AGTGCCAATT CTACTGTACT GGTTAATTAC CCTATCAACA TTTAAGTAGT TTATGTAACG   
  
  
+ TTGTATTAGT TGTTTTTTAA AATAATTTTT TTTTAATATA TTAAAATAAT TTTTTTAAAA AATTTATTTT   
  
  
+ TGATATTAAT ATGTTAAAAC AATTTAAAAA CATTGAAAAA AAATAAAATA AAATAAATTC AAGTTTTGCA   
  
  
+ CAAAAAAACA CTATTGCACC GCGTACATAA ATGATGTCTT AGTGTATTTA GCATTGTGAT AGCTTTTGTG   
  
  
+ ATCATTCTGG TTTTAAAAAA ACAAATTTAA AAGAAATATT TTTAGTTGTG GTTTTAAAAA GTAGATTTTA   
  
  
+ AAAAATACAT GTTTGATTAA ATCTACTATG AGATAAATTT TTACATGTAA AATAAATAAA AACATATTTC   
  
  
+ CATCAATTAA AAAAAAACAT GTTCTTTCAG TTTCTACATG ACTAATATTT AAAATACAAT TACTTATAAA   
  
  
+ GTCCGGTACG AAAGCACCCT CTAAATGGAG AATGAACTGT ATGTATTCTT CCCATGGACC TGTGATTTTT   
  
  
+ CCGTAGGAAA GGCAATTTCC ATTCAGAATA CAAGAAGATA CATGTCTTTT GAAACTCTTA TTAGCTGTCT   
  
  
+ ACCTATCAAC CAAGAATGAC GCTTCTCATC GTATGTCACT ATAAAATTTG GTTGATGACA CCAACACTTT   
  
  
+ CCCATTGGTA TTCATGGCTT TTTAGCTGCA CGATCCAAC  

- AATAGTTTGA AAGTACTACA CTTAGAGTCC AAACTGCCCA ATTGAACCAA ACTTCCCAAT TGGGTCAATT   
  
  
- AAGTTTAAAA AAAAAAGAAG TAATCAAAAA AAAAGAAGGA CAACTAAAAA AAGTAACTTG AATATATAAA   
  
  
- ATTAATATTA TTATATTATT ATTAATGTAT ATGATTTTAT GACTCAACAG AAGAAAAAAA AAATGCAAAA   
  
  
- ATTTAATAAT AAAAAAACAA AAACAAAAAA AGAAACACAA AAAAAATAAA ATTACTTAAA AAAAACAAAT   
  
  
- TAAATCAAAC AATTACAAAT TAAAAAAAAT AAATCAATAG TATAAAAGTA TTATGCCTAG GGCTCAAACT   
  
  
- ACTCAATTAG GTCTTAAAAA CGAAAAGAAG AAAAATTAAT TAAAAAAAAA CAAATCAAAT CAAACAATTA   
  
  
- CAATTTAAAA AAAATAAATT AATAGTCTGA AAATACTGTG CATAGAGCCC AAACTGCCCA ATTGCGCAGT   
  
  
- TAAAAAAAAA GATAAATCAA TAGTTTAAAA ATACTGCGCT TAAAGTCCAA ACTGTCCAAT TGGACCAAAC   
  
  
- TTCCCAATTG GGTAAATTAA GTTAAAAAAA AGAAAAAGAA GTAATCAAAA AAAGAAGGTA AATTAAAAAA   
  
  
- AAGAAAAAGA AGTAATTACA ATAATAATTA TAATATTTAT AGTGAGAACC TAGTCCGCAA CGTCGATTTA   
  
  
- GATTTTGAGT ATCCATATCG AAATATCTTT AAACTTAGAA TCAAAAAAAA ATATAAAAAA TATGCTTTTT   
  
  
- TAATTGGGCA CGTCCATTAC ACTGATCATT ATTGTAATAA AAGACAACTT GGCTTGTTCT TTCTTAGCAT   
  
  
- CAATCCACAG TCTCTGAGTC TCAGCGACGA AGAGTGTAAG TTTGGAACAT TCGCAAACCA GTAACATTCT   
  
  
- GGGAGAGAAA GAAAATAGTT TATTGGAAAG AAATAAAGAA TTTATTTAAT TCCTGGGAAG AAAAAAATAT   
  
  
- TTTTTATTTT TATTTTTTAA GAAGGAAGAA GGGTATTAAC TAAGTTAGCG TTTACTCAGT ACAGTACTGG   
  
  
- GCCTCTGGGA AACTTTTCAG TTTAATCTAT TTCTAACTCT GCTACCAAAG TTCTCTGTGT TGGTGAGTTC   
  
  
- ATAAATGGTA ACACTACTCG ACTGTTCCTA ACAGTAGGTC TTACCAGAGA GGAGTGCTAC AGTGGTCGGT   
  
  
- CACCTTAGGT TTAATTTAAC TTAAATAACC CTTTACCCTA TTGAGAAGGA AAATATATAA TTTTTAATTT   
  
  
- TTTATATATG TCACGGTTAA GATGACATGA CCAATTAATG GGATAGTTGT AAATTCATCA AATACATTGC   
  
  
- AACATAATCA ACAAAAAATT TTATTAAAAA AAAATTATAT AATTTTATTA AAAAAATTTT TTAAATAAAA   
  
  
- ACTATAATTA TACAATTTTG TTAAATTTTT GTAACTTTTT TTTATTTTAT TTTATTTAAG TTCAAAACGT   
  
  
- GTTTTTTTGT GATAACGTGG CGCATGTATT TACTACAGAA TCACATAAAT CGTAACACTA TCGAAAACAC   
  
  
- TAGTAAGACC AAAATTTTTT TGTTTAAATT TTCTTTATAA AAATCAACAC CAAAATTTTT CATCTAAAAT   
  
  
- TTTTTATGTA CAAACTAATT TAGATGATAC TCTATTTAAA AATGTACATT TTATTTATTT TTGTATAAAG   
  
  
- GTAGTTAATT TTTTTTTGTA CAAGAAAGTC AAAGATGTAC TGATTATAAA TTTTATGTTA ATGAATATTT   
  
  
- CAGGCCATGC TTTCGTGGGA GATTTACCTC TTACTTGACA TACATAAGAA GGGTACCTGG ACACTAAAAA   
  
  
- GGCATCCTTT CCGTTAAAGG TAAGTCTTAT GTTCTTCTAT GTACAGAAAA CTTTGAGAAT AATCGACAGA   
  
  
- TGGATAGTTG GTTCTTACTG CGAAGAGTAG CATACAGTGA TATTTTAAAC CAACTACTGT GGTTGTGAAA   
  
  
- GGGTAACCAT AAGTACCGAA AAATCGACGT GCTAGGTTG

+     Unnamed\_\_6

| Site Name | Organism | Position | Strand | Matrix score. | sequence | function |
| --- | --- | --- | --- | --- | --- | --- |
| Unnamed\_\_6 | Zea mays | 663 | + | 10 | taTAAATATct |  |

>Potri.004G051700.1   
+ TTATCAAACT TTCATGATGT GAATCTCAGG TTTGACGGGT TAACTTGGTT TGAAGGGTTA ACCCAGTTAA   
  
  
+ TTCAAATTTT TTTTTTCTTC ATTAGTTTTT TTTTCTTCCT GTTGATTTTT TTCATTGAAC TTATATATTT   
  
  
+ TAATTATAAT AATATAATAA TAATTACATA TACTAAAATA CTGAGTTGTC TTCTTTTTTT TTTACGTTTT   
  
  
+ TAAATTATTA TTTTTTTGTT TTTGTTTTTT TCTTTGTGTT TTTTTTATTT TAATGAATTT TTTTTGTTTA   
  
  
+ ATTTAGTTTG TTAATGTTTA ATTTTTTTTA TTTAGTTATC ATATTTTCAT AATACGGATC CCGAGTTTGA   
  
  
+ TGAGTTAATC CAGAATTTTT GCTTTTCTTC TTTTTAATTA ATTTTTTTTT GTTTAGTTTA GTTTGTTAAT   
  
  
+ GTTAAATTTT TTTTATTTAA TTATCAGACT TTTATGACAC GTATCTCGGG TTTGACGGGT TAACGCGTCA   
  
  
+ ATTTTTTTTT CTATTTAGTT ATCAAATTTT TATGACGCGA ATTTCAGGTT TGACAGGTTA ACCTGGTTTG   
  
  
+ AAGGGTTAAC CCATTTAATT CAATTTTTTT TCTTTTTCTT CATTAGTTTT TTTCTTCCAT TTAATTTTTT   
  
  
+ TTCTTTTTCT TCATTAATGT TATTATTAAT ATTATAAATA TCACTCTTGG ATCAGGCGTT GCAGCTAAAT   
  
  
+ CTAAAACTCA TAGGTATAGC TTTATAGAAA TTTGAATCTT AGTTTTTTTT TATATTTTTT ATACGAAAAA   
  
  
+ ATTAACCCGT GCAGGTAATG TGACTAGTAA TAACATTATT TTCTGTTGAA CCGAACAAGA AAGAATCGTA   
  
  
+ GTTAGGTGTC AGAGACTCAG AGTCGCTGCT TCTCACATTC AAACCTTGTA AGCGTTTGGT CATTGTAAGA   
  
  
+ CCCTCTCTTT CTTTTATCAA ATAACCTTTC TTTATTTCTT AAATAAATTA AGGACCCTTC TTTTTTTATA   
  
  
+ AAAAATAAAA ATAAAAAATT CTTCCTTCTT CCCATAATTG ATTCAATCGC AAATGAGTCA TGTCATGACC   
  
  
+ CGGAGACCCT TTGAAAAGTC AAATTAGATA AAGATTGAGA CGATGGTTTC AAGAGACACA ACCACTCAAG   
  
  
+ TATTTACCAT TGTGATGAGC TGACAAGGAT TGTCATCCAG AATGGTCTCT CCTCACGATG TCACCAGCCA   
  
  
+ GTGGAATCCA AATTAAATTG AATTTATTGG GAAATGGGAT AACTCTTCCT TTTATATATT AAAAATTAAA   
  
  
+ AAATATATAC AGTGCCAATT CTACTGTACT GGTTAATTAC CCTATCAACA TTTAAGTAGT TTATGTAACG   
  
  
+ TTGTATTAGT TGTTTTTTAA AATAATTTTT TTTTAATATA TTAAAATAAT TTTTTTAAAA AATTTATTTT   
  
  
+ TGATATTAAT ATGTTAAAAC AATTTAAAAA CATTGAAAAA AAATAAAATA AAATAAATTC AAGTTTTGCA   
  
  
+ CAAAAAAACA CTATTGCACC GCGTACATAA ATGATGTCTT AGTGTATTTA GCATTGTGAT AGCTTTTGTG   
  
  
+ ATCATTCTGG TTTTAAAAAA ACAAATTTAA AAGAAATATT TTTAGTTGTG GTTTTAAAAA GTAGATTTTA   
  
  
+ AAAAATACAT GTTTGATTAA ATCTACTATG AGATAAATTT TTACATGTAA AATAAATAAA AACATATTTC   
  
  
+ CATCAATTAA AAAAAAACAT GTTCTTTCAG TTTCTACATG ACTAATATTT AAAATACAAT TACTTATAAA   
  
  
+ GTCCGGTACG AAAGCACCCT CTAAATGGAG AATGAACTGT ATGTATTCTT CCCATGGACC TGTGATTTTT   
  
  
+ CCGTAGGAAA GGCAATTTCC ATTCAGAATA CAAGAAGATA CATGTCTTTT GAAACTCTTA TTAGCTGTCT   
  
  
+ ACCTATCAAC CAAGAATGAC GCTTCTCATC GTATGTCACT ATAAAATTTG GTTGATGACA CCAACACTTT   
  
  
+ CCCATTGGTA TTCATGGCTT TTTAGCTGCA CGATCCAAC  

- AATAGTTTGA AAGTACTACA CTTAGAGTCC AAACTGCCCA ATTGAACCAA ACTTCCCAAT TGGGTCAATT   
  
  
- AAGTTTAAAA AAAAAAGAAG TAATCAAAAA AAAAGAAGGA CAACTAAAAA AAGTAACTTG AATATATAAA   
  
  
- ATTAATATTA TTATATTATT ATTAATGTAT ATGATTTTAT GACTCAACAG AAGAAAAAAA AAATGCAAAA   
  
  
- ATTTAATAAT AAAAAAACAA AAACAAAAAA AGAAACACAA AAAAAATAAA ATTACTTAAA AAAAACAAAT   
  
  
- TAAATCAAAC AATTACAAAT TAAAAAAAAT AAATCAATAG TATAAAAGTA TTATGCCTAG GGCTCAAACT   
  
  
- ACTCAATTAG GTCTTAAAAA CGAAAAGAAG AAAAATTAAT TAAAAAAAAA CAAATCAAAT CAAACAATTA   
  
  
- CAATTTAAAA AAAATAAATT AATAGTCTGA AAATACTGTG CATAGAGCCC AAACTGCCCA ATTGCGCAGT   
  
  
- TAAAAAAAAA GATAAATCAA TAGTTTAAAA ATACTGCGCT TAAAGTCCAA ACTGTCCAAT TGGACCAAAC   
  
  
- TTCCCAATTG GGTAAATTAA GTTAAAAAAA AGAAAAAGAA GTAATCAAAA AAAGAAGGTA AATTAAAAAA   
  
  
- AAGAAAAAGA AGTAATTACA ATAATAATTA TAATATTTAT AGTGAGAACC TAGTCCGCAA CGTCGATTTA   
  
  
- GATTTTGAGT ATCCATATCG AAATATCTTT AAACTTAGAA TCAAAAAAAA ATATAAAAAA TATGCTTTTT   
  
  
- TAATTGGGCA CGTCCATTAC ACTGATCATT ATTGTAATAA AAGACAACTT GGCTTGTTCT TTCTTAGCAT   
  
  
- CAATCCACAG TCTCTGAGTC TCAGCGACGA AGAGTGTAAG TTTGGAACAT TCGCAAACCA GTAACATTCT   
  
  
- GGGAGAGAAA GAAAATAGTT TATTGGAAAG AAATAAAGAA TTTATTTAAT TCCTGGGAAG AAAAAAATAT   
  
  
- TTTTTATTTT TATTTTTTAA GAAGGAAGAA GGGTATTAAC TAAGTTAGCG TTTACTCAGT ACAGTACTGG   
  
  
- GCCTCTGGGA AACTTTTCAG TTTAATCTAT TTCTAACTCT GCTACCAAAG TTCTCTGTGT TGGTGAGTTC   
  
  
- ATAAATGGTA ACACTACTCG ACTGTTCCTA ACAGTAGGTC TTACCAGAGA GGAGTGCTAC AGTGGTCGGT   
  
  
- CACCTTAGGT TTAATTTAAC TTAAATAACC CTTTACCCTA TTGAGAAGGA AAATATATAA TTTTTAATTT   
  
  
- TTTATATATG TCACGGTTAA GATGACATGA CCAATTAATG GGATAGTTGT AAATTCATCA AATACATTGC   
  
  
- AACATAATCA ACAAAAAATT TTATTAAAAA AAAATTATAT AATTTTATTA AAAAAATTTT TTAAATAAAA   
  
  
- ACTATAATTA TACAATTTTG TTAAATTTTT GTAACTTTTT TTTATTTTAT TTTATTTAAG TTCAAAACGT   
  
  
- GTTTTTTTGT GATAACGTGG CGCATGTATT TACTACAGAA TCACATAAAT CGTAACACTA TCGAAAACAC   
  
  
- TAGTAAGACC AAAATTTTTT TGTTTAAATT TTCTTTATAA AAATCAACAC CAAAATTTTT CATCTAAAAT   
  
  
- TTTTTATGTA CAAACTAATT TAGATGATAC TCTATTTAAA AATGTACATT TTATTTATTT TTGTATAAAG   
  
  
- GTAGTTAATT TTTTTTTGTA CAAGAAAGTC AAAGATGTAC TGATTATAAA TTTTATGTTA ATGAATATTT   
  
  
- CAGGCCATGC TTTCGTGGGA GATTTACCTC TTACTTGACA TACATAAGAA GGGTACCTGG ACACTAAAAA   
  
  
- GGCATCCTTT CCGTTAAAGG TAAGTCTTAT GTTCTTCTAT GTACAGAAAA CTTTGAGAAT AATCGACAGA   
  
  
- TGGATAGTTG GTTCTTACTG CGAAGAGTAG CATACAGTGA TATTTTAAAC CAACTACTGT GGTTGTGAAA   
  
  
- GGGTAACCAT AAGTACCGAA AAATCGACGT GCTAGGTTG

+     as-1

| Site Name | Organism | Position | Strand | Matrix score. | sequence | function |
| --- | --- | --- | --- | --- | --- | --- |
| as-1 | Arabidopsis thaliana | 1907 | + | 5 | TGACG |  |
| as-1 | Arabidopsis thaliana | 523 | + | 5 | TGACG |  |
| as-1 | Arabidopsis thaliana | 486 | - | 5 | TGACG |  |
| as-1 | Arabidopsis thaliana | 473 | + | 5 | TGACG |  |
| as-1 | Arabidopsis thaliana | 33 | + | 5 | TGACG |  |

>Potri.004G051700.1   
+ TTATCAAACT TTCATGATGT GAATCTCAGG TTTGACGGGT TAACTTGGTT TGAAGGGTTA ACCCAGTTAA   
  
  
+ TTCAAATTTT TTTTTTCTTC ATTAGTTTTT TTTTCTTCCT GTTGATTTTT TTCATTGAAC TTATATATTT   
  
  
+ TAATTATAAT AATATAATAA TAATTACATA TACTAAAATA CTGAGTTGTC TTCTTTTTTT TTTACGTTTT   
  
  
+ TAAATTATTA TTTTTTTGTT TTTGTTTTTT TCTTTGTGTT TTTTTTATTT TAATGAATTT TTTTTGTTTA   
  
  
+ ATTTAGTTTG TTAATGTTTA ATTTTTTTTA TTTAGTTATC ATATTTTCAT AATACGGATC CCGAGTTTGA   
  
  
+ TGAGTTAATC CAGAATTTTT GCTTTTCTTC TTTTTAATTA ATTTTTTTTT GTTTAGTTTA GTTTGTTAAT   
  
  
+ GTTAAATTTT TTTTATTTAA TTATCAGACT TTTATGACAC GTATCTCGGG TTTGACGGGT TAACGCGTCA   
  
  
+ ATTTTTTTTT CTATTTAGTT ATCAAATTTT TATGACGCGA ATTTCAGGTT TGACAGGTTA ACCTGGTTTG   
  
  
+ AAGGGTTAAC CCATTTAATT CAATTTTTTT TCTTTTTCTT CATTAGTTTT TTTCTTCCAT TTAATTTTTT   
  
  
+ TTCTTTTTCT TCATTAATGT TATTATTAAT ATTATAAATA TCACTCTTGG ATCAGGCGTT GCAGCTAAAT   
  
  
+ CTAAAACTCA TAGGTATAGC TTTATAGAAA TTTGAATCTT AGTTTTTTTT TATATTTTTT ATACGAAAAA   
  
  
+ ATTAACCCGT GCAGGTAATG TGACTAGTAA TAACATTATT TTCTGTTGAA CCGAACAAGA AAGAATCGTA   
  
  
+ GTTAGGTGTC AGAGACTCAG AGTCGCTGCT TCTCACATTC AAACCTTGTA AGCGTTTGGT CATTGTAAGA   
  
  
+ CCCTCTCTTT CTTTTATCAA ATAACCTTTC TTTATTTCTT AAATAAATTA AGGACCCTTC TTTTTTTATA   
  
  
+ AAAAATAAAA ATAAAAAATT CTTCCTTCTT CCCATAATTG ATTCAATCGC AAATGAGTCA TGTCATGACC   
  
  
+ CGGAGACCCT TTGAAAAGTC AAATTAGATA AAGATTGAGA CGATGGTTTC AAGAGACACA ACCACTCAAG   
  
  
+ TATTTACCAT TGTGATGAGC TGACAAGGAT TGTCATCCAG AATGGTCTCT CCTCACGATG TCACCAGCCA   
  
  
+ GTGGAATCCA AATTAAATTG AATTTATTGG GAAATGGGAT AACTCTTCCT TTTATATATT AAAAATTAAA   
  
  
+ AAATATATAC AGTGCCAATT CTACTGTACT GGTTAATTAC CCTATCAACA TTTAAGTAGT TTATGTAACG   
  
  
+ TTGTATTAGT TGTTTTTTAA AATAATTTTT TTTTAATATA TTAAAATAAT TTTTTTAAAA AATTTATTTT   
  
  
+ TGATATTAAT ATGTTAAAAC AATTTAAAAA CATTGAAAAA AAATAAAATA AAATAAATTC AAGTTTTGCA   
  
  
+ CAAAAAAACA CTATTGCACC GCGTACATAA ATGATGTCTT AGTGTATTTA GCATTGTGAT AGCTTTTGTG   
  
  
+ ATCATTCTGG TTTTAAAAAA ACAAATTTAA AAGAAATATT TTTAGTTGTG GTTTTAAAAA GTAGATTTTA   
  
  
+ AAAAATACAT GTTTGATTAA ATCTACTATG AGATAAATTT TTACATGTAA AATAAATAAA AACATATTTC   
  
  
+ CATCAATTAA AAAAAAACAT GTTCTTTCAG TTTCTACATG ACTAATATTT AAAATACAAT TACTTATAAA   
  
  
+ GTCCGGTACG AAAGCACCCT CTAAATGGAG AATGAACTGT ATGTATTCTT CCCATGGACC TGTGATTTTT   
  
  
+ CCGTAGGAAA GGCAATTTCC ATTCAGAATA CAAGAAGATA CATGTCTTTT GAAACTCTTA TTAGCTGTCT   
  
  
+ ACCTATCAAC CAAGAATGAC GCTTCTCATC GTATGTCACT ATAAAATTTG GTTGATGACA CCAACACTTT   
  
  
+ CCCATTGGTA TTCATGGCTT TTTAGCTGCA CGATCCAAC  

- AATAGTTTGA AAGTACTACA CTTAGAGTCC AAACTGCCCA ATTGAACCAA ACTTCCCAAT TGGGTCAATT   
  
  
- AAGTTTAAAA AAAAAAGAAG TAATCAAAAA AAAAGAAGGA CAACTAAAAA AAGTAACTTG AATATATAAA   
  
  
- ATTAATATTA TTATATTATT ATTAATGTAT ATGATTTTAT GACTCAACAG AAGAAAAAAA AAATGCAAAA   
  
  
- ATTTAATAAT AAAAAAACAA AAACAAAAAA AGAAACACAA AAAAAATAAA ATTACTTAAA AAAAACAAAT   
  
  
- TAAATCAAAC AATTACAAAT TAAAAAAAAT AAATCAATAG TATAAAAGTA TTATGCCTAG GGCTCAAACT   
  
  
- ACTCAATTAG GTCTTAAAAA CGAAAAGAAG AAAAATTAAT TAAAAAAAAA CAAATCAAAT CAAACAATTA   
  
  
- CAATTTAAAA AAAATAAATT AATAGTCTGA AAATACTGTG CATAGAGCCC AAACTGCCCA ATTGCGCAGT   
  
  
- TAAAAAAAAA GATAAATCAA TAGTTTAAAA ATACTGCGCT TAAAGTCCAA ACTGTCCAAT TGGACCAAAC   
  
  
- TTCCCAATTG GGTAAATTAA GTTAAAAAAA AGAAAAAGAA GTAATCAAAA AAAGAAGGTA AATTAAAAAA   
  
  
- AAGAAAAAGA AGTAATTACA ATAATAATTA TAATATTTAT AGTGAGAACC TAGTCCGCAA CGTCGATTTA   
  
  
- GATTTTGAGT ATCCATATCG AAATATCTTT AAACTTAGAA TCAAAAAAAA ATATAAAAAA TATGCTTTTT   
  
  
- TAATTGGGCA CGTCCATTAC ACTGATCATT ATTGTAATAA AAGACAACTT GGCTTGTTCT TTCTTAGCAT   
  
  
- CAATCCACAG TCTCTGAGTC TCAGCGACGA AGAGTGTAAG TTTGGAACAT TCGCAAACCA GTAACATTCT   
  
  
- GGGAGAGAAA GAAAATAGTT TATTGGAAAG AAATAAAGAA TTTATTTAAT TCCTGGGAAG AAAAAAATAT   
  
  
- TTTTTATTTT TATTTTTTAA GAAGGAAGAA GGGTATTAAC TAAGTTAGCG TTTACTCAGT ACAGTACTGG   
  
  
- GCCTCTGGGA AACTTTTCAG TTTAATCTAT TTCTAACTCT GCTACCAAAG TTCTCTGTGT TGGTGAGTTC   
  
  
- ATAAATGGTA ACACTACTCG ACTGTTCCTA ACAGTAGGTC TTACCAGAGA GGAGTGCTAC AGTGGTCGGT   
  
  
- CACCTTAGGT TTAATTTAAC TTAAATAACC CTTTACCCTA TTGAGAAGGA AAATATATAA TTTTTAATTT   
  
  
- TTTATATATG TCACGGTTAA GATGACATGA CCAATTAATG GGATAGTTGT AAATTCATCA AATACATTGC   
  
  
- AACATAATCA ACAAAAAATT TTATTAAAAA AAAATTATAT AATTTTATTA AAAAAATTTT TTAAATAAAA   
  
  
- ACTATAATTA TACAATTTTG TTAAATTTTT GTAACTTTTT TTTATTTTAT TTTATTTAAG TTCAAAACGT   
  
  
- GTTTTTTTGT GATAACGTGG CGCATGTATT TACTACAGAA TCACATAAAT CGTAACACTA TCGAAAACAC   
  
  
- TAGTAAGACC AAAATTTTTT TGTTTAAATT TTCTTTATAA AAATCAACAC CAAAATTTTT CATCTAAAAT   
  
  
- TTTTTATGTA CAAACTAATT TAGATGATAC TCTATTTAAA AATGTACATT TTATTTATTT TTGTATAAAG   
  
  
- GTAGTTAATT TTTTTTTGTA CAAGAAAGTC AAAGATGTAC TGATTATAAA TTTTATGTTA ATGAATATTT   
  
  
- CAGGCCATGC TTTCGTGGGA GATTTACCTC TTACTTGACA TACATAAGAA GGGTACCTGG ACACTAAAAA   
  
  
- GGCATCCTTT CCGTTAAAGG TAAGTCTTAT GTTCTTCTAT GTACAGAAAA CTTTGAGAAT AATCGACAGA   
  
  
- TGGATAGTTG GTTCTTACTG CGAAGAGTAG CATACAGTGA TATTTTAAAC CAACTACTGT GGTTGTGAAA   
  
  
- GGGTAACCAT AAGTACCGAA AAATCGACGT GCTAGGTTG
