# Supplementary material for: Structure and expression analysis of seven salt-related ERF genes of Populus
Source: PeerJ. 2020 Oct 20;8:e10206. doi: 10.7717/peerj.10206 (PMC7583627; doi:10.7717/peerj.10206)
Supplement: Supplemental Information 17 [file peerj-08-10206-s017.gz › Potri.005G195000.1_plantcare.html]

Content-Type: text/html; charset=ISO-8859-1


PlantCARE


Webmaster Firefox specific output  
To save the result:
click on the frame with the right mouse button and save the source code as a text file with extension .html  
REFERENCE:PlantCARE: a database of plant cis-acting regulatory elements and a portal to tools for in silico analysis of promoter sequences.  
Lescot, M., Déhais, P., Moreau, Y., De Moor, B., Rouzé ,P.,and Rombauts, S.  
Nucleic Acids Res., Database issue(2002), 30(1):325-327.   


---

>Potri.005G195000.1   
+ TCAACACTTT AAAATGATAT GAAAATACTA AAAATATATT AATTTAAATA AAAAAATTAA TATTTTTAAA   
  
  
+ ACATAACAGA TCTATTTGTA TTGTTCACAG GAAAAATGTA ATAATGTTTT TGTCTTTCTG AAAAAAAAAT   
  
  
+ ATTAAATTGG TTATTGGAGG CATGGAAGTT TTTTCATAAT GGGCACTAGT TGTTAACAAA TTAAATAGGG   
  
  
+ GCAAATAAGT CATTTCAAAA ATAGAGTGAG ATGACCTAAA TATCCTTATA AGAAAAGAAA TTAAAAGTGG   
  
  
+ TGTCCAAGAG CTTTATTGTA TTTTTCACAT GATTTTTTTA TTACTATAGG TCAACTAAGG GCCAATTTAG   
  
  
+ TATTTGTATA AATATATGGA AAAAAAAAGC ACAGTGAATG ATCAAAATAT TATTGGAAGC AAAAAAAAAT   
  
  
+ TAAAGTTAGC CTCGAGGCGA TGTTTTTGTA TTTTTACAAT GATTTTTTTT ATCACCAAGT GAACTTAGAG   
  
  
+ GGAATTCGGT ATTTTTCATA AATATAGAAA AATAAAAGTG GTTTGCGGGT GCATAGCAAG AGCCAACATG   
  
  
+ TGTGAGGTGT TTGGGCCTTC ATGTAACGCT TGCGACACCT CCCAACGTTC AAAATACCCT TTCTATCGTG   
  
  
+ TAATTGGAAG CGAAGCGATG TCCTTTTCCT TGCGGGGTGT TGCATGTGGT TGTTGGTGAT ACAGTTTGTT   
  
  
+ GCCGACAAAG CTTTCCCTCT ATATTTTCTC TCTCCTCCCT ACAAAATTTA CTCTGGCCAT GCAAAATTTT   
  
  
+ AGAATTGTCC TTTGATTTAT GGGGATTTCC ACTTAAGTCC TTATTTTTTA TTTCTAATTT TTATTTTTGG   
  
  
+ TCTTTTTGTA AAACTTTAAT TTTTTTTAAT TTCATCTTTA AATCTCAATT TCTAATATTT TTTATTTCAA   
  
  
+ CCTTAATCCT TATTCTTTTA ATTGTTTTTT TAATTTTTTT TGAGCCATTT GTTAAATTGA TATTTCTTAT   
  
  
+ CAATTTCACT CTTTAATAAA AAAATTCGTT TGCATTTCTT ATTTCAACCT TGATCCTCAT TCTTTTAGTT   
  
  
+ TTTTTAGTTT TTATCCTTTT GTTAAATTGT TTTTGCTTTT CAATTTCATT CTTATAGAAT TTATTTTATA   
  
  
+ TTTCAATTTT CATTCTTTTT TTTAATTGCT ATTTTTTAAT CATTTTGTAT AATTGAATTT TTTTTTTCAA   
  
  
+ TTTCATCCTT CAATATTTAA TTGATTTGGA ATTAAGTTTC AAGGTTTTTC CAGATAGGAT GTTTCTAGTC   
  
  
+ TAATAACCTG GATCACAAGT TTAAAAAGTT AACATGGGTT GATATATTTT TTTTATAAAA AAATAGACTT   
  
  
+ TGTGATTTTC TTTGTTTTAT TTTCTATCTA ATTATCTTGA TGTTATAACA TAGGCCGCAT GTTTGGCGGG   
  
  
+ ATAACTTGAG TTAGCTTAAC CCTGATTACT AGGATTATAG GTTTGTCATA TTAACTTGAG TTGACTAAAC   
  
  
+ CTGATTTTTT TATAATTTTG TTTATCTAAT TTTATTCTTT CATATTTAAT GGACTTAGAA TTAAGTTTTG   
  
  
+ TTCTTTATCA TCTTTTAAAA AAAATGTTTT TTTACGTTAT CATGATTTTT TTTTTAATTT ATATCATTTG   
  
  
+ TTATTGTTAT CTATTTATGA TCTATTTAAA ATAAAATTAA CTTCTTTAAA TTAGTTAAAT CTATTATTTG   
  
  
+ AATCACGAAA AAAAATAGTC ACCGCATGAC GAAGGTCCAA AACTAGCCAT GAGGTATTCC TCGATTAATA   
  
  
+ TCAAAGCCCA ATTGTTTAAG CAGTAAGTCG GTTATCGGAA GTCAATTTGG ACACGCTCTG TCTCTCTTAT   
  
  
+ CTAATGTAGA CATGACGGTG TTCTAAGCTT GTAAGGCCTT GTCCTTGGCT CCATGGAACC TTCAATTTAT   
  
  
+ CAGTCAAATC TACAGTGTGA ACGTATATTA TTCGATTCTG TATTTAATTT TTGGTACCAC GCCTCTTTAA   
  
  
+ AAGAAAATAA AAGGTTACAA AATTCACAAA ACGTTTTTA  

- AGTTGTGAAA TTTTACTATA CTTTTATGAT TTTTATATAA TTAAATTTAT TTTTTTAATT ATAAAAATTT   
  
  
- TGTATTGTCT AGATAAACAT AACAAGTGTC CTTTTTACAT TATTACAAAA ACAGAAAGAC TTTTTTTTTA   
  
  
- TAATTTAACC AATAACCTCC GTACCTTCAA AAAAGTATTA CCCGTGATCA ACAATTGTTT AATTTATCCC   
  
  
- CGTTTATTCA GTAAAGTTTT TATCTCACTC TACTGGATTT ATAGGAATAT TCTTTTCTTT AATTTTCACC   
  
  
- ACAGGTTCTC GAAATAACAT AAAAAGTGTA CTAAAAAAAT AATGATATCC AGTTGATTCC CGGTTAAATC   
  
  
- ATAAACATAT TTATATACCT TTTTTTTTCG TGTCACTTAC TAGTTTTATA ATAACCTTCG TTTTTTTTTA   
  
  
- ATTTCAATCG GAGCTCCGCT ACAAAAACAT AAAAATGTTA CTAAAAAAAA TAGTGGTTCA CTTGAATCTC   
  
  
- CCTTAAGCCA TAAAAAGTAT TTATATCTTT TTATTTTCAC CAAACGCCCA CGTATCGTTC TCGGTTGTAC   
  
  
- ACACTCCACA AACCCGGAAG TACATTGCGA ACGCTGTGGA GGGTTGCAAG TTTTATGGGA AAGATAGCAC   
  
  
- ATTAACCTTC GCTTCGCTAC AGGAAAAGGA ACGCCCCACA ACGTACACCA ACAACCACTA TGTCAAACAA   
  
  
- CGGCTGTTTC GAAAGGGAGA TATAAAAGAG AGAGGAGGGA TGTTTTAAAT GAGACCGGTA CGTTTTAAAA   
  
  
- TCTTAACAGG AAACTAAATA CCCCTAAAGG TGAATTCAGG AATAAAAAAT AAAGATTAAA AATAAAAACC   
  
  
- AGAAAAACAT TTTGAAATTA AAAAAAATTA AAGTAGAAAT TTAGAGTTAA AGATTATAAA AAATAAAGTT   
  
  
- GGAATTAGGA ATAAGAAAAT TAACAAAAAA ATTAAAAAAA ACTCGGTAAA CAATTTAACT ATAAAGAATA   
  
  
- GTTAAAGTGA GAAATTATTT TTTTAAGCAA ACGTAAAGAA TAAAGTTGGA ACTAGGAGTA AGAAAATCAA   
  
  
- AAAAATCAAA AATAGGAAAA CAATTTAACA AAAACGAAAA GTTAAAGTAA GAATATCTTA AATAAAATAT   
  
  
- AAAGTTAAAA GTAAGAAAAA AAATTAACGA TAAAAAATTA GTAAAACATA TTAACTTAAA AAAAAAAGTT   
  
  
- AAAGTAGGAA GTTATAAATT AACTAAACCT TAATTCAAAG TTCCAAAAAG GTCTATCCTA CAAAGATCAG   
  
  
- ATTATTGGAC CTAGTGTTCA AATTTTTCAA TTGTACCCAA CTATATAAAA AAAATATTTT TTTATCTGAA   
  
  
- ACACTAAAAG AAACAAAATA AAAGATAGAT TAATAGAACT ACAATATTGT ATCCGGCGTA CAAACCGCCC   
  
  
- TATTGAACTC AATCGAATTG GGACTAATGA TCCTAATATC CAAACAGTAT AATTGAACTC AACTGATTTG   
  
  
- GACTAAAAAA ATATTAAAAC AAATAGATTA AAATAAGAAA GTATAAATTA CCTGAATCTT AATTCAAAAC   
  
  
- AAGAAATAGT AGAAAATTTT TTTTACAAAA AAATGCAATA GTACTAAAAA AAAAATTAAA TATAGTAAAC   
  
  
- AATAACAATA GATAAATACT AGATAAATTT TATTTTAATT GAAGAAATTT AATCAATTTA GATAATAAAC   
  
  
- TTAGTGCTTT TTTTTATCAG TGGCGTACTG CTTCCAGGTT TTGATCGGTA CTCCATAAGG AGCTAATTAT   
  
  
- AGTTTCGGGT TAACAAATTC GTCATTCAGC CAATAGCCTT CAGTTAAACC TGTGCGAGAC AGAGAGAATA   
  
  
- GATTACATCT GTACTGCCAC AAGATTCGAA CATTCCGGAA CAGGAACCGA GGTACCTTGG AAGTTAAATA   
  
  
- GTCAGTTTAG ATGTCACACT TGCATATAAT AAGCTAAGAC ATAAATTAAA AACCATGGTG CGGAGAAATT   
  
  
- TTCTTTTATT TTCCAATGTT TTAAGTGTTT TGCAAAAAT

  
  
Motifs Found  

+   

| Site Name | Organism | Position | Strand | Matrix score. | sequence | function |
| --- | --- | --- | --- | --- | --- | --- |
|  | organism | 730 | + | 4 | motif\_sequence | short\_function |
|  | organism | 379 | - | 4 | motif\_sequence | short\_function |
|  | organism | 1898 | + | 4 | motif\_sequence | short\_function |
|  | organism | 648 | + | 4 | motif\_sequence | short\_function |

>Potri.005G195000.1   
+ TCAACACTTT AAAATGATAT GAAAATACTA AAAATATATT AATTTAAATA AAAAAATTAA TATTTTTAAA   
  
  
+ ACATAACAGA TCTATTTGTA TTGTTCACAG GAAAAATGTA ATAATGTTTT TGTCTTTCTG AAAAAAAAAT   
  
  
+ ATTAAATTGG TTATTGGAGG CATGGAAGTT TTTTCATAAT GGGCACTAGT TGTTAACAAA TTAAATAGGG   
  
  
+ GCAAATAAGT CATTTCAAAA ATAGAGTGAG ATGACCTAAA TATCCTTATA AGAAAAGAAA TTAAAAGTGG   
  
  
+ TGTCCAAGAG CTTTATTGTA TTTTTCACAT GATTTTTTTA TTACTATAGG TCAACTAAGG GCCAATTTAG   
  
  
+ TATTTGTATA AATATATGGA AAAAAAAAGC ACAGTGAATG ATCAAAATAT TATTGGAAGC AAAAAAAAAT   
  
  
+ TAAAGTTAGC CTCGAGGCGA TGTTTTTGTA TTTTTACAAT GATTTTTTTT ATCACCAAGT GAACTTAGAG   
  
  
+ GGAATTCGGT ATTTTTCATA AATATAGAAA AATAAAAGTG GTTTGCGGGT GCATAGCAAG AGCCAACATG   
  
  
+ TGTGAGGTGT TTGGGCCTTC ATGTAACGCT TGCGACACCT CCCAACGTTC AAAATACCCT TTCTATCGTG   
  
  
+ TAATTGGAAG CGAAGCGATG TCCTTTTCCT TGCGGGGTGT TGCATGTGGT TGTTGGTGAT ACAGTTTGTT   
  
  
+ GCCGACAAAG CTTTCCCTCT ATATTTTCTC TCTCCTCCCT ACAAAATTTA CTCTGGCCAT GCAAAATTTT   
  
  
+ AGAATTGTCC TTTGATTTAT GGGGATTTCC ACTTAAGTCC TTATTTTTTA TTTCTAATTT TTATTTTTGG   
  
  
+ TCTTTTTGTA AAACTTTAAT TTTTTTTAAT TTCATCTTTA AATCTCAATT TCTAATATTT TTTATTTCAA   
  
  
+ CCTTAATCCT TATTCTTTTA ATTGTTTTTT TAATTTTTTT TGAGCCATTT GTTAAATTGA TATTTCTTAT   
  
  
+ CAATTTCACT CTTTAATAAA AAAATTCGTT TGCATTTCTT ATTTCAACCT TGATCCTCAT TCTTTTAGTT   
  
  
+ TTTTTAGTTT TTATCCTTTT GTTAAATTGT TTTTGCTTTT CAATTTCATT CTTATAGAAT TTATTTTATA   
  
  
+ TTTCAATTTT CATTCTTTTT TTTAATTGCT ATTTTTTAAT CATTTTGTAT AATTGAATTT TTTTTTTCAA   
  
  
+ TTTCATCCTT CAATATTTAA TTGATTTGGA ATTAAGTTTC AAGGTTTTTC CAGATAGGAT GTTTCTAGTC   
  
  
+ TAATAACCTG GATCACAAGT TTAAAAAGTT AACATGGGTT GATATATTTT TTTTATAAAA AAATAGACTT   
  
  
+ TGTGATTTTC TTTGTTTTAT TTTCTATCTA ATTATCTTGA TGTTATAACA TAGGCCGCAT GTTTGGCGGG   
  
  
+ ATAACTTGAG TTAGCTTAAC CCTGATTACT AGGATTATAG GTTTGTCATA TTAACTTGAG TTGACTAAAC   
  
  
+ CTGATTTTTT TATAATTTTG TTTATCTAAT TTTATTCTTT CATATTTAAT GGACTTAGAA TTAAGTTTTG   
  
  
+ TTCTTTATCA TCTTTTAAAA AAAATGTTTT TTTACGTTAT CATGATTTTT TTTTTAATTT ATATCATTTG   
  
  
+ TTATTGTTAT CTATTTATGA TCTATTTAAA ATAAAATTAA CTTCTTTAAA TTAGTTAAAT CTATTATTTG   
  
  
+ AATCACGAAA AAAAATAGTC ACCGCATGAC GAAGGTCCAA AACTAGCCAT GAGGTATTCC TCGATTAATA   
  
  
+ TCAAAGCCCA ATTGTTTAAG CAGTAAGTCG GTTATCGGAA GTCAATTTGG ACACGCTCTG TCTCTCTTAT   
  
  
+ CTAATGTAGA CATGACGGTG TTCTAAGCTT GTAAGGCCTT GTCCTTGGCT CCATGGAACC TTCAATTTAT   
  
  
+ CAGTCAAATC TACAGTGTGA ACGTATATTA TTCGATTCTG TATTTAATTT TTGGTACCAC GCCTCTTTAA   
  
  
+ AAGAAAATAA AAGGTTACAA AATTCACAAA ACGTTTTTA  

- AGTTGTGAAA TTTTACTATA CTTTTATGAT TTTTATATAA TTAAATTTAT TTTTTTAATT ATAAAAATTT   
  
  
- TGTATTGTCT AGATAAACAT AACAAGTGTC CTTTTTACAT TATTACAAAA ACAGAAAGAC TTTTTTTTTA   
  
  
- TAATTTAACC AATAACCTCC GTACCTTCAA AAAAGTATTA CCCGTGATCA ACAATTGTTT AATTTATCCC   
  
  
- CGTTTATTCA GTAAAGTTTT TATCTCACTC TACTGGATTT ATAGGAATAT TCTTTTCTTT AATTTTCACC   
  
  
- ACAGGTTCTC GAAATAACAT AAAAAGTGTA CTAAAAAAAT AATGATATCC AGTTGATTCC CGGTTAAATC   
  
  
- ATAAACATAT TTATATACCT TTTTTTTTCG TGTCACTTAC TAGTTTTATA ATAACCTTCG TTTTTTTTTA   
  
  
- ATTTCAATCG GAGCTCCGCT ACAAAAACAT AAAAATGTTA CTAAAAAAAA TAGTGGTTCA CTTGAATCTC   
  
  
- CCTTAAGCCA TAAAAAGTAT TTATATCTTT TTATTTTCAC CAAACGCCCA CGTATCGTTC TCGGTTGTAC   
  
  
- ACACTCCACA AACCCGGAAG TACATTGCGA ACGCTGTGGA GGGTTGCAAG TTTTATGGGA AAGATAGCAC   
  
  
- ATTAACCTTC GCTTCGCTAC AGGAAAAGGA ACGCCCCACA ACGTACACCA ACAACCACTA TGTCAAACAA   
  
  
- CGGCTGTTTC GAAAGGGAGA TATAAAAGAG AGAGGAGGGA TGTTTTAAAT GAGACCGGTA CGTTTTAAAA   
  
  
- TCTTAACAGG AAACTAAATA CCCCTAAAGG TGAATTCAGG AATAAAAAAT AAAGATTAAA AATAAAAACC   
  
  
- AGAAAAACAT TTTGAAATTA AAAAAAATTA AAGTAGAAAT TTAGAGTTAA AGATTATAAA AAATAAAGTT   
  
  
- GGAATTAGGA ATAAGAAAAT TAACAAAAAA ATTAAAAAAA ACTCGGTAAA CAATTTAACT ATAAAGAATA   
  
  
- GTTAAAGTGA GAAATTATTT TTTTAAGCAA ACGTAAAGAA TAAAGTTGGA ACTAGGAGTA AGAAAATCAA   
  
  
- AAAAATCAAA AATAGGAAAA CAATTTAACA AAAACGAAAA GTTAAAGTAA GAATATCTTA AATAAAATAT   
  
  
- AAAGTTAAAA GTAAGAAAAA AAATTAACGA TAAAAAATTA GTAAAACATA TTAACTTAAA AAAAAAAGTT   
  
  
- AAAGTAGGAA GTTATAAATT AACTAAACCT TAATTCAAAG TTCCAAAAAG GTCTATCCTA CAAAGATCAG   
  
  
- ATTATTGGAC CTAGTGTTCA AATTTTTCAA TTGTACCCAA CTATATAAAA AAAATATTTT TTTATCTGAA   
  
  
- ACACTAAAAG AAACAAAATA AAAGATAGAT TAATAGAACT ACAATATTGT ATCCGGCGTA CAAACCGCCC   
  
  
- TATTGAACTC AATCGAATTG GGACTAATGA TCCTAATATC CAAACAGTAT AATTGAACTC AACTGATTTG   
  
  
- GACTAAAAAA ATATTAAAAC AAATAGATTA AAATAAGAAA GTATAAATTA CCTGAATCTT AATTCAAAAC   
  
  
- AAGAAATAGT AGAAAATTTT TTTTACAAAA AAATGCAATA GTACTAAAAA AAAAATTAAA TATAGTAAAC   
  
  
- AATAACAATA GATAAATACT AGATAAATTT TATTTTAATT GAAGAAATTT AATCAATTTA GATAATAAAC   
  
  
- TTAGTGCTTT TTTTTATCAG TGGCGTACTG CTTCCAGGTT TTGATCGGTA CTCCATAAGG AGCTAATTAT   
  
  
- AGTTTCGGGT TAACAAATTC GTCATTCAGC CAATAGCCTT CAGTTAAACC TGTGCGAGAC AGAGAGAATA   
  
  
- GATTACATCT GTACTGCCAC AAGATTCGAA CATTCCGGAA CAGGAACCGA GGTACCTTGG AAGTTAAATA   
  
  
- GTCAGTTTAG ATGTCACACT TGCATATAAT AAGCTAAGAC ATAAATTAAA AACCATGGTG CGGAGAAATT   
  
  
- TTCTTTTATT TTCCAATGTT TTAAGTGTTT TGCAAAAAT

+     AAGAA-motif

| Site Name | Organism | Position | Strand | Matrix score. | sequence | function |
| --- | --- | --- | --- | --- | --- | --- |
| AAGAA-motif | Avena sativa | 1505 | - | 7 | GAAAGAA |  |

>Potri.005G195000.1   
+ TCAACACTTT AAAATGATAT GAAAATACTA AAAATATATT AATTTAAATA AAAAAATTAA TATTTTTAAA   
  
  
+ ACATAACAGA TCTATTTGTA TTGTTCACAG GAAAAATGTA ATAATGTTTT TGTCTTTCTG AAAAAAAAAT   
  
  
+ ATTAAATTGG TTATTGGAGG CATGGAAGTT TTTTCATAAT GGGCACTAGT TGTTAACAAA TTAAATAGGG   
  
  
+ GCAAATAAGT CATTTCAAAA ATAGAGTGAG ATGACCTAAA TATCCTTATA AGAAAAGAAA TTAAAAGTGG   
  
  
+ TGTCCAAGAG CTTTATTGTA TTTTTCACAT GATTTTTTTA TTACTATAGG TCAACTAAGG GCCAATTTAG   
  
  
+ TATTTGTATA AATATATGGA AAAAAAAAGC ACAGTGAATG ATCAAAATAT TATTGGAAGC AAAAAAAAAT   
  
  
+ TAAAGTTAGC CTCGAGGCGA TGTTTTTGTA TTTTTACAAT GATTTTTTTT ATCACCAAGT GAACTTAGAG   
  
  
+ GGAATTCGGT ATTTTTCATA AATATAGAAA AATAAAAGTG GTTTGCGGGT GCATAGCAAG AGCCAACATG   
  
  
+ TGTGAGGTGT TTGGGCCTTC ATGTAACGCT TGCGACACCT CCCAACGTTC AAAATACCCT TTCTATCGTG   
  
  
+ TAATTGGAAG CGAAGCGATG TCCTTTTCCT TGCGGGGTGT TGCATGTGGT TGTTGGTGAT ACAGTTTGTT   
  
  
+ GCCGACAAAG CTTTCCCTCT ATATTTTCTC TCTCCTCCCT ACAAAATTTA CTCTGGCCAT GCAAAATTTT   
  
  
+ AGAATTGTCC TTTGATTTAT GGGGATTTCC ACTTAAGTCC TTATTTTTTA TTTCTAATTT TTATTTTTGG   
  
  
+ TCTTTTTGTA AAACTTTAAT TTTTTTTAAT TTCATCTTTA AATCTCAATT TCTAATATTT TTTATTTCAA   
  
  
+ CCTTAATCCT TATTCTTTTA ATTGTTTTTT TAATTTTTTT TGAGCCATTT GTTAAATTGA TATTTCTTAT   
  
  
+ CAATTTCACT CTTTAATAAA AAAATTCGTT TGCATTTCTT ATTTCAACCT TGATCCTCAT TCTTTTAGTT   
  
  
+ TTTTTAGTTT TTATCCTTTT GTTAAATTGT TTTTGCTTTT CAATTTCATT CTTATAGAAT TTATTTTATA   
  
  
+ TTTCAATTTT CATTCTTTTT TTTAATTGCT ATTTTTTAAT CATTTTGTAT AATTGAATTT TTTTTTTCAA   
  
  
+ TTTCATCCTT CAATATTTAA TTGATTTGGA ATTAAGTTTC AAGGTTTTTC CAGATAGGAT GTTTCTAGTC   
  
  
+ TAATAACCTG GATCACAAGT TTAAAAAGTT AACATGGGTT GATATATTTT TTTTATAAAA AAATAGACTT   
  
  
+ TGTGATTTTC TTTGTTTTAT TTTCTATCTA ATTATCTTGA TGTTATAACA TAGGCCGCAT GTTTGGCGGG   
  
  
+ ATAACTTGAG TTAGCTTAAC CCTGATTACT AGGATTATAG GTTTGTCATA TTAACTTGAG TTGACTAAAC   
  
  
+ CTGATTTTTT TATAATTTTG TTTATCTAAT TTTATTCTTT CATATTTAAT GGACTTAGAA TTAAGTTTTG   
  
  
+ TTCTTTATCA TCTTTTAAAA AAAATGTTTT TTTACGTTAT CATGATTTTT TTTTTAATTT ATATCATTTG   
  
  
+ TTATTGTTAT CTATTTATGA TCTATTTAAA ATAAAATTAA CTTCTTTAAA TTAGTTAAAT CTATTATTTG   
  
  
+ AATCACGAAA AAAAATAGTC ACCGCATGAC GAAGGTCCAA AACTAGCCAT GAGGTATTCC TCGATTAATA   
  
  
+ TCAAAGCCCA ATTGTTTAAG CAGTAAGTCG GTTATCGGAA GTCAATTTGG ACACGCTCTG TCTCTCTTAT   
  
  
+ CTAATGTAGA CATGACGGTG TTCTAAGCTT GTAAGGCCTT GTCCTTGGCT CCATGGAACC TTCAATTTAT   
  
  
+ CAGTCAAATC TACAGTGTGA ACGTATATTA TTCGATTCTG TATTTAATTT TTGGTACCAC GCCTCTTTAA   
  
  
+ AAGAAAATAA AAGGTTACAA AATTCACAAA ACGTTTTTA  

- AGTTGTGAAA TTTTACTATA CTTTTATGAT TTTTATATAA TTAAATTTAT TTTTTTAATT ATAAAAATTT   
  
  
- TGTATTGTCT AGATAAACAT AACAAGTGTC CTTTTTACAT TATTACAAAA ACAGAAAGAC TTTTTTTTTA   
  
  
- TAATTTAACC AATAACCTCC GTACCTTCAA AAAAGTATTA CCCGTGATCA ACAATTGTTT AATTTATCCC   
  
  
- CGTTTATTCA GTAAAGTTTT TATCTCACTC TACTGGATTT ATAGGAATAT TCTTTTCTTT AATTTTCACC   
  
  
- ACAGGTTCTC GAAATAACAT AAAAAGTGTA CTAAAAAAAT AATGATATCC AGTTGATTCC CGGTTAAATC   
  
  
- ATAAACATAT TTATATACCT TTTTTTTTCG TGTCACTTAC TAGTTTTATA ATAACCTTCG TTTTTTTTTA   
  
  
- ATTTCAATCG GAGCTCCGCT ACAAAAACAT AAAAATGTTA CTAAAAAAAA TAGTGGTTCA CTTGAATCTC   
  
  
- CCTTAAGCCA TAAAAAGTAT TTATATCTTT TTATTTTCAC CAAACGCCCA CGTATCGTTC TCGGTTGTAC   
  
  
- ACACTCCACA AACCCGGAAG TACATTGCGA ACGCTGTGGA GGGTTGCAAG TTTTATGGGA AAGATAGCAC   
  
  
- ATTAACCTTC GCTTCGCTAC AGGAAAAGGA ACGCCCCACA ACGTACACCA ACAACCACTA TGTCAAACAA   
  
  
- CGGCTGTTTC GAAAGGGAGA TATAAAAGAG AGAGGAGGGA TGTTTTAAAT GAGACCGGTA CGTTTTAAAA   
  
  
- TCTTAACAGG AAACTAAATA CCCCTAAAGG TGAATTCAGG AATAAAAAAT AAAGATTAAA AATAAAAACC   
  
  
- AGAAAAACAT TTTGAAATTA AAAAAAATTA AAGTAGAAAT TTAGAGTTAA AGATTATAAA AAATAAAGTT   
  
  
- GGAATTAGGA ATAAGAAAAT TAACAAAAAA ATTAAAAAAA ACTCGGTAAA CAATTTAACT ATAAAGAATA   
  
  
- GTTAAAGTGA GAAATTATTT TTTTAAGCAA ACGTAAAGAA TAAAGTTGGA ACTAGGAGTA AGAAAATCAA   
  
  
- AAAAATCAAA AATAGGAAAA CAATTTAACA AAAACGAAAA GTTAAAGTAA GAATATCTTA AATAAAATAT   
  
  
- AAAGTTAAAA GTAAGAAAAA AAATTAACGA TAAAAAATTA GTAAAACATA TTAACTTAAA AAAAAAAGTT   
  
  
- AAAGTAGGAA GTTATAAATT AACTAAACCT TAATTCAAAG TTCCAAAAAG GTCTATCCTA CAAAGATCAG   
  
  
- ATTATTGGAC CTAGTGTTCA AATTTTTCAA TTGTACCCAA CTATATAAAA AAAATATTTT TTTATCTGAA   
  
  
- ACACTAAAAG AAACAAAATA AAAGATAGAT TAATAGAACT ACAATATTGT ATCCGGCGTA CAAACCGCCC   
  
  
- TATTGAACTC AATCGAATTG GGACTAATGA TCCTAATATC CAAACAGTAT AATTGAACTC AACTGATTTG   
  
  
- GACTAAAAAA ATATTAAAAC AAATAGATTA AAATAAGAAA GTATAAATTA CCTGAATCTT AATTCAAAAC   
  
  
- AAGAAATAGT AGAAAATTTT TTTTACAAAA AAATGCAATA GTACTAAAAA AAAAATTAAA TATAGTAAAC   
  
  
- AATAACAATA GATAAATACT AGATAAATTT TATTTTAATT GAAGAAATTT AATCAATTTA GATAATAAAC   
  
  
- TTAGTGCTTT TTTTTATCAG TGGCGTACTG CTTCCAGGTT TTGATCGGTA CTCCATAAGG AGCTAATTAT   
  
  
- AGTTTCGGGT TAACAAATTC GTCATTCAGC CAATAGCCTT CAGTTAAACC TGTGCGAGAC AGAGAGAATA   
  
  
- GATTACATCT GTACTGCCAC AAGATTCGAA CATTCCGGAA CAGGAACCGA GGTACCTTGG AAGTTAAATA   
  
  
- GTCAGTTTAG ATGTCACACT TGCATATAAT AAGCTAAGAC ATAAATTAAA AACCATGGTG CGGAGAAATT   
  
  
- TTCTTTTATT TTCCAATGTT TTAAGTGTTT TGCAAAAAT

+     AP-1

| Site Name | Organism | Position | Strand | Matrix score. | sequence | function |
| --- | --- | --- | --- | --- | --- | --- |
| AP-1 | Arabidopsis thaliana | 1407 | + | 8 | TGAGTTAG |  |

>Potri.005G195000.1   
+ TCAACACTTT AAAATGATAT GAAAATACTA AAAATATATT AATTTAAATA AAAAAATTAA TATTTTTAAA   
  
  
+ ACATAACAGA TCTATTTGTA TTGTTCACAG GAAAAATGTA ATAATGTTTT TGTCTTTCTG AAAAAAAAAT   
  
  
+ ATTAAATTGG TTATTGGAGG CATGGAAGTT TTTTCATAAT GGGCACTAGT TGTTAACAAA TTAAATAGGG   
  
  
+ GCAAATAAGT CATTTCAAAA ATAGAGTGAG ATGACCTAAA TATCCTTATA AGAAAAGAAA TTAAAAGTGG   
  
  
+ TGTCCAAGAG CTTTATTGTA TTTTTCACAT GATTTTTTTA TTACTATAGG TCAACTAAGG GCCAATTTAG   
  
  
+ TATTTGTATA AATATATGGA AAAAAAAAGC ACAGTGAATG ATCAAAATAT TATTGGAAGC AAAAAAAAAT   
  
  
+ TAAAGTTAGC CTCGAGGCGA TGTTTTTGTA TTTTTACAAT GATTTTTTTT ATCACCAAGT GAACTTAGAG   
  
  
+ GGAATTCGGT ATTTTTCATA AATATAGAAA AATAAAAGTG GTTTGCGGGT GCATAGCAAG AGCCAACATG   
  
  
+ TGTGAGGTGT TTGGGCCTTC ATGTAACGCT TGCGACACCT CCCAACGTTC AAAATACCCT TTCTATCGTG   
  
  
+ TAATTGGAAG CGAAGCGATG TCCTTTTCCT TGCGGGGTGT TGCATGTGGT TGTTGGTGAT ACAGTTTGTT   
  
  
+ GCCGACAAAG CTTTCCCTCT ATATTTTCTC TCTCCTCCCT ACAAAATTTA CTCTGGCCAT GCAAAATTTT   
  
  
+ AGAATTGTCC TTTGATTTAT GGGGATTTCC ACTTAAGTCC TTATTTTTTA TTTCTAATTT TTATTTTTGG   
  
  
+ TCTTTTTGTA AAACTTTAAT TTTTTTTAAT TTCATCTTTA AATCTCAATT TCTAATATTT TTTATTTCAA   
  
  
+ CCTTAATCCT TATTCTTTTA ATTGTTTTTT TAATTTTTTT TGAGCCATTT GTTAAATTGA TATTTCTTAT   
  
  
+ CAATTTCACT CTTTAATAAA AAAATTCGTT TGCATTTCTT ATTTCAACCT TGATCCTCAT TCTTTTAGTT   
  
  
+ TTTTTAGTTT TTATCCTTTT GTTAAATTGT TTTTGCTTTT CAATTTCATT CTTATAGAAT TTATTTTATA   
  
  
+ TTTCAATTTT CATTCTTTTT TTTAATTGCT ATTTTTTAAT CATTTTGTAT AATTGAATTT TTTTTTTCAA   
  
  
+ TTTCATCCTT CAATATTTAA TTGATTTGGA ATTAAGTTTC AAGGTTTTTC CAGATAGGAT GTTTCTAGTC   
  
  
+ TAATAACCTG GATCACAAGT TTAAAAAGTT AACATGGGTT GATATATTTT TTTTATAAAA AAATAGACTT   
  
  
+ TGTGATTTTC TTTGTTTTAT TTTCTATCTA ATTATCTTGA TGTTATAACA TAGGCCGCAT GTTTGGCGGG   
  
  
+ ATAACTTGAG TTAGCTTAAC CCTGATTACT AGGATTATAG GTTTGTCATA TTAACTTGAG TTGACTAAAC   
  
  
+ CTGATTTTTT TATAATTTTG TTTATCTAAT TTTATTCTTT CATATTTAAT GGACTTAGAA TTAAGTTTTG   
  
  
+ TTCTTTATCA TCTTTTAAAA AAAATGTTTT TTTACGTTAT CATGATTTTT TTTTTAATTT ATATCATTTG   
  
  
+ TTATTGTTAT CTATTTATGA TCTATTTAAA ATAAAATTAA CTTCTTTAAA TTAGTTAAAT CTATTATTTG   
  
  
+ AATCACGAAA AAAAATAGTC ACCGCATGAC GAAGGTCCAA AACTAGCCAT GAGGTATTCC TCGATTAATA   
  
  
+ TCAAAGCCCA ATTGTTTAAG CAGTAAGTCG GTTATCGGAA GTCAATTTGG ACACGCTCTG TCTCTCTTAT   
  
  
+ CTAATGTAGA CATGACGGTG TTCTAAGCTT GTAAGGCCTT GTCCTTGGCT CCATGGAACC TTCAATTTAT   
  
  
+ CAGTCAAATC TACAGTGTGA ACGTATATTA TTCGATTCTG TATTTAATTT TTGGTACCAC GCCTCTTTAA   
  
  
+ AAGAAAATAA AAGGTTACAA AATTCACAAA ACGTTTTTA  

- AGTTGTGAAA TTTTACTATA CTTTTATGAT TTTTATATAA TTAAATTTAT TTTTTTAATT ATAAAAATTT   
  
  
- TGTATTGTCT AGATAAACAT AACAAGTGTC CTTTTTACAT TATTACAAAA ACAGAAAGAC TTTTTTTTTA   
  
  
- TAATTTAACC AATAACCTCC GTACCTTCAA AAAAGTATTA CCCGTGATCA ACAATTGTTT AATTTATCCC   
  
  
- CGTTTATTCA GTAAAGTTTT TATCTCACTC TACTGGATTT ATAGGAATAT TCTTTTCTTT AATTTTCACC   
  
  
- ACAGGTTCTC GAAATAACAT AAAAAGTGTA CTAAAAAAAT AATGATATCC AGTTGATTCC CGGTTAAATC   
  
  
- ATAAACATAT TTATATACCT TTTTTTTTCG TGTCACTTAC TAGTTTTATA ATAACCTTCG TTTTTTTTTA   
  
  
- ATTTCAATCG GAGCTCCGCT ACAAAAACAT AAAAATGTTA CTAAAAAAAA TAGTGGTTCA CTTGAATCTC   
  
  
- CCTTAAGCCA TAAAAAGTAT TTATATCTTT TTATTTTCAC CAAACGCCCA CGTATCGTTC TCGGTTGTAC   
  
  
- ACACTCCACA AACCCGGAAG TACATTGCGA ACGCTGTGGA GGGTTGCAAG TTTTATGGGA AAGATAGCAC   
  
  
- ATTAACCTTC GCTTCGCTAC AGGAAAAGGA ACGCCCCACA ACGTACACCA ACAACCACTA TGTCAAACAA   
  
  
- CGGCTGTTTC GAAAGGGAGA TATAAAAGAG AGAGGAGGGA TGTTTTAAAT GAGACCGGTA CGTTTTAAAA   
  
  
- TCTTAACAGG AAACTAAATA CCCCTAAAGG TGAATTCAGG AATAAAAAAT AAAGATTAAA AATAAAAACC   
  
  
- AGAAAAACAT TTTGAAATTA AAAAAAATTA AAGTAGAAAT TTAGAGTTAA AGATTATAAA AAATAAAGTT   
  
  
- GGAATTAGGA ATAAGAAAAT TAACAAAAAA ATTAAAAAAA ACTCGGTAAA CAATTTAACT ATAAAGAATA   
  
  
- GTTAAAGTGA GAAATTATTT TTTTAAGCAA ACGTAAAGAA TAAAGTTGGA ACTAGGAGTA AGAAAATCAA   
  
  
- AAAAATCAAA AATAGGAAAA CAATTTAACA AAAACGAAAA GTTAAAGTAA GAATATCTTA AATAAAATAT   
  
  
- AAAGTTAAAA GTAAGAAAAA AAATTAACGA TAAAAAATTA GTAAAACATA TTAACTTAAA AAAAAAAGTT   
  
  
- AAAGTAGGAA GTTATAAATT AACTAAACCT TAATTCAAAG TTCCAAAAAG GTCTATCCTA CAAAGATCAG   
  
  
- ATTATTGGAC CTAGTGTTCA AATTTTTCAA TTGTACCCAA CTATATAAAA AAAATATTTT TTTATCTGAA   
  
  
- ACACTAAAAG AAACAAAATA AAAGATAGAT TAATAGAACT ACAATATTGT ATCCGGCGTA CAAACCGCCC   
  
  
- TATTGAACTC AATCGAATTG GGACTAATGA TCCTAATATC CAAACAGTAT AATTGAACTC AACTGATTTG   
  
  
- GACTAAAAAA ATATTAAAAC AAATAGATTA AAATAAGAAA GTATAAATTA CCTGAATCTT AATTCAAAAC   
  
  
- AAGAAATAGT AGAAAATTTT TTTTACAAAA AAATGCAATA GTACTAAAAA AAAAATTAAA TATAGTAAAC   
  
  
- AATAACAATA GATAAATACT AGATAAATTT TATTTTAATT GAAGAAATTT AATCAATTTA GATAATAAAC   
  
  
- TTAGTGCTTT TTTTTATCAG TGGCGTACTG CTTCCAGGTT TTGATCGGTA CTCCATAAGG AGCTAATTAT   
  
  
- AGTTTCGGGT TAACAAATTC GTCATTCAGC CAATAGCCTT CAGTTAAACC TGTGCGAGAC AGAGAGAATA   
  
  
- GATTACATCT GTACTGCCAC AAGATTCGAA CATTCCGGAA CAGGAACCGA GGTACCTTGG AAGTTAAATA   
  
  
- GTCAGTTTAG ATGTCACACT TGCATATAAT AAGCTAAGAC ATAAATTAAA AACCATGGTG CGGAGAAATT   
  
  
- TTCTTTTATT TTCCAATGTT TTAAGTGTTT TGCAAAAAT

+     ARE

| Site Name | Organism | Position | Strand | Matrix score. | sequence | function |
| --- | --- | --- | --- | --- | --- | --- |
| ARE | Zea mays | 529 | - | 6 | AAACCA | cis-acting regulatory element essential for the anaerobic induction |

>Potri.005G195000.1   
+ TCAACACTTT AAAATGATAT GAAAATACTA AAAATATATT AATTTAAATA AAAAAATTAA TATTTTTAAA   
  
  
+ ACATAACAGA TCTATTTGTA TTGTTCACAG GAAAAATGTA ATAATGTTTT TGTCTTTCTG AAAAAAAAAT   
  
  
+ ATTAAATTGG TTATTGGAGG CATGGAAGTT TTTTCATAAT GGGCACTAGT TGTTAACAAA TTAAATAGGG   
  
  
+ GCAAATAAGT CATTTCAAAA ATAGAGTGAG ATGACCTAAA TATCCTTATA AGAAAAGAAA TTAAAAGTGG   
  
  
+ TGTCCAAGAG CTTTATTGTA TTTTTCACAT GATTTTTTTA TTACTATAGG TCAACTAAGG GCCAATTTAG   
  
  
+ TATTTGTATA AATATATGGA AAAAAAAAGC ACAGTGAATG ATCAAAATAT TATTGGAAGC AAAAAAAAAT   
  
  
+ TAAAGTTAGC CTCGAGGCGA TGTTTTTGTA TTTTTACAAT GATTTTTTTT ATCACCAAGT GAACTTAGAG   
  
  
+ GGAATTCGGT ATTTTTCATA AATATAGAAA AATAAAAGTG GTTTGCGGGT GCATAGCAAG AGCCAACATG   
  
  
+ TGTGAGGTGT TTGGGCCTTC ATGTAACGCT TGCGACACCT CCCAACGTTC AAAATACCCT TTCTATCGTG   
  
  
+ TAATTGGAAG CGAAGCGATG TCCTTTTCCT TGCGGGGTGT TGCATGTGGT TGTTGGTGAT ACAGTTTGTT   
  
  
+ GCCGACAAAG CTTTCCCTCT ATATTTTCTC TCTCCTCCCT ACAAAATTTA CTCTGGCCAT GCAAAATTTT   
  
  
+ AGAATTGTCC TTTGATTTAT GGGGATTTCC ACTTAAGTCC TTATTTTTTA TTTCTAATTT TTATTTTTGG   
  
  
+ TCTTTTTGTA AAACTTTAAT TTTTTTTAAT TTCATCTTTA AATCTCAATT TCTAATATTT TTTATTTCAA   
  
  
+ CCTTAATCCT TATTCTTTTA ATTGTTTTTT TAATTTTTTT TGAGCCATTT GTTAAATTGA TATTTCTTAT   
  
  
+ CAATTTCACT CTTTAATAAA AAAATTCGTT TGCATTTCTT ATTTCAACCT TGATCCTCAT TCTTTTAGTT   
  
  
+ TTTTTAGTTT TTATCCTTTT GTTAAATTGT TTTTGCTTTT CAATTTCATT CTTATAGAAT TTATTTTATA   
  
  
+ TTTCAATTTT CATTCTTTTT TTTAATTGCT ATTTTTTAAT CATTTTGTAT AATTGAATTT TTTTTTTCAA   
  
  
+ TTTCATCCTT CAATATTTAA TTGATTTGGA ATTAAGTTTC AAGGTTTTTC CAGATAGGAT GTTTCTAGTC   
  
  
+ TAATAACCTG GATCACAAGT TTAAAAAGTT AACATGGGTT GATATATTTT TTTTATAAAA AAATAGACTT   
  
  
+ TGTGATTTTC TTTGTTTTAT TTTCTATCTA ATTATCTTGA TGTTATAACA TAGGCCGCAT GTTTGGCGGG   
  
  
+ ATAACTTGAG TTAGCTTAAC CCTGATTACT AGGATTATAG GTTTGTCATA TTAACTTGAG TTGACTAAAC   
  
  
+ CTGATTTTTT TATAATTTTG TTTATCTAAT TTTATTCTTT CATATTTAAT GGACTTAGAA TTAAGTTTTG   
  
  
+ TTCTTTATCA TCTTTTAAAA AAAATGTTTT TTTACGTTAT CATGATTTTT TTTTTAATTT ATATCATTTG   
  
  
+ TTATTGTTAT CTATTTATGA TCTATTTAAA ATAAAATTAA CTTCTTTAAA TTAGTTAAAT CTATTATTTG   
  
  
+ AATCACGAAA AAAAATAGTC ACCGCATGAC GAAGGTCCAA AACTAGCCAT GAGGTATTCC TCGATTAATA   
  
  
+ TCAAAGCCCA ATTGTTTAAG CAGTAAGTCG GTTATCGGAA GTCAATTTGG ACACGCTCTG TCTCTCTTAT   
  
  
+ CTAATGTAGA CATGACGGTG TTCTAAGCTT GTAAGGCCTT GTCCTTGGCT CCATGGAACC TTCAATTTAT   
  
  
+ CAGTCAAATC TACAGTGTGA ACGTATATTA TTCGATTCTG TATTTAATTT TTGGTACCAC GCCTCTTTAA   
  
  
+ AAGAAAATAA AAGGTTACAA AATTCACAAA ACGTTTTTA  

- AGTTGTGAAA TTTTACTATA CTTTTATGAT TTTTATATAA TTAAATTTAT TTTTTTAATT ATAAAAATTT   
  
  
- TGTATTGTCT AGATAAACAT AACAAGTGTC CTTTTTACAT TATTACAAAA ACAGAAAGAC TTTTTTTTTA   
  
  
- TAATTTAACC AATAACCTCC GTACCTTCAA AAAAGTATTA CCCGTGATCA ACAATTGTTT AATTTATCCC   
  
  
- CGTTTATTCA GTAAAGTTTT TATCTCACTC TACTGGATTT ATAGGAATAT TCTTTTCTTT AATTTTCACC   
  
  
- ACAGGTTCTC GAAATAACAT AAAAAGTGTA CTAAAAAAAT AATGATATCC AGTTGATTCC CGGTTAAATC   
  
  
- ATAAACATAT TTATATACCT TTTTTTTTCG TGTCACTTAC TAGTTTTATA ATAACCTTCG TTTTTTTTTA   
  
  
- ATTTCAATCG GAGCTCCGCT ACAAAAACAT AAAAATGTTA CTAAAAAAAA TAGTGGTTCA CTTGAATCTC   
  
  
- CCTTAAGCCA TAAAAAGTAT TTATATCTTT TTATTTTCAC CAAACGCCCA CGTATCGTTC TCGGTTGTAC   
  
  
- ACACTCCACA AACCCGGAAG TACATTGCGA ACGCTGTGGA GGGTTGCAAG TTTTATGGGA AAGATAGCAC   
  
  
- ATTAACCTTC GCTTCGCTAC AGGAAAAGGA ACGCCCCACA ACGTACACCA ACAACCACTA TGTCAAACAA   
  
  
- CGGCTGTTTC GAAAGGGAGA TATAAAAGAG AGAGGAGGGA TGTTTTAAAT GAGACCGGTA CGTTTTAAAA   
  
  
- TCTTAACAGG AAACTAAATA CCCCTAAAGG TGAATTCAGG AATAAAAAAT AAAGATTAAA AATAAAAACC   
  
  
- AGAAAAACAT TTTGAAATTA AAAAAAATTA AAGTAGAAAT TTAGAGTTAA AGATTATAAA AAATAAAGTT   
  
  
- GGAATTAGGA ATAAGAAAAT TAACAAAAAA ATTAAAAAAA ACTCGGTAAA CAATTTAACT ATAAAGAATA   
  
  
- GTTAAAGTGA GAAATTATTT TTTTAAGCAA ACGTAAAGAA TAAAGTTGGA ACTAGGAGTA AGAAAATCAA   
  
  
- AAAAATCAAA AATAGGAAAA CAATTTAACA AAAACGAAAA GTTAAAGTAA GAATATCTTA AATAAAATAT   
  
  
- AAAGTTAAAA GTAAGAAAAA AAATTAACGA TAAAAAATTA GTAAAACATA TTAACTTAAA AAAAAAAGTT   
  
  
- AAAGTAGGAA GTTATAAATT AACTAAACCT TAATTCAAAG TTCCAAAAAG GTCTATCCTA CAAAGATCAG   
  
  
- ATTATTGGAC CTAGTGTTCA AATTTTTCAA TTGTACCCAA CTATATAAAA AAAATATTTT TTTATCTGAA   
  
  
- ACACTAAAAG AAACAAAATA AAAGATAGAT TAATAGAACT ACAATATTGT ATCCGGCGTA CAAACCGCCC   
  
  
- TATTGAACTC AATCGAATTG GGACTAATGA TCCTAATATC CAAACAGTAT AATTGAACTC AACTGATTTG   
  
  
- GACTAAAAAA ATATTAAAAC AAATAGATTA AAATAAGAAA GTATAAATTA CCTGAATCTT AATTCAAAAC   
  
  
- AAGAAATAGT AGAAAATTTT TTTTACAAAA AAATGCAATA GTACTAAAAA AAAAATTAAA TATAGTAAAC   
  
  
- AATAACAATA GATAAATACT AGATAAATTT TATTTTAATT GAAGAAATTT AATCAATTTA GATAATAAAC   
  
  
- TTAGTGCTTT TTTTTATCAG TGGCGTACTG CTTCCAGGTT TTGATCGGTA CTCCATAAGG AGCTAATTAT   
  
  
- AGTTTCGGGT TAACAAATTC GTCATTCAGC CAATAGCCTT CAGTTAAACC TGTGCGAGAC AGAGAGAATA   
  
  
- GATTACATCT GTACTGCCAC AAGATTCGAA CATTCCGGAA CAGGAACCGA GGTACCTTGG AAGTTAAATA   
  
  
- GTCAGTTTAG ATGTCACACT TGCATATAAT AAGCTAAGAC ATAAATTAAA AACCATGGTG CGGAGAAATT   
  
  
- TTCTTTTATT TTCCAATGTT TTAAGTGTTT TGCAAAAAT

+     Box 4

| Site Name | Organism | Position | Strand | Matrix score. | sequence | function |
| --- | --- | --- | --- | --- | --- | --- |
| Box 4 | Petroselinum crispum | 1744 | - | 6 | ATTAAT | part of a conserved DNA module involved in light responsiveness |
| Box 4 | Petroselinum crispum | 38 | + | 6 | ATTAAT | part of a conserved DNA module involved in light responsiveness |
| Box 4 | Petroselinum crispum | 56 | + | 6 | ATTAAT | part of a conserved DNA module involved in light responsiveness |

>Potri.005G195000.1   
+ TCAACACTTT AAAATGATAT GAAAATACTA AAAATATATT AATTTAAATA AAAAAATTAA TATTTTTAAA   
  
  
+ ACATAACAGA TCTATTTGTA TTGTTCACAG GAAAAATGTA ATAATGTTTT TGTCTTTCTG AAAAAAAAAT   
  
  
+ ATTAAATTGG TTATTGGAGG CATGGAAGTT TTTTCATAAT GGGCACTAGT TGTTAACAAA TTAAATAGGG   
  
  
+ GCAAATAAGT CATTTCAAAA ATAGAGTGAG ATGACCTAAA TATCCTTATA AGAAAAGAAA TTAAAAGTGG   
  
  
+ TGTCCAAGAG CTTTATTGTA TTTTTCACAT GATTTTTTTA TTACTATAGG TCAACTAAGG GCCAATTTAG   
  
  
+ TATTTGTATA AATATATGGA AAAAAAAAGC ACAGTGAATG ATCAAAATAT TATTGGAAGC AAAAAAAAAT   
  
  
+ TAAAGTTAGC CTCGAGGCGA TGTTTTTGTA TTTTTACAAT GATTTTTTTT ATCACCAAGT GAACTTAGAG   
  
  
+ GGAATTCGGT ATTTTTCATA AATATAGAAA AATAAAAGTG GTTTGCGGGT GCATAGCAAG AGCCAACATG   
  
  
+ TGTGAGGTGT TTGGGCCTTC ATGTAACGCT TGCGACACCT CCCAACGTTC AAAATACCCT TTCTATCGTG   
  
  
+ TAATTGGAAG CGAAGCGATG TCCTTTTCCT TGCGGGGTGT TGCATGTGGT TGTTGGTGAT ACAGTTTGTT   
  
  
+ GCCGACAAAG CTTTCCCTCT ATATTTTCTC TCTCCTCCCT ACAAAATTTA CTCTGGCCAT GCAAAATTTT   
  
  
+ AGAATTGTCC TTTGATTTAT GGGGATTTCC ACTTAAGTCC TTATTTTTTA TTTCTAATTT TTATTTTTGG   
  
  
+ TCTTTTTGTA AAACTTTAAT TTTTTTTAAT TTCATCTTTA AATCTCAATT TCTAATATTT TTTATTTCAA   
  
  
+ CCTTAATCCT TATTCTTTTA ATTGTTTTTT TAATTTTTTT TGAGCCATTT GTTAAATTGA TATTTCTTAT   
  
  
+ CAATTTCACT CTTTAATAAA AAAATTCGTT TGCATTTCTT ATTTCAACCT TGATCCTCAT TCTTTTAGTT   
  
  
+ TTTTTAGTTT TTATCCTTTT GTTAAATTGT TTTTGCTTTT CAATTTCATT CTTATAGAAT TTATTTTATA   
  
  
+ TTTCAATTTT CATTCTTTTT TTTAATTGCT ATTTTTTAAT CATTTTGTAT AATTGAATTT TTTTTTTCAA   
  
  
+ TTTCATCCTT CAATATTTAA TTGATTTGGA ATTAAGTTTC AAGGTTTTTC CAGATAGGAT GTTTCTAGTC   
  
  
+ TAATAACCTG GATCACAAGT TTAAAAAGTT AACATGGGTT GATATATTTT TTTTATAAAA AAATAGACTT   
  
  
+ TGTGATTTTC TTTGTTTTAT TTTCTATCTA ATTATCTTGA TGTTATAACA TAGGCCGCAT GTTTGGCGGG   
  
  
+ ATAACTTGAG TTAGCTTAAC CCTGATTACT AGGATTATAG GTTTGTCATA TTAACTTGAG TTGACTAAAC   
  
  
+ CTGATTTTTT TATAATTTTG TTTATCTAAT TTTATTCTTT CATATTTAAT GGACTTAGAA TTAAGTTTTG   
  
  
+ TTCTTTATCA TCTTTTAAAA AAAATGTTTT TTTACGTTAT CATGATTTTT TTTTTAATTT ATATCATTTG   
  
  
+ TTATTGTTAT CTATTTATGA TCTATTTAAA ATAAAATTAA CTTCTTTAAA TTAGTTAAAT CTATTATTTG   
  
  
+ AATCACGAAA AAAAATAGTC ACCGCATGAC GAAGGTCCAA AACTAGCCAT GAGGTATTCC TCGATTAATA   
  
  
+ TCAAAGCCCA ATTGTTTAAG CAGTAAGTCG GTTATCGGAA GTCAATTTGG ACACGCTCTG TCTCTCTTAT   
  
  
+ CTAATGTAGA CATGACGGTG TTCTAAGCTT GTAAGGCCTT GTCCTTGGCT CCATGGAACC TTCAATTTAT   
  
  
+ CAGTCAAATC TACAGTGTGA ACGTATATTA TTCGATTCTG TATTTAATTT TTGGTACCAC GCCTCTTTAA   
  
  
+ AAGAAAATAA AAGGTTACAA AATTCACAAA ACGTTTTTA  

- AGTTGTGAAA TTTTACTATA CTTTTATGAT TTTTATATAA TTAAATTTAT TTTTTTAATT ATAAAAATTT   
  
  
- TGTATTGTCT AGATAAACAT AACAAGTGTC CTTTTTACAT TATTACAAAA ACAGAAAGAC TTTTTTTTTA   
  
  
- TAATTTAACC AATAACCTCC GTACCTTCAA AAAAGTATTA CCCGTGATCA ACAATTGTTT AATTTATCCC   
  
  
- CGTTTATTCA GTAAAGTTTT TATCTCACTC TACTGGATTT ATAGGAATAT TCTTTTCTTT AATTTTCACC   
  
  
- ACAGGTTCTC GAAATAACAT AAAAAGTGTA CTAAAAAAAT AATGATATCC AGTTGATTCC CGGTTAAATC   
  
  
- ATAAACATAT TTATATACCT TTTTTTTTCG TGTCACTTAC TAGTTTTATA ATAACCTTCG TTTTTTTTTA   
  
  
- ATTTCAATCG GAGCTCCGCT ACAAAAACAT AAAAATGTTA CTAAAAAAAA TAGTGGTTCA CTTGAATCTC   
  
  
- CCTTAAGCCA TAAAAAGTAT TTATATCTTT TTATTTTCAC CAAACGCCCA CGTATCGTTC TCGGTTGTAC   
  
  
- ACACTCCACA AACCCGGAAG TACATTGCGA ACGCTGTGGA GGGTTGCAAG TTTTATGGGA AAGATAGCAC   
  
  
- ATTAACCTTC GCTTCGCTAC AGGAAAAGGA ACGCCCCACA ACGTACACCA ACAACCACTA TGTCAAACAA   
  
  
- CGGCTGTTTC GAAAGGGAGA TATAAAAGAG AGAGGAGGGA TGTTTTAAAT GAGACCGGTA CGTTTTAAAA   
  
  
- TCTTAACAGG AAACTAAATA CCCCTAAAGG TGAATTCAGG AATAAAAAAT AAAGATTAAA AATAAAAACC   
  
  
- AGAAAAACAT TTTGAAATTA AAAAAAATTA AAGTAGAAAT TTAGAGTTAA AGATTATAAA AAATAAAGTT   
  
  
- GGAATTAGGA ATAAGAAAAT TAACAAAAAA ATTAAAAAAA ACTCGGTAAA CAATTTAACT ATAAAGAATA   
  
  
- GTTAAAGTGA GAAATTATTT TTTTAAGCAA ACGTAAAGAA TAAAGTTGGA ACTAGGAGTA AGAAAATCAA   
  
  
- AAAAATCAAA AATAGGAAAA CAATTTAACA AAAACGAAAA GTTAAAGTAA GAATATCTTA AATAAAATAT   
  
  
- AAAGTTAAAA GTAAGAAAAA AAATTAACGA TAAAAAATTA GTAAAACATA TTAACTTAAA AAAAAAAGTT   
  
  
- AAAGTAGGAA GTTATAAATT AACTAAACCT TAATTCAAAG TTCCAAAAAG GTCTATCCTA CAAAGATCAG   
  
  
- ATTATTGGAC CTAGTGTTCA AATTTTTCAA TTGTACCCAA CTATATAAAA AAAATATTTT TTTATCTGAA   
  
  
- ACACTAAAAG AAACAAAATA AAAGATAGAT TAATAGAACT ACAATATTGT ATCCGGCGTA CAAACCGCCC   
  
  
- TATTGAACTC AATCGAATTG GGACTAATGA TCCTAATATC CAAACAGTAT AATTGAACTC AACTGATTTG   
  
  
- GACTAAAAAA ATATTAAAAC AAATAGATTA AAATAAGAAA GTATAAATTA CCTGAATCTT AATTCAAAAC   
  
  
- AAGAAATAGT AGAAAATTTT TTTTACAAAA AAATGCAATA GTACTAAAAA AAAAATTAAA TATAGTAAAC   
  
  
- AATAACAATA GATAAATACT AGATAAATTT TATTTTAATT GAAGAAATTT AATCAATTTA GATAATAAAC   
  
  
- TTAGTGCTTT TTTTTATCAG TGGCGTACTG CTTCCAGGTT TTGATCGGTA CTCCATAAGG AGCTAATTAT   
  
  
- AGTTTCGGGT TAACAAATTC GTCATTCAGC CAATAGCCTT CAGTTAAACC TGTGCGAGAC AGAGAGAATA   
  
  
- GATTACATCT GTACTGCCAC AAGATTCGAA CATTCCGGAA CAGGAACCGA GGTACCTTGG AAGTTAAATA   
  
  
- GTCAGTTTAG ATGTCACACT TGCATATAAT AAGCTAAGAC ATAAATTAAA AACCATGGTG CGGAGAAATT   
  
  
- TTCTTTTATT TTCCAATGTT TTAAGTGTTT TGCAAAAAT

+     Box III

| Site Name | Organism | Position | Strand | Matrix score. | sequence | function |
| --- | --- | --- | --- | --- | --- | --- |
| Box III | Pisum sativum | 979 | + | 11 | atCATTTTCACt | protein binding site |

>Potri.005G195000.1   
+ TCAACACTTT AAAATGATAT GAAAATACTA AAAATATATT AATTTAAATA AAAAAATTAA TATTTTTAAA   
  
  
+ ACATAACAGA TCTATTTGTA TTGTTCACAG GAAAAATGTA ATAATGTTTT TGTCTTTCTG AAAAAAAAAT   
  
  
+ ATTAAATTGG TTATTGGAGG CATGGAAGTT TTTTCATAAT GGGCACTAGT TGTTAACAAA TTAAATAGGG   
  
  
+ GCAAATAAGT CATTTCAAAA ATAGAGTGAG ATGACCTAAA TATCCTTATA AGAAAAGAAA TTAAAAGTGG   
  
  
+ TGTCCAAGAG CTTTATTGTA TTTTTCACAT GATTTTTTTA TTACTATAGG TCAACTAAGG GCCAATTTAG   
  
  
+ TATTTGTATA AATATATGGA AAAAAAAAGC ACAGTGAATG ATCAAAATAT TATTGGAAGC AAAAAAAAAT   
  
  
+ TAAAGTTAGC CTCGAGGCGA TGTTTTTGTA TTTTTACAAT GATTTTTTTT ATCACCAAGT GAACTTAGAG   
  
  
+ GGAATTCGGT ATTTTTCATA AATATAGAAA AATAAAAGTG GTTTGCGGGT GCATAGCAAG AGCCAACATG   
  
  
+ TGTGAGGTGT TTGGGCCTTC ATGTAACGCT TGCGACACCT CCCAACGTTC AAAATACCCT TTCTATCGTG   
  
  
+ TAATTGGAAG CGAAGCGATG TCCTTTTCCT TGCGGGGTGT TGCATGTGGT TGTTGGTGAT ACAGTTTGTT   
  
  
+ GCCGACAAAG CTTTCCCTCT ATATTTTCTC TCTCCTCCCT ACAAAATTTA CTCTGGCCAT GCAAAATTTT   
  
  
+ AGAATTGTCC TTTGATTTAT GGGGATTTCC ACTTAAGTCC TTATTTTTTA TTTCTAATTT TTATTTTTGG   
  
  
+ TCTTTTTGTA AAACTTTAAT TTTTTTTAAT TTCATCTTTA AATCTCAATT TCTAATATTT TTTATTTCAA   
  
  
+ CCTTAATCCT TATTCTTTTA ATTGTTTTTT TAATTTTTTT TGAGCCATTT GTTAAATTGA TATTTCTTAT   
  
  
+ CAATTTCACT CTTTAATAAA AAAATTCGTT TGCATTTCTT ATTTCAACCT TGATCCTCAT TCTTTTAGTT   
  
  
+ TTTTTAGTTT TTATCCTTTT GTTAAATTGT TTTTGCTTTT CAATTTCATT CTTATAGAAT TTATTTTATA   
  
  
+ TTTCAATTTT CATTCTTTTT TTTAATTGCT ATTTTTTAAT CATTTTGTAT AATTGAATTT TTTTTTTCAA   
  
  
+ TTTCATCCTT CAATATTTAA TTGATTTGGA ATTAAGTTTC AAGGTTTTTC CAGATAGGAT GTTTCTAGTC   
  
  
+ TAATAACCTG GATCACAAGT TTAAAAAGTT AACATGGGTT GATATATTTT TTTTATAAAA AAATAGACTT   
  
  
+ TGTGATTTTC TTTGTTTTAT TTTCTATCTA ATTATCTTGA TGTTATAACA TAGGCCGCAT GTTTGGCGGG   
  
  
+ ATAACTTGAG TTAGCTTAAC CCTGATTACT AGGATTATAG GTTTGTCATA TTAACTTGAG TTGACTAAAC   
  
  
+ CTGATTTTTT TATAATTTTG TTTATCTAAT TTTATTCTTT CATATTTAAT GGACTTAGAA TTAAGTTTTG   
  
  
+ TTCTTTATCA TCTTTTAAAA AAAATGTTTT TTTACGTTAT CATGATTTTT TTTTTAATTT ATATCATTTG   
  
  
+ TTATTGTTAT CTATTTATGA TCTATTTAAA ATAAAATTAA CTTCTTTAAA TTAGTTAAAT CTATTATTTG   
  
  
+ AATCACGAAA AAAAATAGTC ACCGCATGAC GAAGGTCCAA AACTAGCCAT GAGGTATTCC TCGATTAATA   
  
  
+ TCAAAGCCCA ATTGTTTAAG CAGTAAGTCG GTTATCGGAA GTCAATTTGG ACACGCTCTG TCTCTCTTAT   
  
  
+ CTAATGTAGA CATGACGGTG TTCTAAGCTT GTAAGGCCTT GTCCTTGGCT CCATGGAACC TTCAATTTAT   
  
  
+ CAGTCAAATC TACAGTGTGA ACGTATATTA TTCGATTCTG TATTTAATTT TTGGTACCAC GCCTCTTTAA   
  
  
+ AAGAAAATAA AAGGTTACAA AATTCACAAA ACGTTTTTA  

- AGTTGTGAAA TTTTACTATA CTTTTATGAT TTTTATATAA TTAAATTTAT TTTTTTAATT ATAAAAATTT   
  
  
- TGTATTGTCT AGATAAACAT AACAAGTGTC CTTTTTACAT TATTACAAAA ACAGAAAGAC TTTTTTTTTA   
  
  
- TAATTTAACC AATAACCTCC GTACCTTCAA AAAAGTATTA CCCGTGATCA ACAATTGTTT AATTTATCCC   
  
  
- CGTTTATTCA GTAAAGTTTT TATCTCACTC TACTGGATTT ATAGGAATAT TCTTTTCTTT AATTTTCACC   
  
  
- ACAGGTTCTC GAAATAACAT AAAAAGTGTA CTAAAAAAAT AATGATATCC AGTTGATTCC CGGTTAAATC   
  
  
- ATAAACATAT TTATATACCT TTTTTTTTCG TGTCACTTAC TAGTTTTATA ATAACCTTCG TTTTTTTTTA   
  
  
- ATTTCAATCG GAGCTCCGCT ACAAAAACAT AAAAATGTTA CTAAAAAAAA TAGTGGTTCA CTTGAATCTC   
  
  
- CCTTAAGCCA TAAAAAGTAT TTATATCTTT TTATTTTCAC CAAACGCCCA CGTATCGTTC TCGGTTGTAC   
  
  
- ACACTCCACA AACCCGGAAG TACATTGCGA ACGCTGTGGA GGGTTGCAAG TTTTATGGGA AAGATAGCAC   
  
  
- ATTAACCTTC GCTTCGCTAC AGGAAAAGGA ACGCCCCACA ACGTACACCA ACAACCACTA TGTCAAACAA   
  
  
- CGGCTGTTTC GAAAGGGAGA TATAAAAGAG AGAGGAGGGA TGTTTTAAAT GAGACCGGTA CGTTTTAAAA   
  
  
- TCTTAACAGG AAACTAAATA CCCCTAAAGG TGAATTCAGG AATAAAAAAT AAAGATTAAA AATAAAAACC   
  
  
- AGAAAAACAT TTTGAAATTA AAAAAAATTA AAGTAGAAAT TTAGAGTTAA AGATTATAAA AAATAAAGTT   
  
  
- GGAATTAGGA ATAAGAAAAT TAACAAAAAA ATTAAAAAAA ACTCGGTAAA CAATTTAACT ATAAAGAATA   
  
  
- GTTAAAGTGA GAAATTATTT TTTTAAGCAA ACGTAAAGAA TAAAGTTGGA ACTAGGAGTA AGAAAATCAA   
  
  
- AAAAATCAAA AATAGGAAAA CAATTTAACA AAAACGAAAA GTTAAAGTAA GAATATCTTA AATAAAATAT   
  
  
- AAAGTTAAAA GTAAGAAAAA AAATTAACGA TAAAAAATTA GTAAAACATA TTAACTTAAA AAAAAAAGTT   
  
  
- AAAGTAGGAA GTTATAAATT AACTAAACCT TAATTCAAAG TTCCAAAAAG GTCTATCCTA CAAAGATCAG   
  
  
- ATTATTGGAC CTAGTGTTCA AATTTTTCAA TTGTACCCAA CTATATAAAA AAAATATTTT TTTATCTGAA   
  
  
- ACACTAAAAG AAACAAAATA AAAGATAGAT TAATAGAACT ACAATATTGT ATCCGGCGTA CAAACCGCCC   
  
  
- TATTGAACTC AATCGAATTG GGACTAATGA TCCTAATATC CAAACAGTAT AATTGAACTC AACTGATTTG   
  
  
- GACTAAAAAA ATATTAAAAC AAATAGATTA AAATAAGAAA GTATAAATTA CCTGAATCTT AATTCAAAAC   
  
  
- AAGAAATAGT AGAAAATTTT TTTTACAAAA AAATGCAATA GTACTAAAAA AAAAATTAAA TATAGTAAAC   
  
  
- AATAACAATA GATAAATACT AGATAAATTT TATTTTAATT GAAGAAATTT AATCAATTTA GATAATAAAC   
  
  
- TTAGTGCTTT TTTTTATCAG TGGCGTACTG CTTCCAGGTT TTGATCGGTA CTCCATAAGG AGCTAATTAT   
  
  
- AGTTTCGGGT TAACAAATTC GTCATTCAGC CAATAGCCTT CAGTTAAACC TGTGCGAGAC AGAGAGAATA   
  
  
- GATTACATCT GTACTGCCAC AAGATTCGAA CATTCCGGAA CAGGAACCGA GGTACCTTGG AAGTTAAATA   
  
  
- GTCAGTTTAG ATGTCACACT TGCATATAAT AAGCTAAGAC ATAAATTAAA AACCATGGTG CGGAGAAATT   
  
  
- TTCTTTTATT TTCCAATGTT TTAAGTGTTT TGCAAAAAT

+     CAAT-box

| Site Name | Organism | Position | Strand | Matrix score. | sequence | function |
| --- | --- | --- | --- | --- | --- | --- |
| CAAT-box | Nicotiana glutinosa | 1883 | + | 4 | CAAT |  |
| CAAT-box | Pisum sativum | 1895 | + | 5 | CAAAT | common cis-acting element in promoter and enhancer regions |
| CAAT-box | Pisum sativum | 1795 | - | 5 | CAAAT | common cis-acting element in promoter and enhancer regions |
| CAAT-box | Nicotiana glutinosa | 1793 | + | 4 | CAAT |  |
| CAAT-box | Nicotiana glutinosa | 1759 | + | 4 | CAAT |  |
| CAAT-box | Nicotiana glutinosa | 1761 | - | 4 | CAAT |  |
| CAAT-box | Arabidopsis thaliana | 1758 | + | 5 | CCAAT | common cis-acting element in promoter and enhancer regions |
| CAAT-box | Pisum sativum | 1676 | - | 5 | CAAAT | common cis-acting element in promoter and enhancer regions |
| CAAT-box | Pisum sativum | 1606 | - | 5 | CAAAT | common cis-acting element in promoter and enhancer regions |
| CAAT-box | Nicotiana glutinosa | 1613 | - | 4 | CAAT |  |
| CAAT-box | Nicotiana glutinosa | 1210 | - | 4 | CAAT |  |
| CAAT-box | Pisum sativum | 1214 | - | 5 | CAAAT | common cis-acting element in promoter and enhancer regions |
| CAAT-box | Nicotiana glutinosa | 1201 | + | 4 | CAAT |  |
| CAAT-box | Nicotiana glutinosa | 1188 | + | 4 | CAAT |  |
| CAAT-box | Nicotiana glutinosa | 1145 | - | 4 | CAAT |  |
| CAAT-box | Nicotiana glutinosa | 1172 | - | 4 | CAAT |  |
| CAAT-box | Nicotiana glutinosa | 1091 | + | 4 | CAAT |  |
| CAAT-box | Nicotiana glutinosa | 1124 | + | 4 | CAAT |  |
| CAAT-box | Nicotiana glutinosa | 1076 | - | 4 | CAAT |  |
| CAAT-box | Nicotiana glutinosa | 981 | + | 4 | CAAT |  |
| CAAT-box | Nicotiana glutinosa | 966 | - | 4 | CAAT |  |
| CAAT-box | Pisum sativum | 957 | - | 5 | CAAAT | common cis-acting element in promoter and enhancer regions |
| CAAT-box | Nicotiana glutinosa | 931 | - | 4 | CAAT |  |
| CAAT-box | Nicotiana glutinosa | 886 | + | 4 | CAAT |  |
| CAAT-box | Arabidopsis thaliana | 633 | - | 5 | CCAAT | common cis-acting element in promoter and enhancer regions |
| CAAT-box | Nicotiana glutinosa | 774 | - | 4 | CAAT |  |
| CAAT-box | Nicotiana glutinosa | 457 | + | 4 | CAAT |  |
| CAAT-box | Arabidopsis thaliana | 402 | - | 5 | CCAAT | common cis-acting element in promoter and enhancer regions |
| CAAT-box | Nicotiana glutinosa | 343 | + | 4 | CAAT |  |
| CAAT-box | Pisum sativum | 352 | - | 5 | CAAAT | common cis-acting element in promoter and enhancer regions |
| CAAT-box | Arabidopsis thaliana | 342 | + | 5 | CCAAT | common cis-acting element in promoter and enhancer regions |
| CAAT-box | Nicotiana glutinosa | 295 | - | 4 | CAAT |  |
| CAAT-box | Pisum sativum | 84 | - | 5 | CAAAT | common cis-acting element in promoter and enhancer regions |
| CAAT-box | Arabidopsis thaliana | 153 | - | 5 | CCAAT | common cis-acting element in promoter and enhancer regions |
| CAAT-box | Arabidopsis thaliana | 146 | - | 5 | CCAAT | common cis-acting element in promoter and enhancer regions |
| CAAT-box | Nicotiana glutinosa | 90 | - | 4 | CAAT |  |
| CAAT-box | Pisum sativum | 197 | + | 5 | CAAAT | common cis-acting element in promoter and enhancer regions |
| CAAT-box | Pisum sativum | 212 | + | 5 | CAAAT | common cis-acting element in promoter and enhancer regions |

>Potri.005G195000.1   
+ TCAACACTTT AAAATGATAT GAAAATACTA AAAATATATT AATTTAAATA AAAAAATTAA TATTTTTAAA   
  
  
+ ACATAACAGA TCTATTTGTA TTGTTCACAG GAAAAATGTA ATAATGTTTT TGTCTTTCTG AAAAAAAAAT   
  
  
+ ATTAAATTGG TTATTGGAGG CATGGAAGTT TTTTCATAAT GGGCACTAGT TGTTAACAAA TTAAATAGGG   
  
  
+ GCAAATAAGT CATTTCAAAA ATAGAGTGAG ATGACCTAAA TATCCTTATA AGAAAAGAAA TTAAAAGTGG   
  
  
+ TGTCCAAGAG CTTTATTGTA TTTTTCACAT GATTTTTTTA TTACTATAGG TCAACTAAGG GCCAATTTAG   
  
  
+ TATTTGTATA AATATATGGA AAAAAAAAGC ACAGTGAATG ATCAAAATAT TATTGGAAGC AAAAAAAAAT   
  
  
+ TAAAGTTAGC CTCGAGGCGA TGTTTTTGTA TTTTTACAAT GATTTTTTTT ATCACCAAGT GAACTTAGAG   
  
  
+ GGAATTCGGT ATTTTTCATA AATATAGAAA AATAAAAGTG GTTTGCGGGT GCATAGCAAG AGCCAACATG   
  
  
+ TGTGAGGTGT TTGGGCCTTC ATGTAACGCT TGCGACACCT CCCAACGTTC AAAATACCCT TTCTATCGTG   
  
  
+ TAATTGGAAG CGAAGCGATG TCCTTTTCCT TGCGGGGTGT TGCATGTGGT TGTTGGTGAT ACAGTTTGTT   
  
  
+ GCCGACAAAG CTTTCCCTCT ATATTTTCTC TCTCCTCCCT ACAAAATTTA CTCTGGCCAT GCAAAATTTT   
  
  
+ AGAATTGTCC TTTGATTTAT GGGGATTTCC ACTTAAGTCC TTATTTTTTA TTTCTAATTT TTATTTTTGG   
  
  
+ TCTTTTTGTA AAACTTTAAT TTTTTTTAAT TTCATCTTTA AATCTCAATT TCTAATATTT TTTATTTCAA   
  
  
+ CCTTAATCCT TATTCTTTTA ATTGTTTTTT TAATTTTTTT TGAGCCATTT GTTAAATTGA TATTTCTTAT   
  
  
+ CAATTTCACT CTTTAATAAA AAAATTCGTT TGCATTTCTT ATTTCAACCT TGATCCTCAT TCTTTTAGTT   
  
  
+ TTTTTAGTTT TTATCCTTTT GTTAAATTGT TTTTGCTTTT CAATTTCATT CTTATAGAAT TTATTTTATA   
  
  
+ TTTCAATTTT CATTCTTTTT TTTAATTGCT ATTTTTTAAT CATTTTGTAT AATTGAATTT TTTTTTTCAA   
  
  
+ TTTCATCCTT CAATATTTAA TTGATTTGGA ATTAAGTTTC AAGGTTTTTC CAGATAGGAT GTTTCTAGTC   
  
  
+ TAATAACCTG GATCACAAGT TTAAAAAGTT AACATGGGTT GATATATTTT TTTTATAAAA AAATAGACTT   
  
  
+ TGTGATTTTC TTTGTTTTAT TTTCTATCTA ATTATCTTGA TGTTATAACA TAGGCCGCAT GTTTGGCGGG   
  
  
+ ATAACTTGAG TTAGCTTAAC CCTGATTACT AGGATTATAG GTTTGTCATA TTAACTTGAG TTGACTAAAC   
  
  
+ CTGATTTTTT TATAATTTTG TTTATCTAAT TTTATTCTTT CATATTTAAT GGACTTAGAA TTAAGTTTTG   
  
  
+ TTCTTTATCA TCTTTTAAAA AAAATGTTTT TTTACGTTAT CATGATTTTT TTTTTAATTT ATATCATTTG   
  
  
+ TTATTGTTAT CTATTTATGA TCTATTTAAA ATAAAATTAA CTTCTTTAAA TTAGTTAAAT CTATTATTTG   
  
  
+ AATCACGAAA AAAAATAGTC ACCGCATGAC GAAGGTCCAA AACTAGCCAT GAGGTATTCC TCGATTAATA   
  
  
+ TCAAAGCCCA ATTGTTTAAG CAGTAAGTCG GTTATCGGAA GTCAATTTGG ACACGCTCTG TCTCTCTTAT   
  
  
+ CTAATGTAGA CATGACGGTG TTCTAAGCTT GTAAGGCCTT GTCCTTGGCT CCATGGAACC TTCAATTTAT   
  
  
+ CAGTCAAATC TACAGTGTGA ACGTATATTA TTCGATTCTG TATTTAATTT TTGGTACCAC GCCTCTTTAA   
  
  
+ AAGAAAATAA AAGGTTACAA AATTCACAAA ACGTTTTTA  

- AGTTGTGAAA TTTTACTATA CTTTTATGAT TTTTATATAA TTAAATTTAT TTTTTTAATT ATAAAAATTT   
  
  
- TGTATTGTCT AGATAAACAT AACAAGTGTC CTTTTTACAT TATTACAAAA ACAGAAAGAC TTTTTTTTTA   
  
  
- TAATTTAACC AATAACCTCC GTACCTTCAA AAAAGTATTA CCCGTGATCA ACAATTGTTT AATTTATCCC   
  
  
- CGTTTATTCA GTAAAGTTTT TATCTCACTC TACTGGATTT ATAGGAATAT TCTTTTCTTT AATTTTCACC   
  
  
- ACAGGTTCTC GAAATAACAT AAAAAGTGTA CTAAAAAAAT AATGATATCC AGTTGATTCC CGGTTAAATC   
  
  
- ATAAACATAT TTATATACCT TTTTTTTTCG TGTCACTTAC TAGTTTTATA ATAACCTTCG TTTTTTTTTA   
  
  
- ATTTCAATCG GAGCTCCGCT ACAAAAACAT AAAAATGTTA CTAAAAAAAA TAGTGGTTCA CTTGAATCTC   
  
  
- CCTTAAGCCA TAAAAAGTAT TTATATCTTT TTATTTTCAC CAAACGCCCA CGTATCGTTC TCGGTTGTAC   
  
  
- ACACTCCACA AACCCGGAAG TACATTGCGA ACGCTGTGGA GGGTTGCAAG TTTTATGGGA AAGATAGCAC   
  
  
- ATTAACCTTC GCTTCGCTAC AGGAAAAGGA ACGCCCCACA ACGTACACCA ACAACCACTA TGTCAAACAA   
  
  
- CGGCTGTTTC GAAAGGGAGA TATAAAAGAG AGAGGAGGGA TGTTTTAAAT GAGACCGGTA CGTTTTAAAA   
  
  
- TCTTAACAGG AAACTAAATA CCCCTAAAGG TGAATTCAGG AATAAAAAAT AAAGATTAAA AATAAAAACC   
  
  
- AGAAAAACAT TTTGAAATTA AAAAAAATTA AAGTAGAAAT TTAGAGTTAA AGATTATAAA AAATAAAGTT   
  
  
- GGAATTAGGA ATAAGAAAAT TAACAAAAAA ATTAAAAAAA ACTCGGTAAA CAATTTAACT ATAAAGAATA   
  
  
- GTTAAAGTGA GAAATTATTT TTTTAAGCAA ACGTAAAGAA TAAAGTTGGA ACTAGGAGTA AGAAAATCAA   
  
  
- AAAAATCAAA AATAGGAAAA CAATTTAACA AAAACGAAAA GTTAAAGTAA GAATATCTTA AATAAAATAT   
  
  
- AAAGTTAAAA GTAAGAAAAA AAATTAACGA TAAAAAATTA GTAAAACATA TTAACTTAAA AAAAAAAGTT   
  
  
- AAAGTAGGAA GTTATAAATT AACTAAACCT TAATTCAAAG TTCCAAAAAG GTCTATCCTA CAAAGATCAG   
  
  
- ATTATTGGAC CTAGTGTTCA AATTTTTCAA TTGTACCCAA CTATATAAAA AAAATATTTT TTTATCTGAA   
  
  
- ACACTAAAAG AAACAAAATA AAAGATAGAT TAATAGAACT ACAATATTGT ATCCGGCGTA CAAACCGCCC   
  
  
- TATTGAACTC AATCGAATTG GGACTAATGA TCCTAATATC CAAACAGTAT AATTGAACTC AACTGATTTG   
  
  
- GACTAAAAAA ATATTAAAAC AAATAGATTA AAATAAGAAA GTATAAATTA CCTGAATCTT AATTCAAAAC   
  
  
- AAGAAATAGT AGAAAATTTT TTTTACAAAA AAATGCAATA GTACTAAAAA AAAAATTAAA TATAGTAAAC   
  
  
- AATAACAATA GATAAATACT AGATAAATTT TATTTTAATT GAAGAAATTT AATCAATTTA GATAATAAAC   
  
  
- TTAGTGCTTT TTTTTATCAG TGGCGTACTG CTTCCAGGTT TTGATCGGTA CTCCATAAGG AGCTAATTAT   
  
  
- AGTTTCGGGT TAACAAATTC GTCATTCAGC CAATAGCCTT CAGTTAAACC TGTGCGAGAC AGAGAGAATA   
  
  
- GATTACATCT GTACTGCCAC AAGATTCGAA CATTCCGGAA CAGGAACCGA GGTACCTTGG AAGTTAAATA   
  
  
- GTCAGTTTAG ATGTCACACT TGCATATAAT AAGCTAAGAC ATAAATTAAA AACCATGGTG CGGAGAAATT   
  
  
- TTCTTTTATT TTCCAATGTT TTAAGTGTTT TGCAAAAAT

+     CGTCA-motif

| Site Name | Organism | Position | Strand | Matrix score. | sequence | function |
| --- | --- | --- | --- | --- | --- | --- |
| CGTCA-motif | Hordeum vulgare | 1707 | - | 5 | CGTCA | cis-acting regulatory element involved in the MeJA-responsiveness |
| CGTCA-motif | Hordeum vulgare | 1833 | - | 5 | CGTCA | cis-acting regulatory element involved in the MeJA-responsiveness |

>Potri.005G195000.1   
+ TCAACACTTT AAAATGATAT GAAAATACTA AAAATATATT AATTTAAATA AAAAAATTAA TATTTTTAAA   
  
  
+ ACATAACAGA TCTATTTGTA TTGTTCACAG GAAAAATGTA ATAATGTTTT TGTCTTTCTG AAAAAAAAAT   
  
  
+ ATTAAATTGG TTATTGGAGG CATGGAAGTT TTTTCATAAT GGGCACTAGT TGTTAACAAA TTAAATAGGG   
  
  
+ GCAAATAAGT CATTTCAAAA ATAGAGTGAG ATGACCTAAA TATCCTTATA AGAAAAGAAA TTAAAAGTGG   
  
  
+ TGTCCAAGAG CTTTATTGTA TTTTTCACAT GATTTTTTTA TTACTATAGG TCAACTAAGG GCCAATTTAG   
  
  
+ TATTTGTATA AATATATGGA AAAAAAAAGC ACAGTGAATG ATCAAAATAT TATTGGAAGC AAAAAAAAAT   
  
  
+ TAAAGTTAGC CTCGAGGCGA TGTTTTTGTA TTTTTACAAT GATTTTTTTT ATCACCAAGT GAACTTAGAG   
  
  
+ GGAATTCGGT ATTTTTCATA AATATAGAAA AATAAAAGTG GTTTGCGGGT GCATAGCAAG AGCCAACATG   
  
  
+ TGTGAGGTGT TTGGGCCTTC ATGTAACGCT TGCGACACCT CCCAACGTTC AAAATACCCT TTCTATCGTG   
  
  
+ TAATTGGAAG CGAAGCGATG TCCTTTTCCT TGCGGGGTGT TGCATGTGGT TGTTGGTGAT ACAGTTTGTT   
  
  
+ GCCGACAAAG CTTTCCCTCT ATATTTTCTC TCTCCTCCCT ACAAAATTTA CTCTGGCCAT GCAAAATTTT   
  
  
+ AGAATTGTCC TTTGATTTAT GGGGATTTCC ACTTAAGTCC TTATTTTTTA TTTCTAATTT TTATTTTTGG   
  
  
+ TCTTTTTGTA AAACTTTAAT TTTTTTTAAT TTCATCTTTA AATCTCAATT TCTAATATTT TTTATTTCAA   
  
  
+ CCTTAATCCT TATTCTTTTA ATTGTTTTTT TAATTTTTTT TGAGCCATTT GTTAAATTGA TATTTCTTAT   
  
  
+ CAATTTCACT CTTTAATAAA AAAATTCGTT TGCATTTCTT ATTTCAACCT TGATCCTCAT TCTTTTAGTT   
  
  
+ TTTTTAGTTT TTATCCTTTT GTTAAATTGT TTTTGCTTTT CAATTTCATT CTTATAGAAT TTATTTTATA   
  
  
+ TTTCAATTTT CATTCTTTTT TTTAATTGCT ATTTTTTAAT CATTTTGTAT AATTGAATTT TTTTTTTCAA   
  
  
+ TTTCATCCTT CAATATTTAA TTGATTTGGA ATTAAGTTTC AAGGTTTTTC CAGATAGGAT GTTTCTAGTC   
  
  
+ TAATAACCTG GATCACAAGT TTAAAAAGTT AACATGGGTT GATATATTTT TTTTATAAAA AAATAGACTT   
  
  
+ TGTGATTTTC TTTGTTTTAT TTTCTATCTA ATTATCTTGA TGTTATAACA TAGGCCGCAT GTTTGGCGGG   
  
  
+ ATAACTTGAG TTAGCTTAAC CCTGATTACT AGGATTATAG GTTTGTCATA TTAACTTGAG TTGACTAAAC   
  
  
+ CTGATTTTTT TATAATTTTG TTTATCTAAT TTTATTCTTT CATATTTAAT GGACTTAGAA TTAAGTTTTG   
  
  
+ TTCTTTATCA TCTTTTAAAA AAAATGTTTT TTTACGTTAT CATGATTTTT TTTTTAATTT ATATCATTTG   
  
  
+ TTATTGTTAT CTATTTATGA TCTATTTAAA ATAAAATTAA CTTCTTTAAA TTAGTTAAAT CTATTATTTG   
  
  
+ AATCACGAAA AAAAATAGTC ACCGCATGAC GAAGGTCCAA AACTAGCCAT GAGGTATTCC TCGATTAATA   
  
  
+ TCAAAGCCCA ATTGTTTAAG CAGTAAGTCG GTTATCGGAA GTCAATTTGG ACACGCTCTG TCTCTCTTAT   
  
  
+ CTAATGTAGA CATGACGGTG TTCTAAGCTT GTAAGGCCTT GTCCTTGGCT CCATGGAACC TTCAATTTAT   
  
  
+ CAGTCAAATC TACAGTGTGA ACGTATATTA TTCGATTCTG TATTTAATTT TTGGTACCAC GCCTCTTTAA   
  
  
+ AAGAAAATAA AAGGTTACAA AATTCACAAA ACGTTTTTA  

- AGTTGTGAAA TTTTACTATA CTTTTATGAT TTTTATATAA TTAAATTTAT TTTTTTAATT ATAAAAATTT   
  
  
- TGTATTGTCT AGATAAACAT AACAAGTGTC CTTTTTACAT TATTACAAAA ACAGAAAGAC TTTTTTTTTA   
  
  
- TAATTTAACC AATAACCTCC GTACCTTCAA AAAAGTATTA CCCGTGATCA ACAATTGTTT AATTTATCCC   
  
  
- CGTTTATTCA GTAAAGTTTT TATCTCACTC TACTGGATTT ATAGGAATAT TCTTTTCTTT AATTTTCACC   
  
  
- ACAGGTTCTC GAAATAACAT AAAAAGTGTA CTAAAAAAAT AATGATATCC AGTTGATTCC CGGTTAAATC   
  
  
- ATAAACATAT TTATATACCT TTTTTTTTCG TGTCACTTAC TAGTTTTATA ATAACCTTCG TTTTTTTTTA   
  
  
- ATTTCAATCG GAGCTCCGCT ACAAAAACAT AAAAATGTTA CTAAAAAAAA TAGTGGTTCA CTTGAATCTC   
  
  
- CCTTAAGCCA TAAAAAGTAT TTATATCTTT TTATTTTCAC CAAACGCCCA CGTATCGTTC TCGGTTGTAC   
  
  
- ACACTCCACA AACCCGGAAG TACATTGCGA ACGCTGTGGA GGGTTGCAAG TTTTATGGGA AAGATAGCAC   
  
  
- ATTAACCTTC GCTTCGCTAC AGGAAAAGGA ACGCCCCACA ACGTACACCA ACAACCACTA TGTCAAACAA   
  
  
- CGGCTGTTTC GAAAGGGAGA TATAAAAGAG AGAGGAGGGA TGTTTTAAAT GAGACCGGTA CGTTTTAAAA   
  
  
- TCTTAACAGG AAACTAAATA CCCCTAAAGG TGAATTCAGG AATAAAAAAT AAAGATTAAA AATAAAAACC   
  
  
- AGAAAAACAT TTTGAAATTA AAAAAAATTA AAGTAGAAAT TTAGAGTTAA AGATTATAAA AAATAAAGTT   
  
  
- GGAATTAGGA ATAAGAAAAT TAACAAAAAA ATTAAAAAAA ACTCGGTAAA CAATTTAACT ATAAAGAATA   
  
  
- GTTAAAGTGA GAAATTATTT TTTTAAGCAA ACGTAAAGAA TAAAGTTGGA ACTAGGAGTA AGAAAATCAA   
  
  
- AAAAATCAAA AATAGGAAAA CAATTTAACA AAAACGAAAA GTTAAAGTAA GAATATCTTA AATAAAATAT   
  
  
- AAAGTTAAAA GTAAGAAAAA AAATTAACGA TAAAAAATTA GTAAAACATA TTAACTTAAA AAAAAAAGTT   
  
  
- AAAGTAGGAA GTTATAAATT AACTAAACCT TAATTCAAAG TTCCAAAAAG GTCTATCCTA CAAAGATCAG   
  
  
- ATTATTGGAC CTAGTGTTCA AATTTTTCAA TTGTACCCAA CTATATAAAA AAAATATTTT TTTATCTGAA   
  
  
- ACACTAAAAG AAACAAAATA AAAGATAGAT TAATAGAACT ACAATATTGT ATCCGGCGTA CAAACCGCCC   
  
  
- TATTGAACTC AATCGAATTG GGACTAATGA TCCTAATATC CAAACAGTAT AATTGAACTC AACTGATTTG   
  
  
- GACTAAAAAA ATATTAAAAC AAATAGATTA AAATAAGAAA GTATAAATTA CCTGAATCTT AATTCAAAAC   
  
  
- AAGAAATAGT AGAAAATTTT TTTTACAAAA AAATGCAATA GTACTAAAAA AAAAATTAAA TATAGTAAAC   
  
  
- AATAACAATA GATAAATACT AGATAAATTT TATTTTAATT GAAGAAATTT AATCAATTTA GATAATAAAC   
  
  
- TTAGTGCTTT TTTTTATCAG TGGCGTACTG CTTCCAGGTT TTGATCGGTA CTCCATAAGG AGCTAATTAT   
  
  
- AGTTTCGGGT TAACAAATTC GTCATTCAGC CAATAGCCTT CAGTTAAACC TGTGCGAGAC AGAGAGAATA   
  
  
- GATTACATCT GTACTGCCAC AAGATTCGAA CATTCCGGAA CAGGAACCGA GGTACCTTGG AAGTTAAATA   
  
  
- GTCAGTTTAG ATGTCACACT TGCATATAAT AAGCTAAGAC ATAAATTAAA AACCATGGTG CGGAGAAATT   
  
  
- TTCTTTTATT TTCCAATGTT TTAAGTGTTT TGCAAAAAT

+     DRE core

| Site Name | Organism | Position | Strand | Matrix score. | sequence | function |
| --- | --- | --- | --- | --- | --- | --- |
| DRE core | Arabidopsis thaliana | 701 | + | 6 | GCCGAC |  |

>Potri.005G195000.1   
+ TCAACACTTT AAAATGATAT GAAAATACTA AAAATATATT AATTTAAATA AAAAAATTAA TATTTTTAAA   
  
  
+ ACATAACAGA TCTATTTGTA TTGTTCACAG GAAAAATGTA ATAATGTTTT TGTCTTTCTG AAAAAAAAAT   
  
  
+ ATTAAATTGG TTATTGGAGG CATGGAAGTT TTTTCATAAT GGGCACTAGT TGTTAACAAA TTAAATAGGG   
  
  
+ GCAAATAAGT CATTTCAAAA ATAGAGTGAG ATGACCTAAA TATCCTTATA AGAAAAGAAA TTAAAAGTGG   
  
  
+ TGTCCAAGAG CTTTATTGTA TTTTTCACAT GATTTTTTTA TTACTATAGG TCAACTAAGG GCCAATTTAG   
  
  
+ TATTTGTATA AATATATGGA AAAAAAAAGC ACAGTGAATG ATCAAAATAT TATTGGAAGC AAAAAAAAAT   
  
  
+ TAAAGTTAGC CTCGAGGCGA TGTTTTTGTA TTTTTACAAT GATTTTTTTT ATCACCAAGT GAACTTAGAG   
  
  
+ GGAATTCGGT ATTTTTCATA AATATAGAAA AATAAAAGTG GTTTGCGGGT GCATAGCAAG AGCCAACATG   
  
  
+ TGTGAGGTGT TTGGGCCTTC ATGTAACGCT TGCGACACCT CCCAACGTTC AAAATACCCT TTCTATCGTG   
  
  
+ TAATTGGAAG CGAAGCGATG TCCTTTTCCT TGCGGGGTGT TGCATGTGGT TGTTGGTGAT ACAGTTTGTT   
  
  
+ GCCGACAAAG CTTTCCCTCT ATATTTTCTC TCTCCTCCCT ACAAAATTTA CTCTGGCCAT GCAAAATTTT   
  
  
+ AGAATTGTCC TTTGATTTAT GGGGATTTCC ACTTAAGTCC TTATTTTTTA TTTCTAATTT TTATTTTTGG   
  
  
+ TCTTTTTGTA AAACTTTAAT TTTTTTTAAT TTCATCTTTA AATCTCAATT TCTAATATTT TTTATTTCAA   
  
  
+ CCTTAATCCT TATTCTTTTA ATTGTTTTTT TAATTTTTTT TGAGCCATTT GTTAAATTGA TATTTCTTAT   
  
  
+ CAATTTCACT CTTTAATAAA AAAATTCGTT TGCATTTCTT ATTTCAACCT TGATCCTCAT TCTTTTAGTT   
  
  
+ TTTTTAGTTT TTATCCTTTT GTTAAATTGT TTTTGCTTTT CAATTTCATT CTTATAGAAT TTATTTTATA   
  
  
+ TTTCAATTTT CATTCTTTTT TTTAATTGCT ATTTTTTAAT CATTTTGTAT AATTGAATTT TTTTTTTCAA   
  
  
+ TTTCATCCTT CAATATTTAA TTGATTTGGA ATTAAGTTTC AAGGTTTTTC CAGATAGGAT GTTTCTAGTC   
  
  
+ TAATAACCTG GATCACAAGT TTAAAAAGTT AACATGGGTT GATATATTTT TTTTATAAAA AAATAGACTT   
  
  
+ TGTGATTTTC TTTGTTTTAT TTTCTATCTA ATTATCTTGA TGTTATAACA TAGGCCGCAT GTTTGGCGGG   
  
  
+ ATAACTTGAG TTAGCTTAAC CCTGATTACT AGGATTATAG GTTTGTCATA TTAACTTGAG TTGACTAAAC   
  
  
+ CTGATTTTTT TATAATTTTG TTTATCTAAT TTTATTCTTT CATATTTAAT GGACTTAGAA TTAAGTTTTG   
  
  
+ TTCTTTATCA TCTTTTAAAA AAAATGTTTT TTTACGTTAT CATGATTTTT TTTTTAATTT ATATCATTTG   
  
  
+ TTATTGTTAT CTATTTATGA TCTATTTAAA ATAAAATTAA CTTCTTTAAA TTAGTTAAAT CTATTATTTG   
  
  
+ AATCACGAAA AAAAATAGTC ACCGCATGAC GAAGGTCCAA AACTAGCCAT GAGGTATTCC TCGATTAATA   
  
  
+ TCAAAGCCCA ATTGTTTAAG CAGTAAGTCG GTTATCGGAA GTCAATTTGG ACACGCTCTG TCTCTCTTAT   
  
  
+ CTAATGTAGA CATGACGGTG TTCTAAGCTT GTAAGGCCTT GTCCTTGGCT CCATGGAACC TTCAATTTAT   
  
  
+ CAGTCAAATC TACAGTGTGA ACGTATATTA TTCGATTCTG TATTTAATTT TTGGTACCAC GCCTCTTTAA   
  
  
+ AAGAAAATAA AAGGTTACAA AATTCACAAA ACGTTTTTA  

- AGTTGTGAAA TTTTACTATA CTTTTATGAT TTTTATATAA TTAAATTTAT TTTTTTAATT ATAAAAATTT   
  
  
- TGTATTGTCT AGATAAACAT AACAAGTGTC CTTTTTACAT TATTACAAAA ACAGAAAGAC TTTTTTTTTA   
  
  
- TAATTTAACC AATAACCTCC GTACCTTCAA AAAAGTATTA CCCGTGATCA ACAATTGTTT AATTTATCCC   
  
  
- CGTTTATTCA GTAAAGTTTT TATCTCACTC TACTGGATTT ATAGGAATAT TCTTTTCTTT AATTTTCACC   
  
  
- ACAGGTTCTC GAAATAACAT AAAAAGTGTA CTAAAAAAAT AATGATATCC AGTTGATTCC CGGTTAAATC   
  
  
- ATAAACATAT TTATATACCT TTTTTTTTCG TGTCACTTAC TAGTTTTATA ATAACCTTCG TTTTTTTTTA   
  
  
- ATTTCAATCG GAGCTCCGCT ACAAAAACAT AAAAATGTTA CTAAAAAAAA TAGTGGTTCA CTTGAATCTC   
  
  
- CCTTAAGCCA TAAAAAGTAT TTATATCTTT TTATTTTCAC CAAACGCCCA CGTATCGTTC TCGGTTGTAC   
  
  
- ACACTCCACA AACCCGGAAG TACATTGCGA ACGCTGTGGA GGGTTGCAAG TTTTATGGGA AAGATAGCAC   
  
  
- ATTAACCTTC GCTTCGCTAC AGGAAAAGGA ACGCCCCACA ACGTACACCA ACAACCACTA TGTCAAACAA   
  
  
- CGGCTGTTTC GAAAGGGAGA TATAAAAGAG AGAGGAGGGA TGTTTTAAAT GAGACCGGTA CGTTTTAAAA   
  
  
- TCTTAACAGG AAACTAAATA CCCCTAAAGG TGAATTCAGG AATAAAAAAT AAAGATTAAA AATAAAAACC   
  
  
- AGAAAAACAT TTTGAAATTA AAAAAAATTA AAGTAGAAAT TTAGAGTTAA AGATTATAAA AAATAAAGTT   
  
  
- GGAATTAGGA ATAAGAAAAT TAACAAAAAA ATTAAAAAAA ACTCGGTAAA CAATTTAACT ATAAAGAATA   
  
  
- GTTAAAGTGA GAAATTATTT TTTTAAGCAA ACGTAAAGAA TAAAGTTGGA ACTAGGAGTA AGAAAATCAA   
  
  
- AAAAATCAAA AATAGGAAAA CAATTTAACA AAAACGAAAA GTTAAAGTAA GAATATCTTA AATAAAATAT   
  
  
- AAAGTTAAAA GTAAGAAAAA AAATTAACGA TAAAAAATTA GTAAAACATA TTAACTTAAA AAAAAAAGTT   
  
  
- AAAGTAGGAA GTTATAAATT AACTAAACCT TAATTCAAAG TTCCAAAAAG GTCTATCCTA CAAAGATCAG   
  
  
- ATTATTGGAC CTAGTGTTCA AATTTTTCAA TTGTACCCAA CTATATAAAA AAAATATTTT TTTATCTGAA   
  
  
- ACACTAAAAG AAACAAAATA AAAGATAGAT TAATAGAACT ACAATATTGT ATCCGGCGTA CAAACCGCCC   
  
  
- TATTGAACTC AATCGAATTG GGACTAATGA TCCTAATATC CAAACAGTAT AATTGAACTC AACTGATTTG   
  
  
- GACTAAAAAA ATATTAAAAC AAATAGATTA AAATAAGAAA GTATAAATTA CCTGAATCTT AATTCAAAAC   
  
  
- AAGAAATAGT AGAAAATTTT TTTTACAAAA AAATGCAATA GTACTAAAAA AAAAATTAAA TATAGTAAAC   
  
  
- AATAACAATA GATAAATACT AGATAAATTT TATTTTAATT GAAGAAATTT AATCAATTTA GATAATAAAC   
  
  
- TTAGTGCTTT TTTTTATCAG TGGCGTACTG CTTCCAGGTT TTGATCGGTA CTCCATAAGG AGCTAATTAT   
  
  
- AGTTTCGGGT TAACAAATTC GTCATTCAGC CAATAGCCTT CAGTTAAACC TGTGCGAGAC AGAGAGAATA   
  
  
- GATTACATCT GTACTGCCAC AAGATTCGAA CATTCCGGAA CAGGAACCGA GGTACCTTGG AAGTTAAATA   
  
  
- GTCAGTTTAG ATGTCACACT TGCATATAAT AAGCTAAGAC ATAAATTAAA AACCATGGTG CGGAGAAATT   
  
  
- TTCTTTTATT TTCCAATGTT TTAAGTGTTT TGCAAAAAT

+     ERE

| Site Name | Organism | Position | Strand | Matrix score. | sequence | function |
| --- | --- | --- | --- | --- | --- | --- |
| ERE | Nicotiana glutinos | 8 | - | 8 | ATTTTAAA |  |
| ERE | Nicotiana glutinos | 1635 | - | 8 | ATTTTAAA |  |

>Potri.005G195000.1   
+ TCAACACTTT AAAATGATAT GAAAATACTA AAAATATATT AATTTAAATA AAAAAATTAA TATTTTTAAA   
  
  
+ ACATAACAGA TCTATTTGTA TTGTTCACAG GAAAAATGTA ATAATGTTTT TGTCTTTCTG AAAAAAAAAT   
  
  
+ ATTAAATTGG TTATTGGAGG CATGGAAGTT TTTTCATAAT GGGCACTAGT TGTTAACAAA TTAAATAGGG   
  
  
+ GCAAATAAGT CATTTCAAAA ATAGAGTGAG ATGACCTAAA TATCCTTATA AGAAAAGAAA TTAAAAGTGG   
  
  
+ TGTCCAAGAG CTTTATTGTA TTTTTCACAT GATTTTTTTA TTACTATAGG TCAACTAAGG GCCAATTTAG   
  
  
+ TATTTGTATA AATATATGGA AAAAAAAAGC ACAGTGAATG ATCAAAATAT TATTGGAAGC AAAAAAAAAT   
  
  
+ TAAAGTTAGC CTCGAGGCGA TGTTTTTGTA TTTTTACAAT GATTTTTTTT ATCACCAAGT GAACTTAGAG   
  
  
+ GGAATTCGGT ATTTTTCATA AATATAGAAA AATAAAAGTG GTTTGCGGGT GCATAGCAAG AGCCAACATG   
  
  
+ TGTGAGGTGT TTGGGCCTTC ATGTAACGCT TGCGACACCT CCCAACGTTC AAAATACCCT TTCTATCGTG   
  
  
+ TAATTGGAAG CGAAGCGATG TCCTTTTCCT TGCGGGGTGT TGCATGTGGT TGTTGGTGAT ACAGTTTGTT   
  
  
+ GCCGACAAAG CTTTCCCTCT ATATTTTCTC TCTCCTCCCT ACAAAATTTA CTCTGGCCAT GCAAAATTTT   
  
  
+ AGAATTGTCC TTTGATTTAT GGGGATTTCC ACTTAAGTCC TTATTTTTTA TTTCTAATTT TTATTTTTGG   
  
  
+ TCTTTTTGTA AAACTTTAAT TTTTTTTAAT TTCATCTTTA AATCTCAATT TCTAATATTT TTTATTTCAA   
  
  
+ CCTTAATCCT TATTCTTTTA ATTGTTTTTT TAATTTTTTT TGAGCCATTT GTTAAATTGA TATTTCTTAT   
  
  
+ CAATTTCACT CTTTAATAAA AAAATTCGTT TGCATTTCTT ATTTCAACCT TGATCCTCAT TCTTTTAGTT   
  
  
+ TTTTTAGTTT TTATCCTTTT GTTAAATTGT TTTTGCTTTT CAATTTCATT CTTATAGAAT TTATTTTATA   
  
  
+ TTTCAATTTT CATTCTTTTT TTTAATTGCT ATTTTTTAAT CATTTTGTAT AATTGAATTT TTTTTTTCAA   
  
  
+ TTTCATCCTT CAATATTTAA TTGATTTGGA ATTAAGTTTC AAGGTTTTTC CAGATAGGAT GTTTCTAGTC   
  
  
+ TAATAACCTG GATCACAAGT TTAAAAAGTT AACATGGGTT GATATATTTT TTTTATAAAA AAATAGACTT   
  
  
+ TGTGATTTTC TTTGTTTTAT TTTCTATCTA ATTATCTTGA TGTTATAACA TAGGCCGCAT GTTTGGCGGG   
  
  
+ ATAACTTGAG TTAGCTTAAC CCTGATTACT AGGATTATAG GTTTGTCATA TTAACTTGAG TTGACTAAAC   
  
  
+ CTGATTTTTT TATAATTTTG TTTATCTAAT TTTATTCTTT CATATTTAAT GGACTTAGAA TTAAGTTTTG   
  
  
+ TTCTTTATCA TCTTTTAAAA AAAATGTTTT TTTACGTTAT CATGATTTTT TTTTTAATTT ATATCATTTG   
  
  
+ TTATTGTTAT CTATTTATGA TCTATTTAAA ATAAAATTAA CTTCTTTAAA TTAGTTAAAT CTATTATTTG   
  
  
+ AATCACGAAA AAAAATAGTC ACCGCATGAC GAAGGTCCAA AACTAGCCAT GAGGTATTCC TCGATTAATA   
  
  
+ TCAAAGCCCA ATTGTTTAAG CAGTAAGTCG GTTATCGGAA GTCAATTTGG ACACGCTCTG TCTCTCTTAT   
  
  
+ CTAATGTAGA CATGACGGTG TTCTAAGCTT GTAAGGCCTT GTCCTTGGCT CCATGGAACC TTCAATTTAT   
  
  
+ CAGTCAAATC TACAGTGTGA ACGTATATTA TTCGATTCTG TATTTAATTT TTGGTACCAC GCCTCTTTAA   
  
  
+ AAGAAAATAA AAGGTTACAA AATTCACAAA ACGTTTTTA  

- AGTTGTGAAA TTTTACTATA CTTTTATGAT TTTTATATAA TTAAATTTAT TTTTTTAATT ATAAAAATTT   
  
  
- TGTATTGTCT AGATAAACAT AACAAGTGTC CTTTTTACAT TATTACAAAA ACAGAAAGAC TTTTTTTTTA   
  
  
- TAATTTAACC AATAACCTCC GTACCTTCAA AAAAGTATTA CCCGTGATCA ACAATTGTTT AATTTATCCC   
  
  
- CGTTTATTCA GTAAAGTTTT TATCTCACTC TACTGGATTT ATAGGAATAT TCTTTTCTTT AATTTTCACC   
  
  
- ACAGGTTCTC GAAATAACAT AAAAAGTGTA CTAAAAAAAT AATGATATCC AGTTGATTCC CGGTTAAATC   
  
  
- ATAAACATAT TTATATACCT TTTTTTTTCG TGTCACTTAC TAGTTTTATA ATAACCTTCG TTTTTTTTTA   
  
  
- ATTTCAATCG GAGCTCCGCT ACAAAAACAT AAAAATGTTA CTAAAAAAAA TAGTGGTTCA CTTGAATCTC   
  
  
- CCTTAAGCCA TAAAAAGTAT TTATATCTTT TTATTTTCAC CAAACGCCCA CGTATCGTTC TCGGTTGTAC   
  
  
- ACACTCCACA AACCCGGAAG TACATTGCGA ACGCTGTGGA GGGTTGCAAG TTTTATGGGA AAGATAGCAC   
  
  
- ATTAACCTTC GCTTCGCTAC AGGAAAAGGA ACGCCCCACA ACGTACACCA ACAACCACTA TGTCAAACAA   
  
  
- CGGCTGTTTC GAAAGGGAGA TATAAAAGAG AGAGGAGGGA TGTTTTAAAT GAGACCGGTA CGTTTTAAAA   
  
  
- TCTTAACAGG AAACTAAATA CCCCTAAAGG TGAATTCAGG AATAAAAAAT AAAGATTAAA AATAAAAACC   
  
  
- AGAAAAACAT TTTGAAATTA AAAAAAATTA AAGTAGAAAT TTAGAGTTAA AGATTATAAA AAATAAAGTT   
  
  
- GGAATTAGGA ATAAGAAAAT TAACAAAAAA ATTAAAAAAA ACTCGGTAAA CAATTTAACT ATAAAGAATA   
  
  
- GTTAAAGTGA GAAATTATTT TTTTAAGCAA ACGTAAAGAA TAAAGTTGGA ACTAGGAGTA AGAAAATCAA   
  
  
- AAAAATCAAA AATAGGAAAA CAATTTAACA AAAACGAAAA GTTAAAGTAA GAATATCTTA AATAAAATAT   
  
  
- AAAGTTAAAA GTAAGAAAAA AAATTAACGA TAAAAAATTA GTAAAACATA TTAACTTAAA AAAAAAAGTT   
  
  
- AAAGTAGGAA GTTATAAATT AACTAAACCT TAATTCAAAG TTCCAAAAAG GTCTATCCTA CAAAGATCAG   
  
  
- ATTATTGGAC CTAGTGTTCA AATTTTTCAA TTGTACCCAA CTATATAAAA AAAATATTTT TTTATCTGAA   
  
  
- ACACTAAAAG AAACAAAATA AAAGATAGAT TAATAGAACT ACAATATTGT ATCCGGCGTA CAAACCGCCC   
  
  
- TATTGAACTC AATCGAATTG GGACTAATGA TCCTAATATC CAAACAGTAT AATTGAACTC AACTGATTTG   
  
  
- GACTAAAAAA ATATTAAAAC AAATAGATTA AAATAAGAAA GTATAAATTA CCTGAATCTT AATTCAAAAC   
  
  
- AAGAAATAGT AGAAAATTTT TTTTACAAAA AAATGCAATA GTACTAAAAA AAAAATTAAA TATAGTAAAC   
  
  
- AATAACAATA GATAAATACT AGATAAATTT TATTTTAATT GAAGAAATTT AATCAATTTA GATAATAAAC   
  
  
- TTAGTGCTTT TTTTTATCAG TGGCGTACTG CTTCCAGGTT TTGATCGGTA CTCCATAAGG AGCTAATTAT   
  
  
- AGTTTCGGGT TAACAAATTC GTCATTCAGC CAATAGCCTT CAGTTAAACC TGTGCGAGAC AGAGAGAATA   
  
  
- GATTACATCT GTACTGCCAC AAGATTCGAA CATTCCGGAA CAGGAACCGA GGTACCTTGG AAGTTAAATA   
  
  
- GTCAGTTTAG ATGTCACACT TGCATATAAT AAGCTAAGAC ATAAATTAAA AACCATGGTG CGGAGAAATT   
  
  
- TTCTTTTATT TTCCAATGTT TTAAGTGTTT TGCAAAAAT

+     GA-motif

| Site Name | Organism | Position | Strand | Matrix score. | sequence | function |
| --- | --- | --- | --- | --- | --- | --- |
| GA-motif | Arabidopsis thaliana | 1617 | - | 8 | ATAGATAA | part of a light responsive element |

>Potri.005G195000.1   
+ TCAACACTTT AAAATGATAT GAAAATACTA AAAATATATT AATTTAAATA AAAAAATTAA TATTTTTAAA   
  
  
+ ACATAACAGA TCTATTTGTA TTGTTCACAG GAAAAATGTA ATAATGTTTT TGTCTTTCTG AAAAAAAAAT   
  
  
+ ATTAAATTGG TTATTGGAGG CATGGAAGTT TTTTCATAAT GGGCACTAGT TGTTAACAAA TTAAATAGGG   
  
  
+ GCAAATAAGT CATTTCAAAA ATAGAGTGAG ATGACCTAAA TATCCTTATA AGAAAAGAAA TTAAAAGTGG   
  
  
+ TGTCCAAGAG CTTTATTGTA TTTTTCACAT GATTTTTTTA TTACTATAGG TCAACTAAGG GCCAATTTAG   
  
  
+ TATTTGTATA AATATATGGA AAAAAAAAGC ACAGTGAATG ATCAAAATAT TATTGGAAGC AAAAAAAAAT   
  
  
+ TAAAGTTAGC CTCGAGGCGA TGTTTTTGTA TTTTTACAAT GATTTTTTTT ATCACCAAGT GAACTTAGAG   
  
  
+ GGAATTCGGT ATTTTTCATA AATATAGAAA AATAAAAGTG GTTTGCGGGT GCATAGCAAG AGCCAACATG   
  
  
+ TGTGAGGTGT TTGGGCCTTC ATGTAACGCT TGCGACACCT CCCAACGTTC AAAATACCCT TTCTATCGTG   
  
  
+ TAATTGGAAG CGAAGCGATG TCCTTTTCCT TGCGGGGTGT TGCATGTGGT TGTTGGTGAT ACAGTTTGTT   
  
  
+ GCCGACAAAG CTTTCCCTCT ATATTTTCTC TCTCCTCCCT ACAAAATTTA CTCTGGCCAT GCAAAATTTT   
  
  
+ AGAATTGTCC TTTGATTTAT GGGGATTTCC ACTTAAGTCC TTATTTTTTA TTTCTAATTT TTATTTTTGG   
  
  
+ TCTTTTTGTA AAACTTTAAT TTTTTTTAAT TTCATCTTTA AATCTCAATT TCTAATATTT TTTATTTCAA   
  
  
+ CCTTAATCCT TATTCTTTTA ATTGTTTTTT TAATTTTTTT TGAGCCATTT GTTAAATTGA TATTTCTTAT   
  
  
+ CAATTTCACT CTTTAATAAA AAAATTCGTT TGCATTTCTT ATTTCAACCT TGATCCTCAT TCTTTTAGTT   
  
  
+ TTTTTAGTTT TTATCCTTTT GTTAAATTGT TTTTGCTTTT CAATTTCATT CTTATAGAAT TTATTTTATA   
  
  
+ TTTCAATTTT CATTCTTTTT TTTAATTGCT ATTTTTTAAT CATTTTGTAT AATTGAATTT TTTTTTTCAA   
  
  
+ TTTCATCCTT CAATATTTAA TTGATTTGGA ATTAAGTTTC AAGGTTTTTC CAGATAGGAT GTTTCTAGTC   
  
  
+ TAATAACCTG GATCACAAGT TTAAAAAGTT AACATGGGTT GATATATTTT TTTTATAAAA AAATAGACTT   
  
  
+ TGTGATTTTC TTTGTTTTAT TTTCTATCTA ATTATCTTGA TGTTATAACA TAGGCCGCAT GTTTGGCGGG   
  
  
+ ATAACTTGAG TTAGCTTAAC CCTGATTACT AGGATTATAG GTTTGTCATA TTAACTTGAG TTGACTAAAC   
  
  
+ CTGATTTTTT TATAATTTTG TTTATCTAAT TTTATTCTTT CATATTTAAT GGACTTAGAA TTAAGTTTTG   
  
  
+ TTCTTTATCA TCTTTTAAAA AAAATGTTTT TTTACGTTAT CATGATTTTT TTTTTAATTT ATATCATTTG   
  
  
+ TTATTGTTAT CTATTTATGA TCTATTTAAA ATAAAATTAA CTTCTTTAAA TTAGTTAAAT CTATTATTTG   
  
  
+ AATCACGAAA AAAAATAGTC ACCGCATGAC GAAGGTCCAA AACTAGCCAT GAGGTATTCC TCGATTAATA   
  
  
+ TCAAAGCCCA ATTGTTTAAG CAGTAAGTCG GTTATCGGAA GTCAATTTGG ACACGCTCTG TCTCTCTTAT   
  
  
+ CTAATGTAGA CATGACGGTG TTCTAAGCTT GTAAGGCCTT GTCCTTGGCT CCATGGAACC TTCAATTTAT   
  
  
+ CAGTCAAATC TACAGTGTGA ACGTATATTA TTCGATTCTG TATTTAATTT TTGGTACCAC GCCTCTTTAA   
  
  
+ AAGAAAATAA AAGGTTACAA AATTCACAAA ACGTTTTTA  

- AGTTGTGAAA TTTTACTATA CTTTTATGAT TTTTATATAA TTAAATTTAT TTTTTTAATT ATAAAAATTT   
  
  
- TGTATTGTCT AGATAAACAT AACAAGTGTC CTTTTTACAT TATTACAAAA ACAGAAAGAC TTTTTTTTTA   
  
  
- TAATTTAACC AATAACCTCC GTACCTTCAA AAAAGTATTA CCCGTGATCA ACAATTGTTT AATTTATCCC   
  
  
- CGTTTATTCA GTAAAGTTTT TATCTCACTC TACTGGATTT ATAGGAATAT TCTTTTCTTT AATTTTCACC   
  
  
- ACAGGTTCTC GAAATAACAT AAAAAGTGTA CTAAAAAAAT AATGATATCC AGTTGATTCC CGGTTAAATC   
  
  
- ATAAACATAT TTATATACCT TTTTTTTTCG TGTCACTTAC TAGTTTTATA ATAACCTTCG TTTTTTTTTA   
  
  
- ATTTCAATCG GAGCTCCGCT ACAAAAACAT AAAAATGTTA CTAAAAAAAA TAGTGGTTCA CTTGAATCTC   
  
  
- CCTTAAGCCA TAAAAAGTAT TTATATCTTT TTATTTTCAC CAAACGCCCA CGTATCGTTC TCGGTTGTAC   
  
  
- ACACTCCACA AACCCGGAAG TACATTGCGA ACGCTGTGGA GGGTTGCAAG TTTTATGGGA AAGATAGCAC   
  
  
- ATTAACCTTC GCTTCGCTAC AGGAAAAGGA ACGCCCCACA ACGTACACCA ACAACCACTA TGTCAAACAA   
  
  
- CGGCTGTTTC GAAAGGGAGA TATAAAAGAG AGAGGAGGGA TGTTTTAAAT GAGACCGGTA CGTTTTAAAA   
  
  
- TCTTAACAGG AAACTAAATA CCCCTAAAGG TGAATTCAGG AATAAAAAAT AAAGATTAAA AATAAAAACC   
  
  
- AGAAAAACAT TTTGAAATTA AAAAAAATTA AAGTAGAAAT TTAGAGTTAA AGATTATAAA AAATAAAGTT   
  
  
- GGAATTAGGA ATAAGAAAAT TAACAAAAAA ATTAAAAAAA ACTCGGTAAA CAATTTAACT ATAAAGAATA   
  
  
- GTTAAAGTGA GAAATTATTT TTTTAAGCAA ACGTAAAGAA TAAAGTTGGA ACTAGGAGTA AGAAAATCAA   
  
  
- AAAAATCAAA AATAGGAAAA CAATTTAACA AAAACGAAAA GTTAAAGTAA GAATATCTTA AATAAAATAT   
  
  
- AAAGTTAAAA GTAAGAAAAA AAATTAACGA TAAAAAATTA GTAAAACATA TTAACTTAAA AAAAAAAGTT   
  
  
- AAAGTAGGAA GTTATAAATT AACTAAACCT TAATTCAAAG TTCCAAAAAG GTCTATCCTA CAAAGATCAG   
  
  
- ATTATTGGAC CTAGTGTTCA AATTTTTCAA TTGTACCCAA CTATATAAAA AAAATATTTT TTTATCTGAA   
  
  
- ACACTAAAAG AAACAAAATA AAAGATAGAT TAATAGAACT ACAATATTGT ATCCGGCGTA CAAACCGCCC   
  
  
- TATTGAACTC AATCGAATTG GGACTAATGA TCCTAATATC CAAACAGTAT AATTGAACTC AACTGATTTG   
  
  
- GACTAAAAAA ATATTAAAAC AAATAGATTA AAATAAGAAA GTATAAATTA CCTGAATCTT AATTCAAAAC   
  
  
- AAGAAATAGT AGAAAATTTT TTTTACAAAA AAATGCAATA GTACTAAAAA AAAAATTAAA TATAGTAAAC   
  
  
- AATAACAATA GATAAATACT AGATAAATTT TATTTTAATT GAAGAAATTT AATCAATTTA GATAATAAAC   
  
  
- TTAGTGCTTT TTTTTATCAG TGGCGTACTG CTTCCAGGTT TTGATCGGTA CTCCATAAGG AGCTAATTAT   
  
  
- AGTTTCGGGT TAACAAATTC GTCATTCAGC CAATAGCCTT CAGTTAAACC TGTGCGAGAC AGAGAGAATA   
  
  
- GATTACATCT GTACTGCCAC AAGATTCGAA CATTCCGGAA CAGGAACCGA GGTACCTTGG AAGTTAAATA   
  
  
- GTCAGTTTAG ATGTCACACT TGCATATAAT AAGCTAAGAC ATAAATTAAA AACCATGGTG CGGAGAAATT   
  
  
- TTCTTTTATT TTCCAATGTT TTAAGTGTTT TGCAAAAAT

+     GATA-motif

| Site Name | Organism | Position | Strand | Matrix score. | sequence | function |
| --- | --- | --- | --- | --- | --- | --- |
| GATA-motif | Arabidopsis thaliana | 1243 | + | 7 | GATAGGA | part of a light responsive element |
| GATA-motif | Solanum tuberosum | 1857 | - | 9 | AAGGATAAGG | part of a light responsive element |
| GATA-motif | Solanum tuberosum | 652 | - | 9 | AAGGATAAGG | part of a light responsive element |
| GATA-motif | Solanum tuberosum | 918 | - | 9 | AAGGATAAGG | part of a light responsive element |

>Potri.005G195000.1   
+ TCAACACTTT AAAATGATAT GAAAATACTA AAAATATATT AATTTAAATA AAAAAATTAA TATTTTTAAA   
  
  
+ ACATAACAGA TCTATTTGTA TTGTTCACAG GAAAAATGTA ATAATGTTTT TGTCTTTCTG AAAAAAAAAT   
  
  
+ ATTAAATTGG TTATTGGAGG CATGGAAGTT TTTTCATAAT GGGCACTAGT TGTTAACAAA TTAAATAGGG   
  
  
+ GCAAATAAGT CATTTCAAAA ATAGAGTGAG ATGACCTAAA TATCCTTATA AGAAAAGAAA TTAAAAGTGG   
  
  
+ TGTCCAAGAG CTTTATTGTA TTTTTCACAT GATTTTTTTA TTACTATAGG TCAACTAAGG GCCAATTTAG   
  
  
+ TATTTGTATA AATATATGGA AAAAAAAAGC ACAGTGAATG ATCAAAATAT TATTGGAAGC AAAAAAAAAT   
  
  
+ TAAAGTTAGC CTCGAGGCGA TGTTTTTGTA TTTTTACAAT GATTTTTTTT ATCACCAAGT GAACTTAGAG   
  
  
+ GGAATTCGGT ATTTTTCATA AATATAGAAA AATAAAAGTG GTTTGCGGGT GCATAGCAAG AGCCAACATG   
  
  
+ TGTGAGGTGT TTGGGCCTTC ATGTAACGCT TGCGACACCT CCCAACGTTC AAAATACCCT TTCTATCGTG   
  
  
+ TAATTGGAAG CGAAGCGATG TCCTTTTCCT TGCGGGGTGT TGCATGTGGT TGTTGGTGAT ACAGTTTGTT   
  
  
+ GCCGACAAAG CTTTCCCTCT ATATTTTCTC TCTCCTCCCT ACAAAATTTA CTCTGGCCAT GCAAAATTTT   
  
  
+ AGAATTGTCC TTTGATTTAT GGGGATTTCC ACTTAAGTCC TTATTTTTTA TTTCTAATTT TTATTTTTGG   
  
  
+ TCTTTTTGTA AAACTTTAAT TTTTTTTAAT TTCATCTTTA AATCTCAATT TCTAATATTT TTTATTTCAA   
  
  
+ CCTTAATCCT TATTCTTTTA ATTGTTTTTT TAATTTTTTT TGAGCCATTT GTTAAATTGA TATTTCTTAT   
  
  
+ CAATTTCACT CTTTAATAAA AAAATTCGTT TGCATTTCTT ATTTCAACCT TGATCCTCAT TCTTTTAGTT   
  
  
+ TTTTTAGTTT TTATCCTTTT GTTAAATTGT TTTTGCTTTT CAATTTCATT CTTATAGAAT TTATTTTATA   
  
  
+ TTTCAATTTT CATTCTTTTT TTTAATTGCT ATTTTTTAAT CATTTTGTAT AATTGAATTT TTTTTTTCAA   
  
  
+ TTTCATCCTT CAATATTTAA TTGATTTGGA ATTAAGTTTC AAGGTTTTTC CAGATAGGAT GTTTCTAGTC   
  
  
+ TAATAACCTG GATCACAAGT TTAAAAAGTT AACATGGGTT GATATATTTT TTTTATAAAA AAATAGACTT   
  
  
+ TGTGATTTTC TTTGTTTTAT TTTCTATCTA ATTATCTTGA TGTTATAACA TAGGCCGCAT GTTTGGCGGG   
  
  
+ ATAACTTGAG TTAGCTTAAC CCTGATTACT AGGATTATAG GTTTGTCATA TTAACTTGAG TTGACTAAAC   
  
  
+ CTGATTTTTT TATAATTTTG TTTATCTAAT TTTATTCTTT CATATTTAAT GGACTTAGAA TTAAGTTTTG   
  
  
+ TTCTTTATCA TCTTTTAAAA AAAATGTTTT TTTACGTTAT CATGATTTTT TTTTTAATTT ATATCATTTG   
  
  
+ TTATTGTTAT CTATTTATGA TCTATTTAAA ATAAAATTAA CTTCTTTAAA TTAGTTAAAT CTATTATTTG   
  
  
+ AATCACGAAA AAAAATAGTC ACCGCATGAC GAAGGTCCAA AACTAGCCAT GAGGTATTCC TCGATTAATA   
  
  
+ TCAAAGCCCA ATTGTTTAAG CAGTAAGTCG GTTATCGGAA GTCAATTTGG ACACGCTCTG TCTCTCTTAT   
  
  
+ CTAATGTAGA CATGACGGTG TTCTAAGCTT GTAAGGCCTT GTCCTTGGCT CCATGGAACC TTCAATTTAT   
  
  
+ CAGTCAAATC TACAGTGTGA ACGTATATTA TTCGATTCTG TATTTAATTT TTGGTACCAC GCCTCTTTAA   
  
  
+ AAGAAAATAA AAGGTTACAA AATTCACAAA ACGTTTTTA  

- AGTTGTGAAA TTTTACTATA CTTTTATGAT TTTTATATAA TTAAATTTAT TTTTTTAATT ATAAAAATTT   
  
  
- TGTATTGTCT AGATAAACAT AACAAGTGTC CTTTTTACAT TATTACAAAA ACAGAAAGAC TTTTTTTTTA   
  
  
- TAATTTAACC AATAACCTCC GTACCTTCAA AAAAGTATTA CCCGTGATCA ACAATTGTTT AATTTATCCC   
  
  
- CGTTTATTCA GTAAAGTTTT TATCTCACTC TACTGGATTT ATAGGAATAT TCTTTTCTTT AATTTTCACC   
  
  
- ACAGGTTCTC GAAATAACAT AAAAAGTGTA CTAAAAAAAT AATGATATCC AGTTGATTCC CGGTTAAATC   
  
  
- ATAAACATAT TTATATACCT TTTTTTTTCG TGTCACTTAC TAGTTTTATA ATAACCTTCG TTTTTTTTTA   
  
  
- ATTTCAATCG GAGCTCCGCT ACAAAAACAT AAAAATGTTA CTAAAAAAAA TAGTGGTTCA CTTGAATCTC   
  
  
- CCTTAAGCCA TAAAAAGTAT TTATATCTTT TTATTTTCAC CAAACGCCCA CGTATCGTTC TCGGTTGTAC   
  
  
- ACACTCCACA AACCCGGAAG TACATTGCGA ACGCTGTGGA GGGTTGCAAG TTTTATGGGA AAGATAGCAC   
  
  
- ATTAACCTTC GCTTCGCTAC AGGAAAAGGA ACGCCCCACA ACGTACACCA ACAACCACTA TGTCAAACAA   
  
  
- CGGCTGTTTC GAAAGGGAGA TATAAAAGAG AGAGGAGGGA TGTTTTAAAT GAGACCGGTA CGTTTTAAAA   
  
  
- TCTTAACAGG AAACTAAATA CCCCTAAAGG TGAATTCAGG AATAAAAAAT AAAGATTAAA AATAAAAACC   
  
  
- AGAAAAACAT TTTGAAATTA AAAAAAATTA AAGTAGAAAT TTAGAGTTAA AGATTATAAA AAATAAAGTT   
  
  
- GGAATTAGGA ATAAGAAAAT TAACAAAAAA ATTAAAAAAA ACTCGGTAAA CAATTTAACT ATAAAGAATA   
  
  
- GTTAAAGTGA GAAATTATTT TTTTAAGCAA ACGTAAAGAA TAAAGTTGGA ACTAGGAGTA AGAAAATCAA   
  
  
- AAAAATCAAA AATAGGAAAA CAATTTAACA AAAACGAAAA GTTAAAGTAA GAATATCTTA AATAAAATAT   
  
  
- AAAGTTAAAA GTAAGAAAAA AAATTAACGA TAAAAAATTA GTAAAACATA TTAACTTAAA AAAAAAAGTT   
  
  
- AAAGTAGGAA GTTATAAATT AACTAAACCT TAATTCAAAG TTCCAAAAAG GTCTATCCTA CAAAGATCAG   
  
  
- ATTATTGGAC CTAGTGTTCA AATTTTTCAA TTGTACCCAA CTATATAAAA AAAATATTTT TTTATCTGAA   
  
  
- ACACTAAAAG AAACAAAATA AAAGATAGAT TAATAGAACT ACAATATTGT ATCCGGCGTA CAAACCGCCC   
  
  
- TATTGAACTC AATCGAATTG GGACTAATGA TCCTAATATC CAAACAGTAT AATTGAACTC AACTGATTTG   
  
  
- GACTAAAAAA ATATTAAAAC AAATAGATTA AAATAAGAAA GTATAAATTA CCTGAATCTT AATTCAAAAC   
  
  
- AAGAAATAGT AGAAAATTTT TTTTACAAAA AAATGCAATA GTACTAAAAA AAAAATTAAA TATAGTAAAC   
  
  
- AATAACAATA GATAAATACT AGATAAATTT TATTTTAATT GAAGAAATTT AATCAATTTA GATAATAAAC   
  
  
- TTAGTGCTTT TTTTTATCAG TGGCGTACTG CTTCCAGGTT TTGATCGGTA CTCCATAAGG AGCTAATTAT   
  
  
- AGTTTCGGGT TAACAAATTC GTCATTCAGC CAATAGCCTT CAGTTAAACC TGTGCGAGAC AGAGAGAATA   
  
  
- GATTACATCT GTACTGCCAC AAGATTCGAA CATTCCGGAA CAGGAACCGA GGTACCTTGG AAGTTAAATA   
  
  
- GTCAGTTTAG ATGTCACACT TGCATATAAT AAGCTAAGAC ATAAATTAAA AACCATGGTG CGGAGAAATT   
  
  
- TTCTTTTATT TTCCAATGTT TTAAGTGTTT TGCAAAAAT

+     GT1-motif

| Site Name | Organism | Position | Strand | Matrix score. | sequence | function |
| --- | --- | --- | --- | --- | --- | --- |
| GT1-motif | Arabidopsis thaliana | 1416 | - | 6 | GGTTAA | light responsive element |

>Potri.005G195000.1   
+ TCAACACTTT AAAATGATAT GAAAATACTA AAAATATATT AATTTAAATA AAAAAATTAA TATTTTTAAA   
  
  
+ ACATAACAGA TCTATTTGTA TTGTTCACAG GAAAAATGTA ATAATGTTTT TGTCTTTCTG AAAAAAAAAT   
  
  
+ ATTAAATTGG TTATTGGAGG CATGGAAGTT TTTTCATAAT GGGCACTAGT TGTTAACAAA TTAAATAGGG   
  
  
+ GCAAATAAGT CATTTCAAAA ATAGAGTGAG ATGACCTAAA TATCCTTATA AGAAAAGAAA TTAAAAGTGG   
  
  
+ TGTCCAAGAG CTTTATTGTA TTTTTCACAT GATTTTTTTA TTACTATAGG TCAACTAAGG GCCAATTTAG   
  
  
+ TATTTGTATA AATATATGGA AAAAAAAAGC ACAGTGAATG ATCAAAATAT TATTGGAAGC AAAAAAAAAT   
  
  
+ TAAAGTTAGC CTCGAGGCGA TGTTTTTGTA TTTTTACAAT GATTTTTTTT ATCACCAAGT GAACTTAGAG   
  
  
+ GGAATTCGGT ATTTTTCATA AATATAGAAA AATAAAAGTG GTTTGCGGGT GCATAGCAAG AGCCAACATG   
  
  
+ TGTGAGGTGT TTGGGCCTTC ATGTAACGCT TGCGACACCT CCCAACGTTC AAAATACCCT TTCTATCGTG   
  
  
+ TAATTGGAAG CGAAGCGATG TCCTTTTCCT TGCGGGGTGT TGCATGTGGT TGTTGGTGAT ACAGTTTGTT   
  
  
+ GCCGACAAAG CTTTCCCTCT ATATTTTCTC TCTCCTCCCT ACAAAATTTA CTCTGGCCAT GCAAAATTTT   
  
  
+ AGAATTGTCC TTTGATTTAT GGGGATTTCC ACTTAAGTCC TTATTTTTTA TTTCTAATTT TTATTTTTGG   
  
  
+ TCTTTTTGTA AAACTTTAAT TTTTTTTAAT TTCATCTTTA AATCTCAATT TCTAATATTT TTTATTTCAA   
  
  
+ CCTTAATCCT TATTCTTTTA ATTGTTTTTT TAATTTTTTT TGAGCCATTT GTTAAATTGA TATTTCTTAT   
  
  
+ CAATTTCACT CTTTAATAAA AAAATTCGTT TGCATTTCTT ATTTCAACCT TGATCCTCAT TCTTTTAGTT   
  
  
+ TTTTTAGTTT TTATCCTTTT GTTAAATTGT TTTTGCTTTT CAATTTCATT CTTATAGAAT TTATTTTATA   
  
  
+ TTTCAATTTT CATTCTTTTT TTTAATTGCT ATTTTTTAAT CATTTTGTAT AATTGAATTT TTTTTTTCAA   
  
  
+ TTTCATCCTT CAATATTTAA TTGATTTGGA ATTAAGTTTC AAGGTTTTTC CAGATAGGAT GTTTCTAGTC   
  
  
+ TAATAACCTG GATCACAAGT TTAAAAAGTT AACATGGGTT GATATATTTT TTTTATAAAA AAATAGACTT   
  
  
+ TGTGATTTTC TTTGTTTTAT TTTCTATCTA ATTATCTTGA TGTTATAACA TAGGCCGCAT GTTTGGCGGG   
  
  
+ ATAACTTGAG TTAGCTTAAC CCTGATTACT AGGATTATAG GTTTGTCATA TTAACTTGAG TTGACTAAAC   
  
  
+ CTGATTTTTT TATAATTTTG TTTATCTAAT TTTATTCTTT CATATTTAAT GGACTTAGAA TTAAGTTTTG   
  
  
+ TTCTTTATCA TCTTTTAAAA AAAATGTTTT TTTACGTTAT CATGATTTTT TTTTTAATTT ATATCATTTG   
  
  
+ TTATTGTTAT CTATTTATGA TCTATTTAAA ATAAAATTAA CTTCTTTAAA TTAGTTAAAT CTATTATTTG   
  
  
+ AATCACGAAA AAAAATAGTC ACCGCATGAC GAAGGTCCAA AACTAGCCAT GAGGTATTCC TCGATTAATA   
  
  
+ TCAAAGCCCA ATTGTTTAAG CAGTAAGTCG GTTATCGGAA GTCAATTTGG ACACGCTCTG TCTCTCTTAT   
  
  
+ CTAATGTAGA CATGACGGTG TTCTAAGCTT GTAAGGCCTT GTCCTTGGCT CCATGGAACC TTCAATTTAT   
  
  
+ CAGTCAAATC TACAGTGTGA ACGTATATTA TTCGATTCTG TATTTAATTT TTGGTACCAC GCCTCTTTAA   
  
  
+ AAGAAAATAA AAGGTTACAA AATTCACAAA ACGTTTTTA  

- AGTTGTGAAA TTTTACTATA CTTTTATGAT TTTTATATAA TTAAATTTAT TTTTTTAATT ATAAAAATTT   
  
  
- TGTATTGTCT AGATAAACAT AACAAGTGTC CTTTTTACAT TATTACAAAA ACAGAAAGAC TTTTTTTTTA   
  
  
- TAATTTAACC AATAACCTCC GTACCTTCAA AAAAGTATTA CCCGTGATCA ACAATTGTTT AATTTATCCC   
  
  
- CGTTTATTCA GTAAAGTTTT TATCTCACTC TACTGGATTT ATAGGAATAT TCTTTTCTTT AATTTTCACC   
  
  
- ACAGGTTCTC GAAATAACAT AAAAAGTGTA CTAAAAAAAT AATGATATCC AGTTGATTCC CGGTTAAATC   
  
  
- ATAAACATAT TTATATACCT TTTTTTTTCG TGTCACTTAC TAGTTTTATA ATAACCTTCG TTTTTTTTTA   
  
  
- ATTTCAATCG GAGCTCCGCT ACAAAAACAT AAAAATGTTA CTAAAAAAAA TAGTGGTTCA CTTGAATCTC   
  
  
- CCTTAAGCCA TAAAAAGTAT TTATATCTTT TTATTTTCAC CAAACGCCCA CGTATCGTTC TCGGTTGTAC   
  
  
- ACACTCCACA AACCCGGAAG TACATTGCGA ACGCTGTGGA GGGTTGCAAG TTTTATGGGA AAGATAGCAC   
  
  
- ATTAACCTTC GCTTCGCTAC AGGAAAAGGA ACGCCCCACA ACGTACACCA ACAACCACTA TGTCAAACAA   
  
  
- CGGCTGTTTC GAAAGGGAGA TATAAAAGAG AGAGGAGGGA TGTTTTAAAT GAGACCGGTA CGTTTTAAAA   
  
  
- TCTTAACAGG AAACTAAATA CCCCTAAAGG TGAATTCAGG AATAAAAAAT AAAGATTAAA AATAAAAACC   
  
  
- AGAAAAACAT TTTGAAATTA AAAAAAATTA AAGTAGAAAT TTAGAGTTAA AGATTATAAA AAATAAAGTT   
  
  
- GGAATTAGGA ATAAGAAAAT TAACAAAAAA ATTAAAAAAA ACTCGGTAAA CAATTTAACT ATAAAGAATA   
  
  
- GTTAAAGTGA GAAATTATTT TTTTAAGCAA ACGTAAAGAA TAAAGTTGGA ACTAGGAGTA AGAAAATCAA   
  
  
- AAAAATCAAA AATAGGAAAA CAATTTAACA AAAACGAAAA GTTAAAGTAA GAATATCTTA AATAAAATAT   
  
  
- AAAGTTAAAA GTAAGAAAAA AAATTAACGA TAAAAAATTA GTAAAACATA TTAACTTAAA AAAAAAAGTT   
  
  
- AAAGTAGGAA GTTATAAATT AACTAAACCT TAATTCAAAG TTCCAAAAAG GTCTATCCTA CAAAGATCAG   
  
  
- ATTATTGGAC CTAGTGTTCA AATTTTTCAA TTGTACCCAA CTATATAAAA AAAATATTTT TTTATCTGAA   
  
  
- ACACTAAAAG AAACAAAATA AAAGATAGAT TAATAGAACT ACAATATTGT ATCCGGCGTA CAAACCGCCC   
  
  
- TATTGAACTC AATCGAATTG GGACTAATGA TCCTAATATC CAAACAGTAT AATTGAACTC AACTGATTTG   
  
  
- GACTAAAAAA ATATTAAAAC AAATAGATTA AAATAAGAAA GTATAAATTA CCTGAATCTT AATTCAAAAC   
  
  
- AAGAAATAGT AGAAAATTTT TTTTACAAAA AAATGCAATA GTACTAAAAA AAAAATTAAA TATAGTAAAC   
  
  
- AATAACAATA GATAAATACT AGATAAATTT TATTTTAATT GAAGAAATTT AATCAATTTA GATAATAAAC   
  
  
- TTAGTGCTTT TTTTTATCAG TGGCGTACTG CTTCCAGGTT TTGATCGGTA CTCCATAAGG AGCTAATTAT   
  
  
- AGTTTCGGGT TAACAAATTC GTCATTCAGC CAATAGCCTT CAGTTAAACC TGTGCGAGAC AGAGAGAATA   
  
  
- GATTACATCT GTACTGCCAC AAGATTCGAA CATTCCGGAA CAGGAACCGA GGTACCTTGG AAGTTAAATA   
  
  
- GTCAGTTTAG ATGTCACACT TGCATATAAT AAGCTAAGAC ATAAATTAAA AACCATGGTG CGGAGAAATT   
  
  
- TTCTTTTATT TTCCAATGTT TTAAGTGTTT TGCAAAAAT

+     LAMP-element

| Site Name | Organism | Position | Strand | Matrix score. | sequence | function |
| --- | --- | --- | --- | --- | --- | --- |
| LAMP-element | Pisum sativum | 1543 | + | 8 | CTTTATCA | part of a light responsive element |

>Potri.005G195000.1   
+ TCAACACTTT AAAATGATAT GAAAATACTA AAAATATATT AATTTAAATA AAAAAATTAA TATTTTTAAA   
  
  
+ ACATAACAGA TCTATTTGTA TTGTTCACAG GAAAAATGTA ATAATGTTTT TGTCTTTCTG AAAAAAAAAT   
  
  
+ ATTAAATTGG TTATTGGAGG CATGGAAGTT TTTTCATAAT GGGCACTAGT TGTTAACAAA TTAAATAGGG   
  
  
+ GCAAATAAGT CATTTCAAAA ATAGAGTGAG ATGACCTAAA TATCCTTATA AGAAAAGAAA TTAAAAGTGG   
  
  
+ TGTCCAAGAG CTTTATTGTA TTTTTCACAT GATTTTTTTA TTACTATAGG TCAACTAAGG GCCAATTTAG   
  
  
+ TATTTGTATA AATATATGGA AAAAAAAAGC ACAGTGAATG ATCAAAATAT TATTGGAAGC AAAAAAAAAT   
  
  
+ TAAAGTTAGC CTCGAGGCGA TGTTTTTGTA TTTTTACAAT GATTTTTTTT ATCACCAAGT GAACTTAGAG   
  
  
+ GGAATTCGGT ATTTTTCATA AATATAGAAA AATAAAAGTG GTTTGCGGGT GCATAGCAAG AGCCAACATG   
  
  
+ TGTGAGGTGT TTGGGCCTTC ATGTAACGCT TGCGACACCT CCCAACGTTC AAAATACCCT TTCTATCGTG   
  
  
+ TAATTGGAAG CGAAGCGATG TCCTTTTCCT TGCGGGGTGT TGCATGTGGT TGTTGGTGAT ACAGTTTGTT   
  
  
+ GCCGACAAAG CTTTCCCTCT ATATTTTCTC TCTCCTCCCT ACAAAATTTA CTCTGGCCAT GCAAAATTTT   
  
  
+ AGAATTGTCC TTTGATTTAT GGGGATTTCC ACTTAAGTCC TTATTTTTTA TTTCTAATTT TTATTTTTGG   
  
  
+ TCTTTTTGTA AAACTTTAAT TTTTTTTAAT TTCATCTTTA AATCTCAATT TCTAATATTT TTTATTTCAA   
  
  
+ CCTTAATCCT TATTCTTTTA ATTGTTTTTT TAATTTTTTT TGAGCCATTT GTTAAATTGA TATTTCTTAT   
  
  
+ CAATTTCACT CTTTAATAAA AAAATTCGTT TGCATTTCTT ATTTCAACCT TGATCCTCAT TCTTTTAGTT   
  
  
+ TTTTTAGTTT TTATCCTTTT GTTAAATTGT TTTTGCTTTT CAATTTCATT CTTATAGAAT TTATTTTATA   
  
  
+ TTTCAATTTT CATTCTTTTT TTTAATTGCT ATTTTTTAAT CATTTTGTAT AATTGAATTT TTTTTTTCAA   
  
  
+ TTTCATCCTT CAATATTTAA TTGATTTGGA ATTAAGTTTC AAGGTTTTTC CAGATAGGAT GTTTCTAGTC   
  
  
+ TAATAACCTG GATCACAAGT TTAAAAAGTT AACATGGGTT GATATATTTT TTTTATAAAA AAATAGACTT   
  
  
+ TGTGATTTTC TTTGTTTTAT TTTCTATCTA ATTATCTTGA TGTTATAACA TAGGCCGCAT GTTTGGCGGG   
  
  
+ ATAACTTGAG TTAGCTTAAC CCTGATTACT AGGATTATAG GTTTGTCATA TTAACTTGAG TTGACTAAAC   
  
  
+ CTGATTTTTT TATAATTTTG TTTATCTAAT TTTATTCTTT CATATTTAAT GGACTTAGAA TTAAGTTTTG   
  
  
+ TTCTTTATCA TCTTTTAAAA AAAATGTTTT TTTACGTTAT CATGATTTTT TTTTTAATTT ATATCATTTG   
  
  
+ TTATTGTTAT CTATTTATGA TCTATTTAAA ATAAAATTAA CTTCTTTAAA TTAGTTAAAT CTATTATTTG   
  
  
+ AATCACGAAA AAAAATAGTC ACCGCATGAC GAAGGTCCAA AACTAGCCAT GAGGTATTCC TCGATTAATA   
  
  
+ TCAAAGCCCA ATTGTTTAAG CAGTAAGTCG GTTATCGGAA GTCAATTTGG ACACGCTCTG TCTCTCTTAT   
  
  
+ CTAATGTAGA CATGACGGTG TTCTAAGCTT GTAAGGCCTT GTCCTTGGCT CCATGGAACC TTCAATTTAT   
  
  
+ CAGTCAAATC TACAGTGTGA ACGTATATTA TTCGATTCTG TATTTAATTT TTGGTACCAC GCCTCTTTAA   
  
  
+ AAGAAAATAA AAGGTTACAA AATTCACAAA ACGTTTTTA  

- AGTTGTGAAA TTTTACTATA CTTTTATGAT TTTTATATAA TTAAATTTAT TTTTTTAATT ATAAAAATTT   
  
  
- TGTATTGTCT AGATAAACAT AACAAGTGTC CTTTTTACAT TATTACAAAA ACAGAAAGAC TTTTTTTTTA   
  
  
- TAATTTAACC AATAACCTCC GTACCTTCAA AAAAGTATTA CCCGTGATCA ACAATTGTTT AATTTATCCC   
  
  
- CGTTTATTCA GTAAAGTTTT TATCTCACTC TACTGGATTT ATAGGAATAT TCTTTTCTTT AATTTTCACC   
  
  
- ACAGGTTCTC GAAATAACAT AAAAAGTGTA CTAAAAAAAT AATGATATCC AGTTGATTCC CGGTTAAATC   
  
  
- ATAAACATAT TTATATACCT TTTTTTTTCG TGTCACTTAC TAGTTTTATA ATAACCTTCG TTTTTTTTTA   
  
  
- ATTTCAATCG GAGCTCCGCT ACAAAAACAT AAAAATGTTA CTAAAAAAAA TAGTGGTTCA CTTGAATCTC   
  
  
- CCTTAAGCCA TAAAAAGTAT TTATATCTTT TTATTTTCAC CAAACGCCCA CGTATCGTTC TCGGTTGTAC   
  
  
- ACACTCCACA AACCCGGAAG TACATTGCGA ACGCTGTGGA GGGTTGCAAG TTTTATGGGA AAGATAGCAC   
  
  
- ATTAACCTTC GCTTCGCTAC AGGAAAAGGA ACGCCCCACA ACGTACACCA ACAACCACTA TGTCAAACAA   
  
  
- CGGCTGTTTC GAAAGGGAGA TATAAAAGAG AGAGGAGGGA TGTTTTAAAT GAGACCGGTA CGTTTTAAAA   
  
  
- TCTTAACAGG AAACTAAATA CCCCTAAAGG TGAATTCAGG AATAAAAAAT AAAGATTAAA AATAAAAACC   
  
  
- AGAAAAACAT TTTGAAATTA AAAAAAATTA AAGTAGAAAT TTAGAGTTAA AGATTATAAA AAATAAAGTT   
  
  
- GGAATTAGGA ATAAGAAAAT TAACAAAAAA ATTAAAAAAA ACTCGGTAAA CAATTTAACT ATAAAGAATA   
  
  
- GTTAAAGTGA GAAATTATTT TTTTAAGCAA ACGTAAAGAA TAAAGTTGGA ACTAGGAGTA AGAAAATCAA   
  
  
- AAAAATCAAA AATAGGAAAA CAATTTAACA AAAACGAAAA GTTAAAGTAA GAATATCTTA AATAAAATAT   
  
  
- AAAGTTAAAA GTAAGAAAAA AAATTAACGA TAAAAAATTA GTAAAACATA TTAACTTAAA AAAAAAAGTT   
  
  
- AAAGTAGGAA GTTATAAATT AACTAAACCT TAATTCAAAG TTCCAAAAAG GTCTATCCTA CAAAGATCAG   
  
  
- ATTATTGGAC CTAGTGTTCA AATTTTTCAA TTGTACCCAA CTATATAAAA AAAATATTTT TTTATCTGAA   
  
  
- ACACTAAAAG AAACAAAATA AAAGATAGAT TAATAGAACT ACAATATTGT ATCCGGCGTA CAAACCGCCC   
  
  
- TATTGAACTC AATCGAATTG GGACTAATGA TCCTAATATC CAAACAGTAT AATTGAACTC AACTGATTTG   
  
  
- GACTAAAAAA ATATTAAAAC AAATAGATTA AAATAAGAAA GTATAAATTA CCTGAATCTT AATTCAAAAC   
  
  
- AAGAAATAGT AGAAAATTTT TTTTACAAAA AAATGCAATA GTACTAAAAA AAAAATTAAA TATAGTAAAC   
  
  
- AATAACAATA GATAAATACT AGATAAATTT TATTTTAATT GAAGAAATTT AATCAATTTA GATAATAAAC   
  
  
- TTAGTGCTTT TTTTTATCAG TGGCGTACTG CTTCCAGGTT TTGATCGGTA CTCCATAAGG AGCTAATTAT   
  
  
- AGTTTCGGGT TAACAAATTC GTCATTCAGC CAATAGCCTT CAGTTAAACC TGTGCGAGAC AGAGAGAATA   
  
  
- GATTACATCT GTACTGCCAC AAGATTCGAA CATTCCGGAA CAGGAACCGA GGTACCTTGG AAGTTAAATA   
  
  
- GTCAGTTTAG ATGTCACACT TGCATATAAT AAGCTAAGAC ATAAATTAAA AACCATGGTG CGGAGAAATT   
  
  
- TTCTTTTATT TTCCAATGTT TTAAGTGTTT TGCAAAAAT

+     MYB

| Site Name | Organism | Position | Strand | Matrix score. | sequence | function |
| --- | --- | --- | --- | --- | --- | --- |
| MYB | Arabidopsis thaliana | 677 | - | 6 | CAACCA |  |
| MYB | Arabidopsis thaliana | 148 | - | 6 | TAACCA |  |

>Potri.005G195000.1   
+ TCAACACTTT AAAATGATAT GAAAATACTA AAAATATATT AATTTAAATA AAAAAATTAA TATTTTTAAA   
  
  
+ ACATAACAGA TCTATTTGTA TTGTTCACAG GAAAAATGTA ATAATGTTTT TGTCTTTCTG AAAAAAAAAT   
  
  
+ ATTAAATTGG TTATTGGAGG CATGGAAGTT TTTTCATAAT GGGCACTAGT TGTTAACAAA TTAAATAGGG   
  
  
+ GCAAATAAGT CATTTCAAAA ATAGAGTGAG ATGACCTAAA TATCCTTATA AGAAAAGAAA TTAAAAGTGG   
  
  
+ TGTCCAAGAG CTTTATTGTA TTTTTCACAT GATTTTTTTA TTACTATAGG TCAACTAAGG GCCAATTTAG   
  
  
+ TATTTGTATA AATATATGGA AAAAAAAAGC ACAGTGAATG ATCAAAATAT TATTGGAAGC AAAAAAAAAT   
  
  
+ TAAAGTTAGC CTCGAGGCGA TGTTTTTGTA TTTTTACAAT GATTTTTTTT ATCACCAAGT GAACTTAGAG   
  
  
+ GGAATTCGGT ATTTTTCATA AATATAGAAA AATAAAAGTG GTTTGCGGGT GCATAGCAAG AGCCAACATG   
  
  
+ TGTGAGGTGT TTGGGCCTTC ATGTAACGCT TGCGACACCT CCCAACGTTC AAAATACCCT TTCTATCGTG   
  
  
+ TAATTGGAAG CGAAGCGATG TCCTTTTCCT TGCGGGGTGT TGCATGTGGT TGTTGGTGAT ACAGTTTGTT   
  
  
+ GCCGACAAAG CTTTCCCTCT ATATTTTCTC TCTCCTCCCT ACAAAATTTA CTCTGGCCAT GCAAAATTTT   
  
  
+ AGAATTGTCC TTTGATTTAT GGGGATTTCC ACTTAAGTCC TTATTTTTTA TTTCTAATTT TTATTTTTGG   
  
  
+ TCTTTTTGTA AAACTTTAAT TTTTTTTAAT TTCATCTTTA AATCTCAATT TCTAATATTT TTTATTTCAA   
  
  
+ CCTTAATCCT TATTCTTTTA ATTGTTTTTT TAATTTTTTT TGAGCCATTT GTTAAATTGA TATTTCTTAT   
  
  
+ CAATTTCACT CTTTAATAAA AAAATTCGTT TGCATTTCTT ATTTCAACCT TGATCCTCAT TCTTTTAGTT   
  
  
+ TTTTTAGTTT TTATCCTTTT GTTAAATTGT TTTTGCTTTT CAATTTCATT CTTATAGAAT TTATTTTATA   
  
  
+ TTTCAATTTT CATTCTTTTT TTTAATTGCT ATTTTTTAAT CATTTTGTAT AATTGAATTT TTTTTTTCAA   
  
  
+ TTTCATCCTT CAATATTTAA TTGATTTGGA ATTAAGTTTC AAGGTTTTTC CAGATAGGAT GTTTCTAGTC   
  
  
+ TAATAACCTG GATCACAAGT TTAAAAAGTT AACATGGGTT GATATATTTT TTTTATAAAA AAATAGACTT   
  
  
+ TGTGATTTTC TTTGTTTTAT TTTCTATCTA ATTATCTTGA TGTTATAACA TAGGCCGCAT GTTTGGCGGG   
  
  
+ ATAACTTGAG TTAGCTTAAC CCTGATTACT AGGATTATAG GTTTGTCATA TTAACTTGAG TTGACTAAAC   
  
  
+ CTGATTTTTT TATAATTTTG TTTATCTAAT TTTATTCTTT CATATTTAAT GGACTTAGAA TTAAGTTTTG   
  
  
+ TTCTTTATCA TCTTTTAAAA AAAATGTTTT TTTACGTTAT CATGATTTTT TTTTTAATTT ATATCATTTG   
  
  
+ TTATTGTTAT CTATTTATGA TCTATTTAAA ATAAAATTAA CTTCTTTAAA TTAGTTAAAT CTATTATTTG   
  
  
+ AATCACGAAA AAAAATAGTC ACCGCATGAC GAAGGTCCAA AACTAGCCAT GAGGTATTCC TCGATTAATA   
  
  
+ TCAAAGCCCA ATTGTTTAAG CAGTAAGTCG GTTATCGGAA GTCAATTTGG ACACGCTCTG TCTCTCTTAT   
  
  
+ CTAATGTAGA CATGACGGTG TTCTAAGCTT GTAAGGCCTT GTCCTTGGCT CCATGGAACC TTCAATTTAT   
  
  
+ CAGTCAAATC TACAGTGTGA ACGTATATTA TTCGATTCTG TATTTAATTT TTGGTACCAC GCCTCTTTAA   
  
  
+ AAGAAAATAA AAGGTTACAA AATTCACAAA ACGTTTTTA  

- AGTTGTGAAA TTTTACTATA CTTTTATGAT TTTTATATAA TTAAATTTAT TTTTTTAATT ATAAAAATTT   
  
  
- TGTATTGTCT AGATAAACAT AACAAGTGTC CTTTTTACAT TATTACAAAA ACAGAAAGAC TTTTTTTTTA   
  
  
- TAATTTAACC AATAACCTCC GTACCTTCAA AAAAGTATTA CCCGTGATCA ACAATTGTTT AATTTATCCC   
  
  
- CGTTTATTCA GTAAAGTTTT TATCTCACTC TACTGGATTT ATAGGAATAT TCTTTTCTTT AATTTTCACC   
  
  
- ACAGGTTCTC GAAATAACAT AAAAAGTGTA CTAAAAAAAT AATGATATCC AGTTGATTCC CGGTTAAATC   
  
  
- ATAAACATAT TTATATACCT TTTTTTTTCG TGTCACTTAC TAGTTTTATA ATAACCTTCG TTTTTTTTTA   
  
  
- ATTTCAATCG GAGCTCCGCT ACAAAAACAT AAAAATGTTA CTAAAAAAAA TAGTGGTTCA CTTGAATCTC   
  
  
- CCTTAAGCCA TAAAAAGTAT TTATATCTTT TTATTTTCAC CAAACGCCCA CGTATCGTTC TCGGTTGTAC   
  
  
- ACACTCCACA AACCCGGAAG TACATTGCGA ACGCTGTGGA GGGTTGCAAG TTTTATGGGA AAGATAGCAC   
  
  
- ATTAACCTTC GCTTCGCTAC AGGAAAAGGA ACGCCCCACA ACGTACACCA ACAACCACTA TGTCAAACAA   
  
  
- CGGCTGTTTC GAAAGGGAGA TATAAAAGAG AGAGGAGGGA TGTTTTAAAT GAGACCGGTA CGTTTTAAAA   
  
  
- TCTTAACAGG AAACTAAATA CCCCTAAAGG TGAATTCAGG AATAAAAAAT AAAGATTAAA AATAAAAACC   
  
  
- AGAAAAACAT TTTGAAATTA AAAAAAATTA AAGTAGAAAT TTAGAGTTAA AGATTATAAA AAATAAAGTT   
  
  
- GGAATTAGGA ATAAGAAAAT TAACAAAAAA ATTAAAAAAA ACTCGGTAAA CAATTTAACT ATAAAGAATA   
  
  
- GTTAAAGTGA GAAATTATTT TTTTAAGCAA ACGTAAAGAA TAAAGTTGGA ACTAGGAGTA AGAAAATCAA   
  
  
- AAAAATCAAA AATAGGAAAA CAATTTAACA AAAACGAAAA GTTAAAGTAA GAATATCTTA AATAAAATAT   
  
  
- AAAGTTAAAA GTAAGAAAAA AAATTAACGA TAAAAAATTA GTAAAACATA TTAACTTAAA AAAAAAAGTT   
  
  
- AAAGTAGGAA GTTATAAATT AACTAAACCT TAATTCAAAG TTCCAAAAAG GTCTATCCTA CAAAGATCAG   
  
  
- ATTATTGGAC CTAGTGTTCA AATTTTTCAA TTGTACCCAA CTATATAAAA AAAATATTTT TTTATCTGAA   
  
  
- ACACTAAAAG AAACAAAATA AAAGATAGAT TAATAGAACT ACAATATTGT ATCCGGCGTA CAAACCGCCC   
  
  
- TATTGAACTC AATCGAATTG GGACTAATGA TCCTAATATC CAAACAGTAT AATTGAACTC AACTGATTTG   
  
  
- GACTAAAAAA ATATTAAAAC AAATAGATTA AAATAAGAAA GTATAAATTA CCTGAATCTT AATTCAAAAC   
  
  
- AAGAAATAGT AGAAAATTTT TTTTACAAAA AAATGCAATA GTACTAAAAA AAAAATTAAA TATAGTAAAC   
  
  
- AATAACAATA GATAAATACT AGATAAATTT TATTTTAATT GAAGAAATTT AATCAATTTA GATAATAAAC   
  
  
- TTAGTGCTTT TTTTTATCAG TGGCGTACTG CTTCCAGGTT TTGATCGGTA CTCCATAAGG AGCTAATTAT   
  
  
- AGTTTCGGGT TAACAAATTC GTCATTCAGC CAATAGCCTT CAGTTAAACC TGTGCGAGAC AGAGAGAATA   
  
  
- GATTACATCT GTACTGCCAC AAGATTCGAA CATTCCGGAA CAGGAACCGA GGTACCTTGG AAGTTAAATA   
  
  
- GTCAGTTTAG ATGTCACACT TGCATATAAT AAGCTAAGAC ATAAATTAAA AACCATGGTG CGGAGAAATT   
  
  
- TTCTTTTATT TTCCAATGTT TTAAGTGTTT TGCAAAAAT

+     MYB-like sequence

| Site Name | Organism | Position | Strand | Matrix score. | sequence | function |
| --- | --- | --- | --- | --- | --- | --- |
| MYB-like sequence | Arabidopsis thaliana | 148 | - | 6 | TAACCA |  |

>Potri.005G195000.1   
+ TCAACACTTT AAAATGATAT GAAAATACTA AAAATATATT AATTTAAATA AAAAAATTAA TATTTTTAAA   
  
  
+ ACATAACAGA TCTATTTGTA TTGTTCACAG GAAAAATGTA ATAATGTTTT TGTCTTTCTG AAAAAAAAAT   
  
  
+ ATTAAATTGG TTATTGGAGG CATGGAAGTT TTTTCATAAT GGGCACTAGT TGTTAACAAA TTAAATAGGG   
  
  
+ GCAAATAAGT CATTTCAAAA ATAGAGTGAG ATGACCTAAA TATCCTTATA AGAAAAGAAA TTAAAAGTGG   
  
  
+ TGTCCAAGAG CTTTATTGTA TTTTTCACAT GATTTTTTTA TTACTATAGG TCAACTAAGG GCCAATTTAG   
  
  
+ TATTTGTATA AATATATGGA AAAAAAAAGC ACAGTGAATG ATCAAAATAT TATTGGAAGC AAAAAAAAAT   
  
  
+ TAAAGTTAGC CTCGAGGCGA TGTTTTTGTA TTTTTACAAT GATTTTTTTT ATCACCAAGT GAACTTAGAG   
  
  
+ GGAATTCGGT ATTTTTCATA AATATAGAAA AATAAAAGTG GTTTGCGGGT GCATAGCAAG AGCCAACATG   
  
  
+ TGTGAGGTGT TTGGGCCTTC ATGTAACGCT TGCGACACCT CCCAACGTTC AAAATACCCT TTCTATCGTG   
  
  
+ TAATTGGAAG CGAAGCGATG TCCTTTTCCT TGCGGGGTGT TGCATGTGGT TGTTGGTGAT ACAGTTTGTT   
  
  
+ GCCGACAAAG CTTTCCCTCT ATATTTTCTC TCTCCTCCCT ACAAAATTTA CTCTGGCCAT GCAAAATTTT   
  
  
+ AGAATTGTCC TTTGATTTAT GGGGATTTCC ACTTAAGTCC TTATTTTTTA TTTCTAATTT TTATTTTTGG   
  
  
+ TCTTTTTGTA AAACTTTAAT TTTTTTTAAT TTCATCTTTA AATCTCAATT TCTAATATTT TTTATTTCAA   
  
  
+ CCTTAATCCT TATTCTTTTA ATTGTTTTTT TAATTTTTTT TGAGCCATTT GTTAAATTGA TATTTCTTAT   
  
  
+ CAATTTCACT CTTTAATAAA AAAATTCGTT TGCATTTCTT ATTTCAACCT TGATCCTCAT TCTTTTAGTT   
  
  
+ TTTTTAGTTT TTATCCTTTT GTTAAATTGT TTTTGCTTTT CAATTTCATT CTTATAGAAT TTATTTTATA   
  
  
+ TTTCAATTTT CATTCTTTTT TTTAATTGCT ATTTTTTAAT CATTTTGTAT AATTGAATTT TTTTTTTCAA   
  
  
+ TTTCATCCTT CAATATTTAA TTGATTTGGA ATTAAGTTTC AAGGTTTTTC CAGATAGGAT GTTTCTAGTC   
  
  
+ TAATAACCTG GATCACAAGT TTAAAAAGTT AACATGGGTT GATATATTTT TTTTATAAAA AAATAGACTT   
  
  
+ TGTGATTTTC TTTGTTTTAT TTTCTATCTA ATTATCTTGA TGTTATAACA TAGGCCGCAT GTTTGGCGGG   
  
  
+ ATAACTTGAG TTAGCTTAAC CCTGATTACT AGGATTATAG GTTTGTCATA TTAACTTGAG TTGACTAAAC   
  
  
+ CTGATTTTTT TATAATTTTG TTTATCTAAT TTTATTCTTT CATATTTAAT GGACTTAGAA TTAAGTTTTG   
  
  
+ TTCTTTATCA TCTTTTAAAA AAAATGTTTT TTTACGTTAT CATGATTTTT TTTTTAATTT ATATCATTTG   
  
  
+ TTATTGTTAT CTATTTATGA TCTATTTAAA ATAAAATTAA CTTCTTTAAA TTAGTTAAAT CTATTATTTG   
  
  
+ AATCACGAAA AAAAATAGTC ACCGCATGAC GAAGGTCCAA AACTAGCCAT GAGGTATTCC TCGATTAATA   
  
  
+ TCAAAGCCCA ATTGTTTAAG CAGTAAGTCG GTTATCGGAA GTCAATTTGG ACACGCTCTG TCTCTCTTAT   
  
  
+ CTAATGTAGA CATGACGGTG TTCTAAGCTT GTAAGGCCTT GTCCTTGGCT CCATGGAACC TTCAATTTAT   
  
  
+ CAGTCAAATC TACAGTGTGA ACGTATATTA TTCGATTCTG TATTTAATTT TTGGTACCAC GCCTCTTTAA   
  
  
+ AAGAAAATAA AAGGTTACAA AATTCACAAA ACGTTTTTA  

- AGTTGTGAAA TTTTACTATA CTTTTATGAT TTTTATATAA TTAAATTTAT TTTTTTAATT ATAAAAATTT   
  
  
- TGTATTGTCT AGATAAACAT AACAAGTGTC CTTTTTACAT TATTACAAAA ACAGAAAGAC TTTTTTTTTA   
  
  
- TAATTTAACC AATAACCTCC GTACCTTCAA AAAAGTATTA CCCGTGATCA ACAATTGTTT AATTTATCCC   
  
  
- CGTTTATTCA GTAAAGTTTT TATCTCACTC TACTGGATTT ATAGGAATAT TCTTTTCTTT AATTTTCACC   
  
  
- ACAGGTTCTC GAAATAACAT AAAAAGTGTA CTAAAAAAAT AATGATATCC AGTTGATTCC CGGTTAAATC   
  
  
- ATAAACATAT TTATATACCT TTTTTTTTCG TGTCACTTAC TAGTTTTATA ATAACCTTCG TTTTTTTTTA   
  
  
- ATTTCAATCG GAGCTCCGCT ACAAAAACAT AAAAATGTTA CTAAAAAAAA TAGTGGTTCA CTTGAATCTC   
  
  
- CCTTAAGCCA TAAAAAGTAT TTATATCTTT TTATTTTCAC CAAACGCCCA CGTATCGTTC TCGGTTGTAC   
  
  
- ACACTCCACA AACCCGGAAG TACATTGCGA ACGCTGTGGA GGGTTGCAAG TTTTATGGGA AAGATAGCAC   
  
  
- ATTAACCTTC GCTTCGCTAC AGGAAAAGGA ACGCCCCACA ACGTACACCA ACAACCACTA TGTCAAACAA   
  
  
- CGGCTGTTTC GAAAGGGAGA TATAAAAGAG AGAGGAGGGA TGTTTTAAAT GAGACCGGTA CGTTTTAAAA   
  
  
- TCTTAACAGG AAACTAAATA CCCCTAAAGG TGAATTCAGG AATAAAAAAT AAAGATTAAA AATAAAAACC   
  
  
- AGAAAAACAT TTTGAAATTA AAAAAAATTA AAGTAGAAAT TTAGAGTTAA AGATTATAAA AAATAAAGTT   
  
  
- GGAATTAGGA ATAAGAAAAT TAACAAAAAA ATTAAAAAAA ACTCGGTAAA CAATTTAACT ATAAAGAATA   
  
  
- GTTAAAGTGA GAAATTATTT TTTTAAGCAA ACGTAAAGAA TAAAGTTGGA ACTAGGAGTA AGAAAATCAA   
  
  
- AAAAATCAAA AATAGGAAAA CAATTTAACA AAAACGAAAA GTTAAAGTAA GAATATCTTA AATAAAATAT   
  
  
- AAAGTTAAAA GTAAGAAAAA AAATTAACGA TAAAAAATTA GTAAAACATA TTAACTTAAA AAAAAAAGTT   
  
  
- AAAGTAGGAA GTTATAAATT AACTAAACCT TAATTCAAAG TTCCAAAAAG GTCTATCCTA CAAAGATCAG   
  
  
- ATTATTGGAC CTAGTGTTCA AATTTTTCAA TTGTACCCAA CTATATAAAA AAAATATTTT TTTATCTGAA   
  
  
- ACACTAAAAG AAACAAAATA AAAGATAGAT TAATAGAACT ACAATATTGT ATCCGGCGTA CAAACCGCCC   
  
  
- TATTGAACTC AATCGAATTG GGACTAATGA TCCTAATATC CAAACAGTAT AATTGAACTC AACTGATTTG   
  
  
- GACTAAAAAA ATATTAAAAC AAATAGATTA AAATAAGAAA GTATAAATTA CCTGAATCTT AATTCAAAAC   
  
  
- AAGAAATAGT AGAAAATTTT TTTTACAAAA AAATGCAATA GTACTAAAAA AAAAATTAAA TATAGTAAAC   
  
  
- AATAACAATA GATAAATACT AGATAAATTT TATTTTAATT GAAGAAATTT AATCAATTTA GATAATAAAC   
  
  
- TTAGTGCTTT TTTTTATCAG TGGCGTACTG CTTCCAGGTT TTGATCGGTA CTCCATAAGG AGCTAATTAT   
  
  
- AGTTTCGGGT TAACAAATTC GTCATTCAGC CAATAGCCTT CAGTTAAACC TGTGCGAGAC AGAGAGAATA   
  
  
- GATTACATCT GTACTGCCAC AAGATTCGAA CATTCCGGAA CAGGAACCGA GGTACCTTGG AAGTTAAATA   
  
  
- GTCAGTTTAG ATGTCACACT TGCATATAAT AAGCTAAGAC ATAAATTAAA AACCATGGTG CGGAGAAATT   
  
  
- TTCTTTTATT TTCCAATGTT TTAAGTGTTT TGCAAAAAT

+     MYC

| Site Name | Organism | Position | Strand | Matrix score. | sequence | function |
| --- | --- | --- | --- | --- | --- | --- |
| MYC | Arabidopsis thaliana | 1759 | - | 6 | CAATTG |  |
| MYC | Arabidopsis thaliana | 1605 | + | 6 | CATTTG |  |
| MYC | Arabidopsis thaliana | 673 | + | 6 | CATGTG |  |
| MYC | Arabidopsis thaliana | 956 | + | 6 | CATTTG |  |
| MYC | Arabidopsis thaliana | 557 | + | 6 | CATGTG |  |
| MYC | Arabidopsis thaliana | 306 | - | 6 | CATGTG |  |

>Potri.005G195000.1   
+ TCAACACTTT AAAATGATAT GAAAATACTA AAAATATATT AATTTAAATA AAAAAATTAA TATTTTTAAA   
  
  
+ ACATAACAGA TCTATTTGTA TTGTTCACAG GAAAAATGTA ATAATGTTTT TGTCTTTCTG AAAAAAAAAT   
  
  
+ ATTAAATTGG TTATTGGAGG CATGGAAGTT TTTTCATAAT GGGCACTAGT TGTTAACAAA TTAAATAGGG   
  
  
+ GCAAATAAGT CATTTCAAAA ATAGAGTGAG ATGACCTAAA TATCCTTATA AGAAAAGAAA TTAAAAGTGG   
  
  
+ TGTCCAAGAG CTTTATTGTA TTTTTCACAT GATTTTTTTA TTACTATAGG TCAACTAAGG GCCAATTTAG   
  
  
+ TATTTGTATA AATATATGGA AAAAAAAAGC ACAGTGAATG ATCAAAATAT TATTGGAAGC AAAAAAAAAT   
  
  
+ TAAAGTTAGC CTCGAGGCGA TGTTTTTGTA TTTTTACAAT GATTTTTTTT ATCACCAAGT GAACTTAGAG   
  
  
+ GGAATTCGGT ATTTTTCATA AATATAGAAA AATAAAAGTG GTTTGCGGGT GCATAGCAAG AGCCAACATG   
  
  
+ TGTGAGGTGT TTGGGCCTTC ATGTAACGCT TGCGACACCT CCCAACGTTC AAAATACCCT TTCTATCGTG   
  
  
+ TAATTGGAAG CGAAGCGATG TCCTTTTCCT TGCGGGGTGT TGCATGTGGT TGTTGGTGAT ACAGTTTGTT   
  
  
+ GCCGACAAAG CTTTCCCTCT ATATTTTCTC TCTCCTCCCT ACAAAATTTA CTCTGGCCAT GCAAAATTTT   
  
  
+ AGAATTGTCC TTTGATTTAT GGGGATTTCC ACTTAAGTCC TTATTTTTTA TTTCTAATTT TTATTTTTGG   
  
  
+ TCTTTTTGTA AAACTTTAAT TTTTTTTAAT TTCATCTTTA AATCTCAATT TCTAATATTT TTTATTTCAA   
  
  
+ CCTTAATCCT TATTCTTTTA ATTGTTTTTT TAATTTTTTT TGAGCCATTT GTTAAATTGA TATTTCTTAT   
  
  
+ CAATTTCACT CTTTAATAAA AAAATTCGTT TGCATTTCTT ATTTCAACCT TGATCCTCAT TCTTTTAGTT   
  
  
+ TTTTTAGTTT TTATCCTTTT GTTAAATTGT TTTTGCTTTT CAATTTCATT CTTATAGAAT TTATTTTATA   
  
  
+ TTTCAATTTT CATTCTTTTT TTTAATTGCT ATTTTTTAAT CATTTTGTAT AATTGAATTT TTTTTTTCAA   
  
  
+ TTTCATCCTT CAATATTTAA TTGATTTGGA ATTAAGTTTC AAGGTTTTTC CAGATAGGAT GTTTCTAGTC   
  
  
+ TAATAACCTG GATCACAAGT TTAAAAAGTT AACATGGGTT GATATATTTT TTTTATAAAA AAATAGACTT   
  
  
+ TGTGATTTTC TTTGTTTTAT TTTCTATCTA ATTATCTTGA TGTTATAACA TAGGCCGCAT GTTTGGCGGG   
  
  
+ ATAACTTGAG TTAGCTTAAC CCTGATTACT AGGATTATAG GTTTGTCATA TTAACTTGAG TTGACTAAAC   
  
  
+ CTGATTTTTT TATAATTTTG TTTATCTAAT TTTATTCTTT CATATTTAAT GGACTTAGAA TTAAGTTTTG   
  
  
+ TTCTTTATCA TCTTTTAAAA AAAATGTTTT TTTACGTTAT CATGATTTTT TTTTTAATTT ATATCATTTG   
  
  
+ TTATTGTTAT CTATTTATGA TCTATTTAAA ATAAAATTAA CTTCTTTAAA TTAGTTAAAT CTATTATTTG   
  
  
+ AATCACGAAA AAAAATAGTC ACCGCATGAC GAAGGTCCAA AACTAGCCAT GAGGTATTCC TCGATTAATA   
  
  
+ TCAAAGCCCA ATTGTTTAAG CAGTAAGTCG GTTATCGGAA GTCAATTTGG ACACGCTCTG TCTCTCTTAT   
  
  
+ CTAATGTAGA CATGACGGTG TTCTAAGCTT GTAAGGCCTT GTCCTTGGCT CCATGGAACC TTCAATTTAT   
  
  
+ CAGTCAAATC TACAGTGTGA ACGTATATTA TTCGATTCTG TATTTAATTT TTGGTACCAC GCCTCTTTAA   
  
  
+ AAGAAAATAA AAGGTTACAA AATTCACAAA ACGTTTTTA  

- AGTTGTGAAA TTTTACTATA CTTTTATGAT TTTTATATAA TTAAATTTAT TTTTTTAATT ATAAAAATTT   
  
  
- TGTATTGTCT AGATAAACAT AACAAGTGTC CTTTTTACAT TATTACAAAA ACAGAAAGAC TTTTTTTTTA   
  
  
- TAATTTAACC AATAACCTCC GTACCTTCAA AAAAGTATTA CCCGTGATCA ACAATTGTTT AATTTATCCC   
  
  
- CGTTTATTCA GTAAAGTTTT TATCTCACTC TACTGGATTT ATAGGAATAT TCTTTTCTTT AATTTTCACC   
  
  
- ACAGGTTCTC GAAATAACAT AAAAAGTGTA CTAAAAAAAT AATGATATCC AGTTGATTCC CGGTTAAATC   
  
  
- ATAAACATAT TTATATACCT TTTTTTTTCG TGTCACTTAC TAGTTTTATA ATAACCTTCG TTTTTTTTTA   
  
  
- ATTTCAATCG GAGCTCCGCT ACAAAAACAT AAAAATGTTA CTAAAAAAAA TAGTGGTTCA CTTGAATCTC   
  
  
- CCTTAAGCCA TAAAAAGTAT TTATATCTTT TTATTTTCAC CAAACGCCCA CGTATCGTTC TCGGTTGTAC   
  
  
- ACACTCCACA AACCCGGAAG TACATTGCGA ACGCTGTGGA GGGTTGCAAG TTTTATGGGA AAGATAGCAC   
  
  
- ATTAACCTTC GCTTCGCTAC AGGAAAAGGA ACGCCCCACA ACGTACACCA ACAACCACTA TGTCAAACAA   
  
  
- CGGCTGTTTC GAAAGGGAGA TATAAAAGAG AGAGGAGGGA TGTTTTAAAT GAGACCGGTA CGTTTTAAAA   
  
  
- TCTTAACAGG AAACTAAATA CCCCTAAAGG TGAATTCAGG AATAAAAAAT AAAGATTAAA AATAAAAACC   
  
  
- AGAAAAACAT TTTGAAATTA AAAAAAATTA AAGTAGAAAT TTAGAGTTAA AGATTATAAA AAATAAAGTT   
  
  
- GGAATTAGGA ATAAGAAAAT TAACAAAAAA ATTAAAAAAA ACTCGGTAAA CAATTTAACT ATAAAGAATA   
  
  
- GTTAAAGTGA GAAATTATTT TTTTAAGCAA ACGTAAAGAA TAAAGTTGGA ACTAGGAGTA AGAAAATCAA   
  
  
- AAAAATCAAA AATAGGAAAA CAATTTAACA AAAACGAAAA GTTAAAGTAA GAATATCTTA AATAAAATAT   
  
  
- AAAGTTAAAA GTAAGAAAAA AAATTAACGA TAAAAAATTA GTAAAACATA TTAACTTAAA AAAAAAAGTT   
  
  
- AAAGTAGGAA GTTATAAATT AACTAAACCT TAATTCAAAG TTCCAAAAAG GTCTATCCTA CAAAGATCAG   
  
  
- ATTATTGGAC CTAGTGTTCA AATTTTTCAA TTGTACCCAA CTATATAAAA AAAATATTTT TTTATCTGAA   
  
  
- ACACTAAAAG AAACAAAATA AAAGATAGAT TAATAGAACT ACAATATTGT ATCCGGCGTA CAAACCGCCC   
  
  
- TATTGAACTC AATCGAATTG GGACTAATGA TCCTAATATC CAAACAGTAT AATTGAACTC AACTGATTTG   
  
  
- GACTAAAAAA ATATTAAAAC AAATAGATTA AAATAAGAAA GTATAAATTA CCTGAATCTT AATTCAAAAC   
  
  
- AAGAAATAGT AGAAAATTTT TTTTACAAAA AAATGCAATA GTACTAAAAA AAAAATTAAA TATAGTAAAC   
  
  
- AATAACAATA GATAAATACT AGATAAATTT TATTTTAATT GAAGAAATTT AATCAATTTA GATAATAAAC   
  
  
- TTAGTGCTTT TTTTTATCAG TGGCGTACTG CTTCCAGGTT TTGATCGGTA CTCCATAAGG AGCTAATTAT   
  
  
- AGTTTCGGGT TAACAAATTC GTCATTCAGC CAATAGCCTT CAGTTAAACC TGTGCGAGAC AGAGAGAATA   
  
  
- GATTACATCT GTACTGCCAC AAGATTCGAA CATTCCGGAA CAGGAACCGA GGTACCTTGG AAGTTAAATA   
  
  
- GTCAGTTTAG ATGTCACACT TGCATATAAT AAGCTAAGAC ATAAATTAAA AACCATGGTG CGGAGAAATT   
  
  
- TTCTTTTATT TTCCAATGTT TTAAGTGTTT TGCAAAAAT

+     Myc

| Site Name | Organism | Position | Strand | Matrix score. | sequence | function |
| --- | --- | --- | --- | --- | --- | --- |
| Myc | Arabidopsis thaliana | 1813 | + | 7 | TCTCTTA |  |

>Potri.005G195000.1   
+ TCAACACTTT AAAATGATAT GAAAATACTA AAAATATATT AATTTAAATA AAAAAATTAA TATTTTTAAA   
  
  
+ ACATAACAGA TCTATTTGTA TTGTTCACAG GAAAAATGTA ATAATGTTTT TGTCTTTCTG AAAAAAAAAT   
  
  
+ ATTAAATTGG TTATTGGAGG CATGGAAGTT TTTTCATAAT GGGCACTAGT TGTTAACAAA TTAAATAGGG   
  
  
+ GCAAATAAGT CATTTCAAAA ATAGAGTGAG ATGACCTAAA TATCCTTATA AGAAAAGAAA TTAAAAGTGG   
  
  
+ TGTCCAAGAG CTTTATTGTA TTTTTCACAT GATTTTTTTA TTACTATAGG TCAACTAAGG GCCAATTTAG   
  
  
+ TATTTGTATA AATATATGGA AAAAAAAAGC ACAGTGAATG ATCAAAATAT TATTGGAAGC AAAAAAAAAT   
  
  
+ TAAAGTTAGC CTCGAGGCGA TGTTTTTGTA TTTTTACAAT GATTTTTTTT ATCACCAAGT GAACTTAGAG   
  
  
+ GGAATTCGGT ATTTTTCATA AATATAGAAA AATAAAAGTG GTTTGCGGGT GCATAGCAAG AGCCAACATG   
  
  
+ TGTGAGGTGT TTGGGCCTTC ATGTAACGCT TGCGACACCT CCCAACGTTC AAAATACCCT TTCTATCGTG   
  
  
+ TAATTGGAAG CGAAGCGATG TCCTTTTCCT TGCGGGGTGT TGCATGTGGT TGTTGGTGAT ACAGTTTGTT   
  
  
+ GCCGACAAAG CTTTCCCTCT ATATTTTCTC TCTCCTCCCT ACAAAATTTA CTCTGGCCAT GCAAAATTTT   
  
  
+ AGAATTGTCC TTTGATTTAT GGGGATTTCC ACTTAAGTCC TTATTTTTTA TTTCTAATTT TTATTTTTGG   
  
  
+ TCTTTTTGTA AAACTTTAAT TTTTTTTAAT TTCATCTTTA AATCTCAATT TCTAATATTT TTTATTTCAA   
  
  
+ CCTTAATCCT TATTCTTTTA ATTGTTTTTT TAATTTTTTT TGAGCCATTT GTTAAATTGA TATTTCTTAT   
  
  
+ CAATTTCACT CTTTAATAAA AAAATTCGTT TGCATTTCTT ATTTCAACCT TGATCCTCAT TCTTTTAGTT   
  
  
+ TTTTTAGTTT TTATCCTTTT GTTAAATTGT TTTTGCTTTT CAATTTCATT CTTATAGAAT TTATTTTATA   
  
  
+ TTTCAATTTT CATTCTTTTT TTTAATTGCT ATTTTTTAAT CATTTTGTAT AATTGAATTT TTTTTTTCAA   
  
  
+ TTTCATCCTT CAATATTTAA TTGATTTGGA ATTAAGTTTC AAGGTTTTTC CAGATAGGAT GTTTCTAGTC   
  
  
+ TAATAACCTG GATCACAAGT TTAAAAAGTT AACATGGGTT GATATATTTT TTTTATAAAA AAATAGACTT   
  
  
+ TGTGATTTTC TTTGTTTTAT TTTCTATCTA ATTATCTTGA TGTTATAACA TAGGCCGCAT GTTTGGCGGG   
  
  
+ ATAACTTGAG TTAGCTTAAC CCTGATTACT AGGATTATAG GTTTGTCATA TTAACTTGAG TTGACTAAAC   
  
  
+ CTGATTTTTT TATAATTTTG TTTATCTAAT TTTATTCTTT CATATTTAAT GGACTTAGAA TTAAGTTTTG   
  
  
+ TTCTTTATCA TCTTTTAAAA AAAATGTTTT TTTACGTTAT CATGATTTTT TTTTTAATTT ATATCATTTG   
  
  
+ TTATTGTTAT CTATTTATGA TCTATTTAAA ATAAAATTAA CTTCTTTAAA TTAGTTAAAT CTATTATTTG   
  
  
+ AATCACGAAA AAAAATAGTC ACCGCATGAC GAAGGTCCAA AACTAGCCAT GAGGTATTCC TCGATTAATA   
  
  
+ TCAAAGCCCA ATTGTTTAAG CAGTAAGTCG GTTATCGGAA GTCAATTTGG ACACGCTCTG TCTCTCTTAT   
  
  
+ CTAATGTAGA CATGACGGTG TTCTAAGCTT GTAAGGCCTT GTCCTTGGCT CCATGGAACC TTCAATTTAT   
  
  
+ CAGTCAAATC TACAGTGTGA ACGTATATTA TTCGATTCTG TATTTAATTT TTGGTACCAC GCCTCTTTAA   
  
  
+ AAGAAAATAA AAGGTTACAA AATTCACAAA ACGTTTTTA  

- AGTTGTGAAA TTTTACTATA CTTTTATGAT TTTTATATAA TTAAATTTAT TTTTTTAATT ATAAAAATTT   
  
  
- TGTATTGTCT AGATAAACAT AACAAGTGTC CTTTTTACAT TATTACAAAA ACAGAAAGAC TTTTTTTTTA   
  
  
- TAATTTAACC AATAACCTCC GTACCTTCAA AAAAGTATTA CCCGTGATCA ACAATTGTTT AATTTATCCC   
  
  
- CGTTTATTCA GTAAAGTTTT TATCTCACTC TACTGGATTT ATAGGAATAT TCTTTTCTTT AATTTTCACC   
  
  
- ACAGGTTCTC GAAATAACAT AAAAAGTGTA CTAAAAAAAT AATGATATCC AGTTGATTCC CGGTTAAATC   
  
  
- ATAAACATAT TTATATACCT TTTTTTTTCG TGTCACTTAC TAGTTTTATA ATAACCTTCG TTTTTTTTTA   
  
  
- ATTTCAATCG GAGCTCCGCT ACAAAAACAT AAAAATGTTA CTAAAAAAAA TAGTGGTTCA CTTGAATCTC   
  
  
- CCTTAAGCCA TAAAAAGTAT TTATATCTTT TTATTTTCAC CAAACGCCCA CGTATCGTTC TCGGTTGTAC   
  
  
- ACACTCCACA AACCCGGAAG TACATTGCGA ACGCTGTGGA GGGTTGCAAG TTTTATGGGA AAGATAGCAC   
  
  
- ATTAACCTTC GCTTCGCTAC AGGAAAAGGA ACGCCCCACA ACGTACACCA ACAACCACTA TGTCAAACAA   
  
  
- CGGCTGTTTC GAAAGGGAGA TATAAAAGAG AGAGGAGGGA TGTTTTAAAT GAGACCGGTA CGTTTTAAAA   
  
  
- TCTTAACAGG AAACTAAATA CCCCTAAAGG TGAATTCAGG AATAAAAAAT AAAGATTAAA AATAAAAACC   
  
  
- AGAAAAACAT TTTGAAATTA AAAAAAATTA AAGTAGAAAT TTAGAGTTAA AGATTATAAA AAATAAAGTT   
  
  
- GGAATTAGGA ATAAGAAAAT TAACAAAAAA ATTAAAAAAA ACTCGGTAAA CAATTTAACT ATAAAGAATA   
  
  
- GTTAAAGTGA GAAATTATTT TTTTAAGCAA ACGTAAAGAA TAAAGTTGGA ACTAGGAGTA AGAAAATCAA   
  
  
- AAAAATCAAA AATAGGAAAA CAATTTAACA AAAACGAAAA GTTAAAGTAA GAATATCTTA AATAAAATAT   
  
  
- AAAGTTAAAA GTAAGAAAAA AAATTAACGA TAAAAAATTA GTAAAACATA TTAACTTAAA AAAAAAAGTT   
  
  
- AAAGTAGGAA GTTATAAATT AACTAAACCT TAATTCAAAG TTCCAAAAAG GTCTATCCTA CAAAGATCAG   
  
  
- ATTATTGGAC CTAGTGTTCA AATTTTTCAA TTGTACCCAA CTATATAAAA AAAATATTTT TTTATCTGAA   
  
  
- ACACTAAAAG AAACAAAATA AAAGATAGAT TAATAGAACT ACAATATTGT ATCCGGCGTA CAAACCGCCC   
  
  
- TATTGAACTC AATCGAATTG GGACTAATGA TCCTAATATC CAAACAGTAT AATTGAACTC AACTGATTTG   
  
  
- GACTAAAAAA ATATTAAAAC AAATAGATTA AAATAAGAAA GTATAAATTA CCTGAATCTT AATTCAAAAC   
  
  
- AAGAAATAGT AGAAAATTTT TTTTACAAAA AAATGCAATA GTACTAAAAA AAAAATTAAA TATAGTAAAC   
  
  
- AATAACAATA GATAAATACT AGATAAATTT TATTTTAATT GAAGAAATTT AATCAATTTA GATAATAAAC   
  
  
- TTAGTGCTTT TTTTTATCAG TGGCGTACTG CTTCCAGGTT TTGATCGGTA CTCCATAAGG AGCTAATTAT   
  
  
- AGTTTCGGGT TAACAAATTC GTCATTCAGC CAATAGCCTT CAGTTAAACC TGTGCGAGAC AGAGAGAATA   
  
  
- GATTACATCT GTACTGCCAC AAGATTCGAA CATTCCGGAA CAGGAACCGA GGTACCTTGG AAGTTAAATA   
  
  
- GTCAGTTTAG ATGTCACACT TGCATATAAT AAGCTAAGAC ATAAATTAAA AACCATGGTG CGGAGAAATT   
  
  
- TTCTTTTATT TTCCAATGTT TTAAGTGTTT TGCAAAAAT

+     P-box

| Site Name | Organism | Position | Strand | Matrix score. | sequence | function |
| --- | --- | --- | --- | --- | --- | --- |
| P-box | Oryza sativa | 1065 | + | 7 | CCTTTTG | gibberellin-responsive element |

>Potri.005G195000.1   
+ TCAACACTTT AAAATGATAT GAAAATACTA AAAATATATT AATTTAAATA AAAAAATTAA TATTTTTAAA   
  
  
+ ACATAACAGA TCTATTTGTA TTGTTCACAG GAAAAATGTA ATAATGTTTT TGTCTTTCTG AAAAAAAAAT   
  
  
+ ATTAAATTGG TTATTGGAGG CATGGAAGTT TTTTCATAAT GGGCACTAGT TGTTAACAAA TTAAATAGGG   
  
  
+ GCAAATAAGT CATTTCAAAA ATAGAGTGAG ATGACCTAAA TATCCTTATA AGAAAAGAAA TTAAAAGTGG   
  
  
+ TGTCCAAGAG CTTTATTGTA TTTTTCACAT GATTTTTTTA TTACTATAGG TCAACTAAGG GCCAATTTAG   
  
  
+ TATTTGTATA AATATATGGA AAAAAAAAGC ACAGTGAATG ATCAAAATAT TATTGGAAGC AAAAAAAAAT   
  
  
+ TAAAGTTAGC CTCGAGGCGA TGTTTTTGTA TTTTTACAAT GATTTTTTTT ATCACCAAGT GAACTTAGAG   
  
  
+ GGAATTCGGT ATTTTTCATA AATATAGAAA AATAAAAGTG GTTTGCGGGT GCATAGCAAG AGCCAACATG   
  
  
+ TGTGAGGTGT TTGGGCCTTC ATGTAACGCT TGCGACACCT CCCAACGTTC AAAATACCCT TTCTATCGTG   
  
  
+ TAATTGGAAG CGAAGCGATG TCCTTTTCCT TGCGGGGTGT TGCATGTGGT TGTTGGTGAT ACAGTTTGTT   
  
  
+ GCCGACAAAG CTTTCCCTCT ATATTTTCTC TCTCCTCCCT ACAAAATTTA CTCTGGCCAT GCAAAATTTT   
  
  
+ AGAATTGTCC TTTGATTTAT GGGGATTTCC ACTTAAGTCC TTATTTTTTA TTTCTAATTT TTATTTTTGG   
  
  
+ TCTTTTTGTA AAACTTTAAT TTTTTTTAAT TTCATCTTTA AATCTCAATT TCTAATATTT TTTATTTCAA   
  
  
+ CCTTAATCCT TATTCTTTTA ATTGTTTTTT TAATTTTTTT TGAGCCATTT GTTAAATTGA TATTTCTTAT   
  
  
+ CAATTTCACT CTTTAATAAA AAAATTCGTT TGCATTTCTT ATTTCAACCT TGATCCTCAT TCTTTTAGTT   
  
  
+ TTTTTAGTTT TTATCCTTTT GTTAAATTGT TTTTGCTTTT CAATTTCATT CTTATAGAAT TTATTTTATA   
  
  
+ TTTCAATTTT CATTCTTTTT TTTAATTGCT ATTTTTTAAT CATTTTGTAT AATTGAATTT TTTTTTTCAA   
  
  
+ TTTCATCCTT CAATATTTAA TTGATTTGGA ATTAAGTTTC AAGGTTTTTC CAGATAGGAT GTTTCTAGTC   
  
  
+ TAATAACCTG GATCACAAGT TTAAAAAGTT AACATGGGTT GATATATTTT TTTTATAAAA AAATAGACTT   
  
  
+ TGTGATTTTC TTTGTTTTAT TTTCTATCTA ATTATCTTGA TGTTATAACA TAGGCCGCAT GTTTGGCGGG   
  
  
+ ATAACTTGAG TTAGCTTAAC CCTGATTACT AGGATTATAG GTTTGTCATA TTAACTTGAG TTGACTAAAC   
  
  
+ CTGATTTTTT TATAATTTTG TTTATCTAAT TTTATTCTTT CATATTTAAT GGACTTAGAA TTAAGTTTTG   
  
  
+ TTCTTTATCA TCTTTTAAAA AAAATGTTTT TTTACGTTAT CATGATTTTT TTTTTAATTT ATATCATTTG   
  
  
+ TTATTGTTAT CTATTTATGA TCTATTTAAA ATAAAATTAA CTTCTTTAAA TTAGTTAAAT CTATTATTTG   
  
  
+ AATCACGAAA AAAAATAGTC ACCGCATGAC GAAGGTCCAA AACTAGCCAT GAGGTATTCC TCGATTAATA   
  
  
+ TCAAAGCCCA ATTGTTTAAG CAGTAAGTCG GTTATCGGAA GTCAATTTGG ACACGCTCTG TCTCTCTTAT   
  
  
+ CTAATGTAGA CATGACGGTG TTCTAAGCTT GTAAGGCCTT GTCCTTGGCT CCATGGAACC TTCAATTTAT   
  
  
+ CAGTCAAATC TACAGTGTGA ACGTATATTA TTCGATTCTG TATTTAATTT TTGGTACCAC GCCTCTTTAA   
  
  
+ AAGAAAATAA AAGGTTACAA AATTCACAAA ACGTTTTTA  

- AGTTGTGAAA TTTTACTATA CTTTTATGAT TTTTATATAA TTAAATTTAT TTTTTTAATT ATAAAAATTT   
  
  
- TGTATTGTCT AGATAAACAT AACAAGTGTC CTTTTTACAT TATTACAAAA ACAGAAAGAC TTTTTTTTTA   
  
  
- TAATTTAACC AATAACCTCC GTACCTTCAA AAAAGTATTA CCCGTGATCA ACAATTGTTT AATTTATCCC   
  
  
- CGTTTATTCA GTAAAGTTTT TATCTCACTC TACTGGATTT ATAGGAATAT TCTTTTCTTT AATTTTCACC   
  
  
- ACAGGTTCTC GAAATAACAT AAAAAGTGTA CTAAAAAAAT AATGATATCC AGTTGATTCC CGGTTAAATC   
  
  
- ATAAACATAT TTATATACCT TTTTTTTTCG TGTCACTTAC TAGTTTTATA ATAACCTTCG TTTTTTTTTA   
  
  
- ATTTCAATCG GAGCTCCGCT ACAAAAACAT AAAAATGTTA CTAAAAAAAA TAGTGGTTCA CTTGAATCTC   
  
  
- CCTTAAGCCA TAAAAAGTAT TTATATCTTT TTATTTTCAC CAAACGCCCA CGTATCGTTC TCGGTTGTAC   
  
  
- ACACTCCACA AACCCGGAAG TACATTGCGA ACGCTGTGGA GGGTTGCAAG TTTTATGGGA AAGATAGCAC   
  
  
- ATTAACCTTC GCTTCGCTAC AGGAAAAGGA ACGCCCCACA ACGTACACCA ACAACCACTA TGTCAAACAA   
  
  
- CGGCTGTTTC GAAAGGGAGA TATAAAAGAG AGAGGAGGGA TGTTTTAAAT GAGACCGGTA CGTTTTAAAA   
  
  
- TCTTAACAGG AAACTAAATA CCCCTAAAGG TGAATTCAGG AATAAAAAAT AAAGATTAAA AATAAAAACC   
  
  
- AGAAAAACAT TTTGAAATTA AAAAAAATTA AAGTAGAAAT TTAGAGTTAA AGATTATAAA AAATAAAGTT   
  
  
- GGAATTAGGA ATAAGAAAAT TAACAAAAAA ATTAAAAAAA ACTCGGTAAA CAATTTAACT ATAAAGAATA   
  
  
- GTTAAAGTGA GAAATTATTT TTTTAAGCAA ACGTAAAGAA TAAAGTTGGA ACTAGGAGTA AGAAAATCAA   
  
  
- AAAAATCAAA AATAGGAAAA CAATTTAACA AAAACGAAAA GTTAAAGTAA GAATATCTTA AATAAAATAT   
  
  
- AAAGTTAAAA GTAAGAAAAA AAATTAACGA TAAAAAATTA GTAAAACATA TTAACTTAAA AAAAAAAGTT   
  
  
- AAAGTAGGAA GTTATAAATT AACTAAACCT TAATTCAAAG TTCCAAAAAG GTCTATCCTA CAAAGATCAG   
  
  
- ATTATTGGAC CTAGTGTTCA AATTTTTCAA TTGTACCCAA CTATATAAAA AAAATATTTT TTTATCTGAA   
  
  
- ACACTAAAAG AAACAAAATA AAAGATAGAT TAATAGAACT ACAATATTGT ATCCGGCGTA CAAACCGCCC   
  
  
- TATTGAACTC AATCGAATTG GGACTAATGA TCCTAATATC CAAACAGTAT AATTGAACTC AACTGATTTG   
  
  
- GACTAAAAAA ATATTAAAAC AAATAGATTA AAATAAGAAA GTATAAATTA CCTGAATCTT AATTCAAAAC   
  
  
- AAGAAATAGT AGAAAATTTT TTTTACAAAA AAATGCAATA GTACTAAAAA AAAAATTAAA TATAGTAAAC   
  
  
- AATAACAATA GATAAATACT AGATAAATTT TATTTTAATT GAAGAAATTT AATCAATTTA GATAATAAAC   
  
  
- TTAGTGCTTT TTTTTATCAG TGGCGTACTG CTTCCAGGTT TTGATCGGTA CTCCATAAGG AGCTAATTAT   
  
  
- AGTTTCGGGT TAACAAATTC GTCATTCAGC CAATAGCCTT CAGTTAAACC TGTGCGAGAC AGAGAGAATA   
  
  
- GATTACATCT GTACTGCCAC AAGATTCGAA CATTCCGGAA CAGGAACCGA GGTACCTTGG AAGTTAAATA   
  
  
- GTCAGTTTAG ATGTCACACT TGCATATAAT AAGCTAAGAC ATAAATTAAA AACCATGGTG CGGAGAAATT   
  
  
- TTCTTTTATT TTCCAATGTT TTAAGTGTTT TGCAAAAAT

+     STRE

| Site Name | Organism | Position | Strand | Matrix score. | sequence | function |
| --- | --- | --- | --- | --- | --- | --- |
| STRE | Arabidopsis thaliana | 207 | + | 5 | AGGGG |  |

>Potri.005G195000.1   
+ TCAACACTTT AAAATGATAT GAAAATACTA AAAATATATT AATTTAAATA AAAAAATTAA TATTTTTAAA   
  
  
+ ACATAACAGA TCTATTTGTA TTGTTCACAG GAAAAATGTA ATAATGTTTT TGTCTTTCTG AAAAAAAAAT   
  
  
+ ATTAAATTGG TTATTGGAGG CATGGAAGTT TTTTCATAAT GGGCACTAGT TGTTAACAAA TTAAATAGGG   
  
  
+ GCAAATAAGT CATTTCAAAA ATAGAGTGAG ATGACCTAAA TATCCTTATA AGAAAAGAAA TTAAAAGTGG   
  
  
+ TGTCCAAGAG CTTTATTGTA TTTTTCACAT GATTTTTTTA TTACTATAGG TCAACTAAGG GCCAATTTAG   
  
  
+ TATTTGTATA AATATATGGA AAAAAAAAGC ACAGTGAATG ATCAAAATAT TATTGGAAGC AAAAAAAAAT   
  
  
+ TAAAGTTAGC CTCGAGGCGA TGTTTTTGTA TTTTTACAAT GATTTTTTTT ATCACCAAGT GAACTTAGAG   
  
  
+ GGAATTCGGT ATTTTTCATA AATATAGAAA AATAAAAGTG GTTTGCGGGT GCATAGCAAG AGCCAACATG   
  
  
+ TGTGAGGTGT TTGGGCCTTC ATGTAACGCT TGCGACACCT CCCAACGTTC AAAATACCCT TTCTATCGTG   
  
  
+ TAATTGGAAG CGAAGCGATG TCCTTTTCCT TGCGGGGTGT TGCATGTGGT TGTTGGTGAT ACAGTTTGTT   
  
  
+ GCCGACAAAG CTTTCCCTCT ATATTTTCTC TCTCCTCCCT ACAAAATTTA CTCTGGCCAT GCAAAATTTT   
  
  
+ AGAATTGTCC TTTGATTTAT GGGGATTTCC ACTTAAGTCC TTATTTTTTA TTTCTAATTT TTATTTTTGG   
  
  
+ TCTTTTTGTA AAACTTTAAT TTTTTTTAAT TTCATCTTTA AATCTCAATT TCTAATATTT TTTATTTCAA   
  
  
+ CCTTAATCCT TATTCTTTTA ATTGTTTTTT TAATTTTTTT TGAGCCATTT GTTAAATTGA TATTTCTTAT   
  
  
+ CAATTTCACT CTTTAATAAA AAAATTCGTT TGCATTTCTT ATTTCAACCT TGATCCTCAT TCTTTTAGTT   
  
  
+ TTTTTAGTTT TTATCCTTTT GTTAAATTGT TTTTGCTTTT CAATTTCATT CTTATAGAAT TTATTTTATA   
  
  
+ TTTCAATTTT CATTCTTTTT TTTAATTGCT ATTTTTTAAT CATTTTGTAT AATTGAATTT TTTTTTTCAA   
  
  
+ TTTCATCCTT CAATATTTAA TTGATTTGGA ATTAAGTTTC AAGGTTTTTC CAGATAGGAT GTTTCTAGTC   
  
  
+ TAATAACCTG GATCACAAGT TTAAAAAGTT AACATGGGTT GATATATTTT TTTTATAAAA AAATAGACTT   
  
  
+ TGTGATTTTC TTTGTTTTAT TTTCTATCTA ATTATCTTGA TGTTATAACA TAGGCCGCAT GTTTGGCGGG   
  
  
+ ATAACTTGAG TTAGCTTAAC CCTGATTACT AGGATTATAG GTTTGTCATA TTAACTTGAG TTGACTAAAC   
  
  
+ CTGATTTTTT TATAATTTTG TTTATCTAAT TTTATTCTTT CATATTTAAT GGACTTAGAA TTAAGTTTTG   
  
  
+ TTCTTTATCA TCTTTTAAAA AAAATGTTTT TTTACGTTAT CATGATTTTT TTTTTAATTT ATATCATTTG   
  
  
+ TTATTGTTAT CTATTTATGA TCTATTTAAA ATAAAATTAA CTTCTTTAAA TTAGTTAAAT CTATTATTTG   
  
  
+ AATCACGAAA AAAAATAGTC ACCGCATGAC GAAGGTCCAA AACTAGCCAT GAGGTATTCC TCGATTAATA   
  
  
+ TCAAAGCCCA ATTGTTTAAG CAGTAAGTCG GTTATCGGAA GTCAATTTGG ACACGCTCTG TCTCTCTTAT   
  
  
+ CTAATGTAGA CATGACGGTG TTCTAAGCTT GTAAGGCCTT GTCCTTGGCT CCATGGAACC TTCAATTTAT   
  
  
+ CAGTCAAATC TACAGTGTGA ACGTATATTA TTCGATTCTG TATTTAATTT TTGGTACCAC GCCTCTTTAA   
  
  
+ AAGAAAATAA AAGGTTACAA AATTCACAAA ACGTTTTTA  

- AGTTGTGAAA TTTTACTATA CTTTTATGAT TTTTATATAA TTAAATTTAT TTTTTTAATT ATAAAAATTT   
  
  
- TGTATTGTCT AGATAAACAT AACAAGTGTC CTTTTTACAT TATTACAAAA ACAGAAAGAC TTTTTTTTTA   
  
  
- TAATTTAACC AATAACCTCC GTACCTTCAA AAAAGTATTA CCCGTGATCA ACAATTGTTT AATTTATCCC   
  
  
- CGTTTATTCA GTAAAGTTTT TATCTCACTC TACTGGATTT ATAGGAATAT TCTTTTCTTT AATTTTCACC   
  
  
- ACAGGTTCTC GAAATAACAT AAAAAGTGTA CTAAAAAAAT AATGATATCC AGTTGATTCC CGGTTAAATC   
  
  
- ATAAACATAT TTATATACCT TTTTTTTTCG TGTCACTTAC TAGTTTTATA ATAACCTTCG TTTTTTTTTA   
  
  
- ATTTCAATCG GAGCTCCGCT ACAAAAACAT AAAAATGTTA CTAAAAAAAA TAGTGGTTCA CTTGAATCTC   
  
  
- CCTTAAGCCA TAAAAAGTAT TTATATCTTT TTATTTTCAC CAAACGCCCA CGTATCGTTC TCGGTTGTAC   
  
  
- ACACTCCACA AACCCGGAAG TACATTGCGA ACGCTGTGGA GGGTTGCAAG TTTTATGGGA AAGATAGCAC   
  
  
- ATTAACCTTC GCTTCGCTAC AGGAAAAGGA ACGCCCCACA ACGTACACCA ACAACCACTA TGTCAAACAA   
  
  
- CGGCTGTTTC GAAAGGGAGA TATAAAAGAG AGAGGAGGGA TGTTTTAAAT GAGACCGGTA CGTTTTAAAA   
  
  
- TCTTAACAGG AAACTAAATA CCCCTAAAGG TGAATTCAGG AATAAAAAAT AAAGATTAAA AATAAAAACC   
  
  
- AGAAAAACAT TTTGAAATTA AAAAAAATTA AAGTAGAAAT TTAGAGTTAA AGATTATAAA AAATAAAGTT   
  
  
- GGAATTAGGA ATAAGAAAAT TAACAAAAAA ATTAAAAAAA ACTCGGTAAA CAATTTAACT ATAAAGAATA   
  
  
- GTTAAAGTGA GAAATTATTT TTTTAAGCAA ACGTAAAGAA TAAAGTTGGA ACTAGGAGTA AGAAAATCAA   
  
  
- AAAAATCAAA AATAGGAAAA CAATTTAACA AAAACGAAAA GTTAAAGTAA GAATATCTTA AATAAAATAT   
  
  
- AAAGTTAAAA GTAAGAAAAA AAATTAACGA TAAAAAATTA GTAAAACATA TTAACTTAAA AAAAAAAGTT   
  
  
- AAAGTAGGAA GTTATAAATT AACTAAACCT TAATTCAAAG TTCCAAAAAG GTCTATCCTA CAAAGATCAG   
  
  
- ATTATTGGAC CTAGTGTTCA AATTTTTCAA TTGTACCCAA CTATATAAAA AAAATATTTT TTTATCTGAA   
  
  
- ACACTAAAAG AAACAAAATA AAAGATAGAT TAATAGAACT ACAATATTGT ATCCGGCGTA CAAACCGCCC   
  
  
- TATTGAACTC AATCGAATTG GGACTAATGA TCCTAATATC CAAACAGTAT AATTGAACTC AACTGATTTG   
  
  
- GACTAAAAAA ATATTAAAAC AAATAGATTA AAATAAGAAA GTATAAATTA CCTGAATCTT AATTCAAAAC   
  
  
- AAGAAATAGT AGAAAATTTT TTTTACAAAA AAATGCAATA GTACTAAAAA AAAAATTAAA TATAGTAAAC   
  
  
- AATAACAATA GATAAATACT AGATAAATTT TATTTTAATT GAAGAAATTT AATCAATTTA GATAATAAAC   
  
  
- TTAGTGCTTT TTTTTATCAG TGGCGTACTG CTTCCAGGTT TTGATCGGTA CTCCATAAGG AGCTAATTAT   
  
  
- AGTTTCGGGT TAACAAATTC GTCATTCAGC CAATAGCCTT CAGTTAAACC TGTGCGAGAC AGAGAGAATA   
  
  
- GATTACATCT GTACTGCCAC AAGATTCGAA CATTCCGGAA CAGGAACCGA GGTACCTTGG AAGTTAAATA   
  
  
- GTCAGTTTAG ATGTCACACT TGCATATAAT AAGCTAAGAC ATAAATTAAA AACCATGGTG CGGAGAAATT   
  
  
- TTCTTTTATT TTCCAATGTT TTAAGTGTTT TGCAAAAAT

+     TATA

| Site Name | Organism | Position | Strand | Matrix score. | sequence | function |
| --- | --- | --- | --- | --- | --- | --- |
| TATA | Arabidopsis thaliana | 1113 | - | 8 | TATAAAAT |  |

>Potri.005G195000.1   
+ TCAACACTTT AAAATGATAT GAAAATACTA AAAATATATT AATTTAAATA AAAAAATTAA TATTTTTAAA   
  
  
+ ACATAACAGA TCTATTTGTA TTGTTCACAG GAAAAATGTA ATAATGTTTT TGTCTTTCTG AAAAAAAAAT   
  
  
+ ATTAAATTGG TTATTGGAGG CATGGAAGTT TTTTCATAAT GGGCACTAGT TGTTAACAAA TTAAATAGGG   
  
  
+ GCAAATAAGT CATTTCAAAA ATAGAGTGAG ATGACCTAAA TATCCTTATA AGAAAAGAAA TTAAAAGTGG   
  
  
+ TGTCCAAGAG CTTTATTGTA TTTTTCACAT GATTTTTTTA TTACTATAGG TCAACTAAGG GCCAATTTAG   
  
  
+ TATTTGTATA AATATATGGA AAAAAAAAGC ACAGTGAATG ATCAAAATAT TATTGGAAGC AAAAAAAAAT   
  
  
+ TAAAGTTAGC CTCGAGGCGA TGTTTTTGTA TTTTTACAAT GATTTTTTTT ATCACCAAGT GAACTTAGAG   
  
  
+ GGAATTCGGT ATTTTTCATA AATATAGAAA AATAAAAGTG GTTTGCGGGT GCATAGCAAG AGCCAACATG   
  
  
+ TGTGAGGTGT TTGGGCCTTC ATGTAACGCT TGCGACACCT CCCAACGTTC AAAATACCCT TTCTATCGTG   
  
  
+ TAATTGGAAG CGAAGCGATG TCCTTTTCCT TGCGGGGTGT TGCATGTGGT TGTTGGTGAT ACAGTTTGTT   
  
  
+ GCCGACAAAG CTTTCCCTCT ATATTTTCTC TCTCCTCCCT ACAAAATTTA CTCTGGCCAT GCAAAATTTT   
  
  
+ AGAATTGTCC TTTGATTTAT GGGGATTTCC ACTTAAGTCC TTATTTTTTA TTTCTAATTT TTATTTTTGG   
  
  
+ TCTTTTTGTA AAACTTTAAT TTTTTTTAAT TTCATCTTTA AATCTCAATT TCTAATATTT TTTATTTCAA   
  
  
+ CCTTAATCCT TATTCTTTTA ATTGTTTTTT TAATTTTTTT TGAGCCATTT GTTAAATTGA TATTTCTTAT   
  
  
+ CAATTTCACT CTTTAATAAA AAAATTCGTT TGCATTTCTT ATTTCAACCT TGATCCTCAT TCTTTTAGTT   
  
  
+ TTTTTAGTTT TTATCCTTTT GTTAAATTGT TTTTGCTTTT CAATTTCATT CTTATAGAAT TTATTTTATA   
  
  
+ TTTCAATTTT CATTCTTTTT TTTAATTGCT ATTTTTTAAT CATTTTGTAT AATTGAATTT TTTTTTTCAA   
  
  
+ TTTCATCCTT CAATATTTAA TTGATTTGGA ATTAAGTTTC AAGGTTTTTC CAGATAGGAT GTTTCTAGTC   
  
  
+ TAATAACCTG GATCACAAGT TTAAAAAGTT AACATGGGTT GATATATTTT TTTTATAAAA AAATAGACTT   
  
  
+ TGTGATTTTC TTTGTTTTAT TTTCTATCTA ATTATCTTGA TGTTATAACA TAGGCCGCAT GTTTGGCGGG   
  
  
+ ATAACTTGAG TTAGCTTAAC CCTGATTACT AGGATTATAG GTTTGTCATA TTAACTTGAG TTGACTAAAC   
  
  
+ CTGATTTTTT TATAATTTTG TTTATCTAAT TTTATTCTTT CATATTTAAT GGACTTAGAA TTAAGTTTTG   
  
  
+ TTCTTTATCA TCTTTTAAAA AAAATGTTTT TTTACGTTAT CATGATTTTT TTTTTAATTT ATATCATTTG   
  
  
+ TTATTGTTAT CTATTTATGA TCTATTTAAA ATAAAATTAA CTTCTTTAAA TTAGTTAAAT CTATTATTTG   
  
  
+ AATCACGAAA AAAAATAGTC ACCGCATGAC GAAGGTCCAA AACTAGCCAT GAGGTATTCC TCGATTAATA   
  
  
+ TCAAAGCCCA ATTGTTTAAG CAGTAAGTCG GTTATCGGAA GTCAATTTGG ACACGCTCTG TCTCTCTTAT   
  
  
+ CTAATGTAGA CATGACGGTG TTCTAAGCTT GTAAGGCCTT GTCCTTGGCT CCATGGAACC TTCAATTTAT   
  
  
+ CAGTCAAATC TACAGTGTGA ACGTATATTA TTCGATTCTG TATTTAATTT TTGGTACCAC GCCTCTTTAA   
  
  
+ AAGAAAATAA AAGGTTACAA AATTCACAAA ACGTTTTTA  

- AGTTGTGAAA TTTTACTATA CTTTTATGAT TTTTATATAA TTAAATTTAT TTTTTTAATT ATAAAAATTT   
  
  
- TGTATTGTCT AGATAAACAT AACAAGTGTC CTTTTTACAT TATTACAAAA ACAGAAAGAC TTTTTTTTTA   
  
  
- TAATTTAACC AATAACCTCC GTACCTTCAA AAAAGTATTA CCCGTGATCA ACAATTGTTT AATTTATCCC   
  
  
- CGTTTATTCA GTAAAGTTTT TATCTCACTC TACTGGATTT ATAGGAATAT TCTTTTCTTT AATTTTCACC   
  
  
- ACAGGTTCTC GAAATAACAT AAAAAGTGTA CTAAAAAAAT AATGATATCC AGTTGATTCC CGGTTAAATC   
  
  
- ATAAACATAT TTATATACCT TTTTTTTTCG TGTCACTTAC TAGTTTTATA ATAACCTTCG TTTTTTTTTA   
  
  
- ATTTCAATCG GAGCTCCGCT ACAAAAACAT AAAAATGTTA CTAAAAAAAA TAGTGGTTCA CTTGAATCTC   
  
  
- CCTTAAGCCA TAAAAAGTAT TTATATCTTT TTATTTTCAC CAAACGCCCA CGTATCGTTC TCGGTTGTAC   
  
  
- ACACTCCACA AACCCGGAAG TACATTGCGA ACGCTGTGGA GGGTTGCAAG TTTTATGGGA AAGATAGCAC   
  
  
- ATTAACCTTC GCTTCGCTAC AGGAAAAGGA ACGCCCCACA ACGTACACCA ACAACCACTA TGTCAAACAA   
  
  
- CGGCTGTTTC GAAAGGGAGA TATAAAAGAG AGAGGAGGGA TGTTTTAAAT GAGACCGGTA CGTTTTAAAA   
  
  
- TCTTAACAGG AAACTAAATA CCCCTAAAGG TGAATTCAGG AATAAAAAAT AAAGATTAAA AATAAAAACC   
  
  
- AGAAAAACAT TTTGAAATTA AAAAAAATTA AAGTAGAAAT TTAGAGTTAA AGATTATAAA AAATAAAGTT   
  
  
- GGAATTAGGA ATAAGAAAAT TAACAAAAAA ATTAAAAAAA ACTCGGTAAA CAATTTAACT ATAAAGAATA   
  
  
- GTTAAAGTGA GAAATTATTT TTTTAAGCAA ACGTAAAGAA TAAAGTTGGA ACTAGGAGTA AGAAAATCAA   
  
  
- AAAAATCAAA AATAGGAAAA CAATTTAACA AAAACGAAAA GTTAAAGTAA GAATATCTTA AATAAAATAT   
  
  
- AAAGTTAAAA GTAAGAAAAA AAATTAACGA TAAAAAATTA GTAAAACATA TTAACTTAAA AAAAAAAGTT   
  
  
- AAAGTAGGAA GTTATAAATT AACTAAACCT TAATTCAAAG TTCCAAAAAG GTCTATCCTA CAAAGATCAG   
  
  
- ATTATTGGAC CTAGTGTTCA AATTTTTCAA TTGTACCCAA CTATATAAAA AAAATATTTT TTTATCTGAA   
  
  
- ACACTAAAAG AAACAAAATA AAAGATAGAT TAATAGAACT ACAATATTGT ATCCGGCGTA CAAACCGCCC   
  
  
- TATTGAACTC AATCGAATTG GGACTAATGA TCCTAATATC CAAACAGTAT AATTGAACTC AACTGATTTG   
  
  
- GACTAAAAAA ATATTAAAAC AAATAGATTA AAATAAGAAA GTATAAATTA CCTGAATCTT AATTCAAAAC   
  
  
- AAGAAATAGT AGAAAATTTT TTTTACAAAA AAATGCAATA GTACTAAAAA AAAAATTAAA TATAGTAAAC   
  
  
- AATAACAATA GATAAATACT AGATAAATTT TATTTTAATT GAAGAAATTT AATCAATTTA GATAATAAAC   
  
  
- TTAGTGCTTT TTTTTATCAG TGGCGTACTG CTTCCAGGTT TTGATCGGTA CTCCATAAGG AGCTAATTAT   
  
  
- AGTTTCGGGT TAACAAATTC GTCATTCAGC CAATAGCCTT CAGTTAAACC TGTGCGAGAC AGAGAGAATA   
  
  
- GATTACATCT GTACTGCCAC AAGATTCGAA CATTCCGGAA CAGGAACCGA GGTACCTTGG AAGTTAAATA   
  
  
- GTCAGTTTAG ATGTCACACT TGCATATAAT AAGCTAAGAC ATAAATTAAA AACCATGGTG CGGAGAAATT   
  
  
- TTCTTTTATT TTCCAATGTT TTAAGTGTTT TGCAAAAAT

+     TATA-box

| Site Name | Organism | Position | Strand | Matrix score. | sequence | function |
| --- | --- | --- | --- | --- | --- | --- |
| TATA-box | Oryza sativa | 1976 | + | 7 | TACAAAA | core promoter element around -30 of transcription start |
| TATA-box | Arabidopsis thaliana | 1633 | + | 8 | TATTTAAA | core promoter element around -30 of transcription start |
| TATA-box | Arabidopsis thaliana | 1914 | - | 4 | TATA | core promoter element around -30 of transcription start |
| TATA-box | Arabidopsis thaliana | 1599 | - | 5 | TATAA | core promoter element around -30 of transcription start |
| TATA-box | Arabidopsis thaliana | 1600 | - | 4 | TATA | core promoter element around -30 of transcription start |
| TATA-box | Brassica juncea | 1597 | - | 7 | TATAAAT | core promoter element around -30 of transcription start |
| TATA-box | Helianthus annuus | 1598 | - | 6 | TATAAA | core promoter element around -30 of transcription start |
| TATA-box | Arabidopsis thaliana | 1481 | - | 4 | TATA | core promoter element around -30 of transcription start |
| TATA-box | Arabidopsis thaliana | 1480 | - | 5 | TATAA | core promoter element around -30 of transcription start |
| TATA-box | Pisum sativum | 1478 | - | 7 | TATAAAA | core promoter element around -30 of transcription start |
| TATA-box | Helianthus annuus | 1479 | - | 6 | TATAAA | core promoter element around -30 of transcription start |
| TATA-box | Arabidopsis thaliana | 1436 | - | 4 | TATA | core promoter element around -30 of transcription start |
| TATA-box | Arabidopsis thaliana | 1435 | - | 5 | TATAA | core promoter element around -30 of transcription start |
| TATA-box | Brassica napus | 1434 | + | 6 | ATTATA | core promoter element around -30 of transcription start |
| TATA-box | Arabidopsis thaliana | 1374 | - | 4 | TATA | core promoter element around -30 of transcription start |
| TATA-box | Arabidopsis thaliana | 1314 | - | 4 | TATA | core promoter element around -30 of transcription start |
| TATA-box | Arabidopsis thaliana | 1373 | - | 5 | TATAA | core promoter element around -30 of transcription start |
| TATA-box | Arabidopsis thaliana | 1313 | - | 5 | TATAA | core promoter element around -30 of transcription start |
| TATA-box | Helianthus annuus | 1312 | - | 6 | TATAAA | core promoter element around -30 of transcription start |
| TATA-box | Pisum sativum | 1311 | - | 7 | TATAAAA | core promoter element around -30 of transcription start |
| TATA-box | Arabidopsis thaliana | 1303 | - | 4 | TATA | core promoter element around -30 of transcription start |
| TATA-box | Brassica napus | 1302 | - | 6 | ATATAT | core promoter element around -30 of transcription start |
| TATA-box | Arabidopsis thaliana | 1168 | - | 4 | TATA | core promoter element around -30 of transcription start |
| TATA-box | Oryza sativa | 1163 | - | 7 | TACAAAA | core promoter element around -30 of transcription start |
| TATA-box | Helianthus annuus | 1166 | - | 6 | TATACA | core promoter element around -30 of transcription start |
| TATA-box | Arabidopsis thaliana | 1117 | - | 4 | TATA | core promoter element around -30 of transcription start |
| TATA-box | Arabidopsis thaliana | 513 | + | 4 | TATA | core promoter element around -30 of transcription start |
| TATA-box | Arabidopsis thaliana | 1116 | - | 5 | TATAA | core promoter element around -30 of transcription start |
| TATA-box | Helianthus annuus | 1115 | - | 6 | TATAAA | core promoter element around -30 of transcription start |
| TATA-box | Pisum sativum | 1114 | - | 7 | TATAAAA | core promoter element around -30 of transcription start |
| TATA-box | Arabidopsis thaliana | 1103 | - | 4 | TATA | core promoter element around -30 of transcription start |
| TATA-box | Arabidopsis thaliana | 1102 | - | 5 | TATAA | core promoter element around -30 of transcription start |
| TATA-box | Zea mays | 1099 | - | 8 | TATAAGAA | core promoter element around -30 of transcription start |
| TATA-box | Oryza sativa | 844 | - | 7 | TACAAAA | core promoter element around -30 of transcription start |
| TATA-box | Oryza sativa | 740 | + | 7 | TACAAAA | core promoter element around -30 of transcription start |
| TATA-box | Arabidopsis thaliana | 720 | + | 4 | TATA | core promoter element around -30 of transcription start |
| TATA-box | Oryza sativa | 444 | - | 7 | TACAAAA | core promoter element around -30 of transcription start |
| TATA-box | Arabidopsis thaliana | 363 | + | 4 | TATA | core promoter element around -30 of transcription start |
| TATA-box | Brassica napus | 362 | + | 6 | ATATAT | core promoter element around -30 of transcription start |
| TATA-box | Arabidopsis thaliana | 357 | + | 4 | TATA | core promoter element around -30 of transcription start |
| TATA-box | Helianthus annuus | 355 | - | 6 | TATACA | core promoter element around -30 of transcription start |
| TATA-box | Arabidopsis thaliana | 325 | + | 4 | TATA | core promoter element around -30 of transcription start |
| TATA-box | Arabidopsis thaliana | 257 | + | 4 | TATA | core promoter element around -30 of transcription start |
| TATA-box | Arabidopsis thaliana | 256 | - | 5 | TATAA | core promoter element around -30 of transcription start |
| TATA-box | Arabidopsis thaliana | 35 | + | 4 | TATA | core promoter element around -30 of transcription start |
| TATA-box | Brassica napus | 34 | + | 6 | ATATAT | core promoter element around -30 of transcription start |
| TATA-box | Arabidopsis thaliana | 43 | - | 8 | TATTTAAA | core promoter element around -30 of transcription start |

>Potri.005G195000.1   
+ TCAACACTTT AAAATGATAT GAAAATACTA AAAATATATT AATTTAAATA AAAAAATTAA TATTTTTAAA   
  
  
+ ACATAACAGA TCTATTTGTA TTGTTCACAG GAAAAATGTA ATAATGTTTT TGTCTTTCTG AAAAAAAAAT   
  
  
+ ATTAAATTGG TTATTGGAGG CATGGAAGTT TTTTCATAAT GGGCACTAGT TGTTAACAAA TTAAATAGGG   
  
  
+ GCAAATAAGT CATTTCAAAA ATAGAGTGAG ATGACCTAAA TATCCTTATA AGAAAAGAAA TTAAAAGTGG   
  
  
+ TGTCCAAGAG CTTTATTGTA TTTTTCACAT GATTTTTTTA TTACTATAGG TCAACTAAGG GCCAATTTAG   
  
  
+ TATTTGTATA AATATATGGA AAAAAAAAGC ACAGTGAATG ATCAAAATAT TATTGGAAGC AAAAAAAAAT   
  
  
+ TAAAGTTAGC CTCGAGGCGA TGTTTTTGTA TTTTTACAAT GATTTTTTTT ATCACCAAGT GAACTTAGAG   
  
  
+ GGAATTCGGT ATTTTTCATA AATATAGAAA AATAAAAGTG GTTTGCGGGT GCATAGCAAG AGCCAACATG   
  
  
+ TGTGAGGTGT TTGGGCCTTC ATGTAACGCT TGCGACACCT CCCAACGTTC AAAATACCCT TTCTATCGTG   
  
  
+ TAATTGGAAG CGAAGCGATG TCCTTTTCCT TGCGGGGTGT TGCATGTGGT TGTTGGTGAT ACAGTTTGTT   
  
  
+ GCCGACAAAG CTTTCCCTCT ATATTTTCTC TCTCCTCCCT ACAAAATTTA CTCTGGCCAT GCAAAATTTT   
  
  
+ AGAATTGTCC TTTGATTTAT GGGGATTTCC ACTTAAGTCC TTATTTTTTA TTTCTAATTT TTATTTTTGG   
  
  
+ TCTTTTTGTA AAACTTTAAT TTTTTTTAAT TTCATCTTTA AATCTCAATT TCTAATATTT TTTATTTCAA   
  
  
+ CCTTAATCCT TATTCTTTTA ATTGTTTTTT TAATTTTTTT TGAGCCATTT GTTAAATTGA TATTTCTTAT   
  
  
+ CAATTTCACT CTTTAATAAA AAAATTCGTT TGCATTTCTT ATTTCAACCT TGATCCTCAT TCTTTTAGTT   
  
  
+ TTTTTAGTTT TTATCCTTTT GTTAAATTGT TTTTGCTTTT CAATTTCATT CTTATAGAAT TTATTTTATA   
  
  
+ TTTCAATTTT CATTCTTTTT TTTAATTGCT ATTTTTTAAT CATTTTGTAT AATTGAATTT TTTTTTTCAA   
  
  
+ TTTCATCCTT CAATATTTAA TTGATTTGGA ATTAAGTTTC AAGGTTTTTC CAGATAGGAT GTTTCTAGTC   
  
  
+ TAATAACCTG GATCACAAGT TTAAAAAGTT AACATGGGTT GATATATTTT TTTTATAAAA AAATAGACTT   
  
  
+ TGTGATTTTC TTTGTTTTAT TTTCTATCTA ATTATCTTGA TGTTATAACA TAGGCCGCAT GTTTGGCGGG   
  
  
+ ATAACTTGAG TTAGCTTAAC CCTGATTACT AGGATTATAG GTTTGTCATA TTAACTTGAG TTGACTAAAC   
  
  
+ CTGATTTTTT TATAATTTTG TTTATCTAAT TTTATTCTTT CATATTTAAT GGACTTAGAA TTAAGTTTTG   
  
  
+ TTCTTTATCA TCTTTTAAAA AAAATGTTTT TTTACGTTAT CATGATTTTT TTTTTAATTT ATATCATTTG   
  
  
+ TTATTGTTAT CTATTTATGA TCTATTTAAA ATAAAATTAA CTTCTTTAAA TTAGTTAAAT CTATTATTTG   
  
  
+ AATCACGAAA AAAAATAGTC ACCGCATGAC GAAGGTCCAA AACTAGCCAT GAGGTATTCC TCGATTAATA   
  
  
+ TCAAAGCCCA ATTGTTTAAG CAGTAAGTCG GTTATCGGAA GTCAATTTGG ACACGCTCTG TCTCTCTTAT   
  
  
+ CTAATGTAGA CATGACGGTG TTCTAAGCTT GTAAGGCCTT GTCCTTGGCT CCATGGAACC TTCAATTTAT   
  
  
+ CAGTCAAATC TACAGTGTGA ACGTATATTA TTCGATTCTG TATTTAATTT TTGGTACCAC GCCTCTTTAA   
  
  
+ AAGAAAATAA AAGGTTACAA AATTCACAAA ACGTTTTTA  

- AGTTGTGAAA TTTTACTATA CTTTTATGAT TTTTATATAA TTAAATTTAT TTTTTTAATT ATAAAAATTT   
  
  
- TGTATTGTCT AGATAAACAT AACAAGTGTC CTTTTTACAT TATTACAAAA ACAGAAAGAC TTTTTTTTTA   
  
  
- TAATTTAACC AATAACCTCC GTACCTTCAA AAAAGTATTA CCCGTGATCA ACAATTGTTT AATTTATCCC   
  
  
- CGTTTATTCA GTAAAGTTTT TATCTCACTC TACTGGATTT ATAGGAATAT TCTTTTCTTT AATTTTCACC   
  
  
- ACAGGTTCTC GAAATAACAT AAAAAGTGTA CTAAAAAAAT AATGATATCC AGTTGATTCC CGGTTAAATC   
  
  
- ATAAACATAT TTATATACCT TTTTTTTTCG TGTCACTTAC TAGTTTTATA ATAACCTTCG TTTTTTTTTA   
  
  
- ATTTCAATCG GAGCTCCGCT ACAAAAACAT AAAAATGTTA CTAAAAAAAA TAGTGGTTCA CTTGAATCTC   
  
  
- CCTTAAGCCA TAAAAAGTAT TTATATCTTT TTATTTTCAC CAAACGCCCA CGTATCGTTC TCGGTTGTAC   
  
  
- ACACTCCACA AACCCGGAAG TACATTGCGA ACGCTGTGGA GGGTTGCAAG TTTTATGGGA AAGATAGCAC   
  
  
- ATTAACCTTC GCTTCGCTAC AGGAAAAGGA ACGCCCCACA ACGTACACCA ACAACCACTA TGTCAAACAA   
  
  
- CGGCTGTTTC GAAAGGGAGA TATAAAAGAG AGAGGAGGGA TGTTTTAAAT GAGACCGGTA CGTTTTAAAA   
  
  
- TCTTAACAGG AAACTAAATA CCCCTAAAGG TGAATTCAGG AATAAAAAAT AAAGATTAAA AATAAAAACC   
  
  
- AGAAAAACAT TTTGAAATTA AAAAAAATTA AAGTAGAAAT TTAGAGTTAA AGATTATAAA AAATAAAGTT   
  
  
- GGAATTAGGA ATAAGAAAAT TAACAAAAAA ATTAAAAAAA ACTCGGTAAA CAATTTAACT ATAAAGAATA   
  
  
- GTTAAAGTGA GAAATTATTT TTTTAAGCAA ACGTAAAGAA TAAAGTTGGA ACTAGGAGTA AGAAAATCAA   
  
  
- AAAAATCAAA AATAGGAAAA CAATTTAACA AAAACGAAAA GTTAAAGTAA GAATATCTTA AATAAAATAT   
  
  
- AAAGTTAAAA GTAAGAAAAA AAATTAACGA TAAAAAATTA GTAAAACATA TTAACTTAAA AAAAAAAGTT   
  
  
- AAAGTAGGAA GTTATAAATT AACTAAACCT TAATTCAAAG TTCCAAAAAG GTCTATCCTA CAAAGATCAG   
  
  
- ATTATTGGAC CTAGTGTTCA AATTTTTCAA TTGTACCCAA CTATATAAAA AAAATATTTT TTTATCTGAA   
  
  
- ACACTAAAAG AAACAAAATA AAAGATAGAT TAATAGAACT ACAATATTGT ATCCGGCGTA CAAACCGCCC   
  
  
- TATTGAACTC AATCGAATTG GGACTAATGA TCCTAATATC CAAACAGTAT AATTGAACTC AACTGATTTG   
  
  
- GACTAAAAAA ATATTAAAAC AAATAGATTA AAATAAGAAA GTATAAATTA CCTGAATCTT AATTCAAAAC   
  
  
- AAGAAATAGT AGAAAATTTT TTTTACAAAA AAATGCAATA GTACTAAAAA AAAAATTAAA TATAGTAAAC   
  
  
- AATAACAATA GATAAATACT AGATAAATTT TATTTTAATT GAAGAAATTT AATCAATTTA GATAATAAAC   
  
  
- TTAGTGCTTT TTTTTATCAG TGGCGTACTG CTTCCAGGTT TTGATCGGTA CTCCATAAGG AGCTAATTAT   
  
  
- AGTTTCGGGT TAACAAATTC GTCATTCAGC CAATAGCCTT CAGTTAAACC TGTGCGAGAC AGAGAGAATA   
  
  
- GATTACATCT GTACTGCCAC AAGATTCGAA CATTCCGGAA CAGGAACCGA GGTACCTTGG AAGTTAAATA   
  
  
- GTCAGTTTAG ATGTCACACT TGCATATAAT AAGCTAAGAC ATAAATTAAA AACCATGGTG CGGAGAAATT   
  
  
- TTCTTTTATT TTCCAATGTT TTAAGTGTTT TGCAAAAAT

+     TC-rich repeats

| Site Name | Organism | Position | Strand | Matrix score. | sequence | function |
| --- | --- | --- | --- | --- | --- | --- |
| TC-rich repeats | Nicotiana tabacum | 1566 | + | 9 | GTTTTCTTAC | cis-acting element involved in defense and stress responsiveness |

>Potri.005G195000.1   
+ TCAACACTTT AAAATGATAT GAAAATACTA AAAATATATT AATTTAAATA AAAAAATTAA TATTTTTAAA   
  
  
+ ACATAACAGA TCTATTTGTA TTGTTCACAG GAAAAATGTA ATAATGTTTT TGTCTTTCTG AAAAAAAAAT   
  
  
+ ATTAAATTGG TTATTGGAGG CATGGAAGTT TTTTCATAAT GGGCACTAGT TGTTAACAAA TTAAATAGGG   
  
  
+ GCAAATAAGT CATTTCAAAA ATAGAGTGAG ATGACCTAAA TATCCTTATA AGAAAAGAAA TTAAAAGTGG   
  
  
+ TGTCCAAGAG CTTTATTGTA TTTTTCACAT GATTTTTTTA TTACTATAGG TCAACTAAGG GCCAATTTAG   
  
  
+ TATTTGTATA AATATATGGA AAAAAAAAGC ACAGTGAATG ATCAAAATAT TATTGGAAGC AAAAAAAAAT   
  
  
+ TAAAGTTAGC CTCGAGGCGA TGTTTTTGTA TTTTTACAAT GATTTTTTTT ATCACCAAGT GAACTTAGAG   
  
  
+ GGAATTCGGT ATTTTTCATA AATATAGAAA AATAAAAGTG GTTTGCGGGT GCATAGCAAG AGCCAACATG   
  
  
+ TGTGAGGTGT TTGGGCCTTC ATGTAACGCT TGCGACACCT CCCAACGTTC AAAATACCCT TTCTATCGTG   
  
  
+ TAATTGGAAG CGAAGCGATG TCCTTTTCCT TGCGGGGTGT TGCATGTGGT TGTTGGTGAT ACAGTTTGTT   
  
  
+ GCCGACAAAG CTTTCCCTCT ATATTTTCTC TCTCCTCCCT ACAAAATTTA CTCTGGCCAT GCAAAATTTT   
  
  
+ AGAATTGTCC TTTGATTTAT GGGGATTTCC ACTTAAGTCC TTATTTTTTA TTTCTAATTT TTATTTTTGG   
  
  
+ TCTTTTTGTA AAACTTTAAT TTTTTTTAAT TTCATCTTTA AATCTCAATT TCTAATATTT TTTATTTCAA   
  
  
+ CCTTAATCCT TATTCTTTTA ATTGTTTTTT TAATTTTTTT TGAGCCATTT GTTAAATTGA TATTTCTTAT   
  
  
+ CAATTTCACT CTTTAATAAA AAAATTCGTT TGCATTTCTT ATTTCAACCT TGATCCTCAT TCTTTTAGTT   
  
  
+ TTTTTAGTTT TTATCCTTTT GTTAAATTGT TTTTGCTTTT CAATTTCATT CTTATAGAAT TTATTTTATA   
  
  
+ TTTCAATTTT CATTCTTTTT TTTAATTGCT ATTTTTTAAT CATTTTGTAT AATTGAATTT TTTTTTTCAA   
  
  
+ TTTCATCCTT CAATATTTAA TTGATTTGGA ATTAAGTTTC AAGGTTTTTC CAGATAGGAT GTTTCTAGTC   
  
  
+ TAATAACCTG GATCACAAGT TTAAAAAGTT AACATGGGTT GATATATTTT TTTTATAAAA AAATAGACTT   
  
  
+ TGTGATTTTC TTTGTTTTAT TTTCTATCTA ATTATCTTGA TGTTATAACA TAGGCCGCAT GTTTGGCGGG   
  
  
+ ATAACTTGAG TTAGCTTAAC CCTGATTACT AGGATTATAG GTTTGTCATA TTAACTTGAG TTGACTAAAC   
  
  
+ CTGATTTTTT TATAATTTTG TTTATCTAAT TTTATTCTTT CATATTTAAT GGACTTAGAA TTAAGTTTTG   
  
  
+ TTCTTTATCA TCTTTTAAAA AAAATGTTTT TTTACGTTAT CATGATTTTT TTTTTAATTT ATATCATTTG   
  
  
+ TTATTGTTAT CTATTTATGA TCTATTTAAA ATAAAATTAA CTTCTTTAAA TTAGTTAAAT CTATTATTTG   
  
  
+ AATCACGAAA AAAAATAGTC ACCGCATGAC GAAGGTCCAA AACTAGCCAT GAGGTATTCC TCGATTAATA   
  
  
+ TCAAAGCCCA ATTGTTTAAG CAGTAAGTCG GTTATCGGAA GTCAATTTGG ACACGCTCTG TCTCTCTTAT   
  
  
+ CTAATGTAGA CATGACGGTG TTCTAAGCTT GTAAGGCCTT GTCCTTGGCT CCATGGAACC TTCAATTTAT   
  
  
+ CAGTCAAATC TACAGTGTGA ACGTATATTA TTCGATTCTG TATTTAATTT TTGGTACCAC GCCTCTTTAA   
  
  
+ AAGAAAATAA AAGGTTACAA AATTCACAAA ACGTTTTTA  

- AGTTGTGAAA TTTTACTATA CTTTTATGAT TTTTATATAA TTAAATTTAT TTTTTTAATT ATAAAAATTT   
  
  
- TGTATTGTCT AGATAAACAT AACAAGTGTC CTTTTTACAT TATTACAAAA ACAGAAAGAC TTTTTTTTTA   
  
  
- TAATTTAACC AATAACCTCC GTACCTTCAA AAAAGTATTA CCCGTGATCA ACAATTGTTT AATTTATCCC   
  
  
- CGTTTATTCA GTAAAGTTTT TATCTCACTC TACTGGATTT ATAGGAATAT TCTTTTCTTT AATTTTCACC   
  
  
- ACAGGTTCTC GAAATAACAT AAAAAGTGTA CTAAAAAAAT AATGATATCC AGTTGATTCC CGGTTAAATC   
  
  
- ATAAACATAT TTATATACCT TTTTTTTTCG TGTCACTTAC TAGTTTTATA ATAACCTTCG TTTTTTTTTA   
  
  
- ATTTCAATCG GAGCTCCGCT ACAAAAACAT AAAAATGTTA CTAAAAAAAA TAGTGGTTCA CTTGAATCTC   
  
  
- CCTTAAGCCA TAAAAAGTAT TTATATCTTT TTATTTTCAC CAAACGCCCA CGTATCGTTC TCGGTTGTAC   
  
  
- ACACTCCACA AACCCGGAAG TACATTGCGA ACGCTGTGGA GGGTTGCAAG TTTTATGGGA AAGATAGCAC   
  
  
- ATTAACCTTC GCTTCGCTAC AGGAAAAGGA ACGCCCCACA ACGTACACCA ACAACCACTA TGTCAAACAA   
  
  
- CGGCTGTTTC GAAAGGGAGA TATAAAAGAG AGAGGAGGGA TGTTTTAAAT GAGACCGGTA CGTTTTAAAA   
  
  
- TCTTAACAGG AAACTAAATA CCCCTAAAGG TGAATTCAGG AATAAAAAAT AAAGATTAAA AATAAAAACC   
  
  
- AGAAAAACAT TTTGAAATTA AAAAAAATTA AAGTAGAAAT TTAGAGTTAA AGATTATAAA AAATAAAGTT   
  
  
- GGAATTAGGA ATAAGAAAAT TAACAAAAAA ATTAAAAAAA ACTCGGTAAA CAATTTAACT ATAAAGAATA   
  
  
- GTTAAAGTGA GAAATTATTT TTTTAAGCAA ACGTAAAGAA TAAAGTTGGA ACTAGGAGTA AGAAAATCAA   
  
  
- AAAAATCAAA AATAGGAAAA CAATTTAACA AAAACGAAAA GTTAAAGTAA GAATATCTTA AATAAAATAT   
  
  
- AAAGTTAAAA GTAAGAAAAA AAATTAACGA TAAAAAATTA GTAAAACATA TTAACTTAAA AAAAAAAGTT   
  
  
- AAAGTAGGAA GTTATAAATT AACTAAACCT TAATTCAAAG TTCCAAAAAG GTCTATCCTA CAAAGATCAG   
  
  
- ATTATTGGAC CTAGTGTTCA AATTTTTCAA TTGTACCCAA CTATATAAAA AAAATATTTT TTTATCTGAA   
  
  
- ACACTAAAAG AAACAAAATA AAAGATAGAT TAATAGAACT ACAATATTGT ATCCGGCGTA CAAACCGCCC   
  
  
- TATTGAACTC AATCGAATTG GGACTAATGA TCCTAATATC CAAACAGTAT AATTGAACTC AACTGATTTG   
  
  
- GACTAAAAAA ATATTAAAAC AAATAGATTA AAATAAGAAA GTATAAATTA CCTGAATCTT AATTCAAAAC   
  
  
- AAGAAATAGT AGAAAATTTT TTTTACAAAA AAATGCAATA GTACTAAAAA AAAAATTAAA TATAGTAAAC   
  
  
- AATAACAATA GATAAATACT AGATAAATTT TATTTTAATT GAAGAAATTT AATCAATTTA GATAATAAAC   
  
  
- TTAGTGCTTT TTTTTATCAG TGGCGTACTG CTTCCAGGTT TTGATCGGTA CTCCATAAGG AGCTAATTAT   
  
  
- AGTTTCGGGT TAACAAATTC GTCATTCAGC CAATAGCCTT CAGTTAAACC TGTGCGAGAC AGAGAGAATA   
  
  
- GATTACATCT GTACTGCCAC AAGATTCGAA CATTCCGGAA CAGGAACCGA GGTACCTTGG AAGTTAAATA   
  
  
- GTCAGTTTAG ATGTCACACT TGCATATAAT AAGCTAAGAC ATAAATTAAA AACCATGGTG CGGAGAAATT   
  
  
- TTCTTTTATT TTCCAATGTT TTAAGTGTTT TGCAAAAAT

+     TGACG-motif

| Site Name | Organism | Position | Strand | Matrix score. | sequence | function |
| --- | --- | --- | --- | --- | --- | --- |
| TGACG-motif | Hordeum vulgare | 1833 | + | 5 | TGACG | cis-acting regulatory element involved in the MeJA-responsiveness |
| TGACG-motif | Hordeum vulgare | 1707 | + | 5 | TGACG | cis-acting regulatory element involved in the MeJA-responsiveness |

>Potri.005G195000.1   
+ TCAACACTTT AAAATGATAT GAAAATACTA AAAATATATT AATTTAAATA AAAAAATTAA TATTTTTAAA   
  
  
+ ACATAACAGA TCTATTTGTA TTGTTCACAG GAAAAATGTA ATAATGTTTT TGTCTTTCTG AAAAAAAAAT   
  
  
+ ATTAAATTGG TTATTGGAGG CATGGAAGTT TTTTCATAAT GGGCACTAGT TGTTAACAAA TTAAATAGGG   
  
  
+ GCAAATAAGT CATTTCAAAA ATAGAGTGAG ATGACCTAAA TATCCTTATA AGAAAAGAAA TTAAAAGTGG   
  
  
+ TGTCCAAGAG CTTTATTGTA TTTTTCACAT GATTTTTTTA TTACTATAGG TCAACTAAGG GCCAATTTAG   
  
  
+ TATTTGTATA AATATATGGA AAAAAAAAGC ACAGTGAATG ATCAAAATAT TATTGGAAGC AAAAAAAAAT   
  
  
+ TAAAGTTAGC CTCGAGGCGA TGTTTTTGTA TTTTTACAAT GATTTTTTTT ATCACCAAGT GAACTTAGAG   
  
  
+ GGAATTCGGT ATTTTTCATA AATATAGAAA AATAAAAGTG GTTTGCGGGT GCATAGCAAG AGCCAACATG   
  
  
+ TGTGAGGTGT TTGGGCCTTC ATGTAACGCT TGCGACACCT CCCAACGTTC AAAATACCCT TTCTATCGTG   
  
  
+ TAATTGGAAG CGAAGCGATG TCCTTTTCCT TGCGGGGTGT TGCATGTGGT TGTTGGTGAT ACAGTTTGTT   
  
  
+ GCCGACAAAG CTTTCCCTCT ATATTTTCTC TCTCCTCCCT ACAAAATTTA CTCTGGCCAT GCAAAATTTT   
  
  
+ AGAATTGTCC TTTGATTTAT GGGGATTTCC ACTTAAGTCC TTATTTTTTA TTTCTAATTT TTATTTTTGG   
  
  
+ TCTTTTTGTA AAACTTTAAT TTTTTTTAAT TTCATCTTTA AATCTCAATT TCTAATATTT TTTATTTCAA   
  
  
+ CCTTAATCCT TATTCTTTTA ATTGTTTTTT TAATTTTTTT TGAGCCATTT GTTAAATTGA TATTTCTTAT   
  
  
+ CAATTTCACT CTTTAATAAA AAAATTCGTT TGCATTTCTT ATTTCAACCT TGATCCTCAT TCTTTTAGTT   
  
  
+ TTTTTAGTTT TTATCCTTTT GTTAAATTGT TTTTGCTTTT CAATTTCATT CTTATAGAAT TTATTTTATA   
  
  
+ TTTCAATTTT CATTCTTTTT TTTAATTGCT ATTTTTTAAT CATTTTGTAT AATTGAATTT TTTTTTTCAA   
  
  
+ TTTCATCCTT CAATATTTAA TTGATTTGGA ATTAAGTTTC AAGGTTTTTC CAGATAGGAT GTTTCTAGTC   
  
  
+ TAATAACCTG GATCACAAGT TTAAAAAGTT AACATGGGTT GATATATTTT TTTTATAAAA AAATAGACTT   
  
  
+ TGTGATTTTC TTTGTTTTAT TTTCTATCTA ATTATCTTGA TGTTATAACA TAGGCCGCAT GTTTGGCGGG   
  
  
+ ATAACTTGAG TTAGCTTAAC CCTGATTACT AGGATTATAG GTTTGTCATA TTAACTTGAG TTGACTAAAC   
  
  
+ CTGATTTTTT TATAATTTTG TTTATCTAAT TTTATTCTTT CATATTTAAT GGACTTAGAA TTAAGTTTTG   
  
  
+ TTCTTTATCA TCTTTTAAAA AAAATGTTTT TTTACGTTAT CATGATTTTT TTTTTAATTT ATATCATTTG   
  
  
+ TTATTGTTAT CTATTTATGA TCTATTTAAA ATAAAATTAA CTTCTTTAAA TTAGTTAAAT CTATTATTTG   
  
  
+ AATCACGAAA AAAAATAGTC ACCGCATGAC GAAGGTCCAA AACTAGCCAT GAGGTATTCC TCGATTAATA   
  
  
+ TCAAAGCCCA ATTGTTTAAG CAGTAAGTCG GTTATCGGAA GTCAATTTGG ACACGCTCTG TCTCTCTTAT   
  
  
+ CTAATGTAGA CATGACGGTG TTCTAAGCTT GTAAGGCCTT GTCCTTGGCT CCATGGAACC TTCAATTTAT   
  
  
+ CAGTCAAATC TACAGTGTGA ACGTATATTA TTCGATTCTG TATTTAATTT TTGGTACCAC GCCTCTTTAA   
  
  
+ AAGAAAATAA AAGGTTACAA AATTCACAAA ACGTTTTTA  

- AGTTGTGAAA TTTTACTATA CTTTTATGAT TTTTATATAA TTAAATTTAT TTTTTTAATT ATAAAAATTT   
  
  
- TGTATTGTCT AGATAAACAT AACAAGTGTC CTTTTTACAT TATTACAAAA ACAGAAAGAC TTTTTTTTTA   
  
  
- TAATTTAACC AATAACCTCC GTACCTTCAA AAAAGTATTA CCCGTGATCA ACAATTGTTT AATTTATCCC   
  
  
- CGTTTATTCA GTAAAGTTTT TATCTCACTC TACTGGATTT ATAGGAATAT TCTTTTCTTT AATTTTCACC   
  
  
- ACAGGTTCTC GAAATAACAT AAAAAGTGTA CTAAAAAAAT AATGATATCC AGTTGATTCC CGGTTAAATC   
  
  
- ATAAACATAT TTATATACCT TTTTTTTTCG TGTCACTTAC TAGTTTTATA ATAACCTTCG TTTTTTTTTA   
  
  
- ATTTCAATCG GAGCTCCGCT ACAAAAACAT AAAAATGTTA CTAAAAAAAA TAGTGGTTCA CTTGAATCTC   
  
  
- CCTTAAGCCA TAAAAAGTAT TTATATCTTT TTATTTTCAC CAAACGCCCA CGTATCGTTC TCGGTTGTAC   
  
  
- ACACTCCACA AACCCGGAAG TACATTGCGA ACGCTGTGGA GGGTTGCAAG TTTTATGGGA AAGATAGCAC   
  
  
- ATTAACCTTC GCTTCGCTAC AGGAAAAGGA ACGCCCCACA ACGTACACCA ACAACCACTA TGTCAAACAA   
  
  
- CGGCTGTTTC GAAAGGGAGA TATAAAAGAG AGAGGAGGGA TGTTTTAAAT GAGACCGGTA CGTTTTAAAA   
  
  
- TCTTAACAGG AAACTAAATA CCCCTAAAGG TGAATTCAGG AATAAAAAAT AAAGATTAAA AATAAAAACC   
  
  
- AGAAAAACAT TTTGAAATTA AAAAAAATTA AAGTAGAAAT TTAGAGTTAA AGATTATAAA AAATAAAGTT   
  
  
- GGAATTAGGA ATAAGAAAAT TAACAAAAAA ATTAAAAAAA ACTCGGTAAA CAATTTAACT ATAAAGAATA   
  
  
- GTTAAAGTGA GAAATTATTT TTTTAAGCAA ACGTAAAGAA TAAAGTTGGA ACTAGGAGTA AGAAAATCAA   
  
  
- AAAAATCAAA AATAGGAAAA CAATTTAACA AAAACGAAAA GTTAAAGTAA GAATATCTTA AATAAAATAT   
  
  
- AAAGTTAAAA GTAAGAAAAA AAATTAACGA TAAAAAATTA GTAAAACATA TTAACTTAAA AAAAAAAGTT   
  
  
- AAAGTAGGAA GTTATAAATT AACTAAACCT TAATTCAAAG TTCCAAAAAG GTCTATCCTA CAAAGATCAG   
  
  
- ATTATTGGAC CTAGTGTTCA AATTTTTCAA TTGTACCCAA CTATATAAAA AAAATATTTT TTTATCTGAA   
  
  
- ACACTAAAAG AAACAAAATA AAAGATAGAT TAATAGAACT ACAATATTGT ATCCGGCGTA CAAACCGCCC   
  
  
- TATTGAACTC AATCGAATTG GGACTAATGA TCCTAATATC CAAACAGTAT AATTGAACTC AACTGATTTG   
  
  
- GACTAAAAAA ATATTAAAAC AAATAGATTA AAATAAGAAA GTATAAATTA CCTGAATCTT AATTCAAAAC   
  
  
- AAGAAATAGT AGAAAATTTT TTTTACAAAA AAATGCAATA GTACTAAAAA AAAAATTAAA TATAGTAAAC   
  
  
- AATAACAATA GATAAATACT AGATAAATTT TATTTTAATT GAAGAAATTT AATCAATTTA GATAATAAAC   
  
  
- TTAGTGCTTT TTTTTATCAG TGGCGTACTG CTTCCAGGTT TTGATCGGTA CTCCATAAGG AGCTAATTAT   
  
  
- AGTTTCGGGT TAACAAATTC GTCATTCAGC CAATAGCCTT CAGTTAAACC TGTGCGAGAC AGAGAGAATA   
  
  
- GATTACATCT GTACTGCCAC AAGATTCGAA CATTCCGGAA CAGGAACCGA GGTACCTTGG AAGTTAAATA   
  
  
- GTCAGTTTAG ATGTCACACT TGCATATAAT AAGCTAAGAC ATAAATTAAA AACCATGGTG CGGAGAAATT   
  
  
- TTCTTTTATT TTCCAATGTT TTAAGTGTTT TGCAAAAAT

+     Unnamed\_\_1

| Site Name | Organism | Position | Strand | Matrix score. | sequence | function |
| --- | --- | --- | --- | --- | --- | --- |
| Unnamed\_\_1 | Zea mays | 1947 | - | 5 | CGTGG |  |

>Potri.005G195000.1   
+ TCAACACTTT AAAATGATAT GAAAATACTA AAAATATATT AATTTAAATA AAAAAATTAA TATTTTTAAA   
  
  
+ ACATAACAGA TCTATTTGTA TTGTTCACAG GAAAAATGTA ATAATGTTTT TGTCTTTCTG AAAAAAAAAT   
  
  
+ ATTAAATTGG TTATTGGAGG CATGGAAGTT TTTTCATAAT GGGCACTAGT TGTTAACAAA TTAAATAGGG   
  
  
+ GCAAATAAGT CATTTCAAAA ATAGAGTGAG ATGACCTAAA TATCCTTATA AGAAAAGAAA TTAAAAGTGG   
  
  
+ TGTCCAAGAG CTTTATTGTA TTTTTCACAT GATTTTTTTA TTACTATAGG TCAACTAAGG GCCAATTTAG   
  
  
+ TATTTGTATA AATATATGGA AAAAAAAAGC ACAGTGAATG ATCAAAATAT TATTGGAAGC AAAAAAAAAT   
  
  
+ TAAAGTTAGC CTCGAGGCGA TGTTTTTGTA TTTTTACAAT GATTTTTTTT ATCACCAAGT GAACTTAGAG   
  
  
+ GGAATTCGGT ATTTTTCATA AATATAGAAA AATAAAAGTG GTTTGCGGGT GCATAGCAAG AGCCAACATG   
  
  
+ TGTGAGGTGT TTGGGCCTTC ATGTAACGCT TGCGACACCT CCCAACGTTC AAAATACCCT TTCTATCGTG   
  
  
+ TAATTGGAAG CGAAGCGATG TCCTTTTCCT TGCGGGGTGT TGCATGTGGT TGTTGGTGAT ACAGTTTGTT   
  
  
+ GCCGACAAAG CTTTCCCTCT ATATTTTCTC TCTCCTCCCT ACAAAATTTA CTCTGGCCAT GCAAAATTTT   
  
  
+ AGAATTGTCC TTTGATTTAT GGGGATTTCC ACTTAAGTCC TTATTTTTTA TTTCTAATTT TTATTTTTGG   
  
  
+ TCTTTTTGTA AAACTTTAAT TTTTTTTAAT TTCATCTTTA AATCTCAATT TCTAATATTT TTTATTTCAA   
  
  
+ CCTTAATCCT TATTCTTTTA ATTGTTTTTT TAATTTTTTT TGAGCCATTT GTTAAATTGA TATTTCTTAT   
  
  
+ CAATTTCACT CTTTAATAAA AAAATTCGTT TGCATTTCTT ATTTCAACCT TGATCCTCAT TCTTTTAGTT   
  
  
+ TTTTTAGTTT TTATCCTTTT GTTAAATTGT TTTTGCTTTT CAATTTCATT CTTATAGAAT TTATTTTATA   
  
  
+ TTTCAATTTT CATTCTTTTT TTTAATTGCT ATTTTTTAAT CATTTTGTAT AATTGAATTT TTTTTTTCAA   
  
  
+ TTTCATCCTT CAATATTTAA TTGATTTGGA ATTAAGTTTC AAGGTTTTTC CAGATAGGAT GTTTCTAGTC   
  
  
+ TAATAACCTG GATCACAAGT TTAAAAAGTT AACATGGGTT GATATATTTT TTTTATAAAA AAATAGACTT   
  
  
+ TGTGATTTTC TTTGTTTTAT TTTCTATCTA ATTATCTTGA TGTTATAACA TAGGCCGCAT GTTTGGCGGG   
  
  
+ ATAACTTGAG TTAGCTTAAC CCTGATTACT AGGATTATAG GTTTGTCATA TTAACTTGAG TTGACTAAAC   
  
  
+ CTGATTTTTT TATAATTTTG TTTATCTAAT TTTATTCTTT CATATTTAAT GGACTTAGAA TTAAGTTTTG   
  
  
+ TTCTTTATCA TCTTTTAAAA AAAATGTTTT TTTACGTTAT CATGATTTTT TTTTTAATTT ATATCATTTG   
  
  
+ TTATTGTTAT CTATTTATGA TCTATTTAAA ATAAAATTAA CTTCTTTAAA TTAGTTAAAT CTATTATTTG   
  
  
+ AATCACGAAA AAAAATAGTC ACCGCATGAC GAAGGTCCAA AACTAGCCAT GAGGTATTCC TCGATTAATA   
  
  
+ TCAAAGCCCA ATTGTTTAAG CAGTAAGTCG GTTATCGGAA GTCAATTTGG ACACGCTCTG TCTCTCTTAT   
  
  
+ CTAATGTAGA CATGACGGTG TTCTAAGCTT GTAAGGCCTT GTCCTTGGCT CCATGGAACC TTCAATTTAT   
  
  
+ CAGTCAAATC TACAGTGTGA ACGTATATTA TTCGATTCTG TATTTAATTT TTGGTACCAC GCCTCTTTAA   
  
  
+ AAGAAAATAA AAGGTTACAA AATTCACAAA ACGTTTTTA  

- AGTTGTGAAA TTTTACTATA CTTTTATGAT TTTTATATAA TTAAATTTAT TTTTTTAATT ATAAAAATTT   
  
  
- TGTATTGTCT AGATAAACAT AACAAGTGTC CTTTTTACAT TATTACAAAA ACAGAAAGAC TTTTTTTTTA   
  
  
- TAATTTAACC AATAACCTCC GTACCTTCAA AAAAGTATTA CCCGTGATCA ACAATTGTTT AATTTATCCC   
  
  
- CGTTTATTCA GTAAAGTTTT TATCTCACTC TACTGGATTT ATAGGAATAT TCTTTTCTTT AATTTTCACC   
  
  
- ACAGGTTCTC GAAATAACAT AAAAAGTGTA CTAAAAAAAT AATGATATCC AGTTGATTCC CGGTTAAATC   
  
  
- ATAAACATAT TTATATACCT TTTTTTTTCG TGTCACTTAC TAGTTTTATA ATAACCTTCG TTTTTTTTTA   
  
  
- ATTTCAATCG GAGCTCCGCT ACAAAAACAT AAAAATGTTA CTAAAAAAAA TAGTGGTTCA CTTGAATCTC   
  
  
- CCTTAAGCCA TAAAAAGTAT TTATATCTTT TTATTTTCAC CAAACGCCCA CGTATCGTTC TCGGTTGTAC   
  
  
- ACACTCCACA AACCCGGAAG TACATTGCGA ACGCTGTGGA GGGTTGCAAG TTTTATGGGA AAGATAGCAC   
  
  
- ATTAACCTTC GCTTCGCTAC AGGAAAAGGA ACGCCCCACA ACGTACACCA ACAACCACTA TGTCAAACAA   
  
  
- CGGCTGTTTC GAAAGGGAGA TATAAAAGAG AGAGGAGGGA TGTTTTAAAT GAGACCGGTA CGTTTTAAAA   
  
  
- TCTTAACAGG AAACTAAATA CCCCTAAAGG TGAATTCAGG AATAAAAAAT AAAGATTAAA AATAAAAACC   
  
  
- AGAAAAACAT TTTGAAATTA AAAAAAATTA AAGTAGAAAT TTAGAGTTAA AGATTATAAA AAATAAAGTT   
  
  
- GGAATTAGGA ATAAGAAAAT TAACAAAAAA ATTAAAAAAA ACTCGGTAAA CAATTTAACT ATAAAGAATA   
  
  
- GTTAAAGTGA GAAATTATTT TTTTAAGCAA ACGTAAAGAA TAAAGTTGGA ACTAGGAGTA AGAAAATCAA   
  
  
- AAAAATCAAA AATAGGAAAA CAATTTAACA AAAACGAAAA GTTAAAGTAA GAATATCTTA AATAAAATAT   
  
  
- AAAGTTAAAA GTAAGAAAAA AAATTAACGA TAAAAAATTA GTAAAACATA TTAACTTAAA AAAAAAAGTT   
  
  
- AAAGTAGGAA GTTATAAATT AACTAAACCT TAATTCAAAG TTCCAAAAAG GTCTATCCTA CAAAGATCAG   
  
  
- ATTATTGGAC CTAGTGTTCA AATTTTTCAA TTGTACCCAA CTATATAAAA AAAATATTTT TTTATCTGAA   
  
  
- ACACTAAAAG AAACAAAATA AAAGATAGAT TAATAGAACT ACAATATTGT ATCCGGCGTA CAAACCGCCC   
  
  
- TATTGAACTC AATCGAATTG GGACTAATGA TCCTAATATC CAAACAGTAT AATTGAACTC AACTGATTTG   
  
  
- GACTAAAAAA ATATTAAAAC AAATAGATTA AAATAAGAAA GTATAAATTA CCTGAATCTT AATTCAAAAC   
  
  
- AAGAAATAGT AGAAAATTTT TTTTACAAAA AAATGCAATA GTACTAAAAA AAAAATTAAA TATAGTAAAC   
  
  
- AATAACAATA GATAAATACT AGATAAATTT TATTTTAATT GAAGAAATTT AATCAATTTA GATAATAAAC   
  
  
- TTAGTGCTTT TTTTTATCAG TGGCGTACTG CTTCCAGGTT TTGATCGGTA CTCCATAAGG AGCTAATTAT   
  
  
- AGTTTCGGGT TAACAAATTC GTCATTCAGC CAATAGCCTT CAGTTAAACC TGTGCGAGAC AGAGAGAATA   
  
  
- GATTACATCT GTACTGCCAC AAGATTCGAA CATTCCGGAA CAGGAACCGA GGTACCTTGG AAGTTAAATA   
  
  
- GTCAGTTTAG ATGTCACACT TGCATATAAT AAGCTAAGAC ATAAATTAAA AACCATGGTG CGGAGAAATT   
  
  
- TTCTTTTATT TTCCAATGTT TTAAGTGTTT TGCAAAAAT

+     Unnamed\_\_4

| Site Name | Organism | Position | Strand | Matrix score. | sequence | function |
| --- | --- | --- | --- | --- | --- | --- |
| Unnamed\_\_4 | Petroselinum hortense | 1869 | + | 4 | CTCC |  |
| Unnamed\_\_4 | Petroselinum hortense | 735 | + | 4 | CTCC |  |
| Unnamed\_\_4 | Petroselinum hortense | 732 | + | 4 | CTCC |  |
| Unnamed\_\_4 | Petroselinum hortense | 599 | + | 4 | CTCC |  |
| Unnamed\_\_4 | Petroselinum hortense | 156 | - | 4 | CTCC |  |

>Potri.005G195000.1   
+ TCAACACTTT AAAATGATAT GAAAATACTA AAAATATATT AATTTAAATA AAAAAATTAA TATTTTTAAA   
  
  
+ ACATAACAGA TCTATTTGTA TTGTTCACAG GAAAAATGTA ATAATGTTTT TGTCTTTCTG AAAAAAAAAT   
  
  
+ ATTAAATTGG TTATTGGAGG CATGGAAGTT TTTTCATAAT GGGCACTAGT TGTTAACAAA TTAAATAGGG   
  
  
+ GCAAATAAGT CATTTCAAAA ATAGAGTGAG ATGACCTAAA TATCCTTATA AGAAAAGAAA TTAAAAGTGG   
  
  
+ TGTCCAAGAG CTTTATTGTA TTTTTCACAT GATTTTTTTA TTACTATAGG TCAACTAAGG GCCAATTTAG   
  
  
+ TATTTGTATA AATATATGGA AAAAAAAAGC ACAGTGAATG ATCAAAATAT TATTGGAAGC AAAAAAAAAT   
  
  
+ TAAAGTTAGC CTCGAGGCGA TGTTTTTGTA TTTTTACAAT GATTTTTTTT ATCACCAAGT GAACTTAGAG   
  
  
+ GGAATTCGGT ATTTTTCATA AATATAGAAA AATAAAAGTG GTTTGCGGGT GCATAGCAAG AGCCAACATG   
  
  
+ TGTGAGGTGT TTGGGCCTTC ATGTAACGCT TGCGACACCT CCCAACGTTC AAAATACCCT TTCTATCGTG   
  
  
+ TAATTGGAAG CGAAGCGATG TCCTTTTCCT TGCGGGGTGT TGCATGTGGT TGTTGGTGAT ACAGTTTGTT   
  
  
+ GCCGACAAAG CTTTCCCTCT ATATTTTCTC TCTCCTCCCT ACAAAATTTA CTCTGGCCAT GCAAAATTTT   
  
  
+ AGAATTGTCC TTTGATTTAT GGGGATTTCC ACTTAAGTCC TTATTTTTTA TTTCTAATTT TTATTTTTGG   
  
  
+ TCTTTTTGTA AAACTTTAAT TTTTTTTAAT TTCATCTTTA AATCTCAATT TCTAATATTT TTTATTTCAA   
  
  
+ CCTTAATCCT TATTCTTTTA ATTGTTTTTT TAATTTTTTT TGAGCCATTT GTTAAATTGA TATTTCTTAT   
  
  
+ CAATTTCACT CTTTAATAAA AAAATTCGTT TGCATTTCTT ATTTCAACCT TGATCCTCAT TCTTTTAGTT   
  
  
+ TTTTTAGTTT TTATCCTTTT GTTAAATTGT TTTTGCTTTT CAATTTCATT CTTATAGAAT TTATTTTATA   
  
  
+ TTTCAATTTT CATTCTTTTT TTTAATTGCT ATTTTTTAAT CATTTTGTAT AATTGAATTT TTTTTTTCAA   
  
  
+ TTTCATCCTT CAATATTTAA TTGATTTGGA ATTAAGTTTC AAGGTTTTTC CAGATAGGAT GTTTCTAGTC   
  
  
+ TAATAACCTG GATCACAAGT TTAAAAAGTT AACATGGGTT GATATATTTT TTTTATAAAA AAATAGACTT   
  
  
+ TGTGATTTTC TTTGTTTTAT TTTCTATCTA ATTATCTTGA TGTTATAACA TAGGCCGCAT GTTTGGCGGG   
  
  
+ ATAACTTGAG TTAGCTTAAC CCTGATTACT AGGATTATAG GTTTGTCATA TTAACTTGAG TTGACTAAAC   
  
  
+ CTGATTTTTT TATAATTTTG TTTATCTAAT TTTATTCTTT CATATTTAAT GGACTTAGAA TTAAGTTTTG   
  
  
+ TTCTTTATCA TCTTTTAAAA AAAATGTTTT TTTACGTTAT CATGATTTTT TTTTTAATTT ATATCATTTG   
  
  
+ TTATTGTTAT CTATTTATGA TCTATTTAAA ATAAAATTAA CTTCTTTAAA TTAGTTAAAT CTATTATTTG   
  
  
+ AATCACGAAA AAAAATAGTC ACCGCATGAC GAAGGTCCAA AACTAGCCAT GAGGTATTCC TCGATTAATA   
  
  
+ TCAAAGCCCA ATTGTTTAAG CAGTAAGTCG GTTATCGGAA GTCAATTTGG ACACGCTCTG TCTCTCTTAT   
  
  
+ CTAATGTAGA CATGACGGTG TTCTAAGCTT GTAAGGCCTT GTCCTTGGCT CCATGGAACC TTCAATTTAT   
  
  
+ CAGTCAAATC TACAGTGTGA ACGTATATTA TTCGATTCTG TATTTAATTT TTGGTACCAC GCCTCTTTAA   
  
  
+ AAGAAAATAA AAGGTTACAA AATTCACAAA ACGTTTTTA  

- AGTTGTGAAA TTTTACTATA CTTTTATGAT TTTTATATAA TTAAATTTAT TTTTTTAATT ATAAAAATTT   
  
  
- TGTATTGTCT AGATAAACAT AACAAGTGTC CTTTTTACAT TATTACAAAA ACAGAAAGAC TTTTTTTTTA   
  
  
- TAATTTAACC AATAACCTCC GTACCTTCAA AAAAGTATTA CCCGTGATCA ACAATTGTTT AATTTATCCC   
  
  
- CGTTTATTCA GTAAAGTTTT TATCTCACTC TACTGGATTT ATAGGAATAT TCTTTTCTTT AATTTTCACC   
  
  
- ACAGGTTCTC GAAATAACAT AAAAAGTGTA CTAAAAAAAT AATGATATCC AGTTGATTCC CGGTTAAATC   
  
  
- ATAAACATAT TTATATACCT TTTTTTTTCG TGTCACTTAC TAGTTTTATA ATAACCTTCG TTTTTTTTTA   
  
  
- ATTTCAATCG GAGCTCCGCT ACAAAAACAT AAAAATGTTA CTAAAAAAAA TAGTGGTTCA CTTGAATCTC   
  
  
- CCTTAAGCCA TAAAAAGTAT TTATATCTTT TTATTTTCAC CAAACGCCCA CGTATCGTTC TCGGTTGTAC   
  
  
- ACACTCCACA AACCCGGAAG TACATTGCGA ACGCTGTGGA GGGTTGCAAG TTTTATGGGA AAGATAGCAC   
  
  
- ATTAACCTTC GCTTCGCTAC AGGAAAAGGA ACGCCCCACA ACGTACACCA ACAACCACTA TGTCAAACAA   
  
  
- CGGCTGTTTC GAAAGGGAGA TATAAAAGAG AGAGGAGGGA TGTTTTAAAT GAGACCGGTA CGTTTTAAAA   
  
  
- TCTTAACAGG AAACTAAATA CCCCTAAAGG TGAATTCAGG AATAAAAAAT AAAGATTAAA AATAAAAACC   
  
  
- AGAAAAACAT TTTGAAATTA AAAAAAATTA AAGTAGAAAT TTAGAGTTAA AGATTATAAA AAATAAAGTT   
  
  
- GGAATTAGGA ATAAGAAAAT TAACAAAAAA ATTAAAAAAA ACTCGGTAAA CAATTTAACT ATAAAGAATA   
  
  
- GTTAAAGTGA GAAATTATTT TTTTAAGCAA ACGTAAAGAA TAAAGTTGGA ACTAGGAGTA AGAAAATCAA   
  
  
- AAAAATCAAA AATAGGAAAA CAATTTAACA AAAACGAAAA GTTAAAGTAA GAATATCTTA AATAAAATAT   
  
  
- AAAGTTAAAA GTAAGAAAAA AAATTAACGA TAAAAAATTA GTAAAACATA TTAACTTAAA AAAAAAAGTT   
  
  
- AAAGTAGGAA GTTATAAATT AACTAAACCT TAATTCAAAG TTCCAAAAAG GTCTATCCTA CAAAGATCAG   
  
  
- ATTATTGGAC CTAGTGTTCA AATTTTTCAA TTGTACCCAA CTATATAAAA AAAATATTTT TTTATCTGAA   
  
  
- ACACTAAAAG AAACAAAATA AAAGATAGAT TAATAGAACT ACAATATTGT ATCCGGCGTA CAAACCGCCC   
  
  
- TATTGAACTC AATCGAATTG GGACTAATGA TCCTAATATC CAAACAGTAT AATTGAACTC AACTGATTTG   
  
  
- GACTAAAAAA ATATTAAAAC AAATAGATTA AAATAAGAAA GTATAAATTA CCTGAATCTT AATTCAAAAC   
  
  
- AAGAAATAGT AGAAAATTTT TTTTACAAAA AAATGCAATA GTACTAAAAA AAAAATTAAA TATAGTAAAC   
  
  
- AATAACAATA GATAAATACT AGATAAATTT TATTTTAATT GAAGAAATTT AATCAATTTA GATAATAAAC   
  
  
- TTAGTGCTTT TTTTTATCAG TGGCGTACTG CTTCCAGGTT TTGATCGGTA CTCCATAAGG AGCTAATTAT   
  
  
- AGTTTCGGGT TAACAAATTC GTCATTCAGC CAATAGCCTT CAGTTAAACC TGTGCGAGAC AGAGAGAATA   
  
  
- GATTACATCT GTACTGCCAC AAGATTCGAA CATTCCGGAA CAGGAACCGA GGTACCTTGG AAGTTAAATA   
  
  
- GTCAGTTTAG ATGTCACACT TGCATATAAT AAGCTAAGAC ATAAATTAAA AACCATGGTG CGGAGAAATT   
  
  
- TTCTTTTATT TTCCAATGTT TTAAGTGTTT TGCAAAAAT

+     Unnamed\_\_6

| Site Name | Organism | Position | Strand | Matrix score. | sequence | function |
| --- | --- | --- | --- | --- | --- | --- |
| Unnamed\_\_6 | Zea mays | 357 | + | 10 | taTAAATATct |  |

>Potri.005G195000.1   
+ TCAACACTTT AAAATGATAT GAAAATACTA AAAATATATT AATTTAAATA AAAAAATTAA TATTTTTAAA   
  
  
+ ACATAACAGA TCTATTTGTA TTGTTCACAG GAAAAATGTA ATAATGTTTT TGTCTTTCTG AAAAAAAAAT   
  
  
+ ATTAAATTGG TTATTGGAGG CATGGAAGTT TTTTCATAAT GGGCACTAGT TGTTAACAAA TTAAATAGGG   
  
  
+ GCAAATAAGT CATTTCAAAA ATAGAGTGAG ATGACCTAAA TATCCTTATA AGAAAAGAAA TTAAAAGTGG   
  
  
+ TGTCCAAGAG CTTTATTGTA TTTTTCACAT GATTTTTTTA TTACTATAGG TCAACTAAGG GCCAATTTAG   
  
  
+ TATTTGTATA AATATATGGA AAAAAAAAGC ACAGTGAATG ATCAAAATAT TATTGGAAGC AAAAAAAAAT   
  
  
+ TAAAGTTAGC CTCGAGGCGA TGTTTTTGTA TTTTTACAAT GATTTTTTTT ATCACCAAGT GAACTTAGAG   
  
  
+ GGAATTCGGT ATTTTTCATA AATATAGAAA AATAAAAGTG GTTTGCGGGT GCATAGCAAG AGCCAACATG   
  
  
+ TGTGAGGTGT TTGGGCCTTC ATGTAACGCT TGCGACACCT CCCAACGTTC AAAATACCCT TTCTATCGTG   
  
  
+ TAATTGGAAG CGAAGCGATG TCCTTTTCCT TGCGGGGTGT TGCATGTGGT TGTTGGTGAT ACAGTTTGTT   
  
  
+ GCCGACAAAG CTTTCCCTCT ATATTTTCTC TCTCCTCCCT ACAAAATTTA CTCTGGCCAT GCAAAATTTT   
  
  
+ AGAATTGTCC TTTGATTTAT GGGGATTTCC ACTTAAGTCC TTATTTTTTA TTTCTAATTT TTATTTTTGG   
  
  
+ TCTTTTTGTA AAACTTTAAT TTTTTTTAAT TTCATCTTTA AATCTCAATT TCTAATATTT TTTATTTCAA   
  
  
+ CCTTAATCCT TATTCTTTTA ATTGTTTTTT TAATTTTTTT TGAGCCATTT GTTAAATTGA TATTTCTTAT   
  
  
+ CAATTTCACT CTTTAATAAA AAAATTCGTT TGCATTTCTT ATTTCAACCT TGATCCTCAT TCTTTTAGTT   
  
  
+ TTTTTAGTTT TTATCCTTTT GTTAAATTGT TTTTGCTTTT CAATTTCATT CTTATAGAAT TTATTTTATA   
  
  
+ TTTCAATTTT CATTCTTTTT TTTAATTGCT ATTTTTTAAT CATTTTGTAT AATTGAATTT TTTTTTTCAA   
  
  
+ TTTCATCCTT CAATATTTAA TTGATTTGGA ATTAAGTTTC AAGGTTTTTC CAGATAGGAT GTTTCTAGTC   
  
  
+ TAATAACCTG GATCACAAGT TTAAAAAGTT AACATGGGTT GATATATTTT TTTTATAAAA AAATAGACTT   
  
  
+ TGTGATTTTC TTTGTTTTAT TTTCTATCTA ATTATCTTGA TGTTATAACA TAGGCCGCAT GTTTGGCGGG   
  
  
+ ATAACTTGAG TTAGCTTAAC CCTGATTACT AGGATTATAG GTTTGTCATA TTAACTTGAG TTGACTAAAC   
  
  
+ CTGATTTTTT TATAATTTTG TTTATCTAAT TTTATTCTTT CATATTTAAT GGACTTAGAA TTAAGTTTTG   
  
  
+ TTCTTTATCA TCTTTTAAAA AAAATGTTTT TTTACGTTAT CATGATTTTT TTTTTAATTT ATATCATTTG   
  
  
+ TTATTGTTAT CTATTTATGA TCTATTTAAA ATAAAATTAA CTTCTTTAAA TTAGTTAAAT CTATTATTTG   
  
  
+ AATCACGAAA AAAAATAGTC ACCGCATGAC GAAGGTCCAA AACTAGCCAT GAGGTATTCC TCGATTAATA   
  
  
+ TCAAAGCCCA ATTGTTTAAG CAGTAAGTCG GTTATCGGAA GTCAATTTGG ACACGCTCTG TCTCTCTTAT   
  
  
+ CTAATGTAGA CATGACGGTG TTCTAAGCTT GTAAGGCCTT GTCCTTGGCT CCATGGAACC TTCAATTTAT   
  
  
+ CAGTCAAATC TACAGTGTGA ACGTATATTA TTCGATTCTG TATTTAATTT TTGGTACCAC GCCTCTTTAA   
  
  
+ AAGAAAATAA AAGGTTACAA AATTCACAAA ACGTTTTTA  

- AGTTGTGAAA TTTTACTATA CTTTTATGAT TTTTATATAA TTAAATTTAT TTTTTTAATT ATAAAAATTT   
  
  
- TGTATTGTCT AGATAAACAT AACAAGTGTC CTTTTTACAT TATTACAAAA ACAGAAAGAC TTTTTTTTTA   
  
  
- TAATTTAACC AATAACCTCC GTACCTTCAA AAAAGTATTA CCCGTGATCA ACAATTGTTT AATTTATCCC   
  
  
- CGTTTATTCA GTAAAGTTTT TATCTCACTC TACTGGATTT ATAGGAATAT TCTTTTCTTT AATTTTCACC   
  
  
- ACAGGTTCTC GAAATAACAT AAAAAGTGTA CTAAAAAAAT AATGATATCC AGTTGATTCC CGGTTAAATC   
  
  
- ATAAACATAT TTATATACCT TTTTTTTTCG TGTCACTTAC TAGTTTTATA ATAACCTTCG TTTTTTTTTA   
  
  
- ATTTCAATCG GAGCTCCGCT ACAAAAACAT AAAAATGTTA CTAAAAAAAA TAGTGGTTCA CTTGAATCTC   
  
  
- CCTTAAGCCA TAAAAAGTAT TTATATCTTT TTATTTTCAC CAAACGCCCA CGTATCGTTC TCGGTTGTAC   
  
  
- ACACTCCACA AACCCGGAAG TACATTGCGA ACGCTGTGGA GGGTTGCAAG TTTTATGGGA AAGATAGCAC   
  
  
- ATTAACCTTC GCTTCGCTAC AGGAAAAGGA ACGCCCCACA ACGTACACCA ACAACCACTA TGTCAAACAA   
  
  
- CGGCTGTTTC GAAAGGGAGA TATAAAAGAG AGAGGAGGGA TGTTTTAAAT GAGACCGGTA CGTTTTAAAA   
  
  
- TCTTAACAGG AAACTAAATA CCCCTAAAGG TGAATTCAGG AATAAAAAAT AAAGATTAAA AATAAAAACC   
  
  
- AGAAAAACAT TTTGAAATTA AAAAAAATTA AAGTAGAAAT TTAGAGTTAA AGATTATAAA AAATAAAGTT   
  
  
- GGAATTAGGA ATAAGAAAAT TAACAAAAAA ATTAAAAAAA ACTCGGTAAA CAATTTAACT ATAAAGAATA   
  
  
- GTTAAAGTGA GAAATTATTT TTTTAAGCAA ACGTAAAGAA TAAAGTTGGA ACTAGGAGTA AGAAAATCAA   
  
  
- AAAAATCAAA AATAGGAAAA CAATTTAACA AAAACGAAAA GTTAAAGTAA GAATATCTTA AATAAAATAT   
  
  
- AAAGTTAAAA GTAAGAAAAA AAATTAACGA TAAAAAATTA GTAAAACATA TTAACTTAAA AAAAAAAGTT   
  
  
- AAAGTAGGAA GTTATAAATT AACTAAACCT TAATTCAAAG TTCCAAAAAG GTCTATCCTA CAAAGATCAG   
  
  
- ATTATTGGAC CTAGTGTTCA AATTTTTCAA TTGTACCCAA CTATATAAAA AAAATATTTT TTTATCTGAA   
  
  
- ACACTAAAAG AAACAAAATA AAAGATAGAT TAATAGAACT ACAATATTGT ATCCGGCGTA CAAACCGCCC   
  
  
- TATTGAACTC AATCGAATTG GGACTAATGA TCCTAATATC CAAACAGTAT AATTGAACTC AACTGATTTG   
  
  
- GACTAAAAAA ATATTAAAAC AAATAGATTA AAATAAGAAA GTATAAATTA CCTGAATCTT AATTCAAAAC   
  
  
- AAGAAATAGT AGAAAATTTT TTTTACAAAA AAATGCAATA GTACTAAAAA AAAAATTAAA TATAGTAAAC   
  
  
- AATAACAATA GATAAATACT AGATAAATTT TATTTTAATT GAAGAAATTT AATCAATTTA GATAATAAAC   
  
  
- TTAGTGCTTT TTTTTATCAG TGGCGTACTG CTTCCAGGTT TTGATCGGTA CTCCATAAGG AGCTAATTAT   
  
  
- AGTTTCGGGT TAACAAATTC GTCATTCAGC CAATAGCCTT CAGTTAAACC TGTGCGAGAC AGAGAGAATA   
  
  
- GATTACATCT GTACTGCCAC AAGATTCGAA CATTCCGGAA CAGGAACCGA GGTACCTTGG AAGTTAAATA   
  
  
- GTCAGTTTAG ATGTCACACT TGCATATAAT AAGCTAAGAC ATAAATTAAA AACCATGGTG CGGAGAAATT   
  
  
- TTCTTTTATT TTCCAATGTT TTAAGTGTTT TGCAAAAAT

+     W box

| Site Name | Organism | Position | Strand | Matrix score. | sequence | function |
| --- | --- | --- | --- | --- | --- | --- |
| W box | Arabidopsis thaliana | 329 | - | 6 | TTGACC |  |

>Potri.005G195000.1   
+ TCAACACTTT AAAATGATAT GAAAATACTA AAAATATATT AATTTAAATA AAAAAATTAA TATTTTTAAA   
  
  
+ ACATAACAGA TCTATTTGTA TTGTTCACAG GAAAAATGTA ATAATGTTTT TGTCTTTCTG AAAAAAAAAT   
  
  
+ ATTAAATTGG TTATTGGAGG CATGGAAGTT TTTTCATAAT GGGCACTAGT TGTTAACAAA TTAAATAGGG   
  
  
+ GCAAATAAGT CATTTCAAAA ATAGAGTGAG ATGACCTAAA TATCCTTATA AGAAAAGAAA TTAAAAGTGG   
  
  
+ TGTCCAAGAG CTTTATTGTA TTTTTCACAT GATTTTTTTA TTACTATAGG TCAACTAAGG GCCAATTTAG   
  
  
+ TATTTGTATA AATATATGGA AAAAAAAAGC ACAGTGAATG ATCAAAATAT TATTGGAAGC AAAAAAAAAT   
  
  
+ TAAAGTTAGC CTCGAGGCGA TGTTTTTGTA TTTTTACAAT GATTTTTTTT ATCACCAAGT GAACTTAGAG   
  
  
+ GGAATTCGGT ATTTTTCATA AATATAGAAA AATAAAAGTG GTTTGCGGGT GCATAGCAAG AGCCAACATG   
  
  
+ TGTGAGGTGT TTGGGCCTTC ATGTAACGCT TGCGACACCT CCCAACGTTC AAAATACCCT TTCTATCGTG   
  
  
+ TAATTGGAAG CGAAGCGATG TCCTTTTCCT TGCGGGGTGT TGCATGTGGT TGTTGGTGAT ACAGTTTGTT   
  
  
+ GCCGACAAAG CTTTCCCTCT ATATTTTCTC TCTCCTCCCT ACAAAATTTA CTCTGGCCAT GCAAAATTTT   
  
  
+ AGAATTGTCC TTTGATTTAT GGGGATTTCC ACTTAAGTCC TTATTTTTTA TTTCTAATTT TTATTTTTGG   
  
  
+ TCTTTTTGTA AAACTTTAAT TTTTTTTAAT TTCATCTTTA AATCTCAATT TCTAATATTT TTTATTTCAA   
  
  
+ CCTTAATCCT TATTCTTTTA ATTGTTTTTT TAATTTTTTT TGAGCCATTT GTTAAATTGA TATTTCTTAT   
  
  
+ CAATTTCACT CTTTAATAAA AAAATTCGTT TGCATTTCTT ATTTCAACCT TGATCCTCAT TCTTTTAGTT   
  
  
+ TTTTTAGTTT TTATCCTTTT GTTAAATTGT TTTTGCTTTT CAATTTCATT CTTATAGAAT TTATTTTATA   
  
  
+ TTTCAATTTT CATTCTTTTT TTTAATTGCT ATTTTTTAAT CATTTTGTAT AATTGAATTT TTTTTTTCAA   
  
  
+ TTTCATCCTT CAATATTTAA TTGATTTGGA ATTAAGTTTC AAGGTTTTTC CAGATAGGAT GTTTCTAGTC   
  
  
+ TAATAACCTG GATCACAAGT TTAAAAAGTT AACATGGGTT GATATATTTT TTTTATAAAA AAATAGACTT   
  
  
+ TGTGATTTTC TTTGTTTTAT TTTCTATCTA ATTATCTTGA TGTTATAACA TAGGCCGCAT GTTTGGCGGG   
  
  
+ ATAACTTGAG TTAGCTTAAC CCTGATTACT AGGATTATAG GTTTGTCATA TTAACTTGAG TTGACTAAAC   
  
  
+ CTGATTTTTT TATAATTTTG TTTATCTAAT TTTATTCTTT CATATTTAAT GGACTTAGAA TTAAGTTTTG   
  
  
+ TTCTTTATCA TCTTTTAAAA AAAATGTTTT TTTACGTTAT CATGATTTTT TTTTTAATTT ATATCATTTG   
  
  
+ TTATTGTTAT CTATTTATGA TCTATTTAAA ATAAAATTAA CTTCTTTAAA TTAGTTAAAT CTATTATTTG   
  
  
+ AATCACGAAA AAAAATAGTC ACCGCATGAC GAAGGTCCAA AACTAGCCAT GAGGTATTCC TCGATTAATA   
  
  
+ TCAAAGCCCA ATTGTTTAAG CAGTAAGTCG GTTATCGGAA GTCAATTTGG ACACGCTCTG TCTCTCTTAT   
  
  
+ CTAATGTAGA CATGACGGTG TTCTAAGCTT GTAAGGCCTT GTCCTTGGCT CCATGGAACC TTCAATTTAT   
  
  
+ CAGTCAAATC TACAGTGTGA ACGTATATTA TTCGATTCTG TATTTAATTT TTGGTACCAC GCCTCTTTAA   
  
  
+ AAGAAAATAA AAGGTTACAA AATTCACAAA ACGTTTTTA  

- AGTTGTGAAA TTTTACTATA CTTTTATGAT TTTTATATAA TTAAATTTAT TTTTTTAATT ATAAAAATTT   
  
  
- TGTATTGTCT AGATAAACAT AACAAGTGTC CTTTTTACAT TATTACAAAA ACAGAAAGAC TTTTTTTTTA   
  
  
- TAATTTAACC AATAACCTCC GTACCTTCAA AAAAGTATTA CCCGTGATCA ACAATTGTTT AATTTATCCC   
  
  
- CGTTTATTCA GTAAAGTTTT TATCTCACTC TACTGGATTT ATAGGAATAT TCTTTTCTTT AATTTTCACC   
  
  
- ACAGGTTCTC GAAATAACAT AAAAAGTGTA CTAAAAAAAT AATGATATCC AGTTGATTCC CGGTTAAATC   
  
  
- ATAAACATAT TTATATACCT TTTTTTTTCG TGTCACTTAC TAGTTTTATA ATAACCTTCG TTTTTTTTTA   
  
  
- ATTTCAATCG GAGCTCCGCT ACAAAAACAT AAAAATGTTA CTAAAAAAAA TAGTGGTTCA CTTGAATCTC   
  
  
- CCTTAAGCCA TAAAAAGTAT TTATATCTTT TTATTTTCAC CAAACGCCCA CGTATCGTTC TCGGTTGTAC   
  
  
- ACACTCCACA AACCCGGAAG TACATTGCGA ACGCTGTGGA GGGTTGCAAG TTTTATGGGA AAGATAGCAC   
  
  
- ATTAACCTTC GCTTCGCTAC AGGAAAAGGA ACGCCCCACA ACGTACACCA ACAACCACTA TGTCAAACAA   
  
  
- CGGCTGTTTC GAAAGGGAGA TATAAAAGAG AGAGGAGGGA TGTTTTAAAT GAGACCGGTA CGTTTTAAAA   
  
  
- TCTTAACAGG AAACTAAATA CCCCTAAAGG TGAATTCAGG AATAAAAAAT AAAGATTAAA AATAAAAACC   
  
  
- AGAAAAACAT TTTGAAATTA AAAAAAATTA AAGTAGAAAT TTAGAGTTAA AGATTATAAA AAATAAAGTT   
  
  
- GGAATTAGGA ATAAGAAAAT TAACAAAAAA ATTAAAAAAA ACTCGGTAAA CAATTTAACT ATAAAGAATA   
  
  
- GTTAAAGTGA GAAATTATTT TTTTAAGCAA ACGTAAAGAA TAAAGTTGGA ACTAGGAGTA AGAAAATCAA   
  
  
- AAAAATCAAA AATAGGAAAA CAATTTAACA AAAACGAAAA GTTAAAGTAA GAATATCTTA AATAAAATAT   
  
  
- AAAGTTAAAA GTAAGAAAAA AAATTAACGA TAAAAAATTA GTAAAACATA TTAACTTAAA AAAAAAAGTT   
  
  
- AAAGTAGGAA GTTATAAATT AACTAAACCT TAATTCAAAG TTCCAAAAAG GTCTATCCTA CAAAGATCAG   
  
  
- ATTATTGGAC CTAGTGTTCA AATTTTTCAA TTGTACCCAA CTATATAAAA AAAATATTTT TTTATCTGAA   
  
  
- ACACTAAAAG AAACAAAATA AAAGATAGAT TAATAGAACT ACAATATTGT ATCCGGCGTA CAAACCGCCC   
  
  
- TATTGAACTC AATCGAATTG GGACTAATGA TCCTAATATC CAAACAGTAT AATTGAACTC AACTGATTTG   
  
  
- GACTAAAAAA ATATTAAAAC AAATAGATTA AAATAAGAAA GTATAAATTA CCTGAATCTT AATTCAAAAC   
  
  
- AAGAAATAGT AGAAAATTTT TTTTACAAAA AAATGCAATA GTACTAAAAA AAAAATTAAA TATAGTAAAC   
  
  
- AATAACAATA GATAAATACT AGATAAATTT TATTTTAATT GAAGAAATTT AATCAATTTA GATAATAAAC   
  
  
- TTAGTGCTTT TTTTTATCAG TGGCGTACTG CTTCCAGGTT TTGATCGGTA CTCCATAAGG AGCTAATTAT   
  
  
- AGTTTCGGGT TAACAAATTC GTCATTCAGC CAATAGCCTT CAGTTAAACC TGTGCGAGAC AGAGAGAATA   
  
  
- GATTACATCT GTACTGCCAC AAGATTCGAA CATTCCGGAA CAGGAACCGA GGTACCTTGG AAGTTAAATA   
  
  
- GTCAGTTTAG ATGTCACACT TGCATATAAT AAGCTAAGAC ATAAATTAAA AACCATGGTG CGGAGAAATT   
  
  
- TTCTTTTATT TTCCAATGTT TTAAGTGTTT TGCAAAAAT

+     WUN-motif

| Site Name | Organism | Position | Strand | Matrix score. | sequence | function |
| --- | --- | --- | --- | --- | --- | --- |
| WUN-motif | Nicotiana glutinosa | 106 | - | 9 | TTATTACAT |  |

>Potri.005G195000.1   
+ TCAACACTTT AAAATGATAT GAAAATACTA AAAATATATT AATTTAAATA AAAAAATTAA TATTTTTAAA   
  
  
+ ACATAACAGA TCTATTTGTA TTGTTCACAG GAAAAATGTA ATAATGTTTT TGTCTTTCTG AAAAAAAAAT   
  
  
+ ATTAAATTGG TTATTGGAGG CATGGAAGTT TTTTCATAAT GGGCACTAGT TGTTAACAAA TTAAATAGGG   
  
  
+ GCAAATAAGT CATTTCAAAA ATAGAGTGAG ATGACCTAAA TATCCTTATA AGAAAAGAAA TTAAAAGTGG   
  
  
+ TGTCCAAGAG CTTTATTGTA TTTTTCACAT GATTTTTTTA TTACTATAGG TCAACTAAGG GCCAATTTAG   
  
  
+ TATTTGTATA AATATATGGA AAAAAAAAGC ACAGTGAATG ATCAAAATAT TATTGGAAGC AAAAAAAAAT   
  
  
+ TAAAGTTAGC CTCGAGGCGA TGTTTTTGTA TTTTTACAAT GATTTTTTTT ATCACCAAGT GAACTTAGAG   
  
  
+ GGAATTCGGT ATTTTTCATA AATATAGAAA AATAAAAGTG GTTTGCGGGT GCATAGCAAG AGCCAACATG   
  
  
+ TGTGAGGTGT TTGGGCCTTC ATGTAACGCT TGCGACACCT CCCAACGTTC AAAATACCCT TTCTATCGTG   
  
  
+ TAATTGGAAG CGAAGCGATG TCCTTTTCCT TGCGGGGTGT TGCATGTGGT TGTTGGTGAT ACAGTTTGTT   
  
  
+ GCCGACAAAG CTTTCCCTCT ATATTTTCTC TCTCCTCCCT ACAAAATTTA CTCTGGCCAT GCAAAATTTT   
  
  
+ AGAATTGTCC TTTGATTTAT GGGGATTTCC ACTTAAGTCC TTATTTTTTA TTTCTAATTT TTATTTTTGG   
  
  
+ TCTTTTTGTA AAACTTTAAT TTTTTTTAAT TTCATCTTTA AATCTCAATT TCTAATATTT TTTATTTCAA   
  
  
+ CCTTAATCCT TATTCTTTTA ATTGTTTTTT TAATTTTTTT TGAGCCATTT GTTAAATTGA TATTTCTTAT   
  
  
+ CAATTTCACT CTTTAATAAA AAAATTCGTT TGCATTTCTT ATTTCAACCT TGATCCTCAT TCTTTTAGTT   
  
  
+ TTTTTAGTTT TTATCCTTTT GTTAAATTGT TTTTGCTTTT CAATTTCATT CTTATAGAAT TTATTTTATA   
  
  
+ TTTCAATTTT CATTCTTTTT TTTAATTGCT ATTTTTTAAT CATTTTGTAT AATTGAATTT TTTTTTTCAA   
  
  
+ TTTCATCCTT CAATATTTAA TTGATTTGGA ATTAAGTTTC AAGGTTTTTC CAGATAGGAT GTTTCTAGTC   
  
  
+ TAATAACCTG GATCACAAGT TTAAAAAGTT AACATGGGTT GATATATTTT TTTTATAAAA AAATAGACTT   
  
  
+ TGTGATTTTC TTTGTTTTAT TTTCTATCTA ATTATCTTGA TGTTATAACA TAGGCCGCAT GTTTGGCGGG   
  
  
+ ATAACTTGAG TTAGCTTAAC CCTGATTACT AGGATTATAG GTTTGTCATA TTAACTTGAG TTGACTAAAC   
  
  
+ CTGATTTTTT TATAATTTTG TTTATCTAAT TTTATTCTTT CATATTTAAT GGACTTAGAA TTAAGTTTTG   
  
  
+ TTCTTTATCA TCTTTTAAAA AAAATGTTTT TTTACGTTAT CATGATTTTT TTTTTAATTT ATATCATTTG   
  
  
+ TTATTGTTAT CTATTTATGA TCTATTTAAA ATAAAATTAA CTTCTTTAAA TTAGTTAAAT CTATTATTTG   
  
  
+ AATCACGAAA AAAAATAGTC ACCGCATGAC GAAGGTCCAA AACTAGCCAT GAGGTATTCC TCGATTAATA   
  
  
+ TCAAAGCCCA ATTGTTTAAG CAGTAAGTCG GTTATCGGAA GTCAATTTGG ACACGCTCTG TCTCTCTTAT   
  
  
+ CTAATGTAGA CATGACGGTG TTCTAAGCTT GTAAGGCCTT GTCCTTGGCT CCATGGAACC TTCAATTTAT   
  
  
+ CAGTCAAATC TACAGTGTGA ACGTATATTA TTCGATTCTG TATTTAATTT TTGGTACCAC GCCTCTTTAA   
  
  
+ AAGAAAATAA AAGGTTACAA AATTCACAAA ACGTTTTTA  

- AGTTGTGAAA TTTTACTATA CTTTTATGAT TTTTATATAA TTAAATTTAT TTTTTTAATT ATAAAAATTT   
  
  
- TGTATTGTCT AGATAAACAT AACAAGTGTC CTTTTTACAT TATTACAAAA ACAGAAAGAC TTTTTTTTTA   
  
  
- TAATTTAACC AATAACCTCC GTACCTTCAA AAAAGTATTA CCCGTGATCA ACAATTGTTT AATTTATCCC   
  
  
- CGTTTATTCA GTAAAGTTTT TATCTCACTC TACTGGATTT ATAGGAATAT TCTTTTCTTT AATTTTCACC   
  
  
- ACAGGTTCTC GAAATAACAT AAAAAGTGTA CTAAAAAAAT AATGATATCC AGTTGATTCC CGGTTAAATC   
  
  
- ATAAACATAT TTATATACCT TTTTTTTTCG TGTCACTTAC TAGTTTTATA ATAACCTTCG TTTTTTTTTA   
  
  
- ATTTCAATCG GAGCTCCGCT ACAAAAACAT AAAAATGTTA CTAAAAAAAA TAGTGGTTCA CTTGAATCTC   
  
  
- CCTTAAGCCA TAAAAAGTAT TTATATCTTT TTATTTTCAC CAAACGCCCA CGTATCGTTC TCGGTTGTAC   
  
  
- ACACTCCACA AACCCGGAAG TACATTGCGA ACGCTGTGGA GGGTTGCAAG TTTTATGGGA AAGATAGCAC   
  
  
- ATTAACCTTC GCTTCGCTAC AGGAAAAGGA ACGCCCCACA ACGTACACCA ACAACCACTA TGTCAAACAA   
  
  
- CGGCTGTTTC GAAAGGGAGA TATAAAAGAG AGAGGAGGGA TGTTTTAAAT GAGACCGGTA CGTTTTAAAA   
  
  
- TCTTAACAGG AAACTAAATA CCCCTAAAGG TGAATTCAGG AATAAAAAAT AAAGATTAAA AATAAAAACC   
  
  
- AGAAAAACAT TTTGAAATTA AAAAAAATTA AAGTAGAAAT TTAGAGTTAA AGATTATAAA AAATAAAGTT   
  
  
- GGAATTAGGA ATAAGAAAAT TAACAAAAAA ATTAAAAAAA ACTCGGTAAA CAATTTAACT ATAAAGAATA   
  
  
- GTTAAAGTGA GAAATTATTT TTTTAAGCAA ACGTAAAGAA TAAAGTTGGA ACTAGGAGTA AGAAAATCAA   
  
  
- AAAAATCAAA AATAGGAAAA CAATTTAACA AAAACGAAAA GTTAAAGTAA GAATATCTTA AATAAAATAT   
  
  
- AAAGTTAAAA GTAAGAAAAA AAATTAACGA TAAAAAATTA GTAAAACATA TTAACTTAAA AAAAAAAGTT   
  
  
- AAAGTAGGAA GTTATAAATT AACTAAACCT TAATTCAAAG TTCCAAAAAG GTCTATCCTA CAAAGATCAG   
  
  
- ATTATTGGAC CTAGTGTTCA AATTTTTCAA TTGTACCCAA CTATATAAAA AAAATATTTT TTTATCTGAA   
  
  
- ACACTAAAAG AAACAAAATA AAAGATAGAT TAATAGAACT ACAATATTGT ATCCGGCGTA CAAACCGCCC   
  
  
- TATTGAACTC AATCGAATTG GGACTAATGA TCCTAATATC CAAACAGTAT AATTGAACTC AACTGATTTG   
  
  
- GACTAAAAAA ATATTAAAAC AAATAGATTA AAATAAGAAA GTATAAATTA CCTGAATCTT AATTCAAAAC   
  
  
- AAGAAATAGT AGAAAATTTT TTTTACAAAA AAATGCAATA GTACTAAAAA AAAAATTAAA TATAGTAAAC   
  
  
- AATAACAATA GATAAATACT AGATAAATTT TATTTTAATT GAAGAAATTT AATCAATTTA GATAATAAAC   
  
  
- TTAGTGCTTT TTTTTATCAG TGGCGTACTG CTTCCAGGTT TTGATCGGTA CTCCATAAGG AGCTAATTAT   
  
  
- AGTTTCGGGT TAACAAATTC GTCATTCAGC CAATAGCCTT CAGTTAAACC TGTGCGAGAC AGAGAGAATA   
  
  
- GATTACATCT GTACTGCCAC AAGATTCGAA CATTCCGGAA CAGGAACCGA GGTACCTTGG AAGTTAAATA   
  
  
- GTCAGTTTAG ATGTCACACT TGCATATAAT AAGCTAAGAC ATAAATTAAA AACCATGGTG CGGAGAAATT   
  
  
- TTCTTTTATT TTCCAATGTT TTAAGTGTTT TGCAAAAAT

+     as-1

| Site Name | Organism | Position | Strand | Matrix score. | sequence | function |
| --- | --- | --- | --- | --- | --- | --- |
| as-1 | Arabidopsis thaliana | 1833 | + | 5 | TGACG |  |
| as-1 | Arabidopsis thaliana | 1707 | + | 5 | TGACG |  |

>Potri.005G195000.1   
+ TCAACACTTT AAAATGATAT GAAAATACTA AAAATATATT AATTTAAATA AAAAAATTAA TATTTTTAAA   
  
  
+ ACATAACAGA TCTATTTGTA TTGTTCACAG GAAAAATGTA ATAATGTTTT TGTCTTTCTG AAAAAAAAAT   
  
  
+ ATTAAATTGG TTATTGGAGG CATGGAAGTT TTTTCATAAT GGGCACTAGT TGTTAACAAA TTAAATAGGG   
  
  
+ GCAAATAAGT CATTTCAAAA ATAGAGTGAG ATGACCTAAA TATCCTTATA AGAAAAGAAA TTAAAAGTGG   
  
  
+ TGTCCAAGAG CTTTATTGTA TTTTTCACAT GATTTTTTTA TTACTATAGG TCAACTAAGG GCCAATTTAG   
  
  
+ TATTTGTATA AATATATGGA AAAAAAAAGC ACAGTGAATG ATCAAAATAT TATTGGAAGC AAAAAAAAAT   
  
  
+ TAAAGTTAGC CTCGAGGCGA TGTTTTTGTA TTTTTACAAT GATTTTTTTT ATCACCAAGT GAACTTAGAG   
  
  
+ GGAATTCGGT ATTTTTCATA AATATAGAAA AATAAAAGTG GTTTGCGGGT GCATAGCAAG AGCCAACATG   
  
  
+ TGTGAGGTGT TTGGGCCTTC ATGTAACGCT TGCGACACCT CCCAACGTTC AAAATACCCT TTCTATCGTG   
  
  
+ TAATTGGAAG CGAAGCGATG TCCTTTTCCT TGCGGGGTGT TGCATGTGGT TGTTGGTGAT ACAGTTTGTT   
  
  
+ GCCGACAAAG CTTTCCCTCT ATATTTTCTC TCTCCTCCCT ACAAAATTTA CTCTGGCCAT GCAAAATTTT   
  
  
+ AGAATTGTCC TTTGATTTAT GGGGATTTCC ACTTAAGTCC TTATTTTTTA TTTCTAATTT TTATTTTTGG   
  
  
+ TCTTTTTGTA AAACTTTAAT TTTTTTTAAT TTCATCTTTA AATCTCAATT TCTAATATTT TTTATTTCAA   
  
  
+ CCTTAATCCT TATTCTTTTA ATTGTTTTTT TAATTTTTTT TGAGCCATTT GTTAAATTGA TATTTCTTAT   
  
  
+ CAATTTCACT CTTTAATAAA AAAATTCGTT TGCATTTCTT ATTTCAACCT TGATCCTCAT TCTTTTAGTT   
  
  
+ TTTTTAGTTT TTATCCTTTT GTTAAATTGT TTTTGCTTTT CAATTTCATT CTTATAGAAT TTATTTTATA   
  
  
+ TTTCAATTTT CATTCTTTTT TTTAATTGCT ATTTTTTAAT CATTTTGTAT AATTGAATTT TTTTTTTCAA   
  
  
+ TTTCATCCTT CAATATTTAA TTGATTTGGA ATTAAGTTTC AAGGTTTTTC CAGATAGGAT GTTTCTAGTC   
  
  
+ TAATAACCTG GATCACAAGT TTAAAAAGTT AACATGGGTT GATATATTTT TTTTATAAAA AAATAGACTT   
  
  
+ TGTGATTTTC TTTGTTTTAT TTTCTATCTA ATTATCTTGA TGTTATAACA TAGGCCGCAT GTTTGGCGGG   
  
  
+ ATAACTTGAG TTAGCTTAAC CCTGATTACT AGGATTATAG GTTTGTCATA TTAACTTGAG TTGACTAAAC   
  
  
+ CTGATTTTTT TATAATTTTG TTTATCTAAT TTTATTCTTT CATATTTAAT GGACTTAGAA TTAAGTTTTG   
  
  
+ TTCTTTATCA TCTTTTAAAA AAAATGTTTT TTTACGTTAT CATGATTTTT TTTTTAATTT ATATCATTTG   
  
  
+ TTATTGTTAT CTATTTATGA TCTATTTAAA ATAAAATTAA CTTCTTTAAA TTAGTTAAAT CTATTATTTG   
  
  
+ AATCACGAAA AAAAATAGTC ACCGCATGAC GAAGGTCCAA AACTAGCCAT GAGGTATTCC TCGATTAATA   
  
  
+ TCAAAGCCCA ATTGTTTAAG CAGTAAGTCG GTTATCGGAA GTCAATTTGG ACACGCTCTG TCTCTCTTAT   
  
  
+ CTAATGTAGA CATGACGGTG TTCTAAGCTT GTAAGGCCTT GTCCTTGGCT CCATGGAACC TTCAATTTAT   
  
  
+ CAGTCAAATC TACAGTGTGA ACGTATATTA TTCGATTCTG TATTTAATTT TTGGTACCAC GCCTCTTTAA   
  
  
+ AAGAAAATAA AAGGTTACAA AATTCACAAA ACGTTTTTA  

- AGTTGTGAAA TTTTACTATA CTTTTATGAT TTTTATATAA TTAAATTTAT TTTTTTAATT ATAAAAATTT   
  
  
- TGTATTGTCT AGATAAACAT AACAAGTGTC CTTTTTACAT TATTACAAAA ACAGAAAGAC TTTTTTTTTA   
  
  
- TAATTTAACC AATAACCTCC GTACCTTCAA AAAAGTATTA CCCGTGATCA ACAATTGTTT AATTTATCCC   
  
  
- CGTTTATTCA GTAAAGTTTT TATCTCACTC TACTGGATTT ATAGGAATAT TCTTTTCTTT AATTTTCACC   
  
  
- ACAGGTTCTC GAAATAACAT AAAAAGTGTA CTAAAAAAAT AATGATATCC AGTTGATTCC CGGTTAAATC   
  
  
- ATAAACATAT TTATATACCT TTTTTTTTCG TGTCACTTAC TAGTTTTATA ATAACCTTCG TTTTTTTTTA   
  
  
- ATTTCAATCG GAGCTCCGCT ACAAAAACAT AAAAATGTTA CTAAAAAAAA TAGTGGTTCA CTTGAATCTC   
  
  
- CCTTAAGCCA TAAAAAGTAT TTATATCTTT TTATTTTCAC CAAACGCCCA CGTATCGTTC TCGGTTGTAC   
  
  
- ACACTCCACA AACCCGGAAG TACATTGCGA ACGCTGTGGA GGGTTGCAAG TTTTATGGGA AAGATAGCAC   
  
  
- ATTAACCTTC GCTTCGCTAC AGGAAAAGGA ACGCCCCACA ACGTACACCA ACAACCACTA TGTCAAACAA   
  
  
- CGGCTGTTTC GAAAGGGAGA TATAAAAGAG AGAGGAGGGA TGTTTTAAAT GAGACCGGTA CGTTTTAAAA   
  
  
- TCTTAACAGG AAACTAAATA CCCCTAAAGG TGAATTCAGG AATAAAAAAT AAAGATTAAA AATAAAAACC   
  
  
- AGAAAAACAT TTTGAAATTA AAAAAAATTA AAGTAGAAAT TTAGAGTTAA AGATTATAAA AAATAAAGTT   
  
  
- GGAATTAGGA ATAAGAAAAT TAACAAAAAA ATTAAAAAAA ACTCGGTAAA CAATTTAACT ATAAAGAATA   
  
  
- GTTAAAGTGA GAAATTATTT TTTTAAGCAA ACGTAAAGAA TAAAGTTGGA ACTAGGAGTA AGAAAATCAA   
  
  
- AAAAATCAAA AATAGGAAAA CAATTTAACA AAAACGAAAA GTTAAAGTAA GAATATCTTA AATAAAATAT   
  
  
- AAAGTTAAAA GTAAGAAAAA AAATTAACGA TAAAAAATTA GTAAAACATA TTAACTTAAA AAAAAAAGTT   
  
  
- AAAGTAGGAA GTTATAAATT AACTAAACCT TAATTCAAAG TTCCAAAAAG GTCTATCCTA CAAAGATCAG   
  
  
- ATTATTGGAC CTAGTGTTCA AATTTTTCAA TTGTACCCAA CTATATAAAA AAAATATTTT TTTATCTGAA   
  
  
- ACACTAAAAG AAACAAAATA AAAGATAGAT TAATAGAACT ACAATATTGT ATCCGGCGTA CAAACCGCCC   
  
  
- TATTGAACTC AATCGAATTG GGACTAATGA TCCTAATATC CAAACAGTAT AATTGAACTC AACTGATTTG   
  
  
- GACTAAAAAA ATATTAAAAC AAATAGATTA AAATAAGAAA GTATAAATTA CCTGAATCTT AATTCAAAAC   
  
  
- AAGAAATAGT AGAAAATTTT TTTTACAAAA AAATGCAATA GTACTAAAAA AAAAATTAAA TATAGTAAAC   
  
  
- AATAACAATA GATAAATACT AGATAAATTT TATTTTAATT GAAGAAATTT AATCAATTTA GATAATAAAC   
  
  
- TTAGTGCTTT TTTTTATCAG TGGCGTACTG CTTCCAGGTT TTGATCGGTA CTCCATAAGG AGCTAATTAT   
  
  
- AGTTTCGGGT TAACAAATTC GTCATTCAGC CAATAGCCTT CAGTTAAACC TGTGCGAGAC AGAGAGAATA   
  
  
- GATTACATCT GTACTGCCAC AAGATTCGAA CATTCCGGAA CAGGAACCGA GGTACCTTGG AAGTTAAATA   
  
  
- GTCAGTTTAG ATGTCACACT TGCATATAAT AAGCTAAGAC ATAAATTAAA AACCATGGTG CGGAGAAATT   
  
  
- TTCTTTTATT TTCCAATGTT TTAAGTGTTT TGCAAAAAT
