# Supplementary material for: Structure and expression analysis of seven salt-related ERF genes of Populus
Source: PeerJ. 2020 Oct 20;8:e10206. doi: 10.7717/peerj.10206 (PMC7583627; doi:10.7717/peerj.10206)
Supplement: Supplemental Information 17 [file peerj-08-10206-s017.gz › Potri.006G138900.1_plantcare.html]

Content-Type: text/html; charset=ISO-8859-1


PlantCARE


Webmaster Firefox specific output  
To save the result:
click on the frame with the right mouse button and save the source code as a text file with extension .html  
REFERENCE:PlantCARE: a database of plant cis-acting regulatory elements and a portal to tools for in silico analysis of promoter sequences.  
Lescot, M., Déhais, P., Moreau, Y., De Moor, B., Rouzé ,P.,and Rombauts, S.  
Nucleic Acids Res., Database issue(2002), 30(1):325-327.   


---

>Potri.006G138900.1   
+ TGATACCAGA TGATGTGTCG TGAATGATTT GAAAATCGAA AGCAATGAAT CTGACGAAAA TGGATGAAGG   
  
  
+ ATGGGTCTGG TTAGTTGTCT TTCTTTTGGT GTTTAGGCTG GATTATAGTG ATTTTAAAGT AAATAAGATG   
  
  
+ GAGGATAGAC TAGAATGATA CTTTGATAAG TCGATCTGGA CGCCACAAAG ATCAATCTAG GATTTCGACA   
  
  
+ CCTATAAAAT ATTTGGATTA GTGTTTTGTG TTTTTTAAAA AACTTTTTTT TGTTTAGTTT TTTTTTCATT   
  
  
+ TCACCGTTCA ACAACTCAAT CTTTTTCTTC TTTTTATAAT TTTTTTTCTA TTTCATCTTT AAAAATTAGG   
  
  
+ TTGTTTTGAA AATTTTGCTT CATATTTTTT TTTTCTGTTG GGTTATATAT TAGTCTCATG GATTTAATTT   
  
  
+ TATTTTCTCG ATTTCAACCT TCAACACTAG ATCTATTGGA AGTGGGGTTT CATAATATTT TTTTTATTTA   
  
  
+ TTTTCTATGA ATTTTTCTTG ATCTCATGAC TAGGTCACAA GTTTAACAAG TTAACTCAAG TTGACTGAGA   
  
  
+ TCATTTTCTT ATTGCTTTTT TTAATTGATT TTTTTATTTC ATCCTTCAAT ATTGGGTTGG ATGGAAATTG   
  
  
+ AGATTCATAT ATTTTTTCTA TTTACTTTCT ATGGGGTTAT CCTGATCTCA TGTGAGTTTA GCAGGTTAAG   
  
  
+ TCGGGTTGAC TTTATTTATT TATTTTTCTT CTTTTTTAAT TGGACTTTGG AATTTTTTTT ATTTTATTTT   
  
  
+ TATGGGATTA TCCAGATCTT ATGACTCAAG TCACGAGTTT AGTTGGTTGA CCTGAGTTGA CTTGATTTAT   
  
  
+ TATTTGGGTT CCTTTTTTAA TTGAATGTTT TTTTTTCTAT TTCACCTTCC AACAACTCAA TTGTTTTATT   
  
  
+ TCACGTTTTT TTTCTTCAGT TTCATCCTTC AATATTAAGT TGTTTGGGAA TTAGATTTTG AAATTTCTTT   
  
  
+ TTCAATTTGG TTTTTGTGGA GTTAACCCGA TCTAATATAT TTAATTTTTT TTCTTGATTT TAATCTTTTA   
  
  
+ CATTGGATCT GTTAGAAAAT GGATCTTTGT AATTTTTTAT TTGATTTTTT TGAAGTTATC TCAGTCTTAT   
  
  
+ GATCTAGGTC ACGAGCTTAA CATATTAGCC TAAGTTAACT TGAGTTGTTT TTTTTAGTTG ATTTTTTTTA   
  
  
+ TTTCATCATT CAACATTAAG TTAGTTGTAA ATTAGGTTTC ATAATTTTTT TATATATGCT TTCTATTGAG   
  
  
+ TTATAAAATA TCCCGATCTC ATGATCCGGG TTGCGAGTTT AACATCTTAA CTTACATTAA CTTGGGTAGT   
  
  
+ TTTTTTTATC CTTTTTTTAA TTTAATATTT TTTTTTCAAT TCCACCCTTC AATATCAAAT TGATTAGAGA   
  
  
+ TTGATCTTCA TAATTTATTT TGATTTACTT TCTATATATA AGGTTATTAT GGTTTCTTGA TTTAAGTTTC   
  
  
+ AAATTTGACA GATTAACTTG TATCAATCTT AGATGTTTTT ATTTTAATAT TTAAAAAATA CATCCAATAT   
  
  
+ ACTTTATGTT TTGAATTTAT AATTAATTTT TTTATTTGAA AATATGTTAA CATCGTAGGT ATATTTTTTT   
  
  
+ ATATTAAAAA ATTAATTGGA TTCAGAGTCA TAAAAATAAT CAGGTTTTCT CTAAAAATCG GTATGCATCA   
  
  
+ CTTTCAAAAC ATGAACGCTC ACATTCTCCC AACGGAGGAA AAATGCCTCT AGATGTTGTC AAAAGAAGAC   
  
  
+ GCCTTTATAA TTACAATTTT TTATACCCAT TTTCCATATC CCAAAAGGCT GATGATTATG GCATTTTCGG   
  
  
+ ATTTTTAAAA AAAAGAAAAA AAATGATAAA TACAGAGGTG TTTAGCATTG AAAAGAAAAA TAAATTAAAG   
  
  
+ GACCTGCCGT CAATAAAATT TGGCAGCAAA GAAGAATATA ATTGACCAGT CAATGAGGAC CTGATAACTA   
  
  
+ TAAAAAAAGA GGCGCATGGA CGGGCGCACC CACATGTTA  

- ACTATGGTCT ACTACACAGC ACTTACTAAA CTTTTAGCTT TCGTTACTTA GACTGCTTTT ACCTACTTCC   
  
  
- TACCCAGACC AATCAACAGA AAGAAAACCA CAAATCCGAC CTAATATCAC TAAAATTTCA TTTATTCTAC   
  
  
- CTCCTATCTG ATCTTACTAT GAAACTATTC AGCTAGACCT GCGGTGTTTC TAGTTAGATC CTAAAGCTGT   
  
  
- GGATATTTTA TAAACCTAAT CACAAAACAC AAAAAATTTT TTGAAAAAAA ACAAATCAAA AAAAAAGTAA   
  
  
- AGTGGCAAGT TGTTGAGTTA GAAAAAGAAG AAAAATATTA AAAAAAAGAT AAAGTAGAAA TTTTTAATCC   
  
  
- AACAAAACTT TTAAAACGAA GTATAAAAAA AAAAGACAAC CCAATATATA ATCAGAGTAC CTAAATTAAA   
  
  
- ATAAAAGAGC TAAAGTTGGA AGTTGTGATC TAGATAACCT TCACCCCAAA GTATTATAAA AAAAATAAAT   
  
  
- AAAAGATACT TAAAAAGAAC TAGAGTACTG ATCCAGTGTT CAAATTGTTC AATTGAGTTC AACTGACTCT   
  
  
- AGTAAAAGAA TAACGAAAAA AATTAACTAA AAAAATAAAG TAGGAAGTTA TAACCCAACC TACCTTTAAC   
  
  
- TCTAAGTATA TAAAAAAGAT AAATGAAAGA TACCCCAATA GGACTAGAGT ACACTCAAAT CGTCCAATTC   
  
  
- AGCCCAACTG AAATAAATAA ATAAAAAGAA GAAAAAATTA ACCTGAAACC TTAAAAAAAA TAAAATAAAA   
  
  
- ATACCCTAAT AGGTCTAGAA TACTGAGTTC AGTGCTCAAA TCAACCAACT GGACTCAACT GAACTAAATA   
  
  
- ATAAACCCAA GGAAAAAATT AACTTACAAA AAAAAAGATA AAGTGGAAGG TTGTTGAGTT AACAAAATAA   
  
  
- AGTGCAAAAA AAAGAAGTCA AAGTAGGAAG TTATAATTCA ACAAACCCTT AATCTAAAAC TTTAAAGAAA   
  
  
- AAGTTAAACC AAAAACACCT CAATTGGGCT AGATTATATA AATTAAAAAA AAGAACTAAA ATTAGAAAAT   
  
  
- GTAACCTAGA CAATCTTTTA CCTAGAAACA TTAAAAAATA AACTAAAAAA ACTTCAATAG AGTCAGAATA   
  
  
- CTAGATCCAG TGCTCGAATT GTATAATCGG ATTCAATTGA ACTCAACAAA AAAAATCAAC TAAAAAAAAT   
  
  
- AAAGTAGTAA GTTGTAATTC AATCAACATT TAATCCAAAG TATTAAAAAA ATATATACGA AAGATAACTC   
  
  
- AATATTTTAT AGGGCTAGAG TACTAGGCCC AACGCTCAAA TTGTAGAATT GAATGTAATT GAACCCATCA   
  
  
- AAAAAAATAG GAAAAAAATT AAATTATAAA AAAAAAGTTA AGGTGGGAAG TTATAGTTTA ACTAATCTCT   
  
  
- AACTAGAAGT ATTAAATAAA ACTAAATGAA AGATATATAT TCCAATAATA CCAAAGAACT AAATTCAAAG   
  
  
- TTTAAACTGT CTAATTGAAC ATAGTTAGAA TCTACAAAAA TAAAATTATA AATTTTTTAT GTAGGTTATA   
  
  
- TGAAATACAA AACTTAAATA TTAATTAAAA AAATAAACTT TTATACAATT GTAGCATCCA TATAAAAAAA   
  
  
- TATAATTTTT TAATTAACCT AAGTCTCAGT ATTTTTATTA GTCCAAAAGA GATTTTTAGC CATACGTAGT   
  
  
- GAAAGTTTTG TACTTGCGAG TGTAAGAGGG TTGCCTCCTT TTTACGGAGA TCTACAACAG TTTTCTTCTG   
  
  
- CGGAAATATT AATGTTAAAA AATATGGGTA AAAGGTATAG GGTTTTCCGA CTACTAATAC CGTAAAAGCC   
  
  
- TAAAAATTTT TTTTCTTTTT TTTACTATTT ATGTCTCCAC AAATCGTAAC TTTTCTTTTT ATTTAATTTC   
  
  
- CTGGACGGCA GTTATTTTAA ACCGTCGTTT CTTCTTATAT TAACTGGTCA GTTACTCCTG GACTATTGAT   
  
  
- ATTTTTTTCT CCGCGTACCT GCCCGCGTGG GTGTACAAT

  
  
Motifs Found  

+   

| Site Name | Organism | Position | Strand | Matrix score. | sequence | function |
| --- | --- | --- | --- | --- | --- | --- |
|  | organism | 1404 | + | 4 | motif\_sequence | short\_function |

>Potri.006G138900.1   
+ TGATACCAGA TGATGTGTCG TGAATGATTT GAAAATCGAA AGCAATGAAT CTGACGAAAA TGGATGAAGG   
  
  
+ ATGGGTCTGG TTAGTTGTCT TTCTTTTGGT GTTTAGGCTG GATTATAGTG ATTTTAAAGT AAATAAGATG   
  
  
+ GAGGATAGAC TAGAATGATA CTTTGATAAG TCGATCTGGA CGCCACAAAG ATCAATCTAG GATTTCGACA   
  
  
+ CCTATAAAAT ATTTGGATTA GTGTTTTGTG TTTTTTAAAA AACTTTTTTT TGTTTAGTTT TTTTTTCATT   
  
  
+ TCACCGTTCA ACAACTCAAT CTTTTTCTTC TTTTTATAAT TTTTTTTCTA TTTCATCTTT AAAAATTAGG   
  
  
+ TTGTTTTGAA AATTTTGCTT CATATTTTTT TTTTCTGTTG GGTTATATAT TAGTCTCATG GATTTAATTT   
  
  
+ TATTTTCTCG ATTTCAACCT TCAACACTAG ATCTATTGGA AGTGGGGTTT CATAATATTT TTTTTATTTA   
  
  
+ TTTTCTATGA ATTTTTCTTG ATCTCATGAC TAGGTCACAA GTTTAACAAG TTAACTCAAG TTGACTGAGA   
  
  
+ TCATTTTCTT ATTGCTTTTT TTAATTGATT TTTTTATTTC ATCCTTCAAT ATTGGGTTGG ATGGAAATTG   
  
  
+ AGATTCATAT ATTTTTTCTA TTTACTTTCT ATGGGGTTAT CCTGATCTCA TGTGAGTTTA GCAGGTTAAG   
  
  
+ TCGGGTTGAC TTTATTTATT TATTTTTCTT CTTTTTTAAT TGGACTTTGG AATTTTTTTT ATTTTATTTT   
  
  
+ TATGGGATTA TCCAGATCTT ATGACTCAAG TCACGAGTTT AGTTGGTTGA CCTGAGTTGA CTTGATTTAT   
  
  
+ TATTTGGGTT CCTTTTTTAA TTGAATGTTT TTTTTTCTAT TTCACCTTCC AACAACTCAA TTGTTTTATT   
  
  
+ TCACGTTTTT TTTCTTCAGT TTCATCCTTC AATATTAAGT TGTTTGGGAA TTAGATTTTG AAATTTCTTT   
  
  
+ TTCAATTTGG TTTTTGTGGA GTTAACCCGA TCTAATATAT TTAATTTTTT TTCTTGATTT TAATCTTTTA   
  
  
+ CATTGGATCT GTTAGAAAAT GGATCTTTGT AATTTTTTAT TTGATTTTTT TGAAGTTATC TCAGTCTTAT   
  
  
+ GATCTAGGTC ACGAGCTTAA CATATTAGCC TAAGTTAACT TGAGTTGTTT TTTTTAGTTG ATTTTTTTTA   
  
  
+ TTTCATCATT CAACATTAAG TTAGTTGTAA ATTAGGTTTC ATAATTTTTT TATATATGCT TTCTATTGAG   
  
  
+ TTATAAAATA TCCCGATCTC ATGATCCGGG TTGCGAGTTT AACATCTTAA CTTACATTAA CTTGGGTAGT   
  
  
+ TTTTTTTATC CTTTTTTTAA TTTAATATTT TTTTTTCAAT TCCACCCTTC AATATCAAAT TGATTAGAGA   
  
  
+ TTGATCTTCA TAATTTATTT TGATTTACTT TCTATATATA AGGTTATTAT GGTTTCTTGA TTTAAGTTTC   
  
  
+ AAATTTGACA GATTAACTTG TATCAATCTT AGATGTTTTT ATTTTAATAT TTAAAAAATA CATCCAATAT   
  
  
+ ACTTTATGTT TTGAATTTAT AATTAATTTT TTTATTTGAA AATATGTTAA CATCGTAGGT ATATTTTTTT   
  
  
+ ATATTAAAAA ATTAATTGGA TTCAGAGTCA TAAAAATAAT CAGGTTTTCT CTAAAAATCG GTATGCATCA   
  
  
+ CTTTCAAAAC ATGAACGCTC ACATTCTCCC AACGGAGGAA AAATGCCTCT AGATGTTGTC AAAAGAAGAC   
  
  
+ GCCTTTATAA TTACAATTTT TTATACCCAT TTTCCATATC CCAAAAGGCT GATGATTATG GCATTTTCGG   
  
  
+ ATTTTTAAAA AAAAGAAAAA AAATGATAAA TACAGAGGTG TTTAGCATTG AAAAGAAAAA TAAATTAAAG   
  
  
+ GACCTGCCGT CAATAAAATT TGGCAGCAAA GAAGAATATA ATTGACCAGT CAATGAGGAC CTGATAACTA   
  
  
+ TAAAAAAAGA GGCGCATGGA CGGGCGCACC CACATGTTA  

- ACTATGGTCT ACTACACAGC ACTTACTAAA CTTTTAGCTT TCGTTACTTA GACTGCTTTT ACCTACTTCC   
  
  
- TACCCAGACC AATCAACAGA AAGAAAACCA CAAATCCGAC CTAATATCAC TAAAATTTCA TTTATTCTAC   
  
  
- CTCCTATCTG ATCTTACTAT GAAACTATTC AGCTAGACCT GCGGTGTTTC TAGTTAGATC CTAAAGCTGT   
  
  
- GGATATTTTA TAAACCTAAT CACAAAACAC AAAAAATTTT TTGAAAAAAA ACAAATCAAA AAAAAAGTAA   
  
  
- AGTGGCAAGT TGTTGAGTTA GAAAAAGAAG AAAAATATTA AAAAAAAGAT AAAGTAGAAA TTTTTAATCC   
  
  
- AACAAAACTT TTAAAACGAA GTATAAAAAA AAAAGACAAC CCAATATATA ATCAGAGTAC CTAAATTAAA   
  
  
- ATAAAAGAGC TAAAGTTGGA AGTTGTGATC TAGATAACCT TCACCCCAAA GTATTATAAA AAAAATAAAT   
  
  
- AAAAGATACT TAAAAAGAAC TAGAGTACTG ATCCAGTGTT CAAATTGTTC AATTGAGTTC AACTGACTCT   
  
  
- AGTAAAAGAA TAACGAAAAA AATTAACTAA AAAAATAAAG TAGGAAGTTA TAACCCAACC TACCTTTAAC   
  
  
- TCTAAGTATA TAAAAAAGAT AAATGAAAGA TACCCCAATA GGACTAGAGT ACACTCAAAT CGTCCAATTC   
  
  
- AGCCCAACTG AAATAAATAA ATAAAAAGAA GAAAAAATTA ACCTGAAACC TTAAAAAAAA TAAAATAAAA   
  
  
- ATACCCTAAT AGGTCTAGAA TACTGAGTTC AGTGCTCAAA TCAACCAACT GGACTCAACT GAACTAAATA   
  
  
- ATAAACCCAA GGAAAAAATT AACTTACAAA AAAAAAGATA AAGTGGAAGG TTGTTGAGTT AACAAAATAA   
  
  
- AGTGCAAAAA AAAGAAGTCA AAGTAGGAAG TTATAATTCA ACAAACCCTT AATCTAAAAC TTTAAAGAAA   
  
  
- AAGTTAAACC AAAAACACCT CAATTGGGCT AGATTATATA AATTAAAAAA AAGAACTAAA ATTAGAAAAT   
  
  
- GTAACCTAGA CAATCTTTTA CCTAGAAACA TTAAAAAATA AACTAAAAAA ACTTCAATAG AGTCAGAATA   
  
  
- CTAGATCCAG TGCTCGAATT GTATAATCGG ATTCAATTGA ACTCAACAAA AAAAATCAAC TAAAAAAAAT   
  
  
- AAAGTAGTAA GTTGTAATTC AATCAACATT TAATCCAAAG TATTAAAAAA ATATATACGA AAGATAACTC   
  
  
- AATATTTTAT AGGGCTAGAG TACTAGGCCC AACGCTCAAA TTGTAGAATT GAATGTAATT GAACCCATCA   
  
  
- AAAAAAATAG GAAAAAAATT AAATTATAAA AAAAAAGTTA AGGTGGGAAG TTATAGTTTA ACTAATCTCT   
  
  
- AACTAGAAGT ATTAAATAAA ACTAAATGAA AGATATATAT TCCAATAATA CCAAAGAACT AAATTCAAAG   
  
  
- TTTAAACTGT CTAATTGAAC ATAGTTAGAA TCTACAAAAA TAAAATTATA AATTTTTTAT GTAGGTTATA   
  
  
- TGAAATACAA AACTTAAATA TTAATTAAAA AAATAAACTT TTATACAATT GTAGCATCCA TATAAAAAAA   
  
  
- TATAATTTTT TAATTAACCT AAGTCTCAGT ATTTTTATTA GTCCAAAAGA GATTTTTAGC CATACGTAGT   
  
  
- GAAAGTTTTG TACTTGCGAG TGTAAGAGGG TTGCCTCCTT TTTACGGAGA TCTACAACAG TTTTCTTCTG   
  
  
- CGGAAATATT AATGTTAAAA AATATGGGTA AAAGGTATAG GGTTTTCCGA CTACTAATAC CGTAAAAGCC   
  
  
- TAAAAATTTT TTTTCTTTTT TTTACTATTT ATGTCTCCAC AAATCGTAAC TTTTCTTTTT ATTTAATTTC   
  
  
- CTGGACGGCA GTTATTTTAA ACCGTCGTTT CTTCTTATAT TAACTGGTCA GTTACTCCTG GACTATTGAT   
  
  
- ATTTTTTTCT CCGCGTACCT GCCCGCGTGG GTGTACAAT

+     A-box

| Site Name | Organism | Position | Strand | Matrix score. | sequence | function |
| --- | --- | --- | --- | --- | --- | --- |
| A-box | Petroselinum crispum | 1978 | - | 6 | CCGTCC | cis-acting regulatory element |

>Potri.006G138900.1   
+ TGATACCAGA TGATGTGTCG TGAATGATTT GAAAATCGAA AGCAATGAAT CTGACGAAAA TGGATGAAGG   
  
  
+ ATGGGTCTGG TTAGTTGTCT TTCTTTTGGT GTTTAGGCTG GATTATAGTG ATTTTAAAGT AAATAAGATG   
  
  
+ GAGGATAGAC TAGAATGATA CTTTGATAAG TCGATCTGGA CGCCACAAAG ATCAATCTAG GATTTCGACA   
  
  
+ CCTATAAAAT ATTTGGATTA GTGTTTTGTG TTTTTTAAAA AACTTTTTTT TGTTTAGTTT TTTTTTCATT   
  
  
+ TCACCGTTCA ACAACTCAAT CTTTTTCTTC TTTTTATAAT TTTTTTTCTA TTTCATCTTT AAAAATTAGG   
  
  
+ TTGTTTTGAA AATTTTGCTT CATATTTTTT TTTTCTGTTG GGTTATATAT TAGTCTCATG GATTTAATTT   
  
  
+ TATTTTCTCG ATTTCAACCT TCAACACTAG ATCTATTGGA AGTGGGGTTT CATAATATTT TTTTTATTTA   
  
  
+ TTTTCTATGA ATTTTTCTTG ATCTCATGAC TAGGTCACAA GTTTAACAAG TTAACTCAAG TTGACTGAGA   
  
  
+ TCATTTTCTT ATTGCTTTTT TTAATTGATT TTTTTATTTC ATCCTTCAAT ATTGGGTTGG ATGGAAATTG   
  
  
+ AGATTCATAT ATTTTTTCTA TTTACTTTCT ATGGGGTTAT CCTGATCTCA TGTGAGTTTA GCAGGTTAAG   
  
  
+ TCGGGTTGAC TTTATTTATT TATTTTTCTT CTTTTTTAAT TGGACTTTGG AATTTTTTTT ATTTTATTTT   
  
  
+ TATGGGATTA TCCAGATCTT ATGACTCAAG TCACGAGTTT AGTTGGTTGA CCTGAGTTGA CTTGATTTAT   
  
  
+ TATTTGGGTT CCTTTTTTAA TTGAATGTTT TTTTTTCTAT TTCACCTTCC AACAACTCAA TTGTTTTATT   
  
  
+ TCACGTTTTT TTTCTTCAGT TTCATCCTTC AATATTAAGT TGTTTGGGAA TTAGATTTTG AAATTTCTTT   
  
  
+ TTCAATTTGG TTTTTGTGGA GTTAACCCGA TCTAATATAT TTAATTTTTT TTCTTGATTT TAATCTTTTA   
  
  
+ CATTGGATCT GTTAGAAAAT GGATCTTTGT AATTTTTTAT TTGATTTTTT TGAAGTTATC TCAGTCTTAT   
  
  
+ GATCTAGGTC ACGAGCTTAA CATATTAGCC TAAGTTAACT TGAGTTGTTT TTTTTAGTTG ATTTTTTTTA   
  
  
+ TTTCATCATT CAACATTAAG TTAGTTGTAA ATTAGGTTTC ATAATTTTTT TATATATGCT TTCTATTGAG   
  
  
+ TTATAAAATA TCCCGATCTC ATGATCCGGG TTGCGAGTTT AACATCTTAA CTTACATTAA CTTGGGTAGT   
  
  
+ TTTTTTTATC CTTTTTTTAA TTTAATATTT TTTTTTCAAT TCCACCCTTC AATATCAAAT TGATTAGAGA   
  
  
+ TTGATCTTCA TAATTTATTT TGATTTACTT TCTATATATA AGGTTATTAT GGTTTCTTGA TTTAAGTTTC   
  
  
+ AAATTTGACA GATTAACTTG TATCAATCTT AGATGTTTTT ATTTTAATAT TTAAAAAATA CATCCAATAT   
  
  
+ ACTTTATGTT TTGAATTTAT AATTAATTTT TTTATTTGAA AATATGTTAA CATCGTAGGT ATATTTTTTT   
  
  
+ ATATTAAAAA ATTAATTGGA TTCAGAGTCA TAAAAATAAT CAGGTTTTCT CTAAAAATCG GTATGCATCA   
  
  
+ CTTTCAAAAC ATGAACGCTC ACATTCTCCC AACGGAGGAA AAATGCCTCT AGATGTTGTC AAAAGAAGAC   
  
  
+ GCCTTTATAA TTACAATTTT TTATACCCAT TTTCCATATC CCAAAAGGCT GATGATTATG GCATTTTCGG   
  
  
+ ATTTTTAAAA AAAAGAAAAA AAATGATAAA TACAGAGGTG TTTAGCATTG AAAAGAAAAA TAAATTAAAG   
  
  
+ GACCTGCCGT CAATAAAATT TGGCAGCAAA GAAGAATATA ATTGACCAGT CAATGAGGAC CTGATAACTA   
  
  
+ TAAAAAAAGA GGCGCATGGA CGGGCGCACC CACATGTTA  

- ACTATGGTCT ACTACACAGC ACTTACTAAA CTTTTAGCTT TCGTTACTTA GACTGCTTTT ACCTACTTCC   
  
  
- TACCCAGACC AATCAACAGA AAGAAAACCA CAAATCCGAC CTAATATCAC TAAAATTTCA TTTATTCTAC   
  
  
- CTCCTATCTG ATCTTACTAT GAAACTATTC AGCTAGACCT GCGGTGTTTC TAGTTAGATC CTAAAGCTGT   
  
  
- GGATATTTTA TAAACCTAAT CACAAAACAC AAAAAATTTT TTGAAAAAAA ACAAATCAAA AAAAAAGTAA   
  
  
- AGTGGCAAGT TGTTGAGTTA GAAAAAGAAG AAAAATATTA AAAAAAAGAT AAAGTAGAAA TTTTTAATCC   
  
  
- AACAAAACTT TTAAAACGAA GTATAAAAAA AAAAGACAAC CCAATATATA ATCAGAGTAC CTAAATTAAA   
  
  
- ATAAAAGAGC TAAAGTTGGA AGTTGTGATC TAGATAACCT TCACCCCAAA GTATTATAAA AAAAATAAAT   
  
  
- AAAAGATACT TAAAAAGAAC TAGAGTACTG ATCCAGTGTT CAAATTGTTC AATTGAGTTC AACTGACTCT   
  
  
- AGTAAAAGAA TAACGAAAAA AATTAACTAA AAAAATAAAG TAGGAAGTTA TAACCCAACC TACCTTTAAC   
  
  
- TCTAAGTATA TAAAAAAGAT AAATGAAAGA TACCCCAATA GGACTAGAGT ACACTCAAAT CGTCCAATTC   
  
  
- AGCCCAACTG AAATAAATAA ATAAAAAGAA GAAAAAATTA ACCTGAAACC TTAAAAAAAA TAAAATAAAA   
  
  
- ATACCCTAAT AGGTCTAGAA TACTGAGTTC AGTGCTCAAA TCAACCAACT GGACTCAACT GAACTAAATA   
  
  
- ATAAACCCAA GGAAAAAATT AACTTACAAA AAAAAAGATA AAGTGGAAGG TTGTTGAGTT AACAAAATAA   
  
  
- AGTGCAAAAA AAAGAAGTCA AAGTAGGAAG TTATAATTCA ACAAACCCTT AATCTAAAAC TTTAAAGAAA   
  
  
- AAGTTAAACC AAAAACACCT CAATTGGGCT AGATTATATA AATTAAAAAA AAGAACTAAA ATTAGAAAAT   
  
  
- GTAACCTAGA CAATCTTTTA CCTAGAAACA TTAAAAAATA AACTAAAAAA ACTTCAATAG AGTCAGAATA   
  
  
- CTAGATCCAG TGCTCGAATT GTATAATCGG ATTCAATTGA ACTCAACAAA AAAAATCAAC TAAAAAAAAT   
  
  
- AAAGTAGTAA GTTGTAATTC AATCAACATT TAATCCAAAG TATTAAAAAA ATATATACGA AAGATAACTC   
  
  
- AATATTTTAT AGGGCTAGAG TACTAGGCCC AACGCTCAAA TTGTAGAATT GAATGTAATT GAACCCATCA   
  
  
- AAAAAAATAG GAAAAAAATT AAATTATAAA AAAAAAGTTA AGGTGGGAAG TTATAGTTTA ACTAATCTCT   
  
  
- AACTAGAAGT ATTAAATAAA ACTAAATGAA AGATATATAT TCCAATAATA CCAAAGAACT AAATTCAAAG   
  
  
- TTTAAACTGT CTAATTGAAC ATAGTTAGAA TCTACAAAAA TAAAATTATA AATTTTTTAT GTAGGTTATA   
  
  
- TGAAATACAA AACTTAAATA TTAATTAAAA AAATAAACTT TTATACAATT GTAGCATCCA TATAAAAAAA   
  
  
- TATAATTTTT TAATTAACCT AAGTCTCAGT ATTTTTATTA GTCCAAAAGA GATTTTTAGC CATACGTAGT   
  
  
- GAAAGTTTTG TACTTGCGAG TGTAAGAGGG TTGCCTCCTT TTTACGGAGA TCTACAACAG TTTTCTTCTG   
  
  
- CGGAAATATT AATGTTAAAA AATATGGGTA AAAGGTATAG GGTTTTCCGA CTACTAATAC CGTAAAAGCC   
  
  
- TAAAAATTTT TTTTCTTTTT TTTACTATTT ATGTCTCCAC AAATCGTAAC TTTTCTTTTT ATTTAATTTC   
  
  
- CTGGACGGCA GTTATTTTAA ACCGTCGTTT CTTCTTATAT TAACTGGTCA GTTACTCCTG GACTATTGAT   
  
  
- ATTTTTTTCT CCGCGTACCT GCCCGCGTGG GTGTACAAT

+     ABRE

| Site Name | Organism | Position | Strand | Matrix score. | sequence | function |
| --- | --- | --- | --- | --- | --- | --- |
| ABRE | Arabidopsis thaliana | 1286 | - | 7 | AACCCGG | cis-acting element involved in the abscisic acid responsiveness |
| ABRE | Arabidopsis thaliana | 912 | - | 5 | ACGTG | cis-acting element involved in the abscisic acid responsiveness |

>Potri.006G138900.1   
+ TGATACCAGA TGATGTGTCG TGAATGATTT GAAAATCGAA AGCAATGAAT CTGACGAAAA TGGATGAAGG   
  
  
+ ATGGGTCTGG TTAGTTGTCT TTCTTTTGGT GTTTAGGCTG GATTATAGTG ATTTTAAAGT AAATAAGATG   
  
  
+ GAGGATAGAC TAGAATGATA CTTTGATAAG TCGATCTGGA CGCCACAAAG ATCAATCTAG GATTTCGACA   
  
  
+ CCTATAAAAT ATTTGGATTA GTGTTTTGTG TTTTTTAAAA AACTTTTTTT TGTTTAGTTT TTTTTTCATT   
  
  
+ TCACCGTTCA ACAACTCAAT CTTTTTCTTC TTTTTATAAT TTTTTTTCTA TTTCATCTTT AAAAATTAGG   
  
  
+ TTGTTTTGAA AATTTTGCTT CATATTTTTT TTTTCTGTTG GGTTATATAT TAGTCTCATG GATTTAATTT   
  
  
+ TATTTTCTCG ATTTCAACCT TCAACACTAG ATCTATTGGA AGTGGGGTTT CATAATATTT TTTTTATTTA   
  
  
+ TTTTCTATGA ATTTTTCTTG ATCTCATGAC TAGGTCACAA GTTTAACAAG TTAACTCAAG TTGACTGAGA   
  
  
+ TCATTTTCTT ATTGCTTTTT TTAATTGATT TTTTTATTTC ATCCTTCAAT ATTGGGTTGG ATGGAAATTG   
  
  
+ AGATTCATAT ATTTTTTCTA TTTACTTTCT ATGGGGTTAT CCTGATCTCA TGTGAGTTTA GCAGGTTAAG   
  
  
+ TCGGGTTGAC TTTATTTATT TATTTTTCTT CTTTTTTAAT TGGACTTTGG AATTTTTTTT ATTTTATTTT   
  
  
+ TATGGGATTA TCCAGATCTT ATGACTCAAG TCACGAGTTT AGTTGGTTGA CCTGAGTTGA CTTGATTTAT   
  
  
+ TATTTGGGTT CCTTTTTTAA TTGAATGTTT TTTTTTCTAT TTCACCTTCC AACAACTCAA TTGTTTTATT   
  
  
+ TCACGTTTTT TTTCTTCAGT TTCATCCTTC AATATTAAGT TGTTTGGGAA TTAGATTTTG AAATTTCTTT   
  
  
+ TTCAATTTGG TTTTTGTGGA GTTAACCCGA TCTAATATAT TTAATTTTTT TTCTTGATTT TAATCTTTTA   
  
  
+ CATTGGATCT GTTAGAAAAT GGATCTTTGT AATTTTTTAT TTGATTTTTT TGAAGTTATC TCAGTCTTAT   
  
  
+ GATCTAGGTC ACGAGCTTAA CATATTAGCC TAAGTTAACT TGAGTTGTTT TTTTTAGTTG ATTTTTTTTA   
  
  
+ TTTCATCATT CAACATTAAG TTAGTTGTAA ATTAGGTTTC ATAATTTTTT TATATATGCT TTCTATTGAG   
  
  
+ TTATAAAATA TCCCGATCTC ATGATCCGGG TTGCGAGTTT AACATCTTAA CTTACATTAA CTTGGGTAGT   
  
  
+ TTTTTTTATC CTTTTTTTAA TTTAATATTT TTTTTTCAAT TCCACCCTTC AATATCAAAT TGATTAGAGA   
  
  
+ TTGATCTTCA TAATTTATTT TGATTTACTT TCTATATATA AGGTTATTAT GGTTTCTTGA TTTAAGTTTC   
  
  
+ AAATTTGACA GATTAACTTG TATCAATCTT AGATGTTTTT ATTTTAATAT TTAAAAAATA CATCCAATAT   
  
  
+ ACTTTATGTT TTGAATTTAT AATTAATTTT TTTATTTGAA AATATGTTAA CATCGTAGGT ATATTTTTTT   
  
  
+ ATATTAAAAA ATTAATTGGA TTCAGAGTCA TAAAAATAAT CAGGTTTTCT CTAAAAATCG GTATGCATCA   
  
  
+ CTTTCAAAAC ATGAACGCTC ACATTCTCCC AACGGAGGAA AAATGCCTCT AGATGTTGTC AAAAGAAGAC   
  
  
+ GCCTTTATAA TTACAATTTT TTATACCCAT TTTCCATATC CCAAAAGGCT GATGATTATG GCATTTTCGG   
  
  
+ ATTTTTAAAA AAAAGAAAAA AAATGATAAA TACAGAGGTG TTTAGCATTG AAAAGAAAAA TAAATTAAAG   
  
  
+ GACCTGCCGT CAATAAAATT TGGCAGCAAA GAAGAATATA ATTGACCAGT CAATGAGGAC CTGATAACTA   
  
  
+ TAAAAAAAGA GGCGCATGGA CGGGCGCACC CACATGTTA  

- ACTATGGTCT ACTACACAGC ACTTACTAAA CTTTTAGCTT TCGTTACTTA GACTGCTTTT ACCTACTTCC   
  
  
- TACCCAGACC AATCAACAGA AAGAAAACCA CAAATCCGAC CTAATATCAC TAAAATTTCA TTTATTCTAC   
  
  
- CTCCTATCTG ATCTTACTAT GAAACTATTC AGCTAGACCT GCGGTGTTTC TAGTTAGATC CTAAAGCTGT   
  
  
- GGATATTTTA TAAACCTAAT CACAAAACAC AAAAAATTTT TTGAAAAAAA ACAAATCAAA AAAAAAGTAA   
  
  
- AGTGGCAAGT TGTTGAGTTA GAAAAAGAAG AAAAATATTA AAAAAAAGAT AAAGTAGAAA TTTTTAATCC   
  
  
- AACAAAACTT TTAAAACGAA GTATAAAAAA AAAAGACAAC CCAATATATA ATCAGAGTAC CTAAATTAAA   
  
  
- ATAAAAGAGC TAAAGTTGGA AGTTGTGATC TAGATAACCT TCACCCCAAA GTATTATAAA AAAAATAAAT   
  
  
- AAAAGATACT TAAAAAGAAC TAGAGTACTG ATCCAGTGTT CAAATTGTTC AATTGAGTTC AACTGACTCT   
  
  
- AGTAAAAGAA TAACGAAAAA AATTAACTAA AAAAATAAAG TAGGAAGTTA TAACCCAACC TACCTTTAAC   
  
  
- TCTAAGTATA TAAAAAAGAT AAATGAAAGA TACCCCAATA GGACTAGAGT ACACTCAAAT CGTCCAATTC   
  
  
- AGCCCAACTG AAATAAATAA ATAAAAAGAA GAAAAAATTA ACCTGAAACC TTAAAAAAAA TAAAATAAAA   
  
  
- ATACCCTAAT AGGTCTAGAA TACTGAGTTC AGTGCTCAAA TCAACCAACT GGACTCAACT GAACTAAATA   
  
  
- ATAAACCCAA GGAAAAAATT AACTTACAAA AAAAAAGATA AAGTGGAAGG TTGTTGAGTT AACAAAATAA   
  
  
- AGTGCAAAAA AAAGAAGTCA AAGTAGGAAG TTATAATTCA ACAAACCCTT AATCTAAAAC TTTAAAGAAA   
  
  
- AAGTTAAACC AAAAACACCT CAATTGGGCT AGATTATATA AATTAAAAAA AAGAACTAAA ATTAGAAAAT   
  
  
- GTAACCTAGA CAATCTTTTA CCTAGAAACA TTAAAAAATA AACTAAAAAA ACTTCAATAG AGTCAGAATA   
  
  
- CTAGATCCAG TGCTCGAATT GTATAATCGG ATTCAATTGA ACTCAACAAA AAAAATCAAC TAAAAAAAAT   
  
  
- AAAGTAGTAA GTTGTAATTC AATCAACATT TAATCCAAAG TATTAAAAAA ATATATACGA AAGATAACTC   
  
  
- AATATTTTAT AGGGCTAGAG TACTAGGCCC AACGCTCAAA TTGTAGAATT GAATGTAATT GAACCCATCA   
  
  
- AAAAAAATAG GAAAAAAATT AAATTATAAA AAAAAAGTTA AGGTGGGAAG TTATAGTTTA ACTAATCTCT   
  
  
- AACTAGAAGT ATTAAATAAA ACTAAATGAA AGATATATAT TCCAATAATA CCAAAGAACT AAATTCAAAG   
  
  
- TTTAAACTGT CTAATTGAAC ATAGTTAGAA TCTACAAAAA TAAAATTATA AATTTTTTAT GTAGGTTATA   
  
  
- TGAAATACAA AACTTAAATA TTAATTAAAA AAATAAACTT TTATACAATT GTAGCATCCA TATAAAAAAA   
  
  
- TATAATTTTT TAATTAACCT AAGTCTCAGT ATTTTTATTA GTCCAAAAGA GATTTTTAGC CATACGTAGT   
  
  
- GAAAGTTTTG TACTTGCGAG TGTAAGAGGG TTGCCTCCTT TTTACGGAGA TCTACAACAG TTTTCTTCTG   
  
  
- CGGAAATATT AATGTTAAAA AATATGGGTA AAAGGTATAG GGTTTTCCGA CTACTAATAC CGTAAAAGCC   
  
  
- TAAAAATTTT TTTTCTTTTT TTTACTATTT ATGTCTCCAC AAATCGTAAC TTTTCTTTTT ATTTAATTTC   
  
  
- CTGGACGGCA GTTATTTTAA ACCGTCGTTT CTTCTTATAT TAACTGGTCA GTTACTCCTG GACTATTGAT   
  
  
- ATTTTTTTCT CCGCGTACCT GCCCGCGTGG GTGTACAAT

+     ARE

| Site Name | Organism | Position | Strand | Matrix score. | sequence | function |
| --- | --- | --- | --- | --- | --- | --- |
| ARE | Zea mays | 988 | - | 6 | AAACCA | cis-acting regulatory element essential for the anaerobic induction |
| ARE | Zea mays | 1450 | - | 6 | AAACCA | cis-acting regulatory element essential for the anaerobic induction |

>Potri.006G138900.1   
+ TGATACCAGA TGATGTGTCG TGAATGATTT GAAAATCGAA AGCAATGAAT CTGACGAAAA TGGATGAAGG   
  
  
+ ATGGGTCTGG TTAGTTGTCT TTCTTTTGGT GTTTAGGCTG GATTATAGTG ATTTTAAAGT AAATAAGATG   
  
  
+ GAGGATAGAC TAGAATGATA CTTTGATAAG TCGATCTGGA CGCCACAAAG ATCAATCTAG GATTTCGACA   
  
  
+ CCTATAAAAT ATTTGGATTA GTGTTTTGTG TTTTTTAAAA AACTTTTTTT TGTTTAGTTT TTTTTTCATT   
  
  
+ TCACCGTTCA ACAACTCAAT CTTTTTCTTC TTTTTATAAT TTTTTTTCTA TTTCATCTTT AAAAATTAGG   
  
  
+ TTGTTTTGAA AATTTTGCTT CATATTTTTT TTTTCTGTTG GGTTATATAT TAGTCTCATG GATTTAATTT   
  
  
+ TATTTTCTCG ATTTCAACCT TCAACACTAG ATCTATTGGA AGTGGGGTTT CATAATATTT TTTTTATTTA   
  
  
+ TTTTCTATGA ATTTTTCTTG ATCTCATGAC TAGGTCACAA GTTTAACAAG TTAACTCAAG TTGACTGAGA   
  
  
+ TCATTTTCTT ATTGCTTTTT TTAATTGATT TTTTTATTTC ATCCTTCAAT ATTGGGTTGG ATGGAAATTG   
  
  
+ AGATTCATAT ATTTTTTCTA TTTACTTTCT ATGGGGTTAT CCTGATCTCA TGTGAGTTTA GCAGGTTAAG   
  
  
+ TCGGGTTGAC TTTATTTATT TATTTTTCTT CTTTTTTAAT TGGACTTTGG AATTTTTTTT ATTTTATTTT   
  
  
+ TATGGGATTA TCCAGATCTT ATGACTCAAG TCACGAGTTT AGTTGGTTGA CCTGAGTTGA CTTGATTTAT   
  
  
+ TATTTGGGTT CCTTTTTTAA TTGAATGTTT TTTTTTCTAT TTCACCTTCC AACAACTCAA TTGTTTTATT   
  
  
+ TCACGTTTTT TTTCTTCAGT TTCATCCTTC AATATTAAGT TGTTTGGGAA TTAGATTTTG AAATTTCTTT   
  
  
+ TTCAATTTGG TTTTTGTGGA GTTAACCCGA TCTAATATAT TTAATTTTTT TTCTTGATTT TAATCTTTTA   
  
  
+ CATTGGATCT GTTAGAAAAT GGATCTTTGT AATTTTTTAT TTGATTTTTT TGAAGTTATC TCAGTCTTAT   
  
  
+ GATCTAGGTC ACGAGCTTAA CATATTAGCC TAAGTTAACT TGAGTTGTTT TTTTTAGTTG ATTTTTTTTA   
  
  
+ TTTCATCATT CAACATTAAG TTAGTTGTAA ATTAGGTTTC ATAATTTTTT TATATATGCT TTCTATTGAG   
  
  
+ TTATAAAATA TCCCGATCTC ATGATCCGGG TTGCGAGTTT AACATCTTAA CTTACATTAA CTTGGGTAGT   
  
  
+ TTTTTTTATC CTTTTTTTAA TTTAATATTT TTTTTTCAAT TCCACCCTTC AATATCAAAT TGATTAGAGA   
  
  
+ TTGATCTTCA TAATTTATTT TGATTTACTT TCTATATATA AGGTTATTAT GGTTTCTTGA TTTAAGTTTC   
  
  
+ AAATTTGACA GATTAACTTG TATCAATCTT AGATGTTTTT ATTTTAATAT TTAAAAAATA CATCCAATAT   
  
  
+ ACTTTATGTT TTGAATTTAT AATTAATTTT TTTATTTGAA AATATGTTAA CATCGTAGGT ATATTTTTTT   
  
  
+ ATATTAAAAA ATTAATTGGA TTCAGAGTCA TAAAAATAAT CAGGTTTTCT CTAAAAATCG GTATGCATCA   
  
  
+ CTTTCAAAAC ATGAACGCTC ACATTCTCCC AACGGAGGAA AAATGCCTCT AGATGTTGTC AAAAGAAGAC   
  
  
+ GCCTTTATAA TTACAATTTT TTATACCCAT TTTCCATATC CCAAAAGGCT GATGATTATG GCATTTTCGG   
  
  
+ ATTTTTAAAA AAAAGAAAAA AAATGATAAA TACAGAGGTG TTTAGCATTG AAAAGAAAAA TAAATTAAAG   
  
  
+ GACCTGCCGT CAATAAAATT TGGCAGCAAA GAAGAATATA ATTGACCAGT CAATGAGGAC CTGATAACTA   
  
  
+ TAAAAAAAGA GGCGCATGGA CGGGCGCACC CACATGTTA  

- ACTATGGTCT ACTACACAGC ACTTACTAAA CTTTTAGCTT TCGTTACTTA GACTGCTTTT ACCTACTTCC   
  
  
- TACCCAGACC AATCAACAGA AAGAAAACCA CAAATCCGAC CTAATATCAC TAAAATTTCA TTTATTCTAC   
  
  
- CTCCTATCTG ATCTTACTAT GAAACTATTC AGCTAGACCT GCGGTGTTTC TAGTTAGATC CTAAAGCTGT   
  
  
- GGATATTTTA TAAACCTAAT CACAAAACAC AAAAAATTTT TTGAAAAAAA ACAAATCAAA AAAAAAGTAA   
  
  
- AGTGGCAAGT TGTTGAGTTA GAAAAAGAAG AAAAATATTA AAAAAAAGAT AAAGTAGAAA TTTTTAATCC   
  
  
- AACAAAACTT TTAAAACGAA GTATAAAAAA AAAAGACAAC CCAATATATA ATCAGAGTAC CTAAATTAAA   
  
  
- ATAAAAGAGC TAAAGTTGGA AGTTGTGATC TAGATAACCT TCACCCCAAA GTATTATAAA AAAAATAAAT   
  
  
- AAAAGATACT TAAAAAGAAC TAGAGTACTG ATCCAGTGTT CAAATTGTTC AATTGAGTTC AACTGACTCT   
  
  
- AGTAAAAGAA TAACGAAAAA AATTAACTAA AAAAATAAAG TAGGAAGTTA TAACCCAACC TACCTTTAAC   
  
  
- TCTAAGTATA TAAAAAAGAT AAATGAAAGA TACCCCAATA GGACTAGAGT ACACTCAAAT CGTCCAATTC   
  
  
- AGCCCAACTG AAATAAATAA ATAAAAAGAA GAAAAAATTA ACCTGAAACC TTAAAAAAAA TAAAATAAAA   
  
  
- ATACCCTAAT AGGTCTAGAA TACTGAGTTC AGTGCTCAAA TCAACCAACT GGACTCAACT GAACTAAATA   
  
  
- ATAAACCCAA GGAAAAAATT AACTTACAAA AAAAAAGATA AAGTGGAAGG TTGTTGAGTT AACAAAATAA   
  
  
- AGTGCAAAAA AAAGAAGTCA AAGTAGGAAG TTATAATTCA ACAAACCCTT AATCTAAAAC TTTAAAGAAA   
  
  
- AAGTTAAACC AAAAACACCT CAATTGGGCT AGATTATATA AATTAAAAAA AAGAACTAAA ATTAGAAAAT   
  
  
- GTAACCTAGA CAATCTTTTA CCTAGAAACA TTAAAAAATA AACTAAAAAA ACTTCAATAG AGTCAGAATA   
  
  
- CTAGATCCAG TGCTCGAATT GTATAATCGG ATTCAATTGA ACTCAACAAA AAAAATCAAC TAAAAAAAAT   
  
  
- AAAGTAGTAA GTTGTAATTC AATCAACATT TAATCCAAAG TATTAAAAAA ATATATACGA AAGATAACTC   
  
  
- AATATTTTAT AGGGCTAGAG TACTAGGCCC AACGCTCAAA TTGTAGAATT GAATGTAATT GAACCCATCA   
  
  
- AAAAAAATAG GAAAAAAATT AAATTATAAA AAAAAAGTTA AGGTGGGAAG TTATAGTTTA ACTAATCTCT   
  
  
- AACTAGAAGT ATTAAATAAA ACTAAATGAA AGATATATAT TCCAATAATA CCAAAGAACT AAATTCAAAG   
  
  
- TTTAAACTGT CTAATTGAAC ATAGTTAGAA TCTACAAAAA TAAAATTATA AATTTTTTAT GTAGGTTATA   
  
  
- TGAAATACAA AACTTAAATA TTAATTAAAA AAATAAACTT TTATACAATT GTAGCATCCA TATAAAAAAA   
  
  
- TATAATTTTT TAATTAACCT AAGTCTCAGT ATTTTTATTA GTCCAAAAGA GATTTTTAGC CATACGTAGT   
  
  
- GAAAGTTTTG TACTTGCGAG TGTAAGAGGG TTGCCTCCTT TTTACGGAGA TCTACAACAG TTTTCTTCTG   
  
  
- CGGAAATATT AATGTTAAAA AATATGGGTA AAAGGTATAG GGTTTTCCGA CTACTAATAC CGTAAAAGCC   
  
  
- TAAAAATTTT TTTTCTTTTT TTTACTATTT ATGTCTCCAC AAATCGTAAC TTTTCTTTTT ATTTAATTTC   
  
  
- CTGGACGGCA GTTATTTTAA ACCGTCGTTT CTTCTTATAT TAACTGGTCA GTTACTCCTG GACTATTGAT   
  
  
- ATTTTTTTCT CCGCGTACCT GCCCGCGTGG GTGTACAAT

+     ATCT-motif

| Site Name | Organism | Position | Strand | Matrix score. | sequence | function |
| --- | --- | --- | --- | --- | --- | --- |
| ATCT-motif | Pisum sativum | 958 | - | 9 | AATCTAATCC | part of a conserved DNA module involved in light responsiveness |

>Potri.006G138900.1   
+ TGATACCAGA TGATGTGTCG TGAATGATTT GAAAATCGAA AGCAATGAAT CTGACGAAAA TGGATGAAGG   
  
  
+ ATGGGTCTGG TTAGTTGTCT TTCTTTTGGT GTTTAGGCTG GATTATAGTG ATTTTAAAGT AAATAAGATG   
  
  
+ GAGGATAGAC TAGAATGATA CTTTGATAAG TCGATCTGGA CGCCACAAAG ATCAATCTAG GATTTCGACA   
  
  
+ CCTATAAAAT ATTTGGATTA GTGTTTTGTG TTTTTTAAAA AACTTTTTTT TGTTTAGTTT TTTTTTCATT   
  
  
+ TCACCGTTCA ACAACTCAAT CTTTTTCTTC TTTTTATAAT TTTTTTTCTA TTTCATCTTT AAAAATTAGG   
  
  
+ TTGTTTTGAA AATTTTGCTT CATATTTTTT TTTTCTGTTG GGTTATATAT TAGTCTCATG GATTTAATTT   
  
  
+ TATTTTCTCG ATTTCAACCT TCAACACTAG ATCTATTGGA AGTGGGGTTT CATAATATTT TTTTTATTTA   
  
  
+ TTTTCTATGA ATTTTTCTTG ATCTCATGAC TAGGTCACAA GTTTAACAAG TTAACTCAAG TTGACTGAGA   
  
  
+ TCATTTTCTT ATTGCTTTTT TTAATTGATT TTTTTATTTC ATCCTTCAAT ATTGGGTTGG ATGGAAATTG   
  
  
+ AGATTCATAT ATTTTTTCTA TTTACTTTCT ATGGGGTTAT CCTGATCTCA TGTGAGTTTA GCAGGTTAAG   
  
  
+ TCGGGTTGAC TTTATTTATT TATTTTTCTT CTTTTTTAAT TGGACTTTGG AATTTTTTTT ATTTTATTTT   
  
  
+ TATGGGATTA TCCAGATCTT ATGACTCAAG TCACGAGTTT AGTTGGTTGA CCTGAGTTGA CTTGATTTAT   
  
  
+ TATTTGGGTT CCTTTTTTAA TTGAATGTTT TTTTTTCTAT TTCACCTTCC AACAACTCAA TTGTTTTATT   
  
  
+ TCACGTTTTT TTTCTTCAGT TTCATCCTTC AATATTAAGT TGTTTGGGAA TTAGATTTTG AAATTTCTTT   
  
  
+ TTCAATTTGG TTTTTGTGGA GTTAACCCGA TCTAATATAT TTAATTTTTT TTCTTGATTT TAATCTTTTA   
  
  
+ CATTGGATCT GTTAGAAAAT GGATCTTTGT AATTTTTTAT TTGATTTTTT TGAAGTTATC TCAGTCTTAT   
  
  
+ GATCTAGGTC ACGAGCTTAA CATATTAGCC TAAGTTAACT TGAGTTGTTT TTTTTAGTTG ATTTTTTTTA   
  
  
+ TTTCATCATT CAACATTAAG TTAGTTGTAA ATTAGGTTTC ATAATTTTTT TATATATGCT TTCTATTGAG   
  
  
+ TTATAAAATA TCCCGATCTC ATGATCCGGG TTGCGAGTTT AACATCTTAA CTTACATTAA CTTGGGTAGT   
  
  
+ TTTTTTTATC CTTTTTTTAA TTTAATATTT TTTTTTCAAT TCCACCCTTC AATATCAAAT TGATTAGAGA   
  
  
+ TTGATCTTCA TAATTTATTT TGATTTACTT TCTATATATA AGGTTATTAT GGTTTCTTGA TTTAAGTTTC   
  
  
+ AAATTTGACA GATTAACTTG TATCAATCTT AGATGTTTTT ATTTTAATAT TTAAAAAATA CATCCAATAT   
  
  
+ ACTTTATGTT TTGAATTTAT AATTAATTTT TTTATTTGAA AATATGTTAA CATCGTAGGT ATATTTTTTT   
  
  
+ ATATTAAAAA ATTAATTGGA TTCAGAGTCA TAAAAATAAT CAGGTTTTCT CTAAAAATCG GTATGCATCA   
  
  
+ CTTTCAAAAC ATGAACGCTC ACATTCTCCC AACGGAGGAA AAATGCCTCT AGATGTTGTC AAAAGAAGAC   
  
  
+ GCCTTTATAA TTACAATTTT TTATACCCAT TTTCCATATC CCAAAAGGCT GATGATTATG GCATTTTCGG   
  
  
+ ATTTTTAAAA AAAAGAAAAA AAATGATAAA TACAGAGGTG TTTAGCATTG AAAAGAAAAA TAAATTAAAG   
  
  
+ GACCTGCCGT CAATAAAATT TGGCAGCAAA GAAGAATATA ATTGACCAGT CAATGAGGAC CTGATAACTA   
  
  
+ TAAAAAAAGA GGCGCATGGA CGGGCGCACC CACATGTTA  

- ACTATGGTCT ACTACACAGC ACTTACTAAA CTTTTAGCTT TCGTTACTTA GACTGCTTTT ACCTACTTCC   
  
  
- TACCCAGACC AATCAACAGA AAGAAAACCA CAAATCCGAC CTAATATCAC TAAAATTTCA TTTATTCTAC   
  
  
- CTCCTATCTG ATCTTACTAT GAAACTATTC AGCTAGACCT GCGGTGTTTC TAGTTAGATC CTAAAGCTGT   
  
  
- GGATATTTTA TAAACCTAAT CACAAAACAC AAAAAATTTT TTGAAAAAAA ACAAATCAAA AAAAAAGTAA   
  
  
- AGTGGCAAGT TGTTGAGTTA GAAAAAGAAG AAAAATATTA AAAAAAAGAT AAAGTAGAAA TTTTTAATCC   
  
  
- AACAAAACTT TTAAAACGAA GTATAAAAAA AAAAGACAAC CCAATATATA ATCAGAGTAC CTAAATTAAA   
  
  
- ATAAAAGAGC TAAAGTTGGA AGTTGTGATC TAGATAACCT TCACCCCAAA GTATTATAAA AAAAATAAAT   
  
  
- AAAAGATACT TAAAAAGAAC TAGAGTACTG ATCCAGTGTT CAAATTGTTC AATTGAGTTC AACTGACTCT   
  
  
- AGTAAAAGAA TAACGAAAAA AATTAACTAA AAAAATAAAG TAGGAAGTTA TAACCCAACC TACCTTTAAC   
  
  
- TCTAAGTATA TAAAAAAGAT AAATGAAAGA TACCCCAATA GGACTAGAGT ACACTCAAAT CGTCCAATTC   
  
  
- AGCCCAACTG AAATAAATAA ATAAAAAGAA GAAAAAATTA ACCTGAAACC TTAAAAAAAA TAAAATAAAA   
  
  
- ATACCCTAAT AGGTCTAGAA TACTGAGTTC AGTGCTCAAA TCAACCAACT GGACTCAACT GAACTAAATA   
  
  
- ATAAACCCAA GGAAAAAATT AACTTACAAA AAAAAAGATA AAGTGGAAGG TTGTTGAGTT AACAAAATAA   
  
  
- AGTGCAAAAA AAAGAAGTCA AAGTAGGAAG TTATAATTCA ACAAACCCTT AATCTAAAAC TTTAAAGAAA   
  
  
- AAGTTAAACC AAAAACACCT CAATTGGGCT AGATTATATA AATTAAAAAA AAGAACTAAA ATTAGAAAAT   
  
  
- GTAACCTAGA CAATCTTTTA CCTAGAAACA TTAAAAAATA AACTAAAAAA ACTTCAATAG AGTCAGAATA   
  
  
- CTAGATCCAG TGCTCGAATT GTATAATCGG ATTCAATTGA ACTCAACAAA AAAAATCAAC TAAAAAAAAT   
  
  
- AAAGTAGTAA GTTGTAATTC AATCAACATT TAATCCAAAG TATTAAAAAA ATATATACGA AAGATAACTC   
  
  
- AATATTTTAT AGGGCTAGAG TACTAGGCCC AACGCTCAAA TTGTAGAATT GAATGTAATT GAACCCATCA   
  
  
- AAAAAAATAG GAAAAAAATT AAATTATAAA AAAAAAGTTA AGGTGGGAAG TTATAGTTTA ACTAATCTCT   
  
  
- AACTAGAAGT ATTAAATAAA ACTAAATGAA AGATATATAT TCCAATAATA CCAAAGAACT AAATTCAAAG   
  
  
- TTTAAACTGT CTAATTGAAC ATAGTTAGAA TCTACAAAAA TAAAATTATA AATTTTTTAT GTAGGTTATA   
  
  
- TGAAATACAA AACTTAAATA TTAATTAAAA AAATAAACTT TTATACAATT GTAGCATCCA TATAAAAAAA   
  
  
- TATAATTTTT TAATTAACCT AAGTCTCAGT ATTTTTATTA GTCCAAAAGA GATTTTTAGC CATACGTAGT   
  
  
- GAAAGTTTTG TACTTGCGAG TGTAAGAGGG TTGCCTCCTT TTTACGGAGA TCTACAACAG TTTTCTTCTG   
  
  
- CGGAAATATT AATGTTAAAA AATATGGGTA AAAGGTATAG GGTTTTCCGA CTACTAATAC CGTAAAAGCC   
  
  
- TAAAAATTTT TTTTCTTTTT TTTACTATTT ATGTCTCCAC AAATCGTAAC TTTTCTTTTT ATTTAATTTC   
  
  
- CTGGACGGCA GTTATTTTAA ACCGTCGTTT CTTCTTATAT TAACTGGTCA GTTACTCCTG GACTATTGAT   
  
  
- ATTTTTTTCT CCGCGTACCT GCCCGCGTGG GTGTACAAT

+     AT~TATA-box

| Site Name | Organism | Position | Strand | Matrix score. | sequence | function |
| --- | --- | --- | --- | --- | --- | --- |
| AT~TATA-box | Arabidopsis thaliana | 1435 | - | 6 | TATATA |  |
| AT~TATA-box | Arabidopsis thaliana | 1433 | - | 6 | TATATA |  |
| AT~TATA-box | Arabidopsis thaliana | 1241 | - | 6 | TATATA |  |
| AT~TATA-box | Arabidopsis thaliana | 1239 | - | 8 | TATATAAA |  |
| AT~TATA-box | Arabidopsis thaliana | 394 | + | 6 | TATATA |  |

>Potri.006G138900.1   
+ TGATACCAGA TGATGTGTCG TGAATGATTT GAAAATCGAA AGCAATGAAT CTGACGAAAA TGGATGAAGG   
  
  
+ ATGGGTCTGG TTAGTTGTCT TTCTTTTGGT GTTTAGGCTG GATTATAGTG ATTTTAAAGT AAATAAGATG   
  
  
+ GAGGATAGAC TAGAATGATA CTTTGATAAG TCGATCTGGA CGCCACAAAG ATCAATCTAG GATTTCGACA   
  
  
+ CCTATAAAAT ATTTGGATTA GTGTTTTGTG TTTTTTAAAA AACTTTTTTT TGTTTAGTTT TTTTTTCATT   
  
  
+ TCACCGTTCA ACAACTCAAT CTTTTTCTTC TTTTTATAAT TTTTTTTCTA TTTCATCTTT AAAAATTAGG   
  
  
+ TTGTTTTGAA AATTTTGCTT CATATTTTTT TTTTCTGTTG GGTTATATAT TAGTCTCATG GATTTAATTT   
  
  
+ TATTTTCTCG ATTTCAACCT TCAACACTAG ATCTATTGGA AGTGGGGTTT CATAATATTT TTTTTATTTA   
  
  
+ TTTTCTATGA ATTTTTCTTG ATCTCATGAC TAGGTCACAA GTTTAACAAG TTAACTCAAG TTGACTGAGA   
  
  
+ TCATTTTCTT ATTGCTTTTT TTAATTGATT TTTTTATTTC ATCCTTCAAT ATTGGGTTGG ATGGAAATTG   
  
  
+ AGATTCATAT ATTTTTTCTA TTTACTTTCT ATGGGGTTAT CCTGATCTCA TGTGAGTTTA GCAGGTTAAG   
  
  
+ TCGGGTTGAC TTTATTTATT TATTTTTCTT CTTTTTTAAT TGGACTTTGG AATTTTTTTT ATTTTATTTT   
  
  
+ TATGGGATTA TCCAGATCTT ATGACTCAAG TCACGAGTTT AGTTGGTTGA CCTGAGTTGA CTTGATTTAT   
  
  
+ TATTTGGGTT CCTTTTTTAA TTGAATGTTT TTTTTTCTAT TTCACCTTCC AACAACTCAA TTGTTTTATT   
  
  
+ TCACGTTTTT TTTCTTCAGT TTCATCCTTC AATATTAAGT TGTTTGGGAA TTAGATTTTG AAATTTCTTT   
  
  
+ TTCAATTTGG TTTTTGTGGA GTTAACCCGA TCTAATATAT TTAATTTTTT TTCTTGATTT TAATCTTTTA   
  
  
+ CATTGGATCT GTTAGAAAAT GGATCTTTGT AATTTTTTAT TTGATTTTTT TGAAGTTATC TCAGTCTTAT   
  
  
+ GATCTAGGTC ACGAGCTTAA CATATTAGCC TAAGTTAACT TGAGTTGTTT TTTTTAGTTG ATTTTTTTTA   
  
  
+ TTTCATCATT CAACATTAAG TTAGTTGTAA ATTAGGTTTC ATAATTTTTT TATATATGCT TTCTATTGAG   
  
  
+ TTATAAAATA TCCCGATCTC ATGATCCGGG TTGCGAGTTT AACATCTTAA CTTACATTAA CTTGGGTAGT   
  
  
+ TTTTTTTATC CTTTTTTTAA TTTAATATTT TTTTTTCAAT TCCACCCTTC AATATCAAAT TGATTAGAGA   
  
  
+ TTGATCTTCA TAATTTATTT TGATTTACTT TCTATATATA AGGTTATTAT GGTTTCTTGA TTTAAGTTTC   
  
  
+ AAATTTGACA GATTAACTTG TATCAATCTT AGATGTTTTT ATTTTAATAT TTAAAAAATA CATCCAATAT   
  
  
+ ACTTTATGTT TTGAATTTAT AATTAATTTT TTTATTTGAA AATATGTTAA CATCGTAGGT ATATTTTTTT   
  
  
+ ATATTAAAAA ATTAATTGGA TTCAGAGTCA TAAAAATAAT CAGGTTTTCT CTAAAAATCG GTATGCATCA   
  
  
+ CTTTCAAAAC ATGAACGCTC ACATTCTCCC AACGGAGGAA AAATGCCTCT AGATGTTGTC AAAAGAAGAC   
  
  
+ GCCTTTATAA TTACAATTTT TTATACCCAT TTTCCATATC CCAAAAGGCT GATGATTATG GCATTTTCGG   
  
  
+ ATTTTTAAAA AAAAGAAAAA AAATGATAAA TACAGAGGTG TTTAGCATTG AAAAGAAAAA TAAATTAAAG   
  
  
+ GACCTGCCGT CAATAAAATT TGGCAGCAAA GAAGAATATA ATTGACCAGT CAATGAGGAC CTGATAACTA   
  
  
+ TAAAAAAAGA GGCGCATGGA CGGGCGCACC CACATGTTA  

- ACTATGGTCT ACTACACAGC ACTTACTAAA CTTTTAGCTT TCGTTACTTA GACTGCTTTT ACCTACTTCC   
  
  
- TACCCAGACC AATCAACAGA AAGAAAACCA CAAATCCGAC CTAATATCAC TAAAATTTCA TTTATTCTAC   
  
  
- CTCCTATCTG ATCTTACTAT GAAACTATTC AGCTAGACCT GCGGTGTTTC TAGTTAGATC CTAAAGCTGT   
  
  
- GGATATTTTA TAAACCTAAT CACAAAACAC AAAAAATTTT TTGAAAAAAA ACAAATCAAA AAAAAAGTAA   
  
  
- AGTGGCAAGT TGTTGAGTTA GAAAAAGAAG AAAAATATTA AAAAAAAGAT AAAGTAGAAA TTTTTAATCC   
  
  
- AACAAAACTT TTAAAACGAA GTATAAAAAA AAAAGACAAC CCAATATATA ATCAGAGTAC CTAAATTAAA   
  
  
- ATAAAAGAGC TAAAGTTGGA AGTTGTGATC TAGATAACCT TCACCCCAAA GTATTATAAA AAAAATAAAT   
  
  
- AAAAGATACT TAAAAAGAAC TAGAGTACTG ATCCAGTGTT CAAATTGTTC AATTGAGTTC AACTGACTCT   
  
  
- AGTAAAAGAA TAACGAAAAA AATTAACTAA AAAAATAAAG TAGGAAGTTA TAACCCAACC TACCTTTAAC   
  
  
- TCTAAGTATA TAAAAAAGAT AAATGAAAGA TACCCCAATA GGACTAGAGT ACACTCAAAT CGTCCAATTC   
  
  
- AGCCCAACTG AAATAAATAA ATAAAAAGAA GAAAAAATTA ACCTGAAACC TTAAAAAAAA TAAAATAAAA   
  
  
- ATACCCTAAT AGGTCTAGAA TACTGAGTTC AGTGCTCAAA TCAACCAACT GGACTCAACT GAACTAAATA   
  
  
- ATAAACCCAA GGAAAAAATT AACTTACAAA AAAAAAGATA AAGTGGAAGG TTGTTGAGTT AACAAAATAA   
  
  
- AGTGCAAAAA AAAGAAGTCA AAGTAGGAAG TTATAATTCA ACAAACCCTT AATCTAAAAC TTTAAAGAAA   
  
  
- AAGTTAAACC AAAAACACCT CAATTGGGCT AGATTATATA AATTAAAAAA AAGAACTAAA ATTAGAAAAT   
  
  
- GTAACCTAGA CAATCTTTTA CCTAGAAACA TTAAAAAATA AACTAAAAAA ACTTCAATAG AGTCAGAATA   
  
  
- CTAGATCCAG TGCTCGAATT GTATAATCGG ATTCAATTGA ACTCAACAAA AAAAATCAAC TAAAAAAAAT   
  
  
- AAAGTAGTAA GTTGTAATTC AATCAACATT TAATCCAAAG TATTAAAAAA ATATATACGA AAGATAACTC   
  
  
- AATATTTTAT AGGGCTAGAG TACTAGGCCC AACGCTCAAA TTGTAGAATT GAATGTAATT GAACCCATCA   
  
  
- AAAAAAATAG GAAAAAAATT AAATTATAAA AAAAAAGTTA AGGTGGGAAG TTATAGTTTA ACTAATCTCT   
  
  
- AACTAGAAGT ATTAAATAAA ACTAAATGAA AGATATATAT TCCAATAATA CCAAAGAACT AAATTCAAAG   
  
  
- TTTAAACTGT CTAATTGAAC ATAGTTAGAA TCTACAAAAA TAAAATTATA AATTTTTTAT GTAGGTTATA   
  
  
- TGAAATACAA AACTTAAATA TTAATTAAAA AAATAAACTT TTATACAATT GTAGCATCCA TATAAAAAAA   
  
  
- TATAATTTTT TAATTAACCT AAGTCTCAGT ATTTTTATTA GTCCAAAAGA GATTTTTAGC CATACGTAGT   
  
  
- GAAAGTTTTG TACTTGCGAG TGTAAGAGGG TTGCCTCCTT TTTACGGAGA TCTACAACAG TTTTCTTCTG   
  
  
- CGGAAATATT AATGTTAAAA AATATGGGTA AAAGGTATAG GGTTTTCCGA CTACTAATAC CGTAAAAGCC   
  
  
- TAAAAATTTT TTTTCTTTTT TTTACTATTT ATGTCTCCAC AAATCGTAAC TTTTCTTTTT ATTTAATTTC   
  
  
- CTGGACGGCA GTTATTTTAA ACCGTCGTTT CTTCTTATAT TAACTGGTCA GTTACTCCTG GACTATTGAT   
  
  
- ATTTTTTTCT CCGCGTACCT GCCCGCGTGG GTGTACAAT

+     Box 4

| Site Name | Organism | Position | Strand | Matrix score. | sequence | function |
| --- | --- | --- | --- | --- | --- | --- |
| Box 4 | Petroselinum crispum | 1621 | - | 6 | ATTAAT | part of a conserved DNA module involved in light responsiveness |
| Box 4 | Petroselinum crispum | 1562 | - | 6 | ATTAAT | part of a conserved DNA module involved in light responsiveness |

>Potri.006G138900.1   
+ TGATACCAGA TGATGTGTCG TGAATGATTT GAAAATCGAA AGCAATGAAT CTGACGAAAA TGGATGAAGG   
  
  
+ ATGGGTCTGG TTAGTTGTCT TTCTTTTGGT GTTTAGGCTG GATTATAGTG ATTTTAAAGT AAATAAGATG   
  
  
+ GAGGATAGAC TAGAATGATA CTTTGATAAG TCGATCTGGA CGCCACAAAG ATCAATCTAG GATTTCGACA   
  
  
+ CCTATAAAAT ATTTGGATTA GTGTTTTGTG TTTTTTAAAA AACTTTTTTT TGTTTAGTTT TTTTTTCATT   
  
  
+ TCACCGTTCA ACAACTCAAT CTTTTTCTTC TTTTTATAAT TTTTTTTCTA TTTCATCTTT AAAAATTAGG   
  
  
+ TTGTTTTGAA AATTTTGCTT CATATTTTTT TTTTCTGTTG GGTTATATAT TAGTCTCATG GATTTAATTT   
  
  
+ TATTTTCTCG ATTTCAACCT TCAACACTAG ATCTATTGGA AGTGGGGTTT CATAATATTT TTTTTATTTA   
  
  
+ TTTTCTATGA ATTTTTCTTG ATCTCATGAC TAGGTCACAA GTTTAACAAG TTAACTCAAG TTGACTGAGA   
  
  
+ TCATTTTCTT ATTGCTTTTT TTAATTGATT TTTTTATTTC ATCCTTCAAT ATTGGGTTGG ATGGAAATTG   
  
  
+ AGATTCATAT ATTTTTTCTA TTTACTTTCT ATGGGGTTAT CCTGATCTCA TGTGAGTTTA GCAGGTTAAG   
  
  
+ TCGGGTTGAC TTTATTTATT TATTTTTCTT CTTTTTTAAT TGGACTTTGG AATTTTTTTT ATTTTATTTT   
  
  
+ TATGGGATTA TCCAGATCTT ATGACTCAAG TCACGAGTTT AGTTGGTTGA CCTGAGTTGA CTTGATTTAT   
  
  
+ TATTTGGGTT CCTTTTTTAA TTGAATGTTT TTTTTTCTAT TTCACCTTCC AACAACTCAA TTGTTTTATT   
  
  
+ TCACGTTTTT TTTCTTCAGT TTCATCCTTC AATATTAAGT TGTTTGGGAA TTAGATTTTG AAATTTCTTT   
  
  
+ TTCAATTTGG TTTTTGTGGA GTTAACCCGA TCTAATATAT TTAATTTTTT TTCTTGATTT TAATCTTTTA   
  
  
+ CATTGGATCT GTTAGAAAAT GGATCTTTGT AATTTTTTAT TTGATTTTTT TGAAGTTATC TCAGTCTTAT   
  
  
+ GATCTAGGTC ACGAGCTTAA CATATTAGCC TAAGTTAACT TGAGTTGTTT TTTTTAGTTG ATTTTTTTTA   
  
  
+ TTTCATCATT CAACATTAAG TTAGTTGTAA ATTAGGTTTC ATAATTTTTT TATATATGCT TTCTATTGAG   
  
  
+ TTATAAAATA TCCCGATCTC ATGATCCGGG TTGCGAGTTT AACATCTTAA CTTACATTAA CTTGGGTAGT   
  
  
+ TTTTTTTATC CTTTTTTTAA TTTAATATTT TTTTTTCAAT TCCACCCTTC AATATCAAAT TGATTAGAGA   
  
  
+ TTGATCTTCA TAATTTATTT TGATTTACTT TCTATATATA AGGTTATTAT GGTTTCTTGA TTTAAGTTTC   
  
  
+ AAATTTGACA GATTAACTTG TATCAATCTT AGATGTTTTT ATTTTAATAT TTAAAAAATA CATCCAATAT   
  
  
+ ACTTTATGTT TTGAATTTAT AATTAATTTT TTTATTTGAA AATATGTTAA CATCGTAGGT ATATTTTTTT   
  
  
+ ATATTAAAAA ATTAATTGGA TTCAGAGTCA TAAAAATAAT CAGGTTTTCT CTAAAAATCG GTATGCATCA   
  
  
+ CTTTCAAAAC ATGAACGCTC ACATTCTCCC AACGGAGGAA AAATGCCTCT AGATGTTGTC AAAAGAAGAC   
  
  
+ GCCTTTATAA TTACAATTTT TTATACCCAT TTTCCATATC CCAAAAGGCT GATGATTATG GCATTTTCGG   
  
  
+ ATTTTTAAAA AAAAGAAAAA AAATGATAAA TACAGAGGTG TTTAGCATTG AAAAGAAAAA TAAATTAAAG   
  
  
+ GACCTGCCGT CAATAAAATT TGGCAGCAAA GAAGAATATA ATTGACCAGT CAATGAGGAC CTGATAACTA   
  
  
+ TAAAAAAAGA GGCGCATGGA CGGGCGCACC CACATGTTA  

- ACTATGGTCT ACTACACAGC ACTTACTAAA CTTTTAGCTT TCGTTACTTA GACTGCTTTT ACCTACTTCC   
  
  
- TACCCAGACC AATCAACAGA AAGAAAACCA CAAATCCGAC CTAATATCAC TAAAATTTCA TTTATTCTAC   
  
  
- CTCCTATCTG ATCTTACTAT GAAACTATTC AGCTAGACCT GCGGTGTTTC TAGTTAGATC CTAAAGCTGT   
  
  
- GGATATTTTA TAAACCTAAT CACAAAACAC AAAAAATTTT TTGAAAAAAA ACAAATCAAA AAAAAAGTAA   
  
  
- AGTGGCAAGT TGTTGAGTTA GAAAAAGAAG AAAAATATTA AAAAAAAGAT AAAGTAGAAA TTTTTAATCC   
  
  
- AACAAAACTT TTAAAACGAA GTATAAAAAA AAAAGACAAC CCAATATATA ATCAGAGTAC CTAAATTAAA   
  
  
- ATAAAAGAGC TAAAGTTGGA AGTTGTGATC TAGATAACCT TCACCCCAAA GTATTATAAA AAAAATAAAT   
  
  
- AAAAGATACT TAAAAAGAAC TAGAGTACTG ATCCAGTGTT CAAATTGTTC AATTGAGTTC AACTGACTCT   
  
  
- AGTAAAAGAA TAACGAAAAA AATTAACTAA AAAAATAAAG TAGGAAGTTA TAACCCAACC TACCTTTAAC   
  
  
- TCTAAGTATA TAAAAAAGAT AAATGAAAGA TACCCCAATA GGACTAGAGT ACACTCAAAT CGTCCAATTC   
  
  
- AGCCCAACTG AAATAAATAA ATAAAAAGAA GAAAAAATTA ACCTGAAACC TTAAAAAAAA TAAAATAAAA   
  
  
- ATACCCTAAT AGGTCTAGAA TACTGAGTTC AGTGCTCAAA TCAACCAACT GGACTCAACT GAACTAAATA   
  
  
- ATAAACCCAA GGAAAAAATT AACTTACAAA AAAAAAGATA AAGTGGAAGG TTGTTGAGTT AACAAAATAA   
  
  
- AGTGCAAAAA AAAGAAGTCA AAGTAGGAAG TTATAATTCA ACAAACCCTT AATCTAAAAC TTTAAAGAAA   
  
  
- AAGTTAAACC AAAAACACCT CAATTGGGCT AGATTATATA AATTAAAAAA AAGAACTAAA ATTAGAAAAT   
  
  
- GTAACCTAGA CAATCTTTTA CCTAGAAACA TTAAAAAATA AACTAAAAAA ACTTCAATAG AGTCAGAATA   
  
  
- CTAGATCCAG TGCTCGAATT GTATAATCGG ATTCAATTGA ACTCAACAAA AAAAATCAAC TAAAAAAAAT   
  
  
- AAAGTAGTAA GTTGTAATTC AATCAACATT TAATCCAAAG TATTAAAAAA ATATATACGA AAGATAACTC   
  
  
- AATATTTTAT AGGGCTAGAG TACTAGGCCC AACGCTCAAA TTGTAGAATT GAATGTAATT GAACCCATCA   
  
  
- AAAAAAATAG GAAAAAAATT AAATTATAAA AAAAAAGTTA AGGTGGGAAG TTATAGTTTA ACTAATCTCT   
  
  
- AACTAGAAGT ATTAAATAAA ACTAAATGAA AGATATATAT TCCAATAATA CCAAAGAACT AAATTCAAAG   
  
  
- TTTAAACTGT CTAATTGAAC ATAGTTAGAA TCTACAAAAA TAAAATTATA AATTTTTTAT GTAGGTTATA   
  
  
- TGAAATACAA AACTTAAATA TTAATTAAAA AAATAAACTT TTATACAATT GTAGCATCCA TATAAAAAAA   
  
  
- TATAATTTTT TAATTAACCT AAGTCTCAGT ATTTTTATTA GTCCAAAAGA GATTTTTAGC CATACGTAGT   
  
  
- GAAAGTTTTG TACTTGCGAG TGTAAGAGGG TTGCCTCCTT TTTACGGAGA TCTACAACAG TTTTCTTCTG   
  
  
- CGGAAATATT AATGTTAAAA AATATGGGTA AAAGGTATAG GGTTTTCCGA CTACTAATAC CGTAAAAGCC   
  
  
- TAAAAATTTT TTTTCTTTTT TTTACTATTT ATGTCTCCAC AAATCGTAAC TTTTCTTTTT ATTTAATTTC   
  
  
- CTGGACGGCA GTTATTTTAA ACCGTCGTTT CTTCTTATAT TAACTGGTCA GTTACTCCTG GACTATTGAT   
  
  
- ATTTTTTTCT CCGCGTACCT GCCCGCGTGG GTGTACAAT

+     CAAT-box

| Site Name | Organism | Position | Strand | Matrix score. | sequence | function |
| --- | --- | --- | --- | --- | --- | --- |
| CAAT-box | Nicotiana glutinosa | 983 | + | 4 | CAAT |  |
| CAAT-box | Arabidopsis thaliana | 1052 | - | 5 | CCAAT | common cis-acting element in promoter and enhancer regions |
| CAAT-box | Nicotiana glutinosa | 607 | + | 4 | CAAT |  |
| CAAT-box | Nicotiana glutinosa | 900 | - | 4 | CAAT |  |
| CAAT-box | Arabidopsis thaliana | 739 | - | 5 | CCAAT | common cis-acting element in promoter and enhancer regions |
| CAAT-box | Nicotiana glutinosa | 898 | + | 4 | CAAT |  |
| CAAT-box | Nicotiana glutinosa | 1931 | - | 4 | CAAT |  |
| CAAT-box | Nicotiana glutinosa | 1901 | + | 4 | CAAT |  |
| CAAT-box | Nicotiana glutinosa | 1867 | - | 4 | CAAT |  |
| CAAT-box | Nicotiana glutinosa | 1494 | + | 4 | CAAT |  |
| CAAT-box | Pisum sativum | 1089 | - | 5 | CAAAT | common cis-acting element in promoter and enhancer regions |
| CAAT-box | Arabidopsis thaliana | 611 | - | 5 | CCAAT | common cis-acting element in promoter and enhancer regions |
| CAAT-box | Arabidopsis thaliana | 1625 | - | 5 | CCAAT | common cis-acting element in promoter and enhancer regions |
| CAAT-box | Nicotiana glutinosa | 1255 | - | 4 | CAAT |  |
| CAAT-box | Nicotiana glutinosa | 1764 | + | 4 | CAAT |  |
| CAAT-box | Nicotiana glutinosa | 571 | - | 4 | CAAT |  |
| CAAT-box | Nicotiana glutinosa | 584 | - | 4 | CAAT |  |
| CAAT-box | Pisum sativum | 1470 | + | 5 | CAAAT | common cis-acting element in promoter and enhancer regions |
| CAAT-box | Pisum sativum | 1908 | - | 5 | CAAAT | common cis-acting element in promoter and enhancer regions |
| CAAT-box | Nicotiana glutinosa | 1941 | + | 4 | CAAT |  |
| CAAT-box | Nicotiana glutinosa | 940 | + | 4 | CAAT |  |
| CAAT-box | Nicotiana glutinosa | 627 | - | 4 | CAAT |  |
| CAAT-box | Pisum sativum | 1386 | + | 5 | CAAAT | common cis-acting element in promoter and enhancer regions |
| CAAT-box | Arabidopsis thaliana | 455 | - | 5 | CCAAT | common cis-acting element in promoter and enhancer regions |
| CAAT-box | Nicotiana glutinosa | 1400 | - | 4 | CAAT |  |
| CAAT-box | Pisum sativum | 985 | - | 5 | CAAAT | common cis-acting element in promoter and enhancer regions |
| CAAT-box | Nicotiana glutinosa | 43 | + | 4 | CAAT |  |
| CAAT-box | Pisum sativum | 1574 | - | 5 | CAAAT | common cis-acting element in promoter and enhancer regions |
| CAAT-box | Nicotiana glutinosa | 1535 | + | 4 | CAAT |  |
| CAAT-box | Arabidopsis thaliana | 1534 | + | 5 | CCAAT | common cis-acting element in promoter and enhancer regions |
| CAAT-box | Nicotiana glutinosa | 193 | + | 4 | CAAT |  |
| CAAT-box | Nicotiana glutinosa | 297 | + | 4 | CAAT |  |
| CAAT-box | Pisum sativum | 842 | - | 5 | CAAAT | common cis-acting element in promoter and enhancer regions |
| CAAT-box | Nicotiana glutinosa | 1367 | + | 4 | CAAT |  |
| CAAT-box | Pisum sativum | 27 | - | 5 | CAAAT | common cis-acting element in promoter and enhancer regions |
| CAAT-box | Pisum sativum | 1473 | - | 5 | CAAAT | common cis-acting element in promoter and enhancer regions |
| CAAT-box | Nicotiana glutinosa | 1389 | - | 4 | CAAT |  |
| CAAT-box | Nicotiana glutinosa | 860 | - | 4 | CAAT |  |
| CAAT-box | Pisum sativum | 221 | - | 5 | CAAAT | common cis-acting element in promoter and enhancer regions |
| CAAT-box | Nicotiana glutinosa | 1380 | + | 4 | CAAT |  |

>Potri.006G138900.1   
+ TGATACCAGA TGATGTGTCG TGAATGATTT GAAAATCGAA AGCAATGAAT CTGACGAAAA TGGATGAAGG   
  
  
+ ATGGGTCTGG TTAGTTGTCT TTCTTTTGGT GTTTAGGCTG GATTATAGTG ATTTTAAAGT AAATAAGATG   
  
  
+ GAGGATAGAC TAGAATGATA CTTTGATAAG TCGATCTGGA CGCCACAAAG ATCAATCTAG GATTTCGACA   
  
  
+ CCTATAAAAT ATTTGGATTA GTGTTTTGTG TTTTTTAAAA AACTTTTTTT TGTTTAGTTT TTTTTTCATT   
  
  
+ TCACCGTTCA ACAACTCAAT CTTTTTCTTC TTTTTATAAT TTTTTTTCTA TTTCATCTTT AAAAATTAGG   
  
  
+ TTGTTTTGAA AATTTTGCTT CATATTTTTT TTTTCTGTTG GGTTATATAT TAGTCTCATG GATTTAATTT   
  
  
+ TATTTTCTCG ATTTCAACCT TCAACACTAG ATCTATTGGA AGTGGGGTTT CATAATATTT TTTTTATTTA   
  
  
+ TTTTCTATGA ATTTTTCTTG ATCTCATGAC TAGGTCACAA GTTTAACAAG TTAACTCAAG TTGACTGAGA   
  
  
+ TCATTTTCTT ATTGCTTTTT TTAATTGATT TTTTTATTTC ATCCTTCAAT ATTGGGTTGG ATGGAAATTG   
  
  
+ AGATTCATAT ATTTTTTCTA TTTACTTTCT ATGGGGTTAT CCTGATCTCA TGTGAGTTTA GCAGGTTAAG   
  
  
+ TCGGGTTGAC TTTATTTATT TATTTTTCTT CTTTTTTAAT TGGACTTTGG AATTTTTTTT ATTTTATTTT   
  
  
+ TATGGGATTA TCCAGATCTT ATGACTCAAG TCACGAGTTT AGTTGGTTGA CCTGAGTTGA CTTGATTTAT   
  
  
+ TATTTGGGTT CCTTTTTTAA TTGAATGTTT TTTTTTCTAT TTCACCTTCC AACAACTCAA TTGTTTTATT   
  
  
+ TCACGTTTTT TTTCTTCAGT TTCATCCTTC AATATTAAGT TGTTTGGGAA TTAGATTTTG AAATTTCTTT   
  
  
+ TTCAATTTGG TTTTTGTGGA GTTAACCCGA TCTAATATAT TTAATTTTTT TTCTTGATTT TAATCTTTTA   
  
  
+ CATTGGATCT GTTAGAAAAT GGATCTTTGT AATTTTTTAT TTGATTTTTT TGAAGTTATC TCAGTCTTAT   
  
  
+ GATCTAGGTC ACGAGCTTAA CATATTAGCC TAAGTTAACT TGAGTTGTTT TTTTTAGTTG ATTTTTTTTA   
  
  
+ TTTCATCATT CAACATTAAG TTAGTTGTAA ATTAGGTTTC ATAATTTTTT TATATATGCT TTCTATTGAG   
  
  
+ TTATAAAATA TCCCGATCTC ATGATCCGGG TTGCGAGTTT AACATCTTAA CTTACATTAA CTTGGGTAGT   
  
  
+ TTTTTTTATC CTTTTTTTAA TTTAATATTT TTTTTTCAAT TCCACCCTTC AATATCAAAT TGATTAGAGA   
  
  
+ TTGATCTTCA TAATTTATTT TGATTTACTT TCTATATATA AGGTTATTAT GGTTTCTTGA TTTAAGTTTC   
  
  
+ AAATTTGACA GATTAACTTG TATCAATCTT AGATGTTTTT ATTTTAATAT TTAAAAAATA CATCCAATAT   
  
  
+ ACTTTATGTT TTGAATTTAT AATTAATTTT TTTATTTGAA AATATGTTAA CATCGTAGGT ATATTTTTTT   
  
  
+ ATATTAAAAA ATTAATTGGA TTCAGAGTCA TAAAAATAAT CAGGTTTTCT CTAAAAATCG GTATGCATCA   
  
  
+ CTTTCAAAAC ATGAACGCTC ACATTCTCCC AACGGAGGAA AAATGCCTCT AGATGTTGTC AAAAGAAGAC   
  
  
+ GCCTTTATAA TTACAATTTT TTATACCCAT TTTCCATATC CCAAAAGGCT GATGATTATG GCATTTTCGG   
  
  
+ ATTTTTAAAA AAAAGAAAAA AAATGATAAA TACAGAGGTG TTTAGCATTG AAAAGAAAAA TAAATTAAAG   
  
  
+ GACCTGCCGT CAATAAAATT TGGCAGCAAA GAAGAATATA ATTGACCAGT CAATGAGGAC CTGATAACTA   
  
  
+ TAAAAAAAGA GGCGCATGGA CGGGCGCACC CACATGTTA  

- ACTATGGTCT ACTACACAGC ACTTACTAAA CTTTTAGCTT TCGTTACTTA GACTGCTTTT ACCTACTTCC   
  
  
- TACCCAGACC AATCAACAGA AAGAAAACCA CAAATCCGAC CTAATATCAC TAAAATTTCA TTTATTCTAC   
  
  
- CTCCTATCTG ATCTTACTAT GAAACTATTC AGCTAGACCT GCGGTGTTTC TAGTTAGATC CTAAAGCTGT   
  
  
- GGATATTTTA TAAACCTAAT CACAAAACAC AAAAAATTTT TTGAAAAAAA ACAAATCAAA AAAAAAGTAA   
  
  
- AGTGGCAAGT TGTTGAGTTA GAAAAAGAAG AAAAATATTA AAAAAAAGAT AAAGTAGAAA TTTTTAATCC   
  
  
- AACAAAACTT TTAAAACGAA GTATAAAAAA AAAAGACAAC CCAATATATA ATCAGAGTAC CTAAATTAAA   
  
  
- ATAAAAGAGC TAAAGTTGGA AGTTGTGATC TAGATAACCT TCACCCCAAA GTATTATAAA AAAAATAAAT   
  
  
- AAAAGATACT TAAAAAGAAC TAGAGTACTG ATCCAGTGTT CAAATTGTTC AATTGAGTTC AACTGACTCT   
  
  
- AGTAAAAGAA TAACGAAAAA AATTAACTAA AAAAATAAAG TAGGAAGTTA TAACCCAACC TACCTTTAAC   
  
  
- TCTAAGTATA TAAAAAAGAT AAATGAAAGA TACCCCAATA GGACTAGAGT ACACTCAAAT CGTCCAATTC   
  
  
- AGCCCAACTG AAATAAATAA ATAAAAAGAA GAAAAAATTA ACCTGAAACC TTAAAAAAAA TAAAATAAAA   
  
  
- ATACCCTAAT AGGTCTAGAA TACTGAGTTC AGTGCTCAAA TCAACCAACT GGACTCAACT GAACTAAATA   
  
  
- ATAAACCCAA GGAAAAAATT AACTTACAAA AAAAAAGATA AAGTGGAAGG TTGTTGAGTT AACAAAATAA   
  
  
- AGTGCAAAAA AAAGAAGTCA AAGTAGGAAG TTATAATTCA ACAAACCCTT AATCTAAAAC TTTAAAGAAA   
  
  
- AAGTTAAACC AAAAACACCT CAATTGGGCT AGATTATATA AATTAAAAAA AAGAACTAAA ATTAGAAAAT   
  
  
- GTAACCTAGA CAATCTTTTA CCTAGAAACA TTAAAAAATA AACTAAAAAA ACTTCAATAG AGTCAGAATA   
  
  
- CTAGATCCAG TGCTCGAATT GTATAATCGG ATTCAATTGA ACTCAACAAA AAAAATCAAC TAAAAAAAAT   
  
  
- AAAGTAGTAA GTTGTAATTC AATCAACATT TAATCCAAAG TATTAAAAAA ATATATACGA AAGATAACTC   
  
  
- AATATTTTAT AGGGCTAGAG TACTAGGCCC AACGCTCAAA TTGTAGAATT GAATGTAATT GAACCCATCA   
  
  
- AAAAAAATAG GAAAAAAATT AAATTATAAA AAAAAAGTTA AGGTGGGAAG TTATAGTTTA ACTAATCTCT   
  
  
- AACTAGAAGT ATTAAATAAA ACTAAATGAA AGATATATAT TCCAATAATA CCAAAGAACT AAATTCAAAG   
  
  
- TTTAAACTGT CTAATTGAAC ATAGTTAGAA TCTACAAAAA TAAAATTATA AATTTTTTAT GTAGGTTATA   
  
  
- TGAAATACAA AACTTAAATA TTAATTAAAA AAATAAACTT TTATACAATT GTAGCATCCA TATAAAAAAA   
  
  
- TATAATTTTT TAATTAACCT AAGTCTCAGT ATTTTTATTA GTCCAAAAGA GATTTTTAGC CATACGTAGT   
  
  
- GAAAGTTTTG TACTTGCGAG TGTAAGAGGG TTGCCTCCTT TTTACGGAGA TCTACAACAG TTTTCTTCTG   
  
  
- CGGAAATATT AATGTTAAAA AATATGGGTA AAAGGTATAG GGTTTTCCGA CTACTAATAC CGTAAAAGCC   
  
  
- TAAAAATTTT TTTTCTTTTT TTTACTATTT ATGTCTCCAC AAATCGTAAC TTTTCTTTTT ATTTAATTTC   
  
  
- CTGGACGGCA GTTATTTTAA ACCGTCGTTT CTTCTTATAT TAACTGGTCA GTTACTCCTG GACTATTGAT   
  
  
- ATTTTTTTCT CCGCGTACCT GCCCGCGTGG GTGTACAAT

+     CCAAT-box

| Site Name | Organism | Position | Strand | Matrix score. | sequence | function |
| --- | --- | --- | --- | --- | --- | --- |
| CCAAT-box | Hordeum vulgare | 1710 | + | 6 | CAACGG | MYBHv1 binding site |

>Potri.006G138900.1   
+ TGATACCAGA TGATGTGTCG TGAATGATTT GAAAATCGAA AGCAATGAAT CTGACGAAAA TGGATGAAGG   
  
  
+ ATGGGTCTGG TTAGTTGTCT TTCTTTTGGT GTTTAGGCTG GATTATAGTG ATTTTAAAGT AAATAAGATG   
  
  
+ GAGGATAGAC TAGAATGATA CTTTGATAAG TCGATCTGGA CGCCACAAAG ATCAATCTAG GATTTCGACA   
  
  
+ CCTATAAAAT ATTTGGATTA GTGTTTTGTG TTTTTTAAAA AACTTTTTTT TGTTTAGTTT TTTTTTCATT   
  
  
+ TCACCGTTCA ACAACTCAAT CTTTTTCTTC TTTTTATAAT TTTTTTTCTA TTTCATCTTT AAAAATTAGG   
  
  
+ TTGTTTTGAA AATTTTGCTT CATATTTTTT TTTTCTGTTG GGTTATATAT TAGTCTCATG GATTTAATTT   
  
  
+ TATTTTCTCG ATTTCAACCT TCAACACTAG ATCTATTGGA AGTGGGGTTT CATAATATTT TTTTTATTTA   
  
  
+ TTTTCTATGA ATTTTTCTTG ATCTCATGAC TAGGTCACAA GTTTAACAAG TTAACTCAAG TTGACTGAGA   
  
  
+ TCATTTTCTT ATTGCTTTTT TTAATTGATT TTTTTATTTC ATCCTTCAAT ATTGGGTTGG ATGGAAATTG   
  
  
+ AGATTCATAT ATTTTTTCTA TTTACTTTCT ATGGGGTTAT CCTGATCTCA TGTGAGTTTA GCAGGTTAAG   
  
  
+ TCGGGTTGAC TTTATTTATT TATTTTTCTT CTTTTTTAAT TGGACTTTGG AATTTTTTTT ATTTTATTTT   
  
  
+ TATGGGATTA TCCAGATCTT ATGACTCAAG TCACGAGTTT AGTTGGTTGA CCTGAGTTGA CTTGATTTAT   
  
  
+ TATTTGGGTT CCTTTTTTAA TTGAATGTTT TTTTTTCTAT TTCACCTTCC AACAACTCAA TTGTTTTATT   
  
  
+ TCACGTTTTT TTTCTTCAGT TTCATCCTTC AATATTAAGT TGTTTGGGAA TTAGATTTTG AAATTTCTTT   
  
  
+ TTCAATTTGG TTTTTGTGGA GTTAACCCGA TCTAATATAT TTAATTTTTT TTCTTGATTT TAATCTTTTA   
  
  
+ CATTGGATCT GTTAGAAAAT GGATCTTTGT AATTTTTTAT TTGATTTTTT TGAAGTTATC TCAGTCTTAT   
  
  
+ GATCTAGGTC ACGAGCTTAA CATATTAGCC TAAGTTAACT TGAGTTGTTT TTTTTAGTTG ATTTTTTTTA   
  
  
+ TTTCATCATT CAACATTAAG TTAGTTGTAA ATTAGGTTTC ATAATTTTTT TATATATGCT TTCTATTGAG   
  
  
+ TTATAAAATA TCCCGATCTC ATGATCCGGG TTGCGAGTTT AACATCTTAA CTTACATTAA CTTGGGTAGT   
  
  
+ TTTTTTTATC CTTTTTTTAA TTTAATATTT TTTTTTCAAT TCCACCCTTC AATATCAAAT TGATTAGAGA   
  
  
+ TTGATCTTCA TAATTTATTT TGATTTACTT TCTATATATA AGGTTATTAT GGTTTCTTGA TTTAAGTTTC   
  
  
+ AAATTTGACA GATTAACTTG TATCAATCTT AGATGTTTTT ATTTTAATAT TTAAAAAATA CATCCAATAT   
  
  
+ ACTTTATGTT TTGAATTTAT AATTAATTTT TTTATTTGAA AATATGTTAA CATCGTAGGT ATATTTTTTT   
  
  
+ ATATTAAAAA ATTAATTGGA TTCAGAGTCA TAAAAATAAT CAGGTTTTCT CTAAAAATCG GTATGCATCA   
  
  
+ CTTTCAAAAC ATGAACGCTC ACATTCTCCC AACGGAGGAA AAATGCCTCT AGATGTTGTC AAAAGAAGAC   
  
  
+ GCCTTTATAA TTACAATTTT TTATACCCAT TTTCCATATC CCAAAAGGCT GATGATTATG GCATTTTCGG   
  
  
+ ATTTTTAAAA AAAAGAAAAA AAATGATAAA TACAGAGGTG TTTAGCATTG AAAAGAAAAA TAAATTAAAG   
  
  
+ GACCTGCCGT CAATAAAATT TGGCAGCAAA GAAGAATATA ATTGACCAGT CAATGAGGAC CTGATAACTA   
  
  
+ TAAAAAAAGA GGCGCATGGA CGGGCGCACC CACATGTTA  

- ACTATGGTCT ACTACACAGC ACTTACTAAA CTTTTAGCTT TCGTTACTTA GACTGCTTTT ACCTACTTCC   
  
  
- TACCCAGACC AATCAACAGA AAGAAAACCA CAAATCCGAC CTAATATCAC TAAAATTTCA TTTATTCTAC   
  
  
- CTCCTATCTG ATCTTACTAT GAAACTATTC AGCTAGACCT GCGGTGTTTC TAGTTAGATC CTAAAGCTGT   
  
  
- GGATATTTTA TAAACCTAAT CACAAAACAC AAAAAATTTT TTGAAAAAAA ACAAATCAAA AAAAAAGTAA   
  
  
- AGTGGCAAGT TGTTGAGTTA GAAAAAGAAG AAAAATATTA AAAAAAAGAT AAAGTAGAAA TTTTTAATCC   
  
  
- AACAAAACTT TTAAAACGAA GTATAAAAAA AAAAGACAAC CCAATATATA ATCAGAGTAC CTAAATTAAA   
  
  
- ATAAAAGAGC TAAAGTTGGA AGTTGTGATC TAGATAACCT TCACCCCAAA GTATTATAAA AAAAATAAAT   
  
  
- AAAAGATACT TAAAAAGAAC TAGAGTACTG ATCCAGTGTT CAAATTGTTC AATTGAGTTC AACTGACTCT   
  
  
- AGTAAAAGAA TAACGAAAAA AATTAACTAA AAAAATAAAG TAGGAAGTTA TAACCCAACC TACCTTTAAC   
  
  
- TCTAAGTATA TAAAAAAGAT AAATGAAAGA TACCCCAATA GGACTAGAGT ACACTCAAAT CGTCCAATTC   
  
  
- AGCCCAACTG AAATAAATAA ATAAAAAGAA GAAAAAATTA ACCTGAAACC TTAAAAAAAA TAAAATAAAA   
  
  
- ATACCCTAAT AGGTCTAGAA TACTGAGTTC AGTGCTCAAA TCAACCAACT GGACTCAACT GAACTAAATA   
  
  
- ATAAACCCAA GGAAAAAATT AACTTACAAA AAAAAAGATA AAGTGGAAGG TTGTTGAGTT AACAAAATAA   
  
  
- AGTGCAAAAA AAAGAAGTCA AAGTAGGAAG TTATAATTCA ACAAACCCTT AATCTAAAAC TTTAAAGAAA   
  
  
- AAGTTAAACC AAAAACACCT CAATTGGGCT AGATTATATA AATTAAAAAA AAGAACTAAA ATTAGAAAAT   
  
  
- GTAACCTAGA CAATCTTTTA CCTAGAAACA TTAAAAAATA AACTAAAAAA ACTTCAATAG AGTCAGAATA   
  
  
- CTAGATCCAG TGCTCGAATT GTATAATCGG ATTCAATTGA ACTCAACAAA AAAAATCAAC TAAAAAAAAT   
  
  
- AAAGTAGTAA GTTGTAATTC AATCAACATT TAATCCAAAG TATTAAAAAA ATATATACGA AAGATAACTC   
  
  
- AATATTTTAT AGGGCTAGAG TACTAGGCCC AACGCTCAAA TTGTAGAATT GAATGTAATT GAACCCATCA   
  
  
- AAAAAAATAG GAAAAAAATT AAATTATAAA AAAAAAGTTA AGGTGGGAAG TTATAGTTTA ACTAATCTCT   
  
  
- AACTAGAAGT ATTAAATAAA ACTAAATGAA AGATATATAT TCCAATAATA CCAAAGAACT AAATTCAAAG   
  
  
- TTTAAACTGT CTAATTGAAC ATAGTTAGAA TCTACAAAAA TAAAATTATA AATTTTTTAT GTAGGTTATA   
  
  
- TGAAATACAA AACTTAAATA TTAATTAAAA AAATAAACTT TTATACAATT GTAGCATCCA TATAAAAAAA   
  
  
- TATAATTTTT TAATTAACCT AAGTCTCAGT ATTTTTATTA GTCCAAAAGA GATTTTTAGC CATACGTAGT   
  
  
- GAAAGTTTTG TACTTGCGAG TGTAAGAGGG TTGCCTCCTT TTTACGGAGA TCTACAACAG TTTTCTTCTG   
  
  
- CGGAAATATT AATGTTAAAA AATATGGGTA AAAGGTATAG GGTTTTCCGA CTACTAATAC CGTAAAAGCC   
  
  
- TAAAAATTTT TTTTCTTTTT TTTACTATTT ATGTCTCCAC AAATCGTAAC TTTTCTTTTT ATTTAATTTC   
  
  
- CTGGACGGCA GTTATTTTAA ACCGTCGTTT CTTCTTATAT TAACTGGTCA GTTACTCCTG GACTATTGAT   
  
  
- ATTTTTTTCT CCGCGTACCT GCCCGCGTGG GTGTACAAT

+     CCGTCC motif

| Site Name | Organism | Position | Strand | Matrix score. | sequence | function |
| --- | --- | --- | --- | --- | --- | --- |
| CCGTCC motif | Nicotiana tabacum | 1978 | - | 6 | CCGTCC |  |

>Potri.006G138900.1   
+ TGATACCAGA TGATGTGTCG TGAATGATTT GAAAATCGAA AGCAATGAAT CTGACGAAAA TGGATGAAGG   
  
  
+ ATGGGTCTGG TTAGTTGTCT TTCTTTTGGT GTTTAGGCTG GATTATAGTG ATTTTAAAGT AAATAAGATG   
  
  
+ GAGGATAGAC TAGAATGATA CTTTGATAAG TCGATCTGGA CGCCACAAAG ATCAATCTAG GATTTCGACA   
  
  
+ CCTATAAAAT ATTTGGATTA GTGTTTTGTG TTTTTTAAAA AACTTTTTTT TGTTTAGTTT TTTTTTCATT   
  
  
+ TCACCGTTCA ACAACTCAAT CTTTTTCTTC TTTTTATAAT TTTTTTTCTA TTTCATCTTT AAAAATTAGG   
  
  
+ TTGTTTTGAA AATTTTGCTT CATATTTTTT TTTTCTGTTG GGTTATATAT TAGTCTCATG GATTTAATTT   
  
  
+ TATTTTCTCG ATTTCAACCT TCAACACTAG ATCTATTGGA AGTGGGGTTT CATAATATTT TTTTTATTTA   
  
  
+ TTTTCTATGA ATTTTTCTTG ATCTCATGAC TAGGTCACAA GTTTAACAAG TTAACTCAAG TTGACTGAGA   
  
  
+ TCATTTTCTT ATTGCTTTTT TTAATTGATT TTTTTATTTC ATCCTTCAAT ATTGGGTTGG ATGGAAATTG   
  
  
+ AGATTCATAT ATTTTTTCTA TTTACTTTCT ATGGGGTTAT CCTGATCTCA TGTGAGTTTA GCAGGTTAAG   
  
  
+ TCGGGTTGAC TTTATTTATT TATTTTTCTT CTTTTTTAAT TGGACTTTGG AATTTTTTTT ATTTTATTTT   
  
  
+ TATGGGATTA TCCAGATCTT ATGACTCAAG TCACGAGTTT AGTTGGTTGA CCTGAGTTGA CTTGATTTAT   
  
  
+ TATTTGGGTT CCTTTTTTAA TTGAATGTTT TTTTTTCTAT TTCACCTTCC AACAACTCAA TTGTTTTATT   
  
  
+ TCACGTTTTT TTTCTTCAGT TTCATCCTTC AATATTAAGT TGTTTGGGAA TTAGATTTTG AAATTTCTTT   
  
  
+ TTCAATTTGG TTTTTGTGGA GTTAACCCGA TCTAATATAT TTAATTTTTT TTCTTGATTT TAATCTTTTA   
  
  
+ CATTGGATCT GTTAGAAAAT GGATCTTTGT AATTTTTTAT TTGATTTTTT TGAAGTTATC TCAGTCTTAT   
  
  
+ GATCTAGGTC ACGAGCTTAA CATATTAGCC TAAGTTAACT TGAGTTGTTT TTTTTAGTTG ATTTTTTTTA   
  
  
+ TTTCATCATT CAACATTAAG TTAGTTGTAA ATTAGGTTTC ATAATTTTTT TATATATGCT TTCTATTGAG   
  
  
+ TTATAAAATA TCCCGATCTC ATGATCCGGG TTGCGAGTTT AACATCTTAA CTTACATTAA CTTGGGTAGT   
  
  
+ TTTTTTTATC CTTTTTTTAA TTTAATATTT TTTTTTCAAT TCCACCCTTC AATATCAAAT TGATTAGAGA   
  
  
+ TTGATCTTCA TAATTTATTT TGATTTACTT TCTATATATA AGGTTATTAT GGTTTCTTGA TTTAAGTTTC   
  
  
+ AAATTTGACA GATTAACTTG TATCAATCTT AGATGTTTTT ATTTTAATAT TTAAAAAATA CATCCAATAT   
  
  
+ ACTTTATGTT TTGAATTTAT AATTAATTTT TTTATTTGAA AATATGTTAA CATCGTAGGT ATATTTTTTT   
  
  
+ ATATTAAAAA ATTAATTGGA TTCAGAGTCA TAAAAATAAT CAGGTTTTCT CTAAAAATCG GTATGCATCA   
  
  
+ CTTTCAAAAC ATGAACGCTC ACATTCTCCC AACGGAGGAA AAATGCCTCT AGATGTTGTC AAAAGAAGAC   
  
  
+ GCCTTTATAA TTACAATTTT TTATACCCAT TTTCCATATC CCAAAAGGCT GATGATTATG GCATTTTCGG   
  
  
+ ATTTTTAAAA AAAAGAAAAA AAATGATAAA TACAGAGGTG TTTAGCATTG AAAAGAAAAA TAAATTAAAG   
  
  
+ GACCTGCCGT CAATAAAATT TGGCAGCAAA GAAGAATATA ATTGACCAGT CAATGAGGAC CTGATAACTA   
  
  
+ TAAAAAAAGA GGCGCATGGA CGGGCGCACC CACATGTTA  

- ACTATGGTCT ACTACACAGC ACTTACTAAA CTTTTAGCTT TCGTTACTTA GACTGCTTTT ACCTACTTCC   
  
  
- TACCCAGACC AATCAACAGA AAGAAAACCA CAAATCCGAC CTAATATCAC TAAAATTTCA TTTATTCTAC   
  
  
- CTCCTATCTG ATCTTACTAT GAAACTATTC AGCTAGACCT GCGGTGTTTC TAGTTAGATC CTAAAGCTGT   
  
  
- GGATATTTTA TAAACCTAAT CACAAAACAC AAAAAATTTT TTGAAAAAAA ACAAATCAAA AAAAAAGTAA   
  
  
- AGTGGCAAGT TGTTGAGTTA GAAAAAGAAG AAAAATATTA AAAAAAAGAT AAAGTAGAAA TTTTTAATCC   
  
  
- AACAAAACTT TTAAAACGAA GTATAAAAAA AAAAGACAAC CCAATATATA ATCAGAGTAC CTAAATTAAA   
  
  
- ATAAAAGAGC TAAAGTTGGA AGTTGTGATC TAGATAACCT TCACCCCAAA GTATTATAAA AAAAATAAAT   
  
  
- AAAAGATACT TAAAAAGAAC TAGAGTACTG ATCCAGTGTT CAAATTGTTC AATTGAGTTC AACTGACTCT   
  
  
- AGTAAAAGAA TAACGAAAAA AATTAACTAA AAAAATAAAG TAGGAAGTTA TAACCCAACC TACCTTTAAC   
  
  
- TCTAAGTATA TAAAAAAGAT AAATGAAAGA TACCCCAATA GGACTAGAGT ACACTCAAAT CGTCCAATTC   
  
  
- AGCCCAACTG AAATAAATAA ATAAAAAGAA GAAAAAATTA ACCTGAAACC TTAAAAAAAA TAAAATAAAA   
  
  
- ATACCCTAAT AGGTCTAGAA TACTGAGTTC AGTGCTCAAA TCAACCAACT GGACTCAACT GAACTAAATA   
  
  
- ATAAACCCAA GGAAAAAATT AACTTACAAA AAAAAAGATA AAGTGGAAGG TTGTTGAGTT AACAAAATAA   
  
  
- AGTGCAAAAA AAAGAAGTCA AAGTAGGAAG TTATAATTCA ACAAACCCTT AATCTAAAAC TTTAAAGAAA   
  
  
- AAGTTAAACC AAAAACACCT CAATTGGGCT AGATTATATA AATTAAAAAA AAGAACTAAA ATTAGAAAAT   
  
  
- GTAACCTAGA CAATCTTTTA CCTAGAAACA TTAAAAAATA AACTAAAAAA ACTTCAATAG AGTCAGAATA   
  
  
- CTAGATCCAG TGCTCGAATT GTATAATCGG ATTCAATTGA ACTCAACAAA AAAAATCAAC TAAAAAAAAT   
  
  
- AAAGTAGTAA GTTGTAATTC AATCAACATT TAATCCAAAG TATTAAAAAA ATATATACGA AAGATAACTC   
  
  
- AATATTTTAT AGGGCTAGAG TACTAGGCCC AACGCTCAAA TTGTAGAATT GAATGTAATT GAACCCATCA   
  
  
- AAAAAAATAG GAAAAAAATT AAATTATAAA AAAAAAGTTA AGGTGGGAAG TTATAGTTTA ACTAATCTCT   
  
  
- AACTAGAAGT ATTAAATAAA ACTAAATGAA AGATATATAT TCCAATAATA CCAAAGAACT AAATTCAAAG   
  
  
- TTTAAACTGT CTAATTGAAC ATAGTTAGAA TCTACAAAAA TAAAATTATA AATTTTTTAT GTAGGTTATA   
  
  
- TGAAATACAA AACTTAAATA TTAATTAAAA AAATAAACTT TTATACAATT GTAGCATCCA TATAAAAAAA   
  
  
- TATAATTTTT TAATTAACCT AAGTCTCAGT ATTTTTATTA GTCCAAAAGA GATTTTTAGC CATACGTAGT   
  
  
- GAAAGTTTTG TACTTGCGAG TGTAAGAGGG TTGCCTCCTT TTTACGGAGA TCTACAACAG TTTTCTTCTG   
  
  
- CGGAAATATT AATGTTAAAA AATATGGGTA AAAGGTATAG GGTTTTCCGA CTACTAATAC CGTAAAAGCC   
  
  
- TAAAAATTTT TTTTCTTTTT TTTACTATTT ATGTCTCCAC AAATCGTAAC TTTTCTTTTT ATTTAATTTC   
  
  
- CTGGACGGCA GTTATTTTAA ACCGTCGTTT CTTCTTATAT TAACTGGTCA GTTACTCCTG GACTATTGAT   
  
  
- ATTTTTTTCT CCGCGTACCT GCCCGCGTGG GTGTACAAT

+     CCGTCC-box

| Site Name | Organism | Position | Strand | Matrix score. | sequence | function |
| --- | --- | --- | --- | --- | --- | --- |
| CCGTCC-box | Petroselinum hortense | 1978 | - | 6 | CCGTCC |  |

>Potri.006G138900.1   
+ TGATACCAGA TGATGTGTCG TGAATGATTT GAAAATCGAA AGCAATGAAT CTGACGAAAA TGGATGAAGG   
  
  
+ ATGGGTCTGG TTAGTTGTCT TTCTTTTGGT GTTTAGGCTG GATTATAGTG ATTTTAAAGT AAATAAGATG   
  
  
+ GAGGATAGAC TAGAATGATA CTTTGATAAG TCGATCTGGA CGCCACAAAG ATCAATCTAG GATTTCGACA   
  
  
+ CCTATAAAAT ATTTGGATTA GTGTTTTGTG TTTTTTAAAA AACTTTTTTT TGTTTAGTTT TTTTTTCATT   
  
  
+ TCACCGTTCA ACAACTCAAT CTTTTTCTTC TTTTTATAAT TTTTTTTCTA TTTCATCTTT AAAAATTAGG   
  
  
+ TTGTTTTGAA AATTTTGCTT CATATTTTTT TTTTCTGTTG GGTTATATAT TAGTCTCATG GATTTAATTT   
  
  
+ TATTTTCTCG ATTTCAACCT TCAACACTAG ATCTATTGGA AGTGGGGTTT CATAATATTT TTTTTATTTA   
  
  
+ TTTTCTATGA ATTTTTCTTG ATCTCATGAC TAGGTCACAA GTTTAACAAG TTAACTCAAG TTGACTGAGA   
  
  
+ TCATTTTCTT ATTGCTTTTT TTAATTGATT TTTTTATTTC ATCCTTCAAT ATTGGGTTGG ATGGAAATTG   
  
  
+ AGATTCATAT ATTTTTTCTA TTTACTTTCT ATGGGGTTAT CCTGATCTCA TGTGAGTTTA GCAGGTTAAG   
  
  
+ TCGGGTTGAC TTTATTTATT TATTTTTCTT CTTTTTTAAT TGGACTTTGG AATTTTTTTT ATTTTATTTT   
  
  
+ TATGGGATTA TCCAGATCTT ATGACTCAAG TCACGAGTTT AGTTGGTTGA CCTGAGTTGA CTTGATTTAT   
  
  
+ TATTTGGGTT CCTTTTTTAA TTGAATGTTT TTTTTTCTAT TTCACCTTCC AACAACTCAA TTGTTTTATT   
  
  
+ TCACGTTTTT TTTCTTCAGT TTCATCCTTC AATATTAAGT TGTTTGGGAA TTAGATTTTG AAATTTCTTT   
  
  
+ TTCAATTTGG TTTTTGTGGA GTTAACCCGA TCTAATATAT TTAATTTTTT TTCTTGATTT TAATCTTTTA   
  
  
+ CATTGGATCT GTTAGAAAAT GGATCTTTGT AATTTTTTAT TTGATTTTTT TGAAGTTATC TCAGTCTTAT   
  
  
+ GATCTAGGTC ACGAGCTTAA CATATTAGCC TAAGTTAACT TGAGTTGTTT TTTTTAGTTG ATTTTTTTTA   
  
  
+ TTTCATCATT CAACATTAAG TTAGTTGTAA ATTAGGTTTC ATAATTTTTT TATATATGCT TTCTATTGAG   
  
  
+ TTATAAAATA TCCCGATCTC ATGATCCGGG TTGCGAGTTT AACATCTTAA CTTACATTAA CTTGGGTAGT   
  
  
+ TTTTTTTATC CTTTTTTTAA TTTAATATTT TTTTTTCAAT TCCACCCTTC AATATCAAAT TGATTAGAGA   
  
  
+ TTGATCTTCA TAATTTATTT TGATTTACTT TCTATATATA AGGTTATTAT GGTTTCTTGA TTTAAGTTTC   
  
  
+ AAATTTGACA GATTAACTTG TATCAATCTT AGATGTTTTT ATTTTAATAT TTAAAAAATA CATCCAATAT   
  
  
+ ACTTTATGTT TTGAATTTAT AATTAATTTT TTTATTTGAA AATATGTTAA CATCGTAGGT ATATTTTTTT   
  
  
+ ATATTAAAAA ATTAATTGGA TTCAGAGTCA TAAAAATAAT CAGGTTTTCT CTAAAAATCG GTATGCATCA   
  
  
+ CTTTCAAAAC ATGAACGCTC ACATTCTCCC AACGGAGGAA AAATGCCTCT AGATGTTGTC AAAAGAAGAC   
  
  
+ GCCTTTATAA TTACAATTTT TTATACCCAT TTTCCATATC CCAAAAGGCT GATGATTATG GCATTTTCGG   
  
  
+ ATTTTTAAAA AAAAGAAAAA AAATGATAAA TACAGAGGTG TTTAGCATTG AAAAGAAAAA TAAATTAAAG   
  
  
+ GACCTGCCGT CAATAAAATT TGGCAGCAAA GAAGAATATA ATTGACCAGT CAATGAGGAC CTGATAACTA   
  
  
+ TAAAAAAAGA GGCGCATGGA CGGGCGCACC CACATGTTA  

- ACTATGGTCT ACTACACAGC ACTTACTAAA CTTTTAGCTT TCGTTACTTA GACTGCTTTT ACCTACTTCC   
  
  
- TACCCAGACC AATCAACAGA AAGAAAACCA CAAATCCGAC CTAATATCAC TAAAATTTCA TTTATTCTAC   
  
  
- CTCCTATCTG ATCTTACTAT GAAACTATTC AGCTAGACCT GCGGTGTTTC TAGTTAGATC CTAAAGCTGT   
  
  
- GGATATTTTA TAAACCTAAT CACAAAACAC AAAAAATTTT TTGAAAAAAA ACAAATCAAA AAAAAAGTAA   
  
  
- AGTGGCAAGT TGTTGAGTTA GAAAAAGAAG AAAAATATTA AAAAAAAGAT AAAGTAGAAA TTTTTAATCC   
  
  
- AACAAAACTT TTAAAACGAA GTATAAAAAA AAAAGACAAC CCAATATATA ATCAGAGTAC CTAAATTAAA   
  
  
- ATAAAAGAGC TAAAGTTGGA AGTTGTGATC TAGATAACCT TCACCCCAAA GTATTATAAA AAAAATAAAT   
  
  
- AAAAGATACT TAAAAAGAAC TAGAGTACTG ATCCAGTGTT CAAATTGTTC AATTGAGTTC AACTGACTCT   
  
  
- AGTAAAAGAA TAACGAAAAA AATTAACTAA AAAAATAAAG TAGGAAGTTA TAACCCAACC TACCTTTAAC   
  
  
- TCTAAGTATA TAAAAAAGAT AAATGAAAGA TACCCCAATA GGACTAGAGT ACACTCAAAT CGTCCAATTC   
  
  
- AGCCCAACTG AAATAAATAA ATAAAAAGAA GAAAAAATTA ACCTGAAACC TTAAAAAAAA TAAAATAAAA   
  
  
- ATACCCTAAT AGGTCTAGAA TACTGAGTTC AGTGCTCAAA TCAACCAACT GGACTCAACT GAACTAAATA   
  
  
- ATAAACCCAA GGAAAAAATT AACTTACAAA AAAAAAGATA AAGTGGAAGG TTGTTGAGTT AACAAAATAA   
  
  
- AGTGCAAAAA AAAGAAGTCA AAGTAGGAAG TTATAATTCA ACAAACCCTT AATCTAAAAC TTTAAAGAAA   
  
  
- AAGTTAAACC AAAAACACCT CAATTGGGCT AGATTATATA AATTAAAAAA AAGAACTAAA ATTAGAAAAT   
  
  
- GTAACCTAGA CAATCTTTTA CCTAGAAACA TTAAAAAATA AACTAAAAAA ACTTCAATAG AGTCAGAATA   
  
  
- CTAGATCCAG TGCTCGAATT GTATAATCGG ATTCAATTGA ACTCAACAAA AAAAATCAAC TAAAAAAAAT   
  
  
- AAAGTAGTAA GTTGTAATTC AATCAACATT TAATCCAAAG TATTAAAAAA ATATATACGA AAGATAACTC   
  
  
- AATATTTTAT AGGGCTAGAG TACTAGGCCC AACGCTCAAA TTGTAGAATT GAATGTAATT GAACCCATCA   
  
  
- AAAAAAATAG GAAAAAAATT AAATTATAAA AAAAAAGTTA AGGTGGGAAG TTATAGTTTA ACTAATCTCT   
  
  
- AACTAGAAGT ATTAAATAAA ACTAAATGAA AGATATATAT TCCAATAATA CCAAAGAACT AAATTCAAAG   
  
  
- TTTAAACTGT CTAATTGAAC ATAGTTAGAA TCTACAAAAA TAAAATTATA AATTTTTTAT GTAGGTTATA   
  
  
- TGAAATACAA AACTTAAATA TTAATTAAAA AAATAAACTT TTATACAATT GTAGCATCCA TATAAAAAAA   
  
  
- TATAATTTTT TAATTAACCT AAGTCTCAGT ATTTTTATTA GTCCAAAAGA GATTTTTAGC CATACGTAGT   
  
  
- GAAAGTTTTG TACTTGCGAG TGTAAGAGGG TTGCCTCCTT TTTACGGAGA TCTACAACAG TTTTCTTCTG   
  
  
- CGGAAATATT AATGTTAAAA AATATGGGTA AAAGGTATAG GGTTTTCCGA CTACTAATAC CGTAAAAGCC   
  
  
- TAAAAATTTT TTTTCTTTTT TTTACTATTT ATGTCTCCAC AAATCGTAAC TTTTCTTTTT ATTTAATTTC   
  
  
- CTGGACGGCA GTTATTTTAA ACCGTCGTTT CTTCTTATAT TAACTGGTCA GTTACTCCTG GACTATTGAT   
  
  
- ATTTTTTTCT CCGCGTACCT GCCCGCGTGG GTGTACAAT

+     CGTCA-motif

| Site Name | Organism | Position | Strand | Matrix score. | sequence | function |
| --- | --- | --- | --- | --- | --- | --- |
| CGTCA-motif | Hordeum vulgare | 1898 | + | 5 | CGTCA | cis-acting regulatory element involved in the MeJA-responsiveness |
| CGTCA-motif | Hordeum vulgare | 52 | - | 5 | CGTCA | cis-acting regulatory element involved in the MeJA-responsiveness |

>Potri.006G138900.1   
+ TGATACCAGA TGATGTGTCG TGAATGATTT GAAAATCGAA AGCAATGAAT CTGACGAAAA TGGATGAAGG   
  
  
+ ATGGGTCTGG TTAGTTGTCT TTCTTTTGGT GTTTAGGCTG GATTATAGTG ATTTTAAAGT AAATAAGATG   
  
  
+ GAGGATAGAC TAGAATGATA CTTTGATAAG TCGATCTGGA CGCCACAAAG ATCAATCTAG GATTTCGACA   
  
  
+ CCTATAAAAT ATTTGGATTA GTGTTTTGTG TTTTTTAAAA AACTTTTTTT TGTTTAGTTT TTTTTTCATT   
  
  
+ TCACCGTTCA ACAACTCAAT CTTTTTCTTC TTTTTATAAT TTTTTTTCTA TTTCATCTTT AAAAATTAGG   
  
  
+ TTGTTTTGAA AATTTTGCTT CATATTTTTT TTTTCTGTTG GGTTATATAT TAGTCTCATG GATTTAATTT   
  
  
+ TATTTTCTCG ATTTCAACCT TCAACACTAG ATCTATTGGA AGTGGGGTTT CATAATATTT TTTTTATTTA   
  
  
+ TTTTCTATGA ATTTTTCTTG ATCTCATGAC TAGGTCACAA GTTTAACAAG TTAACTCAAG TTGACTGAGA   
  
  
+ TCATTTTCTT ATTGCTTTTT TTAATTGATT TTTTTATTTC ATCCTTCAAT ATTGGGTTGG ATGGAAATTG   
  
  
+ AGATTCATAT ATTTTTTCTA TTTACTTTCT ATGGGGTTAT CCTGATCTCA TGTGAGTTTA GCAGGTTAAG   
  
  
+ TCGGGTTGAC TTTATTTATT TATTTTTCTT CTTTTTTAAT TGGACTTTGG AATTTTTTTT ATTTTATTTT   
  
  
+ TATGGGATTA TCCAGATCTT ATGACTCAAG TCACGAGTTT AGTTGGTTGA CCTGAGTTGA CTTGATTTAT   
  
  
+ TATTTGGGTT CCTTTTTTAA TTGAATGTTT TTTTTTCTAT TTCACCTTCC AACAACTCAA TTGTTTTATT   
  
  
+ TCACGTTTTT TTTCTTCAGT TTCATCCTTC AATATTAAGT TGTTTGGGAA TTAGATTTTG AAATTTCTTT   
  
  
+ TTCAATTTGG TTTTTGTGGA GTTAACCCGA TCTAATATAT TTAATTTTTT TTCTTGATTT TAATCTTTTA   
  
  
+ CATTGGATCT GTTAGAAAAT GGATCTTTGT AATTTTTTAT TTGATTTTTT TGAAGTTATC TCAGTCTTAT   
  
  
+ GATCTAGGTC ACGAGCTTAA CATATTAGCC TAAGTTAACT TGAGTTGTTT TTTTTAGTTG ATTTTTTTTA   
  
  
+ TTTCATCATT CAACATTAAG TTAGTTGTAA ATTAGGTTTC ATAATTTTTT TATATATGCT TTCTATTGAG   
  
  
+ TTATAAAATA TCCCGATCTC ATGATCCGGG TTGCGAGTTT AACATCTTAA CTTACATTAA CTTGGGTAGT   
  
  
+ TTTTTTTATC CTTTTTTTAA TTTAATATTT TTTTTTCAAT TCCACCCTTC AATATCAAAT TGATTAGAGA   
  
  
+ TTGATCTTCA TAATTTATTT TGATTTACTT TCTATATATA AGGTTATTAT GGTTTCTTGA TTTAAGTTTC   
  
  
+ AAATTTGACA GATTAACTTG TATCAATCTT AGATGTTTTT ATTTTAATAT TTAAAAAATA CATCCAATAT   
  
  
+ ACTTTATGTT TTGAATTTAT AATTAATTTT TTTATTTGAA AATATGTTAA CATCGTAGGT ATATTTTTTT   
  
  
+ ATATTAAAAA ATTAATTGGA TTCAGAGTCA TAAAAATAAT CAGGTTTTCT CTAAAAATCG GTATGCATCA   
  
  
+ CTTTCAAAAC ATGAACGCTC ACATTCTCCC AACGGAGGAA AAATGCCTCT AGATGTTGTC AAAAGAAGAC   
  
  
+ GCCTTTATAA TTACAATTTT TTATACCCAT TTTCCATATC CCAAAAGGCT GATGATTATG GCATTTTCGG   
  
  
+ ATTTTTAAAA AAAAGAAAAA AAATGATAAA TACAGAGGTG TTTAGCATTG AAAAGAAAAA TAAATTAAAG   
  
  
+ GACCTGCCGT CAATAAAATT TGGCAGCAAA GAAGAATATA ATTGACCAGT CAATGAGGAC CTGATAACTA   
  
  
+ TAAAAAAAGA GGCGCATGGA CGGGCGCACC CACATGTTA  

- ACTATGGTCT ACTACACAGC ACTTACTAAA CTTTTAGCTT TCGTTACTTA GACTGCTTTT ACCTACTTCC   
  
  
- TACCCAGACC AATCAACAGA AAGAAAACCA CAAATCCGAC CTAATATCAC TAAAATTTCA TTTATTCTAC   
  
  
- CTCCTATCTG ATCTTACTAT GAAACTATTC AGCTAGACCT GCGGTGTTTC TAGTTAGATC CTAAAGCTGT   
  
  
- GGATATTTTA TAAACCTAAT CACAAAACAC AAAAAATTTT TTGAAAAAAA ACAAATCAAA AAAAAAGTAA   
  
  
- AGTGGCAAGT TGTTGAGTTA GAAAAAGAAG AAAAATATTA AAAAAAAGAT AAAGTAGAAA TTTTTAATCC   
  
  
- AACAAAACTT TTAAAACGAA GTATAAAAAA AAAAGACAAC CCAATATATA ATCAGAGTAC CTAAATTAAA   
  
  
- ATAAAAGAGC TAAAGTTGGA AGTTGTGATC TAGATAACCT TCACCCCAAA GTATTATAAA AAAAATAAAT   
  
  
- AAAAGATACT TAAAAAGAAC TAGAGTACTG ATCCAGTGTT CAAATTGTTC AATTGAGTTC AACTGACTCT   
  
  
- AGTAAAAGAA TAACGAAAAA AATTAACTAA AAAAATAAAG TAGGAAGTTA TAACCCAACC TACCTTTAAC   
  
  
- TCTAAGTATA TAAAAAAGAT AAATGAAAGA TACCCCAATA GGACTAGAGT ACACTCAAAT CGTCCAATTC   
  
  
- AGCCCAACTG AAATAAATAA ATAAAAAGAA GAAAAAATTA ACCTGAAACC TTAAAAAAAA TAAAATAAAA   
  
  
- ATACCCTAAT AGGTCTAGAA TACTGAGTTC AGTGCTCAAA TCAACCAACT GGACTCAACT GAACTAAATA   
  
  
- ATAAACCCAA GGAAAAAATT AACTTACAAA AAAAAAGATA AAGTGGAAGG TTGTTGAGTT AACAAAATAA   
  
  
- AGTGCAAAAA AAAGAAGTCA AAGTAGGAAG TTATAATTCA ACAAACCCTT AATCTAAAAC TTTAAAGAAA   
  
  
- AAGTTAAACC AAAAACACCT CAATTGGGCT AGATTATATA AATTAAAAAA AAGAACTAAA ATTAGAAAAT   
  
  
- GTAACCTAGA CAATCTTTTA CCTAGAAACA TTAAAAAATA AACTAAAAAA ACTTCAATAG AGTCAGAATA   
  
  
- CTAGATCCAG TGCTCGAATT GTATAATCGG ATTCAATTGA ACTCAACAAA AAAAATCAAC TAAAAAAAAT   
  
  
- AAAGTAGTAA GTTGTAATTC AATCAACATT TAATCCAAAG TATTAAAAAA ATATATACGA AAGATAACTC   
  
  
- AATATTTTAT AGGGCTAGAG TACTAGGCCC AACGCTCAAA TTGTAGAATT GAATGTAATT GAACCCATCA   
  
  
- AAAAAAATAG GAAAAAAATT AAATTATAAA AAAAAAGTTA AGGTGGGAAG TTATAGTTTA ACTAATCTCT   
  
  
- AACTAGAAGT ATTAAATAAA ACTAAATGAA AGATATATAT TCCAATAATA CCAAAGAACT AAATTCAAAG   
  
  
- TTTAAACTGT CTAATTGAAC ATAGTTAGAA TCTACAAAAA TAAAATTATA AATTTTTTAT GTAGGTTATA   
  
  
- TGAAATACAA AACTTAAATA TTAATTAAAA AAATAAACTT TTATACAATT GTAGCATCCA TATAAAAAAA   
  
  
- TATAATTTTT TAATTAACCT AAGTCTCAGT ATTTTTATTA GTCCAAAAGA GATTTTTAGC CATACGTAGT   
  
  
- GAAAGTTTTG TACTTGCGAG TGTAAGAGGG TTGCCTCCTT TTTACGGAGA TCTACAACAG TTTTCTTCTG   
  
  
- CGGAAATATT AATGTTAAAA AATATGGGTA AAAGGTATAG GGTTTTCCGA CTACTAATAC CGTAAAAGCC   
  
  
- TAAAAATTTT TTTTCTTTTT TTTACTATTT ATGTCTCCAC AAATCGTAAC TTTTCTTTTT ATTTAATTTC   
  
  
- CTGGACGGCA GTTATTTTAA ACCGTCGTTT CTTCTTATAT TAACTGGTCA GTTACTCCTG GACTATTGAT   
  
  
- ATTTTTTTCT CCGCGTACCT GCCCGCGTGG GTGTACAAT

+     ERE

| Site Name | Organism | Position | Strand | Matrix score. | sequence | function |
| --- | --- | --- | --- | --- | --- | --- |
| ERE | Nicotiana glutinos | 121 | + | 8 | ATTTTAAA |  |

>Potri.006G138900.1   
+ TGATACCAGA TGATGTGTCG TGAATGATTT GAAAATCGAA AGCAATGAAT CTGACGAAAA TGGATGAAGG   
  
  
+ ATGGGTCTGG TTAGTTGTCT TTCTTTTGGT GTTTAGGCTG GATTATAGTG ATTTTAAAGT AAATAAGATG   
  
  
+ GAGGATAGAC TAGAATGATA CTTTGATAAG TCGATCTGGA CGCCACAAAG ATCAATCTAG GATTTCGACA   
  
  
+ CCTATAAAAT ATTTGGATTA GTGTTTTGTG TTTTTTAAAA AACTTTTTTT TGTTTAGTTT TTTTTTCATT   
  
  
+ TCACCGTTCA ACAACTCAAT CTTTTTCTTC TTTTTATAAT TTTTTTTCTA TTTCATCTTT AAAAATTAGG   
  
  
+ TTGTTTTGAA AATTTTGCTT CATATTTTTT TTTTCTGTTG GGTTATATAT TAGTCTCATG GATTTAATTT   
  
  
+ TATTTTCTCG ATTTCAACCT TCAACACTAG ATCTATTGGA AGTGGGGTTT CATAATATTT TTTTTATTTA   
  
  
+ TTTTCTATGA ATTTTTCTTG ATCTCATGAC TAGGTCACAA GTTTAACAAG TTAACTCAAG TTGACTGAGA   
  
  
+ TCATTTTCTT ATTGCTTTTT TTAATTGATT TTTTTATTTC ATCCTTCAAT ATTGGGTTGG ATGGAAATTG   
  
  
+ AGATTCATAT ATTTTTTCTA TTTACTTTCT ATGGGGTTAT CCTGATCTCA TGTGAGTTTA GCAGGTTAAG   
  
  
+ TCGGGTTGAC TTTATTTATT TATTTTTCTT CTTTTTTAAT TGGACTTTGG AATTTTTTTT ATTTTATTTT   
  
  
+ TATGGGATTA TCCAGATCTT ATGACTCAAG TCACGAGTTT AGTTGGTTGA CCTGAGTTGA CTTGATTTAT   
  
  
+ TATTTGGGTT CCTTTTTTAA TTGAATGTTT TTTTTTCTAT TTCACCTTCC AACAACTCAA TTGTTTTATT   
  
  
+ TCACGTTTTT TTTCTTCAGT TTCATCCTTC AATATTAAGT TGTTTGGGAA TTAGATTTTG AAATTTCTTT   
  
  
+ TTCAATTTGG TTTTTGTGGA GTTAACCCGA TCTAATATAT TTAATTTTTT TTCTTGATTT TAATCTTTTA   
  
  
+ CATTGGATCT GTTAGAAAAT GGATCTTTGT AATTTTTTAT TTGATTTTTT TGAAGTTATC TCAGTCTTAT   
  
  
+ GATCTAGGTC ACGAGCTTAA CATATTAGCC TAAGTTAACT TGAGTTGTTT TTTTTAGTTG ATTTTTTTTA   
  
  
+ TTTCATCATT CAACATTAAG TTAGTTGTAA ATTAGGTTTC ATAATTTTTT TATATATGCT TTCTATTGAG   
  
  
+ TTATAAAATA TCCCGATCTC ATGATCCGGG TTGCGAGTTT AACATCTTAA CTTACATTAA CTTGGGTAGT   
  
  
+ TTTTTTTATC CTTTTTTTAA TTTAATATTT TTTTTTCAAT TCCACCCTTC AATATCAAAT TGATTAGAGA   
  
  
+ TTGATCTTCA TAATTTATTT TGATTTACTT TCTATATATA AGGTTATTAT GGTTTCTTGA TTTAAGTTTC   
  
  
+ AAATTTGACA GATTAACTTG TATCAATCTT AGATGTTTTT ATTTTAATAT TTAAAAAATA CATCCAATAT   
  
  
+ ACTTTATGTT TTGAATTTAT AATTAATTTT TTTATTTGAA AATATGTTAA CATCGTAGGT ATATTTTTTT   
  
  
+ ATATTAAAAA ATTAATTGGA TTCAGAGTCA TAAAAATAAT CAGGTTTTCT CTAAAAATCG GTATGCATCA   
  
  
+ CTTTCAAAAC ATGAACGCTC ACATTCTCCC AACGGAGGAA AAATGCCTCT AGATGTTGTC AAAAGAAGAC   
  
  
+ GCCTTTATAA TTACAATTTT TTATACCCAT TTTCCATATC CCAAAAGGCT GATGATTATG GCATTTTCGG   
  
  
+ ATTTTTAAAA AAAAGAAAAA AAATGATAAA TACAGAGGTG TTTAGCATTG AAAAGAAAAA TAAATTAAAG   
  
  
+ GACCTGCCGT CAATAAAATT TGGCAGCAAA GAAGAATATA ATTGACCAGT CAATGAGGAC CTGATAACTA   
  
  
+ TAAAAAAAGA GGCGCATGGA CGGGCGCACC CACATGTTA  

- ACTATGGTCT ACTACACAGC ACTTACTAAA CTTTTAGCTT TCGTTACTTA GACTGCTTTT ACCTACTTCC   
  
  
- TACCCAGACC AATCAACAGA AAGAAAACCA CAAATCCGAC CTAATATCAC TAAAATTTCA TTTATTCTAC   
  
  
- CTCCTATCTG ATCTTACTAT GAAACTATTC AGCTAGACCT GCGGTGTTTC TAGTTAGATC CTAAAGCTGT   
  
  
- GGATATTTTA TAAACCTAAT CACAAAACAC AAAAAATTTT TTGAAAAAAA ACAAATCAAA AAAAAAGTAA   
  
  
- AGTGGCAAGT TGTTGAGTTA GAAAAAGAAG AAAAATATTA AAAAAAAGAT AAAGTAGAAA TTTTTAATCC   
  
  
- AACAAAACTT TTAAAACGAA GTATAAAAAA AAAAGACAAC CCAATATATA ATCAGAGTAC CTAAATTAAA   
  
  
- ATAAAAGAGC TAAAGTTGGA AGTTGTGATC TAGATAACCT TCACCCCAAA GTATTATAAA AAAAATAAAT   
  
  
- AAAAGATACT TAAAAAGAAC TAGAGTACTG ATCCAGTGTT CAAATTGTTC AATTGAGTTC AACTGACTCT   
  
  
- AGTAAAAGAA TAACGAAAAA AATTAACTAA AAAAATAAAG TAGGAAGTTA TAACCCAACC TACCTTTAAC   
  
  
- TCTAAGTATA TAAAAAAGAT AAATGAAAGA TACCCCAATA GGACTAGAGT ACACTCAAAT CGTCCAATTC   
  
  
- AGCCCAACTG AAATAAATAA ATAAAAAGAA GAAAAAATTA ACCTGAAACC TTAAAAAAAA TAAAATAAAA   
  
  
- ATACCCTAAT AGGTCTAGAA TACTGAGTTC AGTGCTCAAA TCAACCAACT GGACTCAACT GAACTAAATA   
  
  
- ATAAACCCAA GGAAAAAATT AACTTACAAA AAAAAAGATA AAGTGGAAGG TTGTTGAGTT AACAAAATAA   
  
  
- AGTGCAAAAA AAAGAAGTCA AAGTAGGAAG TTATAATTCA ACAAACCCTT AATCTAAAAC TTTAAAGAAA   
  
  
- AAGTTAAACC AAAAACACCT CAATTGGGCT AGATTATATA AATTAAAAAA AAGAACTAAA ATTAGAAAAT   
  
  
- GTAACCTAGA CAATCTTTTA CCTAGAAACA TTAAAAAATA AACTAAAAAA ACTTCAATAG AGTCAGAATA   
  
  
- CTAGATCCAG TGCTCGAATT GTATAATCGG ATTCAATTGA ACTCAACAAA AAAAATCAAC TAAAAAAAAT   
  
  
- AAAGTAGTAA GTTGTAATTC AATCAACATT TAATCCAAAG TATTAAAAAA ATATATACGA AAGATAACTC   
  
  
- AATATTTTAT AGGGCTAGAG TACTAGGCCC AACGCTCAAA TTGTAGAATT GAATGTAATT GAACCCATCA   
  
  
- AAAAAAATAG GAAAAAAATT AAATTATAAA AAAAAAGTTA AGGTGGGAAG TTATAGTTTA ACTAATCTCT   
  
  
- AACTAGAAGT ATTAAATAAA ACTAAATGAA AGATATATAT TCCAATAATA CCAAAGAACT AAATTCAAAG   
  
  
- TTTAAACTGT CTAATTGAAC ATAGTTAGAA TCTACAAAAA TAAAATTATA AATTTTTTAT GTAGGTTATA   
  
  
- TGAAATACAA AACTTAAATA TTAATTAAAA AAATAAACTT TTATACAATT GTAGCATCCA TATAAAAAAA   
  
  
- TATAATTTTT TAATTAACCT AAGTCTCAGT ATTTTTATTA GTCCAAAAGA GATTTTTAGC CATACGTAGT   
  
  
- GAAAGTTTTG TACTTGCGAG TGTAAGAGGG TTGCCTCCTT TTTACGGAGA TCTACAACAG TTTTCTTCTG   
  
  
- CGGAAATATT AATGTTAAAA AATATGGGTA AAAGGTATAG GGTTTTCCGA CTACTAATAC CGTAAAAGCC   
  
  
- TAAAAATTTT TTTTCTTTTT TTTACTATTT ATGTCTCCAC AAATCGTAAC TTTTCTTTTT ATTTAATTTC   
  
  
- CTGGACGGCA GTTATTTTAA ACCGTCGTTT CTTCTTATAT TAACTGGTCA GTTACTCCTG GACTATTGAT   
  
  
- ATTTTTTTCT CCGCGTACCT GCCCGCGTGG GTGTACAAT

+     G-Box

| Site Name | Organism | Position | Strand | Matrix score. | sequence | function |
| --- | --- | --- | --- | --- | --- | --- |
| G-Box | Pisum sativum | 912 | + | 6 | CACGTT | cis-acting regulatory element involved in light responsiveness |

>Potri.006G138900.1   
+ TGATACCAGA TGATGTGTCG TGAATGATTT GAAAATCGAA AGCAATGAAT CTGACGAAAA TGGATGAAGG   
  
  
+ ATGGGTCTGG TTAGTTGTCT TTCTTTTGGT GTTTAGGCTG GATTATAGTG ATTTTAAAGT AAATAAGATG   
  
  
+ GAGGATAGAC TAGAATGATA CTTTGATAAG TCGATCTGGA CGCCACAAAG ATCAATCTAG GATTTCGACA   
  
  
+ CCTATAAAAT ATTTGGATTA GTGTTTTGTG TTTTTTAAAA AACTTTTTTT TGTTTAGTTT TTTTTTCATT   
  
  
+ TCACCGTTCA ACAACTCAAT CTTTTTCTTC TTTTTATAAT TTTTTTTCTA TTTCATCTTT AAAAATTAGG   
  
  
+ TTGTTTTGAA AATTTTGCTT CATATTTTTT TTTTCTGTTG GGTTATATAT TAGTCTCATG GATTTAATTT   
  
  
+ TATTTTCTCG ATTTCAACCT TCAACACTAG ATCTATTGGA AGTGGGGTTT CATAATATTT TTTTTATTTA   
  
  
+ TTTTCTATGA ATTTTTCTTG ATCTCATGAC TAGGTCACAA GTTTAACAAG TTAACTCAAG TTGACTGAGA   
  
  
+ TCATTTTCTT ATTGCTTTTT TTAATTGATT TTTTTATTTC ATCCTTCAAT ATTGGGTTGG ATGGAAATTG   
  
  
+ AGATTCATAT ATTTTTTCTA TTTACTTTCT ATGGGGTTAT CCTGATCTCA TGTGAGTTTA GCAGGTTAAG   
  
  
+ TCGGGTTGAC TTTATTTATT TATTTTTCTT CTTTTTTAAT TGGACTTTGG AATTTTTTTT ATTTTATTTT   
  
  
+ TATGGGATTA TCCAGATCTT ATGACTCAAG TCACGAGTTT AGTTGGTTGA CCTGAGTTGA CTTGATTTAT   
  
  
+ TATTTGGGTT CCTTTTTTAA TTGAATGTTT TTTTTTCTAT TTCACCTTCC AACAACTCAA TTGTTTTATT   
  
  
+ TCACGTTTTT TTTCTTCAGT TTCATCCTTC AATATTAAGT TGTTTGGGAA TTAGATTTTG AAATTTCTTT   
  
  
+ TTCAATTTGG TTTTTGTGGA GTTAACCCGA TCTAATATAT TTAATTTTTT TTCTTGATTT TAATCTTTTA   
  
  
+ CATTGGATCT GTTAGAAAAT GGATCTTTGT AATTTTTTAT TTGATTTTTT TGAAGTTATC TCAGTCTTAT   
  
  
+ GATCTAGGTC ACGAGCTTAA CATATTAGCC TAAGTTAACT TGAGTTGTTT TTTTTAGTTG ATTTTTTTTA   
  
  
+ TTTCATCATT CAACATTAAG TTAGTTGTAA ATTAGGTTTC ATAATTTTTT TATATATGCT TTCTATTGAG   
  
  
+ TTATAAAATA TCCCGATCTC ATGATCCGGG TTGCGAGTTT AACATCTTAA CTTACATTAA CTTGGGTAGT   
  
  
+ TTTTTTTATC CTTTTTTTAA TTTAATATTT TTTTTTCAAT TCCACCCTTC AATATCAAAT TGATTAGAGA   
  
  
+ TTGATCTTCA TAATTTATTT TGATTTACTT TCTATATATA AGGTTATTAT GGTTTCTTGA TTTAAGTTTC   
  
  
+ AAATTTGACA GATTAACTTG TATCAATCTT AGATGTTTTT ATTTTAATAT TTAAAAAATA CATCCAATAT   
  
  
+ ACTTTATGTT TTGAATTTAT AATTAATTTT TTTATTTGAA AATATGTTAA CATCGTAGGT ATATTTTTTT   
  
  
+ ATATTAAAAA ATTAATTGGA TTCAGAGTCA TAAAAATAAT CAGGTTTTCT CTAAAAATCG GTATGCATCA   
  
  
+ CTTTCAAAAC ATGAACGCTC ACATTCTCCC AACGGAGGAA AAATGCCTCT AGATGTTGTC AAAAGAAGAC   
  
  
+ GCCTTTATAA TTACAATTTT TTATACCCAT TTTCCATATC CCAAAAGGCT GATGATTATG GCATTTTCGG   
  
  
+ ATTTTTAAAA AAAAGAAAAA AAATGATAAA TACAGAGGTG TTTAGCATTG AAAAGAAAAA TAAATTAAAG   
  
  
+ GACCTGCCGT CAATAAAATT TGGCAGCAAA GAAGAATATA ATTGACCAGT CAATGAGGAC CTGATAACTA   
  
  
+ TAAAAAAAGA GGCGCATGGA CGGGCGCACC CACATGTTA  

- ACTATGGTCT ACTACACAGC ACTTACTAAA CTTTTAGCTT TCGTTACTTA GACTGCTTTT ACCTACTTCC   
  
  
- TACCCAGACC AATCAACAGA AAGAAAACCA CAAATCCGAC CTAATATCAC TAAAATTTCA TTTATTCTAC   
  
  
- CTCCTATCTG ATCTTACTAT GAAACTATTC AGCTAGACCT GCGGTGTTTC TAGTTAGATC CTAAAGCTGT   
  
  
- GGATATTTTA TAAACCTAAT CACAAAACAC AAAAAATTTT TTGAAAAAAA ACAAATCAAA AAAAAAGTAA   
  
  
- AGTGGCAAGT TGTTGAGTTA GAAAAAGAAG AAAAATATTA AAAAAAAGAT AAAGTAGAAA TTTTTAATCC   
  
  
- AACAAAACTT TTAAAACGAA GTATAAAAAA AAAAGACAAC CCAATATATA ATCAGAGTAC CTAAATTAAA   
  
  
- ATAAAAGAGC TAAAGTTGGA AGTTGTGATC TAGATAACCT TCACCCCAAA GTATTATAAA AAAAATAAAT   
  
  
- AAAAGATACT TAAAAAGAAC TAGAGTACTG ATCCAGTGTT CAAATTGTTC AATTGAGTTC AACTGACTCT   
  
  
- AGTAAAAGAA TAACGAAAAA AATTAACTAA AAAAATAAAG TAGGAAGTTA TAACCCAACC TACCTTTAAC   
  
  
- TCTAAGTATA TAAAAAAGAT AAATGAAAGA TACCCCAATA GGACTAGAGT ACACTCAAAT CGTCCAATTC   
  
  
- AGCCCAACTG AAATAAATAA ATAAAAAGAA GAAAAAATTA ACCTGAAACC TTAAAAAAAA TAAAATAAAA   
  
  
- ATACCCTAAT AGGTCTAGAA TACTGAGTTC AGTGCTCAAA TCAACCAACT GGACTCAACT GAACTAAATA   
  
  
- ATAAACCCAA GGAAAAAATT AACTTACAAA AAAAAAGATA AAGTGGAAGG TTGTTGAGTT AACAAAATAA   
  
  
- AGTGCAAAAA AAAGAAGTCA AAGTAGGAAG TTATAATTCA ACAAACCCTT AATCTAAAAC TTTAAAGAAA   
  
  
- AAGTTAAACC AAAAACACCT CAATTGGGCT AGATTATATA AATTAAAAAA AAGAACTAAA ATTAGAAAAT   
  
  
- GTAACCTAGA CAATCTTTTA CCTAGAAACA TTAAAAAATA AACTAAAAAA ACTTCAATAG AGTCAGAATA   
  
  
- CTAGATCCAG TGCTCGAATT GTATAATCGG ATTCAATTGA ACTCAACAAA AAAAATCAAC TAAAAAAAAT   
  
  
- AAAGTAGTAA GTTGTAATTC AATCAACATT TAATCCAAAG TATTAAAAAA ATATATACGA AAGATAACTC   
  
  
- AATATTTTAT AGGGCTAGAG TACTAGGCCC AACGCTCAAA TTGTAGAATT GAATGTAATT GAACCCATCA   
  
  
- AAAAAAATAG GAAAAAAATT AAATTATAAA AAAAAAGTTA AGGTGGGAAG TTATAGTTTA ACTAATCTCT   
  
  
- AACTAGAAGT ATTAAATAAA ACTAAATGAA AGATATATAT TCCAATAATA CCAAAGAACT AAATTCAAAG   
  
  
- TTTAAACTGT CTAATTGAAC ATAGTTAGAA TCTACAAAAA TAAAATTATA AATTTTTTAT GTAGGTTATA   
  
  
- TGAAATACAA AACTTAAATA TTAATTAAAA AAATAAACTT TTATACAATT GTAGCATCCA TATAAAAAAA   
  
  
- TATAATTTTT TAATTAACCT AAGTCTCAGT ATTTTTATTA GTCCAAAAGA GATTTTTAGC CATACGTAGT   
  
  
- GAAAGTTTTG TACTTGCGAG TGTAAGAGGG TTGCCTCCTT TTTACGGAGA TCTACAACAG TTTTCTTCTG   
  
  
- CGGAAATATT AATGTTAAAA AATATGGGTA AAAGGTATAG GGTTTTCCGA CTACTAATAC CGTAAAAGCC   
  
  
- TAAAAATTTT TTTTCTTTTT TTTACTATTT ATGTCTCCAC AAATCGTAAC TTTTCTTTTT ATTTAATTTC   
  
  
- CTGGACGGCA GTTATTTTAA ACCGTCGTTT CTTCTTATAT TAACTGGTCA GTTACTCCTG GACTATTGAT   
  
  
- ATTTTTTTCT CCGCGTACCT GCCCGCGTGG GTGTACAAT

+     G-box

| Site Name | Organism | Position | Strand | Matrix score. | sequence | function |
| --- | --- | --- | --- | --- | --- | --- |
| G-box | Zea mays | 17 | - | 6 | CACGAC | cis-acting regulatory element involved in light responsiveness |

>Potri.006G138900.1   
+ TGATACCAGA TGATGTGTCG TGAATGATTT GAAAATCGAA AGCAATGAAT CTGACGAAAA TGGATGAAGG   
  
  
+ ATGGGTCTGG TTAGTTGTCT TTCTTTTGGT GTTTAGGCTG GATTATAGTG ATTTTAAAGT AAATAAGATG   
  
  
+ GAGGATAGAC TAGAATGATA CTTTGATAAG TCGATCTGGA CGCCACAAAG ATCAATCTAG GATTTCGACA   
  
  
+ CCTATAAAAT ATTTGGATTA GTGTTTTGTG TTTTTTAAAA AACTTTTTTT TGTTTAGTTT TTTTTTCATT   
  
  
+ TCACCGTTCA ACAACTCAAT CTTTTTCTTC TTTTTATAAT TTTTTTTCTA TTTCATCTTT AAAAATTAGG   
  
  
+ TTGTTTTGAA AATTTTGCTT CATATTTTTT TTTTCTGTTG GGTTATATAT TAGTCTCATG GATTTAATTT   
  
  
+ TATTTTCTCG ATTTCAACCT TCAACACTAG ATCTATTGGA AGTGGGGTTT CATAATATTT TTTTTATTTA   
  
  
+ TTTTCTATGA ATTTTTCTTG ATCTCATGAC TAGGTCACAA GTTTAACAAG TTAACTCAAG TTGACTGAGA   
  
  
+ TCATTTTCTT ATTGCTTTTT TTAATTGATT TTTTTATTTC ATCCTTCAAT ATTGGGTTGG ATGGAAATTG   
  
  
+ AGATTCATAT ATTTTTTCTA TTTACTTTCT ATGGGGTTAT CCTGATCTCA TGTGAGTTTA GCAGGTTAAG   
  
  
+ TCGGGTTGAC TTTATTTATT TATTTTTCTT CTTTTTTAAT TGGACTTTGG AATTTTTTTT ATTTTATTTT   
  
  
+ TATGGGATTA TCCAGATCTT ATGACTCAAG TCACGAGTTT AGTTGGTTGA CCTGAGTTGA CTTGATTTAT   
  
  
+ TATTTGGGTT CCTTTTTTAA TTGAATGTTT TTTTTTCTAT TTCACCTTCC AACAACTCAA TTGTTTTATT   
  
  
+ TCACGTTTTT TTTCTTCAGT TTCATCCTTC AATATTAAGT TGTTTGGGAA TTAGATTTTG AAATTTCTTT   
  
  
+ TTCAATTTGG TTTTTGTGGA GTTAACCCGA TCTAATATAT TTAATTTTTT TTCTTGATTT TAATCTTTTA   
  
  
+ CATTGGATCT GTTAGAAAAT GGATCTTTGT AATTTTTTAT TTGATTTTTT TGAAGTTATC TCAGTCTTAT   
  
  
+ GATCTAGGTC ACGAGCTTAA CATATTAGCC TAAGTTAACT TGAGTTGTTT TTTTTAGTTG ATTTTTTTTA   
  
  
+ TTTCATCATT CAACATTAAG TTAGTTGTAA ATTAGGTTTC ATAATTTTTT TATATATGCT TTCTATTGAG   
  
  
+ TTATAAAATA TCCCGATCTC ATGATCCGGG TTGCGAGTTT AACATCTTAA CTTACATTAA CTTGGGTAGT   
  
  
+ TTTTTTTATC CTTTTTTTAA TTTAATATTT TTTTTTCAAT TCCACCCTTC AATATCAAAT TGATTAGAGA   
  
  
+ TTGATCTTCA TAATTTATTT TGATTTACTT TCTATATATA AGGTTATTAT GGTTTCTTGA TTTAAGTTTC   
  
  
+ AAATTTGACA GATTAACTTG TATCAATCTT AGATGTTTTT ATTTTAATAT TTAAAAAATA CATCCAATAT   
  
  
+ ACTTTATGTT TTGAATTTAT AATTAATTTT TTTATTTGAA AATATGTTAA CATCGTAGGT ATATTTTTTT   
  
  
+ ATATTAAAAA ATTAATTGGA TTCAGAGTCA TAAAAATAAT CAGGTTTTCT CTAAAAATCG GTATGCATCA   
  
  
+ CTTTCAAAAC ATGAACGCTC ACATTCTCCC AACGGAGGAA AAATGCCTCT AGATGTTGTC AAAAGAAGAC   
  
  
+ GCCTTTATAA TTACAATTTT TTATACCCAT TTTCCATATC CCAAAAGGCT GATGATTATG GCATTTTCGG   
  
  
+ ATTTTTAAAA AAAAGAAAAA AAATGATAAA TACAGAGGTG TTTAGCATTG AAAAGAAAAA TAAATTAAAG   
  
  
+ GACCTGCCGT CAATAAAATT TGGCAGCAAA GAAGAATATA ATTGACCAGT CAATGAGGAC CTGATAACTA   
  
  
+ TAAAAAAAGA GGCGCATGGA CGGGCGCACC CACATGTTA  

- ACTATGGTCT ACTACACAGC ACTTACTAAA CTTTTAGCTT TCGTTACTTA GACTGCTTTT ACCTACTTCC   
  
  
- TACCCAGACC AATCAACAGA AAGAAAACCA CAAATCCGAC CTAATATCAC TAAAATTTCA TTTATTCTAC   
  
  
- CTCCTATCTG ATCTTACTAT GAAACTATTC AGCTAGACCT GCGGTGTTTC TAGTTAGATC CTAAAGCTGT   
  
  
- GGATATTTTA TAAACCTAAT CACAAAACAC AAAAAATTTT TTGAAAAAAA ACAAATCAAA AAAAAAGTAA   
  
  
- AGTGGCAAGT TGTTGAGTTA GAAAAAGAAG AAAAATATTA AAAAAAAGAT AAAGTAGAAA TTTTTAATCC   
  
  
- AACAAAACTT TTAAAACGAA GTATAAAAAA AAAAGACAAC CCAATATATA ATCAGAGTAC CTAAATTAAA   
  
  
- ATAAAAGAGC TAAAGTTGGA AGTTGTGATC TAGATAACCT TCACCCCAAA GTATTATAAA AAAAATAAAT   
  
  
- AAAAGATACT TAAAAAGAAC TAGAGTACTG ATCCAGTGTT CAAATTGTTC AATTGAGTTC AACTGACTCT   
  
  
- AGTAAAAGAA TAACGAAAAA AATTAACTAA AAAAATAAAG TAGGAAGTTA TAACCCAACC TACCTTTAAC   
  
  
- TCTAAGTATA TAAAAAAGAT AAATGAAAGA TACCCCAATA GGACTAGAGT ACACTCAAAT CGTCCAATTC   
  
  
- AGCCCAACTG AAATAAATAA ATAAAAAGAA GAAAAAATTA ACCTGAAACC TTAAAAAAAA TAAAATAAAA   
  
  
- ATACCCTAAT AGGTCTAGAA TACTGAGTTC AGTGCTCAAA TCAACCAACT GGACTCAACT GAACTAAATA   
  
  
- ATAAACCCAA GGAAAAAATT AACTTACAAA AAAAAAGATA AAGTGGAAGG TTGTTGAGTT AACAAAATAA   
  
  
- AGTGCAAAAA AAAGAAGTCA AAGTAGGAAG TTATAATTCA ACAAACCCTT AATCTAAAAC TTTAAAGAAA   
  
  
- AAGTTAAACC AAAAACACCT CAATTGGGCT AGATTATATA AATTAAAAAA AAGAACTAAA ATTAGAAAAT   
  
  
- GTAACCTAGA CAATCTTTTA CCTAGAAACA TTAAAAAATA AACTAAAAAA ACTTCAATAG AGTCAGAATA   
  
  
- CTAGATCCAG TGCTCGAATT GTATAATCGG ATTCAATTGA ACTCAACAAA AAAAATCAAC TAAAAAAAAT   
  
  
- AAAGTAGTAA GTTGTAATTC AATCAACATT TAATCCAAAG TATTAAAAAA ATATATACGA AAGATAACTC   
  
  
- AATATTTTAT AGGGCTAGAG TACTAGGCCC AACGCTCAAA TTGTAGAATT GAATGTAATT GAACCCATCA   
  
  
- AAAAAAATAG GAAAAAAATT AAATTATAAA AAAAAAGTTA AGGTGGGAAG TTATAGTTTA ACTAATCTCT   
  
  
- AACTAGAAGT ATTAAATAAA ACTAAATGAA AGATATATAT TCCAATAATA CCAAAGAACT AAATTCAAAG   
  
  
- TTTAAACTGT CTAATTGAAC ATAGTTAGAA TCTACAAAAA TAAAATTATA AATTTTTTAT GTAGGTTATA   
  
  
- TGAAATACAA AACTTAAATA TTAATTAAAA AAATAAACTT TTATACAATT GTAGCATCCA TATAAAAAAA   
  
  
- TATAATTTTT TAATTAACCT AAGTCTCAGT ATTTTTATTA GTCCAAAAGA GATTTTTAGC CATACGTAGT   
  
  
- GAAAGTTTTG TACTTGCGAG TGTAAGAGGG TTGCCTCCTT TTTACGGAGA TCTACAACAG TTTTCTTCTG   
  
  
- CGGAAATATT AATGTTAAAA AATATGGGTA AAAGGTATAG GGTTTTCCGA CTACTAATAC CGTAAAAGCC   
  
  
- TAAAAATTTT TTTTCTTTTT TTTACTATTT ATGTCTCCAC AAATCGTAAC TTTTCTTTTT ATTTAATTTC   
  
  
- CTGGACGGCA GTTATTTTAA ACCGTCGTTT CTTCTTATAT TAACTGGTCA GTTACTCCTG GACTATTGAT   
  
  
- ATTTTTTTCT CCGCGTACCT GCCCGCGTGG GTGTACAAT

+     GARE-motif

| Site Name | Organism | Position | Strand | Matrix score. | sequence | function |
| --- | --- | --- | --- | --- | --- | --- |
| GARE-motif | Brassica oleracea | 384 | + | 7 | TCTGTTG | gibberellin-responsive element |

>Potri.006G138900.1   
+ TGATACCAGA TGATGTGTCG TGAATGATTT GAAAATCGAA AGCAATGAAT CTGACGAAAA TGGATGAAGG   
  
  
+ ATGGGTCTGG TTAGTTGTCT TTCTTTTGGT GTTTAGGCTG GATTATAGTG ATTTTAAAGT AAATAAGATG   
  
  
+ GAGGATAGAC TAGAATGATA CTTTGATAAG TCGATCTGGA CGCCACAAAG ATCAATCTAG GATTTCGACA   
  
  
+ CCTATAAAAT ATTTGGATTA GTGTTTTGTG TTTTTTAAAA AACTTTTTTT TGTTTAGTTT TTTTTTCATT   
  
  
+ TCACCGTTCA ACAACTCAAT CTTTTTCTTC TTTTTATAAT TTTTTTTCTA TTTCATCTTT AAAAATTAGG   
  
  
+ TTGTTTTGAA AATTTTGCTT CATATTTTTT TTTTCTGTTG GGTTATATAT TAGTCTCATG GATTTAATTT   
  
  
+ TATTTTCTCG ATTTCAACCT TCAACACTAG ATCTATTGGA AGTGGGGTTT CATAATATTT TTTTTATTTA   
  
  
+ TTTTCTATGA ATTTTTCTTG ATCTCATGAC TAGGTCACAA GTTTAACAAG TTAACTCAAG TTGACTGAGA   
  
  
+ TCATTTTCTT ATTGCTTTTT TTAATTGATT TTTTTATTTC ATCCTTCAAT ATTGGGTTGG ATGGAAATTG   
  
  
+ AGATTCATAT ATTTTTTCTA TTTACTTTCT ATGGGGTTAT CCTGATCTCA TGTGAGTTTA GCAGGTTAAG   
  
  
+ TCGGGTTGAC TTTATTTATT TATTTTTCTT CTTTTTTAAT TGGACTTTGG AATTTTTTTT ATTTTATTTT   
  
  
+ TATGGGATTA TCCAGATCTT ATGACTCAAG TCACGAGTTT AGTTGGTTGA CCTGAGTTGA CTTGATTTAT   
  
  
+ TATTTGGGTT CCTTTTTTAA TTGAATGTTT TTTTTTCTAT TTCACCTTCC AACAACTCAA TTGTTTTATT   
  
  
+ TCACGTTTTT TTTCTTCAGT TTCATCCTTC AATATTAAGT TGTTTGGGAA TTAGATTTTG AAATTTCTTT   
  
  
+ TTCAATTTGG TTTTTGTGGA GTTAACCCGA TCTAATATAT TTAATTTTTT TTCTTGATTT TAATCTTTTA   
  
  
+ CATTGGATCT GTTAGAAAAT GGATCTTTGT AATTTTTTAT TTGATTTTTT TGAAGTTATC TCAGTCTTAT   
  
  
+ GATCTAGGTC ACGAGCTTAA CATATTAGCC TAAGTTAACT TGAGTTGTTT TTTTTAGTTG ATTTTTTTTA   
  
  
+ TTTCATCATT CAACATTAAG TTAGTTGTAA ATTAGGTTTC ATAATTTTTT TATATATGCT TTCTATTGAG   
  
  
+ TTATAAAATA TCCCGATCTC ATGATCCGGG TTGCGAGTTT AACATCTTAA CTTACATTAA CTTGGGTAGT   
  
  
+ TTTTTTTATC CTTTTTTTAA TTTAATATTT TTTTTTCAAT TCCACCCTTC AATATCAAAT TGATTAGAGA   
  
  
+ TTGATCTTCA TAATTTATTT TGATTTACTT TCTATATATA AGGTTATTAT GGTTTCTTGA TTTAAGTTTC   
  
  
+ AAATTTGACA GATTAACTTG TATCAATCTT AGATGTTTTT ATTTTAATAT TTAAAAAATA CATCCAATAT   
  
  
+ ACTTTATGTT TTGAATTTAT AATTAATTTT TTTATTTGAA AATATGTTAA CATCGTAGGT ATATTTTTTT   
  
  
+ ATATTAAAAA ATTAATTGGA TTCAGAGTCA TAAAAATAAT CAGGTTTTCT CTAAAAATCG GTATGCATCA   
  
  
+ CTTTCAAAAC ATGAACGCTC ACATTCTCCC AACGGAGGAA AAATGCCTCT AGATGTTGTC AAAAGAAGAC   
  
  
+ GCCTTTATAA TTACAATTTT TTATACCCAT TTTCCATATC CCAAAAGGCT GATGATTATG GCATTTTCGG   
  
  
+ ATTTTTAAAA AAAAGAAAAA AAATGATAAA TACAGAGGTG TTTAGCATTG AAAAGAAAAA TAAATTAAAG   
  
  
+ GACCTGCCGT CAATAAAATT TGGCAGCAAA GAAGAATATA ATTGACCAGT CAATGAGGAC CTGATAACTA   
  
  
+ TAAAAAAAGA GGCGCATGGA CGGGCGCACC CACATGTTA  

- ACTATGGTCT ACTACACAGC ACTTACTAAA CTTTTAGCTT TCGTTACTTA GACTGCTTTT ACCTACTTCC   
  
  
- TACCCAGACC AATCAACAGA AAGAAAACCA CAAATCCGAC CTAATATCAC TAAAATTTCA TTTATTCTAC   
  
  
- CTCCTATCTG ATCTTACTAT GAAACTATTC AGCTAGACCT GCGGTGTTTC TAGTTAGATC CTAAAGCTGT   
  
  
- GGATATTTTA TAAACCTAAT CACAAAACAC AAAAAATTTT TTGAAAAAAA ACAAATCAAA AAAAAAGTAA   
  
  
- AGTGGCAAGT TGTTGAGTTA GAAAAAGAAG AAAAATATTA AAAAAAAGAT AAAGTAGAAA TTTTTAATCC   
  
  
- AACAAAACTT TTAAAACGAA GTATAAAAAA AAAAGACAAC CCAATATATA ATCAGAGTAC CTAAATTAAA   
  
  
- ATAAAAGAGC TAAAGTTGGA AGTTGTGATC TAGATAACCT TCACCCCAAA GTATTATAAA AAAAATAAAT   
  
  
- AAAAGATACT TAAAAAGAAC TAGAGTACTG ATCCAGTGTT CAAATTGTTC AATTGAGTTC AACTGACTCT   
  
  
- AGTAAAAGAA TAACGAAAAA AATTAACTAA AAAAATAAAG TAGGAAGTTA TAACCCAACC TACCTTTAAC   
  
  
- TCTAAGTATA TAAAAAAGAT AAATGAAAGA TACCCCAATA GGACTAGAGT ACACTCAAAT CGTCCAATTC   
  
  
- AGCCCAACTG AAATAAATAA ATAAAAAGAA GAAAAAATTA ACCTGAAACC TTAAAAAAAA TAAAATAAAA   
  
  
- ATACCCTAAT AGGTCTAGAA TACTGAGTTC AGTGCTCAAA TCAACCAACT GGACTCAACT GAACTAAATA   
  
  
- ATAAACCCAA GGAAAAAATT AACTTACAAA AAAAAAGATA AAGTGGAAGG TTGTTGAGTT AACAAAATAA   
  
  
- AGTGCAAAAA AAAGAAGTCA AAGTAGGAAG TTATAATTCA ACAAACCCTT AATCTAAAAC TTTAAAGAAA   
  
  
- AAGTTAAACC AAAAACACCT CAATTGGGCT AGATTATATA AATTAAAAAA AAGAACTAAA ATTAGAAAAT   
  
  
- GTAACCTAGA CAATCTTTTA CCTAGAAACA TTAAAAAATA AACTAAAAAA ACTTCAATAG AGTCAGAATA   
  
  
- CTAGATCCAG TGCTCGAATT GTATAATCGG ATTCAATTGA ACTCAACAAA AAAAATCAAC TAAAAAAAAT   
  
  
- AAAGTAGTAA GTTGTAATTC AATCAACATT TAATCCAAAG TATTAAAAAA ATATATACGA AAGATAACTC   
  
  
- AATATTTTAT AGGGCTAGAG TACTAGGCCC AACGCTCAAA TTGTAGAATT GAATGTAATT GAACCCATCA   
  
  
- AAAAAAATAG GAAAAAAATT AAATTATAAA AAAAAAGTTA AGGTGGGAAG TTATAGTTTA ACTAATCTCT   
  
  
- AACTAGAAGT ATTAAATAAA ACTAAATGAA AGATATATAT TCCAATAATA CCAAAGAACT AAATTCAAAG   
  
  
- TTTAAACTGT CTAATTGAAC ATAGTTAGAA TCTACAAAAA TAAAATTATA AATTTTTTAT GTAGGTTATA   
  
  
- TGAAATACAA AACTTAAATA TTAATTAAAA AAATAAACTT TTATACAATT GTAGCATCCA TATAAAAAAA   
  
  
- TATAATTTTT TAATTAACCT AAGTCTCAGT ATTTTTATTA GTCCAAAAGA GATTTTTAGC CATACGTAGT   
  
  
- GAAAGTTTTG TACTTGCGAG TGTAAGAGGG TTGCCTCCTT TTTACGGAGA TCTACAACAG TTTTCTTCTG   
  
  
- CGGAAATATT AATGTTAAAA AATATGGGTA AAAGGTATAG GGTTTTCCGA CTACTAATAC CGTAAAAGCC   
  
  
- TAAAAATTTT TTTTCTTTTT TTTACTATTT ATGTCTCCAC AAATCGTAAC TTTTCTTTTT ATTTAATTTC   
  
  
- CTGGACGGCA GTTATTTTAA ACCGTCGTTT CTTCTTATAT TAACTGGTCA GTTACTCCTG GACTATTGAT   
  
  
- ATTTTTTTCT CCGCGTACCT GCCCGCGTGG GTGTACAAT

+     GCN4\_motif

| Site Name | Organism | Position | Strand | Matrix score. | sequence | function |
| --- | --- | --- | --- | --- | --- | --- |
| GCN4\_motif | Oryza sativa | 792 | - | 7 | TGAGTCA | cis-regulatory element involved in endosperm expression |

>Potri.006G138900.1   
+ TGATACCAGA TGATGTGTCG TGAATGATTT GAAAATCGAA AGCAATGAAT CTGACGAAAA TGGATGAAGG   
  
  
+ ATGGGTCTGG TTAGTTGTCT TTCTTTTGGT GTTTAGGCTG GATTATAGTG ATTTTAAAGT AAATAAGATG   
  
  
+ GAGGATAGAC TAGAATGATA CTTTGATAAG TCGATCTGGA CGCCACAAAG ATCAATCTAG GATTTCGACA   
  
  
+ CCTATAAAAT ATTTGGATTA GTGTTTTGTG TTTTTTAAAA AACTTTTTTT TGTTTAGTTT TTTTTTCATT   
  
  
+ TCACCGTTCA ACAACTCAAT CTTTTTCTTC TTTTTATAAT TTTTTTTCTA TTTCATCTTT AAAAATTAGG   
  
  
+ TTGTTTTGAA AATTTTGCTT CATATTTTTT TTTTCTGTTG GGTTATATAT TAGTCTCATG GATTTAATTT   
  
  
+ TATTTTCTCG ATTTCAACCT TCAACACTAG ATCTATTGGA AGTGGGGTTT CATAATATTT TTTTTATTTA   
  
  
+ TTTTCTATGA ATTTTTCTTG ATCTCATGAC TAGGTCACAA GTTTAACAAG TTAACTCAAG TTGACTGAGA   
  
  
+ TCATTTTCTT ATTGCTTTTT TTAATTGATT TTTTTATTTC ATCCTTCAAT ATTGGGTTGG ATGGAAATTG   
  
  
+ AGATTCATAT ATTTTTTCTA TTTACTTTCT ATGGGGTTAT CCTGATCTCA TGTGAGTTTA GCAGGTTAAG   
  
  
+ TCGGGTTGAC TTTATTTATT TATTTTTCTT CTTTTTTAAT TGGACTTTGG AATTTTTTTT ATTTTATTTT   
  
  
+ TATGGGATTA TCCAGATCTT ATGACTCAAG TCACGAGTTT AGTTGGTTGA CCTGAGTTGA CTTGATTTAT   
  
  
+ TATTTGGGTT CCTTTTTTAA TTGAATGTTT TTTTTTCTAT TTCACCTTCC AACAACTCAA TTGTTTTATT   
  
  
+ TCACGTTTTT TTTCTTCAGT TTCATCCTTC AATATTAAGT TGTTTGGGAA TTAGATTTTG AAATTTCTTT   
  
  
+ TTCAATTTGG TTTTTGTGGA GTTAACCCGA TCTAATATAT TTAATTTTTT TTCTTGATTT TAATCTTTTA   
  
  
+ CATTGGATCT GTTAGAAAAT GGATCTTTGT AATTTTTTAT TTGATTTTTT TGAAGTTATC TCAGTCTTAT   
  
  
+ GATCTAGGTC ACGAGCTTAA CATATTAGCC TAAGTTAACT TGAGTTGTTT TTTTTAGTTG ATTTTTTTTA   
  
  
+ TTTCATCATT CAACATTAAG TTAGTTGTAA ATTAGGTTTC ATAATTTTTT TATATATGCT TTCTATTGAG   
  
  
+ TTATAAAATA TCCCGATCTC ATGATCCGGG TTGCGAGTTT AACATCTTAA CTTACATTAA CTTGGGTAGT   
  
  
+ TTTTTTTATC CTTTTTTTAA TTTAATATTT TTTTTTCAAT TCCACCCTTC AATATCAAAT TGATTAGAGA   
  
  
+ TTGATCTTCA TAATTTATTT TGATTTACTT TCTATATATA AGGTTATTAT GGTTTCTTGA TTTAAGTTTC   
  
  
+ AAATTTGACA GATTAACTTG TATCAATCTT AGATGTTTTT ATTTTAATAT TTAAAAAATA CATCCAATAT   
  
  
+ ACTTTATGTT TTGAATTTAT AATTAATTTT TTTATTTGAA AATATGTTAA CATCGTAGGT ATATTTTTTT   
  
  
+ ATATTAAAAA ATTAATTGGA TTCAGAGTCA TAAAAATAAT CAGGTTTTCT CTAAAAATCG GTATGCATCA   
  
  
+ CTTTCAAAAC ATGAACGCTC ACATTCTCCC AACGGAGGAA AAATGCCTCT AGATGTTGTC AAAAGAAGAC   
  
  
+ GCCTTTATAA TTACAATTTT TTATACCCAT TTTCCATATC CCAAAAGGCT GATGATTATG GCATTTTCGG   
  
  
+ ATTTTTAAAA AAAAGAAAAA AAATGATAAA TACAGAGGTG TTTAGCATTG AAAAGAAAAA TAAATTAAAG   
  
  
+ GACCTGCCGT CAATAAAATT TGGCAGCAAA GAAGAATATA ATTGACCAGT CAATGAGGAC CTGATAACTA   
  
  
+ TAAAAAAAGA GGCGCATGGA CGGGCGCACC CACATGTTA  

- ACTATGGTCT ACTACACAGC ACTTACTAAA CTTTTAGCTT TCGTTACTTA GACTGCTTTT ACCTACTTCC   
  
  
- TACCCAGACC AATCAACAGA AAGAAAACCA CAAATCCGAC CTAATATCAC TAAAATTTCA TTTATTCTAC   
  
  
- CTCCTATCTG ATCTTACTAT GAAACTATTC AGCTAGACCT GCGGTGTTTC TAGTTAGATC CTAAAGCTGT   
  
  
- GGATATTTTA TAAACCTAAT CACAAAACAC AAAAAATTTT TTGAAAAAAA ACAAATCAAA AAAAAAGTAA   
  
  
- AGTGGCAAGT TGTTGAGTTA GAAAAAGAAG AAAAATATTA AAAAAAAGAT AAAGTAGAAA TTTTTAATCC   
  
  
- AACAAAACTT TTAAAACGAA GTATAAAAAA AAAAGACAAC CCAATATATA ATCAGAGTAC CTAAATTAAA   
  
  
- ATAAAAGAGC TAAAGTTGGA AGTTGTGATC TAGATAACCT TCACCCCAAA GTATTATAAA AAAAATAAAT   
  
  
- AAAAGATACT TAAAAAGAAC TAGAGTACTG ATCCAGTGTT CAAATTGTTC AATTGAGTTC AACTGACTCT   
  
  
- AGTAAAAGAA TAACGAAAAA AATTAACTAA AAAAATAAAG TAGGAAGTTA TAACCCAACC TACCTTTAAC   
  
  
- TCTAAGTATA TAAAAAAGAT AAATGAAAGA TACCCCAATA GGACTAGAGT ACACTCAAAT CGTCCAATTC   
  
  
- AGCCCAACTG AAATAAATAA ATAAAAAGAA GAAAAAATTA ACCTGAAACC TTAAAAAAAA TAAAATAAAA   
  
  
- ATACCCTAAT AGGTCTAGAA TACTGAGTTC AGTGCTCAAA TCAACCAACT GGACTCAACT GAACTAAATA   
  
  
- ATAAACCCAA GGAAAAAATT AACTTACAAA AAAAAAGATA AAGTGGAAGG TTGTTGAGTT AACAAAATAA   
  
  
- AGTGCAAAAA AAAGAAGTCA AAGTAGGAAG TTATAATTCA ACAAACCCTT AATCTAAAAC TTTAAAGAAA   
  
  
- AAGTTAAACC AAAAACACCT CAATTGGGCT AGATTATATA AATTAAAAAA AAGAACTAAA ATTAGAAAAT   
  
  
- GTAACCTAGA CAATCTTTTA CCTAGAAACA TTAAAAAATA AACTAAAAAA ACTTCAATAG AGTCAGAATA   
  
  
- CTAGATCCAG TGCTCGAATT GTATAATCGG ATTCAATTGA ACTCAACAAA AAAAATCAAC TAAAAAAAAT   
  
  
- AAAGTAGTAA GTTGTAATTC AATCAACATT TAATCCAAAG TATTAAAAAA ATATATACGA AAGATAACTC   
  
  
- AATATTTTAT AGGGCTAGAG TACTAGGCCC AACGCTCAAA TTGTAGAATT GAATGTAATT GAACCCATCA   
  
  
- AAAAAAATAG GAAAAAAATT AAATTATAAA AAAAAAGTTA AGGTGGGAAG TTATAGTTTA ACTAATCTCT   
  
  
- AACTAGAAGT ATTAAATAAA ACTAAATGAA AGATATATAT TCCAATAATA CCAAAGAACT AAATTCAAAG   
  
  
- TTTAAACTGT CTAATTGAAC ATAGTTAGAA TCTACAAAAA TAAAATTATA AATTTTTTAT GTAGGTTATA   
  
  
- TGAAATACAA AACTTAAATA TTAATTAAAA AAATAAACTT TTATACAATT GTAGCATCCA TATAAAAAAA   
  
  
- TATAATTTTT TAATTAACCT AAGTCTCAGT ATTTTTATTA GTCCAAAAGA GATTTTTAGC CATACGTAGT   
  
  
- GAAAGTTTTG TACTTGCGAG TGTAAGAGGG TTGCCTCCTT TTTACGGAGA TCTACAACAG TTTTCTTCTG   
  
  
- CGGAAATATT AATGTTAAAA AATATGGGTA AAAGGTATAG GGTTTTCCGA CTACTAATAC CGTAAAAGCC   
  
  
- TAAAAATTTT TTTTCTTTTT TTTACTATTT ATGTCTCCAC AAATCGTAAC TTTTCTTTTT ATTTAATTTC   
  
  
- CTGGACGGCA GTTATTTTAA ACCGTCGTTT CTTCTTATAT TAACTGGTCA GTTACTCCTG GACTATTGAT   
  
  
- ATTTTTTTCT CCGCGTACCT GCCCGCGTGG GTGTACAAT

+     GT1-motif

| Site Name | Organism | Position | Strand | Matrix score. | sequence | function |
| --- | --- | --- | --- | --- | --- | --- |
| GT1-motif | Arabidopsis thaliana | 1002 | - | 6 | GGTTAA | light responsive element |
| GT1-motif | Arabidopsis thaliana | 694 | + | 6 | GGTTAA | light responsive element |

>Potri.006G138900.1   
+ TGATACCAGA TGATGTGTCG TGAATGATTT GAAAATCGAA AGCAATGAAT CTGACGAAAA TGGATGAAGG   
  
  
+ ATGGGTCTGG TTAGTTGTCT TTCTTTTGGT GTTTAGGCTG GATTATAGTG ATTTTAAAGT AAATAAGATG   
  
  
+ GAGGATAGAC TAGAATGATA CTTTGATAAG TCGATCTGGA CGCCACAAAG ATCAATCTAG GATTTCGACA   
  
  
+ CCTATAAAAT ATTTGGATTA GTGTTTTGTG TTTTTTAAAA AACTTTTTTT TGTTTAGTTT TTTTTTCATT   
  
  
+ TCACCGTTCA ACAACTCAAT CTTTTTCTTC TTTTTATAAT TTTTTTTCTA TTTCATCTTT AAAAATTAGG   
  
  
+ TTGTTTTGAA AATTTTGCTT CATATTTTTT TTTTCTGTTG GGTTATATAT TAGTCTCATG GATTTAATTT   
  
  
+ TATTTTCTCG ATTTCAACCT TCAACACTAG ATCTATTGGA AGTGGGGTTT CATAATATTT TTTTTATTTA   
  
  
+ TTTTCTATGA ATTTTTCTTG ATCTCATGAC TAGGTCACAA GTTTAACAAG TTAACTCAAG TTGACTGAGA   
  
  
+ TCATTTTCTT ATTGCTTTTT TTAATTGATT TTTTTATTTC ATCCTTCAAT ATTGGGTTGG ATGGAAATTG   
  
  
+ AGATTCATAT ATTTTTTCTA TTTACTTTCT ATGGGGTTAT CCTGATCTCA TGTGAGTTTA GCAGGTTAAG   
  
  
+ TCGGGTTGAC TTTATTTATT TATTTTTCTT CTTTTTTAAT TGGACTTTGG AATTTTTTTT ATTTTATTTT   
  
  
+ TATGGGATTA TCCAGATCTT ATGACTCAAG TCACGAGTTT AGTTGGTTGA CCTGAGTTGA CTTGATTTAT   
  
  
+ TATTTGGGTT CCTTTTTTAA TTGAATGTTT TTTTTTCTAT TTCACCTTCC AACAACTCAA TTGTTTTATT   
  
  
+ TCACGTTTTT TTTCTTCAGT TTCATCCTTC AATATTAAGT TGTTTGGGAA TTAGATTTTG AAATTTCTTT   
  
  
+ TTCAATTTGG TTTTTGTGGA GTTAACCCGA TCTAATATAT TTAATTTTTT TTCTTGATTT TAATCTTTTA   
  
  
+ CATTGGATCT GTTAGAAAAT GGATCTTTGT AATTTTTTAT TTGATTTTTT TGAAGTTATC TCAGTCTTAT   
  
  
+ GATCTAGGTC ACGAGCTTAA CATATTAGCC TAAGTTAACT TGAGTTGTTT TTTTTAGTTG ATTTTTTTTA   
  
  
+ TTTCATCATT CAACATTAAG TTAGTTGTAA ATTAGGTTTC ATAATTTTTT TATATATGCT TTCTATTGAG   
  
  
+ TTATAAAATA TCCCGATCTC ATGATCCGGG TTGCGAGTTT AACATCTTAA CTTACATTAA CTTGGGTAGT   
  
  
+ TTTTTTTATC CTTTTTTTAA TTTAATATTT TTTTTTCAAT TCCACCCTTC AATATCAAAT TGATTAGAGA   
  
  
+ TTGATCTTCA TAATTTATTT TGATTTACTT TCTATATATA AGGTTATTAT GGTTTCTTGA TTTAAGTTTC   
  
  
+ AAATTTGACA GATTAACTTG TATCAATCTT AGATGTTTTT ATTTTAATAT TTAAAAAATA CATCCAATAT   
  
  
+ ACTTTATGTT TTGAATTTAT AATTAATTTT TTTATTTGAA AATATGTTAA CATCGTAGGT ATATTTTTTT   
  
  
+ ATATTAAAAA ATTAATTGGA TTCAGAGTCA TAAAAATAAT CAGGTTTTCT CTAAAAATCG GTATGCATCA   
  
  
+ CTTTCAAAAC ATGAACGCTC ACATTCTCCC AACGGAGGAA AAATGCCTCT AGATGTTGTC AAAAGAAGAC   
  
  
+ GCCTTTATAA TTACAATTTT TTATACCCAT TTTCCATATC CCAAAAGGCT GATGATTATG GCATTTTCGG   
  
  
+ ATTTTTAAAA AAAAGAAAAA AAATGATAAA TACAGAGGTG TTTAGCATTG AAAAGAAAAA TAAATTAAAG   
  
  
+ GACCTGCCGT CAATAAAATT TGGCAGCAAA GAAGAATATA ATTGACCAGT CAATGAGGAC CTGATAACTA   
  
  
+ TAAAAAAAGA GGCGCATGGA CGGGCGCACC CACATGTTA  

- ACTATGGTCT ACTACACAGC ACTTACTAAA CTTTTAGCTT TCGTTACTTA GACTGCTTTT ACCTACTTCC   
  
  
- TACCCAGACC AATCAACAGA AAGAAAACCA CAAATCCGAC CTAATATCAC TAAAATTTCA TTTATTCTAC   
  
  
- CTCCTATCTG ATCTTACTAT GAAACTATTC AGCTAGACCT GCGGTGTTTC TAGTTAGATC CTAAAGCTGT   
  
  
- GGATATTTTA TAAACCTAAT CACAAAACAC AAAAAATTTT TTGAAAAAAA ACAAATCAAA AAAAAAGTAA   
  
  
- AGTGGCAAGT TGTTGAGTTA GAAAAAGAAG AAAAATATTA AAAAAAAGAT AAAGTAGAAA TTTTTAATCC   
  
  
- AACAAAACTT TTAAAACGAA GTATAAAAAA AAAAGACAAC CCAATATATA ATCAGAGTAC CTAAATTAAA   
  
  
- ATAAAAGAGC TAAAGTTGGA AGTTGTGATC TAGATAACCT TCACCCCAAA GTATTATAAA AAAAATAAAT   
  
  
- AAAAGATACT TAAAAAGAAC TAGAGTACTG ATCCAGTGTT CAAATTGTTC AATTGAGTTC AACTGACTCT   
  
  
- AGTAAAAGAA TAACGAAAAA AATTAACTAA AAAAATAAAG TAGGAAGTTA TAACCCAACC TACCTTTAAC   
  
  
- TCTAAGTATA TAAAAAAGAT AAATGAAAGA TACCCCAATA GGACTAGAGT ACACTCAAAT CGTCCAATTC   
  
  
- AGCCCAACTG AAATAAATAA ATAAAAAGAA GAAAAAATTA ACCTGAAACC TTAAAAAAAA TAAAATAAAA   
  
  
- ATACCCTAAT AGGTCTAGAA TACTGAGTTC AGTGCTCAAA TCAACCAACT GGACTCAACT GAACTAAATA   
  
  
- ATAAACCCAA GGAAAAAATT AACTTACAAA AAAAAAGATA AAGTGGAAGG TTGTTGAGTT AACAAAATAA   
  
  
- AGTGCAAAAA AAAGAAGTCA AAGTAGGAAG TTATAATTCA ACAAACCCTT AATCTAAAAC TTTAAAGAAA   
  
  
- AAGTTAAACC AAAAACACCT CAATTGGGCT AGATTATATA AATTAAAAAA AAGAACTAAA ATTAGAAAAT   
  
  
- GTAACCTAGA CAATCTTTTA CCTAGAAACA TTAAAAAATA AACTAAAAAA ACTTCAATAG AGTCAGAATA   
  
  
- CTAGATCCAG TGCTCGAATT GTATAATCGG ATTCAATTGA ACTCAACAAA AAAAATCAAC TAAAAAAAAT   
  
  
- AAAGTAGTAA GTTGTAATTC AATCAACATT TAATCCAAAG TATTAAAAAA ATATATACGA AAGATAACTC   
  
  
- AATATTTTAT AGGGCTAGAG TACTAGGCCC AACGCTCAAA TTGTAGAATT GAATGTAATT GAACCCATCA   
  
  
- AAAAAAATAG GAAAAAAATT AAATTATAAA AAAAAAGTTA AGGTGGGAAG TTATAGTTTA ACTAATCTCT   
  
  
- AACTAGAAGT ATTAAATAAA ACTAAATGAA AGATATATAT TCCAATAATA CCAAAGAACT AAATTCAAAG   
  
  
- TTTAAACTGT CTAATTGAAC ATAGTTAGAA TCTACAAAAA TAAAATTATA AATTTTTTAT GTAGGTTATA   
  
  
- TGAAATACAA AACTTAAATA TTAATTAAAA AAATAAACTT TTATACAATT GTAGCATCCA TATAAAAAAA   
  
  
- TATAATTTTT TAATTAACCT AAGTCTCAGT ATTTTTATTA GTCCAAAAGA GATTTTTAGC CATACGTAGT   
  
  
- GAAAGTTTTG TACTTGCGAG TGTAAGAGGG TTGCCTCCTT TTTACGGAGA TCTACAACAG TTTTCTTCTG   
  
  
- CGGAAATATT AATGTTAAAA AATATGGGTA AAAGGTATAG GGTTTTCCGA CTACTAATAC CGTAAAAGCC   
  
  
- TAAAAATTTT TTTTCTTTTT TTTACTATTT ATGTCTCCAC AAATCGTAAC TTTTCTTTTT ATTTAATTTC   
  
  
- CTGGACGGCA GTTATTTTAA ACCGTCGTTT CTTCTTATAT TAACTGGTCA GTTACTCCTG GACTATTGAT   
  
  
- ATTTTTTTCT CCGCGTACCT GCCCGCGTGG GTGTACAAT

+     LTR

| Site Name | Organism | Position | Strand | Matrix score. | sequence | function |
| --- | --- | --- | --- | --- | --- | --- |
| LTR | Hordeum vulgare | 1815 | - | 6 | CCGAAA | cis-acting element involved in low-temperature responsiveness |

>Potri.006G138900.1   
+ TGATACCAGA TGATGTGTCG TGAATGATTT GAAAATCGAA AGCAATGAAT CTGACGAAAA TGGATGAAGG   
  
  
+ ATGGGTCTGG TTAGTTGTCT TTCTTTTGGT GTTTAGGCTG GATTATAGTG ATTTTAAAGT AAATAAGATG   
  
  
+ GAGGATAGAC TAGAATGATA CTTTGATAAG TCGATCTGGA CGCCACAAAG ATCAATCTAG GATTTCGACA   
  
  
+ CCTATAAAAT ATTTGGATTA GTGTTTTGTG TTTTTTAAAA AACTTTTTTT TGTTTAGTTT TTTTTTCATT   
  
  
+ TCACCGTTCA ACAACTCAAT CTTTTTCTTC TTTTTATAAT TTTTTTTCTA TTTCATCTTT AAAAATTAGG   
  
  
+ TTGTTTTGAA AATTTTGCTT CATATTTTTT TTTTCTGTTG GGTTATATAT TAGTCTCATG GATTTAATTT   
  
  
+ TATTTTCTCG ATTTCAACCT TCAACACTAG ATCTATTGGA AGTGGGGTTT CATAATATTT TTTTTATTTA   
  
  
+ TTTTCTATGA ATTTTTCTTG ATCTCATGAC TAGGTCACAA GTTTAACAAG TTAACTCAAG TTGACTGAGA   
  
  
+ TCATTTTCTT ATTGCTTTTT TTAATTGATT TTTTTATTTC ATCCTTCAAT ATTGGGTTGG ATGGAAATTG   
  
  
+ AGATTCATAT ATTTTTTCTA TTTACTTTCT ATGGGGTTAT CCTGATCTCA TGTGAGTTTA GCAGGTTAAG   
  
  
+ TCGGGTTGAC TTTATTTATT TATTTTTCTT CTTTTTTAAT TGGACTTTGG AATTTTTTTT ATTTTATTTT   
  
  
+ TATGGGATTA TCCAGATCTT ATGACTCAAG TCACGAGTTT AGTTGGTTGA CCTGAGTTGA CTTGATTTAT   
  
  
+ TATTTGGGTT CCTTTTTTAA TTGAATGTTT TTTTTTCTAT TTCACCTTCC AACAACTCAA TTGTTTTATT   
  
  
+ TCACGTTTTT TTTCTTCAGT TTCATCCTTC AATATTAAGT TGTTTGGGAA TTAGATTTTG AAATTTCTTT   
  
  
+ TTCAATTTGG TTTTTGTGGA GTTAACCCGA TCTAATATAT TTAATTTTTT TTCTTGATTT TAATCTTTTA   
  
  
+ CATTGGATCT GTTAGAAAAT GGATCTTTGT AATTTTTTAT TTGATTTTTT TGAAGTTATC TCAGTCTTAT   
  
  
+ GATCTAGGTC ACGAGCTTAA CATATTAGCC TAAGTTAACT TGAGTTGTTT TTTTTAGTTG ATTTTTTTTA   
  
  
+ TTTCATCATT CAACATTAAG TTAGTTGTAA ATTAGGTTTC ATAATTTTTT TATATATGCT TTCTATTGAG   
  
  
+ TTATAAAATA TCCCGATCTC ATGATCCGGG TTGCGAGTTT AACATCTTAA CTTACATTAA CTTGGGTAGT   
  
  
+ TTTTTTTATC CTTTTTTTAA TTTAATATTT TTTTTTCAAT TCCACCCTTC AATATCAAAT TGATTAGAGA   
  
  
+ TTGATCTTCA TAATTTATTT TGATTTACTT TCTATATATA AGGTTATTAT GGTTTCTTGA TTTAAGTTTC   
  
  
+ AAATTTGACA GATTAACTTG TATCAATCTT AGATGTTTTT ATTTTAATAT TTAAAAAATA CATCCAATAT   
  
  
+ ACTTTATGTT TTGAATTTAT AATTAATTTT TTTATTTGAA AATATGTTAA CATCGTAGGT ATATTTTTTT   
  
  
+ ATATTAAAAA ATTAATTGGA TTCAGAGTCA TAAAAATAAT CAGGTTTTCT CTAAAAATCG GTATGCATCA   
  
  
+ CTTTCAAAAC ATGAACGCTC ACATTCTCCC AACGGAGGAA AAATGCCTCT AGATGTTGTC AAAAGAAGAC   
  
  
+ GCCTTTATAA TTACAATTTT TTATACCCAT TTTCCATATC CCAAAAGGCT GATGATTATG GCATTTTCGG   
  
  
+ ATTTTTAAAA AAAAGAAAAA AAATGATAAA TACAGAGGTG TTTAGCATTG AAAAGAAAAA TAAATTAAAG   
  
  
+ GACCTGCCGT CAATAAAATT TGGCAGCAAA GAAGAATATA ATTGACCAGT CAATGAGGAC CTGATAACTA   
  
  
+ TAAAAAAAGA GGCGCATGGA CGGGCGCACC CACATGTTA  

- ACTATGGTCT ACTACACAGC ACTTACTAAA CTTTTAGCTT TCGTTACTTA GACTGCTTTT ACCTACTTCC   
  
  
- TACCCAGACC AATCAACAGA AAGAAAACCA CAAATCCGAC CTAATATCAC TAAAATTTCA TTTATTCTAC   
  
  
- CTCCTATCTG ATCTTACTAT GAAACTATTC AGCTAGACCT GCGGTGTTTC TAGTTAGATC CTAAAGCTGT   
  
  
- GGATATTTTA TAAACCTAAT CACAAAACAC AAAAAATTTT TTGAAAAAAA ACAAATCAAA AAAAAAGTAA   
  
  
- AGTGGCAAGT TGTTGAGTTA GAAAAAGAAG AAAAATATTA AAAAAAAGAT AAAGTAGAAA TTTTTAATCC   
  
  
- AACAAAACTT TTAAAACGAA GTATAAAAAA AAAAGACAAC CCAATATATA ATCAGAGTAC CTAAATTAAA   
  
  
- ATAAAAGAGC TAAAGTTGGA AGTTGTGATC TAGATAACCT TCACCCCAAA GTATTATAAA AAAAATAAAT   
  
  
- AAAAGATACT TAAAAAGAAC TAGAGTACTG ATCCAGTGTT CAAATTGTTC AATTGAGTTC AACTGACTCT   
  
  
- AGTAAAAGAA TAACGAAAAA AATTAACTAA AAAAATAAAG TAGGAAGTTA TAACCCAACC TACCTTTAAC   
  
  
- TCTAAGTATA TAAAAAAGAT AAATGAAAGA TACCCCAATA GGACTAGAGT ACACTCAAAT CGTCCAATTC   
  
  
- AGCCCAACTG AAATAAATAA ATAAAAAGAA GAAAAAATTA ACCTGAAACC TTAAAAAAAA TAAAATAAAA   
  
  
- ATACCCTAAT AGGTCTAGAA TACTGAGTTC AGTGCTCAAA TCAACCAACT GGACTCAACT GAACTAAATA   
  
  
- ATAAACCCAA GGAAAAAATT AACTTACAAA AAAAAAGATA AAGTGGAAGG TTGTTGAGTT AACAAAATAA   
  
  
- AGTGCAAAAA AAAGAAGTCA AAGTAGGAAG TTATAATTCA ACAAACCCTT AATCTAAAAC TTTAAAGAAA   
  
  
- AAGTTAAACC AAAAACACCT CAATTGGGCT AGATTATATA AATTAAAAAA AAGAACTAAA ATTAGAAAAT   
  
  
- GTAACCTAGA CAATCTTTTA CCTAGAAACA TTAAAAAATA AACTAAAAAA ACTTCAATAG AGTCAGAATA   
  
  
- CTAGATCCAG TGCTCGAATT GTATAATCGG ATTCAATTGA ACTCAACAAA AAAAATCAAC TAAAAAAAAT   
  
  
- AAAGTAGTAA GTTGTAATTC AATCAACATT TAATCCAAAG TATTAAAAAA ATATATACGA AAGATAACTC   
  
  
- AATATTTTAT AGGGCTAGAG TACTAGGCCC AACGCTCAAA TTGTAGAATT GAATGTAATT GAACCCATCA   
  
  
- AAAAAAATAG GAAAAAAATT AAATTATAAA AAAAAAGTTA AGGTGGGAAG TTATAGTTTA ACTAATCTCT   
  
  
- AACTAGAAGT ATTAAATAAA ACTAAATGAA AGATATATAT TCCAATAATA CCAAAGAACT AAATTCAAAG   
  
  
- TTTAAACTGT CTAATTGAAC ATAGTTAGAA TCTACAAAAA TAAAATTATA AATTTTTTAT GTAGGTTATA   
  
  
- TGAAATACAA AACTTAAATA TTAATTAAAA AAATAAACTT TTATACAATT GTAGCATCCA TATAAAAAAA   
  
  
- TATAATTTTT TAATTAACCT AAGTCTCAGT ATTTTTATTA GTCCAAAAGA GATTTTTAGC CATACGTAGT   
  
  
- GAAAGTTTTG TACTTGCGAG TGTAAGAGGG TTGCCTCCTT TTTACGGAGA TCTACAACAG TTTTCTTCTG   
  
  
- CGGAAATATT AATGTTAAAA AATATGGGTA AAAGGTATAG GGTTTTCCGA CTACTAATAC CGTAAAAGCC   
  
  
- TAAAAATTTT TTTTCTTTTT TTTACTATTT ATGTCTCCAC AAATCGTAAC TTTTCTTTTT ATTTAATTTC   
  
  
- CTGGACGGCA GTTATTTTAA ACCGTCGTTT CTTCTTATAT TAACTGGTCA GTTACTCCTG GACTATTGAT   
  
  
- ATTTTTTTCT CCGCGTACCT GCCCGCGTGG GTGTACAAT

+     MRE

| Site Name | Organism | Position | Strand | Matrix score. | sequence | function |
| --- | --- | --- | --- | --- | --- | --- |
| MRE | Petroselinum crispum | 1222 | - | 7 | AACCTAA | MYB binding site involved in light responsiveness |
| MRE | Petroselinum crispum | 346 | - | 7 | AACCTAA | MYB binding site involved in light responsiveness |

>Potri.006G138900.1   
+ TGATACCAGA TGATGTGTCG TGAATGATTT GAAAATCGAA AGCAATGAAT CTGACGAAAA TGGATGAAGG   
  
  
+ ATGGGTCTGG TTAGTTGTCT TTCTTTTGGT GTTTAGGCTG GATTATAGTG ATTTTAAAGT AAATAAGATG   
  
  
+ GAGGATAGAC TAGAATGATA CTTTGATAAG TCGATCTGGA CGCCACAAAG ATCAATCTAG GATTTCGACA   
  
  
+ CCTATAAAAT ATTTGGATTA GTGTTTTGTG TTTTTTAAAA AACTTTTTTT TGTTTAGTTT TTTTTTCATT   
  
  
+ TCACCGTTCA ACAACTCAAT CTTTTTCTTC TTTTTATAAT TTTTTTTCTA TTTCATCTTT AAAAATTAGG   
  
  
+ TTGTTTTGAA AATTTTGCTT CATATTTTTT TTTTCTGTTG GGTTATATAT TAGTCTCATG GATTTAATTT   
  
  
+ TATTTTCTCG ATTTCAACCT TCAACACTAG ATCTATTGGA AGTGGGGTTT CATAATATTT TTTTTATTTA   
  
  
+ TTTTCTATGA ATTTTTCTTG ATCTCATGAC TAGGTCACAA GTTTAACAAG TTAACTCAAG TTGACTGAGA   
  
  
+ TCATTTTCTT ATTGCTTTTT TTAATTGATT TTTTTATTTC ATCCTTCAAT ATTGGGTTGG ATGGAAATTG   
  
  
+ AGATTCATAT ATTTTTTCTA TTTACTTTCT ATGGGGTTAT CCTGATCTCA TGTGAGTTTA GCAGGTTAAG   
  
  
+ TCGGGTTGAC TTTATTTATT TATTTTTCTT CTTTTTTAAT TGGACTTTGG AATTTTTTTT ATTTTATTTT   
  
  
+ TATGGGATTA TCCAGATCTT ATGACTCAAG TCACGAGTTT AGTTGGTTGA CCTGAGTTGA CTTGATTTAT   
  
  
+ TATTTGGGTT CCTTTTTTAA TTGAATGTTT TTTTTTCTAT TTCACCTTCC AACAACTCAA TTGTTTTATT   
  
  
+ TCACGTTTTT TTTCTTCAGT TTCATCCTTC AATATTAAGT TGTTTGGGAA TTAGATTTTG AAATTTCTTT   
  
  
+ TTCAATTTGG TTTTTGTGGA GTTAACCCGA TCTAATATAT TTAATTTTTT TTCTTGATTT TAATCTTTTA   
  
  
+ CATTGGATCT GTTAGAAAAT GGATCTTTGT AATTTTTTAT TTGATTTTTT TGAAGTTATC TCAGTCTTAT   
  
  
+ GATCTAGGTC ACGAGCTTAA CATATTAGCC TAAGTTAACT TGAGTTGTTT TTTTTAGTTG ATTTTTTTTA   
  
  
+ TTTCATCATT CAACATTAAG TTAGTTGTAA ATTAGGTTTC ATAATTTTTT TATATATGCT TTCTATTGAG   
  
  
+ TTATAAAATA TCCCGATCTC ATGATCCGGG TTGCGAGTTT AACATCTTAA CTTACATTAA CTTGGGTAGT   
  
  
+ TTTTTTTATC CTTTTTTTAA TTTAATATTT TTTTTTCAAT TCCACCCTTC AATATCAAAT TGATTAGAGA   
  
  
+ TTGATCTTCA TAATTTATTT TGATTTACTT TCTATATATA AGGTTATTAT GGTTTCTTGA TTTAAGTTTC   
  
  
+ AAATTTGACA GATTAACTTG TATCAATCTT AGATGTTTTT ATTTTAATAT TTAAAAAATA CATCCAATAT   
  
  
+ ACTTTATGTT TTGAATTTAT AATTAATTTT TTTATTTGAA AATATGTTAA CATCGTAGGT ATATTTTTTT   
  
  
+ ATATTAAAAA ATTAATTGGA TTCAGAGTCA TAAAAATAAT CAGGTTTTCT CTAAAAATCG GTATGCATCA   
  
  
+ CTTTCAAAAC ATGAACGCTC ACATTCTCCC AACGGAGGAA AAATGCCTCT AGATGTTGTC AAAAGAAGAC   
  
  
+ GCCTTTATAA TTACAATTTT TTATACCCAT TTTCCATATC CCAAAAGGCT GATGATTATG GCATTTTCGG   
  
  
+ ATTTTTAAAA AAAAGAAAAA AAATGATAAA TACAGAGGTG TTTAGCATTG AAAAGAAAAA TAAATTAAAG   
  
  
+ GACCTGCCGT CAATAAAATT TGGCAGCAAA GAAGAATATA ATTGACCAGT CAATGAGGAC CTGATAACTA   
  
  
+ TAAAAAAAGA GGCGCATGGA CGGGCGCACC CACATGTTA  

- ACTATGGTCT ACTACACAGC ACTTACTAAA CTTTTAGCTT TCGTTACTTA GACTGCTTTT ACCTACTTCC   
  
  
- TACCCAGACC AATCAACAGA AAGAAAACCA CAAATCCGAC CTAATATCAC TAAAATTTCA TTTATTCTAC   
  
  
- CTCCTATCTG ATCTTACTAT GAAACTATTC AGCTAGACCT GCGGTGTTTC TAGTTAGATC CTAAAGCTGT   
  
  
- GGATATTTTA TAAACCTAAT CACAAAACAC AAAAAATTTT TTGAAAAAAA ACAAATCAAA AAAAAAGTAA   
  
  
- AGTGGCAAGT TGTTGAGTTA GAAAAAGAAG AAAAATATTA AAAAAAAGAT AAAGTAGAAA TTTTTAATCC   
  
  
- AACAAAACTT TTAAAACGAA GTATAAAAAA AAAAGACAAC CCAATATATA ATCAGAGTAC CTAAATTAAA   
  
  
- ATAAAAGAGC TAAAGTTGGA AGTTGTGATC TAGATAACCT TCACCCCAAA GTATTATAAA AAAAATAAAT   
  
  
- AAAAGATACT TAAAAAGAAC TAGAGTACTG ATCCAGTGTT CAAATTGTTC AATTGAGTTC AACTGACTCT   
  
  
- AGTAAAAGAA TAACGAAAAA AATTAACTAA AAAAATAAAG TAGGAAGTTA TAACCCAACC TACCTTTAAC   
  
  
- TCTAAGTATA TAAAAAAGAT AAATGAAAGA TACCCCAATA GGACTAGAGT ACACTCAAAT CGTCCAATTC   
  
  
- AGCCCAACTG AAATAAATAA ATAAAAAGAA GAAAAAATTA ACCTGAAACC TTAAAAAAAA TAAAATAAAA   
  
  
- ATACCCTAAT AGGTCTAGAA TACTGAGTTC AGTGCTCAAA TCAACCAACT GGACTCAACT GAACTAAATA   
  
  
- ATAAACCCAA GGAAAAAATT AACTTACAAA AAAAAAGATA AAGTGGAAGG TTGTTGAGTT AACAAAATAA   
  
  
- AGTGCAAAAA AAAGAAGTCA AAGTAGGAAG TTATAATTCA ACAAACCCTT AATCTAAAAC TTTAAAGAAA   
  
  
- AAGTTAAACC AAAAACACCT CAATTGGGCT AGATTATATA AATTAAAAAA AAGAACTAAA ATTAGAAAAT   
  
  
- GTAACCTAGA CAATCTTTTA CCTAGAAACA TTAAAAAATA AACTAAAAAA ACTTCAATAG AGTCAGAATA   
  
  
- CTAGATCCAG TGCTCGAATT GTATAATCGG ATTCAATTGA ACTCAACAAA AAAAATCAAC TAAAAAAAAT   
  
  
- AAAGTAGTAA GTTGTAATTC AATCAACATT TAATCCAAAG TATTAAAAAA ATATATACGA AAGATAACTC   
  
  
- AATATTTTAT AGGGCTAGAG TACTAGGCCC AACGCTCAAA TTGTAGAATT GAATGTAATT GAACCCATCA   
  
  
- AAAAAAATAG GAAAAAAATT AAATTATAAA AAAAAAGTTA AGGTGGGAAG TTATAGTTTA ACTAATCTCT   
  
  
- AACTAGAAGT ATTAAATAAA ACTAAATGAA AGATATATAT TCCAATAATA CCAAAGAACT AAATTCAAAG   
  
  
- TTTAAACTGT CTAATTGAAC ATAGTTAGAA TCTACAAAAA TAAAATTATA AATTTTTTAT GTAGGTTATA   
  
  
- TGAAATACAA AACTTAAATA TTAATTAAAA AAATAAACTT TTATACAATT GTAGCATCCA TATAAAAAAA   
  
  
- TATAATTTTT TAATTAACCT AAGTCTCAGT ATTTTTATTA GTCCAAAAGA GATTTTTAGC CATACGTAGT   
  
  
- GAAAGTTTTG TACTTGCGAG TGTAAGAGGG TTGCCTCCTT TTTACGGAGA TCTACAACAG TTTTCTTCTG   
  
  
- CGGAAATATT AATGTTAAAA AATATGGGTA AAAGGTATAG GGTTTTCCGA CTACTAATAC CGTAAAAGCC   
  
  
- TAAAAATTTT TTTTCTTTTT TTTACTATTT ATGTCTCCAC AAATCGTAAC TTTTCTTTTT ATTTAATTTC   
  
  
- CTGGACGGCA GTTATTTTAA ACCGTCGTTT CTTCTTATAT TAACTGGTCA GTTACTCCTG GACTATTGAT   
  
  
- ATTTTTTTCT CCGCGTACCT GCCCGCGTGG GTGTACAAT

+     MYB

| Site Name | Organism | Position | Strand | Matrix score. | sequence | function |
| --- | --- | --- | --- | --- | --- | --- |
| MYB | Arabidopsis thaliana | 814 | - | 6 | CAACCA |  |
| MYB | Arabidopsis thaliana | 385 | - | 6 | CAACAG |  |
| MYB | Arabidopsis thaliana | 78 | - | 6 | TAACCA |  |

>Potri.006G138900.1   
+ TGATACCAGA TGATGTGTCG TGAATGATTT GAAAATCGAA AGCAATGAAT CTGACGAAAA TGGATGAAGG   
  
  
+ ATGGGTCTGG TTAGTTGTCT TTCTTTTGGT GTTTAGGCTG GATTATAGTG ATTTTAAAGT AAATAAGATG   
  
  
+ GAGGATAGAC TAGAATGATA CTTTGATAAG TCGATCTGGA CGCCACAAAG ATCAATCTAG GATTTCGACA   
  
  
+ CCTATAAAAT ATTTGGATTA GTGTTTTGTG TTTTTTAAAA AACTTTTTTT TGTTTAGTTT TTTTTTCATT   
  
  
+ TCACCGTTCA ACAACTCAAT CTTTTTCTTC TTTTTATAAT TTTTTTTCTA TTTCATCTTT AAAAATTAGG   
  
  
+ TTGTTTTGAA AATTTTGCTT CATATTTTTT TTTTCTGTTG GGTTATATAT TAGTCTCATG GATTTAATTT   
  
  
+ TATTTTCTCG ATTTCAACCT TCAACACTAG ATCTATTGGA AGTGGGGTTT CATAATATTT TTTTTATTTA   
  
  
+ TTTTCTATGA ATTTTTCTTG ATCTCATGAC TAGGTCACAA GTTTAACAAG TTAACTCAAG TTGACTGAGA   
  
  
+ TCATTTTCTT ATTGCTTTTT TTAATTGATT TTTTTATTTC ATCCTTCAAT ATTGGGTTGG ATGGAAATTG   
  
  
+ AGATTCATAT ATTTTTTCTA TTTACTTTCT ATGGGGTTAT CCTGATCTCA TGTGAGTTTA GCAGGTTAAG   
  
  
+ TCGGGTTGAC TTTATTTATT TATTTTTCTT CTTTTTTAAT TGGACTTTGG AATTTTTTTT ATTTTATTTT   
  
  
+ TATGGGATTA TCCAGATCTT ATGACTCAAG TCACGAGTTT AGTTGGTTGA CCTGAGTTGA CTTGATTTAT   
  
  
+ TATTTGGGTT CCTTTTTTAA TTGAATGTTT TTTTTTCTAT TTCACCTTCC AACAACTCAA TTGTTTTATT   
  
  
+ TCACGTTTTT TTTCTTCAGT TTCATCCTTC AATATTAAGT TGTTTGGGAA TTAGATTTTG AAATTTCTTT   
  
  
+ TTCAATTTGG TTTTTGTGGA GTTAACCCGA TCTAATATAT TTAATTTTTT TTCTTGATTT TAATCTTTTA   
  
  
+ CATTGGATCT GTTAGAAAAT GGATCTTTGT AATTTTTTAT TTGATTTTTT TGAAGTTATC TCAGTCTTAT   
  
  
+ GATCTAGGTC ACGAGCTTAA CATATTAGCC TAAGTTAACT TGAGTTGTTT TTTTTAGTTG ATTTTTTTTA   
  
  
+ TTTCATCATT CAACATTAAG TTAGTTGTAA ATTAGGTTTC ATAATTTTTT TATATATGCT TTCTATTGAG   
  
  
+ TTATAAAATA TCCCGATCTC ATGATCCGGG TTGCGAGTTT AACATCTTAA CTTACATTAA CTTGGGTAGT   
  
  
+ TTTTTTTATC CTTTTTTTAA TTTAATATTT TTTTTTCAAT TCCACCCTTC AATATCAAAT TGATTAGAGA   
  
  
+ TTGATCTTCA TAATTTATTT TGATTTACTT TCTATATATA AGGTTATTAT GGTTTCTTGA TTTAAGTTTC   
  
  
+ AAATTTGACA GATTAACTTG TATCAATCTT AGATGTTTTT ATTTTAATAT TTAAAAAATA CATCCAATAT   
  
  
+ ACTTTATGTT TTGAATTTAT AATTAATTTT TTTATTTGAA AATATGTTAA CATCGTAGGT ATATTTTTTT   
  
  
+ ATATTAAAAA ATTAATTGGA TTCAGAGTCA TAAAAATAAT CAGGTTTTCT CTAAAAATCG GTATGCATCA   
  
  
+ CTTTCAAAAC ATGAACGCTC ACATTCTCCC AACGGAGGAA AAATGCCTCT AGATGTTGTC AAAAGAAGAC   
  
  
+ GCCTTTATAA TTACAATTTT TTATACCCAT TTTCCATATC CCAAAAGGCT GATGATTATG GCATTTTCGG   
  
  
+ ATTTTTAAAA AAAAGAAAAA AAATGATAAA TACAGAGGTG TTTAGCATTG AAAAGAAAAA TAAATTAAAG   
  
  
+ GACCTGCCGT CAATAAAATT TGGCAGCAAA GAAGAATATA ATTGACCAGT CAATGAGGAC CTGATAACTA   
  
  
+ TAAAAAAAGA GGCGCATGGA CGGGCGCACC CACATGTTA  

- ACTATGGTCT ACTACACAGC ACTTACTAAA CTTTTAGCTT TCGTTACTTA GACTGCTTTT ACCTACTTCC   
  
  
- TACCCAGACC AATCAACAGA AAGAAAACCA CAAATCCGAC CTAATATCAC TAAAATTTCA TTTATTCTAC   
  
  
- CTCCTATCTG ATCTTACTAT GAAACTATTC AGCTAGACCT GCGGTGTTTC TAGTTAGATC CTAAAGCTGT   
  
  
- GGATATTTTA TAAACCTAAT CACAAAACAC AAAAAATTTT TTGAAAAAAA ACAAATCAAA AAAAAAGTAA   
  
  
- AGTGGCAAGT TGTTGAGTTA GAAAAAGAAG AAAAATATTA AAAAAAAGAT AAAGTAGAAA TTTTTAATCC   
  
  
- AACAAAACTT TTAAAACGAA GTATAAAAAA AAAAGACAAC CCAATATATA ATCAGAGTAC CTAAATTAAA   
  
  
- ATAAAAGAGC TAAAGTTGGA AGTTGTGATC TAGATAACCT TCACCCCAAA GTATTATAAA AAAAATAAAT   
  
  
- AAAAGATACT TAAAAAGAAC TAGAGTACTG ATCCAGTGTT CAAATTGTTC AATTGAGTTC AACTGACTCT   
  
  
- AGTAAAAGAA TAACGAAAAA AATTAACTAA AAAAATAAAG TAGGAAGTTA TAACCCAACC TACCTTTAAC   
  
  
- TCTAAGTATA TAAAAAAGAT AAATGAAAGA TACCCCAATA GGACTAGAGT ACACTCAAAT CGTCCAATTC   
  
  
- AGCCCAACTG AAATAAATAA ATAAAAAGAA GAAAAAATTA ACCTGAAACC TTAAAAAAAA TAAAATAAAA   
  
  
- ATACCCTAAT AGGTCTAGAA TACTGAGTTC AGTGCTCAAA TCAACCAACT GGACTCAACT GAACTAAATA   
  
  
- ATAAACCCAA GGAAAAAATT AACTTACAAA AAAAAAGATA AAGTGGAAGG TTGTTGAGTT AACAAAATAA   
  
  
- AGTGCAAAAA AAAGAAGTCA AAGTAGGAAG TTATAATTCA ACAAACCCTT AATCTAAAAC TTTAAAGAAA   
  
  
- AAGTTAAACC AAAAACACCT CAATTGGGCT AGATTATATA AATTAAAAAA AAGAACTAAA ATTAGAAAAT   
  
  
- GTAACCTAGA CAATCTTTTA CCTAGAAACA TTAAAAAATA AACTAAAAAA ACTTCAATAG AGTCAGAATA   
  
  
- CTAGATCCAG TGCTCGAATT GTATAATCGG ATTCAATTGA ACTCAACAAA AAAAATCAAC TAAAAAAAAT   
  
  
- AAAGTAGTAA GTTGTAATTC AATCAACATT TAATCCAAAG TATTAAAAAA ATATATACGA AAGATAACTC   
  
  
- AATATTTTAT AGGGCTAGAG TACTAGGCCC AACGCTCAAA TTGTAGAATT GAATGTAATT GAACCCATCA   
  
  
- AAAAAAATAG GAAAAAAATT AAATTATAAA AAAAAAGTTA AGGTGGGAAG TTATAGTTTA ACTAATCTCT   
  
  
- AACTAGAAGT ATTAAATAAA ACTAAATGAA AGATATATAT TCCAATAATA CCAAAGAACT AAATTCAAAG   
  
  
- TTTAAACTGT CTAATTGAAC ATAGTTAGAA TCTACAAAAA TAAAATTATA AATTTTTTAT GTAGGTTATA   
  
  
- TGAAATACAA AACTTAAATA TTAATTAAAA AAATAAACTT TTATACAATT GTAGCATCCA TATAAAAAAA   
  
  
- TATAATTTTT TAATTAACCT AAGTCTCAGT ATTTTTATTA GTCCAAAAGA GATTTTTAGC CATACGTAGT   
  
  
- GAAAGTTTTG TACTTGCGAG TGTAAGAGGG TTGCCTCCTT TTTACGGAGA TCTACAACAG TTTTCTTCTG   
  
  
- CGGAAATATT AATGTTAAAA AATATGGGTA AAAGGTATAG GGTTTTCCGA CTACTAATAC CGTAAAAGCC   
  
  
- TAAAAATTTT TTTTCTTTTT TTTACTATTT ATGTCTCCAC AAATCGTAAC TTTTCTTTTT ATTTAATTTC   
  
  
- CTGGACGGCA GTTATTTTAA ACCGTCGTTT CTTCTTATAT TAACTGGTCA GTTACTCCTG GACTATTGAT   
  
  
- ATTTTTTTCT CCGCGTACCT GCCCGCGTGG GTGTACAAT

+     MYB recognition site

| Site Name | Organism | Position | Strand | Matrix score. | sequence | function |
| --- | --- | --- | --- | --- | --- | --- |
| MYB recognition site | Arabidopsis thaliana | 1710 | - | 6 | CCGTTG |  |

>Potri.006G138900.1   
+ TGATACCAGA TGATGTGTCG TGAATGATTT GAAAATCGAA AGCAATGAAT CTGACGAAAA TGGATGAAGG   
  
  
+ ATGGGTCTGG TTAGTTGTCT TTCTTTTGGT GTTTAGGCTG GATTATAGTG ATTTTAAAGT AAATAAGATG   
  
  
+ GAGGATAGAC TAGAATGATA CTTTGATAAG TCGATCTGGA CGCCACAAAG ATCAATCTAG GATTTCGACA   
  
  
+ CCTATAAAAT ATTTGGATTA GTGTTTTGTG TTTTTTAAAA AACTTTTTTT TGTTTAGTTT TTTTTTCATT   
  
  
+ TCACCGTTCA ACAACTCAAT CTTTTTCTTC TTTTTATAAT TTTTTTTCTA TTTCATCTTT AAAAATTAGG   
  
  
+ TTGTTTTGAA AATTTTGCTT CATATTTTTT TTTTCTGTTG GGTTATATAT TAGTCTCATG GATTTAATTT   
  
  
+ TATTTTCTCG ATTTCAACCT TCAACACTAG ATCTATTGGA AGTGGGGTTT CATAATATTT TTTTTATTTA   
  
  
+ TTTTCTATGA ATTTTTCTTG ATCTCATGAC TAGGTCACAA GTTTAACAAG TTAACTCAAG TTGACTGAGA   
  
  
+ TCATTTTCTT ATTGCTTTTT TTAATTGATT TTTTTATTTC ATCCTTCAAT ATTGGGTTGG ATGGAAATTG   
  
  
+ AGATTCATAT ATTTTTTCTA TTTACTTTCT ATGGGGTTAT CCTGATCTCA TGTGAGTTTA GCAGGTTAAG   
  
  
+ TCGGGTTGAC TTTATTTATT TATTTTTCTT CTTTTTTAAT TGGACTTTGG AATTTTTTTT ATTTTATTTT   
  
  
+ TATGGGATTA TCCAGATCTT ATGACTCAAG TCACGAGTTT AGTTGGTTGA CCTGAGTTGA CTTGATTTAT   
  
  
+ TATTTGGGTT CCTTTTTTAA TTGAATGTTT TTTTTTCTAT TTCACCTTCC AACAACTCAA TTGTTTTATT   
  
  
+ TCACGTTTTT TTTCTTCAGT TTCATCCTTC AATATTAAGT TGTTTGGGAA TTAGATTTTG AAATTTCTTT   
  
  
+ TTCAATTTGG TTTTTGTGGA GTTAACCCGA TCTAATATAT TTAATTTTTT TTCTTGATTT TAATCTTTTA   
  
  
+ CATTGGATCT GTTAGAAAAT GGATCTTTGT AATTTTTTAT TTGATTTTTT TGAAGTTATC TCAGTCTTAT   
  
  
+ GATCTAGGTC ACGAGCTTAA CATATTAGCC TAAGTTAACT TGAGTTGTTT TTTTTAGTTG ATTTTTTTTA   
  
  
+ TTTCATCATT CAACATTAAG TTAGTTGTAA ATTAGGTTTC ATAATTTTTT TATATATGCT TTCTATTGAG   
  
  
+ TTATAAAATA TCCCGATCTC ATGATCCGGG TTGCGAGTTT AACATCTTAA CTTACATTAA CTTGGGTAGT   
  
  
+ TTTTTTTATC CTTTTTTTAA TTTAATATTT TTTTTTCAAT TCCACCCTTC AATATCAAAT TGATTAGAGA   
  
  
+ TTGATCTTCA TAATTTATTT TGATTTACTT TCTATATATA AGGTTATTAT GGTTTCTTGA TTTAAGTTTC   
  
  
+ AAATTTGACA GATTAACTTG TATCAATCTT AGATGTTTTT ATTTTAATAT TTAAAAAATA CATCCAATAT   
  
  
+ ACTTTATGTT TTGAATTTAT AATTAATTTT TTTATTTGAA AATATGTTAA CATCGTAGGT ATATTTTTTT   
  
  
+ ATATTAAAAA ATTAATTGGA TTCAGAGTCA TAAAAATAAT CAGGTTTTCT CTAAAAATCG GTATGCATCA   
  
  
+ CTTTCAAAAC ATGAACGCTC ACATTCTCCC AACGGAGGAA AAATGCCTCT AGATGTTGTC AAAAGAAGAC   
  
  
+ GCCTTTATAA TTACAATTTT TTATACCCAT TTTCCATATC CCAAAAGGCT GATGATTATG GCATTTTCGG   
  
  
+ ATTTTTAAAA AAAAGAAAAA AAATGATAAA TACAGAGGTG TTTAGCATTG AAAAGAAAAA TAAATTAAAG   
  
  
+ GACCTGCCGT CAATAAAATT TGGCAGCAAA GAAGAATATA ATTGACCAGT CAATGAGGAC CTGATAACTA   
  
  
+ TAAAAAAAGA GGCGCATGGA CGGGCGCACC CACATGTTA  

- ACTATGGTCT ACTACACAGC ACTTACTAAA CTTTTAGCTT TCGTTACTTA GACTGCTTTT ACCTACTTCC   
  
  
- TACCCAGACC AATCAACAGA AAGAAAACCA CAAATCCGAC CTAATATCAC TAAAATTTCA TTTATTCTAC   
  
  
- CTCCTATCTG ATCTTACTAT GAAACTATTC AGCTAGACCT GCGGTGTTTC TAGTTAGATC CTAAAGCTGT   
  
  
- GGATATTTTA TAAACCTAAT CACAAAACAC AAAAAATTTT TTGAAAAAAA ACAAATCAAA AAAAAAGTAA   
  
  
- AGTGGCAAGT TGTTGAGTTA GAAAAAGAAG AAAAATATTA AAAAAAAGAT AAAGTAGAAA TTTTTAATCC   
  
  
- AACAAAACTT TTAAAACGAA GTATAAAAAA AAAAGACAAC CCAATATATA ATCAGAGTAC CTAAATTAAA   
  
  
- ATAAAAGAGC TAAAGTTGGA AGTTGTGATC TAGATAACCT TCACCCCAAA GTATTATAAA AAAAATAAAT   
  
  
- AAAAGATACT TAAAAAGAAC TAGAGTACTG ATCCAGTGTT CAAATTGTTC AATTGAGTTC AACTGACTCT   
  
  
- AGTAAAAGAA TAACGAAAAA AATTAACTAA AAAAATAAAG TAGGAAGTTA TAACCCAACC TACCTTTAAC   
  
  
- TCTAAGTATA TAAAAAAGAT AAATGAAAGA TACCCCAATA GGACTAGAGT ACACTCAAAT CGTCCAATTC   
  
  
- AGCCCAACTG AAATAAATAA ATAAAAAGAA GAAAAAATTA ACCTGAAACC TTAAAAAAAA TAAAATAAAA   
  
  
- ATACCCTAAT AGGTCTAGAA TACTGAGTTC AGTGCTCAAA TCAACCAACT GGACTCAACT GAACTAAATA   
  
  
- ATAAACCCAA GGAAAAAATT AACTTACAAA AAAAAAGATA AAGTGGAAGG TTGTTGAGTT AACAAAATAA   
  
  
- AGTGCAAAAA AAAGAAGTCA AAGTAGGAAG TTATAATTCA ACAAACCCTT AATCTAAAAC TTTAAAGAAA   
  
  
- AAGTTAAACC AAAAACACCT CAATTGGGCT AGATTATATA AATTAAAAAA AAGAACTAAA ATTAGAAAAT   
  
  
- GTAACCTAGA CAATCTTTTA CCTAGAAACA TTAAAAAATA AACTAAAAAA ACTTCAATAG AGTCAGAATA   
  
  
- CTAGATCCAG TGCTCGAATT GTATAATCGG ATTCAATTGA ACTCAACAAA AAAAATCAAC TAAAAAAAAT   
  
  
- AAAGTAGTAA GTTGTAATTC AATCAACATT TAATCCAAAG TATTAAAAAA ATATATACGA AAGATAACTC   
  
  
- AATATTTTAT AGGGCTAGAG TACTAGGCCC AACGCTCAAA TTGTAGAATT GAATGTAATT GAACCCATCA   
  
  
- AAAAAAATAG GAAAAAAATT AAATTATAAA AAAAAAGTTA AGGTGGGAAG TTATAGTTTA ACTAATCTCT   
  
  
- AACTAGAAGT ATTAAATAAA ACTAAATGAA AGATATATAT TCCAATAATA CCAAAGAACT AAATTCAAAG   
  
  
- TTTAAACTGT CTAATTGAAC ATAGTTAGAA TCTACAAAAA TAAAATTATA AATTTTTTAT GTAGGTTATA   
  
  
- TGAAATACAA AACTTAAATA TTAATTAAAA AAATAAACTT TTATACAATT GTAGCATCCA TATAAAAAAA   
  
  
- TATAATTTTT TAATTAACCT AAGTCTCAGT ATTTTTATTA GTCCAAAAGA GATTTTTAGC CATACGTAGT   
  
  
- GAAAGTTTTG TACTTGCGAG TGTAAGAGGG TTGCCTCCTT TTTACGGAGA TCTACAACAG TTTTCTTCTG   
  
  
- CGGAAATATT AATGTTAAAA AATATGGGTA AAAGGTATAG GGTTTTCCGA CTACTAATAC CGTAAAAGCC   
  
  
- TAAAAATTTT TTTTCTTTTT TTTACTATTT ATGTCTCCAC AAATCGTAAC TTTTCTTTTT ATTTAATTTC   
  
  
- CTGGACGGCA GTTATTTTAA ACCGTCGTTT CTTCTTATAT TAACTGGTCA GTTACTCCTG GACTATTGAT   
  
  
- ATTTTTTTCT CCGCGTACCT GCCCGCGTGG GTGTACAAT

+     MYB-like sequence

| Site Name | Organism | Position | Strand | Matrix score. | sequence | function |
| --- | --- | --- | --- | --- | --- | --- |
| MYB-like sequence | Arabidopsis thaliana | 78 | - | 6 | TAACCA |  |

>Potri.006G138900.1   
+ TGATACCAGA TGATGTGTCG TGAATGATTT GAAAATCGAA AGCAATGAAT CTGACGAAAA TGGATGAAGG   
  
  
+ ATGGGTCTGG TTAGTTGTCT TTCTTTTGGT GTTTAGGCTG GATTATAGTG ATTTTAAAGT AAATAAGATG   
  
  
+ GAGGATAGAC TAGAATGATA CTTTGATAAG TCGATCTGGA CGCCACAAAG ATCAATCTAG GATTTCGACA   
  
  
+ CCTATAAAAT ATTTGGATTA GTGTTTTGTG TTTTTTAAAA AACTTTTTTT TGTTTAGTTT TTTTTTCATT   
  
  
+ TCACCGTTCA ACAACTCAAT CTTTTTCTTC TTTTTATAAT TTTTTTTCTA TTTCATCTTT AAAAATTAGG   
  
  
+ TTGTTTTGAA AATTTTGCTT CATATTTTTT TTTTCTGTTG GGTTATATAT TAGTCTCATG GATTTAATTT   
  
  
+ TATTTTCTCG ATTTCAACCT TCAACACTAG ATCTATTGGA AGTGGGGTTT CATAATATTT TTTTTATTTA   
  
  
+ TTTTCTATGA ATTTTTCTTG ATCTCATGAC TAGGTCACAA GTTTAACAAG TTAACTCAAG TTGACTGAGA   
  
  
+ TCATTTTCTT ATTGCTTTTT TTAATTGATT TTTTTATTTC ATCCTTCAAT ATTGGGTTGG ATGGAAATTG   
  
  
+ AGATTCATAT ATTTTTTCTA TTTACTTTCT ATGGGGTTAT CCTGATCTCA TGTGAGTTTA GCAGGTTAAG   
  
  
+ TCGGGTTGAC TTTATTTATT TATTTTTCTT CTTTTTTAAT TGGACTTTGG AATTTTTTTT ATTTTATTTT   
  
  
+ TATGGGATTA TCCAGATCTT ATGACTCAAG TCACGAGTTT AGTTGGTTGA CCTGAGTTGA CTTGATTTAT   
  
  
+ TATTTGGGTT CCTTTTTTAA TTGAATGTTT TTTTTTCTAT TTCACCTTCC AACAACTCAA TTGTTTTATT   
  
  
+ TCACGTTTTT TTTCTTCAGT TTCATCCTTC AATATTAAGT TGTTTGGGAA TTAGATTTTG AAATTTCTTT   
  
  
+ TTCAATTTGG TTTTTGTGGA GTTAACCCGA TCTAATATAT TTAATTTTTT TTCTTGATTT TAATCTTTTA   
  
  
+ CATTGGATCT GTTAGAAAAT GGATCTTTGT AATTTTTTAT TTGATTTTTT TGAAGTTATC TCAGTCTTAT   
  
  
+ GATCTAGGTC ACGAGCTTAA CATATTAGCC TAAGTTAACT TGAGTTGTTT TTTTTAGTTG ATTTTTTTTA   
  
  
+ TTTCATCATT CAACATTAAG TTAGTTGTAA ATTAGGTTTC ATAATTTTTT TATATATGCT TTCTATTGAG   
  
  
+ TTATAAAATA TCCCGATCTC ATGATCCGGG TTGCGAGTTT AACATCTTAA CTTACATTAA CTTGGGTAGT   
  
  
+ TTTTTTTATC CTTTTTTTAA TTTAATATTT TTTTTTCAAT TCCACCCTTC AATATCAAAT TGATTAGAGA   
  
  
+ TTGATCTTCA TAATTTATTT TGATTTACTT TCTATATATA AGGTTATTAT GGTTTCTTGA TTTAAGTTTC   
  
  
+ AAATTTGACA GATTAACTTG TATCAATCTT AGATGTTTTT ATTTTAATAT TTAAAAAATA CATCCAATAT   
  
  
+ ACTTTATGTT TTGAATTTAT AATTAATTTT TTTATTTGAA AATATGTTAA CATCGTAGGT ATATTTTTTT   
  
  
+ ATATTAAAAA ATTAATTGGA TTCAGAGTCA TAAAAATAAT CAGGTTTTCT CTAAAAATCG GTATGCATCA   
  
  
+ CTTTCAAAAC ATGAACGCTC ACATTCTCCC AACGGAGGAA AAATGCCTCT AGATGTTGTC AAAAGAAGAC   
  
  
+ GCCTTTATAA TTACAATTTT TTATACCCAT TTTCCATATC CCAAAAGGCT GATGATTATG GCATTTTCGG   
  
  
+ ATTTTTAAAA AAAAGAAAAA AAATGATAAA TACAGAGGTG TTTAGCATTG AAAAGAAAAA TAAATTAAAG   
  
  
+ GACCTGCCGT CAATAAAATT TGGCAGCAAA GAAGAATATA ATTGACCAGT CAATGAGGAC CTGATAACTA   
  
  
+ TAAAAAAAGA GGCGCATGGA CGGGCGCACC CACATGTTA  

- ACTATGGTCT ACTACACAGC ACTTACTAAA CTTTTAGCTT TCGTTACTTA GACTGCTTTT ACCTACTTCC   
  
  
- TACCCAGACC AATCAACAGA AAGAAAACCA CAAATCCGAC CTAATATCAC TAAAATTTCA TTTATTCTAC   
  
  
- CTCCTATCTG ATCTTACTAT GAAACTATTC AGCTAGACCT GCGGTGTTTC TAGTTAGATC CTAAAGCTGT   
  
  
- GGATATTTTA TAAACCTAAT CACAAAACAC AAAAAATTTT TTGAAAAAAA ACAAATCAAA AAAAAAGTAA   
  
  
- AGTGGCAAGT TGTTGAGTTA GAAAAAGAAG AAAAATATTA AAAAAAAGAT AAAGTAGAAA TTTTTAATCC   
  
  
- AACAAAACTT TTAAAACGAA GTATAAAAAA AAAAGACAAC CCAATATATA ATCAGAGTAC CTAAATTAAA   
  
  
- ATAAAAGAGC TAAAGTTGGA AGTTGTGATC TAGATAACCT TCACCCCAAA GTATTATAAA AAAAATAAAT   
  
  
- AAAAGATACT TAAAAAGAAC TAGAGTACTG ATCCAGTGTT CAAATTGTTC AATTGAGTTC AACTGACTCT   
  
  
- AGTAAAAGAA TAACGAAAAA AATTAACTAA AAAAATAAAG TAGGAAGTTA TAACCCAACC TACCTTTAAC   
  
  
- TCTAAGTATA TAAAAAAGAT AAATGAAAGA TACCCCAATA GGACTAGAGT ACACTCAAAT CGTCCAATTC   
  
  
- AGCCCAACTG AAATAAATAA ATAAAAAGAA GAAAAAATTA ACCTGAAACC TTAAAAAAAA TAAAATAAAA   
  
  
- ATACCCTAAT AGGTCTAGAA TACTGAGTTC AGTGCTCAAA TCAACCAACT GGACTCAACT GAACTAAATA   
  
  
- ATAAACCCAA GGAAAAAATT AACTTACAAA AAAAAAGATA AAGTGGAAGG TTGTTGAGTT AACAAAATAA   
  
  
- AGTGCAAAAA AAAGAAGTCA AAGTAGGAAG TTATAATTCA ACAAACCCTT AATCTAAAAC TTTAAAGAAA   
  
  
- AAGTTAAACC AAAAACACCT CAATTGGGCT AGATTATATA AATTAAAAAA AAGAACTAAA ATTAGAAAAT   
  
  
- GTAACCTAGA CAATCTTTTA CCTAGAAACA TTAAAAAATA AACTAAAAAA ACTTCAATAG AGTCAGAATA   
  
  
- CTAGATCCAG TGCTCGAATT GTATAATCGG ATTCAATTGA ACTCAACAAA AAAAATCAAC TAAAAAAAAT   
  
  
- AAAGTAGTAA GTTGTAATTC AATCAACATT TAATCCAAAG TATTAAAAAA ATATATACGA AAGATAACTC   
  
  
- AATATTTTAT AGGGCTAGAG TACTAGGCCC AACGCTCAAA TTGTAGAATT GAATGTAATT GAACCCATCA   
  
  
- AAAAAAATAG GAAAAAAATT AAATTATAAA AAAAAAGTTA AGGTGGGAAG TTATAGTTTA ACTAATCTCT   
  
  
- AACTAGAAGT ATTAAATAAA ACTAAATGAA AGATATATAT TCCAATAATA CCAAAGAACT AAATTCAAAG   
  
  
- TTTAAACTGT CTAATTGAAC ATAGTTAGAA TCTACAAAAA TAAAATTATA AATTTTTTAT GTAGGTTATA   
  
  
- TGAAATACAA AACTTAAATA TTAATTAAAA AAATAAACTT TTATACAATT GTAGCATCCA TATAAAAAAA   
  
  
- TATAATTTTT TAATTAACCT AAGTCTCAGT ATTTTTATTA GTCCAAAAGA GATTTTTAGC CATACGTAGT   
  
  
- GAAAGTTTTG TACTTGCGAG TGTAAGAGGG TTGCCTCCTT TTTACGGAGA TCTACAACAG TTTTCTTCTG   
  
  
- CGGAAATATT AATGTTAAAA AATATGGGTA AAAGGTATAG GGTTTTCCGA CTACTAATAC CGTAAAAGCC   
  
  
- TAAAAATTTT TTTTCTTTTT TTTACTATTT ATGTCTCCAC AAATCGTAAC TTTTCTTTTT ATTTAATTTC   
  
  
- CTGGACGGCA GTTATTTTAA ACCGTCGTTT CTTCTTATAT TAACTGGTCA GTTACTCCTG GACTATTGAT   
  
  
- ATTTTTTTCT CCGCGTACCT GCCCGCGTGG GTGTACAAT

+     MYC

| Site Name | Organism | Position | Strand | Matrix score. | sequence | function |
| --- | --- | --- | --- | --- | --- | --- |
| MYC | Arabidopsis thaliana | 898 | + | 6 | CAATTG |  |
| MYC | Arabidopsis thaliana | 1991 | - | 6 | CATGTG |  |
| MYC | Arabidopsis thaliana | 679 | + | 6 | CATGTG |  |

>Potri.006G138900.1   
+ TGATACCAGA TGATGTGTCG TGAATGATTT GAAAATCGAA AGCAATGAAT CTGACGAAAA TGGATGAAGG   
  
  
+ ATGGGTCTGG TTAGTTGTCT TTCTTTTGGT GTTTAGGCTG GATTATAGTG ATTTTAAAGT AAATAAGATG   
  
  
+ GAGGATAGAC TAGAATGATA CTTTGATAAG TCGATCTGGA CGCCACAAAG ATCAATCTAG GATTTCGACA   
  
  
+ CCTATAAAAT ATTTGGATTA GTGTTTTGTG TTTTTTAAAA AACTTTTTTT TGTTTAGTTT TTTTTTCATT   
  
  
+ TCACCGTTCA ACAACTCAAT CTTTTTCTTC TTTTTATAAT TTTTTTTCTA TTTCATCTTT AAAAATTAGG   
  
  
+ TTGTTTTGAA AATTTTGCTT CATATTTTTT TTTTCTGTTG GGTTATATAT TAGTCTCATG GATTTAATTT   
  
  
+ TATTTTCTCG ATTTCAACCT TCAACACTAG ATCTATTGGA AGTGGGGTTT CATAATATTT TTTTTATTTA   
  
  
+ TTTTCTATGA ATTTTTCTTG ATCTCATGAC TAGGTCACAA GTTTAACAAG TTAACTCAAG TTGACTGAGA   
  
  
+ TCATTTTCTT ATTGCTTTTT TTAATTGATT TTTTTATTTC ATCCTTCAAT ATTGGGTTGG ATGGAAATTG   
  
  
+ AGATTCATAT ATTTTTTCTA TTTACTTTCT ATGGGGTTAT CCTGATCTCA TGTGAGTTTA GCAGGTTAAG   
  
  
+ TCGGGTTGAC TTTATTTATT TATTTTTCTT CTTTTTTAAT TGGACTTTGG AATTTTTTTT ATTTTATTTT   
  
  
+ TATGGGATTA TCCAGATCTT ATGACTCAAG TCACGAGTTT AGTTGGTTGA CCTGAGTTGA CTTGATTTAT   
  
  
+ TATTTGGGTT CCTTTTTTAA TTGAATGTTT TTTTTTCTAT TTCACCTTCC AACAACTCAA TTGTTTTATT   
  
  
+ TCACGTTTTT TTTCTTCAGT TTCATCCTTC AATATTAAGT TGTTTGGGAA TTAGATTTTG AAATTTCTTT   
  
  
+ TTCAATTTGG TTTTTGTGGA GTTAACCCGA TCTAATATAT TTAATTTTTT TTCTTGATTT TAATCTTTTA   
  
  
+ CATTGGATCT GTTAGAAAAT GGATCTTTGT AATTTTTTAT TTGATTTTTT TGAAGTTATC TCAGTCTTAT   
  
  
+ GATCTAGGTC ACGAGCTTAA CATATTAGCC TAAGTTAACT TGAGTTGTTT TTTTTAGTTG ATTTTTTTTA   
  
  
+ TTTCATCATT CAACATTAAG TTAGTTGTAA ATTAGGTTTC ATAATTTTTT TATATATGCT TTCTATTGAG   
  
  
+ TTATAAAATA TCCCGATCTC ATGATCCGGG TTGCGAGTTT AACATCTTAA CTTACATTAA CTTGGGTAGT   
  
  
+ TTTTTTTATC CTTTTTTTAA TTTAATATTT TTTTTTCAAT TCCACCCTTC AATATCAAAT TGATTAGAGA   
  
  
+ TTGATCTTCA TAATTTATTT TGATTTACTT TCTATATATA AGGTTATTAT GGTTTCTTGA TTTAAGTTTC   
  
  
+ AAATTTGACA GATTAACTTG TATCAATCTT AGATGTTTTT ATTTTAATAT TTAAAAAATA CATCCAATAT   
  
  
+ ACTTTATGTT TTGAATTTAT AATTAATTTT TTTATTTGAA AATATGTTAA CATCGTAGGT ATATTTTTTT   
  
  
+ ATATTAAAAA ATTAATTGGA TTCAGAGTCA TAAAAATAAT CAGGTTTTCT CTAAAAATCG GTATGCATCA   
  
  
+ CTTTCAAAAC ATGAACGCTC ACATTCTCCC AACGGAGGAA AAATGCCTCT AGATGTTGTC AAAAGAAGAC   
  
  
+ GCCTTTATAA TTACAATTTT TTATACCCAT TTTCCATATC CCAAAAGGCT GATGATTATG GCATTTTCGG   
  
  
+ ATTTTTAAAA AAAAGAAAAA AAATGATAAA TACAGAGGTG TTTAGCATTG AAAAGAAAAA TAAATTAAAG   
  
  
+ GACCTGCCGT CAATAAAATT TGGCAGCAAA GAAGAATATA ATTGACCAGT CAATGAGGAC CTGATAACTA   
  
  
+ TAAAAAAAGA GGCGCATGGA CGGGCGCACC CACATGTTA  

- ACTATGGTCT ACTACACAGC ACTTACTAAA CTTTTAGCTT TCGTTACTTA GACTGCTTTT ACCTACTTCC   
  
  
- TACCCAGACC AATCAACAGA AAGAAAACCA CAAATCCGAC CTAATATCAC TAAAATTTCA TTTATTCTAC   
  
  
- CTCCTATCTG ATCTTACTAT GAAACTATTC AGCTAGACCT GCGGTGTTTC TAGTTAGATC CTAAAGCTGT   
  
  
- GGATATTTTA TAAACCTAAT CACAAAACAC AAAAAATTTT TTGAAAAAAA ACAAATCAAA AAAAAAGTAA   
  
  
- AGTGGCAAGT TGTTGAGTTA GAAAAAGAAG AAAAATATTA AAAAAAAGAT AAAGTAGAAA TTTTTAATCC   
  
  
- AACAAAACTT TTAAAACGAA GTATAAAAAA AAAAGACAAC CCAATATATA ATCAGAGTAC CTAAATTAAA   
  
  
- ATAAAAGAGC TAAAGTTGGA AGTTGTGATC TAGATAACCT TCACCCCAAA GTATTATAAA AAAAATAAAT   
  
  
- AAAAGATACT TAAAAAGAAC TAGAGTACTG ATCCAGTGTT CAAATTGTTC AATTGAGTTC AACTGACTCT   
  
  
- AGTAAAAGAA TAACGAAAAA AATTAACTAA AAAAATAAAG TAGGAAGTTA TAACCCAACC TACCTTTAAC   
  
  
- TCTAAGTATA TAAAAAAGAT AAATGAAAGA TACCCCAATA GGACTAGAGT ACACTCAAAT CGTCCAATTC   
  
  
- AGCCCAACTG AAATAAATAA ATAAAAAGAA GAAAAAATTA ACCTGAAACC TTAAAAAAAA TAAAATAAAA   
  
  
- ATACCCTAAT AGGTCTAGAA TACTGAGTTC AGTGCTCAAA TCAACCAACT GGACTCAACT GAACTAAATA   
  
  
- ATAAACCCAA GGAAAAAATT AACTTACAAA AAAAAAGATA AAGTGGAAGG TTGTTGAGTT AACAAAATAA   
  
  
- AGTGCAAAAA AAAGAAGTCA AAGTAGGAAG TTATAATTCA ACAAACCCTT AATCTAAAAC TTTAAAGAAA   
  
  
- AAGTTAAACC AAAAACACCT CAATTGGGCT AGATTATATA AATTAAAAAA AAGAACTAAA ATTAGAAAAT   
  
  
- GTAACCTAGA CAATCTTTTA CCTAGAAACA TTAAAAAATA AACTAAAAAA ACTTCAATAG AGTCAGAATA   
  
  
- CTAGATCCAG TGCTCGAATT GTATAATCGG ATTCAATTGA ACTCAACAAA AAAAATCAAC TAAAAAAAAT   
  
  
- AAAGTAGTAA GTTGTAATTC AATCAACATT TAATCCAAAG TATTAAAAAA ATATATACGA AAGATAACTC   
  
  
- AATATTTTAT AGGGCTAGAG TACTAGGCCC AACGCTCAAA TTGTAGAATT GAATGTAATT GAACCCATCA   
  
  
- AAAAAAATAG GAAAAAAATT AAATTATAAA AAAAAAGTTA AGGTGGGAAG TTATAGTTTA ACTAATCTCT   
  
  
- AACTAGAAGT ATTAAATAAA ACTAAATGAA AGATATATAT TCCAATAATA CCAAAGAACT AAATTCAAAG   
  
  
- TTTAAACTGT CTAATTGAAC ATAGTTAGAA TCTACAAAAA TAAAATTATA AATTTTTTAT GTAGGTTATA   
  
  
- TGAAATACAA AACTTAAATA TTAATTAAAA AAATAAACTT TTATACAATT GTAGCATCCA TATAAAAAAA   
  
  
- TATAATTTTT TAATTAACCT AAGTCTCAGT ATTTTTATTA GTCCAAAAGA GATTTTTAGC CATACGTAGT   
  
  
- GAAAGTTTTG TACTTGCGAG TGTAAGAGGG TTGCCTCCTT TTTACGGAGA TCTACAACAG TTTTCTTCTG   
  
  
- CGGAAATATT AATGTTAAAA AATATGGGTA AAAGGTATAG GGTTTTCCGA CTACTAATAC CGTAAAAGCC   
  
  
- TAAAAATTTT TTTTCTTTTT TTTACTATTT ATGTCTCCAC AAATCGTAAC TTTTCTTTTT ATTTAATTTC   
  
  
- CTGGACGGCA GTTATTTTAA ACCGTCGTTT CTTCTTATAT TAACTGGTCA GTTACTCCTG GACTATTGAT   
  
  
- ATTTTTTTCT CCGCGTACCT GCCCGCGTGG GTGTACAAT

+     Myb-binding site

| Site Name | Organism | Position | Strand | Matrix score. | sequence | function |
| --- | --- | --- | --- | --- | --- | --- |
| Myb-binding site | Nicotiana tabacum | 385 | - | 6 | CAACAG |  |

>Potri.006G138900.1   
+ TGATACCAGA TGATGTGTCG TGAATGATTT GAAAATCGAA AGCAATGAAT CTGACGAAAA TGGATGAAGG   
  
  
+ ATGGGTCTGG TTAGTTGTCT TTCTTTTGGT GTTTAGGCTG GATTATAGTG ATTTTAAAGT AAATAAGATG   
  
  
+ GAGGATAGAC TAGAATGATA CTTTGATAAG TCGATCTGGA CGCCACAAAG ATCAATCTAG GATTTCGACA   
  
  
+ CCTATAAAAT ATTTGGATTA GTGTTTTGTG TTTTTTAAAA AACTTTTTTT TGTTTAGTTT TTTTTTCATT   
  
  
+ TCACCGTTCA ACAACTCAAT CTTTTTCTTC TTTTTATAAT TTTTTTTCTA TTTCATCTTT AAAAATTAGG   
  
  
+ TTGTTTTGAA AATTTTGCTT CATATTTTTT TTTTCTGTTG GGTTATATAT TAGTCTCATG GATTTAATTT   
  
  
+ TATTTTCTCG ATTTCAACCT TCAACACTAG ATCTATTGGA AGTGGGGTTT CATAATATTT TTTTTATTTA   
  
  
+ TTTTCTATGA ATTTTTCTTG ATCTCATGAC TAGGTCACAA GTTTAACAAG TTAACTCAAG TTGACTGAGA   
  
  
+ TCATTTTCTT ATTGCTTTTT TTAATTGATT TTTTTATTTC ATCCTTCAAT ATTGGGTTGG ATGGAAATTG   
  
  
+ AGATTCATAT ATTTTTTCTA TTTACTTTCT ATGGGGTTAT CCTGATCTCA TGTGAGTTTA GCAGGTTAAG   
  
  
+ TCGGGTTGAC TTTATTTATT TATTTTTCTT CTTTTTTAAT TGGACTTTGG AATTTTTTTT ATTTTATTTT   
  
  
+ TATGGGATTA TCCAGATCTT ATGACTCAAG TCACGAGTTT AGTTGGTTGA CCTGAGTTGA CTTGATTTAT   
  
  
+ TATTTGGGTT CCTTTTTTAA TTGAATGTTT TTTTTTCTAT TTCACCTTCC AACAACTCAA TTGTTTTATT   
  
  
+ TCACGTTTTT TTTCTTCAGT TTCATCCTTC AATATTAAGT TGTTTGGGAA TTAGATTTTG AAATTTCTTT   
  
  
+ TTCAATTTGG TTTTTGTGGA GTTAACCCGA TCTAATATAT TTAATTTTTT TTCTTGATTT TAATCTTTTA   
  
  
+ CATTGGATCT GTTAGAAAAT GGATCTTTGT AATTTTTTAT TTGATTTTTT TGAAGTTATC TCAGTCTTAT   
  
  
+ GATCTAGGTC ACGAGCTTAA CATATTAGCC TAAGTTAACT TGAGTTGTTT TTTTTAGTTG ATTTTTTTTA   
  
  
+ TTTCATCATT CAACATTAAG TTAGTTGTAA ATTAGGTTTC ATAATTTTTT TATATATGCT TTCTATTGAG   
  
  
+ TTATAAAATA TCCCGATCTC ATGATCCGGG TTGCGAGTTT AACATCTTAA CTTACATTAA CTTGGGTAGT   
  
  
+ TTTTTTTATC CTTTTTTTAA TTTAATATTT TTTTTTCAAT TCCACCCTTC AATATCAAAT TGATTAGAGA   
  
  
+ TTGATCTTCA TAATTTATTT TGATTTACTT TCTATATATA AGGTTATTAT GGTTTCTTGA TTTAAGTTTC   
  
  
+ AAATTTGACA GATTAACTTG TATCAATCTT AGATGTTTTT ATTTTAATAT TTAAAAAATA CATCCAATAT   
  
  
+ ACTTTATGTT TTGAATTTAT AATTAATTTT TTTATTTGAA AATATGTTAA CATCGTAGGT ATATTTTTTT   
  
  
+ ATATTAAAAA ATTAATTGGA TTCAGAGTCA TAAAAATAAT CAGGTTTTCT CTAAAAATCG GTATGCATCA   
  
  
+ CTTTCAAAAC ATGAACGCTC ACATTCTCCC AACGGAGGAA AAATGCCTCT AGATGTTGTC AAAAGAAGAC   
  
  
+ GCCTTTATAA TTACAATTTT TTATACCCAT TTTCCATATC CCAAAAGGCT GATGATTATG GCATTTTCGG   
  
  
+ ATTTTTAAAA AAAAGAAAAA AAATGATAAA TACAGAGGTG TTTAGCATTG AAAAGAAAAA TAAATTAAAG   
  
  
+ GACCTGCCGT CAATAAAATT TGGCAGCAAA GAAGAATATA ATTGACCAGT CAATGAGGAC CTGATAACTA   
  
  
+ TAAAAAAAGA GGCGCATGGA CGGGCGCACC CACATGTTA  

- ACTATGGTCT ACTACACAGC ACTTACTAAA CTTTTAGCTT TCGTTACTTA GACTGCTTTT ACCTACTTCC   
  
  
- TACCCAGACC AATCAACAGA AAGAAAACCA CAAATCCGAC CTAATATCAC TAAAATTTCA TTTATTCTAC   
  
  
- CTCCTATCTG ATCTTACTAT GAAACTATTC AGCTAGACCT GCGGTGTTTC TAGTTAGATC CTAAAGCTGT   
  
  
- GGATATTTTA TAAACCTAAT CACAAAACAC AAAAAATTTT TTGAAAAAAA ACAAATCAAA AAAAAAGTAA   
  
  
- AGTGGCAAGT TGTTGAGTTA GAAAAAGAAG AAAAATATTA AAAAAAAGAT AAAGTAGAAA TTTTTAATCC   
  
  
- AACAAAACTT TTAAAACGAA GTATAAAAAA AAAAGACAAC CCAATATATA ATCAGAGTAC CTAAATTAAA   
  
  
- ATAAAAGAGC TAAAGTTGGA AGTTGTGATC TAGATAACCT TCACCCCAAA GTATTATAAA AAAAATAAAT   
  
  
- AAAAGATACT TAAAAAGAAC TAGAGTACTG ATCCAGTGTT CAAATTGTTC AATTGAGTTC AACTGACTCT   
  
  
- AGTAAAAGAA TAACGAAAAA AATTAACTAA AAAAATAAAG TAGGAAGTTA TAACCCAACC TACCTTTAAC   
  
  
- TCTAAGTATA TAAAAAAGAT AAATGAAAGA TACCCCAATA GGACTAGAGT ACACTCAAAT CGTCCAATTC   
  
  
- AGCCCAACTG AAATAAATAA ATAAAAAGAA GAAAAAATTA ACCTGAAACC TTAAAAAAAA TAAAATAAAA   
  
  
- ATACCCTAAT AGGTCTAGAA TACTGAGTTC AGTGCTCAAA TCAACCAACT GGACTCAACT GAACTAAATA   
  
  
- ATAAACCCAA GGAAAAAATT AACTTACAAA AAAAAAGATA AAGTGGAAGG TTGTTGAGTT AACAAAATAA   
  
  
- AGTGCAAAAA AAAGAAGTCA AAGTAGGAAG TTATAATTCA ACAAACCCTT AATCTAAAAC TTTAAAGAAA   
  
  
- AAGTTAAACC AAAAACACCT CAATTGGGCT AGATTATATA AATTAAAAAA AAGAACTAAA ATTAGAAAAT   
  
  
- GTAACCTAGA CAATCTTTTA CCTAGAAACA TTAAAAAATA AACTAAAAAA ACTTCAATAG AGTCAGAATA   
  
  
- CTAGATCCAG TGCTCGAATT GTATAATCGG ATTCAATTGA ACTCAACAAA AAAAATCAAC TAAAAAAAAT   
  
  
- AAAGTAGTAA GTTGTAATTC AATCAACATT TAATCCAAAG TATTAAAAAA ATATATACGA AAGATAACTC   
  
  
- AATATTTTAT AGGGCTAGAG TACTAGGCCC AACGCTCAAA TTGTAGAATT GAATGTAATT GAACCCATCA   
  
  
- AAAAAAATAG GAAAAAAATT AAATTATAAA AAAAAAGTTA AGGTGGGAAG TTATAGTTTA ACTAATCTCT   
  
  
- AACTAGAAGT ATTAAATAAA ACTAAATGAA AGATATATAT TCCAATAATA CCAAAGAACT AAATTCAAAG   
  
  
- TTTAAACTGT CTAATTGAAC ATAGTTAGAA TCTACAAAAA TAAAATTATA AATTTTTTAT GTAGGTTATA   
  
  
- TGAAATACAA AACTTAAATA TTAATTAAAA AAATAAACTT TTATACAATT GTAGCATCCA TATAAAAAAA   
  
  
- TATAATTTTT TAATTAACCT AAGTCTCAGT ATTTTTATTA GTCCAAAAGA GATTTTTAGC CATACGTAGT   
  
  
- GAAAGTTTTG TACTTGCGAG TGTAAGAGGG TTGCCTCCTT TTTACGGAGA TCTACAACAG TTTTCTTCTG   
  
  
- CGGAAATATT AATGTTAAAA AATATGGGTA AAAGGTATAG GGTTTTCCGA CTACTAATAC CGTAAAAGCC   
  
  
- TAAAAATTTT TTTTCTTTTT TTTACTATTT ATGTCTCCAC AAATCGTAAC TTTTCTTTTT ATTTAATTTC   
  
  
- CTGGACGGCA GTTATTTTAA ACCGTCGTTT CTTCTTATAT TAACTGGTCA GTTACTCCTG GACTATTGAT   
  
  
- ATTTTTTTCT CCGCGTACCT GCCCGCGTGG GTGTACAAT

+     O2-site

| Site Name | Organism | Position | Strand | Matrix score. | sequence | function |
| --- | --- | --- | --- | --- | --- | --- |
| O2-site | Zea mays | 9 | + | 9 | GATGATGTGG | cis-acting regulatory element involved in zein metabolism regulation |
| O2-site | Zea mays | 826 | + | 9 | GTTGACGTGA | cis-acting regulatory element involved in zein metabolism regulation |
| O2-site | Zea mays | 816 | + | 9 | GTTGACGTGA | cis-acting regulatory element involved in zein metabolism regulation |

>Potri.006G138900.1   
+ TGATACCAGA TGATGTGTCG TGAATGATTT GAAAATCGAA AGCAATGAAT CTGACGAAAA TGGATGAAGG   
  
  
+ ATGGGTCTGG TTAGTTGTCT TTCTTTTGGT GTTTAGGCTG GATTATAGTG ATTTTAAAGT AAATAAGATG   
  
  
+ GAGGATAGAC TAGAATGATA CTTTGATAAG TCGATCTGGA CGCCACAAAG ATCAATCTAG GATTTCGACA   
  
  
+ CCTATAAAAT ATTTGGATTA GTGTTTTGTG TTTTTTAAAA AACTTTTTTT TGTTTAGTTT TTTTTTCATT   
  
  
+ TCACCGTTCA ACAACTCAAT CTTTTTCTTC TTTTTATAAT TTTTTTTCTA TTTCATCTTT AAAAATTAGG   
  
  
+ TTGTTTTGAA AATTTTGCTT CATATTTTTT TTTTCTGTTG GGTTATATAT TAGTCTCATG GATTTAATTT   
  
  
+ TATTTTCTCG ATTTCAACCT TCAACACTAG ATCTATTGGA AGTGGGGTTT CATAATATTT TTTTTATTTA   
  
  
+ TTTTCTATGA ATTTTTCTTG ATCTCATGAC TAGGTCACAA GTTTAACAAG TTAACTCAAG TTGACTGAGA   
  
  
+ TCATTTTCTT ATTGCTTTTT TTAATTGATT TTTTTATTTC ATCCTTCAAT ATTGGGTTGG ATGGAAATTG   
  
  
+ AGATTCATAT ATTTTTTCTA TTTACTTTCT ATGGGGTTAT CCTGATCTCA TGTGAGTTTA GCAGGTTAAG   
  
  
+ TCGGGTTGAC TTTATTTATT TATTTTTCTT CTTTTTTAAT TGGACTTTGG AATTTTTTTT ATTTTATTTT   
  
  
+ TATGGGATTA TCCAGATCTT ATGACTCAAG TCACGAGTTT AGTTGGTTGA CCTGAGTTGA CTTGATTTAT   
  
  
+ TATTTGGGTT CCTTTTTTAA TTGAATGTTT TTTTTTCTAT TTCACCTTCC AACAACTCAA TTGTTTTATT   
  
  
+ TCACGTTTTT TTTCTTCAGT TTCATCCTTC AATATTAAGT TGTTTGGGAA TTAGATTTTG AAATTTCTTT   
  
  
+ TTCAATTTGG TTTTTGTGGA GTTAACCCGA TCTAATATAT TTAATTTTTT TTCTTGATTT TAATCTTTTA   
  
  
+ CATTGGATCT GTTAGAAAAT GGATCTTTGT AATTTTTTAT TTGATTTTTT TGAAGTTATC TCAGTCTTAT   
  
  
+ GATCTAGGTC ACGAGCTTAA CATATTAGCC TAAGTTAACT TGAGTTGTTT TTTTTAGTTG ATTTTTTTTA   
  
  
+ TTTCATCATT CAACATTAAG TTAGTTGTAA ATTAGGTTTC ATAATTTTTT TATATATGCT TTCTATTGAG   
  
  
+ TTATAAAATA TCCCGATCTC ATGATCCGGG TTGCGAGTTT AACATCTTAA CTTACATTAA CTTGGGTAGT   
  
  
+ TTTTTTTATC CTTTTTTTAA TTTAATATTT TTTTTTCAAT TCCACCCTTC AATATCAAAT TGATTAGAGA   
  
  
+ TTGATCTTCA TAATTTATTT TGATTTACTT TCTATATATA AGGTTATTAT GGTTTCTTGA TTTAAGTTTC   
  
  
+ AAATTTGACA GATTAACTTG TATCAATCTT AGATGTTTTT ATTTTAATAT TTAAAAAATA CATCCAATAT   
  
  
+ ACTTTATGTT TTGAATTTAT AATTAATTTT TTTATTTGAA AATATGTTAA CATCGTAGGT ATATTTTTTT   
  
  
+ ATATTAAAAA ATTAATTGGA TTCAGAGTCA TAAAAATAAT CAGGTTTTCT CTAAAAATCG GTATGCATCA   
  
  
+ CTTTCAAAAC ATGAACGCTC ACATTCTCCC AACGGAGGAA AAATGCCTCT AGATGTTGTC AAAAGAAGAC   
  
  
+ GCCTTTATAA TTACAATTTT TTATACCCAT TTTCCATATC CCAAAAGGCT GATGATTATG GCATTTTCGG   
  
  
+ ATTTTTAAAA AAAAGAAAAA AAATGATAAA TACAGAGGTG TTTAGCATTG AAAAGAAAAA TAAATTAAAG   
  
  
+ GACCTGCCGT CAATAAAATT TGGCAGCAAA GAAGAATATA ATTGACCAGT CAATGAGGAC CTGATAACTA   
  
  
+ TAAAAAAAGA GGCGCATGGA CGGGCGCACC CACATGTTA  

- ACTATGGTCT ACTACACAGC ACTTACTAAA CTTTTAGCTT TCGTTACTTA GACTGCTTTT ACCTACTTCC   
  
  
- TACCCAGACC AATCAACAGA AAGAAAACCA CAAATCCGAC CTAATATCAC TAAAATTTCA TTTATTCTAC   
  
  
- CTCCTATCTG ATCTTACTAT GAAACTATTC AGCTAGACCT GCGGTGTTTC TAGTTAGATC CTAAAGCTGT   
  
  
- GGATATTTTA TAAACCTAAT CACAAAACAC AAAAAATTTT TTGAAAAAAA ACAAATCAAA AAAAAAGTAA   
  
  
- AGTGGCAAGT TGTTGAGTTA GAAAAAGAAG AAAAATATTA AAAAAAAGAT AAAGTAGAAA TTTTTAATCC   
  
  
- AACAAAACTT TTAAAACGAA GTATAAAAAA AAAAGACAAC CCAATATATA ATCAGAGTAC CTAAATTAAA   
  
  
- ATAAAAGAGC TAAAGTTGGA AGTTGTGATC TAGATAACCT TCACCCCAAA GTATTATAAA AAAAATAAAT   
  
  
- AAAAGATACT TAAAAAGAAC TAGAGTACTG ATCCAGTGTT CAAATTGTTC AATTGAGTTC AACTGACTCT   
  
  
- AGTAAAAGAA TAACGAAAAA AATTAACTAA AAAAATAAAG TAGGAAGTTA TAACCCAACC TACCTTTAAC   
  
  
- TCTAAGTATA TAAAAAAGAT AAATGAAAGA TACCCCAATA GGACTAGAGT ACACTCAAAT CGTCCAATTC   
  
  
- AGCCCAACTG AAATAAATAA ATAAAAAGAA GAAAAAATTA ACCTGAAACC TTAAAAAAAA TAAAATAAAA   
  
  
- ATACCCTAAT AGGTCTAGAA TACTGAGTTC AGTGCTCAAA TCAACCAACT GGACTCAACT GAACTAAATA   
  
  
- ATAAACCCAA GGAAAAAATT AACTTACAAA AAAAAAGATA AAGTGGAAGG TTGTTGAGTT AACAAAATAA   
  
  
- AGTGCAAAAA AAAGAAGTCA AAGTAGGAAG TTATAATTCA ACAAACCCTT AATCTAAAAC TTTAAAGAAA   
  
  
- AAGTTAAACC AAAAACACCT CAATTGGGCT AGATTATATA AATTAAAAAA AAGAACTAAA ATTAGAAAAT   
  
  
- GTAACCTAGA CAATCTTTTA CCTAGAAACA TTAAAAAATA AACTAAAAAA ACTTCAATAG AGTCAGAATA   
  
  
- CTAGATCCAG TGCTCGAATT GTATAATCGG ATTCAATTGA ACTCAACAAA AAAAATCAAC TAAAAAAAAT   
  
  
- AAAGTAGTAA GTTGTAATTC AATCAACATT TAATCCAAAG TATTAAAAAA ATATATACGA AAGATAACTC   
  
  
- AATATTTTAT AGGGCTAGAG TACTAGGCCC AACGCTCAAA TTGTAGAATT GAATGTAATT GAACCCATCA   
  
  
- AAAAAAATAG GAAAAAAATT AAATTATAAA AAAAAAGTTA AGGTGGGAAG TTATAGTTTA ACTAATCTCT   
  
  
- AACTAGAAGT ATTAAATAAA ACTAAATGAA AGATATATAT TCCAATAATA CCAAAGAACT AAATTCAAAG   
  
  
- TTTAAACTGT CTAATTGAAC ATAGTTAGAA TCTACAAAAA TAAAATTATA AATTTTTTAT GTAGGTTATA   
  
  
- TGAAATACAA AACTTAAATA TTAATTAAAA AAATAAACTT TTATACAATT GTAGCATCCA TATAAAAAAA   
  
  
- TATAATTTTT TAATTAACCT AAGTCTCAGT ATTTTTATTA GTCCAAAAGA GATTTTTAGC CATACGTAGT   
  
  
- GAAAGTTTTG TACTTGCGAG TGTAAGAGGG TTGCCTCCTT TTTACGGAGA TCTACAACAG TTTTCTTCTG   
  
  
- CGGAAATATT AATGTTAAAA AATATGGGTA AAAGGTATAG GGTTTTCCGA CTACTAATAC CGTAAAAGCC   
  
  
- TAAAAATTTT TTTTCTTTTT TTTACTATTT ATGTCTCCAC AAATCGTAAC TTTTCTTTTT ATTTAATTTC   
  
  
- CTGGACGGCA GTTATTTTAA ACCGTCGTTT CTTCTTATAT TAACTGGTCA GTTACTCCTG GACTATTGAT   
  
  
- ATTTTTTTCT CCGCGTACCT GCCCGCGTGG GTGTACAAT

+     P-box

| Site Name | Organism | Position | Strand | Matrix score. | sequence | function |
| --- | --- | --- | --- | --- | --- | --- |
| P-box | Oryza sativa | 1792 | - | 7 | CCTTTTG | gibberellin-responsive element |

>Potri.006G138900.1   
+ TGATACCAGA TGATGTGTCG TGAATGATTT GAAAATCGAA AGCAATGAAT CTGACGAAAA TGGATGAAGG   
  
  
+ ATGGGTCTGG TTAGTTGTCT TTCTTTTGGT GTTTAGGCTG GATTATAGTG ATTTTAAAGT AAATAAGATG   
  
  
+ GAGGATAGAC TAGAATGATA CTTTGATAAG TCGATCTGGA CGCCACAAAG ATCAATCTAG GATTTCGACA   
  
  
+ CCTATAAAAT ATTTGGATTA GTGTTTTGTG TTTTTTAAAA AACTTTTTTT TGTTTAGTTT TTTTTTCATT   
  
  
+ TCACCGTTCA ACAACTCAAT CTTTTTCTTC TTTTTATAAT TTTTTTTCTA TTTCATCTTT AAAAATTAGG   
  
  
+ TTGTTTTGAA AATTTTGCTT CATATTTTTT TTTTCTGTTG GGTTATATAT TAGTCTCATG GATTTAATTT   
  
  
+ TATTTTCTCG ATTTCAACCT TCAACACTAG ATCTATTGGA AGTGGGGTTT CATAATATTT TTTTTATTTA   
  
  
+ TTTTCTATGA ATTTTTCTTG ATCTCATGAC TAGGTCACAA GTTTAACAAG TTAACTCAAG TTGACTGAGA   
  
  
+ TCATTTTCTT ATTGCTTTTT TTAATTGATT TTTTTATTTC ATCCTTCAAT ATTGGGTTGG ATGGAAATTG   
  
  
+ AGATTCATAT ATTTTTTCTA TTTACTTTCT ATGGGGTTAT CCTGATCTCA TGTGAGTTTA GCAGGTTAAG   
  
  
+ TCGGGTTGAC TTTATTTATT TATTTTTCTT CTTTTTTAAT TGGACTTTGG AATTTTTTTT ATTTTATTTT   
  
  
+ TATGGGATTA TCCAGATCTT ATGACTCAAG TCACGAGTTT AGTTGGTTGA CCTGAGTTGA CTTGATTTAT   
  
  
+ TATTTGGGTT CCTTTTTTAA TTGAATGTTT TTTTTTCTAT TTCACCTTCC AACAACTCAA TTGTTTTATT   
  
  
+ TCACGTTTTT TTTCTTCAGT TTCATCCTTC AATATTAAGT TGTTTGGGAA TTAGATTTTG AAATTTCTTT   
  
  
+ TTCAATTTGG TTTTTGTGGA GTTAACCCGA TCTAATATAT TTAATTTTTT TTCTTGATTT TAATCTTTTA   
  
  
+ CATTGGATCT GTTAGAAAAT GGATCTTTGT AATTTTTTAT TTGATTTTTT TGAAGTTATC TCAGTCTTAT   
  
  
+ GATCTAGGTC ACGAGCTTAA CATATTAGCC TAAGTTAACT TGAGTTGTTT TTTTTAGTTG ATTTTTTTTA   
  
  
+ TTTCATCATT CAACATTAAG TTAGTTGTAA ATTAGGTTTC ATAATTTTTT TATATATGCT TTCTATTGAG   
  
  
+ TTATAAAATA TCCCGATCTC ATGATCCGGG TTGCGAGTTT AACATCTTAA CTTACATTAA CTTGGGTAGT   
  
  
+ TTTTTTTATC CTTTTTTTAA TTTAATATTT TTTTTTCAAT TCCACCCTTC AATATCAAAT TGATTAGAGA   
  
  
+ TTGATCTTCA TAATTTATTT TGATTTACTT TCTATATATA AGGTTATTAT GGTTTCTTGA TTTAAGTTTC   
  
  
+ AAATTTGACA GATTAACTTG TATCAATCTT AGATGTTTTT ATTTTAATAT TTAAAAAATA CATCCAATAT   
  
  
+ ACTTTATGTT TTGAATTTAT AATTAATTTT TTTATTTGAA AATATGTTAA CATCGTAGGT ATATTTTTTT   
  
  
+ ATATTAAAAA ATTAATTGGA TTCAGAGTCA TAAAAATAAT CAGGTTTTCT CTAAAAATCG GTATGCATCA   
  
  
+ CTTTCAAAAC ATGAACGCTC ACATTCTCCC AACGGAGGAA AAATGCCTCT AGATGTTGTC AAAAGAAGAC   
  
  
+ GCCTTTATAA TTACAATTTT TTATACCCAT TTTCCATATC CCAAAAGGCT GATGATTATG GCATTTTCGG   
  
  
+ ATTTTTAAAA AAAAGAAAAA AAATGATAAA TACAGAGGTG TTTAGCATTG AAAAGAAAAA TAAATTAAAG   
  
  
+ GACCTGCCGT CAATAAAATT TGGCAGCAAA GAAGAATATA ATTGACCAGT CAATGAGGAC CTGATAACTA   
  
  
+ TAAAAAAAGA GGCGCATGGA CGGGCGCACC CACATGTTA  

- ACTATGGTCT ACTACACAGC ACTTACTAAA CTTTTAGCTT TCGTTACTTA GACTGCTTTT ACCTACTTCC   
  
  
- TACCCAGACC AATCAACAGA AAGAAAACCA CAAATCCGAC CTAATATCAC TAAAATTTCA TTTATTCTAC   
  
  
- CTCCTATCTG ATCTTACTAT GAAACTATTC AGCTAGACCT GCGGTGTTTC TAGTTAGATC CTAAAGCTGT   
  
  
- GGATATTTTA TAAACCTAAT CACAAAACAC AAAAAATTTT TTGAAAAAAA ACAAATCAAA AAAAAAGTAA   
  
  
- AGTGGCAAGT TGTTGAGTTA GAAAAAGAAG AAAAATATTA AAAAAAAGAT AAAGTAGAAA TTTTTAATCC   
  
  
- AACAAAACTT TTAAAACGAA GTATAAAAAA AAAAGACAAC CCAATATATA ATCAGAGTAC CTAAATTAAA   
  
  
- ATAAAAGAGC TAAAGTTGGA AGTTGTGATC TAGATAACCT TCACCCCAAA GTATTATAAA AAAAATAAAT   
  
  
- AAAAGATACT TAAAAAGAAC TAGAGTACTG ATCCAGTGTT CAAATTGTTC AATTGAGTTC AACTGACTCT   
  
  
- AGTAAAAGAA TAACGAAAAA AATTAACTAA AAAAATAAAG TAGGAAGTTA TAACCCAACC TACCTTTAAC   
  
  
- TCTAAGTATA TAAAAAAGAT AAATGAAAGA TACCCCAATA GGACTAGAGT ACACTCAAAT CGTCCAATTC   
  
  
- AGCCCAACTG AAATAAATAA ATAAAAAGAA GAAAAAATTA ACCTGAAACC TTAAAAAAAA TAAAATAAAA   
  
  
- ATACCCTAAT AGGTCTAGAA TACTGAGTTC AGTGCTCAAA TCAACCAACT GGACTCAACT GAACTAAATA   
  
  
- ATAAACCCAA GGAAAAAATT AACTTACAAA AAAAAAGATA AAGTGGAAGG TTGTTGAGTT AACAAAATAA   
  
  
- AGTGCAAAAA AAAGAAGTCA AAGTAGGAAG TTATAATTCA ACAAACCCTT AATCTAAAAC TTTAAAGAAA   
  
  
- AAGTTAAACC AAAAACACCT CAATTGGGCT AGATTATATA AATTAAAAAA AAGAACTAAA ATTAGAAAAT   
  
  
- GTAACCTAGA CAATCTTTTA CCTAGAAACA TTAAAAAATA AACTAAAAAA ACTTCAATAG AGTCAGAATA   
  
  
- CTAGATCCAG TGCTCGAATT GTATAATCGG ATTCAATTGA ACTCAACAAA AAAAATCAAC TAAAAAAAAT   
  
  
- AAAGTAGTAA GTTGTAATTC AATCAACATT TAATCCAAAG TATTAAAAAA ATATATACGA AAGATAACTC   
  
  
- AATATTTTAT AGGGCTAGAG TACTAGGCCC AACGCTCAAA TTGTAGAATT GAATGTAATT GAACCCATCA   
  
  
- AAAAAAATAG GAAAAAAATT AAATTATAAA AAAAAAGTTA AGGTGGGAAG TTATAGTTTA ACTAATCTCT   
  
  
- AACTAGAAGT ATTAAATAAA ACTAAATGAA AGATATATAT TCCAATAATA CCAAAGAACT AAATTCAAAG   
  
  
- TTTAAACTGT CTAATTGAAC ATAGTTAGAA TCTACAAAAA TAAAATTATA AATTTTTTAT GTAGGTTATA   
  
  
- TGAAATACAA AACTTAAATA TTAATTAAAA AAATAAACTT TTATACAATT GTAGCATCCA TATAAAAAAA   
  
  
- TATAATTTTT TAATTAACCT AAGTCTCAGT ATTTTTATTA GTCCAAAAGA GATTTTTAGC CATACGTAGT   
  
  
- GAAAGTTTTG TACTTGCGAG TGTAAGAGGG TTGCCTCCTT TTTACGGAGA TCTACAACAG TTTTCTTCTG   
  
  
- CGGAAATATT AATGTTAAAA AATATGGGTA AAAGGTATAG GGTTTTCCGA CTACTAATAC CGTAAAAGCC   
  
  
- TAAAAATTTT TTTTCTTTTT TTTACTATTT ATGTCTCCAC AAATCGTAAC TTTTCTTTTT ATTTAATTTC   
  
  
- CTGGACGGCA GTTATTTTAA ACCGTCGTTT CTTCTTATAT TAACTGGTCA GTTACTCCTG GACTATTGAT   
  
  
- ATTTTTTTCT CCGCGTACCT GCCCGCGTGG GTGTACAAT

+     TATA

| Site Name | Organism | Position | Strand | Matrix score. | sequence | function |
| --- | --- | --- | --- | --- | --- | --- |
| TATA | Arabidopsis thaliana | 1262 | + | 8 | TATAAAAT |  |
| TATA | Arabidopsis thaliana | 213 | + | 8 | TATAAAAT |  |

>Potri.006G138900.1   
+ TGATACCAGA TGATGTGTCG TGAATGATTT GAAAATCGAA AGCAATGAAT CTGACGAAAA TGGATGAAGG   
  
  
+ ATGGGTCTGG TTAGTTGTCT TTCTTTTGGT GTTTAGGCTG GATTATAGTG ATTTTAAAGT AAATAAGATG   
  
  
+ GAGGATAGAC TAGAATGATA CTTTGATAAG TCGATCTGGA CGCCACAAAG ATCAATCTAG GATTTCGACA   
  
  
+ CCTATAAAAT ATTTGGATTA GTGTTTTGTG TTTTTTAAAA AACTTTTTTT TGTTTAGTTT TTTTTTCATT   
  
  
+ TCACCGTTCA ACAACTCAAT CTTTTTCTTC TTTTTATAAT TTTTTTTCTA TTTCATCTTT AAAAATTAGG   
  
  
+ TTGTTTTGAA AATTTTGCTT CATATTTTTT TTTTCTGTTG GGTTATATAT TAGTCTCATG GATTTAATTT   
  
  
+ TATTTTCTCG ATTTCAACCT TCAACACTAG ATCTATTGGA AGTGGGGTTT CATAATATTT TTTTTATTTA   
  
  
+ TTTTCTATGA ATTTTTCTTG ATCTCATGAC TAGGTCACAA GTTTAACAAG TTAACTCAAG TTGACTGAGA   
  
  
+ TCATTTTCTT ATTGCTTTTT TTAATTGATT TTTTTATTTC ATCCTTCAAT ATTGGGTTGG ATGGAAATTG   
  
  
+ AGATTCATAT ATTTTTTCTA TTTACTTTCT ATGGGGTTAT CCTGATCTCA TGTGAGTTTA GCAGGTTAAG   
  
  
+ TCGGGTTGAC TTTATTTATT TATTTTTCTT CTTTTTTAAT TGGACTTTGG AATTTTTTTT ATTTTATTTT   
  
  
+ TATGGGATTA TCCAGATCTT ATGACTCAAG TCACGAGTTT AGTTGGTTGA CCTGAGTTGA CTTGATTTAT   
  
  
+ TATTTGGGTT CCTTTTTTAA TTGAATGTTT TTTTTTCTAT TTCACCTTCC AACAACTCAA TTGTTTTATT   
  
  
+ TCACGTTTTT TTTCTTCAGT TTCATCCTTC AATATTAAGT TGTTTGGGAA TTAGATTTTG AAATTTCTTT   
  
  
+ TTCAATTTGG TTTTTGTGGA GTTAACCCGA TCTAATATAT TTAATTTTTT TTCTTGATTT TAATCTTTTA   
  
  
+ CATTGGATCT GTTAGAAAAT GGATCTTTGT AATTTTTTAT TTGATTTTTT TGAAGTTATC TCAGTCTTAT   
  
  
+ GATCTAGGTC ACGAGCTTAA CATATTAGCC TAAGTTAACT TGAGTTGTTT TTTTTAGTTG ATTTTTTTTA   
  
  
+ TTTCATCATT CAACATTAAG TTAGTTGTAA ATTAGGTTTC ATAATTTTTT TATATATGCT TTCTATTGAG   
  
  
+ TTATAAAATA TCCCGATCTC ATGATCCGGG TTGCGAGTTT AACATCTTAA CTTACATTAA CTTGGGTAGT   
  
  
+ TTTTTTTATC CTTTTTTTAA TTTAATATTT TTTTTTCAAT TCCACCCTTC AATATCAAAT TGATTAGAGA   
  
  
+ TTGATCTTCA TAATTTATTT TGATTTACTT TCTATATATA AGGTTATTAT GGTTTCTTGA TTTAAGTTTC   
  
  
+ AAATTTGACA GATTAACTTG TATCAATCTT AGATGTTTTT ATTTTAATAT TTAAAAAATA CATCCAATAT   
  
  
+ ACTTTATGTT TTGAATTTAT AATTAATTTT TTTATTTGAA AATATGTTAA CATCGTAGGT ATATTTTTTT   
  
  
+ ATATTAAAAA ATTAATTGGA TTCAGAGTCA TAAAAATAAT CAGGTTTTCT CTAAAAATCG GTATGCATCA   
  
  
+ CTTTCAAAAC ATGAACGCTC ACATTCTCCC AACGGAGGAA AAATGCCTCT AGATGTTGTC AAAAGAAGAC   
  
  
+ GCCTTTATAA TTACAATTTT TTATACCCAT TTTCCATATC CCAAAAGGCT GATGATTATG GCATTTTCGG   
  
  
+ ATTTTTAAAA AAAAGAAAAA AAATGATAAA TACAGAGGTG TTTAGCATTG AAAAGAAAAA TAAATTAAAG   
  
  
+ GACCTGCCGT CAATAAAATT TGGCAGCAAA GAAGAATATA ATTGACCAGT CAATGAGGAC CTGATAACTA   
  
  
+ TAAAAAAAGA GGCGCATGGA CGGGCGCACC CACATGTTA  

- ACTATGGTCT ACTACACAGC ACTTACTAAA CTTTTAGCTT TCGTTACTTA GACTGCTTTT ACCTACTTCC   
  
  
- TACCCAGACC AATCAACAGA AAGAAAACCA CAAATCCGAC CTAATATCAC TAAAATTTCA TTTATTCTAC   
  
  
- CTCCTATCTG ATCTTACTAT GAAACTATTC AGCTAGACCT GCGGTGTTTC TAGTTAGATC CTAAAGCTGT   
  
  
- GGATATTTTA TAAACCTAAT CACAAAACAC AAAAAATTTT TTGAAAAAAA ACAAATCAAA AAAAAAGTAA   
  
  
- AGTGGCAAGT TGTTGAGTTA GAAAAAGAAG AAAAATATTA AAAAAAAGAT AAAGTAGAAA TTTTTAATCC   
  
  
- AACAAAACTT TTAAAACGAA GTATAAAAAA AAAAGACAAC CCAATATATA ATCAGAGTAC CTAAATTAAA   
  
  
- ATAAAAGAGC TAAAGTTGGA AGTTGTGATC TAGATAACCT TCACCCCAAA GTATTATAAA AAAAATAAAT   
  
  
- AAAAGATACT TAAAAAGAAC TAGAGTACTG ATCCAGTGTT CAAATTGTTC AATTGAGTTC AACTGACTCT   
  
  
- AGTAAAAGAA TAACGAAAAA AATTAACTAA AAAAATAAAG TAGGAAGTTA TAACCCAACC TACCTTTAAC   
  
  
- TCTAAGTATA TAAAAAAGAT AAATGAAAGA TACCCCAATA GGACTAGAGT ACACTCAAAT CGTCCAATTC   
  
  
- AGCCCAACTG AAATAAATAA ATAAAAAGAA GAAAAAATTA ACCTGAAACC TTAAAAAAAA TAAAATAAAA   
  
  
- ATACCCTAAT AGGTCTAGAA TACTGAGTTC AGTGCTCAAA TCAACCAACT GGACTCAACT GAACTAAATA   
  
  
- ATAAACCCAA GGAAAAAATT AACTTACAAA AAAAAAGATA AAGTGGAAGG TTGTTGAGTT AACAAAATAA   
  
  
- AGTGCAAAAA AAAGAAGTCA AAGTAGGAAG TTATAATTCA ACAAACCCTT AATCTAAAAC TTTAAAGAAA   
  
  
- AAGTTAAACC AAAAACACCT CAATTGGGCT AGATTATATA AATTAAAAAA AAGAACTAAA ATTAGAAAAT   
  
  
- GTAACCTAGA CAATCTTTTA CCTAGAAACA TTAAAAAATA AACTAAAAAA ACTTCAATAG AGTCAGAATA   
  
  
- CTAGATCCAG TGCTCGAATT GTATAATCGG ATTCAATTGA ACTCAACAAA AAAAATCAAC TAAAAAAAAT   
  
  
- AAAGTAGTAA GTTGTAATTC AATCAACATT TAATCCAAAG TATTAAAAAA ATATATACGA AAGATAACTC   
  
  
- AATATTTTAT AGGGCTAGAG TACTAGGCCC AACGCTCAAA TTGTAGAATT GAATGTAATT GAACCCATCA   
  
  
- AAAAAAATAG GAAAAAAATT AAATTATAAA AAAAAAGTTA AGGTGGGAAG TTATAGTTTA ACTAATCTCT   
  
  
- AACTAGAAGT ATTAAATAAA ACTAAATGAA AGATATATAT TCCAATAATA CCAAAGAACT AAATTCAAAG   
  
  
- TTTAAACTGT CTAATTGAAC ATAGTTAGAA TCTACAAAAA TAAAATTATA AATTTTTTAT GTAGGTTATA   
  
  
- TGAAATACAA AACTTAAATA TTAATTAAAA AAATAAACTT TTATACAATT GTAGCATCCA TATAAAAAAA   
  
  
- TATAATTTTT TAATTAACCT AAGTCTCAGT ATTTTTATTA GTCCAAAAGA GATTTTTAGC CATACGTAGT   
  
  
- GAAAGTTTTG TACTTGCGAG TGTAAGAGGG TTGCCTCCTT TTTACGGAGA TCTACAACAG TTTTCTTCTG   
  
  
- CGGAAATATT AATGTTAAAA AATATGGGTA AAAGGTATAG GGTTTTCCGA CTACTAATAC CGTAAAAGCC   
  
  
- TAAAAATTTT TTTTCTTTTT TTTACTATTT ATGTCTCCAC AAATCGTAAC TTTTCTTTTT ATTTAATTTC   
  
  
- CTGGACGGCA GTTATTTTAA ACCGTCGTTT CTTCTTATAT TAACTGGTCA GTTACTCCTG GACTATTGAT   
  
  
- ATTTTTTTCT CCGCGTACCT GCCCGCGTGG GTGTACAAT

+     TATA-box

| Site Name | Organism | Position | Strand | Matrix score. | sequence | function |
| --- | --- | --- | --- | --- | --- | --- |
| TATA-box | Helianthus annuus | 1239 | - | 6 | TATAAA | core promoter element around -30 of transcription start |
| TATA-box | Arabidopsis thaliana | 213 | + | 4 | TATA | core promoter element around -30 of transcription start |
| TATA-box | Arabidopsis thaliana | 114 | + | 4 | TATA | core promoter element around -30 of transcription start |
| TATA-box | Arabidopsis thaliana | 632 | - | 9 | taTATAAAtc | core promoter element around -30 of transcription start |
| TATA-box | Arabidopsis thaliana | 396 | + | 4 | TATA | core promoter element around -30 of transcription start |
| TATA-box | Arabidopsis thaliana | 1959 | - | 4 | TATA | core promoter element around -30 of transcription start |
| TATA-box | Arabidopsis thaliana | 1957 | + | 9 | ccTATAAAaa | core promoter element around -30 of transcription start |
| TATA-box | Arabidopsis thaliana | 1927 | - | 4 | TATA | core promoter element around -30 of transcription start |
| TATA-box | Arabidopsis thaliana | 1772 | - | 4 | TATA | core promoter element around -30 of transcription start |
| TATA-box | Arabidopsis thaliana | 1609 | - | 5 | TATAA | core promoter element around -30 of transcription start |
| TATA-box | Arabidopsis thaliana | 1771 | - | 5 | TATAA | core promoter element around -30 of transcription start |
| TATA-box | Helianthus annuus | 1608 | - | 6 | TATAAA | core promoter element around -30 of transcription start |
| TATA-box | Arabidopsis thaliana | 1600 | - | 4 | TATA | core promoter element around -30 of transcription start |
| TATA-box | Helianthus annuus | 1770 | - | 6 | TATAAA | core promoter element around -30 of transcription start |
| TATA-box | Arabidopsis thaliana | 1755 | - | 5 | TATAA | core promoter element around -30 of transcription start |
| TATA-box | Arabidopsis thaliana | 1756 | - | 4 | TATA | core promoter element around -30 of transcription start |
| TATA-box | Arabidopsis thaliana | 1610 | - | 4 | TATA | core promoter element around -30 of transcription start |
| TATA-box | Helianthus annuus | 1556 | - | 6 | TATAAA | core promoter element around -30 of transcription start |
| TATA-box | Brassica juncea | 1555 | - | 7 | TATAAAT | core promoter element around -30 of transcription start |
| TATA-box | Brassica oleracea | 1926 | + | 6 | ATATAA | core promoter element around -30 of transcription start |
| TATA-box | Helianthus annuus | 1754 | - | 6 | TATAAA | core promoter element around -30 of transcription start |
| TATA-box | Arabidopsis thaliana | 1557 | - | 5 | TATAA | core promoter element around -30 of transcription start |
| TATA-box | Pisum sativum | 1769 | - | 7 | TATAAAA | core promoter element around -30 of transcription start |
| TATA-box | Arabidopsis thaliana | 1558 | - | 4 | TATA | core promoter element around -30 of transcription start |
| TATA-box | Pisum sativum | 1607 | - | 7 | TATAAAA | core promoter element around -30 of transcription start |
| TATA-box | Arabidopsis thaliana | 1518 | + | 8 | TATTTAAA | core promoter element around -30 of transcription start |
| TATA-box | Arabidopsis thaliana | 1538 | - | 4 | TATA | core promoter element around -30 of transcription start |
| TATA-box | Arabidopsis thaliana | 1437 | - | 4 | TATA | core promoter element around -30 of transcription start |
| TATA-box | Brassica oleracea | 1436 | + | 6 | ATATAA | core promoter element around -30 of transcription start |
| TATA-box | Arabidopsis thaliana | 1435 | - | 6 | TATATA | core promoter element around -30 of transcription start |
| TATA-box | Arabidopsis thaliana | 393 | - | 7 | TATATAA | core promoter element around -30 of transcription start |
| TATA-box | Brassica napus | 1434 | - | 6 | ATATAT | core promoter element around -30 of transcription start |
| TATA-box | Arabidopsis thaliana | 315 | + | 4 | TATA | core promoter element around -30 of transcription start |
| TATA-box | Arabidopsis thaliana | 394 | + | 6 | TATATA | core promoter element around -30 of transcription start |
| TATA-box | Brassica napus | 395 | + | 6 | ATATAT | core promoter element around -30 of transcription start |
| TATA-box | Helianthus annuus | 313 | - | 6 | TATAAA | core promoter element around -30 of transcription start |
| TATA-box | Arabidopsis thaliana | 314 | - | 5 | TATAA | core promoter element around -30 of transcription start |
| TATA-box | Arabidopsis thaliana | 113 | - | 5 | TATAA | core promoter element around -30 of transcription start |
| TATA-box | Arabidopsis thaliana | 211 | + | 9 | ccTATAAAaa | core promoter element around -30 of transcription start |
| TATA-box | Brassica napus | 112 | + | 6 | ATTATA | core promoter element around -30 of transcription start |
| TATA-box | Pisum sativum | 312 | - | 7 | TATAAAA | core promoter element around -30 of transcription start |
| TATA-box | Arabidopsis thaliana | 1240 | - | 7 | TATATAA | core promoter element around -30 of transcription start |
| TATA-box | Arabidopsis thaliana | 1016 | - | 4 | TATA | core promoter element around -30 of transcription start |
| TATA-box | Arabidopsis thaliana | 1433 | - | 6 | TATATA | core promoter element around -30 of transcription start |
| TATA-box | Arabidopsis thaliana | 1261 | - | 5 | TATAA | core promoter element around -30 of transcription start |
| TATA-box | Arabidopsis thaliana | 1262 | - | 4 | TATA | core promoter element around -30 of transcription start |
| TATA-box | Brassica napus | 1242 | - | 6 | ATATAT | core promoter element around -30 of transcription start |
| TATA-box | Pisum sativum | 1238 | - | 7 | TATAAAA | core promoter element around -30 of transcription start |
| TATA-box | Brassica napus | 1015 | - | 6 | ATATAT | core promoter element around -30 of transcription start |
| TATA-box | Arabidopsis thaliana | 767 | - | 9 | ccTATAAAaa | core promoter element around -30 of transcription start |
| TATA-box | Arabidopsis thaliana | 1241 | - | 6 | TATATA | core promoter element around -30 of transcription start |
| TATA-box | Arabidopsis thaliana | 1243 | - | 4 | TATA | core promoter element around -30 of transcription start |
| TATA-box | Arabidopsis thaliana | 638 | + | 4 | TATA | core promoter element around -30 of transcription start |
| TATA-box | Brassica napus | 637 | + | 6 | ATATAT | core promoter element around -30 of transcription start |

>Potri.006G138900.1   
+ TGATACCAGA TGATGTGTCG TGAATGATTT GAAAATCGAA AGCAATGAAT CTGACGAAAA TGGATGAAGG   
  
  
+ ATGGGTCTGG TTAGTTGTCT TTCTTTTGGT GTTTAGGCTG GATTATAGTG ATTTTAAAGT AAATAAGATG   
  
  
+ GAGGATAGAC TAGAATGATA CTTTGATAAG TCGATCTGGA CGCCACAAAG ATCAATCTAG GATTTCGACA   
  
  
+ CCTATAAAAT ATTTGGATTA GTGTTTTGTG TTTTTTAAAA AACTTTTTTT TGTTTAGTTT TTTTTTCATT   
  
  
+ TCACCGTTCA ACAACTCAAT CTTTTTCTTC TTTTTATAAT TTTTTTTCTA TTTCATCTTT AAAAATTAGG   
  
  
+ TTGTTTTGAA AATTTTGCTT CATATTTTTT TTTTCTGTTG GGTTATATAT TAGTCTCATG GATTTAATTT   
  
  
+ TATTTTCTCG ATTTCAACCT TCAACACTAG ATCTATTGGA AGTGGGGTTT CATAATATTT TTTTTATTTA   
  
  
+ TTTTCTATGA ATTTTTCTTG ATCTCATGAC TAGGTCACAA GTTTAACAAG TTAACTCAAG TTGACTGAGA   
  
  
+ TCATTTTCTT ATTGCTTTTT TTAATTGATT TTTTTATTTC ATCCTTCAAT ATTGGGTTGG ATGGAAATTG   
  
  
+ AGATTCATAT ATTTTTTCTA TTTACTTTCT ATGGGGTTAT CCTGATCTCA TGTGAGTTTA GCAGGTTAAG   
  
  
+ TCGGGTTGAC TTTATTTATT TATTTTTCTT CTTTTTTAAT TGGACTTTGG AATTTTTTTT ATTTTATTTT   
  
  
+ TATGGGATTA TCCAGATCTT ATGACTCAAG TCACGAGTTT AGTTGGTTGA CCTGAGTTGA CTTGATTTAT   
  
  
+ TATTTGGGTT CCTTTTTTAA TTGAATGTTT TTTTTTCTAT TTCACCTTCC AACAACTCAA TTGTTTTATT   
  
  
+ TCACGTTTTT TTTCTTCAGT TTCATCCTTC AATATTAAGT TGTTTGGGAA TTAGATTTTG AAATTTCTTT   
  
  
+ TTCAATTTGG TTTTTGTGGA GTTAACCCGA TCTAATATAT TTAATTTTTT TTCTTGATTT TAATCTTTTA   
  
  
+ CATTGGATCT GTTAGAAAAT GGATCTTTGT AATTTTTTAT TTGATTTTTT TGAAGTTATC TCAGTCTTAT   
  
  
+ GATCTAGGTC ACGAGCTTAA CATATTAGCC TAAGTTAACT TGAGTTGTTT TTTTTAGTTG ATTTTTTTTA   
  
  
+ TTTCATCATT CAACATTAAG TTAGTTGTAA ATTAGGTTTC ATAATTTTTT TATATATGCT TTCTATTGAG   
  
  
+ TTATAAAATA TCCCGATCTC ATGATCCGGG TTGCGAGTTT AACATCTTAA CTTACATTAA CTTGGGTAGT   
  
  
+ TTTTTTTATC CTTTTTTTAA TTTAATATTT TTTTTTCAAT TCCACCCTTC AATATCAAAT TGATTAGAGA   
  
  
+ TTGATCTTCA TAATTTATTT TGATTTACTT TCTATATATA AGGTTATTAT GGTTTCTTGA TTTAAGTTTC   
  
  
+ AAATTTGACA GATTAACTTG TATCAATCTT AGATGTTTTT ATTTTAATAT TTAAAAAATA CATCCAATAT   
  
  
+ ACTTTATGTT TTGAATTTAT AATTAATTTT TTTATTTGAA AATATGTTAA CATCGTAGGT ATATTTTTTT   
  
  
+ ATATTAAAAA ATTAATTGGA TTCAGAGTCA TAAAAATAAT CAGGTTTTCT CTAAAAATCG GTATGCATCA   
  
  
+ CTTTCAAAAC ATGAACGCTC ACATTCTCCC AACGGAGGAA AAATGCCTCT AGATGTTGTC AAAAGAAGAC   
  
  
+ GCCTTTATAA TTACAATTTT TTATACCCAT TTTCCATATC CCAAAAGGCT GATGATTATG GCATTTTCGG   
  
  
+ ATTTTTAAAA AAAAGAAAAA AAATGATAAA TACAGAGGTG TTTAGCATTG AAAAGAAAAA TAAATTAAAG   
  
  
+ GACCTGCCGT CAATAAAATT TGGCAGCAAA GAAGAATATA ATTGACCAGT CAATGAGGAC CTGATAACTA   
  
  
+ TAAAAAAAGA GGCGCATGGA CGGGCGCACC CACATGTTA  

- ACTATGGTCT ACTACACAGC ACTTACTAAA CTTTTAGCTT TCGTTACTTA GACTGCTTTT ACCTACTTCC   
  
  
- TACCCAGACC AATCAACAGA AAGAAAACCA CAAATCCGAC CTAATATCAC TAAAATTTCA TTTATTCTAC   
  
  
- CTCCTATCTG ATCTTACTAT GAAACTATTC AGCTAGACCT GCGGTGTTTC TAGTTAGATC CTAAAGCTGT   
  
  
- GGATATTTTA TAAACCTAAT CACAAAACAC AAAAAATTTT TTGAAAAAAA ACAAATCAAA AAAAAAGTAA   
  
  
- AGTGGCAAGT TGTTGAGTTA GAAAAAGAAG AAAAATATTA AAAAAAAGAT AAAGTAGAAA TTTTTAATCC   
  
  
- AACAAAACTT TTAAAACGAA GTATAAAAAA AAAAGACAAC CCAATATATA ATCAGAGTAC CTAAATTAAA   
  
  
- ATAAAAGAGC TAAAGTTGGA AGTTGTGATC TAGATAACCT TCACCCCAAA GTATTATAAA AAAAATAAAT   
  
  
- AAAAGATACT TAAAAAGAAC TAGAGTACTG ATCCAGTGTT CAAATTGTTC AATTGAGTTC AACTGACTCT   
  
  
- AGTAAAAGAA TAACGAAAAA AATTAACTAA AAAAATAAAG TAGGAAGTTA TAACCCAACC TACCTTTAAC   
  
  
- TCTAAGTATA TAAAAAAGAT AAATGAAAGA TACCCCAATA GGACTAGAGT ACACTCAAAT CGTCCAATTC   
  
  
- AGCCCAACTG AAATAAATAA ATAAAAAGAA GAAAAAATTA ACCTGAAACC TTAAAAAAAA TAAAATAAAA   
  
  
- ATACCCTAAT AGGTCTAGAA TACTGAGTTC AGTGCTCAAA TCAACCAACT GGACTCAACT GAACTAAATA   
  
  
- ATAAACCCAA GGAAAAAATT AACTTACAAA AAAAAAGATA AAGTGGAAGG TTGTTGAGTT AACAAAATAA   
  
  
- AGTGCAAAAA AAAGAAGTCA AAGTAGGAAG TTATAATTCA ACAAACCCTT AATCTAAAAC TTTAAAGAAA   
  
  
- AAGTTAAACC AAAAACACCT CAATTGGGCT AGATTATATA AATTAAAAAA AAGAACTAAA ATTAGAAAAT   
  
  
- GTAACCTAGA CAATCTTTTA CCTAGAAACA TTAAAAAATA AACTAAAAAA ACTTCAATAG AGTCAGAATA   
  
  
- CTAGATCCAG TGCTCGAATT GTATAATCGG ATTCAATTGA ACTCAACAAA AAAAATCAAC TAAAAAAAAT   
  
  
- AAAGTAGTAA GTTGTAATTC AATCAACATT TAATCCAAAG TATTAAAAAA ATATATACGA AAGATAACTC   
  
  
- AATATTTTAT AGGGCTAGAG TACTAGGCCC AACGCTCAAA TTGTAGAATT GAATGTAATT GAACCCATCA   
  
  
- AAAAAAATAG GAAAAAAATT AAATTATAAA AAAAAAGTTA AGGTGGGAAG TTATAGTTTA ACTAATCTCT   
  
  
- AACTAGAAGT ATTAAATAAA ACTAAATGAA AGATATATAT TCCAATAATA CCAAAGAACT AAATTCAAAG   
  
  
- TTTAAACTGT CTAATTGAAC ATAGTTAGAA TCTACAAAAA TAAAATTATA AATTTTTTAT GTAGGTTATA   
  
  
- TGAAATACAA AACTTAAATA TTAATTAAAA AAATAAACTT TTATACAATT GTAGCATCCA TATAAAAAAA   
  
  
- TATAATTTTT TAATTAACCT AAGTCTCAGT ATTTTTATTA GTCCAAAAGA GATTTTTAGC CATACGTAGT   
  
  
- GAAAGTTTTG TACTTGCGAG TGTAAGAGGG TTGCCTCCTT TTTACGGAGA TCTACAACAG TTTTCTTCTG   
  
  
- CGGAAATATT AATGTTAAAA AATATGGGTA AAAGGTATAG GGTTTTCCGA CTACTAATAC CGTAAAAGCC   
  
  
- TAAAAATTTT TTTTCTTTTT TTTACTATTT ATGTCTCCAC AAATCGTAAC TTTTCTTTTT ATTTAATTTC   
  
  
- CTGGACGGCA GTTATTTTAA ACCGTCGTTT CTTCTTATAT TAACTGGTCA GTTACTCCTG GACTATTGAT   
  
  
- ATTTTTTTCT CCGCGTACCT GCCCGCGTGG GTGTACAAT

+     TATC-box

| Site Name | Organism | Position | Strand | Matrix score. | sequence | function |
| --- | --- | --- | --- | --- | --- | --- |
| TATC-box | Oryza sativa | 1787 | + | 7 | TATCCCA | cis-acting element involved in gibberellin-responsiveness |

>Potri.006G138900.1   
+ TGATACCAGA TGATGTGTCG TGAATGATTT GAAAATCGAA AGCAATGAAT CTGACGAAAA TGGATGAAGG   
  
  
+ ATGGGTCTGG TTAGTTGTCT TTCTTTTGGT GTTTAGGCTG GATTATAGTG ATTTTAAAGT AAATAAGATG   
  
  
+ GAGGATAGAC TAGAATGATA CTTTGATAAG TCGATCTGGA CGCCACAAAG ATCAATCTAG GATTTCGACA   
  
  
+ CCTATAAAAT ATTTGGATTA GTGTTTTGTG TTTTTTAAAA AACTTTTTTT TGTTTAGTTT TTTTTTCATT   
  
  
+ TCACCGTTCA ACAACTCAAT CTTTTTCTTC TTTTTATAAT TTTTTTTCTA TTTCATCTTT AAAAATTAGG   
  
  
+ TTGTTTTGAA AATTTTGCTT CATATTTTTT TTTTCTGTTG GGTTATATAT TAGTCTCATG GATTTAATTT   
  
  
+ TATTTTCTCG ATTTCAACCT TCAACACTAG ATCTATTGGA AGTGGGGTTT CATAATATTT TTTTTATTTA   
  
  
+ TTTTCTATGA ATTTTTCTTG ATCTCATGAC TAGGTCACAA GTTTAACAAG TTAACTCAAG TTGACTGAGA   
  
  
+ TCATTTTCTT ATTGCTTTTT TTAATTGATT TTTTTATTTC ATCCTTCAAT ATTGGGTTGG ATGGAAATTG   
  
  
+ AGATTCATAT ATTTTTTCTA TTTACTTTCT ATGGGGTTAT CCTGATCTCA TGTGAGTTTA GCAGGTTAAG   
  
  
+ TCGGGTTGAC TTTATTTATT TATTTTTCTT CTTTTTTAAT TGGACTTTGG AATTTTTTTT ATTTTATTTT   
  
  
+ TATGGGATTA TCCAGATCTT ATGACTCAAG TCACGAGTTT AGTTGGTTGA CCTGAGTTGA CTTGATTTAT   
  
  
+ TATTTGGGTT CCTTTTTTAA TTGAATGTTT TTTTTTCTAT TTCACCTTCC AACAACTCAA TTGTTTTATT   
  
  
+ TCACGTTTTT TTTCTTCAGT TTCATCCTTC AATATTAAGT TGTTTGGGAA TTAGATTTTG AAATTTCTTT   
  
  
+ TTCAATTTGG TTTTTGTGGA GTTAACCCGA TCTAATATAT TTAATTTTTT TTCTTGATTT TAATCTTTTA   
  
  
+ CATTGGATCT GTTAGAAAAT GGATCTTTGT AATTTTTTAT TTGATTTTTT TGAAGTTATC TCAGTCTTAT   
  
  
+ GATCTAGGTC ACGAGCTTAA CATATTAGCC TAAGTTAACT TGAGTTGTTT TTTTTAGTTG ATTTTTTTTA   
  
  
+ TTTCATCATT CAACATTAAG TTAGTTGTAA ATTAGGTTTC ATAATTTTTT TATATATGCT TTCTATTGAG   
  
  
+ TTATAAAATA TCCCGATCTC ATGATCCGGG TTGCGAGTTT AACATCTTAA CTTACATTAA CTTGGGTAGT   
  
  
+ TTTTTTTATC CTTTTTTTAA TTTAATATTT TTTTTTCAAT TCCACCCTTC AATATCAAAT TGATTAGAGA   
  
  
+ TTGATCTTCA TAATTTATTT TGATTTACTT TCTATATATA AGGTTATTAT GGTTTCTTGA TTTAAGTTTC   
  
  
+ AAATTTGACA GATTAACTTG TATCAATCTT AGATGTTTTT ATTTTAATAT TTAAAAAATA CATCCAATAT   
  
  
+ ACTTTATGTT TTGAATTTAT AATTAATTTT TTTATTTGAA AATATGTTAA CATCGTAGGT ATATTTTTTT   
  
  
+ ATATTAAAAA ATTAATTGGA TTCAGAGTCA TAAAAATAAT CAGGTTTTCT CTAAAAATCG GTATGCATCA   
  
  
+ CTTTCAAAAC ATGAACGCTC ACATTCTCCC AACGGAGGAA AAATGCCTCT AGATGTTGTC AAAAGAAGAC   
  
  
+ GCCTTTATAA TTACAATTTT TTATACCCAT TTTCCATATC CCAAAAGGCT GATGATTATG GCATTTTCGG   
  
  
+ ATTTTTAAAA AAAAGAAAAA AAATGATAAA TACAGAGGTG TTTAGCATTG AAAAGAAAAA TAAATTAAAG   
  
  
+ GACCTGCCGT CAATAAAATT TGGCAGCAAA GAAGAATATA ATTGACCAGT CAATGAGGAC CTGATAACTA   
  
  
+ TAAAAAAAGA GGCGCATGGA CGGGCGCACC CACATGTTA  

- ACTATGGTCT ACTACACAGC ACTTACTAAA CTTTTAGCTT TCGTTACTTA GACTGCTTTT ACCTACTTCC   
  
  
- TACCCAGACC AATCAACAGA AAGAAAACCA CAAATCCGAC CTAATATCAC TAAAATTTCA TTTATTCTAC   
  
  
- CTCCTATCTG ATCTTACTAT GAAACTATTC AGCTAGACCT GCGGTGTTTC TAGTTAGATC CTAAAGCTGT   
  
  
- GGATATTTTA TAAACCTAAT CACAAAACAC AAAAAATTTT TTGAAAAAAA ACAAATCAAA AAAAAAGTAA   
  
  
- AGTGGCAAGT TGTTGAGTTA GAAAAAGAAG AAAAATATTA AAAAAAAGAT AAAGTAGAAA TTTTTAATCC   
  
  
- AACAAAACTT TTAAAACGAA GTATAAAAAA AAAAGACAAC CCAATATATA ATCAGAGTAC CTAAATTAAA   
  
  
- ATAAAAGAGC TAAAGTTGGA AGTTGTGATC TAGATAACCT TCACCCCAAA GTATTATAAA AAAAATAAAT   
  
  
- AAAAGATACT TAAAAAGAAC TAGAGTACTG ATCCAGTGTT CAAATTGTTC AATTGAGTTC AACTGACTCT   
  
  
- AGTAAAAGAA TAACGAAAAA AATTAACTAA AAAAATAAAG TAGGAAGTTA TAACCCAACC TACCTTTAAC   
  
  
- TCTAAGTATA TAAAAAAGAT AAATGAAAGA TACCCCAATA GGACTAGAGT ACACTCAAAT CGTCCAATTC   
  
  
- AGCCCAACTG AAATAAATAA ATAAAAAGAA GAAAAAATTA ACCTGAAACC TTAAAAAAAA TAAAATAAAA   
  
  
- ATACCCTAAT AGGTCTAGAA TACTGAGTTC AGTGCTCAAA TCAACCAACT GGACTCAACT GAACTAAATA   
  
  
- ATAAACCCAA GGAAAAAATT AACTTACAAA AAAAAAGATA AAGTGGAAGG TTGTTGAGTT AACAAAATAA   
  
  
- AGTGCAAAAA AAAGAAGTCA AAGTAGGAAG TTATAATTCA ACAAACCCTT AATCTAAAAC TTTAAAGAAA   
  
  
- AAGTTAAACC AAAAACACCT CAATTGGGCT AGATTATATA AATTAAAAAA AAGAACTAAA ATTAGAAAAT   
  
  
- GTAACCTAGA CAATCTTTTA CCTAGAAACA TTAAAAAATA AACTAAAAAA ACTTCAATAG AGTCAGAATA   
  
  
- CTAGATCCAG TGCTCGAATT GTATAATCGG ATTCAATTGA ACTCAACAAA AAAAATCAAC TAAAAAAAAT   
  
  
- AAAGTAGTAA GTTGTAATTC AATCAACATT TAATCCAAAG TATTAAAAAA ATATATACGA AAGATAACTC   
  
  
- AATATTTTAT AGGGCTAGAG TACTAGGCCC AACGCTCAAA TTGTAGAATT GAATGTAATT GAACCCATCA   
  
  
- AAAAAAATAG GAAAAAAATT AAATTATAAA AAAAAAGTTA AGGTGGGAAG TTATAGTTTA ACTAATCTCT   
  
  
- AACTAGAAGT ATTAAATAAA ACTAAATGAA AGATATATAT TCCAATAATA CCAAAGAACT AAATTCAAAG   
  
  
- TTTAAACTGT CTAATTGAAC ATAGTTAGAA TCTACAAAAA TAAAATTATA AATTTTTTAT GTAGGTTATA   
  
  
- TGAAATACAA AACTTAAATA TTAATTAAAA AAATAAACTT TTATACAATT GTAGCATCCA TATAAAAAAA   
  
  
- TATAATTTTT TAATTAACCT AAGTCTCAGT ATTTTTATTA GTCCAAAAGA GATTTTTAGC CATACGTAGT   
  
  
- GAAAGTTTTG TACTTGCGAG TGTAAGAGGG TTGCCTCCTT TTTACGGAGA TCTACAACAG TTTTCTTCTG   
  
  
- CGGAAATATT AATGTTAAAA AATATGGGTA AAAGGTATAG GGTTTTCCGA CTACTAATAC CGTAAAAGCC   
  
  
- TAAAAATTTT TTTTCTTTTT TTTACTATTT ATGTCTCCAC AAATCGTAAC TTTTCTTTTT ATTTAATTTC   
  
  
- CTGGACGGCA GTTATTTTAA ACCGTCGTTT CTTCTTATAT TAACTGGTCA GTTACTCCTG GACTATTGAT   
  
  
- ATTTTTTTCT CCGCGTACCT GCCCGCGTGG GTGTACAAT

+     TC-rich repeats

| Site Name | Organism | Position | Strand | Matrix score. | sequence | function |
| --- | --- | --- | --- | --- | --- | --- |
| TC-rich repeats | Nicotiana tabacum | 1061 | - | 9 | ATTCTCTAAC | cis-acting element involved in defense and stress responsiveness |

>Potri.006G138900.1   
+ TGATACCAGA TGATGTGTCG TGAATGATTT GAAAATCGAA AGCAATGAAT CTGACGAAAA TGGATGAAGG   
  
  
+ ATGGGTCTGG TTAGTTGTCT TTCTTTTGGT GTTTAGGCTG GATTATAGTG ATTTTAAAGT AAATAAGATG   
  
  
+ GAGGATAGAC TAGAATGATA CTTTGATAAG TCGATCTGGA CGCCACAAAG ATCAATCTAG GATTTCGACA   
  
  
+ CCTATAAAAT ATTTGGATTA GTGTTTTGTG TTTTTTAAAA AACTTTTTTT TGTTTAGTTT TTTTTTCATT   
  
  
+ TCACCGTTCA ACAACTCAAT CTTTTTCTTC TTTTTATAAT TTTTTTTCTA TTTCATCTTT AAAAATTAGG   
  
  
+ TTGTTTTGAA AATTTTGCTT CATATTTTTT TTTTCTGTTG GGTTATATAT TAGTCTCATG GATTTAATTT   
  
  
+ TATTTTCTCG ATTTCAACCT TCAACACTAG ATCTATTGGA AGTGGGGTTT CATAATATTT TTTTTATTTA   
  
  
+ TTTTCTATGA ATTTTTCTTG ATCTCATGAC TAGGTCACAA GTTTAACAAG TTAACTCAAG TTGACTGAGA   
  
  
+ TCATTTTCTT ATTGCTTTTT TTAATTGATT TTTTTATTTC ATCCTTCAAT ATTGGGTTGG ATGGAAATTG   
  
  
+ AGATTCATAT ATTTTTTCTA TTTACTTTCT ATGGGGTTAT CCTGATCTCA TGTGAGTTTA GCAGGTTAAG   
  
  
+ TCGGGTTGAC TTTATTTATT TATTTTTCTT CTTTTTTAAT TGGACTTTGG AATTTTTTTT ATTTTATTTT   
  
  
+ TATGGGATTA TCCAGATCTT ATGACTCAAG TCACGAGTTT AGTTGGTTGA CCTGAGTTGA CTTGATTTAT   
  
  
+ TATTTGGGTT CCTTTTTTAA TTGAATGTTT TTTTTTCTAT TTCACCTTCC AACAACTCAA TTGTTTTATT   
  
  
+ TCACGTTTTT TTTCTTCAGT TTCATCCTTC AATATTAAGT TGTTTGGGAA TTAGATTTTG AAATTTCTTT   
  
  
+ TTCAATTTGG TTTTTGTGGA GTTAACCCGA TCTAATATAT TTAATTTTTT TTCTTGATTT TAATCTTTTA   
  
  
+ CATTGGATCT GTTAGAAAAT GGATCTTTGT AATTTTTTAT TTGATTTTTT TGAAGTTATC TCAGTCTTAT   
  
  
+ GATCTAGGTC ACGAGCTTAA CATATTAGCC TAAGTTAACT TGAGTTGTTT TTTTTAGTTG ATTTTTTTTA   
  
  
+ TTTCATCATT CAACATTAAG TTAGTTGTAA ATTAGGTTTC ATAATTTTTT TATATATGCT TTCTATTGAG   
  
  
+ TTATAAAATA TCCCGATCTC ATGATCCGGG TTGCGAGTTT AACATCTTAA CTTACATTAA CTTGGGTAGT   
  
  
+ TTTTTTTATC CTTTTTTTAA TTTAATATTT TTTTTTCAAT TCCACCCTTC AATATCAAAT TGATTAGAGA   
  
  
+ TTGATCTTCA TAATTTATTT TGATTTACTT TCTATATATA AGGTTATTAT GGTTTCTTGA TTTAAGTTTC   
  
  
+ AAATTTGACA GATTAACTTG TATCAATCTT AGATGTTTTT ATTTTAATAT TTAAAAAATA CATCCAATAT   
  
  
+ ACTTTATGTT TTGAATTTAT AATTAATTTT TTTATTTGAA AATATGTTAA CATCGTAGGT ATATTTTTTT   
  
  
+ ATATTAAAAA ATTAATTGGA TTCAGAGTCA TAAAAATAAT CAGGTTTTCT CTAAAAATCG GTATGCATCA   
  
  
+ CTTTCAAAAC ATGAACGCTC ACATTCTCCC AACGGAGGAA AAATGCCTCT AGATGTTGTC AAAAGAAGAC   
  
  
+ GCCTTTATAA TTACAATTTT TTATACCCAT TTTCCATATC CCAAAAGGCT GATGATTATG GCATTTTCGG   
  
  
+ ATTTTTAAAA AAAAGAAAAA AAATGATAAA TACAGAGGTG TTTAGCATTG AAAAGAAAAA TAAATTAAAG   
  
  
+ GACCTGCCGT CAATAAAATT TGGCAGCAAA GAAGAATATA ATTGACCAGT CAATGAGGAC CTGATAACTA   
  
  
+ TAAAAAAAGA GGCGCATGGA CGGGCGCACC CACATGTTA  

- ACTATGGTCT ACTACACAGC ACTTACTAAA CTTTTAGCTT TCGTTACTTA GACTGCTTTT ACCTACTTCC   
  
  
- TACCCAGACC AATCAACAGA AAGAAAACCA CAAATCCGAC CTAATATCAC TAAAATTTCA TTTATTCTAC   
  
  
- CTCCTATCTG ATCTTACTAT GAAACTATTC AGCTAGACCT GCGGTGTTTC TAGTTAGATC CTAAAGCTGT   
  
  
- GGATATTTTA TAAACCTAAT CACAAAACAC AAAAAATTTT TTGAAAAAAA ACAAATCAAA AAAAAAGTAA   
  
  
- AGTGGCAAGT TGTTGAGTTA GAAAAAGAAG AAAAATATTA AAAAAAAGAT AAAGTAGAAA TTTTTAATCC   
  
  
- AACAAAACTT TTAAAACGAA GTATAAAAAA AAAAGACAAC CCAATATATA ATCAGAGTAC CTAAATTAAA   
  
  
- ATAAAAGAGC TAAAGTTGGA AGTTGTGATC TAGATAACCT TCACCCCAAA GTATTATAAA AAAAATAAAT   
  
  
- AAAAGATACT TAAAAAGAAC TAGAGTACTG ATCCAGTGTT CAAATTGTTC AATTGAGTTC AACTGACTCT   
  
  
- AGTAAAAGAA TAACGAAAAA AATTAACTAA AAAAATAAAG TAGGAAGTTA TAACCCAACC TACCTTTAAC   
  
  
- TCTAAGTATA TAAAAAAGAT AAATGAAAGA TACCCCAATA GGACTAGAGT ACACTCAAAT CGTCCAATTC   
  
  
- AGCCCAACTG AAATAAATAA ATAAAAAGAA GAAAAAATTA ACCTGAAACC TTAAAAAAAA TAAAATAAAA   
  
  
- ATACCCTAAT AGGTCTAGAA TACTGAGTTC AGTGCTCAAA TCAACCAACT GGACTCAACT GAACTAAATA   
  
  
- ATAAACCCAA GGAAAAAATT AACTTACAAA AAAAAAGATA AAGTGGAAGG TTGTTGAGTT AACAAAATAA   
  
  
- AGTGCAAAAA AAAGAAGTCA AAGTAGGAAG TTATAATTCA ACAAACCCTT AATCTAAAAC TTTAAAGAAA   
  
  
- AAGTTAAACC AAAAACACCT CAATTGGGCT AGATTATATA AATTAAAAAA AAGAACTAAA ATTAGAAAAT   
  
  
- GTAACCTAGA CAATCTTTTA CCTAGAAACA TTAAAAAATA AACTAAAAAA ACTTCAATAG AGTCAGAATA   
  
  
- CTAGATCCAG TGCTCGAATT GTATAATCGG ATTCAATTGA ACTCAACAAA AAAAATCAAC TAAAAAAAAT   
  
  
- AAAGTAGTAA GTTGTAATTC AATCAACATT TAATCCAAAG TATTAAAAAA ATATATACGA AAGATAACTC   
  
  
- AATATTTTAT AGGGCTAGAG TACTAGGCCC AACGCTCAAA TTGTAGAATT GAATGTAATT GAACCCATCA   
  
  
- AAAAAAATAG GAAAAAAATT AAATTATAAA AAAAAAGTTA AGGTGGGAAG TTATAGTTTA ACTAATCTCT   
  
  
- AACTAGAAGT ATTAAATAAA ACTAAATGAA AGATATATAT TCCAATAATA CCAAAGAACT AAATTCAAAG   
  
  
- TTTAAACTGT CTAATTGAAC ATAGTTAGAA TCTACAAAAA TAAAATTATA AATTTTTTAT GTAGGTTATA   
  
  
- TGAAATACAA AACTTAAATA TTAATTAAAA AAATAAACTT TTATACAATT GTAGCATCCA TATAAAAAAA   
  
  
- TATAATTTTT TAATTAACCT AAGTCTCAGT ATTTTTATTA GTCCAAAAGA GATTTTTAGC CATACGTAGT   
  
  
- GAAAGTTTTG TACTTGCGAG TGTAAGAGGG TTGCCTCCTT TTTACGGAGA TCTACAACAG TTTTCTTCTG   
  
  
- CGGAAATATT AATGTTAAAA AATATGGGTA AAAGGTATAG GGTTTTCCGA CTACTAATAC CGTAAAAGCC   
  
  
- TAAAAATTTT TTTTCTTTTT TTTACTATTT ATGTCTCCAC AAATCGTAAC TTTTCTTTTT ATTTAATTTC   
  
  
- CTGGACGGCA GTTATTTTAA ACCGTCGTTT CTTCTTATAT TAACTGGTCA GTTACTCCTG GACTATTGAT   
  
  
- ATTTTTTTCT CCGCGTACCT GCCCGCGTGG GTGTACAAT

+     TCA

| Site Name | Organism | Position | Strand | Matrix score. | sequence | function |
| --- | --- | --- | --- | --- | --- | --- |
| TCA | Pisum sativum | 1402 | + | 9 | TCATCTTCAT |  |

>Potri.006G138900.1   
+ TGATACCAGA TGATGTGTCG TGAATGATTT GAAAATCGAA AGCAATGAAT CTGACGAAAA TGGATGAAGG   
  
  
+ ATGGGTCTGG TTAGTTGTCT TTCTTTTGGT GTTTAGGCTG GATTATAGTG ATTTTAAAGT AAATAAGATG   
  
  
+ GAGGATAGAC TAGAATGATA CTTTGATAAG TCGATCTGGA CGCCACAAAG ATCAATCTAG GATTTCGACA   
  
  
+ CCTATAAAAT ATTTGGATTA GTGTTTTGTG TTTTTTAAAA AACTTTTTTT TGTTTAGTTT TTTTTTCATT   
  
  
+ TCACCGTTCA ACAACTCAAT CTTTTTCTTC TTTTTATAAT TTTTTTTCTA TTTCATCTTT AAAAATTAGG   
  
  
+ TTGTTTTGAA AATTTTGCTT CATATTTTTT TTTTCTGTTG GGTTATATAT TAGTCTCATG GATTTAATTT   
  
  
+ TATTTTCTCG ATTTCAACCT TCAACACTAG ATCTATTGGA AGTGGGGTTT CATAATATTT TTTTTATTTA   
  
  
+ TTTTCTATGA ATTTTTCTTG ATCTCATGAC TAGGTCACAA GTTTAACAAG TTAACTCAAG TTGACTGAGA   
  
  
+ TCATTTTCTT ATTGCTTTTT TTAATTGATT TTTTTATTTC ATCCTTCAAT ATTGGGTTGG ATGGAAATTG   
  
  
+ AGATTCATAT ATTTTTTCTA TTTACTTTCT ATGGGGTTAT CCTGATCTCA TGTGAGTTTA GCAGGTTAAG   
  
  
+ TCGGGTTGAC TTTATTTATT TATTTTTCTT CTTTTTTAAT TGGACTTTGG AATTTTTTTT ATTTTATTTT   
  
  
+ TATGGGATTA TCCAGATCTT ATGACTCAAG TCACGAGTTT AGTTGGTTGA CCTGAGTTGA CTTGATTTAT   
  
  
+ TATTTGGGTT CCTTTTTTAA TTGAATGTTT TTTTTTCTAT TTCACCTTCC AACAACTCAA TTGTTTTATT   
  
  
+ TCACGTTTTT TTTCTTCAGT TTCATCCTTC AATATTAAGT TGTTTGGGAA TTAGATTTTG AAATTTCTTT   
  
  
+ TTCAATTTGG TTTTTGTGGA GTTAACCCGA TCTAATATAT TTAATTTTTT TTCTTGATTT TAATCTTTTA   
  
  
+ CATTGGATCT GTTAGAAAAT GGATCTTTGT AATTTTTTAT TTGATTTTTT TGAAGTTATC TCAGTCTTAT   
  
  
+ GATCTAGGTC ACGAGCTTAA CATATTAGCC TAAGTTAACT TGAGTTGTTT TTTTTAGTTG ATTTTTTTTA   
  
  
+ TTTCATCATT CAACATTAAG TTAGTTGTAA ATTAGGTTTC ATAATTTTTT TATATATGCT TTCTATTGAG   
  
  
+ TTATAAAATA TCCCGATCTC ATGATCCGGG TTGCGAGTTT AACATCTTAA CTTACATTAA CTTGGGTAGT   
  
  
+ TTTTTTTATC CTTTTTTTAA TTTAATATTT TTTTTTCAAT TCCACCCTTC AATATCAAAT TGATTAGAGA   
  
  
+ TTGATCTTCA TAATTTATTT TGATTTACTT TCTATATATA AGGTTATTAT GGTTTCTTGA TTTAAGTTTC   
  
  
+ AAATTTGACA GATTAACTTG TATCAATCTT AGATGTTTTT ATTTTAATAT TTAAAAAATA CATCCAATAT   
  
  
+ ACTTTATGTT TTGAATTTAT AATTAATTTT TTTATTTGAA AATATGTTAA CATCGTAGGT ATATTTTTTT   
  
  
+ ATATTAAAAA ATTAATTGGA TTCAGAGTCA TAAAAATAAT CAGGTTTTCT CTAAAAATCG GTATGCATCA   
  
  
+ CTTTCAAAAC ATGAACGCTC ACATTCTCCC AACGGAGGAA AAATGCCTCT AGATGTTGTC AAAAGAAGAC   
  
  
+ GCCTTTATAA TTACAATTTT TTATACCCAT TTTCCATATC CCAAAAGGCT GATGATTATG GCATTTTCGG   
  
  
+ ATTTTTAAAA AAAAGAAAAA AAATGATAAA TACAGAGGTG TTTAGCATTG AAAAGAAAAA TAAATTAAAG   
  
  
+ GACCTGCCGT CAATAAAATT TGGCAGCAAA GAAGAATATA ATTGACCAGT CAATGAGGAC CTGATAACTA   
  
  
+ TAAAAAAAGA GGCGCATGGA CGGGCGCACC CACATGTTA  

- ACTATGGTCT ACTACACAGC ACTTACTAAA CTTTTAGCTT TCGTTACTTA GACTGCTTTT ACCTACTTCC   
  
  
- TACCCAGACC AATCAACAGA AAGAAAACCA CAAATCCGAC CTAATATCAC TAAAATTTCA TTTATTCTAC   
  
  
- CTCCTATCTG ATCTTACTAT GAAACTATTC AGCTAGACCT GCGGTGTTTC TAGTTAGATC CTAAAGCTGT   
  
  
- GGATATTTTA TAAACCTAAT CACAAAACAC AAAAAATTTT TTGAAAAAAA ACAAATCAAA AAAAAAGTAA   
  
  
- AGTGGCAAGT TGTTGAGTTA GAAAAAGAAG AAAAATATTA AAAAAAAGAT AAAGTAGAAA TTTTTAATCC   
  
  
- AACAAAACTT TTAAAACGAA GTATAAAAAA AAAAGACAAC CCAATATATA ATCAGAGTAC CTAAATTAAA   
  
  
- ATAAAAGAGC TAAAGTTGGA AGTTGTGATC TAGATAACCT TCACCCCAAA GTATTATAAA AAAAATAAAT   
  
  
- AAAAGATACT TAAAAAGAAC TAGAGTACTG ATCCAGTGTT CAAATTGTTC AATTGAGTTC AACTGACTCT   
  
  
- AGTAAAAGAA TAACGAAAAA AATTAACTAA AAAAATAAAG TAGGAAGTTA TAACCCAACC TACCTTTAAC   
  
  
- TCTAAGTATA TAAAAAAGAT AAATGAAAGA TACCCCAATA GGACTAGAGT ACACTCAAAT CGTCCAATTC   
  
  
- AGCCCAACTG AAATAAATAA ATAAAAAGAA GAAAAAATTA ACCTGAAACC TTAAAAAAAA TAAAATAAAA   
  
  
- ATACCCTAAT AGGTCTAGAA TACTGAGTTC AGTGCTCAAA TCAACCAACT GGACTCAACT GAACTAAATA   
  
  
- ATAAACCCAA GGAAAAAATT AACTTACAAA AAAAAAGATA AAGTGGAAGG TTGTTGAGTT AACAAAATAA   
  
  
- AGTGCAAAAA AAAGAAGTCA AAGTAGGAAG TTATAATTCA ACAAACCCTT AATCTAAAAC TTTAAAGAAA   
  
  
- AAGTTAAACC AAAAACACCT CAATTGGGCT AGATTATATA AATTAAAAAA AAGAACTAAA ATTAGAAAAT   
  
  
- GTAACCTAGA CAATCTTTTA CCTAGAAACA TTAAAAAATA AACTAAAAAA ACTTCAATAG AGTCAGAATA   
  
  
- CTAGATCCAG TGCTCGAATT GTATAATCGG ATTCAATTGA ACTCAACAAA AAAAATCAAC TAAAAAAAAT   
  
  
- AAAGTAGTAA GTTGTAATTC AATCAACATT TAATCCAAAG TATTAAAAAA ATATATACGA AAGATAACTC   
  
  
- AATATTTTAT AGGGCTAGAG TACTAGGCCC AACGCTCAAA TTGTAGAATT GAATGTAATT GAACCCATCA   
  
  
- AAAAAAATAG GAAAAAAATT AAATTATAAA AAAAAAGTTA AGGTGGGAAG TTATAGTTTA ACTAATCTCT   
  
  
- AACTAGAAGT ATTAAATAAA ACTAAATGAA AGATATATAT TCCAATAATA CCAAAGAACT AAATTCAAAG   
  
  
- TTTAAACTGT CTAATTGAAC ATAGTTAGAA TCTACAAAAA TAAAATTATA AATTTTTTAT GTAGGTTATA   
  
  
- TGAAATACAA AACTTAAATA TTAATTAAAA AAATAAACTT TTATACAATT GTAGCATCCA TATAAAAAAA   
  
  
- TATAATTTTT TAATTAACCT AAGTCTCAGT ATTTTTATTA GTCCAAAAGA GATTTTTAGC CATACGTAGT   
  
  
- GAAAGTTTTG TACTTGCGAG TGTAAGAGGG TTGCCTCCTT TTTACGGAGA TCTACAACAG TTTTCTTCTG   
  
  
- CGGAAATATT AATGTTAAAA AATATGGGTA AAAGGTATAG GGTTTTCCGA CTACTAATAC CGTAAAAGCC   
  
  
- TAAAAATTTT TTTTCTTTTT TTTACTATTT ATGTCTCCAC AAATCGTAAC TTTTCTTTTT ATTTAATTTC   
  
  
- CTGGACGGCA GTTATTTTAA ACCGTCGTTT CTTCTTATAT TAACTGGTCA GTTACTCCTG GACTATTGAT   
  
  
- ATTTTTTTCT CCGCGTACCT GCCCGCGTGG GTGTACAAT

+     TCA-element

| Site Name | Organism | Position | Strand | Matrix score. | sequence | function |
| --- | --- | --- | --- | --- | --- | --- |
| TCA-element | Nicotiana tabacum | 132 | - | 9 | CCATCTTTTT | cis-acting element involved in salicylic acid responsiveness |
| TCA-element | Nicotiana tabacum | 297 | + | 9 | CCATCTTTTT | cis-acting element involved in salicylic acid responsiveness |

>Potri.006G138900.1   
+ TGATACCAGA TGATGTGTCG TGAATGATTT GAAAATCGAA AGCAATGAAT CTGACGAAAA TGGATGAAGG   
  
  
+ ATGGGTCTGG TTAGTTGTCT TTCTTTTGGT GTTTAGGCTG GATTATAGTG ATTTTAAAGT AAATAAGATG   
  
  
+ GAGGATAGAC TAGAATGATA CTTTGATAAG TCGATCTGGA CGCCACAAAG ATCAATCTAG GATTTCGACA   
  
  
+ CCTATAAAAT ATTTGGATTA GTGTTTTGTG TTTTTTAAAA AACTTTTTTT TGTTTAGTTT TTTTTTCATT   
  
  
+ TCACCGTTCA ACAACTCAAT CTTTTTCTTC TTTTTATAAT TTTTTTTCTA TTTCATCTTT AAAAATTAGG   
  
  
+ TTGTTTTGAA AATTTTGCTT CATATTTTTT TTTTCTGTTG GGTTATATAT TAGTCTCATG GATTTAATTT   
  
  
+ TATTTTCTCG ATTTCAACCT TCAACACTAG ATCTATTGGA AGTGGGGTTT CATAATATTT TTTTTATTTA   
  
  
+ TTTTCTATGA ATTTTTCTTG ATCTCATGAC TAGGTCACAA GTTTAACAAG TTAACTCAAG TTGACTGAGA   
  
  
+ TCATTTTCTT ATTGCTTTTT TTAATTGATT TTTTTATTTC ATCCTTCAAT ATTGGGTTGG ATGGAAATTG   
  
  
+ AGATTCATAT ATTTTTTCTA TTTACTTTCT ATGGGGTTAT CCTGATCTCA TGTGAGTTTA GCAGGTTAAG   
  
  
+ TCGGGTTGAC TTTATTTATT TATTTTTCTT CTTTTTTAAT TGGACTTTGG AATTTTTTTT ATTTTATTTT   
  
  
+ TATGGGATTA TCCAGATCTT ATGACTCAAG TCACGAGTTT AGTTGGTTGA CCTGAGTTGA CTTGATTTAT   
  
  
+ TATTTGGGTT CCTTTTTTAA TTGAATGTTT TTTTTTCTAT TTCACCTTCC AACAACTCAA TTGTTTTATT   
  
  
+ TCACGTTTTT TTTCTTCAGT TTCATCCTTC AATATTAAGT TGTTTGGGAA TTAGATTTTG AAATTTCTTT   
  
  
+ TTCAATTTGG TTTTTGTGGA GTTAACCCGA TCTAATATAT TTAATTTTTT TTCTTGATTT TAATCTTTTA   
  
  
+ CATTGGATCT GTTAGAAAAT GGATCTTTGT AATTTTTTAT TTGATTTTTT TGAAGTTATC TCAGTCTTAT   
  
  
+ GATCTAGGTC ACGAGCTTAA CATATTAGCC TAAGTTAACT TGAGTTGTTT TTTTTAGTTG ATTTTTTTTA   
  
  
+ TTTCATCATT CAACATTAAG TTAGTTGTAA ATTAGGTTTC ATAATTTTTT TATATATGCT TTCTATTGAG   
  
  
+ TTATAAAATA TCCCGATCTC ATGATCCGGG TTGCGAGTTT AACATCTTAA CTTACATTAA CTTGGGTAGT   
  
  
+ TTTTTTTATC CTTTTTTTAA TTTAATATTT TTTTTTCAAT TCCACCCTTC AATATCAAAT TGATTAGAGA   
  
  
+ TTGATCTTCA TAATTTATTT TGATTTACTT TCTATATATA AGGTTATTAT GGTTTCTTGA TTTAAGTTTC   
  
  
+ AAATTTGACA GATTAACTTG TATCAATCTT AGATGTTTTT ATTTTAATAT TTAAAAAATA CATCCAATAT   
  
  
+ ACTTTATGTT TTGAATTTAT AATTAATTTT TTTATTTGAA AATATGTTAA CATCGTAGGT ATATTTTTTT   
  
  
+ ATATTAAAAA ATTAATTGGA TTCAGAGTCA TAAAAATAAT CAGGTTTTCT CTAAAAATCG GTATGCATCA   
  
  
+ CTTTCAAAAC ATGAACGCTC ACATTCTCCC AACGGAGGAA AAATGCCTCT AGATGTTGTC AAAAGAAGAC   
  
  
+ GCCTTTATAA TTACAATTTT TTATACCCAT TTTCCATATC CCAAAAGGCT GATGATTATG GCATTTTCGG   
  
  
+ ATTTTTAAAA AAAAGAAAAA AAATGATAAA TACAGAGGTG TTTAGCATTG AAAAGAAAAA TAAATTAAAG   
  
  
+ GACCTGCCGT CAATAAAATT TGGCAGCAAA GAAGAATATA ATTGACCAGT CAATGAGGAC CTGATAACTA   
  
  
+ TAAAAAAAGA GGCGCATGGA CGGGCGCACC CACATGTTA  

- ACTATGGTCT ACTACACAGC ACTTACTAAA CTTTTAGCTT TCGTTACTTA GACTGCTTTT ACCTACTTCC   
  
  
- TACCCAGACC AATCAACAGA AAGAAAACCA CAAATCCGAC CTAATATCAC TAAAATTTCA TTTATTCTAC   
  
  
- CTCCTATCTG ATCTTACTAT GAAACTATTC AGCTAGACCT GCGGTGTTTC TAGTTAGATC CTAAAGCTGT   
  
  
- GGATATTTTA TAAACCTAAT CACAAAACAC AAAAAATTTT TTGAAAAAAA ACAAATCAAA AAAAAAGTAA   
  
  
- AGTGGCAAGT TGTTGAGTTA GAAAAAGAAG AAAAATATTA AAAAAAAGAT AAAGTAGAAA TTTTTAATCC   
  
  
- AACAAAACTT TTAAAACGAA GTATAAAAAA AAAAGACAAC CCAATATATA ATCAGAGTAC CTAAATTAAA   
  
  
- ATAAAAGAGC TAAAGTTGGA AGTTGTGATC TAGATAACCT TCACCCCAAA GTATTATAAA AAAAATAAAT   
  
  
- AAAAGATACT TAAAAAGAAC TAGAGTACTG ATCCAGTGTT CAAATTGTTC AATTGAGTTC AACTGACTCT   
  
  
- AGTAAAAGAA TAACGAAAAA AATTAACTAA AAAAATAAAG TAGGAAGTTA TAACCCAACC TACCTTTAAC   
  
  
- TCTAAGTATA TAAAAAAGAT AAATGAAAGA TACCCCAATA GGACTAGAGT ACACTCAAAT CGTCCAATTC   
  
  
- AGCCCAACTG AAATAAATAA ATAAAAAGAA GAAAAAATTA ACCTGAAACC TTAAAAAAAA TAAAATAAAA   
  
  
- ATACCCTAAT AGGTCTAGAA TACTGAGTTC AGTGCTCAAA TCAACCAACT GGACTCAACT GAACTAAATA   
  
  
- ATAAACCCAA GGAAAAAATT AACTTACAAA AAAAAAGATA AAGTGGAAGG TTGTTGAGTT AACAAAATAA   
  
  
- AGTGCAAAAA AAAGAAGTCA AAGTAGGAAG TTATAATTCA ACAAACCCTT AATCTAAAAC TTTAAAGAAA   
  
  
- AAGTTAAACC AAAAACACCT CAATTGGGCT AGATTATATA AATTAAAAAA AAGAACTAAA ATTAGAAAAT   
  
  
- GTAACCTAGA CAATCTTTTA CCTAGAAACA TTAAAAAATA AACTAAAAAA ACTTCAATAG AGTCAGAATA   
  
  
- CTAGATCCAG TGCTCGAATT GTATAATCGG ATTCAATTGA ACTCAACAAA AAAAATCAAC TAAAAAAAAT   
  
  
- AAAGTAGTAA GTTGTAATTC AATCAACATT TAATCCAAAG TATTAAAAAA ATATATACGA AAGATAACTC   
  
  
- AATATTTTAT AGGGCTAGAG TACTAGGCCC AACGCTCAAA TTGTAGAATT GAATGTAATT GAACCCATCA   
  
  
- AAAAAAATAG GAAAAAAATT AAATTATAAA AAAAAAGTTA AGGTGGGAAG TTATAGTTTA ACTAATCTCT   
  
  
- AACTAGAAGT ATTAAATAAA ACTAAATGAA AGATATATAT TCCAATAATA CCAAAGAACT AAATTCAAAG   
  
  
- TTTAAACTGT CTAATTGAAC ATAGTTAGAA TCTACAAAAA TAAAATTATA AATTTTTTAT GTAGGTTATA   
  
  
- TGAAATACAA AACTTAAATA TTAATTAAAA AAATAAACTT TTATACAATT GTAGCATCCA TATAAAAAAA   
  
  
- TATAATTTTT TAATTAACCT AAGTCTCAGT ATTTTTATTA GTCCAAAAGA GATTTTTAGC CATACGTAGT   
  
  
- GAAAGTTTTG TACTTGCGAG TGTAAGAGGG TTGCCTCCTT TTTACGGAGA TCTACAACAG TTTTCTTCTG   
  
  
- CGGAAATATT AATGTTAAAA AATATGGGTA AAAGGTATAG GGTTTTCCGA CTACTAATAC CGTAAAAGCC   
  
  
- TAAAAATTTT TTTTCTTTTT TTTACTATTT ATGTCTCCAC AAATCGTAAC TTTTCTTTTT ATTTAATTTC   
  
  
- CTGGACGGCA GTTATTTTAA ACCGTCGTTT CTTCTTATAT TAACTGGTCA GTTACTCCTG GACTATTGAT   
  
  
- ATTTTTTTCT CCGCGTACCT GCCCGCGTGG GTGTACAAT

+     TGACG-motif

| Site Name | Organism | Position | Strand | Matrix score. | sequence | function |
| --- | --- | --- | --- | --- | --- | --- |
| TGACG-motif | Hordeum vulgare | 1898 | - | 5 | TGACG | cis-acting regulatory element involved in the MeJA-responsiveness |
| TGACG-motif | Hordeum vulgare | 52 | + | 5 | TGACG | cis-acting regulatory element involved in the MeJA-responsiveness |

>Potri.006G138900.1   
+ TGATACCAGA TGATGTGTCG TGAATGATTT GAAAATCGAA AGCAATGAAT CTGACGAAAA TGGATGAAGG   
  
  
+ ATGGGTCTGG TTAGTTGTCT TTCTTTTGGT GTTTAGGCTG GATTATAGTG ATTTTAAAGT AAATAAGATG   
  
  
+ GAGGATAGAC TAGAATGATA CTTTGATAAG TCGATCTGGA CGCCACAAAG ATCAATCTAG GATTTCGACA   
  
  
+ CCTATAAAAT ATTTGGATTA GTGTTTTGTG TTTTTTAAAA AACTTTTTTT TGTTTAGTTT TTTTTTCATT   
  
  
+ TCACCGTTCA ACAACTCAAT CTTTTTCTTC TTTTTATAAT TTTTTTTCTA TTTCATCTTT AAAAATTAGG   
  
  
+ TTGTTTTGAA AATTTTGCTT CATATTTTTT TTTTCTGTTG GGTTATATAT TAGTCTCATG GATTTAATTT   
  
  
+ TATTTTCTCG ATTTCAACCT TCAACACTAG ATCTATTGGA AGTGGGGTTT CATAATATTT TTTTTATTTA   
  
  
+ TTTTCTATGA ATTTTTCTTG ATCTCATGAC TAGGTCACAA GTTTAACAAG TTAACTCAAG TTGACTGAGA   
  
  
+ TCATTTTCTT ATTGCTTTTT TTAATTGATT TTTTTATTTC ATCCTTCAAT ATTGGGTTGG ATGGAAATTG   
  
  
+ AGATTCATAT ATTTTTTCTA TTTACTTTCT ATGGGGTTAT CCTGATCTCA TGTGAGTTTA GCAGGTTAAG   
  
  
+ TCGGGTTGAC TTTATTTATT TATTTTTCTT CTTTTTTAAT TGGACTTTGG AATTTTTTTT ATTTTATTTT   
  
  
+ TATGGGATTA TCCAGATCTT ATGACTCAAG TCACGAGTTT AGTTGGTTGA CCTGAGTTGA CTTGATTTAT   
  
  
+ TATTTGGGTT CCTTTTTTAA TTGAATGTTT TTTTTTCTAT TTCACCTTCC AACAACTCAA TTGTTTTATT   
  
  
+ TCACGTTTTT TTTCTTCAGT TTCATCCTTC AATATTAAGT TGTTTGGGAA TTAGATTTTG AAATTTCTTT   
  
  
+ TTCAATTTGG TTTTTGTGGA GTTAACCCGA TCTAATATAT TTAATTTTTT TTCTTGATTT TAATCTTTTA   
  
  
+ CATTGGATCT GTTAGAAAAT GGATCTTTGT AATTTTTTAT TTGATTTTTT TGAAGTTATC TCAGTCTTAT   
  
  
+ GATCTAGGTC ACGAGCTTAA CATATTAGCC TAAGTTAACT TGAGTTGTTT TTTTTAGTTG ATTTTTTTTA   
  
  
+ TTTCATCATT CAACATTAAG TTAGTTGTAA ATTAGGTTTC ATAATTTTTT TATATATGCT TTCTATTGAG   
  
  
+ TTATAAAATA TCCCGATCTC ATGATCCGGG TTGCGAGTTT AACATCTTAA CTTACATTAA CTTGGGTAGT   
  
  
+ TTTTTTTATC CTTTTTTTAA TTTAATATTT TTTTTTCAAT TCCACCCTTC AATATCAAAT TGATTAGAGA   
  
  
+ TTGATCTTCA TAATTTATTT TGATTTACTT TCTATATATA AGGTTATTAT GGTTTCTTGA TTTAAGTTTC   
  
  
+ AAATTTGACA GATTAACTTG TATCAATCTT AGATGTTTTT ATTTTAATAT TTAAAAAATA CATCCAATAT   
  
  
+ ACTTTATGTT TTGAATTTAT AATTAATTTT TTTATTTGAA AATATGTTAA CATCGTAGGT ATATTTTTTT   
  
  
+ ATATTAAAAA ATTAATTGGA TTCAGAGTCA TAAAAATAAT CAGGTTTTCT CTAAAAATCG GTATGCATCA   
  
  
+ CTTTCAAAAC ATGAACGCTC ACATTCTCCC AACGGAGGAA AAATGCCTCT AGATGTTGTC AAAAGAAGAC   
  
  
+ GCCTTTATAA TTACAATTTT TTATACCCAT TTTCCATATC CCAAAAGGCT GATGATTATG GCATTTTCGG   
  
  
+ ATTTTTAAAA AAAAGAAAAA AAATGATAAA TACAGAGGTG TTTAGCATTG AAAAGAAAAA TAAATTAAAG   
  
  
+ GACCTGCCGT CAATAAAATT TGGCAGCAAA GAAGAATATA ATTGACCAGT CAATGAGGAC CTGATAACTA   
  
  
+ TAAAAAAAGA GGCGCATGGA CGGGCGCACC CACATGTTA  

- ACTATGGTCT ACTACACAGC ACTTACTAAA CTTTTAGCTT TCGTTACTTA GACTGCTTTT ACCTACTTCC   
  
  
- TACCCAGACC AATCAACAGA AAGAAAACCA CAAATCCGAC CTAATATCAC TAAAATTTCA TTTATTCTAC   
  
  
- CTCCTATCTG ATCTTACTAT GAAACTATTC AGCTAGACCT GCGGTGTTTC TAGTTAGATC CTAAAGCTGT   
  
  
- GGATATTTTA TAAACCTAAT CACAAAACAC AAAAAATTTT TTGAAAAAAA ACAAATCAAA AAAAAAGTAA   
  
  
- AGTGGCAAGT TGTTGAGTTA GAAAAAGAAG AAAAATATTA AAAAAAAGAT AAAGTAGAAA TTTTTAATCC   
  
  
- AACAAAACTT TTAAAACGAA GTATAAAAAA AAAAGACAAC CCAATATATA ATCAGAGTAC CTAAATTAAA   
  
  
- ATAAAAGAGC TAAAGTTGGA AGTTGTGATC TAGATAACCT TCACCCCAAA GTATTATAAA AAAAATAAAT   
  
  
- AAAAGATACT TAAAAAGAAC TAGAGTACTG ATCCAGTGTT CAAATTGTTC AATTGAGTTC AACTGACTCT   
  
  
- AGTAAAAGAA TAACGAAAAA AATTAACTAA AAAAATAAAG TAGGAAGTTA TAACCCAACC TACCTTTAAC   
  
  
- TCTAAGTATA TAAAAAAGAT AAATGAAAGA TACCCCAATA GGACTAGAGT ACACTCAAAT CGTCCAATTC   
  
  
- AGCCCAACTG AAATAAATAA ATAAAAAGAA GAAAAAATTA ACCTGAAACC TTAAAAAAAA TAAAATAAAA   
  
  
- ATACCCTAAT AGGTCTAGAA TACTGAGTTC AGTGCTCAAA TCAACCAACT GGACTCAACT GAACTAAATA   
  
  
- ATAAACCCAA GGAAAAAATT AACTTACAAA AAAAAAGATA AAGTGGAAGG TTGTTGAGTT AACAAAATAA   
  
  
- AGTGCAAAAA AAAGAAGTCA AAGTAGGAAG TTATAATTCA ACAAACCCTT AATCTAAAAC TTTAAAGAAA   
  
  
- AAGTTAAACC AAAAACACCT CAATTGGGCT AGATTATATA AATTAAAAAA AAGAACTAAA ATTAGAAAAT   
  
  
- GTAACCTAGA CAATCTTTTA CCTAGAAACA TTAAAAAATA AACTAAAAAA ACTTCAATAG AGTCAGAATA   
  
  
- CTAGATCCAG TGCTCGAATT GTATAATCGG ATTCAATTGA ACTCAACAAA AAAAATCAAC TAAAAAAAAT   
  
  
- AAAGTAGTAA GTTGTAATTC AATCAACATT TAATCCAAAG TATTAAAAAA ATATATACGA AAGATAACTC   
  
  
- AATATTTTAT AGGGCTAGAG TACTAGGCCC AACGCTCAAA TTGTAGAATT GAATGTAATT GAACCCATCA   
  
  
- AAAAAAATAG GAAAAAAATT AAATTATAAA AAAAAAGTTA AGGTGGGAAG TTATAGTTTA ACTAATCTCT   
  
  
- AACTAGAAGT ATTAAATAAA ACTAAATGAA AGATATATAT TCCAATAATA CCAAAGAACT AAATTCAAAG   
  
  
- TTTAAACTGT CTAATTGAAC ATAGTTAGAA TCTACAAAAA TAAAATTATA AATTTTTTAT GTAGGTTATA   
  
  
- TGAAATACAA AACTTAAATA TTAATTAAAA AAATAAACTT TTATACAATT GTAGCATCCA TATAAAAAAA   
  
  
- TATAATTTTT TAATTAACCT AAGTCTCAGT ATTTTTATTA GTCCAAAAGA GATTTTTAGC CATACGTAGT   
  
  
- GAAAGTTTTG TACTTGCGAG TGTAAGAGGG TTGCCTCCTT TTTACGGAGA TCTACAACAG TTTTCTTCTG   
  
  
- CGGAAATATT AATGTTAAAA AATATGGGTA AAAGGTATAG GGTTTTCCGA CTACTAATAC CGTAAAAGCC   
  
  
- TAAAAATTTT TTTTCTTTTT TTTACTATTT ATGTCTCCAC AAATCGTAAC TTTTCTTTTT ATTTAATTTC   
  
  
- CTGGACGGCA GTTATTTTAA ACCGTCGTTT CTTCTTATAT TAACTGGTCA GTTACTCCTG GACTATTGAT   
  
  
- ATTTTTTTCT CCGCGTACCT GCCCGCGTGG GTGTACAAT

+     Unnamed\_\_4

| Site Name | Organism | Position | Strand | Matrix score. | sequence | function |
| --- | --- | --- | --- | --- | --- | --- |
| Unnamed\_\_4 | Petroselinum hortense | 140 | - | 4 | CTCC |  |
| Unnamed\_\_4 | Petroselinum hortense | 1706 | + | 4 | CTCC |  |
| Unnamed\_\_4 | Petroselinum hortense | 998 | - | 4 | CTCC |  |
| Unnamed\_\_4 | Petroselinum hortense | 1714 | - | 4 | CTCC |  |

>Potri.006G138900.1   
+ TGATACCAGA TGATGTGTCG TGAATGATTT GAAAATCGAA AGCAATGAAT CTGACGAAAA TGGATGAAGG   
  
  
+ ATGGGTCTGG TTAGTTGTCT TTCTTTTGGT GTTTAGGCTG GATTATAGTG ATTTTAAAGT AAATAAGATG   
  
  
+ GAGGATAGAC TAGAATGATA CTTTGATAAG TCGATCTGGA CGCCACAAAG ATCAATCTAG GATTTCGACA   
  
  
+ CCTATAAAAT ATTTGGATTA GTGTTTTGTG TTTTTTAAAA AACTTTTTTT TGTTTAGTTT TTTTTTCATT   
  
  
+ TCACCGTTCA ACAACTCAAT CTTTTTCTTC TTTTTATAAT TTTTTTTCTA TTTCATCTTT AAAAATTAGG   
  
  
+ TTGTTTTGAA AATTTTGCTT CATATTTTTT TTTTCTGTTG GGTTATATAT TAGTCTCATG GATTTAATTT   
  
  
+ TATTTTCTCG ATTTCAACCT TCAACACTAG ATCTATTGGA AGTGGGGTTT CATAATATTT TTTTTATTTA   
  
  
+ TTTTCTATGA ATTTTTCTTG ATCTCATGAC TAGGTCACAA GTTTAACAAG TTAACTCAAG TTGACTGAGA   
  
  
+ TCATTTTCTT ATTGCTTTTT TTAATTGATT TTTTTATTTC ATCCTTCAAT ATTGGGTTGG ATGGAAATTG   
  
  
+ AGATTCATAT ATTTTTTCTA TTTACTTTCT ATGGGGTTAT CCTGATCTCA TGTGAGTTTA GCAGGTTAAG   
  
  
+ TCGGGTTGAC TTTATTTATT TATTTTTCTT CTTTTTTAAT TGGACTTTGG AATTTTTTTT ATTTTATTTT   
  
  
+ TATGGGATTA TCCAGATCTT ATGACTCAAG TCACGAGTTT AGTTGGTTGA CCTGAGTTGA CTTGATTTAT   
  
  
+ TATTTGGGTT CCTTTTTTAA TTGAATGTTT TTTTTTCTAT TTCACCTTCC AACAACTCAA TTGTTTTATT   
  
  
+ TCACGTTTTT TTTCTTCAGT TTCATCCTTC AATATTAAGT TGTTTGGGAA TTAGATTTTG AAATTTCTTT   
  
  
+ TTCAATTTGG TTTTTGTGGA GTTAACCCGA TCTAATATAT TTAATTTTTT TTCTTGATTT TAATCTTTTA   
  
  
+ CATTGGATCT GTTAGAAAAT GGATCTTTGT AATTTTTTAT TTGATTTTTT TGAAGTTATC TCAGTCTTAT   
  
  
+ GATCTAGGTC ACGAGCTTAA CATATTAGCC TAAGTTAACT TGAGTTGTTT TTTTTAGTTG ATTTTTTTTA   
  
  
+ TTTCATCATT CAACATTAAG TTAGTTGTAA ATTAGGTTTC ATAATTTTTT TATATATGCT TTCTATTGAG   
  
  
+ TTATAAAATA TCCCGATCTC ATGATCCGGG TTGCGAGTTT AACATCTTAA CTTACATTAA CTTGGGTAGT   
  
  
+ TTTTTTTATC CTTTTTTTAA TTTAATATTT TTTTTTCAAT TCCACCCTTC AATATCAAAT TGATTAGAGA   
  
  
+ TTGATCTTCA TAATTTATTT TGATTTACTT TCTATATATA AGGTTATTAT GGTTTCTTGA TTTAAGTTTC   
  
  
+ AAATTTGACA GATTAACTTG TATCAATCTT AGATGTTTTT ATTTTAATAT TTAAAAAATA CATCCAATAT   
  
  
+ ACTTTATGTT TTGAATTTAT AATTAATTTT TTTATTTGAA AATATGTTAA CATCGTAGGT ATATTTTTTT   
  
  
+ ATATTAAAAA ATTAATTGGA TTCAGAGTCA TAAAAATAAT CAGGTTTTCT CTAAAAATCG GTATGCATCA   
  
  
+ CTTTCAAAAC ATGAACGCTC ACATTCTCCC AACGGAGGAA AAATGCCTCT AGATGTTGTC AAAAGAAGAC   
  
  
+ GCCTTTATAA TTACAATTTT TTATACCCAT TTTCCATATC CCAAAAGGCT GATGATTATG GCATTTTCGG   
  
  
+ ATTTTTAAAA AAAAGAAAAA AAATGATAAA TACAGAGGTG TTTAGCATTG AAAAGAAAAA TAAATTAAAG   
  
  
+ GACCTGCCGT CAATAAAATT TGGCAGCAAA GAAGAATATA ATTGACCAGT CAATGAGGAC CTGATAACTA   
  
  
+ TAAAAAAAGA GGCGCATGGA CGGGCGCACC CACATGTTA  

- ACTATGGTCT ACTACACAGC ACTTACTAAA CTTTTAGCTT TCGTTACTTA GACTGCTTTT ACCTACTTCC   
  
  
- TACCCAGACC AATCAACAGA AAGAAAACCA CAAATCCGAC CTAATATCAC TAAAATTTCA TTTATTCTAC   
  
  
- CTCCTATCTG ATCTTACTAT GAAACTATTC AGCTAGACCT GCGGTGTTTC TAGTTAGATC CTAAAGCTGT   
  
  
- GGATATTTTA TAAACCTAAT CACAAAACAC AAAAAATTTT TTGAAAAAAA ACAAATCAAA AAAAAAGTAA   
  
  
- AGTGGCAAGT TGTTGAGTTA GAAAAAGAAG AAAAATATTA AAAAAAAGAT AAAGTAGAAA TTTTTAATCC   
  
  
- AACAAAACTT TTAAAACGAA GTATAAAAAA AAAAGACAAC CCAATATATA ATCAGAGTAC CTAAATTAAA   
  
  
- ATAAAAGAGC TAAAGTTGGA AGTTGTGATC TAGATAACCT TCACCCCAAA GTATTATAAA AAAAATAAAT   
  
  
- AAAAGATACT TAAAAAGAAC TAGAGTACTG ATCCAGTGTT CAAATTGTTC AATTGAGTTC AACTGACTCT   
  
  
- AGTAAAAGAA TAACGAAAAA AATTAACTAA AAAAATAAAG TAGGAAGTTA TAACCCAACC TACCTTTAAC   
  
  
- TCTAAGTATA TAAAAAAGAT AAATGAAAGA TACCCCAATA GGACTAGAGT ACACTCAAAT CGTCCAATTC   
  
  
- AGCCCAACTG AAATAAATAA ATAAAAAGAA GAAAAAATTA ACCTGAAACC TTAAAAAAAA TAAAATAAAA   
  
  
- ATACCCTAAT AGGTCTAGAA TACTGAGTTC AGTGCTCAAA TCAACCAACT GGACTCAACT GAACTAAATA   
  
  
- ATAAACCCAA GGAAAAAATT AACTTACAAA AAAAAAGATA AAGTGGAAGG TTGTTGAGTT AACAAAATAA   
  
  
- AGTGCAAAAA AAAGAAGTCA AAGTAGGAAG TTATAATTCA ACAAACCCTT AATCTAAAAC TTTAAAGAAA   
  
  
- AAGTTAAACC AAAAACACCT CAATTGGGCT AGATTATATA AATTAAAAAA AAGAACTAAA ATTAGAAAAT   
  
  
- GTAACCTAGA CAATCTTTTA CCTAGAAACA TTAAAAAATA AACTAAAAAA ACTTCAATAG AGTCAGAATA   
  
  
- CTAGATCCAG TGCTCGAATT GTATAATCGG ATTCAATTGA ACTCAACAAA AAAAATCAAC TAAAAAAAAT   
  
  
- AAAGTAGTAA GTTGTAATTC AATCAACATT TAATCCAAAG TATTAAAAAA ATATATACGA AAGATAACTC   
  
  
- AATATTTTAT AGGGCTAGAG TACTAGGCCC AACGCTCAAA TTGTAGAATT GAATGTAATT GAACCCATCA   
  
  
- AAAAAAATAG GAAAAAAATT AAATTATAAA AAAAAAGTTA AGGTGGGAAG TTATAGTTTA ACTAATCTCT   
  
  
- AACTAGAAGT ATTAAATAAA ACTAAATGAA AGATATATAT TCCAATAATA CCAAAGAACT AAATTCAAAG   
  
  
- TTTAAACTGT CTAATTGAAC ATAGTTAGAA TCTACAAAAA TAAAATTATA AATTTTTTAT GTAGGTTATA   
  
  
- TGAAATACAA AACTTAAATA TTAATTAAAA AAATAAACTT TTATACAATT GTAGCATCCA TATAAAAAAA   
  
  
- TATAATTTTT TAATTAACCT AAGTCTCAGT ATTTTTATTA GTCCAAAAGA GATTTTTAGC CATACGTAGT   
  
  
- GAAAGTTTTG TACTTGCGAG TGTAAGAGGG TTGCCTCCTT TTTACGGAGA TCTACAACAG TTTTCTTCTG   
  
  
- CGGAAATATT AATGTTAAAA AATATGGGTA AAAGGTATAG GGTTTTCCGA CTACTAATAC CGTAAAAGCC   
  
  
- TAAAAATTTT TTTTCTTTTT TTTACTATTT ATGTCTCCAC AAATCGTAAC TTTTCTTTTT ATTTAATTTC   
  
  
- CTGGACGGCA GTTATTTTAA ACCGTCGTTT CTTCTTATAT TAACTGGTCA GTTACTCCTG GACTATTGAT   
  
  
- ATTTTTTTCT CCGCGTACCT GCCCGCGTGG GTGTACAAT

+     W box

| Site Name | Organism | Position | Strand | Matrix score. | sequence | function |
| --- | --- | --- | --- | --- | --- | --- |
| W box | Arabidopsis thaliana | 817 | + | 6 | TTGACC |  |
| W box | Arabidopsis thaliana | 1932 | + | 6 | TTGACC |  |

>Potri.006G138900.1   
+ TGATACCAGA TGATGTGTCG TGAATGATTT GAAAATCGAA AGCAATGAAT CTGACGAAAA TGGATGAAGG   
  
  
+ ATGGGTCTGG TTAGTTGTCT TTCTTTTGGT GTTTAGGCTG GATTATAGTG ATTTTAAAGT AAATAAGATG   
  
  
+ GAGGATAGAC TAGAATGATA CTTTGATAAG TCGATCTGGA CGCCACAAAG ATCAATCTAG GATTTCGACA   
  
  
+ CCTATAAAAT ATTTGGATTA GTGTTTTGTG TTTTTTAAAA AACTTTTTTT TGTTTAGTTT TTTTTTCATT   
  
  
+ TCACCGTTCA ACAACTCAAT CTTTTTCTTC TTTTTATAAT TTTTTTTCTA TTTCATCTTT AAAAATTAGG   
  
  
+ TTGTTTTGAA AATTTTGCTT CATATTTTTT TTTTCTGTTG GGTTATATAT TAGTCTCATG GATTTAATTT   
  
  
+ TATTTTCTCG ATTTCAACCT TCAACACTAG ATCTATTGGA AGTGGGGTTT CATAATATTT TTTTTATTTA   
  
  
+ TTTTCTATGA ATTTTTCTTG ATCTCATGAC TAGGTCACAA GTTTAACAAG TTAACTCAAG TTGACTGAGA   
  
  
+ TCATTTTCTT ATTGCTTTTT TTAATTGATT TTTTTATTTC ATCCTTCAAT ATTGGGTTGG ATGGAAATTG   
  
  
+ AGATTCATAT ATTTTTTCTA TTTACTTTCT ATGGGGTTAT CCTGATCTCA TGTGAGTTTA GCAGGTTAAG   
  
  
+ TCGGGTTGAC TTTATTTATT TATTTTTCTT CTTTTTTAAT TGGACTTTGG AATTTTTTTT ATTTTATTTT   
  
  
+ TATGGGATTA TCCAGATCTT ATGACTCAAG TCACGAGTTT AGTTGGTTGA CCTGAGTTGA CTTGATTTAT   
  
  
+ TATTTGGGTT CCTTTTTTAA TTGAATGTTT TTTTTTCTAT TTCACCTTCC AACAACTCAA TTGTTTTATT   
  
  
+ TCACGTTTTT TTTCTTCAGT TTCATCCTTC AATATTAAGT TGTTTGGGAA TTAGATTTTG AAATTTCTTT   
  
  
+ TTCAATTTGG TTTTTGTGGA GTTAACCCGA TCTAATATAT TTAATTTTTT TTCTTGATTT TAATCTTTTA   
  
  
+ CATTGGATCT GTTAGAAAAT GGATCTTTGT AATTTTTTAT TTGATTTTTT TGAAGTTATC TCAGTCTTAT   
  
  
+ GATCTAGGTC ACGAGCTTAA CATATTAGCC TAAGTTAACT TGAGTTGTTT TTTTTAGTTG ATTTTTTTTA   
  
  
+ TTTCATCATT CAACATTAAG TTAGTTGTAA ATTAGGTTTC ATAATTTTTT TATATATGCT TTCTATTGAG   
  
  
+ TTATAAAATA TCCCGATCTC ATGATCCGGG TTGCGAGTTT AACATCTTAA CTTACATTAA CTTGGGTAGT   
  
  
+ TTTTTTTATC CTTTTTTTAA TTTAATATTT TTTTTTCAAT TCCACCCTTC AATATCAAAT TGATTAGAGA   
  
  
+ TTGATCTTCA TAATTTATTT TGATTTACTT TCTATATATA AGGTTATTAT GGTTTCTTGA TTTAAGTTTC   
  
  
+ AAATTTGACA GATTAACTTG TATCAATCTT AGATGTTTTT ATTTTAATAT TTAAAAAATA CATCCAATAT   
  
  
+ ACTTTATGTT TTGAATTTAT AATTAATTTT TTTATTTGAA AATATGTTAA CATCGTAGGT ATATTTTTTT   
  
  
+ ATATTAAAAA ATTAATTGGA TTCAGAGTCA TAAAAATAAT CAGGTTTTCT CTAAAAATCG GTATGCATCA   
  
  
+ CTTTCAAAAC ATGAACGCTC ACATTCTCCC AACGGAGGAA AAATGCCTCT AGATGTTGTC AAAAGAAGAC   
  
  
+ GCCTTTATAA TTACAATTTT TTATACCCAT TTTCCATATC CCAAAAGGCT GATGATTATG GCATTTTCGG   
  
  
+ ATTTTTAAAA AAAAGAAAAA AAATGATAAA TACAGAGGTG TTTAGCATTG AAAAGAAAAA TAAATTAAAG   
  
  
+ GACCTGCCGT CAATAAAATT TGGCAGCAAA GAAGAATATA ATTGACCAGT CAATGAGGAC CTGATAACTA   
  
  
+ TAAAAAAAGA GGCGCATGGA CGGGCGCACC CACATGTTA  

- ACTATGGTCT ACTACACAGC ACTTACTAAA CTTTTAGCTT TCGTTACTTA GACTGCTTTT ACCTACTTCC   
  
  
- TACCCAGACC AATCAACAGA AAGAAAACCA CAAATCCGAC CTAATATCAC TAAAATTTCA TTTATTCTAC   
  
  
- CTCCTATCTG ATCTTACTAT GAAACTATTC AGCTAGACCT GCGGTGTTTC TAGTTAGATC CTAAAGCTGT   
  
  
- GGATATTTTA TAAACCTAAT CACAAAACAC AAAAAATTTT TTGAAAAAAA ACAAATCAAA AAAAAAGTAA   
  
  
- AGTGGCAAGT TGTTGAGTTA GAAAAAGAAG AAAAATATTA AAAAAAAGAT AAAGTAGAAA TTTTTAATCC   
  
  
- AACAAAACTT TTAAAACGAA GTATAAAAAA AAAAGACAAC CCAATATATA ATCAGAGTAC CTAAATTAAA   
  
  
- ATAAAAGAGC TAAAGTTGGA AGTTGTGATC TAGATAACCT TCACCCCAAA GTATTATAAA AAAAATAAAT   
  
  
- AAAAGATACT TAAAAAGAAC TAGAGTACTG ATCCAGTGTT CAAATTGTTC AATTGAGTTC AACTGACTCT   
  
  
- AGTAAAAGAA TAACGAAAAA AATTAACTAA AAAAATAAAG TAGGAAGTTA TAACCCAACC TACCTTTAAC   
  
  
- TCTAAGTATA TAAAAAAGAT AAATGAAAGA TACCCCAATA GGACTAGAGT ACACTCAAAT CGTCCAATTC   
  
  
- AGCCCAACTG AAATAAATAA ATAAAAAGAA GAAAAAATTA ACCTGAAACC TTAAAAAAAA TAAAATAAAA   
  
  
- ATACCCTAAT AGGTCTAGAA TACTGAGTTC AGTGCTCAAA TCAACCAACT GGACTCAACT GAACTAAATA   
  
  
- ATAAACCCAA GGAAAAAATT AACTTACAAA AAAAAAGATA AAGTGGAAGG TTGTTGAGTT AACAAAATAA   
  
  
- AGTGCAAAAA AAAGAAGTCA AAGTAGGAAG TTATAATTCA ACAAACCCTT AATCTAAAAC TTTAAAGAAA   
  
  
- AAGTTAAACC AAAAACACCT CAATTGGGCT AGATTATATA AATTAAAAAA AAGAACTAAA ATTAGAAAAT   
  
  
- GTAACCTAGA CAATCTTTTA CCTAGAAACA TTAAAAAATA AACTAAAAAA ACTTCAATAG AGTCAGAATA   
  
  
- CTAGATCCAG TGCTCGAATT GTATAATCGG ATTCAATTGA ACTCAACAAA AAAAATCAAC TAAAAAAAAT   
  
  
- AAAGTAGTAA GTTGTAATTC AATCAACATT TAATCCAAAG TATTAAAAAA ATATATACGA AAGATAACTC   
  
  
- AATATTTTAT AGGGCTAGAG TACTAGGCCC AACGCTCAAA TTGTAGAATT GAATGTAATT GAACCCATCA   
  
  
- AAAAAAATAG GAAAAAAATT AAATTATAAA AAAAAAGTTA AGGTGGGAAG TTATAGTTTA ACTAATCTCT   
  
  
- AACTAGAAGT ATTAAATAAA ACTAAATGAA AGATATATAT TCCAATAATA CCAAAGAACT AAATTCAAAG   
  
  
- TTTAAACTGT CTAATTGAAC ATAGTTAGAA TCTACAAAAA TAAAATTATA AATTTTTTAT GTAGGTTATA   
  
  
- TGAAATACAA AACTTAAATA TTAATTAAAA AAATAAACTT TTATACAATT GTAGCATCCA TATAAAAAAA   
  
  
- TATAATTTTT TAATTAACCT AAGTCTCAGT ATTTTTATTA GTCCAAAAGA GATTTTTAGC CATACGTAGT   
  
  
- GAAAGTTTTG TACTTGCGAG TGTAAGAGGG TTGCCTCCTT TTTACGGAGA TCTACAACAG TTTTCTTCTG   
  
  
- CGGAAATATT AATGTTAAAA AATATGGGTA AAAGGTATAG GGTTTTCCGA CTACTAATAC CGTAAAAGCC   
  
  
- TAAAAATTTT TTTTCTTTTT TTTACTATTT ATGTCTCCAC AAATCGTAAC TTTTCTTTTT ATTTAATTTC   
  
  
- CTGGACGGCA GTTATTTTAA ACCGTCGTTT CTTCTTATAT TAACTGGTCA GTTACTCCTG GACTATTGAT   
  
  
- ATTTTTTTCT CCGCGTACCT GCCCGCGTGG GTGTACAAT

+     WUN-motif

| Site Name | Organism | Position | Strand | Matrix score. | sequence | function |
| --- | --- | --- | --- | --- | --- | --- |
| WUN-motif | Nicotiana glutinosa | 971 | + | 9 | AAATTTCTT |  |

>Potri.006G138900.1   
+ TGATACCAGA TGATGTGTCG TGAATGATTT GAAAATCGAA AGCAATGAAT CTGACGAAAA TGGATGAAGG   
  
  
+ ATGGGTCTGG TTAGTTGTCT TTCTTTTGGT GTTTAGGCTG GATTATAGTG ATTTTAAAGT AAATAAGATG   
  
  
+ GAGGATAGAC TAGAATGATA CTTTGATAAG TCGATCTGGA CGCCACAAAG ATCAATCTAG GATTTCGACA   
  
  
+ CCTATAAAAT ATTTGGATTA GTGTTTTGTG TTTTTTAAAA AACTTTTTTT TGTTTAGTTT TTTTTTCATT   
  
  
+ TCACCGTTCA ACAACTCAAT CTTTTTCTTC TTTTTATAAT TTTTTTTCTA TTTCATCTTT AAAAATTAGG   
  
  
+ TTGTTTTGAA AATTTTGCTT CATATTTTTT TTTTCTGTTG GGTTATATAT TAGTCTCATG GATTTAATTT   
  
  
+ TATTTTCTCG ATTTCAACCT TCAACACTAG ATCTATTGGA AGTGGGGTTT CATAATATTT TTTTTATTTA   
  
  
+ TTTTCTATGA ATTTTTCTTG ATCTCATGAC TAGGTCACAA GTTTAACAAG TTAACTCAAG TTGACTGAGA   
  
  
+ TCATTTTCTT ATTGCTTTTT TTAATTGATT TTTTTATTTC ATCCTTCAAT ATTGGGTTGG ATGGAAATTG   
  
  
+ AGATTCATAT ATTTTTTCTA TTTACTTTCT ATGGGGTTAT CCTGATCTCA TGTGAGTTTA GCAGGTTAAG   
  
  
+ TCGGGTTGAC TTTATTTATT TATTTTTCTT CTTTTTTAAT TGGACTTTGG AATTTTTTTT ATTTTATTTT   
  
  
+ TATGGGATTA TCCAGATCTT ATGACTCAAG TCACGAGTTT AGTTGGTTGA CCTGAGTTGA CTTGATTTAT   
  
  
+ TATTTGGGTT CCTTTTTTAA TTGAATGTTT TTTTTTCTAT TTCACCTTCC AACAACTCAA TTGTTTTATT   
  
  
+ TCACGTTTTT TTTCTTCAGT TTCATCCTTC AATATTAAGT TGTTTGGGAA TTAGATTTTG AAATTTCTTT   
  
  
+ TTCAATTTGG TTTTTGTGGA GTTAACCCGA TCTAATATAT TTAATTTTTT TTCTTGATTT TAATCTTTTA   
  
  
+ CATTGGATCT GTTAGAAAAT GGATCTTTGT AATTTTTTAT TTGATTTTTT TGAAGTTATC TCAGTCTTAT   
  
  
+ GATCTAGGTC ACGAGCTTAA CATATTAGCC TAAGTTAACT TGAGTTGTTT TTTTTAGTTG ATTTTTTTTA   
  
  
+ TTTCATCATT CAACATTAAG TTAGTTGTAA ATTAGGTTTC ATAATTTTTT TATATATGCT TTCTATTGAG   
  
  
+ TTATAAAATA TCCCGATCTC ATGATCCGGG TTGCGAGTTT AACATCTTAA CTTACATTAA CTTGGGTAGT   
  
  
+ TTTTTTTATC CTTTTTTTAA TTTAATATTT TTTTTTCAAT TCCACCCTTC AATATCAAAT TGATTAGAGA   
  
  
+ TTGATCTTCA TAATTTATTT TGATTTACTT TCTATATATA AGGTTATTAT GGTTTCTTGA TTTAAGTTTC   
  
  
+ AAATTTGACA GATTAACTTG TATCAATCTT AGATGTTTTT ATTTTAATAT TTAAAAAATA CATCCAATAT   
  
  
+ ACTTTATGTT TTGAATTTAT AATTAATTTT TTTATTTGAA AATATGTTAA CATCGTAGGT ATATTTTTTT   
  
  
+ ATATTAAAAA ATTAATTGGA TTCAGAGTCA TAAAAATAAT CAGGTTTTCT CTAAAAATCG GTATGCATCA   
  
  
+ CTTTCAAAAC ATGAACGCTC ACATTCTCCC AACGGAGGAA AAATGCCTCT AGATGTTGTC AAAAGAAGAC   
  
  
+ GCCTTTATAA TTACAATTTT TTATACCCAT TTTCCATATC CCAAAAGGCT GATGATTATG GCATTTTCGG   
  
  
+ ATTTTTAAAA AAAAGAAAAA AAATGATAAA TACAGAGGTG TTTAGCATTG AAAAGAAAAA TAAATTAAAG   
  
  
+ GACCTGCCGT CAATAAAATT TGGCAGCAAA GAAGAATATA ATTGACCAGT CAATGAGGAC CTGATAACTA   
  
  
+ TAAAAAAAGA GGCGCATGGA CGGGCGCACC CACATGTTA  

- ACTATGGTCT ACTACACAGC ACTTACTAAA CTTTTAGCTT TCGTTACTTA GACTGCTTTT ACCTACTTCC   
  
  
- TACCCAGACC AATCAACAGA AAGAAAACCA CAAATCCGAC CTAATATCAC TAAAATTTCA TTTATTCTAC   
  
  
- CTCCTATCTG ATCTTACTAT GAAACTATTC AGCTAGACCT GCGGTGTTTC TAGTTAGATC CTAAAGCTGT   
  
  
- GGATATTTTA TAAACCTAAT CACAAAACAC AAAAAATTTT TTGAAAAAAA ACAAATCAAA AAAAAAGTAA   
  
  
- AGTGGCAAGT TGTTGAGTTA GAAAAAGAAG AAAAATATTA AAAAAAAGAT AAAGTAGAAA TTTTTAATCC   
  
  
- AACAAAACTT TTAAAACGAA GTATAAAAAA AAAAGACAAC CCAATATATA ATCAGAGTAC CTAAATTAAA   
  
  
- ATAAAAGAGC TAAAGTTGGA AGTTGTGATC TAGATAACCT TCACCCCAAA GTATTATAAA AAAAATAAAT   
  
  
- AAAAGATACT TAAAAAGAAC TAGAGTACTG ATCCAGTGTT CAAATTGTTC AATTGAGTTC AACTGACTCT   
  
  
- AGTAAAAGAA TAACGAAAAA AATTAACTAA AAAAATAAAG TAGGAAGTTA TAACCCAACC TACCTTTAAC   
  
  
- TCTAAGTATA TAAAAAAGAT AAATGAAAGA TACCCCAATA GGACTAGAGT ACACTCAAAT CGTCCAATTC   
  
  
- AGCCCAACTG AAATAAATAA ATAAAAAGAA GAAAAAATTA ACCTGAAACC TTAAAAAAAA TAAAATAAAA   
  
  
- ATACCCTAAT AGGTCTAGAA TACTGAGTTC AGTGCTCAAA TCAACCAACT GGACTCAACT GAACTAAATA   
  
  
- ATAAACCCAA GGAAAAAATT AACTTACAAA AAAAAAGATA AAGTGGAAGG TTGTTGAGTT AACAAAATAA   
  
  
- AGTGCAAAAA AAAGAAGTCA AAGTAGGAAG TTATAATTCA ACAAACCCTT AATCTAAAAC TTTAAAGAAA   
  
  
- AAGTTAAACC AAAAACACCT CAATTGGGCT AGATTATATA AATTAAAAAA AAGAACTAAA ATTAGAAAAT   
  
  
- GTAACCTAGA CAATCTTTTA CCTAGAAACA TTAAAAAATA AACTAAAAAA ACTTCAATAG AGTCAGAATA   
  
  
- CTAGATCCAG TGCTCGAATT GTATAATCGG ATTCAATTGA ACTCAACAAA AAAAATCAAC TAAAAAAAAT   
  
  
- AAAGTAGTAA GTTGTAATTC AATCAACATT TAATCCAAAG TATTAAAAAA ATATATACGA AAGATAACTC   
  
  
- AATATTTTAT AGGGCTAGAG TACTAGGCCC AACGCTCAAA TTGTAGAATT GAATGTAATT GAACCCATCA   
  
  
- AAAAAAATAG GAAAAAAATT AAATTATAAA AAAAAAGTTA AGGTGGGAAG TTATAGTTTA ACTAATCTCT   
  
  
- AACTAGAAGT ATTAAATAAA ACTAAATGAA AGATATATAT TCCAATAATA CCAAAGAACT AAATTCAAAG   
  
  
- TTTAAACTGT CTAATTGAAC ATAGTTAGAA TCTACAAAAA TAAAATTATA AATTTTTTAT GTAGGTTATA   
  
  
- TGAAATACAA AACTTAAATA TTAATTAAAA AAATAAACTT TTATACAATT GTAGCATCCA TATAAAAAAA   
  
  
- TATAATTTTT TAATTAACCT AAGTCTCAGT ATTTTTATTA GTCCAAAAGA GATTTTTAGC CATACGTAGT   
  
  
- GAAAGTTTTG TACTTGCGAG TGTAAGAGGG TTGCCTCCTT TTTACGGAGA TCTACAACAG TTTTCTTCTG   
  
  
- CGGAAATATT AATGTTAAAA AATATGGGTA AAAGGTATAG GGTTTTCCGA CTACTAATAC CGTAAAAGCC   
  
  
- TAAAAATTTT TTTTCTTTTT TTTACTATTT ATGTCTCCAC AAATCGTAAC TTTTCTTTTT ATTTAATTTC   
  
  
- CTGGACGGCA GTTATTTTAA ACCGTCGTTT CTTCTTATAT TAACTGGTCA GTTACTCCTG GACTATTGAT   
  
  
- ATTTTTTTCT CCGCGTACCT GCCCGCGTGG GTGTACAAT

+     as-1

| Site Name | Organism | Position | Strand | Matrix score. | sequence | function |
| --- | --- | --- | --- | --- | --- | --- |
| as-1 | Arabidopsis thaliana | 52 | + | 5 | TGACG |  |
| as-1 | Arabidopsis thaliana | 1898 | - | 5 | TGACG |  |

>Potri.006G138900.1   
+ TGATACCAGA TGATGTGTCG TGAATGATTT GAAAATCGAA AGCAATGAAT CTGACGAAAA TGGATGAAGG   
  
  
+ ATGGGTCTGG TTAGTTGTCT TTCTTTTGGT GTTTAGGCTG GATTATAGTG ATTTTAAAGT AAATAAGATG   
  
  
+ GAGGATAGAC TAGAATGATA CTTTGATAAG TCGATCTGGA CGCCACAAAG ATCAATCTAG GATTTCGACA   
  
  
+ CCTATAAAAT ATTTGGATTA GTGTTTTGTG TTTTTTAAAA AACTTTTTTT TGTTTAGTTT TTTTTTCATT   
  
  
+ TCACCGTTCA ACAACTCAAT CTTTTTCTTC TTTTTATAAT TTTTTTTCTA TTTCATCTTT AAAAATTAGG   
  
  
+ TTGTTTTGAA AATTTTGCTT CATATTTTTT TTTTCTGTTG GGTTATATAT TAGTCTCATG GATTTAATTT   
  
  
+ TATTTTCTCG ATTTCAACCT TCAACACTAG ATCTATTGGA AGTGGGGTTT CATAATATTT TTTTTATTTA   
  
  
+ TTTTCTATGA ATTTTTCTTG ATCTCATGAC TAGGTCACAA GTTTAACAAG TTAACTCAAG TTGACTGAGA   
  
  
+ TCATTTTCTT ATTGCTTTTT TTAATTGATT TTTTTATTTC ATCCTTCAAT ATTGGGTTGG ATGGAAATTG   
  
  
+ AGATTCATAT ATTTTTTCTA TTTACTTTCT ATGGGGTTAT CCTGATCTCA TGTGAGTTTA GCAGGTTAAG   
  
  
+ TCGGGTTGAC TTTATTTATT TATTTTTCTT CTTTTTTAAT TGGACTTTGG AATTTTTTTT ATTTTATTTT   
  
  
+ TATGGGATTA TCCAGATCTT ATGACTCAAG TCACGAGTTT AGTTGGTTGA CCTGAGTTGA CTTGATTTAT   
  
  
+ TATTTGGGTT CCTTTTTTAA TTGAATGTTT TTTTTTCTAT TTCACCTTCC AACAACTCAA TTGTTTTATT   
  
  
+ TCACGTTTTT TTTCTTCAGT TTCATCCTTC AATATTAAGT TGTTTGGGAA TTAGATTTTG AAATTTCTTT   
  
  
+ TTCAATTTGG TTTTTGTGGA GTTAACCCGA TCTAATATAT TTAATTTTTT TTCTTGATTT TAATCTTTTA   
  
  
+ CATTGGATCT GTTAGAAAAT GGATCTTTGT AATTTTTTAT TTGATTTTTT TGAAGTTATC TCAGTCTTAT   
  
  
+ GATCTAGGTC ACGAGCTTAA CATATTAGCC TAAGTTAACT TGAGTTGTTT TTTTTAGTTG ATTTTTTTTA   
  
  
+ TTTCATCATT CAACATTAAG TTAGTTGTAA ATTAGGTTTC ATAATTTTTT TATATATGCT TTCTATTGAG   
  
  
+ TTATAAAATA TCCCGATCTC ATGATCCGGG TTGCGAGTTT AACATCTTAA CTTACATTAA CTTGGGTAGT   
  
  
+ TTTTTTTATC CTTTTTTTAA TTTAATATTT TTTTTTCAAT TCCACCCTTC AATATCAAAT TGATTAGAGA   
  
  
+ TTGATCTTCA TAATTTATTT TGATTTACTT TCTATATATA AGGTTATTAT GGTTTCTTGA TTTAAGTTTC   
  
  
+ AAATTTGACA GATTAACTTG TATCAATCTT AGATGTTTTT ATTTTAATAT TTAAAAAATA CATCCAATAT   
  
  
+ ACTTTATGTT TTGAATTTAT AATTAATTTT TTTATTTGAA AATATGTTAA CATCGTAGGT ATATTTTTTT   
  
  
+ ATATTAAAAA ATTAATTGGA TTCAGAGTCA TAAAAATAAT CAGGTTTTCT CTAAAAATCG GTATGCATCA   
  
  
+ CTTTCAAAAC ATGAACGCTC ACATTCTCCC AACGGAGGAA AAATGCCTCT AGATGTTGTC AAAAGAAGAC   
  
  
+ GCCTTTATAA TTACAATTTT TTATACCCAT TTTCCATATC CCAAAAGGCT GATGATTATG GCATTTTCGG   
  
  
+ ATTTTTAAAA AAAAGAAAAA AAATGATAAA TACAGAGGTG TTTAGCATTG AAAAGAAAAA TAAATTAAAG   
  
  
+ GACCTGCCGT CAATAAAATT TGGCAGCAAA GAAGAATATA ATTGACCAGT CAATGAGGAC CTGATAACTA   
  
  
+ TAAAAAAAGA GGCGCATGGA CGGGCGCACC CACATGTTA  

- ACTATGGTCT ACTACACAGC ACTTACTAAA CTTTTAGCTT TCGTTACTTA GACTGCTTTT ACCTACTTCC   
  
  
- TACCCAGACC AATCAACAGA AAGAAAACCA CAAATCCGAC CTAATATCAC TAAAATTTCA TTTATTCTAC   
  
  
- CTCCTATCTG ATCTTACTAT GAAACTATTC AGCTAGACCT GCGGTGTTTC TAGTTAGATC CTAAAGCTGT   
  
  
- GGATATTTTA TAAACCTAAT CACAAAACAC AAAAAATTTT TTGAAAAAAA ACAAATCAAA AAAAAAGTAA   
  
  
- AGTGGCAAGT TGTTGAGTTA GAAAAAGAAG AAAAATATTA AAAAAAAGAT AAAGTAGAAA TTTTTAATCC   
  
  
- AACAAAACTT TTAAAACGAA GTATAAAAAA AAAAGACAAC CCAATATATA ATCAGAGTAC CTAAATTAAA   
  
  
- ATAAAAGAGC TAAAGTTGGA AGTTGTGATC TAGATAACCT TCACCCCAAA GTATTATAAA AAAAATAAAT   
  
  
- AAAAGATACT TAAAAAGAAC TAGAGTACTG ATCCAGTGTT CAAATTGTTC AATTGAGTTC AACTGACTCT   
  
  
- AGTAAAAGAA TAACGAAAAA AATTAACTAA AAAAATAAAG TAGGAAGTTA TAACCCAACC TACCTTTAAC   
  
  
- TCTAAGTATA TAAAAAAGAT AAATGAAAGA TACCCCAATA GGACTAGAGT ACACTCAAAT CGTCCAATTC   
  
  
- AGCCCAACTG AAATAAATAA ATAAAAAGAA GAAAAAATTA ACCTGAAACC TTAAAAAAAA TAAAATAAAA   
  
  
- ATACCCTAAT AGGTCTAGAA TACTGAGTTC AGTGCTCAAA TCAACCAACT GGACTCAACT GAACTAAATA   
  
  
- ATAAACCCAA GGAAAAAATT AACTTACAAA AAAAAAGATA AAGTGGAAGG TTGTTGAGTT AACAAAATAA   
  
  
- AGTGCAAAAA AAAGAAGTCA AAGTAGGAAG TTATAATTCA ACAAACCCTT AATCTAAAAC TTTAAAGAAA   
  
  
- AAGTTAAACC AAAAACACCT CAATTGGGCT AGATTATATA AATTAAAAAA AAGAACTAAA ATTAGAAAAT   
  
  
- GTAACCTAGA CAATCTTTTA CCTAGAAACA TTAAAAAATA AACTAAAAAA ACTTCAATAG AGTCAGAATA   
  
  
- CTAGATCCAG TGCTCGAATT GTATAATCGG ATTCAATTGA ACTCAACAAA AAAAATCAAC TAAAAAAAAT   
  
  
- AAAGTAGTAA GTTGTAATTC AATCAACATT TAATCCAAAG TATTAAAAAA ATATATACGA AAGATAACTC   
  
  
- AATATTTTAT AGGGCTAGAG TACTAGGCCC AACGCTCAAA TTGTAGAATT GAATGTAATT GAACCCATCA   
  
  
- AAAAAAATAG GAAAAAAATT AAATTATAAA AAAAAAGTTA AGGTGGGAAG TTATAGTTTA ACTAATCTCT   
  
  
- AACTAGAAGT ATTAAATAAA ACTAAATGAA AGATATATAT TCCAATAATA CCAAAGAACT AAATTCAAAG   
  
  
- TTTAAACTGT CTAATTGAAC ATAGTTAGAA TCTACAAAAA TAAAATTATA AATTTTTTAT GTAGGTTATA   
  
  
- TGAAATACAA AACTTAAATA TTAATTAAAA AAATAAACTT TTATACAATT GTAGCATCCA TATAAAAAAA   
  
  
- TATAATTTTT TAATTAACCT AAGTCTCAGT ATTTTTATTA GTCCAAAAGA GATTTTTAGC CATACGTAGT   
  
  
- GAAAGTTTTG TACTTGCGAG TGTAAGAGGG TTGCCTCCTT TTTACGGAGA TCTACAACAG TTTTCTTCTG   
  
  
- CGGAAATATT AATGTTAAAA AATATGGGTA AAAGGTATAG GGTTTTCCGA CTACTAATAC CGTAAAAGCC   
  
  
- TAAAAATTTT TTTTCTTTTT TTTACTATTT ATGTCTCCAC AAATCGTAAC TTTTCTTTTT ATTTAATTTC   
  
  
- CTGGACGGCA GTTATTTTAA ACCGTCGTTT CTTCTTATAT TAACTGGTCA GTTACTCCTG GACTATTGAT   
  
  
- ATTTTTTTCT CCGCGTACCT GCCCGCGTGG GTGTACAAT
