# Supplementary material for: Structure and expression analysis of seven salt-related ERF genes of Populus
Source: PeerJ. 2020 Oct 20;8:e10206. doi: 10.7717/peerj.10206 (PMC7583627; doi:10.7717/peerj.10206)
Supplement: Supplemental Information 17 [file peerj-08-10206-s017.gz › Potri.011G061700.1_plantcare.html]

Content-Type: text/html; charset=ISO-8859-1


PlantCARE


Webmaster Firefox specific output  
To save the result:
click on the frame with the right mouse button and save the source code as a text file with extension .html  
REFERENCE:PlantCARE: a database of plant cis-acting regulatory elements and a portal to tools for in silico analysis of promoter sequences.  
Lescot, M., Déhais, P., Moreau, Y., De Moor, B., Rouzé ,P.,and Rombauts, S.  
Nucleic Acids Res., Database issue(2002), 30(1):325-327.   


---

>Potri.011G061700.1   
+ TGTATAATAA AACAAGTGCA TATATATCAT CATGACACAA AAATAGAAAT ATAGGCCATA ATATTAAATT   
  
  
+ AGGCATCTAT TGCACCAGCC ATGACAGACA TTTGGTTAAT AATATAATAC TCATTGATAA AGGTGTCAAT   
  
  
+ GACACAAATC TATGATCCTA GTGTTGGAAA ATTTAAATTA TTTAAATAAC ATGGTTAGTT GATATATTAT   
  
  
+ TTTTCTTATA ATTAGGTAGA TTGAGGGGGG AAATGATATT TTTATAAGTA ATAAATATTG ATAAAACAAG   
  
  
+ TAGTAGACAC CTGTGTCGCA CATGTCTACA CGTTGGTGTT GGTGTGCGAC GTTTATTAAT GCTTAAATGG   
  
  
+ TGGTTTCTAG GACTAGTATG CGGGTTTGGA CAGCACGTGC GACGTTTAAT TTCATCCTCA TTAAATTAGT   
  
  
+ GTTTATTTTT TTATTTTTTA TTTTTTATTT TTTTATTTTT TTAATTGAAT TTATTTTTTA ATTTTATCAC   
  
  
+ TCATTATTTA ATTTAATTTT ATTTGTATGT TAAATTTAGT TTTTATTCTT TTTATTTCTA TTTGTTTTAC   
  
  
+ TTTAGAAAAA TTTTTGATTA AAATTTTTTT TTTCAATATA ATCCTTTAGA TTTAGTTTTT TTTTCTTTTT   
  
  
+ AATTTTAGCT CTCAACATTT GAATTGTAAG GATGTGGTTT GACTTAAAAA AATTTCAAGA TAACATGTTT   
  
  
+ TTAAATATTG GAATGACAAC ATATTAGATC AATCCAAGTC GACTCACTAG TAAAAAAAAT AAACTTATTA   
  
  
+ AATCCACATT GGTCATGGAT CCAAGTAGAT TTAATAATTT TTTTAAACCA GTTTTTATTT AATTACATGA   
  
  
+ TAAAAAATAA GCGATAGTAG TTAAACAACC AAATTAAATT TACAAATTGA TTACAATGGA TTGTAAAAAA   
  
  
+ CACTTGCTAA TATTACAACA AATATTTCTT TTGTTTTATT CAAGAACAAA GTGAAAATGA AATGAAATTT   
  
  
+ GATAAAACAA TATAAAAAAT ATATCAATTA ACTTAATATT TTTGAAAACA ATATATAATA TATTTTATAT   
  
  
+ TCATTAATTT TTAAGAAATT AATTTTAATT ATTTGAAGAA AAATAAAAAA AATAGACCAA GCGCGAGGGC   
  
  
+ TTGCCAACCC AGGCCAACAC TTGGGTTTTT TGTTGCTTTT TAAACAAGAC AGATGACATG TCGTCTACAG   
  
  
+ AGGAGAGTGA TATGTCATCT GTAGAGTTAG ACAACATGTC ATCTGCAAAC CTATTTTTTA ACATCTAGAG   
  
  
+ AGTACTGGAA AATCTAGCTG GGTATTTGTT TTTGTCCAAA AACTTAATTT TAATTTATTT TTATCTAAAG   
  
  
+ ACACCTAGAG AAAACCATTA TGAACTCAAA AAAAACTAAT TCATCAACTT GAAAAAAAAA AACAAATACA   
  
  
+ AAATGGATTA AAAACATAAA ACTAGTTTGG GAAAAAAAAT TTCTCTCTCT AACTAGATCT AAACTTTCCA   
  
  
+ GCAATATAAA TACTTAAAAA TATTTTAATA ACATTTTTTT AACATATTGA TATCAAATTT AACCATTATT   
  
  
+ TTCAATAAAA TTCAACCAAC CAATTTCTGT CTCATCATTC GTGTTCCTAT GATTCTCTCT TTTTCTTTCA   
  
  
+ AACTCTCACA AGGTAAATAA AATTATATTA TGTGCTTGAA AGGAAATTTG AAAAATCTAA AGGAAAACTT   
  
  
+ AACTTAAAAG GTGCTGACAT TTTTTATTAA AAAAAGCTCA TATTGTTTTT TCTTTAATTT TGACTTTATT   
  
  
+ CTTTTCTTTT TCAAATAATA AAATTAGATT CGTTTCCATA AAATTTAATG AACTTTTAGG CAATAATGTT   
  
  
+ GTACGAAGTC AATAATGACG TGCTACAATC GTATGTCACG ACAAAAAGAC GACATGAACA CCATCCATTG   
  
  
+ GTATTCATGT GCCTTTTCAC CTTGTAAAAT AACAGGAGAA AGATACCTTC CAGGGGCAAA TTCATCTATG   
  
  
+ CCACGTGCCT ACAAGTTGAA GGTATGTTTG GTATGCAAG  

- ACATATTATT TTGTTCACGT ATATATAGTA GTACTGTGTT TTTATCTTTA TATCCGGTAT TATAATTTAA   
  
  
- TCCGTAGATA ACGTGGTCGG TACTGTCTGT AAACCAATTA TTATATTATG AGTAACTATT TCCACAGTTA   
  
  
- CTGTGTTTAG ATACTAGGAT CACAACCTTT TAAATTTAAT AAATTTATTG TACCAATCAA CTATATAATA   
  
  
- AAAAGAATAT TAATCCATCT AACTCCCCCC TTTACTATAA AAATATTCAT TATTTATAAC TATTTTGTTC   
  
  
- ATCATCTGTG GACACAGCGT GTACAGATGT GCAACCACAA CCACACGCTG CAAATAATTA CGAATTTACC   
  
  
- ACCAAAGATC CTGATCATAC GCCCAAACCT GTCGTGCACG CTGCAAATTA AAGTAGGAGT AATTTAATCA   
  
  
- CAAATAAAAA AATAAAAAAT AAAAAATAAA AAAATAAAAA AATTAACTTA AATAAAAAAT TAAAATAGTG   
  
  
- AGTAATAAAT TAAATTAAAA TAAACATACA ATTTAAATCA AAAATAAGAA AAATAAAGAT AAACAAAATG   
  
  
- AAATCTTTTT AAAAACTAAT TTTAAAAAAA AAAGTTATAT TAGGAAATCT AAATCAAAAA AAAAGAAAAA   
  
  
- TTAAAATCGA GAGTTGTAAA CTTAACATTC CTACACCAAA CTGAATTTTT TTAAAGTTCT ATTGTACAAA   
  
  
- AATTTATAAC CTTACTGTTG TATAATCTAG TTAGGTTCAG CTGAGTGATC ATTTTTTTTA TTTGAATAAT   
  
  
- TTAGGTGTAA CCAGTACCTA GGTTCATCTA AATTATTAAA AAAATTTGGT CAAAAATAAA TTAATGTACT   
  
  
- ATTTTTTATT CGCTATCATC AATTTGTTGG TTTAATTTAA ATGTTTAACT AATGTTACCT AACATTTTTT   
  
  
- GTGAACGATT ATAATGTTGT TTATAAAGAA AACAAAATAA GTTCTTGTTT CACTTTTACT TTACTTTAAA   
  
  
- CTATTTTGTT ATATTTTTTA TATAGTTAAT TGAATTATAA AAACTTTTGT TATATATTAT ATAAAATATA   
  
  
- AGTAATTAAA AATTCTTTAA TTAAAATTAA TAAACTTCTT TTTATTTTTT TTATCTGGTT CGCGCTCCCG   
  
  
- AACGGTTGGG TCCGGTTGTG AACCCAAAAA ACAACGAAAA ATTTGTTCTG TCTACTGTAC AGCAGATGTC   
  
  
- TCCTCTCACT ATACAGTAGA CATCTCAATC TGTTGTACAG TAGACGTTTG GATAAAAAAT TGTAGATCTC   
  
  
- TCATGACCTT TTAGATCGAC CCATAAACAA AAACAGGTTT TTGAATTAAA ATTAAATAAA AATAGATTTC   
  
  
- TGTGGATCTC TTTTGGTAAT ACTTGAGTTT TTTTTGATTA AGTAGTTGAA CTTTTTTTTT TTGTTTATGT   
  
  
- TTTACCTAAT TTTTGTATTT TGATCAAACC CTTTTTTTTA AAGAGAGAGA TTGATCTAGA TTTGAAAGGT   
  
  
- CGTTATATTT ATGAATTTTT ATAAAATTAT TGTAAAAAAA TTGTATAACT ATAGTTTAAA TTGGTAATAA   
  
  
- AAGTTATTTT AAGTTGGTTG GTTAAAGACA GAGTAGTAAG CACAAGGATA CTAAGAGAGA AAAAGAAAGT   
  
  
- TTGAGAGTGT TCCATTTATT TTAATATAAT ACACGAACTT TCCTTTAAAC TTTTTAGATT TCCTTTTGAA   
  
  
- TTGAATTTTC CACGACTGTA AAAAATAATT TTTTTCGAGT ATAACAAAAA AGAAATTAAA ACTGAAATAA   
  
  
- GAAAAGAAAA AGTTTATTAT TTTAATCTAA GCAAAGGTAT TTTAAATTAC TTGAAAATCC GTTATTACAA   
  
  
- CATGCTTCAG TTATTACTGC ACGATGTTAG CATACAGTGC TGTTTTTCTG CTGTACTTGT GGTAGGTAAC   
  
  
- CATAAGTACA CGGAAAAGTG GAACATTTTA TTGTCCTCTT TCTATGGAAG GTCCCCGTTT AAGTAGATAC   
  
  
- GGTGCACGGA TGTTCAACTT CCATACAAAC CATACGTTC

  
  
Motifs Found  

+   

| Site Name | Organism | Position | Strand | Matrix score. | sequence | function |
| --- | --- | --- | --- | --- | --- | --- |
|  | organism | 1897 | + | 4 | motif\_sequence | short\_function |
|  | organism | 1231 | + | 4 | motif\_sequence | short\_function |
|  | organism | 298 | - | 4 | motif\_sequence | short\_function |
|  | organism | 1211 | - | 4 | motif\_sequence | short\_function |
|  | organism | 226 | - | 4 | motif\_sequence | short\_function |
|  | organism | 1192 | - | 4 | motif\_sequence | short\_function |
|  | organism | 795 | - | 4 | motif\_sequence | short\_function |
|  | organism | 378 | - | 4 | motif\_sequence | short\_function |
|  | organism | 1640 | + | 4 | motif\_sequence | short\_function |

>Potri.011G061700.1   
+ TGTATAATAA AACAAGTGCA TATATATCAT CATGACACAA AAATAGAAAT ATAGGCCATA ATATTAAATT   
  
  
+ AGGCATCTAT TGCACCAGCC ATGACAGACA TTTGGTTAAT AATATAATAC TCATTGATAA AGGTGTCAAT   
  
  
+ GACACAAATC TATGATCCTA GTGTTGGAAA ATTTAAATTA TTTAAATAAC ATGGTTAGTT GATATATTAT   
  
  
+ TTTTCTTATA ATTAGGTAGA TTGAGGGGGG AAATGATATT TTTATAAGTA ATAAATATTG ATAAAACAAG   
  
  
+ TAGTAGACAC CTGTGTCGCA CATGTCTACA CGTTGGTGTT GGTGTGCGAC GTTTATTAAT GCTTAAATGG   
  
  
+ TGGTTTCTAG GACTAGTATG CGGGTTTGGA CAGCACGTGC GACGTTTAAT TTCATCCTCA TTAAATTAGT   
  
  
+ GTTTATTTTT TTATTTTTTA TTTTTTATTT TTTTATTTTT TTAATTGAAT TTATTTTTTA ATTTTATCAC   
  
  
+ TCATTATTTA ATTTAATTTT ATTTGTATGT TAAATTTAGT TTTTATTCTT TTTATTTCTA TTTGTTTTAC   
  
  
+ TTTAGAAAAA TTTTTGATTA AAATTTTTTT TTTCAATATA ATCCTTTAGA TTTAGTTTTT TTTTCTTTTT   
  
  
+ AATTTTAGCT CTCAACATTT GAATTGTAAG GATGTGGTTT GACTTAAAAA AATTTCAAGA TAACATGTTT   
  
  
+ TTAAATATTG GAATGACAAC ATATTAGATC AATCCAAGTC GACTCACTAG TAAAAAAAAT AAACTTATTA   
  
  
+ AATCCACATT GGTCATGGAT CCAAGTAGAT TTAATAATTT TTTTAAACCA GTTTTTATTT AATTACATGA   
  
  
+ TAAAAAATAA GCGATAGTAG TTAAACAACC AAATTAAATT TACAAATTGA TTACAATGGA TTGTAAAAAA   
  
  
+ CACTTGCTAA TATTACAACA AATATTTCTT TTGTTTTATT CAAGAACAAA GTGAAAATGA AATGAAATTT   
  
  
+ GATAAAACAA TATAAAAAAT ATATCAATTA ACTTAATATT TTTGAAAACA ATATATAATA TATTTTATAT   
  
  
+ TCATTAATTT TTAAGAAATT AATTTTAATT ATTTGAAGAA AAATAAAAAA AATAGACCAA GCGCGAGGGC   
  
  
+ TTGCCAACCC AGGCCAACAC TTGGGTTTTT TGTTGCTTTT TAAACAAGAC AGATGACATG TCGTCTACAG   
  
  
+ AGGAGAGTGA TATGTCATCT GTAGAGTTAG ACAACATGTC ATCTGCAAAC CTATTTTTTA ACATCTAGAG   
  
  
+ AGTACTGGAA AATCTAGCTG GGTATTTGTT TTTGTCCAAA AACTTAATTT TAATTTATTT TTATCTAAAG   
  
  
+ ACACCTAGAG AAAACCATTA TGAACTCAAA AAAAACTAAT TCATCAACTT GAAAAAAAAA AACAAATACA   
  
  
+ AAATGGATTA AAAACATAAA ACTAGTTTGG GAAAAAAAAT TTCTCTCTCT AACTAGATCT AAACTTTCCA   
  
  
+ GCAATATAAA TACTTAAAAA TATTTTAATA ACATTTTTTT AACATATTGA TATCAAATTT AACCATTATT   
  
  
+ TTCAATAAAA TTCAACCAAC CAATTTCTGT CTCATCATTC GTGTTCCTAT GATTCTCTCT TTTTCTTTCA   
  
  
+ AACTCTCACA AGGTAAATAA AATTATATTA TGTGCTTGAA AGGAAATTTG AAAAATCTAA AGGAAAACTT   
  
  
+ AACTTAAAAG GTGCTGACAT TTTTTATTAA AAAAAGCTCA TATTGTTTTT TCTTTAATTT TGACTTTATT   
  
  
+ CTTTTCTTTT TCAAATAATA AAATTAGATT CGTTTCCATA AAATTTAATG AACTTTTAGG CAATAATGTT   
  
  
+ GTACGAAGTC AATAATGACG TGCTACAATC GTATGTCACG ACAAAAAGAC GACATGAACA CCATCCATTG   
  
  
+ GTATTCATGT GCCTTTTCAC CTTGTAAAAT AACAGGAGAA AGATACCTTC CAGGGGCAAA TTCATCTATG   
  
  
+ CCACGTGCCT ACAAGTTGAA GGTATGTTTG GTATGCAAG  

- ACATATTATT TTGTTCACGT ATATATAGTA GTACTGTGTT TTTATCTTTA TATCCGGTAT TATAATTTAA   
  
  
- TCCGTAGATA ACGTGGTCGG TACTGTCTGT AAACCAATTA TTATATTATG AGTAACTATT TCCACAGTTA   
  
  
- CTGTGTTTAG ATACTAGGAT CACAACCTTT TAAATTTAAT AAATTTATTG TACCAATCAA CTATATAATA   
  
  
- AAAAGAATAT TAATCCATCT AACTCCCCCC TTTACTATAA AAATATTCAT TATTTATAAC TATTTTGTTC   
  
  
- ATCATCTGTG GACACAGCGT GTACAGATGT GCAACCACAA CCACACGCTG CAAATAATTA CGAATTTACC   
  
  
- ACCAAAGATC CTGATCATAC GCCCAAACCT GTCGTGCACG CTGCAAATTA AAGTAGGAGT AATTTAATCA   
  
  
- CAAATAAAAA AATAAAAAAT AAAAAATAAA AAAATAAAAA AATTAACTTA AATAAAAAAT TAAAATAGTG   
  
  
- AGTAATAAAT TAAATTAAAA TAAACATACA ATTTAAATCA AAAATAAGAA AAATAAAGAT AAACAAAATG   
  
  
- AAATCTTTTT AAAAACTAAT TTTAAAAAAA AAAGTTATAT TAGGAAATCT AAATCAAAAA AAAAGAAAAA   
  
  
- TTAAAATCGA GAGTTGTAAA CTTAACATTC CTACACCAAA CTGAATTTTT TTAAAGTTCT ATTGTACAAA   
  
  
- AATTTATAAC CTTACTGTTG TATAATCTAG TTAGGTTCAG CTGAGTGATC ATTTTTTTTA TTTGAATAAT   
  
  
- TTAGGTGTAA CCAGTACCTA GGTTCATCTA AATTATTAAA AAAATTTGGT CAAAAATAAA TTAATGTACT   
  
  
- ATTTTTTATT CGCTATCATC AATTTGTTGG TTTAATTTAA ATGTTTAACT AATGTTACCT AACATTTTTT   
  
  
- GTGAACGATT ATAATGTTGT TTATAAAGAA AACAAAATAA GTTCTTGTTT CACTTTTACT TTACTTTAAA   
  
  
- CTATTTTGTT ATATTTTTTA TATAGTTAAT TGAATTATAA AAACTTTTGT TATATATTAT ATAAAATATA   
  
  
- AGTAATTAAA AATTCTTTAA TTAAAATTAA TAAACTTCTT TTTATTTTTT TTATCTGGTT CGCGCTCCCG   
  
  
- AACGGTTGGG TCCGGTTGTG AACCCAAAAA ACAACGAAAA ATTTGTTCTG TCTACTGTAC AGCAGATGTC   
  
  
- TCCTCTCACT ATACAGTAGA CATCTCAATC TGTTGTACAG TAGACGTTTG GATAAAAAAT TGTAGATCTC   
  
  
- TCATGACCTT TTAGATCGAC CCATAAACAA AAACAGGTTT TTGAATTAAA ATTAAATAAA AATAGATTTC   
  
  
- TGTGGATCTC TTTTGGTAAT ACTTGAGTTT TTTTTGATTA AGTAGTTGAA CTTTTTTTTT TTGTTTATGT   
  
  
- TTTACCTAAT TTTTGTATTT TGATCAAACC CTTTTTTTTA AAGAGAGAGA TTGATCTAGA TTTGAAAGGT   
  
  
- CGTTATATTT ATGAATTTTT ATAAAATTAT TGTAAAAAAA TTGTATAACT ATAGTTTAAA TTGGTAATAA   
  
  
- AAGTTATTTT AAGTTGGTTG GTTAAAGACA GAGTAGTAAG CACAAGGATA CTAAGAGAGA AAAAGAAAGT   
  
  
- TTGAGAGTGT TCCATTTATT TTAATATAAT ACACGAACTT TCCTTTAAAC TTTTTAGATT TCCTTTTGAA   
  
  
- TTGAATTTTC CACGACTGTA AAAAATAATT TTTTTCGAGT ATAACAAAAA AGAAATTAAA ACTGAAATAA   
  
  
- GAAAAGAAAA AGTTTATTAT TTTAATCTAA GCAAAGGTAT TTTAAATTAC TTGAAAATCC GTTATTACAA   
  
  
- CATGCTTCAG TTATTACTGC ACGATGTTAG CATACAGTGC TGTTTTTCTG CTGTACTTGT GGTAGGTAAC   
  
  
- CATAAGTACA CGGAAAAGTG GAACATTTTA TTGTCCTCTT TCTATGGAAG GTCCCCGTTT AAGTAGATAC   
  
  
- GGTGCACGGA TGTTCAACTT CCATACAAAC CATACGTTC

+     AAGAA-motif

| Site Name | Organism | Position | Strand | Matrix score. | sequence | function |
| --- | --- | --- | --- | --- | --- | --- |
| AAGAA-motif | Avena sativa | 1622 | + | 9 | gGTAAAGAAA |  |
| AAGAA-motif | Avena sativa | 1603 | - | 7 | GAAAGAA |  |

>Potri.011G061700.1   
+ TGTATAATAA AACAAGTGCA TATATATCAT CATGACACAA AAATAGAAAT ATAGGCCATA ATATTAAATT   
  
  
+ AGGCATCTAT TGCACCAGCC ATGACAGACA TTTGGTTAAT AATATAATAC TCATTGATAA AGGTGTCAAT   
  
  
+ GACACAAATC TATGATCCTA GTGTTGGAAA ATTTAAATTA TTTAAATAAC ATGGTTAGTT GATATATTAT   
  
  
+ TTTTCTTATA ATTAGGTAGA TTGAGGGGGG AAATGATATT TTTATAAGTA ATAAATATTG ATAAAACAAG   
  
  
+ TAGTAGACAC CTGTGTCGCA CATGTCTACA CGTTGGTGTT GGTGTGCGAC GTTTATTAAT GCTTAAATGG   
  
  
+ TGGTTTCTAG GACTAGTATG CGGGTTTGGA CAGCACGTGC GACGTTTAAT TTCATCCTCA TTAAATTAGT   
  
  
+ GTTTATTTTT TTATTTTTTA TTTTTTATTT TTTTATTTTT TTAATTGAAT TTATTTTTTA ATTTTATCAC   
  
  
+ TCATTATTTA ATTTAATTTT ATTTGTATGT TAAATTTAGT TTTTATTCTT TTTATTTCTA TTTGTTTTAC   
  
  
+ TTTAGAAAAA TTTTTGATTA AAATTTTTTT TTTCAATATA ATCCTTTAGA TTTAGTTTTT TTTTCTTTTT   
  
  
+ AATTTTAGCT CTCAACATTT GAATTGTAAG GATGTGGTTT GACTTAAAAA AATTTCAAGA TAACATGTTT   
  
  
+ TTAAATATTG GAATGACAAC ATATTAGATC AATCCAAGTC GACTCACTAG TAAAAAAAAT AAACTTATTA   
  
  
+ AATCCACATT GGTCATGGAT CCAAGTAGAT TTAATAATTT TTTTAAACCA GTTTTTATTT AATTACATGA   
  
  
+ TAAAAAATAA GCGATAGTAG TTAAACAACC AAATTAAATT TACAAATTGA TTACAATGGA TTGTAAAAAA   
  
  
+ CACTTGCTAA TATTACAACA AATATTTCTT TTGTTTTATT CAAGAACAAA GTGAAAATGA AATGAAATTT   
  
  
+ GATAAAACAA TATAAAAAAT ATATCAATTA ACTTAATATT TTTGAAAACA ATATATAATA TATTTTATAT   
  
  
+ TCATTAATTT TTAAGAAATT AATTTTAATT ATTTGAAGAA AAATAAAAAA AATAGACCAA GCGCGAGGGC   
  
  
+ TTGCCAACCC AGGCCAACAC TTGGGTTTTT TGTTGCTTTT TAAACAAGAC AGATGACATG TCGTCTACAG   
  
  
+ AGGAGAGTGA TATGTCATCT GTAGAGTTAG ACAACATGTC ATCTGCAAAC CTATTTTTTA ACATCTAGAG   
  
  
+ AGTACTGGAA AATCTAGCTG GGTATTTGTT TTTGTCCAAA AACTTAATTT TAATTTATTT TTATCTAAAG   
  
  
+ ACACCTAGAG AAAACCATTA TGAACTCAAA AAAAACTAAT TCATCAACTT GAAAAAAAAA AACAAATACA   
  
  
+ AAATGGATTA AAAACATAAA ACTAGTTTGG GAAAAAAAAT TTCTCTCTCT AACTAGATCT AAACTTTCCA   
  
  
+ GCAATATAAA TACTTAAAAA TATTTTAATA ACATTTTTTT AACATATTGA TATCAAATTT AACCATTATT   
  
  
+ TTCAATAAAA TTCAACCAAC CAATTTCTGT CTCATCATTC GTGTTCCTAT GATTCTCTCT TTTTCTTTCA   
  
  
+ AACTCTCACA AGGTAAATAA AATTATATTA TGTGCTTGAA AGGAAATTTG AAAAATCTAA AGGAAAACTT   
  
  
+ AACTTAAAAG GTGCTGACAT TTTTTATTAA AAAAAGCTCA TATTGTTTTT TCTTTAATTT TGACTTTATT   
  
  
+ CTTTTCTTTT TCAAATAATA AAATTAGATT CGTTTCCATA AAATTTAATG AACTTTTAGG CAATAATGTT   
  
  
+ GTACGAAGTC AATAATGACG TGCTACAATC GTATGTCACG ACAAAAAGAC GACATGAACA CCATCCATTG   
  
  
+ GTATTCATGT GCCTTTTCAC CTTGTAAAAT AACAGGAGAA AGATACCTTC CAGGGGCAAA TTCATCTATG   
  
  
+ CCACGTGCCT ACAAGTTGAA GGTATGTTTG GTATGCAAG  

- ACATATTATT TTGTTCACGT ATATATAGTA GTACTGTGTT TTTATCTTTA TATCCGGTAT TATAATTTAA   
  
  
- TCCGTAGATA ACGTGGTCGG TACTGTCTGT AAACCAATTA TTATATTATG AGTAACTATT TCCACAGTTA   
  
  
- CTGTGTTTAG ATACTAGGAT CACAACCTTT TAAATTTAAT AAATTTATTG TACCAATCAA CTATATAATA   
  
  
- AAAAGAATAT TAATCCATCT AACTCCCCCC TTTACTATAA AAATATTCAT TATTTATAAC TATTTTGTTC   
  
  
- ATCATCTGTG GACACAGCGT GTACAGATGT GCAACCACAA CCACACGCTG CAAATAATTA CGAATTTACC   
  
  
- ACCAAAGATC CTGATCATAC GCCCAAACCT GTCGTGCACG CTGCAAATTA AAGTAGGAGT AATTTAATCA   
  
  
- CAAATAAAAA AATAAAAAAT AAAAAATAAA AAAATAAAAA AATTAACTTA AATAAAAAAT TAAAATAGTG   
  
  
- AGTAATAAAT TAAATTAAAA TAAACATACA ATTTAAATCA AAAATAAGAA AAATAAAGAT AAACAAAATG   
  
  
- AAATCTTTTT AAAAACTAAT TTTAAAAAAA AAAGTTATAT TAGGAAATCT AAATCAAAAA AAAAGAAAAA   
  
  
- TTAAAATCGA GAGTTGTAAA CTTAACATTC CTACACCAAA CTGAATTTTT TTAAAGTTCT ATTGTACAAA   
  
  
- AATTTATAAC CTTACTGTTG TATAATCTAG TTAGGTTCAG CTGAGTGATC ATTTTTTTTA TTTGAATAAT   
  
  
- TTAGGTGTAA CCAGTACCTA GGTTCATCTA AATTATTAAA AAAATTTGGT CAAAAATAAA TTAATGTACT   
  
  
- ATTTTTTATT CGCTATCATC AATTTGTTGG TTTAATTTAA ATGTTTAACT AATGTTACCT AACATTTTTT   
  
  
- GTGAACGATT ATAATGTTGT TTATAAAGAA AACAAAATAA GTTCTTGTTT CACTTTTACT TTACTTTAAA   
  
  
- CTATTTTGTT ATATTTTTTA TATAGTTAAT TGAATTATAA AAACTTTTGT TATATATTAT ATAAAATATA   
  
  
- AGTAATTAAA AATTCTTTAA TTAAAATTAA TAAACTTCTT TTTATTTTTT TTATCTGGTT CGCGCTCCCG   
  
  
- AACGGTTGGG TCCGGTTGTG AACCCAAAAA ACAACGAAAA ATTTGTTCTG TCTACTGTAC AGCAGATGTC   
  
  
- TCCTCTCACT ATACAGTAGA CATCTCAATC TGTTGTACAG TAGACGTTTG GATAAAAAAT TGTAGATCTC   
  
  
- TCATGACCTT TTAGATCGAC CCATAAACAA AAACAGGTTT TTGAATTAAA ATTAAATAAA AATAGATTTC   
  
  
- TGTGGATCTC TTTTGGTAAT ACTTGAGTTT TTTTTGATTA AGTAGTTGAA CTTTTTTTTT TTGTTTATGT   
  
  
- TTTACCTAAT TTTTGTATTT TGATCAAACC CTTTTTTTTA AAGAGAGAGA TTGATCTAGA TTTGAAAGGT   
  
  
- CGTTATATTT ATGAATTTTT ATAAAATTAT TGTAAAAAAA TTGTATAACT ATAGTTTAAA TTGGTAATAA   
  
  
- AAGTTATTTT AAGTTGGTTG GTTAAAGACA GAGTAGTAAG CACAAGGATA CTAAGAGAGA AAAAGAAAGT   
  
  
- TTGAGAGTGT TCCATTTATT TTAATATAAT ACACGAACTT TCCTTTAAAC TTTTTAGATT TCCTTTTGAA   
  
  
- TTGAATTTTC CACGACTGTA AAAAATAATT TTTTTCGAGT ATAACAAAAA AGAAATTAAA ACTGAAATAA   
  
  
- GAAAAGAAAA AGTTTATTAT TTTAATCTAA GCAAAGGTAT TTTAAATTAC TTGAAAATCC GTTATTACAA   
  
  
- CATGCTTCAG TTATTACTGC ACGATGTTAG CATACAGTGC TGTTTTTCTG CTGTACTTGT GGTAGGTAAC   
  
  
- CATAAGTACA CGGAAAAGTG GAACATTTTA TTGTCCTCTT TCTATGGAAG GTCCCCGTTT AAGTAGATAC   
  
  
- GGTGCACGGA TGTTCAACTT CCATACAAAC CATACGTTC

+     ABRE

| Site Name | Organism | Position | Strand | Matrix score. | sequence | function |
| --- | --- | --- | --- | --- | --- | --- |
| ABRE | Arabidopsis thaliana | 1963 | + | 5 | ACGTG | cis-acting element involved in the abscisic acid responsiveness |
| ABRE | Arabidopsis thaliana | 1838 | + | 5 | ACGTG | cis-acting element involved in the abscisic acid responsiveness |
| ABRE | Arabidopsis thaliana | 385 | + | 5 | ACGTG | cis-acting element involved in the abscisic acid responsiveness |
| ABRE | Arabidopsis thaliana | 309 | - | 5 | ACGTG | cis-acting element involved in the abscisic acid responsiveness |
| ABRE | Arabidopsis thaliana | 384 | + | 6 | CACGTG | cis-acting element involved in the abscisic acid responsiveness |
| ABRE | Triticum aestivum | 1960 | - | 9 | GACACGTGGC | cis-acting element involved in the abscisic acid responsiveness |
| ABRE | Hordeum vulgare | 297 | + | 9 | CGCACGTGTC | cis-acting element involved in the abscisic acid responsiveness |
| ABRE | Arabidopsis thaliana | 1962 | - | 6 | CACGTG | cis-acting element involved in the abscisic acid responsiveness |

>Potri.011G061700.1   
+ TGTATAATAA AACAAGTGCA TATATATCAT CATGACACAA AAATAGAAAT ATAGGCCATA ATATTAAATT   
  
  
+ AGGCATCTAT TGCACCAGCC ATGACAGACA TTTGGTTAAT AATATAATAC TCATTGATAA AGGTGTCAAT   
  
  
+ GACACAAATC TATGATCCTA GTGTTGGAAA ATTTAAATTA TTTAAATAAC ATGGTTAGTT GATATATTAT   
  
  
+ TTTTCTTATA ATTAGGTAGA TTGAGGGGGG AAATGATATT TTTATAAGTA ATAAATATTG ATAAAACAAG   
  
  
+ TAGTAGACAC CTGTGTCGCA CATGTCTACA CGTTGGTGTT GGTGTGCGAC GTTTATTAAT GCTTAAATGG   
  
  
+ TGGTTTCTAG GACTAGTATG CGGGTTTGGA CAGCACGTGC GACGTTTAAT TTCATCCTCA TTAAATTAGT   
  
  
+ GTTTATTTTT TTATTTTTTA TTTTTTATTT TTTTATTTTT TTAATTGAAT TTATTTTTTA ATTTTATCAC   
  
  
+ TCATTATTTA ATTTAATTTT ATTTGTATGT TAAATTTAGT TTTTATTCTT TTTATTTCTA TTTGTTTTAC   
  
  
+ TTTAGAAAAA TTTTTGATTA AAATTTTTTT TTTCAATATA ATCCTTTAGA TTTAGTTTTT TTTTCTTTTT   
  
  
+ AATTTTAGCT CTCAACATTT GAATTGTAAG GATGTGGTTT GACTTAAAAA AATTTCAAGA TAACATGTTT   
  
  
+ TTAAATATTG GAATGACAAC ATATTAGATC AATCCAAGTC GACTCACTAG TAAAAAAAAT AAACTTATTA   
  
  
+ AATCCACATT GGTCATGGAT CCAAGTAGAT TTAATAATTT TTTTAAACCA GTTTTTATTT AATTACATGA   
  
  
+ TAAAAAATAA GCGATAGTAG TTAAACAACC AAATTAAATT TACAAATTGA TTACAATGGA TTGTAAAAAA   
  
  
+ CACTTGCTAA TATTACAACA AATATTTCTT TTGTTTTATT CAAGAACAAA GTGAAAATGA AATGAAATTT   
  
  
+ GATAAAACAA TATAAAAAAT ATATCAATTA ACTTAATATT TTTGAAAACA ATATATAATA TATTTTATAT   
  
  
+ TCATTAATTT TTAAGAAATT AATTTTAATT ATTTGAAGAA AAATAAAAAA AATAGACCAA GCGCGAGGGC   
  
  
+ TTGCCAACCC AGGCCAACAC TTGGGTTTTT TGTTGCTTTT TAAACAAGAC AGATGACATG TCGTCTACAG   
  
  
+ AGGAGAGTGA TATGTCATCT GTAGAGTTAG ACAACATGTC ATCTGCAAAC CTATTTTTTA ACATCTAGAG   
  
  
+ AGTACTGGAA AATCTAGCTG GGTATTTGTT TTTGTCCAAA AACTTAATTT TAATTTATTT TTATCTAAAG   
  
  
+ ACACCTAGAG AAAACCATTA TGAACTCAAA AAAAACTAAT TCATCAACTT GAAAAAAAAA AACAAATACA   
  
  
+ AAATGGATTA AAAACATAAA ACTAGTTTGG GAAAAAAAAT TTCTCTCTCT AACTAGATCT AAACTTTCCA   
  
  
+ GCAATATAAA TACTTAAAAA TATTTTAATA ACATTTTTTT AACATATTGA TATCAAATTT AACCATTATT   
  
  
+ TTCAATAAAA TTCAACCAAC CAATTTCTGT CTCATCATTC GTGTTCCTAT GATTCTCTCT TTTTCTTTCA   
  
  
+ AACTCTCACA AGGTAAATAA AATTATATTA TGTGCTTGAA AGGAAATTTG AAAAATCTAA AGGAAAACTT   
  
  
+ AACTTAAAAG GTGCTGACAT TTTTTATTAA AAAAAGCTCA TATTGTTTTT TCTTTAATTT TGACTTTATT   
  
  
+ CTTTTCTTTT TCAAATAATA AAATTAGATT CGTTTCCATA AAATTTAATG AACTTTTAGG CAATAATGTT   
  
  
+ GTACGAAGTC AATAATGACG TGCTACAATC GTATGTCACG ACAAAAAGAC GACATGAACA CCATCCATTG   
  
  
+ GTATTCATGT GCCTTTTCAC CTTGTAAAAT AACAGGAGAA AGATACCTTC CAGGGGCAAA TTCATCTATG   
  
  
+ CCACGTGCCT ACAAGTTGAA GGTATGTTTG GTATGCAAG  

- ACATATTATT TTGTTCACGT ATATATAGTA GTACTGTGTT TTTATCTTTA TATCCGGTAT TATAATTTAA   
  
  
- TCCGTAGATA ACGTGGTCGG TACTGTCTGT AAACCAATTA TTATATTATG AGTAACTATT TCCACAGTTA   
  
  
- CTGTGTTTAG ATACTAGGAT CACAACCTTT TAAATTTAAT AAATTTATTG TACCAATCAA CTATATAATA   
  
  
- AAAAGAATAT TAATCCATCT AACTCCCCCC TTTACTATAA AAATATTCAT TATTTATAAC TATTTTGTTC   
  
  
- ATCATCTGTG GACACAGCGT GTACAGATGT GCAACCACAA CCACACGCTG CAAATAATTA CGAATTTACC   
  
  
- ACCAAAGATC CTGATCATAC GCCCAAACCT GTCGTGCACG CTGCAAATTA AAGTAGGAGT AATTTAATCA   
  
  
- CAAATAAAAA AATAAAAAAT AAAAAATAAA AAAATAAAAA AATTAACTTA AATAAAAAAT TAAAATAGTG   
  
  
- AGTAATAAAT TAAATTAAAA TAAACATACA ATTTAAATCA AAAATAAGAA AAATAAAGAT AAACAAAATG   
  
  
- AAATCTTTTT AAAAACTAAT TTTAAAAAAA AAAGTTATAT TAGGAAATCT AAATCAAAAA AAAAGAAAAA   
  
  
- TTAAAATCGA GAGTTGTAAA CTTAACATTC CTACACCAAA CTGAATTTTT TTAAAGTTCT ATTGTACAAA   
  
  
- AATTTATAAC CTTACTGTTG TATAATCTAG TTAGGTTCAG CTGAGTGATC ATTTTTTTTA TTTGAATAAT   
  
  
- TTAGGTGTAA CCAGTACCTA GGTTCATCTA AATTATTAAA AAAATTTGGT CAAAAATAAA TTAATGTACT   
  
  
- ATTTTTTATT CGCTATCATC AATTTGTTGG TTTAATTTAA ATGTTTAACT AATGTTACCT AACATTTTTT   
  
  
- GTGAACGATT ATAATGTTGT TTATAAAGAA AACAAAATAA GTTCTTGTTT CACTTTTACT TTACTTTAAA   
  
  
- CTATTTTGTT ATATTTTTTA TATAGTTAAT TGAATTATAA AAACTTTTGT TATATATTAT ATAAAATATA   
  
  
- AGTAATTAAA AATTCTTTAA TTAAAATTAA TAAACTTCTT TTTATTTTTT TTATCTGGTT CGCGCTCCCG   
  
  
- AACGGTTGGG TCCGGTTGTG AACCCAAAAA ACAACGAAAA ATTTGTTCTG TCTACTGTAC AGCAGATGTC   
  
  
- TCCTCTCACT ATACAGTAGA CATCTCAATC TGTTGTACAG TAGACGTTTG GATAAAAAAT TGTAGATCTC   
  
  
- TCATGACCTT TTAGATCGAC CCATAAACAA AAACAGGTTT TTGAATTAAA ATTAAATAAA AATAGATTTC   
  
  
- TGTGGATCTC TTTTGGTAAT ACTTGAGTTT TTTTTGATTA AGTAGTTGAA CTTTTTTTTT TTGTTTATGT   
  
  
- TTTACCTAAT TTTTGTATTT TGATCAAACC CTTTTTTTTA AAGAGAGAGA TTGATCTAGA TTTGAAAGGT   
  
  
- CGTTATATTT ATGAATTTTT ATAAAATTAT TGTAAAAAAA TTGTATAACT ATAGTTTAAA TTGGTAATAA   
  
  
- AAGTTATTTT AAGTTGGTTG GTTAAAGACA GAGTAGTAAG CACAAGGATA CTAAGAGAGA AAAAGAAAGT   
  
  
- TTGAGAGTGT TCCATTTATT TTAATATAAT ACACGAACTT TCCTTTAAAC TTTTTAGATT TCCTTTTGAA   
  
  
- TTGAATTTTC CACGACTGTA AAAAATAATT TTTTTCGAGT ATAACAAAAA AGAAATTAAA ACTGAAATAA   
  
  
- GAAAAGAAAA AGTTTATTAT TTTAATCTAA GCAAAGGTAT TTTAAATTAC TTGAAAATCC GTTATTACAA   
  
  
- CATGCTTCAG TTATTACTGC ACGATGTTAG CATACAGTGC TGTTTTTCTG CTGTACTTGT GGTAGGTAAC   
  
  
- CATAAGTACA CGGAAAAGTG GAACATTTTA TTGTCCTCTT TCTATGGAAG GTCCCCGTTT AAGTAGATAC   
  
  
- GGTGCACGGA TGTTCAACTT CCATACAAAC CATACGTTC

+     ARE

| Site Name | Organism | Position | Strand | Matrix score. | sequence | function |
| --- | --- | --- | --- | --- | --- | --- |
| ARE | Zea mays | 665 | - | 6 | AAACCA | cis-acting regulatory element essential for the anaerobic induction |
| ARE | Zea mays | 351 | - | 6 | AAACCA | cis-acting regulatory element essential for the anaerobic induction |
| ARE | Zea mays | 815 | + | 6 | AAACCA | cis-acting regulatory element essential for the anaerobic induction |
| ARE | Zea mays | 1342 | + | 6 | AAACCA | cis-acting regulatory element essential for the anaerobic induction |

>Potri.011G061700.1   
+ TGTATAATAA AACAAGTGCA TATATATCAT CATGACACAA AAATAGAAAT ATAGGCCATA ATATTAAATT   
  
  
+ AGGCATCTAT TGCACCAGCC ATGACAGACA TTTGGTTAAT AATATAATAC TCATTGATAA AGGTGTCAAT   
  
  
+ GACACAAATC TATGATCCTA GTGTTGGAAA ATTTAAATTA TTTAAATAAC ATGGTTAGTT GATATATTAT   
  
  
+ TTTTCTTATA ATTAGGTAGA TTGAGGGGGG AAATGATATT TTTATAAGTA ATAAATATTG ATAAAACAAG   
  
  
+ TAGTAGACAC CTGTGTCGCA CATGTCTACA CGTTGGTGTT GGTGTGCGAC GTTTATTAAT GCTTAAATGG   
  
  
+ TGGTTTCTAG GACTAGTATG CGGGTTTGGA CAGCACGTGC GACGTTTAAT TTCATCCTCA TTAAATTAGT   
  
  
+ GTTTATTTTT TTATTTTTTA TTTTTTATTT TTTTATTTTT TTAATTGAAT TTATTTTTTA ATTTTATCAC   
  
  
+ TCATTATTTA ATTTAATTTT ATTTGTATGT TAAATTTAGT TTTTATTCTT TTTATTTCTA TTTGTTTTAC   
  
  
+ TTTAGAAAAA TTTTTGATTA AAATTTTTTT TTTCAATATA ATCCTTTAGA TTTAGTTTTT TTTTCTTTTT   
  
  
+ AATTTTAGCT CTCAACATTT GAATTGTAAG GATGTGGTTT GACTTAAAAA AATTTCAAGA TAACATGTTT   
  
  
+ TTAAATATTG GAATGACAAC ATATTAGATC AATCCAAGTC GACTCACTAG TAAAAAAAAT AAACTTATTA   
  
  
+ AATCCACATT GGTCATGGAT CCAAGTAGAT TTAATAATTT TTTTAAACCA GTTTTTATTT AATTACATGA   
  
  
+ TAAAAAATAA GCGATAGTAG TTAAACAACC AAATTAAATT TACAAATTGA TTACAATGGA TTGTAAAAAA   
  
  
+ CACTTGCTAA TATTACAACA AATATTTCTT TTGTTTTATT CAAGAACAAA GTGAAAATGA AATGAAATTT   
  
  
+ GATAAAACAA TATAAAAAAT ATATCAATTA ACTTAATATT TTTGAAAACA ATATATAATA TATTTTATAT   
  
  
+ TCATTAATTT TTAAGAAATT AATTTTAATT ATTTGAAGAA AAATAAAAAA AATAGACCAA GCGCGAGGGC   
  
  
+ TTGCCAACCC AGGCCAACAC TTGGGTTTTT TGTTGCTTTT TAAACAAGAC AGATGACATG TCGTCTACAG   
  
  
+ AGGAGAGTGA TATGTCATCT GTAGAGTTAG ACAACATGTC ATCTGCAAAC CTATTTTTTA ACATCTAGAG   
  
  
+ AGTACTGGAA AATCTAGCTG GGTATTTGTT TTTGTCCAAA AACTTAATTT TAATTTATTT TTATCTAAAG   
  
  
+ ACACCTAGAG AAAACCATTA TGAACTCAAA AAAAACTAAT TCATCAACTT GAAAAAAAAA AACAAATACA   
  
  
+ AAATGGATTA AAAACATAAA ACTAGTTTGG GAAAAAAAAT TTCTCTCTCT AACTAGATCT AAACTTTCCA   
  
  
+ GCAATATAAA TACTTAAAAA TATTTTAATA ACATTTTTTT AACATATTGA TATCAAATTT AACCATTATT   
  
  
+ TTCAATAAAA TTCAACCAAC CAATTTCTGT CTCATCATTC GTGTTCCTAT GATTCTCTCT TTTTCTTTCA   
  
  
+ AACTCTCACA AGGTAAATAA AATTATATTA TGTGCTTGAA AGGAAATTTG AAAAATCTAA AGGAAAACTT   
  
  
+ AACTTAAAAG GTGCTGACAT TTTTTATTAA AAAAAGCTCA TATTGTTTTT TCTTTAATTT TGACTTTATT   
  
  
+ CTTTTCTTTT TCAAATAATA AAATTAGATT CGTTTCCATA AAATTTAATG AACTTTTAGG CAATAATGTT   
  
  
+ GTACGAAGTC AATAATGACG TGCTACAATC GTATGTCACG ACAAAAAGAC GACATGAACA CCATCCATTG   
  
  
+ GTATTCATGT GCCTTTTCAC CTTGTAAAAT AACAGGAGAA AGATACCTTC CAGGGGCAAA TTCATCTATG   
  
  
+ CCACGTGCCT ACAAGTTGAA GGTATGTTTG GTATGCAAG  

- ACATATTATT TTGTTCACGT ATATATAGTA GTACTGTGTT TTTATCTTTA TATCCGGTAT TATAATTTAA   
  
  
- TCCGTAGATA ACGTGGTCGG TACTGTCTGT AAACCAATTA TTATATTATG AGTAACTATT TCCACAGTTA   
  
  
- CTGTGTTTAG ATACTAGGAT CACAACCTTT TAAATTTAAT AAATTTATTG TACCAATCAA CTATATAATA   
  
  
- AAAAGAATAT TAATCCATCT AACTCCCCCC TTTACTATAA AAATATTCAT TATTTATAAC TATTTTGTTC   
  
  
- ATCATCTGTG GACACAGCGT GTACAGATGT GCAACCACAA CCACACGCTG CAAATAATTA CGAATTTACC   
  
  
- ACCAAAGATC CTGATCATAC GCCCAAACCT GTCGTGCACG CTGCAAATTA AAGTAGGAGT AATTTAATCA   
  
  
- CAAATAAAAA AATAAAAAAT AAAAAATAAA AAAATAAAAA AATTAACTTA AATAAAAAAT TAAAATAGTG   
  
  
- AGTAATAAAT TAAATTAAAA TAAACATACA ATTTAAATCA AAAATAAGAA AAATAAAGAT AAACAAAATG   
  
  
- AAATCTTTTT AAAAACTAAT TTTAAAAAAA AAAGTTATAT TAGGAAATCT AAATCAAAAA AAAAGAAAAA   
  
  
- TTAAAATCGA GAGTTGTAAA CTTAACATTC CTACACCAAA CTGAATTTTT TTAAAGTTCT ATTGTACAAA   
  
  
- AATTTATAAC CTTACTGTTG TATAATCTAG TTAGGTTCAG CTGAGTGATC ATTTTTTTTA TTTGAATAAT   
  
  
- TTAGGTGTAA CCAGTACCTA GGTTCATCTA AATTATTAAA AAAATTTGGT CAAAAATAAA TTAATGTACT   
  
  
- ATTTTTTATT CGCTATCATC AATTTGTTGG TTTAATTTAA ATGTTTAACT AATGTTACCT AACATTTTTT   
  
  
- GTGAACGATT ATAATGTTGT TTATAAAGAA AACAAAATAA GTTCTTGTTT CACTTTTACT TTACTTTAAA   
  
  
- CTATTTTGTT ATATTTTTTA TATAGTTAAT TGAATTATAA AAACTTTTGT TATATATTAT ATAAAATATA   
  
  
- AGTAATTAAA AATTCTTTAA TTAAAATTAA TAAACTTCTT TTTATTTTTT TTATCTGGTT CGCGCTCCCG   
  
  
- AACGGTTGGG TCCGGTTGTG AACCCAAAAA ACAACGAAAA ATTTGTTCTG TCTACTGTAC AGCAGATGTC   
  
  
- TCCTCTCACT ATACAGTAGA CATCTCAATC TGTTGTACAG TAGACGTTTG GATAAAAAAT TGTAGATCTC   
  
  
- TCATGACCTT TTAGATCGAC CCATAAACAA AAACAGGTTT TTGAATTAAA ATTAAATAAA AATAGATTTC   
  
  
- TGTGGATCTC TTTTGGTAAT ACTTGAGTTT TTTTTGATTA AGTAGTTGAA CTTTTTTTTT TTGTTTATGT   
  
  
- TTTACCTAAT TTTTGTATTT TGATCAAACC CTTTTTTTTA AAGAGAGAGA TTGATCTAGA TTTGAAAGGT   
  
  
- CGTTATATTT ATGAATTTTT ATAAAATTAT TGTAAAAAAA TTGTATAACT ATAGTTTAAA TTGGTAATAA   
  
  
- AAGTTATTTT AAGTTGGTTG GTTAAAGACA GAGTAGTAAG CACAAGGATA CTAAGAGAGA AAAAGAAAGT   
  
  
- TTGAGAGTGT TCCATTTATT TTAATATAAT ACACGAACTT TCCTTTAAAC TTTTTAGATT TCCTTTTGAA   
  
  
- TTGAATTTTC CACGACTGTA AAAAATAATT TTTTTCGAGT ATAACAAAAA AGAAATTAAA ACTGAAATAA   
  
  
- GAAAAGAAAA AGTTTATTAT TTTAATCTAA GCAAAGGTAT TTTAAATTAC TTGAAAATCC GTTATTACAA   
  
  
- CATGCTTCAG TTATTACTGC ACGATGTTAG CATACAGTGC TGTTTTTCTG CTGTACTTGT GGTAGGTAAC   
  
  
- CATAAGTACA CGGAAAAGTG GAACATTTTA TTGTCCTCTT TCTATGGAAG GTCCCCGTTT AAGTAGATAC   
  
  
- GGTGCACGGA TGTTCAACTT CCATACAAAC CATACGTTC

+     AT-rich element

| Site Name | Organism | Position | Strand | Matrix score. | sequence | function |
| --- | --- | --- | --- | --- | --- | --- |
| AT-rich element | Glycine max | 541 | - | 10 | ATAGAAATCAA | binding site of AT-rich DNA binding protein (ATBP-1) |

>Potri.011G061700.1   
+ TGTATAATAA AACAAGTGCA TATATATCAT CATGACACAA AAATAGAAAT ATAGGCCATA ATATTAAATT   
  
  
+ AGGCATCTAT TGCACCAGCC ATGACAGACA TTTGGTTAAT AATATAATAC TCATTGATAA AGGTGTCAAT   
  
  
+ GACACAAATC TATGATCCTA GTGTTGGAAA ATTTAAATTA TTTAAATAAC ATGGTTAGTT GATATATTAT   
  
  
+ TTTTCTTATA ATTAGGTAGA TTGAGGGGGG AAATGATATT TTTATAAGTA ATAAATATTG ATAAAACAAG   
  
  
+ TAGTAGACAC CTGTGTCGCA CATGTCTACA CGTTGGTGTT GGTGTGCGAC GTTTATTAAT GCTTAAATGG   
  
  
+ TGGTTTCTAG GACTAGTATG CGGGTTTGGA CAGCACGTGC GACGTTTAAT TTCATCCTCA TTAAATTAGT   
  
  
+ GTTTATTTTT TTATTTTTTA TTTTTTATTT TTTTATTTTT TTAATTGAAT TTATTTTTTA ATTTTATCAC   
  
  
+ TCATTATTTA ATTTAATTTT ATTTGTATGT TAAATTTAGT TTTTATTCTT TTTATTTCTA TTTGTTTTAC   
  
  
+ TTTAGAAAAA TTTTTGATTA AAATTTTTTT TTTCAATATA ATCCTTTAGA TTTAGTTTTT TTTTCTTTTT   
  
  
+ AATTTTAGCT CTCAACATTT GAATTGTAAG GATGTGGTTT GACTTAAAAA AATTTCAAGA TAACATGTTT   
  
  
+ TTAAATATTG GAATGACAAC ATATTAGATC AATCCAAGTC GACTCACTAG TAAAAAAAAT AAACTTATTA   
  
  
+ AATCCACATT GGTCATGGAT CCAAGTAGAT TTAATAATTT TTTTAAACCA GTTTTTATTT AATTACATGA   
  
  
+ TAAAAAATAA GCGATAGTAG TTAAACAACC AAATTAAATT TACAAATTGA TTACAATGGA TTGTAAAAAA   
  
  
+ CACTTGCTAA TATTACAACA AATATTTCTT TTGTTTTATT CAAGAACAAA GTGAAAATGA AATGAAATTT   
  
  
+ GATAAAACAA TATAAAAAAT ATATCAATTA ACTTAATATT TTTGAAAACA ATATATAATA TATTTTATAT   
  
  
+ TCATTAATTT TTAAGAAATT AATTTTAATT ATTTGAAGAA AAATAAAAAA AATAGACCAA GCGCGAGGGC   
  
  
+ TTGCCAACCC AGGCCAACAC TTGGGTTTTT TGTTGCTTTT TAAACAAGAC AGATGACATG TCGTCTACAG   
  
  
+ AGGAGAGTGA TATGTCATCT GTAGAGTTAG ACAACATGTC ATCTGCAAAC CTATTTTTTA ACATCTAGAG   
  
  
+ AGTACTGGAA AATCTAGCTG GGTATTTGTT TTTGTCCAAA AACTTAATTT TAATTTATTT TTATCTAAAG   
  
  
+ ACACCTAGAG AAAACCATTA TGAACTCAAA AAAAACTAAT TCATCAACTT GAAAAAAAAA AACAAATACA   
  
  
+ AAATGGATTA AAAACATAAA ACTAGTTTGG GAAAAAAAAT TTCTCTCTCT AACTAGATCT AAACTTTCCA   
  
  
+ GCAATATAAA TACTTAAAAA TATTTTAATA ACATTTTTTT AACATATTGA TATCAAATTT AACCATTATT   
  
  
+ TTCAATAAAA TTCAACCAAC CAATTTCTGT CTCATCATTC GTGTTCCTAT GATTCTCTCT TTTTCTTTCA   
  
  
+ AACTCTCACA AGGTAAATAA AATTATATTA TGTGCTTGAA AGGAAATTTG AAAAATCTAA AGGAAAACTT   
  
  
+ AACTTAAAAG GTGCTGACAT TTTTTATTAA AAAAAGCTCA TATTGTTTTT TCTTTAATTT TGACTTTATT   
  
  
+ CTTTTCTTTT TCAAATAATA AAATTAGATT CGTTTCCATA AAATTTAATG AACTTTTAGG CAATAATGTT   
  
  
+ GTACGAAGTC AATAATGACG TGCTACAATC GTATGTCACG ACAAAAAGAC GACATGAACA CCATCCATTG   
  
  
+ GTATTCATGT GCCTTTTCAC CTTGTAAAAT AACAGGAGAA AGATACCTTC CAGGGGCAAA TTCATCTATG   
  
  
+ CCACGTGCCT ACAAGTTGAA GGTATGTTTG GTATGCAAG  

- ACATATTATT TTGTTCACGT ATATATAGTA GTACTGTGTT TTTATCTTTA TATCCGGTAT TATAATTTAA   
  
  
- TCCGTAGATA ACGTGGTCGG TACTGTCTGT AAACCAATTA TTATATTATG AGTAACTATT TCCACAGTTA   
  
  
- CTGTGTTTAG ATACTAGGAT CACAACCTTT TAAATTTAAT AAATTTATTG TACCAATCAA CTATATAATA   
  
  
- AAAAGAATAT TAATCCATCT AACTCCCCCC TTTACTATAA AAATATTCAT TATTTATAAC TATTTTGTTC   
  
  
- ATCATCTGTG GACACAGCGT GTACAGATGT GCAACCACAA CCACACGCTG CAAATAATTA CGAATTTACC   
  
  
- ACCAAAGATC CTGATCATAC GCCCAAACCT GTCGTGCACG CTGCAAATTA AAGTAGGAGT AATTTAATCA   
  
  
- CAAATAAAAA AATAAAAAAT AAAAAATAAA AAAATAAAAA AATTAACTTA AATAAAAAAT TAAAATAGTG   
  
  
- AGTAATAAAT TAAATTAAAA TAAACATACA ATTTAAATCA AAAATAAGAA AAATAAAGAT AAACAAAATG   
  
  
- AAATCTTTTT AAAAACTAAT TTTAAAAAAA AAAGTTATAT TAGGAAATCT AAATCAAAAA AAAAGAAAAA   
  
  
- TTAAAATCGA GAGTTGTAAA CTTAACATTC CTACACCAAA CTGAATTTTT TTAAAGTTCT ATTGTACAAA   
  
  
- AATTTATAAC CTTACTGTTG TATAATCTAG TTAGGTTCAG CTGAGTGATC ATTTTTTTTA TTTGAATAAT   
  
  
- TTAGGTGTAA CCAGTACCTA GGTTCATCTA AATTATTAAA AAAATTTGGT CAAAAATAAA TTAATGTACT   
  
  
- ATTTTTTATT CGCTATCATC AATTTGTTGG TTTAATTTAA ATGTTTAACT AATGTTACCT AACATTTTTT   
  
  
- GTGAACGATT ATAATGTTGT TTATAAAGAA AACAAAATAA GTTCTTGTTT CACTTTTACT TTACTTTAAA   
  
  
- CTATTTTGTT ATATTTTTTA TATAGTTAAT TGAATTATAA AAACTTTTGT TATATATTAT ATAAAATATA   
  
  
- AGTAATTAAA AATTCTTTAA TTAAAATTAA TAAACTTCTT TTTATTTTTT TTATCTGGTT CGCGCTCCCG   
  
  
- AACGGTTGGG TCCGGTTGTG AACCCAAAAA ACAACGAAAA ATTTGTTCTG TCTACTGTAC AGCAGATGTC   
  
  
- TCCTCTCACT ATACAGTAGA CATCTCAATC TGTTGTACAG TAGACGTTTG GATAAAAAAT TGTAGATCTC   
  
  
- TCATGACCTT TTAGATCGAC CCATAAACAA AAACAGGTTT TTGAATTAAA ATTAAATAAA AATAGATTTC   
  
  
- TGTGGATCTC TTTTGGTAAT ACTTGAGTTT TTTTTGATTA AGTAGTTGAA CTTTTTTTTT TTGTTTATGT   
  
  
- TTTACCTAAT TTTTGTATTT TGATCAAACC CTTTTTTTTA AAGAGAGAGA TTGATCTAGA TTTGAAAGGT   
  
  
- CGTTATATTT ATGAATTTTT ATAAAATTAT TGTAAAAAAA TTGTATAACT ATAGTTTAAA TTGGTAATAA   
  
  
- AAGTTATTTT AAGTTGGTTG GTTAAAGACA GAGTAGTAAG CACAAGGATA CTAAGAGAGA AAAAGAAAGT   
  
  
- TTGAGAGTGT TCCATTTATT TTAATATAAT ACACGAACTT TCCTTTAAAC TTTTTAGATT TCCTTTTGAA   
  
  
- TTGAATTTTC CACGACTGTA AAAAATAATT TTTTTCGAGT ATAACAAAAA AGAAATTAAA ACTGAAATAA   
  
  
- GAAAAGAAAA AGTTTATTAT TTTAATCTAA GCAAAGGTAT TTTAAATTAC TTGAAAATCC GTTATTACAA   
  
  
- CATGCTTCAG TTATTACTGC ACGATGTTAG CATACAGTGC TGTTTTTCTG CTGTACTTGT GGTAGGTAAC   
  
  
- CATAAGTACA CGGAAAAGTG GAACATTTTA TTGTCCTCTT TCTATGGAAG GTCCCCGTTT AAGTAGATAC   
  
  
- GGTGCACGGA TGTTCAACTT CCATACAAAC CATACGTTC

+     ATCT-motif

| Site Name | Organism | Position | Strand | Matrix score. | sequence | function |
| --- | --- | --- | --- | --- | --- | --- |
| ATCT-motif | Pisum sativum | 595 | + | 9 | AATCTAATCC | part of a conserved DNA module involved in light responsiveness |

>Potri.011G061700.1   
+ TGTATAATAA AACAAGTGCA TATATATCAT CATGACACAA AAATAGAAAT ATAGGCCATA ATATTAAATT   
  
  
+ AGGCATCTAT TGCACCAGCC ATGACAGACA TTTGGTTAAT AATATAATAC TCATTGATAA AGGTGTCAAT   
  
  
+ GACACAAATC TATGATCCTA GTGTTGGAAA ATTTAAATTA TTTAAATAAC ATGGTTAGTT GATATATTAT   
  
  
+ TTTTCTTATA ATTAGGTAGA TTGAGGGGGG AAATGATATT TTTATAAGTA ATAAATATTG ATAAAACAAG   
  
  
+ TAGTAGACAC CTGTGTCGCA CATGTCTACA CGTTGGTGTT GGTGTGCGAC GTTTATTAAT GCTTAAATGG   
  
  
+ TGGTTTCTAG GACTAGTATG CGGGTTTGGA CAGCACGTGC GACGTTTAAT TTCATCCTCA TTAAATTAGT   
  
  
+ GTTTATTTTT TTATTTTTTA TTTTTTATTT TTTTATTTTT TTAATTGAAT TTATTTTTTA ATTTTATCAC   
  
  
+ TCATTATTTA ATTTAATTTT ATTTGTATGT TAAATTTAGT TTTTATTCTT TTTATTTCTA TTTGTTTTAC   
  
  
+ TTTAGAAAAA TTTTTGATTA AAATTTTTTT TTTCAATATA ATCCTTTAGA TTTAGTTTTT TTTTCTTTTT   
  
  
+ AATTTTAGCT CTCAACATTT GAATTGTAAG GATGTGGTTT GACTTAAAAA AATTTCAAGA TAACATGTTT   
  
  
+ TTAAATATTG GAATGACAAC ATATTAGATC AATCCAAGTC GACTCACTAG TAAAAAAAAT AAACTTATTA   
  
  
+ AATCCACATT GGTCATGGAT CCAAGTAGAT TTAATAATTT TTTTAAACCA GTTTTTATTT AATTACATGA   
  
  
+ TAAAAAATAA GCGATAGTAG TTAAACAACC AAATTAAATT TACAAATTGA TTACAATGGA TTGTAAAAAA   
  
  
+ CACTTGCTAA TATTACAACA AATATTTCTT TTGTTTTATT CAAGAACAAA GTGAAAATGA AATGAAATTT   
  
  
+ GATAAAACAA TATAAAAAAT ATATCAATTA ACTTAATATT TTTGAAAACA ATATATAATA TATTTTATAT   
  
  
+ TCATTAATTT TTAAGAAATT AATTTTAATT ATTTGAAGAA AAATAAAAAA AATAGACCAA GCGCGAGGGC   
  
  
+ TTGCCAACCC AGGCCAACAC TTGGGTTTTT TGTTGCTTTT TAAACAAGAC AGATGACATG TCGTCTACAG   
  
  
+ AGGAGAGTGA TATGTCATCT GTAGAGTTAG ACAACATGTC ATCTGCAAAC CTATTTTTTA ACATCTAGAG   
  
  
+ AGTACTGGAA AATCTAGCTG GGTATTTGTT TTTGTCCAAA AACTTAATTT TAATTTATTT TTATCTAAAG   
  
  
+ ACACCTAGAG AAAACCATTA TGAACTCAAA AAAAACTAAT TCATCAACTT GAAAAAAAAA AACAAATACA   
  
  
+ AAATGGATTA AAAACATAAA ACTAGTTTGG GAAAAAAAAT TTCTCTCTCT AACTAGATCT AAACTTTCCA   
  
  
+ GCAATATAAA TACTTAAAAA TATTTTAATA ACATTTTTTT AACATATTGA TATCAAATTT AACCATTATT   
  
  
+ TTCAATAAAA TTCAACCAAC CAATTTCTGT CTCATCATTC GTGTTCCTAT GATTCTCTCT TTTTCTTTCA   
  
  
+ AACTCTCACA AGGTAAATAA AATTATATTA TGTGCTTGAA AGGAAATTTG AAAAATCTAA AGGAAAACTT   
  
  
+ AACTTAAAAG GTGCTGACAT TTTTTATTAA AAAAAGCTCA TATTGTTTTT TCTTTAATTT TGACTTTATT   
  
  
+ CTTTTCTTTT TCAAATAATA AAATTAGATT CGTTTCCATA AAATTTAATG AACTTTTAGG CAATAATGTT   
  
  
+ GTACGAAGTC AATAATGACG TGCTACAATC GTATGTCACG ACAAAAAGAC GACATGAACA CCATCCATTG   
  
  
+ GTATTCATGT GCCTTTTCAC CTTGTAAAAT AACAGGAGAA AGATACCTTC CAGGGGCAAA TTCATCTATG   
  
  
+ CCACGTGCCT ACAAGTTGAA GGTATGTTTG GTATGCAAG  

- ACATATTATT TTGTTCACGT ATATATAGTA GTACTGTGTT TTTATCTTTA TATCCGGTAT TATAATTTAA   
  
  
- TCCGTAGATA ACGTGGTCGG TACTGTCTGT AAACCAATTA TTATATTATG AGTAACTATT TCCACAGTTA   
  
  
- CTGTGTTTAG ATACTAGGAT CACAACCTTT TAAATTTAAT AAATTTATTG TACCAATCAA CTATATAATA   
  
  
- AAAAGAATAT TAATCCATCT AACTCCCCCC TTTACTATAA AAATATTCAT TATTTATAAC TATTTTGTTC   
  
  
- ATCATCTGTG GACACAGCGT GTACAGATGT GCAACCACAA CCACACGCTG CAAATAATTA CGAATTTACC   
  
  
- ACCAAAGATC CTGATCATAC GCCCAAACCT GTCGTGCACG CTGCAAATTA AAGTAGGAGT AATTTAATCA   
  
  
- CAAATAAAAA AATAAAAAAT AAAAAATAAA AAAATAAAAA AATTAACTTA AATAAAAAAT TAAAATAGTG   
  
  
- AGTAATAAAT TAAATTAAAA TAAACATACA ATTTAAATCA AAAATAAGAA AAATAAAGAT AAACAAAATG   
  
  
- AAATCTTTTT AAAAACTAAT TTTAAAAAAA AAAGTTATAT TAGGAAATCT AAATCAAAAA AAAAGAAAAA   
  
  
- TTAAAATCGA GAGTTGTAAA CTTAACATTC CTACACCAAA CTGAATTTTT TTAAAGTTCT ATTGTACAAA   
  
  
- AATTTATAAC CTTACTGTTG TATAATCTAG TTAGGTTCAG CTGAGTGATC ATTTTTTTTA TTTGAATAAT   
  
  
- TTAGGTGTAA CCAGTACCTA GGTTCATCTA AATTATTAAA AAAATTTGGT CAAAAATAAA TTAATGTACT   
  
  
- ATTTTTTATT CGCTATCATC AATTTGTTGG TTTAATTTAA ATGTTTAACT AATGTTACCT AACATTTTTT   
  
  
- GTGAACGATT ATAATGTTGT TTATAAAGAA AACAAAATAA GTTCTTGTTT CACTTTTACT TTACTTTAAA   
  
  
- CTATTTTGTT ATATTTTTTA TATAGTTAAT TGAATTATAA AAACTTTTGT TATATATTAT ATAAAATATA   
  
  
- AGTAATTAAA AATTCTTTAA TTAAAATTAA TAAACTTCTT TTTATTTTTT TTATCTGGTT CGCGCTCCCG   
  
  
- AACGGTTGGG TCCGGTTGTG AACCCAAAAA ACAACGAAAA ATTTGTTCTG TCTACTGTAC AGCAGATGTC   
  
  
- TCCTCTCACT ATACAGTAGA CATCTCAATC TGTTGTACAG TAGACGTTTG GATAAAAAAT TGTAGATCTC   
  
  
- TCATGACCTT TTAGATCGAC CCATAAACAA AAACAGGTTT TTGAATTAAA ATTAAATAAA AATAGATTTC   
  
  
- TGTGGATCTC TTTTGGTAAT ACTTGAGTTT TTTTTGATTA AGTAGTTGAA CTTTTTTTTT TTGTTTATGT   
  
  
- TTTACCTAAT TTTTGTATTT TGATCAAACC CTTTTTTTTA AAGAGAGAGA TTGATCTAGA TTTGAAAGGT   
  
  
- CGTTATATTT ATGAATTTTT ATAAAATTAT TGTAAAAAAA TTGTATAACT ATAGTTTAAA TTGGTAATAA   
  
  
- AAGTTATTTT AAGTTGGTTG GTTAAAGACA GAGTAGTAAG CACAAGGATA CTAAGAGAGA AAAAGAAAGT   
  
  
- TTGAGAGTGT TCCATTTATT TTAATATAAT ACACGAACTT TCCTTTAAAC TTTTTAGATT TCCTTTTGAA   
  
  
- TTGAATTTTC CACGACTGTA AAAAATAATT TTTTTCGAGT ATAACAAAAA AGAAATTAAA ACTGAAATAA   
  
  
- GAAAAGAAAA AGTTTATTAT TTTAATCTAA GCAAAGGTAT TTTAAATTAC TTGAAAATCC GTTATTACAA   
  
  
- CATGCTTCAG TTATTACTGC ACGATGTTAG CATACAGTGC TGTTTTTCTG CTGTACTTGT GGTAGGTAAC   
  
  
- CATAAGTACA CGGAAAAGTG GAACATTTTA TTGTCCTCTT TCTATGGAAG GTCCCCGTTT AAGTAGATAC   
  
  
- GGTGCACGGA TGTTCAACTT CCATACAAAC CATACGTTC

+     AT~TATA-box

| Site Name | Organism | Position | Strand | Matrix score. | sequence | function |
| --- | --- | --- | --- | --- | --- | --- |
| AT~TATA-box | Arabidopsis thaliana | 1032 | - | 6 | TATATA |  |
| AT~TATA-box | Arabidopsis thaliana | 21 | + | 6 | TATATA |  |

>Potri.011G061700.1   
+ TGTATAATAA AACAAGTGCA TATATATCAT CATGACACAA AAATAGAAAT ATAGGCCATA ATATTAAATT   
  
  
+ AGGCATCTAT TGCACCAGCC ATGACAGACA TTTGGTTAAT AATATAATAC TCATTGATAA AGGTGTCAAT   
  
  
+ GACACAAATC TATGATCCTA GTGTTGGAAA ATTTAAATTA TTTAAATAAC ATGGTTAGTT GATATATTAT   
  
  
+ TTTTCTTATA ATTAGGTAGA TTGAGGGGGG AAATGATATT TTTATAAGTA ATAAATATTG ATAAAACAAG   
  
  
+ TAGTAGACAC CTGTGTCGCA CATGTCTACA CGTTGGTGTT GGTGTGCGAC GTTTATTAAT GCTTAAATGG   
  
  
+ TGGTTTCTAG GACTAGTATG CGGGTTTGGA CAGCACGTGC GACGTTTAAT TTCATCCTCA TTAAATTAGT   
  
  
+ GTTTATTTTT TTATTTTTTA TTTTTTATTT TTTTATTTTT TTAATTGAAT TTATTTTTTA ATTTTATCAC   
  
  
+ TCATTATTTA ATTTAATTTT ATTTGTATGT TAAATTTAGT TTTTATTCTT TTTATTTCTA TTTGTTTTAC   
  
  
+ TTTAGAAAAA TTTTTGATTA AAATTTTTTT TTTCAATATA ATCCTTTAGA TTTAGTTTTT TTTTCTTTTT   
  
  
+ AATTTTAGCT CTCAACATTT GAATTGTAAG GATGTGGTTT GACTTAAAAA AATTTCAAGA TAACATGTTT   
  
  
+ TTAAATATTG GAATGACAAC ATATTAGATC AATCCAAGTC GACTCACTAG TAAAAAAAAT AAACTTATTA   
  
  
+ AATCCACATT GGTCATGGAT CCAAGTAGAT TTAATAATTT TTTTAAACCA GTTTTTATTT AATTACATGA   
  
  
+ TAAAAAATAA GCGATAGTAG TTAAACAACC AAATTAAATT TACAAATTGA TTACAATGGA TTGTAAAAAA   
  
  
+ CACTTGCTAA TATTACAACA AATATTTCTT TTGTTTTATT CAAGAACAAA GTGAAAATGA AATGAAATTT   
  
  
+ GATAAAACAA TATAAAAAAT ATATCAATTA ACTTAATATT TTTGAAAACA ATATATAATA TATTTTATAT   
  
  
+ TCATTAATTT TTAAGAAATT AATTTTAATT ATTTGAAGAA AAATAAAAAA AATAGACCAA GCGCGAGGGC   
  
  
+ TTGCCAACCC AGGCCAACAC TTGGGTTTTT TGTTGCTTTT TAAACAAGAC AGATGACATG TCGTCTACAG   
  
  
+ AGGAGAGTGA TATGTCATCT GTAGAGTTAG ACAACATGTC ATCTGCAAAC CTATTTTTTA ACATCTAGAG   
  
  
+ AGTACTGGAA AATCTAGCTG GGTATTTGTT TTTGTCCAAA AACTTAATTT TAATTTATTT TTATCTAAAG   
  
  
+ ACACCTAGAG AAAACCATTA TGAACTCAAA AAAAACTAAT TCATCAACTT GAAAAAAAAA AACAAATACA   
  
  
+ AAATGGATTA AAAACATAAA ACTAGTTTGG GAAAAAAAAT TTCTCTCTCT AACTAGATCT AAACTTTCCA   
  
  
+ GCAATATAAA TACTTAAAAA TATTTTAATA ACATTTTTTT AACATATTGA TATCAAATTT AACCATTATT   
  
  
+ TTCAATAAAA TTCAACCAAC CAATTTCTGT CTCATCATTC GTGTTCCTAT GATTCTCTCT TTTTCTTTCA   
  
  
+ AACTCTCACA AGGTAAATAA AATTATATTA TGTGCTTGAA AGGAAATTTG AAAAATCTAA AGGAAAACTT   
  
  
+ AACTTAAAAG GTGCTGACAT TTTTTATTAA AAAAAGCTCA TATTGTTTTT TCTTTAATTT TGACTTTATT   
  
  
+ CTTTTCTTTT TCAAATAATA AAATTAGATT CGTTTCCATA AAATTTAATG AACTTTTAGG CAATAATGTT   
  
  
+ GTACGAAGTC AATAATGACG TGCTACAATC GTATGTCACG ACAAAAAGAC GACATGAACA CCATCCATTG   
  
  
+ GTATTCATGT GCCTTTTCAC CTTGTAAAAT AACAGGAGAA AGATACCTTC CAGGGGCAAA TTCATCTATG   
  
  
+ CCACGTGCCT ACAAGTTGAA GGTATGTTTG GTATGCAAG  

- ACATATTATT TTGTTCACGT ATATATAGTA GTACTGTGTT TTTATCTTTA TATCCGGTAT TATAATTTAA   
  
  
- TCCGTAGATA ACGTGGTCGG TACTGTCTGT AAACCAATTA TTATATTATG AGTAACTATT TCCACAGTTA   
  
  
- CTGTGTTTAG ATACTAGGAT CACAACCTTT TAAATTTAAT AAATTTATTG TACCAATCAA CTATATAATA   
  
  
- AAAAGAATAT TAATCCATCT AACTCCCCCC TTTACTATAA AAATATTCAT TATTTATAAC TATTTTGTTC   
  
  
- ATCATCTGTG GACACAGCGT GTACAGATGT GCAACCACAA CCACACGCTG CAAATAATTA CGAATTTACC   
  
  
- ACCAAAGATC CTGATCATAC GCCCAAACCT GTCGTGCACG CTGCAAATTA AAGTAGGAGT AATTTAATCA   
  
  
- CAAATAAAAA AATAAAAAAT AAAAAATAAA AAAATAAAAA AATTAACTTA AATAAAAAAT TAAAATAGTG   
  
  
- AGTAATAAAT TAAATTAAAA TAAACATACA ATTTAAATCA AAAATAAGAA AAATAAAGAT AAACAAAATG   
  
  
- AAATCTTTTT AAAAACTAAT TTTAAAAAAA AAAGTTATAT TAGGAAATCT AAATCAAAAA AAAAGAAAAA   
  
  
- TTAAAATCGA GAGTTGTAAA CTTAACATTC CTACACCAAA CTGAATTTTT TTAAAGTTCT ATTGTACAAA   
  
  
- AATTTATAAC CTTACTGTTG TATAATCTAG TTAGGTTCAG CTGAGTGATC ATTTTTTTTA TTTGAATAAT   
  
  
- TTAGGTGTAA CCAGTACCTA GGTTCATCTA AATTATTAAA AAAATTTGGT CAAAAATAAA TTAATGTACT   
  
  
- ATTTTTTATT CGCTATCATC AATTTGTTGG TTTAATTTAA ATGTTTAACT AATGTTACCT AACATTTTTT   
  
  
- GTGAACGATT ATAATGTTGT TTATAAAGAA AACAAAATAA GTTCTTGTTT CACTTTTACT TTACTTTAAA   
  
  
- CTATTTTGTT ATATTTTTTA TATAGTTAAT TGAATTATAA AAACTTTTGT TATATATTAT ATAAAATATA   
  
  
- AGTAATTAAA AATTCTTTAA TTAAAATTAA TAAACTTCTT TTTATTTTTT TTATCTGGTT CGCGCTCCCG   
  
  
- AACGGTTGGG TCCGGTTGTG AACCCAAAAA ACAACGAAAA ATTTGTTCTG TCTACTGTAC AGCAGATGTC   
  
  
- TCCTCTCACT ATACAGTAGA CATCTCAATC TGTTGTACAG TAGACGTTTG GATAAAAAAT TGTAGATCTC   
  
  
- TCATGACCTT TTAGATCGAC CCATAAACAA AAACAGGTTT TTGAATTAAA ATTAAATAAA AATAGATTTC   
  
  
- TGTGGATCTC TTTTGGTAAT ACTTGAGTTT TTTTTGATTA AGTAGTTGAA CTTTTTTTTT TTGTTTATGT   
  
  
- TTTACCTAAT TTTTGTATTT TGATCAAACC CTTTTTTTTA AAGAGAGAGA TTGATCTAGA TTTGAAAGGT   
  
  
- CGTTATATTT ATGAATTTTT ATAAAATTAT TGTAAAAAAA TTGTATAACT ATAGTTTAAA TTGGTAATAA   
  
  
- AAGTTATTTT AAGTTGGTTG GTTAAAGACA GAGTAGTAAG CACAAGGATA CTAAGAGAGA AAAAGAAAGT   
  
  
- TTGAGAGTGT TCCATTTATT TTAATATAAT ACACGAACTT TCCTTTAAAC TTTTTAGATT TCCTTTTGAA   
  
  
- TTGAATTTTC CACGACTGTA AAAAATAATT TTTTTCGAGT ATAACAAAAA AGAAATTAAA ACTGAAATAA   
  
  
- GAAAAGAAAA AGTTTATTAT TTTAATCTAA GCAAAGGTAT TTTAAATTAC TTGAAAATCC GTTATTACAA   
  
  
- CATGCTTCAG TTATTACTGC ACGATGTTAG CATACAGTGC TGTTTTTCTG CTGTACTTGT GGTAGGTAAC   
  
  
- CATAAGTACA CGGAAAAGTG GAACATTTTA TTGTCCTCTT TCTATGGAAG GTCCCCGTTT AAGTAGATAC   
  
  
- GGTGCACGGA TGTTCAACTT CCATACAAAC CATACGTTC

+     Box 4

| Site Name | Organism | Position | Strand | Matrix score. | sequence | function |
| --- | --- | --- | --- | --- | --- | --- |
| Box 4 | Petroselinum crispum | 1068 | - | 6 | ATTAAT | part of a conserved DNA module involved in light responsiveness |
| Box 4 | Petroselinum crispum | 335 | + | 6 | ATTAAT | part of a conserved DNA module involved in light responsiveness |
| Box 4 | Petroselinum crispum | 1053 | - | 6 | ATTAAT | part of a conserved DNA module involved in light responsiveness |

>Potri.011G061700.1   
+ TGTATAATAA AACAAGTGCA TATATATCAT CATGACACAA AAATAGAAAT ATAGGCCATA ATATTAAATT   
  
  
+ AGGCATCTAT TGCACCAGCC ATGACAGACA TTTGGTTAAT AATATAATAC TCATTGATAA AGGTGTCAAT   
  
  
+ GACACAAATC TATGATCCTA GTGTTGGAAA ATTTAAATTA TTTAAATAAC ATGGTTAGTT GATATATTAT   
  
  
+ TTTTCTTATA ATTAGGTAGA TTGAGGGGGG AAATGATATT TTTATAAGTA ATAAATATTG ATAAAACAAG   
  
  
+ TAGTAGACAC CTGTGTCGCA CATGTCTACA CGTTGGTGTT GGTGTGCGAC GTTTATTAAT GCTTAAATGG   
  
  
+ TGGTTTCTAG GACTAGTATG CGGGTTTGGA CAGCACGTGC GACGTTTAAT TTCATCCTCA TTAAATTAGT   
  
  
+ GTTTATTTTT TTATTTTTTA TTTTTTATTT TTTTATTTTT TTAATTGAAT TTATTTTTTA ATTTTATCAC   
  
  
+ TCATTATTTA ATTTAATTTT ATTTGTATGT TAAATTTAGT TTTTATTCTT TTTATTTCTA TTTGTTTTAC   
  
  
+ TTTAGAAAAA TTTTTGATTA AAATTTTTTT TTTCAATATA ATCCTTTAGA TTTAGTTTTT TTTTCTTTTT   
  
  
+ AATTTTAGCT CTCAACATTT GAATTGTAAG GATGTGGTTT GACTTAAAAA AATTTCAAGA TAACATGTTT   
  
  
+ TTAAATATTG GAATGACAAC ATATTAGATC AATCCAAGTC GACTCACTAG TAAAAAAAAT AAACTTATTA   
  
  
+ AATCCACATT GGTCATGGAT CCAAGTAGAT TTAATAATTT TTTTAAACCA GTTTTTATTT AATTACATGA   
  
  
+ TAAAAAATAA GCGATAGTAG TTAAACAACC AAATTAAATT TACAAATTGA TTACAATGGA TTGTAAAAAA   
  
  
+ CACTTGCTAA TATTACAACA AATATTTCTT TTGTTTTATT CAAGAACAAA GTGAAAATGA AATGAAATTT   
  
  
+ GATAAAACAA TATAAAAAAT ATATCAATTA ACTTAATATT TTTGAAAACA ATATATAATA TATTTTATAT   
  
  
+ TCATTAATTT TTAAGAAATT AATTTTAATT ATTTGAAGAA AAATAAAAAA AATAGACCAA GCGCGAGGGC   
  
  
+ TTGCCAACCC AGGCCAACAC TTGGGTTTTT TGTTGCTTTT TAAACAAGAC AGATGACATG TCGTCTACAG   
  
  
+ AGGAGAGTGA TATGTCATCT GTAGAGTTAG ACAACATGTC ATCTGCAAAC CTATTTTTTA ACATCTAGAG   
  
  
+ AGTACTGGAA AATCTAGCTG GGTATTTGTT TTTGTCCAAA AACTTAATTT TAATTTATTT TTATCTAAAG   
  
  
+ ACACCTAGAG AAAACCATTA TGAACTCAAA AAAAACTAAT TCATCAACTT GAAAAAAAAA AACAAATACA   
  
  
+ AAATGGATTA AAAACATAAA ACTAGTTTGG GAAAAAAAAT TTCTCTCTCT AACTAGATCT AAACTTTCCA   
  
  
+ GCAATATAAA TACTTAAAAA TATTTTAATA ACATTTTTTT AACATATTGA TATCAAATTT AACCATTATT   
  
  
+ TTCAATAAAA TTCAACCAAC CAATTTCTGT CTCATCATTC GTGTTCCTAT GATTCTCTCT TTTTCTTTCA   
  
  
+ AACTCTCACA AGGTAAATAA AATTATATTA TGTGCTTGAA AGGAAATTTG AAAAATCTAA AGGAAAACTT   
  
  
+ AACTTAAAAG GTGCTGACAT TTTTTATTAA AAAAAGCTCA TATTGTTTTT TCTTTAATTT TGACTTTATT   
  
  
+ CTTTTCTTTT TCAAATAATA AAATTAGATT CGTTTCCATA AAATTTAATG AACTTTTAGG CAATAATGTT   
  
  
+ GTACGAAGTC AATAATGACG TGCTACAATC GTATGTCACG ACAAAAAGAC GACATGAACA CCATCCATTG   
  
  
+ GTATTCATGT GCCTTTTCAC CTTGTAAAAT AACAGGAGAA AGATACCTTC CAGGGGCAAA TTCATCTATG   
  
  
+ CCACGTGCCT ACAAGTTGAA GGTATGTTTG GTATGCAAG  

- ACATATTATT TTGTTCACGT ATATATAGTA GTACTGTGTT TTTATCTTTA TATCCGGTAT TATAATTTAA   
  
  
- TCCGTAGATA ACGTGGTCGG TACTGTCTGT AAACCAATTA TTATATTATG AGTAACTATT TCCACAGTTA   
  
  
- CTGTGTTTAG ATACTAGGAT CACAACCTTT TAAATTTAAT AAATTTATTG TACCAATCAA CTATATAATA   
  
  
- AAAAGAATAT TAATCCATCT AACTCCCCCC TTTACTATAA AAATATTCAT TATTTATAAC TATTTTGTTC   
  
  
- ATCATCTGTG GACACAGCGT GTACAGATGT GCAACCACAA CCACACGCTG CAAATAATTA CGAATTTACC   
  
  
- ACCAAAGATC CTGATCATAC GCCCAAACCT GTCGTGCACG CTGCAAATTA AAGTAGGAGT AATTTAATCA   
  
  
- CAAATAAAAA AATAAAAAAT AAAAAATAAA AAAATAAAAA AATTAACTTA AATAAAAAAT TAAAATAGTG   
  
  
- AGTAATAAAT TAAATTAAAA TAAACATACA ATTTAAATCA AAAATAAGAA AAATAAAGAT AAACAAAATG   
  
  
- AAATCTTTTT AAAAACTAAT TTTAAAAAAA AAAGTTATAT TAGGAAATCT AAATCAAAAA AAAAGAAAAA   
  
  
- TTAAAATCGA GAGTTGTAAA CTTAACATTC CTACACCAAA CTGAATTTTT TTAAAGTTCT ATTGTACAAA   
  
  
- AATTTATAAC CTTACTGTTG TATAATCTAG TTAGGTTCAG CTGAGTGATC ATTTTTTTTA TTTGAATAAT   
  
  
- TTAGGTGTAA CCAGTACCTA GGTTCATCTA AATTATTAAA AAAATTTGGT CAAAAATAAA TTAATGTACT   
  
  
- ATTTTTTATT CGCTATCATC AATTTGTTGG TTTAATTTAA ATGTTTAACT AATGTTACCT AACATTTTTT   
  
  
- GTGAACGATT ATAATGTTGT TTATAAAGAA AACAAAATAA GTTCTTGTTT CACTTTTACT TTACTTTAAA   
  
  
- CTATTTTGTT ATATTTTTTA TATAGTTAAT TGAATTATAA AAACTTTTGT TATATATTAT ATAAAATATA   
  
  
- AGTAATTAAA AATTCTTTAA TTAAAATTAA TAAACTTCTT TTTATTTTTT TTATCTGGTT CGCGCTCCCG   
  
  
- AACGGTTGGG TCCGGTTGTG AACCCAAAAA ACAACGAAAA ATTTGTTCTG TCTACTGTAC AGCAGATGTC   
  
  
- TCCTCTCACT ATACAGTAGA CATCTCAATC TGTTGTACAG TAGACGTTTG GATAAAAAAT TGTAGATCTC   
  
  
- TCATGACCTT TTAGATCGAC CCATAAACAA AAACAGGTTT TTGAATTAAA ATTAAATAAA AATAGATTTC   
  
  
- TGTGGATCTC TTTTGGTAAT ACTTGAGTTT TTTTTGATTA AGTAGTTGAA CTTTTTTTTT TTGTTTATGT   
  
  
- TTTACCTAAT TTTTGTATTT TGATCAAACC CTTTTTTTTA AAGAGAGAGA TTGATCTAGA TTTGAAAGGT   
  
  
- CGTTATATTT ATGAATTTTT ATAAAATTAT TGTAAAAAAA TTGTATAACT ATAGTTTAAA TTGGTAATAA   
  
  
- AAGTTATTTT AAGTTGGTTG GTTAAAGACA GAGTAGTAAG CACAAGGATA CTAAGAGAGA AAAAGAAAGT   
  
  
- TTGAGAGTGT TCCATTTATT TTAATATAAT ACACGAACTT TCCTTTAAAC TTTTTAGATT TCCTTTTGAA   
  
  
- TTGAATTTTC CACGACTGTA AAAAATAATT TTTTTCGAGT ATAACAAAAA AGAAATTAAA ACTGAAATAA   
  
  
- GAAAAGAAAA AGTTTATTAT TTTAATCTAA GCAAAGGTAT TTTAAATTAC TTGAAAATCC GTTATTACAA   
  
  
- CATGCTTCAG TTATTACTGC ACGATGTTAG CATACAGTGC TGTTTTTCTG CTGTACTTGT GGTAGGTAAC   
  
  
- CATAAGTACA CGGAAAAGTG GAACATTTTA TTGTCCTCTT TCTATGGAAG GTCCCCGTTT AAGTAGATAC   
  
  
- GGTGCACGGA TGTTCAACTT CCATACAAAC CATACGTTC

+     Box III

| Site Name | Organism | Position | Strand | Matrix score. | sequence | function |
| --- | --- | --- | --- | --- | --- | --- |
| Box III | Pisum sativum | 960 | - | 11 | atCATTTTCACt | protein binding site |

>Potri.011G061700.1   
+ TGTATAATAA AACAAGTGCA TATATATCAT CATGACACAA AAATAGAAAT ATAGGCCATA ATATTAAATT   
  
  
+ AGGCATCTAT TGCACCAGCC ATGACAGACA TTTGGTTAAT AATATAATAC TCATTGATAA AGGTGTCAAT   
  
  
+ GACACAAATC TATGATCCTA GTGTTGGAAA ATTTAAATTA TTTAAATAAC ATGGTTAGTT GATATATTAT   
  
  
+ TTTTCTTATA ATTAGGTAGA TTGAGGGGGG AAATGATATT TTTATAAGTA ATAAATATTG ATAAAACAAG   
  
  
+ TAGTAGACAC CTGTGTCGCA CATGTCTACA CGTTGGTGTT GGTGTGCGAC GTTTATTAAT GCTTAAATGG   
  
  
+ TGGTTTCTAG GACTAGTATG CGGGTTTGGA CAGCACGTGC GACGTTTAAT TTCATCCTCA TTAAATTAGT   
  
  
+ GTTTATTTTT TTATTTTTTA TTTTTTATTT TTTTATTTTT TTAATTGAAT TTATTTTTTA ATTTTATCAC   
  
  
+ TCATTATTTA ATTTAATTTT ATTTGTATGT TAAATTTAGT TTTTATTCTT TTTATTTCTA TTTGTTTTAC   
  
  
+ TTTAGAAAAA TTTTTGATTA AAATTTTTTT TTTCAATATA ATCCTTTAGA TTTAGTTTTT TTTTCTTTTT   
  
  
+ AATTTTAGCT CTCAACATTT GAATTGTAAG GATGTGGTTT GACTTAAAAA AATTTCAAGA TAACATGTTT   
  
  
+ TTAAATATTG GAATGACAAC ATATTAGATC AATCCAAGTC GACTCACTAG TAAAAAAAAT AAACTTATTA   
  
  
+ AATCCACATT GGTCATGGAT CCAAGTAGAT TTAATAATTT TTTTAAACCA GTTTTTATTT AATTACATGA   
  
  
+ TAAAAAATAA GCGATAGTAG TTAAACAACC AAATTAAATT TACAAATTGA TTACAATGGA TTGTAAAAAA   
  
  
+ CACTTGCTAA TATTACAACA AATATTTCTT TTGTTTTATT CAAGAACAAA GTGAAAATGA AATGAAATTT   
  
  
+ GATAAAACAA TATAAAAAAT ATATCAATTA ACTTAATATT TTTGAAAACA ATATATAATA TATTTTATAT   
  
  
+ TCATTAATTT TTAAGAAATT AATTTTAATT ATTTGAAGAA AAATAAAAAA AATAGACCAA GCGCGAGGGC   
  
  
+ TTGCCAACCC AGGCCAACAC TTGGGTTTTT TGTTGCTTTT TAAACAAGAC AGATGACATG TCGTCTACAG   
  
  
+ AGGAGAGTGA TATGTCATCT GTAGAGTTAG ACAACATGTC ATCTGCAAAC CTATTTTTTA ACATCTAGAG   
  
  
+ AGTACTGGAA AATCTAGCTG GGTATTTGTT TTTGTCCAAA AACTTAATTT TAATTTATTT TTATCTAAAG   
  
  
+ ACACCTAGAG AAAACCATTA TGAACTCAAA AAAAACTAAT TCATCAACTT GAAAAAAAAA AACAAATACA   
  
  
+ AAATGGATTA AAAACATAAA ACTAGTTTGG GAAAAAAAAT TTCTCTCTCT AACTAGATCT AAACTTTCCA   
  
  
+ GCAATATAAA TACTTAAAAA TATTTTAATA ACATTTTTTT AACATATTGA TATCAAATTT AACCATTATT   
  
  
+ TTCAATAAAA TTCAACCAAC CAATTTCTGT CTCATCATTC GTGTTCCTAT GATTCTCTCT TTTTCTTTCA   
  
  
+ AACTCTCACA AGGTAAATAA AATTATATTA TGTGCTTGAA AGGAAATTTG AAAAATCTAA AGGAAAACTT   
  
  
+ AACTTAAAAG GTGCTGACAT TTTTTATTAA AAAAAGCTCA TATTGTTTTT TCTTTAATTT TGACTTTATT   
  
  
+ CTTTTCTTTT TCAAATAATA AAATTAGATT CGTTTCCATA AAATTTAATG AACTTTTAGG CAATAATGTT   
  
  
+ GTACGAAGTC AATAATGACG TGCTACAATC GTATGTCACG ACAAAAAGAC GACATGAACA CCATCCATTG   
  
  
+ GTATTCATGT GCCTTTTCAC CTTGTAAAAT AACAGGAGAA AGATACCTTC CAGGGGCAAA TTCATCTATG   
  
  
+ CCACGTGCCT ACAAGTTGAA GGTATGTTTG GTATGCAAG  

- ACATATTATT TTGTTCACGT ATATATAGTA GTACTGTGTT TTTATCTTTA TATCCGGTAT TATAATTTAA   
  
  
- TCCGTAGATA ACGTGGTCGG TACTGTCTGT AAACCAATTA TTATATTATG AGTAACTATT TCCACAGTTA   
  
  
- CTGTGTTTAG ATACTAGGAT CACAACCTTT TAAATTTAAT AAATTTATTG TACCAATCAA CTATATAATA   
  
  
- AAAAGAATAT TAATCCATCT AACTCCCCCC TTTACTATAA AAATATTCAT TATTTATAAC TATTTTGTTC   
  
  
- ATCATCTGTG GACACAGCGT GTACAGATGT GCAACCACAA CCACACGCTG CAAATAATTA CGAATTTACC   
  
  
- ACCAAAGATC CTGATCATAC GCCCAAACCT GTCGTGCACG CTGCAAATTA AAGTAGGAGT AATTTAATCA   
  
  
- CAAATAAAAA AATAAAAAAT AAAAAATAAA AAAATAAAAA AATTAACTTA AATAAAAAAT TAAAATAGTG   
  
  
- AGTAATAAAT TAAATTAAAA TAAACATACA ATTTAAATCA AAAATAAGAA AAATAAAGAT AAACAAAATG   
  
  
- AAATCTTTTT AAAAACTAAT TTTAAAAAAA AAAGTTATAT TAGGAAATCT AAATCAAAAA AAAAGAAAAA   
  
  
- TTAAAATCGA GAGTTGTAAA CTTAACATTC CTACACCAAA CTGAATTTTT TTAAAGTTCT ATTGTACAAA   
  
  
- AATTTATAAC CTTACTGTTG TATAATCTAG TTAGGTTCAG CTGAGTGATC ATTTTTTTTA TTTGAATAAT   
  
  
- TTAGGTGTAA CCAGTACCTA GGTTCATCTA AATTATTAAA AAAATTTGGT CAAAAATAAA TTAATGTACT   
  
  
- ATTTTTTATT CGCTATCATC AATTTGTTGG TTTAATTTAA ATGTTTAACT AATGTTACCT AACATTTTTT   
  
  
- GTGAACGATT ATAATGTTGT TTATAAAGAA AACAAAATAA GTTCTTGTTT CACTTTTACT TTACTTTAAA   
  
  
- CTATTTTGTT ATATTTTTTA TATAGTTAAT TGAATTATAA AAACTTTTGT TATATATTAT ATAAAATATA   
  
  
- AGTAATTAAA AATTCTTTAA TTAAAATTAA TAAACTTCTT TTTATTTTTT TTATCTGGTT CGCGCTCCCG   
  
  
- AACGGTTGGG TCCGGTTGTG AACCCAAAAA ACAACGAAAA ATTTGTTCTG TCTACTGTAC AGCAGATGTC   
  
  
- TCCTCTCACT ATACAGTAGA CATCTCAATC TGTTGTACAG TAGACGTTTG GATAAAAAAT TGTAGATCTC   
  
  
- TCATGACCTT TTAGATCGAC CCATAAACAA AAACAGGTTT TTGAATTAAA ATTAAATAAA AATAGATTTC   
  
  
- TGTGGATCTC TTTTGGTAAT ACTTGAGTTT TTTTTGATTA AGTAGTTGAA CTTTTTTTTT TTGTTTATGT   
  
  
- TTTACCTAAT TTTTGTATTT TGATCAAACC CTTTTTTTTA AAGAGAGAGA TTGATCTAGA TTTGAAAGGT   
  
  
- CGTTATATTT ATGAATTTTT ATAAAATTAT TGTAAAAAAA TTGTATAACT ATAGTTTAAA TTGGTAATAA   
  
  
- AAGTTATTTT AAGTTGGTTG GTTAAAGACA GAGTAGTAAG CACAAGGATA CTAAGAGAGA AAAAGAAAGT   
  
  
- TTGAGAGTGT TCCATTTATT TTAATATAAT ACACGAACTT TCCTTTAAAC TTTTTAGATT TCCTTTTGAA   
  
  
- TTGAATTTTC CACGACTGTA AAAAATAATT TTTTTCGAGT ATAACAAAAA AGAAATTAAA ACTGAAATAA   
  
  
- GAAAAGAAAA AGTTTATTAT TTTAATCTAA GCAAAGGTAT TTTAAATTAC TTGAAAATCC GTTATTACAA   
  
  
- CATGCTTCAG TTATTACTGC ACGATGTTAG CATACAGTGC TGTTTTTCTG CTGTACTTGT GGTAGGTAAC   
  
  
- CATAAGTACA CGGAAAAGTG GAACATTTTA TTGTCCTCTT TCTATGGAAG GTCCCCGTTT AAGTAGATAC   
  
  
- GGTGCACGGA TGTTCAACTT CCATACAAAC CATACGTTC

+     CAAT-box

| Site Name | Organism | Position | Strand | Matrix score. | sequence | function |
| --- | --- | --- | --- | --- | --- | --- |
| CAAT-box | Pisum sativum | 1947 | + | 5 | CAAAT | common cis-acting element in promoter and enhancer regions |
| CAAT-box | Arabidopsis thaliana | 1887 | - | 5 | CCAAT | common cis-acting element in promoter and enhancer regions |
| CAAT-box | Nicotiana glutinosa | 1846 | + | 4 | CAAT |  |
| CAAT-box | Nicotiana glutinosa | 900 | - | 4 | CAAT |  |
| CAAT-box | Nicotiana glutinosa | 1722 | - | 4 | CAAT |  |
| CAAT-box | Pisum sativum | 929 | + | 5 | CAAAT | common cis-acting element in promoter and enhancer regions |
| CAAT-box | Pisum sativum | 1524 | + | 5 | CAAAT | common cis-acting element in promoter and enhancer regions |
| CAAT-box | Nicotiana glutinosa | 894 | + | 4 | CAAT |  |
| CAAT-box | Nicotiana glutinosa | 1472 | + | 4 | CAAT |  |
| CAAT-box | Nicotiana glutinosa | 1830 | + | 4 | CAAT |  |
| CAAT-box | Pisum sativum | 1656 | - | 5 | CAAAT | common cis-acting element in promoter and enhancer regions |
| CAAT-box | Arabidopsis thaliana | 1560 | + | 5 | CCAAT | common cis-acting element in promoter and enhancer regions |
| CAAT-box | Nicotiana glutinosa | 886 | - | 4 | CAAT |  |
| CAAT-box | Arabidopsis thaliana | 778 | - | 5 | CCAAT | common cis-acting element in promoter and enhancer regions |
| CAAT-box | Nicotiana glutinosa | 730 | + | 4 | CAAT |  |
| CAAT-box | Arabidopsis thaliana | 707 | - | 5 | CCAAT | common cis-acting element in promoter and enhancer regions |
| CAAT-box | Nicotiana glutinosa | 653 | - | 4 | CAAT |  |
| CAAT-box | Nicotiana glutinosa | 594 | + | 4 | CAAT |  |
| CAAT-box | Pisum sativum | 883 | + | 5 | CAAAT | common cis-acting element in promoter and enhancer regions |
| CAAT-box | Pisum sativum | 870 | + | 5 | CAAAT | common cis-acting element in promoter and enhancer regions |
| CAAT-box | Pisum sativum | 550 | - | 5 | CAAAT | common cis-acting element in promoter and enhancer regions |
| CAAT-box | Nicotiana glutinosa | 1543 | + | 4 | CAAT |  |
| CAAT-box | Nicotiana glutinosa | 1516 | - | 4 | CAAT |  |
| CAAT-box | Pisum sativum | 1284 | - | 5 | CAAAT | common cis-acting element in promoter and enhancer regions |
| CAAT-box | Pisum sativum | 1393 | + | 5 | CAAAT | common cis-acting element in promoter and enhancer regions |
| CAAT-box | Pisum sativum | 1081 | - | 5 | CAAAT | common cis-acting element in promoter and enhancer regions |
| CAAT-box | Pisum sativum | 1762 | + | 5 | CAAAT | common cis-acting element in promoter and enhancer regions |
| CAAT-box | Petunia hybrida | 1122 | + | 7 | TGCCAAC | common cis-acting element in promoter and enhancer regions |
| CAAT-box | Nicotiana glutinosa | 988 | + | 4 | CAAT |  |
| CAAT-box | Nicotiana glutinosa | 1029 | + | 4 | CAAT |  |
| CAAT-box | Pisum sativum | 647 | - | 5 | CAAAT | common cis-acting element in promoter and enhancer regions |
| CAAT-box | Nicotiana glutinosa | 79 | - | 4 | CAAT |  |
| CAAT-box | Nicotiana glutinosa | 123 | - | 4 | CAAT |  |
| CAAT-box | Pisum sativum | 100 | - | 5 | CAAAT | common cis-acting element in promoter and enhancer regions |
| CAAT-box | Pisum sativum | 145 | + | 5 | CAAAT | common cis-acting element in promoter and enhancer regions |
| CAAT-box | Nicotiana glutinosa | 1561 | + | 4 | CAAT |  |
| CAAT-box | Pisum sativum | 977 | - | 5 | CAAAT | common cis-acting element in promoter and enhancer regions |
| CAAT-box | Nicotiana glutinosa | 267 | - | 4 | CAAT |  |
| CAAT-box | Pisum sativum | 511 | - | 5 | CAAAT | common cis-acting element in promoter and enhancer regions |
| CAAT-box | Nicotiana glutinosa | 464 | - | 4 | CAAT |  |
| CAAT-box | Nicotiana glutinosa | 1811 | + | 4 | CAAT |  |
| CAAT-box | Nicotiana glutinosa | 137 | + | 4 | CAAT |  |
| CAAT-box | Nicotiana glutinosa | 1005 | + | 4 | CAAT |  |
| CAAT-box | Nicotiana glutinosa | 230 | - | 4 | CAAT |  |

>Potri.011G061700.1   
+ TGTATAATAA AACAAGTGCA TATATATCAT CATGACACAA AAATAGAAAT ATAGGCCATA ATATTAAATT   
  
  
+ AGGCATCTAT TGCACCAGCC ATGACAGACA TTTGGTTAAT AATATAATAC TCATTGATAA AGGTGTCAAT   
  
  
+ GACACAAATC TATGATCCTA GTGTTGGAAA ATTTAAATTA TTTAAATAAC ATGGTTAGTT GATATATTAT   
  
  
+ TTTTCTTATA ATTAGGTAGA TTGAGGGGGG AAATGATATT TTTATAAGTA ATAAATATTG ATAAAACAAG   
  
  
+ TAGTAGACAC CTGTGTCGCA CATGTCTACA CGTTGGTGTT GGTGTGCGAC GTTTATTAAT GCTTAAATGG   
  
  
+ TGGTTTCTAG GACTAGTATG CGGGTTTGGA CAGCACGTGC GACGTTTAAT TTCATCCTCA TTAAATTAGT   
  
  
+ GTTTATTTTT TTATTTTTTA TTTTTTATTT TTTTATTTTT TTAATTGAAT TTATTTTTTA ATTTTATCAC   
  
  
+ TCATTATTTA ATTTAATTTT ATTTGTATGT TAAATTTAGT TTTTATTCTT TTTATTTCTA TTTGTTTTAC   
  
  
+ TTTAGAAAAA TTTTTGATTA AAATTTTTTT TTTCAATATA ATCCTTTAGA TTTAGTTTTT TTTTCTTTTT   
  
  
+ AATTTTAGCT CTCAACATTT GAATTGTAAG GATGTGGTTT GACTTAAAAA AATTTCAAGA TAACATGTTT   
  
  
+ TTAAATATTG GAATGACAAC ATATTAGATC AATCCAAGTC GACTCACTAG TAAAAAAAAT AAACTTATTA   
  
  
+ AATCCACATT GGTCATGGAT CCAAGTAGAT TTAATAATTT TTTTAAACCA GTTTTTATTT AATTACATGA   
  
  
+ TAAAAAATAA GCGATAGTAG TTAAACAACC AAATTAAATT TACAAATTGA TTACAATGGA TTGTAAAAAA   
  
  
+ CACTTGCTAA TATTACAACA AATATTTCTT TTGTTTTATT CAAGAACAAA GTGAAAATGA AATGAAATTT   
  
  
+ GATAAAACAA TATAAAAAAT ATATCAATTA ACTTAATATT TTTGAAAACA ATATATAATA TATTTTATAT   
  
  
+ TCATTAATTT TTAAGAAATT AATTTTAATT ATTTGAAGAA AAATAAAAAA AATAGACCAA GCGCGAGGGC   
  
  
+ TTGCCAACCC AGGCCAACAC TTGGGTTTTT TGTTGCTTTT TAAACAAGAC AGATGACATG TCGTCTACAG   
  
  
+ AGGAGAGTGA TATGTCATCT GTAGAGTTAG ACAACATGTC ATCTGCAAAC CTATTTTTTA ACATCTAGAG   
  
  
+ AGTACTGGAA AATCTAGCTG GGTATTTGTT TTTGTCCAAA AACTTAATTT TAATTTATTT TTATCTAAAG   
  
  
+ ACACCTAGAG AAAACCATTA TGAACTCAAA AAAAACTAAT TCATCAACTT GAAAAAAAAA AACAAATACA   
  
  
+ AAATGGATTA AAAACATAAA ACTAGTTTGG GAAAAAAAAT TTCTCTCTCT AACTAGATCT AAACTTTCCA   
  
  
+ GCAATATAAA TACTTAAAAA TATTTTAATA ACATTTTTTT AACATATTGA TATCAAATTT AACCATTATT   
  
  
+ TTCAATAAAA TTCAACCAAC CAATTTCTGT CTCATCATTC GTGTTCCTAT GATTCTCTCT TTTTCTTTCA   
  
  
+ AACTCTCACA AGGTAAATAA AATTATATTA TGTGCTTGAA AGGAAATTTG AAAAATCTAA AGGAAAACTT   
  
  
+ AACTTAAAAG GTGCTGACAT TTTTTATTAA AAAAAGCTCA TATTGTTTTT TCTTTAATTT TGACTTTATT   
  
  
+ CTTTTCTTTT TCAAATAATA AAATTAGATT CGTTTCCATA AAATTTAATG AACTTTTAGG CAATAATGTT   
  
  
+ GTACGAAGTC AATAATGACG TGCTACAATC GTATGTCACG ACAAAAAGAC GACATGAACA CCATCCATTG   
  
  
+ GTATTCATGT GCCTTTTCAC CTTGTAAAAT AACAGGAGAA AGATACCTTC CAGGGGCAAA TTCATCTATG   
  
  
+ CCACGTGCCT ACAAGTTGAA GGTATGTTTG GTATGCAAG  

- ACATATTATT TTGTTCACGT ATATATAGTA GTACTGTGTT TTTATCTTTA TATCCGGTAT TATAATTTAA   
  
  
- TCCGTAGATA ACGTGGTCGG TACTGTCTGT AAACCAATTA TTATATTATG AGTAACTATT TCCACAGTTA   
  
  
- CTGTGTTTAG ATACTAGGAT CACAACCTTT TAAATTTAAT AAATTTATTG TACCAATCAA CTATATAATA   
  
  
- AAAAGAATAT TAATCCATCT AACTCCCCCC TTTACTATAA AAATATTCAT TATTTATAAC TATTTTGTTC   
  
  
- ATCATCTGTG GACACAGCGT GTACAGATGT GCAACCACAA CCACACGCTG CAAATAATTA CGAATTTACC   
  
  
- ACCAAAGATC CTGATCATAC GCCCAAACCT GTCGTGCACG CTGCAAATTA AAGTAGGAGT AATTTAATCA   
  
  
- CAAATAAAAA AATAAAAAAT AAAAAATAAA AAAATAAAAA AATTAACTTA AATAAAAAAT TAAAATAGTG   
  
  
- AGTAATAAAT TAAATTAAAA TAAACATACA ATTTAAATCA AAAATAAGAA AAATAAAGAT AAACAAAATG   
  
  
- AAATCTTTTT AAAAACTAAT TTTAAAAAAA AAAGTTATAT TAGGAAATCT AAATCAAAAA AAAAGAAAAA   
  
  
- TTAAAATCGA GAGTTGTAAA CTTAACATTC CTACACCAAA CTGAATTTTT TTAAAGTTCT ATTGTACAAA   
  
  
- AATTTATAAC CTTACTGTTG TATAATCTAG TTAGGTTCAG CTGAGTGATC ATTTTTTTTA TTTGAATAAT   
  
  
- TTAGGTGTAA CCAGTACCTA GGTTCATCTA AATTATTAAA AAAATTTGGT CAAAAATAAA TTAATGTACT   
  
  
- ATTTTTTATT CGCTATCATC AATTTGTTGG TTTAATTTAA ATGTTTAACT AATGTTACCT AACATTTTTT   
  
  
- GTGAACGATT ATAATGTTGT TTATAAAGAA AACAAAATAA GTTCTTGTTT CACTTTTACT TTACTTTAAA   
  
  
- CTATTTTGTT ATATTTTTTA TATAGTTAAT TGAATTATAA AAACTTTTGT TATATATTAT ATAAAATATA   
  
  
- AGTAATTAAA AATTCTTTAA TTAAAATTAA TAAACTTCTT TTTATTTTTT TTATCTGGTT CGCGCTCCCG   
  
  
- AACGGTTGGG TCCGGTTGTG AACCCAAAAA ACAACGAAAA ATTTGTTCTG TCTACTGTAC AGCAGATGTC   
  
  
- TCCTCTCACT ATACAGTAGA CATCTCAATC TGTTGTACAG TAGACGTTTG GATAAAAAAT TGTAGATCTC   
  
  
- TCATGACCTT TTAGATCGAC CCATAAACAA AAACAGGTTT TTGAATTAAA ATTAAATAAA AATAGATTTC   
  
  
- TGTGGATCTC TTTTGGTAAT ACTTGAGTTT TTTTTGATTA AGTAGTTGAA CTTTTTTTTT TTGTTTATGT   
  
  
- TTTACCTAAT TTTTGTATTT TGATCAAACC CTTTTTTTTA AAGAGAGAGA TTGATCTAGA TTTGAAAGGT   
  
  
- CGTTATATTT ATGAATTTTT ATAAAATTAT TGTAAAAAAA TTGTATAACT ATAGTTTAAA TTGGTAATAA   
  
  
- AAGTTATTTT AAGTTGGTTG GTTAAAGACA GAGTAGTAAG CACAAGGATA CTAAGAGAGA AAAAGAAAGT   
  
  
- TTGAGAGTGT TCCATTTATT TTAATATAAT ACACGAACTT TCCTTTAAAC TTTTTAGATT TCCTTTTGAA   
  
  
- TTGAATTTTC CACGACTGTA AAAAATAATT TTTTTCGAGT ATAACAAAAA AGAAATTAAA ACTGAAATAA   
  
  
- GAAAAGAAAA AGTTTATTAT TTTAATCTAA GCAAAGGTAT TTTAAATTAC TTGAAAATCC GTTATTACAA   
  
  
- CATGCTTCAG TTATTACTGC ACGATGTTAG CATACAGTGC TGTTTTTCTG CTGTACTTGT GGTAGGTAAC   
  
  
- CATAAGTACA CGGAAAAGTG GAACATTTTA TTGTCCTCTT TCTATGGAAG GTCCCCGTTT AAGTAGATAC   
  
  
- GGTGCACGGA TGTTCAACTT CCATACAAAC CATACGTTC

+     CGTCA-motif

| Site Name | Organism | Position | Strand | Matrix score. | sequence | function |
| --- | --- | --- | --- | --- | --- | --- |
| CGTCA-motif | Hordeum vulgare | 1836 | - | 5 | CGTCA | cis-acting regulatory element involved in the MeJA-responsiveness |

>Potri.011G061700.1   
+ TGTATAATAA AACAAGTGCA TATATATCAT CATGACACAA AAATAGAAAT ATAGGCCATA ATATTAAATT   
  
  
+ AGGCATCTAT TGCACCAGCC ATGACAGACA TTTGGTTAAT AATATAATAC TCATTGATAA AGGTGTCAAT   
  
  
+ GACACAAATC TATGATCCTA GTGTTGGAAA ATTTAAATTA TTTAAATAAC ATGGTTAGTT GATATATTAT   
  
  
+ TTTTCTTATA ATTAGGTAGA TTGAGGGGGG AAATGATATT TTTATAAGTA ATAAATATTG ATAAAACAAG   
  
  
+ TAGTAGACAC CTGTGTCGCA CATGTCTACA CGTTGGTGTT GGTGTGCGAC GTTTATTAAT GCTTAAATGG   
  
  
+ TGGTTTCTAG GACTAGTATG CGGGTTTGGA CAGCACGTGC GACGTTTAAT TTCATCCTCA TTAAATTAGT   
  
  
+ GTTTATTTTT TTATTTTTTA TTTTTTATTT TTTTATTTTT TTAATTGAAT TTATTTTTTA ATTTTATCAC   
  
  
+ TCATTATTTA ATTTAATTTT ATTTGTATGT TAAATTTAGT TTTTATTCTT TTTATTTCTA TTTGTTTTAC   
  
  
+ TTTAGAAAAA TTTTTGATTA AAATTTTTTT TTTCAATATA ATCCTTTAGA TTTAGTTTTT TTTTCTTTTT   
  
  
+ AATTTTAGCT CTCAACATTT GAATTGTAAG GATGTGGTTT GACTTAAAAA AATTTCAAGA TAACATGTTT   
  
  
+ TTAAATATTG GAATGACAAC ATATTAGATC AATCCAAGTC GACTCACTAG TAAAAAAAAT AAACTTATTA   
  
  
+ AATCCACATT GGTCATGGAT CCAAGTAGAT TTAATAATTT TTTTAAACCA GTTTTTATTT AATTACATGA   
  
  
+ TAAAAAATAA GCGATAGTAG TTAAACAACC AAATTAAATT TACAAATTGA TTACAATGGA TTGTAAAAAA   
  
  
+ CACTTGCTAA TATTACAACA AATATTTCTT TTGTTTTATT CAAGAACAAA GTGAAAATGA AATGAAATTT   
  
  
+ GATAAAACAA TATAAAAAAT ATATCAATTA ACTTAATATT TTTGAAAACA ATATATAATA TATTTTATAT   
  
  
+ TCATTAATTT TTAAGAAATT AATTTTAATT ATTTGAAGAA AAATAAAAAA AATAGACCAA GCGCGAGGGC   
  
  
+ TTGCCAACCC AGGCCAACAC TTGGGTTTTT TGTTGCTTTT TAAACAAGAC AGATGACATG TCGTCTACAG   
  
  
+ AGGAGAGTGA TATGTCATCT GTAGAGTTAG ACAACATGTC ATCTGCAAAC CTATTTTTTA ACATCTAGAG   
  
  
+ AGTACTGGAA AATCTAGCTG GGTATTTGTT TTTGTCCAAA AACTTAATTT TAATTTATTT TTATCTAAAG   
  
  
+ ACACCTAGAG AAAACCATTA TGAACTCAAA AAAAACTAAT TCATCAACTT GAAAAAAAAA AACAAATACA   
  
  
+ AAATGGATTA AAAACATAAA ACTAGTTTGG GAAAAAAAAT TTCTCTCTCT AACTAGATCT AAACTTTCCA   
  
  
+ GCAATATAAA TACTTAAAAA TATTTTAATA ACATTTTTTT AACATATTGA TATCAAATTT AACCATTATT   
  
  
+ TTCAATAAAA TTCAACCAAC CAATTTCTGT CTCATCATTC GTGTTCCTAT GATTCTCTCT TTTTCTTTCA   
  
  
+ AACTCTCACA AGGTAAATAA AATTATATTA TGTGCTTGAA AGGAAATTTG AAAAATCTAA AGGAAAACTT   
  
  
+ AACTTAAAAG GTGCTGACAT TTTTTATTAA AAAAAGCTCA TATTGTTTTT TCTTTAATTT TGACTTTATT   
  
  
+ CTTTTCTTTT TCAAATAATA AAATTAGATT CGTTTCCATA AAATTTAATG AACTTTTAGG CAATAATGTT   
  
  
+ GTACGAAGTC AATAATGACG TGCTACAATC GTATGTCACG ACAAAAAGAC GACATGAACA CCATCCATTG   
  
  
+ GTATTCATGT GCCTTTTCAC CTTGTAAAAT AACAGGAGAA AGATACCTTC CAGGGGCAAA TTCATCTATG   
  
  
+ CCACGTGCCT ACAAGTTGAA GGTATGTTTG GTATGCAAG  

- ACATATTATT TTGTTCACGT ATATATAGTA GTACTGTGTT TTTATCTTTA TATCCGGTAT TATAATTTAA   
  
  
- TCCGTAGATA ACGTGGTCGG TACTGTCTGT AAACCAATTA TTATATTATG AGTAACTATT TCCACAGTTA   
  
  
- CTGTGTTTAG ATACTAGGAT CACAACCTTT TAAATTTAAT AAATTTATTG TACCAATCAA CTATATAATA   
  
  
- AAAAGAATAT TAATCCATCT AACTCCCCCC TTTACTATAA AAATATTCAT TATTTATAAC TATTTTGTTC   
  
  
- ATCATCTGTG GACACAGCGT GTACAGATGT GCAACCACAA CCACACGCTG CAAATAATTA CGAATTTACC   
  
  
- ACCAAAGATC CTGATCATAC GCCCAAACCT GTCGTGCACG CTGCAAATTA AAGTAGGAGT AATTTAATCA   
  
  
- CAAATAAAAA AATAAAAAAT AAAAAATAAA AAAATAAAAA AATTAACTTA AATAAAAAAT TAAAATAGTG   
  
  
- AGTAATAAAT TAAATTAAAA TAAACATACA ATTTAAATCA AAAATAAGAA AAATAAAGAT AAACAAAATG   
  
  
- AAATCTTTTT AAAAACTAAT TTTAAAAAAA AAAGTTATAT TAGGAAATCT AAATCAAAAA AAAAGAAAAA   
  
  
- TTAAAATCGA GAGTTGTAAA CTTAACATTC CTACACCAAA CTGAATTTTT TTAAAGTTCT ATTGTACAAA   
  
  
- AATTTATAAC CTTACTGTTG TATAATCTAG TTAGGTTCAG CTGAGTGATC ATTTTTTTTA TTTGAATAAT   
  
  
- TTAGGTGTAA CCAGTACCTA GGTTCATCTA AATTATTAAA AAAATTTGGT CAAAAATAAA TTAATGTACT   
  
  
- ATTTTTTATT CGCTATCATC AATTTGTTGG TTTAATTTAA ATGTTTAACT AATGTTACCT AACATTTTTT   
  
  
- GTGAACGATT ATAATGTTGT TTATAAAGAA AACAAAATAA GTTCTTGTTT CACTTTTACT TTACTTTAAA   
  
  
- CTATTTTGTT ATATTTTTTA TATAGTTAAT TGAATTATAA AAACTTTTGT TATATATTAT ATAAAATATA   
  
  
- AGTAATTAAA AATTCTTTAA TTAAAATTAA TAAACTTCTT TTTATTTTTT TTATCTGGTT CGCGCTCCCG   
  
  
- AACGGTTGGG TCCGGTTGTG AACCCAAAAA ACAACGAAAA ATTTGTTCTG TCTACTGTAC AGCAGATGTC   
  
  
- TCCTCTCACT ATACAGTAGA CATCTCAATC TGTTGTACAG TAGACGTTTG GATAAAAAAT TGTAGATCTC   
  
  
- TCATGACCTT TTAGATCGAC CCATAAACAA AAACAGGTTT TTGAATTAAA ATTAAATAAA AATAGATTTC   
  
  
- TGTGGATCTC TTTTGGTAAT ACTTGAGTTT TTTTTGATTA AGTAGTTGAA CTTTTTTTTT TTGTTTATGT   
  
  
- TTTACCTAAT TTTTGTATTT TGATCAAACC CTTTTTTTTA AAGAGAGAGA TTGATCTAGA TTTGAAAGGT   
  
  
- CGTTATATTT ATGAATTTTT ATAAAATTAT TGTAAAAAAA TTGTATAACT ATAGTTTAAA TTGGTAATAA   
  
  
- AAGTTATTTT AAGTTGGTTG GTTAAAGACA GAGTAGTAAG CACAAGGATA CTAAGAGAGA AAAAGAAAGT   
  
  
- TTGAGAGTGT TCCATTTATT TTAATATAAT ACACGAACTT TCCTTTAAAC TTTTTAGATT TCCTTTTGAA   
  
  
- TTGAATTTTC CACGACTGTA AAAAATAATT TTTTTCGAGT ATAACAAAAA AGAAATTAAA ACTGAAATAA   
  
  
- GAAAAGAAAA AGTTTATTAT TTTAATCTAA GCAAAGGTAT TTTAAATTAC TTGAAAATCC GTTATTACAA   
  
  
- CATGCTTCAG TTATTACTGC ACGATGTTAG CATACAGTGC TGTTTTTCTG CTGTACTTGT GGTAGGTAAC   
  
  
- CATAAGTACA CGGAAAAGTG GAACATTTTA TTGTCCTCTT TCTATGGAAG GTCCCCGTTT AAGTAGATAC   
  
  
- GGTGCACGGA TGTTCAACTT CCATACAAAC CATACGTTC

+     G-Box

| Site Name | Organism | Position | Strand | Matrix score. | sequence | function |
| --- | --- | --- | --- | --- | --- | --- |
| G-Box | Pisum sativum | 1962 | - | 6 | CACGTG | cis-acting regulatory element involved in light responsiveness |
| G-Box | Pisum sativum | 309 | + | 6 | CACGTT | cis-acting regulatory element involved in light responsiveness |
| G-Box | Pisum sativum | 384 | + | 6 | CACGTG | cis-acting regulatory element involved in light responsiveness |

>Potri.011G061700.1   
+ TGTATAATAA AACAAGTGCA TATATATCAT CATGACACAA AAATAGAAAT ATAGGCCATA ATATTAAATT   
  
  
+ AGGCATCTAT TGCACCAGCC ATGACAGACA TTTGGTTAAT AATATAATAC TCATTGATAA AGGTGTCAAT   
  
  
+ GACACAAATC TATGATCCTA GTGTTGGAAA ATTTAAATTA TTTAAATAAC ATGGTTAGTT GATATATTAT   
  
  
+ TTTTCTTATA ATTAGGTAGA TTGAGGGGGG AAATGATATT TTTATAAGTA ATAAATATTG ATAAAACAAG   
  
  
+ TAGTAGACAC CTGTGTCGCA CATGTCTACA CGTTGGTGTT GGTGTGCGAC GTTTATTAAT GCTTAAATGG   
  
  
+ TGGTTTCTAG GACTAGTATG CGGGTTTGGA CAGCACGTGC GACGTTTAAT TTCATCCTCA TTAAATTAGT   
  
  
+ GTTTATTTTT TTATTTTTTA TTTTTTATTT TTTTATTTTT TTAATTGAAT TTATTTTTTA ATTTTATCAC   
  
  
+ TCATTATTTA ATTTAATTTT ATTTGTATGT TAAATTTAGT TTTTATTCTT TTTATTTCTA TTTGTTTTAC   
  
  
+ TTTAGAAAAA TTTTTGATTA AAATTTTTTT TTTCAATATA ATCCTTTAGA TTTAGTTTTT TTTTCTTTTT   
  
  
+ AATTTTAGCT CTCAACATTT GAATTGTAAG GATGTGGTTT GACTTAAAAA AATTTCAAGA TAACATGTTT   
  
  
+ TTAAATATTG GAATGACAAC ATATTAGATC AATCCAAGTC GACTCACTAG TAAAAAAAAT AAACTTATTA   
  
  
+ AATCCACATT GGTCATGGAT CCAAGTAGAT TTAATAATTT TTTTAAACCA GTTTTTATTT AATTACATGA   
  
  
+ TAAAAAATAA GCGATAGTAG TTAAACAACC AAATTAAATT TACAAATTGA TTACAATGGA TTGTAAAAAA   
  
  
+ CACTTGCTAA TATTACAACA AATATTTCTT TTGTTTTATT CAAGAACAAA GTGAAAATGA AATGAAATTT   
  
  
+ GATAAAACAA TATAAAAAAT ATATCAATTA ACTTAATATT TTTGAAAACA ATATATAATA TATTTTATAT   
  
  
+ TCATTAATTT TTAAGAAATT AATTTTAATT ATTTGAAGAA AAATAAAAAA AATAGACCAA GCGCGAGGGC   
  
  
+ TTGCCAACCC AGGCCAACAC TTGGGTTTTT TGTTGCTTTT TAAACAAGAC AGATGACATG TCGTCTACAG   
  
  
+ AGGAGAGTGA TATGTCATCT GTAGAGTTAG ACAACATGTC ATCTGCAAAC CTATTTTTTA ACATCTAGAG   
  
  
+ AGTACTGGAA AATCTAGCTG GGTATTTGTT TTTGTCCAAA AACTTAATTT TAATTTATTT TTATCTAAAG   
  
  
+ ACACCTAGAG AAAACCATTA TGAACTCAAA AAAAACTAAT TCATCAACTT GAAAAAAAAA AACAAATACA   
  
  
+ AAATGGATTA AAAACATAAA ACTAGTTTGG GAAAAAAAAT TTCTCTCTCT AACTAGATCT AAACTTTCCA   
  
  
+ GCAATATAAA TACTTAAAAA TATTTTAATA ACATTTTTTT AACATATTGA TATCAAATTT AACCATTATT   
  
  
+ TTCAATAAAA TTCAACCAAC CAATTTCTGT CTCATCATTC GTGTTCCTAT GATTCTCTCT TTTTCTTTCA   
  
  
+ AACTCTCACA AGGTAAATAA AATTATATTA TGTGCTTGAA AGGAAATTTG AAAAATCTAA AGGAAAACTT   
  
  
+ AACTTAAAAG GTGCTGACAT TTTTTATTAA AAAAAGCTCA TATTGTTTTT TCTTTAATTT TGACTTTATT   
  
  
+ CTTTTCTTTT TCAAATAATA AAATTAGATT CGTTTCCATA AAATTTAATG AACTTTTAGG CAATAATGTT   
  
  
+ GTACGAAGTC AATAATGACG TGCTACAATC GTATGTCACG ACAAAAAGAC GACATGAACA CCATCCATTG   
  
  
+ GTATTCATGT GCCTTTTCAC CTTGTAAAAT AACAGGAGAA AGATACCTTC CAGGGGCAAA TTCATCTATG   
  
  
+ CCACGTGCCT ACAAGTTGAA GGTATGTTTG GTATGCAAG  

- ACATATTATT TTGTTCACGT ATATATAGTA GTACTGTGTT TTTATCTTTA TATCCGGTAT TATAATTTAA   
  
  
- TCCGTAGATA ACGTGGTCGG TACTGTCTGT AAACCAATTA TTATATTATG AGTAACTATT TCCACAGTTA   
  
  
- CTGTGTTTAG ATACTAGGAT CACAACCTTT TAAATTTAAT AAATTTATTG TACCAATCAA CTATATAATA   
  
  
- AAAAGAATAT TAATCCATCT AACTCCCCCC TTTACTATAA AAATATTCAT TATTTATAAC TATTTTGTTC   
  
  
- ATCATCTGTG GACACAGCGT GTACAGATGT GCAACCACAA CCACACGCTG CAAATAATTA CGAATTTACC   
  
  
- ACCAAAGATC CTGATCATAC GCCCAAACCT GTCGTGCACG CTGCAAATTA AAGTAGGAGT AATTTAATCA   
  
  
- CAAATAAAAA AATAAAAAAT AAAAAATAAA AAAATAAAAA AATTAACTTA AATAAAAAAT TAAAATAGTG   
  
  
- AGTAATAAAT TAAATTAAAA TAAACATACA ATTTAAATCA AAAATAAGAA AAATAAAGAT AAACAAAATG   
  
  
- AAATCTTTTT AAAAACTAAT TTTAAAAAAA AAAGTTATAT TAGGAAATCT AAATCAAAAA AAAAGAAAAA   
  
  
- TTAAAATCGA GAGTTGTAAA CTTAACATTC CTACACCAAA CTGAATTTTT TTAAAGTTCT ATTGTACAAA   
  
  
- AATTTATAAC CTTACTGTTG TATAATCTAG TTAGGTTCAG CTGAGTGATC ATTTTTTTTA TTTGAATAAT   
  
  
- TTAGGTGTAA CCAGTACCTA GGTTCATCTA AATTATTAAA AAAATTTGGT CAAAAATAAA TTAATGTACT   
  
  
- ATTTTTTATT CGCTATCATC AATTTGTTGG TTTAATTTAA ATGTTTAACT AATGTTACCT AACATTTTTT   
  
  
- GTGAACGATT ATAATGTTGT TTATAAAGAA AACAAAATAA GTTCTTGTTT CACTTTTACT TTACTTTAAA   
  
  
- CTATTTTGTT ATATTTTTTA TATAGTTAAT TGAATTATAA AAACTTTTGT TATATATTAT ATAAAATATA   
  
  
- AGTAATTAAA AATTCTTTAA TTAAAATTAA TAAACTTCTT TTTATTTTTT TTATCTGGTT CGCGCTCCCG   
  
  
- AACGGTTGGG TCCGGTTGTG AACCCAAAAA ACAACGAAAA ATTTGTTCTG TCTACTGTAC AGCAGATGTC   
  
  
- TCCTCTCACT ATACAGTAGA CATCTCAATC TGTTGTACAG TAGACGTTTG GATAAAAAAT TGTAGATCTC   
  
  
- TCATGACCTT TTAGATCGAC CCATAAACAA AAACAGGTTT TTGAATTAAA ATTAAATAAA AATAGATTTC   
  
  
- TGTGGATCTC TTTTGGTAAT ACTTGAGTTT TTTTTGATTA AGTAGTTGAA CTTTTTTTTT TTGTTTATGT   
  
  
- TTTACCTAAT TTTTGTATTT TGATCAAACC CTTTTTTTTA AAGAGAGAGA TTGATCTAGA TTTGAAAGGT   
  
  
- CGTTATATTT ATGAATTTTT ATAAAATTAT TGTAAAAAAA TTGTATAACT ATAGTTTAAA TTGGTAATAA   
  
  
- AAGTTATTTT AAGTTGGTTG GTTAAAGACA GAGTAGTAAG CACAAGGATA CTAAGAGAGA AAAAGAAAGT   
  
  
- TTGAGAGTGT TCCATTTATT TTAATATAAT ACACGAACTT TCCTTTAAAC TTTTTAGATT TCCTTTTGAA   
  
  
- TTGAATTTTC CACGACTGTA AAAAATAATT TTTTTCGAGT ATAACAAAAA AGAAATTAAA ACTGAAATAA   
  
  
- GAAAAGAAAA AGTTTATTAT TTTAATCTAA GCAAAGGTAT TTTAAATTAC TTGAAAATCC GTTATTACAA   
  
  
- CATGCTTCAG TTATTACTGC ACGATGTTAG CATACAGTGC TGTTTTTCTG CTGTACTTGT GGTAGGTAAC   
  
  
- CATAAGTACA CGGAAAAGTG GAACATTTTA TTGTCCTCTT TCTATGGAAG GTCCCCGTTT AAGTAGATAC   
  
  
- GGTGCACGGA TGTTCAACTT CCATACAAAC CATACGTTC

+     G-box

| Site Name | Organism | Position | Strand | Matrix score. | sequence | function |
| --- | --- | --- | --- | --- | --- | --- |
| G-box | Zea mays | 1857 | + | 6 | CACGAC | cis-acting regulatory element involved in light responsiveness |
| G-box | Zea mays | 1837 | - | 6 | CACGTC | cis-acting regulatory element involved in light responsiveness |
| G-box | Arabidopsis thaliana | 1962 | - | 6 | CACGTG | cis-acting regulatory element involved in light responsiveness |
| G-box | Arabidopsis thaliana | 384 | + | 6 | CACGTG | cis-acting regulatory element involved in light responsiveness |

>Potri.011G061700.1   
+ TGTATAATAA AACAAGTGCA TATATATCAT CATGACACAA AAATAGAAAT ATAGGCCATA ATATTAAATT   
  
  
+ AGGCATCTAT TGCACCAGCC ATGACAGACA TTTGGTTAAT AATATAATAC TCATTGATAA AGGTGTCAAT   
  
  
+ GACACAAATC TATGATCCTA GTGTTGGAAA ATTTAAATTA TTTAAATAAC ATGGTTAGTT GATATATTAT   
  
  
+ TTTTCTTATA ATTAGGTAGA TTGAGGGGGG AAATGATATT TTTATAAGTA ATAAATATTG ATAAAACAAG   
  
  
+ TAGTAGACAC CTGTGTCGCA CATGTCTACA CGTTGGTGTT GGTGTGCGAC GTTTATTAAT GCTTAAATGG   
  
  
+ TGGTTTCTAG GACTAGTATG CGGGTTTGGA CAGCACGTGC GACGTTTAAT TTCATCCTCA TTAAATTAGT   
  
  
+ GTTTATTTTT TTATTTTTTA TTTTTTATTT TTTTATTTTT TTAATTGAAT TTATTTTTTA ATTTTATCAC   
  
  
+ TCATTATTTA ATTTAATTTT ATTTGTATGT TAAATTTAGT TTTTATTCTT TTTATTTCTA TTTGTTTTAC   
  
  
+ TTTAGAAAAA TTTTTGATTA AAATTTTTTT TTTCAATATA ATCCTTTAGA TTTAGTTTTT TTTTCTTTTT   
  
  
+ AATTTTAGCT CTCAACATTT GAATTGTAAG GATGTGGTTT GACTTAAAAA AATTTCAAGA TAACATGTTT   
  
  
+ TTAAATATTG GAATGACAAC ATATTAGATC AATCCAAGTC GACTCACTAG TAAAAAAAAT AAACTTATTA   
  
  
+ AATCCACATT GGTCATGGAT CCAAGTAGAT TTAATAATTT TTTTAAACCA GTTTTTATTT AATTACATGA   
  
  
+ TAAAAAATAA GCGATAGTAG TTAAACAACC AAATTAAATT TACAAATTGA TTACAATGGA TTGTAAAAAA   
  
  
+ CACTTGCTAA TATTACAACA AATATTTCTT TTGTTTTATT CAAGAACAAA GTGAAAATGA AATGAAATTT   
  
  
+ GATAAAACAA TATAAAAAAT ATATCAATTA ACTTAATATT TTTGAAAACA ATATATAATA TATTTTATAT   
  
  
+ TCATTAATTT TTAAGAAATT AATTTTAATT ATTTGAAGAA AAATAAAAAA AATAGACCAA GCGCGAGGGC   
  
  
+ TTGCCAACCC AGGCCAACAC TTGGGTTTTT TGTTGCTTTT TAAACAAGAC AGATGACATG TCGTCTACAG   
  
  
+ AGGAGAGTGA TATGTCATCT GTAGAGTTAG ACAACATGTC ATCTGCAAAC CTATTTTTTA ACATCTAGAG   
  
  
+ AGTACTGGAA AATCTAGCTG GGTATTTGTT TTTGTCCAAA AACTTAATTT TAATTTATTT TTATCTAAAG   
  
  
+ ACACCTAGAG AAAACCATTA TGAACTCAAA AAAAACTAAT TCATCAACTT GAAAAAAAAA AACAAATACA   
  
  
+ AAATGGATTA AAAACATAAA ACTAGTTTGG GAAAAAAAAT TTCTCTCTCT AACTAGATCT AAACTTTCCA   
  
  
+ GCAATATAAA TACTTAAAAA TATTTTAATA ACATTTTTTT AACATATTGA TATCAAATTT AACCATTATT   
  
  
+ TTCAATAAAA TTCAACCAAC CAATTTCTGT CTCATCATTC GTGTTCCTAT GATTCTCTCT TTTTCTTTCA   
  
  
+ AACTCTCACA AGGTAAATAA AATTATATTA TGTGCTTGAA AGGAAATTTG AAAAATCTAA AGGAAAACTT   
  
  
+ AACTTAAAAG GTGCTGACAT TTTTTATTAA AAAAAGCTCA TATTGTTTTT TCTTTAATTT TGACTTTATT   
  
  
+ CTTTTCTTTT TCAAATAATA AAATTAGATT CGTTTCCATA AAATTTAATG AACTTTTAGG CAATAATGTT   
  
  
+ GTACGAAGTC AATAATGACG TGCTACAATC GTATGTCACG ACAAAAAGAC GACATGAACA CCATCCATTG   
  
  
+ GTATTCATGT GCCTTTTCAC CTTGTAAAAT AACAGGAGAA AGATACCTTC CAGGGGCAAA TTCATCTATG   
  
  
+ CCACGTGCCT ACAAGTTGAA GGTATGTTTG GTATGCAAG  

- ACATATTATT TTGTTCACGT ATATATAGTA GTACTGTGTT TTTATCTTTA TATCCGGTAT TATAATTTAA   
  
  
- TCCGTAGATA ACGTGGTCGG TACTGTCTGT AAACCAATTA TTATATTATG AGTAACTATT TCCACAGTTA   
  
  
- CTGTGTTTAG ATACTAGGAT CACAACCTTT TAAATTTAAT AAATTTATTG TACCAATCAA CTATATAATA   
  
  
- AAAAGAATAT TAATCCATCT AACTCCCCCC TTTACTATAA AAATATTCAT TATTTATAAC TATTTTGTTC   
  
  
- ATCATCTGTG GACACAGCGT GTACAGATGT GCAACCACAA CCACACGCTG CAAATAATTA CGAATTTACC   
  
  
- ACCAAAGATC CTGATCATAC GCCCAAACCT GTCGTGCACG CTGCAAATTA AAGTAGGAGT AATTTAATCA   
  
  
- CAAATAAAAA AATAAAAAAT AAAAAATAAA AAAATAAAAA AATTAACTTA AATAAAAAAT TAAAATAGTG   
  
  
- AGTAATAAAT TAAATTAAAA TAAACATACA ATTTAAATCA AAAATAAGAA AAATAAAGAT AAACAAAATG   
  
  
- AAATCTTTTT AAAAACTAAT TTTAAAAAAA AAAGTTATAT TAGGAAATCT AAATCAAAAA AAAAGAAAAA   
  
  
- TTAAAATCGA GAGTTGTAAA CTTAACATTC CTACACCAAA CTGAATTTTT TTAAAGTTCT ATTGTACAAA   
  
  
- AATTTATAAC CTTACTGTTG TATAATCTAG TTAGGTTCAG CTGAGTGATC ATTTTTTTTA TTTGAATAAT   
  
  
- TTAGGTGTAA CCAGTACCTA GGTTCATCTA AATTATTAAA AAAATTTGGT CAAAAATAAA TTAATGTACT   
  
  
- ATTTTTTATT CGCTATCATC AATTTGTTGG TTTAATTTAA ATGTTTAACT AATGTTACCT AACATTTTTT   
  
  
- GTGAACGATT ATAATGTTGT TTATAAAGAA AACAAAATAA GTTCTTGTTT CACTTTTACT TTACTTTAAA   
  
  
- CTATTTTGTT ATATTTTTTA TATAGTTAAT TGAATTATAA AAACTTTTGT TATATATTAT ATAAAATATA   
  
  
- AGTAATTAAA AATTCTTTAA TTAAAATTAA TAAACTTCTT TTTATTTTTT TTATCTGGTT CGCGCTCCCG   
  
  
- AACGGTTGGG TCCGGTTGTG AACCCAAAAA ACAACGAAAA ATTTGTTCTG TCTACTGTAC AGCAGATGTC   
  
  
- TCCTCTCACT ATACAGTAGA CATCTCAATC TGTTGTACAG TAGACGTTTG GATAAAAAAT TGTAGATCTC   
  
  
- TCATGACCTT TTAGATCGAC CCATAAACAA AAACAGGTTT TTGAATTAAA ATTAAATAAA AATAGATTTC   
  
  
- TGTGGATCTC TTTTGGTAAT ACTTGAGTTT TTTTTGATTA AGTAGTTGAA CTTTTTTTTT TTGTTTATGT   
  
  
- TTTACCTAAT TTTTGTATTT TGATCAAACC CTTTTTTTTA AAGAGAGAGA TTGATCTAGA TTTGAAAGGT   
  
  
- CGTTATATTT ATGAATTTTT ATAAAATTAT TGTAAAAAAA TTGTATAACT ATAGTTTAAA TTGGTAATAA   
  
  
- AAGTTATTTT AAGTTGGTTG GTTAAAGACA GAGTAGTAAG CACAAGGATA CTAAGAGAGA AAAAGAAAGT   
  
  
- TTGAGAGTGT TCCATTTATT TTAATATAAT ACACGAACTT TCCTTTAAAC TTTTTAGATT TCCTTTTGAA   
  
  
- TTGAATTTTC CACGACTGTA AAAAATAATT TTTTTCGAGT ATAACAAAAA AGAAATTAAA ACTGAAATAA   
  
  
- GAAAAGAAAA AGTTTATTAT TTTAATCTAA GCAAAGGTAT TTTAAATTAC TTGAAAATCC GTTATTACAA   
  
  
- CATGCTTCAG TTATTACTGC ACGATGTTAG CATACAGTGC TGTTTTTCTG CTGTACTTGT GGTAGGTAAC   
  
  
- CATAAGTACA CGGAAAAGTG GAACATTTTA TTGTCCTCTT TCTATGGAAG GTCCCCGTTT AAGTAGATAC   
  
  
- GGTGCACGGA TGTTCAACTT CCATACAAAC CATACGTTC

+     GT1-motif

| Site Name | Organism | Position | Strand | Matrix score. | sequence | function |
| --- | --- | --- | --- | --- | --- | --- |
| GT1-motif | Arabidopsis thaliana | 1529 | - | 6 | GGTTAA | light responsive element |
| GT1-motif | Avena sativa | 104 | + | 7 | GGTTAAT | light responsive element |

>Potri.011G061700.1   
+ TGTATAATAA AACAAGTGCA TATATATCAT CATGACACAA AAATAGAAAT ATAGGCCATA ATATTAAATT   
  
  
+ AGGCATCTAT TGCACCAGCC ATGACAGACA TTTGGTTAAT AATATAATAC TCATTGATAA AGGTGTCAAT   
  
  
+ GACACAAATC TATGATCCTA GTGTTGGAAA ATTTAAATTA TTTAAATAAC ATGGTTAGTT GATATATTAT   
  
  
+ TTTTCTTATA ATTAGGTAGA TTGAGGGGGG AAATGATATT TTTATAAGTA ATAAATATTG ATAAAACAAG   
  
  
+ TAGTAGACAC CTGTGTCGCA CATGTCTACA CGTTGGTGTT GGTGTGCGAC GTTTATTAAT GCTTAAATGG   
  
  
+ TGGTTTCTAG GACTAGTATG CGGGTTTGGA CAGCACGTGC GACGTTTAAT TTCATCCTCA TTAAATTAGT   
  
  
+ GTTTATTTTT TTATTTTTTA TTTTTTATTT TTTTATTTTT TTAATTGAAT TTATTTTTTA ATTTTATCAC   
  
  
+ TCATTATTTA ATTTAATTTT ATTTGTATGT TAAATTTAGT TTTTATTCTT TTTATTTCTA TTTGTTTTAC   
  
  
+ TTTAGAAAAA TTTTTGATTA AAATTTTTTT TTTCAATATA ATCCTTTAGA TTTAGTTTTT TTTTCTTTTT   
  
  
+ AATTTTAGCT CTCAACATTT GAATTGTAAG GATGTGGTTT GACTTAAAAA AATTTCAAGA TAACATGTTT   
  
  
+ TTAAATATTG GAATGACAAC ATATTAGATC AATCCAAGTC GACTCACTAG TAAAAAAAAT AAACTTATTA   
  
  
+ AATCCACATT GGTCATGGAT CCAAGTAGAT TTAATAATTT TTTTAAACCA GTTTTTATTT AATTACATGA   
  
  
+ TAAAAAATAA GCGATAGTAG TTAAACAACC AAATTAAATT TACAAATTGA TTACAATGGA TTGTAAAAAA   
  
  
+ CACTTGCTAA TATTACAACA AATATTTCTT TTGTTTTATT CAAGAACAAA GTGAAAATGA AATGAAATTT   
  
  
+ GATAAAACAA TATAAAAAAT ATATCAATTA ACTTAATATT TTTGAAAACA ATATATAATA TATTTTATAT   
  
  
+ TCATTAATTT TTAAGAAATT AATTTTAATT ATTTGAAGAA AAATAAAAAA AATAGACCAA GCGCGAGGGC   
  
  
+ TTGCCAACCC AGGCCAACAC TTGGGTTTTT TGTTGCTTTT TAAACAAGAC AGATGACATG TCGTCTACAG   
  
  
+ AGGAGAGTGA TATGTCATCT GTAGAGTTAG ACAACATGTC ATCTGCAAAC CTATTTTTTA ACATCTAGAG   
  
  
+ AGTACTGGAA AATCTAGCTG GGTATTTGTT TTTGTCCAAA AACTTAATTT TAATTTATTT TTATCTAAAG   
  
  
+ ACACCTAGAG AAAACCATTA TGAACTCAAA AAAAACTAAT TCATCAACTT GAAAAAAAAA AACAAATACA   
  
  
+ AAATGGATTA AAAACATAAA ACTAGTTTGG GAAAAAAAAT TTCTCTCTCT AACTAGATCT AAACTTTCCA   
  
  
+ GCAATATAAA TACTTAAAAA TATTTTAATA ACATTTTTTT AACATATTGA TATCAAATTT AACCATTATT   
  
  
+ TTCAATAAAA TTCAACCAAC CAATTTCTGT CTCATCATTC GTGTTCCTAT GATTCTCTCT TTTTCTTTCA   
  
  
+ AACTCTCACA AGGTAAATAA AATTATATTA TGTGCTTGAA AGGAAATTTG AAAAATCTAA AGGAAAACTT   
  
  
+ AACTTAAAAG GTGCTGACAT TTTTTATTAA AAAAAGCTCA TATTGTTTTT TCTTTAATTT TGACTTTATT   
  
  
+ CTTTTCTTTT TCAAATAATA AAATTAGATT CGTTTCCATA AAATTTAATG AACTTTTAGG CAATAATGTT   
  
  
+ GTACGAAGTC AATAATGACG TGCTACAATC GTATGTCACG ACAAAAAGAC GACATGAACA CCATCCATTG   
  
  
+ GTATTCATGT GCCTTTTCAC CTTGTAAAAT AACAGGAGAA AGATACCTTC CAGGGGCAAA TTCATCTATG   
  
  
+ CCACGTGCCT ACAAGTTGAA GGTATGTTTG GTATGCAAG  

- ACATATTATT TTGTTCACGT ATATATAGTA GTACTGTGTT TTTATCTTTA TATCCGGTAT TATAATTTAA   
  
  
- TCCGTAGATA ACGTGGTCGG TACTGTCTGT AAACCAATTA TTATATTATG AGTAACTATT TCCACAGTTA   
  
  
- CTGTGTTTAG ATACTAGGAT CACAACCTTT TAAATTTAAT AAATTTATTG TACCAATCAA CTATATAATA   
  
  
- AAAAGAATAT TAATCCATCT AACTCCCCCC TTTACTATAA AAATATTCAT TATTTATAAC TATTTTGTTC   
  
  
- ATCATCTGTG GACACAGCGT GTACAGATGT GCAACCACAA CCACACGCTG CAAATAATTA CGAATTTACC   
  
  
- ACCAAAGATC CTGATCATAC GCCCAAACCT GTCGTGCACG CTGCAAATTA AAGTAGGAGT AATTTAATCA   
  
  
- CAAATAAAAA AATAAAAAAT AAAAAATAAA AAAATAAAAA AATTAACTTA AATAAAAAAT TAAAATAGTG   
  
  
- AGTAATAAAT TAAATTAAAA TAAACATACA ATTTAAATCA AAAATAAGAA AAATAAAGAT AAACAAAATG   
  
  
- AAATCTTTTT AAAAACTAAT TTTAAAAAAA AAAGTTATAT TAGGAAATCT AAATCAAAAA AAAAGAAAAA   
  
  
- TTAAAATCGA GAGTTGTAAA CTTAACATTC CTACACCAAA CTGAATTTTT TTAAAGTTCT ATTGTACAAA   
  
  
- AATTTATAAC CTTACTGTTG TATAATCTAG TTAGGTTCAG CTGAGTGATC ATTTTTTTTA TTTGAATAAT   
  
  
- TTAGGTGTAA CCAGTACCTA GGTTCATCTA AATTATTAAA AAAATTTGGT CAAAAATAAA TTAATGTACT   
  
  
- ATTTTTTATT CGCTATCATC AATTTGTTGG TTTAATTTAA ATGTTTAACT AATGTTACCT AACATTTTTT   
  
  
- GTGAACGATT ATAATGTTGT TTATAAAGAA AACAAAATAA GTTCTTGTTT CACTTTTACT TTACTTTAAA   
  
  
- CTATTTTGTT ATATTTTTTA TATAGTTAAT TGAATTATAA AAACTTTTGT TATATATTAT ATAAAATATA   
  
  
- AGTAATTAAA AATTCTTTAA TTAAAATTAA TAAACTTCTT TTTATTTTTT TTATCTGGTT CGCGCTCCCG   
  
  
- AACGGTTGGG TCCGGTTGTG AACCCAAAAA ACAACGAAAA ATTTGTTCTG TCTACTGTAC AGCAGATGTC   
  
  
- TCCTCTCACT ATACAGTAGA CATCTCAATC TGTTGTACAG TAGACGTTTG GATAAAAAAT TGTAGATCTC   
  
  
- TCATGACCTT TTAGATCGAC CCATAAACAA AAACAGGTTT TTGAATTAAA ATTAAATAAA AATAGATTTC   
  
  
- TGTGGATCTC TTTTGGTAAT ACTTGAGTTT TTTTTGATTA AGTAGTTGAA CTTTTTTTTT TTGTTTATGT   
  
  
- TTTACCTAAT TTTTGTATTT TGATCAAACC CTTTTTTTTA AAGAGAGAGA TTGATCTAGA TTTGAAAGGT   
  
  
- CGTTATATTT ATGAATTTTT ATAAAATTAT TGTAAAAAAA TTGTATAACT ATAGTTTAAA TTGGTAATAA   
  
  
- AAGTTATTTT AAGTTGGTTG GTTAAAGACA GAGTAGTAAG CACAAGGATA CTAAGAGAGA AAAAGAAAGT   
  
  
- TTGAGAGTGT TCCATTTATT TTAATATAAT ACACGAACTT TCCTTTAAAC TTTTTAGATT TCCTTTTGAA   
  
  
- TTGAATTTTC CACGACTGTA AAAAATAATT TTTTTCGAGT ATAACAAAAA AGAAATTAAA ACTGAAATAA   
  
  
- GAAAAGAAAA AGTTTATTAT TTTAATCTAA GCAAAGGTAT TTTAAATTAC TTGAAAATCC GTTATTACAA   
  
  
- CATGCTTCAG TTATTACTGC ACGATGTTAG CATACAGTGC TGTTTTTCTG CTGTACTTGT GGTAGGTAAC   
  
  
- CATAAGTACA CGGAAAAGTG GAACATTTTA TTGTCCTCTT TCTATGGAAG GTCCCCGTTT AAGTAGATAC   
  
  
- GGTGCACGGA TGTTCAACTT CCATACAAAC CATACGTTC

+     LAMP-element

| Site Name | Organism | Position | Strand | Matrix score. | sequence | function |
| --- | --- | --- | --- | --- | --- | --- |
| LAMP-element | Pisum sativum | 125 | - | 8 | CTTTATCA | part of a light responsive element |

>Potri.011G061700.1   
+ TGTATAATAA AACAAGTGCA TATATATCAT CATGACACAA AAATAGAAAT ATAGGCCATA ATATTAAATT   
  
  
+ AGGCATCTAT TGCACCAGCC ATGACAGACA TTTGGTTAAT AATATAATAC TCATTGATAA AGGTGTCAAT   
  
  
+ GACACAAATC TATGATCCTA GTGTTGGAAA ATTTAAATTA TTTAAATAAC ATGGTTAGTT GATATATTAT   
  
  
+ TTTTCTTATA ATTAGGTAGA TTGAGGGGGG AAATGATATT TTTATAAGTA ATAAATATTG ATAAAACAAG   
  
  
+ TAGTAGACAC CTGTGTCGCA CATGTCTACA CGTTGGTGTT GGTGTGCGAC GTTTATTAAT GCTTAAATGG   
  
  
+ TGGTTTCTAG GACTAGTATG CGGGTTTGGA CAGCACGTGC GACGTTTAAT TTCATCCTCA TTAAATTAGT   
  
  
+ GTTTATTTTT TTATTTTTTA TTTTTTATTT TTTTATTTTT TTAATTGAAT TTATTTTTTA ATTTTATCAC   
  
  
+ TCATTATTTA ATTTAATTTT ATTTGTATGT TAAATTTAGT TTTTATTCTT TTTATTTCTA TTTGTTTTAC   
  
  
+ TTTAGAAAAA TTTTTGATTA AAATTTTTTT TTTCAATATA ATCCTTTAGA TTTAGTTTTT TTTTCTTTTT   
  
  
+ AATTTTAGCT CTCAACATTT GAATTGTAAG GATGTGGTTT GACTTAAAAA AATTTCAAGA TAACATGTTT   
  
  
+ TTAAATATTG GAATGACAAC ATATTAGATC AATCCAAGTC GACTCACTAG TAAAAAAAAT AAACTTATTA   
  
  
+ AATCCACATT GGTCATGGAT CCAAGTAGAT TTAATAATTT TTTTAAACCA GTTTTTATTT AATTACATGA   
  
  
+ TAAAAAATAA GCGATAGTAG TTAAACAACC AAATTAAATT TACAAATTGA TTACAATGGA TTGTAAAAAA   
  
  
+ CACTTGCTAA TATTACAACA AATATTTCTT TTGTTTTATT CAAGAACAAA GTGAAAATGA AATGAAATTT   
  
  
+ GATAAAACAA TATAAAAAAT ATATCAATTA ACTTAATATT TTTGAAAACA ATATATAATA TATTTTATAT   
  
  
+ TCATTAATTT TTAAGAAATT AATTTTAATT ATTTGAAGAA AAATAAAAAA AATAGACCAA GCGCGAGGGC   
  
  
+ TTGCCAACCC AGGCCAACAC TTGGGTTTTT TGTTGCTTTT TAAACAAGAC AGATGACATG TCGTCTACAG   
  
  
+ AGGAGAGTGA TATGTCATCT GTAGAGTTAG ACAACATGTC ATCTGCAAAC CTATTTTTTA ACATCTAGAG   
  
  
+ AGTACTGGAA AATCTAGCTG GGTATTTGTT TTTGTCCAAA AACTTAATTT TAATTTATTT TTATCTAAAG   
  
  
+ ACACCTAGAG AAAACCATTA TGAACTCAAA AAAAACTAAT TCATCAACTT GAAAAAAAAA AACAAATACA   
  
  
+ AAATGGATTA AAAACATAAA ACTAGTTTGG GAAAAAAAAT TTCTCTCTCT AACTAGATCT AAACTTTCCA   
  
  
+ GCAATATAAA TACTTAAAAA TATTTTAATA ACATTTTTTT AACATATTGA TATCAAATTT AACCATTATT   
  
  
+ TTCAATAAAA TTCAACCAAC CAATTTCTGT CTCATCATTC GTGTTCCTAT GATTCTCTCT TTTTCTTTCA   
  
  
+ AACTCTCACA AGGTAAATAA AATTATATTA TGTGCTTGAA AGGAAATTTG AAAAATCTAA AGGAAAACTT   
  
  
+ AACTTAAAAG GTGCTGACAT TTTTTATTAA AAAAAGCTCA TATTGTTTTT TCTTTAATTT TGACTTTATT   
  
  
+ CTTTTCTTTT TCAAATAATA AAATTAGATT CGTTTCCATA AAATTTAATG AACTTTTAGG CAATAATGTT   
  
  
+ GTACGAAGTC AATAATGACG TGCTACAATC GTATGTCACG ACAAAAAGAC GACATGAACA CCATCCATTG   
  
  
+ GTATTCATGT GCCTTTTCAC CTTGTAAAAT AACAGGAGAA AGATACCTTC CAGGGGCAAA TTCATCTATG   
  
  
+ CCACGTGCCT ACAAGTTGAA GGTATGTTTG GTATGCAAG  

- ACATATTATT TTGTTCACGT ATATATAGTA GTACTGTGTT TTTATCTTTA TATCCGGTAT TATAATTTAA   
  
  
- TCCGTAGATA ACGTGGTCGG TACTGTCTGT AAACCAATTA TTATATTATG AGTAACTATT TCCACAGTTA   
  
  
- CTGTGTTTAG ATACTAGGAT CACAACCTTT TAAATTTAAT AAATTTATTG TACCAATCAA CTATATAATA   
  
  
- AAAAGAATAT TAATCCATCT AACTCCCCCC TTTACTATAA AAATATTCAT TATTTATAAC TATTTTGTTC   
  
  
- ATCATCTGTG GACACAGCGT GTACAGATGT GCAACCACAA CCACACGCTG CAAATAATTA CGAATTTACC   
  
  
- ACCAAAGATC CTGATCATAC GCCCAAACCT GTCGTGCACG CTGCAAATTA AAGTAGGAGT AATTTAATCA   
  
  
- CAAATAAAAA AATAAAAAAT AAAAAATAAA AAAATAAAAA AATTAACTTA AATAAAAAAT TAAAATAGTG   
  
  
- AGTAATAAAT TAAATTAAAA TAAACATACA ATTTAAATCA AAAATAAGAA AAATAAAGAT AAACAAAATG   
  
  
- AAATCTTTTT AAAAACTAAT TTTAAAAAAA AAAGTTATAT TAGGAAATCT AAATCAAAAA AAAAGAAAAA   
  
  
- TTAAAATCGA GAGTTGTAAA CTTAACATTC CTACACCAAA CTGAATTTTT TTAAAGTTCT ATTGTACAAA   
  
  
- AATTTATAAC CTTACTGTTG TATAATCTAG TTAGGTTCAG CTGAGTGATC ATTTTTTTTA TTTGAATAAT   
  
  
- TTAGGTGTAA CCAGTACCTA GGTTCATCTA AATTATTAAA AAAATTTGGT CAAAAATAAA TTAATGTACT   
  
  
- ATTTTTTATT CGCTATCATC AATTTGTTGG TTTAATTTAA ATGTTTAACT AATGTTACCT AACATTTTTT   
  
  
- GTGAACGATT ATAATGTTGT TTATAAAGAA AACAAAATAA GTTCTTGTTT CACTTTTACT TTACTTTAAA   
  
  
- CTATTTTGTT ATATTTTTTA TATAGTTAAT TGAATTATAA AAACTTTTGT TATATATTAT ATAAAATATA   
  
  
- AGTAATTAAA AATTCTTTAA TTAAAATTAA TAAACTTCTT TTTATTTTTT TTATCTGGTT CGCGCTCCCG   
  
  
- AACGGTTGGG TCCGGTTGTG AACCCAAAAA ACAACGAAAA ATTTGTTCTG TCTACTGTAC AGCAGATGTC   
  
  
- TCCTCTCACT ATACAGTAGA CATCTCAATC TGTTGTACAG TAGACGTTTG GATAAAAAAT TGTAGATCTC   
  
  
- TCATGACCTT TTAGATCGAC CCATAAACAA AAACAGGTTT TTGAATTAAA ATTAAATAAA AATAGATTTC   
  
  
- TGTGGATCTC TTTTGGTAAT ACTTGAGTTT TTTTTGATTA AGTAGTTGAA CTTTTTTTTT TTGTTTATGT   
  
  
- TTTACCTAAT TTTTGTATTT TGATCAAACC CTTTTTTTTA AAGAGAGAGA TTGATCTAGA TTTGAAAGGT   
  
  
- CGTTATATTT ATGAATTTTT ATAAAATTAT TGTAAAAAAA TTGTATAACT ATAGTTTAAA TTGGTAATAA   
  
  
- AAGTTATTTT AAGTTGGTTG GTTAAAGACA GAGTAGTAAG CACAAGGATA CTAAGAGAGA AAAAGAAAGT   
  
  
- TTGAGAGTGT TCCATTTATT TTAATATAAT ACACGAACTT TCCTTTAAAC TTTTTAGATT TCCTTTTGAA   
  
  
- TTGAATTTTC CACGACTGTA AAAAATAATT TTTTTCGAGT ATAACAAAAA AGAAATTAAA ACTGAAATAA   
  
  
- GAAAAGAAAA AGTTTATTAT TTTAATCTAA GCAAAGGTAT TTTAAATTAC TTGAAAATCC GTTATTACAA   
  
  
- CATGCTTCAG TTATTACTGC ACGATGTTAG CATACAGTGC TGTTTTTCTG CTGTACTTGT GGTAGGTAAC   
  
  
- CATAAGTACA CGGAAAAGTG GAACATTTTA TTGTCCTCTT TCTATGGAAG GTCCCCGTTT AAGTAGATAC   
  
  
- GGTGCACGGA TGTTCAACTT CCATACAAAC CATACGTTC

+     MYB

| Site Name | Organism | Position | Strand | Matrix score. | sequence | function |
| --- | --- | --- | --- | --- | --- | --- |
| MYB | Arabidopsis thaliana | 1557 | + | 6 | CAACCA |  |
| MYB | Arabidopsis thaliana | 1553 | + | 6 | CAACCA |  |
| MYB | Arabidopsis thaliana | 1530 | + | 6 | TAACCA |  |
| MYB | Arabidopsis thaliana | 192 | - | 6 | TAACCA |  |
| MYB | Arabidopsis thaliana | 866 | + | 6 | CAACCA |  |
| MYB | Arabidopsis thaliana | 103 | - | 6 | TAACCA |  |

>Potri.011G061700.1   
+ TGTATAATAA AACAAGTGCA TATATATCAT CATGACACAA AAATAGAAAT ATAGGCCATA ATATTAAATT   
  
  
+ AGGCATCTAT TGCACCAGCC ATGACAGACA TTTGGTTAAT AATATAATAC TCATTGATAA AGGTGTCAAT   
  
  
+ GACACAAATC TATGATCCTA GTGTTGGAAA ATTTAAATTA TTTAAATAAC ATGGTTAGTT GATATATTAT   
  
  
+ TTTTCTTATA ATTAGGTAGA TTGAGGGGGG AAATGATATT TTTATAAGTA ATAAATATTG ATAAAACAAG   
  
  
+ TAGTAGACAC CTGTGTCGCA CATGTCTACA CGTTGGTGTT GGTGTGCGAC GTTTATTAAT GCTTAAATGG   
  
  
+ TGGTTTCTAG GACTAGTATG CGGGTTTGGA CAGCACGTGC GACGTTTAAT TTCATCCTCA TTAAATTAGT   
  
  
+ GTTTATTTTT TTATTTTTTA TTTTTTATTT TTTTATTTTT TTAATTGAAT TTATTTTTTA ATTTTATCAC   
  
  
+ TCATTATTTA ATTTAATTTT ATTTGTATGT TAAATTTAGT TTTTATTCTT TTTATTTCTA TTTGTTTTAC   
  
  
+ TTTAGAAAAA TTTTTGATTA AAATTTTTTT TTTCAATATA ATCCTTTAGA TTTAGTTTTT TTTTCTTTTT   
  
  
+ AATTTTAGCT CTCAACATTT GAATTGTAAG GATGTGGTTT GACTTAAAAA AATTTCAAGA TAACATGTTT   
  
  
+ TTAAATATTG GAATGACAAC ATATTAGATC AATCCAAGTC GACTCACTAG TAAAAAAAAT AAACTTATTA   
  
  
+ AATCCACATT GGTCATGGAT CCAAGTAGAT TTAATAATTT TTTTAAACCA GTTTTTATTT AATTACATGA   
  
  
+ TAAAAAATAA GCGATAGTAG TTAAACAACC AAATTAAATT TACAAATTGA TTACAATGGA TTGTAAAAAA   
  
  
+ CACTTGCTAA TATTACAACA AATATTTCTT TTGTTTTATT CAAGAACAAA GTGAAAATGA AATGAAATTT   
  
  
+ GATAAAACAA TATAAAAAAT ATATCAATTA ACTTAATATT TTTGAAAACA ATATATAATA TATTTTATAT   
  
  
+ TCATTAATTT TTAAGAAATT AATTTTAATT ATTTGAAGAA AAATAAAAAA AATAGACCAA GCGCGAGGGC   
  
  
+ TTGCCAACCC AGGCCAACAC TTGGGTTTTT TGTTGCTTTT TAAACAAGAC AGATGACATG TCGTCTACAG   
  
  
+ AGGAGAGTGA TATGTCATCT GTAGAGTTAG ACAACATGTC ATCTGCAAAC CTATTTTTTA ACATCTAGAG   
  
  
+ AGTACTGGAA AATCTAGCTG GGTATTTGTT TTTGTCCAAA AACTTAATTT TAATTTATTT TTATCTAAAG   
  
  
+ ACACCTAGAG AAAACCATTA TGAACTCAAA AAAAACTAAT TCATCAACTT GAAAAAAAAA AACAAATACA   
  
  
+ AAATGGATTA AAAACATAAA ACTAGTTTGG GAAAAAAAAT TTCTCTCTCT AACTAGATCT AAACTTTCCA   
  
  
+ GCAATATAAA TACTTAAAAA TATTTTAATA ACATTTTTTT AACATATTGA TATCAAATTT AACCATTATT   
  
  
+ TTCAATAAAA TTCAACCAAC CAATTTCTGT CTCATCATTC GTGTTCCTAT GATTCTCTCT TTTTCTTTCA   
  
  
+ AACTCTCACA AGGTAAATAA AATTATATTA TGTGCTTGAA AGGAAATTTG AAAAATCTAA AGGAAAACTT   
  
  
+ AACTTAAAAG GTGCTGACAT TTTTTATTAA AAAAAGCTCA TATTGTTTTT TCTTTAATTT TGACTTTATT   
  
  
+ CTTTTCTTTT TCAAATAATA AAATTAGATT CGTTTCCATA AAATTTAATG AACTTTTAGG CAATAATGTT   
  
  
+ GTACGAAGTC AATAATGACG TGCTACAATC GTATGTCACG ACAAAAAGAC GACATGAACA CCATCCATTG   
  
  
+ GTATTCATGT GCCTTTTCAC CTTGTAAAAT AACAGGAGAA AGATACCTTC CAGGGGCAAA TTCATCTATG   
  
  
+ CCACGTGCCT ACAAGTTGAA GGTATGTTTG GTATGCAAG  

- ACATATTATT TTGTTCACGT ATATATAGTA GTACTGTGTT TTTATCTTTA TATCCGGTAT TATAATTTAA   
  
  
- TCCGTAGATA ACGTGGTCGG TACTGTCTGT AAACCAATTA TTATATTATG AGTAACTATT TCCACAGTTA   
  
  
- CTGTGTTTAG ATACTAGGAT CACAACCTTT TAAATTTAAT AAATTTATTG TACCAATCAA CTATATAATA   
  
  
- AAAAGAATAT TAATCCATCT AACTCCCCCC TTTACTATAA AAATATTCAT TATTTATAAC TATTTTGTTC   
  
  
- ATCATCTGTG GACACAGCGT GTACAGATGT GCAACCACAA CCACACGCTG CAAATAATTA CGAATTTACC   
  
  
- ACCAAAGATC CTGATCATAC GCCCAAACCT GTCGTGCACG CTGCAAATTA AAGTAGGAGT AATTTAATCA   
  
  
- CAAATAAAAA AATAAAAAAT AAAAAATAAA AAAATAAAAA AATTAACTTA AATAAAAAAT TAAAATAGTG   
  
  
- AGTAATAAAT TAAATTAAAA TAAACATACA ATTTAAATCA AAAATAAGAA AAATAAAGAT AAACAAAATG   
  
  
- AAATCTTTTT AAAAACTAAT TTTAAAAAAA AAAGTTATAT TAGGAAATCT AAATCAAAAA AAAAGAAAAA   
  
  
- TTAAAATCGA GAGTTGTAAA CTTAACATTC CTACACCAAA CTGAATTTTT TTAAAGTTCT ATTGTACAAA   
  
  
- AATTTATAAC CTTACTGTTG TATAATCTAG TTAGGTTCAG CTGAGTGATC ATTTTTTTTA TTTGAATAAT   
  
  
- TTAGGTGTAA CCAGTACCTA GGTTCATCTA AATTATTAAA AAAATTTGGT CAAAAATAAA TTAATGTACT   
  
  
- ATTTTTTATT CGCTATCATC AATTTGTTGG TTTAATTTAA ATGTTTAACT AATGTTACCT AACATTTTTT   
  
  
- GTGAACGATT ATAATGTTGT TTATAAAGAA AACAAAATAA GTTCTTGTTT CACTTTTACT TTACTTTAAA   
  
  
- CTATTTTGTT ATATTTTTTA TATAGTTAAT TGAATTATAA AAACTTTTGT TATATATTAT ATAAAATATA   
  
  
- AGTAATTAAA AATTCTTTAA TTAAAATTAA TAAACTTCTT TTTATTTTTT TTATCTGGTT CGCGCTCCCG   
  
  
- AACGGTTGGG TCCGGTTGTG AACCCAAAAA ACAACGAAAA ATTTGTTCTG TCTACTGTAC AGCAGATGTC   
  
  
- TCCTCTCACT ATACAGTAGA CATCTCAATC TGTTGTACAG TAGACGTTTG GATAAAAAAT TGTAGATCTC   
  
  
- TCATGACCTT TTAGATCGAC CCATAAACAA AAACAGGTTT TTGAATTAAA ATTAAATAAA AATAGATTTC   
  
  
- TGTGGATCTC TTTTGGTAAT ACTTGAGTTT TTTTTGATTA AGTAGTTGAA CTTTTTTTTT TTGTTTATGT   
  
  
- TTTACCTAAT TTTTGTATTT TGATCAAACC CTTTTTTTTA AAGAGAGAGA TTGATCTAGA TTTGAAAGGT   
  
  
- CGTTATATTT ATGAATTTTT ATAAAATTAT TGTAAAAAAA TTGTATAACT ATAGTTTAAA TTGGTAATAA   
  
  
- AAGTTATTTT AAGTTGGTTG GTTAAAGACA GAGTAGTAAG CACAAGGATA CTAAGAGAGA AAAAGAAAGT   
  
  
- TTGAGAGTGT TCCATTTATT TTAATATAAT ACACGAACTT TCCTTTAAAC TTTTTAGATT TCCTTTTGAA   
  
  
- TTGAATTTTC CACGACTGTA AAAAATAATT TTTTTCGAGT ATAACAAAAA AGAAATTAAA ACTGAAATAA   
  
  
- GAAAAGAAAA AGTTTATTAT TTTAATCTAA GCAAAGGTAT TTTAAATTAC TTGAAAATCC GTTATTACAA   
  
  
- CATGCTTCAG TTATTACTGC ACGATGTTAG CATACAGTGC TGTTTTTCTG CTGTACTTGT GGTAGGTAAC   
  
  
- CATAAGTACA CGGAAAAGTG GAACATTTTA TTGTCCTCTT TCTATGGAAG GTCCCCGTTT AAGTAGATAC   
  
  
- GGTGCACGGA TGTTCAACTT CCATACAAAC CATACGTTC

+     MYB-like sequence

| Site Name | Organism | Position | Strand | Matrix score. | sequence | function |
| --- | --- | --- | --- | --- | --- | --- |
| MYB-like sequence | Arabidopsis thaliana | 1530 | + | 6 | TAACCA |  |
| MYB-like sequence | Arabidopsis thaliana | 192 | - | 6 | TAACCA |  |
| MYB-like sequence | Arabidopsis thaliana | 103 | - | 6 | TAACCA |  |

>Potri.011G061700.1   
+ TGTATAATAA AACAAGTGCA TATATATCAT CATGACACAA AAATAGAAAT ATAGGCCATA ATATTAAATT   
  
  
+ AGGCATCTAT TGCACCAGCC ATGACAGACA TTTGGTTAAT AATATAATAC TCATTGATAA AGGTGTCAAT   
  
  
+ GACACAAATC TATGATCCTA GTGTTGGAAA ATTTAAATTA TTTAAATAAC ATGGTTAGTT GATATATTAT   
  
  
+ TTTTCTTATA ATTAGGTAGA TTGAGGGGGG AAATGATATT TTTATAAGTA ATAAATATTG ATAAAACAAG   
  
  
+ TAGTAGACAC CTGTGTCGCA CATGTCTACA CGTTGGTGTT GGTGTGCGAC GTTTATTAAT GCTTAAATGG   
  
  
+ TGGTTTCTAG GACTAGTATG CGGGTTTGGA CAGCACGTGC GACGTTTAAT TTCATCCTCA TTAAATTAGT   
  
  
+ GTTTATTTTT TTATTTTTTA TTTTTTATTT TTTTATTTTT TTAATTGAAT TTATTTTTTA ATTTTATCAC   
  
  
+ TCATTATTTA ATTTAATTTT ATTTGTATGT TAAATTTAGT TTTTATTCTT TTTATTTCTA TTTGTTTTAC   
  
  
+ TTTAGAAAAA TTTTTGATTA AAATTTTTTT TTTCAATATA ATCCTTTAGA TTTAGTTTTT TTTTCTTTTT   
  
  
+ AATTTTAGCT CTCAACATTT GAATTGTAAG GATGTGGTTT GACTTAAAAA AATTTCAAGA TAACATGTTT   
  
  
+ TTAAATATTG GAATGACAAC ATATTAGATC AATCCAAGTC GACTCACTAG TAAAAAAAAT AAACTTATTA   
  
  
+ AATCCACATT GGTCATGGAT CCAAGTAGAT TTAATAATTT TTTTAAACCA GTTTTTATTT AATTACATGA   
  
  
+ TAAAAAATAA GCGATAGTAG TTAAACAACC AAATTAAATT TACAAATTGA TTACAATGGA TTGTAAAAAA   
  
  
+ CACTTGCTAA TATTACAACA AATATTTCTT TTGTTTTATT CAAGAACAAA GTGAAAATGA AATGAAATTT   
  
  
+ GATAAAACAA TATAAAAAAT ATATCAATTA ACTTAATATT TTTGAAAACA ATATATAATA TATTTTATAT   
  
  
+ TCATTAATTT TTAAGAAATT AATTTTAATT ATTTGAAGAA AAATAAAAAA AATAGACCAA GCGCGAGGGC   
  
  
+ TTGCCAACCC AGGCCAACAC TTGGGTTTTT TGTTGCTTTT TAAACAAGAC AGATGACATG TCGTCTACAG   
  
  
+ AGGAGAGTGA TATGTCATCT GTAGAGTTAG ACAACATGTC ATCTGCAAAC CTATTTTTTA ACATCTAGAG   
  
  
+ AGTACTGGAA AATCTAGCTG GGTATTTGTT TTTGTCCAAA AACTTAATTT TAATTTATTT TTATCTAAAG   
  
  
+ ACACCTAGAG AAAACCATTA TGAACTCAAA AAAAACTAAT TCATCAACTT GAAAAAAAAA AACAAATACA   
  
  
+ AAATGGATTA AAAACATAAA ACTAGTTTGG GAAAAAAAAT TTCTCTCTCT AACTAGATCT AAACTTTCCA   
  
  
+ GCAATATAAA TACTTAAAAA TATTTTAATA ACATTTTTTT AACATATTGA TATCAAATTT AACCATTATT   
  
  
+ TTCAATAAAA TTCAACCAAC CAATTTCTGT CTCATCATTC GTGTTCCTAT GATTCTCTCT TTTTCTTTCA   
  
  
+ AACTCTCACA AGGTAAATAA AATTATATTA TGTGCTTGAA AGGAAATTTG AAAAATCTAA AGGAAAACTT   
  
  
+ AACTTAAAAG GTGCTGACAT TTTTTATTAA AAAAAGCTCA TATTGTTTTT TCTTTAATTT TGACTTTATT   
  
  
+ CTTTTCTTTT TCAAATAATA AAATTAGATT CGTTTCCATA AAATTTAATG AACTTTTAGG CAATAATGTT   
  
  
+ GTACGAAGTC AATAATGACG TGCTACAATC GTATGTCACG ACAAAAAGAC GACATGAACA CCATCCATTG   
  
  
+ GTATTCATGT GCCTTTTCAC CTTGTAAAAT AACAGGAGAA AGATACCTTC CAGGGGCAAA TTCATCTATG   
  
  
+ CCACGTGCCT ACAAGTTGAA GGTATGTTTG GTATGCAAG  

- ACATATTATT TTGTTCACGT ATATATAGTA GTACTGTGTT TTTATCTTTA TATCCGGTAT TATAATTTAA   
  
  
- TCCGTAGATA ACGTGGTCGG TACTGTCTGT AAACCAATTA TTATATTATG AGTAACTATT TCCACAGTTA   
  
  
- CTGTGTTTAG ATACTAGGAT CACAACCTTT TAAATTTAAT AAATTTATTG TACCAATCAA CTATATAATA   
  
  
- AAAAGAATAT TAATCCATCT AACTCCCCCC TTTACTATAA AAATATTCAT TATTTATAAC TATTTTGTTC   
  
  
- ATCATCTGTG GACACAGCGT GTACAGATGT GCAACCACAA CCACACGCTG CAAATAATTA CGAATTTACC   
  
  
- ACCAAAGATC CTGATCATAC GCCCAAACCT GTCGTGCACG CTGCAAATTA AAGTAGGAGT AATTTAATCA   
  
  
- CAAATAAAAA AATAAAAAAT AAAAAATAAA AAAATAAAAA AATTAACTTA AATAAAAAAT TAAAATAGTG   
  
  
- AGTAATAAAT TAAATTAAAA TAAACATACA ATTTAAATCA AAAATAAGAA AAATAAAGAT AAACAAAATG   
  
  
- AAATCTTTTT AAAAACTAAT TTTAAAAAAA AAAGTTATAT TAGGAAATCT AAATCAAAAA AAAAGAAAAA   
  
  
- TTAAAATCGA GAGTTGTAAA CTTAACATTC CTACACCAAA CTGAATTTTT TTAAAGTTCT ATTGTACAAA   
  
  
- AATTTATAAC CTTACTGTTG TATAATCTAG TTAGGTTCAG CTGAGTGATC ATTTTTTTTA TTTGAATAAT   
  
  
- TTAGGTGTAA CCAGTACCTA GGTTCATCTA AATTATTAAA AAAATTTGGT CAAAAATAAA TTAATGTACT   
  
  
- ATTTTTTATT CGCTATCATC AATTTGTTGG TTTAATTTAA ATGTTTAACT AATGTTACCT AACATTTTTT   
  
  
- GTGAACGATT ATAATGTTGT TTATAAAGAA AACAAAATAA GTTCTTGTTT CACTTTTACT TTACTTTAAA   
  
  
- CTATTTTGTT ATATTTTTTA TATAGTTAAT TGAATTATAA AAACTTTTGT TATATATTAT ATAAAATATA   
  
  
- AGTAATTAAA AATTCTTTAA TTAAAATTAA TAAACTTCTT TTTATTTTTT TTATCTGGTT CGCGCTCCCG   
  
  
- AACGGTTGGG TCCGGTTGTG AACCCAAAAA ACAACGAAAA ATTTGTTCTG TCTACTGTAC AGCAGATGTC   
  
  
- TCCTCTCACT ATACAGTAGA CATCTCAATC TGTTGTACAG TAGACGTTTG GATAAAAAAT TGTAGATCTC   
  
  
- TCATGACCTT TTAGATCGAC CCATAAACAA AAACAGGTTT TTGAATTAAA ATTAAATAAA AATAGATTTC   
  
  
- TGTGGATCTC TTTTGGTAAT ACTTGAGTTT TTTTTGATTA AGTAGTTGAA CTTTTTTTTT TTGTTTATGT   
  
  
- TTTACCTAAT TTTTGTATTT TGATCAAACC CTTTTTTTTA AAGAGAGAGA TTGATCTAGA TTTGAAAGGT   
  
  
- CGTTATATTT ATGAATTTTT ATAAAATTAT TGTAAAAAAA TTGTATAACT ATAGTTTAAA TTGGTAATAA   
  
  
- AAGTTATTTT AAGTTGGTTG GTTAAAGACA GAGTAGTAAG CACAAGGATA CTAAGAGAGA AAAAGAAAGT   
  
  
- TTGAGAGTGT TCCATTTATT TTAATATAAT ACACGAACTT TCCTTTAAAC TTTTTAGATT TCCTTTTGAA   
  
  
- TTGAATTTTC CACGACTGTA AAAAATAATT TTTTTCGAGT ATAACAAAAA AGAAATTAAA ACTGAAATAA   
  
  
- GAAAAGAAAA AGTTTATTAT TTTAATCTAA GCAAAGGTAT TTTAAATTAC TTGAAAATCC GTTATTACAA   
  
  
- CATGCTTCAG TTATTACTGC ACGATGTTAG CATACAGTGC TGTTTTTCTG CTGTACTTGT GGTAGGTAAC   
  
  
- CATAAGTACA CGGAAAAGTG GAACATTTTA TTGTCCTCTT TCTATGGAAG GTCCCCGTTT AAGTAGATAC   
  
  
- GGTGCACGGA TGTTCAACTT CCATACAAAC CATACGTTC

+     MYC

| Site Name | Organism | Position | Strand | Matrix score. | sequence | function |
| --- | --- | --- | --- | --- | --- | --- |
| MYC | Arabidopsis thaliana | 1896 | + | 6 | CATGTG |  |
| MYC | Arabidopsis thaliana | 299 | - | 6 | CATGTG |  |
| MYC | Arabidopsis thaliana | 99 | + | 6 | CATTTG |  |
| MYC | Arabidopsis thaliana | 646 | + | 6 | CATTTG |  |

>Potri.011G061700.1   
+ TGTATAATAA AACAAGTGCA TATATATCAT CATGACACAA AAATAGAAAT ATAGGCCATA ATATTAAATT   
  
  
+ AGGCATCTAT TGCACCAGCC ATGACAGACA TTTGGTTAAT AATATAATAC TCATTGATAA AGGTGTCAAT   
  
  
+ GACACAAATC TATGATCCTA GTGTTGGAAA ATTTAAATTA TTTAAATAAC ATGGTTAGTT GATATATTAT   
  
  
+ TTTTCTTATA ATTAGGTAGA TTGAGGGGGG AAATGATATT TTTATAAGTA ATAAATATTG ATAAAACAAG   
  
  
+ TAGTAGACAC CTGTGTCGCA CATGTCTACA CGTTGGTGTT GGTGTGCGAC GTTTATTAAT GCTTAAATGG   
  
  
+ TGGTTTCTAG GACTAGTATG CGGGTTTGGA CAGCACGTGC GACGTTTAAT TTCATCCTCA TTAAATTAGT   
  
  
+ GTTTATTTTT TTATTTTTTA TTTTTTATTT TTTTATTTTT TTAATTGAAT TTATTTTTTA ATTTTATCAC   
  
  
+ TCATTATTTA ATTTAATTTT ATTTGTATGT TAAATTTAGT TTTTATTCTT TTTATTTCTA TTTGTTTTAC   
  
  
+ TTTAGAAAAA TTTTTGATTA AAATTTTTTT TTTCAATATA ATCCTTTAGA TTTAGTTTTT TTTTCTTTTT   
  
  
+ AATTTTAGCT CTCAACATTT GAATTGTAAG GATGTGGTTT GACTTAAAAA AATTTCAAGA TAACATGTTT   
  
  
+ TTAAATATTG GAATGACAAC ATATTAGATC AATCCAAGTC GACTCACTAG TAAAAAAAAT AAACTTATTA   
  
  
+ AATCCACATT GGTCATGGAT CCAAGTAGAT TTAATAATTT TTTTAAACCA GTTTTTATTT AATTACATGA   
  
  
+ TAAAAAATAA GCGATAGTAG TTAAACAACC AAATTAAATT TACAAATTGA TTACAATGGA TTGTAAAAAA   
  
  
+ CACTTGCTAA TATTACAACA AATATTTCTT TTGTTTTATT CAAGAACAAA GTGAAAATGA AATGAAATTT   
  
  
+ GATAAAACAA TATAAAAAAT ATATCAATTA ACTTAATATT TTTGAAAACA ATATATAATA TATTTTATAT   
  
  
+ TCATTAATTT TTAAGAAATT AATTTTAATT ATTTGAAGAA AAATAAAAAA AATAGACCAA GCGCGAGGGC   
  
  
+ TTGCCAACCC AGGCCAACAC TTGGGTTTTT TGTTGCTTTT TAAACAAGAC AGATGACATG TCGTCTACAG   
  
  
+ AGGAGAGTGA TATGTCATCT GTAGAGTTAG ACAACATGTC ATCTGCAAAC CTATTTTTTA ACATCTAGAG   
  
  
+ AGTACTGGAA AATCTAGCTG GGTATTTGTT TTTGTCCAAA AACTTAATTT TAATTTATTT TTATCTAAAG   
  
  
+ ACACCTAGAG AAAACCATTA TGAACTCAAA AAAAACTAAT TCATCAACTT GAAAAAAAAA AACAAATACA   
  
  
+ AAATGGATTA AAAACATAAA ACTAGTTTGG GAAAAAAAAT TTCTCTCTCT AACTAGATCT AAACTTTCCA   
  
  
+ GCAATATAAA TACTTAAAAA TATTTTAATA ACATTTTTTT AACATATTGA TATCAAATTT AACCATTATT   
  
  
+ TTCAATAAAA TTCAACCAAC CAATTTCTGT CTCATCATTC GTGTTCCTAT GATTCTCTCT TTTTCTTTCA   
  
  
+ AACTCTCACA AGGTAAATAA AATTATATTA TGTGCTTGAA AGGAAATTTG AAAAATCTAA AGGAAAACTT   
  
  
+ AACTTAAAAG GTGCTGACAT TTTTTATTAA AAAAAGCTCA TATTGTTTTT TCTTTAATTT TGACTTTATT   
  
  
+ CTTTTCTTTT TCAAATAATA AAATTAGATT CGTTTCCATA AAATTTAATG AACTTTTAGG CAATAATGTT   
  
  
+ GTACGAAGTC AATAATGACG TGCTACAATC GTATGTCACG ACAAAAAGAC GACATGAACA CCATCCATTG   
  
  
+ GTATTCATGT GCCTTTTCAC CTTGTAAAAT AACAGGAGAA AGATACCTTC CAGGGGCAAA TTCATCTATG   
  
  
+ CCACGTGCCT ACAAGTTGAA GGTATGTTTG GTATGCAAG  

- ACATATTATT TTGTTCACGT ATATATAGTA GTACTGTGTT TTTATCTTTA TATCCGGTAT TATAATTTAA   
  
  
- TCCGTAGATA ACGTGGTCGG TACTGTCTGT AAACCAATTA TTATATTATG AGTAACTATT TCCACAGTTA   
  
  
- CTGTGTTTAG ATACTAGGAT CACAACCTTT TAAATTTAAT AAATTTATTG TACCAATCAA CTATATAATA   
  
  
- AAAAGAATAT TAATCCATCT AACTCCCCCC TTTACTATAA AAATATTCAT TATTTATAAC TATTTTGTTC   
  
  
- ATCATCTGTG GACACAGCGT GTACAGATGT GCAACCACAA CCACACGCTG CAAATAATTA CGAATTTACC   
  
  
- ACCAAAGATC CTGATCATAC GCCCAAACCT GTCGTGCACG CTGCAAATTA AAGTAGGAGT AATTTAATCA   
  
  
- CAAATAAAAA AATAAAAAAT AAAAAATAAA AAAATAAAAA AATTAACTTA AATAAAAAAT TAAAATAGTG   
  
  
- AGTAATAAAT TAAATTAAAA TAAACATACA ATTTAAATCA AAAATAAGAA AAATAAAGAT AAACAAAATG   
  
  
- AAATCTTTTT AAAAACTAAT TTTAAAAAAA AAAGTTATAT TAGGAAATCT AAATCAAAAA AAAAGAAAAA   
  
  
- TTAAAATCGA GAGTTGTAAA CTTAACATTC CTACACCAAA CTGAATTTTT TTAAAGTTCT ATTGTACAAA   
  
  
- AATTTATAAC CTTACTGTTG TATAATCTAG TTAGGTTCAG CTGAGTGATC ATTTTTTTTA TTTGAATAAT   
  
  
- TTAGGTGTAA CCAGTACCTA GGTTCATCTA AATTATTAAA AAAATTTGGT CAAAAATAAA TTAATGTACT   
  
  
- ATTTTTTATT CGCTATCATC AATTTGTTGG TTTAATTTAA ATGTTTAACT AATGTTACCT AACATTTTTT   
  
  
- GTGAACGATT ATAATGTTGT TTATAAAGAA AACAAAATAA GTTCTTGTTT CACTTTTACT TTACTTTAAA   
  
  
- CTATTTTGTT ATATTTTTTA TATAGTTAAT TGAATTATAA AAACTTTTGT TATATATTAT ATAAAATATA   
  
  
- AGTAATTAAA AATTCTTTAA TTAAAATTAA TAAACTTCTT TTTATTTTTT TTATCTGGTT CGCGCTCCCG   
  
  
- AACGGTTGGG TCCGGTTGTG AACCCAAAAA ACAACGAAAA ATTTGTTCTG TCTACTGTAC AGCAGATGTC   
  
  
- TCCTCTCACT ATACAGTAGA CATCTCAATC TGTTGTACAG TAGACGTTTG GATAAAAAAT TGTAGATCTC   
  
  
- TCATGACCTT TTAGATCGAC CCATAAACAA AAACAGGTTT TTGAATTAAA ATTAAATAAA AATAGATTTC   
  
  
- TGTGGATCTC TTTTGGTAAT ACTTGAGTTT TTTTTGATTA AGTAGTTGAA CTTTTTTTTT TTGTTTATGT   
  
  
- TTTACCTAAT TTTTGTATTT TGATCAAACC CTTTTTTTTA AAGAGAGAGA TTGATCTAGA TTTGAAAGGT   
  
  
- CGTTATATTT ATGAATTTTT ATAAAATTAT TGTAAAAAAA TTGTATAACT ATAGTTTAAA TTGGTAATAA   
  
  
- AAGTTATTTT AAGTTGGTTG GTTAAAGACA GAGTAGTAAG CACAAGGATA CTAAGAGAGA AAAAGAAAGT   
  
  
- TTGAGAGTGT TCCATTTATT TTAATATAAT ACACGAACTT TCCTTTAAAC TTTTTAGATT TCCTTTTGAA   
  
  
- TTGAATTTTC CACGACTGTA AAAAATAATT TTTTTCGAGT ATAACAAAAA AGAAATTAAA ACTGAAATAA   
  
  
- GAAAAGAAAA AGTTTATTAT TTTAATCTAA GCAAAGGTAT TTTAAATTAC TTGAAAATCC GTTATTACAA   
  
  
- CATGCTTCAG TTATTACTGC ACGATGTTAG CATACAGTGC TGTTTTTCTG CTGTACTTGT GGTAGGTAAC   
  
  
- CATAAGTACA CGGAAAAGTG GAACATTTTA TTGTCCTCTT TCTATGGAAG GTCCCCGTTT AAGTAGATAC   
  
  
- GGTGCACGGA TGTTCAACTT CCATACAAAC CATACGTTC

+     O2-site

| Site Name | Organism | Position | Strand | Matrix score. | sequence | function |
| --- | --- | --- | --- | --- | --- | --- |
| O2-site | Zea mays | 1172 | + | 9 | GATGACATGG | cis-acting regulatory element involved in zein metabolism regulation |
| O2-site | Zea mays | 1224 | - | 9 | GATGACATGG | cis-acting regulatory element involved in zein metabolism regulation |

>Potri.011G061700.1   
+ TGTATAATAA AACAAGTGCA TATATATCAT CATGACACAA AAATAGAAAT ATAGGCCATA ATATTAAATT   
  
  
+ AGGCATCTAT TGCACCAGCC ATGACAGACA TTTGGTTAAT AATATAATAC TCATTGATAA AGGTGTCAAT   
  
  
+ GACACAAATC TATGATCCTA GTGTTGGAAA ATTTAAATTA TTTAAATAAC ATGGTTAGTT GATATATTAT   
  
  
+ TTTTCTTATA ATTAGGTAGA TTGAGGGGGG AAATGATATT TTTATAAGTA ATAAATATTG ATAAAACAAG   
  
  
+ TAGTAGACAC CTGTGTCGCA CATGTCTACA CGTTGGTGTT GGTGTGCGAC GTTTATTAAT GCTTAAATGG   
  
  
+ TGGTTTCTAG GACTAGTATG CGGGTTTGGA CAGCACGTGC GACGTTTAAT TTCATCCTCA TTAAATTAGT   
  
  
+ GTTTATTTTT TTATTTTTTA TTTTTTATTT TTTTATTTTT TTAATTGAAT TTATTTTTTA ATTTTATCAC   
  
  
+ TCATTATTTA ATTTAATTTT ATTTGTATGT TAAATTTAGT TTTTATTCTT TTTATTTCTA TTTGTTTTAC   
  
  
+ TTTAGAAAAA TTTTTGATTA AAATTTTTTT TTTCAATATA ATCCTTTAGA TTTAGTTTTT TTTTCTTTTT   
  
  
+ AATTTTAGCT CTCAACATTT GAATTGTAAG GATGTGGTTT GACTTAAAAA AATTTCAAGA TAACATGTTT   
  
  
+ TTAAATATTG GAATGACAAC ATATTAGATC AATCCAAGTC GACTCACTAG TAAAAAAAAT AAACTTATTA   
  
  
+ AATCCACATT GGTCATGGAT CCAAGTAGAT TTAATAATTT TTTTAAACCA GTTTTTATTT AATTACATGA   
  
  
+ TAAAAAATAA GCGATAGTAG TTAAACAACC AAATTAAATT TACAAATTGA TTACAATGGA TTGTAAAAAA   
  
  
+ CACTTGCTAA TATTACAACA AATATTTCTT TTGTTTTATT CAAGAACAAA GTGAAAATGA AATGAAATTT   
  
  
+ GATAAAACAA TATAAAAAAT ATATCAATTA ACTTAATATT TTTGAAAACA ATATATAATA TATTTTATAT   
  
  
+ TCATTAATTT TTAAGAAATT AATTTTAATT ATTTGAAGAA AAATAAAAAA AATAGACCAA GCGCGAGGGC   
  
  
+ TTGCCAACCC AGGCCAACAC TTGGGTTTTT TGTTGCTTTT TAAACAAGAC AGATGACATG TCGTCTACAG   
  
  
+ AGGAGAGTGA TATGTCATCT GTAGAGTTAG ACAACATGTC ATCTGCAAAC CTATTTTTTA ACATCTAGAG   
  
  
+ AGTACTGGAA AATCTAGCTG GGTATTTGTT TTTGTCCAAA AACTTAATTT TAATTTATTT TTATCTAAAG   
  
  
+ ACACCTAGAG AAAACCATTA TGAACTCAAA AAAAACTAAT TCATCAACTT GAAAAAAAAA AACAAATACA   
  
  
+ AAATGGATTA AAAACATAAA ACTAGTTTGG GAAAAAAAAT TTCTCTCTCT AACTAGATCT AAACTTTCCA   
  
  
+ GCAATATAAA TACTTAAAAA TATTTTAATA ACATTTTTTT AACATATTGA TATCAAATTT AACCATTATT   
  
  
+ TTCAATAAAA TTCAACCAAC CAATTTCTGT CTCATCATTC GTGTTCCTAT GATTCTCTCT TTTTCTTTCA   
  
  
+ AACTCTCACA AGGTAAATAA AATTATATTA TGTGCTTGAA AGGAAATTTG AAAAATCTAA AGGAAAACTT   
  
  
+ AACTTAAAAG GTGCTGACAT TTTTTATTAA AAAAAGCTCA TATTGTTTTT TCTTTAATTT TGACTTTATT   
  
  
+ CTTTTCTTTT TCAAATAATA AAATTAGATT CGTTTCCATA AAATTTAATG AACTTTTAGG CAATAATGTT   
  
  
+ GTACGAAGTC AATAATGACG TGCTACAATC GTATGTCACG ACAAAAAGAC GACATGAACA CCATCCATTG   
  
  
+ GTATTCATGT GCCTTTTCAC CTTGTAAAAT AACAGGAGAA AGATACCTTC CAGGGGCAAA TTCATCTATG   
  
  
+ CCACGTGCCT ACAAGTTGAA GGTATGTTTG GTATGCAAG  

- ACATATTATT TTGTTCACGT ATATATAGTA GTACTGTGTT TTTATCTTTA TATCCGGTAT TATAATTTAA   
  
  
- TCCGTAGATA ACGTGGTCGG TACTGTCTGT AAACCAATTA TTATATTATG AGTAACTATT TCCACAGTTA   
  
  
- CTGTGTTTAG ATACTAGGAT CACAACCTTT TAAATTTAAT AAATTTATTG TACCAATCAA CTATATAATA   
  
  
- AAAAGAATAT TAATCCATCT AACTCCCCCC TTTACTATAA AAATATTCAT TATTTATAAC TATTTTGTTC   
  
  
- ATCATCTGTG GACACAGCGT GTACAGATGT GCAACCACAA CCACACGCTG CAAATAATTA CGAATTTACC   
  
  
- ACCAAAGATC CTGATCATAC GCCCAAACCT GTCGTGCACG CTGCAAATTA AAGTAGGAGT AATTTAATCA   
  
  
- CAAATAAAAA AATAAAAAAT AAAAAATAAA AAAATAAAAA AATTAACTTA AATAAAAAAT TAAAATAGTG   
  
  
- AGTAATAAAT TAAATTAAAA TAAACATACA ATTTAAATCA AAAATAAGAA AAATAAAGAT AAACAAAATG   
  
  
- AAATCTTTTT AAAAACTAAT TTTAAAAAAA AAAGTTATAT TAGGAAATCT AAATCAAAAA AAAAGAAAAA   
  
  
- TTAAAATCGA GAGTTGTAAA CTTAACATTC CTACACCAAA CTGAATTTTT TTAAAGTTCT ATTGTACAAA   
  
  
- AATTTATAAC CTTACTGTTG TATAATCTAG TTAGGTTCAG CTGAGTGATC ATTTTTTTTA TTTGAATAAT   
  
  
- TTAGGTGTAA CCAGTACCTA GGTTCATCTA AATTATTAAA AAAATTTGGT CAAAAATAAA TTAATGTACT   
  
  
- ATTTTTTATT CGCTATCATC AATTTGTTGG TTTAATTTAA ATGTTTAACT AATGTTACCT AACATTTTTT   
  
  
- GTGAACGATT ATAATGTTGT TTATAAAGAA AACAAAATAA GTTCTTGTTT CACTTTTACT TTACTTTAAA   
  
  
- CTATTTTGTT ATATTTTTTA TATAGTTAAT TGAATTATAA AAACTTTTGT TATATATTAT ATAAAATATA   
  
  
- AGTAATTAAA AATTCTTTAA TTAAAATTAA TAAACTTCTT TTTATTTTTT TTATCTGGTT CGCGCTCCCG   
  
  
- AACGGTTGGG TCCGGTTGTG AACCCAAAAA ACAACGAAAA ATTTGTTCTG TCTACTGTAC AGCAGATGTC   
  
  
- TCCTCTCACT ATACAGTAGA CATCTCAATC TGTTGTACAG TAGACGTTTG GATAAAAAAT TGTAGATCTC   
  
  
- TCATGACCTT TTAGATCGAC CCATAAACAA AAACAGGTTT TTGAATTAAA ATTAAATAAA AATAGATTTC   
  
  
- TGTGGATCTC TTTTGGTAAT ACTTGAGTTT TTTTTGATTA AGTAGTTGAA CTTTTTTTTT TTGTTTATGT   
  
  
- TTTACCTAAT TTTTGTATTT TGATCAAACC CTTTTTTTTA AAGAGAGAGA TTGATCTAGA TTTGAAAGGT   
  
  
- CGTTATATTT ATGAATTTTT ATAAAATTAT TGTAAAAAAA TTGTATAACT ATAGTTTAAA TTGGTAATAA   
  
  
- AAGTTATTTT AAGTTGGTTG GTTAAAGACA GAGTAGTAAG CACAAGGATA CTAAGAGAGA AAAAGAAAGT   
  
  
- TTGAGAGTGT TCCATTTATT TTAATATAAT ACACGAACTT TCCTTTAAAC TTTTTAGATT TCCTTTTGAA   
  
  
- TTGAATTTTC CACGACTGTA AAAAATAATT TTTTTCGAGT ATAACAAAAA AGAAATTAAA ACTGAAATAA   
  
  
- GAAAAGAAAA AGTTTATTAT TTTAATCTAA GCAAAGGTAT TTTAAATTAC TTGAAAATCC GTTATTACAA   
  
  
- CATGCTTCAG TTATTACTGC ACGATGTTAG CATACAGTGC TGTTTTTCTG CTGTACTTGT GGTAGGTAAC   
  
  
- CATAAGTACA CGGAAAAGTG GAACATTTTA TTGTCCTCTT TCTATGGAAG GTCCCCGTTT AAGTAGATAC   
  
  
- GGTGCACGGA TGTTCAACTT CCATACAAAC CATACGTTC

+     STRE

| Site Name | Organism | Position | Strand | Matrix score. | sequence | function |
| --- | --- | --- | --- | --- | --- | --- |
| STRE | Arabidopsis thaliana | 1942 | + | 5 | AGGGG |  |
| STRE | Arabidopsis thaliana | 234 | + | 5 | AGGGG |  |

>Potri.011G061700.1   
+ TGTATAATAA AACAAGTGCA TATATATCAT CATGACACAA AAATAGAAAT ATAGGCCATA ATATTAAATT   
  
  
+ AGGCATCTAT TGCACCAGCC ATGACAGACA TTTGGTTAAT AATATAATAC TCATTGATAA AGGTGTCAAT   
  
  
+ GACACAAATC TATGATCCTA GTGTTGGAAA ATTTAAATTA TTTAAATAAC ATGGTTAGTT GATATATTAT   
  
  
+ TTTTCTTATA ATTAGGTAGA TTGAGGGGGG AAATGATATT TTTATAAGTA ATAAATATTG ATAAAACAAG   
  
  
+ TAGTAGACAC CTGTGTCGCA CATGTCTACA CGTTGGTGTT GGTGTGCGAC GTTTATTAAT GCTTAAATGG   
  
  
+ TGGTTTCTAG GACTAGTATG CGGGTTTGGA CAGCACGTGC GACGTTTAAT TTCATCCTCA TTAAATTAGT   
  
  
+ GTTTATTTTT TTATTTTTTA TTTTTTATTT TTTTATTTTT TTAATTGAAT TTATTTTTTA ATTTTATCAC   
  
  
+ TCATTATTTA ATTTAATTTT ATTTGTATGT TAAATTTAGT TTTTATTCTT TTTATTTCTA TTTGTTTTAC   
  
  
+ TTTAGAAAAA TTTTTGATTA AAATTTTTTT TTTCAATATA ATCCTTTAGA TTTAGTTTTT TTTTCTTTTT   
  
  
+ AATTTTAGCT CTCAACATTT GAATTGTAAG GATGTGGTTT GACTTAAAAA AATTTCAAGA TAACATGTTT   
  
  
+ TTAAATATTG GAATGACAAC ATATTAGATC AATCCAAGTC GACTCACTAG TAAAAAAAAT AAACTTATTA   
  
  
+ AATCCACATT GGTCATGGAT CCAAGTAGAT TTAATAATTT TTTTAAACCA GTTTTTATTT AATTACATGA   
  
  
+ TAAAAAATAA GCGATAGTAG TTAAACAACC AAATTAAATT TACAAATTGA TTACAATGGA TTGTAAAAAA   
  
  
+ CACTTGCTAA TATTACAACA AATATTTCTT TTGTTTTATT CAAGAACAAA GTGAAAATGA AATGAAATTT   
  
  
+ GATAAAACAA TATAAAAAAT ATATCAATTA ACTTAATATT TTTGAAAACA ATATATAATA TATTTTATAT   
  
  
+ TCATTAATTT TTAAGAAATT AATTTTAATT ATTTGAAGAA AAATAAAAAA AATAGACCAA GCGCGAGGGC   
  
  
+ TTGCCAACCC AGGCCAACAC TTGGGTTTTT TGTTGCTTTT TAAACAAGAC AGATGACATG TCGTCTACAG   
  
  
+ AGGAGAGTGA TATGTCATCT GTAGAGTTAG ACAACATGTC ATCTGCAAAC CTATTTTTTA ACATCTAGAG   
  
  
+ AGTACTGGAA AATCTAGCTG GGTATTTGTT TTTGTCCAAA AACTTAATTT TAATTTATTT TTATCTAAAG   
  
  
+ ACACCTAGAG AAAACCATTA TGAACTCAAA AAAAACTAAT TCATCAACTT GAAAAAAAAA AACAAATACA   
  
  
+ AAATGGATTA AAAACATAAA ACTAGTTTGG GAAAAAAAAT TTCTCTCTCT AACTAGATCT AAACTTTCCA   
  
  
+ GCAATATAAA TACTTAAAAA TATTTTAATA ACATTTTTTT AACATATTGA TATCAAATTT AACCATTATT   
  
  
+ TTCAATAAAA TTCAACCAAC CAATTTCTGT CTCATCATTC GTGTTCCTAT GATTCTCTCT TTTTCTTTCA   
  
  
+ AACTCTCACA AGGTAAATAA AATTATATTA TGTGCTTGAA AGGAAATTTG AAAAATCTAA AGGAAAACTT   
  
  
+ AACTTAAAAG GTGCTGACAT TTTTTATTAA AAAAAGCTCA TATTGTTTTT TCTTTAATTT TGACTTTATT   
  
  
+ CTTTTCTTTT TCAAATAATA AAATTAGATT CGTTTCCATA AAATTTAATG AACTTTTAGG CAATAATGTT   
  
  
+ GTACGAAGTC AATAATGACG TGCTACAATC GTATGTCACG ACAAAAAGAC GACATGAACA CCATCCATTG   
  
  
+ GTATTCATGT GCCTTTTCAC CTTGTAAAAT AACAGGAGAA AGATACCTTC CAGGGGCAAA TTCATCTATG   
  
  
+ CCACGTGCCT ACAAGTTGAA GGTATGTTTG GTATGCAAG  

- ACATATTATT TTGTTCACGT ATATATAGTA GTACTGTGTT TTTATCTTTA TATCCGGTAT TATAATTTAA   
  
  
- TCCGTAGATA ACGTGGTCGG TACTGTCTGT AAACCAATTA TTATATTATG AGTAACTATT TCCACAGTTA   
  
  
- CTGTGTTTAG ATACTAGGAT CACAACCTTT TAAATTTAAT AAATTTATTG TACCAATCAA CTATATAATA   
  
  
- AAAAGAATAT TAATCCATCT AACTCCCCCC TTTACTATAA AAATATTCAT TATTTATAAC TATTTTGTTC   
  
  
- ATCATCTGTG GACACAGCGT GTACAGATGT GCAACCACAA CCACACGCTG CAAATAATTA CGAATTTACC   
  
  
- ACCAAAGATC CTGATCATAC GCCCAAACCT GTCGTGCACG CTGCAAATTA AAGTAGGAGT AATTTAATCA   
  
  
- CAAATAAAAA AATAAAAAAT AAAAAATAAA AAAATAAAAA AATTAACTTA AATAAAAAAT TAAAATAGTG   
  
  
- AGTAATAAAT TAAATTAAAA TAAACATACA ATTTAAATCA AAAATAAGAA AAATAAAGAT AAACAAAATG   
  
  
- AAATCTTTTT AAAAACTAAT TTTAAAAAAA AAAGTTATAT TAGGAAATCT AAATCAAAAA AAAAGAAAAA   
  
  
- TTAAAATCGA GAGTTGTAAA CTTAACATTC CTACACCAAA CTGAATTTTT TTAAAGTTCT ATTGTACAAA   
  
  
- AATTTATAAC CTTACTGTTG TATAATCTAG TTAGGTTCAG CTGAGTGATC ATTTTTTTTA TTTGAATAAT   
  
  
- TTAGGTGTAA CCAGTACCTA GGTTCATCTA AATTATTAAA AAAATTTGGT CAAAAATAAA TTAATGTACT   
  
  
- ATTTTTTATT CGCTATCATC AATTTGTTGG TTTAATTTAA ATGTTTAACT AATGTTACCT AACATTTTTT   
  
  
- GTGAACGATT ATAATGTTGT TTATAAAGAA AACAAAATAA GTTCTTGTTT CACTTTTACT TTACTTTAAA   
  
  
- CTATTTTGTT ATATTTTTTA TATAGTTAAT TGAATTATAA AAACTTTTGT TATATATTAT ATAAAATATA   
  
  
- AGTAATTAAA AATTCTTTAA TTAAAATTAA TAAACTTCTT TTTATTTTTT TTATCTGGTT CGCGCTCCCG   
  
  
- AACGGTTGGG TCCGGTTGTG AACCCAAAAA ACAACGAAAA ATTTGTTCTG TCTACTGTAC AGCAGATGTC   
  
  
- TCCTCTCACT ATACAGTAGA CATCTCAATC TGTTGTACAG TAGACGTTTG GATAAAAAAT TGTAGATCTC   
  
  
- TCATGACCTT TTAGATCGAC CCATAAACAA AAACAGGTTT TTGAATTAAA ATTAAATAAA AATAGATTTC   
  
  
- TGTGGATCTC TTTTGGTAAT ACTTGAGTTT TTTTTGATTA AGTAGTTGAA CTTTTTTTTT TTGTTTATGT   
  
  
- TTTACCTAAT TTTTGTATTT TGATCAAACC CTTTTTTTTA AAGAGAGAGA TTGATCTAGA TTTGAAAGGT   
  
  
- CGTTATATTT ATGAATTTTT ATAAAATTAT TGTAAAAAAA TTGTATAACT ATAGTTTAAA TTGGTAATAA   
  
  
- AAGTTATTTT AAGTTGGTTG GTTAAAGACA GAGTAGTAAG CACAAGGATA CTAAGAGAGA AAAAGAAAGT   
  
  
- TTGAGAGTGT TCCATTTATT TTAATATAAT ACACGAACTT TCCTTTAAAC TTTTTAGATT TCCTTTTGAA   
  
  
- TTGAATTTTC CACGACTGTA AAAAATAATT TTTTTCGAGT ATAACAAAAA AGAAATTAAA ACTGAAATAA   
  
  
- GAAAAGAAAA AGTTTATTAT TTTAATCTAA GCAAAGGTAT TTTAAATTAC TTGAAAATCC GTTATTACAA   
  
  
- CATGCTTCAG TTATTACTGC ACGATGTTAG CATACAGTGC TGTTTTTCTG CTGTACTTGT GGTAGGTAAC   
  
  
- CATAAGTACA CGGAAAAGTG GAACATTTTA TTGTCCTCTT TCTATGGAAG GTCCCCGTTT AAGTAGATAC   
  
  
- GGTGCACGGA TGTTCAACTT CCATACAAAC CATACGTTC

+     TATA

| Site Name | Organism | Position | Strand | Matrix score. | sequence | function |
| --- | --- | --- | --- | --- | --- | --- |
| TATA | Arabidopsis thaliana | 1042 | - | 8 | TATAAAAT |  |

>Potri.011G061700.1   
+ TGTATAATAA AACAAGTGCA TATATATCAT CATGACACAA AAATAGAAAT ATAGGCCATA ATATTAAATT   
  
  
+ AGGCATCTAT TGCACCAGCC ATGACAGACA TTTGGTTAAT AATATAATAC TCATTGATAA AGGTGTCAAT   
  
  
+ GACACAAATC TATGATCCTA GTGTTGGAAA ATTTAAATTA TTTAAATAAC ATGGTTAGTT GATATATTAT   
  
  
+ TTTTCTTATA ATTAGGTAGA TTGAGGGGGG AAATGATATT TTTATAAGTA ATAAATATTG ATAAAACAAG   
  
  
+ TAGTAGACAC CTGTGTCGCA CATGTCTACA CGTTGGTGTT GGTGTGCGAC GTTTATTAAT GCTTAAATGG   
  
  
+ TGGTTTCTAG GACTAGTATG CGGGTTTGGA CAGCACGTGC GACGTTTAAT TTCATCCTCA TTAAATTAGT   
  
  
+ GTTTATTTTT TTATTTTTTA TTTTTTATTT TTTTATTTTT TTAATTGAAT TTATTTTTTA ATTTTATCAC   
  
  
+ TCATTATTTA ATTTAATTTT ATTTGTATGT TAAATTTAGT TTTTATTCTT TTTATTTCTA TTTGTTTTAC   
  
  
+ TTTAGAAAAA TTTTTGATTA AAATTTTTTT TTTCAATATA ATCCTTTAGA TTTAGTTTTT TTTTCTTTTT   
  
  
+ AATTTTAGCT CTCAACATTT GAATTGTAAG GATGTGGTTT GACTTAAAAA AATTTCAAGA TAACATGTTT   
  
  
+ TTAAATATTG GAATGACAAC ATATTAGATC AATCCAAGTC GACTCACTAG TAAAAAAAAT AAACTTATTA   
  
  
+ AATCCACATT GGTCATGGAT CCAAGTAGAT TTAATAATTT TTTTAAACCA GTTTTTATTT AATTACATGA   
  
  
+ TAAAAAATAA GCGATAGTAG TTAAACAACC AAATTAAATT TACAAATTGA TTACAATGGA TTGTAAAAAA   
  
  
+ CACTTGCTAA TATTACAACA AATATTTCTT TTGTTTTATT CAAGAACAAA GTGAAAATGA AATGAAATTT   
  
  
+ GATAAAACAA TATAAAAAAT ATATCAATTA ACTTAATATT TTTGAAAACA ATATATAATA TATTTTATAT   
  
  
+ TCATTAATTT TTAAGAAATT AATTTTAATT ATTTGAAGAA AAATAAAAAA AATAGACCAA GCGCGAGGGC   
  
  
+ TTGCCAACCC AGGCCAACAC TTGGGTTTTT TGTTGCTTTT TAAACAAGAC AGATGACATG TCGTCTACAG   
  
  
+ AGGAGAGTGA TATGTCATCT GTAGAGTTAG ACAACATGTC ATCTGCAAAC CTATTTTTTA ACATCTAGAG   
  
  
+ AGTACTGGAA AATCTAGCTG GGTATTTGTT TTTGTCCAAA AACTTAATTT TAATTTATTT TTATCTAAAG   
  
  
+ ACACCTAGAG AAAACCATTA TGAACTCAAA AAAAACTAAT TCATCAACTT GAAAAAAAAA AACAAATACA   
  
  
+ AAATGGATTA AAAACATAAA ACTAGTTTGG GAAAAAAAAT TTCTCTCTCT AACTAGATCT AAACTTTCCA   
  
  
+ GCAATATAAA TACTTAAAAA TATTTTAATA ACATTTTTTT AACATATTGA TATCAAATTT AACCATTATT   
  
  
+ TTCAATAAAA TTCAACCAAC CAATTTCTGT CTCATCATTC GTGTTCCTAT GATTCTCTCT TTTTCTTTCA   
  
  
+ AACTCTCACA AGGTAAATAA AATTATATTA TGTGCTTGAA AGGAAATTTG AAAAATCTAA AGGAAAACTT   
  
  
+ AACTTAAAAG GTGCTGACAT TTTTTATTAA AAAAAGCTCA TATTGTTTTT TCTTTAATTT TGACTTTATT   
  
  
+ CTTTTCTTTT TCAAATAATA AAATTAGATT CGTTTCCATA AAATTTAATG AACTTTTAGG CAATAATGTT   
  
  
+ GTACGAAGTC AATAATGACG TGCTACAATC GTATGTCACG ACAAAAAGAC GACATGAACA CCATCCATTG   
  
  
+ GTATTCATGT GCCTTTTCAC CTTGTAAAAT AACAGGAGAA AGATACCTTC CAGGGGCAAA TTCATCTATG   
  
  
+ CCACGTGCCT ACAAGTTGAA GGTATGTTTG GTATGCAAG  

- ACATATTATT TTGTTCACGT ATATATAGTA GTACTGTGTT TTTATCTTTA TATCCGGTAT TATAATTTAA   
  
  
- TCCGTAGATA ACGTGGTCGG TACTGTCTGT AAACCAATTA TTATATTATG AGTAACTATT TCCACAGTTA   
  
  
- CTGTGTTTAG ATACTAGGAT CACAACCTTT TAAATTTAAT AAATTTATTG TACCAATCAA CTATATAATA   
  
  
- AAAAGAATAT TAATCCATCT AACTCCCCCC TTTACTATAA AAATATTCAT TATTTATAAC TATTTTGTTC   
  
  
- ATCATCTGTG GACACAGCGT GTACAGATGT GCAACCACAA CCACACGCTG CAAATAATTA CGAATTTACC   
  
  
- ACCAAAGATC CTGATCATAC GCCCAAACCT GTCGTGCACG CTGCAAATTA AAGTAGGAGT AATTTAATCA   
  
  
- CAAATAAAAA AATAAAAAAT AAAAAATAAA AAAATAAAAA AATTAACTTA AATAAAAAAT TAAAATAGTG   
  
  
- AGTAATAAAT TAAATTAAAA TAAACATACA ATTTAAATCA AAAATAAGAA AAATAAAGAT AAACAAAATG   
  
  
- AAATCTTTTT AAAAACTAAT TTTAAAAAAA AAAGTTATAT TAGGAAATCT AAATCAAAAA AAAAGAAAAA   
  
  
- TTAAAATCGA GAGTTGTAAA CTTAACATTC CTACACCAAA CTGAATTTTT TTAAAGTTCT ATTGTACAAA   
  
  
- AATTTATAAC CTTACTGTTG TATAATCTAG TTAGGTTCAG CTGAGTGATC ATTTTTTTTA TTTGAATAAT   
  
  
- TTAGGTGTAA CCAGTACCTA GGTTCATCTA AATTATTAAA AAAATTTGGT CAAAAATAAA TTAATGTACT   
  
  
- ATTTTTTATT CGCTATCATC AATTTGTTGG TTTAATTTAA ATGTTTAACT AATGTTACCT AACATTTTTT   
  
  
- GTGAACGATT ATAATGTTGT TTATAAAGAA AACAAAATAA GTTCTTGTTT CACTTTTACT TTACTTTAAA   
  
  
- CTATTTTGTT ATATTTTTTA TATAGTTAAT TGAATTATAA AAACTTTTGT TATATATTAT ATAAAATATA   
  
  
- AGTAATTAAA AATTCTTTAA TTAAAATTAA TAAACTTCTT TTTATTTTTT TTATCTGGTT CGCGCTCCCG   
  
  
- AACGGTTGGG TCCGGTTGTG AACCCAAAAA ACAACGAAAA ATTTGTTCTG TCTACTGTAC AGCAGATGTC   
  
  
- TCCTCTCACT ATACAGTAGA CATCTCAATC TGTTGTACAG TAGACGTTTG GATAAAAAAT TGTAGATCTC   
  
  
- TCATGACCTT TTAGATCGAC CCATAAACAA AAACAGGTTT TTGAATTAAA ATTAAATAAA AATAGATTTC   
  
  
- TGTGGATCTC TTTTGGTAAT ACTTGAGTTT TTTTTGATTA AGTAGTTGAA CTTTTTTTTT TTGTTTATGT   
  
  
- TTTACCTAAT TTTTGTATTT TGATCAAACC CTTTTTTTTA AAGAGAGAGA TTGATCTAGA TTTGAAAGGT   
  
  
- CGTTATATTT ATGAATTTTT ATAAAATTAT TGTAAAAAAA TTGTATAACT ATAGTTTAAA TTGGTAATAA   
  
  
- AAGTTATTTT AAGTTGGTTG GTTAAAGACA GAGTAGTAAG CACAAGGATA CTAAGAGAGA AAAAGAAAGT   
  
  
- TTGAGAGTGT TCCATTTATT TTAATATAAT ACACGAACTT TCCTTTAAAC TTTTTAGATT TCCTTTTGAA   
  
  
- TTGAATTTTC CACGACTGTA AAAAATAATT TTTTTCGAGT ATAACAAAAA AGAAATTAAA ACTGAAATAA   
  
  
- GAAAAGAAAA AGTTTATTAT TTTAATCTAA GCAAAGGTAT TTTAAATTAC TTGAAAATCC GTTATTACAA   
  
  
- CATGCTTCAG TTATTACTGC ACGATGTTAG CATACAGTGC TGTTTTTCTG CTGTACTTGT GGTAGGTAAC   
  
  
- CATAAGTACA CGGAAAAGTG GAACATTTTA TTGTCCTCTT TCTATGGAAG GTCCCCGTTT AAGTAGATAC   
  
  
- GGTGCACGGA TGTTCAACTT CCATACAAAC CATACGTTC

+     TATA-box

| Site Name | Organism | Position | Strand | Matrix score. | sequence | function |
| --- | --- | --- | --- | --- | --- | --- |
| TATA-box | Arabidopsis thaliana | 1045 | - | 5 | TATAA | core promoter element around -30 of transcription start |
| TATA-box | Pisum sativum | 1043 | - | 7 | TATAAAA | core promoter element around -30 of transcription start |
| TATA-box | Brassica napus | 999 | - | 6 | ATATAT | core promoter element around -30 of transcription start |
| TATA-box | Arabidopsis thaliana | 700 | - | 8 | TATTTAAA | core promoter element around -30 of transcription start |
| TATA-box | Brassica oleracea | 990 | + | 6 | ATATAA | core promoter element around -30 of transcription start |
| TATA-box | Arabidopsis thaliana | 1000 | - | 4 | TATA | core promoter element around -30 of transcription start |
| TATA-box | Arabidopsis thaliana | 1475 | - | 4 | TATA | core promoter element around -30 of transcription start |
| TATA-box | Brassica oleracea | 1474 | + | 6 | ATATAA | core promoter element around -30 of transcription start |
| TATA-box | Helianthus annuus | 1044 | - | 6 | TATAAA | core promoter element around -30 of transcription start |
| TATA-box | Arabidopsis thaliana | 1633 | - | 5 | TATAA | core promoter element around -30 of transcription start |
| TATA-box | Brassica napus | 1632 | + | 6 | ATTATA | core promoter element around -30 of transcription start |
| TATA-box | Brassica oleracea | 596 | + | 6 | ATATAA | core promoter element around -30 of transcription start |
| TATA-box | Arabidopsis thaliana | 1039 | - | 4 | TATA | core promoter element around -30 of transcription start |
| TATA-box | Arabidopsis thaliana | 253 | + | 4 | TATA | core promoter element around -30 of transcription start |
| TATA-box | Arabidopsis thaliana | 252 | - | 5 | TATAA | core promoter element around -30 of transcription start |
| TATA-box | Brassica oleracea | 112 | + | 6 | ATATAA | core promoter element around -30 of transcription start |
| TATA-box | Pisum sativum | 250 | - | 7 | TATAAAA | core promoter element around -30 of transcription start |
| TATA-box | Helianthus annuus | 251 | - | 6 | TATAAA | core promoter element around -30 of transcription start |
| TATA-box | Arabidopsis thaliana | 249 | - | 9 | ccTATAAAaa | core promoter element around -30 of transcription start |
| TATA-box | Brassica napus | 20 | + | 6 | ATATAT | core promoter element around -30 of transcription start |
| TATA-box | Arabidopsis thaliana | 50 | + | 4 | TATA | core promoter element around -30 of transcription start |
| TATA-box | Avena sativa | 41 | - | 12 | TATATTTATATTT | core promoter element around -30 of transcription start |
| TATA-box | Arabidopsis thaliana | 21 | + | 6 | TATATA | core promoter element around -30 of transcription start |
| TATA-box | Arabidopsis thaliana | 181 | - | 8 | TATTTAAA | core promoter element around -30 of transcription start |
| TATA-box | Arabidopsis thaliana | 3 | + | 4 | TATA | core promoter element around -30 of transcription start |
| TATA-box | Helianthus annuus | 1 | - | 6 | TATACA | core promoter element around -30 of transcription start |
| TATA-box | Brassica napus | 22 | + | 6 | ATATAT | core promoter element around -30 of transcription start |
| TATA-box | Arabidopsis thaliana | 216 | - | 5 | TATAA | core promoter element around -30 of transcription start |
| TATA-box | Zea mays | 213 | - | 8 | TATAAGAA | core promoter element around -30 of transcription start |
| TATA-box | Brassica napus | 202 | + | 6 | ATATAT | core promoter element around -30 of transcription start |
| TATA-box | Arabidopsis thaliana | 991 | + | 4 | TATA | core promoter element around -30 of transcription start |
| TATA-box | Brassica napus | 1031 | - | 6 | ATATAT | core promoter element around -30 of transcription start |
| TATA-box | Arabidopsis thaliana | 1034 | - | 4 | TATA | core promoter element around -30 of transcription start |
| TATA-box | Brassica napus | 1038 | - | 6 | ATATAT | core promoter element around -30 of transcription start |
| TATA-box | Arabidopsis thaliana | 1032 | - | 6 | TATATA | core promoter element around -30 of transcription start |
| TATA-box | Arabidopsis thaliana | 113 | + | 4 | TATA | core promoter element around -30 of transcription start |
| TATA-box | Arabidopsis thaliana | 23 | + | 4 | TATA | core promoter element around -30 of transcription start |
| TATA-box | Arabidopsis thaliana | 1046 | - | 4 | TATA | core promoter element around -30 of transcription start |
| TATA-box | Arabidopsis thaliana | 597 | + | 4 | TATA | core promoter element around -30 of transcription start |
| TATA-box | Oryza sativa | 1397 | + | 7 | TACAAAA | core promoter element around -30 of transcription start |
| TATA-box | Arabidopsis thaliana | 217 | + | 4 | TATA | core promoter element around -30 of transcription start |
| TATA-box | Brassica oleracea | 1033 | + | 6 | ATATAA | core promoter element around -30 of transcription start |
| TATA-box | Arabidopsis thaliana | 179 | + | 8 | TATTTAAA | core promoter element around -30 of transcription start |
| TATA-box | Arabidopsis thaliana | 1634 | - | 4 | TATA | core promoter element around -30 of transcription start |
| TATA-box | Arabidopsis thaliana | 203 | + | 4 | TATA | core promoter element around -30 of transcription start |

>Potri.011G061700.1   
+ TGTATAATAA AACAAGTGCA TATATATCAT CATGACACAA AAATAGAAAT ATAGGCCATA ATATTAAATT   
  
  
+ AGGCATCTAT TGCACCAGCC ATGACAGACA TTTGGTTAAT AATATAATAC TCATTGATAA AGGTGTCAAT   
  
  
+ GACACAAATC TATGATCCTA GTGTTGGAAA ATTTAAATTA TTTAAATAAC ATGGTTAGTT GATATATTAT   
  
  
+ TTTTCTTATA ATTAGGTAGA TTGAGGGGGG AAATGATATT TTTATAAGTA ATAAATATTG ATAAAACAAG   
  
  
+ TAGTAGACAC CTGTGTCGCA CATGTCTACA CGTTGGTGTT GGTGTGCGAC GTTTATTAAT GCTTAAATGG   
  
  
+ TGGTTTCTAG GACTAGTATG CGGGTTTGGA CAGCACGTGC GACGTTTAAT TTCATCCTCA TTAAATTAGT   
  
  
+ GTTTATTTTT TTATTTTTTA TTTTTTATTT TTTTATTTTT TTAATTGAAT TTATTTTTTA ATTTTATCAC   
  
  
+ TCATTATTTA ATTTAATTTT ATTTGTATGT TAAATTTAGT TTTTATTCTT TTTATTTCTA TTTGTTTTAC   
  
  
+ TTTAGAAAAA TTTTTGATTA AAATTTTTTT TTTCAATATA ATCCTTTAGA TTTAGTTTTT TTTTCTTTTT   
  
  
+ AATTTTAGCT CTCAACATTT GAATTGTAAG GATGTGGTTT GACTTAAAAA AATTTCAAGA TAACATGTTT   
  
  
+ TTAAATATTG GAATGACAAC ATATTAGATC AATCCAAGTC GACTCACTAG TAAAAAAAAT AAACTTATTA   
  
  
+ AATCCACATT GGTCATGGAT CCAAGTAGAT TTAATAATTT TTTTAAACCA GTTTTTATTT AATTACATGA   
  
  
+ TAAAAAATAA GCGATAGTAG TTAAACAACC AAATTAAATT TACAAATTGA TTACAATGGA TTGTAAAAAA   
  
  
+ CACTTGCTAA TATTACAACA AATATTTCTT TTGTTTTATT CAAGAACAAA GTGAAAATGA AATGAAATTT   
  
  
+ GATAAAACAA TATAAAAAAT ATATCAATTA ACTTAATATT TTTGAAAACA ATATATAATA TATTTTATAT   
  
  
+ TCATTAATTT TTAAGAAATT AATTTTAATT ATTTGAAGAA AAATAAAAAA AATAGACCAA GCGCGAGGGC   
  
  
+ TTGCCAACCC AGGCCAACAC TTGGGTTTTT TGTTGCTTTT TAAACAAGAC AGATGACATG TCGTCTACAG   
  
  
+ AGGAGAGTGA TATGTCATCT GTAGAGTTAG ACAACATGTC ATCTGCAAAC CTATTTTTTA ACATCTAGAG   
  
  
+ AGTACTGGAA AATCTAGCTG GGTATTTGTT TTTGTCCAAA AACTTAATTT TAATTTATTT TTATCTAAAG   
  
  
+ ACACCTAGAG AAAACCATTA TGAACTCAAA AAAAACTAAT TCATCAACTT GAAAAAAAAA AACAAATACA   
  
  
+ AAATGGATTA AAAACATAAA ACTAGTTTGG GAAAAAAAAT TTCTCTCTCT AACTAGATCT AAACTTTCCA   
  
  
+ GCAATATAAA TACTTAAAAA TATTTTAATA ACATTTTTTT AACATATTGA TATCAAATTT AACCATTATT   
  
  
+ TTCAATAAAA TTCAACCAAC CAATTTCTGT CTCATCATTC GTGTTCCTAT GATTCTCTCT TTTTCTTTCA   
  
  
+ AACTCTCACA AGGTAAATAA AATTATATTA TGTGCTTGAA AGGAAATTTG AAAAATCTAA AGGAAAACTT   
  
  
+ AACTTAAAAG GTGCTGACAT TTTTTATTAA AAAAAGCTCA TATTGTTTTT TCTTTAATTT TGACTTTATT   
  
  
+ CTTTTCTTTT TCAAATAATA AAATTAGATT CGTTTCCATA AAATTTAATG AACTTTTAGG CAATAATGTT   
  
  
+ GTACGAAGTC AATAATGACG TGCTACAATC GTATGTCACG ACAAAAAGAC GACATGAACA CCATCCATTG   
  
  
+ GTATTCATGT GCCTTTTCAC CTTGTAAAAT AACAGGAGAA AGATACCTTC CAGGGGCAAA TTCATCTATG   
  
  
+ CCACGTGCCT ACAAGTTGAA GGTATGTTTG GTATGCAAG  

- ACATATTATT TTGTTCACGT ATATATAGTA GTACTGTGTT TTTATCTTTA TATCCGGTAT TATAATTTAA   
  
  
- TCCGTAGATA ACGTGGTCGG TACTGTCTGT AAACCAATTA TTATATTATG AGTAACTATT TCCACAGTTA   
  
  
- CTGTGTTTAG ATACTAGGAT CACAACCTTT TAAATTTAAT AAATTTATTG TACCAATCAA CTATATAATA   
  
  
- AAAAGAATAT TAATCCATCT AACTCCCCCC TTTACTATAA AAATATTCAT TATTTATAAC TATTTTGTTC   
  
  
- ATCATCTGTG GACACAGCGT GTACAGATGT GCAACCACAA CCACACGCTG CAAATAATTA CGAATTTACC   
  
  
- ACCAAAGATC CTGATCATAC GCCCAAACCT GTCGTGCACG CTGCAAATTA AAGTAGGAGT AATTTAATCA   
  
  
- CAAATAAAAA AATAAAAAAT AAAAAATAAA AAAATAAAAA AATTAACTTA AATAAAAAAT TAAAATAGTG   
  
  
- AGTAATAAAT TAAATTAAAA TAAACATACA ATTTAAATCA AAAATAAGAA AAATAAAGAT AAACAAAATG   
  
  
- AAATCTTTTT AAAAACTAAT TTTAAAAAAA AAAGTTATAT TAGGAAATCT AAATCAAAAA AAAAGAAAAA   
  
  
- TTAAAATCGA GAGTTGTAAA CTTAACATTC CTACACCAAA CTGAATTTTT TTAAAGTTCT ATTGTACAAA   
  
  
- AATTTATAAC CTTACTGTTG TATAATCTAG TTAGGTTCAG CTGAGTGATC ATTTTTTTTA TTTGAATAAT   
  
  
- TTAGGTGTAA CCAGTACCTA GGTTCATCTA AATTATTAAA AAAATTTGGT CAAAAATAAA TTAATGTACT   
  
  
- ATTTTTTATT CGCTATCATC AATTTGTTGG TTTAATTTAA ATGTTTAACT AATGTTACCT AACATTTTTT   
  
  
- GTGAACGATT ATAATGTTGT TTATAAAGAA AACAAAATAA GTTCTTGTTT CACTTTTACT TTACTTTAAA   
  
  
- CTATTTTGTT ATATTTTTTA TATAGTTAAT TGAATTATAA AAACTTTTGT TATATATTAT ATAAAATATA   
  
  
- AGTAATTAAA AATTCTTTAA TTAAAATTAA TAAACTTCTT TTTATTTTTT TTATCTGGTT CGCGCTCCCG   
  
  
- AACGGTTGGG TCCGGTTGTG AACCCAAAAA ACAACGAAAA ATTTGTTCTG TCTACTGTAC AGCAGATGTC   
  
  
- TCCTCTCACT ATACAGTAGA CATCTCAATC TGTTGTACAG TAGACGTTTG GATAAAAAAT TGTAGATCTC   
  
  
- TCATGACCTT TTAGATCGAC CCATAAACAA AAACAGGTTT TTGAATTAAA ATTAAATAAA AATAGATTTC   
  
  
- TGTGGATCTC TTTTGGTAAT ACTTGAGTTT TTTTTGATTA AGTAGTTGAA CTTTTTTTTT TTGTTTATGT   
  
  
- TTTACCTAAT TTTTGTATTT TGATCAAACC CTTTTTTTTA AAGAGAGAGA TTGATCTAGA TTTGAAAGGT   
  
  
- CGTTATATTT ATGAATTTTT ATAAAATTAT TGTAAAAAAA TTGTATAACT ATAGTTTAAA TTGGTAATAA   
  
  
- AAGTTATTTT AAGTTGGTTG GTTAAAGACA GAGTAGTAAG CACAAGGATA CTAAGAGAGA AAAAGAAAGT   
  
  
- TTGAGAGTGT TCCATTTATT TTAATATAAT ACACGAACTT TCCTTTAAAC TTTTTAGATT TCCTTTTGAA   
  
  
- TTGAATTTTC CACGACTGTA AAAAATAATT TTTTTCGAGT ATAACAAAAA AGAAATTAAA ACTGAAATAA   
  
  
- GAAAAGAAAA AGTTTATTAT TTTAATCTAA GCAAAGGTAT TTTAAATTAC TTGAAAATCC GTTATTACAA   
  
  
- CATGCTTCAG TTATTACTGC ACGATGTTAG CATACAGTGC TGTTTTTCTG CTGTACTTGT GGTAGGTAAC   
  
  
- CATAAGTACA CGGAAAAGTG GAACATTTTA TTGTCCTCTT TCTATGGAAG GTCCCCGTTT AAGTAGATAC   
  
  
- GGTGCACGGA TGTTCAACTT CCATACAAAC CATACGTTC

+     TCA

| Site Name | Organism | Position | Strand | Matrix score. | sequence | function |
| --- | --- | --- | --- | --- | --- | --- |
| TCA | Pisum sativum | 402 | + | 9 | TCATCTTCAT |  |

>Potri.011G061700.1   
+ TGTATAATAA AACAAGTGCA TATATATCAT CATGACACAA AAATAGAAAT ATAGGCCATA ATATTAAATT   
  
  
+ AGGCATCTAT TGCACCAGCC ATGACAGACA TTTGGTTAAT AATATAATAC TCATTGATAA AGGTGTCAAT   
  
  
+ GACACAAATC TATGATCCTA GTGTTGGAAA ATTTAAATTA TTTAAATAAC ATGGTTAGTT GATATATTAT   
  
  
+ TTTTCTTATA ATTAGGTAGA TTGAGGGGGG AAATGATATT TTTATAAGTA ATAAATATTG ATAAAACAAG   
  
  
+ TAGTAGACAC CTGTGTCGCA CATGTCTACA CGTTGGTGTT GGTGTGCGAC GTTTATTAAT GCTTAAATGG   
  
  
+ TGGTTTCTAG GACTAGTATG CGGGTTTGGA CAGCACGTGC GACGTTTAAT TTCATCCTCA TTAAATTAGT   
  
  
+ GTTTATTTTT TTATTTTTTA TTTTTTATTT TTTTATTTTT TTAATTGAAT TTATTTTTTA ATTTTATCAC   
  
  
+ TCATTATTTA ATTTAATTTT ATTTGTATGT TAAATTTAGT TTTTATTCTT TTTATTTCTA TTTGTTTTAC   
  
  
+ TTTAGAAAAA TTTTTGATTA AAATTTTTTT TTTCAATATA ATCCTTTAGA TTTAGTTTTT TTTTCTTTTT   
  
  
+ AATTTTAGCT CTCAACATTT GAATTGTAAG GATGTGGTTT GACTTAAAAA AATTTCAAGA TAACATGTTT   
  
  
+ TTAAATATTG GAATGACAAC ATATTAGATC AATCCAAGTC GACTCACTAG TAAAAAAAAT AAACTTATTA   
  
  
+ AATCCACATT GGTCATGGAT CCAAGTAGAT TTAATAATTT TTTTAAACCA GTTTTTATTT AATTACATGA   
  
  
+ TAAAAAATAA GCGATAGTAG TTAAACAACC AAATTAAATT TACAAATTGA TTACAATGGA TTGTAAAAAA   
  
  
+ CACTTGCTAA TATTACAACA AATATTTCTT TTGTTTTATT CAAGAACAAA GTGAAAATGA AATGAAATTT   
  
  
+ GATAAAACAA TATAAAAAAT ATATCAATTA ACTTAATATT TTTGAAAACA ATATATAATA TATTTTATAT   
  
  
+ TCATTAATTT TTAAGAAATT AATTTTAATT ATTTGAAGAA AAATAAAAAA AATAGACCAA GCGCGAGGGC   
  
  
+ TTGCCAACCC AGGCCAACAC TTGGGTTTTT TGTTGCTTTT TAAACAAGAC AGATGACATG TCGTCTACAG   
  
  
+ AGGAGAGTGA TATGTCATCT GTAGAGTTAG ACAACATGTC ATCTGCAAAC CTATTTTTTA ACATCTAGAG   
  
  
+ AGTACTGGAA AATCTAGCTG GGTATTTGTT TTTGTCCAAA AACTTAATTT TAATTTATTT TTATCTAAAG   
  
  
+ ACACCTAGAG AAAACCATTA TGAACTCAAA AAAAACTAAT TCATCAACTT GAAAAAAAAA AACAAATACA   
  
  
+ AAATGGATTA AAAACATAAA ACTAGTTTGG GAAAAAAAAT TTCTCTCTCT AACTAGATCT AAACTTTCCA   
  
  
+ GCAATATAAA TACTTAAAAA TATTTTAATA ACATTTTTTT AACATATTGA TATCAAATTT AACCATTATT   
  
  
+ TTCAATAAAA TTCAACCAAC CAATTTCTGT CTCATCATTC GTGTTCCTAT GATTCTCTCT TTTTCTTTCA   
  
  
+ AACTCTCACA AGGTAAATAA AATTATATTA TGTGCTTGAA AGGAAATTTG AAAAATCTAA AGGAAAACTT   
  
  
+ AACTTAAAAG GTGCTGACAT TTTTTATTAA AAAAAGCTCA TATTGTTTTT TCTTTAATTT TGACTTTATT   
  
  
+ CTTTTCTTTT TCAAATAATA AAATTAGATT CGTTTCCATA AAATTTAATG AACTTTTAGG CAATAATGTT   
  
  
+ GTACGAAGTC AATAATGACG TGCTACAATC GTATGTCACG ACAAAAAGAC GACATGAACA CCATCCATTG   
  
  
+ GTATTCATGT GCCTTTTCAC CTTGTAAAAT AACAGGAGAA AGATACCTTC CAGGGGCAAA TTCATCTATG   
  
  
+ CCACGTGCCT ACAAGTTGAA GGTATGTTTG GTATGCAAG  

- ACATATTATT TTGTTCACGT ATATATAGTA GTACTGTGTT TTTATCTTTA TATCCGGTAT TATAATTTAA   
  
  
- TCCGTAGATA ACGTGGTCGG TACTGTCTGT AAACCAATTA TTATATTATG AGTAACTATT TCCACAGTTA   
  
  
- CTGTGTTTAG ATACTAGGAT CACAACCTTT TAAATTTAAT AAATTTATTG TACCAATCAA CTATATAATA   
  
  
- AAAAGAATAT TAATCCATCT AACTCCCCCC TTTACTATAA AAATATTCAT TATTTATAAC TATTTTGTTC   
  
  
- ATCATCTGTG GACACAGCGT GTACAGATGT GCAACCACAA CCACACGCTG CAAATAATTA CGAATTTACC   
  
  
- ACCAAAGATC CTGATCATAC GCCCAAACCT GTCGTGCACG CTGCAAATTA AAGTAGGAGT AATTTAATCA   
  
  
- CAAATAAAAA AATAAAAAAT AAAAAATAAA AAAATAAAAA AATTAACTTA AATAAAAAAT TAAAATAGTG   
  
  
- AGTAATAAAT TAAATTAAAA TAAACATACA ATTTAAATCA AAAATAAGAA AAATAAAGAT AAACAAAATG   
  
  
- AAATCTTTTT AAAAACTAAT TTTAAAAAAA AAAGTTATAT TAGGAAATCT AAATCAAAAA AAAAGAAAAA   
  
  
- TTAAAATCGA GAGTTGTAAA CTTAACATTC CTACACCAAA CTGAATTTTT TTAAAGTTCT ATTGTACAAA   
  
  
- AATTTATAAC CTTACTGTTG TATAATCTAG TTAGGTTCAG CTGAGTGATC ATTTTTTTTA TTTGAATAAT   
  
  
- TTAGGTGTAA CCAGTACCTA GGTTCATCTA AATTATTAAA AAAATTTGGT CAAAAATAAA TTAATGTACT   
  
  
- ATTTTTTATT CGCTATCATC AATTTGTTGG TTTAATTTAA ATGTTTAACT AATGTTACCT AACATTTTTT   
  
  
- GTGAACGATT ATAATGTTGT TTATAAAGAA AACAAAATAA GTTCTTGTTT CACTTTTACT TTACTTTAAA   
  
  
- CTATTTTGTT ATATTTTTTA TATAGTTAAT TGAATTATAA AAACTTTTGT TATATATTAT ATAAAATATA   
  
  
- AGTAATTAAA AATTCTTTAA TTAAAATTAA TAAACTTCTT TTTATTTTTT TTATCTGGTT CGCGCTCCCG   
  
  
- AACGGTTGGG TCCGGTTGTG AACCCAAAAA ACAACGAAAA ATTTGTTCTG TCTACTGTAC AGCAGATGTC   
  
  
- TCCTCTCACT ATACAGTAGA CATCTCAATC TGTTGTACAG TAGACGTTTG GATAAAAAAT TGTAGATCTC   
  
  
- TCATGACCTT TTAGATCGAC CCATAAACAA AAACAGGTTT TTGAATTAAA ATTAAATAAA AATAGATTTC   
  
  
- TGTGGATCTC TTTTGGTAAT ACTTGAGTTT TTTTTGATTA AGTAGTTGAA CTTTTTTTTT TTGTTTATGT   
  
  
- TTTACCTAAT TTTTGTATTT TGATCAAACC CTTTTTTTTA AAGAGAGAGA TTGATCTAGA TTTGAAAGGT   
  
  
- CGTTATATTT ATGAATTTTT ATAAAATTAT TGTAAAAAAA TTGTATAACT ATAGTTTAAA TTGGTAATAA   
  
  
- AAGTTATTTT AAGTTGGTTG GTTAAAGACA GAGTAGTAAG CACAAGGATA CTAAGAGAGA AAAAGAAAGT   
  
  
- TTGAGAGTGT TCCATTTATT TTAATATAAT ACACGAACTT TCCTTTAAAC TTTTTAGATT TCCTTTTGAA   
  
  
- TTGAATTTTC CACGACTGTA AAAAATAATT TTTTTCGAGT ATAACAAAAA AGAAATTAAA ACTGAAATAA   
  
  
- GAAAAGAAAA AGTTTATTAT TTTAATCTAA GCAAAGGTAT TTTAAATTAC TTGAAAATCC GTTATTACAA   
  
  
- CATGCTTCAG TTATTACTGC ACGATGTTAG CATACAGTGC TGTTTTTCTG CTGTACTTGT GGTAGGTAAC   
  
  
- CATAAGTACA CGGAAAAGTG GAACATTTTA TTGTCCTCTT TCTATGGAAG GTCCCCGTTT AAGTAGATAC   
  
  
- GGTGCACGGA TGTTCAACTT CCATACAAAC CATACGTTC

+     TGACG-motif

| Site Name | Organism | Position | Strand | Matrix score. | sequence | function |
| --- | --- | --- | --- | --- | --- | --- |
| TGACG-motif | Hordeum vulgare | 1836 | + | 5 | TGACG | cis-acting regulatory element involved in the MeJA-responsiveness |

>Potri.011G061700.1   
+ TGTATAATAA AACAAGTGCA TATATATCAT CATGACACAA AAATAGAAAT ATAGGCCATA ATATTAAATT   
  
  
+ AGGCATCTAT TGCACCAGCC ATGACAGACA TTTGGTTAAT AATATAATAC TCATTGATAA AGGTGTCAAT   
  
  
+ GACACAAATC TATGATCCTA GTGTTGGAAA ATTTAAATTA TTTAAATAAC ATGGTTAGTT GATATATTAT   
  
  
+ TTTTCTTATA ATTAGGTAGA TTGAGGGGGG AAATGATATT TTTATAAGTA ATAAATATTG ATAAAACAAG   
  
  
+ TAGTAGACAC CTGTGTCGCA CATGTCTACA CGTTGGTGTT GGTGTGCGAC GTTTATTAAT GCTTAAATGG   
  
  
+ TGGTTTCTAG GACTAGTATG CGGGTTTGGA CAGCACGTGC GACGTTTAAT TTCATCCTCA TTAAATTAGT   
  
  
+ GTTTATTTTT TTATTTTTTA TTTTTTATTT TTTTATTTTT TTAATTGAAT TTATTTTTTA ATTTTATCAC   
  
  
+ TCATTATTTA ATTTAATTTT ATTTGTATGT TAAATTTAGT TTTTATTCTT TTTATTTCTA TTTGTTTTAC   
  
  
+ TTTAGAAAAA TTTTTGATTA AAATTTTTTT TTTCAATATA ATCCTTTAGA TTTAGTTTTT TTTTCTTTTT   
  
  
+ AATTTTAGCT CTCAACATTT GAATTGTAAG GATGTGGTTT GACTTAAAAA AATTTCAAGA TAACATGTTT   
  
  
+ TTAAATATTG GAATGACAAC ATATTAGATC AATCCAAGTC GACTCACTAG TAAAAAAAAT AAACTTATTA   
  
  
+ AATCCACATT GGTCATGGAT CCAAGTAGAT TTAATAATTT TTTTAAACCA GTTTTTATTT AATTACATGA   
  
  
+ TAAAAAATAA GCGATAGTAG TTAAACAACC AAATTAAATT TACAAATTGA TTACAATGGA TTGTAAAAAA   
  
  
+ CACTTGCTAA TATTACAACA AATATTTCTT TTGTTTTATT CAAGAACAAA GTGAAAATGA AATGAAATTT   
  
  
+ GATAAAACAA TATAAAAAAT ATATCAATTA ACTTAATATT TTTGAAAACA ATATATAATA TATTTTATAT   
  
  
+ TCATTAATTT TTAAGAAATT AATTTTAATT ATTTGAAGAA AAATAAAAAA AATAGACCAA GCGCGAGGGC   
  
  
+ TTGCCAACCC AGGCCAACAC TTGGGTTTTT TGTTGCTTTT TAAACAAGAC AGATGACATG TCGTCTACAG   
  
  
+ AGGAGAGTGA TATGTCATCT GTAGAGTTAG ACAACATGTC ATCTGCAAAC CTATTTTTTA ACATCTAGAG   
  
  
+ AGTACTGGAA AATCTAGCTG GGTATTTGTT TTTGTCCAAA AACTTAATTT TAATTTATTT TTATCTAAAG   
  
  
+ ACACCTAGAG AAAACCATTA TGAACTCAAA AAAAACTAAT TCATCAACTT GAAAAAAAAA AACAAATACA   
  
  
+ AAATGGATTA AAAACATAAA ACTAGTTTGG GAAAAAAAAT TTCTCTCTCT AACTAGATCT AAACTTTCCA   
  
  
+ GCAATATAAA TACTTAAAAA TATTTTAATA ACATTTTTTT AACATATTGA TATCAAATTT AACCATTATT   
  
  
+ TTCAATAAAA TTCAACCAAC CAATTTCTGT CTCATCATTC GTGTTCCTAT GATTCTCTCT TTTTCTTTCA   
  
  
+ AACTCTCACA AGGTAAATAA AATTATATTA TGTGCTTGAA AGGAAATTTG AAAAATCTAA AGGAAAACTT   
  
  
+ AACTTAAAAG GTGCTGACAT TTTTTATTAA AAAAAGCTCA TATTGTTTTT TCTTTAATTT TGACTTTATT   
  
  
+ CTTTTCTTTT TCAAATAATA AAATTAGATT CGTTTCCATA AAATTTAATG AACTTTTAGG CAATAATGTT   
  
  
+ GTACGAAGTC AATAATGACG TGCTACAATC GTATGTCACG ACAAAAAGAC GACATGAACA CCATCCATTG   
  
  
+ GTATTCATGT GCCTTTTCAC CTTGTAAAAT AACAGGAGAA AGATACCTTC CAGGGGCAAA TTCATCTATG   
  
  
+ CCACGTGCCT ACAAGTTGAA GGTATGTTTG GTATGCAAG  

- ACATATTATT TTGTTCACGT ATATATAGTA GTACTGTGTT TTTATCTTTA TATCCGGTAT TATAATTTAA   
  
  
- TCCGTAGATA ACGTGGTCGG TACTGTCTGT AAACCAATTA TTATATTATG AGTAACTATT TCCACAGTTA   
  
  
- CTGTGTTTAG ATACTAGGAT CACAACCTTT TAAATTTAAT AAATTTATTG TACCAATCAA CTATATAATA   
  
  
- AAAAGAATAT TAATCCATCT AACTCCCCCC TTTACTATAA AAATATTCAT TATTTATAAC TATTTTGTTC   
  
  
- ATCATCTGTG GACACAGCGT GTACAGATGT GCAACCACAA CCACACGCTG CAAATAATTA CGAATTTACC   
  
  
- ACCAAAGATC CTGATCATAC GCCCAAACCT GTCGTGCACG CTGCAAATTA AAGTAGGAGT AATTTAATCA   
  
  
- CAAATAAAAA AATAAAAAAT AAAAAATAAA AAAATAAAAA AATTAACTTA AATAAAAAAT TAAAATAGTG   
  
  
- AGTAATAAAT TAAATTAAAA TAAACATACA ATTTAAATCA AAAATAAGAA AAATAAAGAT AAACAAAATG   
  
  
- AAATCTTTTT AAAAACTAAT TTTAAAAAAA AAAGTTATAT TAGGAAATCT AAATCAAAAA AAAAGAAAAA   
  
  
- TTAAAATCGA GAGTTGTAAA CTTAACATTC CTACACCAAA CTGAATTTTT TTAAAGTTCT ATTGTACAAA   
  
  
- AATTTATAAC CTTACTGTTG TATAATCTAG TTAGGTTCAG CTGAGTGATC ATTTTTTTTA TTTGAATAAT   
  
  
- TTAGGTGTAA CCAGTACCTA GGTTCATCTA AATTATTAAA AAAATTTGGT CAAAAATAAA TTAATGTACT   
  
  
- ATTTTTTATT CGCTATCATC AATTTGTTGG TTTAATTTAA ATGTTTAACT AATGTTACCT AACATTTTTT   
  
  
- GTGAACGATT ATAATGTTGT TTATAAAGAA AACAAAATAA GTTCTTGTTT CACTTTTACT TTACTTTAAA   
  
  
- CTATTTTGTT ATATTTTTTA TATAGTTAAT TGAATTATAA AAACTTTTGT TATATATTAT ATAAAATATA   
  
  
- AGTAATTAAA AATTCTTTAA TTAAAATTAA TAAACTTCTT TTTATTTTTT TTATCTGGTT CGCGCTCCCG   
  
  
- AACGGTTGGG TCCGGTTGTG AACCCAAAAA ACAACGAAAA ATTTGTTCTG TCTACTGTAC AGCAGATGTC   
  
  
- TCCTCTCACT ATACAGTAGA CATCTCAATC TGTTGTACAG TAGACGTTTG GATAAAAAAT TGTAGATCTC   
  
  
- TCATGACCTT TTAGATCGAC CCATAAACAA AAACAGGTTT TTGAATTAAA ATTAAATAAA AATAGATTTC   
  
  
- TGTGGATCTC TTTTGGTAAT ACTTGAGTTT TTTTTGATTA AGTAGTTGAA CTTTTTTTTT TTGTTTATGT   
  
  
- TTTACCTAAT TTTTGTATTT TGATCAAACC CTTTTTTTTA AAGAGAGAGA TTGATCTAGA TTTGAAAGGT   
  
  
- CGTTATATTT ATGAATTTTT ATAAAATTAT TGTAAAAAAA TTGTATAACT ATAGTTTAAA TTGGTAATAA   
  
  
- AAGTTATTTT AAGTTGGTTG GTTAAAGACA GAGTAGTAAG CACAAGGATA CTAAGAGAGA AAAAGAAAGT   
  
  
- TTGAGAGTGT TCCATTTATT TTAATATAAT ACACGAACTT TCCTTTAAAC TTTTTAGATT TCCTTTTGAA   
  
  
- TTGAATTTTC CACGACTGTA AAAAATAATT TTTTTCGAGT ATAACAAAAA AGAAATTAAA ACTGAAATAA   
  
  
- GAAAAGAAAA AGTTTATTAT TTTAATCTAA GCAAAGGTAT TTTAAATTAC TTGAAAATCC GTTATTACAA   
  
  
- CATGCTTCAG TTATTACTGC ACGATGTTAG CATACAGTGC TGTTTTTCTG CTGTACTTGT GGTAGGTAAC   
  
  
- CATAAGTACA CGGAAAAGTG GAACATTTTA TTGTCCTCTT TCTATGGAAG GTCCCCGTTT AAGTAGATAC   
  
  
- GGTGCACGGA TGTTCAACTT CCATACAAAC CATACGTTC

+     Unnamed\_\_1

| Site Name | Organism | Position | Strand | Matrix score. | sequence | function |
| --- | --- | --- | --- | --- | --- | --- |
| Unnamed\_\_1 | Zea mays | 1961 | - | 5 | CGTGG |  |
| Unnamed\_\_1 | Petunia sp. | 1960 | - | 9 | GCCACGTGGC |  |

>Potri.011G061700.1   
+ TGTATAATAA AACAAGTGCA TATATATCAT CATGACACAA AAATAGAAAT ATAGGCCATA ATATTAAATT   
  
  
+ AGGCATCTAT TGCACCAGCC ATGACAGACA TTTGGTTAAT AATATAATAC TCATTGATAA AGGTGTCAAT   
  
  
+ GACACAAATC TATGATCCTA GTGTTGGAAA ATTTAAATTA TTTAAATAAC ATGGTTAGTT GATATATTAT   
  
  
+ TTTTCTTATA ATTAGGTAGA TTGAGGGGGG AAATGATATT TTTATAAGTA ATAAATATTG ATAAAACAAG   
  
  
+ TAGTAGACAC CTGTGTCGCA CATGTCTACA CGTTGGTGTT GGTGTGCGAC GTTTATTAAT GCTTAAATGG   
  
  
+ TGGTTTCTAG GACTAGTATG CGGGTTTGGA CAGCACGTGC GACGTTTAAT TTCATCCTCA TTAAATTAGT   
  
  
+ GTTTATTTTT TTATTTTTTA TTTTTTATTT TTTTATTTTT TTAATTGAAT TTATTTTTTA ATTTTATCAC   
  
  
+ TCATTATTTA ATTTAATTTT ATTTGTATGT TAAATTTAGT TTTTATTCTT TTTATTTCTA TTTGTTTTAC   
  
  
+ TTTAGAAAAA TTTTTGATTA AAATTTTTTT TTTCAATATA ATCCTTTAGA TTTAGTTTTT TTTTCTTTTT   
  
  
+ AATTTTAGCT CTCAACATTT GAATTGTAAG GATGTGGTTT GACTTAAAAA AATTTCAAGA TAACATGTTT   
  
  
+ TTAAATATTG GAATGACAAC ATATTAGATC AATCCAAGTC GACTCACTAG TAAAAAAAAT AAACTTATTA   
  
  
+ AATCCACATT GGTCATGGAT CCAAGTAGAT TTAATAATTT TTTTAAACCA GTTTTTATTT AATTACATGA   
  
  
+ TAAAAAATAA GCGATAGTAG TTAAACAACC AAATTAAATT TACAAATTGA TTACAATGGA TTGTAAAAAA   
  
  
+ CACTTGCTAA TATTACAACA AATATTTCTT TTGTTTTATT CAAGAACAAA GTGAAAATGA AATGAAATTT   
  
  
+ GATAAAACAA TATAAAAAAT ATATCAATTA ACTTAATATT TTTGAAAACA ATATATAATA TATTTTATAT   
  
  
+ TCATTAATTT TTAAGAAATT AATTTTAATT ATTTGAAGAA AAATAAAAAA AATAGACCAA GCGCGAGGGC   
  
  
+ TTGCCAACCC AGGCCAACAC TTGGGTTTTT TGTTGCTTTT TAAACAAGAC AGATGACATG TCGTCTACAG   
  
  
+ AGGAGAGTGA TATGTCATCT GTAGAGTTAG ACAACATGTC ATCTGCAAAC CTATTTTTTA ACATCTAGAG   
  
  
+ AGTACTGGAA AATCTAGCTG GGTATTTGTT TTTGTCCAAA AACTTAATTT TAATTTATTT TTATCTAAAG   
  
  
+ ACACCTAGAG AAAACCATTA TGAACTCAAA AAAAACTAAT TCATCAACTT GAAAAAAAAA AACAAATACA   
  
  
+ AAATGGATTA AAAACATAAA ACTAGTTTGG GAAAAAAAAT TTCTCTCTCT AACTAGATCT AAACTTTCCA   
  
  
+ GCAATATAAA TACTTAAAAA TATTTTAATA ACATTTTTTT AACATATTGA TATCAAATTT AACCATTATT   
  
  
+ TTCAATAAAA TTCAACCAAC CAATTTCTGT CTCATCATTC GTGTTCCTAT GATTCTCTCT TTTTCTTTCA   
  
  
+ AACTCTCACA AGGTAAATAA AATTATATTA TGTGCTTGAA AGGAAATTTG AAAAATCTAA AGGAAAACTT   
  
  
+ AACTTAAAAG GTGCTGACAT TTTTTATTAA AAAAAGCTCA TATTGTTTTT TCTTTAATTT TGACTTTATT   
  
  
+ CTTTTCTTTT TCAAATAATA AAATTAGATT CGTTTCCATA AAATTTAATG AACTTTTAGG CAATAATGTT   
  
  
+ GTACGAAGTC AATAATGACG TGCTACAATC GTATGTCACG ACAAAAAGAC GACATGAACA CCATCCATTG   
  
  
+ GTATTCATGT GCCTTTTCAC CTTGTAAAAT AACAGGAGAA AGATACCTTC CAGGGGCAAA TTCATCTATG   
  
  
+ CCACGTGCCT ACAAGTTGAA GGTATGTTTG GTATGCAAG  

- ACATATTATT TTGTTCACGT ATATATAGTA GTACTGTGTT TTTATCTTTA TATCCGGTAT TATAATTTAA   
  
  
- TCCGTAGATA ACGTGGTCGG TACTGTCTGT AAACCAATTA TTATATTATG AGTAACTATT TCCACAGTTA   
  
  
- CTGTGTTTAG ATACTAGGAT CACAACCTTT TAAATTTAAT AAATTTATTG TACCAATCAA CTATATAATA   
  
  
- AAAAGAATAT TAATCCATCT AACTCCCCCC TTTACTATAA AAATATTCAT TATTTATAAC TATTTTGTTC   
  
  
- ATCATCTGTG GACACAGCGT GTACAGATGT GCAACCACAA CCACACGCTG CAAATAATTA CGAATTTACC   
  
  
- ACCAAAGATC CTGATCATAC GCCCAAACCT GTCGTGCACG CTGCAAATTA AAGTAGGAGT AATTTAATCA   
  
  
- CAAATAAAAA AATAAAAAAT AAAAAATAAA AAAATAAAAA AATTAACTTA AATAAAAAAT TAAAATAGTG   
  
  
- AGTAATAAAT TAAATTAAAA TAAACATACA ATTTAAATCA AAAATAAGAA AAATAAAGAT AAACAAAATG   
  
  
- AAATCTTTTT AAAAACTAAT TTTAAAAAAA AAAGTTATAT TAGGAAATCT AAATCAAAAA AAAAGAAAAA   
  
  
- TTAAAATCGA GAGTTGTAAA CTTAACATTC CTACACCAAA CTGAATTTTT TTAAAGTTCT ATTGTACAAA   
  
  
- AATTTATAAC CTTACTGTTG TATAATCTAG TTAGGTTCAG CTGAGTGATC ATTTTTTTTA TTTGAATAAT   
  
  
- TTAGGTGTAA CCAGTACCTA GGTTCATCTA AATTATTAAA AAAATTTGGT CAAAAATAAA TTAATGTACT   
  
  
- ATTTTTTATT CGCTATCATC AATTTGTTGG TTTAATTTAA ATGTTTAACT AATGTTACCT AACATTTTTT   
  
  
- GTGAACGATT ATAATGTTGT TTATAAAGAA AACAAAATAA GTTCTTGTTT CACTTTTACT TTACTTTAAA   
  
  
- CTATTTTGTT ATATTTTTTA TATAGTTAAT TGAATTATAA AAACTTTTGT TATATATTAT ATAAAATATA   
  
  
- AGTAATTAAA AATTCTTTAA TTAAAATTAA TAAACTTCTT TTTATTTTTT TTATCTGGTT CGCGCTCCCG   
  
  
- AACGGTTGGG TCCGGTTGTG AACCCAAAAA ACAACGAAAA ATTTGTTCTG TCTACTGTAC AGCAGATGTC   
  
  
- TCCTCTCACT ATACAGTAGA CATCTCAATC TGTTGTACAG TAGACGTTTG GATAAAAAAT TGTAGATCTC   
  
  
- TCATGACCTT TTAGATCGAC CCATAAACAA AAACAGGTTT TTGAATTAAA ATTAAATAAA AATAGATTTC   
  
  
- TGTGGATCTC TTTTGGTAAT ACTTGAGTTT TTTTTGATTA AGTAGTTGAA CTTTTTTTTT TTGTTTATGT   
  
  
- TTTACCTAAT TTTTGTATTT TGATCAAACC CTTTTTTTTA AAGAGAGAGA TTGATCTAGA TTTGAAAGGT   
  
  
- CGTTATATTT ATGAATTTTT ATAAAATTAT TGTAAAAAAA TTGTATAACT ATAGTTTAAA TTGGTAATAA   
  
  
- AAGTTATTTT AAGTTGGTTG GTTAAAGACA GAGTAGTAAG CACAAGGATA CTAAGAGAGA AAAAGAAAGT   
  
  
- TTGAGAGTGT TCCATTTATT TTAATATAAT ACACGAACTT TCCTTTAAAC TTTTTAGATT TCCTTTTGAA   
  
  
- TTGAATTTTC CACGACTGTA AAAAATAATT TTTTTCGAGT ATAACAAAAA AGAAATTAAA ACTGAAATAA   
  
  
- GAAAAGAAAA AGTTTATTAT TTTAATCTAA GCAAAGGTAT TTTAAATTAC TTGAAAATCC GTTATTACAA   
  
  
- CATGCTTCAG TTATTACTGC ACGATGTTAG CATACAGTGC TGTTTTTCTG CTGTACTTGT GGTAGGTAAC   
  
  
- CATAAGTACA CGGAAAAGTG GAACATTTTA TTGTCCTCTT TCTATGGAAG GTCCCCGTTT AAGTAGATAC   
  
  
- GGTGCACGGA TGTTCAACTT CCATACAAAC CATACGTTC

+     Unnamed\_\_10

| Site Name | Organism | Position | Strand | Matrix score. | sequence | function |
| --- | --- | --- | --- | --- | --- | --- |
| Unnamed\_\_10 | Zea mays | 790 | + | 9 | TCCACGTAGA |  |

>Potri.011G061700.1   
+ TGTATAATAA AACAAGTGCA TATATATCAT CATGACACAA AAATAGAAAT ATAGGCCATA ATATTAAATT   
  
  
+ AGGCATCTAT TGCACCAGCC ATGACAGACA TTTGGTTAAT AATATAATAC TCATTGATAA AGGTGTCAAT   
  
  
+ GACACAAATC TATGATCCTA GTGTTGGAAA ATTTAAATTA TTTAAATAAC ATGGTTAGTT GATATATTAT   
  
  
+ TTTTCTTATA ATTAGGTAGA TTGAGGGGGG AAATGATATT TTTATAAGTA ATAAATATTG ATAAAACAAG   
  
  
+ TAGTAGACAC CTGTGTCGCA CATGTCTACA CGTTGGTGTT GGTGTGCGAC GTTTATTAAT GCTTAAATGG   
  
  
+ TGGTTTCTAG GACTAGTATG CGGGTTTGGA CAGCACGTGC GACGTTTAAT TTCATCCTCA TTAAATTAGT   
  
  
+ GTTTATTTTT TTATTTTTTA TTTTTTATTT TTTTATTTTT TTAATTGAAT TTATTTTTTA ATTTTATCAC   
  
  
+ TCATTATTTA ATTTAATTTT ATTTGTATGT TAAATTTAGT TTTTATTCTT TTTATTTCTA TTTGTTTTAC   
  
  
+ TTTAGAAAAA TTTTTGATTA AAATTTTTTT TTTCAATATA ATCCTTTAGA TTTAGTTTTT TTTTCTTTTT   
  
  
+ AATTTTAGCT CTCAACATTT GAATTGTAAG GATGTGGTTT GACTTAAAAA AATTTCAAGA TAACATGTTT   
  
  
+ TTAAATATTG GAATGACAAC ATATTAGATC AATCCAAGTC GACTCACTAG TAAAAAAAAT AAACTTATTA   
  
  
+ AATCCACATT GGTCATGGAT CCAAGTAGAT TTAATAATTT TTTTAAACCA GTTTTTATTT AATTACATGA   
  
  
+ TAAAAAATAA GCGATAGTAG TTAAACAACC AAATTAAATT TACAAATTGA TTACAATGGA TTGTAAAAAA   
  
  
+ CACTTGCTAA TATTACAACA AATATTTCTT TTGTTTTATT CAAGAACAAA GTGAAAATGA AATGAAATTT   
  
  
+ GATAAAACAA TATAAAAAAT ATATCAATTA ACTTAATATT TTTGAAAACA ATATATAATA TATTTTATAT   
  
  
+ TCATTAATTT TTAAGAAATT AATTTTAATT ATTTGAAGAA AAATAAAAAA AATAGACCAA GCGCGAGGGC   
  
  
+ TTGCCAACCC AGGCCAACAC TTGGGTTTTT TGTTGCTTTT TAAACAAGAC AGATGACATG TCGTCTACAG   
  
  
+ AGGAGAGTGA TATGTCATCT GTAGAGTTAG ACAACATGTC ATCTGCAAAC CTATTTTTTA ACATCTAGAG   
  
  
+ AGTACTGGAA AATCTAGCTG GGTATTTGTT TTTGTCCAAA AACTTAATTT TAATTTATTT TTATCTAAAG   
  
  
+ ACACCTAGAG AAAACCATTA TGAACTCAAA AAAAACTAAT TCATCAACTT GAAAAAAAAA AACAAATACA   
  
  
+ AAATGGATTA AAAACATAAA ACTAGTTTGG GAAAAAAAAT TTCTCTCTCT AACTAGATCT AAACTTTCCA   
  
  
+ GCAATATAAA TACTTAAAAA TATTTTAATA ACATTTTTTT AACATATTGA TATCAAATTT AACCATTATT   
  
  
+ TTCAATAAAA TTCAACCAAC CAATTTCTGT CTCATCATTC GTGTTCCTAT GATTCTCTCT TTTTCTTTCA   
  
  
+ AACTCTCACA AGGTAAATAA AATTATATTA TGTGCTTGAA AGGAAATTTG AAAAATCTAA AGGAAAACTT   
  
  
+ AACTTAAAAG GTGCTGACAT TTTTTATTAA AAAAAGCTCA TATTGTTTTT TCTTTAATTT TGACTTTATT   
  
  
+ CTTTTCTTTT TCAAATAATA AAATTAGATT CGTTTCCATA AAATTTAATG AACTTTTAGG CAATAATGTT   
  
  
+ GTACGAAGTC AATAATGACG TGCTACAATC GTATGTCACG ACAAAAAGAC GACATGAACA CCATCCATTG   
  
  
+ GTATTCATGT GCCTTTTCAC CTTGTAAAAT AACAGGAGAA AGATACCTTC CAGGGGCAAA TTCATCTATG   
  
  
+ CCACGTGCCT ACAAGTTGAA GGTATGTTTG GTATGCAAG  

- ACATATTATT TTGTTCACGT ATATATAGTA GTACTGTGTT TTTATCTTTA TATCCGGTAT TATAATTTAA   
  
  
- TCCGTAGATA ACGTGGTCGG TACTGTCTGT AAACCAATTA TTATATTATG AGTAACTATT TCCACAGTTA   
  
  
- CTGTGTTTAG ATACTAGGAT CACAACCTTT TAAATTTAAT AAATTTATTG TACCAATCAA CTATATAATA   
  
  
- AAAAGAATAT TAATCCATCT AACTCCCCCC TTTACTATAA AAATATTCAT TATTTATAAC TATTTTGTTC   
  
  
- ATCATCTGTG GACACAGCGT GTACAGATGT GCAACCACAA CCACACGCTG CAAATAATTA CGAATTTACC   
  
  
- ACCAAAGATC CTGATCATAC GCCCAAACCT GTCGTGCACG CTGCAAATTA AAGTAGGAGT AATTTAATCA   
  
  
- CAAATAAAAA AATAAAAAAT AAAAAATAAA AAAATAAAAA AATTAACTTA AATAAAAAAT TAAAATAGTG   
  
  
- AGTAATAAAT TAAATTAAAA TAAACATACA ATTTAAATCA AAAATAAGAA AAATAAAGAT AAACAAAATG   
  
  
- AAATCTTTTT AAAAACTAAT TTTAAAAAAA AAAGTTATAT TAGGAAATCT AAATCAAAAA AAAAGAAAAA   
  
  
- TTAAAATCGA GAGTTGTAAA CTTAACATTC CTACACCAAA CTGAATTTTT TTAAAGTTCT ATTGTACAAA   
  
  
- AATTTATAAC CTTACTGTTG TATAATCTAG TTAGGTTCAG CTGAGTGATC ATTTTTTTTA TTTGAATAAT   
  
  
- TTAGGTGTAA CCAGTACCTA GGTTCATCTA AATTATTAAA AAAATTTGGT CAAAAATAAA TTAATGTACT   
  
  
- ATTTTTTATT CGCTATCATC AATTTGTTGG TTTAATTTAA ATGTTTAACT AATGTTACCT AACATTTTTT   
  
  
- GTGAACGATT ATAATGTTGT TTATAAAGAA AACAAAATAA GTTCTTGTTT CACTTTTACT TTACTTTAAA   
  
  
- CTATTTTGTT ATATTTTTTA TATAGTTAAT TGAATTATAA AAACTTTTGT TATATATTAT ATAAAATATA   
  
  
- AGTAATTAAA AATTCTTTAA TTAAAATTAA TAAACTTCTT TTTATTTTTT TTATCTGGTT CGCGCTCCCG   
  
  
- AACGGTTGGG TCCGGTTGTG AACCCAAAAA ACAACGAAAA ATTTGTTCTG TCTACTGTAC AGCAGATGTC   
  
  
- TCCTCTCACT ATACAGTAGA CATCTCAATC TGTTGTACAG TAGACGTTTG GATAAAAAAT TGTAGATCTC   
  
  
- TCATGACCTT TTAGATCGAC CCATAAACAA AAACAGGTTT TTGAATTAAA ATTAAATAAA AATAGATTTC   
  
  
- TGTGGATCTC TTTTGGTAAT ACTTGAGTTT TTTTTGATTA AGTAGTTGAA CTTTTTTTTT TTGTTTATGT   
  
  
- TTTACCTAAT TTTTGTATTT TGATCAAACC CTTTTTTTTA AAGAGAGAGA TTGATCTAGA TTTGAAAGGT   
  
  
- CGTTATATTT ATGAATTTTT ATAAAATTAT TGTAAAAAAA TTGTATAACT ATAGTTTAAA TTGGTAATAA   
  
  
- AAGTTATTTT AAGTTGGTTG GTTAAAGACA GAGTAGTAAG CACAAGGATA CTAAGAGAGA AAAAGAAAGT   
  
  
- TTGAGAGTGT TCCATTTATT TTAATATAAT ACACGAACTT TCCTTTAAAC TTTTTAGATT TCCTTTTGAA   
  
  
- TTGAATTTTC CACGACTGTA AAAAATAATT TTTTTCGAGT ATAACAAAAA AGAAATTAAA ACTGAAATAA   
  
  
- GAAAAGAAAA AGTTTATTAT TTTAATCTAA GCAAAGGTAT TTTAAATTAC TTGAAAATCC GTTATTACAA   
  
  
- CATGCTTCAG TTATTACTGC ACGATGTTAG CATACAGTGC TGTTTTTCTG CTGTACTTGT GGTAGGTAAC   
  
  
- CATAAGTACA CGGAAAAGTG GAACATTTTA TTGTCCTCTT TCTATGGAAG GTCCCCGTTT AAGTAGATAC   
  
  
- GGTGCACGGA TGTTCAACTT CCATACAAAC CATACGTTC

+     Unnamed\_\_12

| Site Name | Organism | Position | Strand | Matrix score. | sequence | function |
| --- | --- | --- | --- | --- | --- | --- |
| Unnamed\_\_12 | Zea mays | 790 | + | 9 | TCCACGTAGA |  |

>Potri.011G061700.1   
+ TGTATAATAA AACAAGTGCA TATATATCAT CATGACACAA AAATAGAAAT ATAGGCCATA ATATTAAATT   
  
  
+ AGGCATCTAT TGCACCAGCC ATGACAGACA TTTGGTTAAT AATATAATAC TCATTGATAA AGGTGTCAAT   
  
  
+ GACACAAATC TATGATCCTA GTGTTGGAAA ATTTAAATTA TTTAAATAAC ATGGTTAGTT GATATATTAT   
  
  
+ TTTTCTTATA ATTAGGTAGA TTGAGGGGGG AAATGATATT TTTATAAGTA ATAAATATTG ATAAAACAAG   
  
  
+ TAGTAGACAC CTGTGTCGCA CATGTCTACA CGTTGGTGTT GGTGTGCGAC GTTTATTAAT GCTTAAATGG   
  
  
+ TGGTTTCTAG GACTAGTATG CGGGTTTGGA CAGCACGTGC GACGTTTAAT TTCATCCTCA TTAAATTAGT   
  
  
+ GTTTATTTTT TTATTTTTTA TTTTTTATTT TTTTATTTTT TTAATTGAAT TTATTTTTTA ATTTTATCAC   
  
  
+ TCATTATTTA ATTTAATTTT ATTTGTATGT TAAATTTAGT TTTTATTCTT TTTATTTCTA TTTGTTTTAC   
  
  
+ TTTAGAAAAA TTTTTGATTA AAATTTTTTT TTTCAATATA ATCCTTTAGA TTTAGTTTTT TTTTCTTTTT   
  
  
+ AATTTTAGCT CTCAACATTT GAATTGTAAG GATGTGGTTT GACTTAAAAA AATTTCAAGA TAACATGTTT   
  
  
+ TTAAATATTG GAATGACAAC ATATTAGATC AATCCAAGTC GACTCACTAG TAAAAAAAAT AAACTTATTA   
  
  
+ AATCCACATT GGTCATGGAT CCAAGTAGAT TTAATAATTT TTTTAAACCA GTTTTTATTT AATTACATGA   
  
  
+ TAAAAAATAA GCGATAGTAG TTAAACAACC AAATTAAATT TACAAATTGA TTACAATGGA TTGTAAAAAA   
  
  
+ CACTTGCTAA TATTACAACA AATATTTCTT TTGTTTTATT CAAGAACAAA GTGAAAATGA AATGAAATTT   
  
  
+ GATAAAACAA TATAAAAAAT ATATCAATTA ACTTAATATT TTTGAAAACA ATATATAATA TATTTTATAT   
  
  
+ TCATTAATTT TTAAGAAATT AATTTTAATT ATTTGAAGAA AAATAAAAAA AATAGACCAA GCGCGAGGGC   
  
  
+ TTGCCAACCC AGGCCAACAC TTGGGTTTTT TGTTGCTTTT TAAACAAGAC AGATGACATG TCGTCTACAG   
  
  
+ AGGAGAGTGA TATGTCATCT GTAGAGTTAG ACAACATGTC ATCTGCAAAC CTATTTTTTA ACATCTAGAG   
  
  
+ AGTACTGGAA AATCTAGCTG GGTATTTGTT TTTGTCCAAA AACTTAATTT TAATTTATTT TTATCTAAAG   
  
  
+ ACACCTAGAG AAAACCATTA TGAACTCAAA AAAAACTAAT TCATCAACTT GAAAAAAAAA AACAAATACA   
  
  
+ AAATGGATTA AAAACATAAA ACTAGTTTGG GAAAAAAAAT TTCTCTCTCT AACTAGATCT AAACTTTCCA   
  
  
+ GCAATATAAA TACTTAAAAA TATTTTAATA ACATTTTTTT AACATATTGA TATCAAATTT AACCATTATT   
  
  
+ TTCAATAAAA TTCAACCAAC CAATTTCTGT CTCATCATTC GTGTTCCTAT GATTCTCTCT TTTTCTTTCA   
  
  
+ AACTCTCACA AGGTAAATAA AATTATATTA TGTGCTTGAA AGGAAATTTG AAAAATCTAA AGGAAAACTT   
  
  
+ AACTTAAAAG GTGCTGACAT TTTTTATTAA AAAAAGCTCA TATTGTTTTT TCTTTAATTT TGACTTTATT   
  
  
+ CTTTTCTTTT TCAAATAATA AAATTAGATT CGTTTCCATA AAATTTAATG AACTTTTAGG CAATAATGTT   
  
  
+ GTACGAAGTC AATAATGACG TGCTACAATC GTATGTCACG ACAAAAAGAC GACATGAACA CCATCCATTG   
  
  
+ GTATTCATGT GCCTTTTCAC CTTGTAAAAT AACAGGAGAA AGATACCTTC CAGGGGCAAA TTCATCTATG   
  
  
+ CCACGTGCCT ACAAGTTGAA GGTATGTTTG GTATGCAAG  

- ACATATTATT TTGTTCACGT ATATATAGTA GTACTGTGTT TTTATCTTTA TATCCGGTAT TATAATTTAA   
  
  
- TCCGTAGATA ACGTGGTCGG TACTGTCTGT AAACCAATTA TTATATTATG AGTAACTATT TCCACAGTTA   
  
  
- CTGTGTTTAG ATACTAGGAT CACAACCTTT TAAATTTAAT AAATTTATTG TACCAATCAA CTATATAATA   
  
  
- AAAAGAATAT TAATCCATCT AACTCCCCCC TTTACTATAA AAATATTCAT TATTTATAAC TATTTTGTTC   
  
  
- ATCATCTGTG GACACAGCGT GTACAGATGT GCAACCACAA CCACACGCTG CAAATAATTA CGAATTTACC   
  
  
- ACCAAAGATC CTGATCATAC GCCCAAACCT GTCGTGCACG CTGCAAATTA AAGTAGGAGT AATTTAATCA   
  
  
- CAAATAAAAA AATAAAAAAT AAAAAATAAA AAAATAAAAA AATTAACTTA AATAAAAAAT TAAAATAGTG   
  
  
- AGTAATAAAT TAAATTAAAA TAAACATACA ATTTAAATCA AAAATAAGAA AAATAAAGAT AAACAAAATG   
  
  
- AAATCTTTTT AAAAACTAAT TTTAAAAAAA AAAGTTATAT TAGGAAATCT AAATCAAAAA AAAAGAAAAA   
  
  
- TTAAAATCGA GAGTTGTAAA CTTAACATTC CTACACCAAA CTGAATTTTT TTAAAGTTCT ATTGTACAAA   
  
  
- AATTTATAAC CTTACTGTTG TATAATCTAG TTAGGTTCAG CTGAGTGATC ATTTTTTTTA TTTGAATAAT   
  
  
- TTAGGTGTAA CCAGTACCTA GGTTCATCTA AATTATTAAA AAAATTTGGT CAAAAATAAA TTAATGTACT   
  
  
- ATTTTTTATT CGCTATCATC AATTTGTTGG TTTAATTTAA ATGTTTAACT AATGTTACCT AACATTTTTT   
  
  
- GTGAACGATT ATAATGTTGT TTATAAAGAA AACAAAATAA GTTCTTGTTT CACTTTTACT TTACTTTAAA   
  
  
- CTATTTTGTT ATATTTTTTA TATAGTTAAT TGAATTATAA AAACTTTTGT TATATATTAT ATAAAATATA   
  
  
- AGTAATTAAA AATTCTTTAA TTAAAATTAA TAAACTTCTT TTTATTTTTT TTATCTGGTT CGCGCTCCCG   
  
  
- AACGGTTGGG TCCGGTTGTG AACCCAAAAA ACAACGAAAA ATTTGTTCTG TCTACTGTAC AGCAGATGTC   
  
  
- TCCTCTCACT ATACAGTAGA CATCTCAATC TGTTGTACAG TAGACGTTTG GATAAAAAAT TGTAGATCTC   
  
  
- TCATGACCTT TTAGATCGAC CCATAAACAA AAACAGGTTT TTGAATTAAA ATTAAATAAA AATAGATTTC   
  
  
- TGTGGATCTC TTTTGGTAAT ACTTGAGTTT TTTTTGATTA AGTAGTTGAA CTTTTTTTTT TTGTTTATGT   
  
  
- TTTACCTAAT TTTTGTATTT TGATCAAACC CTTTTTTTTA AAGAGAGAGA TTGATCTAGA TTTGAAAGGT   
  
  
- CGTTATATTT ATGAATTTTT ATAAAATTAT TGTAAAAAAA TTGTATAACT ATAGTTTAAA TTGGTAATAA   
  
  
- AAGTTATTTT AAGTTGGTTG GTTAAAGACA GAGTAGTAAG CACAAGGATA CTAAGAGAGA AAAAGAAAGT   
  
  
- TTGAGAGTGT TCCATTTATT TTAATATAAT ACACGAACTT TCCTTTAAAC TTTTTAGATT TCCTTTTGAA   
  
  
- TTGAATTTTC CACGACTGTA AAAAATAATT TTTTTCGAGT ATAACAAAAA AGAAATTAAA ACTGAAATAA   
  
  
- GAAAAGAAAA AGTTTATTAT TTTAATCTAA GCAAAGGTAT TTTAAATTAC TTGAAAATCC GTTATTACAA   
  
  
- CATGCTTCAG TTATTACTGC ACGATGTTAG CATACAGTGC TGTTTTTCTG CTGTACTTGT GGTAGGTAAC   
  
  
- CATAAGTACA CGGAAAAGTG GAACATTTTA TTGTCCTCTT TCTATGGAAG GTCCCCGTTT AAGTAGATAC   
  
  
- GGTGCACGGA TGTTCAACTT CCATACAAAC CATACGTTC

+     Unnamed\_\_14

| Site Name | Organism | Position | Strand | Matrix score. | sequence | function |
| --- | --- | --- | --- | --- | --- | --- |
| Unnamed\_\_14 | Zea mays | 790 | + | 9 | TCCACGTAGA |  |

>Potri.011G061700.1   
+ TGTATAATAA AACAAGTGCA TATATATCAT CATGACACAA AAATAGAAAT ATAGGCCATA ATATTAAATT   
  
  
+ AGGCATCTAT TGCACCAGCC ATGACAGACA TTTGGTTAAT AATATAATAC TCATTGATAA AGGTGTCAAT   
  
  
+ GACACAAATC TATGATCCTA GTGTTGGAAA ATTTAAATTA TTTAAATAAC ATGGTTAGTT GATATATTAT   
  
  
+ TTTTCTTATA ATTAGGTAGA TTGAGGGGGG AAATGATATT TTTATAAGTA ATAAATATTG ATAAAACAAG   
  
  
+ TAGTAGACAC CTGTGTCGCA CATGTCTACA CGTTGGTGTT GGTGTGCGAC GTTTATTAAT GCTTAAATGG   
  
  
+ TGGTTTCTAG GACTAGTATG CGGGTTTGGA CAGCACGTGC GACGTTTAAT TTCATCCTCA TTAAATTAGT   
  
  
+ GTTTATTTTT TTATTTTTTA TTTTTTATTT TTTTATTTTT TTAATTGAAT TTATTTTTTA ATTTTATCAC   
  
  
+ TCATTATTTA ATTTAATTTT ATTTGTATGT TAAATTTAGT TTTTATTCTT TTTATTTCTA TTTGTTTTAC   
  
  
+ TTTAGAAAAA TTTTTGATTA AAATTTTTTT TTTCAATATA ATCCTTTAGA TTTAGTTTTT TTTTCTTTTT   
  
  
+ AATTTTAGCT CTCAACATTT GAATTGTAAG GATGTGGTTT GACTTAAAAA AATTTCAAGA TAACATGTTT   
  
  
+ TTAAATATTG GAATGACAAC ATATTAGATC AATCCAAGTC GACTCACTAG TAAAAAAAAT AAACTTATTA   
  
  
+ AATCCACATT GGTCATGGAT CCAAGTAGAT TTAATAATTT TTTTAAACCA GTTTTTATTT AATTACATGA   
  
  
+ TAAAAAATAA GCGATAGTAG TTAAACAACC AAATTAAATT TACAAATTGA TTACAATGGA TTGTAAAAAA   
  
  
+ CACTTGCTAA TATTACAACA AATATTTCTT TTGTTTTATT CAAGAACAAA GTGAAAATGA AATGAAATTT   
  
  
+ GATAAAACAA TATAAAAAAT ATATCAATTA ACTTAATATT TTTGAAAACA ATATATAATA TATTTTATAT   
  
  
+ TCATTAATTT TTAAGAAATT AATTTTAATT ATTTGAAGAA AAATAAAAAA AATAGACCAA GCGCGAGGGC   
  
  
+ TTGCCAACCC AGGCCAACAC TTGGGTTTTT TGTTGCTTTT TAAACAAGAC AGATGACATG TCGTCTACAG   
  
  
+ AGGAGAGTGA TATGTCATCT GTAGAGTTAG ACAACATGTC ATCTGCAAAC CTATTTTTTA ACATCTAGAG   
  
  
+ AGTACTGGAA AATCTAGCTG GGTATTTGTT TTTGTCCAAA AACTTAATTT TAATTTATTT TTATCTAAAG   
  
  
+ ACACCTAGAG AAAACCATTA TGAACTCAAA AAAAACTAAT TCATCAACTT GAAAAAAAAA AACAAATACA   
  
  
+ AAATGGATTA AAAACATAAA ACTAGTTTGG GAAAAAAAAT TTCTCTCTCT AACTAGATCT AAACTTTCCA   
  
  
+ GCAATATAAA TACTTAAAAA TATTTTAATA ACATTTTTTT AACATATTGA TATCAAATTT AACCATTATT   
  
  
+ TTCAATAAAA TTCAACCAAC CAATTTCTGT CTCATCATTC GTGTTCCTAT GATTCTCTCT TTTTCTTTCA   
  
  
+ AACTCTCACA AGGTAAATAA AATTATATTA TGTGCTTGAA AGGAAATTTG AAAAATCTAA AGGAAAACTT   
  
  
+ AACTTAAAAG GTGCTGACAT TTTTTATTAA AAAAAGCTCA TATTGTTTTT TCTTTAATTT TGACTTTATT   
  
  
+ CTTTTCTTTT TCAAATAATA AAATTAGATT CGTTTCCATA AAATTTAATG AACTTTTAGG CAATAATGTT   
  
  
+ GTACGAAGTC AATAATGACG TGCTACAATC GTATGTCACG ACAAAAAGAC GACATGAACA CCATCCATTG   
  
  
+ GTATTCATGT GCCTTTTCAC CTTGTAAAAT AACAGGAGAA AGATACCTTC CAGGGGCAAA TTCATCTATG   
  
  
+ CCACGTGCCT ACAAGTTGAA GGTATGTTTG GTATGCAAG  

- ACATATTATT TTGTTCACGT ATATATAGTA GTACTGTGTT TTTATCTTTA TATCCGGTAT TATAATTTAA   
  
  
- TCCGTAGATA ACGTGGTCGG TACTGTCTGT AAACCAATTA TTATATTATG AGTAACTATT TCCACAGTTA   
  
  
- CTGTGTTTAG ATACTAGGAT CACAACCTTT TAAATTTAAT AAATTTATTG TACCAATCAA CTATATAATA   
  
  
- AAAAGAATAT TAATCCATCT AACTCCCCCC TTTACTATAA AAATATTCAT TATTTATAAC TATTTTGTTC   
  
  
- ATCATCTGTG GACACAGCGT GTACAGATGT GCAACCACAA CCACACGCTG CAAATAATTA CGAATTTACC   
  
  
- ACCAAAGATC CTGATCATAC GCCCAAACCT GTCGTGCACG CTGCAAATTA AAGTAGGAGT AATTTAATCA   
  
  
- CAAATAAAAA AATAAAAAAT AAAAAATAAA AAAATAAAAA AATTAACTTA AATAAAAAAT TAAAATAGTG   
  
  
- AGTAATAAAT TAAATTAAAA TAAACATACA ATTTAAATCA AAAATAAGAA AAATAAAGAT AAACAAAATG   
  
  
- AAATCTTTTT AAAAACTAAT TTTAAAAAAA AAAGTTATAT TAGGAAATCT AAATCAAAAA AAAAGAAAAA   
  
  
- TTAAAATCGA GAGTTGTAAA CTTAACATTC CTACACCAAA CTGAATTTTT TTAAAGTTCT ATTGTACAAA   
  
  
- AATTTATAAC CTTACTGTTG TATAATCTAG TTAGGTTCAG CTGAGTGATC ATTTTTTTTA TTTGAATAAT   
  
  
- TTAGGTGTAA CCAGTACCTA GGTTCATCTA AATTATTAAA AAAATTTGGT CAAAAATAAA TTAATGTACT   
  
  
- ATTTTTTATT CGCTATCATC AATTTGTTGG TTTAATTTAA ATGTTTAACT AATGTTACCT AACATTTTTT   
  
  
- GTGAACGATT ATAATGTTGT TTATAAAGAA AACAAAATAA GTTCTTGTTT CACTTTTACT TTACTTTAAA   
  
  
- CTATTTTGTT ATATTTTTTA TATAGTTAAT TGAATTATAA AAACTTTTGT TATATATTAT ATAAAATATA   
  
  
- AGTAATTAAA AATTCTTTAA TTAAAATTAA TAAACTTCTT TTTATTTTTT TTATCTGGTT CGCGCTCCCG   
  
  
- AACGGTTGGG TCCGGTTGTG AACCCAAAAA ACAACGAAAA ATTTGTTCTG TCTACTGTAC AGCAGATGTC   
  
  
- TCCTCTCACT ATACAGTAGA CATCTCAATC TGTTGTACAG TAGACGTTTG GATAAAAAAT TGTAGATCTC   
  
  
- TCATGACCTT TTAGATCGAC CCATAAACAA AAACAGGTTT TTGAATTAAA ATTAAATAAA AATAGATTTC   
  
  
- TGTGGATCTC TTTTGGTAAT ACTTGAGTTT TTTTTGATTA AGTAGTTGAA CTTTTTTTTT TTGTTTATGT   
  
  
- TTTACCTAAT TTTTGTATTT TGATCAAACC CTTTTTTTTA AAGAGAGAGA TTGATCTAGA TTTGAAAGGT   
  
  
- CGTTATATTT ATGAATTTTT ATAAAATTAT TGTAAAAAAA TTGTATAACT ATAGTTTAAA TTGGTAATAA   
  
  
- AAGTTATTTT AAGTTGGTTG GTTAAAGACA GAGTAGTAAG CACAAGGATA CTAAGAGAGA AAAAGAAAGT   
  
  
- TTGAGAGTGT TCCATTTATT TTAATATAAT ACACGAACTT TCCTTTAAAC TTTTTAGATT TCCTTTTGAA   
  
  
- TTGAATTTTC CACGACTGTA AAAAATAATT TTTTTCGAGT ATAACAAAAA AGAAATTAAA ACTGAAATAA   
  
  
- GAAAAGAAAA AGTTTATTAT TTTAATCTAA GCAAAGGTAT TTTAAATTAC TTGAAAATCC GTTATTACAA   
  
  
- CATGCTTCAG TTATTACTGC ACGATGTTAG CATACAGTGC TGTTTTTCTG CTGTACTTGT GGTAGGTAAC   
  
  
- CATAAGTACA CGGAAAAGTG GAACATTTTA TTGTCCTCTT TCTATGGAAG GTCCCCGTTT AAGTAGATAC   
  
  
- GGTGCACGGA TGTTCAACTT CCATACAAAC CATACGTTC

+     Unnamed\_\_4

| Site Name | Organism | Position | Strand | Matrix score. | sequence | function |
| --- | --- | --- | --- | --- | --- | --- |
| Unnamed\_\_4 | Petroselinum hortense | 1192 | - | 4 | CTCC |  |
| Unnamed\_\_4 | Petroselinum hortense | 1925 | - | 4 | CTCC |  |

>Potri.011G061700.1   
+ TGTATAATAA AACAAGTGCA TATATATCAT CATGACACAA AAATAGAAAT ATAGGCCATA ATATTAAATT   
  
  
+ AGGCATCTAT TGCACCAGCC ATGACAGACA TTTGGTTAAT AATATAATAC TCATTGATAA AGGTGTCAAT   
  
  
+ GACACAAATC TATGATCCTA GTGTTGGAAA ATTTAAATTA TTTAAATAAC ATGGTTAGTT GATATATTAT   
  
  
+ TTTTCTTATA ATTAGGTAGA TTGAGGGGGG AAATGATATT TTTATAAGTA ATAAATATTG ATAAAACAAG   
  
  
+ TAGTAGACAC CTGTGTCGCA CATGTCTACA CGTTGGTGTT GGTGTGCGAC GTTTATTAAT GCTTAAATGG   
  
  
+ TGGTTTCTAG GACTAGTATG CGGGTTTGGA CAGCACGTGC GACGTTTAAT TTCATCCTCA TTAAATTAGT   
  
  
+ GTTTATTTTT TTATTTTTTA TTTTTTATTT TTTTATTTTT TTAATTGAAT TTATTTTTTA ATTTTATCAC   
  
  
+ TCATTATTTA ATTTAATTTT ATTTGTATGT TAAATTTAGT TTTTATTCTT TTTATTTCTA TTTGTTTTAC   
  
  
+ TTTAGAAAAA TTTTTGATTA AAATTTTTTT TTTCAATATA ATCCTTTAGA TTTAGTTTTT TTTTCTTTTT   
  
  
+ AATTTTAGCT CTCAACATTT GAATTGTAAG GATGTGGTTT GACTTAAAAA AATTTCAAGA TAACATGTTT   
  
  
+ TTAAATATTG GAATGACAAC ATATTAGATC AATCCAAGTC GACTCACTAG TAAAAAAAAT AAACTTATTA   
  
  
+ AATCCACATT GGTCATGGAT CCAAGTAGAT TTAATAATTT TTTTAAACCA GTTTTTATTT AATTACATGA   
  
  
+ TAAAAAATAA GCGATAGTAG TTAAACAACC AAATTAAATT TACAAATTGA TTACAATGGA TTGTAAAAAA   
  
  
+ CACTTGCTAA TATTACAACA AATATTTCTT TTGTTTTATT CAAGAACAAA GTGAAAATGA AATGAAATTT   
  
  
+ GATAAAACAA TATAAAAAAT ATATCAATTA ACTTAATATT TTTGAAAACA ATATATAATA TATTTTATAT   
  
  
+ TCATTAATTT TTAAGAAATT AATTTTAATT ATTTGAAGAA AAATAAAAAA AATAGACCAA GCGCGAGGGC   
  
  
+ TTGCCAACCC AGGCCAACAC TTGGGTTTTT TGTTGCTTTT TAAACAAGAC AGATGACATG TCGTCTACAG   
  
  
+ AGGAGAGTGA TATGTCATCT GTAGAGTTAG ACAACATGTC ATCTGCAAAC CTATTTTTTA ACATCTAGAG   
  
  
+ AGTACTGGAA AATCTAGCTG GGTATTTGTT TTTGTCCAAA AACTTAATTT TAATTTATTT TTATCTAAAG   
  
  
+ ACACCTAGAG AAAACCATTA TGAACTCAAA AAAAACTAAT TCATCAACTT GAAAAAAAAA AACAAATACA   
  
  
+ AAATGGATTA AAAACATAAA ACTAGTTTGG GAAAAAAAAT TTCTCTCTCT AACTAGATCT AAACTTTCCA   
  
  
+ GCAATATAAA TACTTAAAAA TATTTTAATA ACATTTTTTT AACATATTGA TATCAAATTT AACCATTATT   
  
  
+ TTCAATAAAA TTCAACCAAC CAATTTCTGT CTCATCATTC GTGTTCCTAT GATTCTCTCT TTTTCTTTCA   
  
  
+ AACTCTCACA AGGTAAATAA AATTATATTA TGTGCTTGAA AGGAAATTTG AAAAATCTAA AGGAAAACTT   
  
  
+ AACTTAAAAG GTGCTGACAT TTTTTATTAA AAAAAGCTCA TATTGTTTTT TCTTTAATTT TGACTTTATT   
  
  
+ CTTTTCTTTT TCAAATAATA AAATTAGATT CGTTTCCATA AAATTTAATG AACTTTTAGG CAATAATGTT   
  
  
+ GTACGAAGTC AATAATGACG TGCTACAATC GTATGTCACG ACAAAAAGAC GACATGAACA CCATCCATTG   
  
  
+ GTATTCATGT GCCTTTTCAC CTTGTAAAAT AACAGGAGAA AGATACCTTC CAGGGGCAAA TTCATCTATG   
  
  
+ CCACGTGCCT ACAAGTTGAA GGTATGTTTG GTATGCAAG  

- ACATATTATT TTGTTCACGT ATATATAGTA GTACTGTGTT TTTATCTTTA TATCCGGTAT TATAATTTAA   
  
  
- TCCGTAGATA ACGTGGTCGG TACTGTCTGT AAACCAATTA TTATATTATG AGTAACTATT TCCACAGTTA   
  
  
- CTGTGTTTAG ATACTAGGAT CACAACCTTT TAAATTTAAT AAATTTATTG TACCAATCAA CTATATAATA   
  
  
- AAAAGAATAT TAATCCATCT AACTCCCCCC TTTACTATAA AAATATTCAT TATTTATAAC TATTTTGTTC   
  
  
- ATCATCTGTG GACACAGCGT GTACAGATGT GCAACCACAA CCACACGCTG CAAATAATTA CGAATTTACC   
  
  
- ACCAAAGATC CTGATCATAC GCCCAAACCT GTCGTGCACG CTGCAAATTA AAGTAGGAGT AATTTAATCA   
  
  
- CAAATAAAAA AATAAAAAAT AAAAAATAAA AAAATAAAAA AATTAACTTA AATAAAAAAT TAAAATAGTG   
  
  
- AGTAATAAAT TAAATTAAAA TAAACATACA ATTTAAATCA AAAATAAGAA AAATAAAGAT AAACAAAATG   
  
  
- AAATCTTTTT AAAAACTAAT TTTAAAAAAA AAAGTTATAT TAGGAAATCT AAATCAAAAA AAAAGAAAAA   
  
  
- TTAAAATCGA GAGTTGTAAA CTTAACATTC CTACACCAAA CTGAATTTTT TTAAAGTTCT ATTGTACAAA   
  
  
- AATTTATAAC CTTACTGTTG TATAATCTAG TTAGGTTCAG CTGAGTGATC ATTTTTTTTA TTTGAATAAT   
  
  
- TTAGGTGTAA CCAGTACCTA GGTTCATCTA AATTATTAAA AAAATTTGGT CAAAAATAAA TTAATGTACT   
  
  
- ATTTTTTATT CGCTATCATC AATTTGTTGG TTTAATTTAA ATGTTTAACT AATGTTACCT AACATTTTTT   
  
  
- GTGAACGATT ATAATGTTGT TTATAAAGAA AACAAAATAA GTTCTTGTTT CACTTTTACT TTACTTTAAA   
  
  
- CTATTTTGTT ATATTTTTTA TATAGTTAAT TGAATTATAA AAACTTTTGT TATATATTAT ATAAAATATA   
  
  
- AGTAATTAAA AATTCTTTAA TTAAAATTAA TAAACTTCTT TTTATTTTTT TTATCTGGTT CGCGCTCCCG   
  
  
- AACGGTTGGG TCCGGTTGTG AACCCAAAAA ACAACGAAAA ATTTGTTCTG TCTACTGTAC AGCAGATGTC   
  
  
- TCCTCTCACT ATACAGTAGA CATCTCAATC TGTTGTACAG TAGACGTTTG GATAAAAAAT TGTAGATCTC   
  
  
- TCATGACCTT TTAGATCGAC CCATAAACAA AAACAGGTTT TTGAATTAAA ATTAAATAAA AATAGATTTC   
  
  
- TGTGGATCTC TTTTGGTAAT ACTTGAGTTT TTTTTGATTA AGTAGTTGAA CTTTTTTTTT TTGTTTATGT   
  
  
- TTTACCTAAT TTTTGTATTT TGATCAAACC CTTTTTTTTA AAGAGAGAGA TTGATCTAGA TTTGAAAGGT   
  
  
- CGTTATATTT ATGAATTTTT ATAAAATTAT TGTAAAAAAA TTGTATAACT ATAGTTTAAA TTGGTAATAA   
  
  
- AAGTTATTTT AAGTTGGTTG GTTAAAGACA GAGTAGTAAG CACAAGGATA CTAAGAGAGA AAAAGAAAGT   
  
  
- TTGAGAGTGT TCCATTTATT TTAATATAAT ACACGAACTT TCCTTTAAAC TTTTTAGATT TCCTTTTGAA   
  
  
- TTGAATTTTC CACGACTGTA AAAAATAATT TTTTTCGAGT ATAACAAAAA AGAAATTAAA ACTGAAATAA   
  
  
- GAAAAGAAAA AGTTTATTAT TTTAATCTAA GCAAAGGTAT TTTAAATTAC TTGAAAATCC GTTATTACAA   
  
  
- CATGCTTCAG TTATTACTGC ACGATGTTAG CATACAGTGC TGTTTTTCTG CTGTACTTGT GGTAGGTAAC   
  
  
- CATAAGTACA CGGAAAAGTG GAACATTTTA TTGTCCTCTT TCTATGGAAG GTCCCCGTTT AAGTAGATAC   
  
  
- GGTGCACGGA TGTTCAACTT CCATACAAAC CATACGTTC

+     Unnamed\_\_8

| Site Name | Organism | Position | Strand | Matrix score. | sequence | function |
| --- | --- | --- | --- | --- | --- | --- |
| Unnamed\_\_8 | Zea mays | 790 | + | 9 | TCCACGTAGA |  |

>Potri.011G061700.1   
+ TGTATAATAA AACAAGTGCA TATATATCAT CATGACACAA AAATAGAAAT ATAGGCCATA ATATTAAATT   
  
  
+ AGGCATCTAT TGCACCAGCC ATGACAGACA TTTGGTTAAT AATATAATAC TCATTGATAA AGGTGTCAAT   
  
  
+ GACACAAATC TATGATCCTA GTGTTGGAAA ATTTAAATTA TTTAAATAAC ATGGTTAGTT GATATATTAT   
  
  
+ TTTTCTTATA ATTAGGTAGA TTGAGGGGGG AAATGATATT TTTATAAGTA ATAAATATTG ATAAAACAAG   
  
  
+ TAGTAGACAC CTGTGTCGCA CATGTCTACA CGTTGGTGTT GGTGTGCGAC GTTTATTAAT GCTTAAATGG   
  
  
+ TGGTTTCTAG GACTAGTATG CGGGTTTGGA CAGCACGTGC GACGTTTAAT TTCATCCTCA TTAAATTAGT   
  
  
+ GTTTATTTTT TTATTTTTTA TTTTTTATTT TTTTATTTTT TTAATTGAAT TTATTTTTTA ATTTTATCAC   
  
  
+ TCATTATTTA ATTTAATTTT ATTTGTATGT TAAATTTAGT TTTTATTCTT TTTATTTCTA TTTGTTTTAC   
  
  
+ TTTAGAAAAA TTTTTGATTA AAATTTTTTT TTTCAATATA ATCCTTTAGA TTTAGTTTTT TTTTCTTTTT   
  
  
+ AATTTTAGCT CTCAACATTT GAATTGTAAG GATGTGGTTT GACTTAAAAA AATTTCAAGA TAACATGTTT   
  
  
+ TTAAATATTG GAATGACAAC ATATTAGATC AATCCAAGTC GACTCACTAG TAAAAAAAAT AAACTTATTA   
  
  
+ AATCCACATT GGTCATGGAT CCAAGTAGAT TTAATAATTT TTTTAAACCA GTTTTTATTT AATTACATGA   
  
  
+ TAAAAAATAA GCGATAGTAG TTAAACAACC AAATTAAATT TACAAATTGA TTACAATGGA TTGTAAAAAA   
  
  
+ CACTTGCTAA TATTACAACA AATATTTCTT TTGTTTTATT CAAGAACAAA GTGAAAATGA AATGAAATTT   
  
  
+ GATAAAACAA TATAAAAAAT ATATCAATTA ACTTAATATT TTTGAAAACA ATATATAATA TATTTTATAT   
  
  
+ TCATTAATTT TTAAGAAATT AATTTTAATT ATTTGAAGAA AAATAAAAAA AATAGACCAA GCGCGAGGGC   
  
  
+ TTGCCAACCC AGGCCAACAC TTGGGTTTTT TGTTGCTTTT TAAACAAGAC AGATGACATG TCGTCTACAG   
  
  
+ AGGAGAGTGA TATGTCATCT GTAGAGTTAG ACAACATGTC ATCTGCAAAC CTATTTTTTA ACATCTAGAG   
  
  
+ AGTACTGGAA AATCTAGCTG GGTATTTGTT TTTGTCCAAA AACTTAATTT TAATTTATTT TTATCTAAAG   
  
  
+ ACACCTAGAG AAAACCATTA TGAACTCAAA AAAAACTAAT TCATCAACTT GAAAAAAAAA AACAAATACA   
  
  
+ AAATGGATTA AAAACATAAA ACTAGTTTGG GAAAAAAAAT TTCTCTCTCT AACTAGATCT AAACTTTCCA   
  
  
+ GCAATATAAA TACTTAAAAA TATTTTAATA ACATTTTTTT AACATATTGA TATCAAATTT AACCATTATT   
  
  
+ TTCAATAAAA TTCAACCAAC CAATTTCTGT CTCATCATTC GTGTTCCTAT GATTCTCTCT TTTTCTTTCA   
  
  
+ AACTCTCACA AGGTAAATAA AATTATATTA TGTGCTTGAA AGGAAATTTG AAAAATCTAA AGGAAAACTT   
  
  
+ AACTTAAAAG GTGCTGACAT TTTTTATTAA AAAAAGCTCA TATTGTTTTT TCTTTAATTT TGACTTTATT   
  
  
+ CTTTTCTTTT TCAAATAATA AAATTAGATT CGTTTCCATA AAATTTAATG AACTTTTAGG CAATAATGTT   
  
  
+ GTACGAAGTC AATAATGACG TGCTACAATC GTATGTCACG ACAAAAAGAC GACATGAACA CCATCCATTG   
  
  
+ GTATTCATGT GCCTTTTCAC CTTGTAAAAT AACAGGAGAA AGATACCTTC CAGGGGCAAA TTCATCTATG   
  
  
+ CCACGTGCCT ACAAGTTGAA GGTATGTTTG GTATGCAAG  

- ACATATTATT TTGTTCACGT ATATATAGTA GTACTGTGTT TTTATCTTTA TATCCGGTAT TATAATTTAA   
  
  
- TCCGTAGATA ACGTGGTCGG TACTGTCTGT AAACCAATTA TTATATTATG AGTAACTATT TCCACAGTTA   
  
  
- CTGTGTTTAG ATACTAGGAT CACAACCTTT TAAATTTAAT AAATTTATTG TACCAATCAA CTATATAATA   
  
  
- AAAAGAATAT TAATCCATCT AACTCCCCCC TTTACTATAA AAATATTCAT TATTTATAAC TATTTTGTTC   
  
  
- ATCATCTGTG GACACAGCGT GTACAGATGT GCAACCACAA CCACACGCTG CAAATAATTA CGAATTTACC   
  
  
- ACCAAAGATC CTGATCATAC GCCCAAACCT GTCGTGCACG CTGCAAATTA AAGTAGGAGT AATTTAATCA   
  
  
- CAAATAAAAA AATAAAAAAT AAAAAATAAA AAAATAAAAA AATTAACTTA AATAAAAAAT TAAAATAGTG   
  
  
- AGTAATAAAT TAAATTAAAA TAAACATACA ATTTAAATCA AAAATAAGAA AAATAAAGAT AAACAAAATG   
  
  
- AAATCTTTTT AAAAACTAAT TTTAAAAAAA AAAGTTATAT TAGGAAATCT AAATCAAAAA AAAAGAAAAA   
  
  
- TTAAAATCGA GAGTTGTAAA CTTAACATTC CTACACCAAA CTGAATTTTT TTAAAGTTCT ATTGTACAAA   
  
  
- AATTTATAAC CTTACTGTTG TATAATCTAG TTAGGTTCAG CTGAGTGATC ATTTTTTTTA TTTGAATAAT   
  
  
- TTAGGTGTAA CCAGTACCTA GGTTCATCTA AATTATTAAA AAAATTTGGT CAAAAATAAA TTAATGTACT   
  
  
- ATTTTTTATT CGCTATCATC AATTTGTTGG TTTAATTTAA ATGTTTAACT AATGTTACCT AACATTTTTT   
  
  
- GTGAACGATT ATAATGTTGT TTATAAAGAA AACAAAATAA GTTCTTGTTT CACTTTTACT TTACTTTAAA   
  
  
- CTATTTTGTT ATATTTTTTA TATAGTTAAT TGAATTATAA AAACTTTTGT TATATATTAT ATAAAATATA   
  
  
- AGTAATTAAA AATTCTTTAA TTAAAATTAA TAAACTTCTT TTTATTTTTT TTATCTGGTT CGCGCTCCCG   
  
  
- AACGGTTGGG TCCGGTTGTG AACCCAAAAA ACAACGAAAA ATTTGTTCTG TCTACTGTAC AGCAGATGTC   
  
  
- TCCTCTCACT ATACAGTAGA CATCTCAATC TGTTGTACAG TAGACGTTTG GATAAAAAAT TGTAGATCTC   
  
  
- TCATGACCTT TTAGATCGAC CCATAAACAA AAACAGGTTT TTGAATTAAA ATTAAATAAA AATAGATTTC   
  
  
- TGTGGATCTC TTTTGGTAAT ACTTGAGTTT TTTTTGATTA AGTAGTTGAA CTTTTTTTTT TTGTTTATGT   
  
  
- TTTACCTAAT TTTTGTATTT TGATCAAACC CTTTTTTTTA AAGAGAGAGA TTGATCTAGA TTTGAAAGGT   
  
  
- CGTTATATTT ATGAATTTTT ATAAAATTAT TGTAAAAAAA TTGTATAACT ATAGTTTAAA TTGGTAATAA   
  
  
- AAGTTATTTT AAGTTGGTTG GTTAAAGACA GAGTAGTAAG CACAAGGATA CTAAGAGAGA AAAAGAAAGT   
  
  
- TTGAGAGTGT TCCATTTATT TTAATATAAT ACACGAACTT TCCTTTAAAC TTTTTAGATT TCCTTTTGAA   
  
  
- TTGAATTTTC CACGACTGTA AAAAATAATT TTTTTCGAGT ATAACAAAAA AGAAATTAAA ACTGAAATAA   
  
  
- GAAAAGAAAA AGTTTATTAT TTTAATCTAA GCAAAGGTAT TTTAAATTAC TTGAAAATCC GTTATTACAA   
  
  
- CATGCTTCAG TTATTACTGC ACGATGTTAG CATACAGTGC TGTTTTTCTG CTGTACTTGT GGTAGGTAAC   
  
  
- CATAAGTACA CGGAAAAGTG GAACATTTTA TTGTCCTCTT TCTATGGAAG GTCCCCGTTT AAGTAGATAC   
  
  
- GGTGCACGGA TGTTCAACTT CCATACAAAC CATACGTTC

+     WUN-motif

| Site Name | Organism | Position | Strand | Matrix score. | sequence | function |
| --- | --- | --- | --- | --- | --- | --- |
| WUN-motif | Brassica oleracea | 1651 | - | 9 | AAATTTCCT | wound-responsive element |

>Potri.011G061700.1   
+ TGTATAATAA AACAAGTGCA TATATATCAT CATGACACAA AAATAGAAAT ATAGGCCATA ATATTAAATT   
  
  
+ AGGCATCTAT TGCACCAGCC ATGACAGACA TTTGGTTAAT AATATAATAC TCATTGATAA AGGTGTCAAT   
  
  
+ GACACAAATC TATGATCCTA GTGTTGGAAA ATTTAAATTA TTTAAATAAC ATGGTTAGTT GATATATTAT   
  
  
+ TTTTCTTATA ATTAGGTAGA TTGAGGGGGG AAATGATATT TTTATAAGTA ATAAATATTG ATAAAACAAG   
  
  
+ TAGTAGACAC CTGTGTCGCA CATGTCTACA CGTTGGTGTT GGTGTGCGAC GTTTATTAAT GCTTAAATGG   
  
  
+ TGGTTTCTAG GACTAGTATG CGGGTTTGGA CAGCACGTGC GACGTTTAAT TTCATCCTCA TTAAATTAGT   
  
  
+ GTTTATTTTT TTATTTTTTA TTTTTTATTT TTTTATTTTT TTAATTGAAT TTATTTTTTA ATTTTATCAC   
  
  
+ TCATTATTTA ATTTAATTTT ATTTGTATGT TAAATTTAGT TTTTATTCTT TTTATTTCTA TTTGTTTTAC   
  
  
+ TTTAGAAAAA TTTTTGATTA AAATTTTTTT TTTCAATATA ATCCTTTAGA TTTAGTTTTT TTTTCTTTTT   
  
  
+ AATTTTAGCT CTCAACATTT GAATTGTAAG GATGTGGTTT GACTTAAAAA AATTTCAAGA TAACATGTTT   
  
  
+ TTAAATATTG GAATGACAAC ATATTAGATC AATCCAAGTC GACTCACTAG TAAAAAAAAT AAACTTATTA   
  
  
+ AATCCACATT GGTCATGGAT CCAAGTAGAT TTAATAATTT TTTTAAACCA GTTTTTATTT AATTACATGA   
  
  
+ TAAAAAATAA GCGATAGTAG TTAAACAACC AAATTAAATT TACAAATTGA TTACAATGGA TTGTAAAAAA   
  
  
+ CACTTGCTAA TATTACAACA AATATTTCTT TTGTTTTATT CAAGAACAAA GTGAAAATGA AATGAAATTT   
  
  
+ GATAAAACAA TATAAAAAAT ATATCAATTA ACTTAATATT TTTGAAAACA ATATATAATA TATTTTATAT   
  
  
+ TCATTAATTT TTAAGAAATT AATTTTAATT ATTTGAAGAA AAATAAAAAA AATAGACCAA GCGCGAGGGC   
  
  
+ TTGCCAACCC AGGCCAACAC TTGGGTTTTT TGTTGCTTTT TAAACAAGAC AGATGACATG TCGTCTACAG   
  
  
+ AGGAGAGTGA TATGTCATCT GTAGAGTTAG ACAACATGTC ATCTGCAAAC CTATTTTTTA ACATCTAGAG   
  
  
+ AGTACTGGAA AATCTAGCTG GGTATTTGTT TTTGTCCAAA AACTTAATTT TAATTTATTT TTATCTAAAG   
  
  
+ ACACCTAGAG AAAACCATTA TGAACTCAAA AAAAACTAAT TCATCAACTT GAAAAAAAAA AACAAATACA   
  
  
+ AAATGGATTA AAAACATAAA ACTAGTTTGG GAAAAAAAAT TTCTCTCTCT AACTAGATCT AAACTTTCCA   
  
  
+ GCAATATAAA TACTTAAAAA TATTTTAATA ACATTTTTTT AACATATTGA TATCAAATTT AACCATTATT   
  
  
+ TTCAATAAAA TTCAACCAAC CAATTTCTGT CTCATCATTC GTGTTCCTAT GATTCTCTCT TTTTCTTTCA   
  
  
+ AACTCTCACA AGGTAAATAA AATTATATTA TGTGCTTGAA AGGAAATTTG AAAAATCTAA AGGAAAACTT   
  
  
+ AACTTAAAAG GTGCTGACAT TTTTTATTAA AAAAAGCTCA TATTGTTTTT TCTTTAATTT TGACTTTATT   
  
  
+ CTTTTCTTTT TCAAATAATA AAATTAGATT CGTTTCCATA AAATTTAATG AACTTTTAGG CAATAATGTT   
  
  
+ GTACGAAGTC AATAATGACG TGCTACAATC GTATGTCACG ACAAAAAGAC GACATGAACA CCATCCATTG   
  
  
+ GTATTCATGT GCCTTTTCAC CTTGTAAAAT AACAGGAGAA AGATACCTTC CAGGGGCAAA TTCATCTATG   
  
  
+ CCACGTGCCT ACAAGTTGAA GGTATGTTTG GTATGCAAG  

- ACATATTATT TTGTTCACGT ATATATAGTA GTACTGTGTT TTTATCTTTA TATCCGGTAT TATAATTTAA   
  
  
- TCCGTAGATA ACGTGGTCGG TACTGTCTGT AAACCAATTA TTATATTATG AGTAACTATT TCCACAGTTA   
  
  
- CTGTGTTTAG ATACTAGGAT CACAACCTTT TAAATTTAAT AAATTTATTG TACCAATCAA CTATATAATA   
  
  
- AAAAGAATAT TAATCCATCT AACTCCCCCC TTTACTATAA AAATATTCAT TATTTATAAC TATTTTGTTC   
  
  
- ATCATCTGTG GACACAGCGT GTACAGATGT GCAACCACAA CCACACGCTG CAAATAATTA CGAATTTACC   
  
  
- ACCAAAGATC CTGATCATAC GCCCAAACCT GTCGTGCACG CTGCAAATTA AAGTAGGAGT AATTTAATCA   
  
  
- CAAATAAAAA AATAAAAAAT AAAAAATAAA AAAATAAAAA AATTAACTTA AATAAAAAAT TAAAATAGTG   
  
  
- AGTAATAAAT TAAATTAAAA TAAACATACA ATTTAAATCA AAAATAAGAA AAATAAAGAT AAACAAAATG   
  
  
- AAATCTTTTT AAAAACTAAT TTTAAAAAAA AAAGTTATAT TAGGAAATCT AAATCAAAAA AAAAGAAAAA   
  
  
- TTAAAATCGA GAGTTGTAAA CTTAACATTC CTACACCAAA CTGAATTTTT TTAAAGTTCT ATTGTACAAA   
  
  
- AATTTATAAC CTTACTGTTG TATAATCTAG TTAGGTTCAG CTGAGTGATC ATTTTTTTTA TTTGAATAAT   
  
  
- TTAGGTGTAA CCAGTACCTA GGTTCATCTA AATTATTAAA AAAATTTGGT CAAAAATAAA TTAATGTACT   
  
  
- ATTTTTTATT CGCTATCATC AATTTGTTGG TTTAATTTAA ATGTTTAACT AATGTTACCT AACATTTTTT   
  
  
- GTGAACGATT ATAATGTTGT TTATAAAGAA AACAAAATAA GTTCTTGTTT CACTTTTACT TTACTTTAAA   
  
  
- CTATTTTGTT ATATTTTTTA TATAGTTAAT TGAATTATAA AAACTTTTGT TATATATTAT ATAAAATATA   
  
  
- AGTAATTAAA AATTCTTTAA TTAAAATTAA TAAACTTCTT TTTATTTTTT TTATCTGGTT CGCGCTCCCG   
  
  
- AACGGTTGGG TCCGGTTGTG AACCCAAAAA ACAACGAAAA ATTTGTTCTG TCTACTGTAC AGCAGATGTC   
  
  
- TCCTCTCACT ATACAGTAGA CATCTCAATC TGTTGTACAG TAGACGTTTG GATAAAAAAT TGTAGATCTC   
  
  
- TCATGACCTT TTAGATCGAC CCATAAACAA AAACAGGTTT TTGAATTAAA ATTAAATAAA AATAGATTTC   
  
  
- TGTGGATCTC TTTTGGTAAT ACTTGAGTTT TTTTTGATTA AGTAGTTGAA CTTTTTTTTT TTGTTTATGT   
  
  
- TTTACCTAAT TTTTGTATTT TGATCAAACC CTTTTTTTTA AAGAGAGAGA TTGATCTAGA TTTGAAAGGT   
  
  
- CGTTATATTT ATGAATTTTT ATAAAATTAT TGTAAAAAAA TTGTATAACT ATAGTTTAAA TTGGTAATAA   
  
  
- AAGTTATTTT AAGTTGGTTG GTTAAAGACA GAGTAGTAAG CACAAGGATA CTAAGAGAGA AAAAGAAAGT   
  
  
- TTGAGAGTGT TCCATTTATT TTAATATAAT ACACGAACTT TCCTTTAAAC TTTTTAGATT TCCTTTTGAA   
  
  
- TTGAATTTTC CACGACTGTA AAAAATAATT TTTTTCGAGT ATAACAAAAA AGAAATTAAA ACTGAAATAA   
  
  
- GAAAAGAAAA AGTTTATTAT TTTAATCTAA GCAAAGGTAT TTTAAATTAC TTGAAAATCC GTTATTACAA   
  
  
- CATGCTTCAG TTATTACTGC ACGATGTTAG CATACAGTGC TGTTTTTCTG CTGTACTTGT GGTAGGTAAC   
  
  
- CATAAGTACA CGGAAAAGTG GAACATTTTA TTGTCCTCTT TCTATGGAAG GTCCCCGTTT AAGTAGATAC   
  
  
- GGTGCACGGA TGTTCAACTT CCATACAAAC CATACGTTC

+     as-1

| Site Name | Organism | Position | Strand | Matrix score. | sequence | function |
| --- | --- | --- | --- | --- | --- | --- |
| as-1 | Arabidopsis thaliana | 1836 | + | 5 | TGACG |  |

>Potri.011G061700.1   
+ TGTATAATAA AACAAGTGCA TATATATCAT CATGACACAA AAATAGAAAT ATAGGCCATA ATATTAAATT   
  
  
+ AGGCATCTAT TGCACCAGCC ATGACAGACA TTTGGTTAAT AATATAATAC TCATTGATAA AGGTGTCAAT   
  
  
+ GACACAAATC TATGATCCTA GTGTTGGAAA ATTTAAATTA TTTAAATAAC ATGGTTAGTT GATATATTAT   
  
  
+ TTTTCTTATA ATTAGGTAGA TTGAGGGGGG AAATGATATT TTTATAAGTA ATAAATATTG ATAAAACAAG   
  
  
+ TAGTAGACAC CTGTGTCGCA CATGTCTACA CGTTGGTGTT GGTGTGCGAC GTTTATTAAT GCTTAAATGG   
  
  
+ TGGTTTCTAG GACTAGTATG CGGGTTTGGA CAGCACGTGC GACGTTTAAT TTCATCCTCA TTAAATTAGT   
  
  
+ GTTTATTTTT TTATTTTTTA TTTTTTATTT TTTTATTTTT TTAATTGAAT TTATTTTTTA ATTTTATCAC   
  
  
+ TCATTATTTA ATTTAATTTT ATTTGTATGT TAAATTTAGT TTTTATTCTT TTTATTTCTA TTTGTTTTAC   
  
  
+ TTTAGAAAAA TTTTTGATTA AAATTTTTTT TTTCAATATA ATCCTTTAGA TTTAGTTTTT TTTTCTTTTT   
  
  
+ AATTTTAGCT CTCAACATTT GAATTGTAAG GATGTGGTTT GACTTAAAAA AATTTCAAGA TAACATGTTT   
  
  
+ TTAAATATTG GAATGACAAC ATATTAGATC AATCCAAGTC GACTCACTAG TAAAAAAAAT AAACTTATTA   
  
  
+ AATCCACATT GGTCATGGAT CCAAGTAGAT TTAATAATTT TTTTAAACCA GTTTTTATTT AATTACATGA   
  
  
+ TAAAAAATAA GCGATAGTAG TTAAACAACC AAATTAAATT TACAAATTGA TTACAATGGA TTGTAAAAAA   
  
  
+ CACTTGCTAA TATTACAACA AATATTTCTT TTGTTTTATT CAAGAACAAA GTGAAAATGA AATGAAATTT   
  
  
+ GATAAAACAA TATAAAAAAT ATATCAATTA ACTTAATATT TTTGAAAACA ATATATAATA TATTTTATAT   
  
  
+ TCATTAATTT TTAAGAAATT AATTTTAATT ATTTGAAGAA AAATAAAAAA AATAGACCAA GCGCGAGGGC   
  
  
+ TTGCCAACCC AGGCCAACAC TTGGGTTTTT TGTTGCTTTT TAAACAAGAC AGATGACATG TCGTCTACAG   
  
  
+ AGGAGAGTGA TATGTCATCT GTAGAGTTAG ACAACATGTC ATCTGCAAAC CTATTTTTTA ACATCTAGAG   
  
  
+ AGTACTGGAA AATCTAGCTG GGTATTTGTT TTTGTCCAAA AACTTAATTT TAATTTATTT TTATCTAAAG   
  
  
+ ACACCTAGAG AAAACCATTA TGAACTCAAA AAAAACTAAT TCATCAACTT GAAAAAAAAA AACAAATACA   
  
  
+ AAATGGATTA AAAACATAAA ACTAGTTTGG GAAAAAAAAT TTCTCTCTCT AACTAGATCT AAACTTTCCA   
  
  
+ GCAATATAAA TACTTAAAAA TATTTTAATA ACATTTTTTT AACATATTGA TATCAAATTT AACCATTATT   
  
  
+ TTCAATAAAA TTCAACCAAC CAATTTCTGT CTCATCATTC GTGTTCCTAT GATTCTCTCT TTTTCTTTCA   
  
  
+ AACTCTCACA AGGTAAATAA AATTATATTA TGTGCTTGAA AGGAAATTTG AAAAATCTAA AGGAAAACTT   
  
  
+ AACTTAAAAG GTGCTGACAT TTTTTATTAA AAAAAGCTCA TATTGTTTTT TCTTTAATTT TGACTTTATT   
  
  
+ CTTTTCTTTT TCAAATAATA AAATTAGATT CGTTTCCATA AAATTTAATG AACTTTTAGG CAATAATGTT   
  
  
+ GTACGAAGTC AATAATGACG TGCTACAATC GTATGTCACG ACAAAAAGAC GACATGAACA CCATCCATTG   
  
  
+ GTATTCATGT GCCTTTTCAC CTTGTAAAAT AACAGGAGAA AGATACCTTC CAGGGGCAAA TTCATCTATG   
  
  
+ CCACGTGCCT ACAAGTTGAA GGTATGTTTG GTATGCAAG  

- ACATATTATT TTGTTCACGT ATATATAGTA GTACTGTGTT TTTATCTTTA TATCCGGTAT TATAATTTAA   
  
  
- TCCGTAGATA ACGTGGTCGG TACTGTCTGT AAACCAATTA TTATATTATG AGTAACTATT TCCACAGTTA   
  
  
- CTGTGTTTAG ATACTAGGAT CACAACCTTT TAAATTTAAT AAATTTATTG TACCAATCAA CTATATAATA   
  
  
- AAAAGAATAT TAATCCATCT AACTCCCCCC TTTACTATAA AAATATTCAT TATTTATAAC TATTTTGTTC   
  
  
- ATCATCTGTG GACACAGCGT GTACAGATGT GCAACCACAA CCACACGCTG CAAATAATTA CGAATTTACC   
  
  
- ACCAAAGATC CTGATCATAC GCCCAAACCT GTCGTGCACG CTGCAAATTA AAGTAGGAGT AATTTAATCA   
  
  
- CAAATAAAAA AATAAAAAAT AAAAAATAAA AAAATAAAAA AATTAACTTA AATAAAAAAT TAAAATAGTG   
  
  
- AGTAATAAAT TAAATTAAAA TAAACATACA ATTTAAATCA AAAATAAGAA AAATAAAGAT AAACAAAATG   
  
  
- AAATCTTTTT AAAAACTAAT TTTAAAAAAA AAAGTTATAT TAGGAAATCT AAATCAAAAA AAAAGAAAAA   
  
  
- TTAAAATCGA GAGTTGTAAA CTTAACATTC CTACACCAAA CTGAATTTTT TTAAAGTTCT ATTGTACAAA   
  
  
- AATTTATAAC CTTACTGTTG TATAATCTAG TTAGGTTCAG CTGAGTGATC ATTTTTTTTA TTTGAATAAT   
  
  
- TTAGGTGTAA CCAGTACCTA GGTTCATCTA AATTATTAAA AAAATTTGGT CAAAAATAAA TTAATGTACT   
  
  
- ATTTTTTATT CGCTATCATC AATTTGTTGG TTTAATTTAA ATGTTTAACT AATGTTACCT AACATTTTTT   
  
  
- GTGAACGATT ATAATGTTGT TTATAAAGAA AACAAAATAA GTTCTTGTTT CACTTTTACT TTACTTTAAA   
  
  
- CTATTTTGTT ATATTTTTTA TATAGTTAAT TGAATTATAA AAACTTTTGT TATATATTAT ATAAAATATA   
  
  
- AGTAATTAAA AATTCTTTAA TTAAAATTAA TAAACTTCTT TTTATTTTTT TTATCTGGTT CGCGCTCCCG   
  
  
- AACGGTTGGG TCCGGTTGTG AACCCAAAAA ACAACGAAAA ATTTGTTCTG TCTACTGTAC AGCAGATGTC   
  
  
- TCCTCTCACT ATACAGTAGA CATCTCAATC TGTTGTACAG TAGACGTTTG GATAAAAAAT TGTAGATCTC   
  
  
- TCATGACCTT TTAGATCGAC CCATAAACAA AAACAGGTTT TTGAATTAAA ATTAAATAAA AATAGATTTC   
  
  
- TGTGGATCTC TTTTGGTAAT ACTTGAGTTT TTTTTGATTA AGTAGTTGAA CTTTTTTTTT TTGTTTATGT   
  
  
- TTTACCTAAT TTTTGTATTT TGATCAAACC CTTTTTTTTA AAGAGAGAGA TTGATCTAGA TTTGAAAGGT   
  
  
- CGTTATATTT ATGAATTTTT ATAAAATTAT TGTAAAAAAA TTGTATAACT ATAGTTTAAA TTGGTAATAA   
  
  
- AAGTTATTTT AAGTTGGTTG GTTAAAGACA GAGTAGTAAG CACAAGGATA CTAAGAGAGA AAAAGAAAGT   
  
  
- TTGAGAGTGT TCCATTTATT TTAATATAAT ACACGAACTT TCCTTTAAAC TTTTTAGATT TCCTTTTGAA   
  
  
- TTGAATTTTC CACGACTGTA AAAAATAATT TTTTTCGAGT ATAACAAAAA AGAAATTAAA ACTGAAATAA   
  
  
- GAAAAGAAAA AGTTTATTAT TTTAATCTAA GCAAAGGTAT TTTAAATTAC TTGAAAATCC GTTATTACAA   
  
  
- CATGCTTCAG TTATTACTGC ACGATGTTAG CATACAGTGC TGTTTTTCTG CTGTACTTGT GGTAGGTAAC   
  
  
- CATAAGTACA CGGAAAAGTG GAACATTTTA TTGTCCTCTT TCTATGGAAG GTCCCCGTTT AAGTAGATAC   
  
  
- GGTGCACGGA TGTTCAACTT CCATACAAAC CATACGTTC
