# Supplementary material for: Structure and expression analysis of seven salt-related ERF genes of Populus
Source: PeerJ. 2020 Oct 20;8:e10206. doi: 10.7717/peerj.10206 (PMC7583627; doi:10.7717/peerj.10206)
Supplement: Supplemental Information 17 [file peerj-08-10206-s017.gz › Potri.018G038100.1_plantcare.html]

Content-Type: text/html; charset=ISO-8859-1


PlantCARE


Webmaster Firefox specific output  
To save the result:
click on the frame with the right mouse button and save the source code as a text file with extension .html  
REFERENCE:PlantCARE: a database of plant cis-acting regulatory elements and a portal to tools for in silico analysis of promoter sequences.  
Lescot, M., Déhais, P., Moreau, Y., De Moor, B., Rouzé ,P.,and Rombauts, S.  
Nucleic Acids Res., Database issue(2002), 30(1):325-327.   


---

>Potri.018G038100.1   
+ GTTGCTGTCT TTTTTTTTTA TCATGTTATC AAATTAATGG AAATTTAACT CTATTATCAA AATTCTCTTT   
  
  
+ TTTATAAACA TGATAATCTT TTTTATTTTA TTTTCAAGTT AAAAATACTT GGTCCGGCCT GGGACGATGC   
  
  
+ GCTTGCCAAT CTAGTCAATC CTAAATTTAC TAATACGAGT AAGTAATAGC CGAAGGAGCA AAGCTCCAGT   
  
  
+ CCGGCATCTT GTTCAGAACA ATATTACATC CCTGAAATGC CTCGTGAATT GGGTGTCTCT TCCAACTTTC   
  
  
+ TCCAGCAATT TTCTCCCAAC TTCTGCAAGC AGTGATTATC AATTTGAATT ACAGGAGCTA CCTAACATAT   
  
  
+ CCGCCCTAGA ACGAAGGCAG GAATCATCAG CTTTGCCACC TCTTTGCATA ACTGCTACTG AAAGCCATTA   
  
  
+ GCTGGTGAAT GCTCCATTTC AAACTTCCAT TGGCCCGAGT ACTGCTCCTG CTTCCATTGG AAATATCGCT   
  
  
+ GCGCAATCAG ACGAGGCAGG CATTCATATC TGATGATAAA TGTTGCAGGA TTAATTCTAT TTTCGCCGGC   
  
  
+ AGCAAATGGT GGGATTTTTT ACACTGAAAA GTGTTTCAAA AGACTCGAAT TAATGTGGTC GATTTTGGCG   
  
  
+ GGGGCGTGGA ATGGATTCCT CTCTTCCTCA ATCATCAAAC ATCCAGATAC CTGAAACAGA GAGAAGCCCA   
  
  
+ TCTTCAAAAT CATATCGTCA TCGGAGGTCC TTCGCTGCAA TCGGCTGATG ACGGTGCACT TTTTGATGGT   
  
  
+ AGCGAACCAG ATCAGTCAAT GGTCAAATCA TTTGGAAATC AACGAGCTAC AGTTTATCCC TCCTGAATCC   
  
  
+ ATCTTTGAGA ACCTAAAAGA AATTAATTAA AATTAACGAA AGAGAAAAGA GAGAGGTGGT AGAGTTCATA   
  
  
+ GTTGTCGGAT CGGTCCGGAA ATTGATTTAA TTTAGAAATT AATATAGAAA AGAGTTTTGA GTAATTATAT   
  
  
+ TATCTTTAAC GGAGGCAAAC TAAATTAATA TTTTTATTAA AAAAATAATT TTTTTAAAAA AAACTTTTTA   
  
  
+ GACTTTTGTA TAAATTAATC AAATTAAATT AACAAGATTT TACTAAATTA ATAATTTATT AAATTTAACT   
  
  
+ TAAAATCTAA TTTAATATCA CAGCCTGCTT GATATTATAA TATCGGTTGT TTCTGGTTTA AAAATATATT   
  
  
+ AAAATATTAT TTTTTAATTT TTTAAAATTT AATTTTAACA TTAATAAATT AAAATGATCT AATTTTAATT   
  
  
+ TTTTTAAAAA AACTAAGATT CTTTTCAAAA ACATCTTTTT ACGGTGGCTT AGACTCTATT CGTTTTTATA   
  
  
+ TTTTAAAAAT TCTTTTAAAA AAATTAAAAA TTTTTAATTT TTTTCACTTT AATTTTTTTT ATATTTTTAA   
  
  
+ ATTATTTTAA TATGTTAATA TTAAAAATAA AATTTTAAAA TAAAAAAATA TTATTTTAAT AAATTTTAAA   
  
  
+ ATTAAAAATA TTTTAAAAAC CAACCAAAAA AGGCAACCTA TTACGAACGA ACTAGGAGAC ATGATGATCG   
  
  
+ CTGGAAGAAG AGCAACAAGA AGCAATGACA TCACTGAAAG AATAACGGTT AGTCCGGTGA ATTTTCATTA   
  
  
+ GCTGGAAGTT ACTGCTAGTA GTTAATTAGT TAGAAGAGCT GTTGAGTTAG TTAGTAGTTA GTAGTTAATT   
  
  
+ AGTTAGAAAA GCTCTTGAGT TGGTTAGTAG TTAGCAGTTG AATAAAGGGG TTAGATCAAT TGTAATTAGG   
  
  
+ AAAAAACCAT TGTATATAAA CAGGTGTATG AAAACGGGTT GAGGATAAGA AAATACAGAA GATTAATCTC   
  
  
+ ATTTCTCTGC TCATTCTTCT CTGCTCTCTC TTGTTTCTGT TCTTTCTCAA TTTCTGTTCT CTTTTCTTTC   
  
  
+ TAATCTTGTA AAGCTGAGCA TATCATCACC AAGTCATCAC GTGTAGAAGC AAAGGGACAA CAAGTCATCA   
  
  
+ CCGAAGAAGA ATATAGTTGA CCTTCGAGAA CCCAGTCAA  

- CAACGACAGA AAAAAAAAAT AGTACAATAG TTTAATTACC TTTAAATTGA GATAATAGTT TTAAGAGAAA   
  
  
- AAATATTTGT ACTATTAGAA AAAATAAAAT AAAAGTTCAA TTTTTATGAA CCAGGCCGGA CCCTGCTACG   
  
  
- CGAACGGTTA GATCAGTTAG GATTTAAATG ATTATGCTCA TTCATTATCG GCTTCCTCGT TTCGAGGTCA   
  
  
- GGCCGTAGAA CAAGTCTTGT TATAATGTAG GGACTTTACG GAGCACTTAA CCCACAGAGA AGGTTGAAAG   
  
  
- AGGTCGTTAA AAGAGGGTTG AAGACGTTCG TCACTAATAG TTAAACTTAA TGTCCTCGAT GGATTGTATA   
  
  
- GGCGGGATCT TGCTTCCGTC CTTAGTAGTC GAAACGGTGG AGAAACGTAT TGACGATGAC TTTCGGTAAT   
  
  
- CGACCACTTA CGAGGTAAAG TTTGAAGGTA ACCGGGCTCA TGACGAGGAC GAAGGTAACC TTTATAGCGA   
  
  
- CGCGTTAGTC TGCTCCGTCC GTAAGTATAG ACTACTATTT ACAACGTCCT AATTAAGATA AAAGCGGCCG   
  
  
- TCGTTTACCA CCCTAAAAAA TGTGACTTTT CACAAAGTTT TCTGAGCTTA ATTACACCAG CTAAAACCGC   
  
  
- CCCCGCACCT TACCTAAGGA GAGAAGGAGT TAGTAGTTTG TAGGTCTATG GACTTTGTCT CTCTTCGGGT   
  
  
- AGAAGTTTTA GTATAGCAGT AGCCTCCAGG AAGCGACGTT AGCCGACTAC TGCCACGTGA AAAACTACCA   
  
  
- TCGCTTGGTC TAGTCAGTTA CCAGTTTAGT AAACCTTTAG TTGCTCGATG TCAAATAGGG AGGACTTAGG   
  
  
- TAGAAACTCT TGGATTTTCT TTAATTAATT TTAATTGCTT TCTCTTTTCT CTCTCCACCA TCTCAAGTAT   
  
  
- CAACAGCCTA GCCAGGCCTT TAACTAAATT AAATCTTTAA TTATATCTTT TCTCAAAACT CATTAATATA   
  
  
- ATAGAAATTG CCTCCGTTTG ATTTAATTAT AAAAATAATT TTTTTATTAA AAAAATTTTT TTTGAAAAAT   
  
  
- CTGAAAACAT ATTTAATTAG TTTAATTTAA TTGTTCTAAA ATGATTTAAT TATTAAATAA TTTAAATTGA   
  
  
- ATTTTAGATT AAATTATAGT GTCGGACGAA CTATAATATT ATAGCCAACA AAGACCAAAT TTTTATATAA   
  
  
- TTTTATAATA AAAAATTAAA AAATTTTAAA TTAAAATTGT AATTATTTAA TTTTACTAGA TTAAAATTAA   
  
  
- AAAAATTTTT TTGATTCTAA GAAAAGTTTT TGTAGAAAAA TGCCACCGAA TCTGAGATAA GCAAAAATAT   
  
  
- AAAATTTTTA AGAAAATTTT TTTAATTTTT AAAAATTAAA AAAAGTGAAA TTAAAAAAAA TATAAAAATT   
  
  
- TAATAAAATT ATACAATTAT AATTTTTATT TTAAAATTTT ATTTTTTTAT AATAAAATTA TTTAAAATTT   
  
  
- TAATTTTTAT AAAATTTTTG GTTGGTTTTT TCCGTTGGAT AATGCTTGCT TGATCCTCTG TACTACTAGC   
  
  
- GACCTTCTTC TCGTTGTTCT TCGTTACTGT AGTGACTTTC TTATTGCCAA TCAGGCCACT TAAAAGTAAT   
  
  
- CGACCTTCAA TGACGATCAT CAATTAATCA ATCTTCTCGA CAACTCAATC AATCATCAAT CATCAATTAA   
  
  
- TCAATCTTTT CGAGAACTCA ACCAATCATC AATCGTCAAC TTATTTCCCC AATCTAGTTA ACATTAATCC   
  
  
- TTTTTTGGTA ACATATATTT GTCCACATAC TTTTGCCCAA CTCCTATTCT TTTATGTCTT CTAATTAGAG   
  
  
- TAAAGAGACG AGTAAGAAGA GACGAGAGAG AACAAAGACA AGAAAGAGTT AAAGACAAGA GAAAAGAAAG   
  
  
- ATTAGAACAT TTCGACTCGT ATAGTAGTGG TTCAGTAGTG CACATCTTCG TTTCCCTGTT GTTCAGTAGT   
  
  
- GGCTTCTTCT TATATCAACT GGAAGCTCTT GGGTCAGTT

  
  
Motifs Found  

+   

| Site Name | Organism | Position | Strand | Matrix score. | sequence | function |
| --- | --- | --- | --- | --- | --- | --- |
|  | organism | 1874 | + | 4 | motif\_sequence | short\_function |
|  | organism | 1643 | - | 4 | motif\_sequence | short\_function |
|  | organism | 1808 | - | 4 | motif\_sequence | short\_function |
|  | organism | 899 | - | 4 | motif\_sequence | short\_function |
|  | organism | 1547 | - | 4 | motif\_sequence | short\_function |
|  | organism | 267 | + | 4 | motif\_sequence | short\_function |
|  | organism | 1857 | + | 4 | motif\_sequence | short\_function |
|  | organism | 1839 | + | 4 | motif\_sequence | short\_function |
|  | organism | 1825 | + | 4 | motif\_sequence | short\_function |
|  | organism | 651 | + | 4 | motif\_sequence | short\_function |
|  | organism | 700 | + | 4 | motif\_sequence | short\_function |

>Potri.018G038100.1   
+ GTTGCTGTCT TTTTTTTTTA TCATGTTATC AAATTAATGG AAATTTAACT CTATTATCAA AATTCTCTTT   
  
  
+ TTTATAAACA TGATAATCTT TTTTATTTTA TTTTCAAGTT AAAAATACTT GGTCCGGCCT GGGACGATGC   
  
  
+ GCTTGCCAAT CTAGTCAATC CTAAATTTAC TAATACGAGT AAGTAATAGC CGAAGGAGCA AAGCTCCAGT   
  
  
+ CCGGCATCTT GTTCAGAACA ATATTACATC CCTGAAATGC CTCGTGAATT GGGTGTCTCT TCCAACTTTC   
  
  
+ TCCAGCAATT TTCTCCCAAC TTCTGCAAGC AGTGATTATC AATTTGAATT ACAGGAGCTA CCTAACATAT   
  
  
+ CCGCCCTAGA ACGAAGGCAG GAATCATCAG CTTTGCCACC TCTTTGCATA ACTGCTACTG AAAGCCATTA   
  
  
+ GCTGGTGAAT GCTCCATTTC AAACTTCCAT TGGCCCGAGT ACTGCTCCTG CTTCCATTGG AAATATCGCT   
  
  
+ GCGCAATCAG ACGAGGCAGG CATTCATATC TGATGATAAA TGTTGCAGGA TTAATTCTAT TTTCGCCGGC   
  
  
+ AGCAAATGGT GGGATTTTTT ACACTGAAAA GTGTTTCAAA AGACTCGAAT TAATGTGGTC GATTTTGGCG   
  
  
+ GGGGCGTGGA ATGGATTCCT CTCTTCCTCA ATCATCAAAC ATCCAGATAC CTGAAACAGA GAGAAGCCCA   
  
  
+ TCTTCAAAAT CATATCGTCA TCGGAGGTCC TTCGCTGCAA TCGGCTGATG ACGGTGCACT TTTTGATGGT   
  
  
+ AGCGAACCAG ATCAGTCAAT GGTCAAATCA TTTGGAAATC AACGAGCTAC AGTTTATCCC TCCTGAATCC   
  
  
+ ATCTTTGAGA ACCTAAAAGA AATTAATTAA AATTAACGAA AGAGAAAAGA GAGAGGTGGT AGAGTTCATA   
  
  
+ GTTGTCGGAT CGGTCCGGAA ATTGATTTAA TTTAGAAATT AATATAGAAA AGAGTTTTGA GTAATTATAT   
  
  
+ TATCTTTAAC GGAGGCAAAC TAAATTAATA TTTTTATTAA AAAAATAATT TTTTTAAAAA AAACTTTTTA   
  
  
+ GACTTTTGTA TAAATTAATC AAATTAAATT AACAAGATTT TACTAAATTA ATAATTTATT AAATTTAACT   
  
  
+ TAAAATCTAA TTTAATATCA CAGCCTGCTT GATATTATAA TATCGGTTGT TTCTGGTTTA AAAATATATT   
  
  
+ AAAATATTAT TTTTTAATTT TTTAAAATTT AATTTTAACA TTAATAAATT AAAATGATCT AATTTTAATT   
  
  
+ TTTTTAAAAA AACTAAGATT CTTTTCAAAA ACATCTTTTT ACGGTGGCTT AGACTCTATT CGTTTTTATA   
  
  
+ TTTTAAAAAT TCTTTTAAAA AAATTAAAAA TTTTTAATTT TTTTCACTTT AATTTTTTTT ATATTTTTAA   
  
  
+ ATTATTTTAA TATGTTAATA TTAAAAATAA AATTTTAAAA TAAAAAAATA TTATTTTAAT AAATTTTAAA   
  
  
+ ATTAAAAATA TTTTAAAAAC CAACCAAAAA AGGCAACCTA TTACGAACGA ACTAGGAGAC ATGATGATCG   
  
  
+ CTGGAAGAAG AGCAACAAGA AGCAATGACA TCACTGAAAG AATAACGGTT AGTCCGGTGA ATTTTCATTA   
  
  
+ GCTGGAAGTT ACTGCTAGTA GTTAATTAGT TAGAAGAGCT GTTGAGTTAG TTAGTAGTTA GTAGTTAATT   
  
  
+ AGTTAGAAAA GCTCTTGAGT TGGTTAGTAG TTAGCAGTTG AATAAAGGGG TTAGATCAAT TGTAATTAGG   
  
  
+ AAAAAACCAT TGTATATAAA CAGGTGTATG AAAACGGGTT GAGGATAAGA AAATACAGAA GATTAATCTC   
  
  
+ ATTTCTCTGC TCATTCTTCT CTGCTCTCTC TTGTTTCTGT TCTTTCTCAA TTTCTGTTCT CTTTTCTTTC   
  
  
+ TAATCTTGTA AAGCTGAGCA TATCATCACC AAGTCATCAC GTGTAGAAGC AAAGGGACAA CAAGTCATCA   
  
  
+ CCGAAGAAGA ATATAGTTGA CCTTCGAGAA CCCAGTCAA  

- CAACGACAGA AAAAAAAAAT AGTACAATAG TTTAATTACC TTTAAATTGA GATAATAGTT TTAAGAGAAA   
  
  
- AAATATTTGT ACTATTAGAA AAAATAAAAT AAAAGTTCAA TTTTTATGAA CCAGGCCGGA CCCTGCTACG   
  
  
- CGAACGGTTA GATCAGTTAG GATTTAAATG ATTATGCTCA TTCATTATCG GCTTCCTCGT TTCGAGGTCA   
  
  
- GGCCGTAGAA CAAGTCTTGT TATAATGTAG GGACTTTACG GAGCACTTAA CCCACAGAGA AGGTTGAAAG   
  
  
- AGGTCGTTAA AAGAGGGTTG AAGACGTTCG TCACTAATAG TTAAACTTAA TGTCCTCGAT GGATTGTATA   
  
  
- GGCGGGATCT TGCTTCCGTC CTTAGTAGTC GAAACGGTGG AGAAACGTAT TGACGATGAC TTTCGGTAAT   
  
  
- CGACCACTTA CGAGGTAAAG TTTGAAGGTA ACCGGGCTCA TGACGAGGAC GAAGGTAACC TTTATAGCGA   
  
  
- CGCGTTAGTC TGCTCCGTCC GTAAGTATAG ACTACTATTT ACAACGTCCT AATTAAGATA AAAGCGGCCG   
  
  
- TCGTTTACCA CCCTAAAAAA TGTGACTTTT CACAAAGTTT TCTGAGCTTA ATTACACCAG CTAAAACCGC   
  
  
- CCCCGCACCT TACCTAAGGA GAGAAGGAGT TAGTAGTTTG TAGGTCTATG GACTTTGTCT CTCTTCGGGT   
  
  
- AGAAGTTTTA GTATAGCAGT AGCCTCCAGG AAGCGACGTT AGCCGACTAC TGCCACGTGA AAAACTACCA   
  
  
- TCGCTTGGTC TAGTCAGTTA CCAGTTTAGT AAACCTTTAG TTGCTCGATG TCAAATAGGG AGGACTTAGG   
  
  
- TAGAAACTCT TGGATTTTCT TTAATTAATT TTAATTGCTT TCTCTTTTCT CTCTCCACCA TCTCAAGTAT   
  
  
- CAACAGCCTA GCCAGGCCTT TAACTAAATT AAATCTTTAA TTATATCTTT TCTCAAAACT CATTAATATA   
  
  
- ATAGAAATTG CCTCCGTTTG ATTTAATTAT AAAAATAATT TTTTTATTAA AAAAATTTTT TTTGAAAAAT   
  
  
- CTGAAAACAT ATTTAATTAG TTTAATTTAA TTGTTCTAAA ATGATTTAAT TATTAAATAA TTTAAATTGA   
  
  
- ATTTTAGATT AAATTATAGT GTCGGACGAA CTATAATATT ATAGCCAACA AAGACCAAAT TTTTATATAA   
  
  
- TTTTATAATA AAAAATTAAA AAATTTTAAA TTAAAATTGT AATTATTTAA TTTTACTAGA TTAAAATTAA   
  
  
- AAAAATTTTT TTGATTCTAA GAAAAGTTTT TGTAGAAAAA TGCCACCGAA TCTGAGATAA GCAAAAATAT   
  
  
- AAAATTTTTA AGAAAATTTT TTTAATTTTT AAAAATTAAA AAAAGTGAAA TTAAAAAAAA TATAAAAATT   
  
  
- TAATAAAATT ATACAATTAT AATTTTTATT TTAAAATTTT ATTTTTTTAT AATAAAATTA TTTAAAATTT   
  
  
- TAATTTTTAT AAAATTTTTG GTTGGTTTTT TCCGTTGGAT AATGCTTGCT TGATCCTCTG TACTACTAGC   
  
  
- GACCTTCTTC TCGTTGTTCT TCGTTACTGT AGTGACTTTC TTATTGCCAA TCAGGCCACT TAAAAGTAAT   
  
  
- CGACCTTCAA TGACGATCAT CAATTAATCA ATCTTCTCGA CAACTCAATC AATCATCAAT CATCAATTAA   
  
  
- TCAATCTTTT CGAGAACTCA ACCAATCATC AATCGTCAAC TTATTTCCCC AATCTAGTTA ACATTAATCC   
  
  
- TTTTTTGGTA ACATATATTT GTCCACATAC TTTTGCCCAA CTCCTATTCT TTTATGTCTT CTAATTAGAG   
  
  
- TAAAGAGACG AGTAAGAAGA GACGAGAGAG AACAAAGACA AGAAAGAGTT AAAGACAAGA GAAAAGAAAG   
  
  
- ATTAGAACAT TTCGACTCGT ATAGTAGTGG TTCAGTAGTG CACATCTTCG TTTCCCTGTT GTTCAGTAGT   
  
  
- GGCTTCTTCT TATATCAACT GGAAGCTCTT GGGTCAGTT

+     3-AF1 binding site

| Site Name | Organism | Position | Strand | Matrix score. | sequence | function |
| --- | --- | --- | --- | --- | --- | --- |
| 3-AF1 binding site | Solanum tuberosum | 646 | - | 10 | TAAGAGAGGAA | light responsive element |

>Potri.018G038100.1   
+ GTTGCTGTCT TTTTTTTTTA TCATGTTATC AAATTAATGG AAATTTAACT CTATTATCAA AATTCTCTTT   
  
  
+ TTTATAAACA TGATAATCTT TTTTATTTTA TTTTCAAGTT AAAAATACTT GGTCCGGCCT GGGACGATGC   
  
  
+ GCTTGCCAAT CTAGTCAATC CTAAATTTAC TAATACGAGT AAGTAATAGC CGAAGGAGCA AAGCTCCAGT   
  
  
+ CCGGCATCTT GTTCAGAACA ATATTACATC CCTGAAATGC CTCGTGAATT GGGTGTCTCT TCCAACTTTC   
  
  
+ TCCAGCAATT TTCTCCCAAC TTCTGCAAGC AGTGATTATC AATTTGAATT ACAGGAGCTA CCTAACATAT   
  
  
+ CCGCCCTAGA ACGAAGGCAG GAATCATCAG CTTTGCCACC TCTTTGCATA ACTGCTACTG AAAGCCATTA   
  
  
+ GCTGGTGAAT GCTCCATTTC AAACTTCCAT TGGCCCGAGT ACTGCTCCTG CTTCCATTGG AAATATCGCT   
  
  
+ GCGCAATCAG ACGAGGCAGG CATTCATATC TGATGATAAA TGTTGCAGGA TTAATTCTAT TTTCGCCGGC   
  
  
+ AGCAAATGGT GGGATTTTTT ACACTGAAAA GTGTTTCAAA AGACTCGAAT TAATGTGGTC GATTTTGGCG   
  
  
+ GGGGCGTGGA ATGGATTCCT CTCTTCCTCA ATCATCAAAC ATCCAGATAC CTGAAACAGA GAGAAGCCCA   
  
  
+ TCTTCAAAAT CATATCGTCA TCGGAGGTCC TTCGCTGCAA TCGGCTGATG ACGGTGCACT TTTTGATGGT   
  
  
+ AGCGAACCAG ATCAGTCAAT GGTCAAATCA TTTGGAAATC AACGAGCTAC AGTTTATCCC TCCTGAATCC   
  
  
+ ATCTTTGAGA ACCTAAAAGA AATTAATTAA AATTAACGAA AGAGAAAAGA GAGAGGTGGT AGAGTTCATA   
  
  
+ GTTGTCGGAT CGGTCCGGAA ATTGATTTAA TTTAGAAATT AATATAGAAA AGAGTTTTGA GTAATTATAT   
  
  
+ TATCTTTAAC GGAGGCAAAC TAAATTAATA TTTTTATTAA AAAAATAATT TTTTTAAAAA AAACTTTTTA   
  
  
+ GACTTTTGTA TAAATTAATC AAATTAAATT AACAAGATTT TACTAAATTA ATAATTTATT AAATTTAACT   
  
  
+ TAAAATCTAA TTTAATATCA CAGCCTGCTT GATATTATAA TATCGGTTGT TTCTGGTTTA AAAATATATT   
  
  
+ AAAATATTAT TTTTTAATTT TTTAAAATTT AATTTTAACA TTAATAAATT AAAATGATCT AATTTTAATT   
  
  
+ TTTTTAAAAA AACTAAGATT CTTTTCAAAA ACATCTTTTT ACGGTGGCTT AGACTCTATT CGTTTTTATA   
  
  
+ TTTTAAAAAT TCTTTTAAAA AAATTAAAAA TTTTTAATTT TTTTCACTTT AATTTTTTTT ATATTTTTAA   
  
  
+ ATTATTTTAA TATGTTAATA TTAAAAATAA AATTTTAAAA TAAAAAAATA TTATTTTAAT AAATTTTAAA   
  
  
+ ATTAAAAATA TTTTAAAAAC CAACCAAAAA AGGCAACCTA TTACGAACGA ACTAGGAGAC ATGATGATCG   
  
  
+ CTGGAAGAAG AGCAACAAGA AGCAATGACA TCACTGAAAG AATAACGGTT AGTCCGGTGA ATTTTCATTA   
  
  
+ GCTGGAAGTT ACTGCTAGTA GTTAATTAGT TAGAAGAGCT GTTGAGTTAG TTAGTAGTTA GTAGTTAATT   
  
  
+ AGTTAGAAAA GCTCTTGAGT TGGTTAGTAG TTAGCAGTTG AATAAAGGGG TTAGATCAAT TGTAATTAGG   
  
  
+ AAAAAACCAT TGTATATAAA CAGGTGTATG AAAACGGGTT GAGGATAAGA AAATACAGAA GATTAATCTC   
  
  
+ ATTTCTCTGC TCATTCTTCT CTGCTCTCTC TTGTTTCTGT TCTTTCTCAA TTTCTGTTCT CTTTTCTTTC   
  
  
+ TAATCTTGTA AAGCTGAGCA TATCATCACC AAGTCATCAC GTGTAGAAGC AAAGGGACAA CAAGTCATCA   
  
  
+ CCGAAGAAGA ATATAGTTGA CCTTCGAGAA CCCAGTCAA  

- CAACGACAGA AAAAAAAAAT AGTACAATAG TTTAATTACC TTTAAATTGA GATAATAGTT TTAAGAGAAA   
  
  
- AAATATTTGT ACTATTAGAA AAAATAAAAT AAAAGTTCAA TTTTTATGAA CCAGGCCGGA CCCTGCTACG   
  
  
- CGAACGGTTA GATCAGTTAG GATTTAAATG ATTATGCTCA TTCATTATCG GCTTCCTCGT TTCGAGGTCA   
  
  
- GGCCGTAGAA CAAGTCTTGT TATAATGTAG GGACTTTACG GAGCACTTAA CCCACAGAGA AGGTTGAAAG   
  
  
- AGGTCGTTAA AAGAGGGTTG AAGACGTTCG TCACTAATAG TTAAACTTAA TGTCCTCGAT GGATTGTATA   
  
  
- GGCGGGATCT TGCTTCCGTC CTTAGTAGTC GAAACGGTGG AGAAACGTAT TGACGATGAC TTTCGGTAAT   
  
  
- CGACCACTTA CGAGGTAAAG TTTGAAGGTA ACCGGGCTCA TGACGAGGAC GAAGGTAACC TTTATAGCGA   
  
  
- CGCGTTAGTC TGCTCCGTCC GTAAGTATAG ACTACTATTT ACAACGTCCT AATTAAGATA AAAGCGGCCG   
  
  
- TCGTTTACCA CCCTAAAAAA TGTGACTTTT CACAAAGTTT TCTGAGCTTA ATTACACCAG CTAAAACCGC   
  
  
- CCCCGCACCT TACCTAAGGA GAGAAGGAGT TAGTAGTTTG TAGGTCTATG GACTTTGTCT CTCTTCGGGT   
  
  
- AGAAGTTTTA GTATAGCAGT AGCCTCCAGG AAGCGACGTT AGCCGACTAC TGCCACGTGA AAAACTACCA   
  
  
- TCGCTTGGTC TAGTCAGTTA CCAGTTTAGT AAACCTTTAG TTGCTCGATG TCAAATAGGG AGGACTTAGG   
  
  
- TAGAAACTCT TGGATTTTCT TTAATTAATT TTAATTGCTT TCTCTTTTCT CTCTCCACCA TCTCAAGTAT   
  
  
- CAACAGCCTA GCCAGGCCTT TAACTAAATT AAATCTTTAA TTATATCTTT TCTCAAAACT CATTAATATA   
  
  
- ATAGAAATTG CCTCCGTTTG ATTTAATTAT AAAAATAATT TTTTTATTAA AAAAATTTTT TTTGAAAAAT   
  
  
- CTGAAAACAT ATTTAATTAG TTTAATTTAA TTGTTCTAAA ATGATTTAAT TATTAAATAA TTTAAATTGA   
  
  
- ATTTTAGATT AAATTATAGT GTCGGACGAA CTATAATATT ATAGCCAACA AAGACCAAAT TTTTATATAA   
  
  
- TTTTATAATA AAAAATTAAA AAATTTTAAA TTAAAATTGT AATTATTTAA TTTTACTAGA TTAAAATTAA   
  
  
- AAAAATTTTT TTGATTCTAA GAAAAGTTTT TGTAGAAAAA TGCCACCGAA TCTGAGATAA GCAAAAATAT   
  
  
- AAAATTTTTA AGAAAATTTT TTTAATTTTT AAAAATTAAA AAAAGTGAAA TTAAAAAAAA TATAAAAATT   
  
  
- TAATAAAATT ATACAATTAT AATTTTTATT TTAAAATTTT ATTTTTTTAT AATAAAATTA TTTAAAATTT   
  
  
- TAATTTTTAT AAAATTTTTG GTTGGTTTTT TCCGTTGGAT AATGCTTGCT TGATCCTCTG TACTACTAGC   
  
  
- GACCTTCTTC TCGTTGTTCT TCGTTACTGT AGTGACTTTC TTATTGCCAA TCAGGCCACT TAAAAGTAAT   
  
  
- CGACCTTCAA TGACGATCAT CAATTAATCA ATCTTCTCGA CAACTCAATC AATCATCAAT CATCAATTAA   
  
  
- TCAATCTTTT CGAGAACTCA ACCAATCATC AATCGTCAAC TTATTTCCCC AATCTAGTTA ACATTAATCC   
  
  
- TTTTTTGGTA ACATATATTT GTCCACATAC TTTTGCCCAA CTCCTATTCT TTTATGTCTT CTAATTAGAG   
  
  
- TAAAGAGACG AGTAAGAAGA GACGAGAGAG AACAAAGACA AGAAAGAGTT AAAGACAAGA GAAAAGAAAG   
  
  
- ATTAGAACAT TTCGACTCGT ATAGTAGTGG TTCAGTAGTG CACATCTTCG TTTCCCTGTT GTTCAGTAGT   
  
  
- GGCTTCTTCT TATATCAACT GGAAGCTCTT GGGTCAGTT

+     AAGAA-motif

| Site Name | Organism | Position | Strand | Matrix score. | sequence | function |
| --- | --- | --- | --- | --- | --- | --- |
| AAGAA-motif | Avena sativa | 1884 | - | 7 | GAAAGAA |  |
| AAGAA-motif | Avena sativa | 1576 | + | 7 | GAAAGAA |  |
| AAGAA-motif | Avena sativa | 1860 | - | 7 | GAAAGAA |  |

>Potri.018G038100.1   
+ GTTGCTGTCT TTTTTTTTTA TCATGTTATC AAATTAATGG AAATTTAACT CTATTATCAA AATTCTCTTT   
  
  
+ TTTATAAACA TGATAATCTT TTTTATTTTA TTTTCAAGTT AAAAATACTT GGTCCGGCCT GGGACGATGC   
  
  
+ GCTTGCCAAT CTAGTCAATC CTAAATTTAC TAATACGAGT AAGTAATAGC CGAAGGAGCA AAGCTCCAGT   
  
  
+ CCGGCATCTT GTTCAGAACA ATATTACATC CCTGAAATGC CTCGTGAATT GGGTGTCTCT TCCAACTTTC   
  
  
+ TCCAGCAATT TTCTCCCAAC TTCTGCAAGC AGTGATTATC AATTTGAATT ACAGGAGCTA CCTAACATAT   
  
  
+ CCGCCCTAGA ACGAAGGCAG GAATCATCAG CTTTGCCACC TCTTTGCATA ACTGCTACTG AAAGCCATTA   
  
  
+ GCTGGTGAAT GCTCCATTTC AAACTTCCAT TGGCCCGAGT ACTGCTCCTG CTTCCATTGG AAATATCGCT   
  
  
+ GCGCAATCAG ACGAGGCAGG CATTCATATC TGATGATAAA TGTTGCAGGA TTAATTCTAT TTTCGCCGGC   
  
  
+ AGCAAATGGT GGGATTTTTT ACACTGAAAA GTGTTTCAAA AGACTCGAAT TAATGTGGTC GATTTTGGCG   
  
  
+ GGGGCGTGGA ATGGATTCCT CTCTTCCTCA ATCATCAAAC ATCCAGATAC CTGAAACAGA GAGAAGCCCA   
  
  
+ TCTTCAAAAT CATATCGTCA TCGGAGGTCC TTCGCTGCAA TCGGCTGATG ACGGTGCACT TTTTGATGGT   
  
  
+ AGCGAACCAG ATCAGTCAAT GGTCAAATCA TTTGGAAATC AACGAGCTAC AGTTTATCCC TCCTGAATCC   
  
  
+ ATCTTTGAGA ACCTAAAAGA AATTAATTAA AATTAACGAA AGAGAAAAGA GAGAGGTGGT AGAGTTCATA   
  
  
+ GTTGTCGGAT CGGTCCGGAA ATTGATTTAA TTTAGAAATT AATATAGAAA AGAGTTTTGA GTAATTATAT   
  
  
+ TATCTTTAAC GGAGGCAAAC TAAATTAATA TTTTTATTAA AAAAATAATT TTTTTAAAAA AAACTTTTTA   
  
  
+ GACTTTTGTA TAAATTAATC AAATTAAATT AACAAGATTT TACTAAATTA ATAATTTATT AAATTTAACT   
  
  
+ TAAAATCTAA TTTAATATCA CAGCCTGCTT GATATTATAA TATCGGTTGT TTCTGGTTTA AAAATATATT   
  
  
+ AAAATATTAT TTTTTAATTT TTTAAAATTT AATTTTAACA TTAATAAATT AAAATGATCT AATTTTAATT   
  
  
+ TTTTTAAAAA AACTAAGATT CTTTTCAAAA ACATCTTTTT ACGGTGGCTT AGACTCTATT CGTTTTTATA   
  
  
+ TTTTAAAAAT TCTTTTAAAA AAATTAAAAA TTTTTAATTT TTTTCACTTT AATTTTTTTT ATATTTTTAA   
  
  
+ ATTATTTTAA TATGTTAATA TTAAAAATAA AATTTTAAAA TAAAAAAATA TTATTTTAAT AAATTTTAAA   
  
  
+ ATTAAAAATA TTTTAAAAAC CAACCAAAAA AGGCAACCTA TTACGAACGA ACTAGGAGAC ATGATGATCG   
  
  
+ CTGGAAGAAG AGCAACAAGA AGCAATGACA TCACTGAAAG AATAACGGTT AGTCCGGTGA ATTTTCATTA   
  
  
+ GCTGGAAGTT ACTGCTAGTA GTTAATTAGT TAGAAGAGCT GTTGAGTTAG TTAGTAGTTA GTAGTTAATT   
  
  
+ AGTTAGAAAA GCTCTTGAGT TGGTTAGTAG TTAGCAGTTG AATAAAGGGG TTAGATCAAT TGTAATTAGG   
  
  
+ AAAAAACCAT TGTATATAAA CAGGTGTATG AAAACGGGTT GAGGATAAGA AAATACAGAA GATTAATCTC   
  
  
+ ATTTCTCTGC TCATTCTTCT CTGCTCTCTC TTGTTTCTGT TCTTTCTCAA TTTCTGTTCT CTTTTCTTTC   
  
  
+ TAATCTTGTA AAGCTGAGCA TATCATCACC AAGTCATCAC GTGTAGAAGC AAAGGGACAA CAAGTCATCA   
  
  
+ CCGAAGAAGA ATATAGTTGA CCTTCGAGAA CCCAGTCAA  

- CAACGACAGA AAAAAAAAAT AGTACAATAG TTTAATTACC TTTAAATTGA GATAATAGTT TTAAGAGAAA   
  
  
- AAATATTTGT ACTATTAGAA AAAATAAAAT AAAAGTTCAA TTTTTATGAA CCAGGCCGGA CCCTGCTACG   
  
  
- CGAACGGTTA GATCAGTTAG GATTTAAATG ATTATGCTCA TTCATTATCG GCTTCCTCGT TTCGAGGTCA   
  
  
- GGCCGTAGAA CAAGTCTTGT TATAATGTAG GGACTTTACG GAGCACTTAA CCCACAGAGA AGGTTGAAAG   
  
  
- AGGTCGTTAA AAGAGGGTTG AAGACGTTCG TCACTAATAG TTAAACTTAA TGTCCTCGAT GGATTGTATA   
  
  
- GGCGGGATCT TGCTTCCGTC CTTAGTAGTC GAAACGGTGG AGAAACGTAT TGACGATGAC TTTCGGTAAT   
  
  
- CGACCACTTA CGAGGTAAAG TTTGAAGGTA ACCGGGCTCA TGACGAGGAC GAAGGTAACC TTTATAGCGA   
  
  
- CGCGTTAGTC TGCTCCGTCC GTAAGTATAG ACTACTATTT ACAACGTCCT AATTAAGATA AAAGCGGCCG   
  
  
- TCGTTTACCA CCCTAAAAAA TGTGACTTTT CACAAAGTTT TCTGAGCTTA ATTACACCAG CTAAAACCGC   
  
  
- CCCCGCACCT TACCTAAGGA GAGAAGGAGT TAGTAGTTTG TAGGTCTATG GACTTTGTCT CTCTTCGGGT   
  
  
- AGAAGTTTTA GTATAGCAGT AGCCTCCAGG AAGCGACGTT AGCCGACTAC TGCCACGTGA AAAACTACCA   
  
  
- TCGCTTGGTC TAGTCAGTTA CCAGTTTAGT AAACCTTTAG TTGCTCGATG TCAAATAGGG AGGACTTAGG   
  
  
- TAGAAACTCT TGGATTTTCT TTAATTAATT TTAATTGCTT TCTCTTTTCT CTCTCCACCA TCTCAAGTAT   
  
  
- CAACAGCCTA GCCAGGCCTT TAACTAAATT AAATCTTTAA TTATATCTTT TCTCAAAACT CATTAATATA   
  
  
- ATAGAAATTG CCTCCGTTTG ATTTAATTAT AAAAATAATT TTTTTATTAA AAAAATTTTT TTTGAAAAAT   
  
  
- CTGAAAACAT ATTTAATTAG TTTAATTTAA TTGTTCTAAA ATGATTTAAT TATTAAATAA TTTAAATTGA   
  
  
- ATTTTAGATT AAATTATAGT GTCGGACGAA CTATAATATT ATAGCCAACA AAGACCAAAT TTTTATATAA   
  
  
- TTTTATAATA AAAAATTAAA AAATTTTAAA TTAAAATTGT AATTATTTAA TTTTACTAGA TTAAAATTAA   
  
  
- AAAAATTTTT TTGATTCTAA GAAAAGTTTT TGTAGAAAAA TGCCACCGAA TCTGAGATAA GCAAAAATAT   
  
  
- AAAATTTTTA AGAAAATTTT TTTAATTTTT AAAAATTAAA AAAAGTGAAA TTAAAAAAAA TATAAAAATT   
  
  
- TAATAAAATT ATACAATTAT AATTTTTATT TTAAAATTTT ATTTTTTTAT AATAAAATTA TTTAAAATTT   
  
  
- TAATTTTTAT AAAATTTTTG GTTGGTTTTT TCCGTTGGAT AATGCTTGCT TGATCCTCTG TACTACTAGC   
  
  
- GACCTTCTTC TCGTTGTTCT TCGTTACTGT AGTGACTTTC TTATTGCCAA TCAGGCCACT TAAAAGTAAT   
  
  
- CGACCTTCAA TGACGATCAT CAATTAATCA ATCTTCTCGA CAACTCAATC AATCATCAAT CATCAATTAA   
  
  
- TCAATCTTTT CGAGAACTCA ACCAATCATC AATCGTCAAC TTATTTCCCC AATCTAGTTA ACATTAATCC   
  
  
- TTTTTTGGTA ACATATATTT GTCCACATAC TTTTGCCCAA CTCCTATTCT TTTATGTCTT CTAATTAGAG   
  
  
- TAAAGAGACG AGTAAGAAGA GACGAGAGAG AACAAAGACA AGAAAGAGTT AAAGACAAGA GAAAAGAAAG   
  
  
- ATTAGAACAT TTCGACTCGT ATAGTAGTGG TTCAGTAGTG CACATCTTCG TTTCCCTGTT GTTCAGTAGT   
  
  
- GGCTTCTTCT TATATCAACT GGAAGCTCTT GGGTCAGTT

+     ABRE

| Site Name | Organism | Position | Strand | Matrix score. | sequence | function |
| --- | --- | --- | --- | --- | --- | --- |
| ABRE | Arabidopsis thaliana | 1928 | - | 6 | CACGTG | cis-acting element involved in the abscisic acid responsiveness |
| ABRE | Arabidopsis thaliana | 1929 | + | 5 | ACGTG | cis-acting element involved in the abscisic acid responsiveness |

>Potri.018G038100.1   
+ GTTGCTGTCT TTTTTTTTTA TCATGTTATC AAATTAATGG AAATTTAACT CTATTATCAA AATTCTCTTT   
  
  
+ TTTATAAACA TGATAATCTT TTTTATTTTA TTTTCAAGTT AAAAATACTT GGTCCGGCCT GGGACGATGC   
  
  
+ GCTTGCCAAT CTAGTCAATC CTAAATTTAC TAATACGAGT AAGTAATAGC CGAAGGAGCA AAGCTCCAGT   
  
  
+ CCGGCATCTT GTTCAGAACA ATATTACATC CCTGAAATGC CTCGTGAATT GGGTGTCTCT TCCAACTTTC   
  
  
+ TCCAGCAATT TTCTCCCAAC TTCTGCAAGC AGTGATTATC AATTTGAATT ACAGGAGCTA CCTAACATAT   
  
  
+ CCGCCCTAGA ACGAAGGCAG GAATCATCAG CTTTGCCACC TCTTTGCATA ACTGCTACTG AAAGCCATTA   
  
  
+ GCTGGTGAAT GCTCCATTTC AAACTTCCAT TGGCCCGAGT ACTGCTCCTG CTTCCATTGG AAATATCGCT   
  
  
+ GCGCAATCAG ACGAGGCAGG CATTCATATC TGATGATAAA TGTTGCAGGA TTAATTCTAT TTTCGCCGGC   
  
  
+ AGCAAATGGT GGGATTTTTT ACACTGAAAA GTGTTTCAAA AGACTCGAAT TAATGTGGTC GATTTTGGCG   
  
  
+ GGGGCGTGGA ATGGATTCCT CTCTTCCTCA ATCATCAAAC ATCCAGATAC CTGAAACAGA GAGAAGCCCA   
  
  
+ TCTTCAAAAT CATATCGTCA TCGGAGGTCC TTCGCTGCAA TCGGCTGATG ACGGTGCACT TTTTGATGGT   
  
  
+ AGCGAACCAG ATCAGTCAAT GGTCAAATCA TTTGGAAATC AACGAGCTAC AGTTTATCCC TCCTGAATCC   
  
  
+ ATCTTTGAGA ACCTAAAAGA AATTAATTAA AATTAACGAA AGAGAAAAGA GAGAGGTGGT AGAGTTCATA   
  
  
+ GTTGTCGGAT CGGTCCGGAA ATTGATTTAA TTTAGAAATT AATATAGAAA AGAGTTTTGA GTAATTATAT   
  
  
+ TATCTTTAAC GGAGGCAAAC TAAATTAATA TTTTTATTAA AAAAATAATT TTTTTAAAAA AAACTTTTTA   
  
  
+ GACTTTTGTA TAAATTAATC AAATTAAATT AACAAGATTT TACTAAATTA ATAATTTATT AAATTTAACT   
  
  
+ TAAAATCTAA TTTAATATCA CAGCCTGCTT GATATTATAA TATCGGTTGT TTCTGGTTTA AAAATATATT   
  
  
+ AAAATATTAT TTTTTAATTT TTTAAAATTT AATTTTAACA TTAATAAATT AAAATGATCT AATTTTAATT   
  
  
+ TTTTTAAAAA AACTAAGATT CTTTTCAAAA ACATCTTTTT ACGGTGGCTT AGACTCTATT CGTTTTTATA   
  
  
+ TTTTAAAAAT TCTTTTAAAA AAATTAAAAA TTTTTAATTT TTTTCACTTT AATTTTTTTT ATATTTTTAA   
  
  
+ ATTATTTTAA TATGTTAATA TTAAAAATAA AATTTTAAAA TAAAAAAATA TTATTTTAAT AAATTTTAAA   
  
  
+ ATTAAAAATA TTTTAAAAAC CAACCAAAAA AGGCAACCTA TTACGAACGA ACTAGGAGAC ATGATGATCG   
  
  
+ CTGGAAGAAG AGCAACAAGA AGCAATGACA TCACTGAAAG AATAACGGTT AGTCCGGTGA ATTTTCATTA   
  
  
+ GCTGGAAGTT ACTGCTAGTA GTTAATTAGT TAGAAGAGCT GTTGAGTTAG TTAGTAGTTA GTAGTTAATT   
  
  
+ AGTTAGAAAA GCTCTTGAGT TGGTTAGTAG TTAGCAGTTG AATAAAGGGG TTAGATCAAT TGTAATTAGG   
  
  
+ AAAAAACCAT TGTATATAAA CAGGTGTATG AAAACGGGTT GAGGATAAGA AAATACAGAA GATTAATCTC   
  
  
+ ATTTCTCTGC TCATTCTTCT CTGCTCTCTC TTGTTTCTGT TCTTTCTCAA TTTCTGTTCT CTTTTCTTTC   
  
  
+ TAATCTTGTA AAGCTGAGCA TATCATCACC AAGTCATCAC GTGTAGAAGC AAAGGGACAA CAAGTCATCA   
  
  
+ CCGAAGAAGA ATATAGTTGA CCTTCGAGAA CCCAGTCAA  

- CAACGACAGA AAAAAAAAAT AGTACAATAG TTTAATTACC TTTAAATTGA GATAATAGTT TTAAGAGAAA   
  
  
- AAATATTTGT ACTATTAGAA AAAATAAAAT AAAAGTTCAA TTTTTATGAA CCAGGCCGGA CCCTGCTACG   
  
  
- CGAACGGTTA GATCAGTTAG GATTTAAATG ATTATGCTCA TTCATTATCG GCTTCCTCGT TTCGAGGTCA   
  
  
- GGCCGTAGAA CAAGTCTTGT TATAATGTAG GGACTTTACG GAGCACTTAA CCCACAGAGA AGGTTGAAAG   
  
  
- AGGTCGTTAA AAGAGGGTTG AAGACGTTCG TCACTAATAG TTAAACTTAA TGTCCTCGAT GGATTGTATA   
  
  
- GGCGGGATCT TGCTTCCGTC CTTAGTAGTC GAAACGGTGG AGAAACGTAT TGACGATGAC TTTCGGTAAT   
  
  
- CGACCACTTA CGAGGTAAAG TTTGAAGGTA ACCGGGCTCA TGACGAGGAC GAAGGTAACC TTTATAGCGA   
  
  
- CGCGTTAGTC TGCTCCGTCC GTAAGTATAG ACTACTATTT ACAACGTCCT AATTAAGATA AAAGCGGCCG   
  
  
- TCGTTTACCA CCCTAAAAAA TGTGACTTTT CACAAAGTTT TCTGAGCTTA ATTACACCAG CTAAAACCGC   
  
  
- CCCCGCACCT TACCTAAGGA GAGAAGGAGT TAGTAGTTTG TAGGTCTATG GACTTTGTCT CTCTTCGGGT   
  
  
- AGAAGTTTTA GTATAGCAGT AGCCTCCAGG AAGCGACGTT AGCCGACTAC TGCCACGTGA AAAACTACCA   
  
  
- TCGCTTGGTC TAGTCAGTTA CCAGTTTAGT AAACCTTTAG TTGCTCGATG TCAAATAGGG AGGACTTAGG   
  
  
- TAGAAACTCT TGGATTTTCT TTAATTAATT TTAATTGCTT TCTCTTTTCT CTCTCCACCA TCTCAAGTAT   
  
  
- CAACAGCCTA GCCAGGCCTT TAACTAAATT AAATCTTTAA TTATATCTTT TCTCAAAACT CATTAATATA   
  
  
- ATAGAAATTG CCTCCGTTTG ATTTAATTAT AAAAATAATT TTTTTATTAA AAAAATTTTT TTTGAAAAAT   
  
  
- CTGAAAACAT ATTTAATTAG TTTAATTTAA TTGTTCTAAA ATGATTTAAT TATTAAATAA TTTAAATTGA   
  
  
- ATTTTAGATT AAATTATAGT GTCGGACGAA CTATAATATT ATAGCCAACA AAGACCAAAT TTTTATATAA   
  
  
- TTTTATAATA AAAAATTAAA AAATTTTAAA TTAAAATTGT AATTATTTAA TTTTACTAGA TTAAAATTAA   
  
  
- AAAAATTTTT TTGATTCTAA GAAAAGTTTT TGTAGAAAAA TGCCACCGAA TCTGAGATAA GCAAAAATAT   
  
  
- AAAATTTTTA AGAAAATTTT TTTAATTTTT AAAAATTAAA AAAAGTGAAA TTAAAAAAAA TATAAAAATT   
  
  
- TAATAAAATT ATACAATTAT AATTTTTATT TTAAAATTTT ATTTTTTTAT AATAAAATTA TTTAAAATTT   
  
  
- TAATTTTTAT AAAATTTTTG GTTGGTTTTT TCCGTTGGAT AATGCTTGCT TGATCCTCTG TACTACTAGC   
  
  
- GACCTTCTTC TCGTTGTTCT TCGTTACTGT AGTGACTTTC TTATTGCCAA TCAGGCCACT TAAAAGTAAT   
  
  
- CGACCTTCAA TGACGATCAT CAATTAATCA ATCTTCTCGA CAACTCAATC AATCATCAAT CATCAATTAA   
  
  
- TCAATCTTTT CGAGAACTCA ACCAATCATC AATCGTCAAC TTATTTCCCC AATCTAGTTA ACATTAATCC   
  
  
- TTTTTTGGTA ACATATATTT GTCCACATAC TTTTGCCCAA CTCCTATTCT TTTATGTCTT CTAATTAGAG   
  
  
- TAAAGAGACG AGTAAGAAGA GACGAGAGAG AACAAAGACA AGAAAGAGTT AAAGACAAGA GAAAAGAAAG   
  
  
- ATTAGAACAT TTCGACTCGT ATAGTAGTGG TTCAGTAGTG CACATCTTCG TTTCCCTGTT GTTCAGTAGT   
  
  
- GGCTTCTTCT TATATCAACT GGAAGCTCTT GGGTCAGTT

+     AE-box

| Site Name | Organism | Position | Strand | Matrix score. | sequence | function |
| --- | --- | --- | --- | --- | --- | --- |
| AE-box | Arabidopsis thaliana | 1167 | - | 8 | AGAAACAA | part of a module for light response |
| AE-box | Arabidopsis thaliana | 1851 | - | 8 | AGAAACAA | part of a module for light response |

>Potri.018G038100.1   
+ GTTGCTGTCT TTTTTTTTTA TCATGTTATC AAATTAATGG AAATTTAACT CTATTATCAA AATTCTCTTT   
  
  
+ TTTATAAACA TGATAATCTT TTTTATTTTA TTTTCAAGTT AAAAATACTT GGTCCGGCCT GGGACGATGC   
  
  
+ GCTTGCCAAT CTAGTCAATC CTAAATTTAC TAATACGAGT AAGTAATAGC CGAAGGAGCA AAGCTCCAGT   
  
  
+ CCGGCATCTT GTTCAGAACA ATATTACATC CCTGAAATGC CTCGTGAATT GGGTGTCTCT TCCAACTTTC   
  
  
+ TCCAGCAATT TTCTCCCAAC TTCTGCAAGC AGTGATTATC AATTTGAATT ACAGGAGCTA CCTAACATAT   
  
  
+ CCGCCCTAGA ACGAAGGCAG GAATCATCAG CTTTGCCACC TCTTTGCATA ACTGCTACTG AAAGCCATTA   
  
  
+ GCTGGTGAAT GCTCCATTTC AAACTTCCAT TGGCCCGAGT ACTGCTCCTG CTTCCATTGG AAATATCGCT   
  
  
+ GCGCAATCAG ACGAGGCAGG CATTCATATC TGATGATAAA TGTTGCAGGA TTAATTCTAT TTTCGCCGGC   
  
  
+ AGCAAATGGT GGGATTTTTT ACACTGAAAA GTGTTTCAAA AGACTCGAAT TAATGTGGTC GATTTTGGCG   
  
  
+ GGGGCGTGGA ATGGATTCCT CTCTTCCTCA ATCATCAAAC ATCCAGATAC CTGAAACAGA GAGAAGCCCA   
  
  
+ TCTTCAAAAT CATATCGTCA TCGGAGGTCC TTCGCTGCAA TCGGCTGATG ACGGTGCACT TTTTGATGGT   
  
  
+ AGCGAACCAG ATCAGTCAAT GGTCAAATCA TTTGGAAATC AACGAGCTAC AGTTTATCCC TCCTGAATCC   
  
  
+ ATCTTTGAGA ACCTAAAAGA AATTAATTAA AATTAACGAA AGAGAAAAGA GAGAGGTGGT AGAGTTCATA   
  
  
+ GTTGTCGGAT CGGTCCGGAA ATTGATTTAA TTTAGAAATT AATATAGAAA AGAGTTTTGA GTAATTATAT   
  
  
+ TATCTTTAAC GGAGGCAAAC TAAATTAATA TTTTTATTAA AAAAATAATT TTTTTAAAAA AAACTTTTTA   
  
  
+ GACTTTTGTA TAAATTAATC AAATTAAATT AACAAGATTT TACTAAATTA ATAATTTATT AAATTTAACT   
  
  
+ TAAAATCTAA TTTAATATCA CAGCCTGCTT GATATTATAA TATCGGTTGT TTCTGGTTTA AAAATATATT   
  
  
+ AAAATATTAT TTTTTAATTT TTTAAAATTT AATTTTAACA TTAATAAATT AAAATGATCT AATTTTAATT   
  
  
+ TTTTTAAAAA AACTAAGATT CTTTTCAAAA ACATCTTTTT ACGGTGGCTT AGACTCTATT CGTTTTTATA   
  
  
+ TTTTAAAAAT TCTTTTAAAA AAATTAAAAA TTTTTAATTT TTTTCACTTT AATTTTTTTT ATATTTTTAA   
  
  
+ ATTATTTTAA TATGTTAATA TTAAAAATAA AATTTTAAAA TAAAAAAATA TTATTTTAAT AAATTTTAAA   
  
  
+ ATTAAAAATA TTTTAAAAAC CAACCAAAAA AGGCAACCTA TTACGAACGA ACTAGGAGAC ATGATGATCG   
  
  
+ CTGGAAGAAG AGCAACAAGA AGCAATGACA TCACTGAAAG AATAACGGTT AGTCCGGTGA ATTTTCATTA   
  
  
+ GCTGGAAGTT ACTGCTAGTA GTTAATTAGT TAGAAGAGCT GTTGAGTTAG TTAGTAGTTA GTAGTTAATT   
  
  
+ AGTTAGAAAA GCTCTTGAGT TGGTTAGTAG TTAGCAGTTG AATAAAGGGG TTAGATCAAT TGTAATTAGG   
  
  
+ AAAAAACCAT TGTATATAAA CAGGTGTATG AAAACGGGTT GAGGATAAGA AAATACAGAA GATTAATCTC   
  
  
+ ATTTCTCTGC TCATTCTTCT CTGCTCTCTC TTGTTTCTGT TCTTTCTCAA TTTCTGTTCT CTTTTCTTTC   
  
  
+ TAATCTTGTA AAGCTGAGCA TATCATCACC AAGTCATCAC GTGTAGAAGC AAAGGGACAA CAAGTCATCA   
  
  
+ CCGAAGAAGA ATATAGTTGA CCTTCGAGAA CCCAGTCAA  

- CAACGACAGA AAAAAAAAAT AGTACAATAG TTTAATTACC TTTAAATTGA GATAATAGTT TTAAGAGAAA   
  
  
- AAATATTTGT ACTATTAGAA AAAATAAAAT AAAAGTTCAA TTTTTATGAA CCAGGCCGGA CCCTGCTACG   
  
  
- CGAACGGTTA GATCAGTTAG GATTTAAATG ATTATGCTCA TTCATTATCG GCTTCCTCGT TTCGAGGTCA   
  
  
- GGCCGTAGAA CAAGTCTTGT TATAATGTAG GGACTTTACG GAGCACTTAA CCCACAGAGA AGGTTGAAAG   
  
  
- AGGTCGTTAA AAGAGGGTTG AAGACGTTCG TCACTAATAG TTAAACTTAA TGTCCTCGAT GGATTGTATA   
  
  
- GGCGGGATCT TGCTTCCGTC CTTAGTAGTC GAAACGGTGG AGAAACGTAT TGACGATGAC TTTCGGTAAT   
  
  
- CGACCACTTA CGAGGTAAAG TTTGAAGGTA ACCGGGCTCA TGACGAGGAC GAAGGTAACC TTTATAGCGA   
  
  
- CGCGTTAGTC TGCTCCGTCC GTAAGTATAG ACTACTATTT ACAACGTCCT AATTAAGATA AAAGCGGCCG   
  
  
- TCGTTTACCA CCCTAAAAAA TGTGACTTTT CACAAAGTTT TCTGAGCTTA ATTACACCAG CTAAAACCGC   
  
  
- CCCCGCACCT TACCTAAGGA GAGAAGGAGT TAGTAGTTTG TAGGTCTATG GACTTTGTCT CTCTTCGGGT   
  
  
- AGAAGTTTTA GTATAGCAGT AGCCTCCAGG AAGCGACGTT AGCCGACTAC TGCCACGTGA AAAACTACCA   
  
  
- TCGCTTGGTC TAGTCAGTTA CCAGTTTAGT AAACCTTTAG TTGCTCGATG TCAAATAGGG AGGACTTAGG   
  
  
- TAGAAACTCT TGGATTTTCT TTAATTAATT TTAATTGCTT TCTCTTTTCT CTCTCCACCA TCTCAAGTAT   
  
  
- CAACAGCCTA GCCAGGCCTT TAACTAAATT AAATCTTTAA TTATATCTTT TCTCAAAACT CATTAATATA   
  
  
- ATAGAAATTG CCTCCGTTTG ATTTAATTAT AAAAATAATT TTTTTATTAA AAAAATTTTT TTTGAAAAAT   
  
  
- CTGAAAACAT ATTTAATTAG TTTAATTTAA TTGTTCTAAA ATGATTTAAT TATTAAATAA TTTAAATTGA   
  
  
- ATTTTAGATT AAATTATAGT GTCGGACGAA CTATAATATT ATAGCCAACA AAGACCAAAT TTTTATATAA   
  
  
- TTTTATAATA AAAAATTAAA AAATTTTAAA TTAAAATTGT AATTATTTAA TTTTACTAGA TTAAAATTAA   
  
  
- AAAAATTTTT TTGATTCTAA GAAAAGTTTT TGTAGAAAAA TGCCACCGAA TCTGAGATAA GCAAAAATAT   
  
  
- AAAATTTTTA AGAAAATTTT TTTAATTTTT AAAAATTAAA AAAAGTGAAA TTAAAAAAAA TATAAAAATT   
  
  
- TAATAAAATT ATACAATTAT AATTTTTATT TTAAAATTTT ATTTTTTTAT AATAAAATTA TTTAAAATTT   
  
  
- TAATTTTTAT AAAATTTTTG GTTGGTTTTT TCCGTTGGAT AATGCTTGCT TGATCCTCTG TACTACTAGC   
  
  
- GACCTTCTTC TCGTTGTTCT TCGTTACTGT AGTGACTTTC TTATTGCCAA TCAGGCCACT TAAAAGTAAT   
  
  
- CGACCTTCAA TGACGATCAT CAATTAATCA ATCTTCTCGA CAACTCAATC AATCATCAAT CATCAATTAA   
  
  
- TCAATCTTTT CGAGAACTCA ACCAATCATC AATCGTCAAC TTATTTCCCC AATCTAGTTA ACATTAATCC   
  
  
- TTTTTTGGTA ACATATATTT GTCCACATAC TTTTGCCCAA CTCCTATTCT TTTATGTCTT CTAATTAGAG   
  
  
- TAAAGAGACG AGTAAGAAGA GACGAGAGAG AACAAAGACA AGAAAGAGTT AAAGACAAGA GAAAAGAAAG   
  
  
- ATTAGAACAT TTCGACTCGT ATAGTAGTGG TTCAGTAGTG CACATCTTCG TTTCCCTGTT GTTCAGTAGT   
  
  
- GGCTTCTTCT TATATCAACT GGAAGCTCTT GGGTCAGTT

+     AP-1

| Site Name | Organism | Position | Strand | Matrix score. | sequence | function |
| --- | --- | --- | --- | --- | --- | --- |
| AP-1 | Arabidopsis thaliana | 1653 | + | 8 | TGAGTTAG |  |

>Potri.018G038100.1   
+ GTTGCTGTCT TTTTTTTTTA TCATGTTATC AAATTAATGG AAATTTAACT CTATTATCAA AATTCTCTTT   
  
  
+ TTTATAAACA TGATAATCTT TTTTATTTTA TTTTCAAGTT AAAAATACTT GGTCCGGCCT GGGACGATGC   
  
  
+ GCTTGCCAAT CTAGTCAATC CTAAATTTAC TAATACGAGT AAGTAATAGC CGAAGGAGCA AAGCTCCAGT   
  
  
+ CCGGCATCTT GTTCAGAACA ATATTACATC CCTGAAATGC CTCGTGAATT GGGTGTCTCT TCCAACTTTC   
  
  
+ TCCAGCAATT TTCTCCCAAC TTCTGCAAGC AGTGATTATC AATTTGAATT ACAGGAGCTA CCTAACATAT   
  
  
+ CCGCCCTAGA ACGAAGGCAG GAATCATCAG CTTTGCCACC TCTTTGCATA ACTGCTACTG AAAGCCATTA   
  
  
+ GCTGGTGAAT GCTCCATTTC AAACTTCCAT TGGCCCGAGT ACTGCTCCTG CTTCCATTGG AAATATCGCT   
  
  
+ GCGCAATCAG ACGAGGCAGG CATTCATATC TGATGATAAA TGTTGCAGGA TTAATTCTAT TTTCGCCGGC   
  
  
+ AGCAAATGGT GGGATTTTTT ACACTGAAAA GTGTTTCAAA AGACTCGAAT TAATGTGGTC GATTTTGGCG   
  
  
+ GGGGCGTGGA ATGGATTCCT CTCTTCCTCA ATCATCAAAC ATCCAGATAC CTGAAACAGA GAGAAGCCCA   
  
  
+ TCTTCAAAAT CATATCGTCA TCGGAGGTCC TTCGCTGCAA TCGGCTGATG ACGGTGCACT TTTTGATGGT   
  
  
+ AGCGAACCAG ATCAGTCAAT GGTCAAATCA TTTGGAAATC AACGAGCTAC AGTTTATCCC TCCTGAATCC   
  
  
+ ATCTTTGAGA ACCTAAAAGA AATTAATTAA AATTAACGAA AGAGAAAAGA GAGAGGTGGT AGAGTTCATA   
  
  
+ GTTGTCGGAT CGGTCCGGAA ATTGATTTAA TTTAGAAATT AATATAGAAA AGAGTTTTGA GTAATTATAT   
  
  
+ TATCTTTAAC GGAGGCAAAC TAAATTAATA TTTTTATTAA AAAAATAATT TTTTTAAAAA AAACTTTTTA   
  
  
+ GACTTTTGTA TAAATTAATC AAATTAAATT AACAAGATTT TACTAAATTA ATAATTTATT AAATTTAACT   
  
  
+ TAAAATCTAA TTTAATATCA CAGCCTGCTT GATATTATAA TATCGGTTGT TTCTGGTTTA AAAATATATT   
  
  
+ AAAATATTAT TTTTTAATTT TTTAAAATTT AATTTTAACA TTAATAAATT AAAATGATCT AATTTTAATT   
  
  
+ TTTTTAAAAA AACTAAGATT CTTTTCAAAA ACATCTTTTT ACGGTGGCTT AGACTCTATT CGTTTTTATA   
  
  
+ TTTTAAAAAT TCTTTTAAAA AAATTAAAAA TTTTTAATTT TTTTCACTTT AATTTTTTTT ATATTTTTAA   
  
  
+ ATTATTTTAA TATGTTAATA TTAAAAATAA AATTTTAAAA TAAAAAAATA TTATTTTAAT AAATTTTAAA   
  
  
+ ATTAAAAATA TTTTAAAAAC CAACCAAAAA AGGCAACCTA TTACGAACGA ACTAGGAGAC ATGATGATCG   
  
  
+ CTGGAAGAAG AGCAACAAGA AGCAATGACA TCACTGAAAG AATAACGGTT AGTCCGGTGA ATTTTCATTA   
  
  
+ GCTGGAAGTT ACTGCTAGTA GTTAATTAGT TAGAAGAGCT GTTGAGTTAG TTAGTAGTTA GTAGTTAATT   
  
  
+ AGTTAGAAAA GCTCTTGAGT TGGTTAGTAG TTAGCAGTTG AATAAAGGGG TTAGATCAAT TGTAATTAGG   
  
  
+ AAAAAACCAT TGTATATAAA CAGGTGTATG AAAACGGGTT GAGGATAAGA AAATACAGAA GATTAATCTC   
  
  
+ ATTTCTCTGC TCATTCTTCT CTGCTCTCTC TTGTTTCTGT TCTTTCTCAA TTTCTGTTCT CTTTTCTTTC   
  
  
+ TAATCTTGTA AAGCTGAGCA TATCATCACC AAGTCATCAC GTGTAGAAGC AAAGGGACAA CAAGTCATCA   
  
  
+ CCGAAGAAGA ATATAGTTGA CCTTCGAGAA CCCAGTCAA  

- CAACGACAGA AAAAAAAAAT AGTACAATAG TTTAATTACC TTTAAATTGA GATAATAGTT TTAAGAGAAA   
  
  
- AAATATTTGT ACTATTAGAA AAAATAAAAT AAAAGTTCAA TTTTTATGAA CCAGGCCGGA CCCTGCTACG   
  
  
- CGAACGGTTA GATCAGTTAG GATTTAAATG ATTATGCTCA TTCATTATCG GCTTCCTCGT TTCGAGGTCA   
  
  
- GGCCGTAGAA CAAGTCTTGT TATAATGTAG GGACTTTACG GAGCACTTAA CCCACAGAGA AGGTTGAAAG   
  
  
- AGGTCGTTAA AAGAGGGTTG AAGACGTTCG TCACTAATAG TTAAACTTAA TGTCCTCGAT GGATTGTATA   
  
  
- GGCGGGATCT TGCTTCCGTC CTTAGTAGTC GAAACGGTGG AGAAACGTAT TGACGATGAC TTTCGGTAAT   
  
  
- CGACCACTTA CGAGGTAAAG TTTGAAGGTA ACCGGGCTCA TGACGAGGAC GAAGGTAACC TTTATAGCGA   
  
  
- CGCGTTAGTC TGCTCCGTCC GTAAGTATAG ACTACTATTT ACAACGTCCT AATTAAGATA AAAGCGGCCG   
  
  
- TCGTTTACCA CCCTAAAAAA TGTGACTTTT CACAAAGTTT TCTGAGCTTA ATTACACCAG CTAAAACCGC   
  
  
- CCCCGCACCT TACCTAAGGA GAGAAGGAGT TAGTAGTTTG TAGGTCTATG GACTTTGTCT CTCTTCGGGT   
  
  
- AGAAGTTTTA GTATAGCAGT AGCCTCCAGG AAGCGACGTT AGCCGACTAC TGCCACGTGA AAAACTACCA   
  
  
- TCGCTTGGTC TAGTCAGTTA CCAGTTTAGT AAACCTTTAG TTGCTCGATG TCAAATAGGG AGGACTTAGG   
  
  
- TAGAAACTCT TGGATTTTCT TTAATTAATT TTAATTGCTT TCTCTTTTCT CTCTCCACCA TCTCAAGTAT   
  
  
- CAACAGCCTA GCCAGGCCTT TAACTAAATT AAATCTTTAA TTATATCTTT TCTCAAAACT CATTAATATA   
  
  
- ATAGAAATTG CCTCCGTTTG ATTTAATTAT AAAAATAATT TTTTTATTAA AAAAATTTTT TTTGAAAAAT   
  
  
- CTGAAAACAT ATTTAATTAG TTTAATTTAA TTGTTCTAAA ATGATTTAAT TATTAAATAA TTTAAATTGA   
  
  
- ATTTTAGATT AAATTATAGT GTCGGACGAA CTATAATATT ATAGCCAACA AAGACCAAAT TTTTATATAA   
  
  
- TTTTATAATA AAAAATTAAA AAATTTTAAA TTAAAATTGT AATTATTTAA TTTTACTAGA TTAAAATTAA   
  
  
- AAAAATTTTT TTGATTCTAA GAAAAGTTTT TGTAGAAAAA TGCCACCGAA TCTGAGATAA GCAAAAATAT   
  
  
- AAAATTTTTA AGAAAATTTT TTTAATTTTT AAAAATTAAA AAAAGTGAAA TTAAAAAAAA TATAAAAATT   
  
  
- TAATAAAATT ATACAATTAT AATTTTTATT TTAAAATTTT ATTTTTTTAT AATAAAATTA TTTAAAATTT   
  
  
- TAATTTTTAT AAAATTTTTG GTTGGTTTTT TCCGTTGGAT AATGCTTGCT TGATCCTCTG TACTACTAGC   
  
  
- GACCTTCTTC TCGTTGTTCT TCGTTACTGT AGTGACTTTC TTATTGCCAA TCAGGCCACT TAAAAGTAAT   
  
  
- CGACCTTCAA TGACGATCAT CAATTAATCA ATCTTCTCGA CAACTCAATC AATCATCAAT CATCAATTAA   
  
  
- TCAATCTTTT CGAGAACTCA ACCAATCATC AATCGTCAAC TTATTTCCCC AATCTAGTTA ACATTAATCC   
  
  
- TTTTTTGGTA ACATATATTT GTCCACATAC TTTTGCCCAA CTCCTATTCT TTTATGTCTT CTAATTAGAG   
  
  
- TAAAGAGACG AGTAAGAAGA GACGAGAGAG AACAAAGACA AGAAAGAGTT AAAGACAAGA GAAAAGAAAG   
  
  
- ATTAGAACAT TTCGACTCGT ATAGTAGTGG TTCAGTAGTG CACATCTTCG TTTCCCTGTT GTTCAGTAGT   
  
  
- GGCTTCTTCT TATATCAACT GGAAGCTCTT GGGTCAGTT

+     ARE

| Site Name | Organism | Position | Strand | Matrix score. | sequence | function |
| --- | --- | --- | --- | --- | --- | --- |
| ARE | Zea mays | 1754 | + | 6 | AAACCA | cis-acting regulatory element essential for the anaerobic induction |
| ARE | Zea mays | 1174 | - | 6 | AAACCA | cis-acting regulatory element essential for the anaerobic induction |
| ARE | Zea mays | 1487 | + | 6 | AAACCA | cis-acting regulatory element essential for the anaerobic induction |

>Potri.018G038100.1   
+ GTTGCTGTCT TTTTTTTTTA TCATGTTATC AAATTAATGG AAATTTAACT CTATTATCAA AATTCTCTTT   
  
  
+ TTTATAAACA TGATAATCTT TTTTATTTTA TTTTCAAGTT AAAAATACTT GGTCCGGCCT GGGACGATGC   
  
  
+ GCTTGCCAAT CTAGTCAATC CTAAATTTAC TAATACGAGT AAGTAATAGC CGAAGGAGCA AAGCTCCAGT   
  
  
+ CCGGCATCTT GTTCAGAACA ATATTACATC CCTGAAATGC CTCGTGAATT GGGTGTCTCT TCCAACTTTC   
  
  
+ TCCAGCAATT TTCTCCCAAC TTCTGCAAGC AGTGATTATC AATTTGAATT ACAGGAGCTA CCTAACATAT   
  
  
+ CCGCCCTAGA ACGAAGGCAG GAATCATCAG CTTTGCCACC TCTTTGCATA ACTGCTACTG AAAGCCATTA   
  
  
+ GCTGGTGAAT GCTCCATTTC AAACTTCCAT TGGCCCGAGT ACTGCTCCTG CTTCCATTGG AAATATCGCT   
  
  
+ GCGCAATCAG ACGAGGCAGG CATTCATATC TGATGATAAA TGTTGCAGGA TTAATTCTAT TTTCGCCGGC   
  
  
+ AGCAAATGGT GGGATTTTTT ACACTGAAAA GTGTTTCAAA AGACTCGAAT TAATGTGGTC GATTTTGGCG   
  
  
+ GGGGCGTGGA ATGGATTCCT CTCTTCCTCA ATCATCAAAC ATCCAGATAC CTGAAACAGA GAGAAGCCCA   
  
  
+ TCTTCAAAAT CATATCGTCA TCGGAGGTCC TTCGCTGCAA TCGGCTGATG ACGGTGCACT TTTTGATGGT   
  
  
+ AGCGAACCAG ATCAGTCAAT GGTCAAATCA TTTGGAAATC AACGAGCTAC AGTTTATCCC TCCTGAATCC   
  
  
+ ATCTTTGAGA ACCTAAAAGA AATTAATTAA AATTAACGAA AGAGAAAAGA GAGAGGTGGT AGAGTTCATA   
  
  
+ GTTGTCGGAT CGGTCCGGAA ATTGATTTAA TTTAGAAATT AATATAGAAA AGAGTTTTGA GTAATTATAT   
  
  
+ TATCTTTAAC GGAGGCAAAC TAAATTAATA TTTTTATTAA AAAAATAATT TTTTTAAAAA AAACTTTTTA   
  
  
+ GACTTTTGTA TAAATTAATC AAATTAAATT AACAAGATTT TACTAAATTA ATAATTTATT AAATTTAACT   
  
  
+ TAAAATCTAA TTTAATATCA CAGCCTGCTT GATATTATAA TATCGGTTGT TTCTGGTTTA AAAATATATT   
  
  
+ AAAATATTAT TTTTTAATTT TTTAAAATTT AATTTTAACA TTAATAAATT AAAATGATCT AATTTTAATT   
  
  
+ TTTTTAAAAA AACTAAGATT CTTTTCAAAA ACATCTTTTT ACGGTGGCTT AGACTCTATT CGTTTTTATA   
  
  
+ TTTTAAAAAT TCTTTTAAAA AAATTAAAAA TTTTTAATTT TTTTCACTTT AATTTTTTTT ATATTTTTAA   
  
  
+ ATTATTTTAA TATGTTAATA TTAAAAATAA AATTTTAAAA TAAAAAAATA TTATTTTAAT AAATTTTAAA   
  
  
+ ATTAAAAATA TTTTAAAAAC CAACCAAAAA AGGCAACCTA TTACGAACGA ACTAGGAGAC ATGATGATCG   
  
  
+ CTGGAAGAAG AGCAACAAGA AGCAATGACA TCACTGAAAG AATAACGGTT AGTCCGGTGA ATTTTCATTA   
  
  
+ GCTGGAAGTT ACTGCTAGTA GTTAATTAGT TAGAAGAGCT GTTGAGTTAG TTAGTAGTTA GTAGTTAATT   
  
  
+ AGTTAGAAAA GCTCTTGAGT TGGTTAGTAG TTAGCAGTTG AATAAAGGGG TTAGATCAAT TGTAATTAGG   
  
  
+ AAAAAACCAT TGTATATAAA CAGGTGTATG AAAACGGGTT GAGGATAAGA AAATACAGAA GATTAATCTC   
  
  
+ ATTTCTCTGC TCATTCTTCT CTGCTCTCTC TTGTTTCTGT TCTTTCTCAA TTTCTGTTCT CTTTTCTTTC   
  
  
+ TAATCTTGTA AAGCTGAGCA TATCATCACC AAGTCATCAC GTGTAGAAGC AAAGGGACAA CAAGTCATCA   
  
  
+ CCGAAGAAGA ATATAGTTGA CCTTCGAGAA CCCAGTCAA  

- CAACGACAGA AAAAAAAAAT AGTACAATAG TTTAATTACC TTTAAATTGA GATAATAGTT TTAAGAGAAA   
  
  
- AAATATTTGT ACTATTAGAA AAAATAAAAT AAAAGTTCAA TTTTTATGAA CCAGGCCGGA CCCTGCTACG   
  
  
- CGAACGGTTA GATCAGTTAG GATTTAAATG ATTATGCTCA TTCATTATCG GCTTCCTCGT TTCGAGGTCA   
  
  
- GGCCGTAGAA CAAGTCTTGT TATAATGTAG GGACTTTACG GAGCACTTAA CCCACAGAGA AGGTTGAAAG   
  
  
- AGGTCGTTAA AAGAGGGTTG AAGACGTTCG TCACTAATAG TTAAACTTAA TGTCCTCGAT GGATTGTATA   
  
  
- GGCGGGATCT TGCTTCCGTC CTTAGTAGTC GAAACGGTGG AGAAACGTAT TGACGATGAC TTTCGGTAAT   
  
  
- CGACCACTTA CGAGGTAAAG TTTGAAGGTA ACCGGGCTCA TGACGAGGAC GAAGGTAACC TTTATAGCGA   
  
  
- CGCGTTAGTC TGCTCCGTCC GTAAGTATAG ACTACTATTT ACAACGTCCT AATTAAGATA AAAGCGGCCG   
  
  
- TCGTTTACCA CCCTAAAAAA TGTGACTTTT CACAAAGTTT TCTGAGCTTA ATTACACCAG CTAAAACCGC   
  
  
- CCCCGCACCT TACCTAAGGA GAGAAGGAGT TAGTAGTTTG TAGGTCTATG GACTTTGTCT CTCTTCGGGT   
  
  
- AGAAGTTTTA GTATAGCAGT AGCCTCCAGG AAGCGACGTT AGCCGACTAC TGCCACGTGA AAAACTACCA   
  
  
- TCGCTTGGTC TAGTCAGTTA CCAGTTTAGT AAACCTTTAG TTGCTCGATG TCAAATAGGG AGGACTTAGG   
  
  
- TAGAAACTCT TGGATTTTCT TTAATTAATT TTAATTGCTT TCTCTTTTCT CTCTCCACCA TCTCAAGTAT   
  
  
- CAACAGCCTA GCCAGGCCTT TAACTAAATT AAATCTTTAA TTATATCTTT TCTCAAAACT CATTAATATA   
  
  
- ATAGAAATTG CCTCCGTTTG ATTTAATTAT AAAAATAATT TTTTTATTAA AAAAATTTTT TTTGAAAAAT   
  
  
- CTGAAAACAT ATTTAATTAG TTTAATTTAA TTGTTCTAAA ATGATTTAAT TATTAAATAA TTTAAATTGA   
  
  
- ATTTTAGATT AAATTATAGT GTCGGACGAA CTATAATATT ATAGCCAACA AAGACCAAAT TTTTATATAA   
  
  
- TTTTATAATA AAAAATTAAA AAATTTTAAA TTAAAATTGT AATTATTTAA TTTTACTAGA TTAAAATTAA   
  
  
- AAAAATTTTT TTGATTCTAA GAAAAGTTTT TGTAGAAAAA TGCCACCGAA TCTGAGATAA GCAAAAATAT   
  
  
- AAAATTTTTA AGAAAATTTT TTTAATTTTT AAAAATTAAA AAAAGTGAAA TTAAAAAAAA TATAAAAATT   
  
  
- TAATAAAATT ATACAATTAT AATTTTTATT TTAAAATTTT ATTTTTTTAT AATAAAATTA TTTAAAATTT   
  
  
- TAATTTTTAT AAAATTTTTG GTTGGTTTTT TCCGTTGGAT AATGCTTGCT TGATCCTCTG TACTACTAGC   
  
  
- GACCTTCTTC TCGTTGTTCT TCGTTACTGT AGTGACTTTC TTATTGCCAA TCAGGCCACT TAAAAGTAAT   
  
  
- CGACCTTCAA TGACGATCAT CAATTAATCA ATCTTCTCGA CAACTCAATC AATCATCAAT CATCAATTAA   
  
  
- TCAATCTTTT CGAGAACTCA ACCAATCATC AATCGTCAAC TTATTTCCCC AATCTAGTTA ACATTAATCC   
  
  
- TTTTTTGGTA ACATATATTT GTCCACATAC TTTTGCCCAA CTCCTATTCT TTTATGTCTT CTAATTAGAG   
  
  
- TAAAGAGACG AGTAAGAAGA GACGAGAGAG AACAAAGACA AGAAAGAGTT AAAGACAAGA GAAAAGAAAG   
  
  
- ATTAGAACAT TTCGACTCGT ATAGTAGTGG TTCAGTAGTG CACATCTTCG TTTCCCTGTT GTTCAGTAGT   
  
  
- GGCTTCTTCT TATATCAACT GGAAGCTCTT GGGTCAGTT

+     AT~TATA-box

| Site Name | Organism | Position | Strand | Matrix score. | sequence | function |
| --- | --- | --- | --- | --- | --- | --- |
| AT~TATA-box | Arabidopsis thaliana | 1763 | - | 6 | TATATA |  |

>Potri.018G038100.1   
+ GTTGCTGTCT TTTTTTTTTA TCATGTTATC AAATTAATGG AAATTTAACT CTATTATCAA AATTCTCTTT   
  
  
+ TTTATAAACA TGATAATCTT TTTTATTTTA TTTTCAAGTT AAAAATACTT GGTCCGGCCT GGGACGATGC   
  
  
+ GCTTGCCAAT CTAGTCAATC CTAAATTTAC TAATACGAGT AAGTAATAGC CGAAGGAGCA AAGCTCCAGT   
  
  
+ CCGGCATCTT GTTCAGAACA ATATTACATC CCTGAAATGC CTCGTGAATT GGGTGTCTCT TCCAACTTTC   
  
  
+ TCCAGCAATT TTCTCCCAAC TTCTGCAAGC AGTGATTATC AATTTGAATT ACAGGAGCTA CCTAACATAT   
  
  
+ CCGCCCTAGA ACGAAGGCAG GAATCATCAG CTTTGCCACC TCTTTGCATA ACTGCTACTG AAAGCCATTA   
  
  
+ GCTGGTGAAT GCTCCATTTC AAACTTCCAT TGGCCCGAGT ACTGCTCCTG CTTCCATTGG AAATATCGCT   
  
  
+ GCGCAATCAG ACGAGGCAGG CATTCATATC TGATGATAAA TGTTGCAGGA TTAATTCTAT TTTCGCCGGC   
  
  
+ AGCAAATGGT GGGATTTTTT ACACTGAAAA GTGTTTCAAA AGACTCGAAT TAATGTGGTC GATTTTGGCG   
  
  
+ GGGGCGTGGA ATGGATTCCT CTCTTCCTCA ATCATCAAAC ATCCAGATAC CTGAAACAGA GAGAAGCCCA   
  
  
+ TCTTCAAAAT CATATCGTCA TCGGAGGTCC TTCGCTGCAA TCGGCTGATG ACGGTGCACT TTTTGATGGT   
  
  
+ AGCGAACCAG ATCAGTCAAT GGTCAAATCA TTTGGAAATC AACGAGCTAC AGTTTATCCC TCCTGAATCC   
  
  
+ ATCTTTGAGA ACCTAAAAGA AATTAATTAA AATTAACGAA AGAGAAAAGA GAGAGGTGGT AGAGTTCATA   
  
  
+ GTTGTCGGAT CGGTCCGGAA ATTGATTTAA TTTAGAAATT AATATAGAAA AGAGTTTTGA GTAATTATAT   
  
  
+ TATCTTTAAC GGAGGCAAAC TAAATTAATA TTTTTATTAA AAAAATAATT TTTTTAAAAA AAACTTTTTA   
  
  
+ GACTTTTGTA TAAATTAATC AAATTAAATT AACAAGATTT TACTAAATTA ATAATTTATT AAATTTAACT   
  
  
+ TAAAATCTAA TTTAATATCA CAGCCTGCTT GATATTATAA TATCGGTTGT TTCTGGTTTA AAAATATATT   
  
  
+ AAAATATTAT TTTTTAATTT TTTAAAATTT AATTTTAACA TTAATAAATT AAAATGATCT AATTTTAATT   
  
  
+ TTTTTAAAAA AACTAAGATT CTTTTCAAAA ACATCTTTTT ACGGTGGCTT AGACTCTATT CGTTTTTATA   
  
  
+ TTTTAAAAAT TCTTTTAAAA AAATTAAAAA TTTTTAATTT TTTTCACTTT AATTTTTTTT ATATTTTTAA   
  
  
+ ATTATTTTAA TATGTTAATA TTAAAAATAA AATTTTAAAA TAAAAAAATA TTATTTTAAT AAATTTTAAA   
  
  
+ ATTAAAAATA TTTTAAAAAC CAACCAAAAA AGGCAACCTA TTACGAACGA ACTAGGAGAC ATGATGATCG   
  
  
+ CTGGAAGAAG AGCAACAAGA AGCAATGACA TCACTGAAAG AATAACGGTT AGTCCGGTGA ATTTTCATTA   
  
  
+ GCTGGAAGTT ACTGCTAGTA GTTAATTAGT TAGAAGAGCT GTTGAGTTAG TTAGTAGTTA GTAGTTAATT   
  
  
+ AGTTAGAAAA GCTCTTGAGT TGGTTAGTAG TTAGCAGTTG AATAAAGGGG TTAGATCAAT TGTAATTAGG   
  
  
+ AAAAAACCAT TGTATATAAA CAGGTGTATG AAAACGGGTT GAGGATAAGA AAATACAGAA GATTAATCTC   
  
  
+ ATTTCTCTGC TCATTCTTCT CTGCTCTCTC TTGTTTCTGT TCTTTCTCAA TTTCTGTTCT CTTTTCTTTC   
  
  
+ TAATCTTGTA AAGCTGAGCA TATCATCACC AAGTCATCAC GTGTAGAAGC AAAGGGACAA CAAGTCATCA   
  
  
+ CCGAAGAAGA ATATAGTTGA CCTTCGAGAA CCCAGTCAA  

- CAACGACAGA AAAAAAAAAT AGTACAATAG TTTAATTACC TTTAAATTGA GATAATAGTT TTAAGAGAAA   
  
  
- AAATATTTGT ACTATTAGAA AAAATAAAAT AAAAGTTCAA TTTTTATGAA CCAGGCCGGA CCCTGCTACG   
  
  
- CGAACGGTTA GATCAGTTAG GATTTAAATG ATTATGCTCA TTCATTATCG GCTTCCTCGT TTCGAGGTCA   
  
  
- GGCCGTAGAA CAAGTCTTGT TATAATGTAG GGACTTTACG GAGCACTTAA CCCACAGAGA AGGTTGAAAG   
  
  
- AGGTCGTTAA AAGAGGGTTG AAGACGTTCG TCACTAATAG TTAAACTTAA TGTCCTCGAT GGATTGTATA   
  
  
- GGCGGGATCT TGCTTCCGTC CTTAGTAGTC GAAACGGTGG AGAAACGTAT TGACGATGAC TTTCGGTAAT   
  
  
- CGACCACTTA CGAGGTAAAG TTTGAAGGTA ACCGGGCTCA TGACGAGGAC GAAGGTAACC TTTATAGCGA   
  
  
- CGCGTTAGTC TGCTCCGTCC GTAAGTATAG ACTACTATTT ACAACGTCCT AATTAAGATA AAAGCGGCCG   
  
  
- TCGTTTACCA CCCTAAAAAA TGTGACTTTT CACAAAGTTT TCTGAGCTTA ATTACACCAG CTAAAACCGC   
  
  
- CCCCGCACCT TACCTAAGGA GAGAAGGAGT TAGTAGTTTG TAGGTCTATG GACTTTGTCT CTCTTCGGGT   
  
  
- AGAAGTTTTA GTATAGCAGT AGCCTCCAGG AAGCGACGTT AGCCGACTAC TGCCACGTGA AAAACTACCA   
  
  
- TCGCTTGGTC TAGTCAGTTA CCAGTTTAGT AAACCTTTAG TTGCTCGATG TCAAATAGGG AGGACTTAGG   
  
  
- TAGAAACTCT TGGATTTTCT TTAATTAATT TTAATTGCTT TCTCTTTTCT CTCTCCACCA TCTCAAGTAT   
  
  
- CAACAGCCTA GCCAGGCCTT TAACTAAATT AAATCTTTAA TTATATCTTT TCTCAAAACT CATTAATATA   
  
  
- ATAGAAATTG CCTCCGTTTG ATTTAATTAT AAAAATAATT TTTTTATTAA AAAAATTTTT TTTGAAAAAT   
  
  
- CTGAAAACAT ATTTAATTAG TTTAATTTAA TTGTTCTAAA ATGATTTAAT TATTAAATAA TTTAAATTGA   
  
  
- ATTTTAGATT AAATTATAGT GTCGGACGAA CTATAATATT ATAGCCAACA AAGACCAAAT TTTTATATAA   
  
  
- TTTTATAATA AAAAATTAAA AAATTTTAAA TTAAAATTGT AATTATTTAA TTTTACTAGA TTAAAATTAA   
  
  
- AAAAATTTTT TTGATTCTAA GAAAAGTTTT TGTAGAAAAA TGCCACCGAA TCTGAGATAA GCAAAAATAT   
  
  
- AAAATTTTTA AGAAAATTTT TTTAATTTTT AAAAATTAAA AAAAGTGAAA TTAAAAAAAA TATAAAAATT   
  
  
- TAATAAAATT ATACAATTAT AATTTTTATT TTAAAATTTT ATTTTTTTAT AATAAAATTA TTTAAAATTT   
  
  
- TAATTTTTAT AAAATTTTTG GTTGGTTTTT TCCGTTGGAT AATGCTTGCT TGATCCTCTG TACTACTAGC   
  
  
- GACCTTCTTC TCGTTGTTCT TCGTTACTGT AGTGACTTTC TTATTGCCAA TCAGGCCACT TAAAAGTAAT   
  
  
- CGACCTTCAA TGACGATCAT CAATTAATCA ATCTTCTCGA CAACTCAATC AATCATCAAT CATCAATTAA   
  
  
- TCAATCTTTT CGAGAACTCA ACCAATCATC AATCGTCAAC TTATTTCCCC AATCTAGTTA ACATTAATCC   
  
  
- TTTTTTGGTA ACATATATTT GTCCACATAC TTTTGCCCAA CTCCTATTCT TTTATGTCTT CTAATTAGAG   
  
  
- TAAAGAGACG AGTAAGAAGA GACGAGAGAG AACAAAGACA AGAAAGAGTT AAAGACAAGA GAAAAGAAAG   
  
  
- ATTAGAACAT TTCGACTCGT ATAGTAGTGG TTCAGTAGTG CACATCTTCG TTTCCCTGTT GTTCAGTAGT   
  
  
- GGCTTCTTCT TATATCAACT GGAAGCTCTT GGGTCAGTT

+     Box 4

| Site Name | Organism | Position | Strand | Matrix score. | sequence | function |
| --- | --- | --- | --- | --- | --- | --- |
| Box 4 | Petroselinum crispum | 1812 | - | 6 | ATTAAT | part of a conserved DNA module involved in light responsiveness |
| Box 4 | Petroselinum crispum | 1230 | - | 6 | ATTAAT | part of a conserved DNA module involved in light responsiveness |
| Box 4 | Petroselinum crispum | 1097 | - | 6 | ATTAAT | part of a conserved DNA module involved in light responsiveness |
| Box 4 | Petroselinum crispum | 1064 | - | 6 | ATTAAT | part of a conserved DNA module involved in light responsiveness |
| Box 4 | Petroselinum crispum | 862 | + | 6 | ATTAAT | part of a conserved DNA module involved in light responsiveness |
| Box 4 | Petroselinum crispum | 609 | + | 6 | ATTAAT | part of a conserved DNA module involved in light responsiveness |
| Box 4 | Petroselinum crispum | 948 | + | 6 | ATTAAT | part of a conserved DNA module involved in light responsiveness |
| Box 4 | Petroselinum crispum | 33 | + | 6 | ATTAAT | part of a conserved DNA module involved in light responsiveness |
| Box 4 | Petroselinum crispum | 540 | + | 6 | ATTAAT | part of a conserved DNA module involved in light responsiveness |
| Box 4 | Petroselinum crispum | 1004 | - | 6 | ATTAAT | part of a conserved DNA module involved in light responsiveness |

>Potri.018G038100.1   
+ GTTGCTGTCT TTTTTTTTTA TCATGTTATC AAATTAATGG AAATTTAACT CTATTATCAA AATTCTCTTT   
  
  
+ TTTATAAACA TGATAATCTT TTTTATTTTA TTTTCAAGTT AAAAATACTT GGTCCGGCCT GGGACGATGC   
  
  
+ GCTTGCCAAT CTAGTCAATC CTAAATTTAC TAATACGAGT AAGTAATAGC CGAAGGAGCA AAGCTCCAGT   
  
  
+ CCGGCATCTT GTTCAGAACA ATATTACATC CCTGAAATGC CTCGTGAATT GGGTGTCTCT TCCAACTTTC   
  
  
+ TCCAGCAATT TTCTCCCAAC TTCTGCAAGC AGTGATTATC AATTTGAATT ACAGGAGCTA CCTAACATAT   
  
  
+ CCGCCCTAGA ACGAAGGCAG GAATCATCAG CTTTGCCACC TCTTTGCATA ACTGCTACTG AAAGCCATTA   
  
  
+ GCTGGTGAAT GCTCCATTTC AAACTTCCAT TGGCCCGAGT ACTGCTCCTG CTTCCATTGG AAATATCGCT   
  
  
+ GCGCAATCAG ACGAGGCAGG CATTCATATC TGATGATAAA TGTTGCAGGA TTAATTCTAT TTTCGCCGGC   
  
  
+ AGCAAATGGT GGGATTTTTT ACACTGAAAA GTGTTTCAAA AGACTCGAAT TAATGTGGTC GATTTTGGCG   
  
  
+ GGGGCGTGGA ATGGATTCCT CTCTTCCTCA ATCATCAAAC ATCCAGATAC CTGAAACAGA GAGAAGCCCA   
  
  
+ TCTTCAAAAT CATATCGTCA TCGGAGGTCC TTCGCTGCAA TCGGCTGATG ACGGTGCACT TTTTGATGGT   
  
  
+ AGCGAACCAG ATCAGTCAAT GGTCAAATCA TTTGGAAATC AACGAGCTAC AGTTTATCCC TCCTGAATCC   
  
  
+ ATCTTTGAGA ACCTAAAAGA AATTAATTAA AATTAACGAA AGAGAAAAGA GAGAGGTGGT AGAGTTCATA   
  
  
+ GTTGTCGGAT CGGTCCGGAA ATTGATTTAA TTTAGAAATT AATATAGAAA AGAGTTTTGA GTAATTATAT   
  
  
+ TATCTTTAAC GGAGGCAAAC TAAATTAATA TTTTTATTAA AAAAATAATT TTTTTAAAAA AAACTTTTTA   
  
  
+ GACTTTTGTA TAAATTAATC AAATTAAATT AACAAGATTT TACTAAATTA ATAATTTATT AAATTTAACT   
  
  
+ TAAAATCTAA TTTAATATCA CAGCCTGCTT GATATTATAA TATCGGTTGT TTCTGGTTTA AAAATATATT   
  
  
+ AAAATATTAT TTTTTAATTT TTTAAAATTT AATTTTAACA TTAATAAATT AAAATGATCT AATTTTAATT   
  
  
+ TTTTTAAAAA AACTAAGATT CTTTTCAAAA ACATCTTTTT ACGGTGGCTT AGACTCTATT CGTTTTTATA   
  
  
+ TTTTAAAAAT TCTTTTAAAA AAATTAAAAA TTTTTAATTT TTTTCACTTT AATTTTTTTT ATATTTTTAA   
  
  
+ ATTATTTTAA TATGTTAATA TTAAAAATAA AATTTTAAAA TAAAAAAATA TTATTTTAAT AAATTTTAAA   
  
  
+ ATTAAAAATA TTTTAAAAAC CAACCAAAAA AGGCAACCTA TTACGAACGA ACTAGGAGAC ATGATGATCG   
  
  
+ CTGGAAGAAG AGCAACAAGA AGCAATGACA TCACTGAAAG AATAACGGTT AGTCCGGTGA ATTTTCATTA   
  
  
+ GCTGGAAGTT ACTGCTAGTA GTTAATTAGT TAGAAGAGCT GTTGAGTTAG TTAGTAGTTA GTAGTTAATT   
  
  
+ AGTTAGAAAA GCTCTTGAGT TGGTTAGTAG TTAGCAGTTG AATAAAGGGG TTAGATCAAT TGTAATTAGG   
  
  
+ AAAAAACCAT TGTATATAAA CAGGTGTATG AAAACGGGTT GAGGATAAGA AAATACAGAA GATTAATCTC   
  
  
+ ATTTCTCTGC TCATTCTTCT CTGCTCTCTC TTGTTTCTGT TCTTTCTCAA TTTCTGTTCT CTTTTCTTTC   
  
  
+ TAATCTTGTA AAGCTGAGCA TATCATCACC AAGTCATCAC GTGTAGAAGC AAAGGGACAA CAAGTCATCA   
  
  
+ CCGAAGAAGA ATATAGTTGA CCTTCGAGAA CCCAGTCAA  

- CAACGACAGA AAAAAAAAAT AGTACAATAG TTTAATTACC TTTAAATTGA GATAATAGTT TTAAGAGAAA   
  
  
- AAATATTTGT ACTATTAGAA AAAATAAAAT AAAAGTTCAA TTTTTATGAA CCAGGCCGGA CCCTGCTACG   
  
  
- CGAACGGTTA GATCAGTTAG GATTTAAATG ATTATGCTCA TTCATTATCG GCTTCCTCGT TTCGAGGTCA   
  
  
- GGCCGTAGAA CAAGTCTTGT TATAATGTAG GGACTTTACG GAGCACTTAA CCCACAGAGA AGGTTGAAAG   
  
  
- AGGTCGTTAA AAGAGGGTTG AAGACGTTCG TCACTAATAG TTAAACTTAA TGTCCTCGAT GGATTGTATA   
  
  
- GGCGGGATCT TGCTTCCGTC CTTAGTAGTC GAAACGGTGG AGAAACGTAT TGACGATGAC TTTCGGTAAT   
  
  
- CGACCACTTA CGAGGTAAAG TTTGAAGGTA ACCGGGCTCA TGACGAGGAC GAAGGTAACC TTTATAGCGA   
  
  
- CGCGTTAGTC TGCTCCGTCC GTAAGTATAG ACTACTATTT ACAACGTCCT AATTAAGATA AAAGCGGCCG   
  
  
- TCGTTTACCA CCCTAAAAAA TGTGACTTTT CACAAAGTTT TCTGAGCTTA ATTACACCAG CTAAAACCGC   
  
  
- CCCCGCACCT TACCTAAGGA GAGAAGGAGT TAGTAGTTTG TAGGTCTATG GACTTTGTCT CTCTTCGGGT   
  
  
- AGAAGTTTTA GTATAGCAGT AGCCTCCAGG AAGCGACGTT AGCCGACTAC TGCCACGTGA AAAACTACCA   
  
  
- TCGCTTGGTC TAGTCAGTTA CCAGTTTAGT AAACCTTTAG TTGCTCGATG TCAAATAGGG AGGACTTAGG   
  
  
- TAGAAACTCT TGGATTTTCT TTAATTAATT TTAATTGCTT TCTCTTTTCT CTCTCCACCA TCTCAAGTAT   
  
  
- CAACAGCCTA GCCAGGCCTT TAACTAAATT AAATCTTTAA TTATATCTTT TCTCAAAACT CATTAATATA   
  
  
- ATAGAAATTG CCTCCGTTTG ATTTAATTAT AAAAATAATT TTTTTATTAA AAAAATTTTT TTTGAAAAAT   
  
  
- CTGAAAACAT ATTTAATTAG TTTAATTTAA TTGTTCTAAA ATGATTTAAT TATTAAATAA TTTAAATTGA   
  
  
- ATTTTAGATT AAATTATAGT GTCGGACGAA CTATAATATT ATAGCCAACA AAGACCAAAT TTTTATATAA   
  
  
- TTTTATAATA AAAAATTAAA AAATTTTAAA TTAAAATTGT AATTATTTAA TTTTACTAGA TTAAAATTAA   
  
  
- AAAAATTTTT TTGATTCTAA GAAAAGTTTT TGTAGAAAAA TGCCACCGAA TCTGAGATAA GCAAAAATAT   
  
  
- AAAATTTTTA AGAAAATTTT TTTAATTTTT AAAAATTAAA AAAAGTGAAA TTAAAAAAAA TATAAAAATT   
  
  
- TAATAAAATT ATACAATTAT AATTTTTATT TTAAAATTTT ATTTTTTTAT AATAAAATTA TTTAAAATTT   
  
  
- TAATTTTTAT AAAATTTTTG GTTGGTTTTT TCCGTTGGAT AATGCTTGCT TGATCCTCTG TACTACTAGC   
  
  
- GACCTTCTTC TCGTTGTTCT TCGTTACTGT AGTGACTTTC TTATTGCCAA TCAGGCCACT TAAAAGTAAT   
  
  
- CGACCTTCAA TGACGATCAT CAATTAATCA ATCTTCTCGA CAACTCAATC AATCATCAAT CATCAATTAA   
  
  
- TCAATCTTTT CGAGAACTCA ACCAATCATC AATCGTCAAC TTATTTCCCC AATCTAGTTA ACATTAATCC   
  
  
- TTTTTTGGTA ACATATATTT GTCCACATAC TTTTGCCCAA CTCCTATTCT TTTATGTCTT CTAATTAGAG   
  
  
- TAAAGAGACG AGTAAGAAGA GACGAGAGAG AACAAAGACA AGAAAGAGTT AAAGACAAGA GAAAAGAAAG   
  
  
- ATTAGAACAT TTCGACTCGT ATAGTAGTGG TTCAGTAGTG CACATCTTCG TTTCCCTGTT GTTCAGTAGT   
  
  
- GGCTTCTTCT TATATCAACT GGAAGCTCTT GGGTCAGTT

+     CAAT-box

| Site Name | Organism | Position | Strand | Matrix score. | sequence | function |
| --- | --- | --- | --- | --- | --- | --- |
| CAAT-box | Nicotiana glutinosa | 1997 | + | 4 | CAAT |  |
| CAAT-box | Nicotiana glutinosa | 1868 | + | 4 | CAAT |  |
| CAAT-box | Nicotiana glutinosa | 1737 | + | 4 | CAAT |  |
| CAAT-box | Nicotiana glutinosa | 1739 | - | 4 | CAAT |  |
| CAAT-box | Pisum sativum | 1070 | + | 5 | CAAAT | common cis-acting element in promoter and enhancer regions |
| CAAT-box | Pisum sativum | 800 | - | 5 | CAAAT | common cis-acting element in promoter and enhancer regions |
| CAAT-box | Nicotiana glutinosa | 659 | + | 4 | CAAT |  |
| CAAT-box | Nicotiana glutinosa | 1563 | + | 4 | CAAT |  |
| CAAT-box | Nicotiana glutinosa | 931 | - | 4 | CAAT |  |
| CAAT-box | Nicotiana glutinosa | 738 | + | 4 | CAAT |  |
| CAAT-box | Nicotiana glutinosa | 787 | + | 4 | CAAT |  |
| CAAT-box | Nicotiana glutinosa | 494 | + | 4 | CAAT |  |
| CAAT-box | Arabidopsis thaliana | 449 | - | 5 | CCAAT | common cis-acting element in promoter and enhancer regions |
| CAAT-box | Nicotiana glutinosa | 1759 | - | 4 | CAAT |  |
| CAAT-box | Nicotiana glutinosa | 229 | + | 4 | CAAT |  |
| CAAT-box | Nicotiana glutinosa | 320 | + | 4 | CAAT |  |
| CAAT-box | Pisum sativum | 794 | + | 5 | CAAAT | common cis-acting element in promoter and enhancer regions |
| CAAT-box | Pisum sativum | 30 | + | 5 | CAAAT | common cis-acting element in promoter and enhancer regions |
| CAAT-box | Nicotiana glutinosa | 147 | + | 4 | CAAT |  |
| CAAT-box | Pisum sativum | 322 | - | 5 | CAAAT | common cis-acting element in promoter and enhancer regions |
| CAAT-box | Arabidopsis thaliana | 258 | - | 5 | CCAAT | common cis-acting element in promoter and enhancer regions |
| CAAT-box | Nicotiana glutinosa | 286 | + | 4 | CAAT |  |
| CAAT-box | Pisum sativum | 563 | + | 5 | CAAAT | common cis-acting element in promoter and enhancer regions |
| CAAT-box | Arabidopsis thaliana | 146 | + | 5 | CCAAT | common cis-acting element in promoter and enhancer regions |
| CAAT-box | Arabidopsis thaliana | 476 | - | 5 | CCAAT | common cis-acting element in promoter and enhancer regions |
| CAAT-box | Nicotiana glutinosa | 156 | + | 4 | CAAT |  |

>Potri.018G038100.1   
+ GTTGCTGTCT TTTTTTTTTA TCATGTTATC AAATTAATGG AAATTTAACT CTATTATCAA AATTCTCTTT   
  
  
+ TTTATAAACA TGATAATCTT TTTTATTTTA TTTTCAAGTT AAAAATACTT GGTCCGGCCT GGGACGATGC   
  
  
+ GCTTGCCAAT CTAGTCAATC CTAAATTTAC TAATACGAGT AAGTAATAGC CGAAGGAGCA AAGCTCCAGT   
  
  
+ CCGGCATCTT GTTCAGAACA ATATTACATC CCTGAAATGC CTCGTGAATT GGGTGTCTCT TCCAACTTTC   
  
  
+ TCCAGCAATT TTCTCCCAAC TTCTGCAAGC AGTGATTATC AATTTGAATT ACAGGAGCTA CCTAACATAT   
  
  
+ CCGCCCTAGA ACGAAGGCAG GAATCATCAG CTTTGCCACC TCTTTGCATA ACTGCTACTG AAAGCCATTA   
  
  
+ GCTGGTGAAT GCTCCATTTC AAACTTCCAT TGGCCCGAGT ACTGCTCCTG CTTCCATTGG AAATATCGCT   
  
  
+ GCGCAATCAG ACGAGGCAGG CATTCATATC TGATGATAAA TGTTGCAGGA TTAATTCTAT TTTCGCCGGC   
  
  
+ AGCAAATGGT GGGATTTTTT ACACTGAAAA GTGTTTCAAA AGACTCGAAT TAATGTGGTC GATTTTGGCG   
  
  
+ GGGGCGTGGA ATGGATTCCT CTCTTCCTCA ATCATCAAAC ATCCAGATAC CTGAAACAGA GAGAAGCCCA   
  
  
+ TCTTCAAAAT CATATCGTCA TCGGAGGTCC TTCGCTGCAA TCGGCTGATG ACGGTGCACT TTTTGATGGT   
  
  
+ AGCGAACCAG ATCAGTCAAT GGTCAAATCA TTTGGAAATC AACGAGCTAC AGTTTATCCC TCCTGAATCC   
  
  
+ ATCTTTGAGA ACCTAAAAGA AATTAATTAA AATTAACGAA AGAGAAAAGA GAGAGGTGGT AGAGTTCATA   
  
  
+ GTTGTCGGAT CGGTCCGGAA ATTGATTTAA TTTAGAAATT AATATAGAAA AGAGTTTTGA GTAATTATAT   
  
  
+ TATCTTTAAC GGAGGCAAAC TAAATTAATA TTTTTATTAA AAAAATAATT TTTTTAAAAA AAACTTTTTA   
  
  
+ GACTTTTGTA TAAATTAATC AAATTAAATT AACAAGATTT TACTAAATTA ATAATTTATT AAATTTAACT   
  
  
+ TAAAATCTAA TTTAATATCA CAGCCTGCTT GATATTATAA TATCGGTTGT TTCTGGTTTA AAAATATATT   
  
  
+ AAAATATTAT TTTTTAATTT TTTAAAATTT AATTTTAACA TTAATAAATT AAAATGATCT AATTTTAATT   
  
  
+ TTTTTAAAAA AACTAAGATT CTTTTCAAAA ACATCTTTTT ACGGTGGCTT AGACTCTATT CGTTTTTATA   
  
  
+ TTTTAAAAAT TCTTTTAAAA AAATTAAAAA TTTTTAATTT TTTTCACTTT AATTTTTTTT ATATTTTTAA   
  
  
+ ATTATTTTAA TATGTTAATA TTAAAAATAA AATTTTAAAA TAAAAAAATA TTATTTTAAT AAATTTTAAA   
  
  
+ ATTAAAAATA TTTTAAAAAC CAACCAAAAA AGGCAACCTA TTACGAACGA ACTAGGAGAC ATGATGATCG   
  
  
+ CTGGAAGAAG AGCAACAAGA AGCAATGACA TCACTGAAAG AATAACGGTT AGTCCGGTGA ATTTTCATTA   
  
  
+ GCTGGAAGTT ACTGCTAGTA GTTAATTAGT TAGAAGAGCT GTTGAGTTAG TTAGTAGTTA GTAGTTAATT   
  
  
+ AGTTAGAAAA GCTCTTGAGT TGGTTAGTAG TTAGCAGTTG AATAAAGGGG TTAGATCAAT TGTAATTAGG   
  
  
+ AAAAAACCAT TGTATATAAA CAGGTGTATG AAAACGGGTT GAGGATAAGA AAATACAGAA GATTAATCTC   
  
  
+ ATTTCTCTGC TCATTCTTCT CTGCTCTCTC TTGTTTCTGT TCTTTCTCAA TTTCTGTTCT CTTTTCTTTC   
  
  
+ TAATCTTGTA AAGCTGAGCA TATCATCACC AAGTCATCAC GTGTAGAAGC AAAGGGACAA CAAGTCATCA   
  
  
+ CCGAAGAAGA ATATAGTTGA CCTTCGAGAA CCCAGTCAAT   

- CAACGACAGA AAAAAAAAAT AGTACAATAG TTTAATTACC TTTAAATTGA GATAATAGTT TTAAGAGAAA   
  
  
- AAATATTTGT ACTATTAGAA AAAATAAAAT AAAAGTTCAA TTTTTATGAA CCAGGCCGGA CCCTGCTACG   
  
  
- CGAACGGTTA GATCAGTTAG GATTTAAATG ATTATGCTCA TTCATTATCG GCTTCCTCGT TTCGAGGTCA   
  
  
- GGCCGTAGAA CAAGTCTTGT TATAATGTAG GGACTTTACG GAGCACTTAA CCCACAGAGA AGGTTGAAAG   
  
  
- AGGTCGTTAA AAGAGGGTTG AAGACGTTCG TCACTAATAG TTAAACTTAA TGTCCTCGAT GGATTGTATA   
  
  
- GGCGGGATCT TGCTTCCGTC CTTAGTAGTC GAAACGGTGG AGAAACGTAT TGACGATGAC TTTCGGTAAT   
  
  
- CGACCACTTA CGAGGTAAAG TTTGAAGGTA ACCGGGCTCA TGACGAGGAC GAAGGTAACC TTTATAGCGA   
  
  
- CGCGTTAGTC TGCTCCGTCC GTAAGTATAG ACTACTATTT ACAACGTCCT AATTAAGATA AAAGCGGCCG   
  
  
- TCGTTTACCA CCCTAAAAAA TGTGACTTTT CACAAAGTTT TCTGAGCTTA ATTACACCAG CTAAAACCGC   
  
  
- CCCCGCACCT TACCTAAGGA GAGAAGGAGT TAGTAGTTTG TAGGTCTATG GACTTTGTCT CTCTTCGGGT   
  
  
- AGAAGTTTTA GTATAGCAGT AGCCTCCAGG AAGCGACGTT AGCCGACTAC TGCCACGTGA AAAACTACCA   
  
  
- TCGCTTGGTC TAGTCAGTTA CCAGTTTAGT AAACCTTTAG TTGCTCGATG TCAAATAGGG AGGACTTAGG   
  
  
- TAGAAACTCT TGGATTTTCT TTAATTAATT TTAATTGCTT TCTCTTTTCT CTCTCCACCA TCTCAAGTAT   
  
  
- CAACAGCCTA GCCAGGCCTT TAACTAAATT AAATCTTTAA TTATATCTTT TCTCAAAACT CATTAATATA   
  
  
- ATAGAAATTG CCTCCGTTTG ATTTAATTAT AAAAATAATT TTTTTATTAA AAAAATTTTT TTTGAAAAAT   
  
  
- CTGAAAACAT ATTTAATTAG TTTAATTTAA TTGTTCTAAA ATGATTTAAT TATTAAATAA TTTAAATTGA   
  
  
- ATTTTAGATT AAATTATAGT GTCGGACGAA CTATAATATT ATAGCCAACA AAGACCAAAT TTTTATATAA   
  
  
- TTTTATAATA AAAAATTAAA AAATTTTAAA TTAAAATTGT AATTATTTAA TTTTACTAGA TTAAAATTAA   
  
  
- AAAAATTTTT TTGATTCTAA GAAAAGTTTT TGTAGAAAAA TGCCACCGAA TCTGAGATAA GCAAAAATAT   
  
  
- AAAATTTTTA AGAAAATTTT TTTAATTTTT AAAAATTAAA AAAAGTGAAA TTAAAAAAAA TATAAAAATT   
  
  
- TAATAAAATT ATACAATTAT AATTTTTATT TTAAAATTTT ATTTTTTTAT AATAAAATTA TTTAAAATTT   
  
  
- TAATTTTTAT AAAATTTTTG GTTGGTTTTT TCCGTTGGAT AATGCTTGCT TGATCCTCTG TACTACTAGC   
  
  
- GACCTTCTTC TCGTTGTTCT TCGTTACTGT AGTGACTTTC TTATTGCCAA TCAGGCCACT TAAAAGTAAT   
  
  
- CGACCTTCAA TGACGATCAT CAATTAATCA ATCTTCTCGA CAACTCAATC AATCATCAAT CATCAATTAA   
  
  
- TCAATCTTTT CGAGAACTCA ACCAATCATC AATCGTCAAC TTATTTCCCC AATCTAGTTA ACATTAATCC   
  
  
- TTTTTTGGTA ACATATATTT GTCCACATAC TTTTGCCCAA CTCCTATTCT TTTATGTCTT CTAATTAGAG   
  
  
- TAAAGAGACG AGTAAGAAGA GACGAGAGAG AACAAAGACA AGAAAGAGTT AAAGACAAGA GAAAAGAAAG   
  
  
- ATTAGAACAT TTCGACTCGT ATAGTAGTGG TTCAGTAGTG CACATCTTCG TTTCCCTGTT GTTCAGTAGT   
  
  
- GGCTTCTTCT TATATCAACT GGAAGCTCTT GGGTCAGTT

+     CGTCA-motif

| Site Name | Organism | Position | Strand | Matrix score. | sequence | function |
| --- | --- | --- | --- | --- | --- | --- |
| CGTCA-motif | Hordeum vulgare | 716 | + | 5 | CGTCA | cis-acting regulatory element involved in the MeJA-responsiveness |
| CGTCA-motif | Hordeum vulgare | 749 | - | 5 | CGTCA | cis-acting regulatory element involved in the MeJA-responsiveness |

>Potri.018G038100.1   
+ GTTGCTGTCT TTTTTTTTTA TCATGTTATC AAATTAATGG AAATTTAACT CTATTATCAA AATTCTCTTT   
  
  
+ TTTATAAACA TGATAATCTT TTTTATTTTA TTTTCAAGTT AAAAATACTT GGTCCGGCCT GGGACGATGC   
  
  
+ GCTTGCCAAT CTAGTCAATC CTAAATTTAC TAATACGAGT AAGTAATAGC CGAAGGAGCA AAGCTCCAGT   
  
  
+ CCGGCATCTT GTTCAGAACA ATATTACATC CCTGAAATGC CTCGTGAATT GGGTGTCTCT TCCAACTTTC   
  
  
+ TCCAGCAATT TTCTCCCAAC TTCTGCAAGC AGTGATTATC AATTTGAATT ACAGGAGCTA CCTAACATAT   
  
  
+ CCGCCCTAGA ACGAAGGCAG GAATCATCAG CTTTGCCACC TCTTTGCATA ACTGCTACTG AAAGCCATTA   
  
  
+ GCTGGTGAAT GCTCCATTTC AAACTTCCAT TGGCCCGAGT ACTGCTCCTG CTTCCATTGG AAATATCGCT   
  
  
+ GCGCAATCAG ACGAGGCAGG CATTCATATC TGATGATAAA TGTTGCAGGA TTAATTCTAT TTTCGCCGGC   
  
  
+ AGCAAATGGT GGGATTTTTT ACACTGAAAA GTGTTTCAAA AGACTCGAAT TAATGTGGTC GATTTTGGCG   
  
  
+ GGGGCGTGGA ATGGATTCCT CTCTTCCTCA ATCATCAAAC ATCCAGATAC CTGAAACAGA GAGAAGCCCA   
  
  
+ TCTTCAAAAT CATATCGTCA TCGGAGGTCC TTCGCTGCAA TCGGCTGATG ACGGTGCACT TTTTGATGGT   
  
  
+ AGCGAACCAG ATCAGTCAAT GGTCAAATCA TTTGGAAATC AACGAGCTAC AGTTTATCCC TCCTGAATCC   
  
  
+ ATCTTTGAGA ACCTAAAAGA AATTAATTAA AATTAACGAA AGAGAAAAGA GAGAGGTGGT AGAGTTCATA   
  
  
+ GTTGTCGGAT CGGTCCGGAA ATTGATTTAA TTTAGAAATT AATATAGAAA AGAGTTTTGA GTAATTATAT   
  
  
+ TATCTTTAAC GGAGGCAAAC TAAATTAATA TTTTTATTAA AAAAATAATT TTTTTAAAAA AAACTTTTTA   
  
  
+ GACTTTTGTA TAAATTAATC AAATTAAATT AACAAGATTT TACTAAATTA ATAATTTATT AAATTTAACT   
  
  
+ TAAAATCTAA TTTAATATCA CAGCCTGCTT GATATTATAA TATCGGTTGT TTCTGGTTTA AAAATATATT   
  
  
+ AAAATATTAT TTTTTAATTT TTTAAAATTT AATTTTAACA TTAATAAATT AAAATGATCT AATTTTAATT   
  
  
+ TTTTTAAAAA AACTAAGATT CTTTTCAAAA ACATCTTTTT ACGGTGGCTT AGACTCTATT CGTTTTTATA   
  
  
+ TTTTAAAAAT TCTTTTAAAA AAATTAAAAA TTTTTAATTT TTTTCACTTT AATTTTTTTT ATATTTTTAA   
  
  
+ ATTATTTTAA TATGTTAATA TTAAAAATAA AATTTTAAAA TAAAAAAATA TTATTTTAAT AAATTTTAAA   
  
  
+ ATTAAAAATA TTTTAAAAAC CAACCAAAAA AGGCAACCTA TTACGAACGA ACTAGGAGAC ATGATGATCG   
  
  
+ CTGGAAGAAG AGCAACAAGA AGCAATGACA TCACTGAAAG AATAACGGTT AGTCCGGTGA ATTTTCATTA   
  
  
+ GCTGGAAGTT ACTGCTAGTA GTTAATTAGT TAGAAGAGCT GTTGAGTTAG TTAGTAGTTA GTAGTTAATT   
  
  
+ AGTTAGAAAA GCTCTTGAGT TGGTTAGTAG TTAGCAGTTG AATAAAGGGG TTAGATCAAT TGTAATTAGG   
  
  
+ AAAAAACCAT TGTATATAAA CAGGTGTATG AAAACGGGTT GAGGATAAGA AAATACAGAA GATTAATCTC   
  
  
+ ATTTCTCTGC TCATTCTTCT CTGCTCTCTC TTGTTTCTGT TCTTTCTCAA TTTCTGTTCT CTTTTCTTTC   
  
  
+ TAATCTTGTA AAGCTGAGCA TATCATCACC AAGTCATCAC GTGTAGAAGC AAAGGGACAA CAAGTCATCA   
  
  
+ CCGAAGAAGA ATATAGTTGA CCTTCGAGAA CCCAGTCAA  

- CAACGACAGA AAAAAAAAAT AGTACAATAG TTTAATTACC TTTAAATTGA GATAATAGTT TTAAGAGAAA   
  
  
- AAATATTTGT ACTATTAGAA AAAATAAAAT AAAAGTTCAA TTTTTATGAA CCAGGCCGGA CCCTGCTACG   
  
  
- CGAACGGTTA GATCAGTTAG GATTTAAATG ATTATGCTCA TTCATTATCG GCTTCCTCGT TTCGAGGTCA   
  
  
- GGCCGTAGAA CAAGTCTTGT TATAATGTAG GGACTTTACG GAGCACTTAA CCCACAGAGA AGGTTGAAAG   
  
  
- AGGTCGTTAA AAGAGGGTTG AAGACGTTCG TCACTAATAG TTAAACTTAA TGTCCTCGAT GGATTGTATA   
  
  
- GGCGGGATCT TGCTTCCGTC CTTAGTAGTC GAAACGGTGG AGAAACGTAT TGACGATGAC TTTCGGTAAT   
  
  
- CGACCACTTA CGAGGTAAAG TTTGAAGGTA ACCGGGCTCA TGACGAGGAC GAAGGTAACC TTTATAGCGA   
  
  
- CGCGTTAGTC TGCTCCGTCC GTAAGTATAG ACTACTATTT ACAACGTCCT AATTAAGATA AAAGCGGCCG   
  
  
- TCGTTTACCA CCCTAAAAAA TGTGACTTTT CACAAAGTTT TCTGAGCTTA ATTACACCAG CTAAAACCGC   
  
  
- CCCCGCACCT TACCTAAGGA GAGAAGGAGT TAGTAGTTTG TAGGTCTATG GACTTTGTCT CTCTTCGGGT   
  
  
- AGAAGTTTTA GTATAGCAGT AGCCTCCAGG AAGCGACGTT AGCCGACTAC TGCCACGTGA AAAACTACCA   
  
  
- TCGCTTGGTC TAGTCAGTTA CCAGTTTAGT AAACCTTTAG TTGCTCGATG TCAAATAGGG AGGACTTAGG   
  
  
- TAGAAACTCT TGGATTTTCT TTAATTAATT TTAATTGCTT TCTCTTTTCT CTCTCCACCA TCTCAAGTAT   
  
  
- CAACAGCCTA GCCAGGCCTT TAACTAAATT AAATCTTTAA TTATATCTTT TCTCAAAACT CATTAATATA   
  
  
- ATAGAAATTG CCTCCGTTTG ATTTAATTAT AAAAATAATT TTTTTATTAA AAAAATTTTT TTTGAAAAAT   
  
  
- CTGAAAACAT ATTTAATTAG TTTAATTTAA TTGTTCTAAA ATGATTTAAT TATTAAATAA TTTAAATTGA   
  
  
- ATTTTAGATT AAATTATAGT GTCGGACGAA CTATAATATT ATAGCCAACA AAGACCAAAT TTTTATATAA   
  
  
- TTTTATAATA AAAAATTAAA AAATTTTAAA TTAAAATTGT AATTATTTAA TTTTACTAGA TTAAAATTAA   
  
  
- AAAAATTTTT TTGATTCTAA GAAAAGTTTT TGTAGAAAAA TGCCACCGAA TCTGAGATAA GCAAAAATAT   
  
  
- AAAATTTTTA AGAAAATTTT TTTAATTTTT AAAAATTAAA AAAAGTGAAA TTAAAAAAAA TATAAAAATT   
  
  
- TAATAAAATT ATACAATTAT AATTTTTATT TTAAAATTTT ATTTTTTTAT AATAAAATTA TTTAAAATTT   
  
  
- TAATTTTTAT AAAATTTTTG GTTGGTTTTT TCCGTTGGAT AATGCTTGCT TGATCCTCTG TACTACTAGC   
  
  
- GACCTTCTTC TCGTTGTTCT TCGTTACTGT AGTGACTTTC TTATTGCCAA TCAGGCCACT TAAAAGTAAT   
  
  
- CGACCTTCAA TGACGATCAT CAATTAATCA ATCTTCTCGA CAACTCAATC AATCATCAAT CATCAATTAA   
  
  
- TCAATCTTTT CGAGAACTCA ACCAATCATC AATCGTCAAC TTATTTCCCC AATCTAGTTA ACATTAATCC   
  
  
- TTTTTTGGTA ACATATATTT GTCCACATAC TTTTGCCCAA CTCCTATTCT TTTATGTCTT CTAATTAGAG   
  
  
- TAAAGAGACG AGTAAGAAGA GACGAGAGAG AACAAAGACA AGAAAGAGTT AAAGACAAGA GAAAAGAAAG   
  
  
- ATTAGAACAT TTCGACTCGT ATAGTAGTGG TTCAGTAGTG CACATCTTCG TTTCCCTGTT GTTCAGTAGT   
  
  
- GGCTTCTTCT TATATCAACT GGAAGCTCTT GGGTCAGTT

+     CTAG-motif

| Site Name | Organism | Position | Strand | Matrix score. | sequence | function |
| --- | --- | --- | --- | --- | --- | --- |
| CTAG-motif | Avena sativa | 1620 | - | 9 | ACTAGCAGAA |  |

>Potri.018G038100.1   
+ GTTGCTGTCT TTTTTTTTTA TCATGTTATC AAATTAATGG AAATTTAACT CTATTATCAA AATTCTCTTT   
  
  
+ TTTATAAACA TGATAATCTT TTTTATTTTA TTTTCAAGTT AAAAATACTT GGTCCGGCCT GGGACGATGC   
  
  
+ GCTTGCCAAT CTAGTCAATC CTAAATTTAC TAATACGAGT AAGTAATAGC CGAAGGAGCA AAGCTCCAGT   
  
  
+ CCGGCATCTT GTTCAGAACA ATATTACATC CCTGAAATGC CTCGTGAATT GGGTGTCTCT TCCAACTTTC   
  
  
+ TCCAGCAATT TTCTCCCAAC TTCTGCAAGC AGTGATTATC AATTTGAATT ACAGGAGCTA CCTAACATAT   
  
  
+ CCGCCCTAGA ACGAAGGCAG GAATCATCAG CTTTGCCACC TCTTTGCATA ACTGCTACTG AAAGCCATTA   
  
  
+ GCTGGTGAAT GCTCCATTTC AAACTTCCAT TGGCCCGAGT ACTGCTCCTG CTTCCATTGG AAATATCGCT   
  
  
+ GCGCAATCAG ACGAGGCAGG CATTCATATC TGATGATAAA TGTTGCAGGA TTAATTCTAT TTTCGCCGGC   
  
  
+ AGCAAATGGT GGGATTTTTT ACACTGAAAA GTGTTTCAAA AGACTCGAAT TAATGTGGTC GATTTTGGCG   
  
  
+ GGGGCGTGGA ATGGATTCCT CTCTTCCTCA ATCATCAAAC ATCCAGATAC CTGAAACAGA GAGAAGCCCA   
  
  
+ TCTTCAAAAT CATATCGTCA TCGGAGGTCC TTCGCTGCAA TCGGCTGATG ACGGTGCACT TTTTGATGGT   
  
  
+ AGCGAACCAG ATCAGTCAAT GGTCAAATCA TTTGGAAATC AACGAGCTAC AGTTTATCCC TCCTGAATCC   
  
  
+ ATCTTTGAGA ACCTAAAAGA AATTAATTAA AATTAACGAA AGAGAAAAGA GAGAGGTGGT AGAGTTCATA   
  
  
+ GTTGTCGGAT CGGTCCGGAA ATTGATTTAA TTTAGAAATT AATATAGAAA AGAGTTTTGA GTAATTATAT   
  
  
+ TATCTTTAAC GGAGGCAAAC TAAATTAATA TTTTTATTAA AAAAATAATT TTTTTAAAAA AAACTTTTTA   
  
  
+ GACTTTTGTA TAAATTAATC AAATTAAATT AACAAGATTT TACTAAATTA ATAATTTATT AAATTTAACT   
  
  
+ TAAAATCTAA TTTAATATCA CAGCCTGCTT GATATTATAA TATCGGTTGT TTCTGGTTTA AAAATATATT   
  
  
+ AAAATATTAT TTTTTAATTT TTTAAAATTT AATTTTAACA TTAATAAATT AAAATGATCT AATTTTAATT   
  
  
+ TTTTTAAAAA AACTAAGATT CTTTTCAAAA ACATCTTTTT ACGGTGGCTT AGACTCTATT CGTTTTTATA   
  
  
+ TTTTAAAAAT TCTTTTAAAA AAATTAAAAA TTTTTAATTT TTTTCACTTT AATTTTTTTT ATATTTTTAA   
  
  
+ ATTATTTTAA TATGTTAATA TTAAAAATAA AATTTTAAAA TAAAAAAATA TTATTTTAAT AAATTTTAAA   
  
  
+ ATTAAAAATA TTTTAAAAAC CAACCAAAAA AGGCAACCTA TTACGAACGA ACTAGGAGAC ATGATGATCG   
  
  
+ CTGGAAGAAG AGCAACAAGA AGCAATGACA TCACTGAAAG AATAACGGTT AGTCCGGTGA ATTTTCATTA   
  
  
+ GCTGGAAGTT ACTGCTAGTA GTTAATTAGT TAGAAGAGCT GTTGAGTTAG TTAGTAGTTA GTAGTTAATT   
  
  
+ AGTTAGAAAA GCTCTTGAGT TGGTTAGTAG TTAGCAGTTG AATAAAGGGG TTAGATCAAT TGTAATTAGG   
  
  
+ AAAAAACCAT TGTATATAAA CAGGTGTATG AAAACGGGTT GAGGATAAGA AAATACAGAA GATTAATCTC   
  
  
+ ATTTCTCTGC TCATTCTTCT CTGCTCTCTC TTGTTTCTGT TCTTTCTCAA TTTCTGTTCT CTTTTCTTTC   
  
  
+ TAATCTTGTA AAGCTGAGCA TATCATCACC AAGTCATCAC GTGTAGAAGC AAAGGGACAA CAAGTCATCA   
  
  
+ CCGAAGAAGA ATATAGTTGA CCTTCGAGAA CCCAGTCAA  

- CAACGACAGA AAAAAAAAAT AGTACAATAG TTTAATTACC TTTAAATTGA GATAATAGTT TTAAGAGAAA   
  
  
- AAATATTTGT ACTATTAGAA AAAATAAAAT AAAAGTTCAA TTTTTATGAA CCAGGCCGGA CCCTGCTACG   
  
  
- CGAACGGTTA GATCAGTTAG GATTTAAATG ATTATGCTCA TTCATTATCG GCTTCCTCGT TTCGAGGTCA   
  
  
- GGCCGTAGAA CAAGTCTTGT TATAATGTAG GGACTTTACG GAGCACTTAA CCCACAGAGA AGGTTGAAAG   
  
  
- AGGTCGTTAA AAGAGGGTTG AAGACGTTCG TCACTAATAG TTAAACTTAA TGTCCTCGAT GGATTGTATA   
  
  
- GGCGGGATCT TGCTTCCGTC CTTAGTAGTC GAAACGGTGG AGAAACGTAT TGACGATGAC TTTCGGTAAT   
  
  
- CGACCACTTA CGAGGTAAAG TTTGAAGGTA ACCGGGCTCA TGACGAGGAC GAAGGTAACC TTTATAGCGA   
  
  
- CGCGTTAGTC TGCTCCGTCC GTAAGTATAG ACTACTATTT ACAACGTCCT AATTAAGATA AAAGCGGCCG   
  
  
- TCGTTTACCA CCCTAAAAAA TGTGACTTTT CACAAAGTTT TCTGAGCTTA ATTACACCAG CTAAAACCGC   
  
  
- CCCCGCACCT TACCTAAGGA GAGAAGGAGT TAGTAGTTTG TAGGTCTATG GACTTTGTCT CTCTTCGGGT   
  
  
- AGAAGTTTTA GTATAGCAGT AGCCTCCAGG AAGCGACGTT AGCCGACTAC TGCCACGTGA AAAACTACCA   
  
  
- TCGCTTGGTC TAGTCAGTTA CCAGTTTAGT AAACCTTTAG TTGCTCGATG TCAAATAGGG AGGACTTAGG   
  
  
- TAGAAACTCT TGGATTTTCT TTAATTAATT TTAATTGCTT TCTCTTTTCT CTCTCCACCA TCTCAAGTAT   
  
  
- CAACAGCCTA GCCAGGCCTT TAACTAAATT AAATCTTTAA TTATATCTTT TCTCAAAACT CATTAATATA   
  
  
- ATAGAAATTG CCTCCGTTTG ATTTAATTAT AAAAATAATT TTTTTATTAA AAAAATTTTT TTTGAAAAAT   
  
  
- CTGAAAACAT ATTTAATTAG TTTAATTTAA TTGTTCTAAA ATGATTTAAT TATTAAATAA TTTAAATTGA   
  
  
- ATTTTAGATT AAATTATAGT GTCGGACGAA CTATAATATT ATAGCCAACA AAGACCAAAT TTTTATATAA   
  
  
- TTTTATAATA AAAAATTAAA AAATTTTAAA TTAAAATTGT AATTATTTAA TTTTACTAGA TTAAAATTAA   
  
  
- AAAAATTTTT TTGATTCTAA GAAAAGTTTT TGTAGAAAAA TGCCACCGAA TCTGAGATAA GCAAAAATAT   
  
  
- AAAATTTTTA AGAAAATTTT TTTAATTTTT AAAAATTAAA AAAAGTGAAA TTAAAAAAAA TATAAAAATT   
  
  
- TAATAAAATT ATACAATTAT AATTTTTATT TTAAAATTTT ATTTTTTTAT AATAAAATTA TTTAAAATTT   
  
  
- TAATTTTTAT AAAATTTTTG GTTGGTTTTT TCCGTTGGAT AATGCTTGCT TGATCCTCTG TACTACTAGC   
  
  
- GACCTTCTTC TCGTTGTTCT TCGTTACTGT AGTGACTTTC TTATTGCCAA TCAGGCCACT TAAAAGTAAT   
  
  
- CGACCTTCAA TGACGATCAT CAATTAATCA ATCTTCTCGA CAACTCAATC AATCATCAAT CATCAATTAA   
  
  
- TCAATCTTTT CGAGAACTCA ACCAATCATC AATCGTCAAC TTATTTCCCC AATCTAGTTA ACATTAATCC   
  
  
- TTTTTTGGTA ACATATATTT GTCCACATAC TTTTGCCCAA CTCCTATTCT TTTATGTCTT CTAATTAGAG   
  
  
- TAAAGAGACG AGTAAGAAGA GACGAGAGAG AACAAAGACA AGAAAGAGTT AAAGACAAGA GAAAAGAAAG   
  
  
- ATTAGAACAT TTCGACTCGT ATAGTAGTGG TTCAGTAGTG CACATCTTCG TTTCCCTGTT GTTCAGTAGT   
  
  
- GGCTTCTTCT TATATCAACT GGAAGCTCTT GGGTCAGTT

+     ERE

| Site Name | Organism | Position | Strand | Matrix score. | sequence | function |
| --- | --- | --- | --- | --- | --- | --- |
| ERE | Nicotiana glutinos | 1211 | - | 8 | ATTTTAAA |  |
| ERE | Nicotiana glutinos | 1465 | - | 8 | ATTTTAAA |  |
| ERE | Nicotiana glutinos | 1463 | + | 8 | ATTTTAAA |  |
| ERE | Nicotiana glutinos | 1432 | + | 8 | ATTTTAAA |  |
| ERE | Nicotiana glutinos | 1330 | + | 8 | ATTTTAAA |  |
| ERE | Nicotiana glutinos | 1434 | - | 8 | ATTTTAAA |  |
| ERE | Nicotiana glutinos | 1480 | + | 8 | ATTTTAAA |  |

>Potri.018G038100.1   
+ GTTGCTGTCT TTTTTTTTTA TCATGTTATC AAATTAATGG AAATTTAACT CTATTATCAA AATTCTCTTT   
  
  
+ TTTATAAACA TGATAATCTT TTTTATTTTA TTTTCAAGTT AAAAATACTT GGTCCGGCCT GGGACGATGC   
  
  
+ GCTTGCCAAT CTAGTCAATC CTAAATTTAC TAATACGAGT AAGTAATAGC CGAAGGAGCA AAGCTCCAGT   
  
  
+ CCGGCATCTT GTTCAGAACA ATATTACATC CCTGAAATGC CTCGTGAATT GGGTGTCTCT TCCAACTTTC   
  
  
+ TCCAGCAATT TTCTCCCAAC TTCTGCAAGC AGTGATTATC AATTTGAATT ACAGGAGCTA CCTAACATAT   
  
  
+ CCGCCCTAGA ACGAAGGCAG GAATCATCAG CTTTGCCACC TCTTTGCATA ACTGCTACTG AAAGCCATTA   
  
  
+ GCTGGTGAAT GCTCCATTTC AAACTTCCAT TGGCCCGAGT ACTGCTCCTG CTTCCATTGG AAATATCGCT   
  
  
+ GCGCAATCAG ACGAGGCAGG CATTCATATC TGATGATAAA TGTTGCAGGA TTAATTCTAT TTTCGCCGGC   
  
  
+ AGCAAATGGT GGGATTTTTT ACACTGAAAA GTGTTTCAAA AGACTCGAAT TAATGTGGTC GATTTTGGCG   
  
  
+ GGGGCGTGGA ATGGATTCCT CTCTTCCTCA ATCATCAAAC ATCCAGATAC CTGAAACAGA GAGAAGCCCA   
  
  
+ TCTTCAAAAT CATATCGTCA TCGGAGGTCC TTCGCTGCAA TCGGCTGATG ACGGTGCACT TTTTGATGGT   
  
  
+ AGCGAACCAG ATCAGTCAAT GGTCAAATCA TTTGGAAATC AACGAGCTAC AGTTTATCCC TCCTGAATCC   
  
  
+ ATCTTTGAGA ACCTAAAAGA AATTAATTAA AATTAACGAA AGAGAAAAGA GAGAGGTGGT AGAGTTCATA   
  
  
+ GTTGTCGGAT CGGTCCGGAA ATTGATTTAA TTTAGAAATT AATATAGAAA AGAGTTTTGA GTAATTATAT   
  
  
+ TATCTTTAAC GGAGGCAAAC TAAATTAATA TTTTTATTAA AAAAATAATT TTTTTAAAAA AAACTTTTTA   
  
  
+ GACTTTTGTA TAAATTAATC AAATTAAATT AACAAGATTT TACTAAATTA ATAATTTATT AAATTTAACT   
  
  
+ TAAAATCTAA TTTAATATCA CAGCCTGCTT GATATTATAA TATCGGTTGT TTCTGGTTTA AAAATATATT   
  
  
+ AAAATATTAT TTTTTAATTT TTTAAAATTT AATTTTAACA TTAATAAATT AAAATGATCT AATTTTAATT   
  
  
+ TTTTTAAAAA AACTAAGATT CTTTTCAAAA ACATCTTTTT ACGGTGGCTT AGACTCTATT CGTTTTTATA   
  
  
+ TTTTAAAAAT TCTTTTAAAA AAATTAAAAA TTTTTAATTT TTTTCACTTT AATTTTTTTT ATATTTTTAA   
  
  
+ ATTATTTTAA TATGTTAATA TTAAAAATAA AATTTTAAAA TAAAAAAATA TTATTTTAAT AAATTTTAAA   
  
  
+ ATTAAAAATA TTTTAAAAAC CAACCAAAAA AGGCAACCTA TTACGAACGA ACTAGGAGAC ATGATGATCG   
  
  
+ CTGGAAGAAG AGCAACAAGA AGCAATGACA TCACTGAAAG AATAACGGTT AGTCCGGTGA ATTTTCATTA   
  
  
+ GCTGGAAGTT ACTGCTAGTA GTTAATTAGT TAGAAGAGCT GTTGAGTTAG TTAGTAGTTA GTAGTTAATT   
  
  
+ AGTTAGAAAA GCTCTTGAGT TGGTTAGTAG TTAGCAGTTG AATAAAGGGG TTAGATCAAT TGTAATTAGG   
  
  
+ AAAAAACCAT TGTATATAAA CAGGTGTATG AAAACGGGTT GAGGATAAGA AAATACAGAA GATTAATCTC   
  
  
+ ATTTCTCTGC TCATTCTTCT CTGCTCTCTC TTGTTTCTGT TCTTTCTCAA TTTCTGTTCT CTTTTCTTTC   
  
  
+ TAATCTTGTA AAGCTGAGCA TATCATCACC AAGTCATCAC GTGTAGAAGC AAAGGGACAA CAAGTCATCA   
  
  
+ CCGAAGAAGA ATATAGTTGA CCTTCGAGAA CCCAGTCAA  

- CAACGACAGA AAAAAAAAAT AGTACAATAG TTTAATTACC TTTAAATTGA GATAATAGTT TTAAGAGAAA   
  
  
- AAATATTTGT ACTATTAGAA AAAATAAAAT AAAAGTTCAA TTTTTATGAA CCAGGCCGGA CCCTGCTACG   
  
  
- CGAACGGTTA GATCAGTTAG GATTTAAATG ATTATGCTCA TTCATTATCG GCTTCCTCGT TTCGAGGTCA   
  
  
- GGCCGTAGAA CAAGTCTTGT TATAATGTAG GGACTTTACG GAGCACTTAA CCCACAGAGA AGGTTGAAAG   
  
  
- AGGTCGTTAA AAGAGGGTTG AAGACGTTCG TCACTAATAG TTAAACTTAA TGTCCTCGAT GGATTGTATA   
  
  
- GGCGGGATCT TGCTTCCGTC CTTAGTAGTC GAAACGGTGG AGAAACGTAT TGACGATGAC TTTCGGTAAT   
  
  
- CGACCACTTA CGAGGTAAAG TTTGAAGGTA ACCGGGCTCA TGACGAGGAC GAAGGTAACC TTTATAGCGA   
  
  
- CGCGTTAGTC TGCTCCGTCC GTAAGTATAG ACTACTATTT ACAACGTCCT AATTAAGATA AAAGCGGCCG   
  
  
- TCGTTTACCA CCCTAAAAAA TGTGACTTTT CACAAAGTTT TCTGAGCTTA ATTACACCAG CTAAAACCGC   
  
  
- CCCCGCACCT TACCTAAGGA GAGAAGGAGT TAGTAGTTTG TAGGTCTATG GACTTTGTCT CTCTTCGGGT   
  
  
- AGAAGTTTTA GTATAGCAGT AGCCTCCAGG AAGCGACGTT AGCCGACTAC TGCCACGTGA AAAACTACCA   
  
  
- TCGCTTGGTC TAGTCAGTTA CCAGTTTAGT AAACCTTTAG TTGCTCGATG TCAAATAGGG AGGACTTAGG   
  
  
- TAGAAACTCT TGGATTTTCT TTAATTAATT TTAATTGCTT TCTCTTTTCT CTCTCCACCA TCTCAAGTAT   
  
  
- CAACAGCCTA GCCAGGCCTT TAACTAAATT AAATCTTTAA TTATATCTTT TCTCAAAACT CATTAATATA   
  
  
- ATAGAAATTG CCTCCGTTTG ATTTAATTAT AAAAATAATT TTTTTATTAA AAAAATTTTT TTTGAAAAAT   
  
  
- CTGAAAACAT ATTTAATTAG TTTAATTTAA TTGTTCTAAA ATGATTTAAT TATTAAATAA TTTAAATTGA   
  
  
- ATTTTAGATT AAATTATAGT GTCGGACGAA CTATAATATT ATAGCCAACA AAGACCAAAT TTTTATATAA   
  
  
- TTTTATAATA AAAAATTAAA AAATTTTAAA TTAAAATTGT AATTATTTAA TTTTACTAGA TTAAAATTAA   
  
  
- AAAAATTTTT TTGATTCTAA GAAAAGTTTT TGTAGAAAAA TGCCACCGAA TCTGAGATAA GCAAAAATAT   
  
  
- AAAATTTTTA AGAAAATTTT TTTAATTTTT AAAAATTAAA AAAAGTGAAA TTAAAAAAAA TATAAAAATT   
  
  
- TAATAAAATT ATACAATTAT AATTTTTATT TTAAAATTTT ATTTTTTTAT AATAAAATTA TTTAAAATTT   
  
  
- TAATTTTTAT AAAATTTTTG GTTGGTTTTT TCCGTTGGAT AATGCTTGCT TGATCCTCTG TACTACTAGC   
  
  
- GACCTTCTTC TCGTTGTTCT TCGTTACTGT AGTGACTTTC TTATTGCCAA TCAGGCCACT TAAAAGTAAT   
  
  
- CGACCTTCAA TGACGATCAT CAATTAATCA ATCTTCTCGA CAACTCAATC AATCATCAAT CATCAATTAA   
  
  
- TCAATCTTTT CGAGAACTCA ACCAATCATC AATCGTCAAC TTATTTCCCC AATCTAGTTA ACATTAATCC   
  
  
- TTTTTTGGTA ACATATATTT GTCCACATAC TTTTGCCCAA CTCCTATTCT TTTATGTCTT CTAATTAGAG   
  
  
- TAAAGAGACG AGTAAGAAGA GACGAGAGAG AACAAAGACA AGAAAGAGTT AAAGACAAGA GAAAAGAAAG   
  
  
- ATTAGAACAT TTCGACTCGT ATAGTAGTGG TTCAGTAGTG CACATCTTCG TTTCCCTGTT GTTCAGTAGT   
  
  
- GGCTTCTTCT TATATCAACT GGAAGCTCTT GGGTCAGTT

+     G-Box

| Site Name | Organism | Position | Strand | Matrix score. | sequence | function |
| --- | --- | --- | --- | --- | --- | --- |
| G-Box | Pisum sativum | 1928 | - | 6 | CACGTG | cis-acting regulatory element involved in light responsiveness |

>Potri.018G038100.1   
+ GTTGCTGTCT TTTTTTTTTA TCATGTTATC AAATTAATGG AAATTTAACT CTATTATCAA AATTCTCTTT   
  
  
+ TTTATAAACA TGATAATCTT TTTTATTTTA TTTTCAAGTT AAAAATACTT GGTCCGGCCT GGGACGATGC   
  
  
+ GCTTGCCAAT CTAGTCAATC CTAAATTTAC TAATACGAGT AAGTAATAGC CGAAGGAGCA AAGCTCCAGT   
  
  
+ CCGGCATCTT GTTCAGAACA ATATTACATC CCTGAAATGC CTCGTGAATT GGGTGTCTCT TCCAACTTTC   
  
  
+ TCCAGCAATT TTCTCCCAAC TTCTGCAAGC AGTGATTATC AATTTGAATT ACAGGAGCTA CCTAACATAT   
  
  
+ CCGCCCTAGA ACGAAGGCAG GAATCATCAG CTTTGCCACC TCTTTGCATA ACTGCTACTG AAAGCCATTA   
  
  
+ GCTGGTGAAT GCTCCATTTC AAACTTCCAT TGGCCCGAGT ACTGCTCCTG CTTCCATTGG AAATATCGCT   
  
  
+ GCGCAATCAG ACGAGGCAGG CATTCATATC TGATGATAAA TGTTGCAGGA TTAATTCTAT TTTCGCCGGC   
  
  
+ AGCAAATGGT GGGATTTTTT ACACTGAAAA GTGTTTCAAA AGACTCGAAT TAATGTGGTC GATTTTGGCG   
  
  
+ GGGGCGTGGA ATGGATTCCT CTCTTCCTCA ATCATCAAAC ATCCAGATAC CTGAAACAGA GAGAAGCCCA   
  
  
+ TCTTCAAAAT CATATCGTCA TCGGAGGTCC TTCGCTGCAA TCGGCTGATG ACGGTGCACT TTTTGATGGT   
  
  
+ AGCGAACCAG ATCAGTCAAT GGTCAAATCA TTTGGAAATC AACGAGCTAC AGTTTATCCC TCCTGAATCC   
  
  
+ ATCTTTGAGA ACCTAAAAGA AATTAATTAA AATTAACGAA AGAGAAAAGA GAGAGGTGGT AGAGTTCATA   
  
  
+ GTTGTCGGAT CGGTCCGGAA ATTGATTTAA TTTAGAAATT AATATAGAAA AGAGTTTTGA GTAATTATAT   
  
  
+ TATCTTTAAC GGAGGCAAAC TAAATTAATA TTTTTATTAA AAAAATAATT TTTTTAAAAA AAACTTTTTA   
  
  
+ GACTTTTGTA TAAATTAATC AAATTAAATT AACAAGATTT TACTAAATTA ATAATTTATT AAATTTAACT   
  
  
+ TAAAATCTAA TTTAATATCA CAGCCTGCTT GATATTATAA TATCGGTTGT TTCTGGTTTA AAAATATATT   
  
  
+ AAAATATTAT TTTTTAATTT TTTAAAATTT AATTTTAACA TTAATAAATT AAAATGATCT AATTTTAATT   
  
  
+ TTTTTAAAAA AACTAAGATT CTTTTCAAAA ACATCTTTTT ACGGTGGCTT AGACTCTATT CGTTTTTATA   
  
  
+ TTTTAAAAAT TCTTTTAAAA AAATTAAAAA TTTTTAATTT TTTTCACTTT AATTTTTTTT ATATTTTTAA   
  
  
+ ATTATTTTAA TATGTTAATA TTAAAAATAA AATTTTAAAA TAAAAAAATA TTATTTTAAT AAATTTTAAA   
  
  
+ ATTAAAAATA TTTTAAAAAC CAACCAAAAA AGGCAACCTA TTACGAACGA ACTAGGAGAC ATGATGATCG   
  
  
+ CTGGAAGAAG AGCAACAAGA AGCAATGACA TCACTGAAAG AATAACGGTT AGTCCGGTGA ATTTTCATTA   
  
  
+ GCTGGAAGTT ACTGCTAGTA GTTAATTAGT TAGAAGAGCT GTTGAGTTAG TTAGTAGTTA GTAGTTAATT   
  
  
+ AGTTAGAAAA GCTCTTGAGT TGGTTAGTAG TTAGCAGTTG AATAAAGGGG TTAGATCAAT TGTAATTAGG   
  
  
+ AAAAAACCAT TGTATATAAA CAGGTGTATG AAAACGGGTT GAGGATAAGA AAATACAGAA GATTAATCTC   
  
  
+ ATTTCTCTGC TCATTCTTCT CTGCTCTCTC TTGTTTCTGT TCTTTCTCAA TTTCTGTTCT CTTTTCTTTC   
  
  
+ TAATCTTGTA AAGCTGAGCA TATCATCACC AAGTCATCAC GTGTAGAAGC AAAGGGACAA CAAGTCATCA   
  
  
+ CCGAAGAAGA ATATAGTTGA CCTTCGAGAA CCCAGTCAA  

- CAACGACAGA AAAAAAAAAT AGTACAATAG TTTAATTACC TTTAAATTGA GATAATAGTT TTAAGAGAAA   
  
  
- AAATATTTGT ACTATTAGAA AAAATAAAAT AAAAGTTCAA TTTTTATGAA CCAGGCCGGA CCCTGCTACG   
  
  
- CGAACGGTTA GATCAGTTAG GATTTAAATG ATTATGCTCA TTCATTATCG GCTTCCTCGT TTCGAGGTCA   
  
  
- GGCCGTAGAA CAAGTCTTGT TATAATGTAG GGACTTTACG GAGCACTTAA CCCACAGAGA AGGTTGAAAG   
  
  
- AGGTCGTTAA AAGAGGGTTG AAGACGTTCG TCACTAATAG TTAAACTTAA TGTCCTCGAT GGATTGTATA   
  
  
- GGCGGGATCT TGCTTCCGTC CTTAGTAGTC GAAACGGTGG AGAAACGTAT TGACGATGAC TTTCGGTAAT   
  
  
- CGACCACTTA CGAGGTAAAG TTTGAAGGTA ACCGGGCTCA TGACGAGGAC GAAGGTAACC TTTATAGCGA   
  
  
- CGCGTTAGTC TGCTCCGTCC GTAAGTATAG ACTACTATTT ACAACGTCCT AATTAAGATA AAAGCGGCCG   
  
  
- TCGTTTACCA CCCTAAAAAA TGTGACTTTT CACAAAGTTT TCTGAGCTTA ATTACACCAG CTAAAACCGC   
  
  
- CCCCGCACCT TACCTAAGGA GAGAAGGAGT TAGTAGTTTG TAGGTCTATG GACTTTGTCT CTCTTCGGGT   
  
  
- AGAAGTTTTA GTATAGCAGT AGCCTCCAGG AAGCGACGTT AGCCGACTAC TGCCACGTGA AAAACTACCA   
  
  
- TCGCTTGGTC TAGTCAGTTA CCAGTTTAGT AAACCTTTAG TTGCTCGATG TCAAATAGGG AGGACTTAGG   
  
  
- TAGAAACTCT TGGATTTTCT TTAATTAATT TTAATTGCTT TCTCTTTTCT CTCTCCACCA TCTCAAGTAT   
  
  
- CAACAGCCTA GCCAGGCCTT TAACTAAATT AAATCTTTAA TTATATCTTT TCTCAAAACT CATTAATATA   
  
  
- ATAGAAATTG CCTCCGTTTG ATTTAATTAT AAAAATAATT TTTTTATTAA AAAAATTTTT TTTGAAAAAT   
  
  
- CTGAAAACAT ATTTAATTAG TTTAATTTAA TTGTTCTAAA ATGATTTAAT TATTAAATAA TTTAAATTGA   
  
  
- ATTTTAGATT AAATTATAGT GTCGGACGAA CTATAATATT ATAGCCAACA AAGACCAAAT TTTTATATAA   
  
  
- TTTTATAATA AAAAATTAAA AAATTTTAAA TTAAAATTGT AATTATTTAA TTTTACTAGA TTAAAATTAA   
  
  
- AAAAATTTTT TTGATTCTAA GAAAAGTTTT TGTAGAAAAA TGCCACCGAA TCTGAGATAA GCAAAAATAT   
  
  
- AAAATTTTTA AGAAAATTTT TTTAATTTTT AAAAATTAAA AAAAGTGAAA TTAAAAAAAA TATAAAAATT   
  
  
- TAATAAAATT ATACAATTAT AATTTTTATT TTAAAATTTT ATTTTTTTAT AATAAAATTA TTTAAAATTT   
  
  
- TAATTTTTAT AAAATTTTTG GTTGGTTTTT TCCGTTGGAT AATGCTTGCT TGATCCTCTG TACTACTAGC   
  
  
- GACCTTCTTC TCGTTGTTCT TCGTTACTGT AGTGACTTTC TTATTGCCAA TCAGGCCACT TAAAAGTAAT   
  
  
- CGACCTTCAA TGACGATCAT CAATTAATCA ATCTTCTCGA CAACTCAATC AATCATCAAT CATCAATTAA   
  
  
- TCAATCTTTT CGAGAACTCA ACCAATCATC AATCGTCAAC TTATTTCCCC AATCTAGTTA ACATTAATCC   
  
  
- TTTTTTGGTA ACATATATTT GTCCACATAC TTTTGCCCAA CTCCTATTCT TTTATGTCTT CTAATTAGAG   
  
  
- TAAAGAGACG AGTAAGAAGA GACGAGAGAG AACAAAGACA AGAAAGAGTT AAAGACAAGA GAAAAGAAAG   
  
  
- ATTAGAACAT TTCGACTCGT ATAGTAGTGG TTCAGTAGTG CACATCTTCG TTTCCCTGTT GTTCAGTAGT   
  
  
- GGCTTCTTCT TATATCAACT GGAAGCTCTT GGGTCAGTT

+     G-box

| Site Name | Organism | Position | Strand | Matrix score. | sequence | function |
| --- | --- | --- | --- | --- | --- | --- |
| G-box | Nicotiana plumbaginifolia | 498 | + | 10 | CAGACGTGGCA | cis-acting regulatory element involved in light responsiveness |
| G-box | Arabidopsis thaliana | 1928 | - | 6 | CACGTG | cis-acting regulatory element involved in light responsiveness |

>Potri.018G038100.1   
+ GTTGCTGTCT TTTTTTTTTA TCATGTTATC AAATTAATGG AAATTTAACT CTATTATCAA AATTCTCTTT   
  
  
+ TTTATAAACA TGATAATCTT TTTTATTTTA TTTTCAAGTT AAAAATACTT GGTCCGGCCT GGGACGATGC   
  
  
+ GCTTGCCAAT CTAGTCAATC CTAAATTTAC TAATACGAGT AAGTAATAGC CGAAGGAGCA AAGCTCCAGT   
  
  
+ CCGGCATCTT GTTCAGAACA ATATTACATC CCTGAAATGC CTCGTGAATT GGGTGTCTCT TCCAACTTTC   
  
  
+ TCCAGCAATT TTCTCCCAAC TTCTGCAAGC AGTGATTATC AATTTGAATT ACAGGAGCTA CCTAACATAT   
  
  
+ CCGCCCTAGA ACGAAGGCAG GAATCATCAG CTTTGCCACC TCTTTGCATA ACTGCTACTG AAAGCCATTA   
  
  
+ GCTGGTGAAT GCTCCATTTC AAACTTCCAT TGGCCCGAGT ACTGCTCCTG CTTCCATTGG AAATATCGCT   
  
  
+ GCGCAATCAG ACGAGGCAGG CATTCATATC TGATGATAAA TGTTGCAGGA TTAATTCTAT TTTCGCCGGC   
  
  
+ AGCAAATGGT GGGATTTTTT ACACTGAAAA GTGTTTCAAA AGACTCGAAT TAATGTGGTC GATTTTGGCG   
  
  
+ GGGGCGTGGA ATGGATTCCT CTCTTCCTCA ATCATCAAAC ATCCAGATAC CTGAAACAGA GAGAAGCCCA   
  
  
+ TCTTCAAAAT CATATCGTCA TCGGAGGTCC TTCGCTGCAA TCGGCTGATG ACGGTGCACT TTTTGATGGT   
  
  
+ AGCGAACCAG ATCAGTCAAT GGTCAAATCA TTTGGAAATC AACGAGCTAC AGTTTATCCC TCCTGAATCC   
  
  
+ ATCTTTGAGA ACCTAAAAGA AATTAATTAA AATTAACGAA AGAGAAAAGA GAGAGGTGGT AGAGTTCATA   
  
  
+ GTTGTCGGAT CGGTCCGGAA ATTGATTTAA TTTAGAAATT AATATAGAAA AGAGTTTTGA GTAATTATAT   
  
  
+ TATCTTTAAC GGAGGCAAAC TAAATTAATA TTTTTATTAA AAAAATAATT TTTTTAAAAA AAACTTTTTA   
  
  
+ GACTTTTGTA TAAATTAATC AAATTAAATT AACAAGATTT TACTAAATTA ATAATTTATT AAATTTAACT   
  
  
+ TAAAATCTAA TTTAATATCA CAGCCTGCTT GATATTATAA TATCGGTTGT TTCTGGTTTA AAAATATATT   
  
  
+ AAAATATTAT TTTTTAATTT TTTAAAATTT AATTTTAACA TTAATAAATT AAAATGATCT AATTTTAATT   
  
  
+ TTTTTAAAAA AACTAAGATT CTTTTCAAAA ACATCTTTTT ACGGTGGCTT AGACTCTATT CGTTTTTATA   
  
  
+ TTTTAAAAAT TCTTTTAAAA AAATTAAAAA TTTTTAATTT TTTTCACTTT AATTTTTTTT ATATTTTTAA   
  
  
+ ATTATTTTAA TATGTTAATA TTAAAAATAA AATTTTAAAA TAAAAAAATA TTATTTTAAT AAATTTTAAA   
  
  
+ ATTAAAAATA TTTTAAAAAC CAACCAAAAA AGGCAACCTA TTACGAACGA ACTAGGAGAC ATGATGATCG   
  
  
+ CTGGAAGAAG AGCAACAAGA AGCAATGACA TCACTGAAAG AATAACGGTT AGTCCGGTGA ATTTTCATTA   
  
  
+ GCTGGAAGTT ACTGCTAGTA GTTAATTAGT TAGAAGAGCT GTTGAGTTAG TTAGTAGTTA GTAGTTAATT   
  
  
+ AGTTAGAAAA GCTCTTGAGT TGGTTAGTAG TTAGCAGTTG AATAAAGGGG TTAGATCAAT TGTAATTAGG   
  
  
+ AAAAAACCAT TGTATATAAA CAGGTGTATG AAAACGGGTT GAGGATAAGA AAATACAGAA GATTAATCTC   
  
  
+ ATTTCTCTGC TCATTCTTCT CTGCTCTCTC TTGTTTCTGT TCTTTCTCAA TTTCTGTTCT CTTTTCTTTC   
  
  
+ TAATCTTGTA AAGCTGAGCA TATCATCACC AAGTCATCAC GTGTAGAAGC AAAGGGACAA CAAGTCATCA   
  
  
+ CCGAAGAAGA ATATAGTTGA CCTTCGAGAA CCCAGTCAA  

- CAACGACAGA AAAAAAAAAT AGTACAATAG TTTAATTACC TTTAAATTGA GATAATAGTT TTAAGAGAAA   
  
  
- AAATATTTGT ACTATTAGAA AAAATAAAAT AAAAGTTCAA TTTTTATGAA CCAGGCCGGA CCCTGCTACG   
  
  
- CGAACGGTTA GATCAGTTAG GATTTAAATG ATTATGCTCA TTCATTATCG GCTTCCTCGT TTCGAGGTCA   
  
  
- GGCCGTAGAA CAAGTCTTGT TATAATGTAG GGACTTTACG GAGCACTTAA CCCACAGAGA AGGTTGAAAG   
  
  
- AGGTCGTTAA AAGAGGGTTG AAGACGTTCG TCACTAATAG TTAAACTTAA TGTCCTCGAT GGATTGTATA   
  
  
- GGCGGGATCT TGCTTCCGTC CTTAGTAGTC GAAACGGTGG AGAAACGTAT TGACGATGAC TTTCGGTAAT   
  
  
- CGACCACTTA CGAGGTAAAG TTTGAAGGTA ACCGGGCTCA TGACGAGGAC GAAGGTAACC TTTATAGCGA   
  
  
- CGCGTTAGTC TGCTCCGTCC GTAAGTATAG ACTACTATTT ACAACGTCCT AATTAAGATA AAAGCGGCCG   
  
  
- TCGTTTACCA CCCTAAAAAA TGTGACTTTT CACAAAGTTT TCTGAGCTTA ATTACACCAG CTAAAACCGC   
  
  
- CCCCGCACCT TACCTAAGGA GAGAAGGAGT TAGTAGTTTG TAGGTCTATG GACTTTGTCT CTCTTCGGGT   
  
  
- AGAAGTTTTA GTATAGCAGT AGCCTCCAGG AAGCGACGTT AGCCGACTAC TGCCACGTGA AAAACTACCA   
  
  
- TCGCTTGGTC TAGTCAGTTA CCAGTTTAGT AAACCTTTAG TTGCTCGATG TCAAATAGGG AGGACTTAGG   
  
  
- TAGAAACTCT TGGATTTTCT TTAATTAATT TTAATTGCTT TCTCTTTTCT CTCTCCACCA TCTCAAGTAT   
  
  
- CAACAGCCTA GCCAGGCCTT TAACTAAATT AAATCTTTAA TTATATCTTT TCTCAAAACT CATTAATATA   
  
  
- ATAGAAATTG CCTCCGTTTG ATTTAATTAT AAAAATAATT TTTTTATTAA AAAAATTTTT TTTGAAAAAT   
  
  
- CTGAAAACAT ATTTAATTAG TTTAATTTAA TTGTTCTAAA ATGATTTAAT TATTAAATAA TTTAAATTGA   
  
  
- ATTTTAGATT AAATTATAGT GTCGGACGAA CTATAATATT ATAGCCAACA AAGACCAAAT TTTTATATAA   
  
  
- TTTTATAATA AAAAATTAAA AAATTTTAAA TTAAAATTGT AATTATTTAA TTTTACTAGA TTAAAATTAA   
  
  
- AAAAATTTTT TTGATTCTAA GAAAAGTTTT TGTAGAAAAA TGCCACCGAA TCTGAGATAA GCAAAAATAT   
  
  
- AAAATTTTTA AGAAAATTTT TTTAATTTTT AAAAATTAAA AAAAGTGAAA TTAAAAAAAA TATAAAAATT   
  
  
- TAATAAAATT ATACAATTAT AATTTTTATT TTAAAATTTT ATTTTTTTAT AATAAAATTA TTTAAAATTT   
  
  
- TAATTTTTAT AAAATTTTTG GTTGGTTTTT TCCGTTGGAT AATGCTTGCT TGATCCTCTG TACTACTAGC   
  
  
- GACCTTCTTC TCGTTGTTCT TCGTTACTGT AGTGACTTTC TTATTGCCAA TCAGGCCACT TAAAAGTAAT   
  
  
- CGACCTTCAA TGACGATCAT CAATTAATCA ATCTTCTCGA CAACTCAATC AATCATCAAT CATCAATTAA   
  
  
- TCAATCTTTT CGAGAACTCA ACCAATCATC AATCGTCAAC TTATTTCCCC AATCTAGTTA ACATTAATCC   
  
  
- TTTTTTGGTA ACATATATTT GTCCACATAC TTTTGCCCAA CTCCTATTCT TTTATGTCTT CTAATTAGAG   
  
  
- TAAAGAGACG AGTAAGAAGA GACGAGAGAG AACAAAGACA AGAAAGAGTT AAAGACAAGA GAAAAGAAAG   
  
  
- ATTAGAACAT TTCGACTCGT ATAGTAGTGG TTCAGTAGTG CACATCTTCG TTTCCCTGTT GTTCAGTAGT   
  
  
- GGCTTCTTCT TATATCAACT GGAAGCTCTT GGGTCAGTT

+     GC-motif

| Site Name | Organism | Position | Strand | Matrix score. | sequence | function |
| --- | --- | --- | --- | --- | --- | --- |
| GC-motif | Zea mays | 629 | - | 6 | CCCCCG | enhancer-like element involved in anoxic specific inducibility |

>Potri.018G038100.1   
+ GTTGCTGTCT TTTTTTTTTA TCATGTTATC AAATTAATGG AAATTTAACT CTATTATCAA AATTCTCTTT   
  
  
+ TTTATAAACA TGATAATCTT TTTTATTTTA TTTTCAAGTT AAAAATACTT GGTCCGGCCT GGGACGATGC   
  
  
+ GCTTGCCAAT CTAGTCAATC CTAAATTTAC TAATACGAGT AAGTAATAGC CGAAGGAGCA AAGCTCCAGT   
  
  
+ CCGGCATCTT GTTCAGAACA ATATTACATC CCTGAAATGC CTCGTGAATT GGGTGTCTCT TCCAACTTTC   
  
  
+ TCCAGCAATT TTCTCCCAAC TTCTGCAAGC AGTGATTATC AATTTGAATT ACAGGAGCTA CCTAACATAT   
  
  
+ CCGCCCTAGA ACGAAGGCAG GAATCATCAG CTTTGCCACC TCTTTGCATA ACTGCTACTG AAAGCCATTA   
  
  
+ GCTGGTGAAT GCTCCATTTC AAACTTCCAT TGGCCCGAGT ACTGCTCCTG CTTCCATTGG AAATATCGCT   
  
  
+ GCGCAATCAG ACGAGGCAGG CATTCATATC TGATGATAAA TGTTGCAGGA TTAATTCTAT TTTCGCCGGC   
  
  
+ AGCAAATGGT GGGATTTTTT ACACTGAAAA GTGTTTCAAA AGACTCGAAT TAATGTGGTC GATTTTGGCG   
  
  
+ GGGGCGTGGA ATGGATTCCT CTCTTCCTCA ATCATCAAAC ATCCAGATAC CTGAAACAGA GAGAAGCCCA   
  
  
+ TCTTCAAAAT CATATCGTCA TCGGAGGTCC TTCGCTGCAA TCGGCTGATG ACGGTGCACT TTTTGATGGT   
  
  
+ AGCGAACCAG ATCAGTCAAT GGTCAAATCA TTTGGAAATC AACGAGCTAC AGTTTATCCC TCCTGAATCC   
  
  
+ ATCTTTGAGA ACCTAAAAGA AATTAATTAA AATTAACGAA AGAGAAAAGA GAGAGGTGGT AGAGTTCATA   
  
  
+ GTTGTCGGAT CGGTCCGGAA ATTGATTTAA TTTAGAAATT AATATAGAAA AGAGTTTTGA GTAATTATAT   
  
  
+ TATCTTTAAC GGAGGCAAAC TAAATTAATA TTTTTATTAA AAAAATAATT TTTTTAAAAA AAACTTTTTA   
  
  
+ GACTTTTGTA TAAATTAATC AAATTAAATT AACAAGATTT TACTAAATTA ATAATTTATT AAATTTAACT   
  
  
+ TAAAATCTAA TTTAATATCA CAGCCTGCTT GATATTATAA TATCGGTTGT TTCTGGTTTA AAAATATATT   
  
  
+ AAAATATTAT TTTTTAATTT TTTAAAATTT AATTTTAACA TTAATAAATT AAAATGATCT AATTTTAATT   
  
  
+ TTTTTAAAAA AACTAAGATT CTTTTCAAAA ACATCTTTTT ACGGTGGCTT AGACTCTATT CGTTTTTATA   
  
  
+ TTTTAAAAAT TCTTTTAAAA AAATTAAAAA TTTTTAATTT TTTTCACTTT AATTTTTTTT ATATTTTTAA   
  
  
+ ATTATTTTAA TATGTTAATA TTAAAAATAA AATTTTAAAA TAAAAAAATA TTATTTTAAT AAATTTTAAA   
  
  
+ ATTAAAAATA TTTTAAAAAC CAACCAAAAA AGGCAACCTA TTACGAACGA ACTAGGAGAC ATGATGATCG   
  
  
+ CTGGAAGAAG AGCAACAAGA AGCAATGACA TCACTGAAAG AATAACGGTT AGTCCGGTGA ATTTTCATTA   
  
  
+ GCTGGAAGTT ACTGCTAGTA GTTAATTAGT TAGAAGAGCT GTTGAGTTAG TTAGTAGTTA GTAGTTAATT   
  
  
+ AGTTAGAAAA GCTCTTGAGT TGGTTAGTAG TTAGCAGTTG AATAAAGGGG TTAGATCAAT TGTAATTAGG   
  
  
+ AAAAAACCAT TGTATATAAA CAGGTGTATG AAAACGGGTT GAGGATAAGA AAATACAGAA GATTAATCTC   
  
  
+ ATTTCTCTGC TCATTCTTCT CTGCTCTCTC TTGTTTCTGT TCTTTCTCAA TTTCTGTTCT CTTTTCTTTC   
  
  
+ TAATCTTGTA AAGCTGAGCA TATCATCACC AAGTCATCAC GTGTAGAAGC AAAGGGACAA CAAGTCATCA   
  
  
+ CCGAAGAAGA ATATAGTTGA CCTTCGAGAA CCCAGTCAA  

- CAACGACAGA AAAAAAAAAT AGTACAATAG TTTAATTACC TTTAAATTGA GATAATAGTT TTAAGAGAAA   
  
  
- AAATATTTGT ACTATTAGAA AAAATAAAAT AAAAGTTCAA TTTTTATGAA CCAGGCCGGA CCCTGCTACG   
  
  
- CGAACGGTTA GATCAGTTAG GATTTAAATG ATTATGCTCA TTCATTATCG GCTTCCTCGT TTCGAGGTCA   
  
  
- GGCCGTAGAA CAAGTCTTGT TATAATGTAG GGACTTTACG GAGCACTTAA CCCACAGAGA AGGTTGAAAG   
  
  
- AGGTCGTTAA AAGAGGGTTG AAGACGTTCG TCACTAATAG TTAAACTTAA TGTCCTCGAT GGATTGTATA   
  
  
- GGCGGGATCT TGCTTCCGTC CTTAGTAGTC GAAACGGTGG AGAAACGTAT TGACGATGAC TTTCGGTAAT   
  
  
- CGACCACTTA CGAGGTAAAG TTTGAAGGTA ACCGGGCTCA TGACGAGGAC GAAGGTAACC TTTATAGCGA   
  
  
- CGCGTTAGTC TGCTCCGTCC GTAAGTATAG ACTACTATTT ACAACGTCCT AATTAAGATA AAAGCGGCCG   
  
  
- TCGTTTACCA CCCTAAAAAA TGTGACTTTT CACAAAGTTT TCTGAGCTTA ATTACACCAG CTAAAACCGC   
  
  
- CCCCGCACCT TACCTAAGGA GAGAAGGAGT TAGTAGTTTG TAGGTCTATG GACTTTGTCT CTCTTCGGGT   
  
  
- AGAAGTTTTA GTATAGCAGT AGCCTCCAGG AAGCGACGTT AGCCGACTAC TGCCACGTGA AAAACTACCA   
  
  
- TCGCTTGGTC TAGTCAGTTA CCAGTTTAGT AAACCTTTAG TTGCTCGATG TCAAATAGGG AGGACTTAGG   
  
  
- TAGAAACTCT TGGATTTTCT TTAATTAATT TTAATTGCTT TCTCTTTTCT CTCTCCACCA TCTCAAGTAT   
  
  
- CAACAGCCTA GCCAGGCCTT TAACTAAATT AAATCTTTAA TTATATCTTT TCTCAAAACT CATTAATATA   
  
  
- ATAGAAATTG CCTCCGTTTG ATTTAATTAT AAAAATAATT TTTTTATTAA AAAAATTTTT TTTGAAAAAT   
  
  
- CTGAAAACAT ATTTAATTAG TTTAATTTAA TTGTTCTAAA ATGATTTAAT TATTAAATAA TTTAAATTGA   
  
  
- ATTTTAGATT AAATTATAGT GTCGGACGAA CTATAATATT ATAGCCAACA AAGACCAAAT TTTTATATAA   
  
  
- TTTTATAATA AAAAATTAAA AAATTTTAAA TTAAAATTGT AATTATTTAA TTTTACTAGA TTAAAATTAA   
  
  
- AAAAATTTTT TTGATTCTAA GAAAAGTTTT TGTAGAAAAA TGCCACCGAA TCTGAGATAA GCAAAAATAT   
  
  
- AAAATTTTTA AGAAAATTTT TTTAATTTTT AAAAATTAAA AAAAGTGAAA TTAAAAAAAA TATAAAAATT   
  
  
- TAATAAAATT ATACAATTAT AATTTTTATT TTAAAATTTT ATTTTTTTAT AATAAAATTA TTTAAAATTT   
  
  
- TAATTTTTAT AAAATTTTTG GTTGGTTTTT TCCGTTGGAT AATGCTTGCT TGATCCTCTG TACTACTAGC   
  
  
- GACCTTCTTC TCGTTGTTCT TCGTTACTGT AGTGACTTTC TTATTGCCAA TCAGGCCACT TAAAAGTAAT   
  
  
- CGACCTTCAA TGACGATCAT CAATTAATCA ATCTTCTCGA CAACTCAATC AATCATCAAT CATCAATTAA   
  
  
- TCAATCTTTT CGAGAACTCA ACCAATCATC AATCGTCAAC TTATTTCCCC AATCTAGTTA ACATTAATCC   
  
  
- TTTTTTGGTA ACATATATTT GTCCACATAC TTTTGCCCAA CTCCTATTCT TTTATGTCTT CTAATTAGAG   
  
  
- TAAAGAGACG AGTAAGAAGA GACGAGAGAG AACAAAGACA AGAAAGAGTT AAAGACAAGA GAAAAGAAAG   
  
  
- ATTAGAACAT TTCGACTCGT ATAGTAGTGG TTCAGTAGTG CACATCTTCG TTTCCCTGTT GTTCAGTAGT   
  
  
- GGCTTCTTCT TATATCAACT GGAAGCTCTT GGGTCAGTT

+     MBS

| Site Name | Organism | Position | Strand | Matrix score. | sequence | function |
| --- | --- | --- | --- | --- | --- | --- |
| MBS | Arabidopsis thaliana | 1715 | - | 6 | CAACTG | MYB binding site involved in drought-inducibility |

>Potri.018G038100.1   
+ GTTGCTGTCT TTTTTTTTTA TCATGTTATC AAATTAATGG AAATTTAACT CTATTATCAA AATTCTCTTT   
  
  
+ TTTATAAACA TGATAATCTT TTTTATTTTA TTTTCAAGTT AAAAATACTT GGTCCGGCCT GGGACGATGC   
  
  
+ GCTTGCCAAT CTAGTCAATC CTAAATTTAC TAATACGAGT AAGTAATAGC CGAAGGAGCA AAGCTCCAGT   
  
  
+ CCGGCATCTT GTTCAGAACA ATATTACATC CCTGAAATGC CTCGTGAATT GGGTGTCTCT TCCAACTTTC   
  
  
+ TCCAGCAATT TTCTCCCAAC TTCTGCAAGC AGTGATTATC AATTTGAATT ACAGGAGCTA CCTAACATAT   
  
  
+ CCGCCCTAGA ACGAAGGCAG GAATCATCAG CTTTGCCACC TCTTTGCATA ACTGCTACTG AAAGCCATTA   
  
  
+ GCTGGTGAAT GCTCCATTTC AAACTTCCAT TGGCCCGAGT ACTGCTCCTG CTTCCATTGG AAATATCGCT   
  
  
+ GCGCAATCAG ACGAGGCAGG CATTCATATC TGATGATAAA TGTTGCAGGA TTAATTCTAT TTTCGCCGGC   
  
  
+ AGCAAATGGT GGGATTTTTT ACACTGAAAA GTGTTTCAAA AGACTCGAAT TAATGTGGTC GATTTTGGCG   
  
  
+ GGGGCGTGGA ATGGATTCCT CTCTTCCTCA ATCATCAAAC ATCCAGATAC CTGAAACAGA GAGAAGCCCA   
  
  
+ TCTTCAAAAT CATATCGTCA TCGGAGGTCC TTCGCTGCAA TCGGCTGATG ACGGTGCACT TTTTGATGGT   
  
  
+ AGCGAACCAG ATCAGTCAAT GGTCAAATCA TTTGGAAATC AACGAGCTAC AGTTTATCCC TCCTGAATCC   
  
  
+ ATCTTTGAGA ACCTAAAAGA AATTAATTAA AATTAACGAA AGAGAAAAGA GAGAGGTGGT AGAGTTCATA   
  
  
+ GTTGTCGGAT CGGTCCGGAA ATTGATTTAA TTTAGAAATT AATATAGAAA AGAGTTTTGA GTAATTATAT   
  
  
+ TATCTTTAAC GGAGGCAAAC TAAATTAATA TTTTTATTAA AAAAATAATT TTTTTAAAAA AAACTTTTTA   
  
  
+ GACTTTTGTA TAAATTAATC AAATTAAATT AACAAGATTT TACTAAATTA ATAATTTATT AAATTTAACT   
  
  
+ TAAAATCTAA TTTAATATCA CAGCCTGCTT GATATTATAA TATCGGTTGT TTCTGGTTTA AAAATATATT   
  
  
+ AAAATATTAT TTTTTAATTT TTTAAAATTT AATTTTAACA TTAATAAATT AAAATGATCT AATTTTAATT   
  
  
+ TTTTTAAAAA AACTAAGATT CTTTTCAAAA ACATCTTTTT ACGGTGGCTT AGACTCTATT CGTTTTTATA   
  
  
+ TTTTAAAAAT TCTTTTAAAA AAATTAAAAA TTTTTAATTT TTTTCACTTT AATTTTTTTT ATATTTTTAA   
  
  
+ ATTATTTTAA TATGTTAATA TTAAAAATAA AATTTTAAAA TAAAAAAATA TTATTTTAAT AAATTTTAAA   
  
  
+ ATTAAAAATA TTTTAAAAAC CAACCAAAAA AGGCAACCTA TTACGAACGA ACTAGGAGAC ATGATGATCG   
  
  
+ CTGGAAGAAG AGCAACAAGA AGCAATGACA TCACTGAAAG AATAACGGTT AGTCCGGTGA ATTTTCATTA   
  
  
+ GCTGGAAGTT ACTGCTAGTA GTTAATTAGT TAGAAGAGCT GTTGAGTTAG TTAGTAGTTA GTAGTTAATT   
  
  
+ AGTTAGAAAA GCTCTTGAGT TGGTTAGTAG TTAGCAGTTG AATAAAGGGG TTAGATCAAT TGTAATTAGG   
  
  
+ AAAAAACCAT TGTATATAAA CAGGTGTATG AAAACGGGTT GAGGATAAGA AAATACAGAA GATTAATCTC   
  
  
+ ATTTCTCTGC TCATTCTTCT CTGCTCTCTC TTGTTTCTGT TCTTTCTCAA TTTCTGTTCT CTTTTCTTTC   
  
  
+ TAATCTTGTA AAGCTGAGCA TATCATCACC AAGTCATCAC GTGTAGAAGC AAAGGGACAA CAAGTCATCA   
  
  
+ CCGAAGAAGA ATATAGTTGA CCTTCGAGAA CCCAGTCAA  

- CAACGACAGA AAAAAAAAAT AGTACAATAG TTTAATTACC TTTAAATTGA GATAATAGTT TTAAGAGAAA   
  
  
- AAATATTTGT ACTATTAGAA AAAATAAAAT AAAAGTTCAA TTTTTATGAA CCAGGCCGGA CCCTGCTACG   
  
  
- CGAACGGTTA GATCAGTTAG GATTTAAATG ATTATGCTCA TTCATTATCG GCTTCCTCGT TTCGAGGTCA   
  
  
- GGCCGTAGAA CAAGTCTTGT TATAATGTAG GGACTTTACG GAGCACTTAA CCCACAGAGA AGGTTGAAAG   
  
  
- AGGTCGTTAA AAGAGGGTTG AAGACGTTCG TCACTAATAG TTAAACTTAA TGTCCTCGAT GGATTGTATA   
  
  
- GGCGGGATCT TGCTTCCGTC CTTAGTAGTC GAAACGGTGG AGAAACGTAT TGACGATGAC TTTCGGTAAT   
  
  
- CGACCACTTA CGAGGTAAAG TTTGAAGGTA ACCGGGCTCA TGACGAGGAC GAAGGTAACC TTTATAGCGA   
  
  
- CGCGTTAGTC TGCTCCGTCC GTAAGTATAG ACTACTATTT ACAACGTCCT AATTAAGATA AAAGCGGCCG   
  
  
- TCGTTTACCA CCCTAAAAAA TGTGACTTTT CACAAAGTTT TCTGAGCTTA ATTACACCAG CTAAAACCGC   
  
  
- CCCCGCACCT TACCTAAGGA GAGAAGGAGT TAGTAGTTTG TAGGTCTATG GACTTTGTCT CTCTTCGGGT   
  
  
- AGAAGTTTTA GTATAGCAGT AGCCTCCAGG AAGCGACGTT AGCCGACTAC TGCCACGTGA AAAACTACCA   
  
  
- TCGCTTGGTC TAGTCAGTTA CCAGTTTAGT AAACCTTTAG TTGCTCGATG TCAAATAGGG AGGACTTAGG   
  
  
- TAGAAACTCT TGGATTTTCT TTAATTAATT TTAATTGCTT TCTCTTTTCT CTCTCCACCA TCTCAAGTAT   
  
  
- CAACAGCCTA GCCAGGCCTT TAACTAAATT AAATCTTTAA TTATATCTTT TCTCAAAACT CATTAATATA   
  
  
- ATAGAAATTG CCTCCGTTTG ATTTAATTAT AAAAATAATT TTTTTATTAA AAAAATTTTT TTTGAAAAAT   
  
  
- CTGAAAACAT ATTTAATTAG TTTAATTTAA TTGTTCTAAA ATGATTTAAT TATTAAATAA TTTAAATTGA   
  
  
- ATTTTAGATT AAATTATAGT GTCGGACGAA CTATAATATT ATAGCCAACA AAGACCAAAT TTTTATATAA   
  
  
- TTTTATAATA AAAAATTAAA AAATTTTAAA TTAAAATTGT AATTATTTAA TTTTACTAGA TTAAAATTAA   
  
  
- AAAAATTTTT TTGATTCTAA GAAAAGTTTT TGTAGAAAAA TGCCACCGAA TCTGAGATAA GCAAAAATAT   
  
  
- AAAATTTTTA AGAAAATTTT TTTAATTTTT AAAAATTAAA AAAAGTGAAA TTAAAAAAAA TATAAAAATT   
  
  
- TAATAAAATT ATACAATTAT AATTTTTATT TTAAAATTTT ATTTTTTTAT AATAAAATTA TTTAAAATTT   
  
  
- TAATTTTTAT AAAATTTTTG GTTGGTTTTT TCCGTTGGAT AATGCTTGCT TGATCCTCTG TACTACTAGC   
  
  
- GACCTTCTTC TCGTTGTTCT TCGTTACTGT AGTGACTTTC TTATTGCCAA TCAGGCCACT TAAAAGTAAT   
  
  
- CGACCTTCAA TGACGATCAT CAATTAATCA ATCTTCTCGA CAACTCAATC AATCATCAAT CATCAATTAA   
  
  
- TCAATCTTTT CGAGAACTCA ACCAATCATC AATCGTCAAC TTATTTCCCC AATCTAGTTA ACATTAATCC   
  
  
- TTTTTTGGTA ACATATATTT GTCCACATAC TTTTGCCCAA CTCCTATTCT TTTATGTCTT CTAATTAGAG   
  
  
- TAAAGAGACG AGTAAGAAGA GACGAGAGAG AACAAAGACA AGAAAGAGTT AAAGACAAGA GAAAAGAAAG   
  
  
- ATTAGAACAT TTCGACTCGT ATAGTAGTGG TTCAGTAGTG CACATCTTCG TTTCCCTGTT GTTCAGTAGT   
  
  
- GGCTTCTTCT TATATCAACT GGAAGCTCTT GGGTCAGTT

+     MRE

| Site Name | Organism | Position | Strand | Matrix score. | sequence | function |
| --- | --- | --- | --- | --- | --- | --- |
| MRE | Petroselinum crispum | 850 | + | 7 | AACCTAA | MYB binding site involved in light responsiveness |

>Potri.018G038100.1   
+ GTTGCTGTCT TTTTTTTTTA TCATGTTATC AAATTAATGG AAATTTAACT CTATTATCAA AATTCTCTTT   
  
  
+ TTTATAAACA TGATAATCTT TTTTATTTTA TTTTCAAGTT AAAAATACTT GGTCCGGCCT GGGACGATGC   
  
  
+ GCTTGCCAAT CTAGTCAATC CTAAATTTAC TAATACGAGT AAGTAATAGC CGAAGGAGCA AAGCTCCAGT   
  
  
+ CCGGCATCTT GTTCAGAACA ATATTACATC CCTGAAATGC CTCGTGAATT GGGTGTCTCT TCCAACTTTC   
  
  
+ TCCAGCAATT TTCTCCCAAC TTCTGCAAGC AGTGATTATC AATTTGAATT ACAGGAGCTA CCTAACATAT   
  
  
+ CCGCCCTAGA ACGAAGGCAG GAATCATCAG CTTTGCCACC TCTTTGCATA ACTGCTACTG AAAGCCATTA   
  
  
+ GCTGGTGAAT GCTCCATTTC AAACTTCCAT TGGCCCGAGT ACTGCTCCTG CTTCCATTGG AAATATCGCT   
  
  
+ GCGCAATCAG ACGAGGCAGG CATTCATATC TGATGATAAA TGTTGCAGGA TTAATTCTAT TTTCGCCGGC   
  
  
+ AGCAAATGGT GGGATTTTTT ACACTGAAAA GTGTTTCAAA AGACTCGAAT TAATGTGGTC GATTTTGGCG   
  
  
+ GGGGCGTGGA ATGGATTCCT CTCTTCCTCA ATCATCAAAC ATCCAGATAC CTGAAACAGA GAGAAGCCCA   
  
  
+ TCTTCAAAAT CATATCGTCA TCGGAGGTCC TTCGCTGCAA TCGGCTGATG ACGGTGCACT TTTTGATGGT   
  
  
+ AGCGAACCAG ATCAGTCAAT GGTCAAATCA TTTGGAAATC AACGAGCTAC AGTTTATCCC TCCTGAATCC   
  
  
+ ATCTTTGAGA ACCTAAAAGA AATTAATTAA AATTAACGAA AGAGAAAAGA GAGAGGTGGT AGAGTTCATA   
  
  
+ GTTGTCGGAT CGGTCCGGAA ATTGATTTAA TTTAGAAATT AATATAGAAA AGAGTTTTGA GTAATTATAT   
  
  
+ TATCTTTAAC GGAGGCAAAC TAAATTAATA TTTTTATTAA AAAAATAATT TTTTTAAAAA AAACTTTTTA   
  
  
+ GACTTTTGTA TAAATTAATC AAATTAAATT AACAAGATTT TACTAAATTA ATAATTTATT AAATTTAACT   
  
  
+ TAAAATCTAA TTTAATATCA CAGCCTGCTT GATATTATAA TATCGGTTGT TTCTGGTTTA AAAATATATT   
  
  
+ AAAATATTAT TTTTTAATTT TTTAAAATTT AATTTTAACA TTAATAAATT AAAATGATCT AATTTTAATT   
  
  
+ TTTTTAAAAA AACTAAGATT CTTTTCAAAA ACATCTTTTT ACGGTGGCTT AGACTCTATT CGTTTTTATA   
  
  
+ TTTTAAAAAT TCTTTTAAAA AAATTAAAAA TTTTTAATTT TTTTCACTTT AATTTTTTTT ATATTTTTAA   
  
  
+ ATTATTTTAA TATGTTAATA TTAAAAATAA AATTTTAAAA TAAAAAAATA TTATTTTAAT AAATTTTAAA   
  
  
+ ATTAAAAATA TTTTAAAAAC CAACCAAAAA AGGCAACCTA TTACGAACGA ACTAGGAGAC ATGATGATCG   
  
  
+ CTGGAAGAAG AGCAACAAGA AGCAATGACA TCACTGAAAG AATAACGGTT AGTCCGGTGA ATTTTCATTA   
  
  
+ GCTGGAAGTT ACTGCTAGTA GTTAATTAGT TAGAAGAGCT GTTGAGTTAG TTAGTAGTTA GTAGTTAATT   
  
  
+ AGTTAGAAAA GCTCTTGAGT TGGTTAGTAG TTAGCAGTTG AATAAAGGGG TTAGATCAAT TGTAATTAGG   
  
  
+ AAAAAACCAT TGTATATAAA CAGGTGTATG AAAACGGGTT GAGGATAAGA AAATACAGAA GATTAATCTC   
  
  
+ ATTTCTCTGC TCATTCTTCT CTGCTCTCTC TTGTTTCTGT TCTTTCTCAA TTTCTGTTCT CTTTTCTTTC   
  
  
+ TAATCTTGTA AAGCTGAGCA TATCATCACC AAGTCATCAC GTGTAGAAGC AAAGGGACAA CAAGTCATCA   
  
  
+ CCGAAGAAGA ATATAGTTGA CCTTCGAGAA CCCAGTCAA  

- CAACGACAGA AAAAAAAAAT AGTACAATAG TTTAATTACC TTTAAATTGA GATAATAGTT TTAAGAGAAA   
  
  
- AAATATTTGT ACTATTAGAA AAAATAAAAT AAAAGTTCAA TTTTTATGAA CCAGGCCGGA CCCTGCTACG   
  
  
- CGAACGGTTA GATCAGTTAG GATTTAAATG ATTATGCTCA TTCATTATCG GCTTCCTCGT TTCGAGGTCA   
  
  
- GGCCGTAGAA CAAGTCTTGT TATAATGTAG GGACTTTACG GAGCACTTAA CCCACAGAGA AGGTTGAAAG   
  
  
- AGGTCGTTAA AAGAGGGTTG AAGACGTTCG TCACTAATAG TTAAACTTAA TGTCCTCGAT GGATTGTATA   
  
  
- GGCGGGATCT TGCTTCCGTC CTTAGTAGTC GAAACGGTGG AGAAACGTAT TGACGATGAC TTTCGGTAAT   
  
  
- CGACCACTTA CGAGGTAAAG TTTGAAGGTA ACCGGGCTCA TGACGAGGAC GAAGGTAACC TTTATAGCGA   
  
  
- CGCGTTAGTC TGCTCCGTCC GTAAGTATAG ACTACTATTT ACAACGTCCT AATTAAGATA AAAGCGGCCG   
  
  
- TCGTTTACCA CCCTAAAAAA TGTGACTTTT CACAAAGTTT TCTGAGCTTA ATTACACCAG CTAAAACCGC   
  
  
- CCCCGCACCT TACCTAAGGA GAGAAGGAGT TAGTAGTTTG TAGGTCTATG GACTTTGTCT CTCTTCGGGT   
  
  
- AGAAGTTTTA GTATAGCAGT AGCCTCCAGG AAGCGACGTT AGCCGACTAC TGCCACGTGA AAAACTACCA   
  
  
- TCGCTTGGTC TAGTCAGTTA CCAGTTTAGT AAACCTTTAG TTGCTCGATG TCAAATAGGG AGGACTTAGG   
  
  
- TAGAAACTCT TGGATTTTCT TTAATTAATT TTAATTGCTT TCTCTTTTCT CTCTCCACCA TCTCAAGTAT   
  
  
- CAACAGCCTA GCCAGGCCTT TAACTAAATT AAATCTTTAA TTATATCTTT TCTCAAAACT CATTAATATA   
  
  
- ATAGAAATTG CCTCCGTTTG ATTTAATTAT AAAAATAATT TTTTTATTAA AAAAATTTTT TTTGAAAAAT   
  
  
- CTGAAAACAT ATTTAATTAG TTTAATTTAA TTGTTCTAAA ATGATTTAAT TATTAAATAA TTTAAATTGA   
  
  
- ATTTTAGATT AAATTATAGT GTCGGACGAA CTATAATATT ATAGCCAACA AAGACCAAAT TTTTATATAA   
  
  
- TTTTATAATA AAAAATTAAA AAATTTTAAA TTAAAATTGT AATTATTTAA TTTTACTAGA TTAAAATTAA   
  
  
- AAAAATTTTT TTGATTCTAA GAAAAGTTTT TGTAGAAAAA TGCCACCGAA TCTGAGATAA GCAAAAATAT   
  
  
- AAAATTTTTA AGAAAATTTT TTTAATTTTT AAAAATTAAA AAAAGTGAAA TTAAAAAAAA TATAAAAATT   
  
  
- TAATAAAATT ATACAATTAT AATTTTTATT TTAAAATTTT ATTTTTTTAT AATAAAATTA TTTAAAATTT   
  
  
- TAATTTTTAT AAAATTTTTG GTTGGTTTTT TCCGTTGGAT AATGCTTGCT TGATCCTCTG TACTACTAGC   
  
  
- GACCTTCTTC TCGTTGTTCT TCGTTACTGT AGTGACTTTC TTATTGCCAA TCAGGCCACT TAAAAGTAAT   
  
  
- CGACCTTCAA TGACGATCAT CAATTAATCA ATCTTCTCGA CAACTCAATC AATCATCAAT CATCAATTAA   
  
  
- TCAATCTTTT CGAGAACTCA ACCAATCATC AATCGTCAAC TTATTTCCCC AATCTAGTTA ACATTAATCC   
  
  
- TTTTTTGGTA ACATATATTT GTCCACATAC TTTTGCCCAA CTCCTATTCT TTTATGTCTT CTAATTAGAG   
  
  
- TAAAGAGACG AGTAAGAAGA GACGAGAGAG AACAAAGACA AGAAAGAGTT AAAGACAAGA GAAAAGAAAG   
  
  
- ATTAGAACAT TTCGACTCGT ATAGTAGTGG TTCAGTAGTG CACATCTTCG TTTCCCTGTT GTTCAGTAGT   
  
  
- GGCTTCTTCT TATATCAACT GGAAGCTCTT GGGTCAGTT

+     MYB

| Site Name | Organism | Position | Strand | Matrix score. | sequence | function |
| --- | --- | --- | --- | --- | --- | --- |
| MYB | Arabidopsis thaliana | 1649 | - | 6 | CAACAG |  |
| MYB | Arabidopsis thaliana | 1701 | - | 6 | TAACCA |  |
| MYB | Arabidopsis thaliana | 1491 | + | 6 | CAACCA |  |

>Potri.018G038100.1   
+ GTTGCTGTCT TTTTTTTTTA TCATGTTATC AAATTAATGG AAATTTAACT CTATTATCAA AATTCTCTTT   
  
  
+ TTTATAAACA TGATAATCTT TTTTATTTTA TTTTCAAGTT AAAAATACTT GGTCCGGCCT GGGACGATGC   
  
  
+ GCTTGCCAAT CTAGTCAATC CTAAATTTAC TAATACGAGT AAGTAATAGC CGAAGGAGCA AAGCTCCAGT   
  
  
+ CCGGCATCTT GTTCAGAACA ATATTACATC CCTGAAATGC CTCGTGAATT GGGTGTCTCT TCCAACTTTC   
  
  
+ TCCAGCAATT TTCTCCCAAC TTCTGCAAGC AGTGATTATC AATTTGAATT ACAGGAGCTA CCTAACATAT   
  
  
+ CCGCCCTAGA ACGAAGGCAG GAATCATCAG CTTTGCCACC TCTTTGCATA ACTGCTACTG AAAGCCATTA   
  
  
+ GCTGGTGAAT GCTCCATTTC AAACTTCCAT TGGCCCGAGT ACTGCTCCTG CTTCCATTGG AAATATCGCT   
  
  
+ GCGCAATCAG ACGAGGCAGG CATTCATATC TGATGATAAA TGTTGCAGGA TTAATTCTAT TTTCGCCGGC   
  
  
+ AGCAAATGGT GGGATTTTTT ACACTGAAAA GTGTTTCAAA AGACTCGAAT TAATGTGGTC GATTTTGGCG   
  
  
+ GGGGCGTGGA ATGGATTCCT CTCTTCCTCA ATCATCAAAC ATCCAGATAC CTGAAACAGA GAGAAGCCCA   
  
  
+ TCTTCAAAAT CATATCGTCA TCGGAGGTCC TTCGCTGCAA TCGGCTGATG ACGGTGCACT TTTTGATGGT   
  
  
+ AGCGAACCAG ATCAGTCAAT GGTCAAATCA TTTGGAAATC AACGAGCTAC AGTTTATCCC TCCTGAATCC   
  
  
+ ATCTTTGAGA ACCTAAAAGA AATTAATTAA AATTAACGAA AGAGAAAAGA GAGAGGTGGT AGAGTTCATA   
  
  
+ GTTGTCGGAT CGGTCCGGAA ATTGATTTAA TTTAGAAATT AATATAGAAA AGAGTTTTGA GTAATTATAT   
  
  
+ TATCTTTAAC GGAGGCAAAC TAAATTAATA TTTTTATTAA AAAAATAATT TTTTTAAAAA AAACTTTTTA   
  
  
+ GACTTTTGTA TAAATTAATC AAATTAAATT AACAAGATTT TACTAAATTA ATAATTTATT AAATTTAACT   
  
  
+ TAAAATCTAA TTTAATATCA CAGCCTGCTT GATATTATAA TATCGGTTGT TTCTGGTTTA AAAATATATT   
  
  
+ AAAATATTAT TTTTTAATTT TTTAAAATTT AATTTTAACA TTAATAAATT AAAATGATCT AATTTTAATT   
  
  
+ TTTTTAAAAA AACTAAGATT CTTTTCAAAA ACATCTTTTT ACGGTGGCTT AGACTCTATT CGTTTTTATA   
  
  
+ TTTTAAAAAT TCTTTTAAAA AAATTAAAAA TTTTTAATTT TTTTCACTTT AATTTTTTTT ATATTTTTAA   
  
  
+ ATTATTTTAA TATGTTAATA TTAAAAATAA AATTTTAAAA TAAAAAAATA TTATTTTAAT AAATTTTAAA   
  
  
+ ATTAAAAATA TTTTAAAAAC CAACCAAAAA AGGCAACCTA TTACGAACGA ACTAGGAGAC ATGATGATCG   
  
  
+ CTGGAAGAAG AGCAACAAGA AGCAATGACA TCACTGAAAG AATAACGGTT AGTCCGGTGA ATTTTCATTA   
  
  
+ GCTGGAAGTT ACTGCTAGTA GTTAATTAGT TAGAAGAGCT GTTGAGTTAG TTAGTAGTTA GTAGTTAATT   
  
  
+ AGTTAGAAAA GCTCTTGAGT TGGTTAGTAG TTAGCAGTTG AATAAAGGGG TTAGATCAAT TGTAATTAGG   
  
  
+ AAAAAACCAT TGTATATAAA CAGGTGTATG AAAACGGGTT GAGGATAAGA AAATACAGAA GATTAATCTC   
  
  
+ ATTTCTCTGC TCATTCTTCT CTGCTCTCTC TTGTTTCTGT TCTTTCTCAA TTTCTGTTCT CTTTTCTTTC   
  
  
+ TAATCTTGTA AAGCTGAGCA TATCATCACC AAGTCATCAC GTGTAGAAGC AAAGGGACAA CAAGTCATCA   
  
  
+ CCGAAGAAGA ATATAGTTGA CCTTCGAGAA CCCAGTCAA  

- CAACGACAGA AAAAAAAAAT AGTACAATAG TTTAATTACC TTTAAATTGA GATAATAGTT TTAAGAGAAA   
  
  
- AAATATTTGT ACTATTAGAA AAAATAAAAT AAAAGTTCAA TTTTTATGAA CCAGGCCGGA CCCTGCTACG   
  
  
- CGAACGGTTA GATCAGTTAG GATTTAAATG ATTATGCTCA TTCATTATCG GCTTCCTCGT TTCGAGGTCA   
  
  
- GGCCGTAGAA CAAGTCTTGT TATAATGTAG GGACTTTACG GAGCACTTAA CCCACAGAGA AGGTTGAAAG   
  
  
- AGGTCGTTAA AAGAGGGTTG AAGACGTTCG TCACTAATAG TTAAACTTAA TGTCCTCGAT GGATTGTATA   
  
  
- GGCGGGATCT TGCTTCCGTC CTTAGTAGTC GAAACGGTGG AGAAACGTAT TGACGATGAC TTTCGGTAAT   
  
  
- CGACCACTTA CGAGGTAAAG TTTGAAGGTA ACCGGGCTCA TGACGAGGAC GAAGGTAACC TTTATAGCGA   
  
  
- CGCGTTAGTC TGCTCCGTCC GTAAGTATAG ACTACTATTT ACAACGTCCT AATTAAGATA AAAGCGGCCG   
  
  
- TCGTTTACCA CCCTAAAAAA TGTGACTTTT CACAAAGTTT TCTGAGCTTA ATTACACCAG CTAAAACCGC   
  
  
- CCCCGCACCT TACCTAAGGA GAGAAGGAGT TAGTAGTTTG TAGGTCTATG GACTTTGTCT CTCTTCGGGT   
  
  
- AGAAGTTTTA GTATAGCAGT AGCCTCCAGG AAGCGACGTT AGCCGACTAC TGCCACGTGA AAAACTACCA   
  
  
- TCGCTTGGTC TAGTCAGTTA CCAGTTTAGT AAACCTTTAG TTGCTCGATG TCAAATAGGG AGGACTTAGG   
  
  
- TAGAAACTCT TGGATTTTCT TTAATTAATT TTAATTGCTT TCTCTTTTCT CTCTCCACCA TCTCAAGTAT   
  
  
- CAACAGCCTA GCCAGGCCTT TAACTAAATT AAATCTTTAA TTATATCTTT TCTCAAAACT CATTAATATA   
  
  
- ATAGAAATTG CCTCCGTTTG ATTTAATTAT AAAAATAATT TTTTTATTAA AAAAATTTTT TTTGAAAAAT   
  
  
- CTGAAAACAT ATTTAATTAG TTTAATTTAA TTGTTCTAAA ATGATTTAAT TATTAAATAA TTTAAATTGA   
  
  
- ATTTTAGATT AAATTATAGT GTCGGACGAA CTATAATATT ATAGCCAACA AAGACCAAAT TTTTATATAA   
  
  
- TTTTATAATA AAAAATTAAA AAATTTTAAA TTAAAATTGT AATTATTTAA TTTTACTAGA TTAAAATTAA   
  
  
- AAAAATTTTT TTGATTCTAA GAAAAGTTTT TGTAGAAAAA TGCCACCGAA TCTGAGATAA GCAAAAATAT   
  
  
- AAAATTTTTA AGAAAATTTT TTTAATTTTT AAAAATTAAA AAAAGTGAAA TTAAAAAAAA TATAAAAATT   
  
  
- TAATAAAATT ATACAATTAT AATTTTTATT TTAAAATTTT ATTTTTTTAT AATAAAATTA TTTAAAATTT   
  
  
- TAATTTTTAT AAAATTTTTG GTTGGTTTTT TCCGTTGGAT AATGCTTGCT TGATCCTCTG TACTACTAGC   
  
  
- GACCTTCTTC TCGTTGTTCT TCGTTACTGT AGTGACTTTC TTATTGCCAA TCAGGCCACT TAAAAGTAAT   
  
  
- CGACCTTCAA TGACGATCAT CAATTAATCA ATCTTCTCGA CAACTCAATC AATCATCAAT CATCAATTAA   
  
  
- TCAATCTTTT CGAGAACTCA ACCAATCATC AATCGTCAAC TTATTTCCCC AATCTAGTTA ACATTAATCC   
  
  
- TTTTTTGGTA ACATATATTT GTCCACATAC TTTTGCCCAA CTCCTATTCT TTTATGTCTT CTAATTAGAG   
  
  
- TAAAGAGACG AGTAAGAAGA GACGAGAGAG AACAAAGACA AGAAAGAGTT AAAGACAAGA GAAAAGAAAG   
  
  
- ATTAGAACAT TTCGACTCGT ATAGTAGTGG TTCAGTAGTG CACATCTTCG TTTCCCTGTT GTTCAGTAGT   
  
  
- GGCTTCTTCT TATATCAACT GGAAGCTCTT GGGTCAGTT

+     MYB-like sequence

| Site Name | Organism | Position | Strand | Matrix score. | sequence | function |
| --- | --- | --- | --- | --- | --- | --- |
| MYB-like sequence | Arabidopsis thaliana | 1701 | - | 6 | TAACCA |  |

>Potri.018G038100.1   
+ GTTGCTGTCT TTTTTTTTTA TCATGTTATC AAATTAATGG AAATTTAACT CTATTATCAA AATTCTCTTT   
  
  
+ TTTATAAACA TGATAATCTT TTTTATTTTA TTTTCAAGTT AAAAATACTT GGTCCGGCCT GGGACGATGC   
  
  
+ GCTTGCCAAT CTAGTCAATC CTAAATTTAC TAATACGAGT AAGTAATAGC CGAAGGAGCA AAGCTCCAGT   
  
  
+ CCGGCATCTT GTTCAGAACA ATATTACATC CCTGAAATGC CTCGTGAATT GGGTGTCTCT TCCAACTTTC   
  
  
+ TCCAGCAATT TTCTCCCAAC TTCTGCAAGC AGTGATTATC AATTTGAATT ACAGGAGCTA CCTAACATAT   
  
  
+ CCGCCCTAGA ACGAAGGCAG GAATCATCAG CTTTGCCACC TCTTTGCATA ACTGCTACTG AAAGCCATTA   
  
  
+ GCTGGTGAAT GCTCCATTTC AAACTTCCAT TGGCCCGAGT ACTGCTCCTG CTTCCATTGG AAATATCGCT   
  
  
+ GCGCAATCAG ACGAGGCAGG CATTCATATC TGATGATAAA TGTTGCAGGA TTAATTCTAT TTTCGCCGGC   
  
  
+ AGCAAATGGT GGGATTTTTT ACACTGAAAA GTGTTTCAAA AGACTCGAAT TAATGTGGTC GATTTTGGCG   
  
  
+ GGGGCGTGGA ATGGATTCCT CTCTTCCTCA ATCATCAAAC ATCCAGATAC CTGAAACAGA GAGAAGCCCA   
  
  
+ TCTTCAAAAT CATATCGTCA TCGGAGGTCC TTCGCTGCAA TCGGCTGATG ACGGTGCACT TTTTGATGGT   
  
  
+ AGCGAACCAG ATCAGTCAAT GGTCAAATCA TTTGGAAATC AACGAGCTAC AGTTTATCCC TCCTGAATCC   
  
  
+ ATCTTTGAGA ACCTAAAAGA AATTAATTAA AATTAACGAA AGAGAAAAGA GAGAGGTGGT AGAGTTCATA   
  
  
+ GTTGTCGGAT CGGTCCGGAA ATTGATTTAA TTTAGAAATT AATATAGAAA AGAGTTTTGA GTAATTATAT   
  
  
+ TATCTTTAAC GGAGGCAAAC TAAATTAATA TTTTTATTAA AAAAATAATT TTTTTAAAAA AAACTTTTTA   
  
  
+ GACTTTTGTA TAAATTAATC AAATTAAATT AACAAGATTT TACTAAATTA ATAATTTATT AAATTTAACT   
  
  
+ TAAAATCTAA TTTAATATCA CAGCCTGCTT GATATTATAA TATCGGTTGT TTCTGGTTTA AAAATATATT   
  
  
+ AAAATATTAT TTTTTAATTT TTTAAAATTT AATTTTAACA TTAATAAATT AAAATGATCT AATTTTAATT   
  
  
+ TTTTTAAAAA AACTAAGATT CTTTTCAAAA ACATCTTTTT ACGGTGGCTT AGACTCTATT CGTTTTTATA   
  
  
+ TTTTAAAAAT TCTTTTAAAA AAATTAAAAA TTTTTAATTT TTTTCACTTT AATTTTTTTT ATATTTTTAA   
  
  
+ ATTATTTTAA TATGTTAATA TTAAAAATAA AATTTTAAAA TAAAAAAATA TTATTTTAAT AAATTTTAAA   
  
  
+ ATTAAAAATA TTTTAAAAAC CAACCAAAAA AGGCAACCTA TTACGAACGA ACTAGGAGAC ATGATGATCG   
  
  
+ CTGGAAGAAG AGCAACAAGA AGCAATGACA TCACTGAAAG AATAACGGTT AGTCCGGTGA ATTTTCATTA   
  
  
+ GCTGGAAGTT ACTGCTAGTA GTTAATTAGT TAGAAGAGCT GTTGAGTTAG TTAGTAGTTA GTAGTTAATT   
  
  
+ AGTTAGAAAA GCTCTTGAGT TGGTTAGTAG TTAGCAGTTG AATAAAGGGG TTAGATCAAT TGTAATTAGG   
  
  
+ AAAAAACCAT TGTATATAAA CAGGTGTATG AAAACGGGTT GAGGATAAGA AAATACAGAA GATTAATCTC   
  
  
+ ATTTCTCTGC TCATTCTTCT CTGCTCTCTC TTGTTTCTGT TCTTTCTCAA TTTCTGTTCT CTTTTCTTTC   
  
  
+ TAATCTTGTA AAGCTGAGCA TATCATCACC AAGTCATCAC GTGTAGAAGC AAAGGGACAA CAAGTCATCA   
  
  
+ CCGAAGAAGA ATATAGTTGA CCTTCGAGAA CCCAGTCAA  

- CAACGACAGA AAAAAAAAAT AGTACAATAG TTTAATTACC TTTAAATTGA GATAATAGTT TTAAGAGAAA   
  
  
- AAATATTTGT ACTATTAGAA AAAATAAAAT AAAAGTTCAA TTTTTATGAA CCAGGCCGGA CCCTGCTACG   
  
  
- CGAACGGTTA GATCAGTTAG GATTTAAATG ATTATGCTCA TTCATTATCG GCTTCCTCGT TTCGAGGTCA   
  
  
- GGCCGTAGAA CAAGTCTTGT TATAATGTAG GGACTTTACG GAGCACTTAA CCCACAGAGA AGGTTGAAAG   
  
  
- AGGTCGTTAA AAGAGGGTTG AAGACGTTCG TCACTAATAG TTAAACTTAA TGTCCTCGAT GGATTGTATA   
  
  
- GGCGGGATCT TGCTTCCGTC CTTAGTAGTC GAAACGGTGG AGAAACGTAT TGACGATGAC TTTCGGTAAT   
  
  
- CGACCACTTA CGAGGTAAAG TTTGAAGGTA ACCGGGCTCA TGACGAGGAC GAAGGTAACC TTTATAGCGA   
  
  
- CGCGTTAGTC TGCTCCGTCC GTAAGTATAG ACTACTATTT ACAACGTCCT AATTAAGATA AAAGCGGCCG   
  
  
- TCGTTTACCA CCCTAAAAAA TGTGACTTTT CACAAAGTTT TCTGAGCTTA ATTACACCAG CTAAAACCGC   
  
  
- CCCCGCACCT TACCTAAGGA GAGAAGGAGT TAGTAGTTTG TAGGTCTATG GACTTTGTCT CTCTTCGGGT   
  
  
- AGAAGTTTTA GTATAGCAGT AGCCTCCAGG AAGCGACGTT AGCCGACTAC TGCCACGTGA AAAACTACCA   
  
  
- TCGCTTGGTC TAGTCAGTTA CCAGTTTAGT AAACCTTTAG TTGCTCGATG TCAAATAGGG AGGACTTAGG   
  
  
- TAGAAACTCT TGGATTTTCT TTAATTAATT TTAATTGCTT TCTCTTTTCT CTCTCCACCA TCTCAAGTAT   
  
  
- CAACAGCCTA GCCAGGCCTT TAACTAAATT AAATCTTTAA TTATATCTTT TCTCAAAACT CATTAATATA   
  
  
- ATAGAAATTG CCTCCGTTTG ATTTAATTAT AAAAATAATT TTTTTATTAA AAAAATTTTT TTTGAAAAAT   
  
  
- CTGAAAACAT ATTTAATTAG TTTAATTTAA TTGTTCTAAA ATGATTTAAT TATTAAATAA TTTAAATTGA   
  
  
- ATTTTAGATT AAATTATAGT GTCGGACGAA CTATAATATT ATAGCCAACA AAGACCAAAT TTTTATATAA   
  
  
- TTTTATAATA AAAAATTAAA AAATTTTAAA TTAAAATTGT AATTATTTAA TTTTACTAGA TTAAAATTAA   
  
  
- AAAAATTTTT TTGATTCTAA GAAAAGTTTT TGTAGAAAAA TGCCACCGAA TCTGAGATAA GCAAAAATAT   
  
  
- AAAATTTTTA AGAAAATTTT TTTAATTTTT AAAAATTAAA AAAAGTGAAA TTAAAAAAAA TATAAAAATT   
  
  
- TAATAAAATT ATACAATTAT AATTTTTATT TTAAAATTTT ATTTTTTTAT AATAAAATTA TTTAAAATTT   
  
  
- TAATTTTTAT AAAATTTTTG GTTGGTTTTT TCCGTTGGAT AATGCTTGCT TGATCCTCTG TACTACTAGC   
  
  
- GACCTTCTTC TCGTTGTTCT TCGTTACTGT AGTGACTTTC TTATTGCCAA TCAGGCCACT TAAAAGTAAT   
  
  
- CGACCTTCAA TGACGATCAT CAATTAATCA ATCTTCTCGA CAACTCAATC AATCATCAAT CATCAATTAA   
  
  
- TCAATCTTTT CGAGAACTCA ACCAATCATC AATCGTCAAC TTATTTCCCC AATCTAGTTA ACATTAATCC   
  
  
- TTTTTTGGTA ACATATATTT GTCCACATAC TTTTGCCCAA CTCCTATTCT TTTATGTCTT CTAATTAGAG   
  
  
- TAAAGAGACG AGTAAGAAGA GACGAGAGAG AACAAAGACA AGAAAGAGTT AAAGACAAGA GAAAAGAAAG   
  
  
- ATTAGAACAT TTCGACTCGT ATAGTAGTGG TTCAGTAGTG CACATCTTCG TTTCCCTGTT GTTCAGTAGT   
  
  
- GGCTTCTTCT TATATCAACT GGAAGCTCTT GGGTCAGTT

+     MYC

| Site Name | Organism | Position | Strand | Matrix score. | sequence | function |
| --- | --- | --- | --- | --- | --- | --- |
| MYC | Arabidopsis thaliana | 1737 | - | 6 | CAATTG |  |
| MYC | Arabidopsis thaliana | 799 | + | 6 | CATTTG |  |
| MYC | Arabidopsis thaliana | 563 | - | 6 | CATTTG |  |

>Potri.018G038100.1   
+ GTTGCTGTCT TTTTTTTTTA TCATGTTATC AAATTAATGG AAATTTAACT CTATTATCAA AATTCTCTTT   
  
  
+ TTTATAAACA TGATAATCTT TTTTATTTTA TTTTCAAGTT AAAAATACTT GGTCCGGCCT GGGACGATGC   
  
  
+ GCTTGCCAAT CTAGTCAATC CTAAATTTAC TAATACGAGT AAGTAATAGC CGAAGGAGCA AAGCTCCAGT   
  
  
+ CCGGCATCTT GTTCAGAACA ATATTACATC CCTGAAATGC CTCGTGAATT GGGTGTCTCT TCCAACTTTC   
  
  
+ TCCAGCAATT TTCTCCCAAC TTCTGCAAGC AGTGATTATC AATTTGAATT ACAGGAGCTA CCTAACATAT   
  
  
+ CCGCCCTAGA ACGAAGGCAG GAATCATCAG CTTTGCCACC TCTTTGCATA ACTGCTACTG AAAGCCATTA   
  
  
+ GCTGGTGAAT GCTCCATTTC AAACTTCCAT TGGCCCGAGT ACTGCTCCTG CTTCCATTGG AAATATCGCT   
  
  
+ GCGCAATCAG ACGAGGCAGG CATTCATATC TGATGATAAA TGTTGCAGGA TTAATTCTAT TTTCGCCGGC   
  
  
+ AGCAAATGGT GGGATTTTTT ACACTGAAAA GTGTTTCAAA AGACTCGAAT TAATGTGGTC GATTTTGGCG   
  
  
+ GGGGCGTGGA ATGGATTCCT CTCTTCCTCA ATCATCAAAC ATCCAGATAC CTGAAACAGA GAGAAGCCCA   
  
  
+ TCTTCAAAAT CATATCGTCA TCGGAGGTCC TTCGCTGCAA TCGGCTGATG ACGGTGCACT TTTTGATGGT   
  
  
+ AGCGAACCAG ATCAGTCAAT GGTCAAATCA TTTGGAAATC AACGAGCTAC AGTTTATCCC TCCTGAATCC   
  
  
+ ATCTTTGAGA ACCTAAAAGA AATTAATTAA AATTAACGAA AGAGAAAAGA GAGAGGTGGT AGAGTTCATA   
  
  
+ GTTGTCGGAT CGGTCCGGAA ATTGATTTAA TTTAGAAATT AATATAGAAA AGAGTTTTGA GTAATTATAT   
  
  
+ TATCTTTAAC GGAGGCAAAC TAAATTAATA TTTTTATTAA AAAAATAATT TTTTTAAAAA AAACTTTTTA   
  
  
+ GACTTTTGTA TAAATTAATC AAATTAAATT AACAAGATTT TACTAAATTA ATAATTTATT AAATTTAACT   
  
  
+ TAAAATCTAA TTTAATATCA CAGCCTGCTT GATATTATAA TATCGGTTGT TTCTGGTTTA AAAATATATT   
  
  
+ AAAATATTAT TTTTTAATTT TTTAAAATTT AATTTTAACA TTAATAAATT AAAATGATCT AATTTTAATT   
  
  
+ TTTTTAAAAA AACTAAGATT CTTTTCAAAA ACATCTTTTT ACGGTGGCTT AGACTCTATT CGTTTTTATA   
  
  
+ TTTTAAAAAT TCTTTTAAAA AAATTAAAAA TTTTTAATTT TTTTCACTTT AATTTTTTTT ATATTTTTAA   
  
  
+ ATTATTTTAA TATGTTAATA TTAAAAATAA AATTTTAAAA TAAAAAAATA TTATTTTAAT AAATTTTAAA   
  
  
+ ATTAAAAATA TTTTAAAAAC CAACCAAAAA AGGCAACCTA TTACGAACGA ACTAGGAGAC ATGATGATCG   
  
  
+ CTGGAAGAAG AGCAACAAGA AGCAATGACA TCACTGAAAG AATAACGGTT AGTCCGGTGA ATTTTCATTA   
  
  
+ GCTGGAAGTT ACTGCTAGTA GTTAATTAGT TAGAAGAGCT GTTGAGTTAG TTAGTAGTTA GTAGTTAATT   
  
  
+ AGTTAGAAAA GCTCTTGAGT TGGTTAGTAG TTAGCAGTTG AATAAAGGGG TTAGATCAAT TGTAATTAGG   
  
  
+ AAAAAACCAT TGTATATAAA CAGGTGTATG AAAACGGGTT GAGGATAAGA AAATACAGAA GATTAATCTC   
  
  
+ ATTTCTCTGC TCATTCTTCT CTGCTCTCTC TTGTTTCTGT TCTTTCTCAA TTTCTGTTCT CTTTTCTTTC   
  
  
+ TAATCTTGTA AAGCTGAGCA TATCATCACC AAGTCATCAC GTGTAGAAGC AAAGGGACAA CAAGTCATCA   
  
  
+ CCGAAGAAGA ATATAGTTGA CCTTCGAGAA CCCAGTCAA  

- CAACGACAGA AAAAAAAAAT AGTACAATAG TTTAATTACC TTTAAATTGA GATAATAGTT TTAAGAGAAA   
  
  
- AAATATTTGT ACTATTAGAA AAAATAAAAT AAAAGTTCAA TTTTTATGAA CCAGGCCGGA CCCTGCTACG   
  
  
- CGAACGGTTA GATCAGTTAG GATTTAAATG ATTATGCTCA TTCATTATCG GCTTCCTCGT TTCGAGGTCA   
  
  
- GGCCGTAGAA CAAGTCTTGT TATAATGTAG GGACTTTACG GAGCACTTAA CCCACAGAGA AGGTTGAAAG   
  
  
- AGGTCGTTAA AAGAGGGTTG AAGACGTTCG TCACTAATAG TTAAACTTAA TGTCCTCGAT GGATTGTATA   
  
  
- GGCGGGATCT TGCTTCCGTC CTTAGTAGTC GAAACGGTGG AGAAACGTAT TGACGATGAC TTTCGGTAAT   
  
  
- CGACCACTTA CGAGGTAAAG TTTGAAGGTA ACCGGGCTCA TGACGAGGAC GAAGGTAACC TTTATAGCGA   
  
  
- CGCGTTAGTC TGCTCCGTCC GTAAGTATAG ACTACTATTT ACAACGTCCT AATTAAGATA AAAGCGGCCG   
  
  
- TCGTTTACCA CCCTAAAAAA TGTGACTTTT CACAAAGTTT TCTGAGCTTA ATTACACCAG CTAAAACCGC   
  
  
- CCCCGCACCT TACCTAAGGA GAGAAGGAGT TAGTAGTTTG TAGGTCTATG GACTTTGTCT CTCTTCGGGT   
  
  
- AGAAGTTTTA GTATAGCAGT AGCCTCCAGG AAGCGACGTT AGCCGACTAC TGCCACGTGA AAAACTACCA   
  
  
- TCGCTTGGTC TAGTCAGTTA CCAGTTTAGT AAACCTTTAG TTGCTCGATG TCAAATAGGG AGGACTTAGG   
  
  
- TAGAAACTCT TGGATTTTCT TTAATTAATT TTAATTGCTT TCTCTTTTCT CTCTCCACCA TCTCAAGTAT   
  
  
- CAACAGCCTA GCCAGGCCTT TAACTAAATT AAATCTTTAA TTATATCTTT TCTCAAAACT CATTAATATA   
  
  
- ATAGAAATTG CCTCCGTTTG ATTTAATTAT AAAAATAATT TTTTTATTAA AAAAATTTTT TTTGAAAAAT   
  
  
- CTGAAAACAT ATTTAATTAG TTTAATTTAA TTGTTCTAAA ATGATTTAAT TATTAAATAA TTTAAATTGA   
  
  
- ATTTTAGATT AAATTATAGT GTCGGACGAA CTATAATATT ATAGCCAACA AAGACCAAAT TTTTATATAA   
  
  
- TTTTATAATA AAAAATTAAA AAATTTTAAA TTAAAATTGT AATTATTTAA TTTTACTAGA TTAAAATTAA   
  
  
- AAAAATTTTT TTGATTCTAA GAAAAGTTTT TGTAGAAAAA TGCCACCGAA TCTGAGATAA GCAAAAATAT   
  
  
- AAAATTTTTA AGAAAATTTT TTTAATTTTT AAAAATTAAA AAAAGTGAAA TTAAAAAAAA TATAAAAATT   
  
  
- TAATAAAATT ATACAATTAT AATTTTTATT TTAAAATTTT ATTTTTTTAT AATAAAATTA TTTAAAATTT   
  
  
- TAATTTTTAT AAAATTTTTG GTTGGTTTTT TCCGTTGGAT AATGCTTGCT TGATCCTCTG TACTACTAGC   
  
  
- GACCTTCTTC TCGTTGTTCT TCGTTACTGT AGTGACTTTC TTATTGCCAA TCAGGCCACT TAAAAGTAAT   
  
  
- CGACCTTCAA TGACGATCAT CAATTAATCA ATCTTCTCGA CAACTCAATC AATCATCAAT CATCAATTAA   
  
  
- TCAATCTTTT CGAGAACTCA ACCAATCATC AATCGTCAAC TTATTTCCCC AATCTAGTTA ACATTAATCC   
  
  
- TTTTTTGGTA ACATATATTT GTCCACATAC TTTTGCCCAA CTCCTATTCT TTTATGTCTT CTAATTAGAG   
  
  
- TAAAGAGACG AGTAAGAAGA GACGAGAGAG AACAAAGACA AGAAAGAGTT AAAGACAAGA GAAAAGAAAG   
  
  
- ATTAGAACAT TTCGACTCGT ATAGTAGTGG TTCAGTAGTG CACATCTTCG TTTCCCTGTT GTTCAGTAGT   
  
  
- GGCTTCTTCT TATATCAACT GGAAGCTCTT GGGTCAGTT

+     Myb

| Site Name | Organism | Position | Strand | Matrix score. | sequence | function |
| --- | --- | --- | --- | --- | --- | --- |
| Myb | Arabidopsis thaliana | 399 | + | 6 | TAACTG |  |
| Myb | Arabidopsis thaliana | 1715 | - | 6 | CAACTG |  |

>Potri.018G038100.1   
+ GTTGCTGTCT TTTTTTTTTA TCATGTTATC AAATTAATGG AAATTTAACT CTATTATCAA AATTCTCTTT   
  
  
+ TTTATAAACA TGATAATCTT TTTTATTTTA TTTTCAAGTT AAAAATACTT GGTCCGGCCT GGGACGATGC   
  
  
+ GCTTGCCAAT CTAGTCAATC CTAAATTTAC TAATACGAGT AAGTAATAGC CGAAGGAGCA AAGCTCCAGT   
  
  
+ CCGGCATCTT GTTCAGAACA ATATTACATC CCTGAAATGC CTCGTGAATT GGGTGTCTCT TCCAACTTTC   
  
  
+ TCCAGCAATT TTCTCCCAAC TTCTGCAAGC AGTGATTATC AATTTGAATT ACAGGAGCTA CCTAACATAT   
  
  
+ CCGCCCTAGA ACGAAGGCAG GAATCATCAG CTTTGCCACC TCTTTGCATA ACTGCTACTG AAAGCCATTA   
  
  
+ GCTGGTGAAT GCTCCATTTC AAACTTCCAT TGGCCCGAGT ACTGCTCCTG CTTCCATTGG AAATATCGCT   
  
  
+ GCGCAATCAG ACGAGGCAGG CATTCATATC TGATGATAAA TGTTGCAGGA TTAATTCTAT TTTCGCCGGC   
  
  
+ AGCAAATGGT GGGATTTTTT ACACTGAAAA GTGTTTCAAA AGACTCGAAT TAATGTGGTC GATTTTGGCG   
  
  
+ GGGGCGTGGA ATGGATTCCT CTCTTCCTCA ATCATCAAAC ATCCAGATAC CTGAAACAGA GAGAAGCCCA   
  
  
+ TCTTCAAAAT CATATCGTCA TCGGAGGTCC TTCGCTGCAA TCGGCTGATG ACGGTGCACT TTTTGATGGT   
  
  
+ AGCGAACCAG ATCAGTCAAT GGTCAAATCA TTTGGAAATC AACGAGCTAC AGTTTATCCC TCCTGAATCC   
  
  
+ ATCTTTGAGA ACCTAAAAGA AATTAATTAA AATTAACGAA AGAGAAAAGA GAGAGGTGGT AGAGTTCATA   
  
  
+ GTTGTCGGAT CGGTCCGGAA ATTGATTTAA TTTAGAAATT AATATAGAAA AGAGTTTTGA GTAATTATAT   
  
  
+ TATCTTTAAC GGAGGCAAAC TAAATTAATA TTTTTATTAA AAAAATAATT TTTTTAAAAA AAACTTTTTA   
  
  
+ GACTTTTGTA TAAATTAATC AAATTAAATT AACAAGATTT TACTAAATTA ATAATTTATT AAATTTAACT   
  
  
+ TAAAATCTAA TTTAATATCA CAGCCTGCTT GATATTATAA TATCGGTTGT TTCTGGTTTA AAAATATATT   
  
  
+ AAAATATTAT TTTTTAATTT TTTAAAATTT AATTTTAACA TTAATAAATT AAAATGATCT AATTTTAATT   
  
  
+ TTTTTAAAAA AACTAAGATT CTTTTCAAAA ACATCTTTTT ACGGTGGCTT AGACTCTATT CGTTTTTATA   
  
  
+ TTTTAAAAAT TCTTTTAAAA AAATTAAAAA TTTTTAATTT TTTTCACTTT AATTTTTTTT ATATTTTTAA   
  
  
+ ATTATTTTAA TATGTTAATA TTAAAAATAA AATTTTAAAA TAAAAAAATA TTATTTTAAT AAATTTTAAA   
  
  
+ ATTAAAAATA TTTTAAAAAC CAACCAAAAA AGGCAACCTA TTACGAACGA ACTAGGAGAC ATGATGATCG   
  
  
+ CTGGAAGAAG AGCAACAAGA AGCAATGACA TCACTGAAAG AATAACGGTT AGTCCGGTGA ATTTTCATTA   
  
  
+ GCTGGAAGTT ACTGCTAGTA GTTAATTAGT TAGAAGAGCT GTTGAGTTAG TTAGTAGTTA GTAGTTAATT   
  
  
+ AGTTAGAAAA GCTCTTGAGT TGGTTAGTAG TTAGCAGTTG AATAAAGGGG TTAGATCAAT TGTAATTAGG   
  
  
+ AAAAAACCAT TGTATATAAA CAGGTGTATG AAAACGGGTT GAGGATAAGA AAATACAGAA GATTAATCTC   
  
  
+ ATTTCTCTGC TCATTCTTCT CTGCTCTCTC TTGTTTCTGT TCTTTCTCAA TTTCTGTTCT CTTTTCTTTC   
  
  
+ TAATCTTGTA AAGCTGAGCA TATCATCACC AAGTCATCAC GTGTAGAAGC AAAGGGACAA CAAGTCATCA   
  
  
+ CCGAAGAAGA ATATAGTTGA CCTTCGAGAA CCCAGTCAA  

- CAACGACAGA AAAAAAAAAT AGTACAATAG TTTAATTACC TTTAAATTGA GATAATAGTT TTAAGAGAAA   
  
  
- AAATATTTGT ACTATTAGAA AAAATAAAAT AAAAGTTCAA TTTTTATGAA CCAGGCCGGA CCCTGCTACG   
  
  
- CGAACGGTTA GATCAGTTAG GATTTAAATG ATTATGCTCA TTCATTATCG GCTTCCTCGT TTCGAGGTCA   
  
  
- GGCCGTAGAA CAAGTCTTGT TATAATGTAG GGACTTTACG GAGCACTTAA CCCACAGAGA AGGTTGAAAG   
  
  
- AGGTCGTTAA AAGAGGGTTG AAGACGTTCG TCACTAATAG TTAAACTTAA TGTCCTCGAT GGATTGTATA   
  
  
- GGCGGGATCT TGCTTCCGTC CTTAGTAGTC GAAACGGTGG AGAAACGTAT TGACGATGAC TTTCGGTAAT   
  
  
- CGACCACTTA CGAGGTAAAG TTTGAAGGTA ACCGGGCTCA TGACGAGGAC GAAGGTAACC TTTATAGCGA   
  
  
- CGCGTTAGTC TGCTCCGTCC GTAAGTATAG ACTACTATTT ACAACGTCCT AATTAAGATA AAAGCGGCCG   
  
  
- TCGTTTACCA CCCTAAAAAA TGTGACTTTT CACAAAGTTT TCTGAGCTTA ATTACACCAG CTAAAACCGC   
  
  
- CCCCGCACCT TACCTAAGGA GAGAAGGAGT TAGTAGTTTG TAGGTCTATG GACTTTGTCT CTCTTCGGGT   
  
  
- AGAAGTTTTA GTATAGCAGT AGCCTCCAGG AAGCGACGTT AGCCGACTAC TGCCACGTGA AAAACTACCA   
  
  
- TCGCTTGGTC TAGTCAGTTA CCAGTTTAGT AAACCTTTAG TTGCTCGATG TCAAATAGGG AGGACTTAGG   
  
  
- TAGAAACTCT TGGATTTTCT TTAATTAATT TTAATTGCTT TCTCTTTTCT CTCTCCACCA TCTCAAGTAT   
  
  
- CAACAGCCTA GCCAGGCCTT TAACTAAATT AAATCTTTAA TTATATCTTT TCTCAAAACT CATTAATATA   
  
  
- ATAGAAATTG CCTCCGTTTG ATTTAATTAT AAAAATAATT TTTTTATTAA AAAAATTTTT TTTGAAAAAT   
  
  
- CTGAAAACAT ATTTAATTAG TTTAATTTAA TTGTTCTAAA ATGATTTAAT TATTAAATAA TTTAAATTGA   
  
  
- ATTTTAGATT AAATTATAGT GTCGGACGAA CTATAATATT ATAGCCAACA AAGACCAAAT TTTTATATAA   
  
  
- TTTTATAATA AAAAATTAAA AAATTTTAAA TTAAAATTGT AATTATTTAA TTTTACTAGA TTAAAATTAA   
  
  
- AAAAATTTTT TTGATTCTAA GAAAAGTTTT TGTAGAAAAA TGCCACCGAA TCTGAGATAA GCAAAAATAT   
  
  
- AAAATTTTTA AGAAAATTTT TTTAATTTTT AAAAATTAAA AAAAGTGAAA TTAAAAAAAA TATAAAAATT   
  
  
- TAATAAAATT ATACAATTAT AATTTTTATT TTAAAATTTT ATTTTTTTAT AATAAAATTA TTTAAAATTT   
  
  
- TAATTTTTAT AAAATTTTTG GTTGGTTTTT TCCGTTGGAT AATGCTTGCT TGATCCTCTG TACTACTAGC   
  
  
- GACCTTCTTC TCGTTGTTCT TCGTTACTGT AGTGACTTTC TTATTGCCAA TCAGGCCACT TAAAAGTAAT   
  
  
- CGACCTTCAA TGACGATCAT CAATTAATCA ATCTTCTCGA CAACTCAATC AATCATCAAT CATCAATTAA   
  
  
- TCAATCTTTT CGAGAACTCA ACCAATCATC AATCGTCAAC TTATTTCCCC AATCTAGTTA ACATTAATCC   
  
  
- TTTTTTGGTA ACATATATTT GTCCACATAC TTTTGCCCAA CTCCTATTCT TTTATGTCTT CTAATTAGAG   
  
  
- TAAAGAGACG AGTAAGAAGA GACGAGAGAG AACAAAGACA AGAAAGAGTT AAAGACAAGA GAAAAGAAAG   
  
  
- ATTAGAACAT TTCGACTCGT ATAGTAGTGG TTCAGTAGTG CACATCTTCG TTTCCCTGTT GTTCAGTAGT   
  
  
- GGCTTCTTCT TATATCAACT GGAAGCTCTT GGGTCAGTT

+     Myb-binding site

| Site Name | Organism | Position | Strand | Matrix score. | sequence | function |
| --- | --- | --- | --- | --- | --- | --- |
| Myb-binding site | Nicotiana tabacum | 1649 | - | 6 | CAACAG |  |

>Potri.018G038100.1   
+ GTTGCTGTCT TTTTTTTTTA TCATGTTATC AAATTAATGG AAATTTAACT CTATTATCAA AATTCTCTTT   
  
  
+ TTTATAAACA TGATAATCTT TTTTATTTTA TTTTCAAGTT AAAAATACTT GGTCCGGCCT GGGACGATGC   
  
  
+ GCTTGCCAAT CTAGTCAATC CTAAATTTAC TAATACGAGT AAGTAATAGC CGAAGGAGCA AAGCTCCAGT   
  
  
+ CCGGCATCTT GTTCAGAACA ATATTACATC CCTGAAATGC CTCGTGAATT GGGTGTCTCT TCCAACTTTC   
  
  
+ TCCAGCAATT TTCTCCCAAC TTCTGCAAGC AGTGATTATC AATTTGAATT ACAGGAGCTA CCTAACATAT   
  
  
+ CCGCCCTAGA ACGAAGGCAG GAATCATCAG CTTTGCCACC TCTTTGCATA ACTGCTACTG AAAGCCATTA   
  
  
+ GCTGGTGAAT GCTCCATTTC AAACTTCCAT TGGCCCGAGT ACTGCTCCTG CTTCCATTGG AAATATCGCT   
  
  
+ GCGCAATCAG ACGAGGCAGG CATTCATATC TGATGATAAA TGTTGCAGGA TTAATTCTAT TTTCGCCGGC   
  
  
+ AGCAAATGGT GGGATTTTTT ACACTGAAAA GTGTTTCAAA AGACTCGAAT TAATGTGGTC GATTTTGGCG   
  
  
+ GGGGCGTGGA ATGGATTCCT CTCTTCCTCA ATCATCAAAC ATCCAGATAC CTGAAACAGA GAGAAGCCCA   
  
  
+ TCTTCAAAAT CATATCGTCA TCGGAGGTCC TTCGCTGCAA TCGGCTGATG ACGGTGCACT TTTTGATGGT   
  
  
+ AGCGAACCAG ATCAGTCAAT GGTCAAATCA TTTGGAAATC AACGAGCTAC AGTTTATCCC TCCTGAATCC   
  
  
+ ATCTTTGAGA ACCTAAAAGA AATTAATTAA AATTAACGAA AGAGAAAAGA GAGAGGTGGT AGAGTTCATA   
  
  
+ GTTGTCGGAT CGGTCCGGAA ATTGATTTAA TTTAGAAATT AATATAGAAA AGAGTTTTGA GTAATTATAT   
  
  
+ TATCTTTAAC GGAGGCAAAC TAAATTAATA TTTTTATTAA AAAAATAATT TTTTTAAAAA AAACTTTTTA   
  
  
+ GACTTTTGTA TAAATTAATC AAATTAAATT AACAAGATTT TACTAAATTA ATAATTTATT AAATTTAACT   
  
  
+ TAAAATCTAA TTTAATATCA CAGCCTGCTT GATATTATAA TATCGGTTGT TTCTGGTTTA AAAATATATT   
  
  
+ AAAATATTAT TTTTTAATTT TTTAAAATTT AATTTTAACA TTAATAAATT AAAATGATCT AATTTTAATT   
  
  
+ TTTTTAAAAA AACTAAGATT CTTTTCAAAA ACATCTTTTT ACGGTGGCTT AGACTCTATT CGTTTTTATA   
  
  
+ TTTTAAAAAT TCTTTTAAAA AAATTAAAAA TTTTTAATTT TTTTCACTTT AATTTTTTTT ATATTTTTAA   
  
  
+ ATTATTTTAA TATGTTAATA TTAAAAATAA AATTTTAAAA TAAAAAAATA TTATTTTAAT AAATTTTAAA   
  
  
+ ATTAAAAATA TTTTAAAAAC CAACCAAAAA AGGCAACCTA TTACGAACGA ACTAGGAGAC ATGATGATCG   
  
  
+ CTGGAAGAAG AGCAACAAGA AGCAATGACA TCACTGAAAG AATAACGGTT AGTCCGGTGA ATTTTCATTA   
  
  
+ GCTGGAAGTT ACTGCTAGTA GTTAATTAGT TAGAAGAGCT GTTGAGTTAG TTAGTAGTTA GTAGTTAATT   
  
  
+ AGTTAGAAAA GCTCTTGAGT TGGTTAGTAG TTAGCAGTTG AATAAAGGGG TTAGATCAAT TGTAATTAGG   
  
  
+ AAAAAACCAT TGTATATAAA CAGGTGTATG AAAACGGGTT GAGGATAAGA AAATACAGAA GATTAATCTC   
  
  
+ ATTTCTCTGC TCATTCTTCT CTGCTCTCTC TTGTTTCTGT TCTTTCTCAA TTTCTGTTCT CTTTTCTTTC   
  
  
+ TAATCTTGTA AAGCTGAGCA TATCATCACC AAGTCATCAC GTGTAGAAGC AAAGGGACAA CAAGTCATCA   
  
  
+ CCGAAGAAGA ATATAGTTGA CCTTCGAGAA CCCAGTCAA  

- CAACGACAGA AAAAAAAAAT AGTACAATAG TTTAATTACC TTTAAATTGA GATAATAGTT TTAAGAGAAA   
  
  
- AAATATTTGT ACTATTAGAA AAAATAAAAT AAAAGTTCAA TTTTTATGAA CCAGGCCGGA CCCTGCTACG   
  
  
- CGAACGGTTA GATCAGTTAG GATTTAAATG ATTATGCTCA TTCATTATCG GCTTCCTCGT TTCGAGGTCA   
  
  
- GGCCGTAGAA CAAGTCTTGT TATAATGTAG GGACTTTACG GAGCACTTAA CCCACAGAGA AGGTTGAAAG   
  
  
- AGGTCGTTAA AAGAGGGTTG AAGACGTTCG TCACTAATAG TTAAACTTAA TGTCCTCGAT GGATTGTATA   
  
  
- GGCGGGATCT TGCTTCCGTC CTTAGTAGTC GAAACGGTGG AGAAACGTAT TGACGATGAC TTTCGGTAAT   
  
  
- CGACCACTTA CGAGGTAAAG TTTGAAGGTA ACCGGGCTCA TGACGAGGAC GAAGGTAACC TTTATAGCGA   
  
  
- CGCGTTAGTC TGCTCCGTCC GTAAGTATAG ACTACTATTT ACAACGTCCT AATTAAGATA AAAGCGGCCG   
  
  
- TCGTTTACCA CCCTAAAAAA TGTGACTTTT CACAAAGTTT TCTGAGCTTA ATTACACCAG CTAAAACCGC   
  
  
- CCCCGCACCT TACCTAAGGA GAGAAGGAGT TAGTAGTTTG TAGGTCTATG GACTTTGTCT CTCTTCGGGT   
  
  
- AGAAGTTTTA GTATAGCAGT AGCCTCCAGG AAGCGACGTT AGCCGACTAC TGCCACGTGA AAAACTACCA   
  
  
- TCGCTTGGTC TAGTCAGTTA CCAGTTTAGT AAACCTTTAG TTGCTCGATG TCAAATAGGG AGGACTTAGG   
  
  
- TAGAAACTCT TGGATTTTCT TTAATTAATT TTAATTGCTT TCTCTTTTCT CTCTCCACCA TCTCAAGTAT   
  
  
- CAACAGCCTA GCCAGGCCTT TAACTAAATT AAATCTTTAA TTATATCTTT TCTCAAAACT CATTAATATA   
  
  
- ATAGAAATTG CCTCCGTTTG ATTTAATTAT AAAAATAATT TTTTTATTAA AAAAATTTTT TTTGAAAAAT   
  
  
- CTGAAAACAT ATTTAATTAG TTTAATTTAA TTGTTCTAAA ATGATTTAAT TATTAAATAA TTTAAATTGA   
  
  
- ATTTTAGATT AAATTATAGT GTCGGACGAA CTATAATATT ATAGCCAACA AAGACCAAAT TTTTATATAA   
  
  
- TTTTATAATA AAAAATTAAA AAATTTTAAA TTAAAATTGT AATTATTTAA TTTTACTAGA TTAAAATTAA   
  
  
- AAAAATTTTT TTGATTCTAA GAAAAGTTTT TGTAGAAAAA TGCCACCGAA TCTGAGATAA GCAAAAATAT   
  
  
- AAAATTTTTA AGAAAATTTT TTTAATTTTT AAAAATTAAA AAAAGTGAAA TTAAAAAAAA TATAAAAATT   
  
  
- TAATAAAATT ATACAATTAT AATTTTTATT TTAAAATTTT ATTTTTTTAT AATAAAATTA TTTAAAATTT   
  
  
- TAATTTTTAT AAAATTTTTG GTTGGTTTTT TCCGTTGGAT AATGCTTGCT TGATCCTCTG TACTACTAGC   
  
  
- GACCTTCTTC TCGTTGTTCT TCGTTACTGT AGTGACTTTC TTATTGCCAA TCAGGCCACT TAAAAGTAAT   
  
  
- CGACCTTCAA TGACGATCAT CAATTAATCA ATCTTCTCGA CAACTCAATC AATCATCAAT CATCAATTAA   
  
  
- TCAATCTTTT CGAGAACTCA ACCAATCATC AATCGTCAAC TTATTTCCCC AATCTAGTTA ACATTAATCC   
  
  
- TTTTTTGGTA ACATATATTT GTCCACATAC TTTTGCCCAA CTCCTATTCT TTTATGTCTT CTAATTAGAG   
  
  
- TAAAGAGACG AGTAAGAAGA GACGAGAGAG AACAAAGACA AGAAAGAGTT AAAGACAAGA GAAAAGAAAG   
  
  
- ATTAGAACAT TTCGACTCGT ATAGTAGTGG TTCAGTAGTG CACATCTTCG TTTCCCTGTT GTTCAGTAGT   
  
  
- GGCTTCTTCT TATATCAACT GGAAGCTCTT GGGTCAGTT

+     O2-site

| Site Name | Organism | Position | Strand | Matrix score. | sequence | function |
| --- | --- | --- | --- | --- | --- | --- |
| O2-site | Zea mays | 1919 | - | 9 | GATGACATGG | cis-acting regulatory element involved in zein metabolism regulation |
| O2-site | Zea mays | 1908 | - | 8 | GATGA(C/T)(A/G)TG(A/G) | cis-acting regulatory element involved in zein metabolism regulation |

>Potri.018G038100.1   
+ GTTGCTGTCT TTTTTTTTTA TCATGTTATC AAATTAATGG AAATTTAACT CTATTATCAA AATTCTCTTT   
  
  
+ TTTATAAACA TGATAATCTT TTTTATTTTA TTTTCAAGTT AAAAATACTT GGTCCGGCCT GGGACGATGC   
  
  
+ GCTTGCCAAT CTAGTCAATC CTAAATTTAC TAATACGAGT AAGTAATAGC CGAAGGAGCA AAGCTCCAGT   
  
  
+ CCGGCATCTT GTTCAGAACA ATATTACATC CCTGAAATGC CTCGTGAATT GGGTGTCTCT TCCAACTTTC   
  
  
+ TCCAGCAATT TTCTCCCAAC TTCTGCAAGC AGTGATTATC AATTTGAATT ACAGGAGCTA CCTAACATAT   
  
  
+ CCGCCCTAGA ACGAAGGCAG GAATCATCAG CTTTGCCACC TCTTTGCATA ACTGCTACTG AAAGCCATTA   
  
  
+ GCTGGTGAAT GCTCCATTTC AAACTTCCAT TGGCCCGAGT ACTGCTCCTG CTTCCATTGG AAATATCGCT   
  
  
+ GCGCAATCAG ACGAGGCAGG CATTCATATC TGATGATAAA TGTTGCAGGA TTAATTCTAT TTTCGCCGGC   
  
  
+ AGCAAATGGT GGGATTTTTT ACACTGAAAA GTGTTTCAAA AGACTCGAAT TAATGTGGTC GATTTTGGCG   
  
  
+ GGGGCGTGGA ATGGATTCCT CTCTTCCTCA ATCATCAAAC ATCCAGATAC CTGAAACAGA GAGAAGCCCA   
  
  
+ TCTTCAAAAT CATATCGTCA TCGGAGGTCC TTCGCTGCAA TCGGCTGATG ACGGTGCACT TTTTGATGGT   
  
  
+ AGCGAACCAG ATCAGTCAAT GGTCAAATCA TTTGGAAATC AACGAGCTAC AGTTTATCCC TCCTGAATCC   
  
  
+ ATCTTTGAGA ACCTAAAAGA AATTAATTAA AATTAACGAA AGAGAAAAGA GAGAGGTGGT AGAGTTCATA   
  
  
+ GTTGTCGGAT CGGTCCGGAA ATTGATTTAA TTTAGAAATT AATATAGAAA AGAGTTTTGA GTAATTATAT   
  
  
+ TATCTTTAAC GGAGGCAAAC TAAATTAATA TTTTTATTAA AAAAATAATT TTTTTAAAAA AAACTTTTTA   
  
  
+ GACTTTTGTA TAAATTAATC AAATTAAATT AACAAGATTT TACTAAATTA ATAATTTATT AAATTTAACT   
  
  
+ TAAAATCTAA TTTAATATCA CAGCCTGCTT GATATTATAA TATCGGTTGT TTCTGGTTTA AAAATATATT   
  
  
+ AAAATATTAT TTTTTAATTT TTTAAAATTT AATTTTAACA TTAATAAATT AAAATGATCT AATTTTAATT   
  
  
+ TTTTTAAAAA AACTAAGATT CTTTTCAAAA ACATCTTTTT ACGGTGGCTT AGACTCTATT CGTTTTTATA   
  
  
+ TTTTAAAAAT TCTTTTAAAA AAATTAAAAA TTTTTAATTT TTTTCACTTT AATTTTTTTT ATATTTTTAA   
  
  
+ ATTATTTTAA TATGTTAATA TTAAAAATAA AATTTTAAAA TAAAAAAATA TTATTTTAAT AAATTTTAAA   
  
  
+ ATTAAAAATA TTTTAAAAAC CAACCAAAAA AGGCAACCTA TTACGAACGA ACTAGGAGAC ATGATGATCG   
  
  
+ CTGGAAGAAG AGCAACAAGA AGCAATGACA TCACTGAAAG AATAACGGTT AGTCCGGTGA ATTTTCATTA   
  
  
+ GCTGGAAGTT ACTGCTAGTA GTTAATTAGT TAGAAGAGCT GTTGAGTTAG TTAGTAGTTA GTAGTTAATT   
  
  
+ AGTTAGAAAA GCTCTTGAGT TGGTTAGTAG TTAGCAGTTG AATAAAGGGG TTAGATCAAT TGTAATTAGG   
  
  
+ AAAAAACCAT TGTATATAAA CAGGTGTATG AAAACGGGTT GAGGATAAGA AAATACAGAA GATTAATCTC   
  
  
+ ATTTCTCTGC TCATTCTTCT CTGCTCTCTC TTGTTTCTGT TCTTTCTCAA TTTCTGTTCT CTTTTCTTTC   
  
  
+ TAATCTTGTA AAGCTGAGCA TATCATCACC AAGTCATCAC GTGTAGAAGC AAAGGGACAA CAAGTCATCA   
  
  
+ CCGAAGAAGA ATATAGTTGA CCTTCGAGAA CCCAGTCAA  

- CAACGACAGA AAAAAAAAAT AGTACAATAG TTTAATTACC TTTAAATTGA GATAATAGTT TTAAGAGAAA   
  
  
- AAATATTTGT ACTATTAGAA AAAATAAAAT AAAAGTTCAA TTTTTATGAA CCAGGCCGGA CCCTGCTACG   
  
  
- CGAACGGTTA GATCAGTTAG GATTTAAATG ATTATGCTCA TTCATTATCG GCTTCCTCGT TTCGAGGTCA   
  
  
- GGCCGTAGAA CAAGTCTTGT TATAATGTAG GGACTTTACG GAGCACTTAA CCCACAGAGA AGGTTGAAAG   
  
  
- AGGTCGTTAA AAGAGGGTTG AAGACGTTCG TCACTAATAG TTAAACTTAA TGTCCTCGAT GGATTGTATA   
  
  
- GGCGGGATCT TGCTTCCGTC CTTAGTAGTC GAAACGGTGG AGAAACGTAT TGACGATGAC TTTCGGTAAT   
  
  
- CGACCACTTA CGAGGTAAAG TTTGAAGGTA ACCGGGCTCA TGACGAGGAC GAAGGTAACC TTTATAGCGA   
  
  
- CGCGTTAGTC TGCTCCGTCC GTAAGTATAG ACTACTATTT ACAACGTCCT AATTAAGATA AAAGCGGCCG   
  
  
- TCGTTTACCA CCCTAAAAAA TGTGACTTTT CACAAAGTTT TCTGAGCTTA ATTACACCAG CTAAAACCGC   
  
  
- CCCCGCACCT TACCTAAGGA GAGAAGGAGT TAGTAGTTTG TAGGTCTATG GACTTTGTCT CTCTTCGGGT   
  
  
- AGAAGTTTTA GTATAGCAGT AGCCTCCAGG AAGCGACGTT AGCCGACTAC TGCCACGTGA AAAACTACCA   
  
  
- TCGCTTGGTC TAGTCAGTTA CCAGTTTAGT AAACCTTTAG TTGCTCGATG TCAAATAGGG AGGACTTAGG   
  
  
- TAGAAACTCT TGGATTTTCT TTAATTAATT TTAATTGCTT TCTCTTTTCT CTCTCCACCA TCTCAAGTAT   
  
  
- CAACAGCCTA GCCAGGCCTT TAACTAAATT AAATCTTTAA TTATATCTTT TCTCAAAACT CATTAATATA   
  
  
- ATAGAAATTG CCTCCGTTTG ATTTAATTAT AAAAATAATT TTTTTATTAA AAAAATTTTT TTTGAAAAAT   
  
  
- CTGAAAACAT ATTTAATTAG TTTAATTTAA TTGTTCTAAA ATGATTTAAT TATTAAATAA TTTAAATTGA   
  
  
- ATTTTAGATT AAATTATAGT GTCGGACGAA CTATAATATT ATAGCCAACA AAGACCAAAT TTTTATATAA   
  
  
- TTTTATAATA AAAAATTAAA AAATTTTAAA TTAAAATTGT AATTATTTAA TTTTACTAGA TTAAAATTAA   
  
  
- AAAAATTTTT TTGATTCTAA GAAAAGTTTT TGTAGAAAAA TGCCACCGAA TCTGAGATAA GCAAAAATAT   
  
  
- AAAATTTTTA AGAAAATTTT TTTAATTTTT AAAAATTAAA AAAAGTGAAA TTAAAAAAAA TATAAAAATT   
  
  
- TAATAAAATT ATACAATTAT AATTTTTATT TTAAAATTTT ATTTTTTTAT AATAAAATTA TTTAAAATTT   
  
  
- TAATTTTTAT AAAATTTTTG GTTGGTTTTT TCCGTTGGAT AATGCTTGCT TGATCCTCTG TACTACTAGC   
  
  
- GACCTTCTTC TCGTTGTTCT TCGTTACTGT AGTGACTTTC TTATTGCCAA TCAGGCCACT TAAAAGTAAT   
  
  
- CGACCTTCAA TGACGATCAT CAATTAATCA ATCTTCTCGA CAACTCAATC AATCATCAAT CATCAATTAA   
  
  
- TCAATCTTTT CGAGAACTCA ACCAATCATC AATCGTCAAC TTATTTCCCC AATCTAGTTA ACATTAATCC   
  
  
- TTTTTTGGTA ACATATATTT GTCCACATAC TTTTGCCCAA CTCCTATTCT TTTATGTCTT CTAATTAGAG   
  
  
- TAAAGAGACG AGTAAGAAGA GACGAGAGAG AACAAAGACA AGAAAGAGTT AAAGACAAGA GAAAAGAAAG   
  
  
- ATTAGAACAT TTCGACTCGT ATAGTAGTGG TTCAGTAGTG CACATCTTCG TTTCCCTGTT GTTCAGTAGT   
  
  
- GGCTTCTTCT TATATCAACT GGAAGCTCTT GGGTCAGTT

+     STRE

| Site Name | Organism | Position | Strand | Matrix score. | sequence | function |
| --- | --- | --- | --- | --- | --- | --- |
| STRE | Arabidopsis thaliana | 1726 | + | 5 | AGGGG |  |

>Potri.018G038100.1   
+ GTTGCTGTCT TTTTTTTTTA TCATGTTATC AAATTAATGG AAATTTAACT CTATTATCAA AATTCTCTTT   
  
  
+ TTTATAAACA TGATAATCTT TTTTATTTTA TTTTCAAGTT AAAAATACTT GGTCCGGCCT GGGACGATGC   
  
  
+ GCTTGCCAAT CTAGTCAATC CTAAATTTAC TAATACGAGT AAGTAATAGC CGAAGGAGCA AAGCTCCAGT   
  
  
+ CCGGCATCTT GTTCAGAACA ATATTACATC CCTGAAATGC CTCGTGAATT GGGTGTCTCT TCCAACTTTC   
  
  
+ TCCAGCAATT TTCTCCCAAC TTCTGCAAGC AGTGATTATC AATTTGAATT ACAGGAGCTA CCTAACATAT   
  
  
+ CCGCCCTAGA ACGAAGGCAG GAATCATCAG CTTTGCCACC TCTTTGCATA ACTGCTACTG AAAGCCATTA   
  
  
+ GCTGGTGAAT GCTCCATTTC AAACTTCCAT TGGCCCGAGT ACTGCTCCTG CTTCCATTGG AAATATCGCT   
  
  
+ GCGCAATCAG ACGAGGCAGG CATTCATATC TGATGATAAA TGTTGCAGGA TTAATTCTAT TTTCGCCGGC   
  
  
+ AGCAAATGGT GGGATTTTTT ACACTGAAAA GTGTTTCAAA AGACTCGAAT TAATGTGGTC GATTTTGGCG   
  
  
+ GGGGCGTGGA ATGGATTCCT CTCTTCCTCA ATCATCAAAC ATCCAGATAC CTGAAACAGA GAGAAGCCCA   
  
  
+ TCTTCAAAAT CATATCGTCA TCGGAGGTCC TTCGCTGCAA TCGGCTGATG ACGGTGCACT TTTTGATGGT   
  
  
+ AGCGAACCAG ATCAGTCAAT GGTCAAATCA TTTGGAAATC AACGAGCTAC AGTTTATCCC TCCTGAATCC   
  
  
+ ATCTTTGAGA ACCTAAAAGA AATTAATTAA AATTAACGAA AGAGAAAAGA GAGAGGTGGT AGAGTTCATA   
  
  
+ GTTGTCGGAT CGGTCCGGAA ATTGATTTAA TTTAGAAATT AATATAGAAA AGAGTTTTGA GTAATTATAT   
  
  
+ TATCTTTAAC GGAGGCAAAC TAAATTAATA TTTTTATTAA AAAAATAATT TTTTTAAAAA AAACTTTTTA   
  
  
+ GACTTTTGTA TAAATTAATC AAATTAAATT AACAAGATTT TACTAAATTA ATAATTTATT AAATTTAACT   
  
  
+ TAAAATCTAA TTTAATATCA CAGCCTGCTT GATATTATAA TATCGGTTGT TTCTGGTTTA AAAATATATT   
  
  
+ AAAATATTAT TTTTTAATTT TTTAAAATTT AATTTTAACA TTAATAAATT AAAATGATCT AATTTTAATT   
  
  
+ TTTTTAAAAA AACTAAGATT CTTTTCAAAA ACATCTTTTT ACGGTGGCTT AGACTCTATT CGTTTTTATA   
  
  
+ TTTTAAAAAT TCTTTTAAAA AAATTAAAAA TTTTTAATTT TTTTCACTTT AATTTTTTTT ATATTTTTAA   
  
  
+ ATTATTTTAA TATGTTAATA TTAAAAATAA AATTTTAAAA TAAAAAAATA TTATTTTAAT AAATTTTAAA   
  
  
+ ATTAAAAATA TTTTAAAAAC CAACCAAAAA AGGCAACCTA TTACGAACGA ACTAGGAGAC ATGATGATCG   
  
  
+ CTGGAAGAAG AGCAACAAGA AGCAATGACA TCACTGAAAG AATAACGGTT AGTCCGGTGA ATTTTCATTA   
  
  
+ GCTGGAAGTT ACTGCTAGTA GTTAATTAGT TAGAAGAGCT GTTGAGTTAG TTAGTAGTTA GTAGTTAATT   
  
  
+ AGTTAGAAAA GCTCTTGAGT TGGTTAGTAG TTAGCAGTTG AATAAAGGGG TTAGATCAAT TGTAATTAGG   
  
  
+ AAAAAACCAT TGTATATAAA CAGGTGTATG AAAACGGGTT GAGGATAAGA AAATACAGAA GATTAATCTC   
  
  
+ ATTTCTCTGC TCATTCTTCT CTGCTCTCTC TTGTTTCTGT TCTTTCTCAA TTTCTGTTCT CTTTTCTTTC   
  
  
+ TAATCTTGTA AAGCTGAGCA TATCATCACC AAGTCATCAC GTGTAGAAGC AAAGGGACAA CAAGTCATCA   
  
  
+ CCGAAGAAGA ATATAGTTGA CCTTCGAGAA CCCAGTCAA  

- CAACGACAGA AAAAAAAAAT AGTACAATAG TTTAATTACC TTTAAATTGA GATAATAGTT TTAAGAGAAA   
  
  
- AAATATTTGT ACTATTAGAA AAAATAAAAT AAAAGTTCAA TTTTTATGAA CCAGGCCGGA CCCTGCTACG   
  
  
- CGAACGGTTA GATCAGTTAG GATTTAAATG ATTATGCTCA TTCATTATCG GCTTCCTCGT TTCGAGGTCA   
  
  
- GGCCGTAGAA CAAGTCTTGT TATAATGTAG GGACTTTACG GAGCACTTAA CCCACAGAGA AGGTTGAAAG   
  
  
- AGGTCGTTAA AAGAGGGTTG AAGACGTTCG TCACTAATAG TTAAACTTAA TGTCCTCGAT GGATTGTATA   
  
  
- GGCGGGATCT TGCTTCCGTC CTTAGTAGTC GAAACGGTGG AGAAACGTAT TGACGATGAC TTTCGGTAAT   
  
  
- CGACCACTTA CGAGGTAAAG TTTGAAGGTA ACCGGGCTCA TGACGAGGAC GAAGGTAACC TTTATAGCGA   
  
  
- CGCGTTAGTC TGCTCCGTCC GTAAGTATAG ACTACTATTT ACAACGTCCT AATTAAGATA AAAGCGGCCG   
  
  
- TCGTTTACCA CCCTAAAAAA TGTGACTTTT CACAAAGTTT TCTGAGCTTA ATTACACCAG CTAAAACCGC   
  
  
- CCCCGCACCT TACCTAAGGA GAGAAGGAGT TAGTAGTTTG TAGGTCTATG GACTTTGTCT CTCTTCGGGT   
  
  
- AGAAGTTTTA GTATAGCAGT AGCCTCCAGG AAGCGACGTT AGCCGACTAC TGCCACGTGA AAAACTACCA   
  
  
- TCGCTTGGTC TAGTCAGTTA CCAGTTTAGT AAACCTTTAG TTGCTCGATG TCAAATAGGG AGGACTTAGG   
  
  
- TAGAAACTCT TGGATTTTCT TTAATTAATT TTAATTGCTT TCTCTTTTCT CTCTCCACCA TCTCAAGTAT   
  
  
- CAACAGCCTA GCCAGGCCTT TAACTAAATT AAATCTTTAA TTATATCTTT TCTCAAAACT CATTAATATA   
  
  
- ATAGAAATTG CCTCCGTTTG ATTTAATTAT AAAAATAATT TTTTTATTAA AAAAATTTTT TTTGAAAAAT   
  
  
- CTGAAAACAT ATTTAATTAG TTTAATTTAA TTGTTCTAAA ATGATTTAAT TATTAAATAA TTTAAATTGA   
  
  
- ATTTTAGATT AAATTATAGT GTCGGACGAA CTATAATATT ATAGCCAACA AAGACCAAAT TTTTATATAA   
  
  
- TTTTATAATA AAAAATTAAA AAATTTTAAA TTAAAATTGT AATTATTTAA TTTTACTAGA TTAAAATTAA   
  
  
- AAAAATTTTT TTGATTCTAA GAAAAGTTTT TGTAGAAAAA TGCCACCGAA TCTGAGATAA GCAAAAATAT   
  
  
- AAAATTTTTA AGAAAATTTT TTTAATTTTT AAAAATTAAA AAAAGTGAAA TTAAAAAAAA TATAAAAATT   
  
  
- TAATAAAATT ATACAATTAT AATTTTTATT TTAAAATTTT ATTTTTTTAT AATAAAATTA TTTAAAATTT   
  
  
- TAATTTTTAT AAAATTTTTG GTTGGTTTTT TCCGTTGGAT AATGCTTGCT TGATCCTCTG TACTACTAGC   
  
  
- GACCTTCTTC TCGTTGTTCT TCGTTACTGT AGTGACTTTC TTATTGCCAA TCAGGCCACT TAAAAGTAAT   
  
  
- CGACCTTCAA TGACGATCAT CAATTAATCA ATCTTCTCGA CAACTCAATC AATCATCAAT CATCAATTAA   
  
  
- TCAATCTTTT CGAGAACTCA ACCAATCATC AATCGTCAAC TTATTTCCCC AATCTAGTTA ACATTAATCC   
  
  
- TTTTTTGGTA ACATATATTT GTCCACATAC TTTTGCCCAA CTCCTATTCT TTTATGTCTT CTAATTAGAG   
  
  
- TAAAGAGACG AGTAAGAAGA GACGAGAGAG AACAAAGACA AGAAAGAGTT AAAGACAAGA GAAAAGAAAG   
  
  
- ATTAGAACAT TTCGACTCGT ATAGTAGTGG TTCAGTAGTG CACATCTTCG TTTCCCTGTT GTTCAGTAGT   
  
  
- GGCTTCTTCT TATATCAACT GGAAGCTCTT GGGTCAGTT

+     Sp1

| Site Name | Organism | Position | Strand | Matrix score. | sequence | function |
| --- | --- | --- | --- | --- | --- | --- |
| Sp1 | Oryza sativa | 351 | - | 6 | GGGCGG | light responsive element |

>Potri.018G038100.1   
+ GTTGCTGTCT TTTTTTTTTA TCATGTTATC AAATTAATGG AAATTTAACT CTATTATCAA AATTCTCTTT   
  
  
+ TTTATAAACA TGATAATCTT TTTTATTTTA TTTTCAAGTT AAAAATACTT GGTCCGGCCT GGGACGATGC   
  
  
+ GCTTGCCAAT CTAGTCAATC CTAAATTTAC TAATACGAGT AAGTAATAGC CGAAGGAGCA AAGCTCCAGT   
  
  
+ CCGGCATCTT GTTCAGAACA ATATTACATC CCTGAAATGC CTCGTGAATT GGGTGTCTCT TCCAACTTTC   
  
  
+ TCCAGCAATT TTCTCCCAAC TTCTGCAAGC AGTGATTATC AATTTGAATT ACAGGAGCTA CCTAACATAT   
  
  
+ CCGCCCTAGA ACGAAGGCAG GAATCATCAG CTTTGCCACC TCTTTGCATA ACTGCTACTG AAAGCCATTA   
  
  
+ GCTGGTGAAT GCTCCATTTC AAACTTCCAT TGGCCCGAGT ACTGCTCCTG CTTCCATTGG AAATATCGCT   
  
  
+ GCGCAATCAG ACGAGGCAGG CATTCATATC TGATGATAAA TGTTGCAGGA TTAATTCTAT TTTCGCCGGC   
  
  
+ AGCAAATGGT GGGATTTTTT ACACTGAAAA GTGTTTCAAA AGACTCGAAT TAATGTGGTC GATTTTGGCG   
  
  
+ GGGGCGTGGA ATGGATTCCT CTCTTCCTCA ATCATCAAAC ATCCAGATAC CTGAAACAGA GAGAAGCCCA   
  
  
+ TCTTCAAAAT CATATCGTCA TCGGAGGTCC TTCGCTGCAA TCGGCTGATG ACGGTGCACT TTTTGATGGT   
  
  
+ AGCGAACCAG ATCAGTCAAT GGTCAAATCA TTTGGAAATC AACGAGCTAC AGTTTATCCC TCCTGAATCC   
  
  
+ ATCTTTGAGA ACCTAAAAGA AATTAATTAA AATTAACGAA AGAGAAAAGA GAGAGGTGGT AGAGTTCATA   
  
  
+ GTTGTCGGAT CGGTCCGGAA ATTGATTTAA TTTAGAAATT AATATAGAAA AGAGTTTTGA GTAATTATAT   
  
  
+ TATCTTTAAC GGAGGCAAAC TAAATTAATA TTTTTATTAA AAAAATAATT TTTTTAAAAA AAACTTTTTA   
  
  
+ GACTTTTGTA TAAATTAATC AAATTAAATT AACAAGATTT TACTAAATTA ATAATTTATT AAATTTAACT   
  
  
+ TAAAATCTAA TTTAATATCA CAGCCTGCTT GATATTATAA TATCGGTTGT TTCTGGTTTA AAAATATATT   
  
  
+ AAAATATTAT TTTTTAATTT TTTAAAATTT AATTTTAACA TTAATAAATT AAAATGATCT AATTTTAATT   
  
  
+ TTTTTAAAAA AACTAAGATT CTTTTCAAAA ACATCTTTTT ACGGTGGCTT AGACTCTATT CGTTTTTATA   
  
  
+ TTTTAAAAAT TCTTTTAAAA AAATTAAAAA TTTTTAATTT TTTTCACTTT AATTTTTTTT ATATTTTTAA   
  
  
+ ATTATTTTAA TATGTTAATA TTAAAAATAA AATTTTAAAA TAAAAAAATA TTATTTTAAT AAATTTTAAA   
  
  
+ ATTAAAAATA TTTTAAAAAC CAACCAAAAA AGGCAACCTA TTACGAACGA ACTAGGAGAC ATGATGATCG   
  
  
+ CTGGAAGAAG AGCAACAAGA AGCAATGACA TCACTGAAAG AATAACGGTT AGTCCGGTGA ATTTTCATTA   
  
  
+ GCTGGAAGTT ACTGCTAGTA GTTAATTAGT TAGAAGAGCT GTTGAGTTAG TTAGTAGTTA GTAGTTAATT   
  
  
+ AGTTAGAAAA GCTCTTGAGT TGGTTAGTAG TTAGCAGTTG AATAAAGGGG TTAGATCAAT TGTAATTAGG   
  
  
+ AAAAAACCAT TGTATATAAA CAGGTGTATG AAAACGGGTT GAGGATAAGA AAATACAGAA GATTAATCTC   
  
  
+ ATTTCTCTGC TCATTCTTCT CTGCTCTCTC TTGTTTCTGT TCTTTCTCAA TTTCTGTTCT CTTTTCTTTC   
  
  
+ TAATCTTGTA AAGCTGAGCA TATCATCACC AAGTCATCAC GTGTAGAAGC AAAGGGACAA CAAGTCATCA   
  
  
+ CCGAAGAAGA ATATAGTTGA CCTTCGAGAA CCCAGTCAA  

- CAACGACAGA AAAAAAAAAT AGTACAATAG TTTAATTACC TTTAAATTGA GATAATAGTT TTAAGAGAAA   
  
  
- AAATATTTGT ACTATTAGAA AAAATAAAAT AAAAGTTCAA TTTTTATGAA CCAGGCCGGA CCCTGCTACG   
  
  
- CGAACGGTTA GATCAGTTAG GATTTAAATG ATTATGCTCA TTCATTATCG GCTTCCTCGT TTCGAGGTCA   
  
  
- GGCCGTAGAA CAAGTCTTGT TATAATGTAG GGACTTTACG GAGCACTTAA CCCACAGAGA AGGTTGAAAG   
  
  
- AGGTCGTTAA AAGAGGGTTG AAGACGTTCG TCACTAATAG TTAAACTTAA TGTCCTCGAT GGATTGTATA   
  
  
- GGCGGGATCT TGCTTCCGTC CTTAGTAGTC GAAACGGTGG AGAAACGTAT TGACGATGAC TTTCGGTAAT   
  
  
- CGACCACTTA CGAGGTAAAG TTTGAAGGTA ACCGGGCTCA TGACGAGGAC GAAGGTAACC TTTATAGCGA   
  
  
- CGCGTTAGTC TGCTCCGTCC GTAAGTATAG ACTACTATTT ACAACGTCCT AATTAAGATA AAAGCGGCCG   
  
  
- TCGTTTACCA CCCTAAAAAA TGTGACTTTT CACAAAGTTT TCTGAGCTTA ATTACACCAG CTAAAACCGC   
  
  
- CCCCGCACCT TACCTAAGGA GAGAAGGAGT TAGTAGTTTG TAGGTCTATG GACTTTGTCT CTCTTCGGGT   
  
  
- AGAAGTTTTA GTATAGCAGT AGCCTCCAGG AAGCGACGTT AGCCGACTAC TGCCACGTGA AAAACTACCA   
  
  
- TCGCTTGGTC TAGTCAGTTA CCAGTTTAGT AAACCTTTAG TTGCTCGATG TCAAATAGGG AGGACTTAGG   
  
  
- TAGAAACTCT TGGATTTTCT TTAATTAATT TTAATTGCTT TCTCTTTTCT CTCTCCACCA TCTCAAGTAT   
  
  
- CAACAGCCTA GCCAGGCCTT TAACTAAATT AAATCTTTAA TTATATCTTT TCTCAAAACT CATTAATATA   
  
  
- ATAGAAATTG CCTCCGTTTG ATTTAATTAT AAAAATAATT TTTTTATTAA AAAAATTTTT TTTGAAAAAT   
  
  
- CTGAAAACAT ATTTAATTAG TTTAATTTAA TTGTTCTAAA ATGATTTAAT TATTAAATAA TTTAAATTGA   
  
  
- ATTTTAGATT AAATTATAGT GTCGGACGAA CTATAATATT ATAGCCAACA AAGACCAAAT TTTTATATAA   
  
  
- TTTTATAATA AAAAATTAAA AAATTTTAAA TTAAAATTGT AATTATTTAA TTTTACTAGA TTAAAATTAA   
  
  
- AAAAATTTTT TTGATTCTAA GAAAAGTTTT TGTAGAAAAA TGCCACCGAA TCTGAGATAA GCAAAAATAT   
  
  
- AAAATTTTTA AGAAAATTTT TTTAATTTTT AAAAATTAAA AAAAGTGAAA TTAAAAAAAA TATAAAAATT   
  
  
- TAATAAAATT ATACAATTAT AATTTTTATT TTAAAATTTT ATTTTTTTAT AATAAAATTA TTTAAAATTT   
  
  
- TAATTTTTAT AAAATTTTTG GTTGGTTTTT TCCGTTGGAT AATGCTTGCT TGATCCTCTG TACTACTAGC   
  
  
- GACCTTCTTC TCGTTGTTCT TCGTTACTGT AGTGACTTTC TTATTGCCAA TCAGGCCACT TAAAAGTAAT   
  
  
- CGACCTTCAA TGACGATCAT CAATTAATCA ATCTTCTCGA CAACTCAATC AATCATCAAT CATCAATTAA   
  
  
- TCAATCTTTT CGAGAACTCA ACCAATCATC AATCGTCAAC TTATTTCCCC AATCTAGTTA ACATTAATCC   
  
  
- TTTTTTGGTA ACATATATTT GTCCACATAC TTTTGCCCAA CTCCTATTCT TTTATGTCTT CTAATTAGAG   
  
  
- TAAAGAGACG AGTAAGAAGA GACGAGAGAG AACAAAGACA AGAAAGAGTT AAAGACAAGA GAAAAGAAAG   
  
  
- ATTAGAACAT TTCGACTCGT ATAGTAGTGG TTCAGTAGTG CACATCTTCG TTTCCCTGTT GTTCAGTAGT   
  
  
- GGCTTCTTCT TATATCAACT GGAAGCTCTT GGGTCAGTT

+     TATA-box

| Site Name | Organism | Position | Strand | Matrix score. | sequence | function |
| --- | --- | --- | --- | --- | --- | --- |
| TATA-box | Arabidopsis thaliana | 1765 | - | 4 | TATA | core promoter element around -30 of transcription start |
| TATA-box | Arabidopsis thaliana | 1327 | - | 4 | TATA | core promoter element around -30 of transcription start |
| TATA-box | Pisum sativum | 1324 | - | 7 | TATAAAA | core promoter element around -30 of transcription start |
| TATA-box | Arabidopsis thaliana | 1390 | - | 4 | TATA | core promoter element around -30 of transcription start |
| TATA-box | Brassica napus | 1184 | - | 6 | ATATAT | core promoter element around -30 of transcription start |
| TATA-box | Helianthus annuus | 1325 | - | 6 | TATAAA | core promoter element around -30 of transcription start |
| TATA-box | Pisum sativum | 1387 | - | 7 | TATAAAA | core promoter element around -30 of transcription start |
| TATA-box | Brassica napus | 974 | + | 6 | ATTATA | core promoter element around -30 of transcription start |
| TATA-box | Helianthus annuus | 1057 | - | 6 | TATACA | core promoter element around -30 of transcription start |
| TATA-box | Oryza sativa | 1054 | - | 7 | TACAAAA | core promoter element around -30 of transcription start |
| TATA-box | Arabidopsis thaliana | 1972 | - | 4 | TATA | core promoter element around -30 of transcription start |
| TATA-box | Helianthus annuus | 71 | - | 6 | TATAAA | core promoter element around -30 of transcription start |
| TATA-box | Arabidopsis thaliana | 1326 | - | 5 | TATAA | core promoter element around -30 of transcription start |
| TATA-box | Arabidopsis thaliana | 72 | - | 5 | TATAA | core promoter element around -30 of transcription start |
| TATA-box | Helianthus annuus | 1761 | - | 6 | TATACA | core promoter element around -30 of transcription start |
| TATA-box | Arabidopsis thaliana | 1155 | - | 5 | TATAA | core promoter element around -30 of transcription start |
| TATA-box | Arabidopsis thaliana | 1059 | - | 4 | TATA | core promoter element around -30 of transcription start |
| TATA-box | Arabidopsis thaliana | 1156 | - | 4 | TATA | core promoter element around -30 of transcription start |
| TATA-box | Arabidopsis thaliana | 1389 | - | 5 | TATAA | core promoter element around -30 of transcription start |
| TATA-box | Pisum sativum | 70 | - | 7 | TATAAAA | core promoter element around -30 of transcription start |
| TATA-box | Brassica oleracea | 1764 | + | 6 | ATATAA | core promoter element around -30 of transcription start |
| TATA-box | Arabidopsis thaliana | 953 | + | 4 | TATA | core promoter element around -30 of transcription start |
| TATA-box | Helianthus annuus | 1388 | - | 6 | TATAAA | core promoter element around -30 of transcription start |
| TATA-box | Arabidopsis thaliana | 975 | - | 5 | TATAA | core promoter element around -30 of transcription start |
| TATA-box | Arabidopsis thaliana | 1763 | - | 6 | TATATA | core promoter element around -30 of transcription start |
| TATA-box | Arabidopsis thaliana | 1185 | - | 4 | TATA | core promoter element around -30 of transcription start |
| TATA-box | Arabidopsis thaliana | 976 | + | 4 | TATA | core promoter element around -30 of transcription start |
| TATA-box | Brassica napus | 1154 | + | 6 | ATTATA | core promoter element around -30 of transcription start |
| TATA-box | Arabidopsis thaliana | 73 | + | 4 | TATA | core promoter element around -30 of transcription start |

>Potri.018G038100.1   
+ GTTGCTGTCT TTTTTTTTTA TCATGTTATC AAATTAATGG AAATTTAACT CTATTATCAA AATTCTCTTT   
  
  
+ TTTATAAACA TGATAATCTT TTTTATTTTA TTTTCAAGTT AAAAATACTT GGTCCGGCCT GGGACGATGC   
  
  
+ GCTTGCCAAT CTAGTCAATC CTAAATTTAC TAATACGAGT AAGTAATAGC CGAAGGAGCA AAGCTCCAGT   
  
  
+ CCGGCATCTT GTTCAGAACA ATATTACATC CCTGAAATGC CTCGTGAATT GGGTGTCTCT TCCAACTTTC   
  
  
+ TCCAGCAATT TTCTCCCAAC TTCTGCAAGC AGTGATTATC AATTTGAATT ACAGGAGCTA CCTAACATAT   
  
  
+ CCGCCCTAGA ACGAAGGCAG GAATCATCAG CTTTGCCACC TCTTTGCATA ACTGCTACTG AAAGCCATTA   
  
  
+ GCTGGTGAAT GCTCCATTTC AAACTTCCAT TGGCCCGAGT ACTGCTCCTG CTTCCATTGG AAATATCGCT   
  
  
+ GCGCAATCAG ACGAGGCAGG CATTCATATC TGATGATAAA TGTTGCAGGA TTAATTCTAT TTTCGCCGGC   
  
  
+ AGCAAATGGT GGGATTTTTT ACACTGAAAA GTGTTTCAAA AGACTCGAAT TAATGTGGTC GATTTTGGCG   
  
  
+ GGGGCGTGGA ATGGATTCCT CTCTTCCTCA ATCATCAAAC ATCCAGATAC CTGAAACAGA GAGAAGCCCA   
  
  
+ TCTTCAAAAT CATATCGTCA TCGGAGGTCC TTCGCTGCAA TCGGCTGATG ACGGTGCACT TTTTGATGGT   
  
  
+ AGCGAACCAG ATCAGTCAAT GGTCAAATCA TTTGGAAATC AACGAGCTAC AGTTTATCCC TCCTGAATCC   
  
  
+ ATCTTTGAGA ACCTAAAAGA AATTAATTAA AATTAACGAA AGAGAAAAGA GAGAGGTGGT AGAGTTCATA   
  
  
+ GTTGTCGGAT CGGTCCGGAA ATTGATTTAA TTTAGAAATT AATATAGAAA AGAGTTTTGA GTAATTATAT   
  
  
+ TATCTTTAAC GGAGGCAAAC TAAATTAATA TTTTTATTAA AAAAATAATT TTTTTAAAAA AAACTTTTTA   
  
  
+ GACTTTTGTA TAAATTAATC AAATTAAATT AACAAGATTT TACTAAATTA ATAATTTATT AAATTTAACT   
  
  
+ TAAAATCTAA TTTAATATCA CAGCCTGCTT GATATTATAA TATCGGTTGT TTCTGGTTTA AAAATATATT   
  
  
+ AAAATATTAT TTTTTAATTT TTTAAAATTT AATTTTAACA TTAATAAATT AAAATGATCT AATTTTAATT   
  
  
+ TTTTTAAAAA AACTAAGATT CTTTTCAAAA ACATCTTTTT ACGGTGGCTT AGACTCTATT CGTTTTTATA   
  
  
+ TTTTAAAAAT TCTTTTAAAA AAATTAAAAA TTTTTAATTT TTTTCACTTT AATTTTTTTT ATATTTTTAA   
  
  
+ ATTATTTTAA TATGTTAATA TTAAAAATAA AATTTTAAAA TAAAAAAATA TTATTTTAAT AAATTTTAAA   
  
  
+ ATTAAAAATA TTTTAAAAAC CAACCAAAAA AGGCAACCTA TTACGAACGA ACTAGGAGAC ATGATGATCG   
  
  
+ CTGGAAGAAG AGCAACAAGA AGCAATGACA TCACTGAAAG AATAACGGTT AGTCCGGTGA ATTTTCATTA   
  
  
+ GCTGGAAGTT ACTGCTAGTA GTTAATTAGT TAGAAGAGCT GTTGAGTTAG TTAGTAGTTA GTAGTTAATT   
  
  
+ AGTTAGAAAA GCTCTTGAGT TGGTTAGTAG TTAGCAGTTG AATAAAGGGG TTAGATCAAT TGTAATTAGG   
  
  
+ AAAAAACCAT TGTATATAAA CAGGTGTATG AAAACGGGTT GAGGATAAGA AAATACAGAA GATTAATCTC   
  
  
+ ATTTCTCTGC TCATTCTTCT CTGCTCTCTC TTGTTTCTGT TCTTTCTCAA TTTCTGTTCT CTTTTCTTTC   
  
  
+ TAATCTTGTA AAGCTGAGCA TATCATCACC AAGTCATCAC GTGTAGAAGC AAAGGGACAA CAAGTCATCA   
  
  
+ CCGAAGAAGA ATATAGTTGA CCTTCGAGAA CCCAGTCAA  

- CAACGACAGA AAAAAAAAAT AGTACAATAG TTTAATTACC TTTAAATTGA GATAATAGTT TTAAGAGAAA   
  
  
- AAATATTTGT ACTATTAGAA AAAATAAAAT AAAAGTTCAA TTTTTATGAA CCAGGCCGGA CCCTGCTACG   
  
  
- CGAACGGTTA GATCAGTTAG GATTTAAATG ATTATGCTCA TTCATTATCG GCTTCCTCGT TTCGAGGTCA   
  
  
- GGCCGTAGAA CAAGTCTTGT TATAATGTAG GGACTTTACG GAGCACTTAA CCCACAGAGA AGGTTGAAAG   
  
  
- AGGTCGTTAA AAGAGGGTTG AAGACGTTCG TCACTAATAG TTAAACTTAA TGTCCTCGAT GGATTGTATA   
  
  
- GGCGGGATCT TGCTTCCGTC CTTAGTAGTC GAAACGGTGG AGAAACGTAT TGACGATGAC TTTCGGTAAT   
  
  
- CGACCACTTA CGAGGTAAAG TTTGAAGGTA ACCGGGCTCA TGACGAGGAC GAAGGTAACC TTTATAGCGA   
  
  
- CGCGTTAGTC TGCTCCGTCC GTAAGTATAG ACTACTATTT ACAACGTCCT AATTAAGATA AAAGCGGCCG   
  
  
- TCGTTTACCA CCCTAAAAAA TGTGACTTTT CACAAAGTTT TCTGAGCTTA ATTACACCAG CTAAAACCGC   
  
  
- CCCCGCACCT TACCTAAGGA GAGAAGGAGT TAGTAGTTTG TAGGTCTATG GACTTTGTCT CTCTTCGGGT   
  
  
- AGAAGTTTTA GTATAGCAGT AGCCTCCAGG AAGCGACGTT AGCCGACTAC TGCCACGTGA AAAACTACCA   
  
  
- TCGCTTGGTC TAGTCAGTTA CCAGTTTAGT AAACCTTTAG TTGCTCGATG TCAAATAGGG AGGACTTAGG   
  
  
- TAGAAACTCT TGGATTTTCT TTAATTAATT TTAATTGCTT TCTCTTTTCT CTCTCCACCA TCTCAAGTAT   
  
  
- CAACAGCCTA GCCAGGCCTT TAACTAAATT AAATCTTTAA TTATATCTTT TCTCAAAACT CATTAATATA   
  
  
- ATAGAAATTG CCTCCGTTTG ATTTAATTAT AAAAATAATT TTTTTATTAA AAAAATTTTT TTTGAAAAAT   
  
  
- CTGAAAACAT ATTTAATTAG TTTAATTTAA TTGTTCTAAA ATGATTTAAT TATTAAATAA TTTAAATTGA   
  
  
- ATTTTAGATT AAATTATAGT GTCGGACGAA CTATAATATT ATAGCCAACA AAGACCAAAT TTTTATATAA   
  
  
- TTTTATAATA AAAAATTAAA AAATTTTAAA TTAAAATTGT AATTATTTAA TTTTACTAGA TTAAAATTAA   
  
  
- AAAAATTTTT TTGATTCTAA GAAAAGTTTT TGTAGAAAAA TGCCACCGAA TCTGAGATAA GCAAAAATAT   
  
  
- AAAATTTTTA AGAAAATTTT TTTAATTTTT AAAAATTAAA AAAAGTGAAA TTAAAAAAAA TATAAAAATT   
  
  
- TAATAAAATT ATACAATTAT AATTTTTATT TTAAAATTTT ATTTTTTTAT AATAAAATTA TTTAAAATTT   
  
  
- TAATTTTTAT AAAATTTTTG GTTGGTTTTT TCCGTTGGAT AATGCTTGCT TGATCCTCTG TACTACTAGC   
  
  
- GACCTTCTTC TCGTTGTTCT TCGTTACTGT AGTGACTTTC TTATTGCCAA TCAGGCCACT TAAAAGTAAT   
  
  
- CGACCTTCAA TGACGATCAT CAATTAATCA ATCTTCTCGA CAACTCAATC AATCATCAAT CATCAATTAA   
  
  
- TCAATCTTTT CGAGAACTCA ACCAATCATC AATCGTCAAC TTATTTCCCC AATCTAGTTA ACATTAATCC   
  
  
- TTTTTTGGTA ACATATATTT GTCCACATAC TTTTGCCCAA CTCCTATTCT TTTATGTCTT CTAATTAGAG   
  
  
- TAAAGAGACG AGTAAGAAGA GACGAGAGAG AACAAAGACA AGAAAGAGTT AAAGACAAGA GAAAAGAAAG   
  
  
- ATTAGAACAT TTCGACTCGT ATAGTAGTGG TTCAGTAGTG CACATCTTCG TTTCCCTGTT GTTCAGTAGT   
  
  
- GGCTTCTTCT TATATCAACT GGAAGCTCTT GGGTCAGTT

+     TC-rich repeats

| Site Name | Organism | Position | Strand | Matrix score. | sequence | function |
| --- | --- | --- | --- | --- | --- | --- |
| TC-rich repeats | Nicotiana tabacum | 1776 | - | 9 | GTTTTCTTAC | cis-acting element involved in defense and stress responsiveness |

>Potri.018G038100.1   
+ GTTGCTGTCT TTTTTTTTTA TCATGTTATC AAATTAATGG AAATTTAACT CTATTATCAA AATTCTCTTT   
  
  
+ TTTATAAACA TGATAATCTT TTTTATTTTA TTTTCAAGTT AAAAATACTT GGTCCGGCCT GGGACGATGC   
  
  
+ GCTTGCCAAT CTAGTCAATC CTAAATTTAC TAATACGAGT AAGTAATAGC CGAAGGAGCA AAGCTCCAGT   
  
  
+ CCGGCATCTT GTTCAGAACA ATATTACATC CCTGAAATGC CTCGTGAATT GGGTGTCTCT TCCAACTTTC   
  
  
+ TCCAGCAATT TTCTCCCAAC TTCTGCAAGC AGTGATTATC AATTTGAATT ACAGGAGCTA CCTAACATAT   
  
  
+ CCGCCCTAGA ACGAAGGCAG GAATCATCAG CTTTGCCACC TCTTTGCATA ACTGCTACTG AAAGCCATTA   
  
  
+ GCTGGTGAAT GCTCCATTTC AAACTTCCAT TGGCCCGAGT ACTGCTCCTG CTTCCATTGG AAATATCGCT   
  
  
+ GCGCAATCAG ACGAGGCAGG CATTCATATC TGATGATAAA TGTTGCAGGA TTAATTCTAT TTTCGCCGGC   
  
  
+ AGCAAATGGT GGGATTTTTT ACACTGAAAA GTGTTTCAAA AGACTCGAAT TAATGTGGTC GATTTTGGCG   
  
  
+ GGGGCGTGGA ATGGATTCCT CTCTTCCTCA ATCATCAAAC ATCCAGATAC CTGAAACAGA GAGAAGCCCA   
  
  
+ TCTTCAAAAT CATATCGTCA TCGGAGGTCC TTCGCTGCAA TCGGCTGATG ACGGTGCACT TTTTGATGGT   
  
  
+ AGCGAACCAG ATCAGTCAAT GGTCAAATCA TTTGGAAATC AACGAGCTAC AGTTTATCCC TCCTGAATCC   
  
  
+ ATCTTTGAGA ACCTAAAAGA AATTAATTAA AATTAACGAA AGAGAAAAGA GAGAGGTGGT AGAGTTCATA   
  
  
+ GTTGTCGGAT CGGTCCGGAA ATTGATTTAA TTTAGAAATT AATATAGAAA AGAGTTTTGA GTAATTATAT   
  
  
+ TATCTTTAAC GGAGGCAAAC TAAATTAATA TTTTTATTAA AAAAATAATT TTTTTAAAAA AAACTTTTTA   
  
  
+ GACTTTTGTA TAAATTAATC AAATTAAATT AACAAGATTT TACTAAATTA ATAATTTATT AAATTTAACT   
  
  
+ TAAAATCTAA TTTAATATCA CAGCCTGCTT GATATTATAA TATCGGTTGT TTCTGGTTTA AAAATATATT   
  
  
+ AAAATATTAT TTTTTAATTT TTTAAAATTT AATTTTAACA TTAATAAATT AAAATGATCT AATTTTAATT   
  
  
+ TTTTTAAAAA AACTAAGATT CTTTTCAAAA ACATCTTTTT ACGGTGGCTT AGACTCTATT CGTTTTTATA   
  
  
+ TTTTAAAAAT TCTTTTAAAA AAATTAAAAA TTTTTAATTT TTTTCACTTT AATTTTTTTT ATATTTTTAA   
  
  
+ ATTATTTTAA TATGTTAATA TTAAAAATAA AATTTTAAAA TAAAAAAATA TTATTTTAAT AAATTTTAAA   
  
  
+ ATTAAAAATA TTTTAAAAAC CAACCAAAAA AGGCAACCTA TTACGAACGA ACTAGGAGAC ATGATGATCG   
  
  
+ CTGGAAGAAG AGCAACAAGA AGCAATGACA TCACTGAAAG AATAACGGTT AGTCCGGTGA ATTTTCATTA   
  
  
+ GCTGGAAGTT ACTGCTAGTA GTTAATTAGT TAGAAGAGCT GTTGAGTTAG TTAGTAGTTA GTAGTTAATT   
  
  
+ AGTTAGAAAA GCTCTTGAGT TGGTTAGTAG TTAGCAGTTG AATAAAGGGG TTAGATCAAT TGTAATTAGG   
  
  
+ AAAAAACCAT TGTATATAAA CAGGTGTATG AAAACGGGTT GAGGATAAGA AAATACAGAA GATTAATCTC   
  
  
+ ATTTCTCTGC TCATTCTTCT CTGCTCTCTC TTGTTTCTGT TCTTTCTCAA TTTCTGTTCT CTTTTCTTTC   
  
  
+ TAATCTTGTA AAGCTGAGCA TATCATCACC AAGTCATCAC GTGTAGAAGC AAAGGGACAA CAAGTCATCA   
  
  
+ CCGAAGAAGA ATATAGTTGA CCTTCGAGAA CCCAGTCAA  

- CAACGACAGA AAAAAAAAAT AGTACAATAG TTTAATTACC TTTAAATTGA GATAATAGTT TTAAGAGAAA   
  
  
- AAATATTTGT ACTATTAGAA AAAATAAAAT AAAAGTTCAA TTTTTATGAA CCAGGCCGGA CCCTGCTACG   
  
  
- CGAACGGTTA GATCAGTTAG GATTTAAATG ATTATGCTCA TTCATTATCG GCTTCCTCGT TTCGAGGTCA   
  
  
- GGCCGTAGAA CAAGTCTTGT TATAATGTAG GGACTTTACG GAGCACTTAA CCCACAGAGA AGGTTGAAAG   
  
  
- AGGTCGTTAA AAGAGGGTTG AAGACGTTCG TCACTAATAG TTAAACTTAA TGTCCTCGAT GGATTGTATA   
  
  
- GGCGGGATCT TGCTTCCGTC CTTAGTAGTC GAAACGGTGG AGAAACGTAT TGACGATGAC TTTCGGTAAT   
  
  
- CGACCACTTA CGAGGTAAAG TTTGAAGGTA ACCGGGCTCA TGACGAGGAC GAAGGTAACC TTTATAGCGA   
  
  
- CGCGTTAGTC TGCTCCGTCC GTAAGTATAG ACTACTATTT ACAACGTCCT AATTAAGATA AAAGCGGCCG   
  
  
- TCGTTTACCA CCCTAAAAAA TGTGACTTTT CACAAAGTTT TCTGAGCTTA ATTACACCAG CTAAAACCGC   
  
  
- CCCCGCACCT TACCTAAGGA GAGAAGGAGT TAGTAGTTTG TAGGTCTATG GACTTTGTCT CTCTTCGGGT   
  
  
- AGAAGTTTTA GTATAGCAGT AGCCTCCAGG AAGCGACGTT AGCCGACTAC TGCCACGTGA AAAACTACCA   
  
  
- TCGCTTGGTC TAGTCAGTTA CCAGTTTAGT AAACCTTTAG TTGCTCGATG TCAAATAGGG AGGACTTAGG   
  
  
- TAGAAACTCT TGGATTTTCT TTAATTAATT TTAATTGCTT TCTCTTTTCT CTCTCCACCA TCTCAAGTAT   
  
  
- CAACAGCCTA GCCAGGCCTT TAACTAAATT AAATCTTTAA TTATATCTTT TCTCAAAACT CATTAATATA   
  
  
- ATAGAAATTG CCTCCGTTTG ATTTAATTAT AAAAATAATT TTTTTATTAA AAAAATTTTT TTTGAAAAAT   
  
  
- CTGAAAACAT ATTTAATTAG TTTAATTTAA TTGTTCTAAA ATGATTTAAT TATTAAATAA TTTAAATTGA   
  
  
- ATTTTAGATT AAATTATAGT GTCGGACGAA CTATAATATT ATAGCCAACA AAGACCAAAT TTTTATATAA   
  
  
- TTTTATAATA AAAAATTAAA AAATTTTAAA TTAAAATTGT AATTATTTAA TTTTACTAGA TTAAAATTAA   
  
  
- AAAAATTTTT TTGATTCTAA GAAAAGTTTT TGTAGAAAAA TGCCACCGAA TCTGAGATAA GCAAAAATAT   
  
  
- AAAATTTTTA AGAAAATTTT TTTAATTTTT AAAAATTAAA AAAAGTGAAA TTAAAAAAAA TATAAAAATT   
  
  
- TAATAAAATT ATACAATTAT AATTTTTATT TTAAAATTTT ATTTTTTTAT AATAAAATTA TTTAAAATTT   
  
  
- TAATTTTTAT AAAATTTTTG GTTGGTTTTT TCCGTTGGAT AATGCTTGCT TGATCCTCTG TACTACTAGC   
  
  
- GACCTTCTTC TCGTTGTTCT TCGTTACTGT AGTGACTTTC TTATTGCCAA TCAGGCCACT TAAAAGTAAT   
  
  
- CGACCTTCAA TGACGATCAT CAATTAATCA ATCTTCTCGA CAACTCAATC AATCATCAAT CATCAATTAA   
  
  
- TCAATCTTTT CGAGAACTCA ACCAATCATC AATCGTCAAC TTATTTCCCC AATCTAGTTA ACATTAATCC   
  
  
- TTTTTTGGTA ACATATATTT GTCCACATAC TTTTGCCCAA CTCCTATTCT TTTATGTCTT CTAATTAGAG   
  
  
- TAAAGAGACG AGTAAGAAGA GACGAGAGAG AACAAAGACA AGAAAGAGTT AAAGACAAGA GAAAAGAAAG   
  
  
- ATTAGAACAT TTCGACTCGT ATAGTAGTGG TTCAGTAGTG CACATCTTCG TTTCCCTGTT GTTCAGTAGT   
  
  
- GGCTTCTTCT TATATCAACT GGAAGCTCTT GGGTCAGTT

+     TCA-element

| Site Name | Organism | Position | Strand | Matrix score. | sequence | function |
| --- | --- | --- | --- | --- | --- | --- |
| TCA-element | Nicotiana tabacum | 1291 | + | 9 | CCATCTTTTT | cis-acting element involved in salicylic acid responsiveness |
| TCA-element | Brassica oleracea | 497 | + | 9 | TCAGAAGAGG | cis-acting element involved in salicylic acid responsiveness |

>Potri.018G038100.1   
+ GTTGCTGTCT TTTTTTTTTA TCATGTTATC AAATTAATGG AAATTTAACT CTATTATCAA AATTCTCTTT   
  
  
+ TTTATAAACA TGATAATCTT TTTTATTTTA TTTTCAAGTT AAAAATACTT GGTCCGGCCT GGGACGATGC   
  
  
+ GCTTGCCAAT CTAGTCAATC CTAAATTTAC TAATACGAGT AAGTAATAGC CGAAGGAGCA AAGCTCCAGT   
  
  
+ CCGGCATCTT GTTCAGAACA ATATTACATC CCTGAAATGC CTCGTGAATT GGGTGTCTCT TCCAACTTTC   
  
  
+ TCCAGCAATT TTCTCCCAAC TTCTGCAAGC AGTGATTATC AATTTGAATT ACAGGAGCTA CCTAACATAT   
  
  
+ CCGCCCTAGA ACGAAGGCAG GAATCATCAG CTTTGCCACC TCTTTGCATA ACTGCTACTG AAAGCCATTA   
  
  
+ GCTGGTGAAT GCTCCATTTC AAACTTCCAT TGGCCCGAGT ACTGCTCCTG CTTCCATTGG AAATATCGCT   
  
  
+ GCGCAATCAG ACGAGGCAGG CATTCATATC TGATGATAAA TGTTGCAGGA TTAATTCTAT TTTCGCCGGC   
  
  
+ AGCAAATGGT GGGATTTTTT ACACTGAAAA GTGTTTCAAA AGACTCGAAT TAATGTGGTC GATTTTGGCG   
  
  
+ GGGGCGTGGA ATGGATTCCT CTCTTCCTCA ATCATCAAAC ATCCAGATAC CTGAAACAGA GAGAAGCCCA   
  
  
+ TCTTCAAAAT CATATCGTCA TCGGAGGTCC TTCGCTGCAA TCGGCTGATG ACGGTGCACT TTTTGATGGT   
  
  
+ AGCGAACCAG ATCAGTCAAT GGTCAAATCA TTTGGAAATC AACGAGCTAC AGTTTATCCC TCCTGAATCC   
  
  
+ ATCTTTGAGA ACCTAAAAGA AATTAATTAA AATTAACGAA AGAGAAAAGA GAGAGGTGGT AGAGTTCATA   
  
  
+ GTTGTCGGAT CGGTCCGGAA ATTGATTTAA TTTAGAAATT AATATAGAAA AGAGTTTTGA GTAATTATAT   
  
  
+ TATCTTTAAC GGAGGCAAAC TAAATTAATA TTTTTATTAA AAAAATAATT TTTTTAAAAA AAACTTTTTA   
  
  
+ GACTTTTGTA TAAATTAATC AAATTAAATT AACAAGATTT TACTAAATTA ATAATTTATT AAATTTAACT   
  
  
+ TAAAATCTAA TTTAATATCA CAGCCTGCTT GATATTATAA TATCGGTTGT TTCTGGTTTA AAAATATATT   
  
  
+ AAAATATTAT TTTTTAATTT TTTAAAATTT AATTTTAACA TTAATAAATT AAAATGATCT AATTTTAATT   
  
  
+ TTTTTAAAAA AACTAAGATT CTTTTCAAAA ACATCTTTTT ACGGTGGCTT AGACTCTATT CGTTTTTATA   
  
  
+ TTTTAAAAAT TCTTTTAAAA AAATTAAAAA TTTTTAATTT TTTTCACTTT AATTTTTTTT ATATTTTTAA   
  
  
+ ATTATTTTAA TATGTTAATA TTAAAAATAA AATTTTAAAA TAAAAAAATA TTATTTTAAT AAATTTTAAA   
  
  
+ ATTAAAAATA TTTTAAAAAC CAACCAAAAA AGGCAACCTA TTACGAACGA ACTAGGAGAC ATGATGATCG   
  
  
+ CTGGAAGAAG AGCAACAAGA AGCAATGACA TCACTGAAAG AATAACGGTT AGTCCGGTGA ATTTTCATTA   
  
  
+ GCTGGAAGTT ACTGCTAGTA GTTAATTAGT TAGAAGAGCT GTTGAGTTAG TTAGTAGTTA GTAGTTAATT   
  
  
+ AGTTAGAAAA GCTCTTGAGT TGGTTAGTAG TTAGCAGTTG AATAAAGGGG TTAGATCAAT TGTAATTAGG   
  
  
+ AAAAAACCAT TGTATATAAA CAGGTGTATG AAAACGGGTT GAGGATAAGA AAATACAGAA GATTAATCTC   
  
  
+ ATTTCTCTGC TCATTCTTCT CTGCTCTCTC TTGTTTCTGT TCTTTCTCAA TTTCTGTTCT CTTTTCTTTC   
  
  
+ TAATCTTGTA AAGCTGAGCA TATCATCACC AAGTCATCAC GTGTAGAAGC AAAGGGACAA CAAGTCATCA   
  
  
+ CCGAAGAAGA ATATAGTTGA CCTTCGAGAA CCCAGTCAA  

- CAACGACAGA AAAAAAAAAT AGTACAATAG TTTAATTACC TTTAAATTGA GATAATAGTT TTAAGAGAAA   
  
  
- AAATATTTGT ACTATTAGAA AAAATAAAAT AAAAGTTCAA TTTTTATGAA CCAGGCCGGA CCCTGCTACG   
  
  
- CGAACGGTTA GATCAGTTAG GATTTAAATG ATTATGCTCA TTCATTATCG GCTTCCTCGT TTCGAGGTCA   
  
  
- GGCCGTAGAA CAAGTCTTGT TATAATGTAG GGACTTTACG GAGCACTTAA CCCACAGAGA AGGTTGAAAG   
  
  
- AGGTCGTTAA AAGAGGGTTG AAGACGTTCG TCACTAATAG TTAAACTTAA TGTCCTCGAT GGATTGTATA   
  
  
- GGCGGGATCT TGCTTCCGTC CTTAGTAGTC GAAACGGTGG AGAAACGTAT TGACGATGAC TTTCGGTAAT   
  
  
- CGACCACTTA CGAGGTAAAG TTTGAAGGTA ACCGGGCTCA TGACGAGGAC GAAGGTAACC TTTATAGCGA   
  
  
- CGCGTTAGTC TGCTCCGTCC GTAAGTATAG ACTACTATTT ACAACGTCCT AATTAAGATA AAAGCGGCCG   
  
  
- TCGTTTACCA CCCTAAAAAA TGTGACTTTT CACAAAGTTT TCTGAGCTTA ATTACACCAG CTAAAACCGC   
  
  
- CCCCGCACCT TACCTAAGGA GAGAAGGAGT TAGTAGTTTG TAGGTCTATG GACTTTGTCT CTCTTCGGGT   
  
  
- AGAAGTTTTA GTATAGCAGT AGCCTCCAGG AAGCGACGTT AGCCGACTAC TGCCACGTGA AAAACTACCA   
  
  
- TCGCTTGGTC TAGTCAGTTA CCAGTTTAGT AAACCTTTAG TTGCTCGATG TCAAATAGGG AGGACTTAGG   
  
  
- TAGAAACTCT TGGATTTTCT TTAATTAATT TTAATTGCTT TCTCTTTTCT CTCTCCACCA TCTCAAGTAT   
  
  
- CAACAGCCTA GCCAGGCCTT TAACTAAATT AAATCTTTAA TTATATCTTT TCTCAAAACT CATTAATATA   
  
  
- ATAGAAATTG CCTCCGTTTG ATTTAATTAT AAAAATAATT TTTTTATTAA AAAAATTTTT TTTGAAAAAT   
  
  
- CTGAAAACAT ATTTAATTAG TTTAATTTAA TTGTTCTAAA ATGATTTAAT TATTAAATAA TTTAAATTGA   
  
  
- ATTTTAGATT AAATTATAGT GTCGGACGAA CTATAATATT ATAGCCAACA AAGACCAAAT TTTTATATAA   
  
  
- TTTTATAATA AAAAATTAAA AAATTTTAAA TTAAAATTGT AATTATTTAA TTTTACTAGA TTAAAATTAA   
  
  
- AAAAATTTTT TTGATTCTAA GAAAAGTTTT TGTAGAAAAA TGCCACCGAA TCTGAGATAA GCAAAAATAT   
  
  
- AAAATTTTTA AGAAAATTTT TTTAATTTTT AAAAATTAAA AAAAGTGAAA TTAAAAAAAA TATAAAAATT   
  
  
- TAATAAAATT ATACAATTAT AATTTTTATT TTAAAATTTT ATTTTTTTAT AATAAAATTA TTTAAAATTT   
  
  
- TAATTTTTAT AAAATTTTTG GTTGGTTTTT TCCGTTGGAT AATGCTTGCT TGATCCTCTG TACTACTAGC   
  
  
- GACCTTCTTC TCGTTGTTCT TCGTTACTGT AGTGACTTTC TTATTGCCAA TCAGGCCACT TAAAAGTAAT   
  
  
- CGACCTTCAA TGACGATCAT CAATTAATCA ATCTTCTCGA CAACTCAATC AATCATCAAT CATCAATTAA   
  
  
- TCAATCTTTT CGAGAACTCA ACCAATCATC AATCGTCAAC TTATTTCCCC AATCTAGTTA ACATTAATCC   
  
  
- TTTTTTGGTA ACATATATTT GTCCACATAC TTTTGCCCAA CTCCTATTCT TTTATGTCTT CTAATTAGAG   
  
  
- TAAAGAGACG AGTAAGAAGA GACGAGAGAG AACAAAGACA AGAAAGAGTT AAAGACAAGA GAAAAGAAAG   
  
  
- ATTAGAACAT TTCGACTCGT ATAGTAGTGG TTCAGTAGTG CACATCTTCG TTTCCCTGTT GTTCAGTAGT   
  
  
- GGCTTCTTCT TATATCAACT GGAAGCTCTT GGGTCAGTT

+     TGACG-motif

| Site Name | Organism | Position | Strand | Matrix score. | sequence | function |
| --- | --- | --- | --- | --- | --- | --- |
| TGACG-motif | Hordeum vulgare | 749 | + | 5 | TGACG | cis-acting regulatory element involved in the MeJA-responsiveness |
| TGACG-motif | Hordeum vulgare | 716 | - | 5 | TGACG | cis-acting regulatory element involved in the MeJA-responsiveness |

>Potri.018G038100.1   
+ GTTGCTGTCT TTTTTTTTTA TCATGTTATC AAATTAATGG AAATTTAACT CTATTATCAA AATTCTCTTT   
  
  
+ TTTATAAACA TGATAATCTT TTTTATTTTA TTTTCAAGTT AAAAATACTT GGTCCGGCCT GGGACGATGC   
  
  
+ GCTTGCCAAT CTAGTCAATC CTAAATTTAC TAATACGAGT AAGTAATAGC CGAAGGAGCA AAGCTCCAGT   
  
  
+ CCGGCATCTT GTTCAGAACA ATATTACATC CCTGAAATGC CTCGTGAATT GGGTGTCTCT TCCAACTTTC   
  
  
+ TCCAGCAATT TTCTCCCAAC TTCTGCAAGC AGTGATTATC AATTTGAATT ACAGGAGCTA CCTAACATAT   
  
  
+ CCGCCCTAGA ACGAAGGCAG GAATCATCAG CTTTGCCACC TCTTTGCATA ACTGCTACTG AAAGCCATTA   
  
  
+ GCTGGTGAAT GCTCCATTTC AAACTTCCAT TGGCCCGAGT ACTGCTCCTG CTTCCATTGG AAATATCGCT   
  
  
+ GCGCAATCAG ACGAGGCAGG CATTCATATC TGATGATAAA TGTTGCAGGA TTAATTCTAT TTTCGCCGGC   
  
  
+ AGCAAATGGT GGGATTTTTT ACACTGAAAA GTGTTTCAAA AGACTCGAAT TAATGTGGTC GATTTTGGCG   
  
  
+ GGGGCGTGGA ATGGATTCCT CTCTTCCTCA ATCATCAAAC ATCCAGATAC CTGAAACAGA GAGAAGCCCA   
  
  
+ TCTTCAAAAT CATATCGTCA TCGGAGGTCC TTCGCTGCAA TCGGCTGATG ACGGTGCACT TTTTGATGGT   
  
  
+ AGCGAACCAG ATCAGTCAAT GGTCAAATCA TTTGGAAATC AACGAGCTAC AGTTTATCCC TCCTGAATCC   
  
  
+ ATCTTTGAGA ACCTAAAAGA AATTAATTAA AATTAACGAA AGAGAAAAGA GAGAGGTGGT AGAGTTCATA   
  
  
+ GTTGTCGGAT CGGTCCGGAA ATTGATTTAA TTTAGAAATT AATATAGAAA AGAGTTTTGA GTAATTATAT   
  
  
+ TATCTTTAAC GGAGGCAAAC TAAATTAATA TTTTTATTAA AAAAATAATT TTTTTAAAAA AAACTTTTTA   
  
  
+ GACTTTTGTA TAAATTAATC AAATTAAATT AACAAGATTT TACTAAATTA ATAATTTATT AAATTTAACT   
  
  
+ TAAAATCTAA TTTAATATCA CAGCCTGCTT GATATTATAA TATCGGTTGT TTCTGGTTTA AAAATATATT   
  
  
+ AAAATATTAT TTTTTAATTT TTTAAAATTT AATTTTAACA TTAATAAATT AAAATGATCT AATTTTAATT   
  
  
+ TTTTTAAAAA AACTAAGATT CTTTTCAAAA ACATCTTTTT ACGGTGGCTT AGACTCTATT CGTTTTTATA   
  
  
+ TTTTAAAAAT TCTTTTAAAA AAATTAAAAA TTTTTAATTT TTTTCACTTT AATTTTTTTT ATATTTTTAA   
  
  
+ ATTATTTTAA TATGTTAATA TTAAAAATAA AATTTTAAAA TAAAAAAATA TTATTTTAAT AAATTTTAAA   
  
  
+ ATTAAAAATA TTTTAAAAAC CAACCAAAAA AGGCAACCTA TTACGAACGA ACTAGGAGAC ATGATGATCG   
  
  
+ CTGGAAGAAG AGCAACAAGA AGCAATGACA TCACTGAAAG AATAACGGTT AGTCCGGTGA ATTTTCATTA   
  
  
+ GCTGGAAGTT ACTGCTAGTA GTTAATTAGT TAGAAGAGCT GTTGAGTTAG TTAGTAGTTA GTAGTTAATT   
  
  
+ AGTTAGAAAA GCTCTTGAGT TGGTTAGTAG TTAGCAGTTG AATAAAGGGG TTAGATCAAT TGTAATTAGG   
  
  
+ AAAAAACCAT TGTATATAAA CAGGTGTATG AAAACGGGTT GAGGATAAGA AAATACAGAA GATTAATCTC   
  
  
+ ATTTCTCTGC TCATTCTTCT CTGCTCTCTC TTGTTTCTGT TCTTTCTCAA TTTCTGTTCT CTTTTCTTTC   
  
  
+ TAATCTTGTA AAGCTGAGCA TATCATCACC AAGTCATCAC GTGTAGAAGC AAAGGGACAA CAAGTCATCA   
  
  
+ CCGAAGAAGA ATATAGTTGA CCTTCGAGAA CCCAGTCAA  

- CAACGACAGA AAAAAAAAAT AGTACAATAG TTTAATTACC TTTAAATTGA GATAATAGTT TTAAGAGAAA   
  
  
- AAATATTTGT ACTATTAGAA AAAATAAAAT AAAAGTTCAA TTTTTATGAA CCAGGCCGGA CCCTGCTACG   
  
  
- CGAACGGTTA GATCAGTTAG GATTTAAATG ATTATGCTCA TTCATTATCG GCTTCCTCGT TTCGAGGTCA   
  
  
- GGCCGTAGAA CAAGTCTTGT TATAATGTAG GGACTTTACG GAGCACTTAA CCCACAGAGA AGGTTGAAAG   
  
  
- AGGTCGTTAA AAGAGGGTTG AAGACGTTCG TCACTAATAG TTAAACTTAA TGTCCTCGAT GGATTGTATA   
  
  
- GGCGGGATCT TGCTTCCGTC CTTAGTAGTC GAAACGGTGG AGAAACGTAT TGACGATGAC TTTCGGTAAT   
  
  
- CGACCACTTA CGAGGTAAAG TTTGAAGGTA ACCGGGCTCA TGACGAGGAC GAAGGTAACC TTTATAGCGA   
  
  
- CGCGTTAGTC TGCTCCGTCC GTAAGTATAG ACTACTATTT ACAACGTCCT AATTAAGATA AAAGCGGCCG   
  
  
- TCGTTTACCA CCCTAAAAAA TGTGACTTTT CACAAAGTTT TCTGAGCTTA ATTACACCAG CTAAAACCGC   
  
  
- CCCCGCACCT TACCTAAGGA GAGAAGGAGT TAGTAGTTTG TAGGTCTATG GACTTTGTCT CTCTTCGGGT   
  
  
- AGAAGTTTTA GTATAGCAGT AGCCTCCAGG AAGCGACGTT AGCCGACTAC TGCCACGTGA AAAACTACCA   
  
  
- TCGCTTGGTC TAGTCAGTTA CCAGTTTAGT AAACCTTTAG TTGCTCGATG TCAAATAGGG AGGACTTAGG   
  
  
- TAGAAACTCT TGGATTTTCT TTAATTAATT TTAATTGCTT TCTCTTTTCT CTCTCCACCA TCTCAAGTAT   
  
  
- CAACAGCCTA GCCAGGCCTT TAACTAAATT AAATCTTTAA TTATATCTTT TCTCAAAACT CATTAATATA   
  
  
- ATAGAAATTG CCTCCGTTTG ATTTAATTAT AAAAATAATT TTTTTATTAA AAAAATTTTT TTTGAAAAAT   
  
  
- CTGAAAACAT ATTTAATTAG TTTAATTTAA TTGTTCTAAA ATGATTTAAT TATTAAATAA TTTAAATTGA   
  
  
- ATTTTAGATT AAATTATAGT GTCGGACGAA CTATAATATT ATAGCCAACA AAGACCAAAT TTTTATATAA   
  
  
- TTTTATAATA AAAAATTAAA AAATTTTAAA TTAAAATTGT AATTATTTAA TTTTACTAGA TTAAAATTAA   
  
  
- AAAAATTTTT TTGATTCTAA GAAAAGTTTT TGTAGAAAAA TGCCACCGAA TCTGAGATAA GCAAAAATAT   
  
  
- AAAATTTTTA AGAAAATTTT TTTAATTTTT AAAAATTAAA AAAAGTGAAA TTAAAAAAAA TATAAAAATT   
  
  
- TAATAAAATT ATACAATTAT AATTTTTATT TTAAAATTTT ATTTTTTTAT AATAAAATTA TTTAAAATTT   
  
  
- TAATTTTTAT AAAATTTTTG GTTGGTTTTT TCCGTTGGAT AATGCTTGCT TGATCCTCTG TACTACTAGC   
  
  
- GACCTTCTTC TCGTTGTTCT TCGTTACTGT AGTGACTTTC TTATTGCCAA TCAGGCCACT TAAAAGTAAT   
  
  
- CGACCTTCAA TGACGATCAT CAATTAATCA ATCTTCTCGA CAACTCAATC AATCATCAAT CATCAATTAA   
  
  
- TCAATCTTTT CGAGAACTCA ACCAATCATC AATCGTCAAC TTATTTCCCC AATCTAGTTA ACATTAATCC   
  
  
- TTTTTTGGTA ACATATATTT GTCCACATAC TTTTGCCCAA CTCCTATTCT TTTATGTCTT CTAATTAGAG   
  
  
- TAAAGAGACG AGTAAGAAGA GACGAGAGAG AACAAAGACA AGAAAGAGTT AAAGACAAGA GAAAAGAAAG   
  
  
- ATTAGAACAT TTCGACTCGT ATAGTAGTGG TTCAGTAGTG CACATCTTCG TTTCCCTGTT GTTCAGTAGT   
  
  
- GGCTTCTTCT TATATCAACT GGAAGCTCTT GGGTCAGTT

+     Unnamed\_\_1

| Site Name | Organism | Position | Strand | Matrix score. | sequence | function |
| --- | --- | --- | --- | --- | --- | --- |
| Unnamed\_\_1 | Glycine max | 859 | + | 11 | GAATTTAATTAA | 60K protein binding site |
| Unnamed\_\_1 | Zea mays | 635 | + | 5 | CGTGG |  |

>Potri.018G038100.1   
+ GTTGCTGTCT TTTTTTTTTA TCATGTTATC AAATTAATGG AAATTTAACT CTATTATCAA AATTCTCTTT   
  
  
+ TTTATAAACA TGATAATCTT TTTTATTTTA TTTTCAAGTT AAAAATACTT GGTCCGGCCT GGGACGATGC   
  
  
+ GCTTGCCAAT CTAGTCAATC CTAAATTTAC TAATACGAGT AAGTAATAGC CGAAGGAGCA AAGCTCCAGT   
  
  
+ CCGGCATCTT GTTCAGAACA ATATTACATC CCTGAAATGC CTCGTGAATT GGGTGTCTCT TCCAACTTTC   
  
  
+ TCCAGCAATT TTCTCCCAAC TTCTGCAAGC AGTGATTATC AATTTGAATT ACAGGAGCTA CCTAACATAT   
  
  
+ CCGCCCTAGA ACGAAGGCAG GAATCATCAG CTTTGCCACC TCTTTGCATA ACTGCTACTG AAAGCCATTA   
  
  
+ GCTGGTGAAT GCTCCATTTC AAACTTCCAT TGGCCCGAGT ACTGCTCCTG CTTCCATTGG AAATATCGCT   
  
  
+ GCGCAATCAG ACGAGGCAGG CATTCATATC TGATGATAAA TGTTGCAGGA TTAATTCTAT TTTCGCCGGC   
  
  
+ AGCAAATGGT GGGATTTTTT ACACTGAAAA GTGTTTCAAA AGACTCGAAT TAATGTGGTC GATTTTGGCG   
  
  
+ GGGGCGTGGA ATGGATTCCT CTCTTCCTCA ATCATCAAAC ATCCAGATAC CTGAAACAGA GAGAAGCCCA   
  
  
+ TCTTCAAAAT CATATCGTCA TCGGAGGTCC TTCGCTGCAA TCGGCTGATG ACGGTGCACT TTTTGATGGT   
  
  
+ AGCGAACCAG ATCAGTCAAT GGTCAAATCA TTTGGAAATC AACGAGCTAC AGTTTATCCC TCCTGAATCC   
  
  
+ ATCTTTGAGA ACCTAAAAGA AATTAATTAA AATTAACGAA AGAGAAAAGA GAGAGGTGGT AGAGTTCATA   
  
  
+ GTTGTCGGAT CGGTCCGGAA ATTGATTTAA TTTAGAAATT AATATAGAAA AGAGTTTTGA GTAATTATAT   
  
  
+ TATCTTTAAC GGAGGCAAAC TAAATTAATA TTTTTATTAA AAAAATAATT TTTTTAAAAA AAACTTTTTA   
  
  
+ GACTTTTGTA TAAATTAATC AAATTAAATT AACAAGATTT TACTAAATTA ATAATTTATT AAATTTAACT   
  
  
+ TAAAATCTAA TTTAATATCA CAGCCTGCTT GATATTATAA TATCGGTTGT TTCTGGTTTA AAAATATATT   
  
  
+ AAAATATTAT TTTTTAATTT TTTAAAATTT AATTTTAACA TTAATAAATT AAAATGATCT AATTTTAATT   
  
  
+ TTTTTAAAAA AACTAAGATT CTTTTCAAAA ACATCTTTTT ACGGTGGCTT AGACTCTATT CGTTTTTATA   
  
  
+ TTTTAAAAAT TCTTTTAAAA AAATTAAAAA TTTTTAATTT TTTTCACTTT AATTTTTTTT ATATTTTTAA   
  
  
+ ATTATTTTAA TATGTTAATA TTAAAAATAA AATTTTAAAA TAAAAAAATA TTATTTTAAT AAATTTTAAA   
  
  
+ ATTAAAAATA TTTTAAAAAC CAACCAAAAA AGGCAACCTA TTACGAACGA ACTAGGAGAC ATGATGATCG   
  
  
+ CTGGAAGAAG AGCAACAAGA AGCAATGACA TCACTGAAAG AATAACGGTT AGTCCGGTGA ATTTTCATTA   
  
  
+ GCTGGAAGTT ACTGCTAGTA GTTAATTAGT TAGAAGAGCT GTTGAGTTAG TTAGTAGTTA GTAGTTAATT   
  
  
+ AGTTAGAAAA GCTCTTGAGT TGGTTAGTAG TTAGCAGTTG AATAAAGGGG TTAGATCAAT TGTAATTAGG   
  
  
+ AAAAAACCAT TGTATATAAA CAGGTGTATG AAAACGGGTT GAGGATAAGA AAATACAGAA GATTAATCTC   
  
  
+ ATTTCTCTGC TCATTCTTCT CTGCTCTCTC TTGTTTCTGT TCTTTCTCAA TTTCTGTTCT CTTTTCTTTC   
  
  
+ TAATCTTGTA AAGCTGAGCA TATCATCACC AAGTCATCAC GTGTAGAAGC AAAGGGACAA CAAGTCATCA   
  
  
+ CCGAAGAAGA ATATAGTTGA CCTTCGAGAA CCCAGTCAA  

- CAACGACAGA AAAAAAAAAT AGTACAATAG TTTAATTACC TTTAAATTGA GATAATAGTT TTAAGAGAAA   
  
  
- AAATATTTGT ACTATTAGAA AAAATAAAAT AAAAGTTCAA TTTTTATGAA CCAGGCCGGA CCCTGCTACG   
  
  
- CGAACGGTTA GATCAGTTAG GATTTAAATG ATTATGCTCA TTCATTATCG GCTTCCTCGT TTCGAGGTCA   
  
  
- GGCCGTAGAA CAAGTCTTGT TATAATGTAG GGACTTTACG GAGCACTTAA CCCACAGAGA AGGTTGAAAG   
  
  
- AGGTCGTTAA AAGAGGGTTG AAGACGTTCG TCACTAATAG TTAAACTTAA TGTCCTCGAT GGATTGTATA   
  
  
- GGCGGGATCT TGCTTCCGTC CTTAGTAGTC GAAACGGTGG AGAAACGTAT TGACGATGAC TTTCGGTAAT   
  
  
- CGACCACTTA CGAGGTAAAG TTTGAAGGTA ACCGGGCTCA TGACGAGGAC GAAGGTAACC TTTATAGCGA   
  
  
- CGCGTTAGTC TGCTCCGTCC GTAAGTATAG ACTACTATTT ACAACGTCCT AATTAAGATA AAAGCGGCCG   
  
  
- TCGTTTACCA CCCTAAAAAA TGTGACTTTT CACAAAGTTT TCTGAGCTTA ATTACACCAG CTAAAACCGC   
  
  
- CCCCGCACCT TACCTAAGGA GAGAAGGAGT TAGTAGTTTG TAGGTCTATG GACTTTGTCT CTCTTCGGGT   
  
  
- AGAAGTTTTA GTATAGCAGT AGCCTCCAGG AAGCGACGTT AGCCGACTAC TGCCACGTGA AAAACTACCA   
  
  
- TCGCTTGGTC TAGTCAGTTA CCAGTTTAGT AAACCTTTAG TTGCTCGATG TCAAATAGGG AGGACTTAGG   
  
  
- TAGAAACTCT TGGATTTTCT TTAATTAATT TTAATTGCTT TCTCTTTTCT CTCTCCACCA TCTCAAGTAT   
  
  
- CAACAGCCTA GCCAGGCCTT TAACTAAATT AAATCTTTAA TTATATCTTT TCTCAAAACT CATTAATATA   
  
  
- ATAGAAATTG CCTCCGTTTG ATTTAATTAT AAAAATAATT TTTTTATTAA AAAAATTTTT TTTGAAAAAT   
  
  
- CTGAAAACAT ATTTAATTAG TTTAATTTAA TTGTTCTAAA ATGATTTAAT TATTAAATAA TTTAAATTGA   
  
  
- ATTTTAGATT AAATTATAGT GTCGGACGAA CTATAATATT ATAGCCAACA AAGACCAAAT TTTTATATAA   
  
  
- TTTTATAATA AAAAATTAAA AAATTTTAAA TTAAAATTGT AATTATTTAA TTTTACTAGA TTAAAATTAA   
  
  
- AAAAATTTTT TTGATTCTAA GAAAAGTTTT TGTAGAAAAA TGCCACCGAA TCTGAGATAA GCAAAAATAT   
  
  
- AAAATTTTTA AGAAAATTTT TTTAATTTTT AAAAATTAAA AAAAGTGAAA TTAAAAAAAA TATAAAAATT   
  
  
- TAATAAAATT ATACAATTAT AATTTTTATT TTAAAATTTT ATTTTTTTAT AATAAAATTA TTTAAAATTT   
  
  
- TAATTTTTAT AAAATTTTTG GTTGGTTTTT TCCGTTGGAT AATGCTTGCT TGATCCTCTG TACTACTAGC   
  
  
- GACCTTCTTC TCGTTGTTCT TCGTTACTGT AGTGACTTTC TTATTGCCAA TCAGGCCACT TAAAAGTAAT   
  
  
- CGACCTTCAA TGACGATCAT CAATTAATCA ATCTTCTCGA CAACTCAATC AATCATCAAT CATCAATTAA   
  
  
- TCAATCTTTT CGAGAACTCA ACCAATCATC AATCGTCAAC TTATTTCCCC AATCTAGTTA ACATTAATCC   
  
  
- TTTTTTGGTA ACATATATTT GTCCACATAC TTTTGCCCAA CTCCTATTCT TTTATGTCTT CTAATTAGAG   
  
  
- TAAAGAGACG AGTAAGAAGA GACGAGAGAG AACAAAGACA AGAAAGAGTT AAAGACAAGA GAAAAGAAAG   
  
  
- ATTAGAACAT TTCGACTCGT ATAGTAGTGG TTCAGTAGTG CACATCTTCG TTTCCCTGTT GTTCAGTAGT   
  
  
- GGCTTCTTCT TATATCAACT GGAAGCTCTT GGGTCAGTT

+     Unnamed\_\_4

| Site Name | Organism | Position | Strand | Matrix score. | sequence | function |
| --- | --- | --- | --- | --- | --- | --- |
| Unnamed\_\_4 | Petroselinum hortense | 432 | + | 4 | CTCC |  |
| Unnamed\_\_4 | Petroselinum hortense | 334 | - | 4 | CTCC |  |
| Unnamed\_\_4 | Petroselinum hortense | 204 | + | 4 | CTCC |  |
| Unnamed\_\_4 | Petroselinum hortense | 1525 | - | 4 | CTCC |  |
| Unnamed\_\_4 | Petroselinum hortense | 830 | + | 4 | CTCC |  |
| Unnamed\_\_4 | Petroselinum hortense | 465 | + | 4 | CTCC |  |
| Unnamed\_\_4 | Petroselinum hortense | 280 | + | 4 | CTCC |  |
| Unnamed\_\_4 | Petroselinum hortense | 195 | - | 4 | CTCC |  |
| Unnamed\_\_4 | Petroselinum hortense | 723 | - | 4 | CTCC |  |
| Unnamed\_\_4 | Petroselinum hortense | 293 | + | 4 | CTCC |  |
| Unnamed\_\_4 | Petroselinum hortense | 991 | - | 4 | CTCC |  |

>Potri.018G038100.1   
+ GTTGCTGTCT TTTTTTTTTA TCATGTTATC AAATTAATGG AAATTTAACT CTATTATCAA AATTCTCTTT   
  
  
+ TTTATAAACA TGATAATCTT TTTTATTTTA TTTTCAAGTT AAAAATACTT GGTCCGGCCT GGGACGATGC   
  
  
+ GCTTGCCAAT CTAGTCAATC CTAAATTTAC TAATACGAGT AAGTAATAGC CGAAGGAGCA AAGCTCCAGT   
  
  
+ CCGGCATCTT GTTCAGAACA ATATTACATC CCTGAAATGC CTCGTGAATT GGGTGTCTCT TCCAACTTTC   
  
  
+ TCCAGCAATT TTCTCCCAAC TTCTGCAAGC AGTGATTATC AATTTGAATT ACAGGAGCTA CCTAACATAT   
  
  
+ CCGCCCTAGA ACGAAGGCAG GAATCATCAG CTTTGCCACC TCTTTGCATA ACTGCTACTG AAAGCCATTA   
  
  
+ GCTGGTGAAT GCTCCATTTC AAACTTCCAT TGGCCCGAGT ACTGCTCCTG CTTCCATTGG AAATATCGCT   
  
  
+ GCGCAATCAG ACGAGGCAGG CATTCATATC TGATGATAAA TGTTGCAGGA TTAATTCTAT TTTCGCCGGC   
  
  
+ AGCAAATGGT GGGATTTTTT ACACTGAAAA GTGTTTCAAA AGACTCGAAT TAATGTGGTC GATTTTGGCG   
  
  
+ GGGGCGTGGA ATGGATTCCT CTCTTCCTCA ATCATCAAAC ATCCAGATAC CTGAAACAGA GAGAAGCCCA   
  
  
+ TCTTCAAAAT CATATCGTCA TCGGAGGTCC TTCGCTGCAA TCGGCTGATG ACGGTGCACT TTTTGATGGT   
  
  
+ AGCGAACCAG ATCAGTCAAT GGTCAAATCA TTTGGAAATC AACGAGCTAC AGTTTATCCC TCCTGAATCC   
  
  
+ ATCTTTGAGA ACCTAAAAGA AATTAATTAA AATTAACGAA AGAGAAAAGA GAGAGGTGGT AGAGTTCATA   
  
  
+ GTTGTCGGAT CGGTCCGGAA ATTGATTTAA TTTAGAAATT AATATAGAAA AGAGTTTTGA GTAATTATAT   
  
  
+ TATCTTTAAC GGAGGCAAAC TAAATTAATA TTTTTATTAA AAAAATAATT TTTTTAAAAA AAACTTTTTA   
  
  
+ GACTTTTGTA TAAATTAATC AAATTAAATT AACAAGATTT TACTAAATTA ATAATTTATT AAATTTAACT   
  
  
+ TAAAATCTAA TTTAATATCA CAGCCTGCTT GATATTATAA TATCGGTTGT TTCTGGTTTA AAAATATATT   
  
  
+ AAAATATTAT TTTTTAATTT TTTAAAATTT AATTTTAACA TTAATAAATT AAAATGATCT AATTTTAATT   
  
  
+ TTTTTAAAAA AACTAAGATT CTTTTCAAAA ACATCTTTTT ACGGTGGCTT AGACTCTATT CGTTTTTATA   
  
  
+ TTTTAAAAAT TCTTTTAAAA AAATTAAAAA TTTTTAATTT TTTTCACTTT AATTTTTTTT ATATTTTTAA   
  
  
+ ATTATTTTAA TATGTTAATA TTAAAAATAA AATTTTAAAA TAAAAAAATA TTATTTTAAT AAATTTTAAA   
  
  
+ ATTAAAAATA TTTTAAAAAC CAACCAAAAA AGGCAACCTA TTACGAACGA ACTAGGAGAC ATGATGATCG   
  
  
+ CTGGAAGAAG AGCAACAAGA AGCAATGACA TCACTGAAAG AATAACGGTT AGTCCGGTGA ATTTTCATTA   
  
  
+ GCTGGAAGTT ACTGCTAGTA GTTAATTAGT TAGAAGAGCT GTTGAGTTAG TTAGTAGTTA GTAGTTAATT   
  
  
+ AGTTAGAAAA GCTCTTGAGT TGGTTAGTAG TTAGCAGTTG AATAAAGGGG TTAGATCAAT TGTAATTAGG   
  
  
+ AAAAAACCAT TGTATATAAA CAGGTGTATG AAAACGGGTT GAGGATAAGA AAATACAGAA GATTAATCTC   
  
  
+ ATTTCTCTGC TCATTCTTCT CTGCTCTCTC TTGTTTCTGT TCTTTCTCAA TTTCTGTTCT CTTTTCTTTC   
  
  
+ TAATCTTGTA AAGCTGAGCA TATCATCACC AAGTCATCAC GTGTAGAAGC AAAGGGACAA CAAGTCATCA   
  
  
+ CCGAAGAAGA ATATAGTTGA CCTTCGAGAA CCCAGTCAA  

- CAACGACAGA AAAAAAAAAT AGTACAATAG TTTAATTACC TTTAAATTGA GATAATAGTT TTAAGAGAAA   
  
  
- AAATATTTGT ACTATTAGAA AAAATAAAAT AAAAGTTCAA TTTTTATGAA CCAGGCCGGA CCCTGCTACG   
  
  
- CGAACGGTTA GATCAGTTAG GATTTAAATG ATTATGCTCA TTCATTATCG GCTTCCTCGT TTCGAGGTCA   
  
  
- GGCCGTAGAA CAAGTCTTGT TATAATGTAG GGACTTTACG GAGCACTTAA CCCACAGAGA AGGTTGAAAG   
  
  
- AGGTCGTTAA AAGAGGGTTG AAGACGTTCG TCACTAATAG TTAAACTTAA TGTCCTCGAT GGATTGTATA   
  
  
- GGCGGGATCT TGCTTCCGTC CTTAGTAGTC GAAACGGTGG AGAAACGTAT TGACGATGAC TTTCGGTAAT   
  
  
- CGACCACTTA CGAGGTAAAG TTTGAAGGTA ACCGGGCTCA TGACGAGGAC GAAGGTAACC TTTATAGCGA   
  
  
- CGCGTTAGTC TGCTCCGTCC GTAAGTATAG ACTACTATTT ACAACGTCCT AATTAAGATA AAAGCGGCCG   
  
  
- TCGTTTACCA CCCTAAAAAA TGTGACTTTT CACAAAGTTT TCTGAGCTTA ATTACACCAG CTAAAACCGC   
  
  
- CCCCGCACCT TACCTAAGGA GAGAAGGAGT TAGTAGTTTG TAGGTCTATG GACTTTGTCT CTCTTCGGGT   
  
  
- AGAAGTTTTA GTATAGCAGT AGCCTCCAGG AAGCGACGTT AGCCGACTAC TGCCACGTGA AAAACTACCA   
  
  
- TCGCTTGGTC TAGTCAGTTA CCAGTTTAGT AAACCTTTAG TTGCTCGATG TCAAATAGGG AGGACTTAGG   
  
  
- TAGAAACTCT TGGATTTTCT TTAATTAATT TTAATTGCTT TCTCTTTTCT CTCTCCACCA TCTCAAGTAT   
  
  
- CAACAGCCTA GCCAGGCCTT TAACTAAATT AAATCTTTAA TTATATCTTT TCTCAAAACT CATTAATATA   
  
  
- ATAGAAATTG CCTCCGTTTG ATTTAATTAT AAAAATAATT TTTTTATTAA AAAAATTTTT TTTGAAAAAT   
  
  
- CTGAAAACAT ATTTAATTAG TTTAATTTAA TTGTTCTAAA ATGATTTAAT TATTAAATAA TTTAAATTGA   
  
  
- ATTTTAGATT AAATTATAGT GTCGGACGAA CTATAATATT ATAGCCAACA AAGACCAAAT TTTTATATAA   
  
  
- TTTTATAATA AAAAATTAAA AAATTTTAAA TTAAAATTGT AATTATTTAA TTTTACTAGA TTAAAATTAA   
  
  
- AAAAATTTTT TTGATTCTAA GAAAAGTTTT TGTAGAAAAA TGCCACCGAA TCTGAGATAA GCAAAAATAT   
  
  
- AAAATTTTTA AGAAAATTTT TTTAATTTTT AAAAATTAAA AAAAGTGAAA TTAAAAAAAA TATAAAAATT   
  
  
- TAATAAAATT ATACAATTAT AATTTTTATT TTAAAATTTT ATTTTTTTAT AATAAAATTA TTTAAAATTT   
  
  
- TAATTTTTAT AAAATTTTTG GTTGGTTTTT TCCGTTGGAT AATGCTTGCT TGATCCTCTG TACTACTAGC   
  
  
- GACCTTCTTC TCGTTGTTCT TCGTTACTGT AGTGACTTTC TTATTGCCAA TCAGGCCACT TAAAAGTAAT   
  
  
- CGACCTTCAA TGACGATCAT CAATTAATCA ATCTTCTCGA CAACTCAATC AATCATCAAT CATCAATTAA   
  
  
- TCAATCTTTT CGAGAACTCA ACCAATCATC AATCGTCAAC TTATTTCCCC AATCTAGTTA ACATTAATCC   
  
  
- TTTTTTGGTA ACATATATTT GTCCACATAC TTTTGCCCAA CTCCTATTCT TTTATGTCTT CTAATTAGAG   
  
  
- TAAAGAGACG AGTAAGAAGA GACGAGAGAG AACAAAGACA AGAAAGAGTT AAAGACAAGA GAAAAGAAAG   
  
  
- ATTAGAACAT TTCGACTCGT ATAGTAGTGG TTCAGTAGTG CACATCTTCG TTTCCCTGTT GTTCAGTAGT   
  
  
- GGCTTCTTCT TATATCAACT GGAAGCTCTT GGGTCAGTT

+     W box

| Site Name | Organism | Position | Strand | Matrix score. | sequence | function |
| --- | --- | --- | --- | --- | --- | --- |
| W box | Arabidopsis thaliana | 791 | - | 6 | TTGACC |  |
| W box | Arabidopsis thaliana | 1977 | + | 6 | TTGACC |  |

>Potri.018G038100.1   
+ GTTGCTGTCT TTTTTTTTTA TCATGTTATC AAATTAATGG AAATTTAACT CTATTATCAA AATTCTCTTT   
  
  
+ TTTATAAACA TGATAATCTT TTTTATTTTA TTTTCAAGTT AAAAATACTT GGTCCGGCCT GGGACGATGC   
  
  
+ GCTTGCCAAT CTAGTCAATC CTAAATTTAC TAATACGAGT AAGTAATAGC CGAAGGAGCA AAGCTCCAGT   
  
  
+ CCGGCATCTT GTTCAGAACA ATATTACATC CCTGAAATGC CTCGTGAATT GGGTGTCTCT TCCAACTTTC   
  
  
+ TCCAGCAATT TTCTCCCAAC TTCTGCAAGC AGTGATTATC AATTTGAATT ACAGGAGCTA CCTAACATAT   
  
  
+ CCGCCCTAGA ACGAAGGCAG GAATCATCAG CTTTGCCACC TCTTTGCATA ACTGCTACTG AAAGCCATTA   
  
  
+ GCTGGTGAAT GCTCCATTTC AAACTTCCAT TGGCCCGAGT ACTGCTCCTG CTTCCATTGG AAATATCGCT   
  
  
+ GCGCAATCAG ACGAGGCAGG CATTCATATC TGATGATAAA TGTTGCAGGA TTAATTCTAT TTTCGCCGGC   
  
  
+ AGCAAATGGT GGGATTTTTT ACACTGAAAA GTGTTTCAAA AGACTCGAAT TAATGTGGTC GATTTTGGCG   
  
  
+ GGGGCGTGGA ATGGATTCCT CTCTTCCTCA ATCATCAAAC ATCCAGATAC CTGAAACAGA GAGAAGCCCA   
  
  
+ TCTTCAAAAT CATATCGTCA TCGGAGGTCC TTCGCTGCAA TCGGCTGATG ACGGTGCACT TTTTGATGGT   
  
  
+ AGCGAACCAG ATCAGTCAAT GGTCAAATCA TTTGGAAATC AACGAGCTAC AGTTTATCCC TCCTGAATCC   
  
  
+ ATCTTTGAGA ACCTAAAAGA AATTAATTAA AATTAACGAA AGAGAAAAGA GAGAGGTGGT AGAGTTCATA   
  
  
+ GTTGTCGGAT CGGTCCGGAA ATTGATTTAA TTTAGAAATT AATATAGAAA AGAGTTTTGA GTAATTATAT   
  
  
+ TATCTTTAAC GGAGGCAAAC TAAATTAATA TTTTTATTAA AAAAATAATT TTTTTAAAAA AAACTTTTTA   
  
  
+ GACTTTTGTA TAAATTAATC AAATTAAATT AACAAGATTT TACTAAATTA ATAATTTATT AAATTTAACT   
  
  
+ TAAAATCTAA TTTAATATCA CAGCCTGCTT GATATTATAA TATCGGTTGT TTCTGGTTTA AAAATATATT   
  
  
+ AAAATATTAT TTTTTAATTT TTTAAAATTT AATTTTAACA TTAATAAATT AAAATGATCT AATTTTAATT   
  
  
+ TTTTTAAAAA AACTAAGATT CTTTTCAAAA ACATCTTTTT ACGGTGGCTT AGACTCTATT CGTTTTTATA   
  
  
+ TTTTAAAAAT TCTTTTAAAA AAATTAAAAA TTTTTAATTT TTTTCACTTT AATTTTTTTT ATATTTTTAA   
  
  
+ ATTATTTTAA TATGTTAATA TTAAAAATAA AATTTTAAAA TAAAAAAATA TTATTTTAAT AAATTTTAAA   
  
  
+ ATTAAAAATA TTTTAAAAAC CAACCAAAAA AGGCAACCTA TTACGAACGA ACTAGGAGAC ATGATGATCG   
  
  
+ CTGGAAGAAG AGCAACAAGA AGCAATGACA TCACTGAAAG AATAACGGTT AGTCCGGTGA ATTTTCATTA   
  
  
+ GCTGGAAGTT ACTGCTAGTA GTTAATTAGT TAGAAGAGCT GTTGAGTTAG TTAGTAGTTA GTAGTTAATT   
  
  
+ AGTTAGAAAA GCTCTTGAGT TGGTTAGTAG TTAGCAGTTG AATAAAGGGG TTAGATCAAT TGTAATTAGG   
  
  
+ AAAAAACCAT TGTATATAAA CAGGTGTATG AAAACGGGTT GAGGATAAGA AAATACAGAA GATTAATCTC   
  
  
+ ATTTCTCTGC TCATTCTTCT CTGCTCTCTC TTGTTTCTGT TCTTTCTCAA TTTCTGTTCT CTTTTCTTTC   
  
  
+ TAATCTTGTA AAGCTGAGCA TATCATCACC AAGTCATCAC GTGTAGAAGC AAAGGGACAA CAAGTCATCA   
  
  
+ CCGAAGAAGA ATATAGTTGA CCTTCGAGAA CCCAGTCAA  

- CAACGACAGA AAAAAAAAAT AGTACAATAG TTTAATTACC TTTAAATTGA GATAATAGTT TTAAGAGAAA   
  
  
- AAATATTTGT ACTATTAGAA AAAATAAAAT AAAAGTTCAA TTTTTATGAA CCAGGCCGGA CCCTGCTACG   
  
  
- CGAACGGTTA GATCAGTTAG GATTTAAATG ATTATGCTCA TTCATTATCG GCTTCCTCGT TTCGAGGTCA   
  
  
- GGCCGTAGAA CAAGTCTTGT TATAATGTAG GGACTTTACG GAGCACTTAA CCCACAGAGA AGGTTGAAAG   
  
  
- AGGTCGTTAA AAGAGGGTTG AAGACGTTCG TCACTAATAG TTAAACTTAA TGTCCTCGAT GGATTGTATA   
  
  
- GGCGGGATCT TGCTTCCGTC CTTAGTAGTC GAAACGGTGG AGAAACGTAT TGACGATGAC TTTCGGTAAT   
  
  
- CGACCACTTA CGAGGTAAAG TTTGAAGGTA ACCGGGCTCA TGACGAGGAC GAAGGTAACC TTTATAGCGA   
  
  
- CGCGTTAGTC TGCTCCGTCC GTAAGTATAG ACTACTATTT ACAACGTCCT AATTAAGATA AAAGCGGCCG   
  
  
- TCGTTTACCA CCCTAAAAAA TGTGACTTTT CACAAAGTTT TCTGAGCTTA ATTACACCAG CTAAAACCGC   
  
  
- CCCCGCACCT TACCTAAGGA GAGAAGGAGT TAGTAGTTTG TAGGTCTATG GACTTTGTCT CTCTTCGGGT   
  
  
- AGAAGTTTTA GTATAGCAGT AGCCTCCAGG AAGCGACGTT AGCCGACTAC TGCCACGTGA AAAACTACCA   
  
  
- TCGCTTGGTC TAGTCAGTTA CCAGTTTAGT AAACCTTTAG TTGCTCGATG TCAAATAGGG AGGACTTAGG   
  
  
- TAGAAACTCT TGGATTTTCT TTAATTAATT TTAATTGCTT TCTCTTTTCT CTCTCCACCA TCTCAAGTAT   
  
  
- CAACAGCCTA GCCAGGCCTT TAACTAAATT AAATCTTTAA TTATATCTTT TCTCAAAACT CATTAATATA   
  
  
- ATAGAAATTG CCTCCGTTTG ATTTAATTAT AAAAATAATT TTTTTATTAA AAAAATTTTT TTTGAAAAAT   
  
  
- CTGAAAACAT ATTTAATTAG TTTAATTTAA TTGTTCTAAA ATGATTTAAT TATTAAATAA TTTAAATTGA   
  
  
- ATTTTAGATT AAATTATAGT GTCGGACGAA CTATAATATT ATAGCCAACA AAGACCAAAT TTTTATATAA   
  
  
- TTTTATAATA AAAAATTAAA AAATTTTAAA TTAAAATTGT AATTATTTAA TTTTACTAGA TTAAAATTAA   
  
  
- AAAAATTTTT TTGATTCTAA GAAAAGTTTT TGTAGAAAAA TGCCACCGAA TCTGAGATAA GCAAAAATAT   
  
  
- AAAATTTTTA AGAAAATTTT TTTAATTTTT AAAAATTAAA AAAAGTGAAA TTAAAAAAAA TATAAAAATT   
  
  
- TAATAAAATT ATACAATTAT AATTTTTATT TTAAAATTTT ATTTTTTTAT AATAAAATTA TTTAAAATTT   
  
  
- TAATTTTTAT AAAATTTTTG GTTGGTTTTT TCCGTTGGAT AATGCTTGCT TGATCCTCTG TACTACTAGC   
  
  
- GACCTTCTTC TCGTTGTTCT TCGTTACTGT AGTGACTTTC TTATTGCCAA TCAGGCCACT TAAAAGTAAT   
  
  
- CGACCTTCAA TGACGATCAT CAATTAATCA ATCTTCTCGA CAACTCAATC AATCATCAAT CATCAATTAA   
  
  
- TCAATCTTTT CGAGAACTCA ACCAATCATC AATCGTCAAC TTATTTCCCC AATCTAGTTA ACATTAATCC   
  
  
- TTTTTTGGTA ACATATATTT GTCCACATAC TTTTGCCCAA CTCCTATTCT TTTATGTCTT CTAATTAGAG   
  
  
- TAAAGAGACG AGTAAGAAGA GACGAGAGAG AACAAAGACA AGAAAGAGTT AAAGACAAGA GAAAAGAAAG   
  
  
- ATTAGAACAT TTCGACTCGT ATAGTAGTGG TTCAGTAGTG CACATCTTCG TTTCCCTGTT GTTCAGTAGT   
  
  
- GGCTTCTTCT TATATCAACT GGAAGCTCTT GGGTCAGTT

+     WRE3

| Site Name | Organism | Position | Strand | Matrix score. | sequence | function |
| --- | --- | --- | --- | --- | --- | --- |
| WRE3 | Pisum sativum | 894 | - | 6 | CCACCT |  |
| WRE3 | Pisum sativum | 386 | + | 6 | CCACCT |  |

>Potri.018G038100.1   
+ GTTGCTGTCT TTTTTTTTTA TCATGTTATC AAATTAATGG AAATTTAACT CTATTATCAA AATTCTCTTT   
  
  
+ TTTATAAACA TGATAATCTT TTTTATTTTA TTTTCAAGTT AAAAATACTT GGTCCGGCCT GGGACGATGC   
  
  
+ GCTTGCCAAT CTAGTCAATC CTAAATTTAC TAATACGAGT AAGTAATAGC CGAAGGAGCA AAGCTCCAGT   
  
  
+ CCGGCATCTT GTTCAGAACA ATATTACATC CCTGAAATGC CTCGTGAATT GGGTGTCTCT TCCAACTTTC   
  
  
+ TCCAGCAATT TTCTCCCAAC TTCTGCAAGC AGTGATTATC AATTTGAATT ACAGGAGCTA CCTAACATAT   
  
  
+ CCGCCCTAGA ACGAAGGCAG GAATCATCAG CTTTGCCACC TCTTTGCATA ACTGCTACTG AAAGCCATTA   
  
  
+ GCTGGTGAAT GCTCCATTTC AAACTTCCAT TGGCCCGAGT ACTGCTCCTG CTTCCATTGG AAATATCGCT   
  
  
+ GCGCAATCAG ACGAGGCAGG CATTCATATC TGATGATAAA TGTTGCAGGA TTAATTCTAT TTTCGCCGGC   
  
  
+ AGCAAATGGT GGGATTTTTT ACACTGAAAA GTGTTTCAAA AGACTCGAAT TAATGTGGTC GATTTTGGCG   
  
  
+ GGGGCGTGGA ATGGATTCCT CTCTTCCTCA ATCATCAAAC ATCCAGATAC CTGAAACAGA GAGAAGCCCA   
  
  
+ TCTTCAAAAT CATATCGTCA TCGGAGGTCC TTCGCTGCAA TCGGCTGATG ACGGTGCACT TTTTGATGGT   
  
  
+ AGCGAACCAG ATCAGTCAAT GGTCAAATCA TTTGGAAATC AACGAGCTAC AGTTTATCCC TCCTGAATCC   
  
  
+ ATCTTTGAGA ACCTAAAAGA AATTAATTAA AATTAACGAA AGAGAAAAGA GAGAGGTGGT AGAGTTCATA   
  
  
+ GTTGTCGGAT CGGTCCGGAA ATTGATTTAA TTTAGAAATT AATATAGAAA AGAGTTTTGA GTAATTATAT   
  
  
+ TATCTTTAAC GGAGGCAAAC TAAATTAATA TTTTTATTAA AAAAATAATT TTTTTAAAAA AAACTTTTTA   
  
  
+ GACTTTTGTA TAAATTAATC AAATTAAATT AACAAGATTT TACTAAATTA ATAATTTATT AAATTTAACT   
  
  
+ TAAAATCTAA TTTAATATCA CAGCCTGCTT GATATTATAA TATCGGTTGT TTCTGGTTTA AAAATATATT   
  
  
+ AAAATATTAT TTTTTAATTT TTTAAAATTT AATTTTAACA TTAATAAATT AAAATGATCT AATTTTAATT   
  
  
+ TTTTTAAAAA AACTAAGATT CTTTTCAAAA ACATCTTTTT ACGGTGGCTT AGACTCTATT CGTTTTTATA   
  
  
+ TTTTAAAAAT TCTTTTAAAA AAATTAAAAA TTTTTAATTT TTTTCACTTT AATTTTTTTT ATATTTTTAA   
  
  
+ ATTATTTTAA TATGTTAATA TTAAAAATAA AATTTTAAAA TAAAAAAATA TTATTTTAAT AAATTTTAAA   
  
  
+ ATTAAAAATA TTTTAAAAAC CAACCAAAAA AGGCAACCTA TTACGAACGA ACTAGGAGAC ATGATGATCG   
  
  
+ CTGGAAGAAG AGCAACAAGA AGCAATGACA TCACTGAAAG AATAACGGTT AGTCCGGTGA ATTTTCATTA   
  
  
+ GCTGGAAGTT ACTGCTAGTA GTTAATTAGT TAGAAGAGCT GTTGAGTTAG TTAGTAGTTA GTAGTTAATT   
  
  
+ AGTTAGAAAA GCTCTTGAGT TGGTTAGTAG TTAGCAGTTG AATAAAGGGG TTAGATCAAT TGTAATTAGG   
  
  
+ AAAAAACCAT TGTATATAAA CAGGTGTATG AAAACGGGTT GAGGATAAGA AAATACAGAA GATTAATCTC   
  
  
+ ATTTCTCTGC TCATTCTTCT CTGCTCTCTC TTGTTTCTGT TCTTTCTCAA TTTCTGTTCT CTTTTCTTTC   
  
  
+ TAATCTTGTA AAGCTGAGCA TATCATCACC AAGTCATCAC GTGTAGAAGC AAAGGGACAA CAAGTCATCA   
  
  
+ CCGAAGAAGA ATATAGTTGA CCTTCGAGAA CCCAGTCAA  

- CAACGACAGA AAAAAAAAAT AGTACAATAG TTTAATTACC TTTAAATTGA GATAATAGTT TTAAGAGAAA   
  
  
- AAATATTTGT ACTATTAGAA AAAATAAAAT AAAAGTTCAA TTTTTATGAA CCAGGCCGGA CCCTGCTACG   
  
  
- CGAACGGTTA GATCAGTTAG GATTTAAATG ATTATGCTCA TTCATTATCG GCTTCCTCGT TTCGAGGTCA   
  
  
- GGCCGTAGAA CAAGTCTTGT TATAATGTAG GGACTTTACG GAGCACTTAA CCCACAGAGA AGGTTGAAAG   
  
  
- AGGTCGTTAA AAGAGGGTTG AAGACGTTCG TCACTAATAG TTAAACTTAA TGTCCTCGAT GGATTGTATA   
  
  
- GGCGGGATCT TGCTTCCGTC CTTAGTAGTC GAAACGGTGG AGAAACGTAT TGACGATGAC TTTCGGTAAT   
  
  
- CGACCACTTA CGAGGTAAAG TTTGAAGGTA ACCGGGCTCA TGACGAGGAC GAAGGTAACC TTTATAGCGA   
  
  
- CGCGTTAGTC TGCTCCGTCC GTAAGTATAG ACTACTATTT ACAACGTCCT AATTAAGATA AAAGCGGCCG   
  
  
- TCGTTTACCA CCCTAAAAAA TGTGACTTTT CACAAAGTTT TCTGAGCTTA ATTACACCAG CTAAAACCGC   
  
  
- CCCCGCACCT TACCTAAGGA GAGAAGGAGT TAGTAGTTTG TAGGTCTATG GACTTTGTCT CTCTTCGGGT   
  
  
- AGAAGTTTTA GTATAGCAGT AGCCTCCAGG AAGCGACGTT AGCCGACTAC TGCCACGTGA AAAACTACCA   
  
  
- TCGCTTGGTC TAGTCAGTTA CCAGTTTAGT AAACCTTTAG TTGCTCGATG TCAAATAGGG AGGACTTAGG   
  
  
- TAGAAACTCT TGGATTTTCT TTAATTAATT TTAATTGCTT TCTCTTTTCT CTCTCCACCA TCTCAAGTAT   
  
  
- CAACAGCCTA GCCAGGCCTT TAACTAAATT AAATCTTTAA TTATATCTTT TCTCAAAACT CATTAATATA   
  
  
- ATAGAAATTG CCTCCGTTTG ATTTAATTAT AAAAATAATT TTTTTATTAA AAAAATTTTT TTTGAAAAAT   
  
  
- CTGAAAACAT ATTTAATTAG TTTAATTTAA TTGTTCTAAA ATGATTTAAT TATTAAATAA TTTAAATTGA   
  
  
- ATTTTAGATT AAATTATAGT GTCGGACGAA CTATAATATT ATAGCCAACA AAGACCAAAT TTTTATATAA   
  
  
- TTTTATAATA AAAAATTAAA AAATTTTAAA TTAAAATTGT AATTATTTAA TTTTACTAGA TTAAAATTAA   
  
  
- AAAAATTTTT TTGATTCTAA GAAAAGTTTT TGTAGAAAAA TGCCACCGAA TCTGAGATAA GCAAAAATAT   
  
  
- AAAATTTTTA AGAAAATTTT TTTAATTTTT AAAAATTAAA AAAAGTGAAA TTAAAAAAAA TATAAAAATT   
  
  
- TAATAAAATT ATACAATTAT AATTTTTATT TTAAAATTTT ATTTTTTTAT AATAAAATTA TTTAAAATTT   
  
  
- TAATTTTTAT AAAATTTTTG GTTGGTTTTT TCCGTTGGAT AATGCTTGCT TGATCCTCTG TACTACTAGC   
  
  
- GACCTTCTTC TCGTTGTTCT TCGTTACTGT AGTGACTTTC TTATTGCCAA TCAGGCCACT TAAAAGTAAT   
  
  
- CGACCTTCAA TGACGATCAT CAATTAATCA ATCTTCTCGA CAACTCAATC AATCATCAAT CATCAATTAA   
  
  
- TCAATCTTTT CGAGAACTCA ACCAATCATC AATCGTCAAC TTATTTCCCC AATCTAGTTA ACATTAATCC   
  
  
- TTTTTTGGTA ACATATATTT GTCCACATAC TTTTGCCCAA CTCCTATTCT TTTATGTCTT CTAATTAGAG   
  
  
- TAAAGAGACG AGTAAGAAGA GACGAGAGAG AACAAAGACA AGAAAGAGTT AAAGACAAGA GAAAAGAAAG   
  
  
- ATTAGAACAT TTCGACTCGT ATAGTAGTGG TTCAGTAGTG CACATCTTCG TTTCCCTGTT GTTCAGTAGT   
  
  
- GGCTTCTTCT TATATCAACT GGAAGCTCTT GGGTCAGTT

+     WUN-motif

| Site Name | Organism | Position | Strand | Matrix score. | sequence | function |
| --- | --- | --- | --- | --- | --- | --- |
| WUN-motif | Nicotiana glutinosa | 969 | - | 9 | TAATTACTC |  |
| WUN-motif | Nicotiana glutinosa | 434 | + | 9 | CCATTTCAA |  |

>Potri.018G038100.1   
+ GTTGCTGTCT TTTTTTTTTA TCATGTTATC AAATTAATGG AAATTTAACT CTATTATCAA AATTCTCTTT   
  
  
+ TTTATAAACA TGATAATCTT TTTTATTTTA TTTTCAAGTT AAAAATACTT GGTCCGGCCT GGGACGATGC   
  
  
+ GCTTGCCAAT CTAGTCAATC CTAAATTTAC TAATACGAGT AAGTAATAGC CGAAGGAGCA AAGCTCCAGT   
  
  
+ CCGGCATCTT GTTCAGAACA ATATTACATC CCTGAAATGC CTCGTGAATT GGGTGTCTCT TCCAACTTTC   
  
  
+ TCCAGCAATT TTCTCCCAAC TTCTGCAAGC AGTGATTATC AATTTGAATT ACAGGAGCTA CCTAACATAT   
  
  
+ CCGCCCTAGA ACGAAGGCAG GAATCATCAG CTTTGCCACC TCTTTGCATA ACTGCTACTG AAAGCCATTA   
  
  
+ GCTGGTGAAT GCTCCATTTC AAACTTCCAT TGGCCCGAGT ACTGCTCCTG CTTCCATTGG AAATATCGCT   
  
  
+ GCGCAATCAG ACGAGGCAGG CATTCATATC TGATGATAAA TGTTGCAGGA TTAATTCTAT TTTCGCCGGC   
  
  
+ AGCAAATGGT GGGATTTTTT ACACTGAAAA GTGTTTCAAA AGACTCGAAT TAATGTGGTC GATTTTGGCG   
  
  
+ GGGGCGTGGA ATGGATTCCT CTCTTCCTCA ATCATCAAAC ATCCAGATAC CTGAAACAGA GAGAAGCCCA   
  
  
+ TCTTCAAAAT CATATCGTCA TCGGAGGTCC TTCGCTGCAA TCGGCTGATG ACGGTGCACT TTTTGATGGT   
  
  
+ AGCGAACCAG ATCAGTCAAT GGTCAAATCA TTTGGAAATC AACGAGCTAC AGTTTATCCC TCCTGAATCC   
  
  
+ ATCTTTGAGA ACCTAAAAGA AATTAATTAA AATTAACGAA AGAGAAAAGA GAGAGGTGGT AGAGTTCATA   
  
  
+ GTTGTCGGAT CGGTCCGGAA ATTGATTTAA TTTAGAAATT AATATAGAAA AGAGTTTTGA GTAATTATAT   
  
  
+ TATCTTTAAC GGAGGCAAAC TAAATTAATA TTTTTATTAA AAAAATAATT TTTTTAAAAA AAACTTTTTA   
  
  
+ GACTTTTGTA TAAATTAATC AAATTAAATT AACAAGATTT TACTAAATTA ATAATTTATT AAATTTAACT   
  
  
+ TAAAATCTAA TTTAATATCA CAGCCTGCTT GATATTATAA TATCGGTTGT TTCTGGTTTA AAAATATATT   
  
  
+ AAAATATTAT TTTTTAATTT TTTAAAATTT AATTTTAACA TTAATAAATT AAAATGATCT AATTTTAATT   
  
  
+ TTTTTAAAAA AACTAAGATT CTTTTCAAAA ACATCTTTTT ACGGTGGCTT AGACTCTATT CGTTTTTATA   
  
  
+ TTTTAAAAAT TCTTTTAAAA AAATTAAAAA TTTTTAATTT TTTTCACTTT AATTTTTTTT ATATTTTTAA   
  
  
+ ATTATTTTAA TATGTTAATA TTAAAAATAA AATTTTAAAA TAAAAAAATA TTATTTTAAT AAATTTTAAA   
  
  
+ ATTAAAAATA TTTTAAAAAC CAACCAAAAA AGGCAACCTA TTACGAACGA ACTAGGAGAC ATGATGATCG   
  
  
+ CTGGAAGAAG AGCAACAAGA AGCAATGACA TCACTGAAAG AATAACGGTT AGTCCGGTGA ATTTTCATTA   
  
  
+ GCTGGAAGTT ACTGCTAGTA GTTAATTAGT TAGAAGAGCT GTTGAGTTAG TTAGTAGTTA GTAGTTAATT   
  
  
+ AGTTAGAAAA GCTCTTGAGT TGGTTAGTAG TTAGCAGTTG AATAAAGGGG TTAGATCAAT TGTAATTAGG   
  
  
+ AAAAAACCAT TGTATATAAA CAGGTGTATG AAAACGGGTT GAGGATAAGA AAATACAGAA GATTAATCTC   
  
  
+ ATTTCTCTGC TCATTCTTCT CTGCTCTCTC TTGTTTCTGT TCTTTCTCAA TTTCTGTTCT CTTTTCTTTC   
  
  
+ TAATCTTGTA AAGCTGAGCA TATCATCACC AAGTCATCAC GTGTAGAAGC AAAGGGACAA CAAGTCATCA   
  
  
+ CCGAAGAAGA ATATAGTTGA CCTTCGAGAA CCCAGTCAA  

- CAACGACAGA AAAAAAAAAT AGTACAATAG TTTAATTACC TTTAAATTGA GATAATAGTT TTAAGAGAAA   
  
  
- AAATATTTGT ACTATTAGAA AAAATAAAAT AAAAGTTCAA TTTTTATGAA CCAGGCCGGA CCCTGCTACG   
  
  
- CGAACGGTTA GATCAGTTAG GATTTAAATG ATTATGCTCA TTCATTATCG GCTTCCTCGT TTCGAGGTCA   
  
  
- GGCCGTAGAA CAAGTCTTGT TATAATGTAG GGACTTTACG GAGCACTTAA CCCACAGAGA AGGTTGAAAG   
  
  
- AGGTCGTTAA AAGAGGGTTG AAGACGTTCG TCACTAATAG TTAAACTTAA TGTCCTCGAT GGATTGTATA   
  
  
- GGCGGGATCT TGCTTCCGTC CTTAGTAGTC GAAACGGTGG AGAAACGTAT TGACGATGAC TTTCGGTAAT   
  
  
- CGACCACTTA CGAGGTAAAG TTTGAAGGTA ACCGGGCTCA TGACGAGGAC GAAGGTAACC TTTATAGCGA   
  
  
- CGCGTTAGTC TGCTCCGTCC GTAAGTATAG ACTACTATTT ACAACGTCCT AATTAAGATA AAAGCGGCCG   
  
  
- TCGTTTACCA CCCTAAAAAA TGTGACTTTT CACAAAGTTT TCTGAGCTTA ATTACACCAG CTAAAACCGC   
  
  
- CCCCGCACCT TACCTAAGGA GAGAAGGAGT TAGTAGTTTG TAGGTCTATG GACTTTGTCT CTCTTCGGGT   
  
  
- AGAAGTTTTA GTATAGCAGT AGCCTCCAGG AAGCGACGTT AGCCGACTAC TGCCACGTGA AAAACTACCA   
  
  
- TCGCTTGGTC TAGTCAGTTA CCAGTTTAGT AAACCTTTAG TTGCTCGATG TCAAATAGGG AGGACTTAGG   
  
  
- TAGAAACTCT TGGATTTTCT TTAATTAATT TTAATTGCTT TCTCTTTTCT CTCTCCACCA TCTCAAGTAT   
  
  
- CAACAGCCTA GCCAGGCCTT TAACTAAATT AAATCTTTAA TTATATCTTT TCTCAAAACT CATTAATATA   
  
  
- ATAGAAATTG CCTCCGTTTG ATTTAATTAT AAAAATAATT TTTTTATTAA AAAAATTTTT TTTGAAAAAT   
  
  
- CTGAAAACAT ATTTAATTAG TTTAATTTAA TTGTTCTAAA ATGATTTAAT TATTAAATAA TTTAAATTGA   
  
  
- ATTTTAGATT AAATTATAGT GTCGGACGAA CTATAATATT ATAGCCAACA AAGACCAAAT TTTTATATAA   
  
  
- TTTTATAATA AAAAATTAAA AAATTTTAAA TTAAAATTGT AATTATTTAA TTTTACTAGA TTAAAATTAA   
  
  
- AAAAATTTTT TTGATTCTAA GAAAAGTTTT TGTAGAAAAA TGCCACCGAA TCTGAGATAA GCAAAAATAT   
  
  
- AAAATTTTTA AGAAAATTTT TTTAATTTTT AAAAATTAAA AAAAGTGAAA TTAAAAAAAA TATAAAAATT   
  
  
- TAATAAAATT ATACAATTAT AATTTTTATT TTAAAATTTT ATTTTTTTAT AATAAAATTA TTTAAAATTT   
  
  
- TAATTTTTAT AAAATTTTTG GTTGGTTTTT TCCGTTGGAT AATGCTTGCT TGATCCTCTG TACTACTAGC   
  
  
- GACCTTCTTC TCGTTGTTCT TCGTTACTGT AGTGACTTTC TTATTGCCAA TCAGGCCACT TAAAAGTAAT   
  
  
- CGACCTTCAA TGACGATCAT CAATTAATCA ATCTTCTCGA CAACTCAATC AATCATCAAT CATCAATTAA   
  
  
- TCAATCTTTT CGAGAACTCA ACCAATCATC AATCGTCAAC TTATTTCCCC AATCTAGTTA ACATTAATCC   
  
  
- TTTTTTGGTA ACATATATTT GTCCACATAC TTTTGCCCAA CTCCTATTCT TTTATGTCTT CTAATTAGAG   
  
  
- TAAAGAGACG AGTAAGAAGA GACGAGAGAG AACAAAGACA AGAAAGAGTT AAAGACAAGA GAAAAGAAAG   
  
  
- ATTAGAACAT TTCGACTCGT ATAGTAGTGG TTCAGTAGTG CACATCTTCG TTTCCCTGTT GTTCAGTAGT   
  
  
- GGCTTCTTCT TATATCAACT GGAAGCTCTT GGGTCAGTT

+     as-1

| Site Name | Organism | Position | Strand | Matrix score. | sequence | function |
| --- | --- | --- | --- | --- | --- | --- |
| as-1 | Arabidopsis thaliana | 716 | - | 5 | TGACG |  |
| as-1 | Arabidopsis thaliana | 749 | + | 5 | TGACG |  |

>Potri.018G038100.1   
+ GTTGCTGTCT TTTTTTTTTA TCATGTTATC AAATTAATGG AAATTTAACT CTATTATCAA AATTCTCTTT   
  
  
+ TTTATAAACA TGATAATCTT TTTTATTTTA TTTTCAAGTT AAAAATACTT GGTCCGGCCT GGGACGATGC   
  
  
+ GCTTGCCAAT CTAGTCAATC CTAAATTTAC TAATACGAGT AAGTAATAGC CGAAGGAGCA AAGCTCCAGT   
  
  
+ CCGGCATCTT GTTCAGAACA ATATTACATC CCTGAAATGC CTCGTGAATT GGGTGTCTCT TCCAACTTTC   
  
  
+ TCCAGCAATT TTCTCCCAAC TTCTGCAAGC AGTGATTATC AATTTGAATT ACAGGAGCTA CCTAACATAT   
  
  
+ CCGCCCTAGA ACGAAGGCAG GAATCATCAG CTTTGCCACC TCTTTGCATA ACTGCTACTG AAAGCCATTA   
  
  
+ GCTGGTGAAT GCTCCATTTC AAACTTCCAT TGGCCCGAGT ACTGCTCCTG CTTCCATTGG AAATATCGCT   
  
  
+ GCGCAATCAG ACGAGGCAGG CATTCATATC TGATGATAAA TGTTGCAGGA TTAATTCTAT TTTCGCCGGC   
  
  
+ AGCAAATGGT GGGATTTTTT ACACTGAAAA GTGTTTCAAA AGACTCGAAT TAATGTGGTC GATTTTGGCG   
  
  
+ GGGGCGTGGA ATGGATTCCT CTCTTCCTCA ATCATCAAAC ATCCAGATAC CTGAAACAGA GAGAAGCCCA   
  
  
+ TCTTCAAAAT CATATCGTCA TCGGAGGTCC TTCGCTGCAA TCGGCTGATG ACGGTGCACT TTTTGATGGT   
  
  
+ AGCGAACCAG ATCAGTCAAT GGTCAAATCA TTTGGAAATC AACGAGCTAC AGTTTATCCC TCCTGAATCC   
  
  
+ ATCTTTGAGA ACCTAAAAGA AATTAATTAA AATTAACGAA AGAGAAAAGA GAGAGGTGGT AGAGTTCATA   
  
  
+ GTTGTCGGAT CGGTCCGGAA ATTGATTTAA TTTAGAAATT AATATAGAAA AGAGTTTTGA GTAATTATAT   
  
  
+ TATCTTTAAC GGAGGCAAAC TAAATTAATA TTTTTATTAA AAAAATAATT TTTTTAAAAA AAACTTTTTA   
  
  
+ GACTTTTGTA TAAATTAATC AAATTAAATT AACAAGATTT TACTAAATTA ATAATTTATT AAATTTAACT   
  
  
+ TAAAATCTAA TTTAATATCA CAGCCTGCTT GATATTATAA TATCGGTTGT TTCTGGTTTA AAAATATATT   
  
  
+ AAAATATTAT TTTTTAATTT TTTAAAATTT AATTTTAACA TTAATAAATT AAAATGATCT AATTTTAATT   
  
  
+ TTTTTAAAAA AACTAAGATT CTTTTCAAAA ACATCTTTTT ACGGTGGCTT AGACTCTATT CGTTTTTATA   
  
  
+ TTTTAAAAAT TCTTTTAAAA AAATTAAAAA TTTTTAATTT TTTTCACTTT AATTTTTTTT ATATTTTTAA   
  
  
+ ATTATTTTAA TATGTTAATA TTAAAAATAA AATTTTAAAA TAAAAAAATA TTATTTTAAT AAATTTTAAA   
  
  
+ ATTAAAAATA TTTTAAAAAC CAACCAAAAA AGGCAACCTA TTACGAACGA ACTAGGAGAC ATGATGATCG   
  
  
+ CTGGAAGAAG AGCAACAAGA AGCAATGACA TCACTGAAAG AATAACGGTT AGTCCGGTGA ATTTTCATTA   
  
  
+ GCTGGAAGTT ACTGCTAGTA GTTAATTAGT TAGAAGAGCT GTTGAGTTAG TTAGTAGTTA GTAGTTAATT   
  
  
+ AGTTAGAAAA GCTCTTGAGT TGGTTAGTAG TTAGCAGTTG AATAAAGGGG TTAGATCAAT TGTAATTAGG   
  
  
+ AAAAAACCAT TGTATATAAA CAGGTGTATG AAAACGGGTT GAGGATAAGA AAATACAGAA GATTAATCTC   
  
  
+ ATTTCTCTGC TCATTCTTCT CTGCTCTCTC TTGTTTCTGT TCTTTCTCAA TTTCTGTTCT CTTTTCTTTC   
  
  
+ TAATCTTGTA AAGCTGAGCA TATCATCACC AAGTCATCAC GTGTAGAAGC AAAGGGACAA CAAGTCATCA   
  
  
+ CCGAAGAAGA ATATAGTTGA CCTTCGAGAA CCCAGTCAA  

- CAACGACAGA AAAAAAAAAT AGTACAATAG TTTAATTACC TTTAAATTGA GATAATAGTT TTAAGAGAAA   
  
  
- AAATATTTGT ACTATTAGAA AAAATAAAAT AAAAGTTCAA TTTTTATGAA CCAGGCCGGA CCCTGCTACG   
  
  
- CGAACGGTTA GATCAGTTAG GATTTAAATG ATTATGCTCA TTCATTATCG GCTTCCTCGT TTCGAGGTCA   
  
  
- GGCCGTAGAA CAAGTCTTGT TATAATGTAG GGACTTTACG GAGCACTTAA CCCACAGAGA AGGTTGAAAG   
  
  
- AGGTCGTTAA AAGAGGGTTG AAGACGTTCG TCACTAATAG TTAAACTTAA TGTCCTCGAT GGATTGTATA   
  
  
- GGCGGGATCT TGCTTCCGTC CTTAGTAGTC GAAACGGTGG AGAAACGTAT TGACGATGAC TTTCGGTAAT   
  
  
- CGACCACTTA CGAGGTAAAG TTTGAAGGTA ACCGGGCTCA TGACGAGGAC GAAGGTAACC TTTATAGCGA   
  
  
- CGCGTTAGTC TGCTCCGTCC GTAAGTATAG ACTACTATTT ACAACGTCCT AATTAAGATA AAAGCGGCCG   
  
  
- TCGTTTACCA CCCTAAAAAA TGTGACTTTT CACAAAGTTT TCTGAGCTTA ATTACACCAG CTAAAACCGC   
  
  
- CCCCGCACCT TACCTAAGGA GAGAAGGAGT TAGTAGTTTG TAGGTCTATG GACTTTGTCT CTCTTCGGGT   
  
  
- AGAAGTTTTA GTATAGCAGT AGCCTCCAGG AAGCGACGTT AGCCGACTAC TGCCACGTGA AAAACTACCA   
  
  
- TCGCTTGGTC TAGTCAGTTA CCAGTTTAGT AAACCTTTAG TTGCTCGATG TCAAATAGGG AGGACTTAGG   
  
  
- TAGAAACTCT TGGATTTTCT TTAATTAATT TTAATTGCTT TCTCTTTTCT CTCTCCACCA TCTCAAGTAT   
  
  
- CAACAGCCTA GCCAGGCCTT TAACTAAATT AAATCTTTAA TTATATCTTT TCTCAAAACT CATTAATATA   
  
  
- ATAGAAATTG CCTCCGTTTG ATTTAATTAT AAAAATAATT TTTTTATTAA AAAAATTTTT TTTGAAAAAT   
  
  
- CTGAAAACAT ATTTAATTAG TTTAATTTAA TTGTTCTAAA ATGATTTAAT TATTAAATAA TTTAAATTGA   
  
  
- ATTTTAGATT AAATTATAGT GTCGGACGAA CTATAATATT ATAGCCAACA AAGACCAAAT TTTTATATAA   
  
  
- TTTTATAATA AAAAATTAAA AAATTTTAAA TTAAAATTGT AATTATTTAA TTTTACTAGA TTAAAATTAA   
  
  
- AAAAATTTTT TTGATTCTAA GAAAAGTTTT TGTAGAAAAA TGCCACCGAA TCTGAGATAA GCAAAAATAT   
  
  
- AAAATTTTTA AGAAAATTTT TTTAATTTTT AAAAATTAAA AAAAGTGAAA TTAAAAAAAA TATAAAAATT   
  
  
- TAATAAAATT ATACAATTAT AATTTTTATT TTAAAATTTT ATTTTTTTAT AATAAAATTA TTTAAAATTT   
  
  
- TAATTTTTAT AAAATTTTTG GTTGGTTTTT TCCGTTGGAT AATGCTTGCT TGATCCTCTG TACTACTAGC   
  
  
- GACCTTCTTC TCGTTGTTCT TCGTTACTGT AGTGACTTTC TTATTGCCAA TCAGGCCACT TAAAAGTAAT   
  
  
- CGACCTTCAA TGACGATCAT CAATTAATCA ATCTTCTCGA CAACTCAATC AATCATCAAT CATCAATTAA   
  
  
- TCAATCTTTT CGAGAACTCA ACCAATCATC AATCGTCAAC TTATTTCCCC AATCTAGTTA ACATTAATCC   
  
  
- TTTTTTGGTA ACATATATTT GTCCACATAC TTTTGCCCAA CTCCTATTCT TTTATGTCTT CTAATTAGAG   
  
  
- TAAAGAGACG AGTAAGAAGA GACGAGAGAG AACAAAGACA AGAAAGAGTT AAAGACAAGA GAAAAGAAAG   
  
  
- ATTAGAACAT TTCGACTCGT ATAGTAGTGG TTCAGTAGTG CACATCTTCG TTTCCCTGTT GTTCAGTAGT   
  
  
- GGCTTCTTCT TATATCAACT GGAAGCTCTT GGGTCAGTT

+     box S

| Site Name | Organism | Position | Strand | Matrix score. | sequence | function |
| --- | --- | --- | --- | --- | --- | --- |
| box S | Arabidopsis thaliana | 1303 | - | 7 | AGCCACC |  |

>Potri.018G038100.1   
+ GTTGCTGTCT TTTTTTTTTA TCATGTTATC AAATTAATGG AAATTTAACT CTATTATCAA AATTCTCTTT   
  
  
+ TTTATAAACA TGATAATCTT TTTTATTTTA TTTTCAAGTT AAAAATACTT GGTCCGGCCT GGGACGATGC   
  
  
+ GCTTGCCAAT CTAGTCAATC CTAAATTTAC TAATACGAGT AAGTAATAGC CGAAGGAGCA AAGCTCCAGT   
  
  
+ CCGGCATCTT GTTCAGAACA ATATTACATC CCTGAAATGC CTCGTGAATT GGGTGTCTCT TCCAACTTTC   
  
  
+ TCCAGCAATT TTCTCCCAAC TTCTGCAAGC AGTGATTATC AATTTGAATT ACAGGAGCTA CCTAACATAT   
  
  
+ CCGCCCTAGA ACGAAGGCAG GAATCATCAG CTTTGCCACC TCTTTGCATA ACTGCTACTG AAAGCCATTA   
  
  
+ GCTGGTGAAT GCTCCATTTC AAACTTCCAT TGGCCCGAGT ACTGCTCCTG CTTCCATTGG AAATATCGCT   
  
  
+ GCGCAATCAG ACGAGGCAGG CATTCATATC TGATGATAAA TGTTGCAGGA TTAATTCTAT TTTCGCCGGC   
  
  
+ AGCAAATGGT GGGATTTTTT ACACTGAAAA GTGTTTCAAA AGACTCGAAT TAATGTGGTC GATTTTGGCG   
  
  
+ GGGGCGTGGA ATGGATTCCT CTCTTCCTCA ATCATCAAAC ATCCAGATAC CTGAAACAGA GAGAAGCCCA   
  
  
+ TCTTCAAAAT CATATCGTCA TCGGAGGTCC TTCGCTGCAA TCGGCTGATG ACGGTGCACT TTTTGATGGT   
  
  
+ AGCGAACCAG ATCAGTCAAT GGTCAAATCA TTTGGAAATC AACGAGCTAC AGTTTATCCC TCCTGAATCC   
  
  
+ ATCTTTGAGA ACCTAAAAGA AATTAATTAA AATTAACGAA AGAGAAAAGA GAGAGGTGGT AGAGTTCATA   
  
  
+ GTTGTCGGAT CGGTCCGGAA ATTGATTTAA TTTAGAAATT AATATAGAAA AGAGTTTTGA GTAATTATAT   
  
  
+ TATCTTTAAC GGAGGCAAAC TAAATTAATA TTTTTATTAA AAAAATAATT TTTTTAAAAA AAACTTTTTA   
  
  
+ GACTTTTGTA TAAATTAATC AAATTAAATT AACAAGATTT TACTAAATTA ATAATTTATT AAATTTAACT   
  
  
+ TAAAATCTAA TTTAATATCA CAGCCTGCTT GATATTATAA TATCGGTTGT TTCTGGTTTA AAAATATATT   
  
  
+ AAAATATTAT TTTTTAATTT TTTAAAATTT AATTTTAACA TTAATAAATT AAAATGATCT AATTTTAATT   
  
  
+ TTTTTAAAAA AACTAAGATT CTTTTCAAAA ACATCTTTTT ACGGTGGCTT AGACTCTATT CGTTTTTATA   
  
  
+ TTTTAAAAAT TCTTTTAAAA AAATTAAAAA TTTTTAATTT TTTTCACTTT AATTTTTTTT ATATTTTTAA   
  
  
+ ATTATTTTAA TATGTTAATA TTAAAAATAA AATTTTAAAA TAAAAAAATA TTATTTTAAT AAATTTTAAA   
  
  
+ ATTAAAAATA TTTTAAAAAC CAACCAAAAA AGGCAACCTA TTACGAACGA ACTAGGAGAC ATGATGATCG   
  
  
+ CTGGAAGAAG AGCAACAAGA AGCAATGACA TCACTGAAAG AATAACGGTT AGTCCGGTGA ATTTTCATTA   
  
  
+ GCTGGAAGTT ACTGCTAGTA GTTAATTAGT TAGAAGAGCT GTTGAGTTAG TTAGTAGTTA GTAGTTAATT   
  
  
+ AGTTAGAAAA GCTCTTGAGT TGGTTAGTAG TTAGCAGTTG AATAAAGGGG TTAGATCAAT TGTAATTAGG   
  
  
+ AAAAAACCAT TGTATATAAA CAGGTGTATG AAAACGGGTT GAGGATAAGA AAATACAGAA GATTAATCTC   
  
  
+ ATTTCTCTGC TCATTCTTCT CTGCTCTCTC TTGTTTCTGT TCTTTCTCAA TTTCTGTTCT CTTTTCTTTC   
  
  
+ TAATCTTGTA AAGCTGAGCA TATCATCACC AAGTCATCAC GTGTAGAAGC AAAGGGACAA CAAGTCATCA   
  
  
+ CCGAAGAAGA ATATAGTTGA CCTTCGAGAA CCCAGTCAA  

- CAACGACAGA AAAAAAAAAT AGTACAATAG TTTAATTACC TTTAAATTGA GATAATAGTT TTAAGAGAAA   
  
  
- AAATATTTGT ACTATTAGAA AAAATAAAAT AAAAGTTCAA TTTTTATGAA CCAGGCCGGA CCCTGCTACG   
  
  
- CGAACGGTTA GATCAGTTAG GATTTAAATG ATTATGCTCA TTCATTATCG GCTTCCTCGT TTCGAGGTCA   
  
  
- GGCCGTAGAA CAAGTCTTGT TATAATGTAG GGACTTTACG GAGCACTTAA CCCACAGAGA AGGTTGAAAG   
  
  
- AGGTCGTTAA AAGAGGGTTG AAGACGTTCG TCACTAATAG TTAAACTTAA TGTCCTCGAT GGATTGTATA   
  
  
- GGCGGGATCT TGCTTCCGTC CTTAGTAGTC GAAACGGTGG AGAAACGTAT TGACGATGAC TTTCGGTAAT   
  
  
- CGACCACTTA CGAGGTAAAG TTTGAAGGTA ACCGGGCTCA TGACGAGGAC GAAGGTAACC TTTATAGCGA   
  
  
- CGCGTTAGTC TGCTCCGTCC GTAAGTATAG ACTACTATTT ACAACGTCCT AATTAAGATA AAAGCGGCCG   
  
  
- TCGTTTACCA CCCTAAAAAA TGTGACTTTT CACAAAGTTT TCTGAGCTTA ATTACACCAG CTAAAACCGC   
  
  
- CCCCGCACCT TACCTAAGGA GAGAAGGAGT TAGTAGTTTG TAGGTCTATG GACTTTGTCT CTCTTCGGGT   
  
  
- AGAAGTTTTA GTATAGCAGT AGCCTCCAGG AAGCGACGTT AGCCGACTAC TGCCACGTGA AAAACTACCA   
  
  
- TCGCTTGGTC TAGTCAGTTA CCAGTTTAGT AAACCTTTAG TTGCTCGATG TCAAATAGGG AGGACTTAGG   
  
  
- TAGAAACTCT TGGATTTTCT TTAATTAATT TTAATTGCTT TCTCTTTTCT CTCTCCACCA TCTCAAGTAT   
  
  
- CAACAGCCTA GCCAGGCCTT TAACTAAATT AAATCTTTAA TTATATCTTT TCTCAAAACT CATTAATATA   
  
  
- ATAGAAATTG CCTCCGTTTG ATTTAATTAT AAAAATAATT TTTTTATTAA AAAAATTTTT TTTGAAAAAT   
  
  
- CTGAAAACAT ATTTAATTAG TTTAATTTAA TTGTTCTAAA ATGATTTAAT TATTAAATAA TTTAAATTGA   
  
  
- ATTTTAGATT AAATTATAGT GTCGGACGAA CTATAATATT ATAGCCAACA AAGACCAAAT TTTTATATAA   
  
  
- TTTTATAATA AAAAATTAAA AAATTTTAAA TTAAAATTGT AATTATTTAA TTTTACTAGA TTAAAATTAA   
  
  
- AAAAATTTTT TTGATTCTAA GAAAAGTTTT TGTAGAAAAA TGCCACCGAA TCTGAGATAA GCAAAAATAT   
  
  
- AAAATTTTTA AGAAAATTTT TTTAATTTTT AAAAATTAAA AAAAGTGAAA TTAAAAAAAA TATAAAAATT   
  
  
- TAATAAAATT ATACAATTAT AATTTTTATT TTAAAATTTT ATTTTTTTAT AATAAAATTA TTTAAAATTT   
  
  
- TAATTTTTAT AAAATTTTTG GTTGGTTTTT TCCGTTGGAT AATGCTTGCT TGATCCTCTG TACTACTAGC   
  
  
- GACCTTCTTC TCGTTGTTCT TCGTTACTGT AGTGACTTTC TTATTGCCAA TCAGGCCACT TAAAAGTAAT   
  
  
- CGACCTTCAA TGACGATCAT CAATTAATCA ATCTTCTCGA CAACTCAATC AATCATCAAT CATCAATTAA   
  
  
- TCAATCTTTT CGAGAACTCA ACCAATCATC AATCGTCAAC TTATTTCCCC AATCTAGTTA ACATTAATCC   
  
  
- TTTTTTGGTA ACATATATTT GTCCACATAC TTTTGCCCAA CTCCTATTCT TTTATGTCTT CTAATTAGAG   
  
  
- TAAAGAGACG AGTAAGAAGA GACGAGAGAG AACAAAGACA AGAAAGAGTT AAAGACAAGA GAAAAGAAAG   
  
  
- ATTAGAACAT TTCGACTCGT ATAGTAGTGG TTCAGTAGTG CACATCTTCG TTTCCCTGTT GTTCAGTAGT   
  
  
- GGCTTCTTCT TATATCAACT GGAAGCTCTT GGGTCAGTT
